# Unveiling Chemical Profile and Insecticidal Potential of Essential Oils from Leaves of Seven *Eugenia* L. Species (Myrtaceae)

**DOI:** 10.3390/plants15091406

**Published:** 2026-05-05

**Authors:** Lorene Armstrong, Nayana Figueiredo Pereira, Diefrey Ribeiro Campos, Yara Peluso Cid, Irailson Thierry Monchak, Neide Mara Menezes Epifânio, Douglas Siqueira Almeida Chaves, Jane Manfron

**Affiliations:** 1 Postgraduate Program in Pharmaceutical Sciences, State University of Ponta Grossa, Ponta Grossa 84030900, Paraná, Brazil; thierry.monchak@gmail.com (I.T.M.); jane@uepg.br (J.M.); 2 Postgraduate Program in Health Sciences, State University of Ponta Grossa, Ponta Grossa 84030900, Paraná, Brazil; 3 Programa de Pós-Graduação em Química, Universidade Federal Rural do Rio de Janeiro, Seropédica, Rio de Janeiro 23897000, Brazil; nayanafpereira@hotmail.com; 4 Clinical Research and Technological Innovation Center—Laerte Grisi, Federal Rural University of Rio de Janeiro, Seropédica, Rio de Janeiro 23897000, Brazil; diefrey8@gmail.com (D.R.C.); yarapcid@gmail.com (Y.P.C.); 5 Laboratório de Farmacognosia, Universidade Federal Rural do Rio de Janeiro, Seropédica, Rio de Janeiro 23897000, Brazil; neide.epifanio@gmail.com (N.M.M.E.);

**Keywords:** *Ctenocephalides felis felis*, GC-MS, Myrtaceae, PCA

## Abstract

The genus *Eugenia* (Myrtaceae) is widely distributed in Brazil and is known for producing diverse secondary metabolites with various biological activities, although several species remain poorly explored. This study aimed to characterize the chemical composition of essential oils (EOs) from the leaves of seven *Eugenia* species (*E. brasiliensis*, *E. involucrata*, *E. longipedunculata*, *E. myrcianthes*, *E. neoverrucosa*, *E. pyriformis*, and *E. uniflora*), compare their chemical profiles using multivariate analysis, and evaluate their insecticidal activity against the flea *Ctenocephalides felis felis*. EOs were obtained from dried leaves by hydrodistillation using a Clevenger apparatus and analyzed by gas chromatography–mass spectrometry (GC–MS). Principal component analysis (PCA) was applied to compare chemical compositions, and contact bioassays were conducted to assess insecticidal activity against adult fleas. The EOs showed distinct chemical compositions, with major constituents varying by species, including α-pinene, (E)-caryophyllene, viridiflorene, β-selinene, limonene, and germacrone. PCA revealed clear differences among species, particularly highlighting oils dominated by *α*-pinene and sesquiterpene-derived compounds. In the bioassays, *E. uniflora* showed the highest insecticidal activity, reaching 95.1% mortality at 800 µg·cm^−2^ and presenting an LC_50_ of 9.12 µg·cm^−2^, whereas *E. brasiliensis* showed moderate activity (LC_50_ = 157.82 µg·cm^−2^). These findings expand the chemical knowledge of the genus and indicate the potential of *E. uniflora* EO as a natural source of insecticidal compounds against *C. felis felis*.

## 1. Introduction

*Ctenocephalides felis felis* is the most common flea species found in cats and dogs. These ectoparasites have a remarkable ability to jump and feed on their hosts’ blood, from which they may remain for several consecutive days. In addition to causing skin allergies in pets, they can act as vectors of pathogens harmful to humans and other animals, including the Gram-negative bacteria *Rickettsia felis* and *Bartonella henselae*. These microorganisms are associated with typhus-like illness, flea-borne spotted fever, and cat-scratch disease in humans, respectively, representing a relevant public health concern [1,2,3,4].

Several classes of natural products contain compounds with antifeedant, insecticidal, and repellent properties, such as alkaloids, flavonoids, and terpenoids. Essential oils (EOs) are primarily composed of monoterpenes, sesquiterpenes, and phenylpropanoids [1,4]. Previous studies have reported the insecticidal potential of EOs from *Cannabis sativa* and *Piper aduncum* L. against *C. felis felis* [5,6].

The genus *Eugenia* L. (Myrtaceae) comprises approximately 1239 accepted species worldwide [7] and is widely distributed in Brazil, with around 421 species [8]. Species of this genus are known to contain diverse chemical classes, including flavonoids, phenolic acids, tannins, and terpenoids [9]. Numerous *Eugenia* species have had their EOs characterized and described in the literature, and these findings will be discussed in further detail in the Section 3 [9,10].

*Eugenia* species have demonstrated biological activity against various parasites. *Eugenia uniflora* L. exhibits anti-*Leishmania* activity [11], while *Eugenia stipitata* McVaugh shows larvicidal and pupicidal effects against *Aedes aegypti*, with LC_50_ values of 0.34 mg/mL and 2.33 mg/mL, respectively [12]. The EO from the leaves and fruits of *Eugenia langsdorffii* O. Berg has also been reported to possess acaricidal activity against *Tetranychus urticae* [13]. Despite evidence of antiparasitic activity in some *Eugenia* species, no study has evaluated the genus against *C. felis felis*.

The aims of this study are: (i) to identify compounds not previously described for *Eugenia longipedunculata* Nied.; (ii) to analyze the chemical profiles of seven *Eugenia* species by gas chromatography coupled with mass spectrometry (GC–MS); (iii) to compare the profiles using principal component analysis (PCA); and (iv) to evaluate the insecticidal activity against *C. felis felis*, which has not yet been studied for this genus. Therefore, the chemical investigation of *Eugenia* species is relevant for generating new data on understudied taxa, for comparing their chemical profiles with regionally related species, and for contributing to the understanding of biological activities not yet reported in the literature for the parasite evaluated.

## 2. Results

### 2.1. Chemical Composition of Essential Oil

It is noted that among the *Eugenia* species: *E. brasiliensis* Lam.; *E. longipedunculata*; *E. neoverrucosa* Sobral. and *E. uniflora* had higher yields (Table 1). *Eugenia neoverrucosa* stands out, presenting a yield of 0.90%. The color of the oil varies from colorless to yellow in *E. involucrata*, *E. myrcianthes*, *E. neoverrucosa*, and *E. pyriformis*, transparent and very slightly yellowish in *E. brasiliensis* and *E. longipedunculata*, and yellow in *E. uniflora*. Regarding the smell, all species have an aromatic, fresh, and slightly citrusy odor, with *E. brasiliensis* and *E. neoverrucosa* being sweeter.

**Figure 1:**
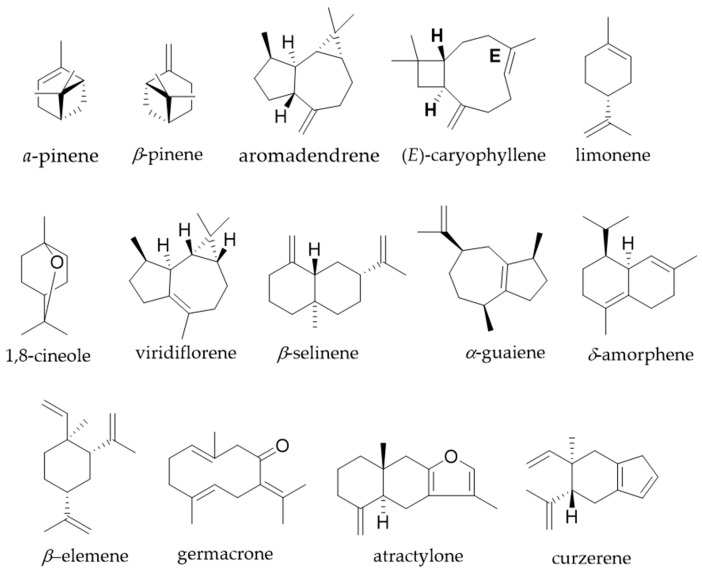
Major structures in *Eugenia* species.

According to Table 2, the major components identified are *α*-pinene (20.51%), (*E*)-caryophyllene (17.52%) and 1,8-cineole (17.01%) in *E. brasiliensis*; (*E*)-caryophyllene (25.59%), viridiflorene (26.32%) and aromadendrene (18.96%) in *E. involucrata*; (*E*)-caryophyllene (19.19%) and viridiflorene (9.31%) in *E. longipedunculata*; *β*-selinene (22.88%), *α*-guaiene (16.23%), *δ*-amorphene (12.21%) and *β*-elemene (9.73%) in *E. myrcianthes*; *α*-pinene (79.92%) in *E. neoverrucosa*; *α*-pinene (32.94%) and limonene (24.56%) in *E. pyriformis*, and the germacrene-type sesquiterpenoids germacrone (26.48%), atractylone (11.08%) and curzerene (9.94%) in *E. uniflora*, Figure 1. Both hydrocarbon and oxygenated mono- and sesquiterpenes were identified. Minor compounds are listed in Table 2.

### 2.2. Chemical Analysis of Essential Oils from Eugenia Species Using Molecular Networking and Principal Component Analysis (PCA)

**Figure 2:**
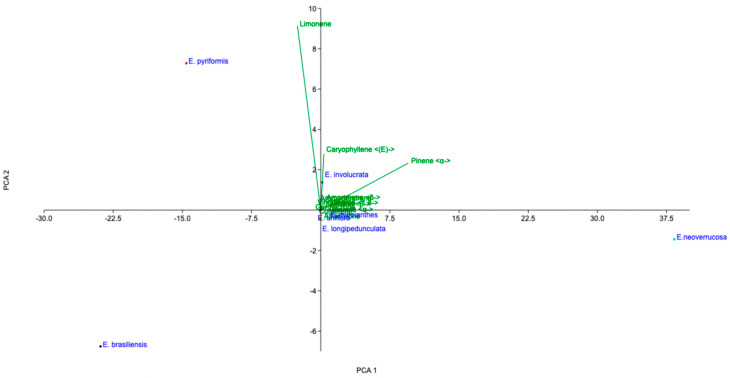
Principal component analysis (PCA1 = 94.53, PCA2 = 4.31%, and PCA3 = 1.15) of 7 essential oil samples from *Eugenia* species, obtained by hydrodistillation and analyzed by GC–MS, and the correlation of all chemical components was identified in the analysis. Only compounds with concentrations above 10% were considered for analysis.

Correlation and multivariate analyses were conducted to highlight differences in chemical composition among the seven *Eugenia* species (Table 2) and the compounds presented (Figure 2). The loadings were calculated and are shown in Figure S1. Similarities among the EOs are evident in the PCA (Figure 2).

PC1 was mainly associated with monoterpenes such as α-pinene and sesquiterpenes like (*E*)-caryophyllene, which contributed positively to the separation along the horizontal axis. In contrast, PC2 was strongly influenced by limonene, which showed a high loading and was the primary driver of vertical separation.

The score plot revealed distinct clustering patterns among the samples. *E. neoverrucosa* was clearly separated along the positive PC1 axis, indicating a strong association with compounds such as α-pinene and (*E*)-caryophyllene. On the opposite side, *E. pyriformis* was positioned in the negative PC1 region, suggesting a different chemical profile with lower contributions from these compounds.

Additionally, *E. brasiliensis* was distinctly separated along the negative PC2 axis, indicating a unique composition likely characterized by lower limonene content or the presence of other compounds not strongly represented in the first two components. Samples such as *E. involucrata* and *E. longipedunculata* were grouped near the origin, suggesting similar and less chemically distinct profiles compared to the other species.

The loading plot corroborated these observations, showing that limonene was the most influential variable for PC2, while α-pinene and (*E*)-caryophyllene contributed significantly to PC1. The clustering pattern indicates clear chemical differentiation among species, reflecting variability in their secondary metabolite profiles.

**Figure 3:**
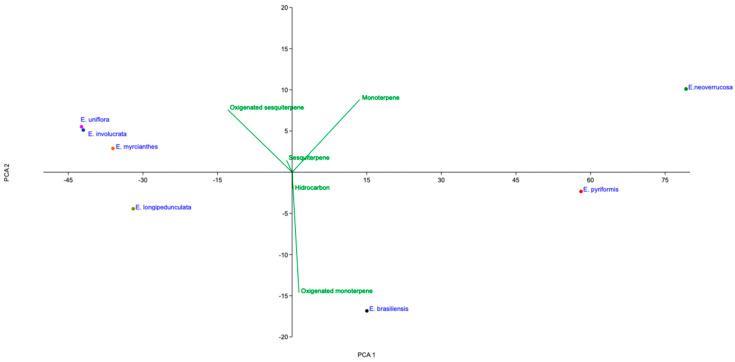
Principal component analysis (PCA1 = 96.70, PCA2 = 2.90%) of 7 essential oil samples from *Eugenia* species, obtained by hydrodistillation and analyzed by GC–MS, and the correlation of the presented class (hydrocarbons, monoterpenes, oxygenated monoterpenes, sesquiterpenes, and oxygenated sesquiterpenes).

In Figure 3, the score plot showed distinct groupings among the species. *E. neoverrucosa* was clearly separated along the positive PC1 and PC2 axes, indicating a profile rich in monoterpenes and, to some extent, oxygenated sesquiterpenes. *E. pyriformis* was also positioned on the positive side of PC1 but closer to the negative side of PC2, suggesting a composition dominated by monoterpenes with lower contributions from oxygenated compounds.

In contrast, *E. uniflora*, *E. involucrata*, and *E. myrcianthes* clustered in the negative PC1 and positive PC2 regions, indicating a stronger association with oxygenated sesquiterpenes. *E. longipedunculata* fell in the negative regions of both PC1 and PC2, suggesting a distinct profile with reduced contributions from both monoterpenes and oxygenated compounds.

Notably, *E. brasiliensis* was separated along the negative PC2 axis, strongly associated with oxygenated monoterpenes, indicating a unique chemical profile compared to the other species.

The loading plot confirmed that monoterpenes, oxygenated monoterpenes, and oxygenated sesquiterpenes were the main variables responsible for sample discrimination, while hydrocarbons and sesquiterpenes showed lower influence on the separation pattern.

### 2.3. Insecticidal Activity Against Adult Fleas

A general increase in insecticidal activity was observed with increasing concentration for most of the evaluated EOs, although the intensity and consistency of this response varied among species. The EO of *E. uniflora* was the most active across nearly the entire concentration range, achieving 95.1% mortality at 800 µg·cm^−2^, demonstrating a strong dose–response relationship. The EO of *E. brasiliensis* showed moderate activity at low and intermediate concentrations but exhibited a marked increase at higher doses, achieving 80.0% mortality at 800 µg·cm^−2^.

In contrast, EOs *pyriformis*, *E. neoverrucosa*, and *E. myrcianthes* showed intermediate insecticidal activity, with mortalities of 60.0%, 52.4%, and 50.0%, respectively, at the highest tested concentration. Finally, *E. involucrata* was the least active oil among those evaluated, with a maximum mortality of 38.9% at 800 µg·cm^−2^ (Table 3).

Based on the mortality data, LC_50_ estimation was possible only for *E. uniflora* and *E. brasiliensis*. *E. uniflora* showed an LC_50_ of 9.12 µg·cm^−2^ (95% CI: 6.59–12.17), whereas *E. brasiliensis* presented an LC_50_ of 157.82 µg/cm^2^ (95% CI: 116.16–225.18). The slope coefficients were 1.19 for *E. uniflora* and 1.09 for *E. brasiliensis*. Chi-square tests indicated no significant deviation from the model (*p* ≥ 0.999) (Table 4).

## 3. Discussion

### 3.1. Chemistry of Essential Oils

According to Borsoi et al. [14], the EO yield from dried leaves of *E. brasiliensis* by hydrodistillation was 0.39%, similar to the value observed in this study and higher than yields from fresh leaves (0.08–0.14%) [15]. For *E. involucrata*, yields of 0.45% and 0.21% from dried leaves have been reported [16], which are higher than those obtained in the present study. Ref. [17] reported yields of 0.06% for *E. myrcianthes*, 0.42% for *E. neoverrucosa*, and 0.17% for *E. pyriformis*. In *E. uniflora*, yields reported in the literature vary widely (0.15–3.1%), and the yield obtained in this study (0.55%) falls within this range [14]. Overall, the yields obtained here are consistent with the literature, except for *E. neoverrucosa*, which exhibited a considerably higher yield.

Similar chemical profiles are observed in *E. brasiliensis*, *E. neoverrucosa,* and *E. pyriformis,* particularly due to the presence of *α*-pinene, which was most abundant in *E. neoverrucosa.* The substance (*E*)-caryophyllene was common to *E. brasiliensis* and *E. longipedunculata.* Viridiflorene was found in *E. involucrata* and *E. longipedunculata*. Differences among species and variations in minor constituents are shown in Table 2.

The major compounds observed in *E. brasiliensis* have previously been reported by [18], whose samples collected from different cities revealed that this species generally presents the same major constituents. These authors identified *α*-selinene (13.3–14.8%) and *β*-selinene (12.6–17.3%) as the main compounds and reported the bicyclic sesquiterpene *(E)*-caryophyllene (8.7–12.6%), which was also reported in the present study. The remaining compounds were also detected, but only in minor amounts. In fresh leaves of *E. brasiliensis*, the major constituent was *α*-muurolol (12.1%), and *α*-pinene was not detected [19]. In contrast, *α*-pinene (15.94%) was identified as the major compound in winter leaves of *E. brasiliensis* [15]. Germacrene B (22.17 ± 1.72), byciclogermacrene (19.76 ± 1.28), and β-elemene (10.86 ± 0.93) were detected as major compounds in *E. involucrata* by [16]. Elixene (26.53%) and caryophyllene (13.16%) were identified by [20]. This last compound corroborates with the current study: aromadendrene was found in minor amounts, and viridiflorene was not found in the studies mentioned.

There are no reports on the EO composition of *E. longipedunculata*; however, *(E)*-caryophyllene was common in other species examined in this study and in other species within the genus. The compound viridiflorene has been reported in dried leaves of *E. uniflora* [21], and *E. myrcianthes* [17,22] also reported *E. myrcianthes* (syn.: *Hexachlamys edulis* (O. Berg) Kausel & D. Legrand), in which the hydrocarbon sesquiterpene *β*-selinene (16.1%) was the major compound; *β*-elemene (1.0%) was detected in very small amounts, while the remaining constituents were not reported. The compound β-copaen-4-*α*-ol (31.7%) was identified by [17].

Nonpolar fractions of *E. myrcianthes* contained the following volatile compounds: in the hexane fraction, the triterpenes lupenyl acetate (45.39%), *β*-amyrone (12.69%), and squalene (12.18%); the cadinane sesquiterpenoid *τ*-muurolol (10.80%); viridiflorene (7.21%); and the tricyclic sesquiterpenoid spathulenol (4.57%). In the chloroform fraction, *δ*-cadinene (9.0%) and α-muurolene (4.15%) were detected, whereas in this study, they were present at 0.71% [23]. Different compounds were observed in extractions using nonpolar solvents, with sesquiterpenes and triterpenes predominating, whereas a distinct profile was obtained from Clevenger hydrodistillation in the present work.

In *E. neoverrucosa*, ref. [17] reported a high α-pinene concentration of 94.5%. In *E. pyriformis*, previous research [17] identified the bicyclic monoterpene isomers *β*-pinene (39.7%) and *α*-pinene (31.5%) as major compounds. Hydrodistillation of dried leaves identified *β*-caryophyllene (17.82%), bicyclogermacrene (12.84%), and globulol (5.96%) [24]. A seasonal analysis showed *β*-pinene as the most prevalent, with levels fluctuating between 0.2% and 25.7% and peaking in January. *α*-Pinene was present in small amounts (0.5–7.4%), and limonene varied from 0.3% to 22.0%, peaking in October [25]. 

This study identified high levels of *α*-pinene and limonene, along with a small amount of *β*-pinene (2.78%). Although seasonality was not examined here, the same compounds were present. The EO composition of E. uniflora has been widely studied using both fresh and dried leaves. The main compounds identified by hydrodistillation of dried leaves generally align with those reported in this study, though their proportions vary across studies. Frequently reported compounds include germacrone, curzerene, germacrene B, caryophyllene oxide, spathulenol, and *α*-selinene [14,21,26]. Additionally, atractylone, a sesquiterpenoid identified here, has been detected in minor amounts in other samples [14,26,27]. 

### 3.2. Insecticidal Activity of the Essential Oils

Among the species tested, only *E. brasiliensis* and *E. uniflora* showed insecticidal activity against adult *C. felis felis*. *E. uniflora* produced higher mortality than *E. brasiliensis* at concentrations of 5000, 10,000, 20,000, and 40,000 µg/mL, with mortality rates of 88.3%, 89.5%, 94.7%, and 95.1%, respectively. By comparison, *E. brasiliensis* produced mortalities of 25.1%, 55.0%, 75.0%, and 80.0% at the same concentrations. Accordingly, the LC_50_ for *E. uniflora* was about 17 times lower than that of *E. brasiliensis* (Table 4), indicating much higher toxicity. These results suggest that *E. uniflora* has greater insecticidal potential than *E. brasiliensis* against adult *C. felis felis*.

Recent studies have highlighted the potential of plant-derived essential oils to control parasite infestations in dogs and cats, including *C. felis felis* (fleas). EOs from *Alpinia zerumbet* B.L. Burtt & R.M. Smith, *Cinnamomum* spp., *Cymbopogon nardus* (L.) Rendle, *Laurus nobilis* L., *Mentha spicata* L., and *Ocimum gratissimum* L. have demonstrated activity against *C. felis felis* across developmental stages, including adults, larvae, and eggs [28]. In these species, 1,8-cineole (eucalyptol) was the major compound in *A. zerumbet*, *O. gratissimum*, and *L. nobilis*, as in *E. brasiliensis*. 1,8-cineole (24.11%), camphor (12.13%), and curzerenone (9.68%) were detected in the EO of fresh *Curcuma zedoaria* (Christm.) Roscoe rhizomes produced 100%, 94.23%, 100%, and 98% mortality against adult fleas, pupal stages, larvae, and eggs of *C. felis felis*, respectively, at concentrations of 800 µg·cm^−2^, 396 µg·cm^−2^, 117.5 µg·cm^−2^, and 396 µg·cm^−2^ [29]. 

The EO from fresh leaves of *Piper aduncum* L., rich in the phenylpropanoid dillapiole (77.56–85.52%), promoted 100% mortality of flea eggs at 100 µg/mL and of adults at 1000 µg/mL [6], a chemical profile not observed in the present study. Ref. [30] demonstrated that the EO from *Baccharis trimera* (Less.) DC. and *Mimosa verrucosa* Benth exhibited strong insecticidal activity against adult fleas, achieving 100% mortality at 800 µg·cm^−2^, with residual activity lasting up to three days and low toxicity (LC_50_ = 369.22 µg·cm^−2^). Interestingly, *M. verrucosa* contains *α*-pinene and (*E*)-caryophyllene as major constituents, compounds also identified in *E. brasiliensis*. The major compounds found in *E. brasiliensis* and *E. uniflora* have previously been associated with antiparasitic activity in other organisms. The compound *(E)*-caryophyllene, isolated from *Ageratum conyzoides* L. and tested orally in cross-bred male calves, was effective against tick species, such as the IVRI-I strain of *Rhipicephalus microplus, R. annulatus,* and *Hyalomma anatolicum* [31].

The EO of *Psidium brownianum* Mart. ex DC. contains isogermafurene (52.93%) and germacrone (16.02%), and the tested oil inhibited the parasites *Leishmania braziliensis* (92.61%), *Leishmania infantum* (84.16%), and *Trypanosoma cruzi* (57.05%) at 1000 µ/mL [32]. Additionally, Pretel et al. [33] synthesized various germacrene-derived compounds, among which 7,11-epoxieudesma-3,7(11)-dien-8-one exhibited insecticidal activity against the aphid Rhopalosiphum padi, a plant parasite, while 1,10-epoxygermacrone showed acaricidal activity against the tick *Hyalomma lusitanicum* and demonstrated greater activity than germacrone. These findings suggest that germacrone, identified in *E. uniflora* in the present study, may also serve as a promising precursor for bioactive derivatives.

The chemical component *α*-pinene has been reported to cause toxicity by fumigation and contact exposure in *Sitophilus zeamais*, with an LC_50_ value of 4.133 ppm after 14 days [34]. This compound has also shown activity against the cattle tick *Rhipicephalus microplus* at high concentrations [35], supporting its potential contribution to the insecticidal activity observed in the present study.

Despite the promising results, the use of essential oils in animals requires caution, as adverse reactions such as depression, incoordination, muscle tremors, pruritus, scratching, and weakness have been reported in pets [36,37]. Therefore, the findings presented here should be considered preliminary, highlighting the need for further toxicological and formulation studies before practical applications involving *Eugenia* essential oils.

## 4. Materials and Methods

Species of the genus *Eugenia* were collected in the state of Paraná, southern Brazil, within the Atlantic Forest biome. Botanical identification was performed, and voucher specimens were deposited in the Herbarium of the State University of Ponta Grossa, as detailed in Appendix A, Table A1. The collections were registered in the National System for the Management of Genetic Heritage and Associated Traditional Knowledge (SisGen) under registration code ADDD35A. Plant material for essential oil extraction was collected between September and December of 2024.

### 4.1. Preparation of Plant Material and Essential Oil Extraction

For the procedures below, the leaves were dried in an oven (LUCA-82, Lucadema^®^, São José do Rio Preto, São Paulo, Brazil) at 26 ± 4 °C and crushed in a blender. They were immediately subjected to essential oil extraction. The EO extraction was performed using a Clevenger apparatus via hydrodistillation for 2 h [38].

### 4.2. Gas Chromatography Coupled with Mass Spectrometry (GC-MS) Analyses

To separate, detect, and quantify the constituents, 1 μL of the volatile oil samples diluted in dichloromethane (10 μL/mL) at the defined times was injected into a gas chromatography (GC) instrument. A Hewlett-Packard 5890 Series II (Palo Alto, CA, USA), equipped with flame ionization detection and a split/splitless injector, in a split ratio of 1:20, was used to separate and detect the constituents in the volatile oil. The compounds were separated with a fused silica capillary column (5% phenyl and 95% dimethylpolysiloxane), with 30 m × 0.25 mm (i.d.) × 0.25 μm (film thickness). Helium was used as the carrier gas at a flow rate of 1 mL/min. The column temperature was programmed as follows: 60 °C for 2 min, then heating at 5 °C/min to 110 °C, at 3 °C/min to 150 °C, and finally at 15 °C/min to 290 °C, with a hold of 15 min. The injector temperature was 220 °C, and the detector temperature was 290 °C. To separate and identify the substances, 1 μL of the volatile oil samples, diluted in dichloromethane (10 μL/mL), was injected at defined times into the gas chromatograph coupled to a mass spectrometer (GC-MS) QP-2010 Plus (Shimadzu, Kyoto, Japan). The flow of the helium gas carrier, the capillary column, and the temperature conditions for the GC-MS analysis were the same as described for the GC. The temperature of the injector was 220 °C and the interface temperature was 250 °C. Mass spectra were obtained with a quadrupole detector operating at 70 eV, with a 40–400 m/z mass range and a scanning rate equal to 0.5 scan/s. The identification of volatile compounds in the volatile oil has been based on linear retention indices (LRI) and mass spectra of the samples, compared with authentic standards injected under the same conditions, with the NIST database (2008). The LRI was calculated based on the co-injection of an alkane series [39,40].

### 4.3. Insecticidal Activity Against Ctenocephalides felis felis

For each replicate, ten unfed adult fleas (five males and five females), 14 days post-emergence from the pupal stage, of the subspecies *Ctenocephalides felis felis* were used. Fleas originated from a laboratory colony maintained on cats, with approval from the Animal Use Ethics Committee (CEUA-IV-UFRRJ), protocol number 4313110419.

EOs of *Eugenia* spp. were diluted in analytical-grade acetone and prepared through a 1:2 serial dilution to obtain a range of ten concentrations from 40,000 to 78.125 µg/mL. For the bioassay, filter paper strips (Whatman No. 1, 80 g; area = 10 cm^2^) were impregnated with 200 µL of each solution. After impregnation, papers were left on the bench for at least 30 min to allow solvent evaporation. Final concentrations corresponded to the mass of EO remaining on the filter paper after acetone evaporation, resulting in a surface concentration range of 800 to 1.5 µg/cm^2^.

Each concentration was tested in six replicates. A negative control consisting of filter paper strips treated with acetone only was included to confirm the absence of vehicle effects. A positive control was performed using fipronil at 8 µg/cm^2^.

Bioassays were conducted to evaluate insecticidal activity by contact exposure of adult fleas. After drying, impregnated filter paper strips were placed inside test tubes, and fleas were introduced. Tubes were maintained in a climatic chamber at 27 ± 1 °C and 70 ± 10% relative humidity for 24 h.

After the exposure period, specimens were examined under a stereomicroscope to determine the biological effect. Mortality was assessed based on motility; individuals showing no movement were considered dead. The mortality percentage was calculated for each concentration according to the formula:

Mortality (%) = 100 × (number of dead individuals/total individuals exposed)

Lethal concentrations causing 50% (LC_50_) and 90% (LC_90_) mortality were estimated by probit analysis. Statistical analyses were performed in RStudio interface (version 4.3.2) (R Core Team) using the ecotoxicoly package, with a 95% confidence interval.

### 4.4. Data Analysis

Principal component analysis was performed using the PAST program, version 3.13.12. The data used for the multivariate analyses were the dependent variables compounds of the EOs and classes such as monoterpene hydrocarbons (MHs), oxygenated monoterpenes (OMs), sesquiterpene hydrocarbons (SHs), oxygenated sesquiterpenes (OSs), and diterpene (DIT), while the independent variables were essential oil samples based on species.

## 5. Conclusions

The findings of this work demonstrate significant chemical variability among EOs obtained from the leaves of the *Eugenia* species studied, with monoterpenes and oxygenated sesquiterpenes as the predominant constituents. Major compounds such as *α*-pinene, (*E*)-caryophyllene, viridiflorene, *β*-selinene, aromadendrene, limonene, and germacrone characterized distinct chemical profiles among species, as confirmed by principal component analysis. Among the evaluated samples, *E. neoverrucosa* presents the highest essential oil yield, while *E. uniflora* exhibited a distinctive composition rich in germacrone-type sesquiterpenoids. In the insecticidal assays against adult *C. felis felis*, *E. uniflora* shows the highest activity, reaching 95.1% mortality at 800 µg·cm^−2^ and presenting the lowest LC_50_ (9.12 µg·cm^−2^), whereas *E. brasiliensis* displays moderate activity; *E. pyriformis*, *E. neoverrucosa*, and *E. myrcianthes* show intermediate activity, and *E. involucrata* demonstrates lower effects. These findings expand the chemical knowledge of the genus and represent the first report of the EO composition of *E. longipedunculata*, and the insecticidal activity of *Eugenia* EOs against *C. felis felis*, highlighting *E. uniflora* as a promising source of natural compounds for ectoparasite control, although further studies on toxicity, active constituents, and formulation are still required.

## Figures and Tables

**Table 1:** Essential oil yield observed in seven species of *Eugenia* genus.

	Mass (g)	Yield (mL)	Yield (%)
*E. brasiliensis*	806.35	2.4	0.30
*E. involucrata*	2577.69	2.3	0.09
*E. longipedunculata*	356.80	1.2	0.34
*E. myrcianthes*	1543.98	1.3	0.08
*E. neoverrucosa*	498.61	4.5	0.90
*E. pyriformis*	2370.23	2.3	0.1
*E. uniflora*	829.65	4.5	0.55

Source: The author, 2026.

**Table 2:** Chemical composition of essential oils from the leaves of *Eugenia* species.

Compounds	LRI	*E.* *brasiliensis*	*E.* *involucrata*	*E.* *longipedunculata*	*E.* *myrcianthes*	*E.* *neoverrucosa*	*E.* *pyriformis*	*E.* *uniflora*	Class
2-Hexanol	796	2.18	2.07	-	1.3	-	3.2	-	AL
(2E)-Hexenal	846	0.73	-	-	0.12	-	-	-	ALD
(3Z)-Hexenol	850	0.67	-	-	-	-	-	0.44	AL
Santene	884	-	-	-	-	-	2.1	-	MH
*α*-Pinene	932	**20.51**	-	7.76	-	**81.9**	**32.94**	-	MH
Camphene	946	-	-	-	-	0.32	-	-	MH
*β*-Pinene	974	-	-	-	-	3.71	2.78	-	MH
Myrcene	988	-	-	-	1.03	0.37	2	-	MH
*δ*-3-Carene (based on RI)	1001	0.96	-	-	-	-	0.58	-	MH
*α*-Phellandrene	1002	-	-	-	-	-	2.8	-	MH
ρ-Cymene	1020	5.9	-	-	-	-	-	-	MH
Limonene	1024	3.14	-	1.42	-	2.67	**24.56**	-	MH
1,8-Cineole	1026	**17.01**	-	5.99	-	-	0.41	-	OM
(Z)*-*β-Ocimene (based on RI)	1032	-	-	-	-	-	3.36	-	MH
α-Campholenal	1122	-	-	-	-	-	6.44	-	MH
γ-Terpinene	1054	2.08	-	1.01	-	0.27	-	-	MH
Isoborneol	1155	-	-	-	-	-	1.87	-	OM
γ-Terpineol	1162	-	-	-	-	3.9	-	-	OM
Borneol	1165	0.72	-	-	-	-	-	-	OM
α-Terpineol	1186	1.25	-	-	-	0.27	-	-	OM
Cubebene	1348	2.38	0.64	-	2.27	0.53	0.6	3.35	SH
Isoledene	1374	-	0.28	-	-	-	-	1.26	SH
α-Copaene	1376	-	-	3.32	-	-	-	-	SH
β-Panasinsene	1381	-	0.96	-	-	-	-	-	SH
β-Elemene	1389	-	3.39	1.55	**9.73**	-	-	1.29	SH
Bornyl acetate	1284	-	-	-	-	0.25	-	-	OM
α-Ylangene	1373	-	-	-	-	1.52	-	-	SH
(E)-Caryophyllene	1417	**17.52**	**25.59**	**19.19**	6.97	4.29	8.35	5.28	SH
α-Guaiene	1437	-	-	-	**16.23**	-	4.02	-	SH
Aromadendrene	1439	-	**18.96**	3.93	-	-	-	-	SH
(*Z*)-β-Farnesene	1440	0.66	-	-	-	-	-	-	SH
cis-Muurola-3.5-diene	1448	1.99	0.66	-	-	-	-	-	SH
Himachalene	1449	-	-	-	-	-	-	0.09	SH
trans-Muurola-3.5-diene	1451	-	-	-	-	-	1	-	SH
*α*-Humulene	1452	2.04	3.03	2.75	3.16	-	-	-	SH
β-Farnesene	1454	-	-	-	-	-	0.1	-	SH
allo-Aromadendrene	1458	-	-	-	0.56	-	2.9	4.58	SH
Dehydroaromadendrane	1460	-	-	4.37	-	-	-	-	SH
9-epi-(E)-Caryophyllene	1464	-	-	1.8	-	-	-	-	SH
γ-Gurjunene	1475	-	-	-	3.1	-	-	-	SH
γ-Muurolene	1478	-	-	-	5.7	-	-	0.39	SH
Germacrene D	1480	0.78	1.16	-	-	-	-	2.76	SH
α-Amorphene	1483	-	-	-	0.22	-	-	0.4	SH
β-Selinene	1489	-	7.36	0.74	**22.88**	-	-	1.01	SH
δ-Selinene	1492	-	-	0.65	-	-	-	-	SH
γ-Amorphene	1495	1.42	-	2.49	1.3	-	-	0.59	SH
Viridiflorene	1496	2.01	**26.32**	**9.31**	-	-	-	-	SH
Curzerene	1499	-	-	-	-	-	-	**11.2**	SH
*α*-Muurolene	1500	-	-	0.89	0.71	-	-	1.45	SH
trans-β-Guaiene	1502	-	-	1.72	-	-	-	-	SH
β-Bisabolene	1505	5.81	0.57	6.83	-	-	-	-	SH
Germacrene A	1508	-	-	-	0.85	-	-	-	SH
δ-Amorphene	1511	-	-	4.4	**12.21**	-	-	7.51	SH
7-epi-α-Selinene	1520	-	-	-	0.17	-	-	-	SH
δ-Cadinene	1522	2.66	3.96	-	-	-	-	-	SH
Zonarene	1529	-	-	0.74	-	-	-	-	SH
Z-Nerolidol (based on RI)	1531	-	-	-	-	-	-	1.57	SH
α-Cadinene	1537	-	-	-	-	-	-	6.36	SH
Selina-3,7(11)-diene	1545	-	-	-	-	-	-	2.98	SH
Spathulenol	1577	4.87	-	1.6	-	-	-	-	OS
Globulol	1590	-	-	3.57	-	-	-	-	OS
Viridiflorol	1592	-	-	2.04	-	-	-	-	OS
Cubeban-11-ol	1595	-	-	0.88	-	-	-	-	OS
Rosifoliol	1600	-	-	1.82	-	-	-	-	OS
1-epi-Cubenol	1627	-	-	1.81	-	-	-	-	OS
Cedranone	1628	-	0.68	-	-	-	-	-	OS
epi-*α*-Muurolol	1640	-	-	1.73	-	-	-	-	OS
α-Cadinol	1652	-	-	-	1.37	-	-	-	OS
neo-Intermedol	1658	-	-	2.65	0.41	-	-	-	OS
Atractylone	1657	-	-	-	-	-	-	**16.08**	OS
Selin-11-en-4-*α*-ol	1658	-	-	-	4.26	-	-		OS
Germacrone	1693	-	-	-	-	-	-	**26.48**	OS
Hydrocarbons		3.58	2.07	0	1.42	0	3.2	0.44	
Monoterpene		30.51	0	1.42	1.03	88.97	67.76	0	
Oxygenated monoterpene		21.06	0	7.0	0	4.44	12.08	0	
Sesquiterpene		2.38	5.27	4.87	12.0	0.53	0.6	5.9	
Oxygenated sesquiterpene		39.76	88.29	75.91	80.1	6.06	15.37	88.73	
Total		97.3	95.6	97.0	94.6	100	100	95.1	

Footnote: -: not detected; bold: major components; Al: alcohol; ADL: aldehyde; LRI: Linear Retention Index; MHs: monoterpene hydrocarbons; OMs: oxygenated monoterpenes; OSs: oxygenated sesquiterpenes; SHs: sesquiterpene hydrocarbons.

**Table 3:** Insecticidal activity (%) of essential oils from *Eugenia* spp. against adults of *Ctenocephalides felis felis*.

Concentration (µg·cm^−2^)	*E.* *brasiliensis*	*E.* *involucrata*	*E.* *longipedunculata*	*E.* *myrcianthes*	*E.* *neoverrucosa*	*E.* *pyriformis*	*E.* *uniflora*
1.5	0.0	0.0	0.0	0.0	0.0	20.0	5.3
3	0.0	0.0	0.0	4.8	9.5	55.0	20.0
6	15.0	5.3	5.6	9.5	17.6	9.5	21.1
12	17.0	0.0	10.0	4.8	19.4	16.7	47.3
25	19.2	5.3	10.6	15.0	20.0	13.6	55.0
50	23.8	5.0	42.2	28.6	23.8	10.0	70.6
100	25.1	19.0	38.9	42.1	24.3	45.0	88.3
200	55.0	31.6	65.3	63.2	22.2	31.6	89.5
400	75.0	31.6	60.0	35.0	25.5	42.9	94.7
800	80.0	38.9	65.3	50.0	52.4	60.0	95.1

Note: The positive control resulted in 100% mortality in all bioassays, while the negative control produced no mortality.

**Table 4:** Lethal concentration (LC_50_) values of essential oils from *Eugenia* uniflora and *Eugenia brasiliensis* obtained by probit analysis.

Essential Oil	LC_50_ (µg/cm^2^)	95% CI (Lower–Upper)	Slope ± SE	R^2^	χ^2^	*p*-Value
*E. uniflora*	9.12	6.59–12.17	1.19 ± 3.86	0.911	13.240	1.000
*E. brasiliensis*	157.82	116.16–225.18	1.09 ± 2.59	0.890	21.770	0.999

## Data Availability

Data are contained within the article and Supplemental Material.

## Supplementary Materials

The following supporting information can be downloaded at: https://www.mdpi.com/article/10.3390/plants15091406/s1, Figure S1, Loadings calculated for the multivariate analysis of essential oils from *Eugenia* species.

## Abbreviations

The following abbreviations are used in this manuscript:

AL	alcohol
ALD	aldehyde
DIT	diterpene
EOs	essential oils
LRI	Linear Retention Index
MHs	monoterpene hydrocarbons
GC-MS	gas chromatography–mass spectrometry
OMs	oxygenated monoterpenes
OSs	oxygenated sesquiterpenes
PCA	Principal component analysis
SH	sesquiterpenes hydrocarbons

## Appendix A

**Table A1:** Data collection of the *Eugenia* species.

Species	Popular Name	Site of Collection	Coordinates	Register
*E. brasiliensis*	grumixama, grumixameira ibaropoti, cumbixaba	Ponta Grossa	25°07′30.2″ S 50°09′25.5″ W	HUPG23392
*E. involucrata*	cereja-do-mato, cerejeira-do-mato, cereja-do-rio-grande	Ponta Grossa	25°05′42.0″ S 50°09′42.8″ W	HUPG 2824
*E. longipedunculata*	pitanga-laranja, grumixama-mirim, grumixama-da-mata, grumixama-miúda	Ponta Grossa	25°07′30.2″ S 50°09′25.5″ W	HUEPG23518
*E. myrcianthes*	pessegueiro-do-mato, ubajaí,	Carambeí	24°54′35.9″ S 50°09′41.5″ W	HUEPG23349
*E. neoverrucosa*	ibirubá, guamirim-ripa, ibacurú, araçá-ripa	Ponta Grossa	25°15′10.8″ S 50°00′06.1″ W	HUEPG23391
*E. pyriformis*	uvaia, uvalha, uvaieira, uvalheira	Carambeí	25°07′30.2″ S 50°09′25.5″ W	HUEPG23346
*E. uniflora*	pitanga, pitangueira	Ponta Grossa	25°07′30.2″ S 50°09′25.5″ W	HUEPG23347
