# Supplementary material for: Unveiling Chemical Profile and Insecticidal Potential of Essential Oils from Leaves of Seven Eugenia L. Species (Myrtaceae)
Source: Plants (Basel). 2026 May 5;15(9):1406. doi: 10.3390/plants15091406 (PMC13165059; doi:10.3390/plants15091406)

UNIVERSIDADE FERAL RURAL DO RIO DE JANEIRO
INSTITUTO DE QUÍMICA

CENTRAL ANALÍTICA MULTIUSUÁRIO
INSTITUTO DE QUÍMICA · UFRRJ

CGMS

Analyzed by: Cristiane Cardoso
Analyzed: 17/6/2025
Solicitante:Douglas
Sample Name: C7_C30 170625
Injection Volume: 1,0 uL   Solvente: Diclorometano
Data File: C:\GCMSsolution\Data\Project1\Douglas\2025\MLLENA\170625\C7_C30 170625.qgd
Method File: C:\GCMSsolution\Data\Project1\Douglas\Essencial Adams-Inj.qgm
EQUIPAMENTO: Modelo:GCMS-QP2010 Plus (Shimadzu)
Coluna: VF-5m (30X0.25X0.25)

Chromatogram C7_C30 170625 C:\GCMSsolution\Data\Project1\Douglas\2025\MLLENA\170625\C7_C30 170625.qgd

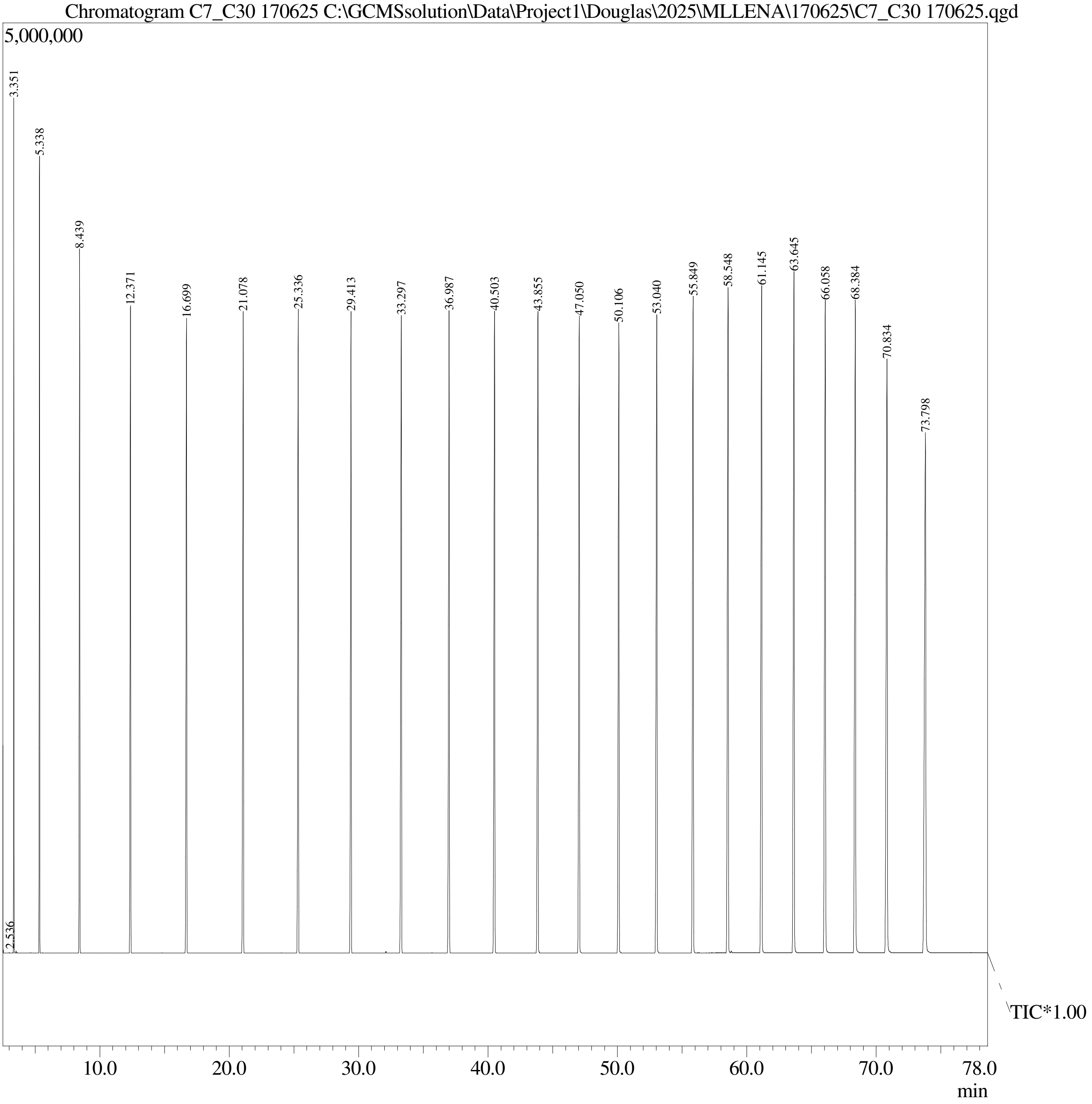

Library

<< Target >>
Line#:1   R.Time:2.533(Scan#:5) MassPeaks:10
RawMode:Averaged 2.525-2.542(4-6) BasePeak:55.00(3490)
BG Mode:Calc. from Peak Group 1 - Event 1 Scan

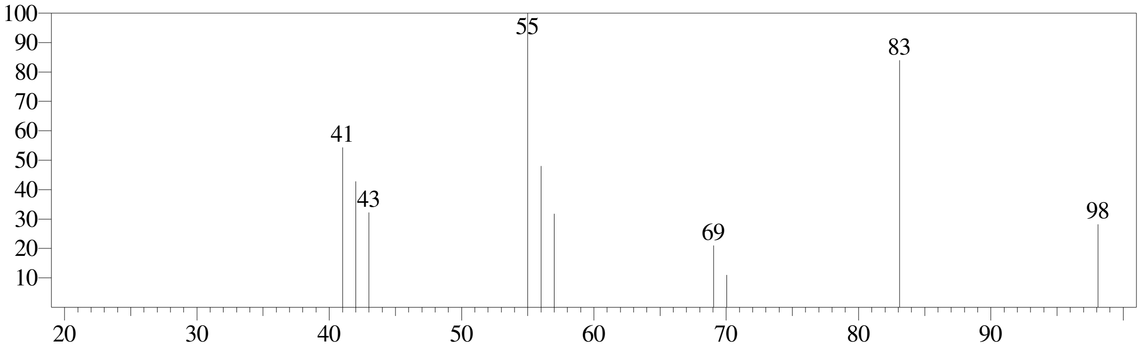

Hit#:1 Entry:2017 Library:NIST23s.lib
SI:87 Formula:C7H14 CAS:762-63-0 MolWeight:98 RetIndex:626
CompName:2-Pentene, 4,4-dimethyl-, (Z)- $$ (Z)-4,4-Dimethyl-2-pentene $$ cis-4,4-Dimethyl-2-Pentene $$ (Z)-(CH3)3C

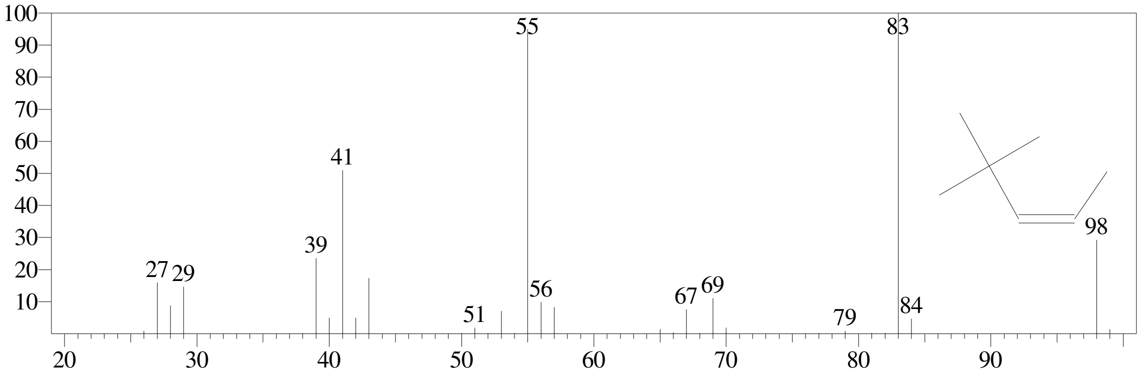

Hit#:2 Entry:2020 Library:NIST23s.lib
SI:86 Formula:C7H14 CAS:108-87-2 MolWeight:98 RetIndex:734
CompName:Cyclohexane, methyl- $$ Cyclohexylmethane $$ Hexahydrotoluene $$ Methylcyclohexane $$ Sextone B $$

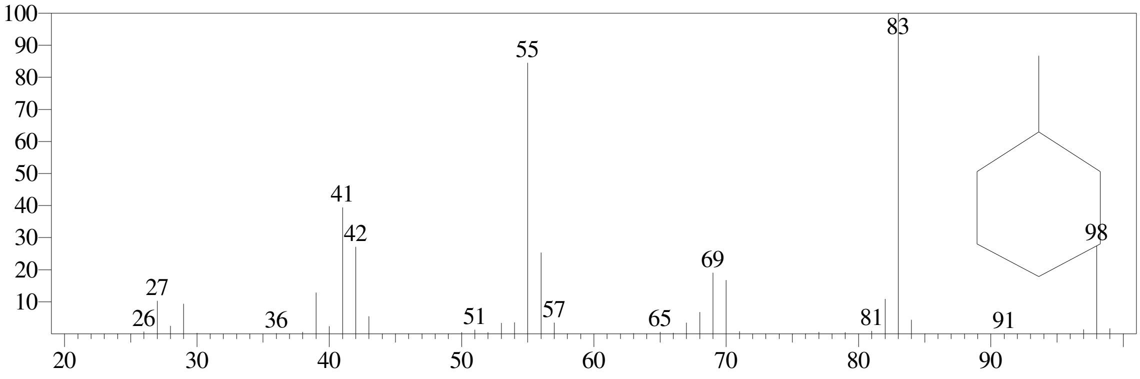

<< Target >>
Line#:1   R.Time:2.533(Scan#:5) MassPeaks:10
RawMode:Averaged 2.525-2.542(4-6) BasePeak:55.00(3490)
BG Mode:Calc. from Peak Group 1 - Event 1 Scan

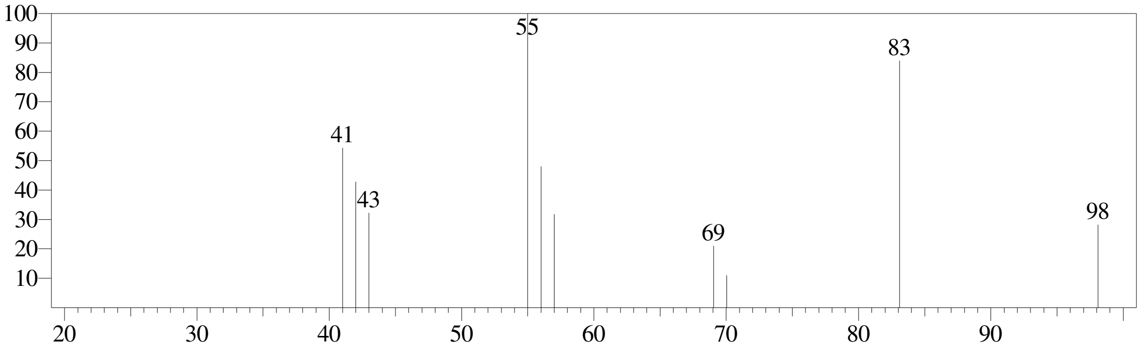

Hit#:3 Entry:1983 Library:NIST23s.lib
SI:86 Formula:C7H14 CAS:4127-47-3 MolWeight:98 RetIndex:626
CompName:Cyclopropane, 1,1,2,2-tetramethyl- $$ 1,1,2,2-Tetramethylcyclopropane $$

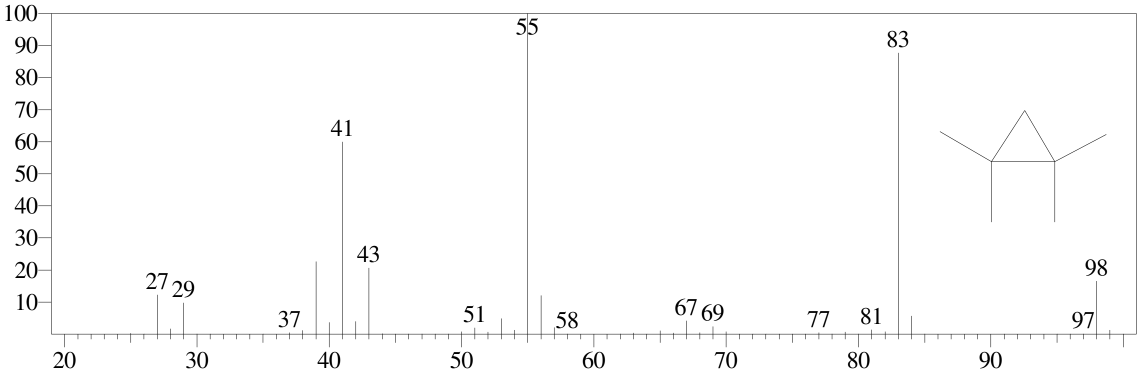

Hit#:4 Entry:1973 Library:NIST23s.lib
SI:85 Formula:C7H14 CAS:74752-93-5 MolWeight:98 RetIndex:660
CompName:Cyclopropane, 1,1,2,3-tetramethyl- $$ 1,1,2,3-Tetramethylcyclopropane # $$

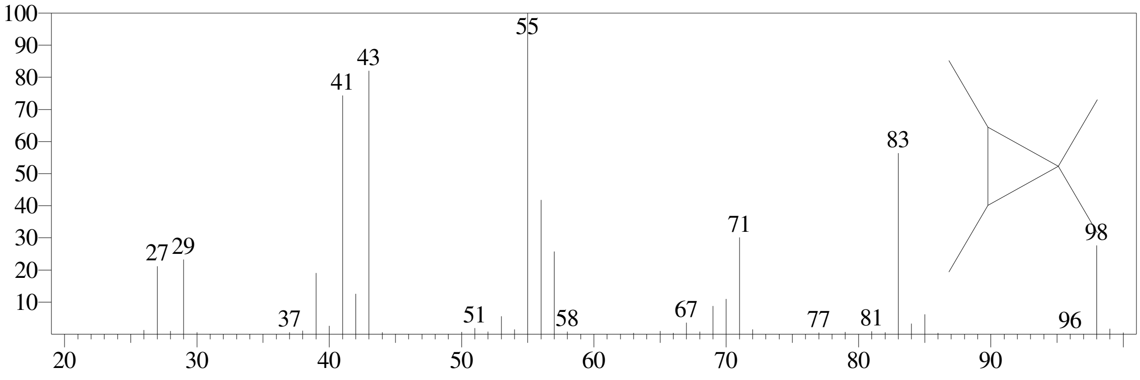

<< Target >>
Line#:1 R.Time:2.533(Scan#:5) MassPeaks:10
RawMode:Averaged 2.525-2.542(4-6) BasePeak:55.00(3490)
BG Mode:Calc. from Peak Group 1 - Event 1 Scan

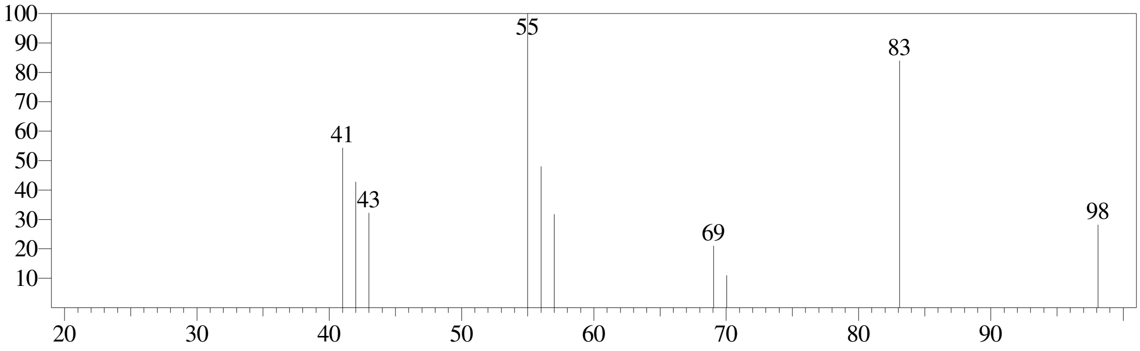

Hit#:5 Entry:1977 Library:NIST23s.lib
SI:85 Formula:C7H14 CAS:24910-63-2 MolWeight:98 RetIndex:672
CompName:2-Pentene, 3,4-dimethyl- $$ 3,4-Dimethyl-2-pentene $$

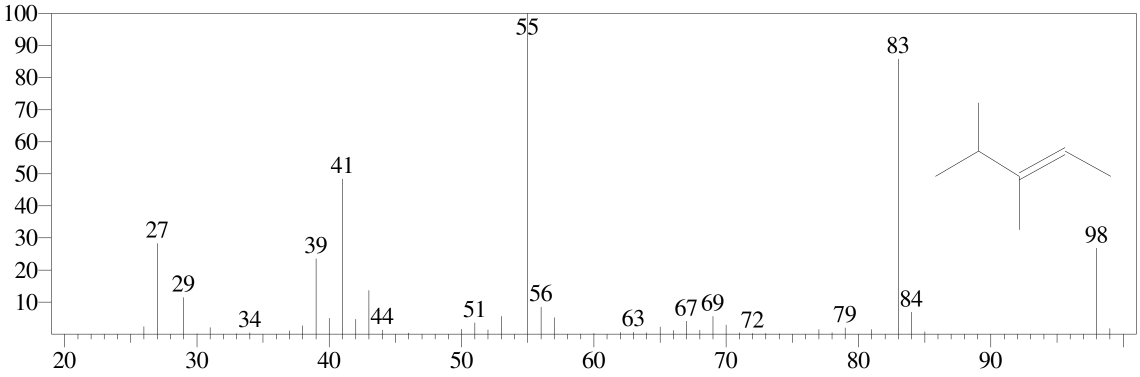

<< Target >>
Line#:2   R.Time:3.350(Scan#:103) MassPeaks:31
RawMode:Averaged 3.342-3.358(102-104) BasePeak:43.05(1175554)
BG Mode:None Group 1 - Event 1 Scan

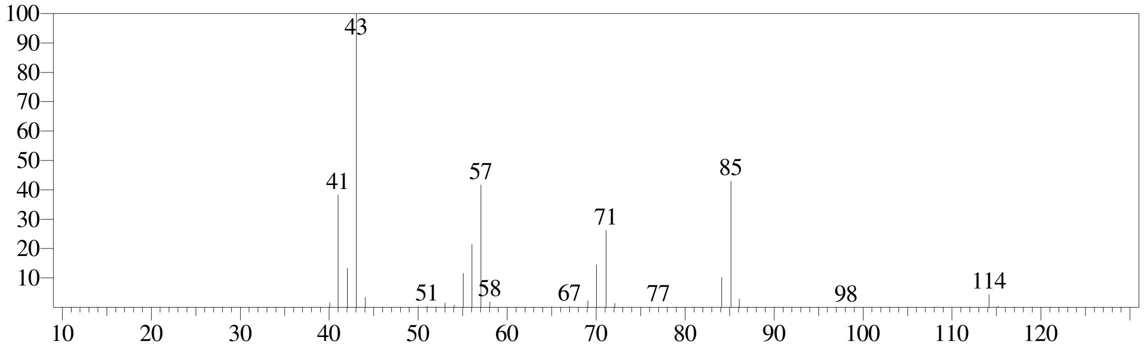

Hit#:1 Entry:4224 Library:NIST23s.lib
SI:97 Formula:C8H18 CAS:111-65-9 MolWeight:114 RetIndex:793
CompName:Octane $$ n-Octane $$ n-C8H18 $$ Oktan $$ Oktanen $$ Ottani $$ UN 1262 $$

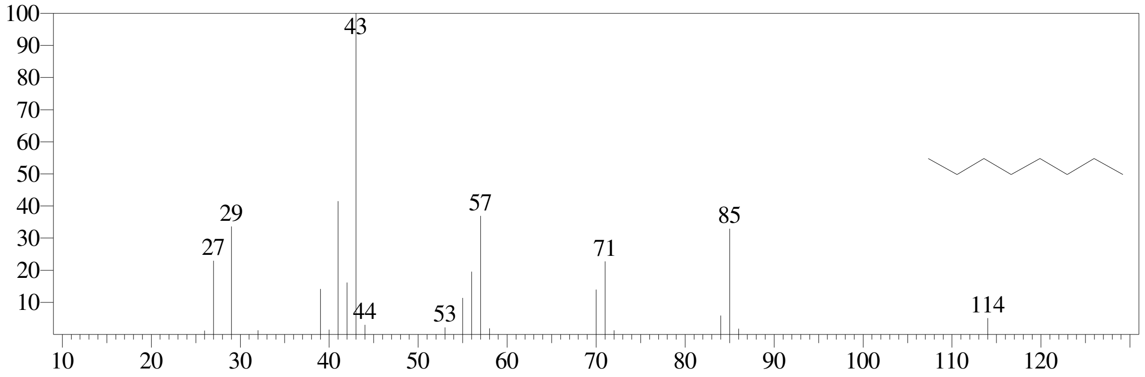

Hit#:2 Entry:4247 Library:NIST23s.lib
SI:97 Formula:C8H18 CAS:111-65-9 MolWeight:114 RetIndex:793
CompName:Octane $$ n-Octane $$ n-C8H18 $$ Oktan $$ Oktanen $$ Ottani $$ UN 1262 $$

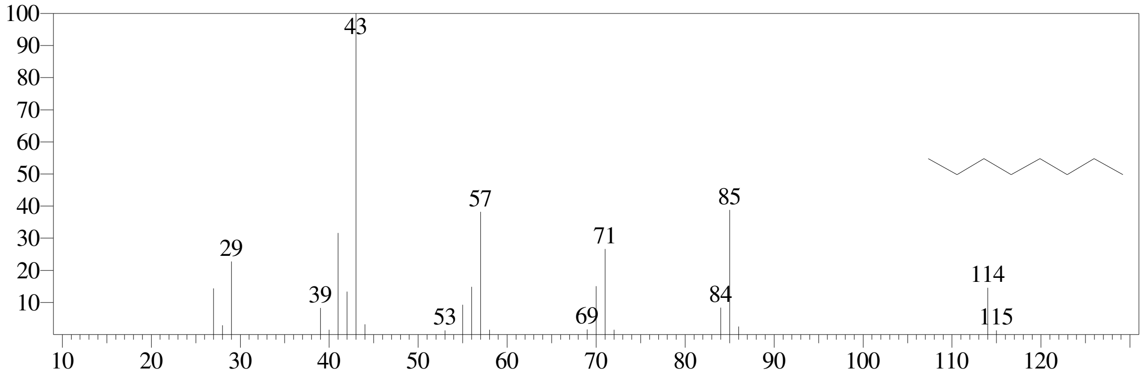

<< Target >>
Line#:2   R.Time:3.350(Scan#:103) MassPeaks:31
RawMode:Averaged 3.342-3.358(102-104) BasePeak:43.05(1175554)
BG Mode:None Group 1 - Event 1 Scan

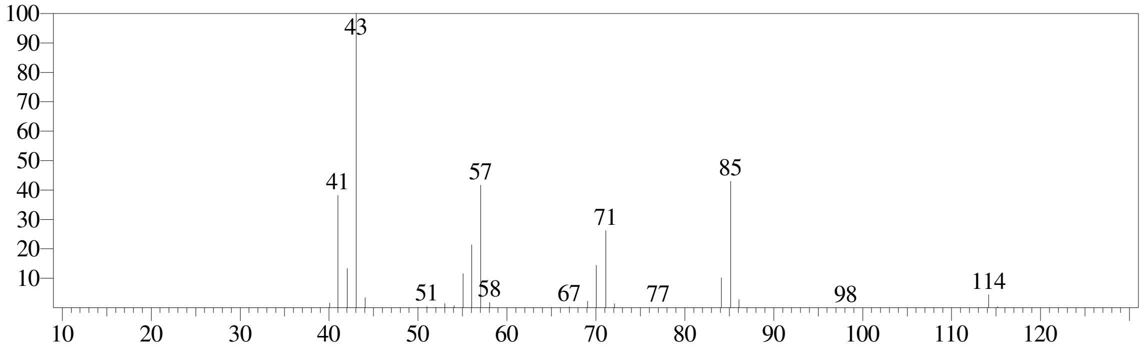

Hit#:3 Entry:4706 Library:NIST23-1.lib
SI:96 Formula:C8H18 CAS:111-65-9 MolWeight:114 RetIndex:793
CompName:Octane $$ n-Octane $$ n-C8H18 $$ Oktan $$ Oktanen $$ Ottani $$ UN 1262 $$

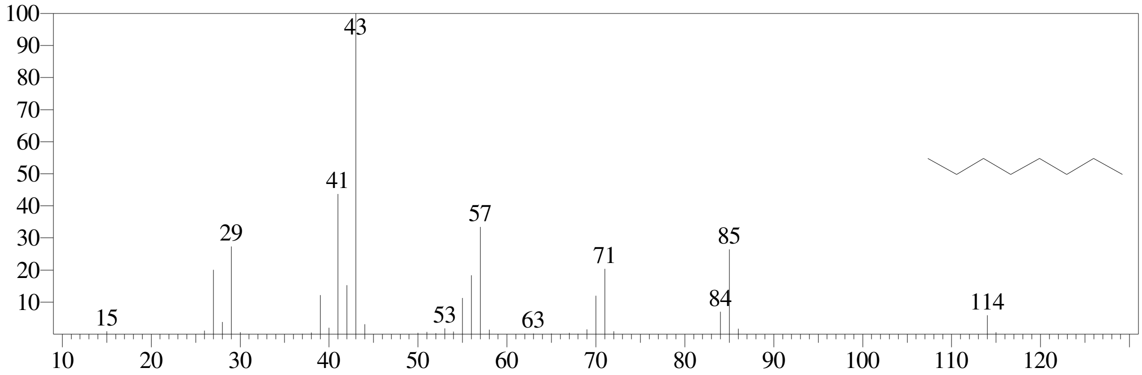

Hit#:4 Entry:4225 Library:NIST23s.lib
SI:96 Formula:C8H18 CAS:111-65-9 MolWeight:114 RetIndex:793
CompName:Octane $$ n-Octane $$ n-C8H18 $$ Oktan $$ Oktanen $$ Ottani $$ UN 1262 $$

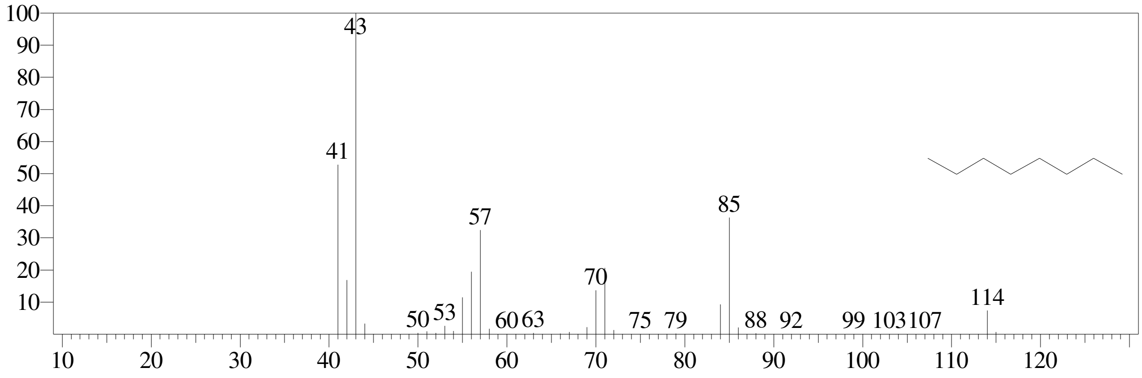

<< Target >>
Line#:2   R.Time:3.350(Scan#:103) MassPeaks:31
RawMode:Averaged 3.342-3.358(102-104) BasePeak:43.05(1175554)
BG Mode:None Group 1 - Event 1 Scan

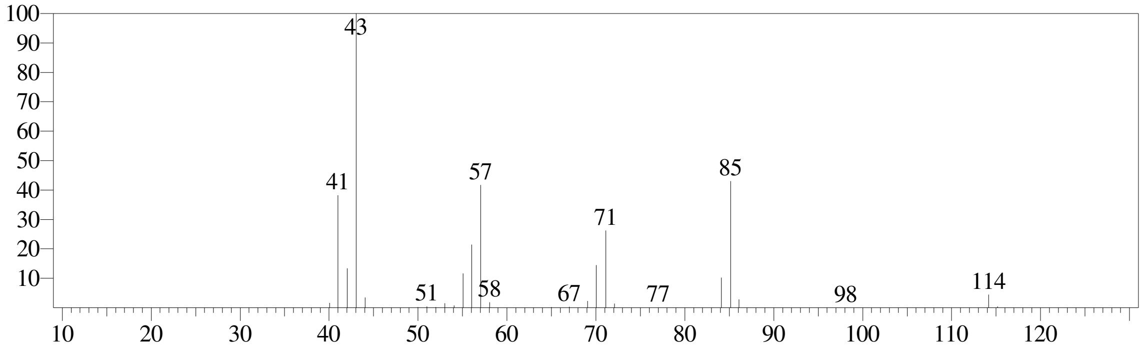

Hit#:5 Entry:8543 Library:NIST23-1.lib
SI:94 Formula:C9H20 CAS:2213-23-2 MolWeight:128 RetIndex:823
CompName:Heptane, 2,4-dimethyl- $$ 2,4-Dimethylheptane $$

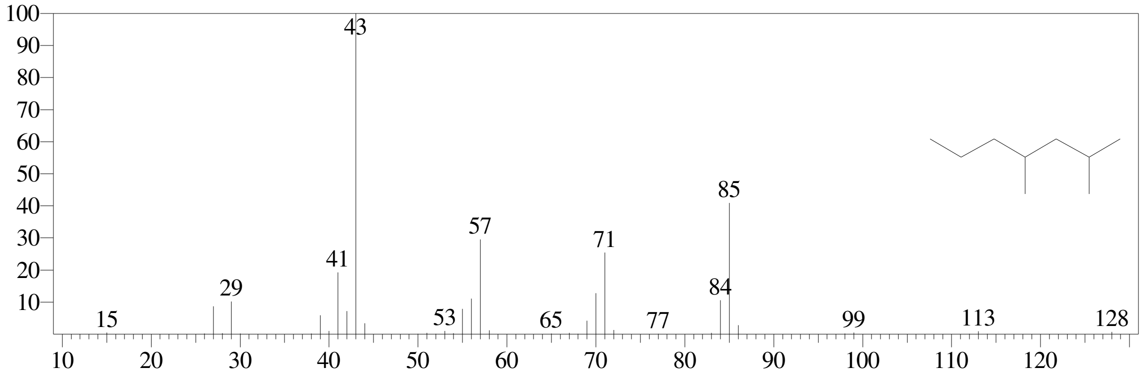

<< Target >>
Line#:3 R.Time:5.342(Scan#:342) MassPeaks:32
RawMode:Averaged 5.333-5.350(341-343) BasePeak:43.05(990160)
BG Mode:None Group 1 - Event 1 Scan

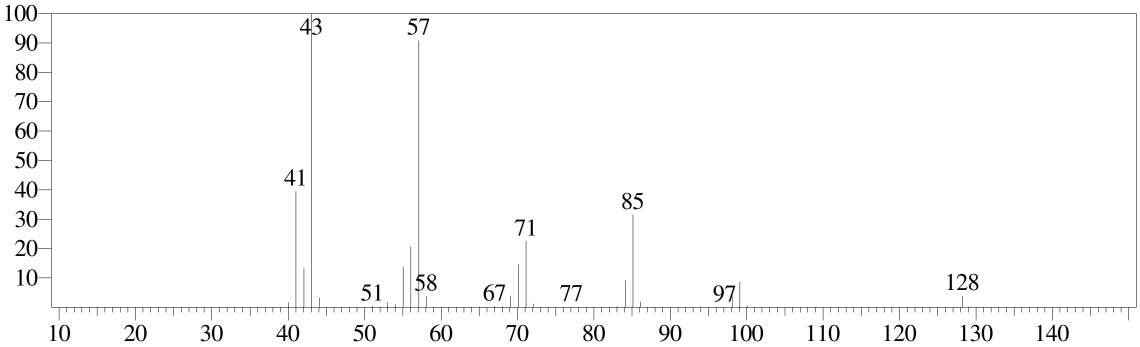

Hit#:1 Entry:8535 Library:NIST23-1.lib
SI:97 Formula:C9H20 CAS:111-84-2 MolWeight:128 RetIndex:894
CompName:Nonane $$ n-Nonane $$ Shellsol 140 $$ n-C9H20 $$ UN 1920 $$

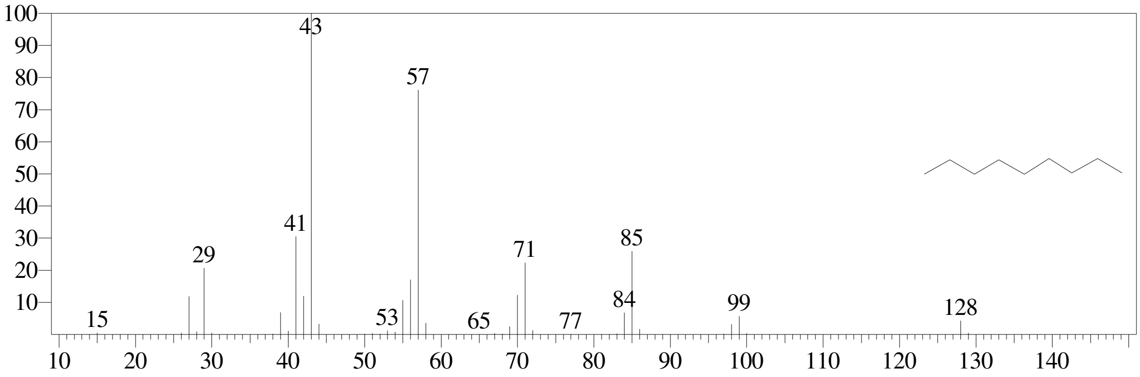

Hit#:2 Entry:6663 Library:NIST23s.lib
SI:96 Formula:C9H20 CAS:111-84-2 MolWeight:128 RetIndex:894
CompName:Nonane $$ n-Nonane $$ Shellsol 140 $$ n-C9H20 $$ UN 1920 $$

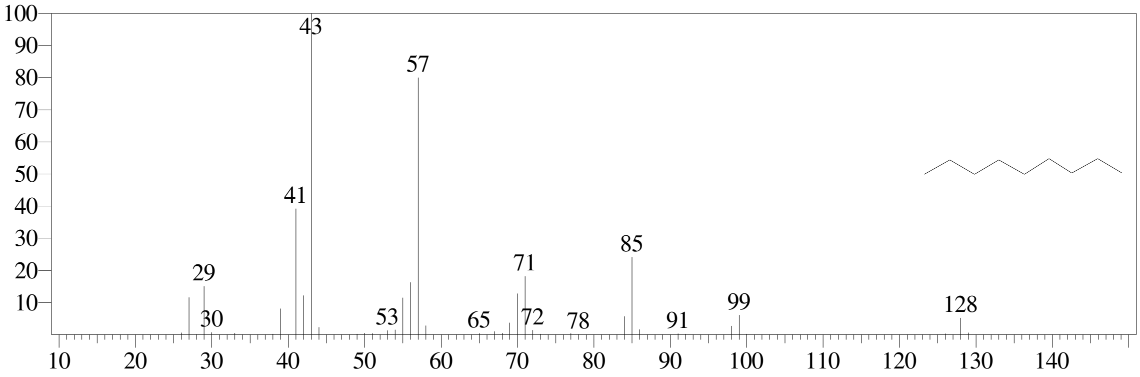

<< Target >>
Line#:3 R.Time:5.342(Scan#:342) MassPeaks:32
RawMode:Averaged 5.333-5.350(341-343) BasePeak:43.05(990160)
BG Mode:None Group 1 - Event 1 Scan

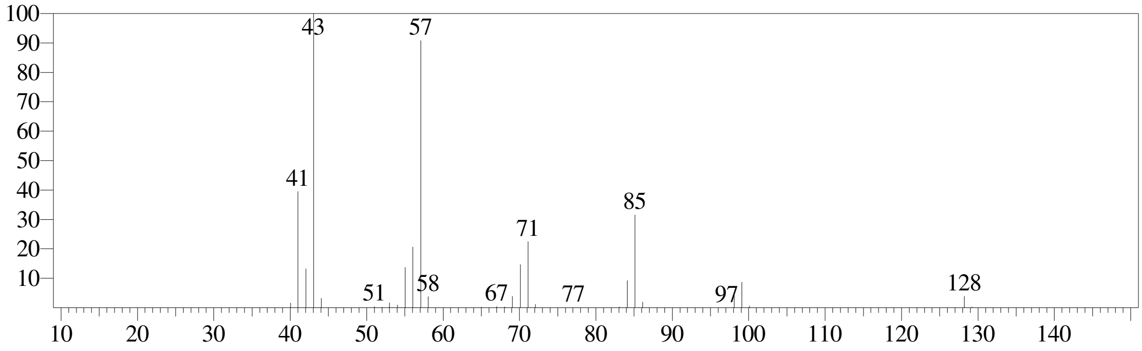

Hit#:3 Entry:6665 Library:NIST23s.lib
SI:96 Formula:C9H20 CAS:111-84-2 MolWeight:128 RetIndex:894
CompName:Nonane $$ n-Nonane $$ Shellsol 140 $$ n-C9H20 $$ UN 1920 $$

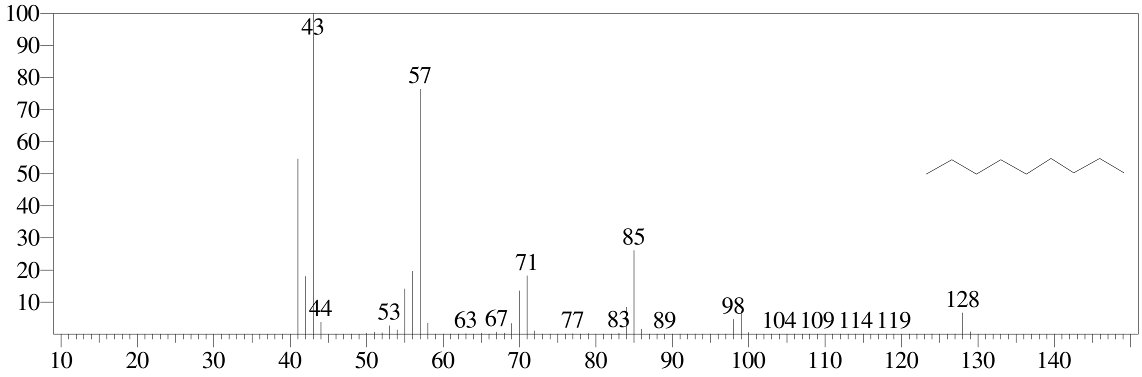

Hit#:4 Entry:6662 Library:NIST23s.lib
SI:96 Formula:C9H20 CAS:111-84-2 MolWeight:128 RetIndex:894
CompName:Nonane $$ n-Nonane $$ Shellsol 140 $$ n-C9H20 $$ UN 1920 $$

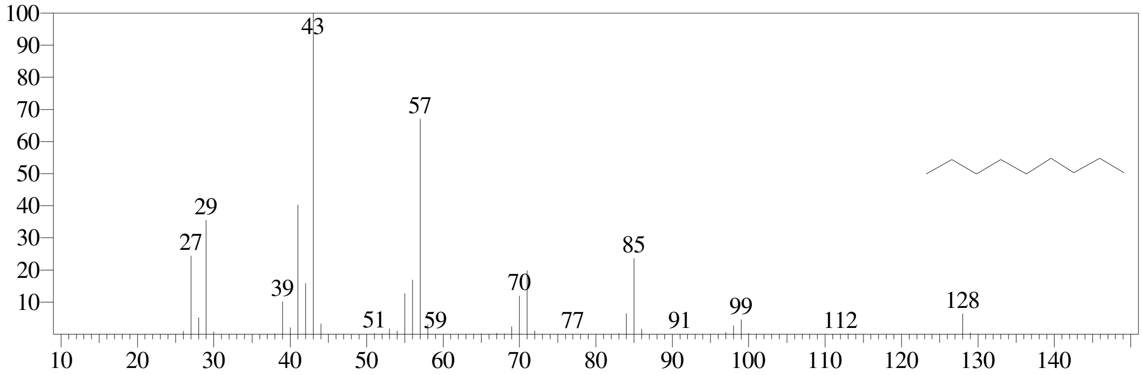

<< Target >>
Line#:3 R.Time:5.342(Scan#:342) MassPeaks:32
RawMode:Averaged 5.333-5.350(341-343) BasePeak:43.05(990160)
BG Mode:None Group 1 - Event 1 Scan

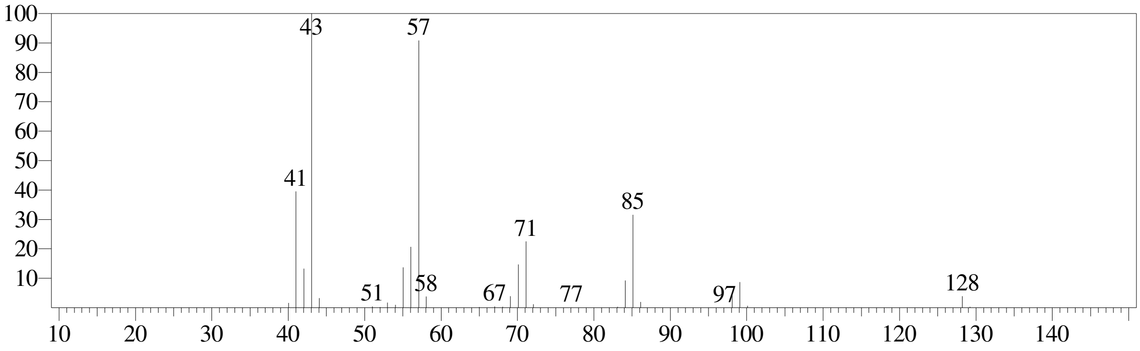

Hit#:5 Entry:9829 Library:NIST23s.lib
SI:95 Formula:C10H22 CAS:124-18-5 MolWeight:142 RetIndex:994
CompName:Decane $$ n-Decane $$ n-C10H22 $$ UN 2247 $$

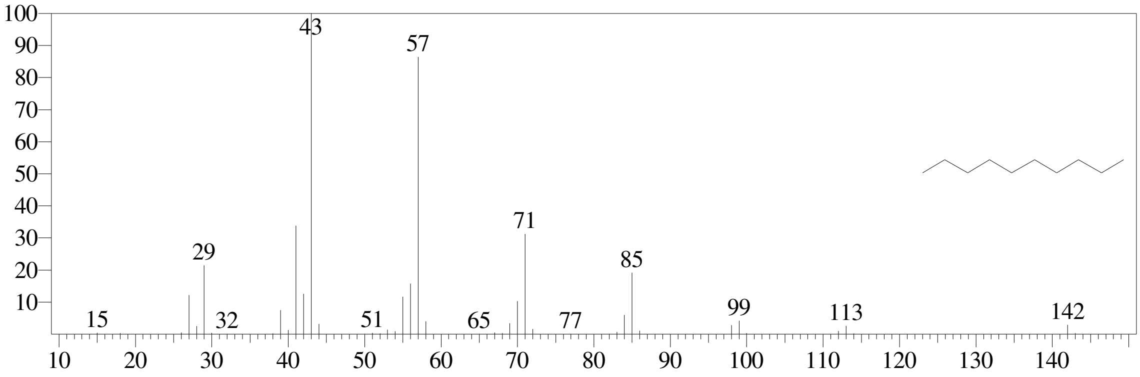

<< Target >>
Line#:4 R.Time:8.442(Scan#:714) MassPeaks:36
RawMode:Averaged 8.433-8.450(713-715) BasePeak:57.10(894452)
BG Mode:None Group 1 - Event 1 Scan

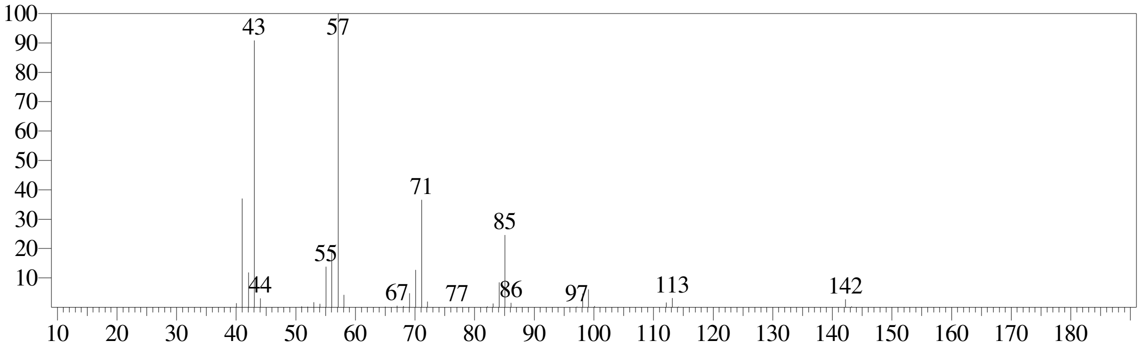

Hit#:1 Entry:9829 Library:NIST23s.lib
SI:96 Formula:C10H22 CAS:124-18-5 MolWeight:142 RetIndex:994
CompName:Decane $$ n-Decane $$ n-C10H22 $$ UN 2247 $$

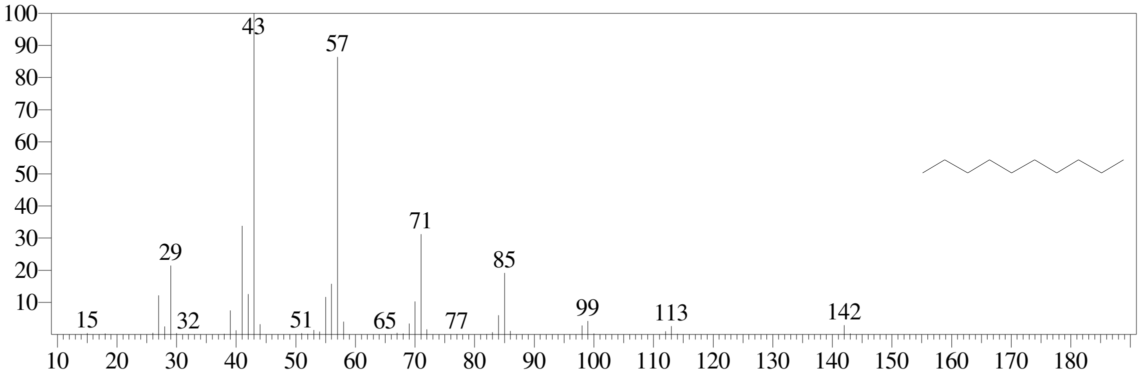

Hit#:2 Entry:22562 Library:NIST23-1.lib
SI:96 Formula:C11H24 CAS:1120-21-4 MolWeight:156 RetIndex:1094
CompName:Undecane $$ n-Undecane $$ Hendecane $$ n-C11H24 $$ UN 2330 $$

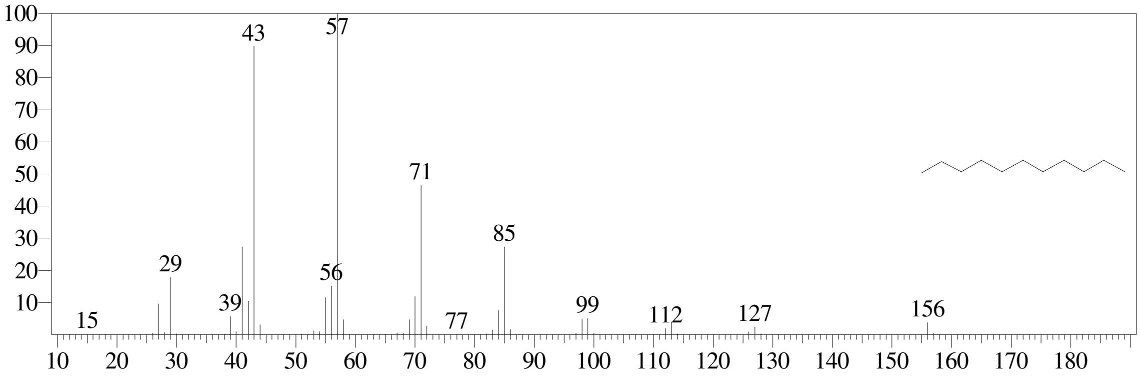

<< Target >>
Line#:4   R.Time:8.442(Scan#:714) MassPeaks:36
RawMode:Averaged 8.433-8.450(713-715) BasePeak:57.10(894452)
BG Mode:None Group 1 - Event 1 Scan

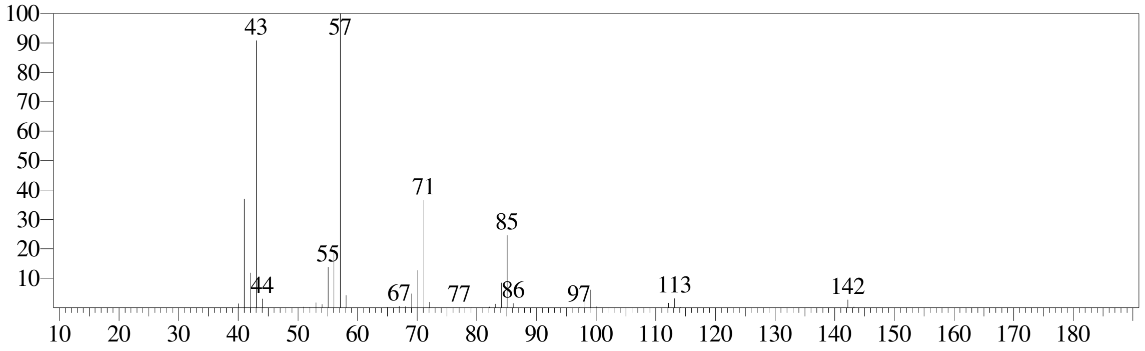

Hit#:3 Entry:20394 Library:NIST23s.lib
SI:96 Formula:C13H28 CAS:629-50-5 MolWeight:184 RetIndex:1293
CompName:Tridecane $$ n-Tridecane $$ Tridecane, n- $$

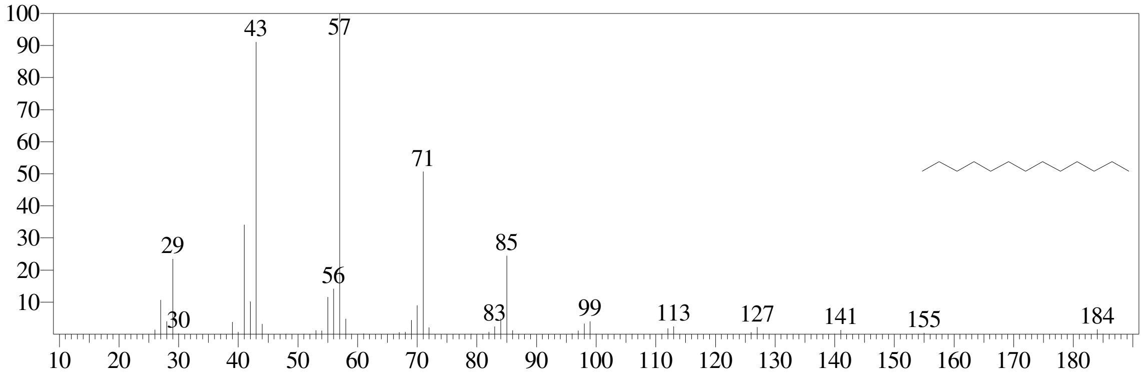

Hit#:4 Entry:9828 Library:NIST23s.lib
SI:96 Formula:C10H22 CAS:124-18-5 MolWeight:142 RetIndex:994
CompName:Decane $$ n-Decane $$ n-C10H22 $$ UN 2247 $$

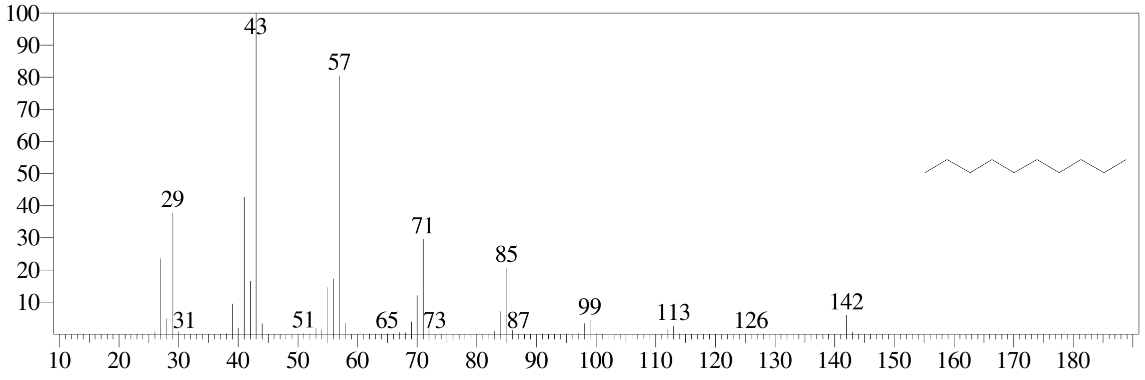

<< Target >>
Line#:4   R.Time:8.442(Scan#:714) MassPeaks:36
RawMode:Averaged 8.433-8.450(713-715) BasePeak:57.10(894452)
BG Mode:None Group 1 - Event 1 Scan

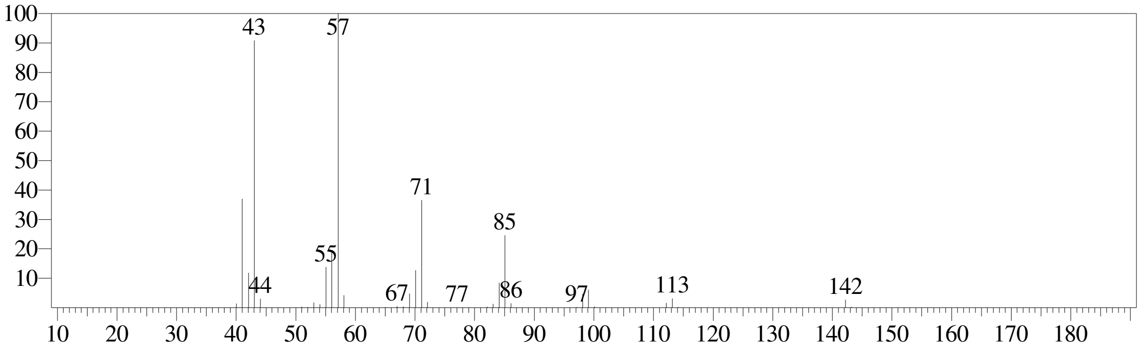

Hit#:5 Entry:9831 Library:NIST23s.lib
SI:96 Formula:C10H22 CAS:124-18-5 MolWeight:142 RetIndex:994
CompName:Decane $$ n-Decane $$ n-C10H22 $$ UN 2247 $$

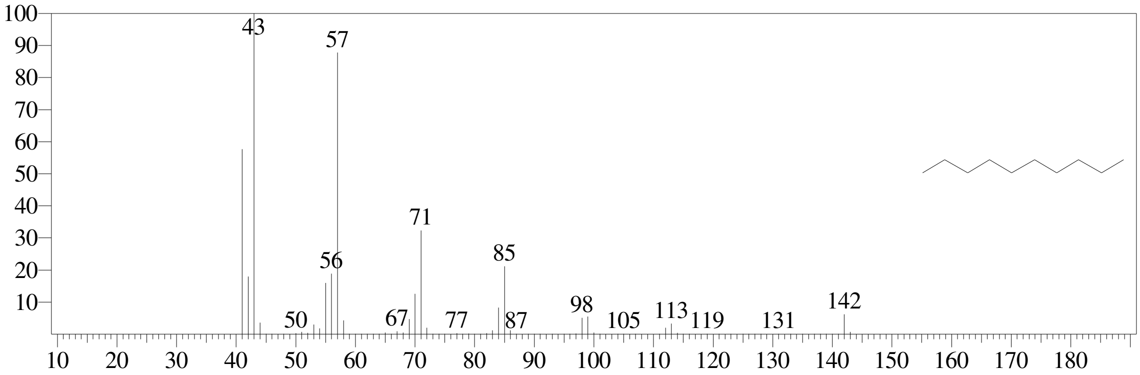

<< Target >>
Line#:5   R.Time:12.375(Scan#:1186) MassPeaks:39
RawMode:Averaged 12.367-12.383(1185-1187) BasePeak:57.05(835904)
BG Mode:None Group 1 - Event 1 Scan

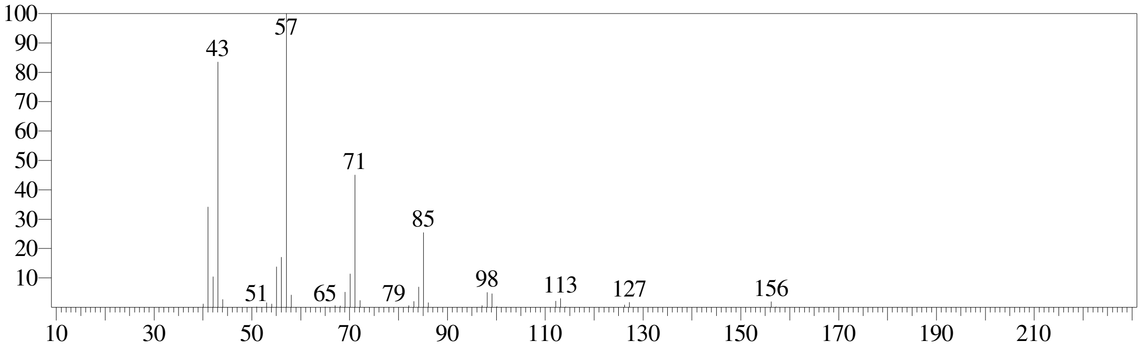

Hit#:1 Entry:22562 Library:NIST23-1.lib
SI:98 Formula:C11H24 CAS:1120-21-4 MolWeight:156 RetIndex:1094
CompName:Undecane $$ n-Undecane $$ Hendecane $$ n-C11H24 $$ UN 2330 $$

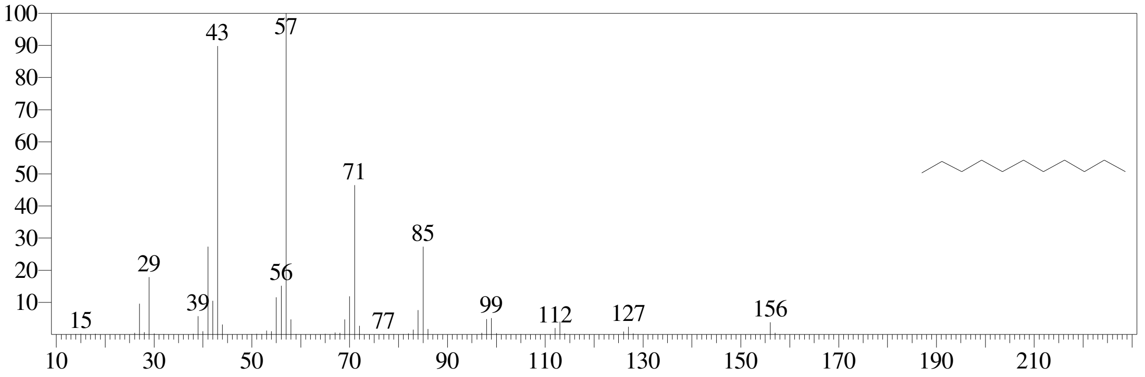

Hit#:2 Entry:20394 Library:NIST23s.lib
SI:97 Formula:C13H28 CAS:629-50-5 MolWeight:184 RetIndex:1293
CompName:Tridecane $$ n-Tridecane $$ Tridecane, n- $$

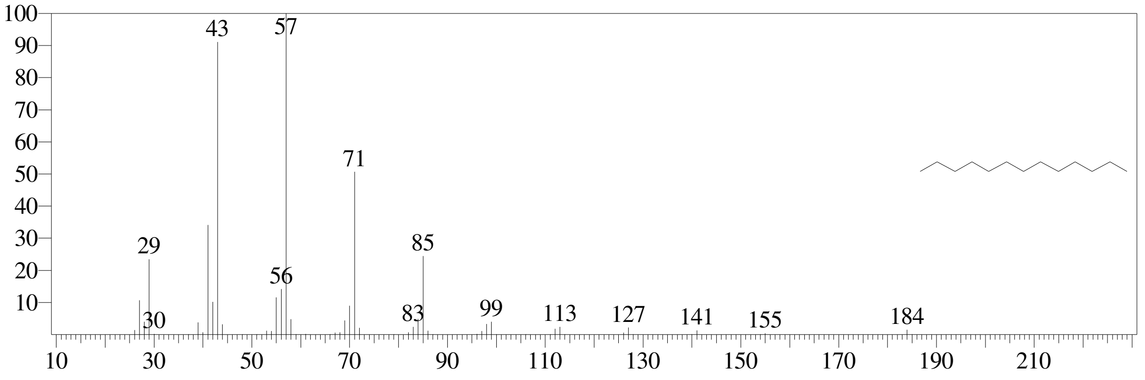

<< Target >>
Line#:5 R.Time:12.375(Scan#:1186) MassPeaks:39
RawMode:Averaged 12.367-12.383(1185-1187) BasePeak:57.05(835904)
BG Mode:None Group 1 - Event 1 Scan

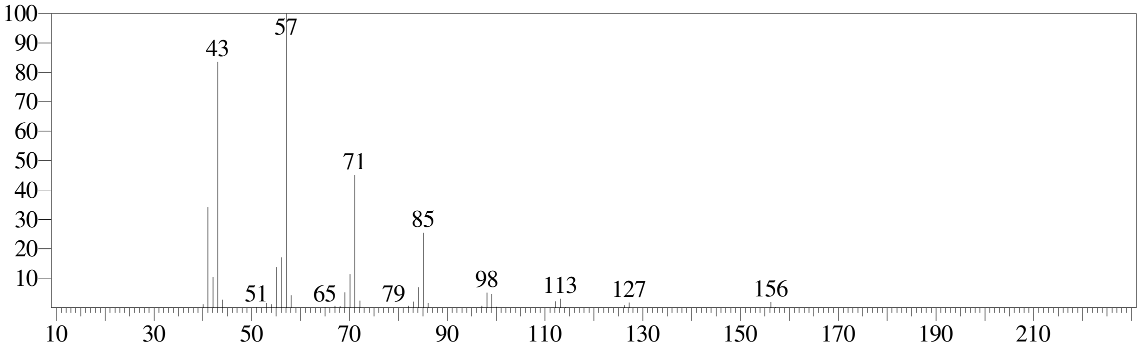

Hit#:3 Entry:13547 Library:NIST23s.lib
SI:97 Formula:C11H24 CAS:1120-21-4 MolWeight:156 RetIndex:1094
CompName:Undecane $$ n-Undecane $$ Hendecane $$ n-C11H24 $$ UN 2330 $$

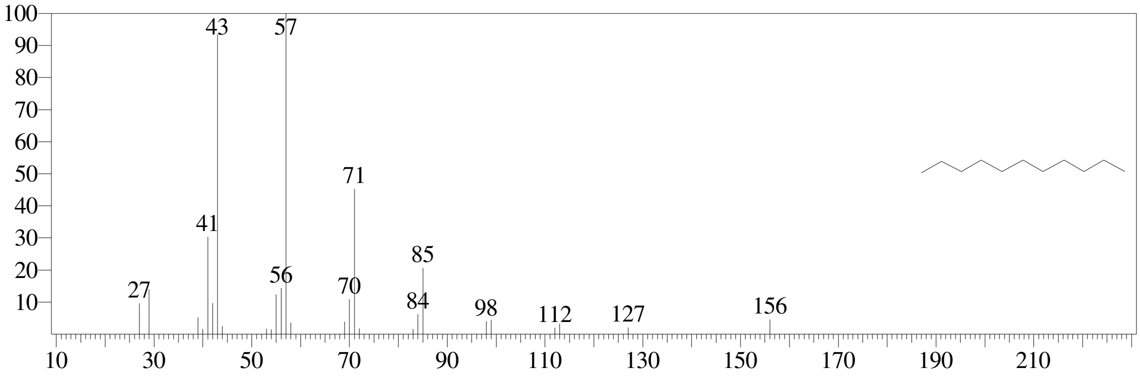

Hit#:4 Entry:13544 Library:NIST23s.lib
SI:96 Formula:C11H24 CAS:1120-21-4 MolWeight:156 RetIndex:1094
CompName:Undecane $$ n-Undecane $$ Hendecane $$ n-C11H24 $$ UN 2330 $$

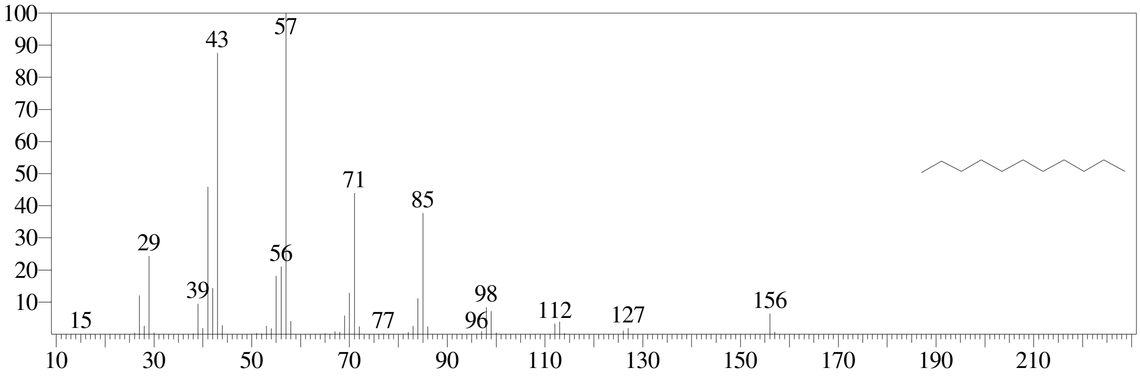

<< Target >>
Line#:5 R.Time:12.375(Scan#:1186) MassPeaks:39
RawMode:Averaged 12.367-12.383(1185-1187) BasePeak:57.05(835904)
BG Mode:None Group 1 - Event 1 Scan

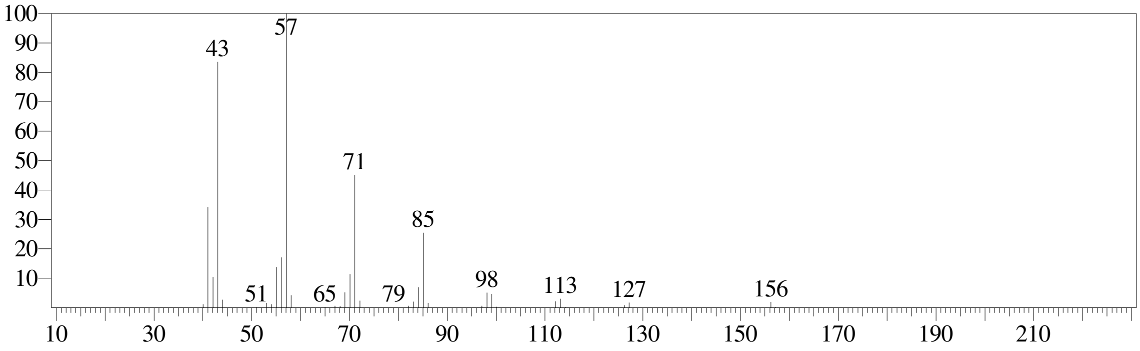

Hit#:5 Entry:29528 Library:NIST23s.lib
SI:96 Formula:C16H34 CAS:544-76-3 MolWeight:226 RetIndex:1599
CompName:Hexadecane $$ n-Cetane $$ n-Hexadecane $$ Cetane $$

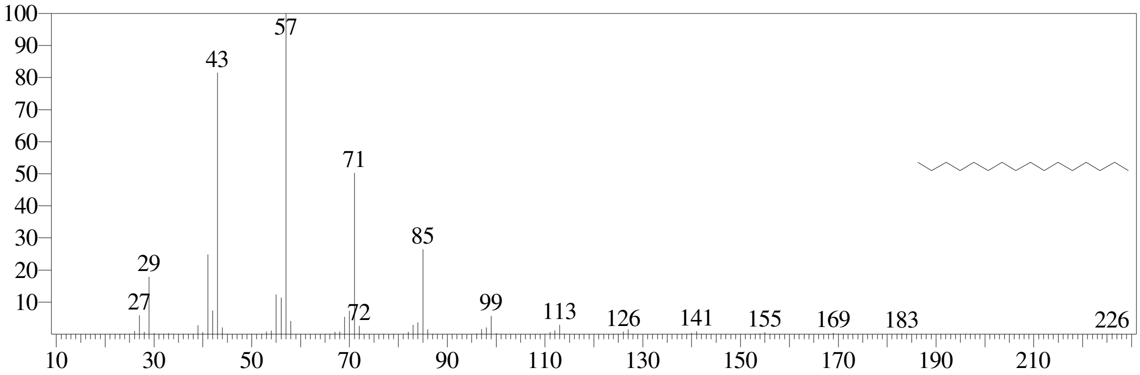

<< Target >>
Line#:6   R.Time:16.700(Scan#:1705) MassPeaks:43
RawMode:Averaged 16.692-16.708(1704-1706) BasePeak:57.05(791267)
BG Mode:None Group 1 - Event 1 Scan

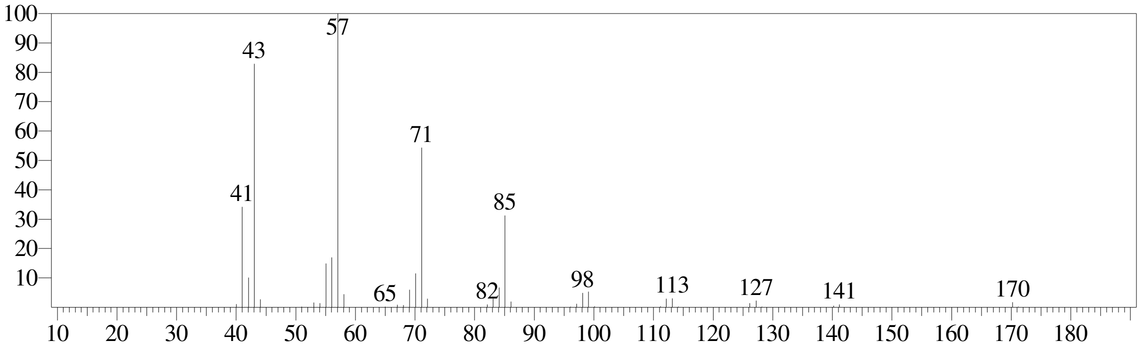

Hit#:1 Entry:43802 Library:NIST23-1.lib
SI:98 Formula:C13H28 CAS:629-50-5 MolWeight:184 RetIndex:1293
CompName:Tridecane $$ n-Tridecane $$ Tridecane, n- $$

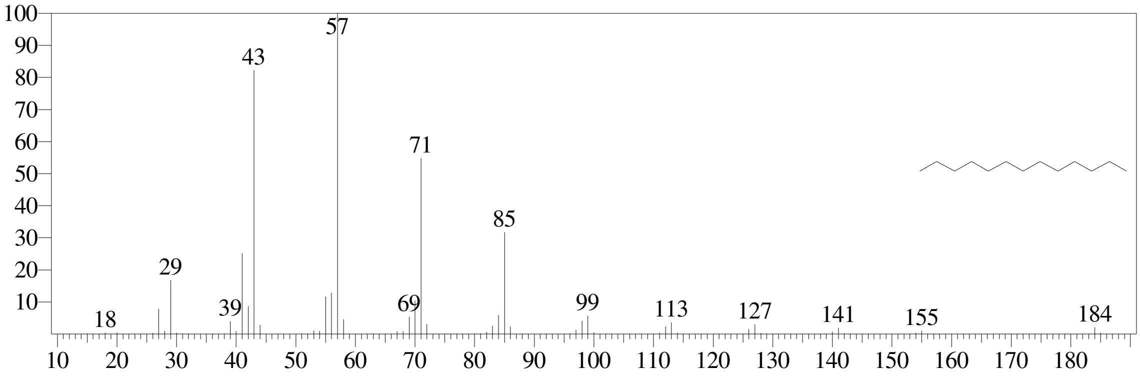

Hit#:2 Entry:32515 Library:NIST23-1.lib
SI:97 Formula:C12H26 CAS:112-40-3 MolWeight:170 RetIndex:1193
CompName:Dodecane $$ n-Dodecane $$ Adakane 12 $$ Ba 51-090453 $$ CH3(CH2)10CH3 $$ Bihexyl $$ Dihexyl $$ I

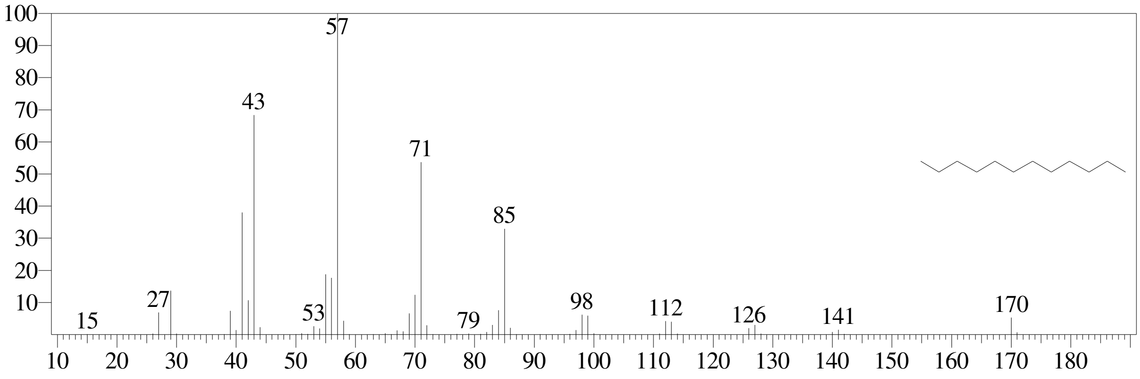

<< Target >>
Line#:6 R.Time:16.700(Scan#:1705) MassPeaks:43
RawMode:Averaged 16.692-16.708(1704-1706) BasePeak:57.05(791267)
BG Mode:None Group 1 - Event 1 Scan

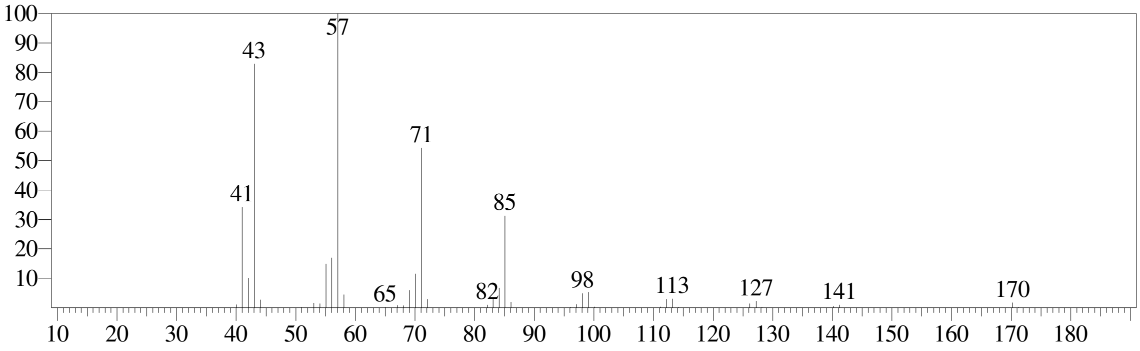

Hit#:3 Entry:17003 Library:NIST23s.lib
SI:97 Formula:C12H26 CAS:112-40-3 MolWeight:170 RetIndex:1193
CompName:Dodecane $$ n-Dodecane $$ Adakane 12 $$ Ba 51-090453 $$ CH3(CH2)10CH3 $$ Bihexyl $$ Dihexyl $$ I

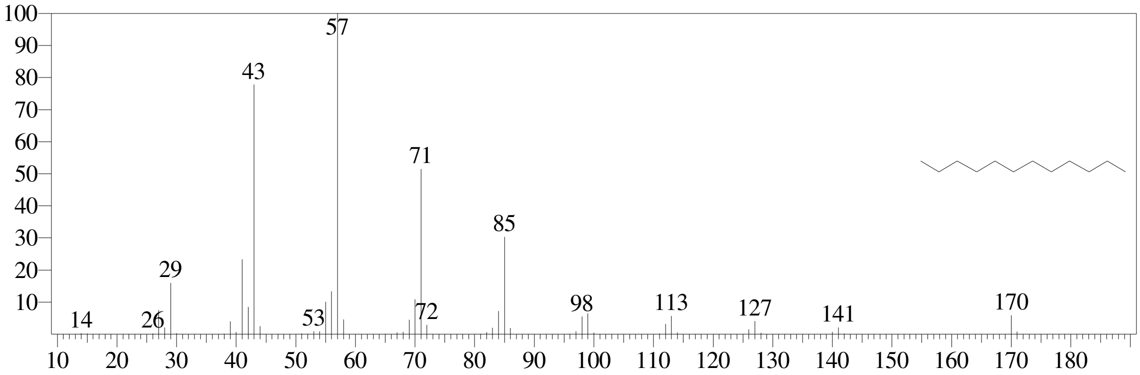

Hit#:4 Entry:20394 Library:NIST23s.lib
SI:97 Formula:C13H28 CAS:629-50-5 MolWeight:184 RetIndex:1293
CompName:Tridecane $$ n-Tridecane $$ Tridecane, n- $$

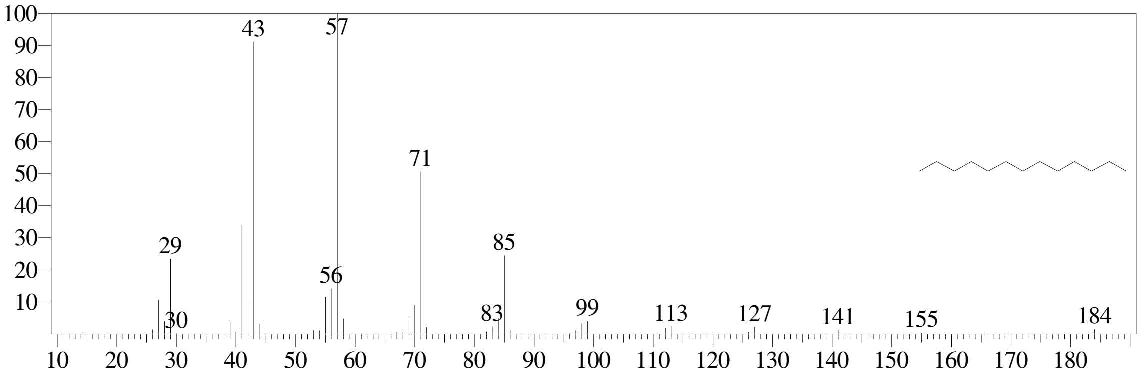

<< Target >>
Line#:6   R.Time:16.700(Scan#:1705) MassPeaks:43
RawMode:Averaged 16.692-16.708(1704-1706) BasePeak:57.05(791267)
BG Mode:None Group 1 - Event 1 Scan

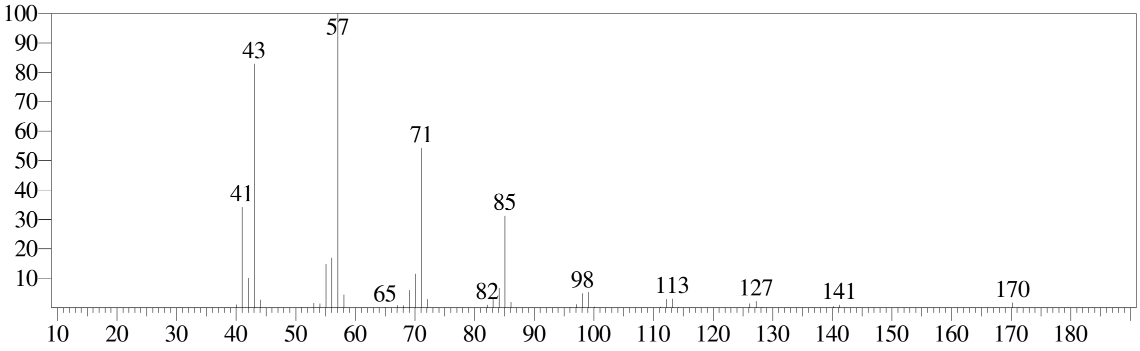

Hit#:5 Entry:22562 Library:NIST23-1.lib
SI:96 Formula:C11H24 CAS:1120-21-4 MolWeight:156 RetIndex:1094
CompName:Undecane $$ n-Undecane $$ Hendecane $$ n-C11H24 $$ UN 2330 $$

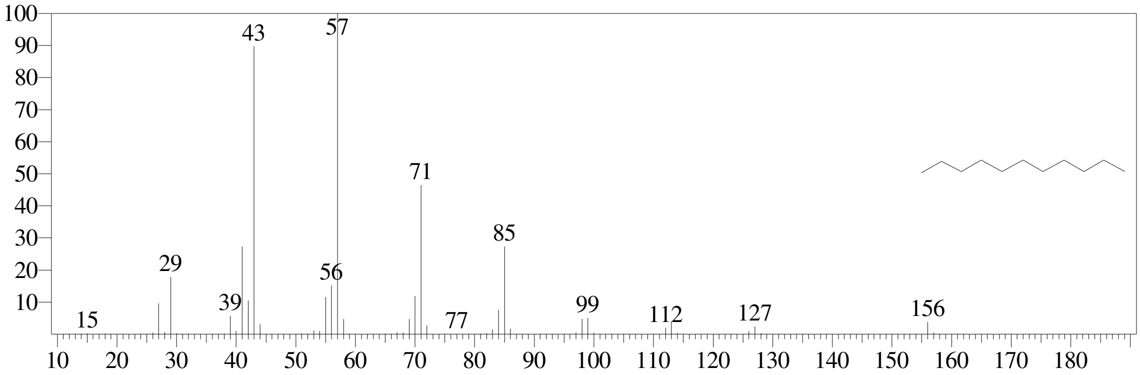

<< Target >>
Line#:7 R.Time:21.075(Scan#:2230) MassPeaks:46
RawMode:Averaged 21.067-21.083(2229-2231) BasePeak:57.05(814594)
BG Mode:None Group 1 - Event 1 Scan

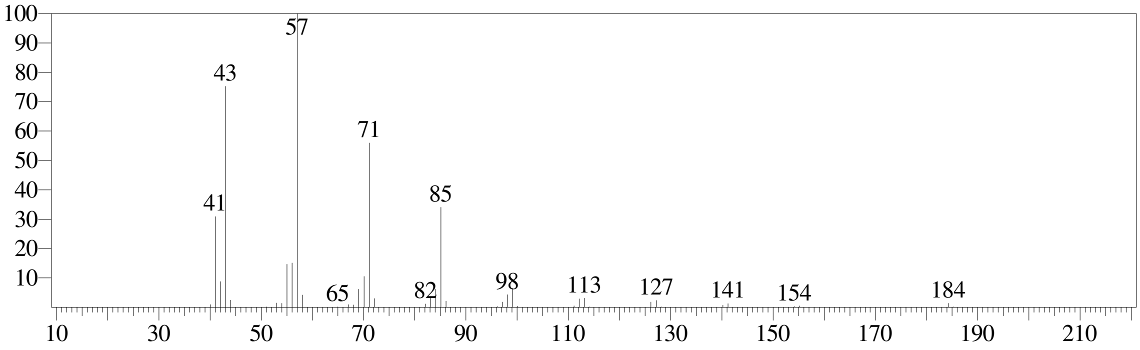

Hit#:1 Entry:43802 Library:NIST23-1.lib
SI:98 Formula:C13H28 CAS:629-50-5 MolWeight:184 RetIndex:1293
CompName:Tridecane $$ n-Tridecane $$ Tridecane, n- $$

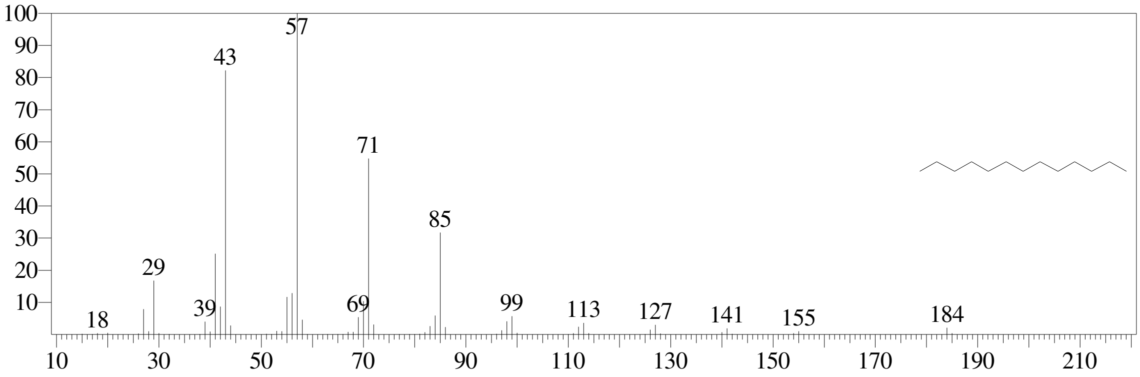

Hit#:2 Entry:72073 Library:NIST23-1.lib
SI:97 Formula:C15H32 CAS:629-62-9 MolWeight:212 RetIndex:1497
CompName:Pentadecane $$ n-Pentadecane $$ CH3(CH2)13CH3 $$

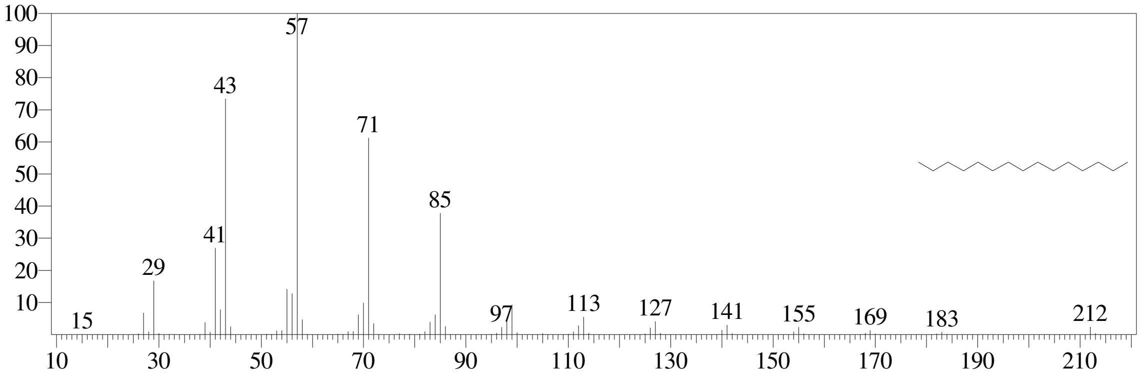

<< Target >>
Line#:7   R.Time:21.075(Scan#:2230) MassPeaks:46
RawMode:Averaged 21.067-21.083(2229-2231) BasePeak:57.05(814594)
BG Mode:None Group 1 - Event 1 Scan

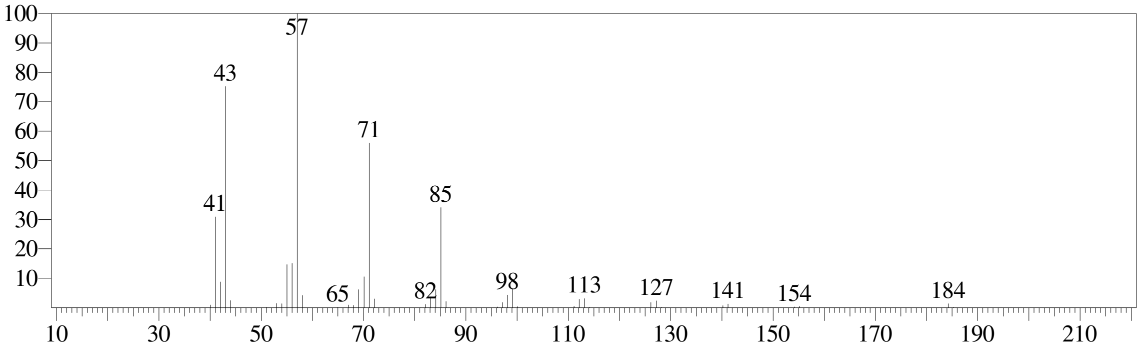

Hit#:3 Entry:23688 Library:NIST23s.lib
SI:97 Formula:C14H30 CAS:629-59-4 MolWeight:198 RetIndex:1395
CompName:Tetradecane $$ n-Tetradecane $$

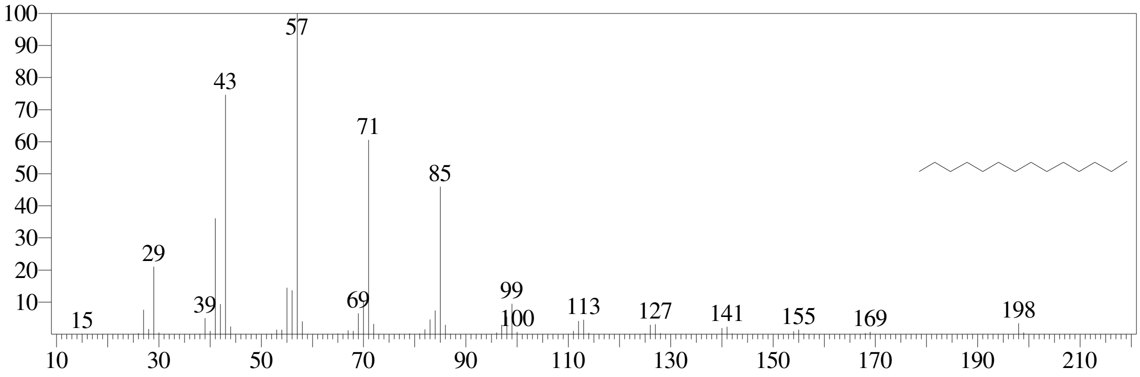

Hit#:4 Entry:32515 Library:NIST23-1.lib
SI:97 Formula:C12H26 CAS:112-40-3 MolWeight:170 RetIndex:1193
CompName:Dodecane $$ n-Dodecane $$ Adakane 12 $$ Ba 51-090453 $$ CH3(CH2)10CH3 $$ Bihexyl $$ Dihexyl $$ I

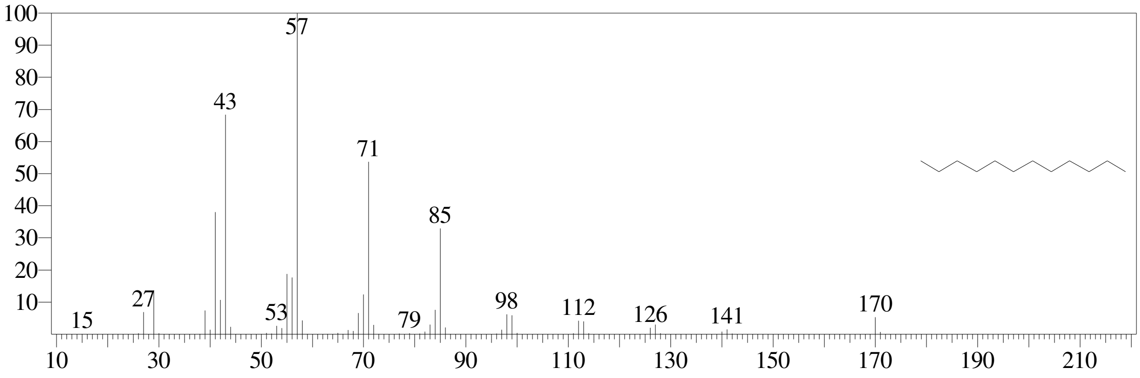

<< Target >>
Line#:7 R.Time:21.075(Scan#:2230) MassPeaks:46
RawMode:Averaged 21.067-21.083(2229-2231) BasePeak:57.05(814594)
BG Mode:None Group 1 - Event 1 Scan

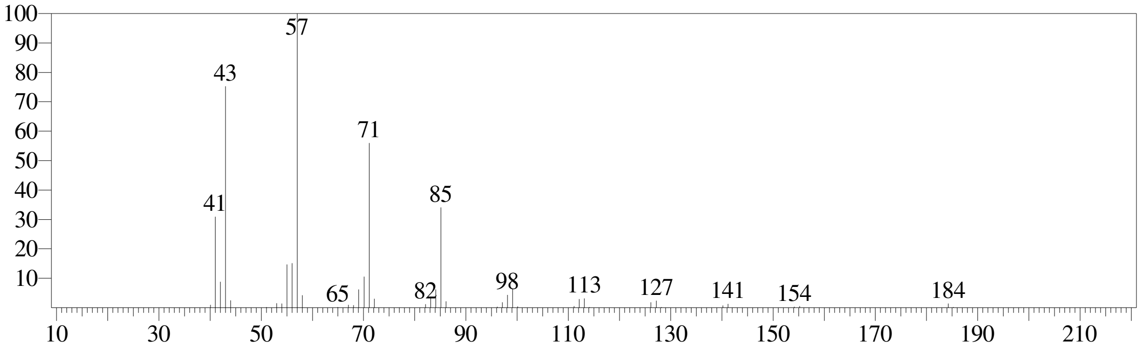

Hit#:5 Entry:57112 Library:NIST23-1.lib
SI:96 Formula:C14H30 CAS:629-59-4 MolWeight:198 RetIndex:1395
CompName:Tetradecane $$ n-Tetradecane $$

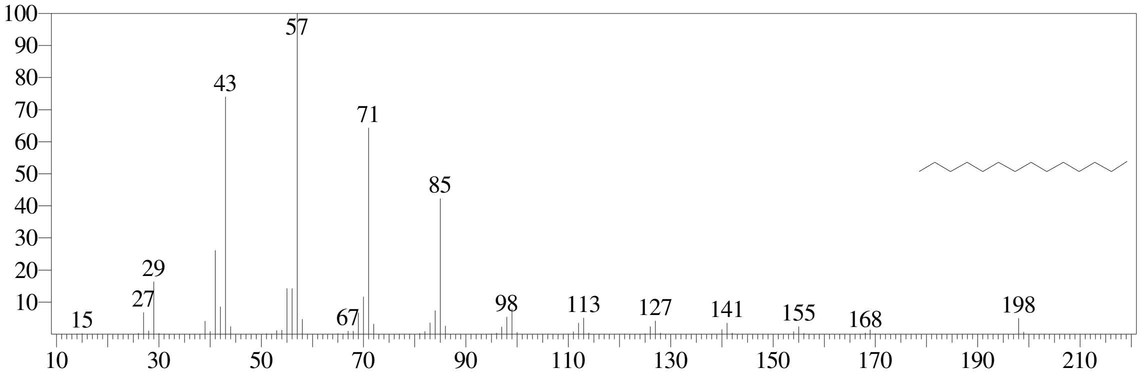

<< Target >>
Line#:8 R.Time:25.333(Scan#:2741) MassPeaks:49
RawMode:Averaged 25.325-25.342(2740-2742) BasePeak:57.05(809477)
BG Mode:None Group 1 - Event 1 Scan

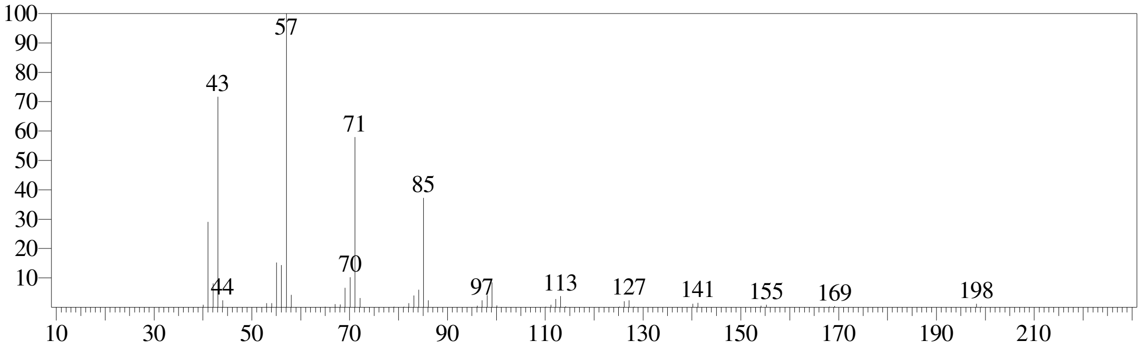

Hit#:1 Entry:72073 Library:NIST23-1.lib
SI:98 Formula:C15H32 CAS:629-62-9 MolWeight:212 RetIndex:1497
CompName:Pentadecane $$ n-Pentadecane $$ CH3(CH2)13CH3 $$

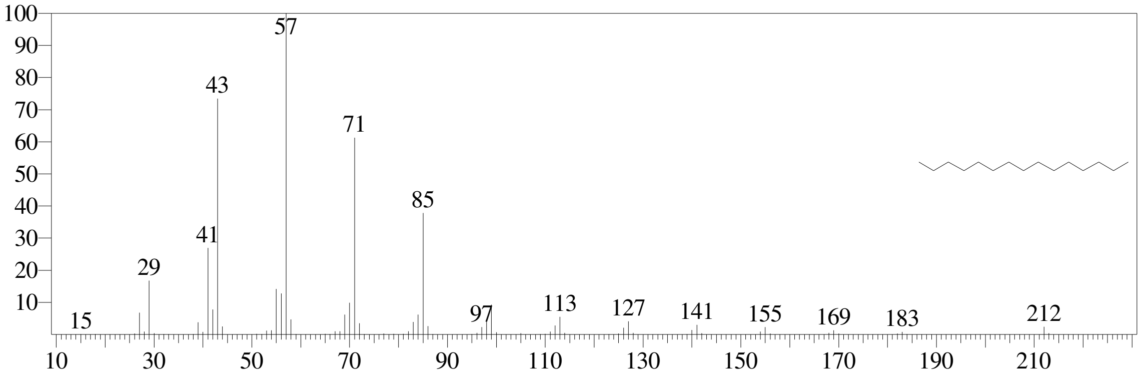

Hit#:2 Entry:57112 Library:NIST23-1.lib
SI:98 Formula:C14H30 CAS:629-59-4 MolWeight:198 RetIndex:1395
CompName:Tetradecane $$ n-Tetradecane $$

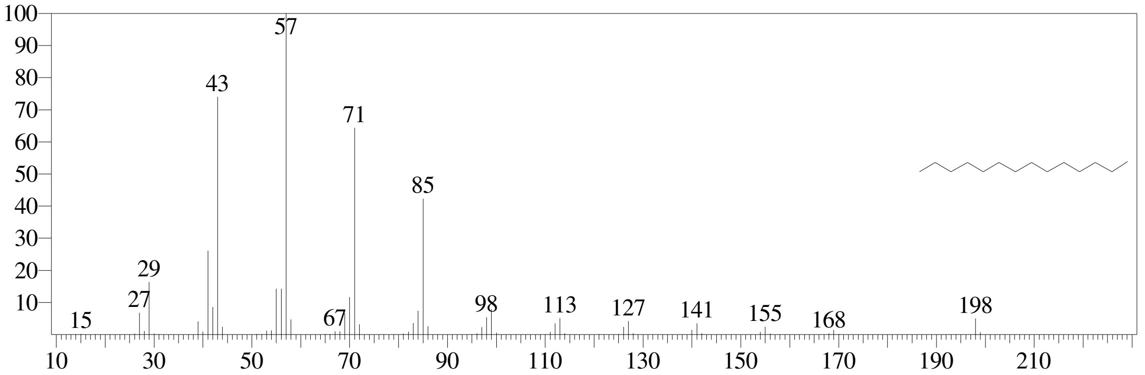

<< Target >>
Line#:8 R.Time:25.333(Scan#:2741) MassPeaks:49
RawMode:Averaged 25.325-25.342(2740-2742) BasePeak:57.05(809477)
BG Mode:None Group 1 - Event 1 Scan

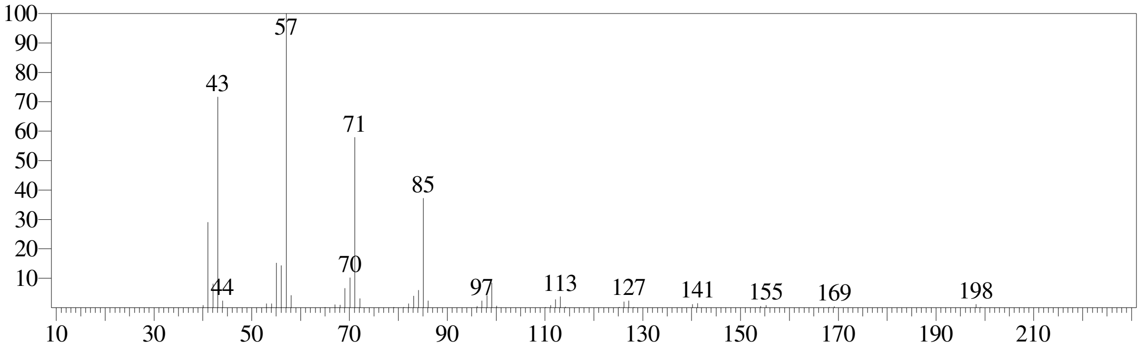

Hit#:3 Entry:23688 Library:NIST23s.lib
SI:98 Formula:C14H30 CAS:629-59-4 MolWeight:198 RetIndex:1395
CompName:Tetradecane $$ n-Tetradecane $$

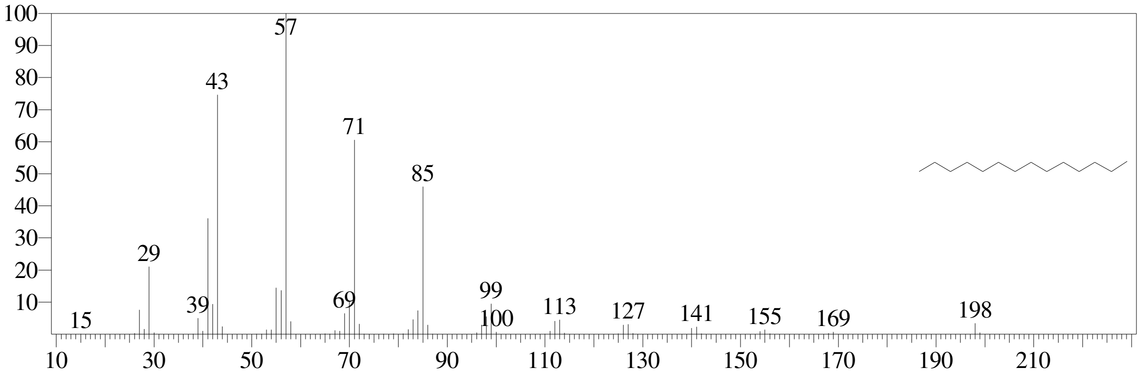

Hit#:4 Entry:88527 Library:NIST23-1.lib
SI:97 Formula:C16H34 CAS:544-76-3 MolWeight:226 RetIndex:1599
CompName:Hexadecane $$ n-Cetane $$ n-Hexadecane $$ Cetane $$

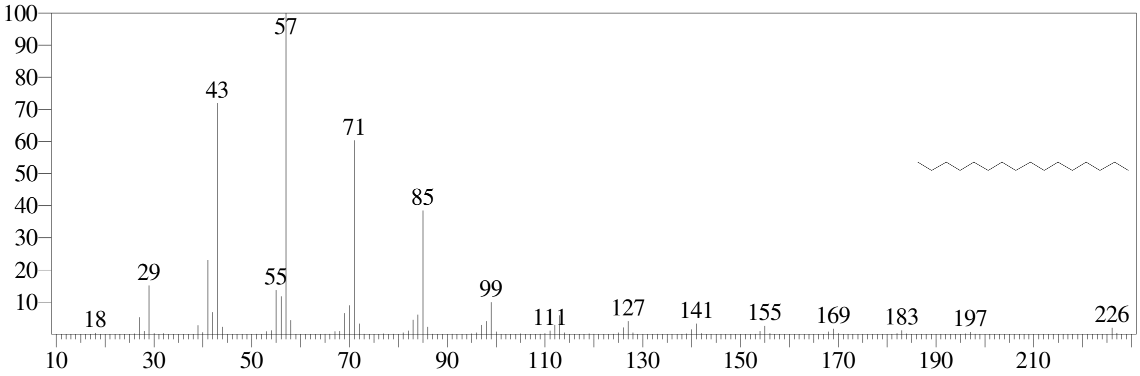

<< Target >>
Line#:8   R.Time:25.333(Scan#:2741) MassPeaks:49
RawMode:Averaged 25.325-25.342(2740-2742) BasePeak:57.05(809477)
BG Mode:None Group 1 - Event 1 Scan

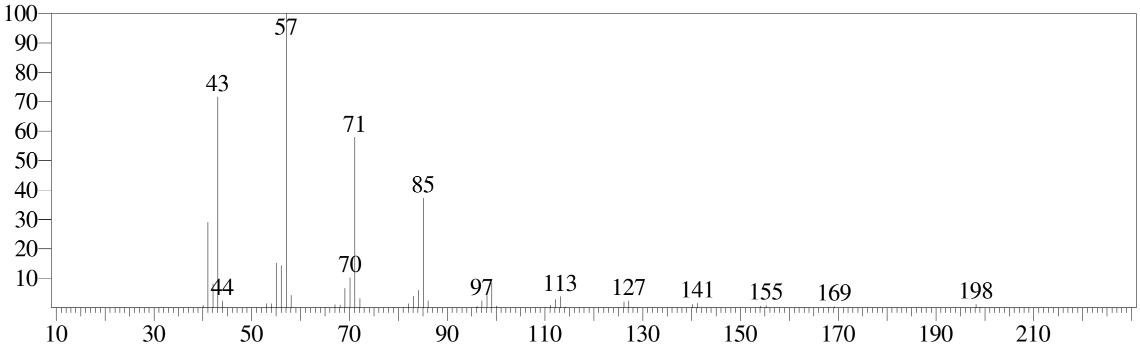

Hit#:5 Entry:43802 Library:NIST23-1.lib
SI:97 Formula:C13H28 CAS:629-50-5 MolWeight:184 RetIndex:1293
CompName:Tridecane $$ n-Tridecane $$ Tridecane, n- $$

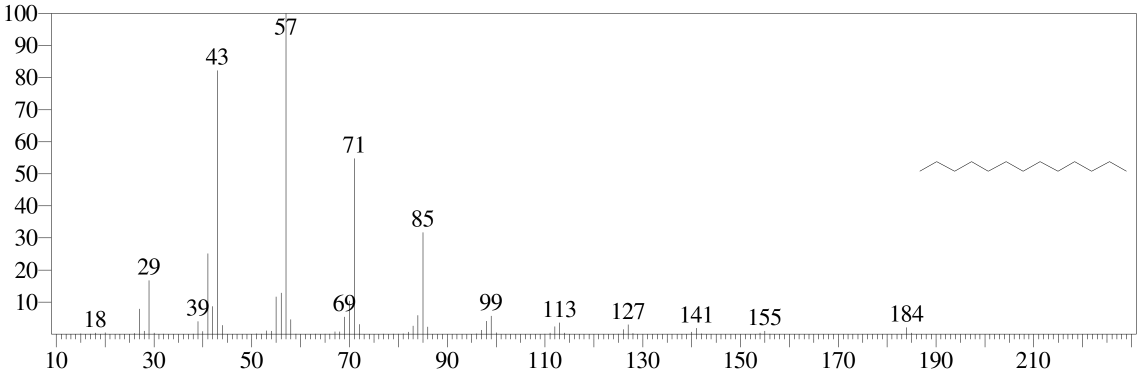

<< Target >>
Line#:9   R.Time:29.417(Scan#:3231) MassPeaks:51
RawMode:Averaged 29.408-29.425(3230-3232) BasePeak:57.05(780726)
BG Mode:None Group 1 - Event 1 Scan

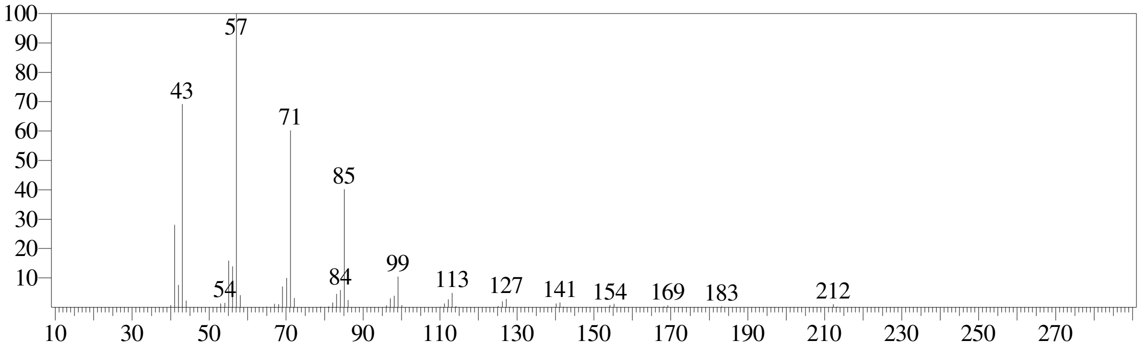

Hit#:1 Entry:72073 Library:NIST23-1.lib
SI:98 Formula:C15H32 CAS:629-62-9 MolWeight:212 RetIndex:1497
CompName:Pentadecane $$ n-Pentadecane $$ CH3(CH2)13CH3 $$

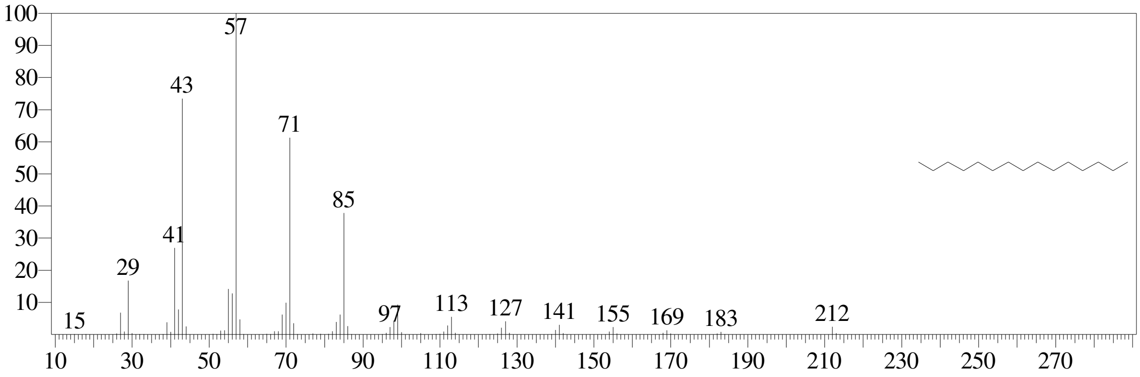

Hit#:2 Entry:88527 Library:NIST23-1.lib
SI:98 Formula:C16H34 CAS:544-76-3 MolWeight:226 RetIndex:1599
CompName:Hexadecane $$ n-Cetane $$ n-Hexadecane $$ Cetane $$

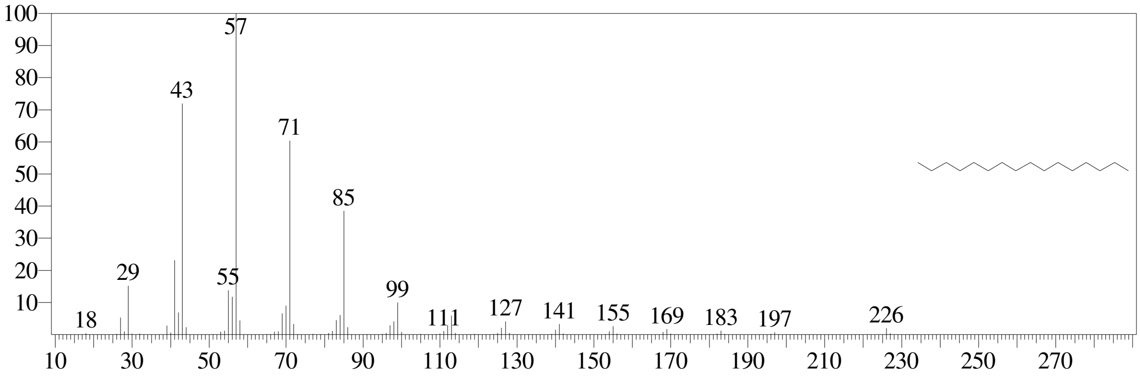

<< Target >>
Line#:9 R.Time:29.417(Scan#:3231) MassPeaks:51
RawMode:Averaged 29.408-29.425(3230-3232) BasePeak:57.05(780726)
BG Mode:None Group 1 - Event 1 Scan

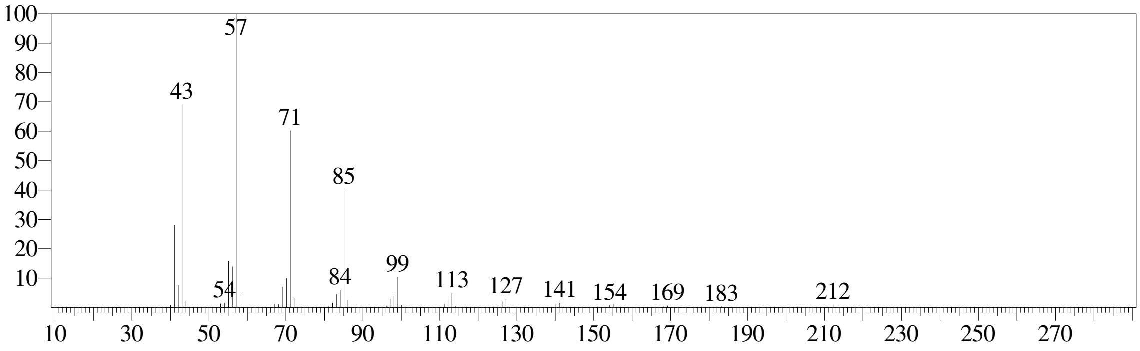

Hit#:3 Entry:23688 Library:NIST23s.lib
SI:97 Formula:C14H30 CAS:629-59-4 MolWeight:198 RetIndex:1395
CompName:Tetradecane $$ n-Tetradecane $$

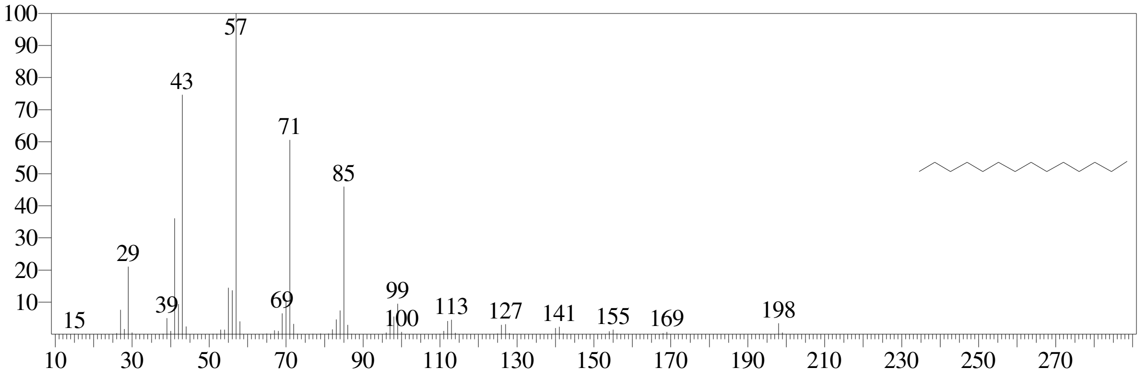

Hit#:4 Entry:57112 Library:NIST23-1.lib
SI:97 Formula:C14H30 CAS:629-59-4 MolWeight:198 RetIndex:1395
CompName:Tetradecane $$ n-Tetradecane $$

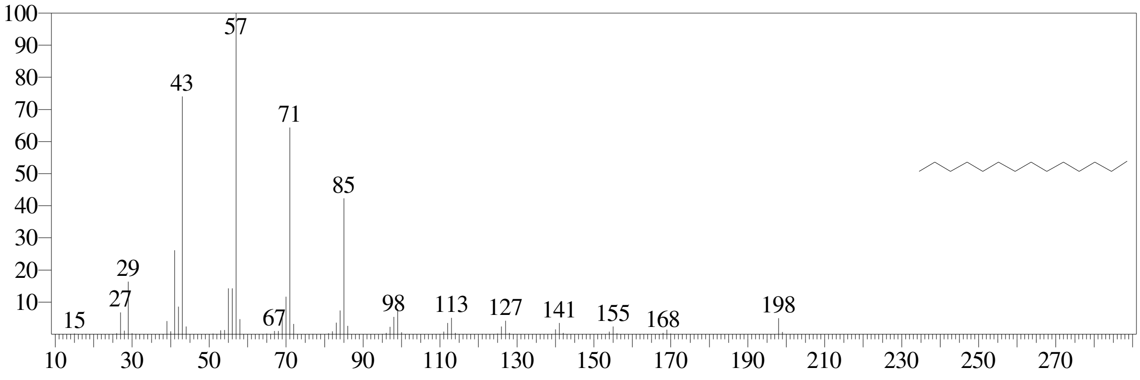

<< Target >>
Line#:9   R.Time:29.417(Scan#:3231) MassPeaks:51
RawMode:Averaged 29.408-29.425(3230-3232) BasePeak:57.05(780726)
BG Mode:None Group 1 - Event 1 Scan

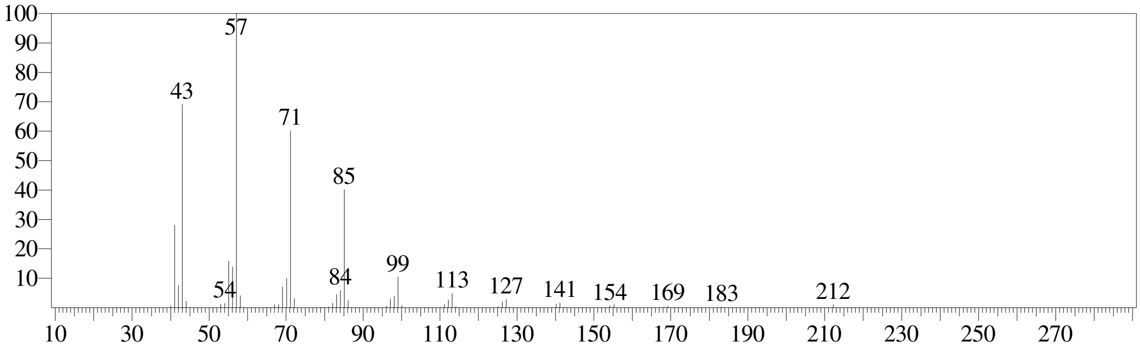

Hit#:5 Entry:36522 Library:NIST23s.lib
SI:97 Formula:C20H42 CAS:112-95-8 MolWeight:282 RetIndex:2004
CompName:Eicosane $$ n-Eicosane $$ Icosane # $$ n-Icosane $$

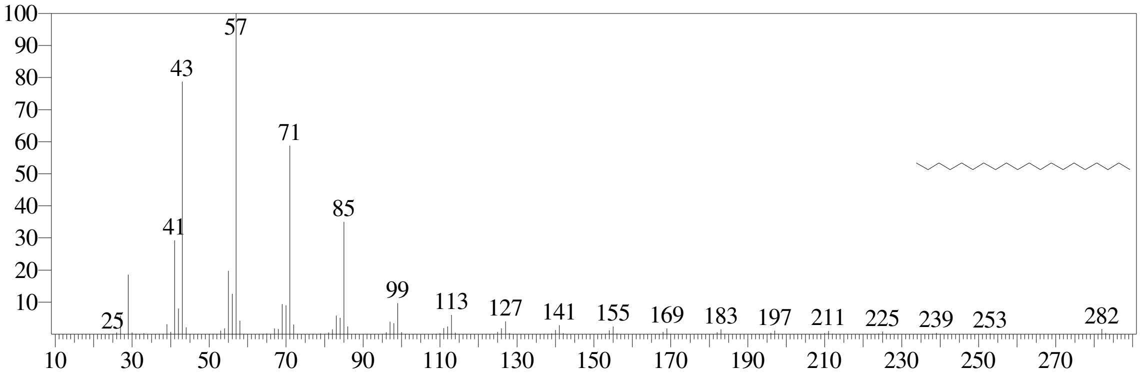

<< Target >>
Line#:10 R.Time:33.300(Scan#:3697) MassPeaks:56
RawMode:Averaged 33.292-33.308(3696-3698) BasePeak:57.05(767666)
BG Mode:None Group 1 - Event 1 Scan

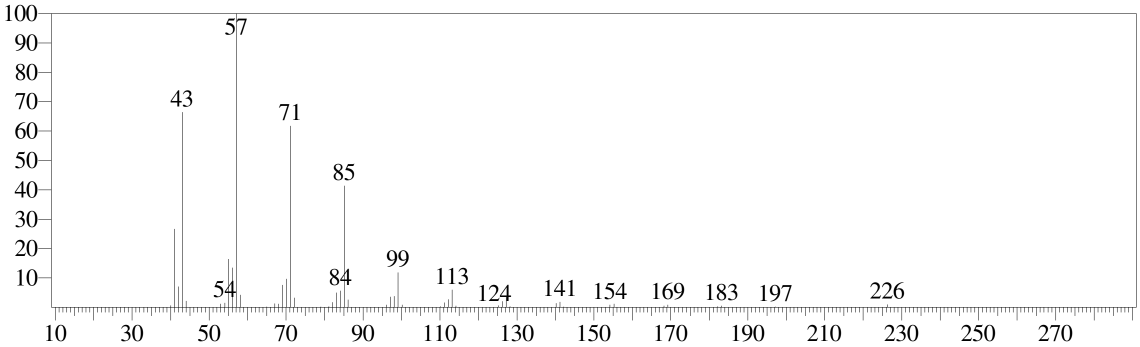

Hit#:1 Entry:88527 Library:NIST23-1.lib
SI:98 Formula:C16H34 CAS:544-76-3 MolWeight:226 RetIndex:1599
CompName:Hexadecane $$ n-Cetane $$ n-Hexadecane $$ Cetane $$

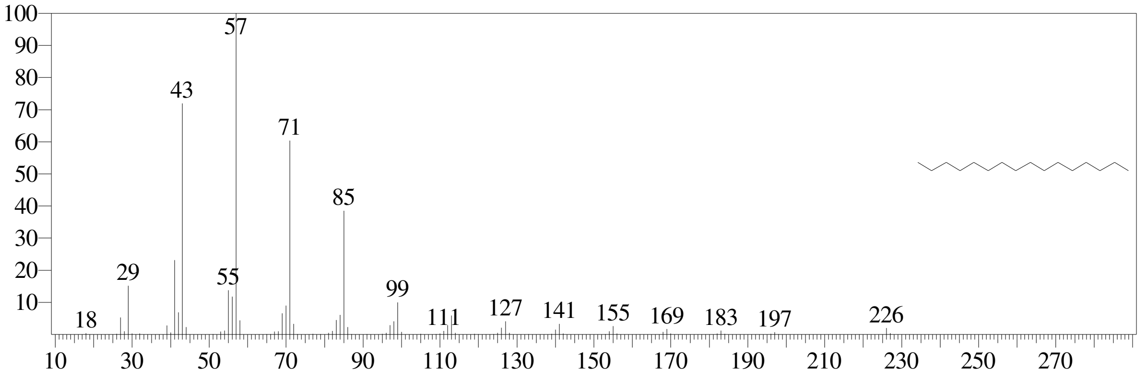

Hit#:2 Entry:72073 Library:NIST23-1.lib
SI:98 Formula:C15H32 CAS:629-62-9 MolWeight:212 RetIndex:1497
CompName:Pentadecane $$ n-Pentadecane $$ CH3(CH2)13CH3 $$

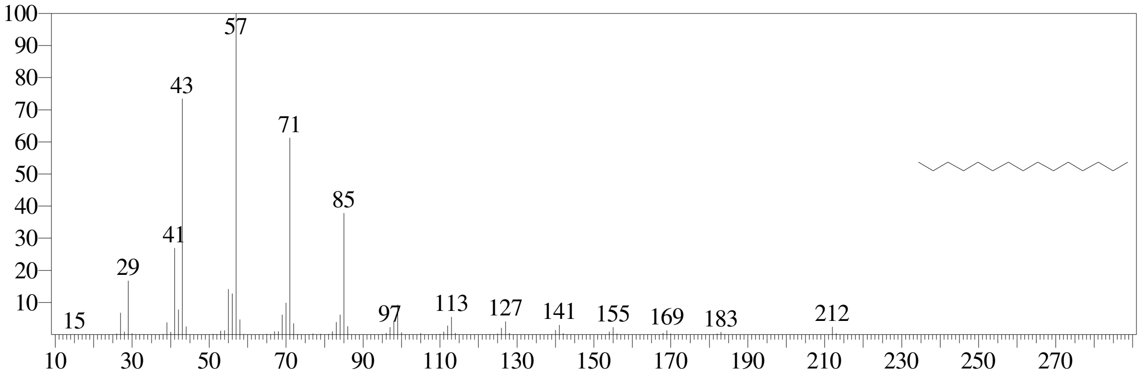

<< Target >>
Line#:10   R.Time:33.300(Scan#:3697) MassPeaks:56
RawMode:Averaged 33.292-33.308(3696-3698) BasePeak:57.05(767666)
BG Mode:None Group 1 - Event 1 Scan

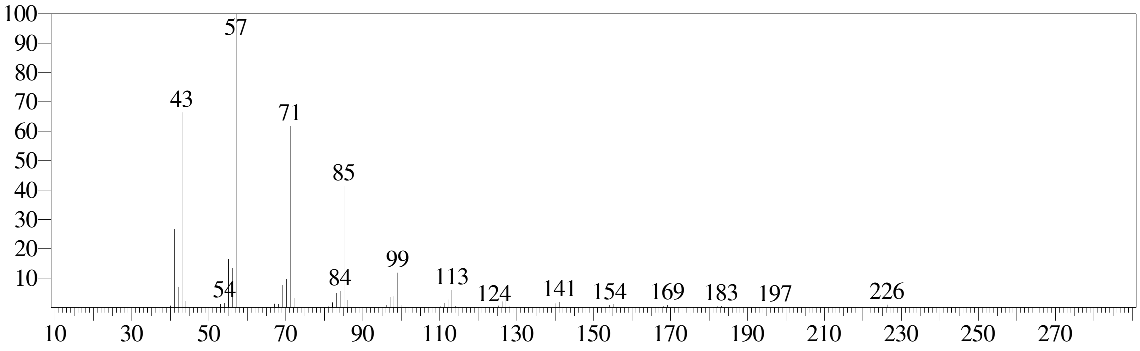

Hit#:3 Entry:33431 Library:NIST23s.lib
SI:97 Formula:C18H38 CAS:593-45-3 MolWeight:254 RetIndex:1802
CompName:Octadecane $$ n-Octadecane $$ Octadecan $$

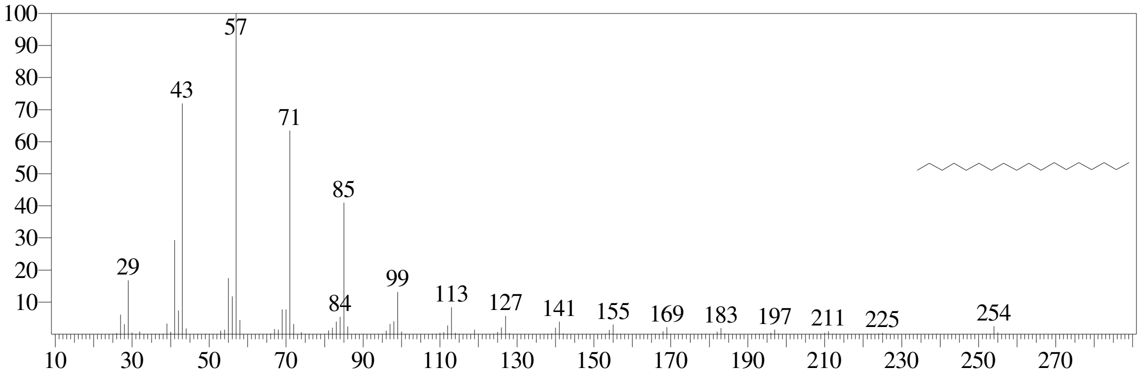

Hit#:4 Entry:36522 Library:NIST23s.lib
SI:97 Formula:C20H42 CAS:112-95-8 MolWeight:282 RetIndex:2004
CompName:Eicosane $$ n-Eicosane $$ Icosane # $$ n-Icosane $$

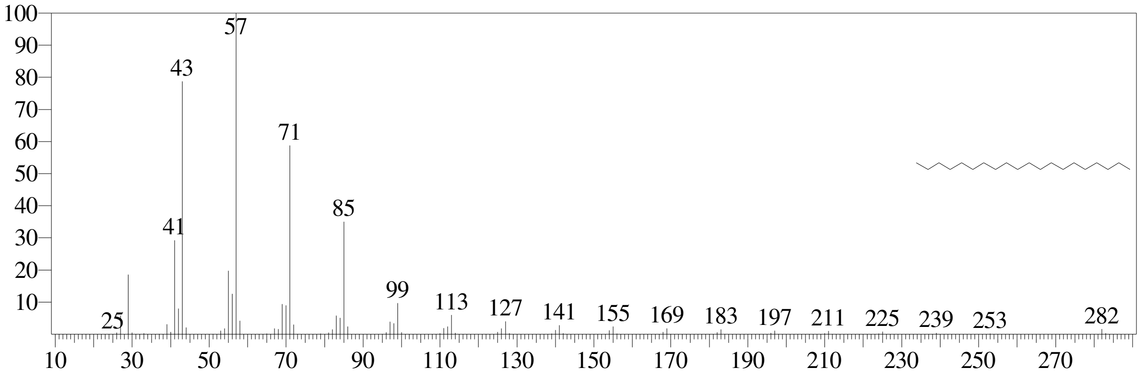

<< Target >>
Line#:10   R.Time:33.300(Scan#:3697) MassPeaks:56
RawMode:Averaged 33.292-33.308(3696-3698) BasePeak:57.05(767666)
BG Mode:None Group 1 - Event 1 Scan

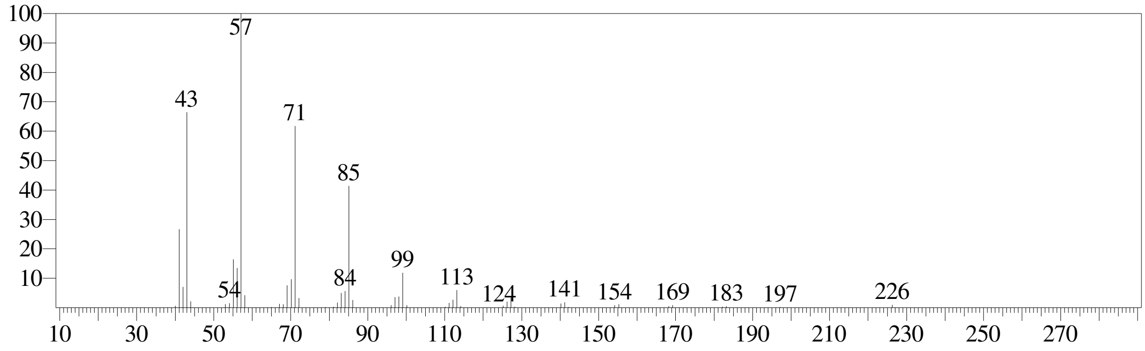

Hit#:5 Entry:57112 Library:NIST23-1.lib
SI:97 Formula:C14H30 CAS:629-59-4 MolWeight:198 RetIndex:1395
CompName:Tetradecane $$ n-Tetradecane $$

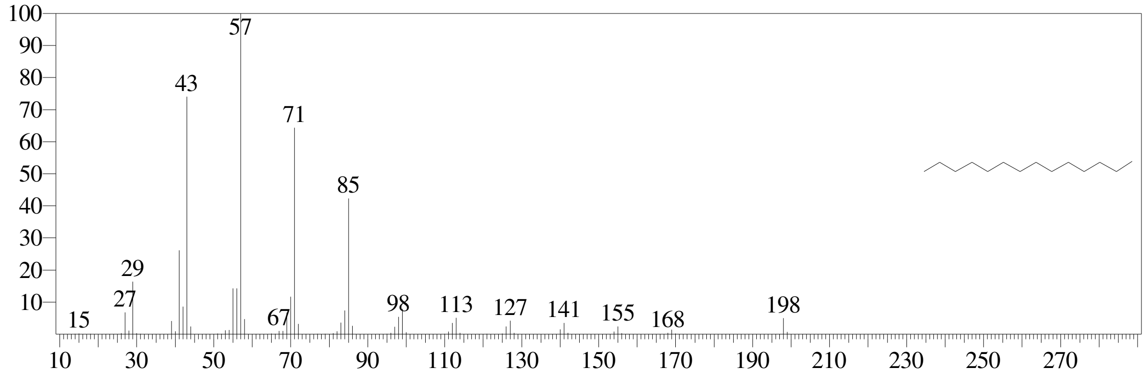

<< Target >>
Line#:11 R.Time:36.983(Scan#:4139) MassPeaks:58
RawMode:Averaged 36.975-36.992(4138-4140) BasePeak:57.05(768227)
BG Mode:Calc. from Peak Group 1 - Event 1 Scan

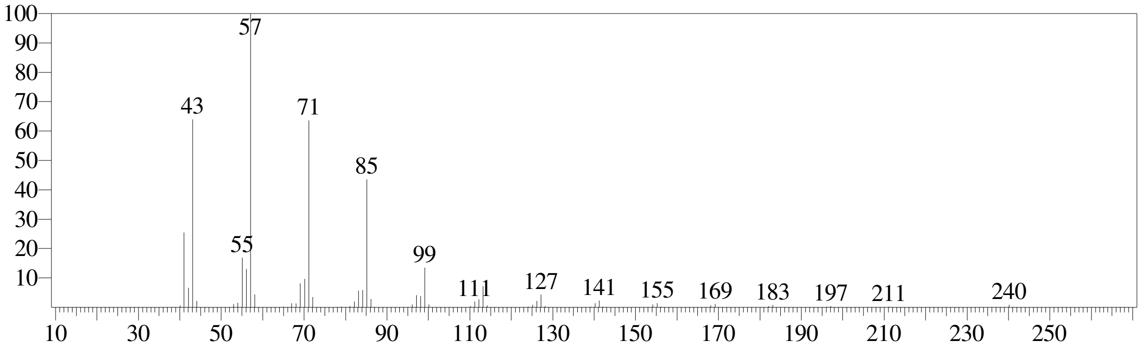

Hit#:1 Entry:105397 Library:NIST23-1.lib
SI:98 Formula:C17H36 CAS:629-78-7 MolWeight:240 RetIndex:1701
CompName:Heptadecane $$ n-Heptadecane $$ Normal-heptadecane $$

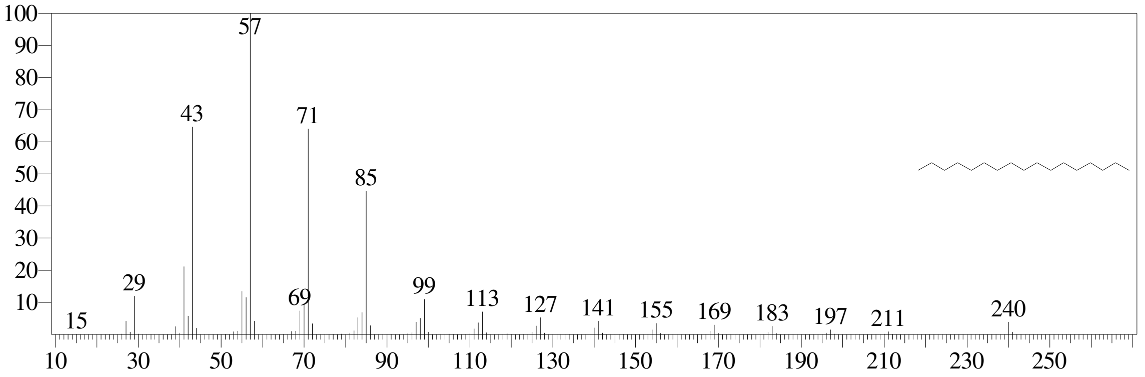

Hit#:2 Entry:88527 Library:NIST23-1.lib
SI:97 Formula:C16H34 CAS:544-76-3 MolWeight:226 RetIndex:1599
CompName:Hexadecane $$ n-Cetane $$ n-Hexadecane $$ Cetane $$

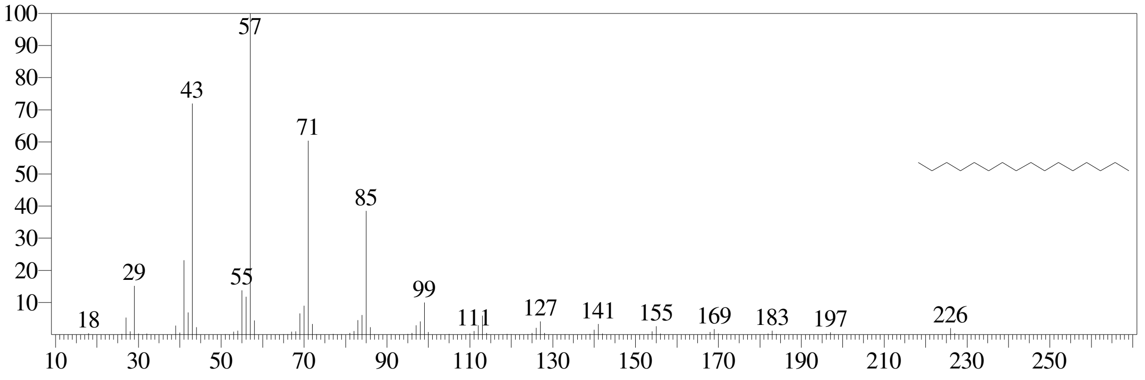

<< Target >>
Line#:11   R.Time:36.983(Scan#:4139) MassPeaks:58
RawMode:Averaged 36.975-36.992(4138-4140) BasePeak:57.05(768227)
BG Mode:Calc. from Peak Group 1 - Event 1 Scan

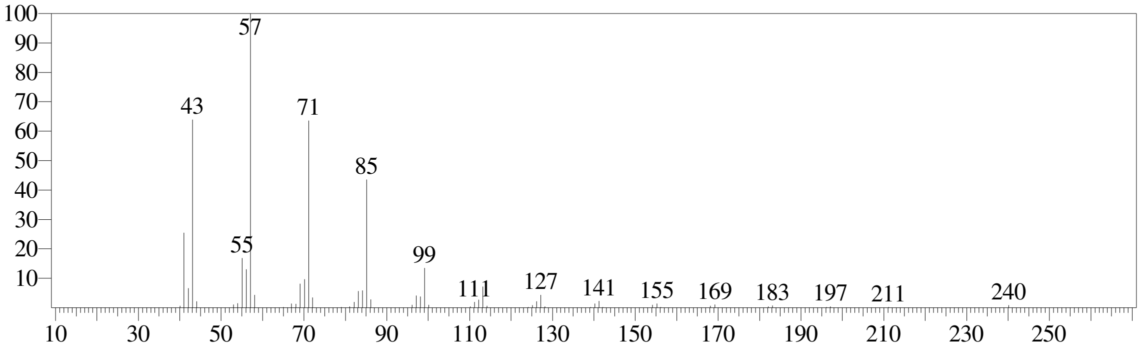

Hit#:3 Entry:31590 Library:NIST23s.lib
SI:97 Formula:C17H36 CAS:629-78-7 MolWeight:240 RetIndex:1701
CompName:Heptadecane $$ n-Heptadecane $$ Normal-heptadecane $$

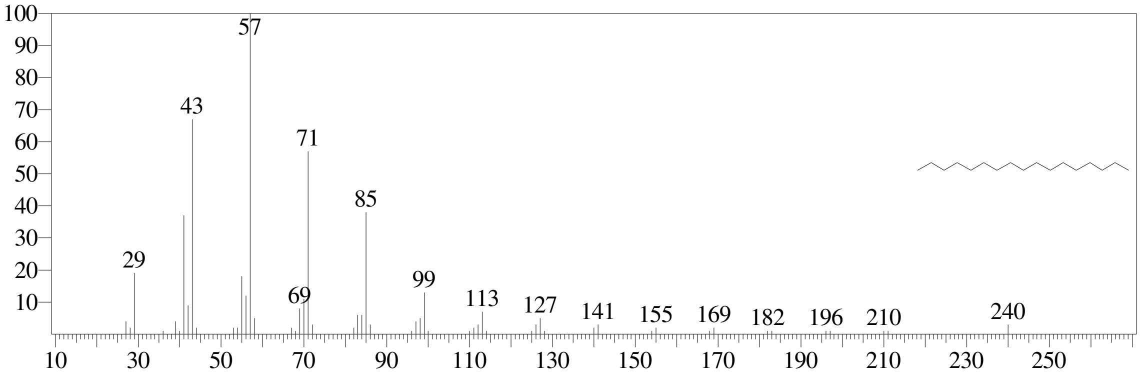

Hit#:4 Entry:141042 Library:NIST23-1.lib
SI:97 Formula:C19H40 CAS:629-92-5 MolWeight:268 RetIndex:1903
CompName:Nonadecane $$ n-Nonadecane $$

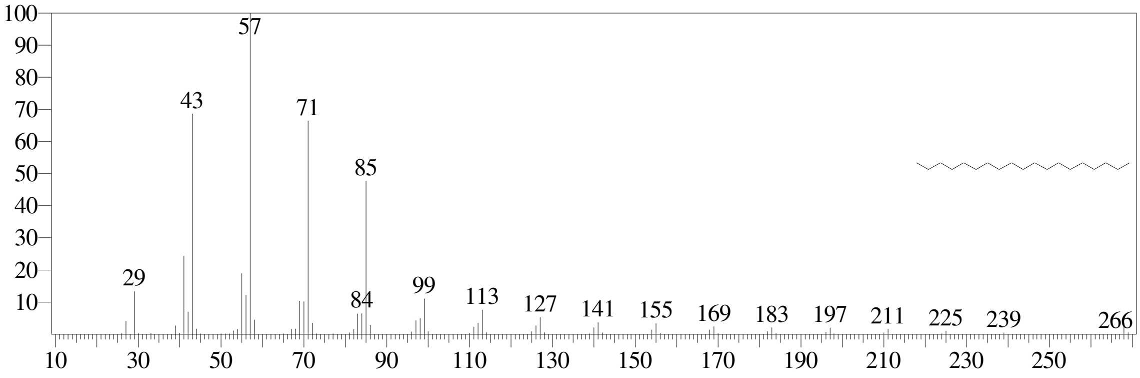

<< Target >>
Line#:11 R.Time:36.983(Scan#:4139) MassPeaks:58
RawMode:Averaged 36.975-36.992(4138-4140) BasePeak:57.05(768227)
BG Mode:Calc. from Peak Group 1 - Event 1 Scan

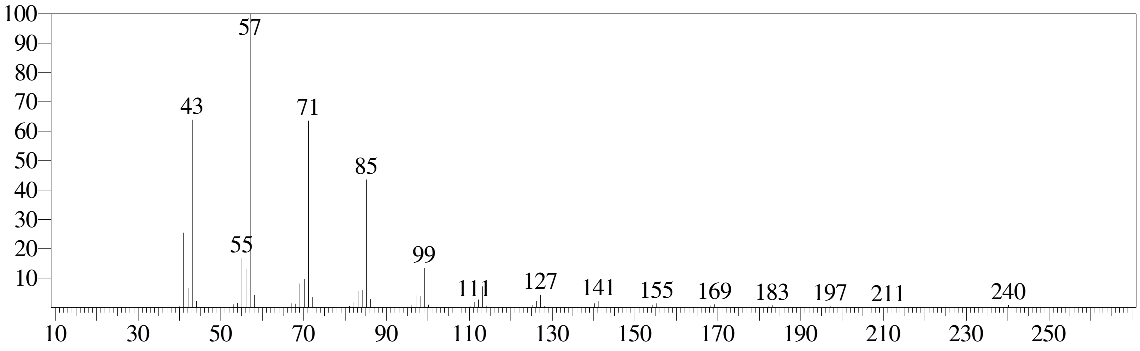

Hit#:5 Entry:33431 Library:NIST23s.lib
SI:97 Formula:C18H38 CAS:593-45-3 MolWeight:254 RetIndex:1802
CompName:Octadecane $$ n-Octadecane $$ Octadecan $$

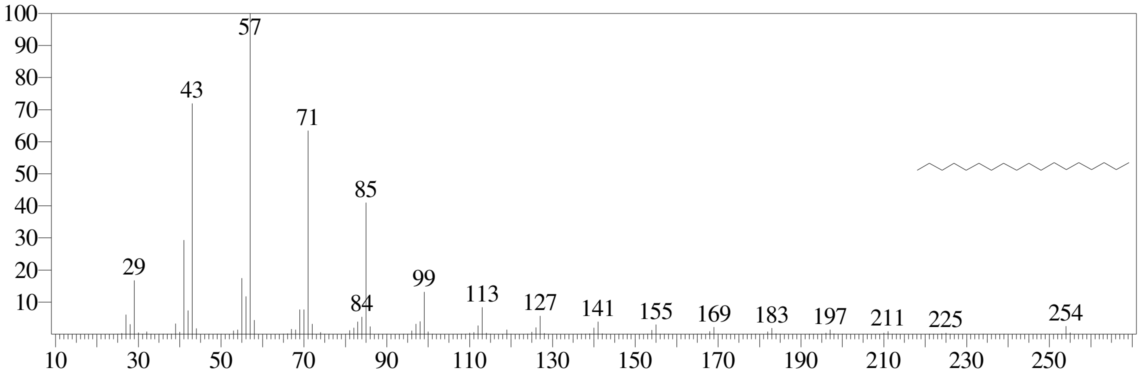

<< Target >>
Line#:12   R.Time:40.500(Scan#:4561) MassPeaks:61
RawMode:Averaged 40.492-40.508(4560-4562) BasePeak:57.05(756044)
BG Mode:Calc. from Peak Group 1 - Event 1 Scan

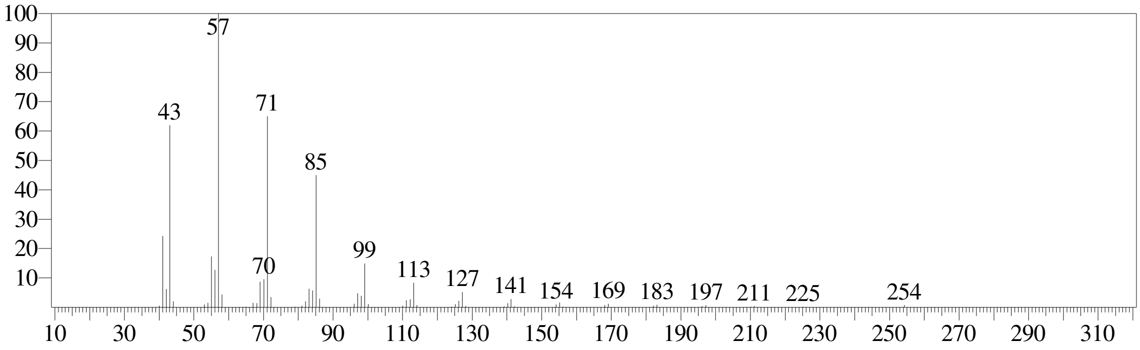

Hit#:1 Entry:141042 Library:NIST23-1.lib
SI:97 Formula:C19H40 CAS:629-92-5 MolWeight:268 RetIndex:1903
CompName:Nonadecane $$ n-Nonadecane $$

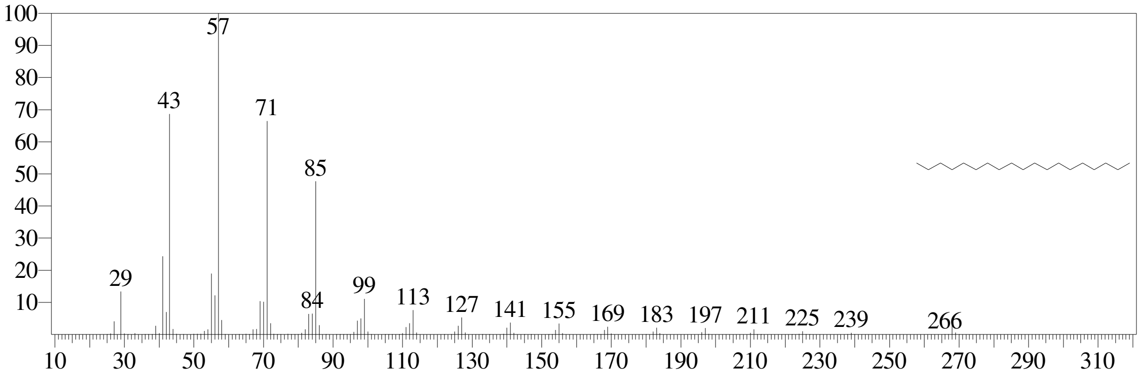

Hit#:2 Entry:122857 Library:NIST23-1.lib
SI:97 Formula:C18H38 CAS:593-45-3 MolWeight:254 RetIndex:1802
CompName:Octadecane $$ n-Octadecane $$ Octadecan $$

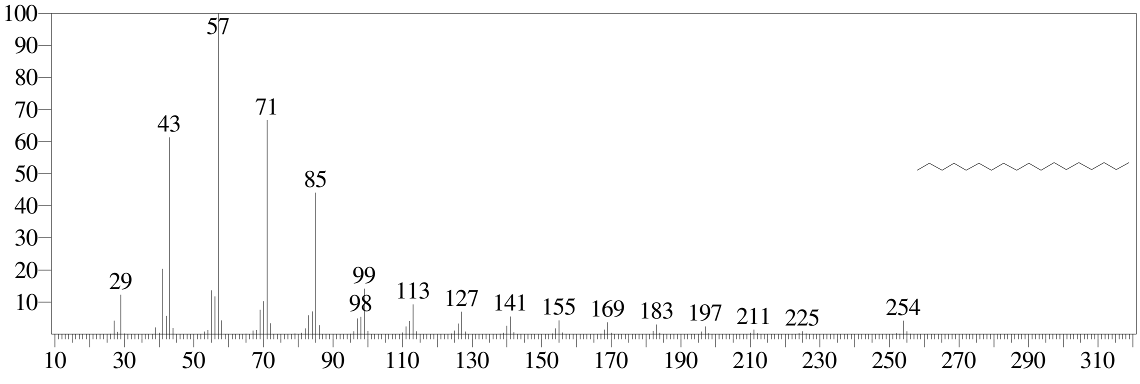

<< Target >>
Line#:12   R.Time:40.500(Scan#:4561) MassPeaks:61
RawMode:Averaged 40.492-40.508(4560-4562) BasePeak:57.05(756044)
BG Mode:Calc. from Peak Group 1 - Event 1 Scan

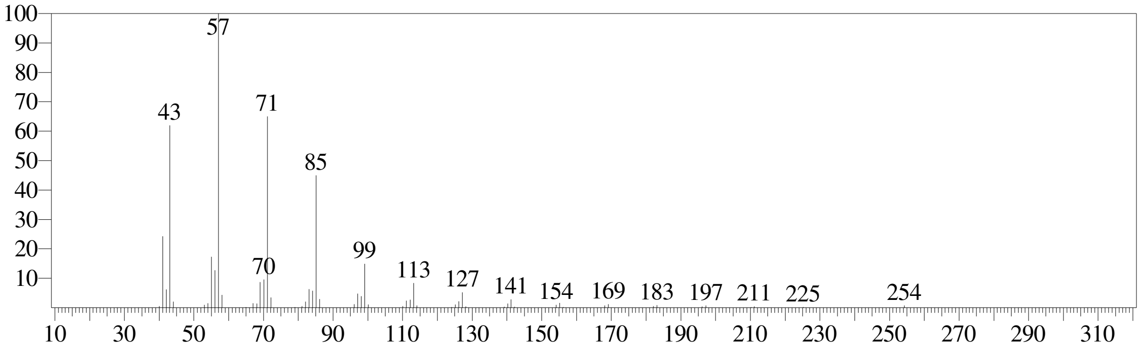

Hit#:3 Entry:105397 Library:NIST23-1.lib
SI:97 Formula:C17H36 CAS:629-78-7 MolWeight:240 RetIndex:1701
CompName:Heptadecane $$ n-Heptadecane $$ Normal-heptadecane $$

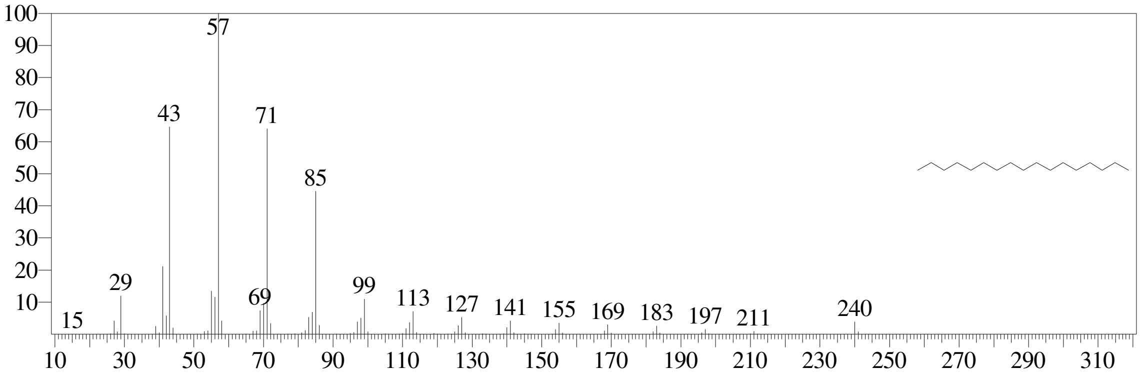

Hit#:4 Entry:33431 Library:NIST23s.lib
SI:97 Formula:C18H38 CAS:593-45-3 MolWeight:254 RetIndex:1802
CompName:Octadecane $$ n-Octadecane $$ Octadecan $$

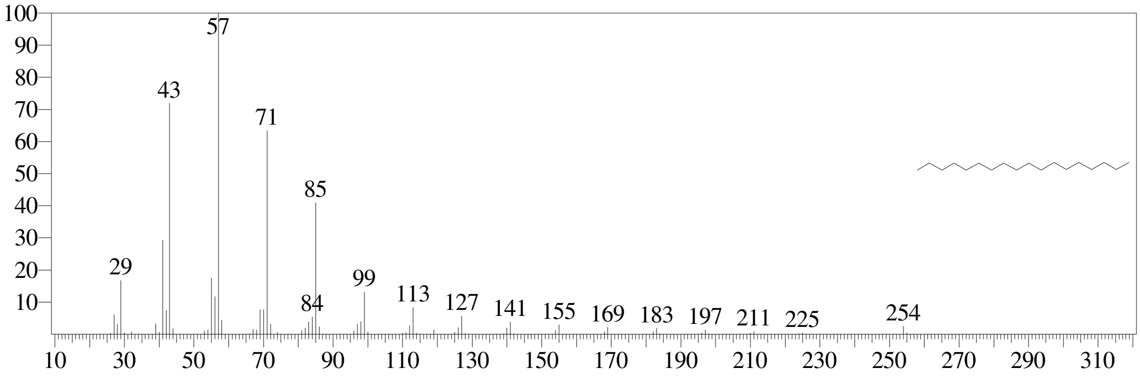

<< Target >>
Line#:12 R.Time:40.500(Scan#:4561) MassPeaks:61
RawMode:Averaged 40.492-40.508(4560-4562) BasePeak:57.05(756044)
BG Mode:Calc. from Peak Group 1 - Event 1 Scan

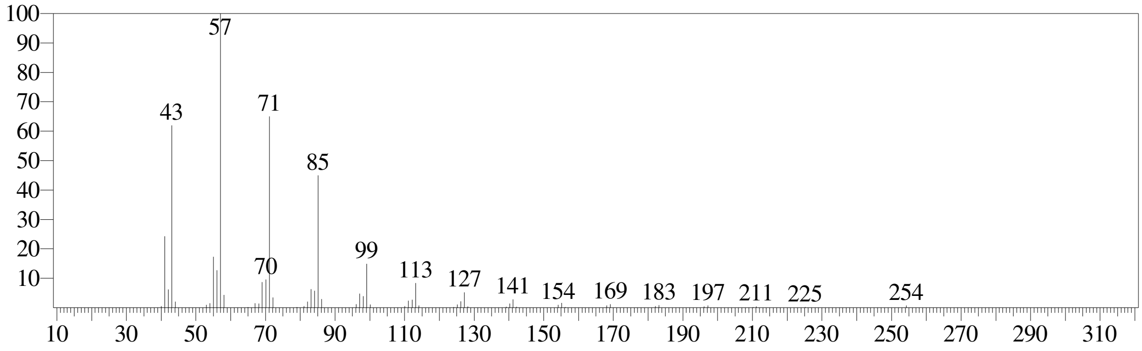

Hit#:5 Entry:39191 Library:NIST23s.lib
SI:97 Formula:C22H46 CAS:629-97-0 MolWeight:310 RetIndex:2203
CompName:Docosane $$ n-Docosane $$ Normal-docosane $$

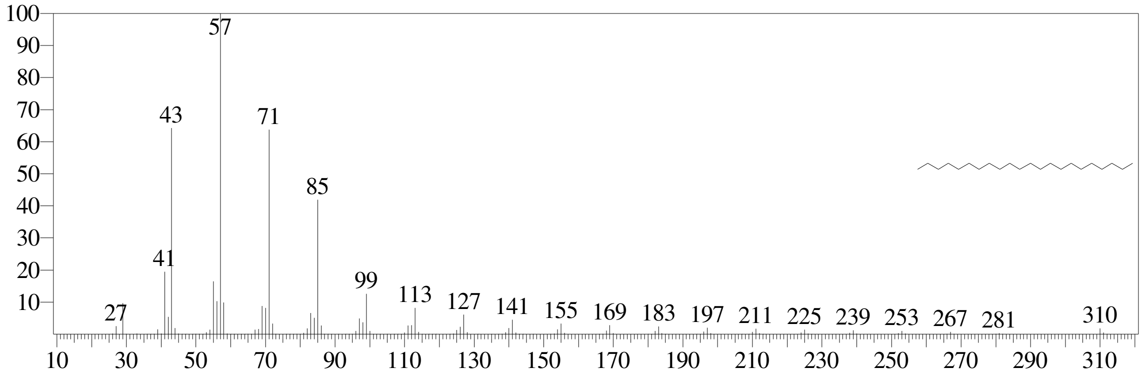

<< Target >>
Line#:13   R.Time:43.858(Scan#:4964) MassPeaks:65
RawMode:Averaged 43.850-43.867(4963-4965) BasePeak:57.05(725925)
BG Mode:Calc. from Peak Group 1 - Event 1 Scan

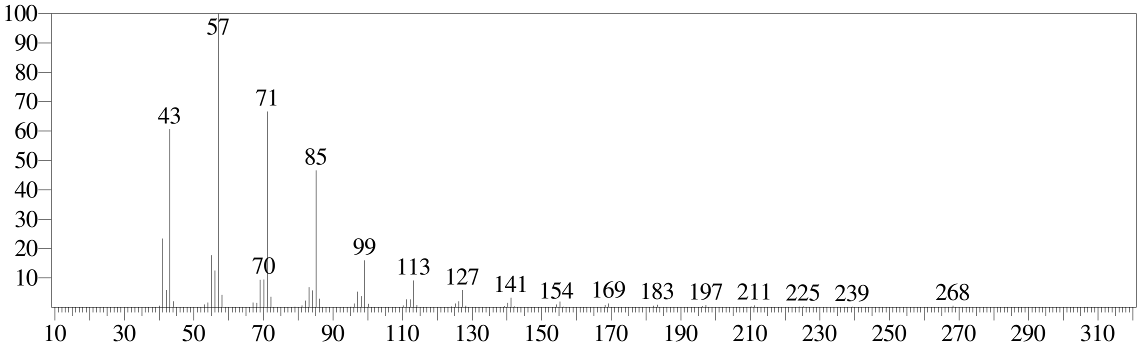

Hit#:1 Entry:141042 Library:NIST23-1.lib
SI:98 Formula:C19H40 CAS:629-92-5 MolWeight:268 RetIndex:1903
CompName:Nonadecane $$ n-Nonadecane $$

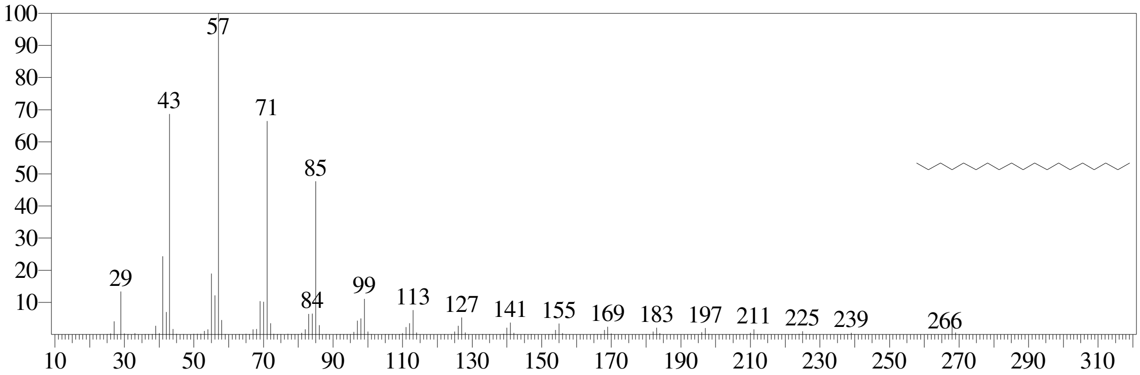

Hit#:2 Entry:122857 Library:NIST23-1.lib
SI:97 Formula:C18H38 CAS:593-45-3 MolWeight:254 RetIndex:1802
CompName:Octadecane $$ n-Octadecane $$ Octadecan $$

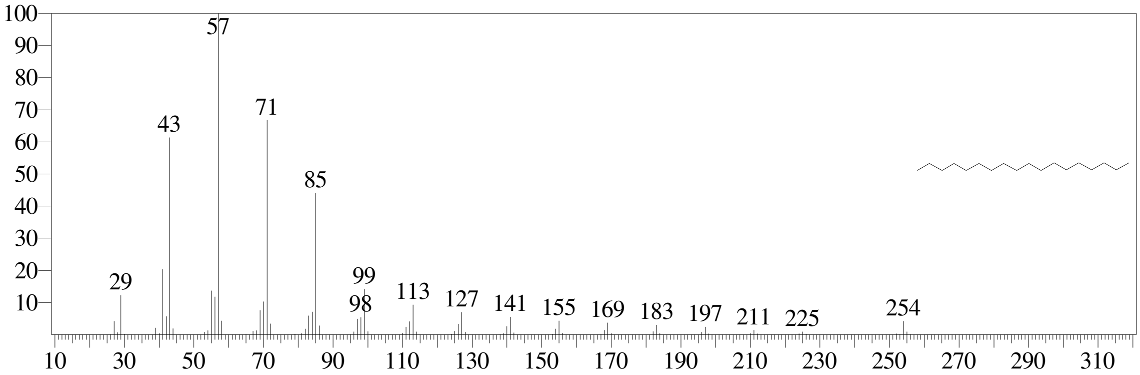

<< Target >>
Line#:13   R.Time:43.858(Scan#:4964) MassPeaks:65
RawMode:Averaged 43.850-43.867(4963-4965) BasePeak:57.05(725925)
BG Mode:Calc. from Peak Group 1 - Event 1 Scan

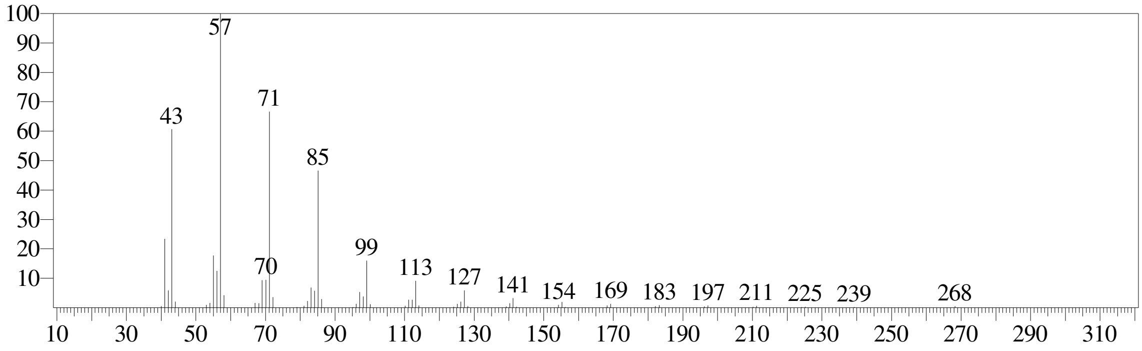

Hit#:3 Entry:105397 Library:NIST23-1.lib
SI:97 Formula:C17H36 CAS:629-78-7 MolWeight:240 RetIndex:1701
CompName:Heptadecane $$ n-Heptadecane $$ Normal-heptadecane $$

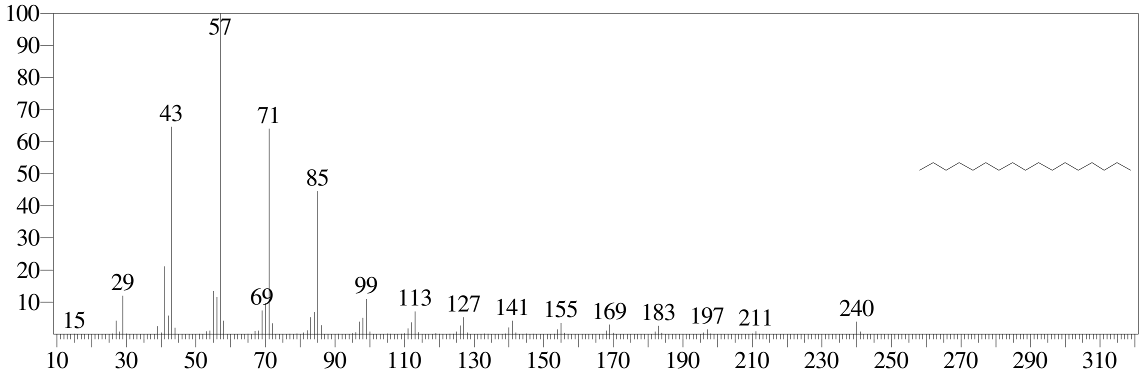

Hit#:4 Entry:37977 Library:NIST23s.lib
SI:97 Formula:C21H44 CAS:629-94-7 MolWeight:296 RetIndex:2103
CompName:Heneicosane $$ n-Heneicosane $$ Henicosane # $$

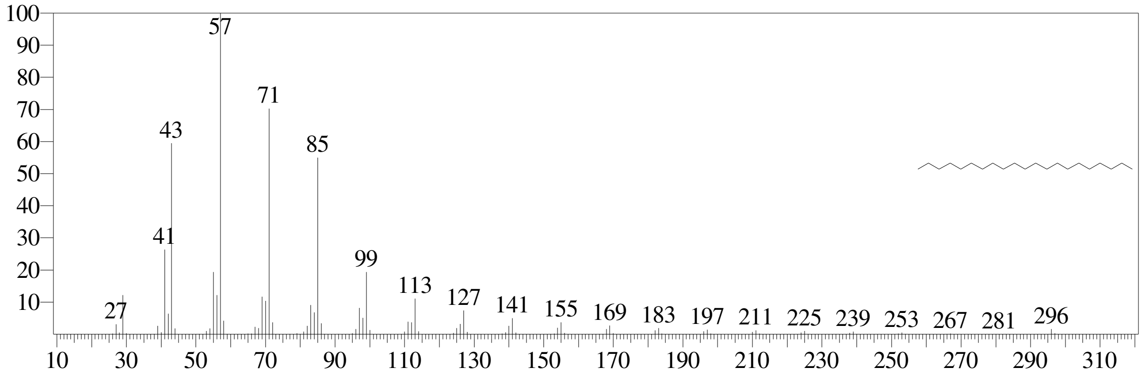

<< Target >>
Line#:13 R.Time:43.858(Scan#:4964) MassPeaks:65
RawMode:Averaged 43.850-43.867(4963-4965) BasePeak:57.05(725925)
BG Mode:Calc. from Peak Group 1 - Event 1 Scan

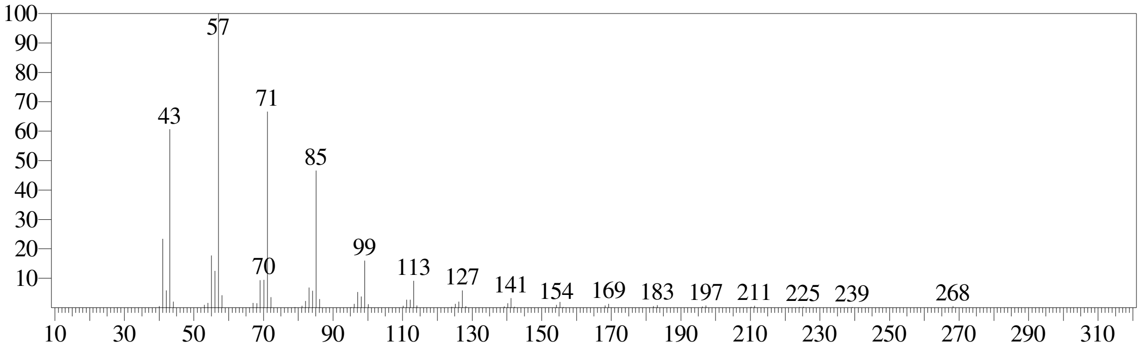

Hit#:5 Entry:39191 Library:NIST23s.lib
SI:96 Formula:C22H46 CAS:629-97-0 MolWeight:310 RetIndex:2203
CompName:Docosane $$ n-Docosane $$ Normal-docosane $$

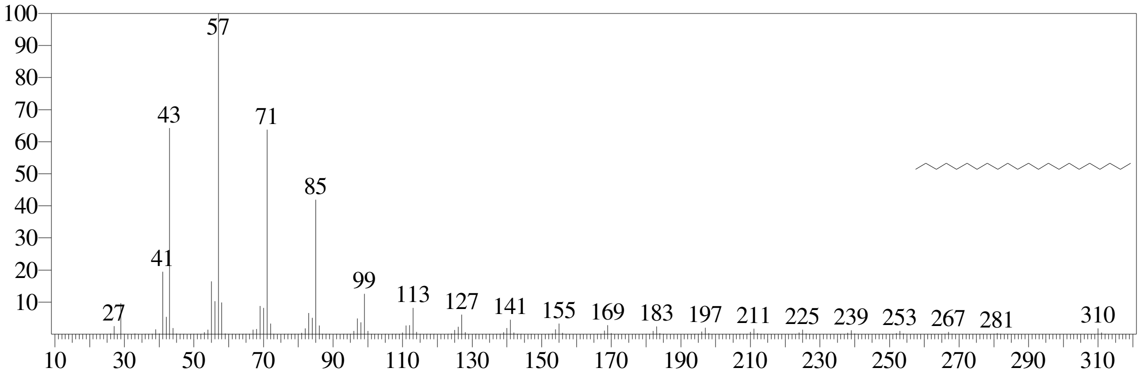

<< Target >>
Line#:14   R.Time:47.050(Scan#:5347) MassPeaks:67
RawMode:Averaged 47.042-47.058(5346-5348) BasePeak:57.05(719659)
BG Mode:Calc. from Peak Group 1 - Event 1 Scan

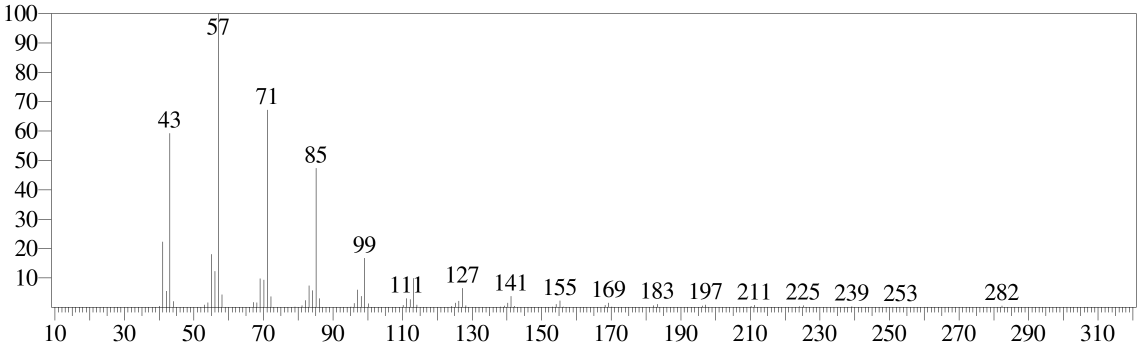

Hit#:1 Entry:37977 Library:NIST23s.lib
SI:97 Formula:C21H44 CAS:629-94-7 MolWeight:296 RetIndex:2103
CompName:Heneicosane $$ n-Heneicosane $$ Henicosane # $$

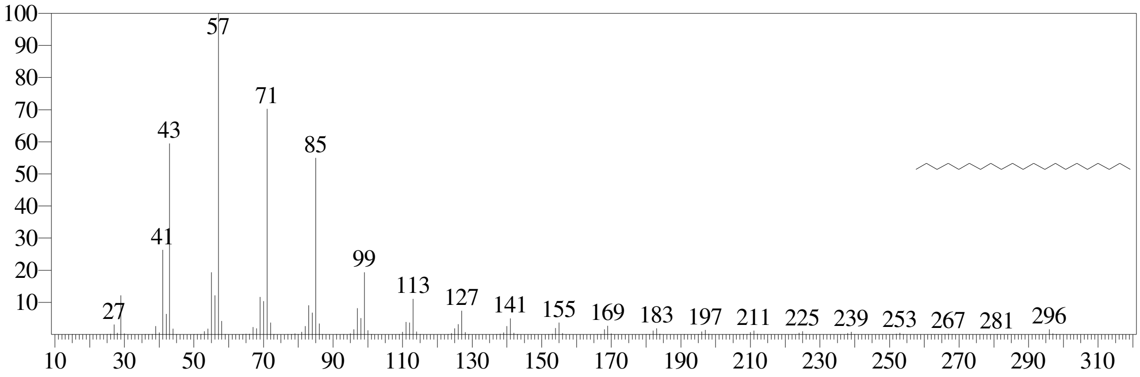

Hit#:2 Entry:141042 Library:NIST23-1.lib
SI:97 Formula:C19H40 CAS:629-92-5 MolWeight:268 RetIndex:1903
CompName:Nonadecane $$ n-Nonadecane $$

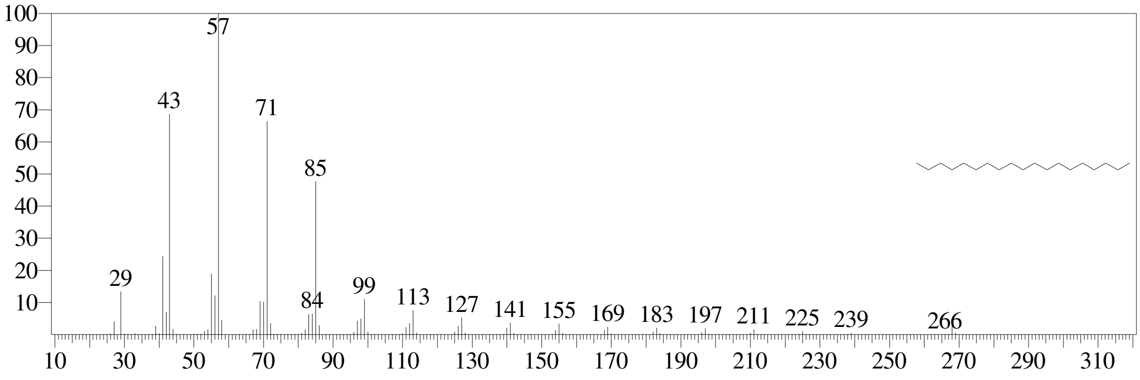

<< Target >>
Line#:14 R.Time:47.050(Scan#:5347) MassPeaks:67
RawMode:Averaged 47.042-47.058(5346-5348) BasePeak:57.05(719659)
BG Mode:Calc. from Peak Group 1 - Event 1 Scan

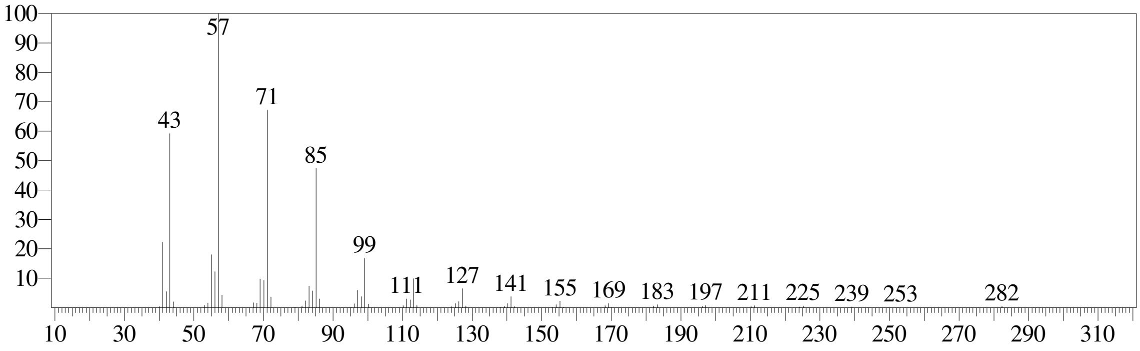

Hit#:3 Entry:122857 Library:NIST23-1.lib
SI:97 Formula:C18H38 CAS:593-45-3 MolWeight:254 RetIndex:1802
CompName:Octadecane $$ n-Octadecane $$ Octadecan $$

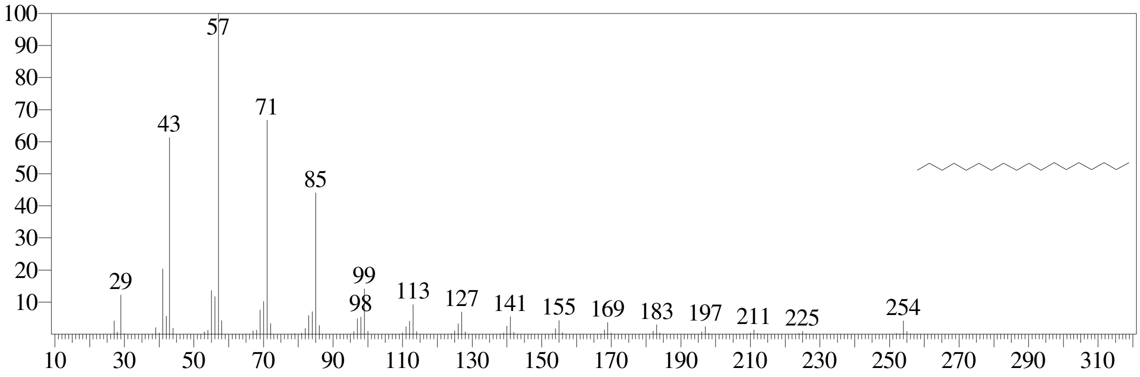

Hit#:4 Entry:39191 Library:NIST23s.lib
SI:96 Formula:C22H46 CAS:629-97-0 MolWeight:310 RetIndex:2203
CompName:Docosane $$ n-Docosane $$ Normal-docosane $$

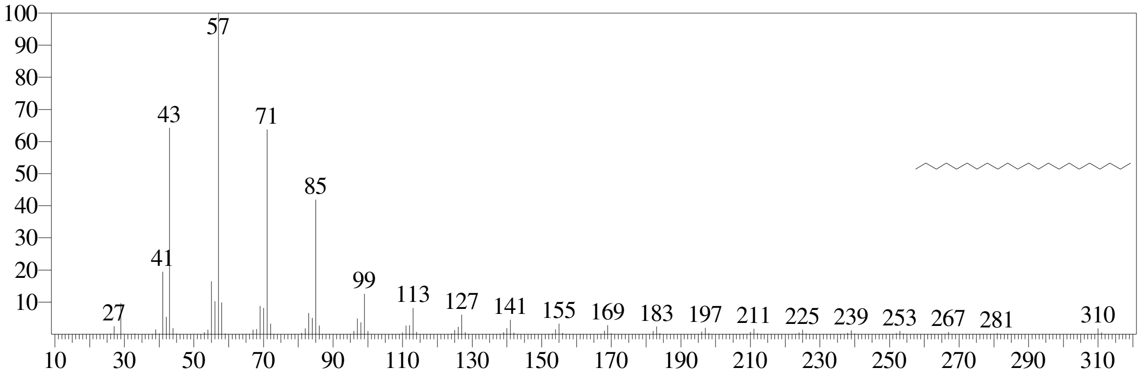

<< Target >>
Line#:14 R.Time:47.050(Scan#:5347) MassPeaks:67
RawMode:Averaged 47.042-47.058(5346-5348) BasePeak:57.05(719659)
BG Mode:Calc. from Peak Group 1 - Event 1 Scan

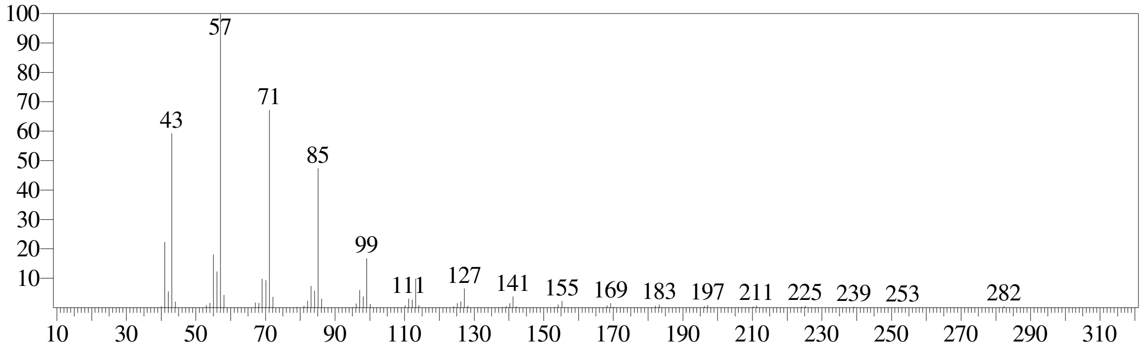

Hit#:5 Entry:105397 Library:NIST23-1.lib
SI:96 Formula:C17H36 CAS:629-78-7 MolWeight:240 RetIndex:1701
CompName:Heptadecane $$ n-Heptadecane $$ Normal-heptadecane $$

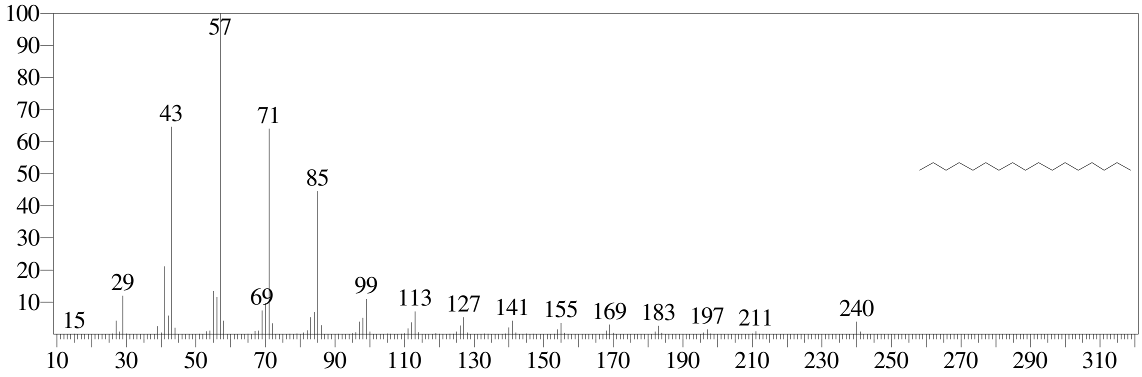

<< Target >>
Line#:15   R.Time:50.108(Scan#:5714) MassPeaks:68
RawMode:Averaged 50.100-50.117(5713-5715) BasePeak:57.05(701803)
BG Mode:Calc. from Peak Group 1 - Event 1 Scan

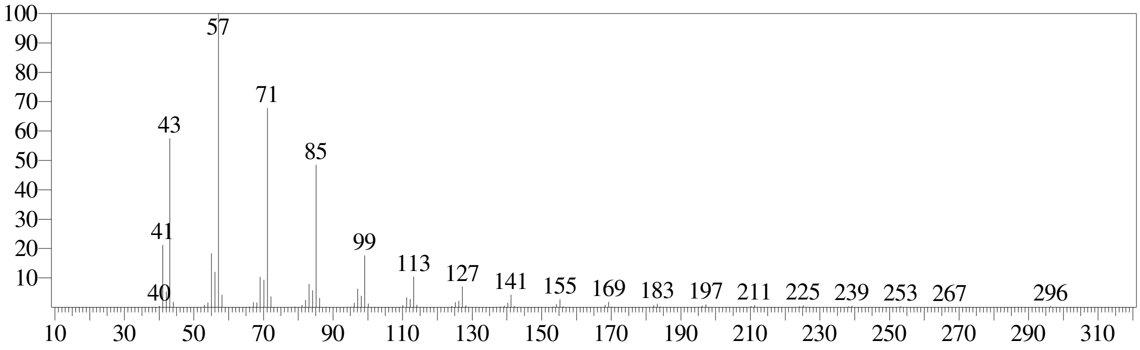

Hit#:1 Entry:37977 Library:NIST23s.lib
SI:97 Formula:C21H44 CAS:629-94-7 MolWeight:296 RetIndex:2103
CompName:Heneicosane $$ n-Heneicosane $$ Henicosane # $$

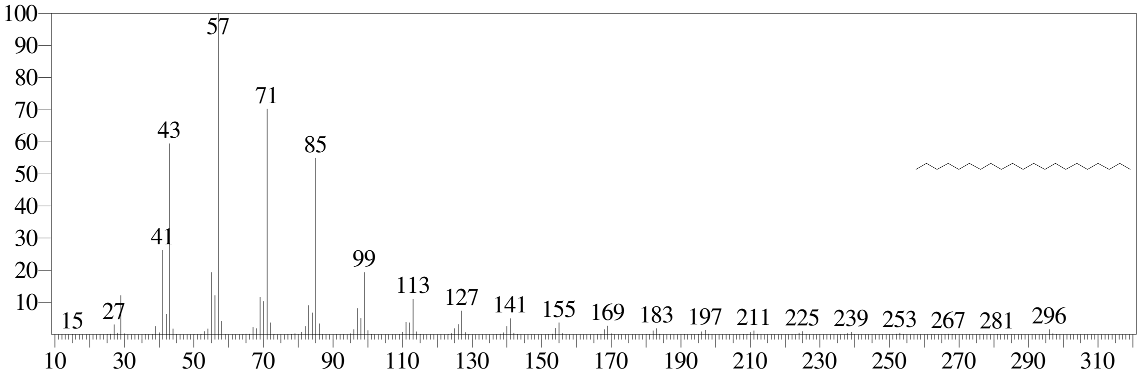

Hit#:2 Entry:141042 Library:NIST23-1.lib
SI:97 Formula:C19H40 CAS:629-92-5 MolWeight:268 RetIndex:1903
CompName:Nonadecane $$ n-Nonadecane $$

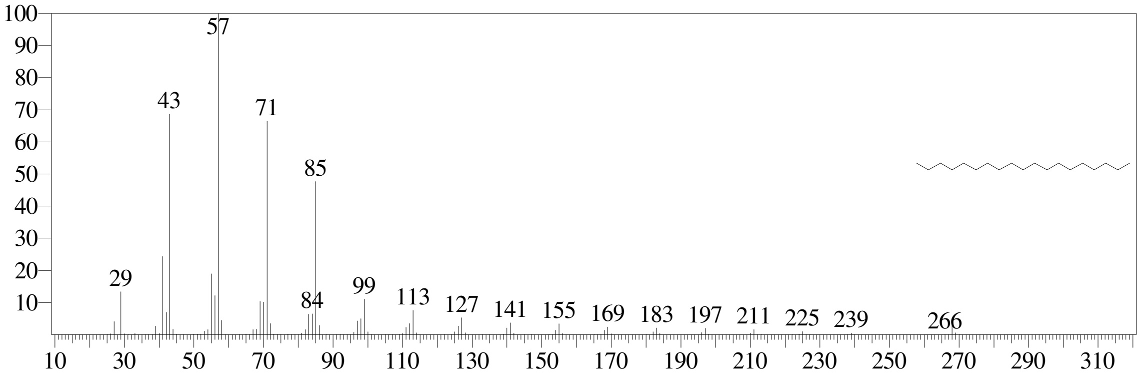

<< Target >>
Line#:15   R.Time:50.108(Scan#:5714) MassPeaks:68
RawMode:Averaged 50.100-50.117(5713-5715) BasePeak:57.05(701803)
BG Mode:Calc. from Peak Group 1 - Event 1 Scan

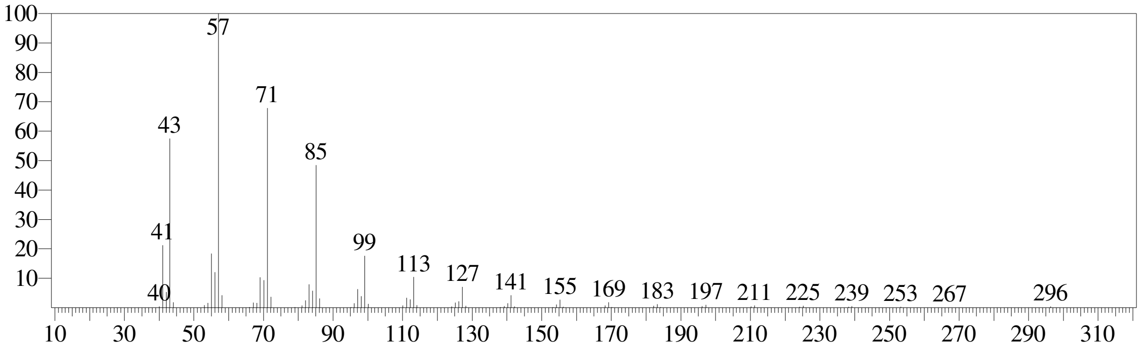

Hit#:3 Entry:122857 Library:NIST23-1.lib
SI:97 Formula:C18H38 CAS:593-45-3 MolWeight:254 RetIndex:1802
CompName:Octadecane $$ n-Octadecane $$ Octadecan $$

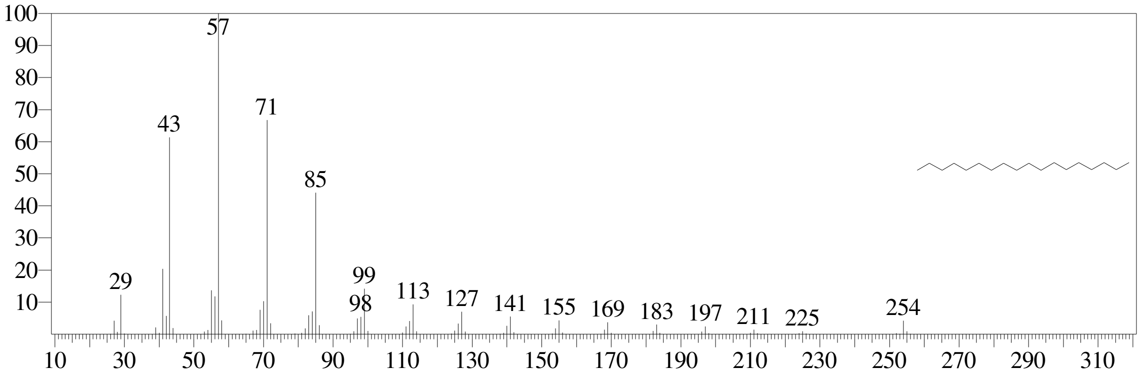

Hit#:4 Entry:178877 Library:NIST23-1.lib
SI:96 Formula:C21H44 CAS:629-94-7 MolWeight:296 RetIndex:2103
CompName:Heneicosane $$ n-Heneicosane $$ Henicosane # $$

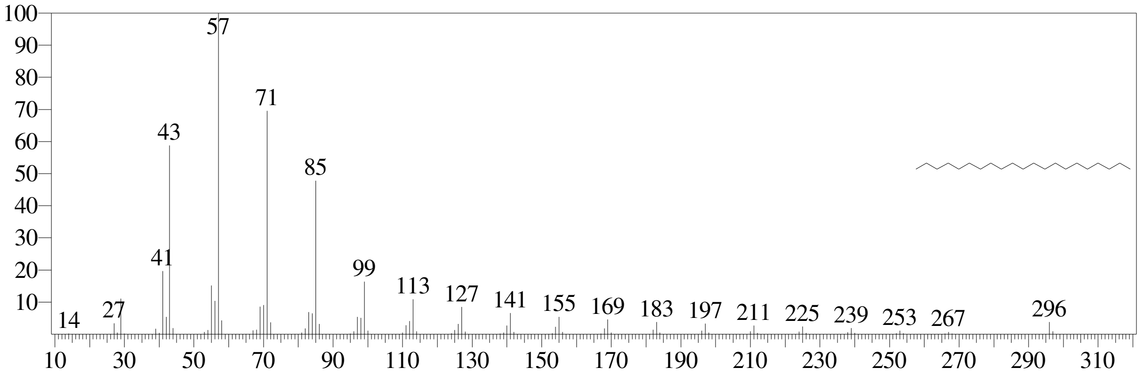

<< Target >>
Line#:15   R.Time:50.108(Scan#:5714) MassPeaks:68
RawMode:Averaged 50.100-50.117(5713-5715) BasePeak:57.05(701803)
BG Mode:Calc. from Peak Group 1 - Event 1 Scan

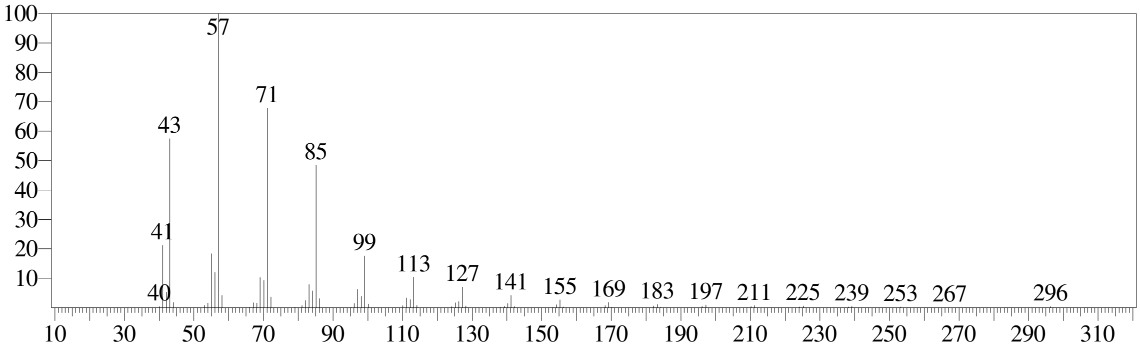

Hit#:5 Entry:39191 Library:NIST23s.lib
SI:96 Formula:C22H46 CAS:629-97-0 MolWeight:310 RetIndex:2203
CompName:Docosane $$ n-Docosane $$ Normal-docosane $$

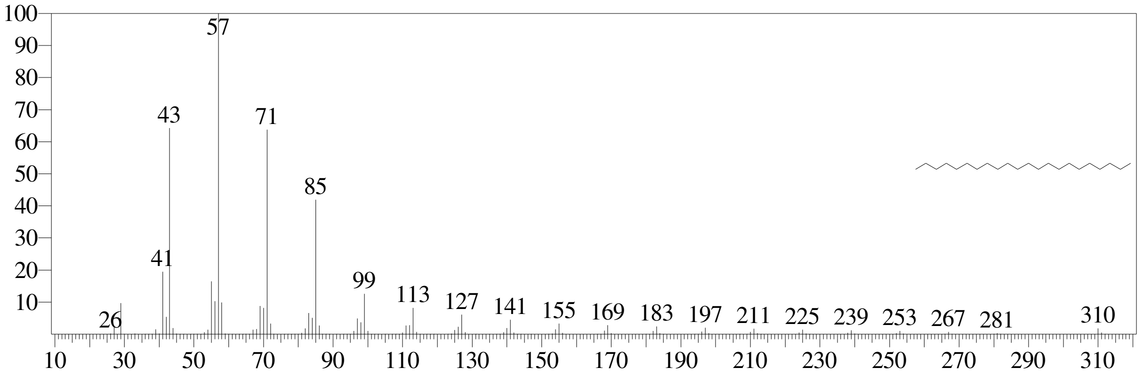

<< Target >>
Line#:16 R.Time:53.042(Scan#:6066) MassPeaks:71
RawMode:Averaged 53.033-53.050(6065-6067) BasePeak:57.05(697559)
BG Mode:Calc. from Peak Group 1 - Event 1 Scan

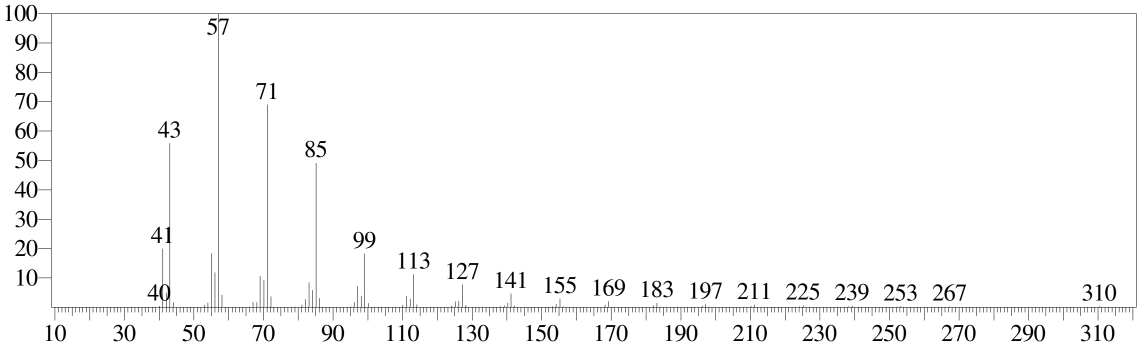

Hit#:1 Entry:37977 Library:NIST23s.lib
SI:98 Formula:C21H44 CAS:629-94-7 MolWeight:296 RetIndex:2103
CompName:Heneicosane $$ n-Heneicosane $$ Henicosane # $$

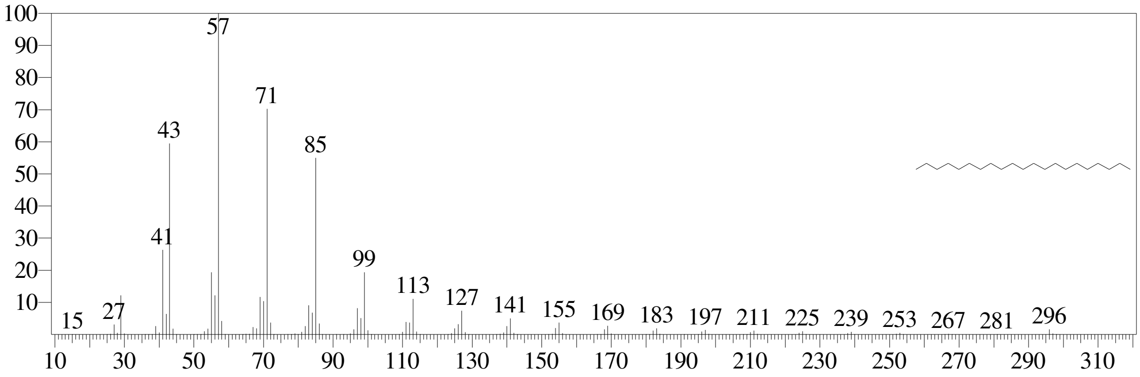

Hit#:2 Entry:178877 Library:NIST23-1.lib
SI:96 Formula:C21H44 CAS:629-94-7 MolWeight:296 RetIndex:2103
CompName:Heneicosane $$ n-Heneicosane $$ Henicosane # $$

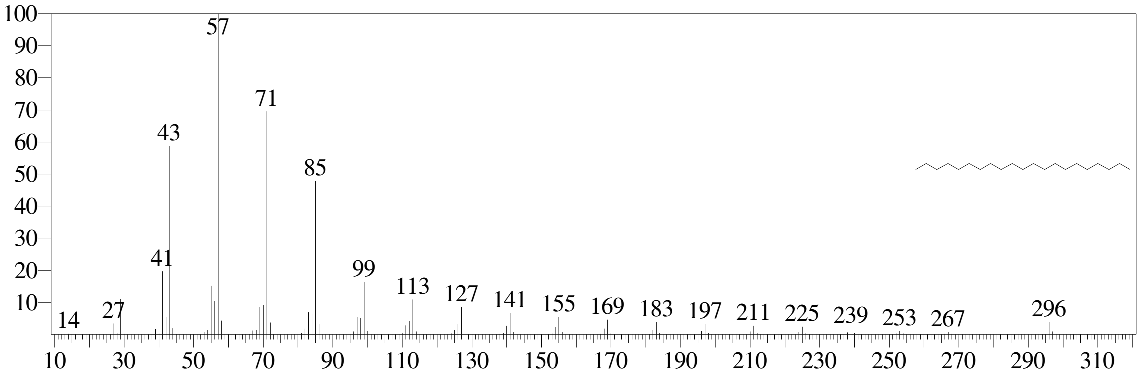

<< Target >>
Line#:16   R.Time:53.042(Scan#:6066) MassPeaks:71
RawMode:Averaged 53.033-53.050(6065-6067) BasePeak:57.05(697559)
BG Mode:Calc. from Peak Group 1 - Event 1 Scan

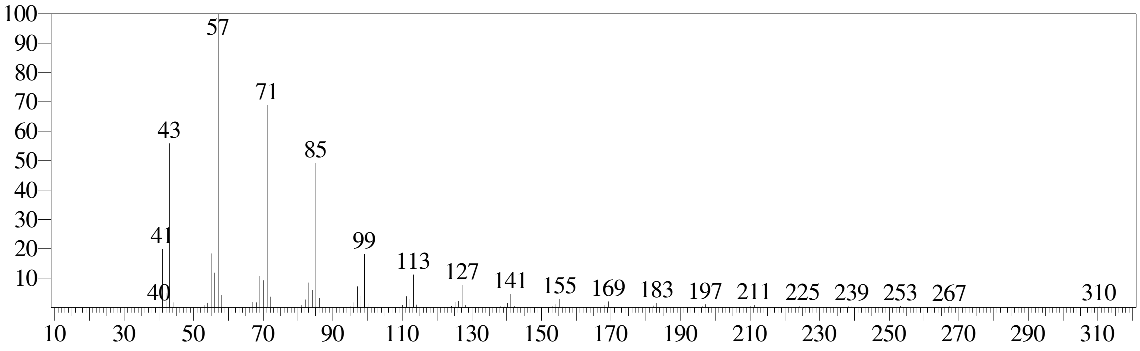

Hit#:3 Entry:39194 Library:NIST23s.lib
SI:96 Formula:C22H46 CAS:629-97-0 MolWeight:310 RetIndex:2203
CompName:Docosane $$ n-Docosane $$ Normal-docosane $$

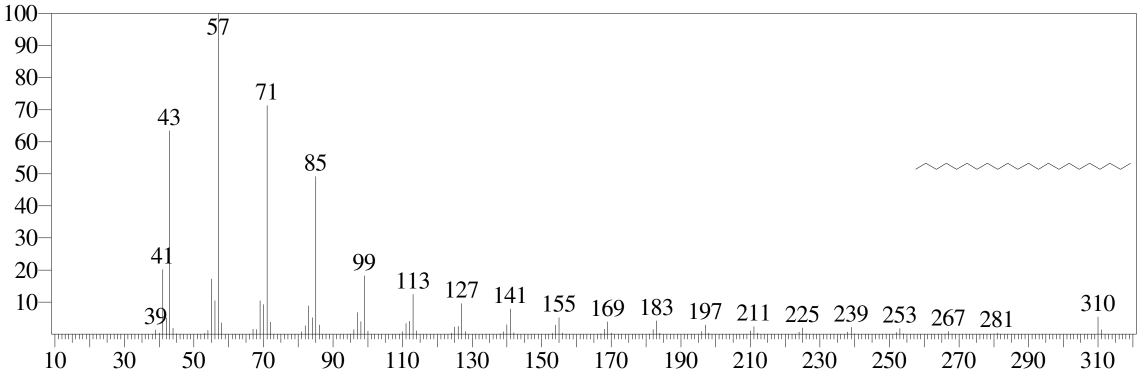

Hit#:4 Entry:141042 Library:NIST23-1.lib
SI:96 Formula:C19H40 CAS:629-92-5 MolWeight:268 RetIndex:1903
CompName:Nonadecane $$ n-Nonadecane $$

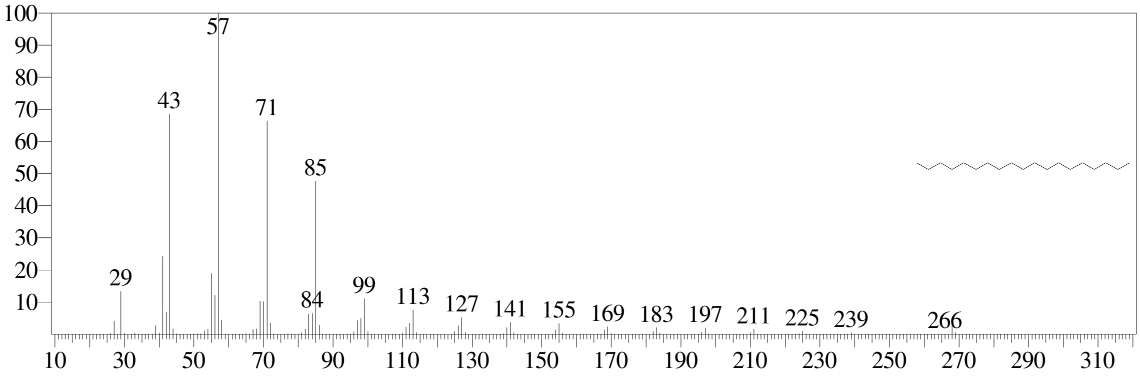

<< Target >>
Line#:16   R.Time:53.042(Scan#:6066) MassPeaks:71
RawMode:Averaged 53.033-53.050(6065-6067) BasePeak:57.05(697559)
BG Mode:Calc. from Peak Group 1 - Event 1 Scan

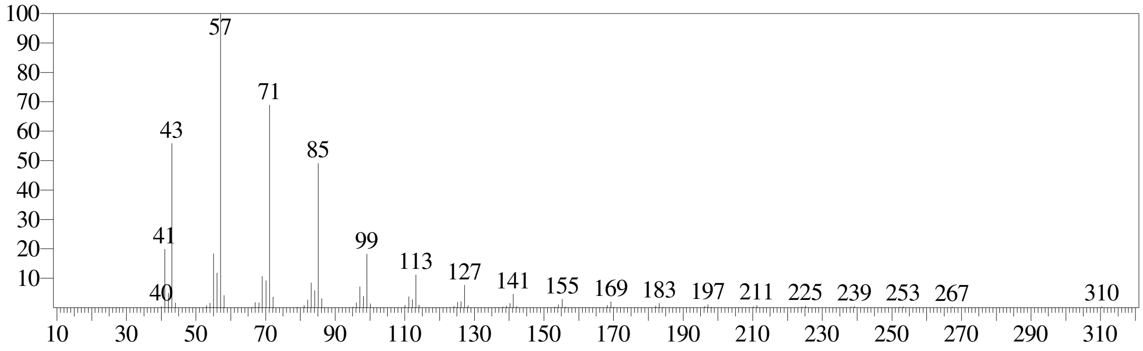

Hit#:5 Entry:122857 Library:NIST23-1.lib
SI:96 Formula:C18H38 CAS:593-45-3 MolWeight:254 RetIndex:1802
CompName:Octadecane $$ n-Octadecane $$ Octadecan $$

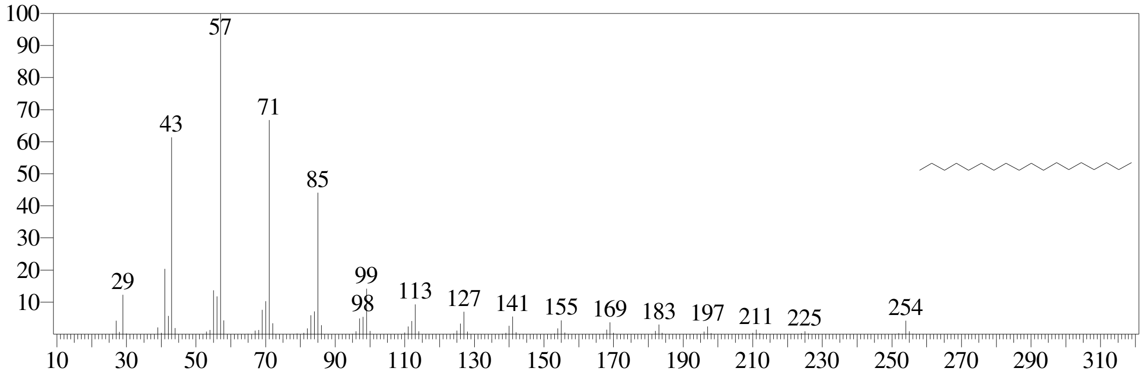

<< Target >>
Line#:17   R.Time:55.850(Scan#:6403) MassPeaks:74
RawMode:Averaged 55.842-55.858(6402-6404) BasePeak:57.05(707294)
BG Mode:Calc. from Peak Group 1 - Event 1 Scan

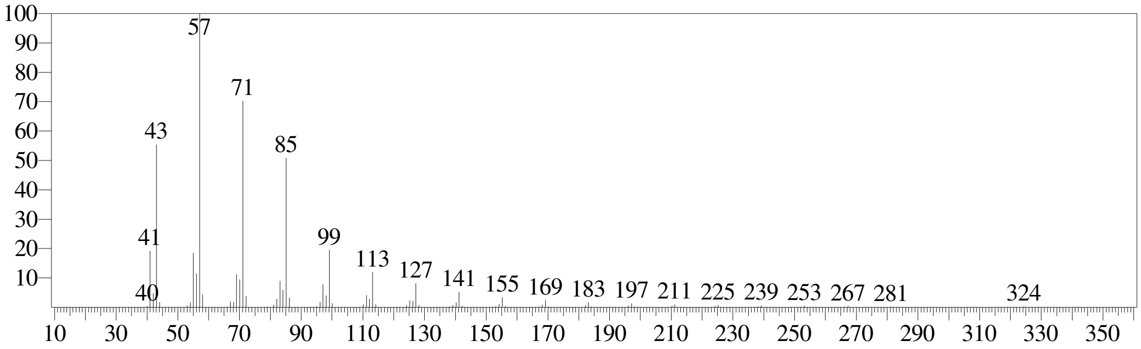

Hit#:1 Entry:37977 Library:NIST23s.lib
SI:98 Formula:C21H44 CAS:629-94-7 MolWeight:296 RetIndex:2103
CompName:Heneicosane $$ n-Heneicosane $$ Henicosane # $$

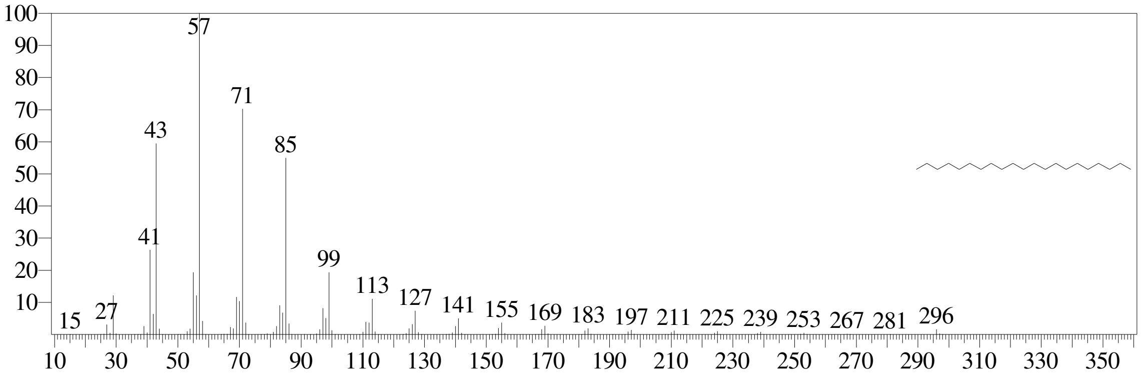

Hit#:2 Entry:39194 Library:NIST23s.lib
SI:96 Formula:C22H46 CAS:629-97-0 MolWeight:310 RetIndex:2203
CompName:Docosane $$ n-Docosane $$ Normal-docosane $$

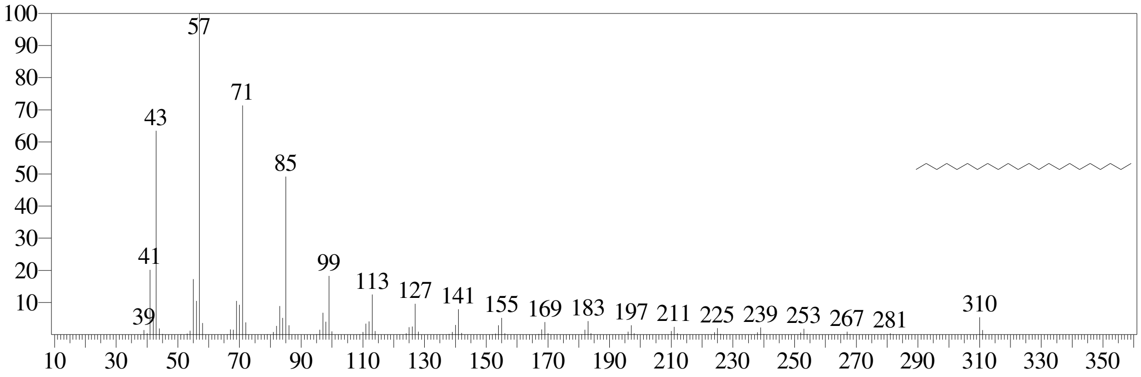

<< Target >>
Line#:17   R.Time:55.850(Scan#:6403) MassPeaks:74
RawMode:Averaged 55.842-55.858(6402-6404) BasePeak:57.05(707294)
BG Mode:Calc. from Peak Group 1 - Event 1 Scan

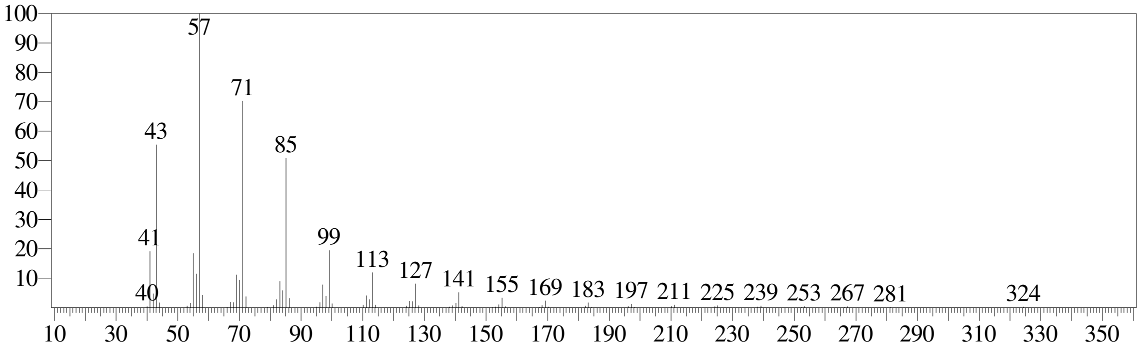

Hit#:3 Entry:2191 Library:NIST23-2.lib
SI:96 Formula:C25H52 CAS:629-99-2 MolWeight:352 RetIndex:2503
CompName:Pentacosane $$ n-Pentacosane $$

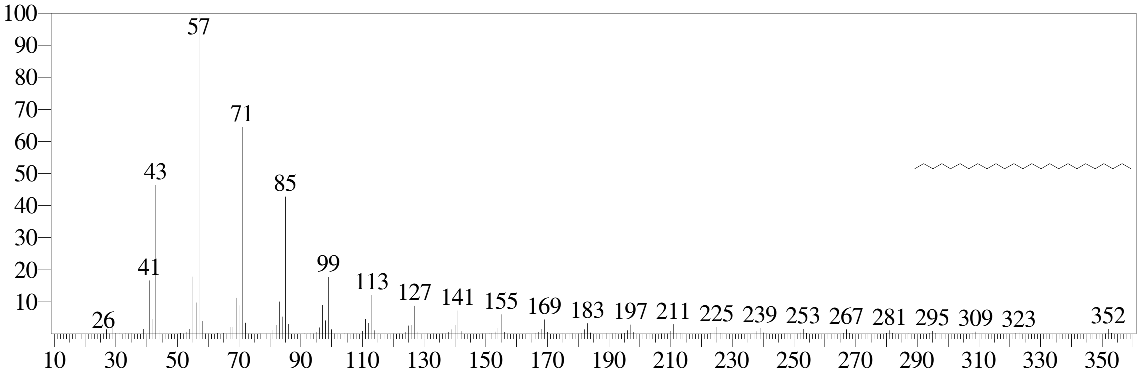

Hit#:4 Entry:178877 Library:NIST23-1.lib
SI:96 Formula:C21H44 CAS:629-94-7 MolWeight:296 RetIndex:2103
CompName:Heneicosane $$ n-Heneicosane $$ Henicosane # $$

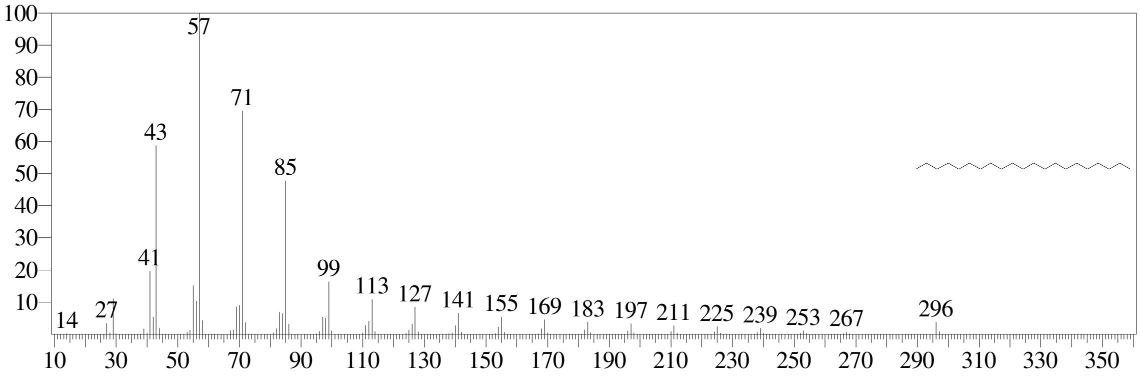

<< Target >>
Line#:17 R.Time:55.850(Scan#:6403) MassPeaks:74
RawMode:Averaged 55.842-55.858(6402-6404) BasePeak:57.05(707294)
BG Mode:Calc. from Peak Group 1 - Event 1 Scan

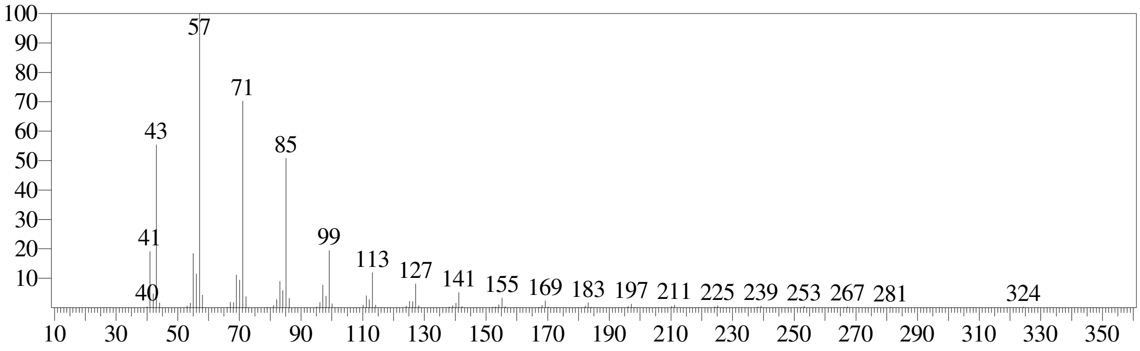

Hit#:5 Entry:233797 Library:NIST23-1.lib
SI:96 Formula:C24H50 CAS:646-31-1 MolWeight:338 RetIndex:2404
CompName:Tetracosane $$ n-Tetracosane $$

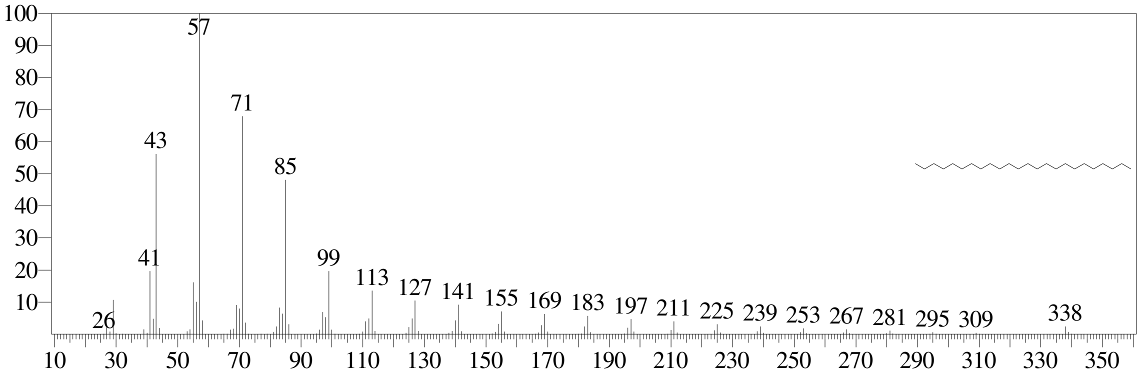

<< Target >>
Line#:18   R.Time:58.550(Scan#:6727) MassPeaks:78
RawMode:Averaged 58.542-58.558(6726-6728) BasePeak:57.05(705162)
BG Mode:Calc. from Peak Group 1 - Event 1 Scan

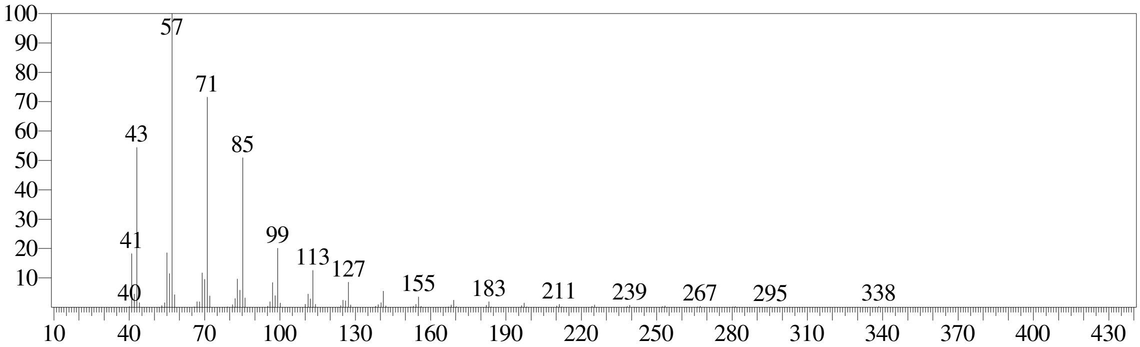

Hit#:1 Entry:37977 Library:NIST23s.lib
SI:98 Formula:C21H44 CAS:629-94-7 MolWeight:296 RetIndex:2103
CompName:Heneicosane $$ n-Heneicosane $$ Henicosane # $$

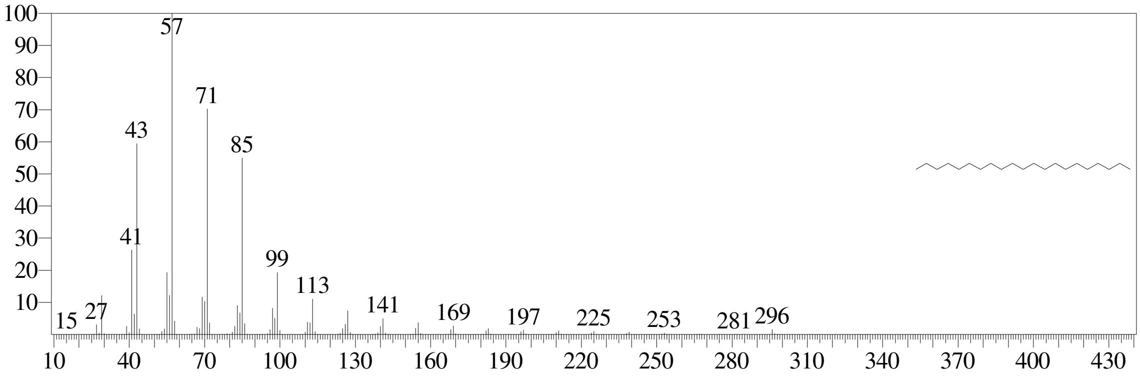

Hit#:2 Entry:2191 Library:NIST23-2.lib
SI:96 Formula:C25H52 CAS:629-99-2 MolWeight:352 RetIndex:2503
CompName:Pentacosane $$ n-Pentacosane $$

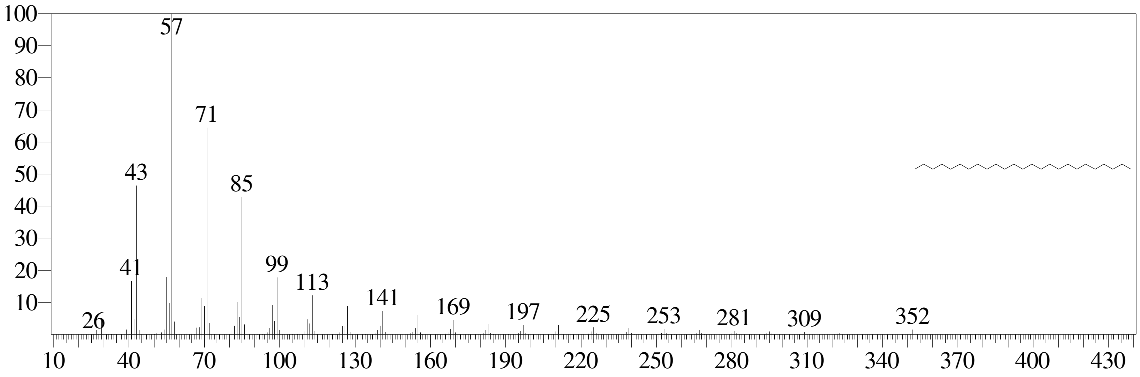

<< Target >>
Line#:18   R.Time:58.550(Scan#:6727) MassPeaks:78
RawMode:Averaged 58.542-58.558(6726-6728) BasePeak:57.05(705162)
BG Mode:Calc. from Peak Group 1 - Event 1 Scan

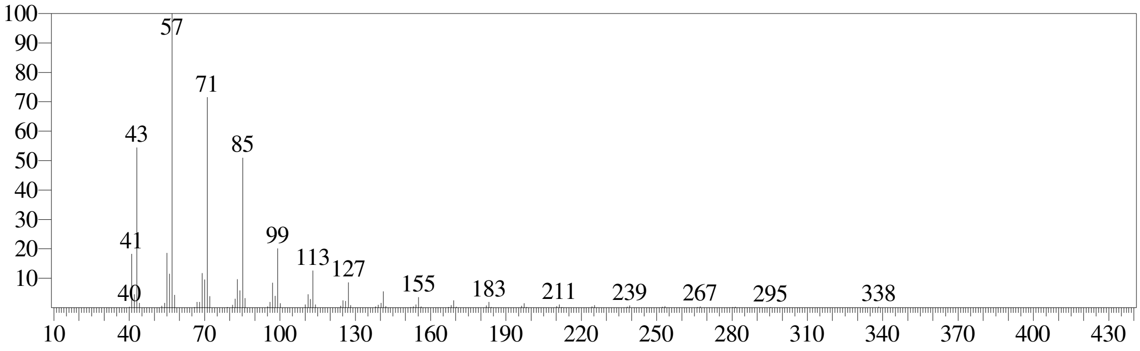

Hit#:3 Entry:39194 Library:NIST23s.lib
SI:96 Formula:C22H46 CAS:629-97-0 MolWeight:310 RetIndex:2203
CompName:Docosane $$ n-Docosane $$ Normal-docosane $$

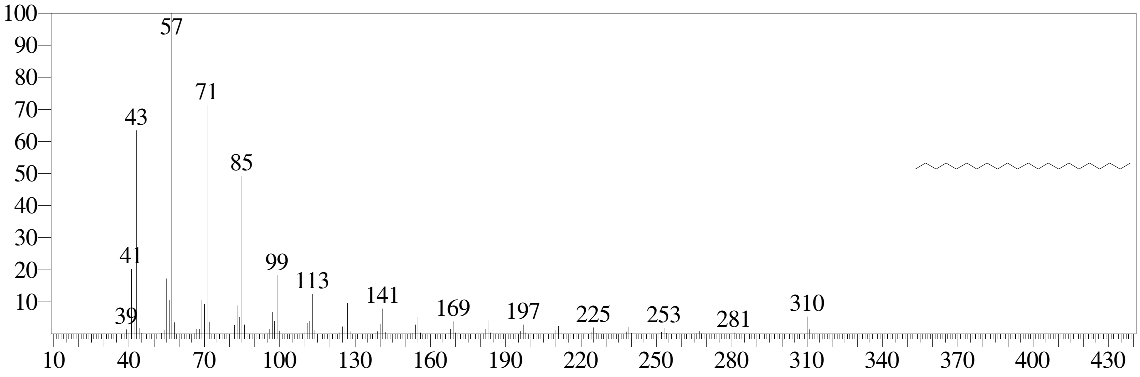

Hit#:4 Entry:178877 Library:NIST23-1.lib
SI:96 Formula:C21H44 CAS:629-94-7 MolWeight:296 RetIndex:2103
CompName:Heneicosane $$ n-Heneicosane $$ Henicosane # $$

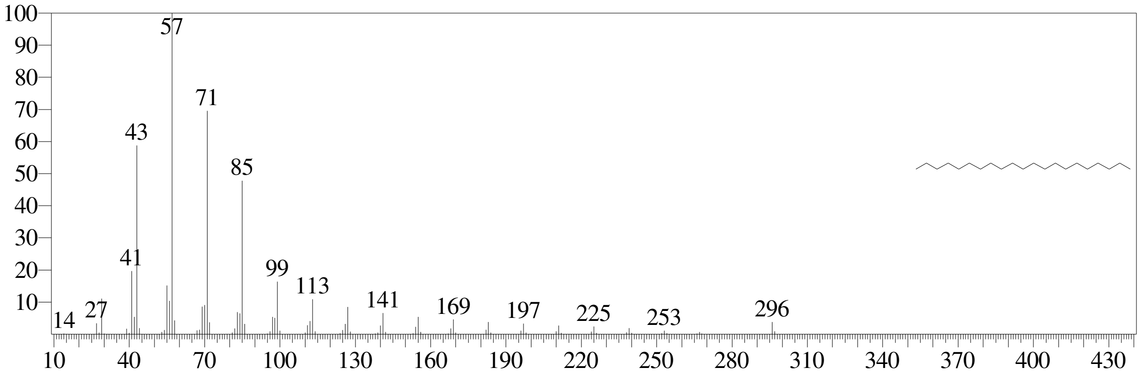

<< Target >>
Line#:18 R.Time:58.550(Scan#:6727) MassPeaks:78
RawMode:Averaged 58.542-58.558(6726-6728) BasePeak:57.05(705162)
BG Mode:Calc. from Peak Group 1 - Event 1 Scan

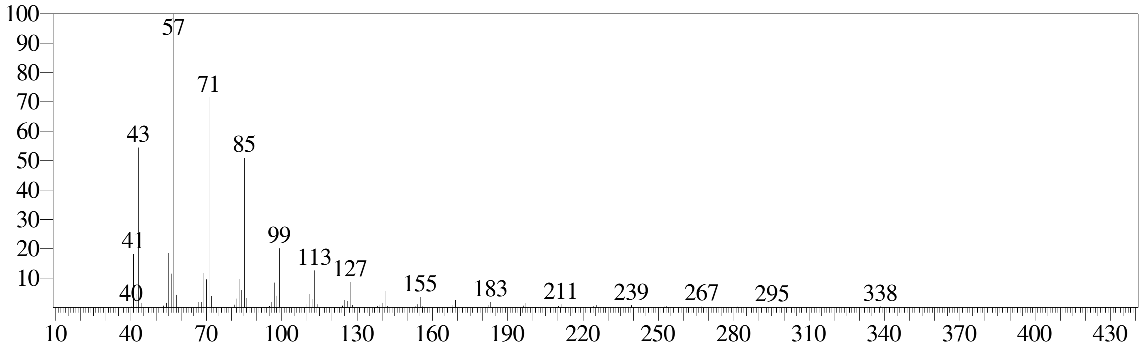

Hit#:5 Entry:65699 Library:NIST23-2.lib
SI:95 Formula:C31H64 CAS:630-04-6 MolWeight:436 RetIndex:3102
CompName:Hentriacontane $$ n-Hentriacontane $$ Untriacontane $$

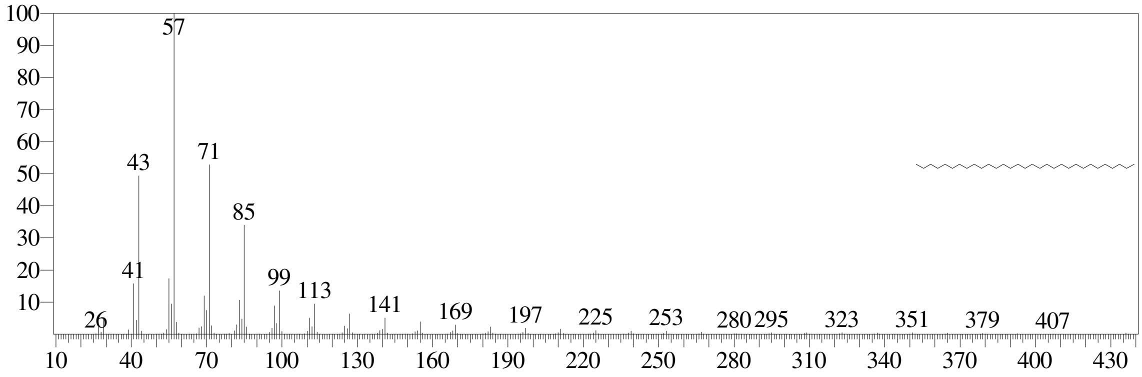

<< Target >>
Line#:19   R.Time:61.142(Scan#:7038) MassPeaks:83
RawMode:Averaged 61.133-61.150(7037-7039) BasePeak:57.05(696893)
BG Mode:Calc. from Peak Group 1 - Event 1 Scan

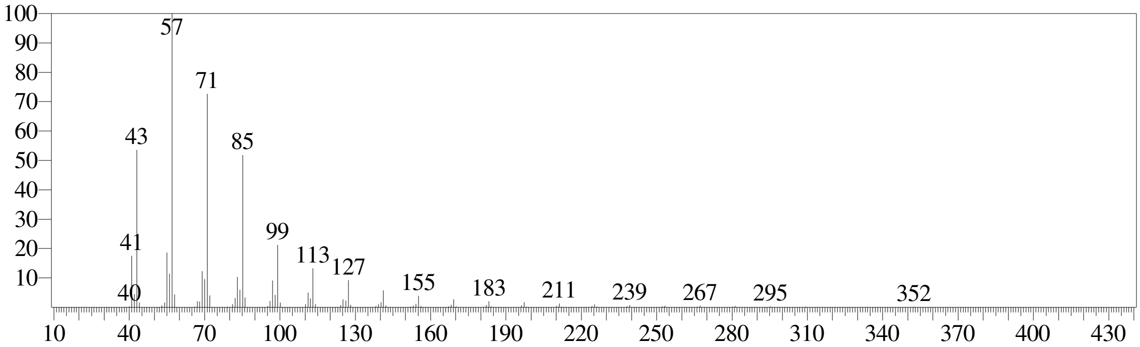

Hit#:1 Entry:37977 Library:NIST23s.lib
SI:97 Formula:C21H44 CAS:629-94-7 MolWeight:296 RetIndex:2103
CompName:Heneicosane $$ n-Heneicosane $$ Henicosane # $$

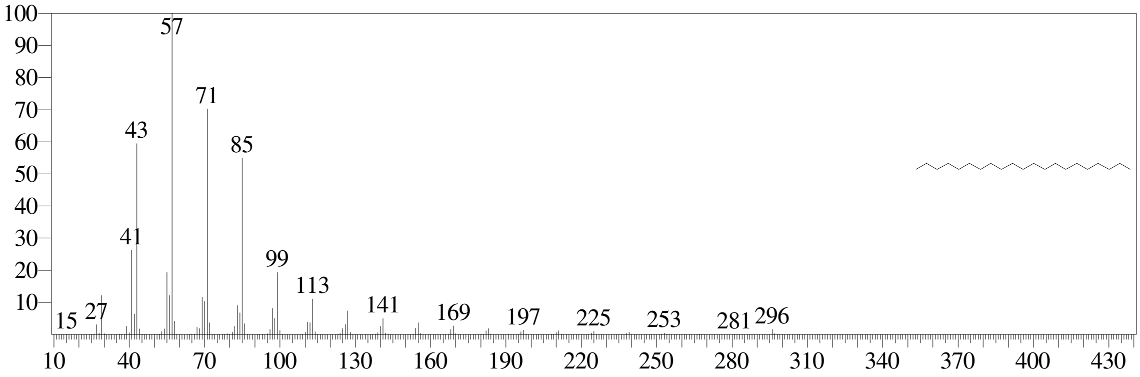

Hit#:2 Entry:2191 Library:NIST23-2.lib
SI:96 Formula:C25H52 CAS:629-99-2 MolWeight:352 RetIndex:2503
CompName:Pentacosane $$ n-Pentacosane $$

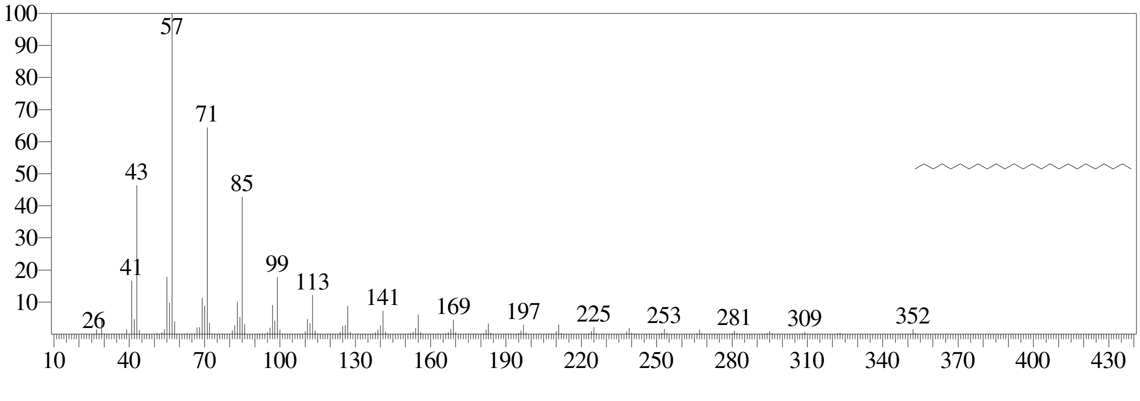

<< Target >>
Line#:19   R.Time:61.142(Scan#:7038) MassPeaks:83
RawMode:Averaged 61.133-61.150(7037-7039) BasePeak:57.05(696893)
BG Mode:Calc. from Peak Group 1 - Event 1 Scan

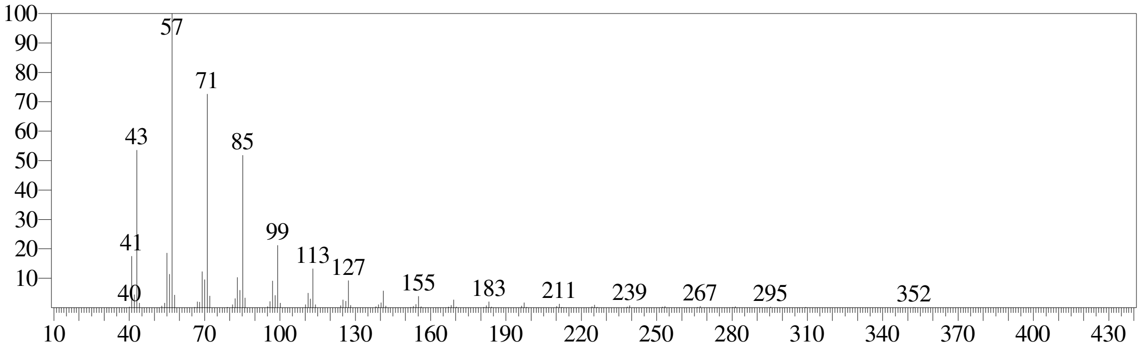

Hit#:3 Entry:39194 Library:NIST23s.lib
SI:96 Formula:C22H46 CAS:629-97-0 MolWeight:310 RetIndex:2203
CompName:Docosane $$ n-Docosane $$ Normal-docosane $$

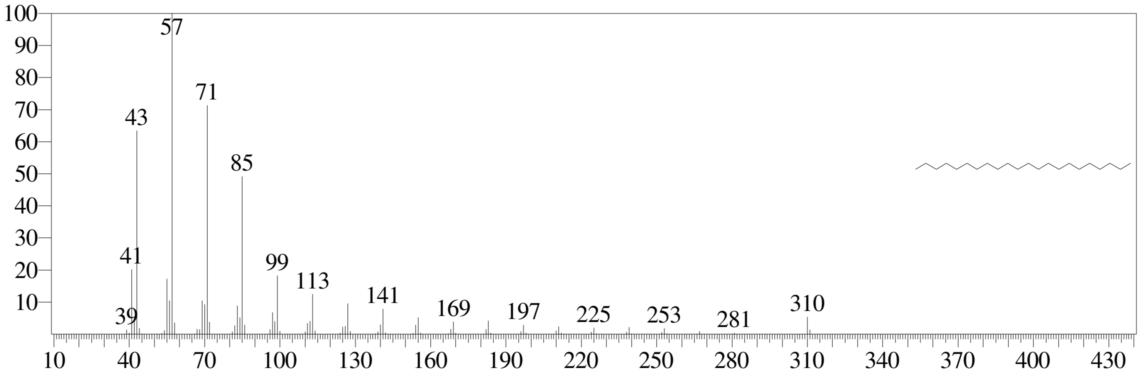

Hit#:4 Entry:65699 Library:NIST23-2.lib
SI:95 Formula:C31H64 CAS:630-04-6 MolWeight:436 RetIndex:3102
CompName:Hentriacontane $$ n-Hentriacontane $$ Untriacontane $$

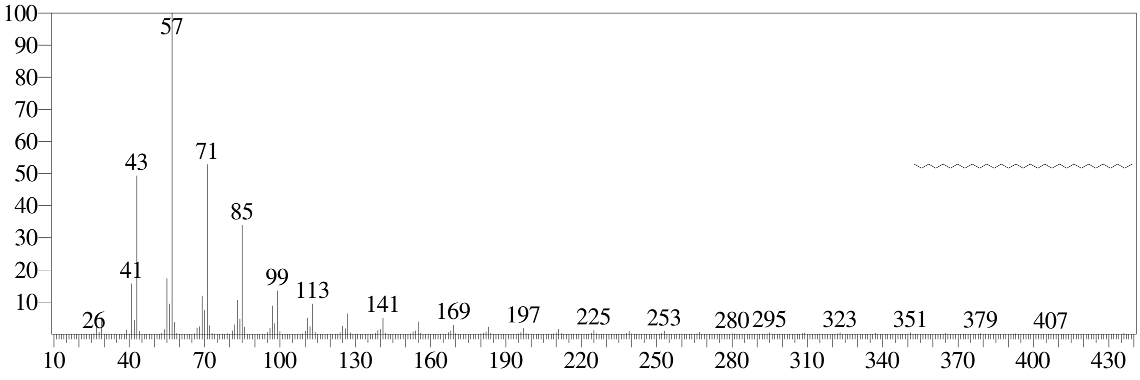

<< Target >>
Line#:19   R.Time:61.142(Scan#:7038) MassPeaks:83
RawMode:Averaged 61.133-61.150(7037-7039) BasePeak:57.05(696893)
BG Mode:Calc. from Peak Group 1 - Event 1 Scan

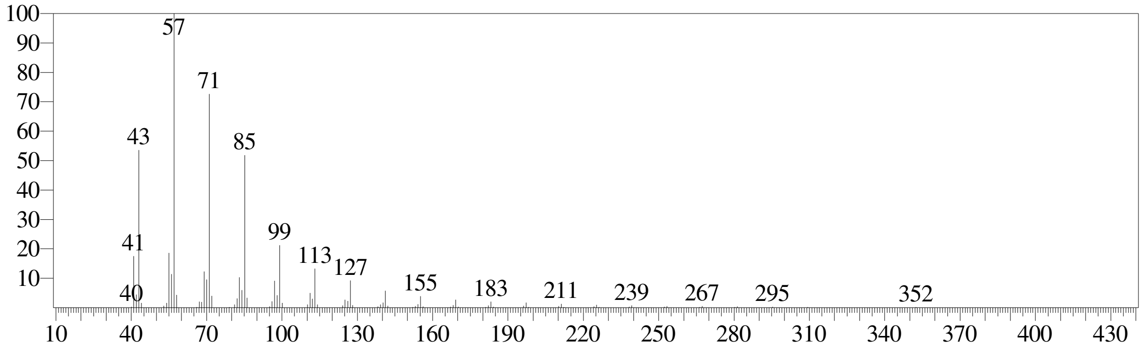

Hit#:5 Entry:178877 Library:NIST23-1.lib
SI:95 Formula:C21H44 CAS:629-94-7 MolWeight:296 RetIndex:2103
CompName:Heneicosane $$ n-Heneicosane $$ Henicosane # $$

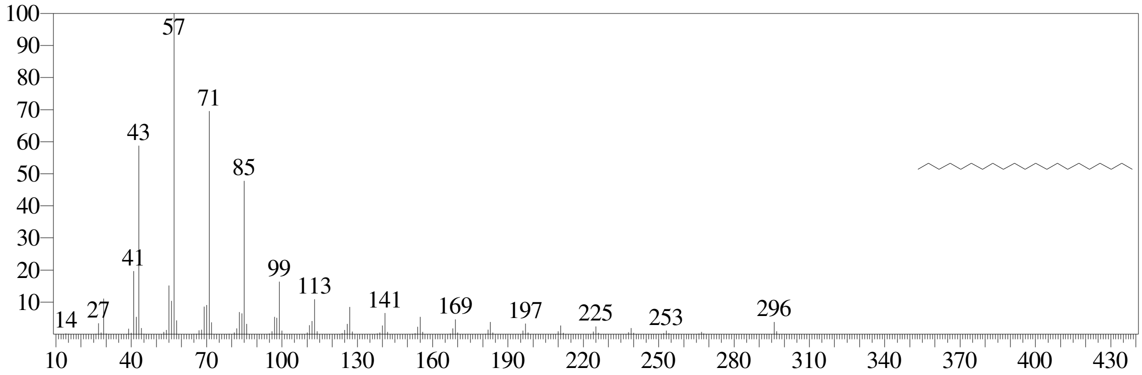

<< Target >>
Line#:20   R.Time:63.642(Scan#:7338) MassPeaks:86
RawMode:Averaged 63.633-63.650(7337-7339) BasePeak:57.05(702222)
BG Mode:Calc. from Peak Group 1 - Event 1 Scan

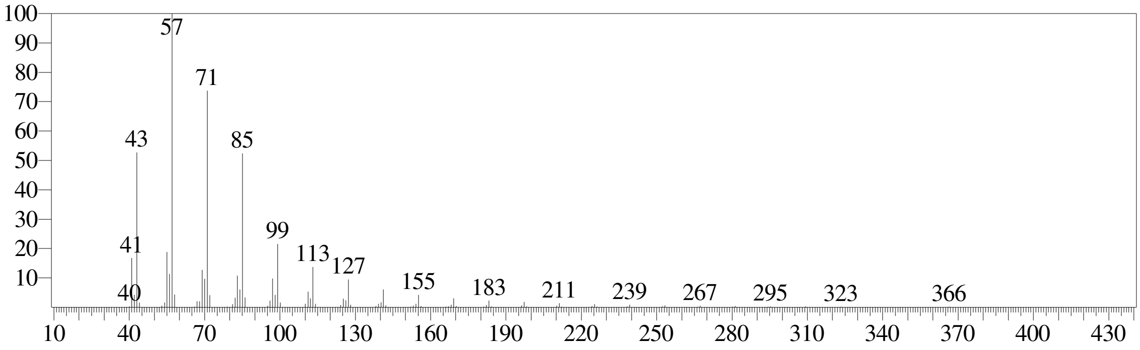

Hit#:1 Entry:37977 Library:NIST23s.lib
SI:97 Formula:C21H44 CAS:629-94-7 MolWeight:296 RetIndex:2103
CompName:Heneicosane $$ n-Heneicosane $$ Henicosane # $$

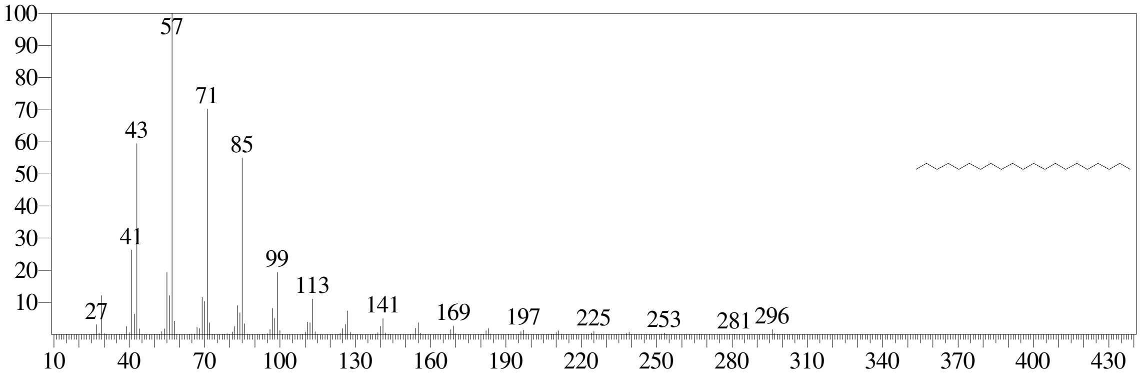

Hit#:2 Entry:2191 Library:NIST23-2.lib
SI:96 Formula:C25H52 CAS:629-99-2 MolWeight:352 RetIndex:2503
CompName:Pentacosane $$ n-Pentacosane $$

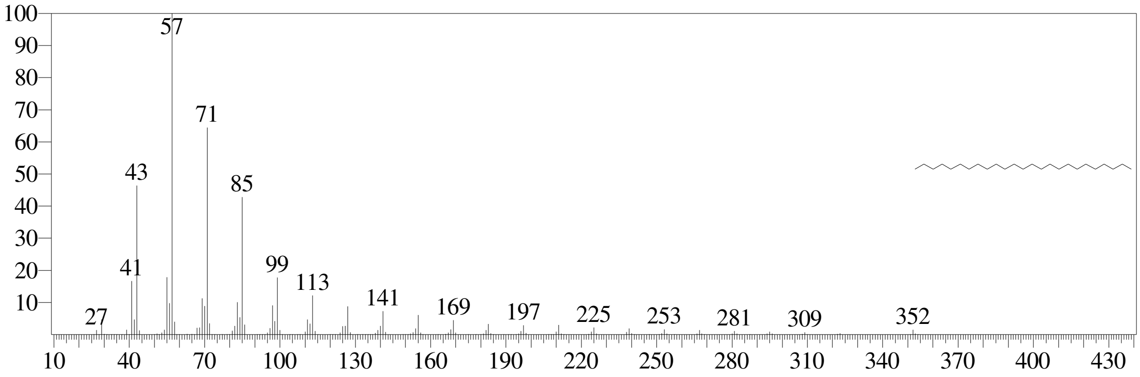

<< Target >>
Line#:20   R.Time:63.642(Scan#:7338) MassPeaks:86
RawMode:Averaged 63.633-63.650(7337-7339) BasePeak:57.05(702222)
BG Mode:Calc. from Peak Group 1 - Event 1 Scan

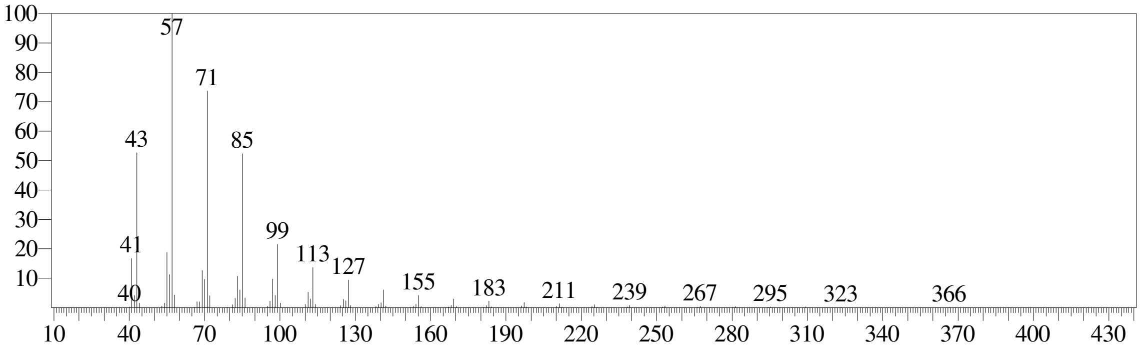

Hit#:3 Entry:39194 Library:NIST23s.lib
SI:96 Formula:C22H46 CAS:629-97-0 MolWeight:310 RetIndex:2203
CompName:Docosane $$ n-Docosane $$ Normal-docosane $$

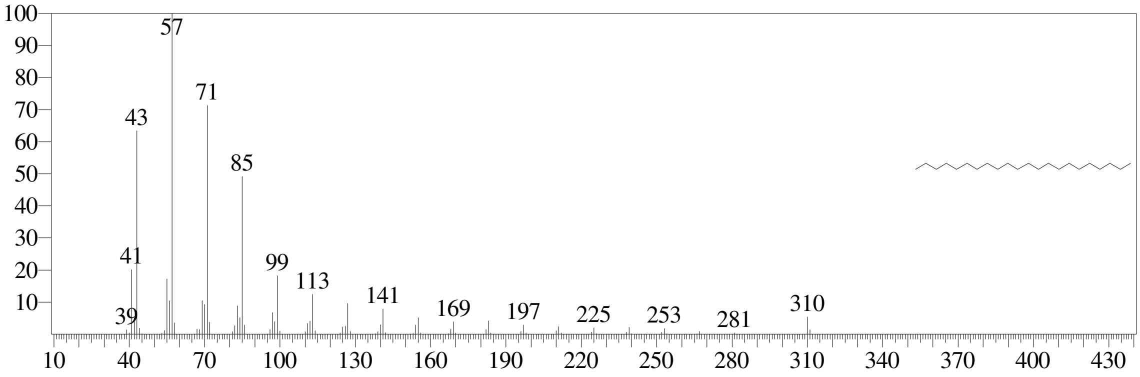

Hit#:4 Entry:65699 Library:NIST23-2.lib
SI:95 Formula:C31H64 CAS:630-04-6 MolWeight:436 RetIndex:3102
CompName:Hentriacontane $$ n-Hentriacontane $$ Untriacontane $$

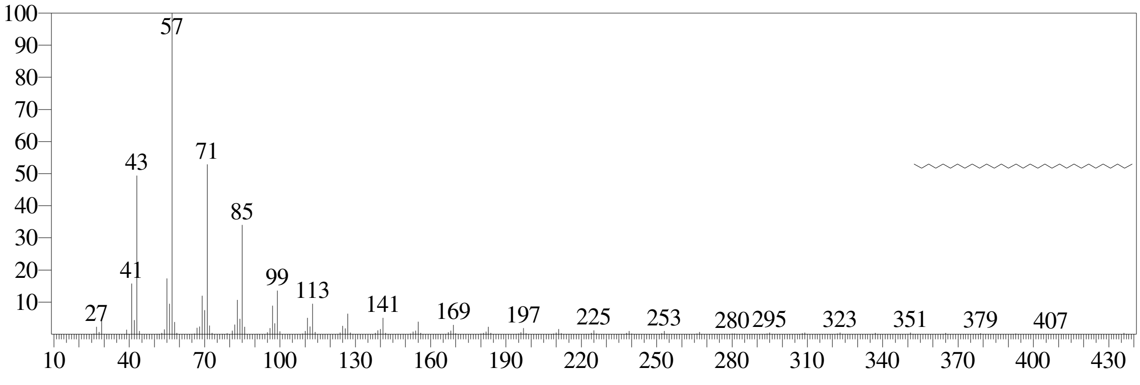

<< Target >>
Line#:20 R.Time:63.642(Scan#:7338) MassPeaks:86
RawMode:Averaged 63.633-63.650(7337-7339) BasePeak:57.05(702222)
BG Mode:Calc. from Peak Group 1 - Event 1 Scan

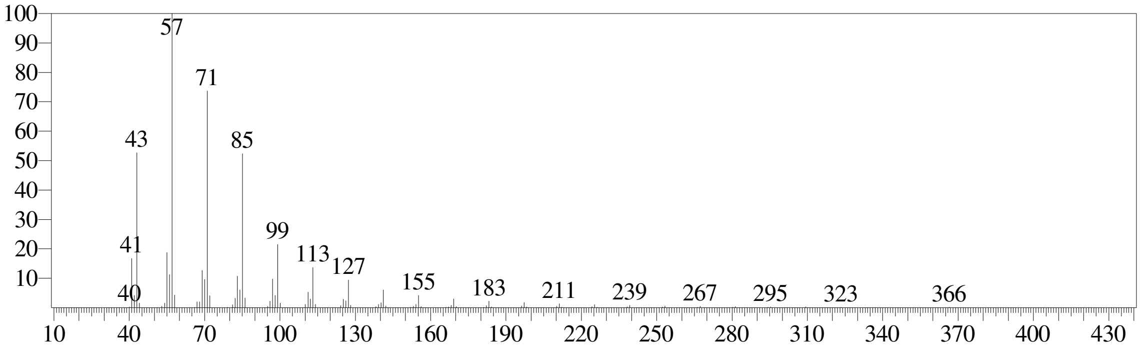

Hit#:5 Entry:43100 Library:NIST23s.lib
SI:95 Formula:C26H54 CAS:630-01-3 MolWeight:366 RetIndex:2604
CompName:Hexacosane $$ n-Hexacosane $$

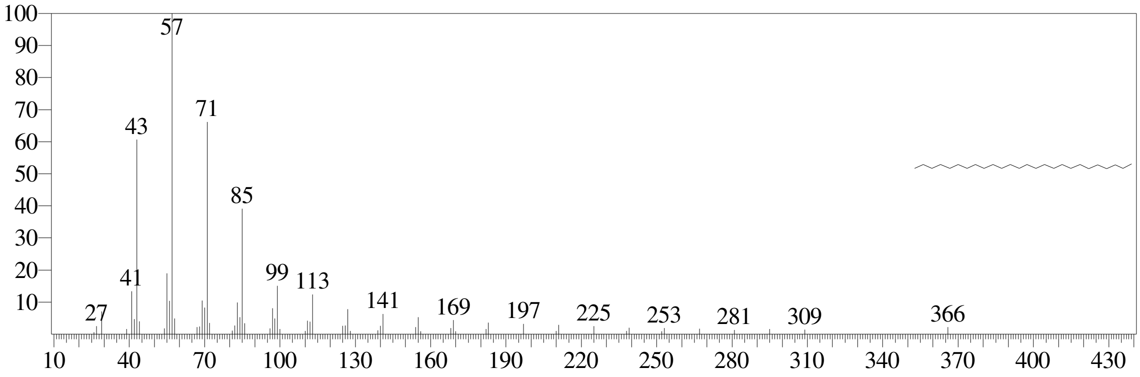

<< Target >>
Line#:21 R.Time:66.058(Scan#:7628) MassPeaks:88
RawMode:Averaged 66.050-66.067(7627-7629) BasePeak:57.05(666737)
BG Mode:Calc. from Peak Group 1 - Event 1 Scan

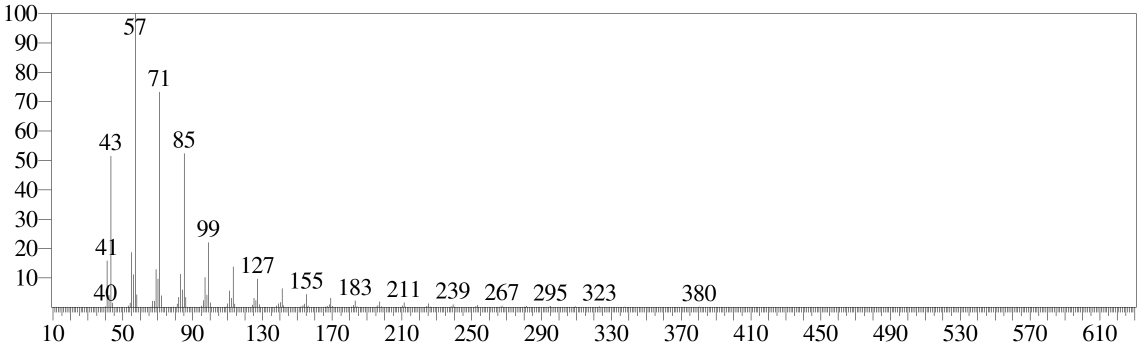

Hit#:1 Entry:37977 Library:NIST23s.lib
SI:96 Formula:C21H44 CAS:629-94-7 MolWeight:296 RetIndex:2103
CompName:Heneicosane $$ n-Heneicosane $$ Henicosane # $$

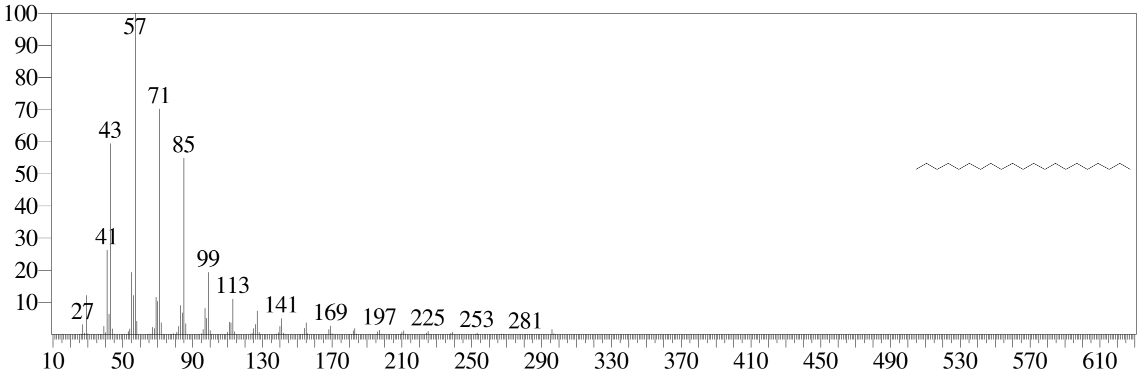

Hit#:2 Entry:2191 Library:NIST23-2.lib
SI:96 Formula:C25H52 CAS:629-99-2 MolWeight:352 RetIndex:2503
CompName:Pentacosane $$ n-Pentacosane $$

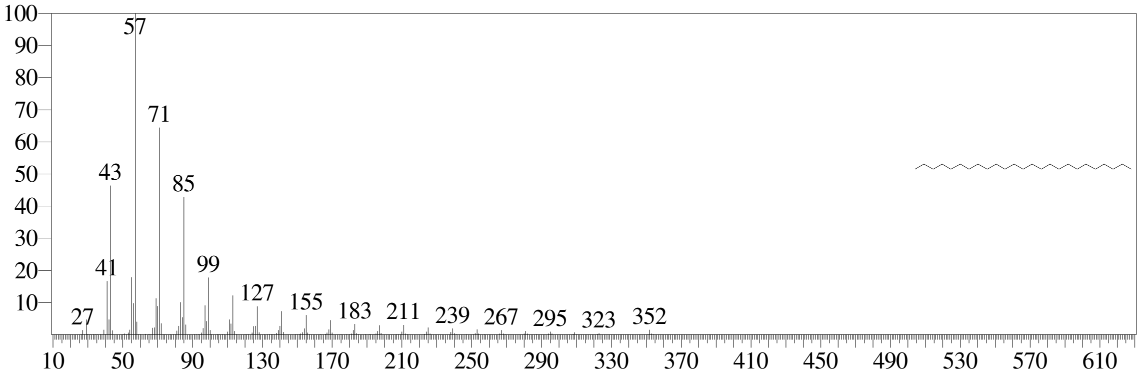

<< Target >>
Line#:21 R.Time:66.058(Scan#:7628) MassPeaks:88
RawMode:Averaged 66.050-66.067(7627-7629) BasePeak:57.05(666737)
BG Mode:Calc. from Peak Group 1 - Event 1 Scan

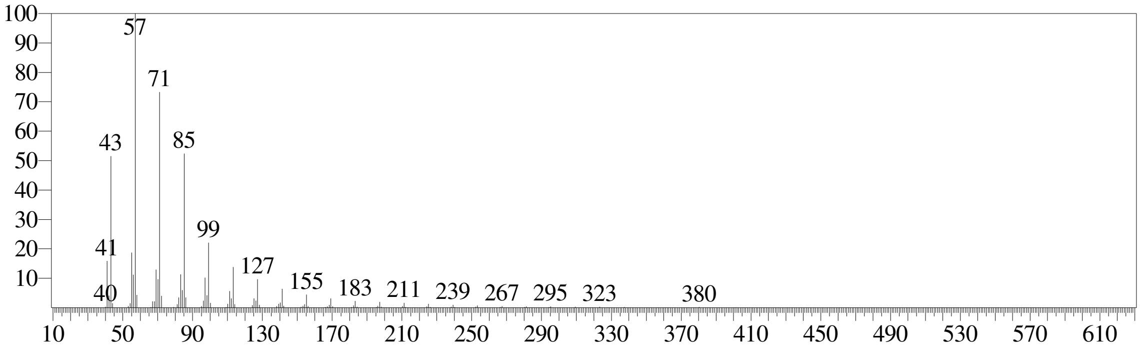

Hit#:3 Entry:39194 Library:NIST23s.lib
SI:96 Formula:C22H46 CAS:629-97-0 MolWeight:310 RetIndex:2203
CompName:Docosane $$ n-Docosane $$ Normal-docosane $$

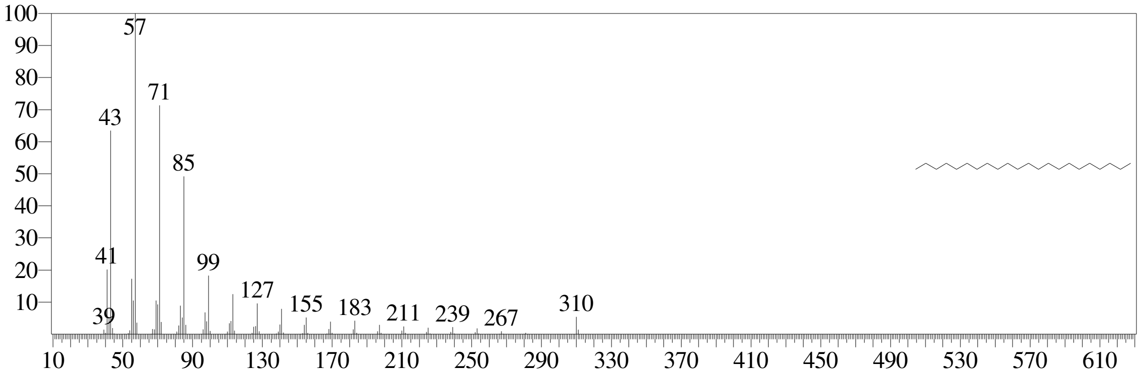

Hit#:4 Entry:95221 Library:NIST23-2.lib
SI:95 Formula:C44H90 CAS:7098-22-8 MolWeight:618 RetIndex:4390
CompName:Tetratetracontane $$ n-Tetratetracontane $$

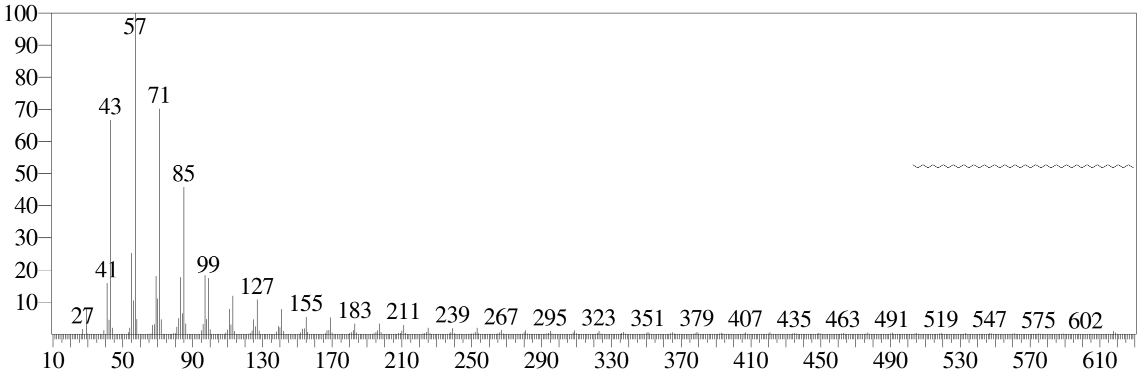

<< Target >>
Line#:21   R.Time:66.058(Scan#:7628) MassPeaks:88
RawMode:Averaged 66.050-66.067(7627-7629) BasePeak:57.05(666737)
BG Mode:Calc. from Peak Group 1 - Event 1 Scan

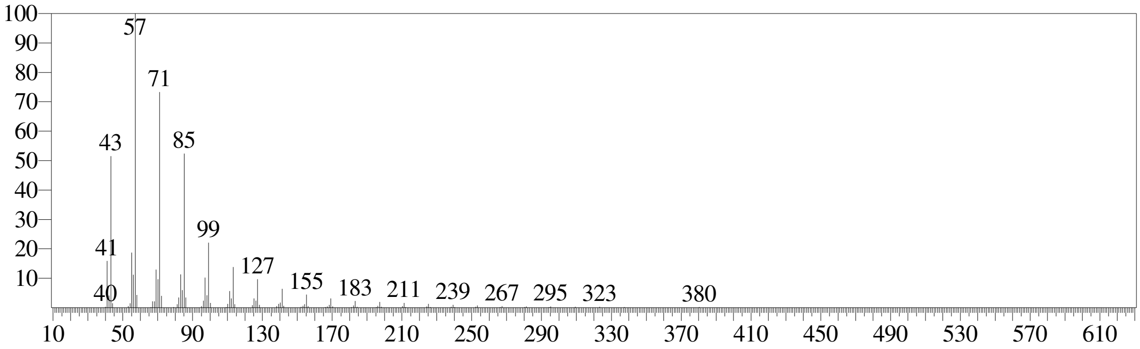

Hit#:5 Entry:65699 Library:NIST23-2.lib
SI:95 Formula:C31H64 CAS:630-04-6 MolWeight:436 RetIndex:3102
CompName:Hentriacontane $$ n-Hentriacontane $$ Untriacontane $$

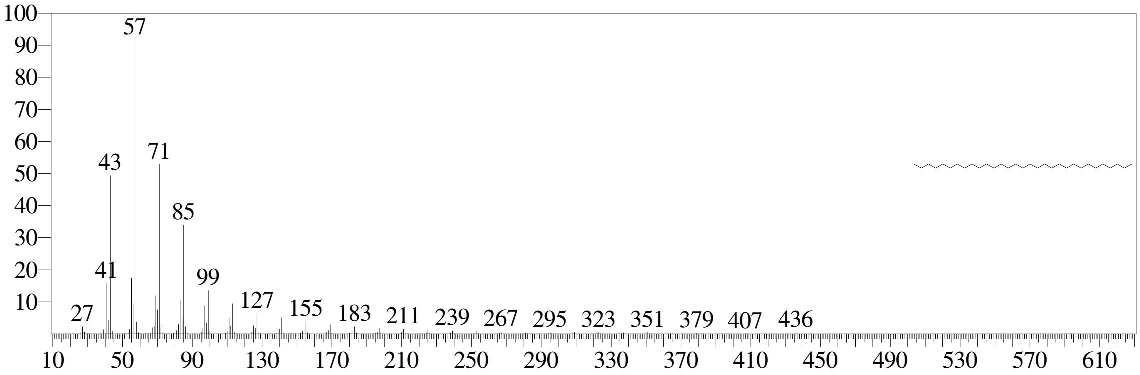

<< Target >>
Line#:22   R.Time:68.383(Scan#:7907) MassPeaks:91
RawMode:Averaged 68.375-68.392(7906-7908) BasePeak:57.05(657283)
BG Mode:Calc. from Peak Group 1 - Event 1 Scan

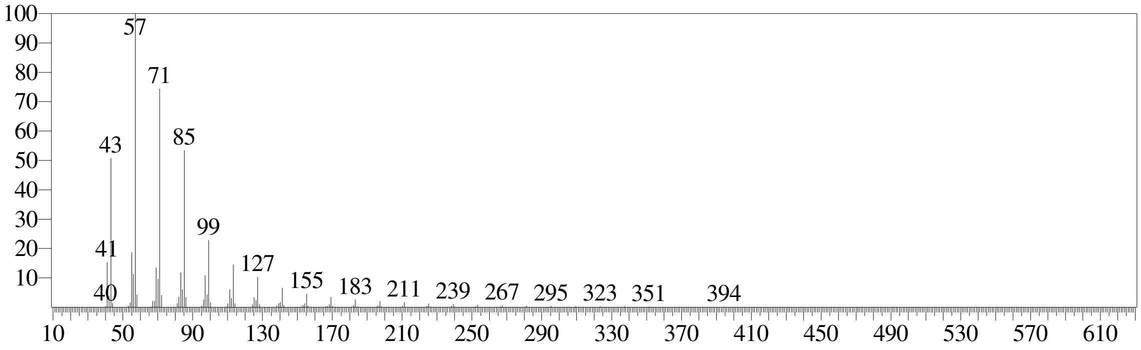

Hit#:1 Entry:2191 Library:NIST23-2.lib
SI:96 Formula:C25H52 CAS:629-99-2 MolWeight:352 RetIndex:2503
CompName:Pentacosane $$ n-Pentacosane $$

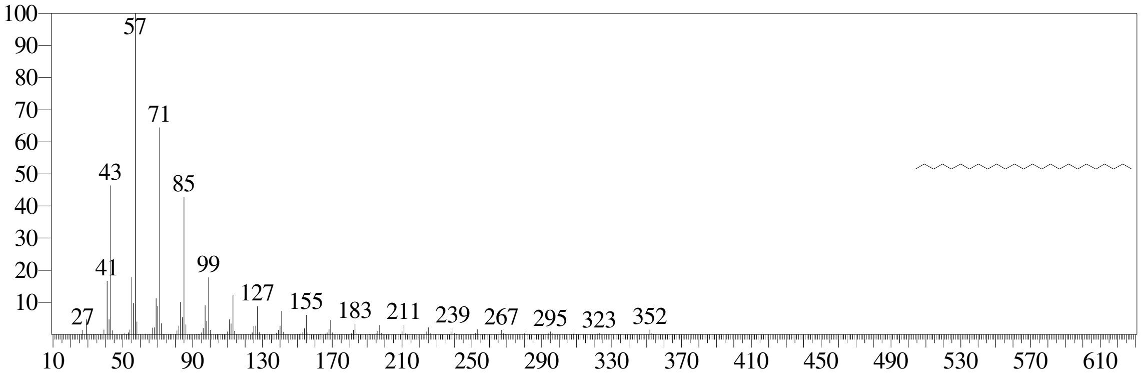

Hit#:2 Entry:37977 Library:NIST23s.lib
SI:96 Formula:C21H44 CAS:629-94-7 MolWeight:296 RetIndex:2103
CompName:Heneicosane $$ n-Heneicosane $$ Henicosane # $$

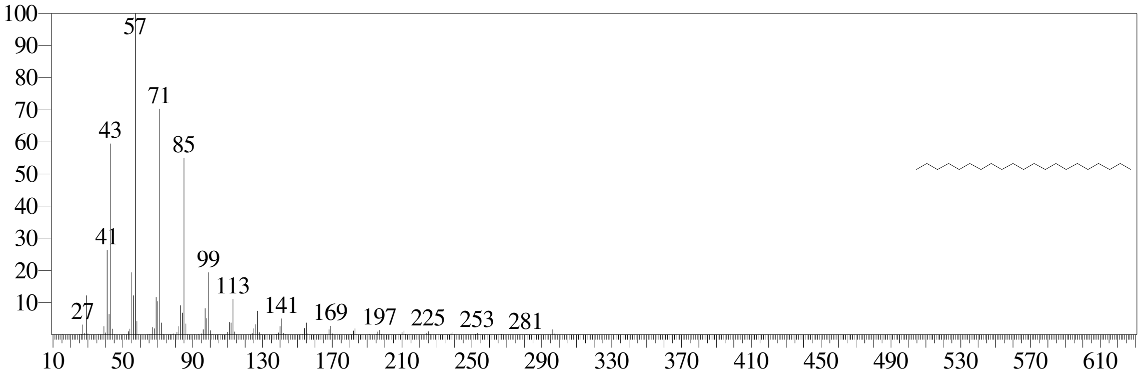

<< Target >>
Line#:22   R.Time:68.383(Scan#:7907) MassPeaks:91
RawMode:Averaged 68.375-68.392(7906-7908) BasePeak:57.05(657283)
BG Mode:Calc. from Peak Group 1 - Event 1 Scan

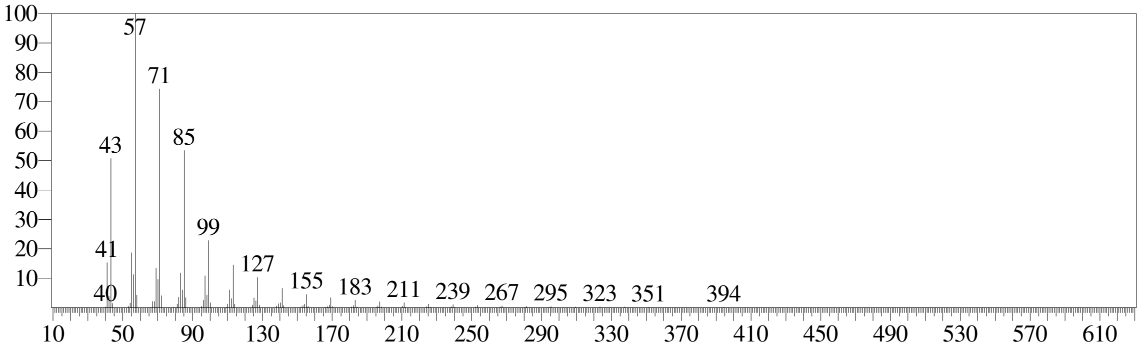

Hit#:3 Entry:46601 Library:NIST23s.lib
SI:96 Formula:C40H82 CAS:4181-95-7 MolWeight:562 RetIndex:3993
CompName:Tetracontane $$ n-Tetracontane $$

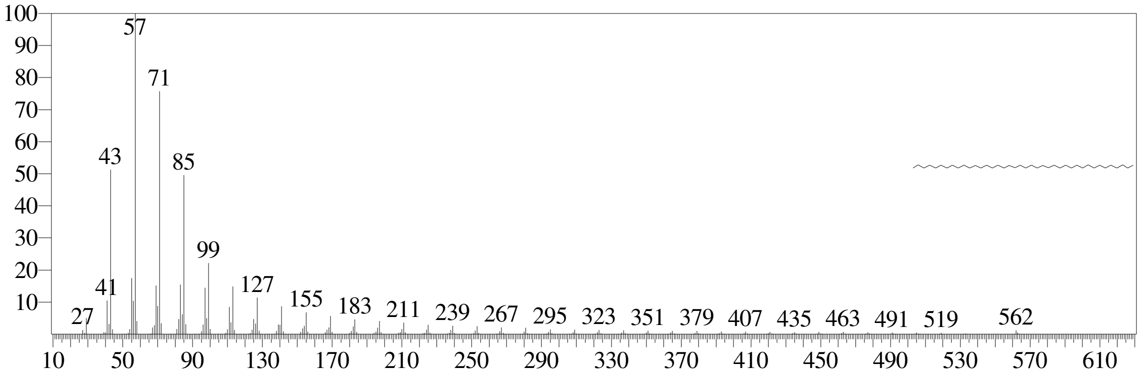

Hit#:4 Entry:95221 Library:NIST23-2.lib
SI:95 Formula:C44H90 CAS:7098-22-8 MolWeight:618 RetIndex:4390
CompName:Tetratetracontane $$ n-Tetratetracontane $$

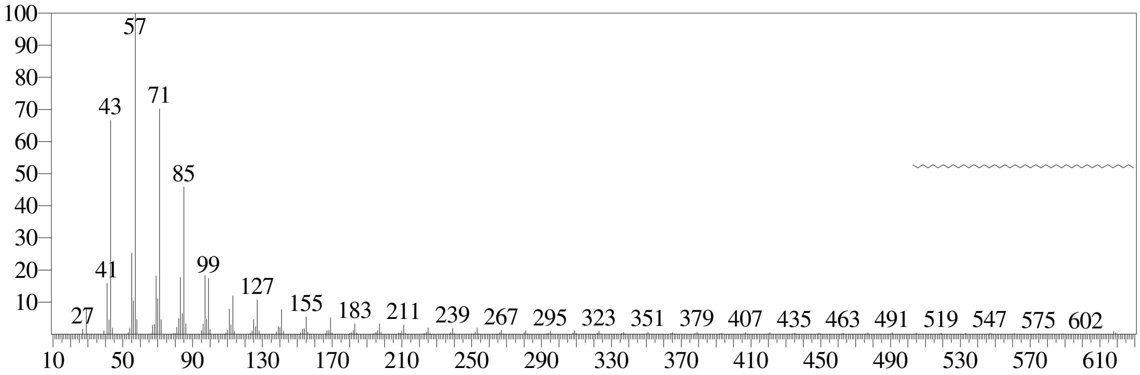

<< Target >>
Line#:22   R.Time:68.383(Scan#:7907) MassPeaks:91
RawMode:Averaged 68.375-68.392(7906-7908) BasePeak:57.05(657283)
BG Mode:Calc. from Peak Group 1 - Event 1 Scan

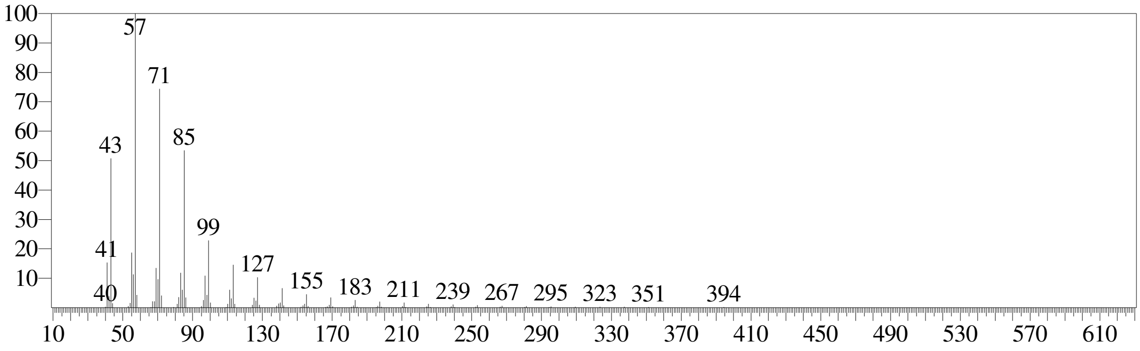

Hit#:5 Entry:93275 Library:NIST23-2.lib
SI:95 Formula:C32H65I CAS:0-00-0 MolWeight:576 RetIndex:3768
CompName:Dotriacontane, 1-iodo-

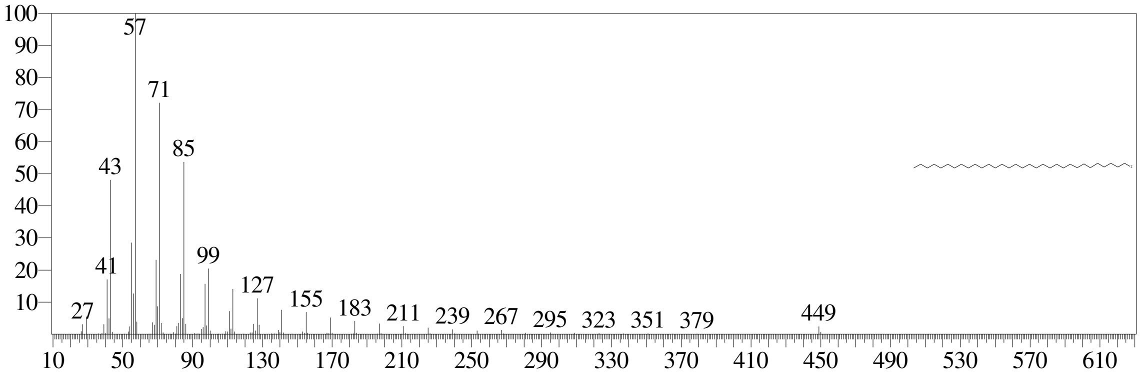

<< Target >>
Line#:23 R.Time:70.833(Scan#:8201) MassPeaks:92
RawMode:Averaged 70.825-70.842(8200-8202) BasePeak:57.05(592947)
BG Mode:Calc. from Peak Group 1 - Event 1 Scan

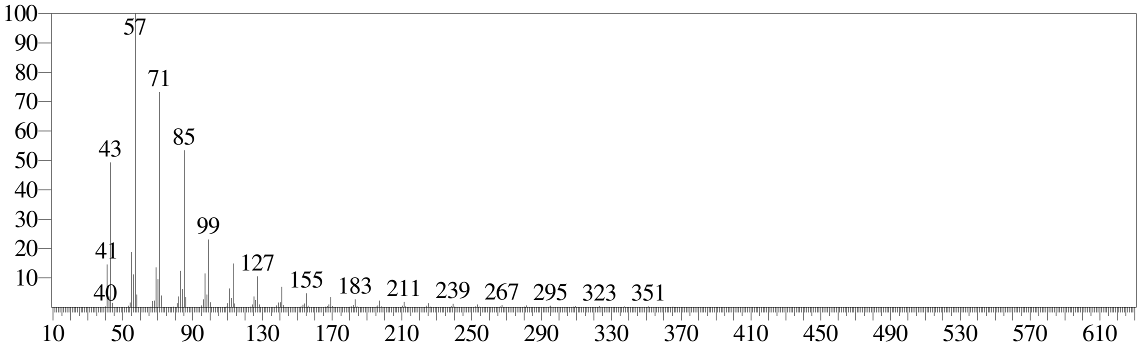

Hit#:1 Entry:2191 Library:NIST23-2.lib
SI:96 Formula:C25H52 CAS:629-99-2 MolWeight:352 RetIndex:2503
CompName:Pentacosane $$ n-Pentacosane $$

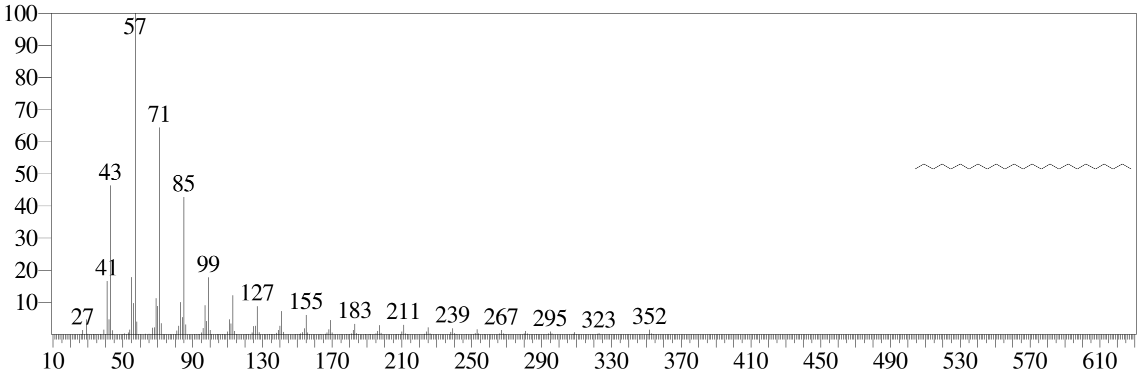

Hit#:2 Entry:46601 Library:NIST23s.lib
SI:96 Formula:C40H82 CAS:4181-95-7 MolWeight:562 RetIndex:3993
CompName:Tetracontane $$ n-Tetracontane $$

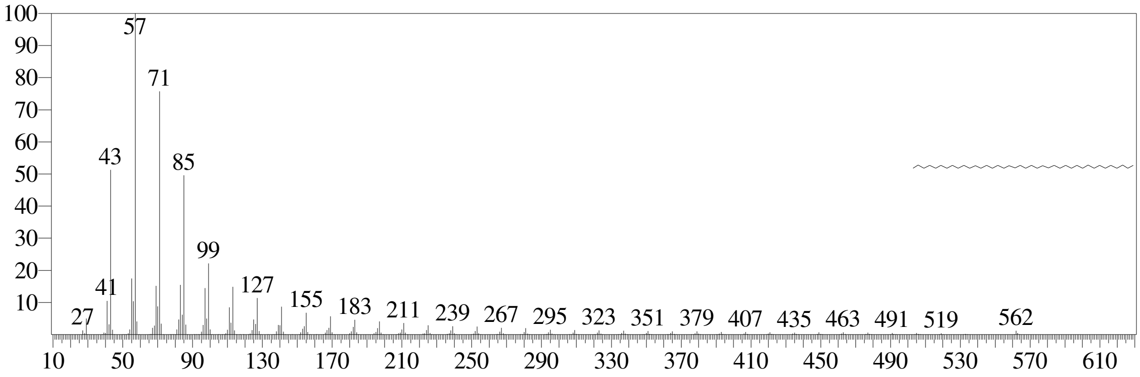

<< Target >>
Line#:23 R.Time:70.833(Scan#:8201) MassPeaks:92
RawMode:Averaged 70.825-70.842(8200-8202) BasePeak:57.05(592947)
BG Mode:Calc. from Peak Group 1 - Event 1 Scan

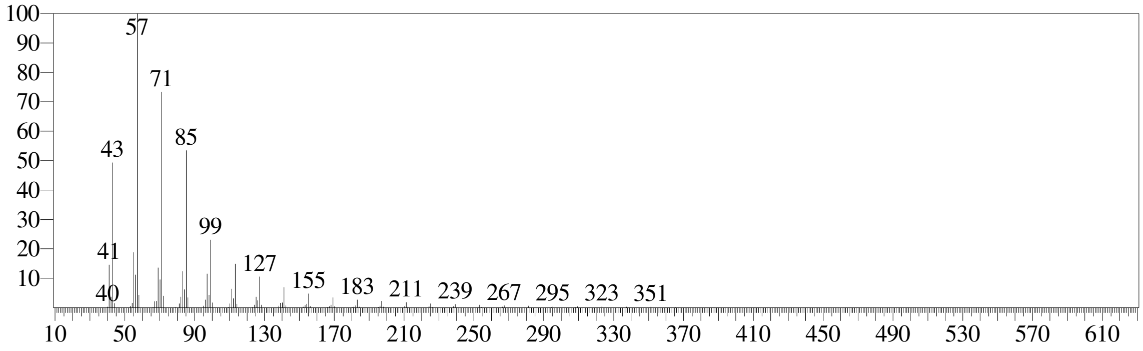

Hit#:3 Entry:95221 Library:NIST23-2.lib
SI:96 Formula:C44H90 CAS:7098-22-8 MolWeight:618 RetIndex:4390
CompName:Tetratetracontane $$ n-Tetratetracontane $$

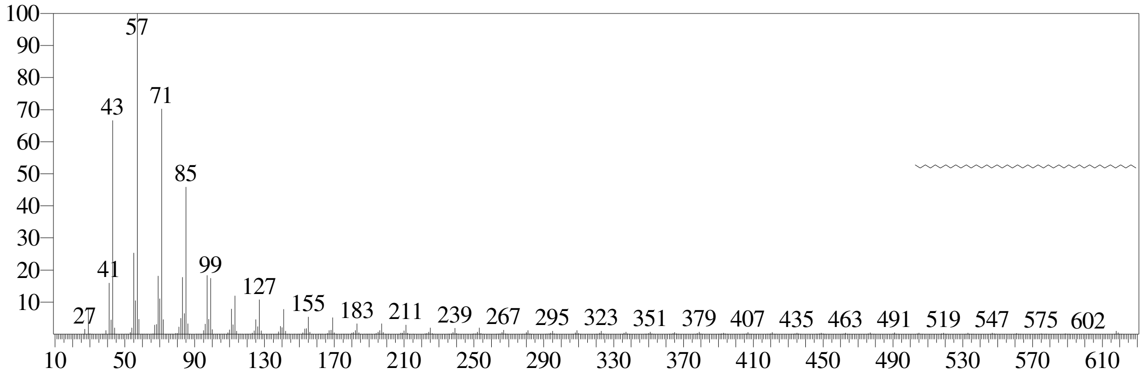

Hit#:4 Entry:93275 Library:NIST23-2.lib
SI:96 Formula:C32H65I CAS:0-00-0 MolWeight:576 RetIndex:3768
CompName:Dotriacontane, 1-iodo-

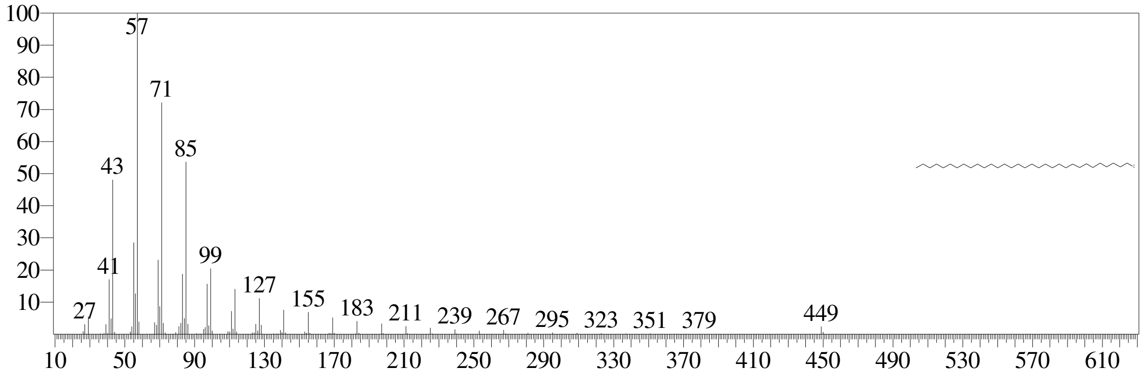

<< Target >>
Line#:23   R.Time:70.833(Scan#:8201) MassPeaks:92
RawMode:Averaged 70.825-70.842(8200-8202) BasePeak:57.05(592947)
BG Mode:Calc. from Peak Group 1 - Event 1 Scan

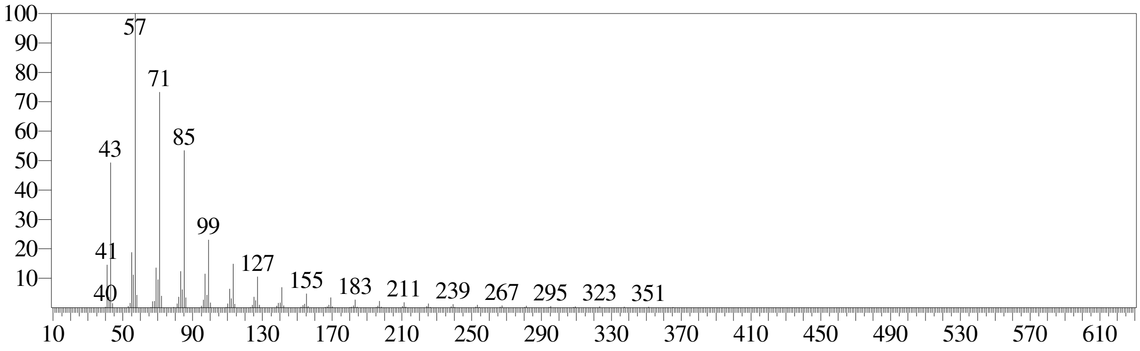

Hit#:5 Entry:37977 Library:NIST23s.lib
SI:95 Formula:C21H44 CAS:629-94-7 MolWeight:296 RetIndex:2103
CompName:Heneicosane $$ n-Heneicosane $$ Henicosane # $$

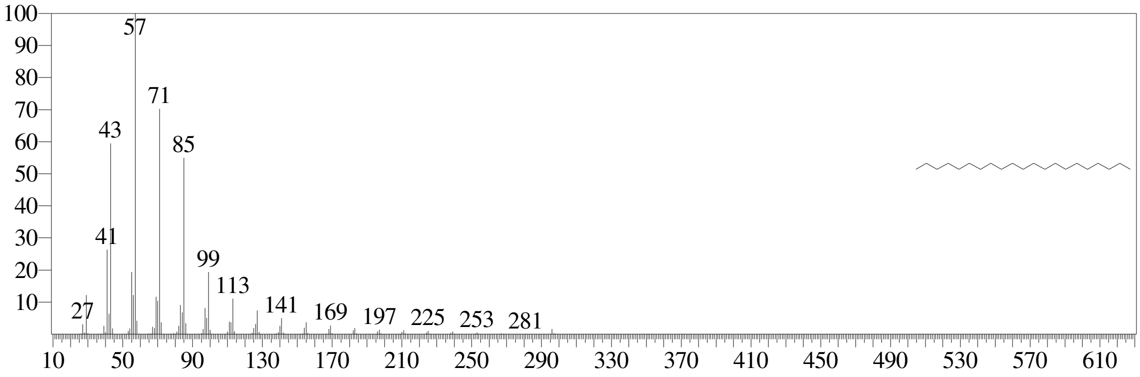

<< Target >>
Line#:24 R.Time:73.800(Scan#:8557) MassPeaks:91
RawMode:Averaged 73.792-73.808(8556-8558) BasePeak:57.05(516753)
BG Mode:Calc. from Peak Group 1 - Event 1 Scan

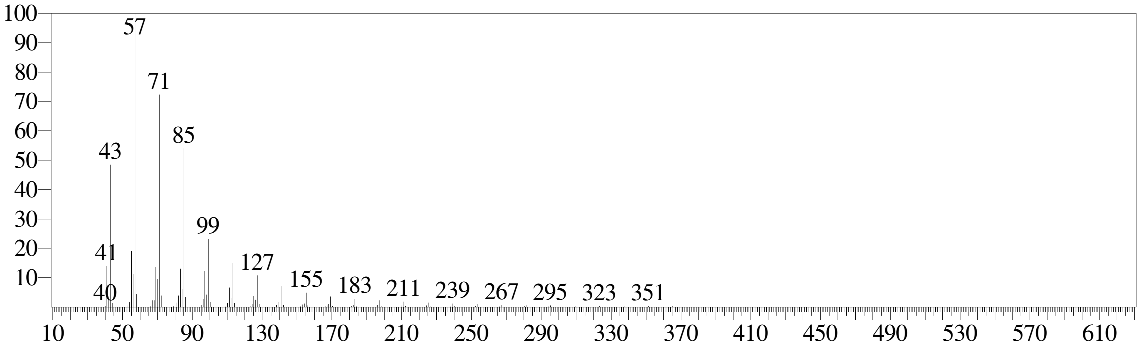

Hit#:1 Entry:46601 Library:NIST23s.lib
SI:96 Formula:C40H82 CAS:4181-95-7 MolWeight:562 RetIndex:3993
CompName:Tetracontane $$ n-Tetracontane $$

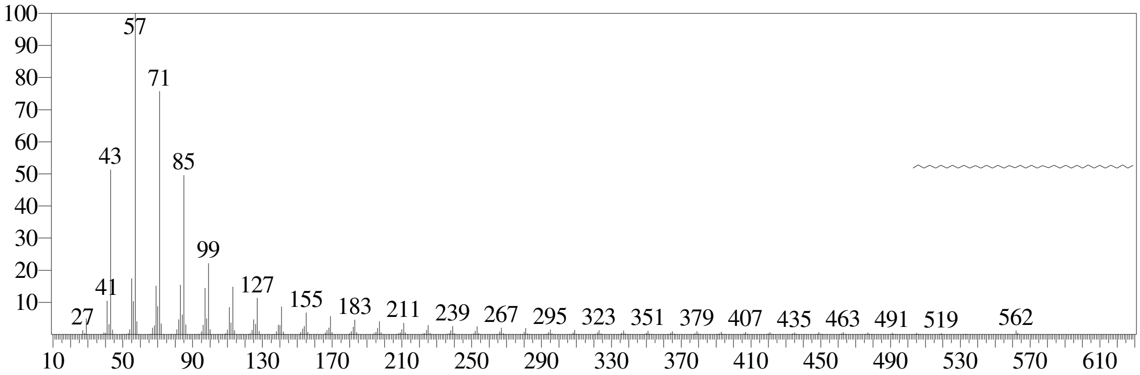

Hit#:2 Entry:95221 Library:NIST23-2.lib
SI:96 Formula:C44H90 CAS:7098-22-8 MolWeight:618 RetIndex:4390
CompName:Tetratetracontane $$ n-Tetratetracontane $$

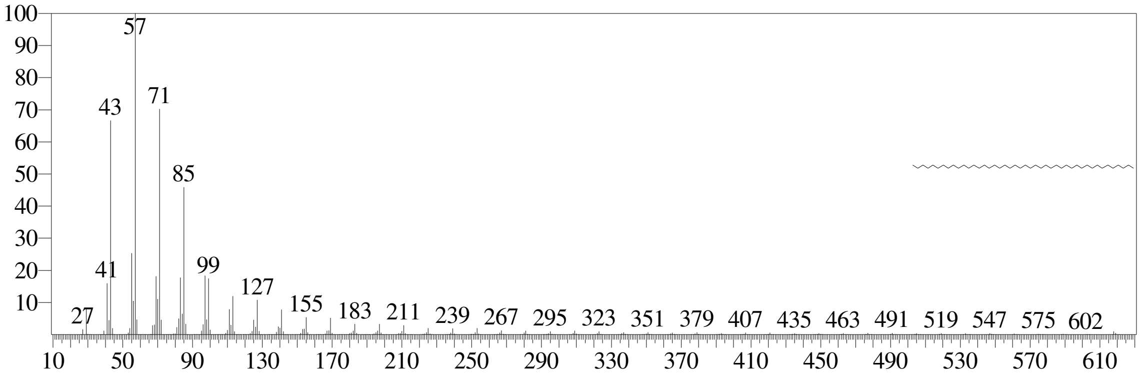

<< Target >>
Line#:24 R.Time:73.800(Scan#:8557) MassPeaks:91
RawMode:Averaged 73.792-73.808(8556-8558) BasePeak:57.05(516753)
BG Mode:Calc. from Peak Group 1 - Event 1 Scan

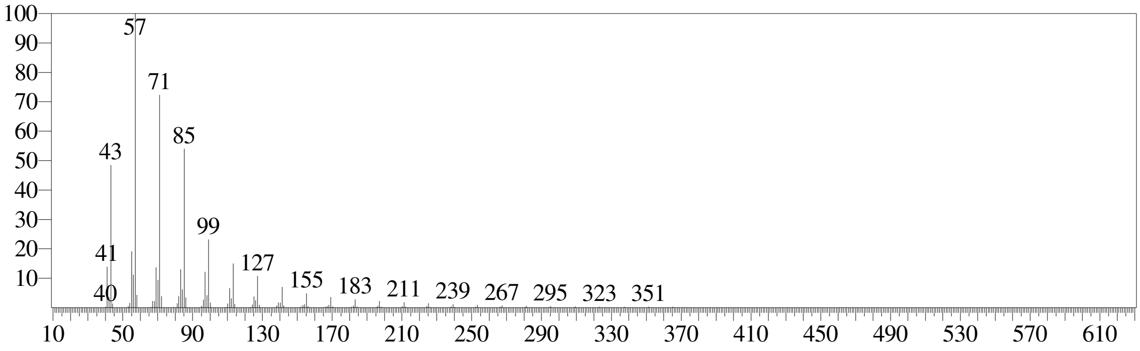

Hit#:3 Entry:2191 Library:NIST23-2.lib
SI:96 Formula:C25H52 CAS:629-99-2 MolWeight:352 RetIndex:2503
CompName:Pentacosane $$ n-Pentacosane $$

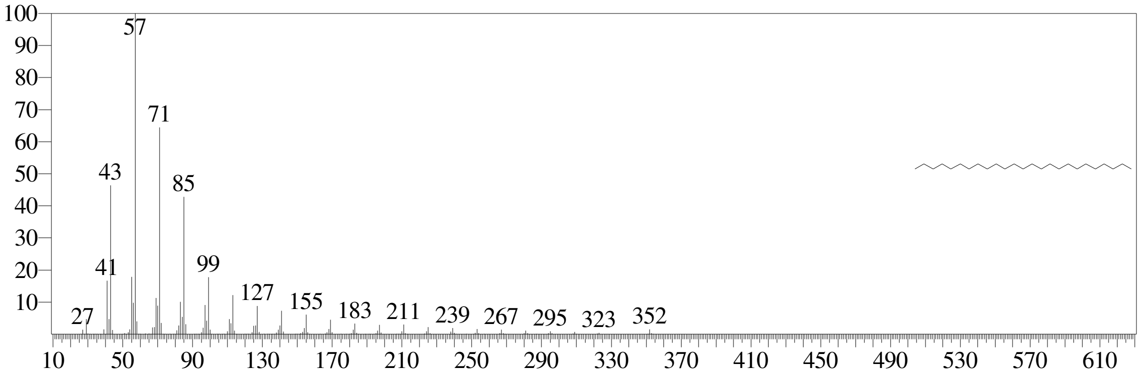

Hit#:4 Entry:93275 Library:NIST23-2.lib
SI:96 Formula:C32H65I CAS:0-00-0 MolWeight:576 RetIndex:3768
CompName:Dotriacontane, 1-iodo-

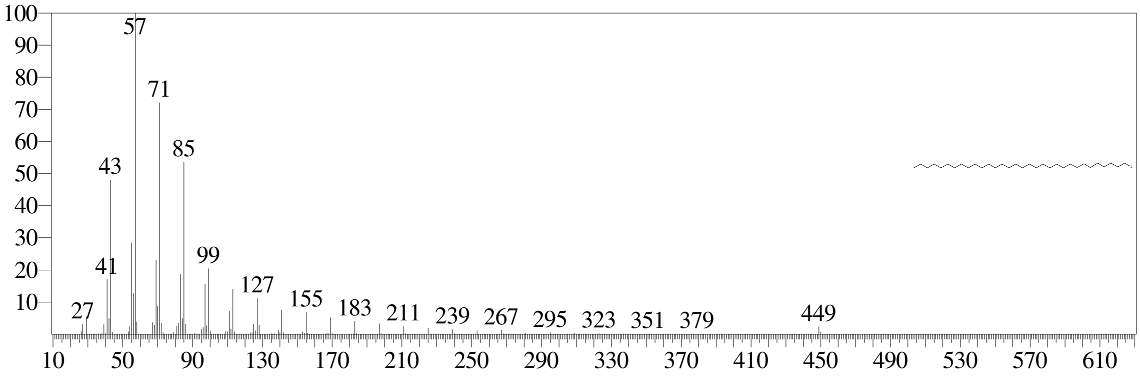

<< Target >>
Line#:24   R.Time:73.800(Scan#:8557) MassPeaks:91
RawMode:Averaged 73.792-73.808(8556-8558) BasePeak:57.05(516753)
BG Mode:Calc. from Peak Group 1 - Event 1 Scan

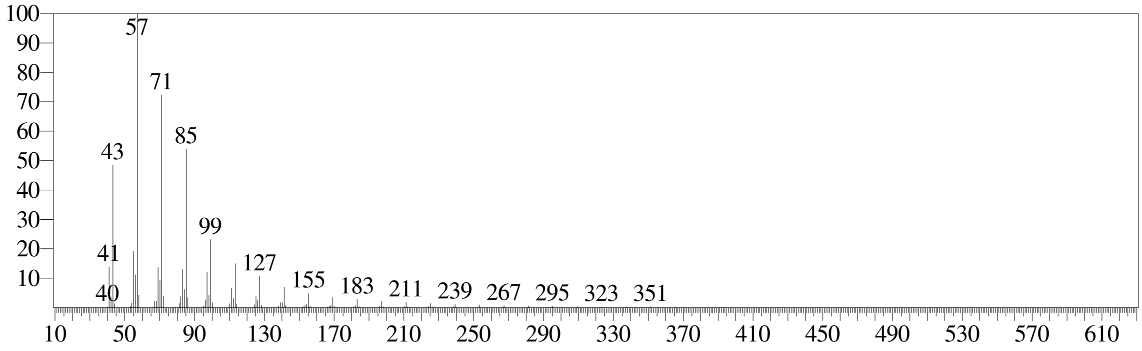

Hit#:5 Entry:37977 Library:NIST23s.lib
SI:95 Formula:C21H44 CAS:629-94-7 MolWeight:296 RetIndex:2103
CompName:Heneicosane $$ n-Heneicosane $$ Henicosane # $$

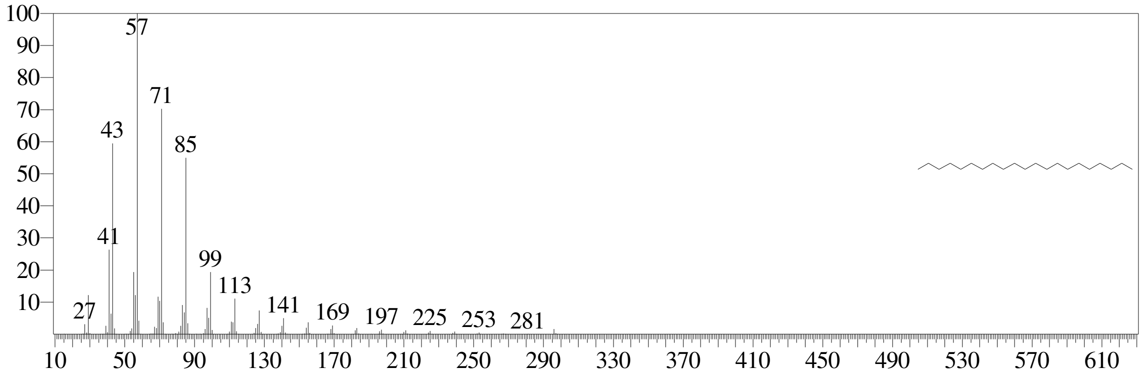

Supplement: Supplementary file 1 [file plants-15-01406-s001.zip › C7_C30 170625bb.pdf]
