# Supplementary material for: Unveiling Chemical Profile and Insecticidal Potential of Essential Oils from Leaves of Seven Eugenia L. Species (Myrtaceae)
Source: Plants (Basel). 2026 May 5;15(9):1406. doi: 10.3390/plants15091406 (PMC13165059; doi:10.3390/plants15091406)

UNIVERSIDADE FERAL RURAL DO RIO DE JANEIRO
INSTITUTO DE QUÍMICA

LabCrom — Laboratório de Cromatografia (CAM · UFRRJ)

CENTRAL ANALÍTICA MULTIUSUÁRIO
INSTITUTO DE QUÍMICA · UFRRJ

CGMS

Analyzed by: Cristiane Cardoso
Analyzed: 17/6/2025
Solicitante:Douglas
Sample Name: EB
Injection Volume: 1,0 uL   Solvente: Diclorometano
Data File: C:\GCMSsolution\Data\Project1\Douglas\2025\MLLENA\170625\EB.qgd
Method File: C:\GCMSsolution\Data\Project1\Douglas\Essencial Adams-Inj.qgm
EQUIPAMENTO: Modelo:GCMS-QP2010 Plus (Shimadzu)
Coluna: VF-5m (30X0.25X0.25)

Chromatogram EB C:\GCMSsolution\Data\Project1\Douglas\2025\MLLENA\170625\EB.qgd

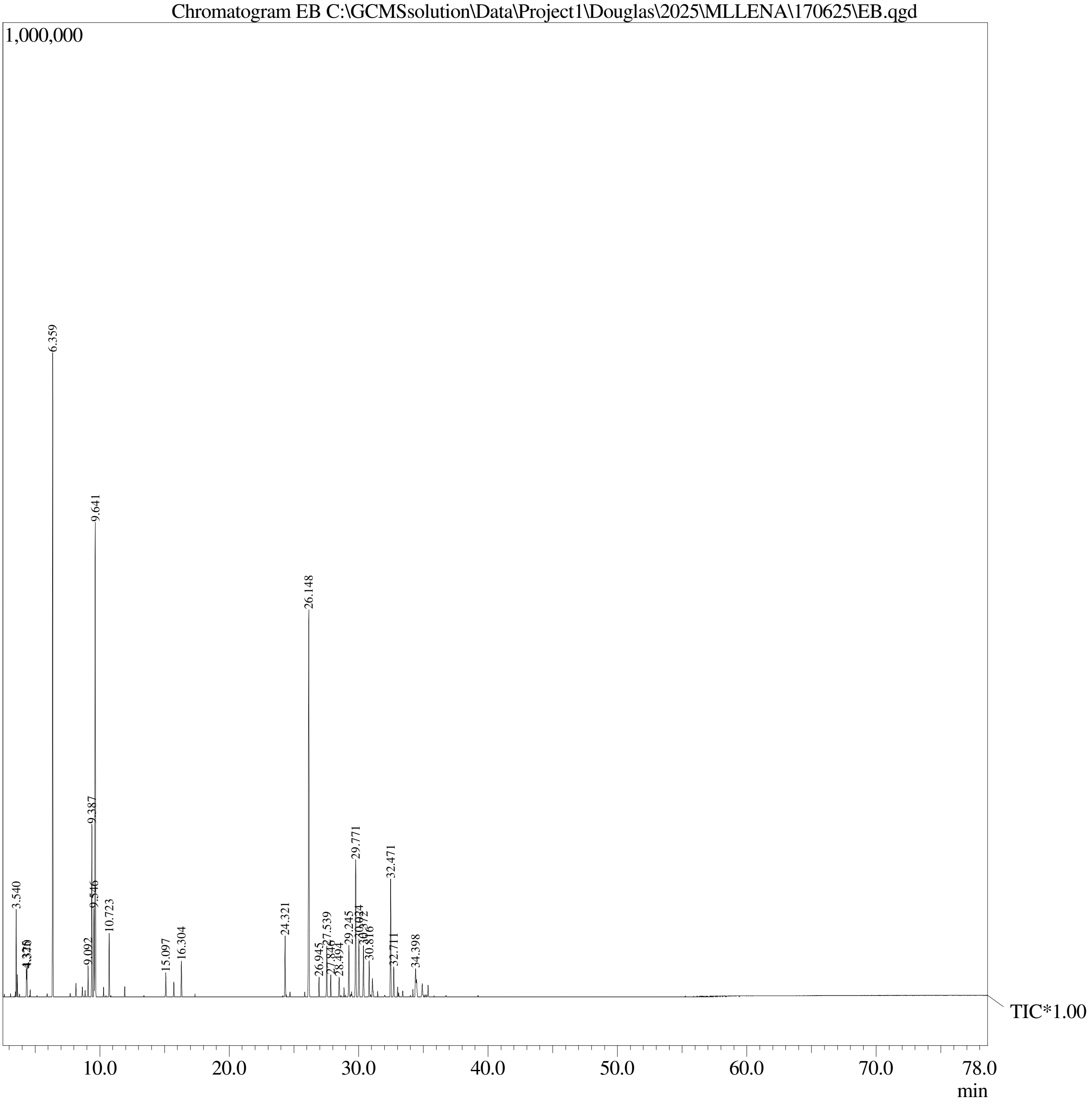

Library

<< Target >>
Line#:1 R.Time:3.542(Scan#:126) MassPeaks:11
RawMode:Averaged 3.533-3.550(125-127) BasePeak:45.00(47519)
BG Mode:None Group 1 - Event 1 Scan

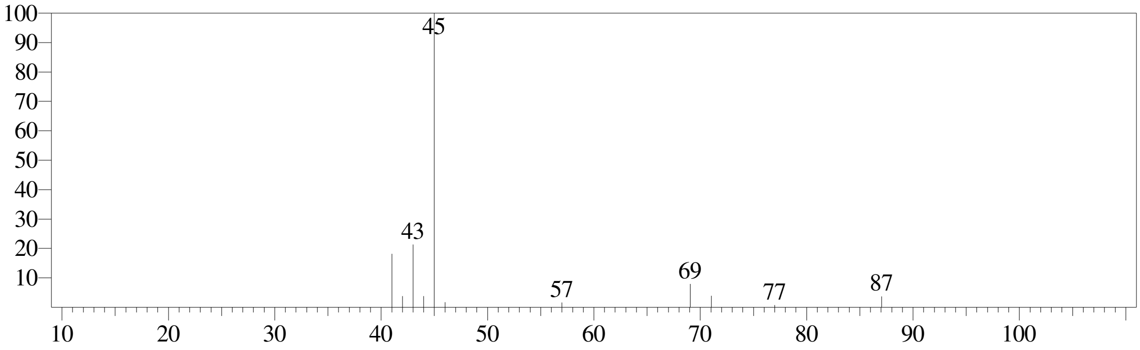

Hit#:1 Entry:2534 Library:NIST23-1.lib
SI:93 Formula:C6H14O CAS:26549-24-6 MolWeight:102 RetIndex:791
CompName:2-Hexanol, (R)- $$ (R)-(-)-2-Hexanol $$ 2-Hexanol # $$

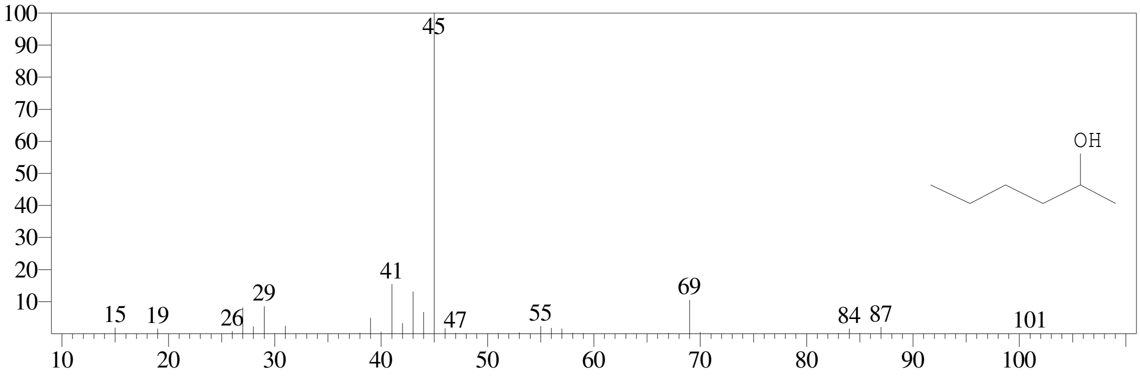

Hit#:2 Entry:2533 Library:NIST23-1.lib
SI:93 Formula:C6H14O CAS:52019-78-0 MolWeight:102 RetIndex:791
CompName:2-Hexanol, (S)- $$ (S)-(+)-2-Hexanol $$ 2-Hexanol # $$

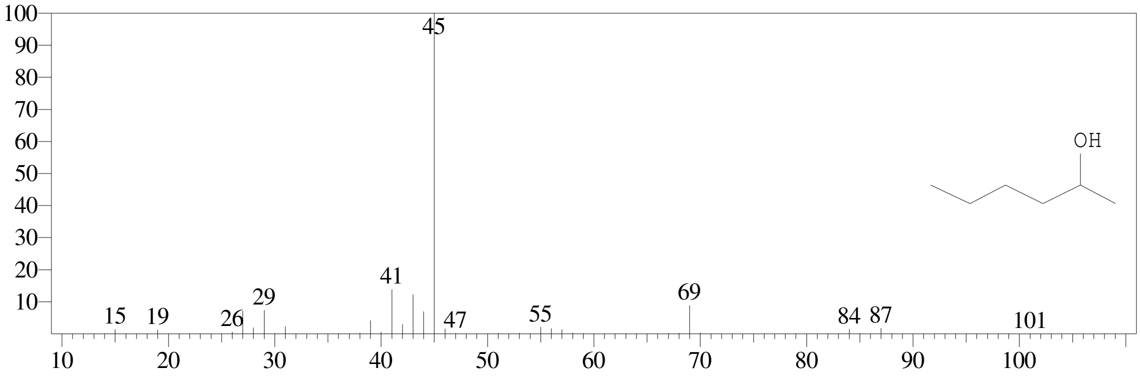

<< Target >>
Line#:1   R.Time:3.542(Scan#:126) MassPeaks:11
RawMode:Averaged 3.533-3.550(125-127) BasePeak:45.00(47519)
BG Mode:None Group 1 - Event 1 Scan

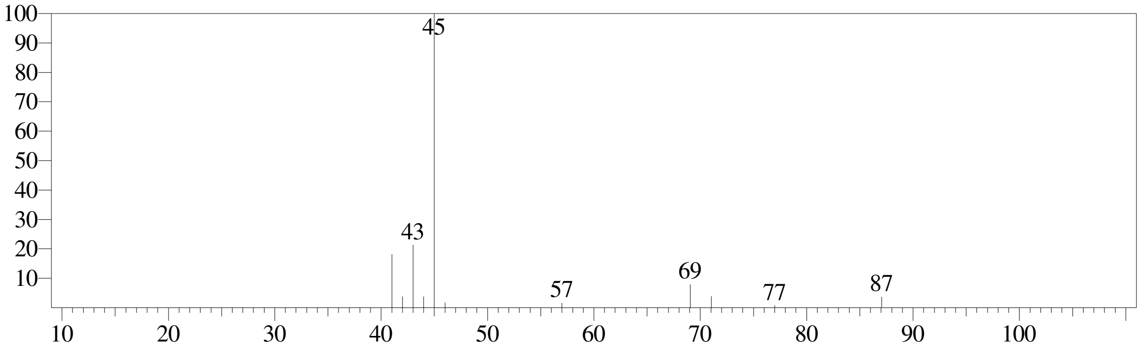

Hit#:3 Entry:2597 Library:NIST23s.lib
SI:93 Formula:C6H14O CAS:108-11-2 MolWeight:102 RetIndex:752
CompName:2-Pentanol, 4-methyl- $$ Isobutylmethylcarbinol $$ Isobutylmethylmethanol $$ Methylisobutylcarbinol $$ M

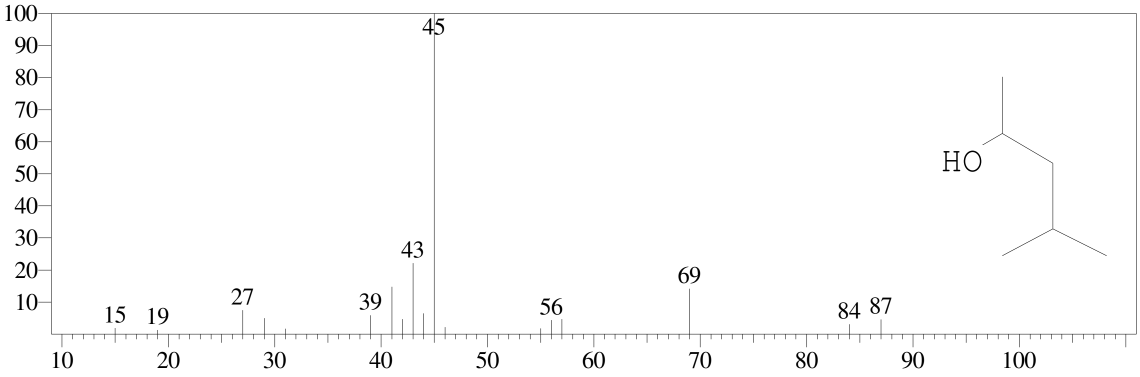

Hit#:4 Entry:1082 Library:NIST23s.lib
SI:92 Formula:C5H10O CAS:625-31-0 MolWeight:86 RetIndex:658
CompName:4-Penten-2-ol $$ 1-Penten-4-ol $$ 4-Hydroxypent-1-ene $$ CH2=CHCH2CH(OH)CH3 $$

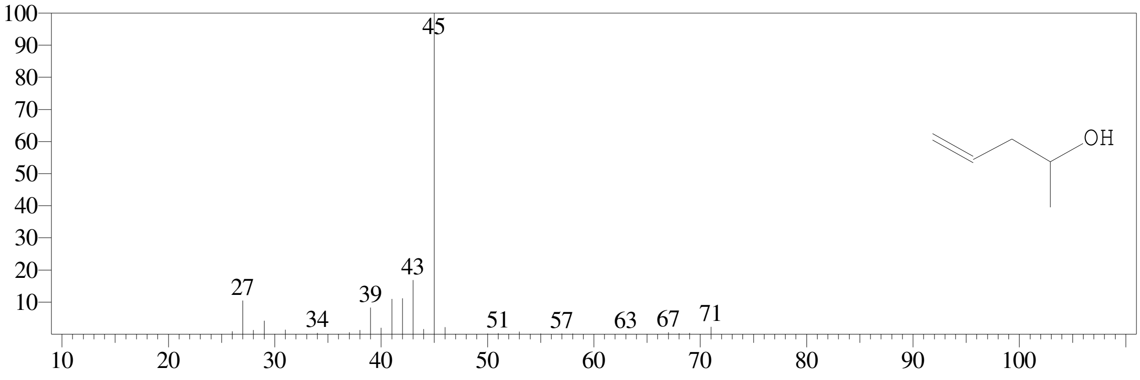

<< Target >>
Line#:1   R.Time:3.542(Scan#:126) MassPeaks:11
RawMode:Averaged 3.533-3.550(125-127) BasePeak:45.00(47519)
BG Mode:None Group 1 - Event 1 Scan

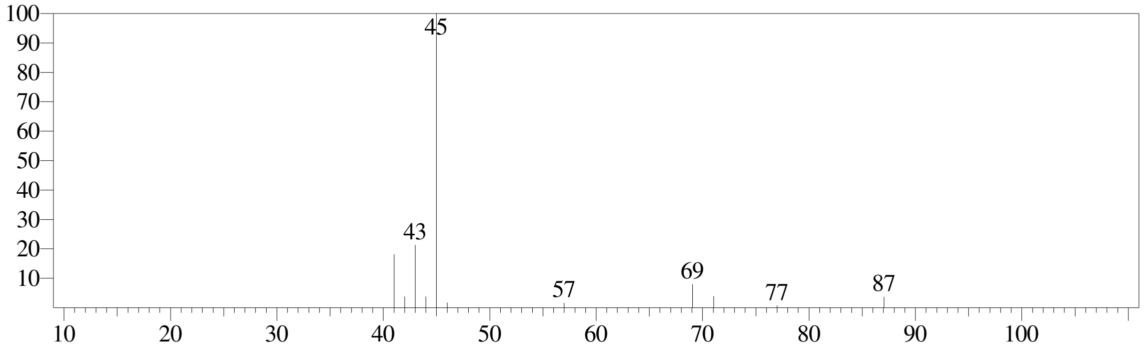

Hit#:5 Entry:897 Library:NIST23-1.lib
SI:92 Formula:C5H10O CAS:625-31-0 MolWeight:86 RetIndex:658
CompName:4-Penten-2-ol $$ 1-Penten-4-ol $$ 4-Hydroxypent-1-ene $$ CH2=CHCH2CH(OH)CH3 $$

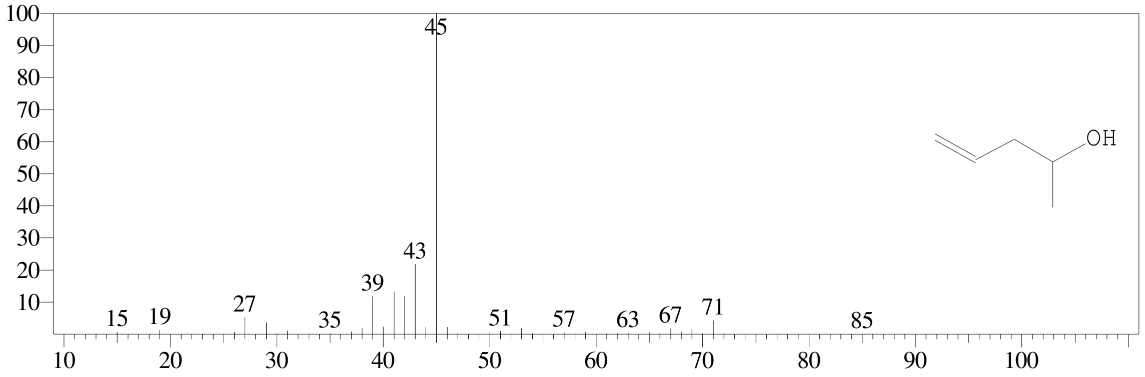

<< Target >>
Line#:2 R.Time:4.325(Scan#:220) MassPeaks:9
RawMode:Averaged 4.317-4.333(219-221) BasePeak:55.00(3189)
BG Mode:Calc. from Peak Group 1 - Event 1 Scan

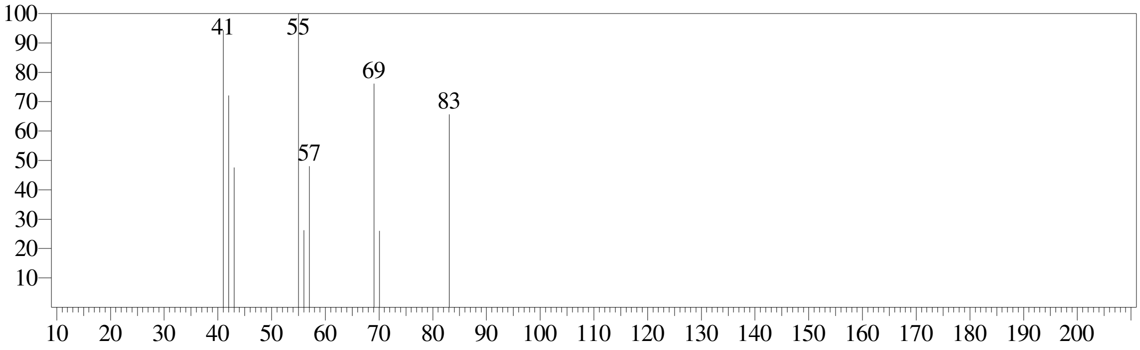

Hit#:1 Entry:1298 Library:NIST23s.lib
SI:85 Formula:C5H12O CAS:123-51-3 MolWeight:88 RetIndex:730
CompName:1-Butanol, 3-methyl- $$ Isopentyl alcohol $$ Fermentation amyl alcohol $$ Fusel Oil $$ Isoamyl alcohol $$ I

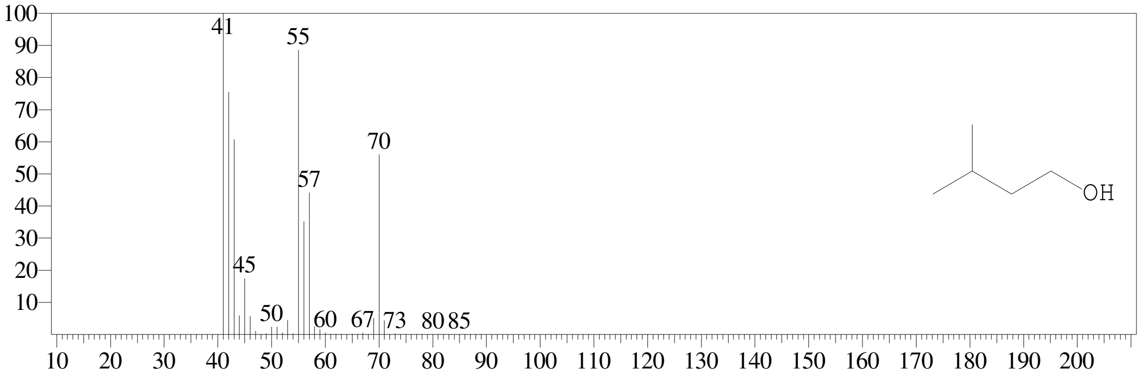

Hit#:2 Entry:1896 Library:NIST23s.lib
SI:85 Formula:C6H10O CAS:505-57-7 MolWeight:98 RetIndex:849
CompName:2-Hexenal $$ 2-Hexen-1-al $$ n-C3H7CH=CHCHO $$ Hex-2-enal $$ Hex-2-en-1-al $$ Hexylenic aldehyde

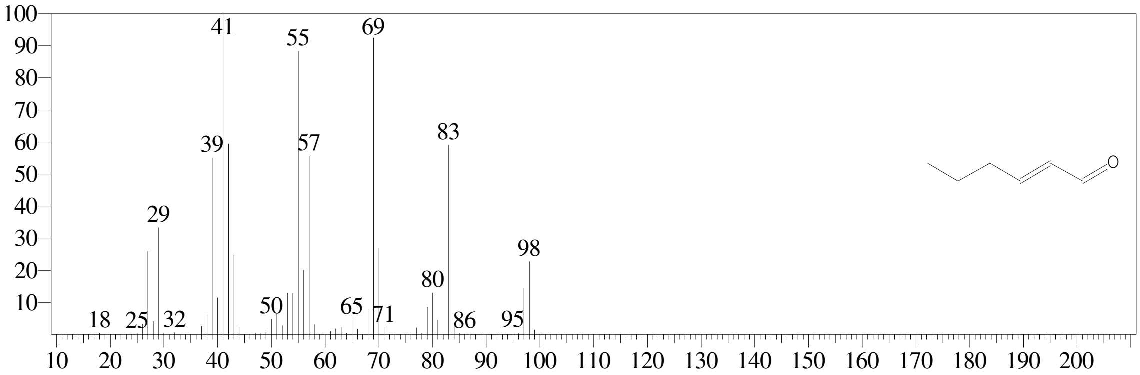

<< Target >>
Line#:2   R.Time:4.325(Scan#:220) MassPeaks:9
RawMode:Averaged 4.317-4.333(219-221) BasePeak:55.00(3189)
BG Mode:Calc. from Peak Group 1 - Event 1 Scan

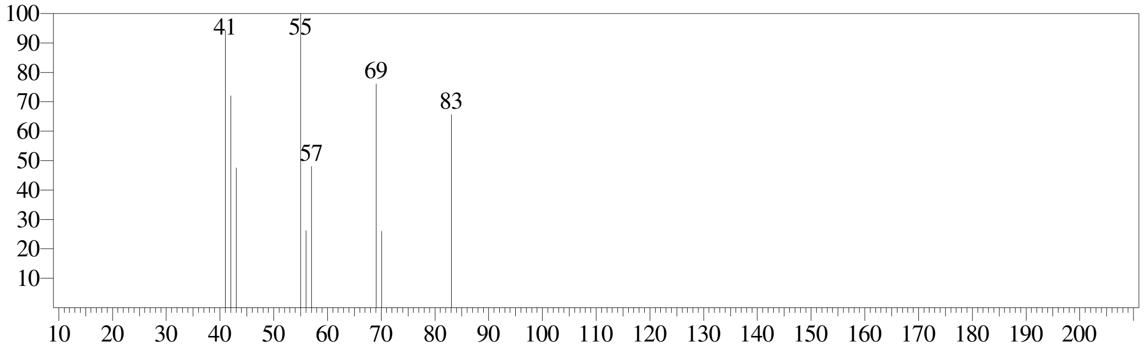

Hit#:3 Entry:1892 Library:NIST23s.lib
SI:84 Formula:C6H10O CAS:6728-26-3 MolWeight:98 RetIndex:849
CompName:2-Hexenal, (E)- $$ (E)-2-Hexenal $$ n-Hex-trans-2-enal $$ trans-Hex-2-enal $$ trans-2-Hexen-1-al $$ trans-

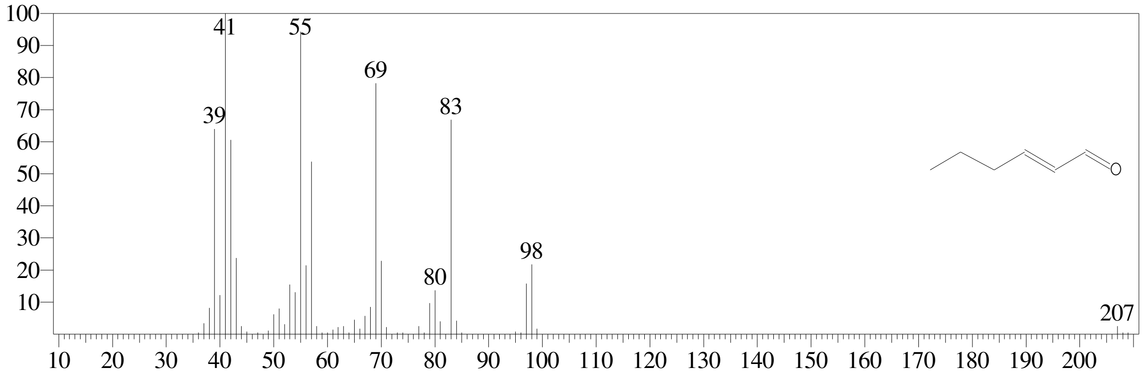

Hit#:4 Entry:1888 Library:NIST23s.lib
SI:84 Formula:C6H10O CAS:6728-26-3 MolWeight:98 RetIndex:849
CompName:2-Hexenal, (E)- $$ (E)-2-Hexenal $$ n-Hex-trans-2-enal $$ trans-Hex-2-enal $$ trans-2-Hexen-1-al $$ trans-

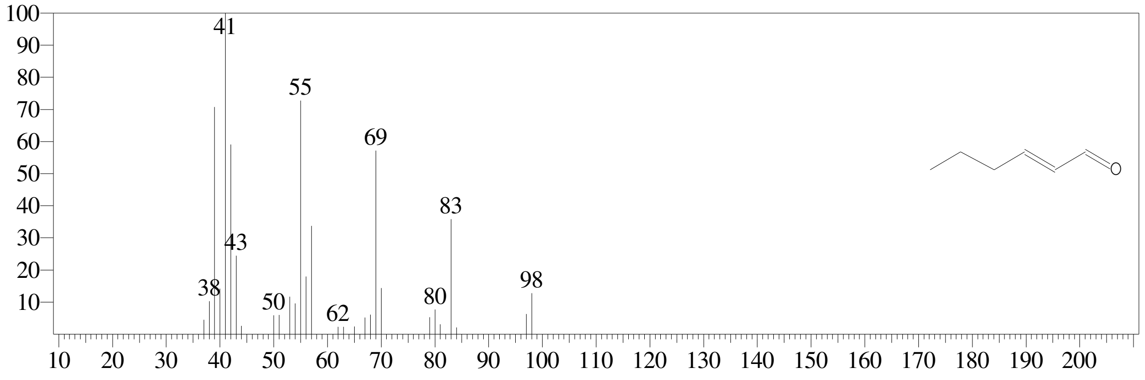

<< Target >>
Line#:2   R.Time:4.325(Scan#:220) MassPeaks:9
RawMode:Averaged 4.317-4.333(219-221) BasePeak:55.00(3189)
BG Mode:Calc. from Peak Group 1 - Event 1 Scan

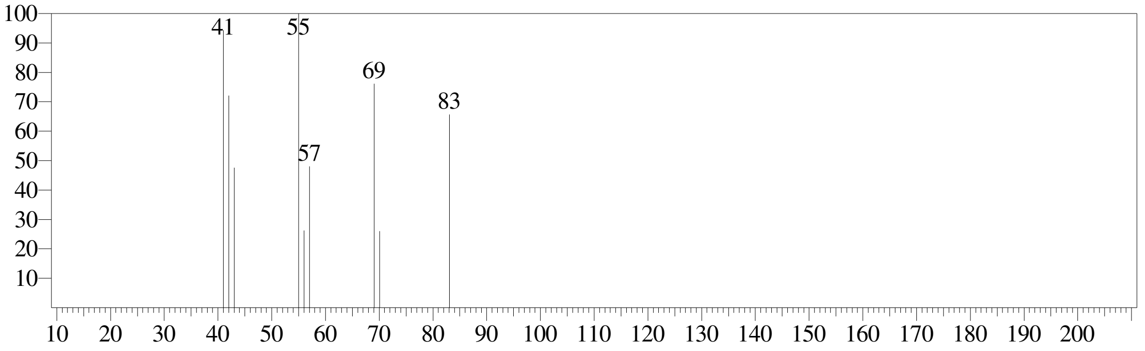

Hit#:5 Entry:1891 Library:NIST23s.lib
SI:84 Formula:C6H10O CAS:6728-26-3 MolWeight:98 RetIndex:849
CompName:2-Hexenal, (E)- $$ (E)-2-Hexenal $$ n-Hex-trans-2-enal $$ trans-Hex-2-enal $$ trans-2-Hexen-1-al $$ trans-

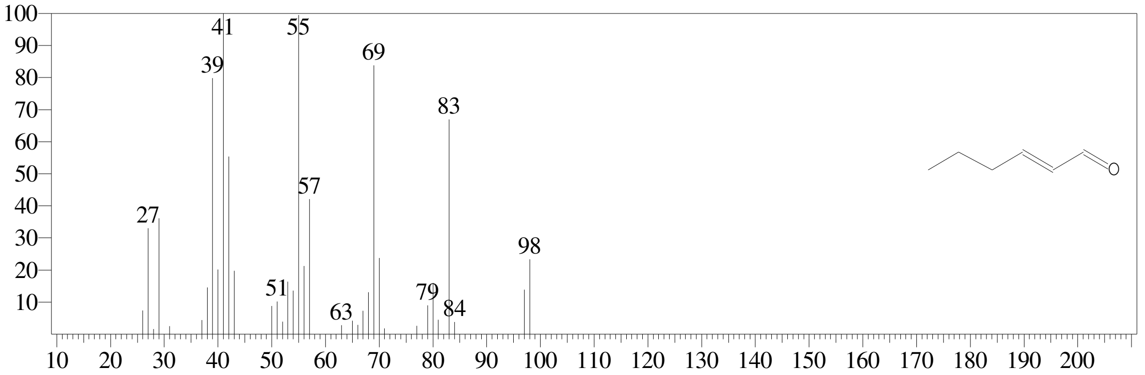

<< Target >>
Line#:3 R.Time:4.367(Scan#:225) MassPeaks:9
RawMode:Averaged 4.358-4.375(224-226) BasePeak:41.00(4269)
BG Mode:Calc. from Peak Group 1 - Event 1 Scan

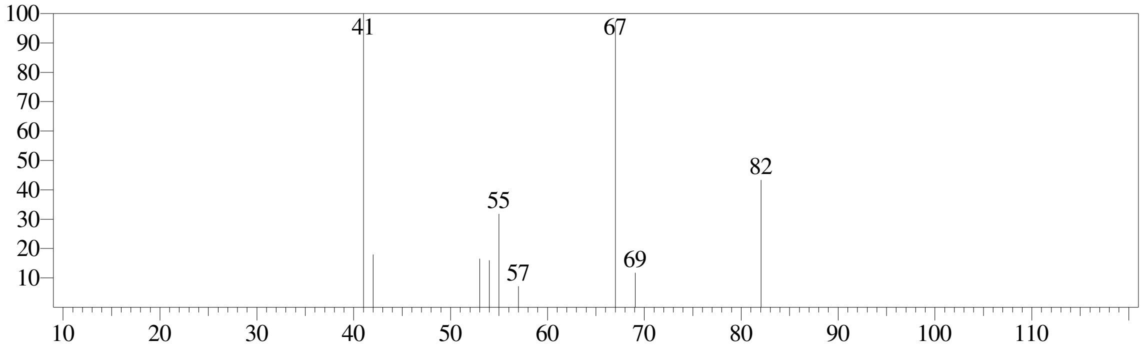

Hit#:1 Entry:6482 Library:NIST23s.lib
SI:89 Formula:C7H12O2 CAS:33467-73-1 MolWeight:128 RetIndex:931
CompName:3-Hexen-1-ol, formate, (Z)- $$ cis-.beta.-Hexenyl formate $$ cis-3-Hexenyl formate $$ cis-3-Hexen-1-ol forr

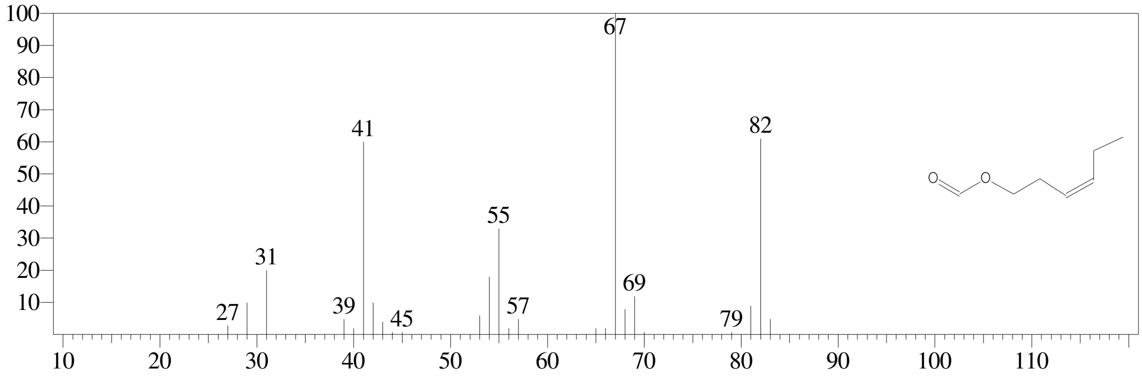

Hit#:2 Entry:2217 Library:NIST23s.lib
SI:87 Formula:C6H12O CAS:544-12-7 MolWeight:100 RetIndex:857
CompName:3-Hexen-1-ol $$ 3-Hexen-1-ol (c,t) $$ 3-Hexenol $$ Hex-3-en-1-ol $$

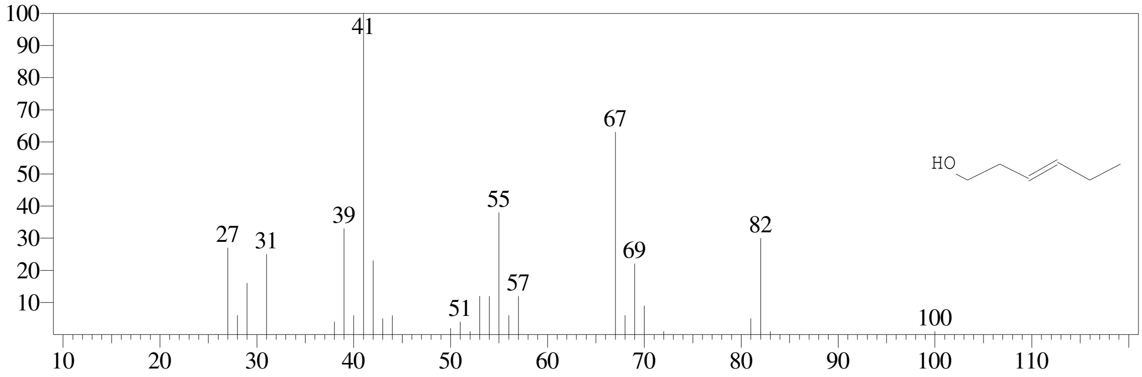

<< Target >>
Line#:3 R.Time:4.367(Scan#:225) MassPeaks:9
RawMode:Averaged 4.358-4.375(224-226) BasePeak:41.00(4269)
BG Mode:Calc. from Peak Group 1 - Event 1 Scan

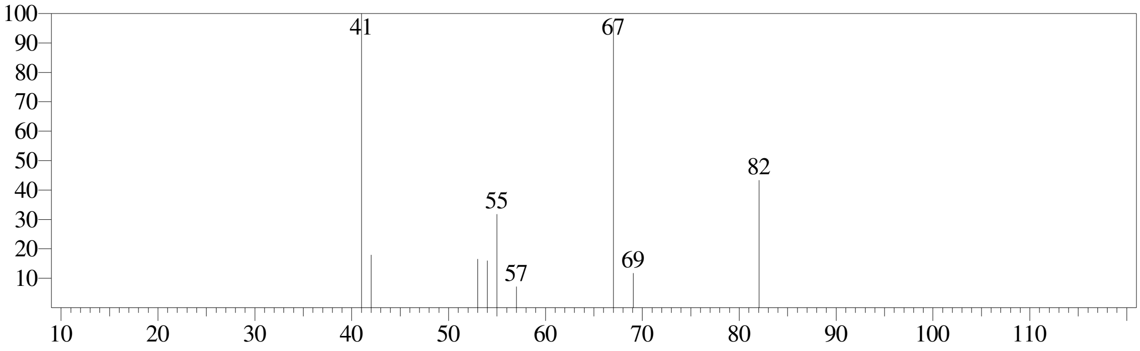

Hit#:3 Entry:2219 Library:NIST23s.lib
SI:87 Formula:C6H12O CAS:544-12-7 MolWeight:100 RetIndex:857
CompName:3-Hexen-1-ol $$ 3-Hexen-1-ol (c,t) $$ 3-Hexenol $$ Hex-3-en-1-ol $$

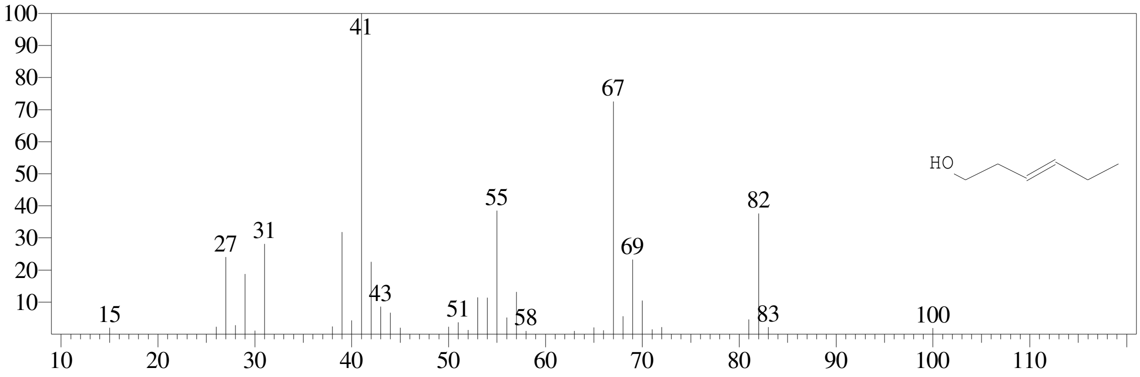

Hit#:4 Entry:6479 Library:NIST23s.lib
SI:87 Formula:C7H12O2 CAS:33467-73-1 MolWeight:128 RetIndex:931
CompName:3-Hexen-1-ol, formate, (Z)- $$ cis-.beta.-Hexenyl formate $$ cis-3-Hexenyl formate $$ cis-3-Hexen-1-ol forr

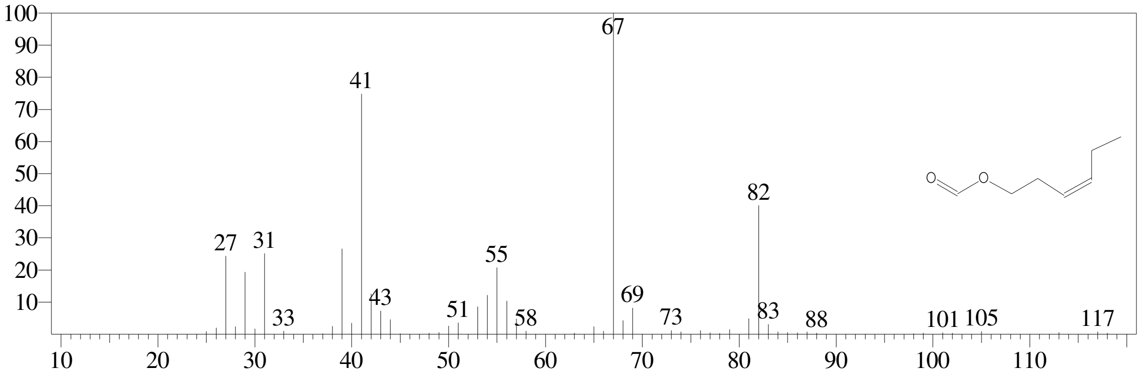

<< Target >>
Line#:3   R.Time:4.367(Scan#:225) MassPeaks:9
RawMode:Averaged 4.358-4.375(224-226) BasePeak:41.00(4269)
BG Mode:Calc. from Peak Group 1 - Event 1 Scan

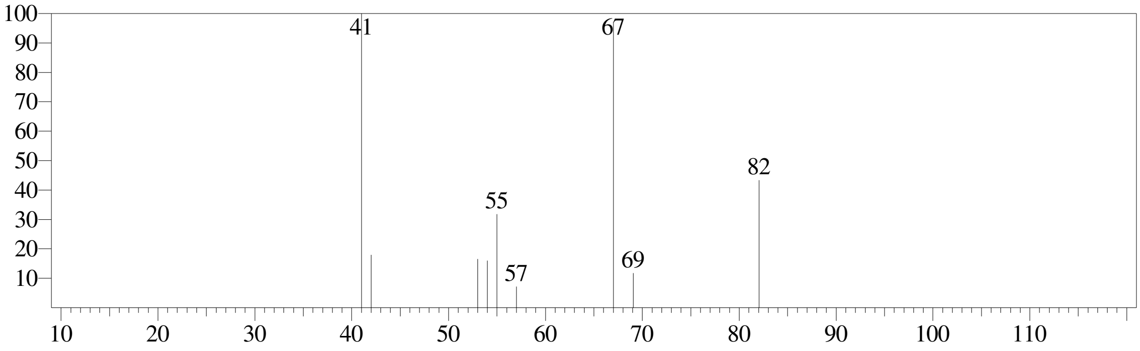

Hit#:5 Entry:8273 Library:NIST23-1.lib
SI:86 Formula:C7H12O2 CAS:33467-73-1 MolWeight:128 RetIndex:931
CompName:3-Hexen-1-ol, formate, (Z)- $$ cis-.beta.-Hexenyl formate $$ cis-3-Hexenyl formate $$ cis-3-Hexen-1-ol forr

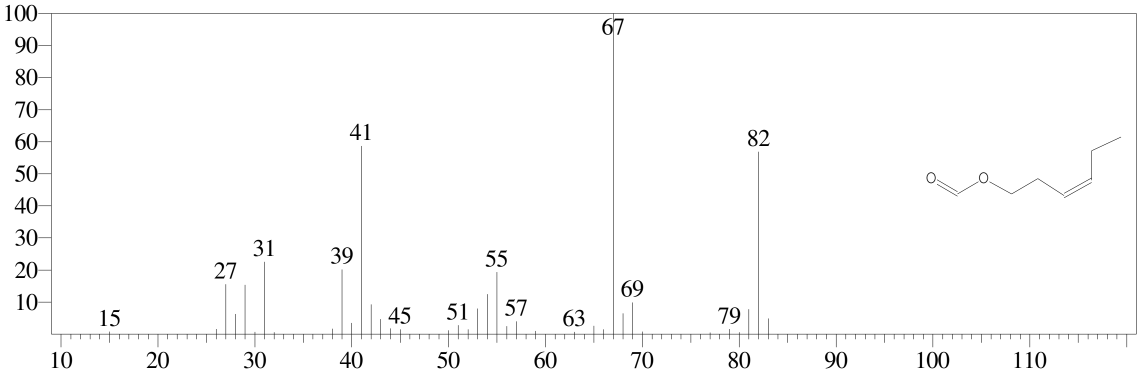

<< Target >>
Line#:4   R.Time:6.358(Scan#:464) MassPeaks:38
RawMode:Averaged 6.350-6.367(463-465) BasePeak:93.05(153161)
BG Mode:None Group 1 - Event 1 Scan

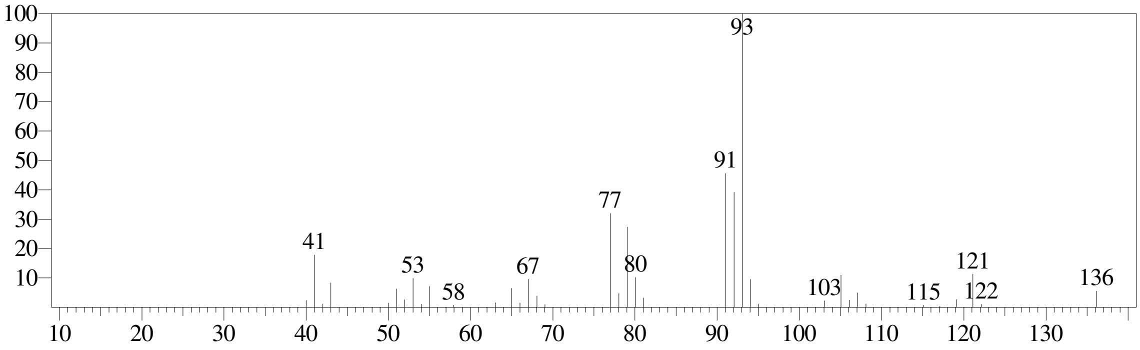

Hit#:1 Entry:8445 Library:NIST23s.lib
SI:97 Formula:C10H16 CAS:80-56-8 MolWeight:136 RetIndex:947
CompName:.alpha.-Pinene $$ Bicyclo[3.1.1]hept-2-ene, 2,6,6-trimethyl- $$ 2-Pinene $$ 2,6,6-Trimethylbicyclo[3.1.1]hep

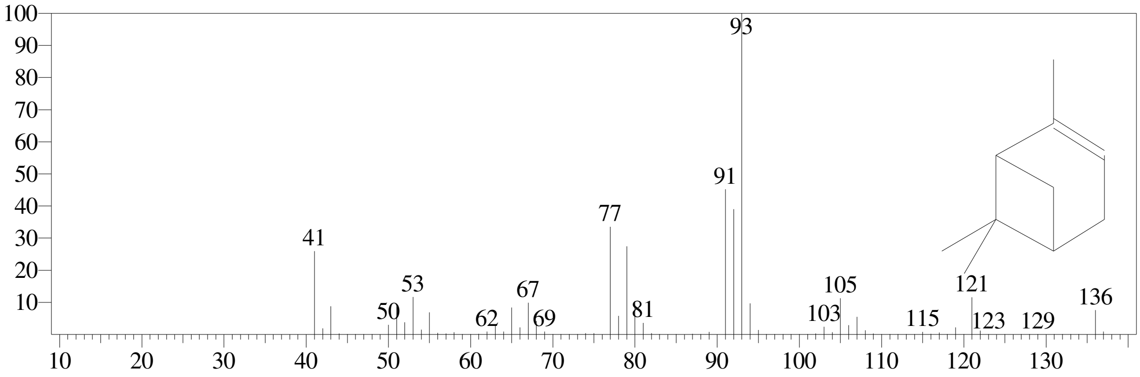

Hit#:2 Entry:8451 Library:NIST23s.lib
SI:97 Formula:C10H16 CAS:80-56-8 MolWeight:136 RetIndex:947
CompName:.alpha.-Pinene $$ Bicyclo[3.1.1]hept-2-ene, 2,6,6-trimethyl- $$ 2-Pinene $$ 2,6,6-Trimethylbicyclo[3.1.1]hep

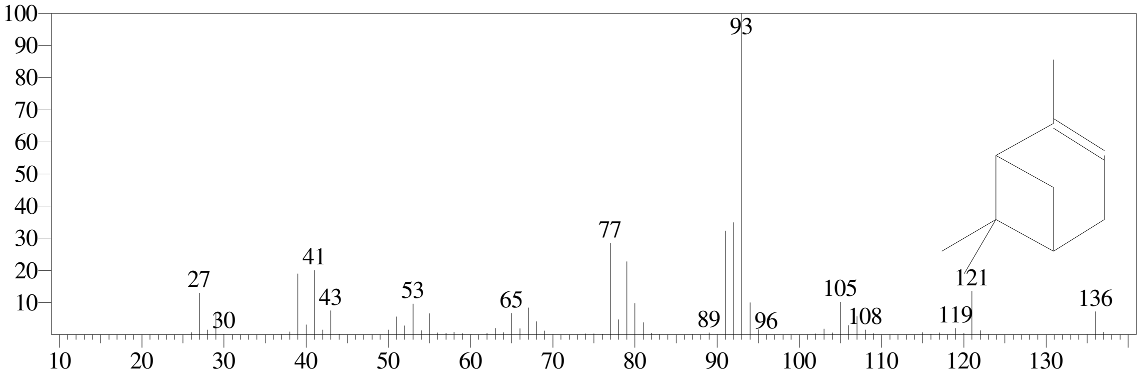

<< Target >>
Line#:4   R.Time:6.358(Scan#:464) MassPeaks:38
RawMode:Averaged 6.350-6.367(463-465) BasePeak:93.05(153161)
BG Mode:None Group 1 - Event 1 Scan

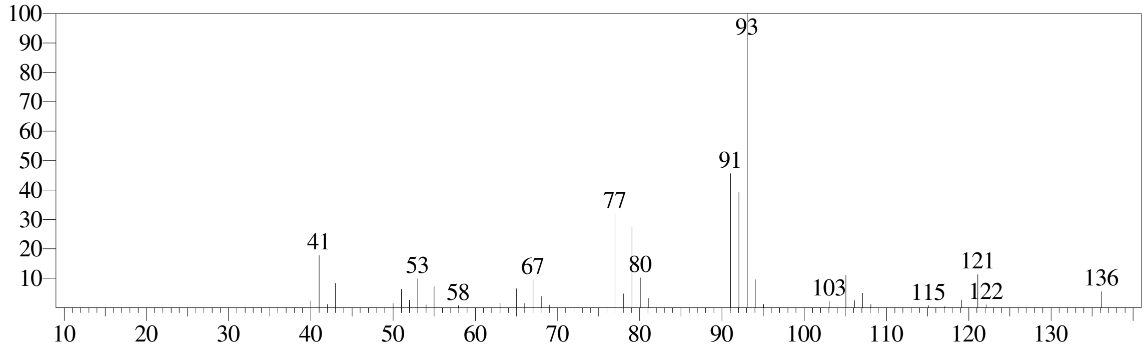

Hit#:3 Entry:11410 Library:NIST23-1.lib
SI:96 Formula:C10H16 CAS:80-56-8 MolWeight:136 RetIndex:947
CompName:.alpha.-Pinene $$ Bicyclo[3.1.1]hept-2-ene, 2,6,6-trimethyl- $$ 2-Pinene $$ 2,6,6-Trimethylbicyclo[3.1.1]hep

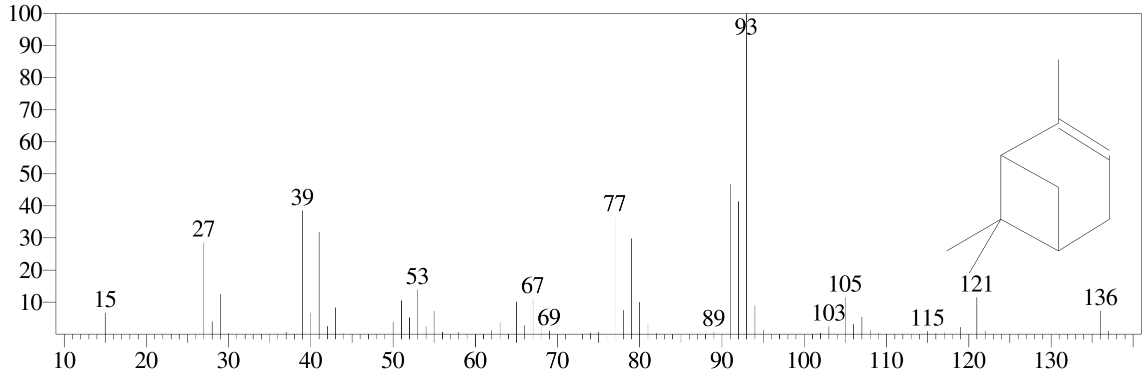

Hit#:4 Entry:8443 Library:NIST23s.lib
SI:96 Formula:C10H16 CAS:3779-61-1 MolWeight:136 RetIndex:1047
CompName:trans-.beta.-Ocimene $$ 1,3,6-Octatriene, 3,7-dimethyl-, (E)- $$ .beta.-trans-Ocimene $$ trans-3,7-Dimethyl-

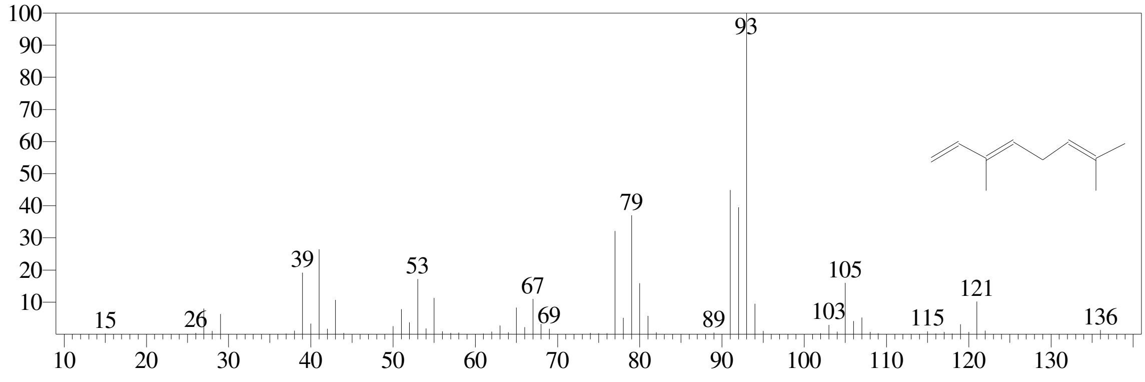

<< Target >>
Line#:4 R.Time:6.358(Scan#:464) MassPeaks:38
RawMode:Averaged 6.350-6.367(463-465) BasePeak:93.05(153161)
BG Mode:None Group 1 - Event 1 Scan

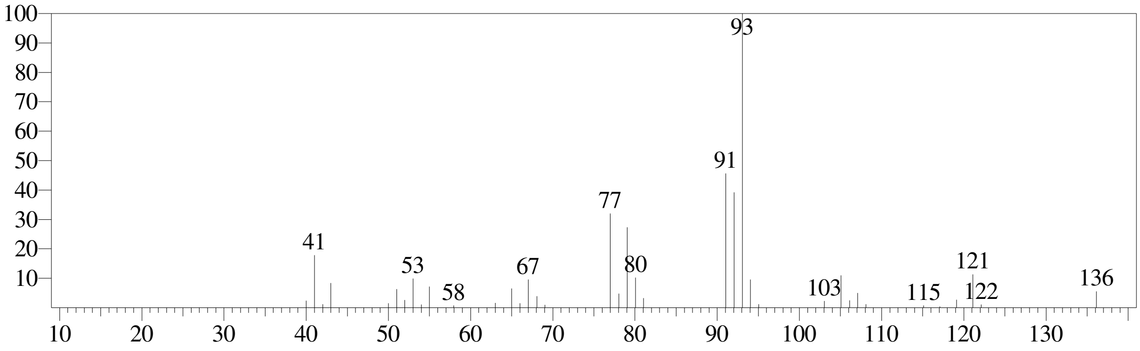

Hit#:5 Entry:8450 Library:NIST23s.lib
SI:95 Formula:C10H16 CAS:80-56-8 MolWeight:136 RetIndex:947
CompName:.alpha.-Pinene $$ Bicyclo[3.1.1]hept-2-ene, 2,6,6-trimethyl- $$ 2-Pinene $$ 2,6,6-Trimethylbicyclo[3.1.1]hep

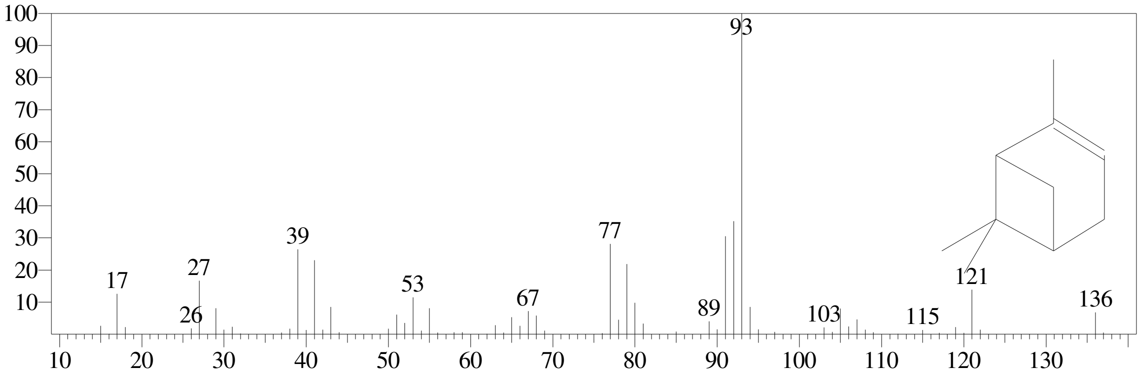

<< Target >>
Line#:5 R.Time:9.092(Scan#:792) MassPeaks:10
RawMode:Averaged 9.083-9.100(791-793) BasePeak:93.05(6699)
BG Mode:None Group 1 - Event 1 Scan

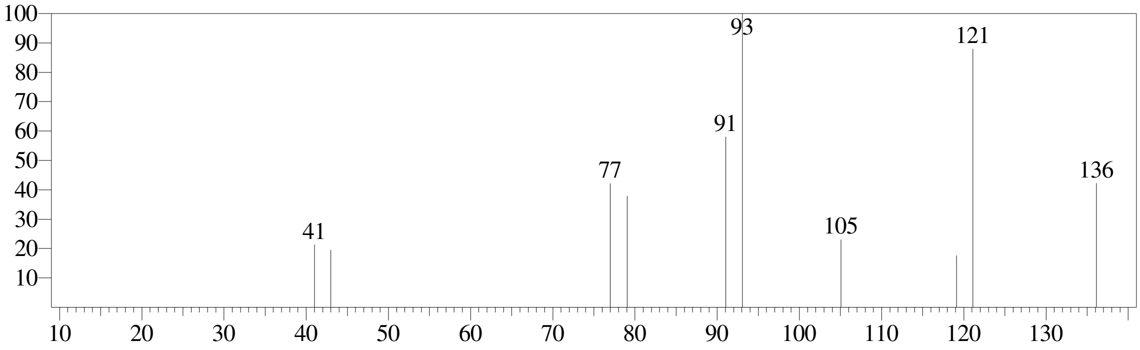

Hit#:1 Entry:8478 Library:NIST23s.lib
SI:83 Formula:C10H16 CAS:586-63-0 MolWeight:136 RetIndex:1083
CompName:Cyclohexene, 3-methyl-6-(1-methylethylidene)- $$ p-Mentha-2,4(8)-diene $$ Isoterpinolene $$ 3-Methyl-6-(

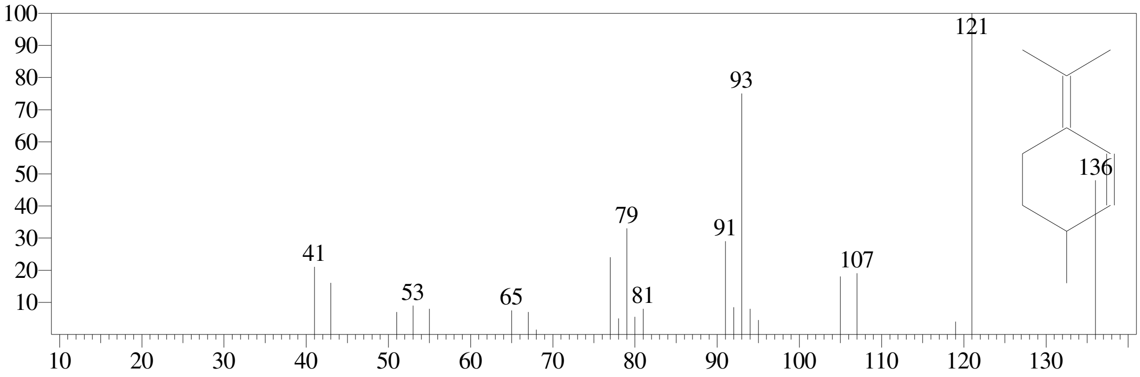

Hit#:2 Entry:11428 Library:NIST23-1.lib
SI:83 Formula:C10H16 CAS:29050-33-7 MolWeight:136 RetIndex:970
CompName:(+)-4-Carene $$ 4,7,7-Trimethylbicyclo[4.1.0]hept-2-ene # $$

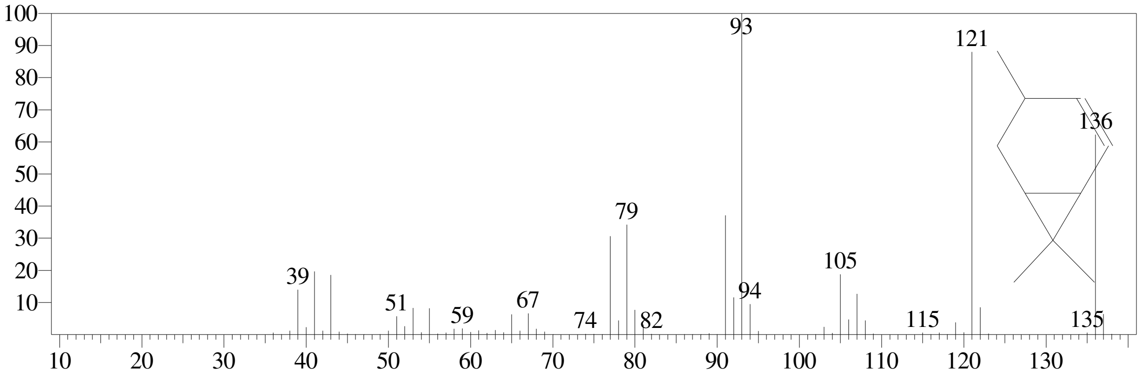

<< Target >>
Line#:5   R.Time:9.092(Scan#:792) MassPeaks:10
RawMode:Averaged 9.083-9.100(791-793) BasePeak:93.05(6699)
BG Mode:None Group 1 - Event 1 Scan

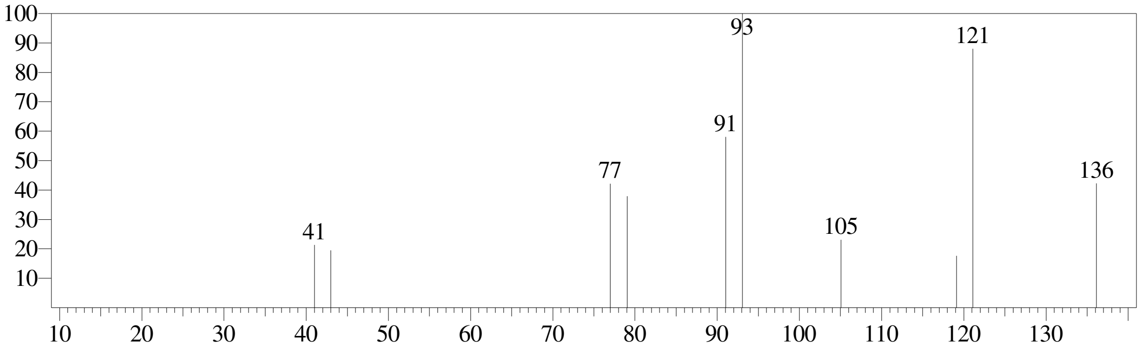

Hit#:3 Entry:11427 Library:NIST23-1.lib
SI:82 Formula:C10H16 CAS:554-61-0 MolWeight:136 RetIndex:986
CompName:2-Carene $$ Bicyclo[4.1.0]hept-2-ene, 3,7,7-trimethyl- $$ .delta.-2-Carene $$ (.+/-.)-2-Carene $$ 3,7,7-Trime

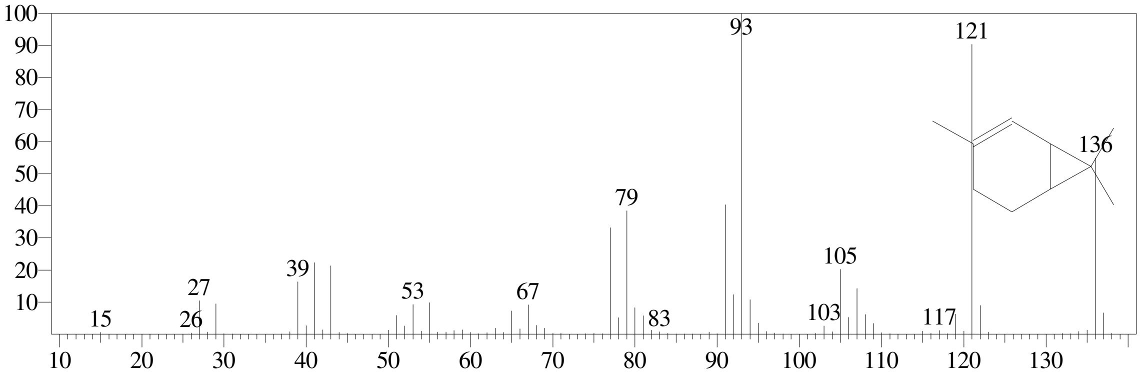

Hit#:4 Entry:11417 Library:NIST23-1.lib
SI:82 Formula:C10H16 CAS:4497-92-1 MolWeight:136 RetIndex:985
CompName:Bicyclo[4.1.0]hept-2-ene, 3,7,7-trimethyl-, (1S-cis)-

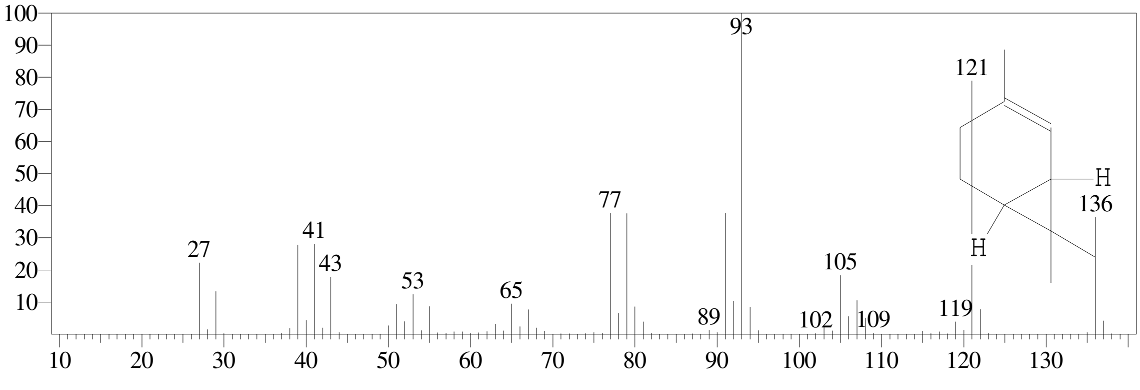

<< Target >>
Line#:5   R.Time:9.092(Scan#:792) MassPeaks:10
RawMode:Averaged 9.083-9.100(791-793) BasePeak:93.05(6699)
BG Mode:None Group 1 - Event 1 Scan

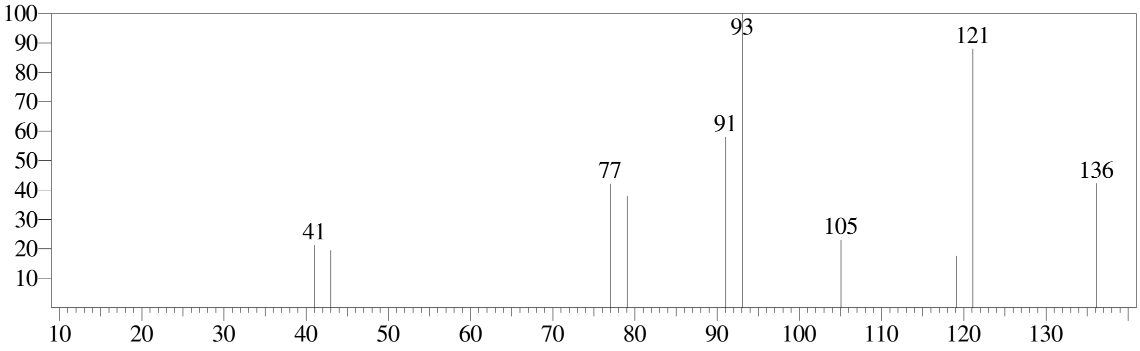

Hit#:5 Entry:8472 Library:NIST23s.lib
SI:81 Formula:C10H16 CAS:99-86-5 MolWeight:136 RetIndex:1036
CompName:1,3-Cyclohexadiene, 1-methyl-4-(1-methylethyl)- $$ .alpha.-Terpinene $$ .alpha.-Terpinen $$ p-Mentha-1,3-

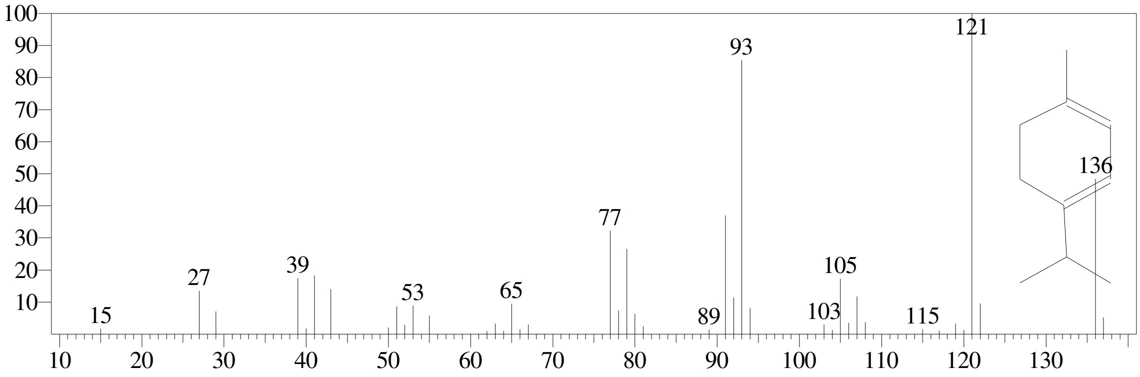

<< Target >>
Line#:6 R.Time:9.383(Scan#:827) MassPeaks:25
RawMode:Averaged 9.375-9.392(826-828) BasePeak:119.10(60519)
BG Mode:None Group 1 - Event 1 Scan

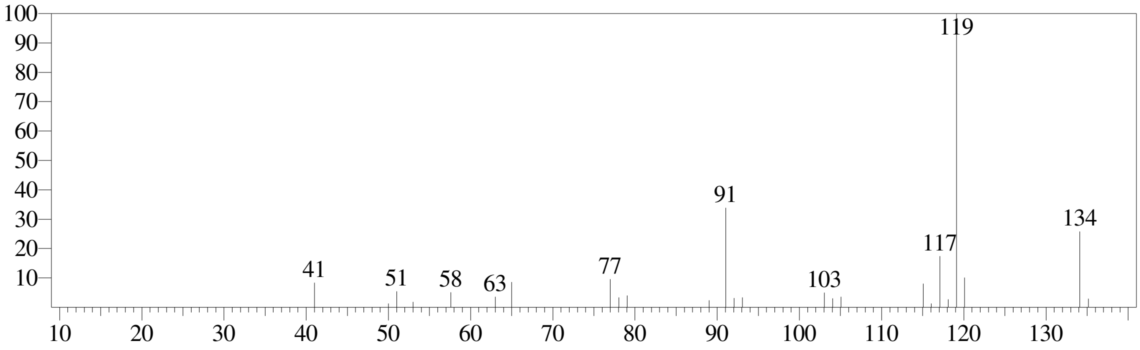

Hit#:1 Entry:7837 Library:NIST23s.lib
SI:96 Formula:C10H14 CAS:99-87-6 MolWeight:134 RetIndex:1017
CompName:p-Cymene $$ Benzene, 1-methyl-4-(1-methylethyl)- $$ p-Cimene $$ p-Cymol $$ p-Isopropyltoluene $$ p-M

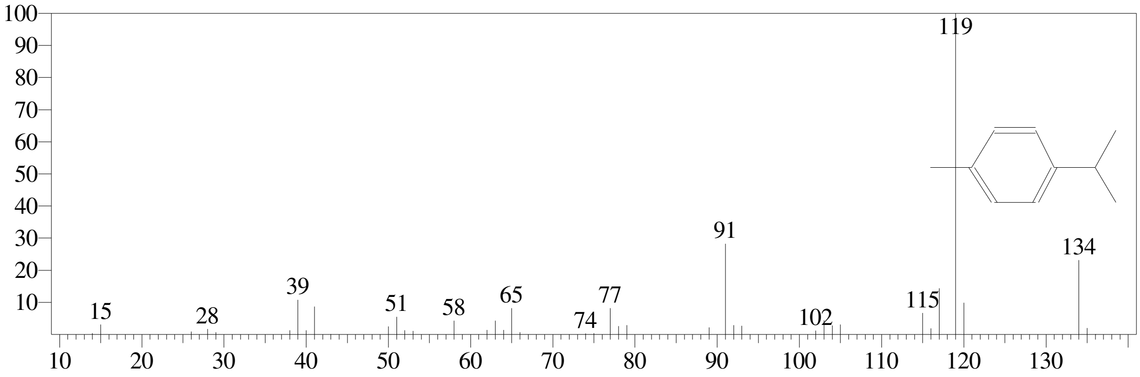

Hit#:2 Entry:7838 Library:NIST23s.lib
SI:95 Formula:C10H14 CAS:99-87-6 MolWeight:134 RetIndex:1017
CompName:p-Cymene $$ Benzene, 1-methyl-4-(1-methylethyl)- $$ p-Cimene $$ p-Cymol $$ p-Isopropyltoluene $$ p-M

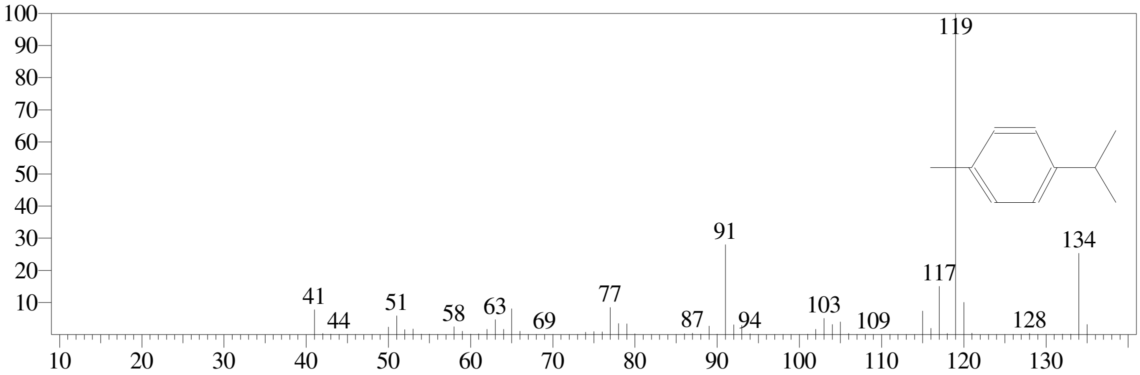

<< Target >>
Line#:6   R.Time:9.383(Scan#:827) MassPeaks:25
RawMode:Averaged 9.375-9.392(826-828) BasePeak:119.10(60519)
BG Mode:None Group 1 - Event 1 Scan

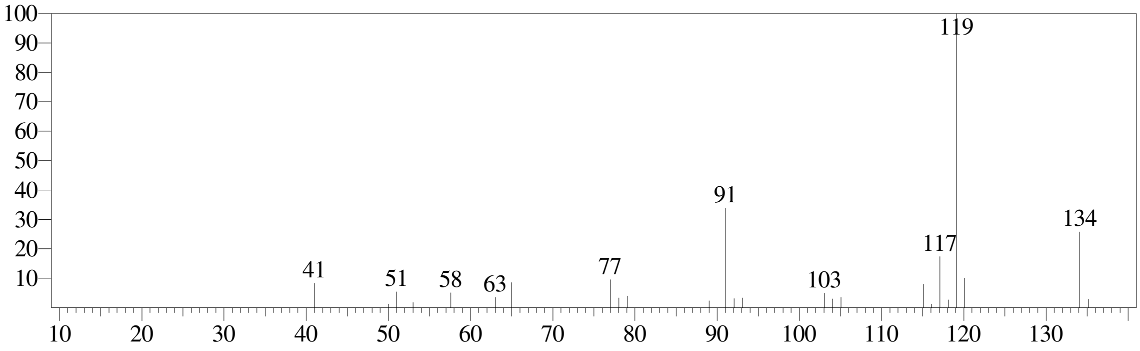

Hit#:3 Entry:10589 Library:NIST23-1.lib
SI:95 Formula:C10H14 CAS:99-87-6 MolWeight:134 RetIndex:1017
CompName:p-Cymene $$ Benzene, 1-methyl-4-(1-methylethyl)- $$ p-Cimene $$ p-Cymol $$ p-Isopropyltoluene $$ p-M

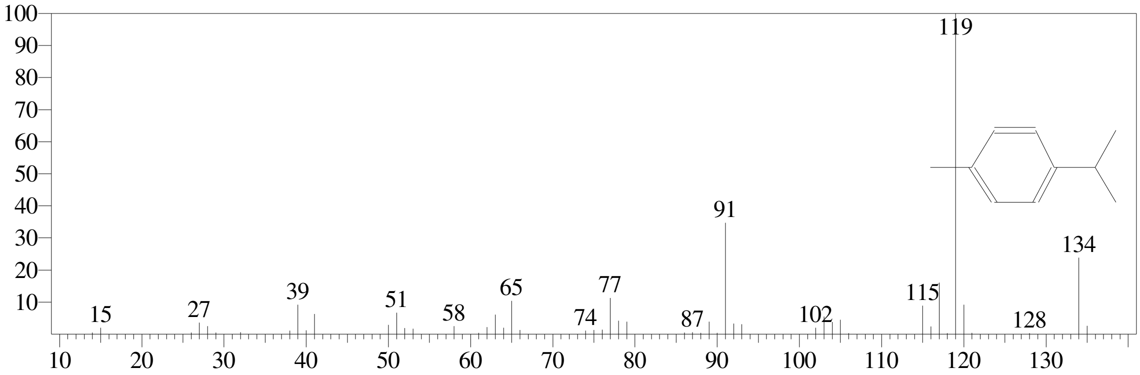

Hit#:4 Entry:7842 Library:NIST23s.lib
SI:94 Formula:C10H14 CAS:535-77-3 MolWeight:134 RetIndex:1013
CompName:Benzene, 1-methyl-3-(1-methylethyl)- $$ m-Cymene $$ .beta.-Cymene $$ m-Cymol $$ m-Isopropyltoluene $

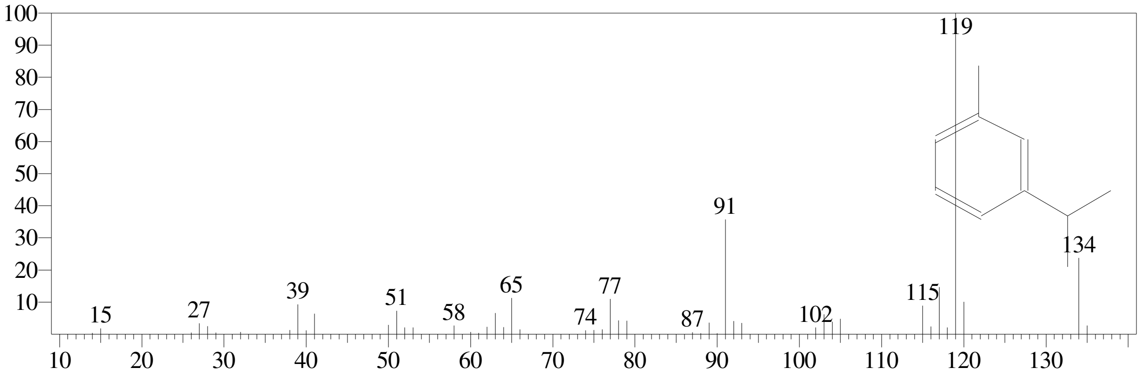

<< Target >>
Line#:6 R.Time:9.383(Scan#:827) MassPeaks:25
RawMode:Averaged 9.375-9.392(826-828) BasePeak:119.10(60519)
BG Mode:None Group 1 - Event 1 Scan

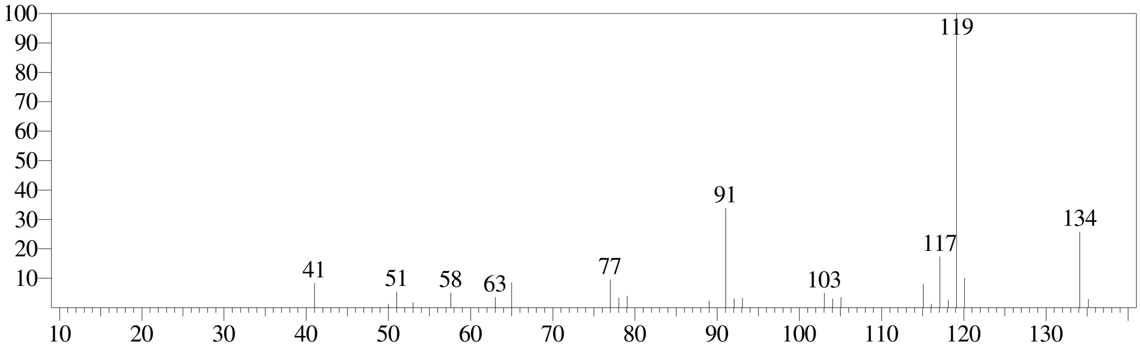

Hit#:5 Entry:7843 Library:NIST23s.lib
SI:94 Formula:C10H14 CAS:527-84-4 MolWeight:134 RetIndex:1022
CompName:o-Cymene $$ Benzene, 1-methyl-2-(1-methylethyl)- $$ o-Cymol $$ o-Isopropyltoluene $$ 1-Isopropyl-2-met

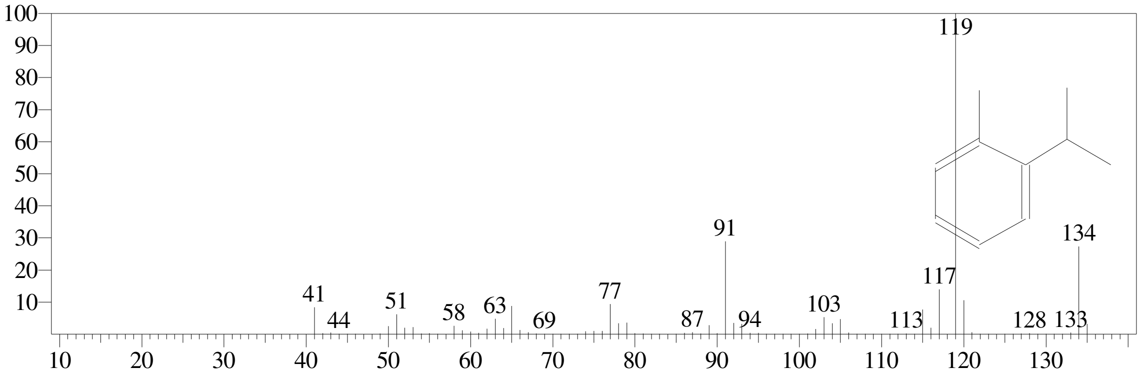

<< Target >>
Line#:7 R.Time:9.550(Scan#:847) MassPeaks:21
RawMode:Averaged 9.542-9.558(846-848) BasePeak:68.05(13221)
BG Mode:Calc. from Peak Group 1 - Event 1 Scan

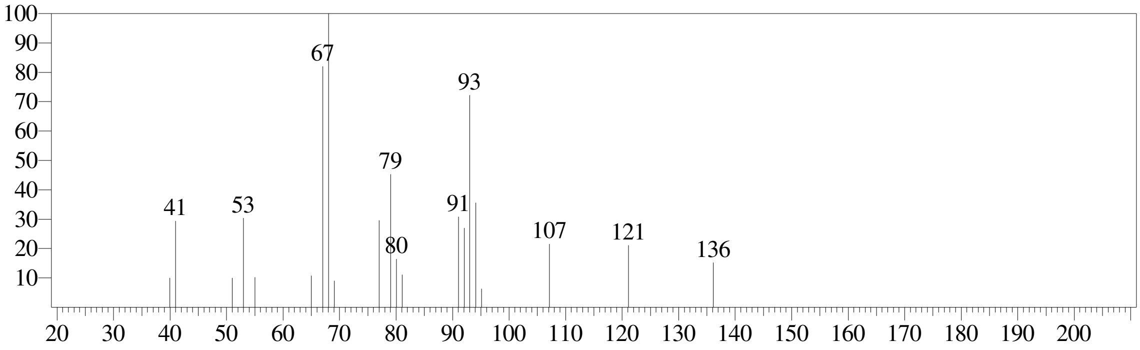

Hit#:1 Entry:8371 Library:NIST23s.lib
SI:91 Formula:C10H16 CAS:5989-27-5 MolWeight:136 RetIndex:1031
CompName:D-Limonene $$ Cyclohexene, 1-methyl-4-(1-methylethenyl)-, (R)- $$ p-Mentha-1,8-diene, (R)-(+)- $$ (+)-(R

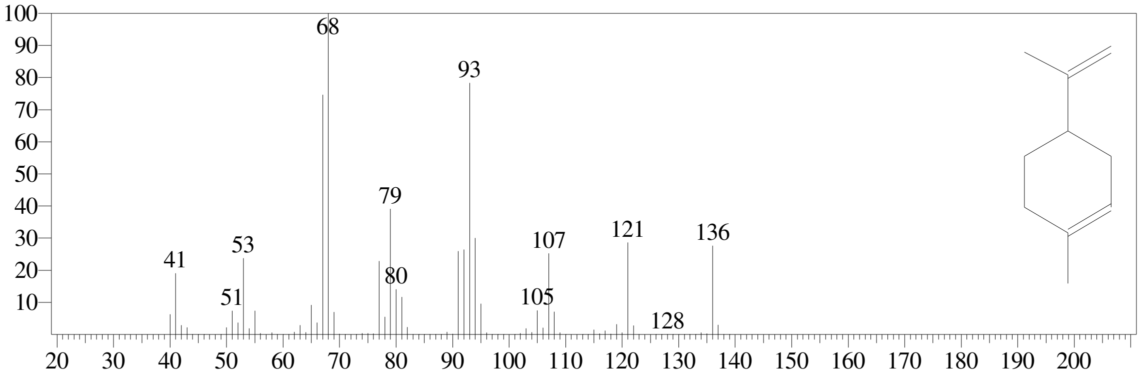

Hit#:2 Entry:8365 Library:NIST23s.lib
SI:90 Formula:C10H16 CAS:5989-54-8 MolWeight:136 RetIndex:1031
CompName:Cyclohexene, 1-methyl-4-(1-methylethenyl)-, (S)- $$ p-Mentha-1,8-diene, (S)-(-)- $$ (-)-Limonene $$ L-Lim

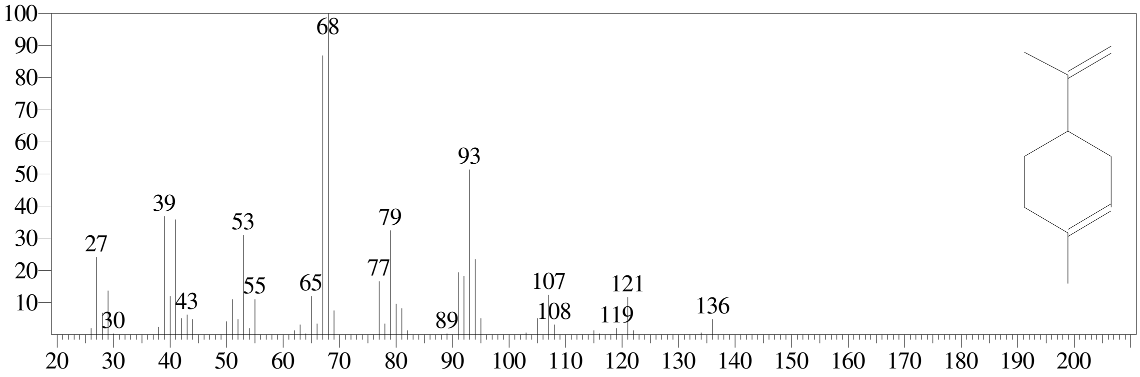

<< Target >>
Line#:7   R.Time:9.550(Scan#:847) MassPeaks:21
RawMode:Averaged 9.542-9.558(846-848) BasePeak:68.05(13221)
BG Mode:Calc. from Peak Group 1 - Event 1 Scan

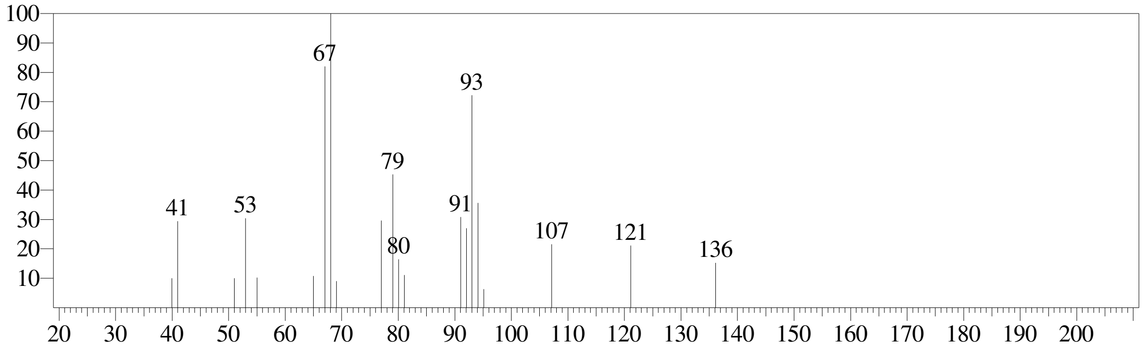

Hit#:3 Entry:8364 Library:NIST23s.lib
SI:89 Formula:C10H16 CAS:138-86-3 MolWeight:136 RetIndex:1031
CompName:Limonene $$ Cyclohexene, 1-methyl-4-(1-methylethenyl)- $$ p-Mentha-1,8-diene $$ .alpha.-Limonene $$ C

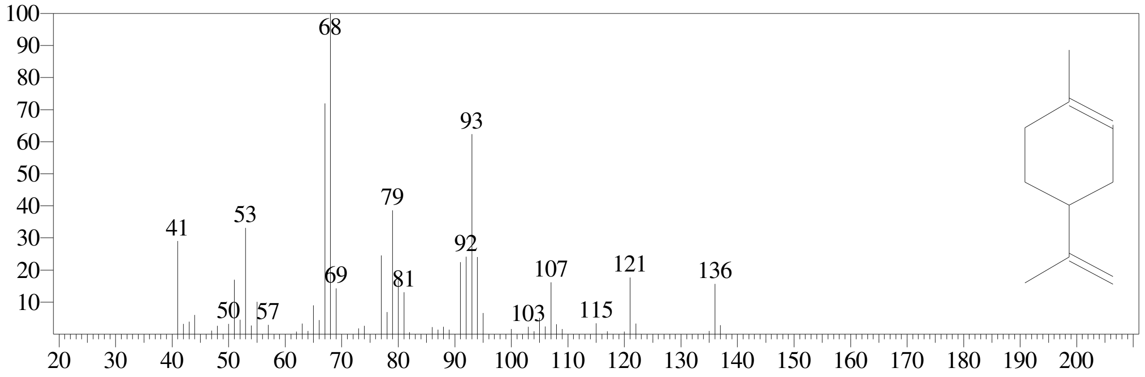

Hit#:4 Entry:11340 Library:NIST23-1.lib
SI:89 Formula:C10H16 CAS:138-86-3 MolWeight:136 RetIndex:1031
CompName:Limonene $$ Cyclohexene, 1-methyl-4-(1-methylethenyl)- $$ p-Mentha-1,8-diene $$ .alpha.-Limonene $$ C

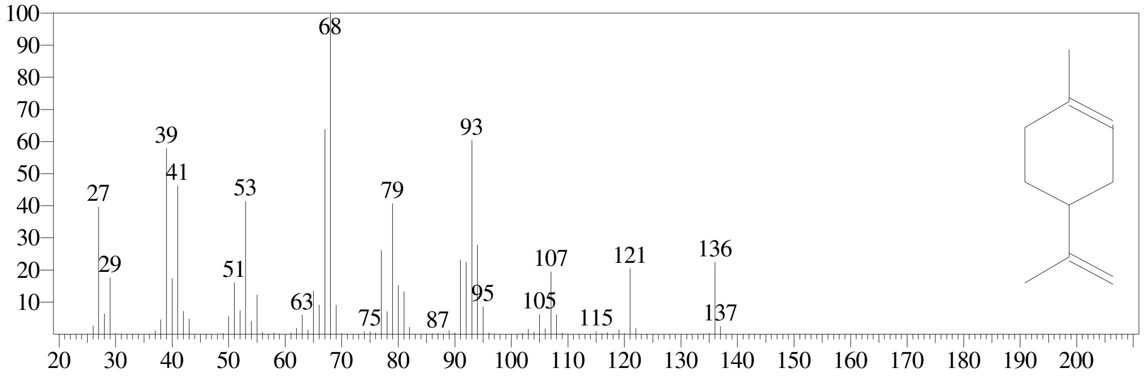

<< Target >>
Line#:7   R.Time:9.550(Scan#:847) MassPeaks:21
RawMode:Averaged 9.542-9.558(846-848) BasePeak:68.05(13221)
BG Mode:Calc. from Peak Group 1 - Event 1 Scan

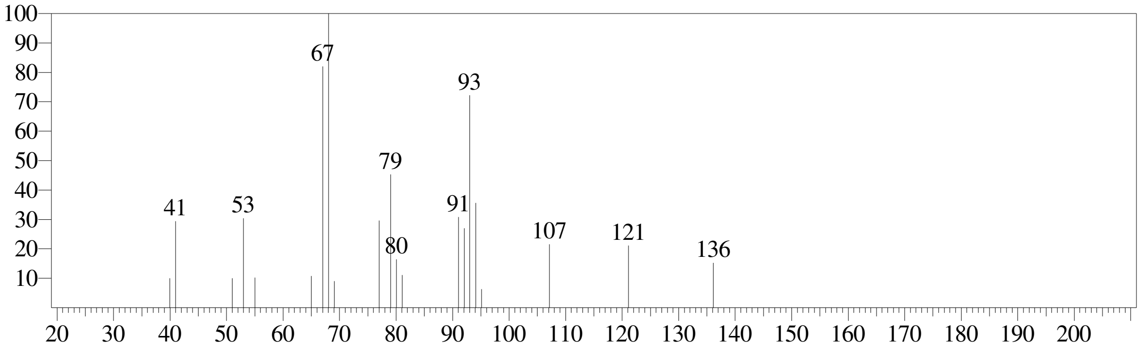

Hit#:5 Entry:11345 Library:NIST23-1.lib
SI:89 Formula:C10H16 CAS:19465-02-2 MolWeight:136 RetIndex:968
CompName:Cyclobutane, 1,2-bis(1-methylethenyl)-, trans- $$ 1,2-Diisopropenylcyclobutane # $$

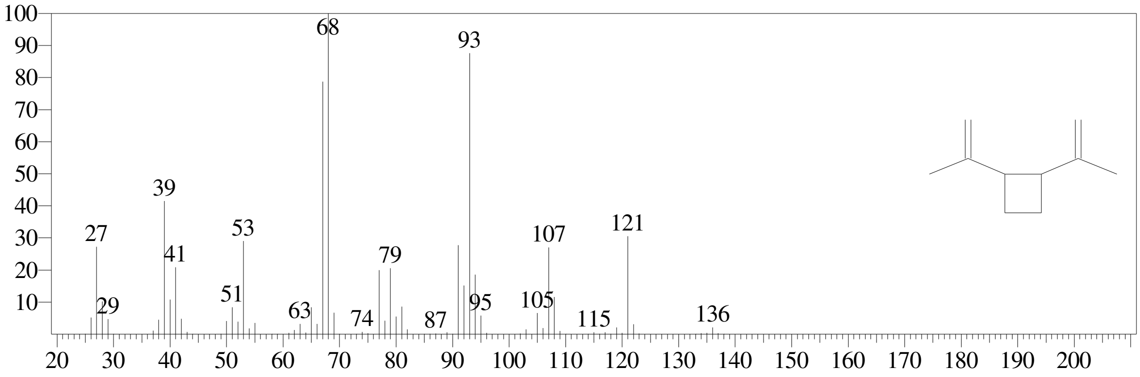

<< Target >>
Line#:8   R.Time:9.642(Scan#:858) MassPeaks:48
RawMode:Averaged 9.633-9.650(857-859) BasePeak:43.00(65888)
BG Mode:Calc. from Peak Group 1 - Event 1 Scan

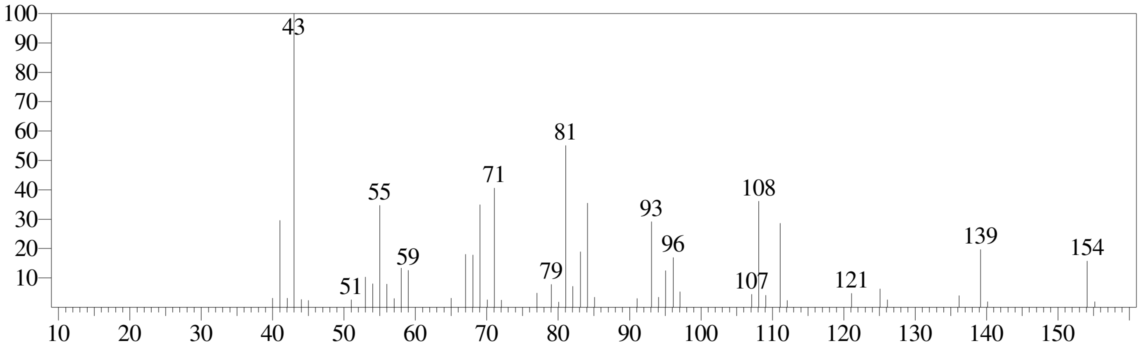

Hit#:1 Entry:12890 Library:NIST23s.lib
SI:95 Formula:C10H18O CAS:470-82-6 MolWeight:154 RetIndex:1028
CompName:Eucalyptol $$ Cineole $$ 2-Oxabicyclo[2.2.2]octane, 1,3,3-trimethyl- $$ p-Menthane, 1,8-epoxy- $$ p-Cineo

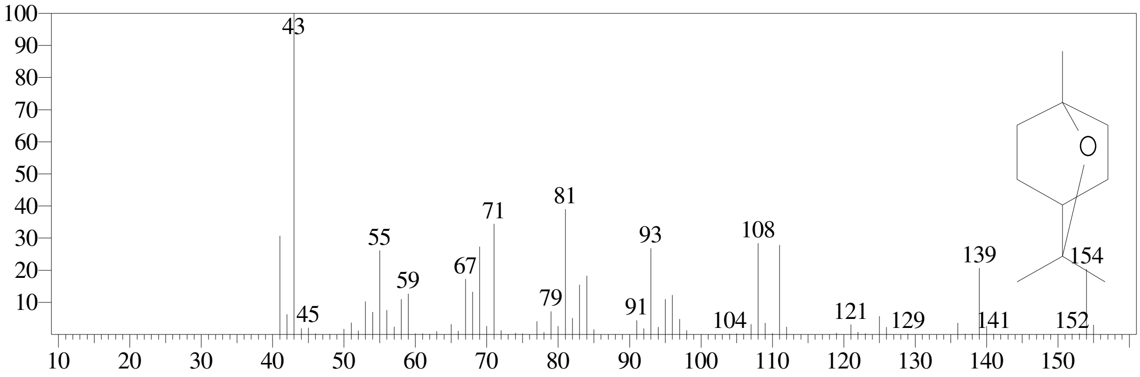

Hit#:2 Entry:12891 Library:NIST23s.lib
SI:94 Formula:C10H18O CAS:470-82-6 MolWeight:154 RetIndex:1028
CompName:Eucalyptol $$ Cineole $$ 2-Oxabicyclo[2.2.2]octane, 1,3,3-trimethyl- $$ p-Menthane, 1,8-epoxy- $$ p-Cineo

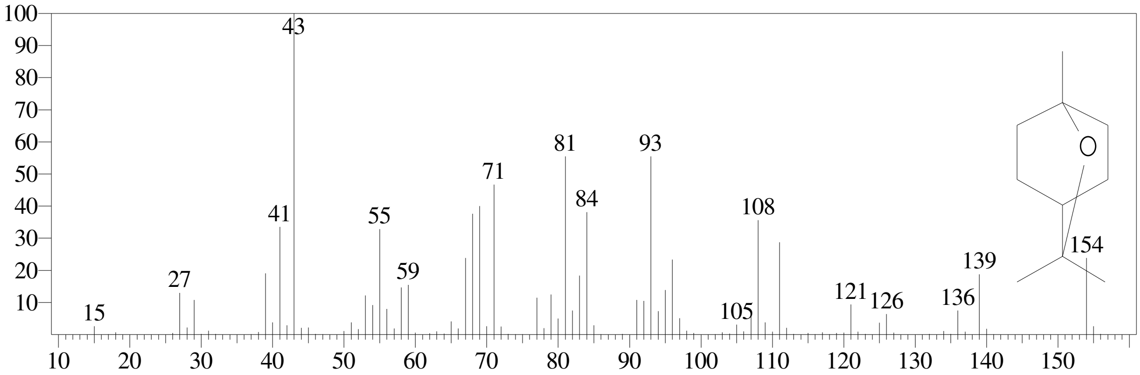

<< Target >>
Line#:8 R.Time:9.642(Scan#:858) MassPeaks:48
RawMode:Averaged 9.633-9.650(857-859) BasePeak:43.00(65888)
BG Mode:Calc. from Peak Group 1 - Event 1 Scan

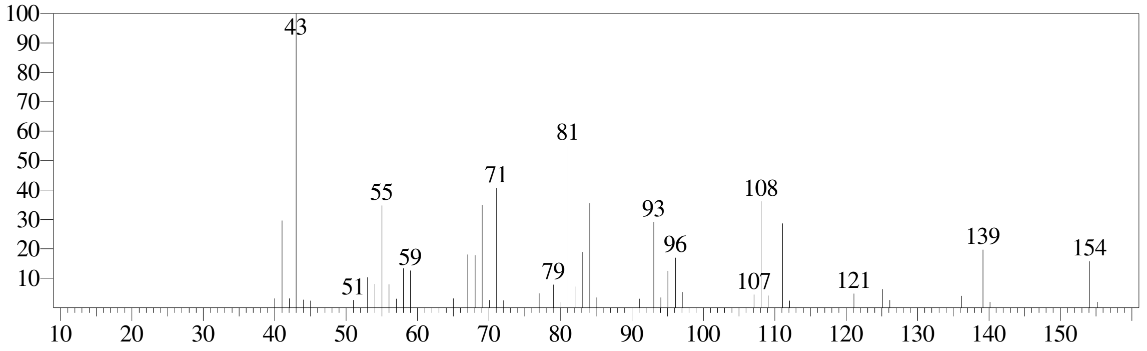

Hit#:3 Entry:20951 Library:NIST23-1.lib
SI:94 Formula:C10H18O CAS:470-82-6 MolWeight:154 RetIndex:1028
CompName:Eucalyptol $$ Cineole $$ 2-Oxabicyclo[2.2.2]octane, 1,3,3-trimethyl- $$ p-Menthane, 1,8-epoxy- $$ p-Cineo

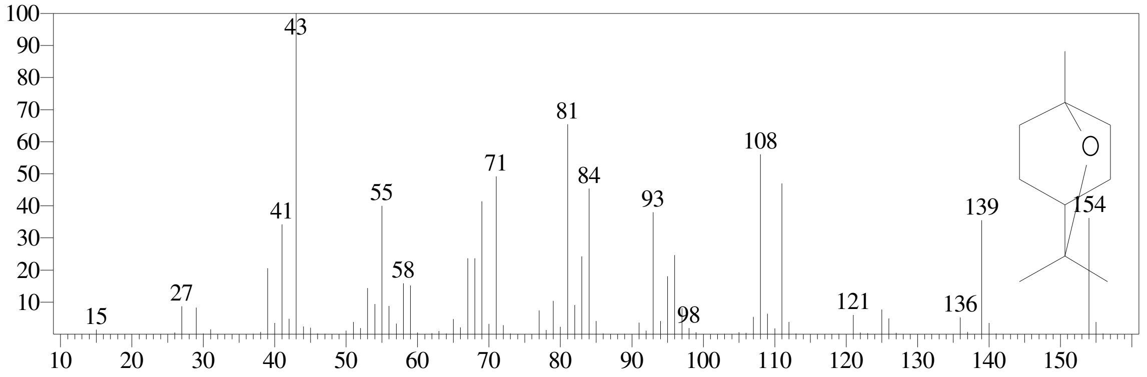

Hit#:4 Entry:12889 Library:NIST23s.lib
SI:93 Formula:C10H18O CAS:470-82-6 MolWeight:154 RetIndex:1028
CompName:Eucalyptol $$ Cineole $$ 2-Oxabicyclo[2.2.2]octane, 1,3,3-trimethyl- $$ p-Menthane, 1,8-epoxy- $$ p-Cineo

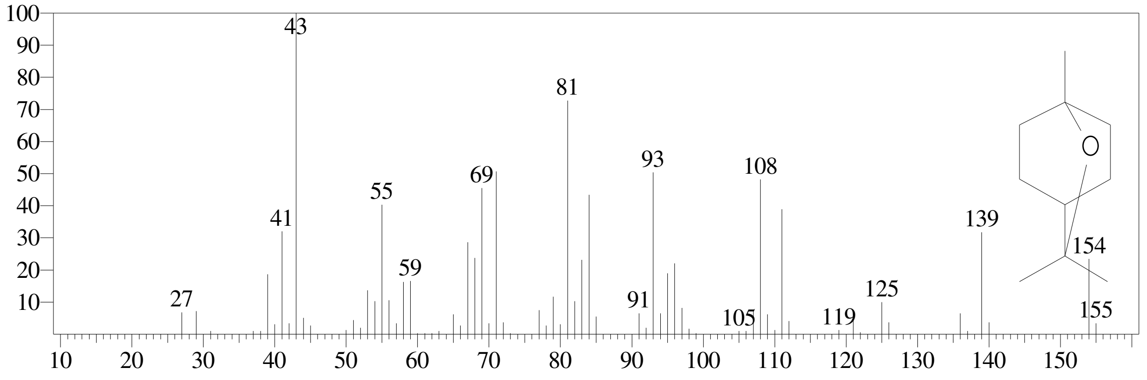

<< Target >>
Line#:8   R.Time:9.642(Scan#:858) MassPeaks:48
RawMode:Averaged 9.633-9.650(857-859) BasePeak:43.00(65888)
BG Mode:Calc. from Peak Group 1 - Event 1 Scan

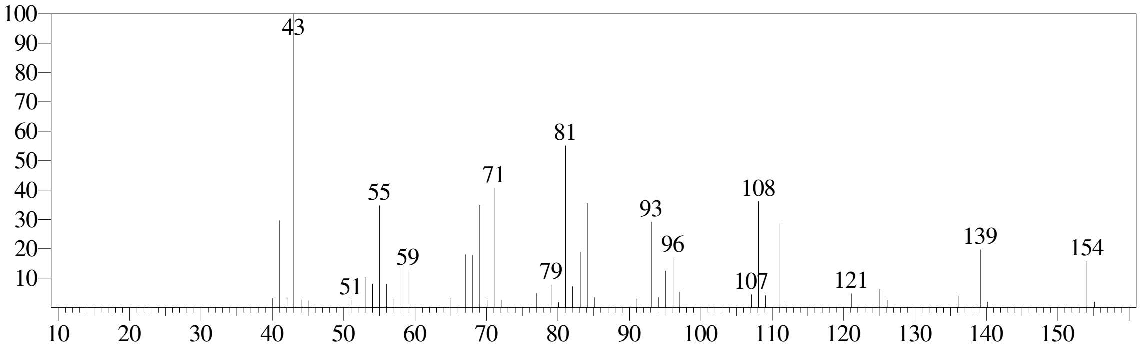

Hit#:5 Entry:12893 Library:NIST23s.lib
SI:93 Formula:C10H18O CAS:470-82-6 MolWeight:154 RetIndex:1028
CompName:Eucalyptol $$ Cineole $$ 2-Oxabicyclo[2.2.2]octane, 1,3,3-trimethyl- $$ p-Menthane, 1,8-epoxy- $$ p-Cineo

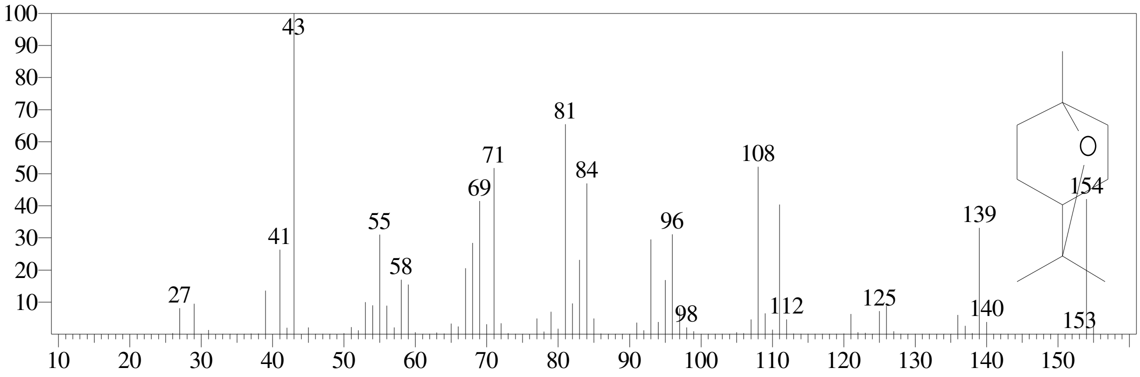

<< Target >>
Line#:9 R.Time:10.725(Scan#:988) MassPeaks:18
RawMode:Averaged 10.717-10.733(987-989) BasePeak:93.05(14148)
BG Mode:None Group 1 - Event 1 Scan

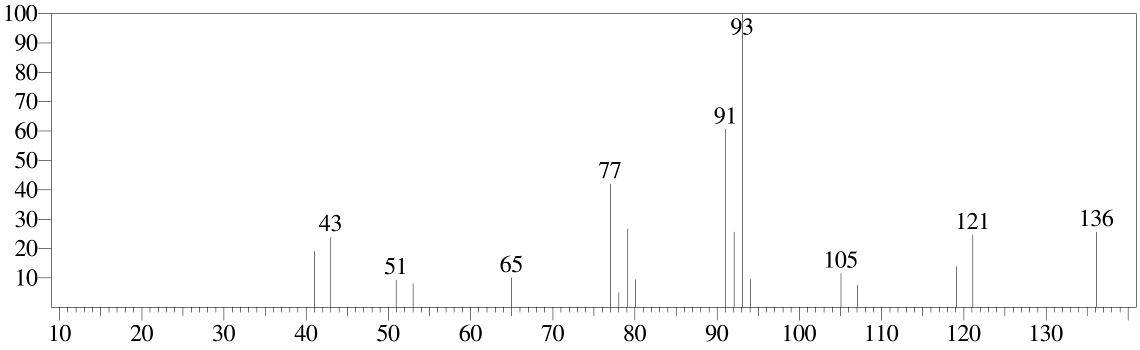

Hit#:1 Entry:8426 Library:NIST23s.lib
SI:92 Formula:C10H16 CAS:99-85-4 MolWeight:136 RetIndex:1038
CompName:.gamma.-Terpinene $$ 1,4-Cyclohexadiene, 1-methyl-4-(1-methylethyl)- $$ .gamma.-Terpinen $$ p-Mentha-

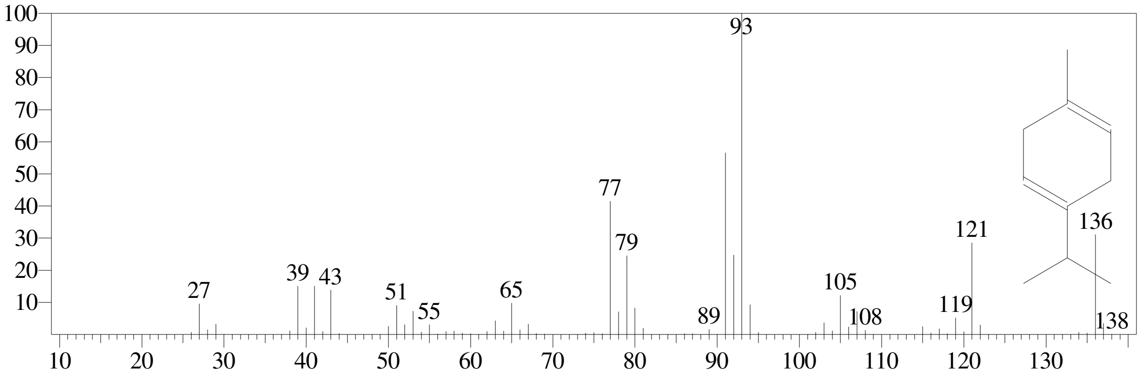

Hit#:2 Entry:8427 Library:NIST23s.lib
SI:91 Formula:C10H16 CAS:99-85-4 MolWeight:136 RetIndex:1038
CompName:.gamma.-Terpinene $$ 1,4-Cyclohexadiene, 1-methyl-4-(1-methylethyl)- $$ .gamma.-Terpinen $$ p-Mentha-

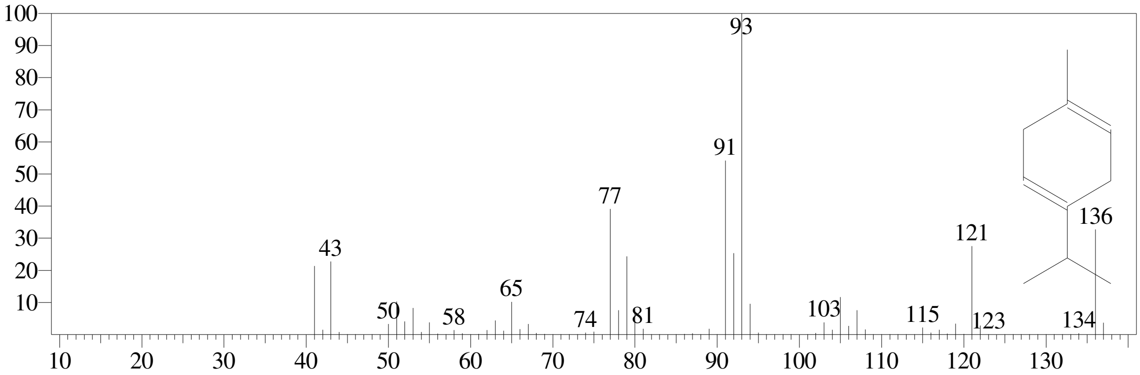

<< Target >>
Line#:9 R.Time:10.725(Scan#:988) MassPeaks:18
RawMode:Averaged 10.717-10.733(987-989) BasePeak:93.05(14148)
BG Mode:None Group 1 - Event 1 Scan

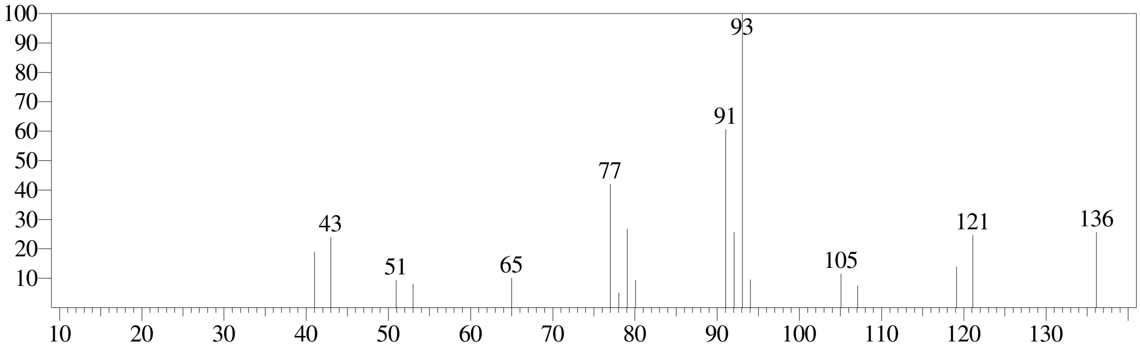

Hit#:3 Entry:8424 Library:NIST23s.lib
SI:90 Formula:C10H16 CAS:99-85-4 MolWeight:136 RetIndex:1038
CompName:.gamma.-Terpinene $$ 1,4-Cyclohexadiene, 1-methyl-4-(1-methylethyl)- $$ .gamma.-Terpinen $$ p-Mentha-

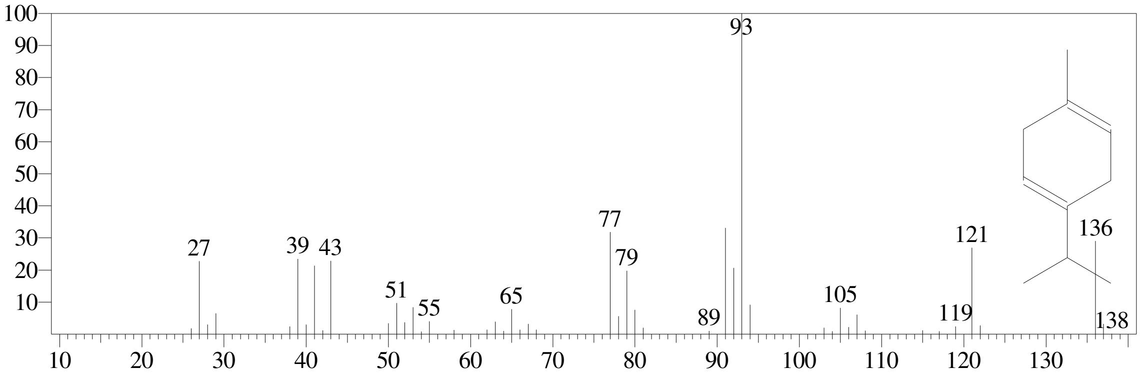

Hit#:4 Entry:8425 Library:NIST23s.lib
SI:89 Formula:C10H16 CAS:99-85-4 MolWeight:136 RetIndex:1038
CompName:.gamma.-Terpinene $$ 1,4-Cyclohexadiene, 1-methyl-4-(1-methylethyl)- $$ .gamma.-Terpinen $$ p-Mentha-

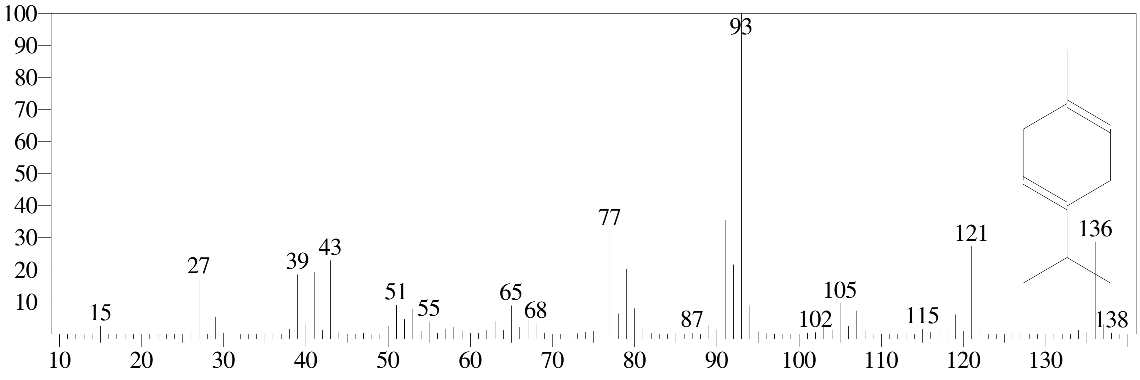

<< Target >>
Line#:9 R.Time:10.725(Scan#:988) MassPeaks:18
RawMode:Averaged 10.717-10.733(987-989) BasePeak:93.05(14148)
BG Mode:None Group 1 - Event 1 Scan

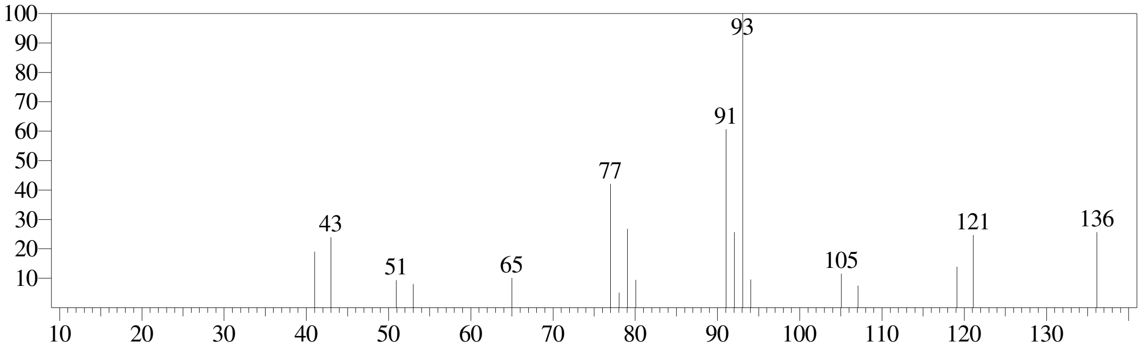

Hit#:5 Entry:8446 Library:NIST23s.lib
SI:89 Formula:C10H16 CAS:99-85-4 MolWeight:136 RetIndex:1038
CompName:.gamma.-Terpinene $$ 1,4-Cyclohexadiene, 1-methyl-4-(1-methylethyl)- $$ .gamma.-Terpinen $$ p-Mentha-

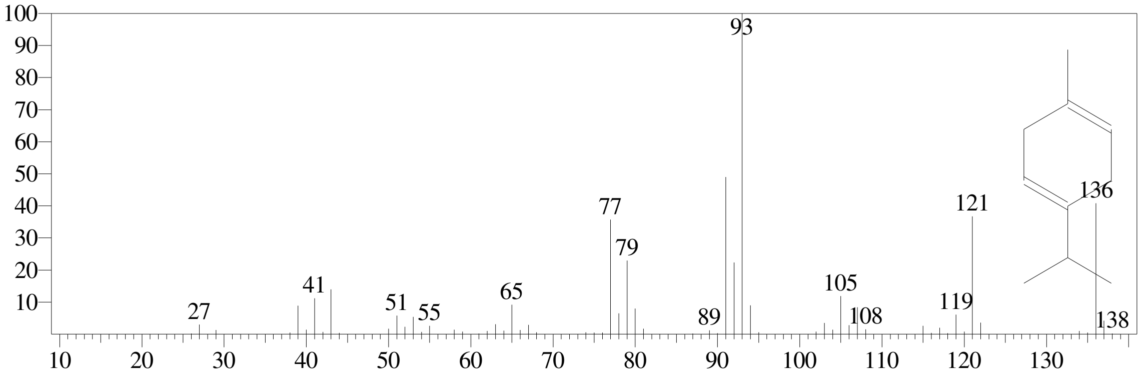

<< Target >>
Line#:10 R.Time:15.100(Scan#:1513) MassPeaks:12
RawMode:Averaged 15.092-15.108(1512-1514) BasePeak:95.10(6311)
BG Mode:Calc. from Peak Group 1 - Event 1 Scan

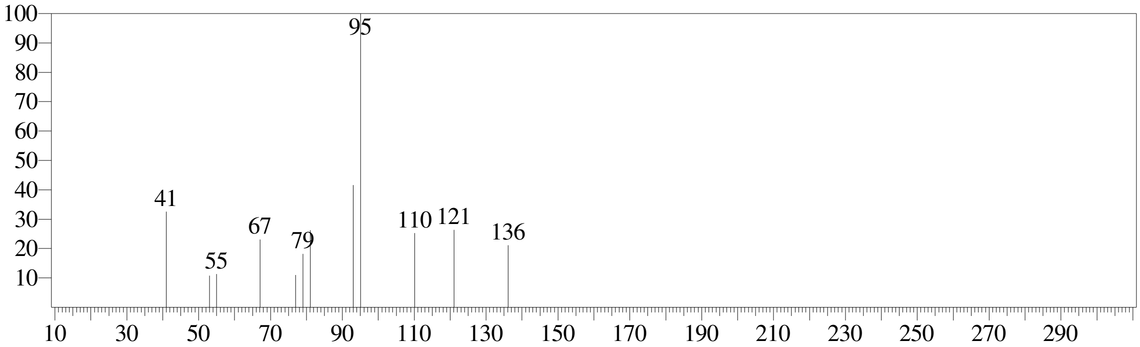

Hit#:1 Entry:33826 Library:NIST23-1.lib
SI:78 Formula:C10H17Cl CAS:30462-53-4 MolWeight:172 RetIndex:1165
CompName:Bicyclo[2.2.1]heptane, 2-chloro-1,7,7-trimethyl-, (1R-endo)- $$ 2-Chloro-1,7,7-trimethylbicyclo[2.2.1]heptar

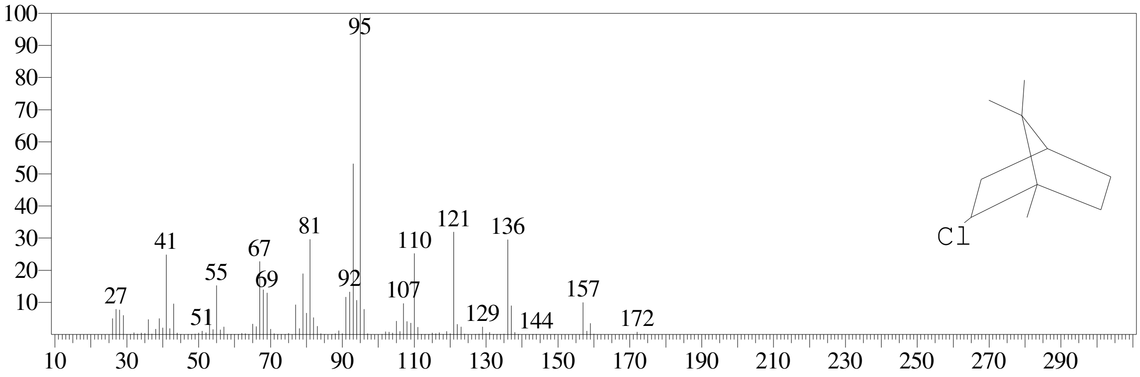

Hit#:2 Entry:32803 Library:NIST23s.lib
SI:78 Formula:C12H17F3O2 CAS:28587-55-5 MolWeight:250 RetIndex:1141
CompName:Borneol, trifluoroacetate (ester) $$ 1,7,7-Trimethylbicyclo[2.2.1]hept-2-yl trifluoroacetate # $$

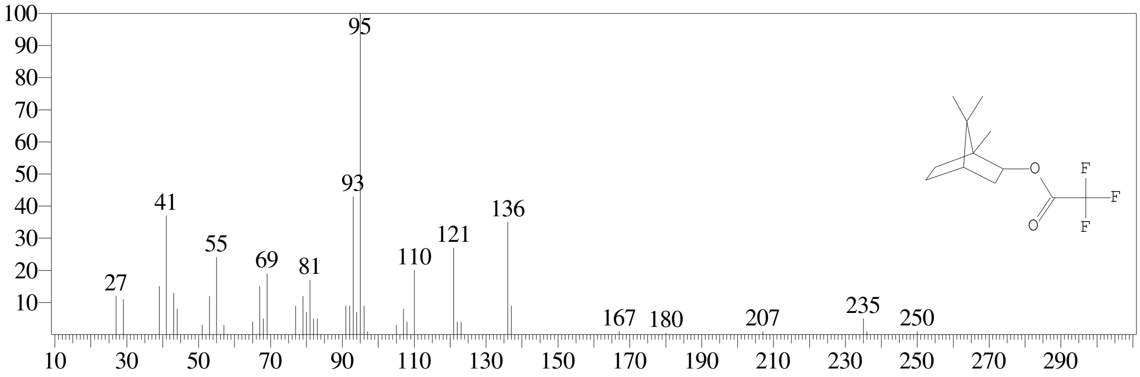

<< Target >>
Line#:10   R.Time:15.100(Scan#:1513) MassPeaks:12
RawMode:Averaged 15.092-15.108(1512-1514) BasePeak:95.10(6311)
BG Mode:Calc. from Peak Group 1 - Event 1 Scan

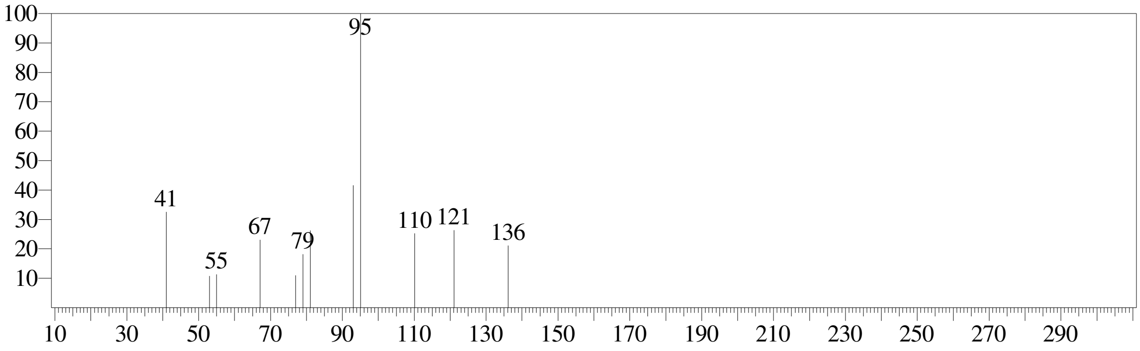

Hit#:3 Entry:116904 Library:NIST23-1.lib
SI:77 Formula:C12H17F3O2 CAS:28587-55-5 MolWeight:250 RetIndex:1141
CompName:Borneol, trifluoroacetate (ester) $$ 1,7,7-Trimethylbicyclo[2.2.1]hept-2-yl trifluoroacetate # $$

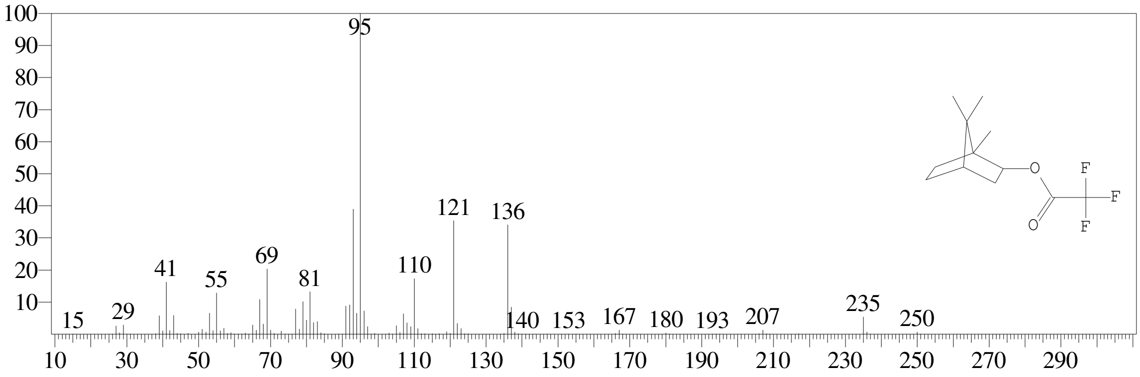

Hit#:4 Entry:19801 Library:NIST23s.lib
SI:76 Formula:C11H18O2 CAS:7492-41-3 MolWeight:182 RetIndex:1240
CompName:Bicyclo[2.2.1]heptan-2-ol, 1,7,7-trimethyl-, formate, endo- $$ Borneol, formate $$ Bornyl formate $$ 1,7,7-T

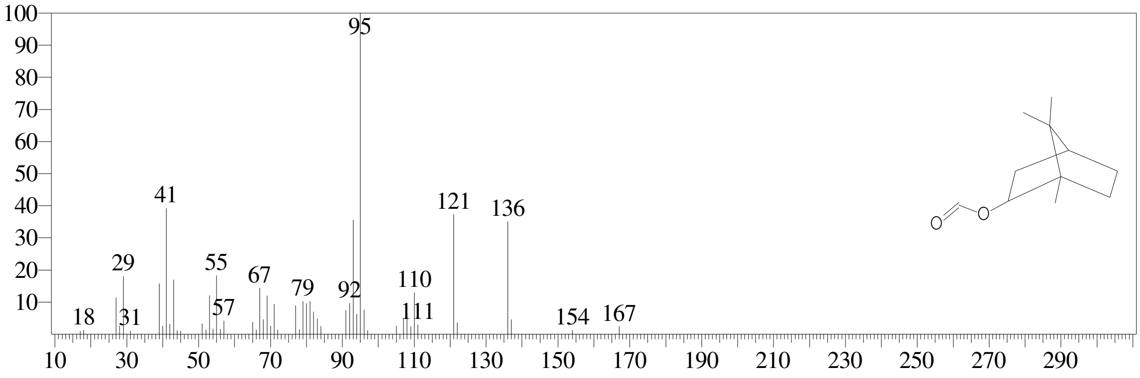

<< Target >>
Line#:10 R.Time:15.100(Scan#:1513) MassPeaks:12
RawMode:Averaged 15.092-15.108(1512-1514) BasePeak:95.10(6311)
BG Mode:Calc. from Peak Group 1 - Event 1 Scan

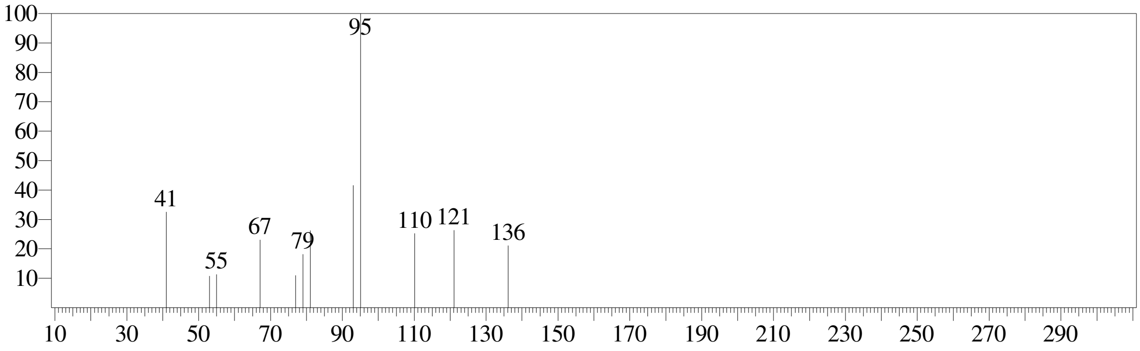

Hit#:5 Entry:183318 Library:NIST23-1.lib
SI:76 Formula:C13H17F5O2 CAS:0-00-0 MolWeight:300 RetIndex:1162
CompName:Borneol, pentafluoropropionate

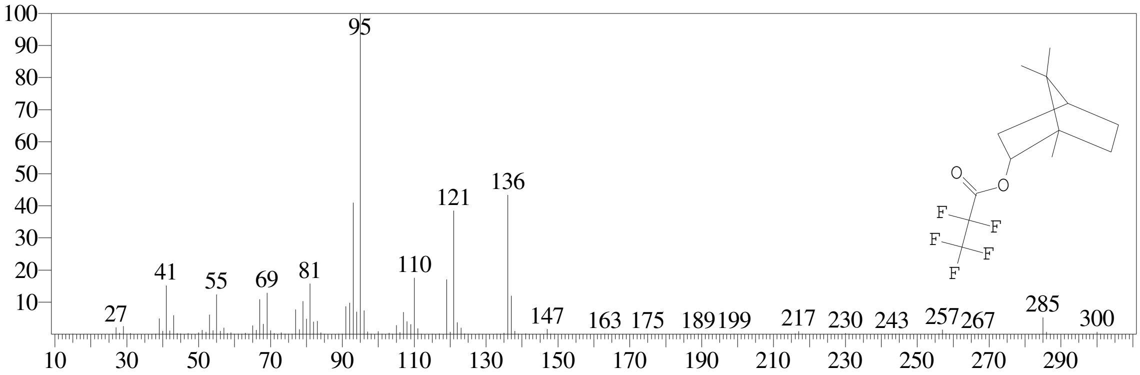

<< Target >>
Line#:11 R.Time:16.300(Scan#:1657) MassPeaks:15
RawMode:Averaged 16.292-16.308(1656-1658) BasePeak:59.00(7571)
BG Mode:None Group 1 - Event 1 Scan

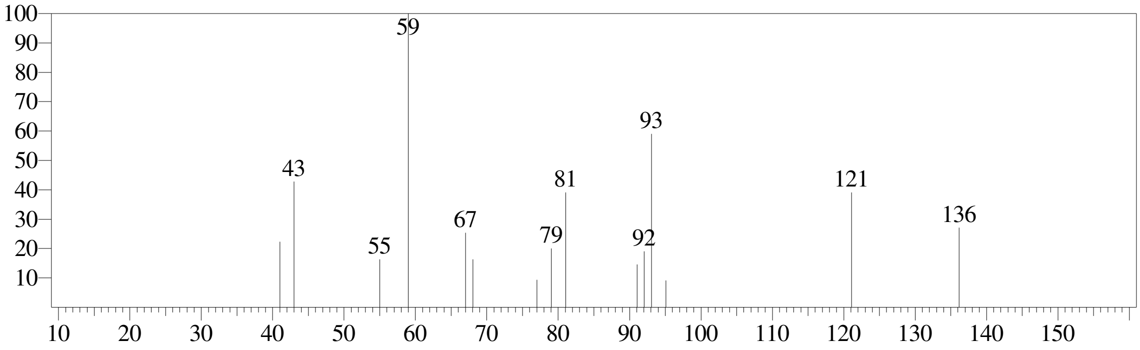

Hit#:1 Entry:21000 Library:NIST23-1.lib
SI:87 Formula:C10H18O CAS:98-55-5 MolWeight:154 RetIndex:1183
CompName:.alpha.-Terpineol $$ 3-Cyclohexene-1-methanol, .alpha.,.alpha.4-trimethyl- $$ p-Menth-1-en-8-ol $$ Terpine

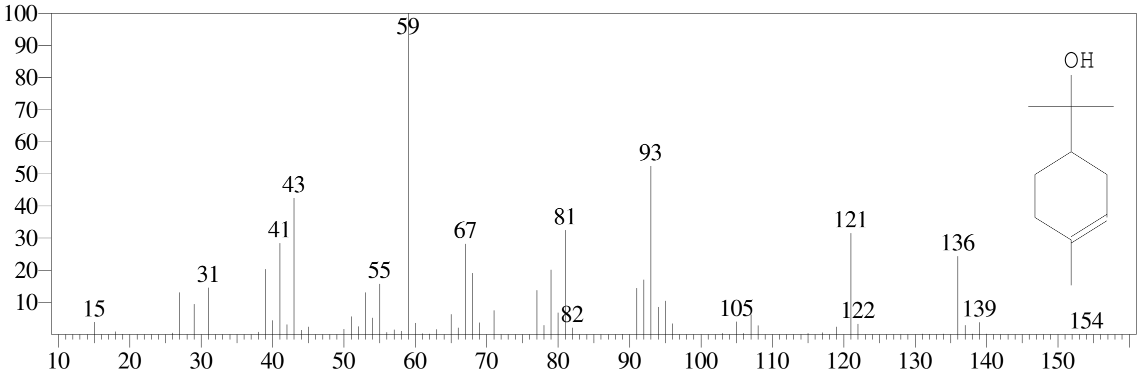

Hit#:2 Entry:20999 Library:NIST23-1.lib
SI:86 Formula:C10H18O CAS:7785-53-7 MolWeight:154 RetIndex:1183
CompName:3-Cyclohexene-1-methanol, .alpha.,.alpha.,4-trimethyl-, (R)- $$ 3-Cyclohexene-1-methanol, .alpha.,.alpha.,4-

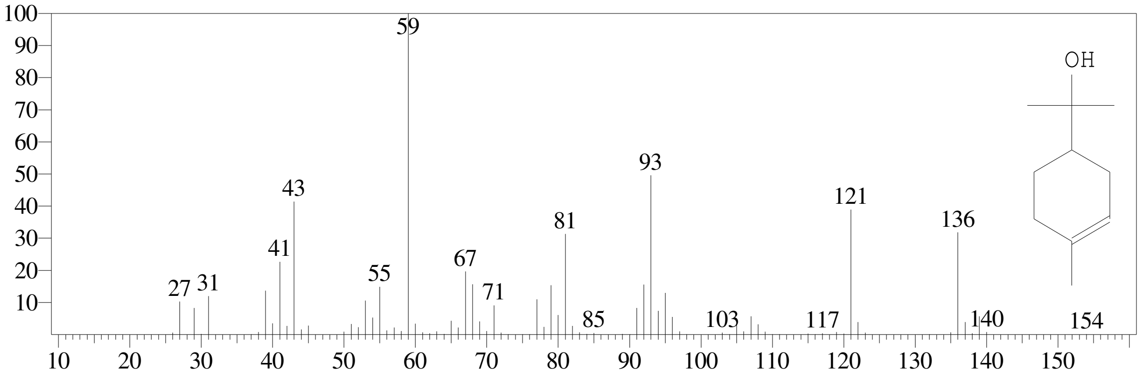

<< Target >>
Line#:11   R.Time:16.300(Scan#:1657) MassPeaks:15
RawMode:Averaged 16.292-16.308(1656-1658) BasePeak:59.00(7571)
BG Mode:None Group 1 - Event 1 Scan

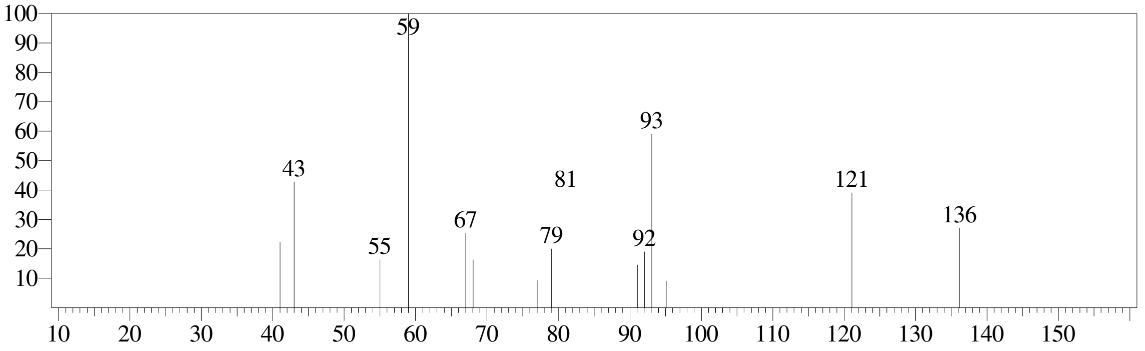

Hit#:3 Entry:12921 Library:NIST23s.lib
SI:86 Formula:C10H18O CAS:98-55-5 MolWeight:154 RetIndex:1183
CompName:.alpha.-Terpineol $$ 3-Cyclohexene-1-methanol, .alpha.,.alpha.4-trimethyl- $$ p-Menth-1-en-8-ol $$ Terpine

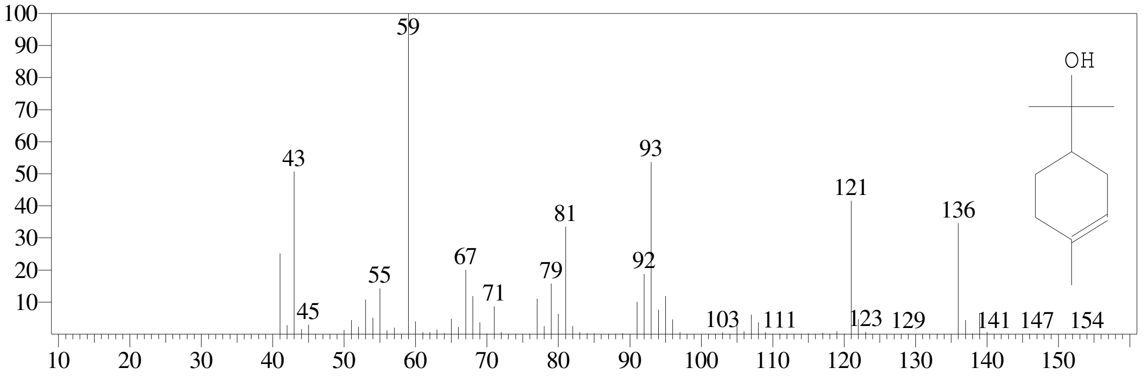

Hit#:4 Entry:12922 Library:NIST23s.lib
SI:84 Formula:C10H18O CAS:10482-56-1 MolWeight:154 RetIndex:1183
CompName:L-.alpha.-Terpineol $$ 3-Cyclohexene-1-methanol, .alpha.,.alpha.,4-trimethyl-, (S)- $$ p-Menth-1-en-8-ol, (S

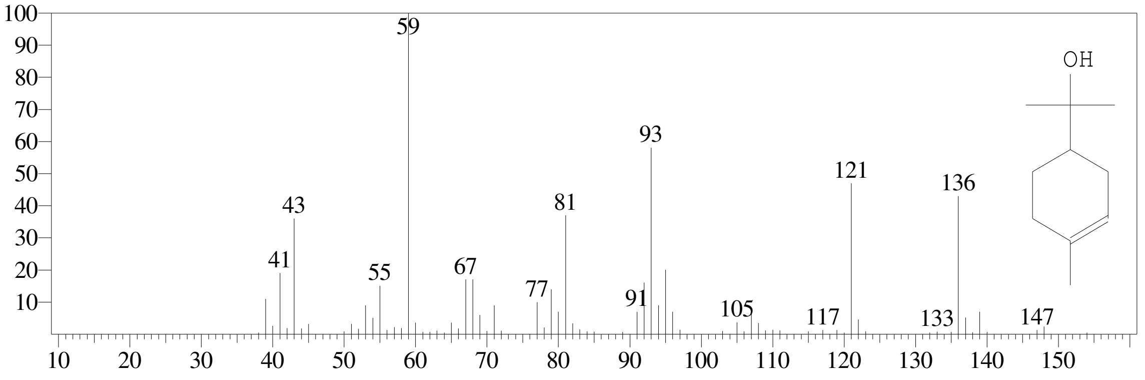

<< Target >>
Line#:11 R.Time:16.300(Scan#:1657) MassPeaks:15
RawMode:Averaged 16.292-16.308(1656-1658) BasePeak:59.00(7571)
BG Mode:None Group 1 - Event 1 Scan

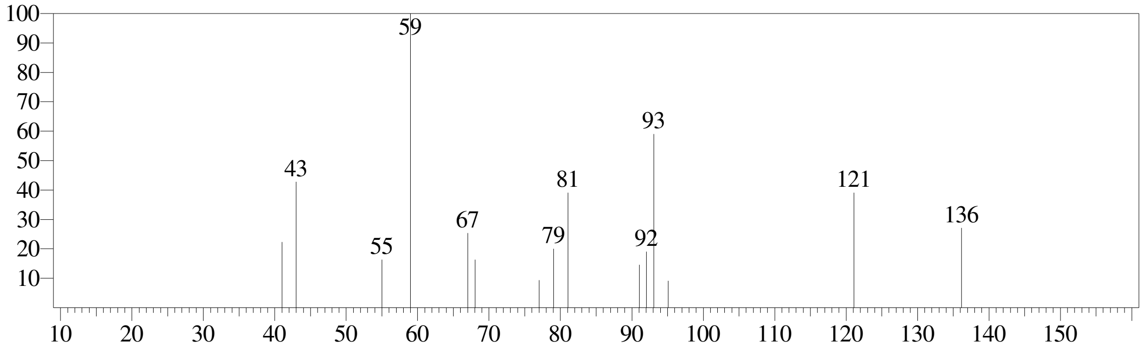

Hit#:5 Entry:12925 Library:NIST23s.lib
SI:84 Formula:C10H18O CAS:98-55-5 MolWeight:154 RetIndex:1183
CompName:.alpha.-Terpineol $$ 3-Cyclohexene-1-methanol, .alpha.,.alpha.4-trimethyl- $$ p-Menth-1-en-8-ol $$ Terpine

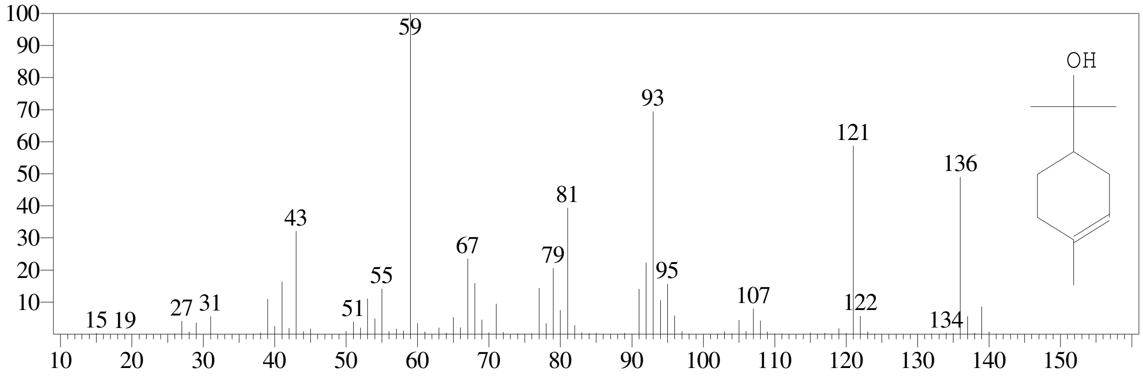

<< Target >>
Line#:12 R.Time:24.317(Scan#:2619) MassPeaks:21
RawMode:Averaged 24.308-24.325(2618-2620) BasePeak:105.10(9711)
BG Mode:None Group 1 - Event 1 Scan

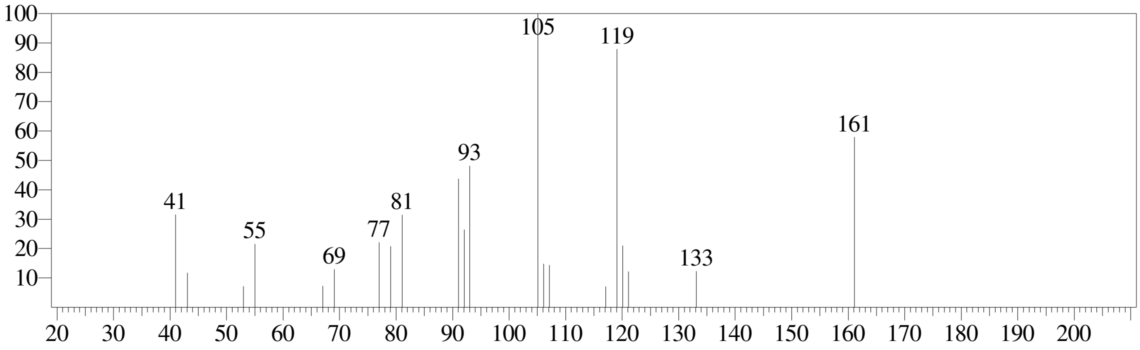

Hit#:1 Entry:24948 Library:NIST23s.lib
SI:87 Formula:C15H24 CAS:17699-14-8 MolWeight:204 RetIndex:1381
CompName:.alpha.-Cubebene $$ 1H-Cyclopenta[1,3]cyclopropa[1,2]benzene, 3a,3b,4,5,6,7-hexahydro-3,7-dimethyl-4-(1-

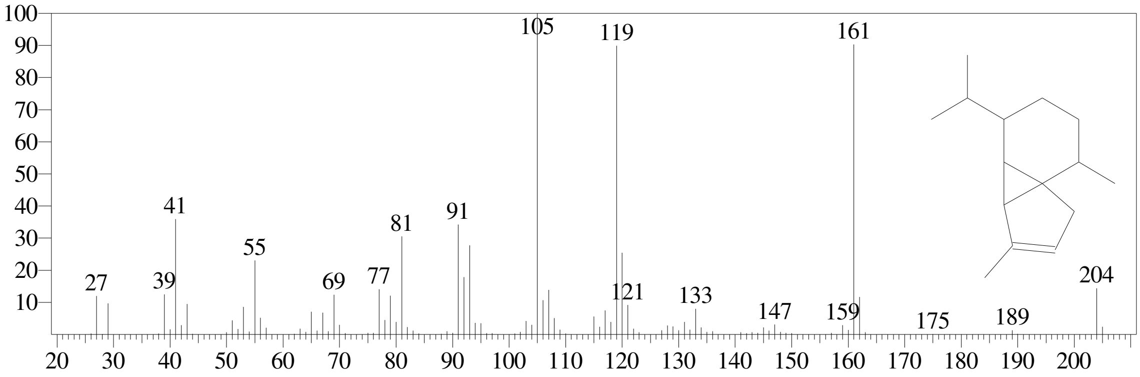

Hit#:2 Entry:62910 Library:NIST23-1.lib
SI:85 Formula:C15H24 CAS:3856-25-5 MolWeight:204 RetIndex:1407
CompName:Copaene $$ Tricyclo[4.4.0.02,7]dec-3-ene, 1,3-dimethyl-8-(1-methylethyl)-, stereoisomer $$ Tricyclo[4.4.0.0

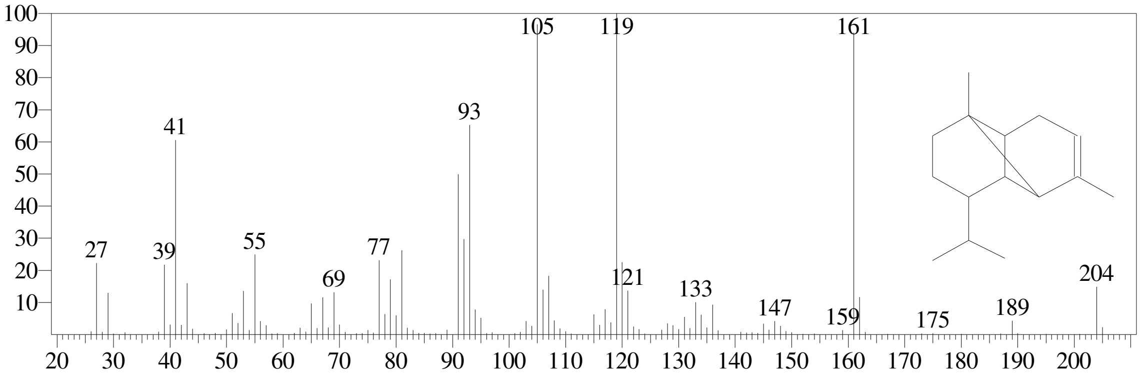

<< Target >>
Line#:12   R.Time:24.317(Scan#:2619) MassPeaks:21
RawMode:Averaged 24.308-24.325(2618-2620) BasePeak:105.10(9711)
BG Mode:None Group 1 - Event 1 Scan

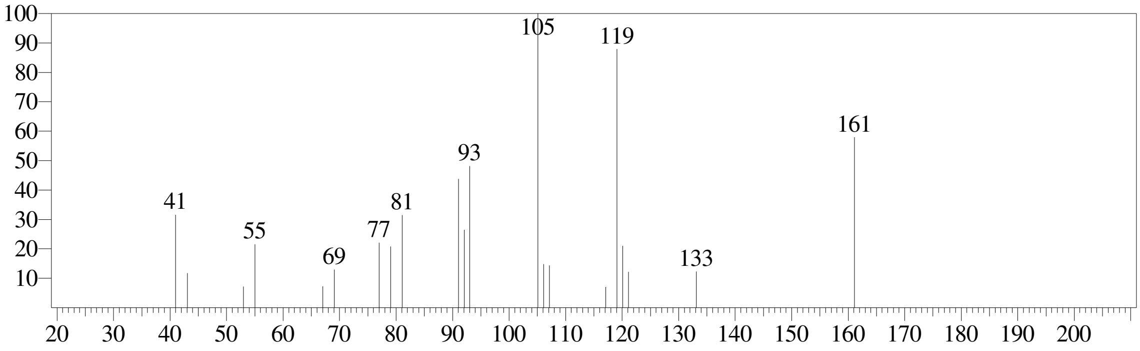

Hit#:3 Entry:25087 Library:NIST23s.lib
SI:85 Formula:C15H24 CAS:3856-25-5 MolWeight:204 RetIndex:1407
CompName:Copaene $$ Tricyclo[4.4.0.02,7]dec-3-ene, 1,3-dimethyl-8-(1-methylethyl)-, stereoisomer $$ Tricyclo[4.4.0.0

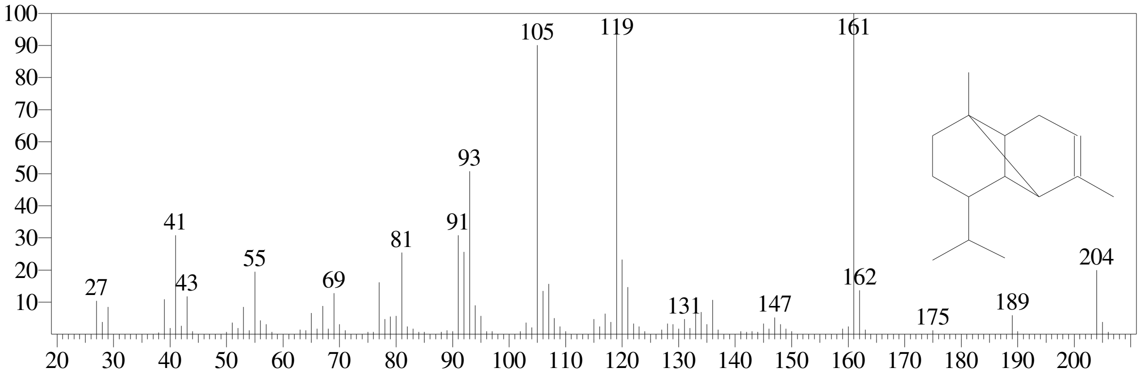

Hit#:4 Entry:25089 Library:NIST23s.lib
SI:85 Formula:C15H24 CAS:3856-25-5 MolWeight:204 RetIndex:1407
CompName:Copaene $$ Tricyclo[4.4.0.02,7]dec-3-ene, 1,3-dimethyl-8-(1-methylethyl)-, stereoisomer $$ Tricyclo[4.4.0.0

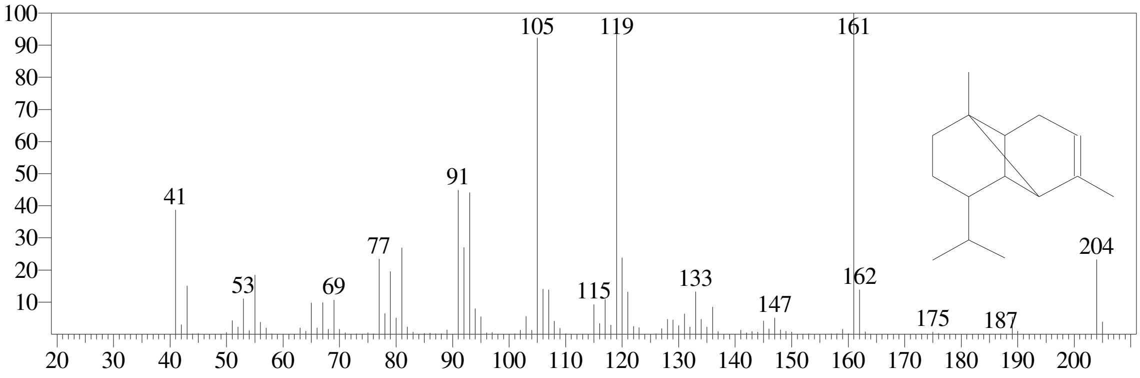

<< Target >>
Line#:12 R.Time:24.317(Scan#:2619) MassPeaks:21
RawMode:Averaged 24.308-24.325(2618-2620) BasePeak:105.10(9711)
BG Mode:None Group 1 - Event 1 Scan

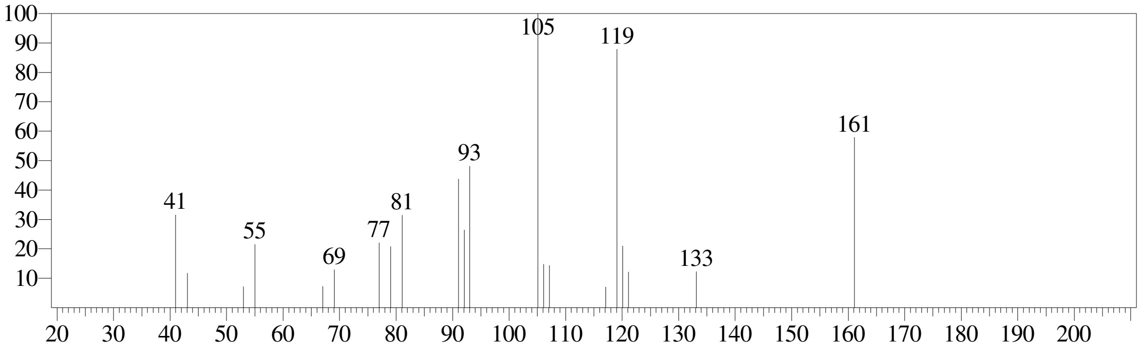

Hit#:5 Entry:25077 Library:NIST23s.lib
SI:85 Formula:C15H24 CAS:17699-14-8 MolWeight:204 RetIndex:1381
CompName:.alpha.-Cubebene $$ 1H-Cyclopenta[1,3]cyclopropa[1,2]benzene, 3a,3b,4,5,6,7-hexahydro-3,7-dimethyl-4-(1

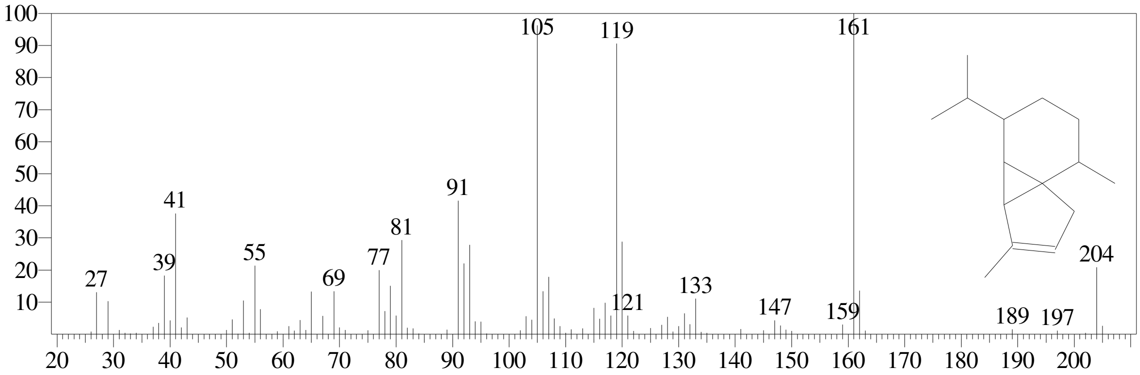

<< Target >>
Line#:13 R.Time:26.150(Scan#:2839) MassPeaks:56
RawMode:Averaged 26.142-26.158(2838-2840) BasePeak:41.00(30057)
BG Mode:None Group 1 - Event 1 Scan

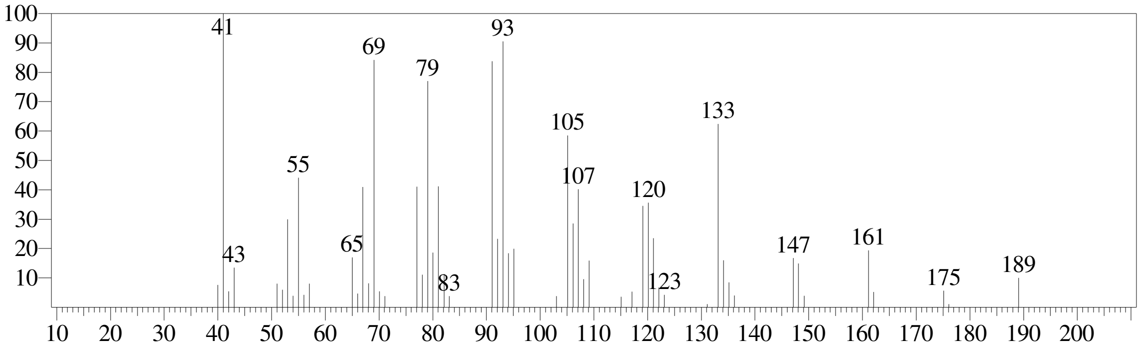

Hit#:1 Entry:24804 Library:NIST23s.lib
SI:95 Formula:C15H24 CAS:87-44-5 MolWeight:204 RetIndex:1448
CompName:Caryophyllene $$ Bicyclo[7.2.0]undec-4-ene, 4,11,11-trimethyl-8-methylene-, [1R-(1R*,4E,9S*)]- $$ Bicycl

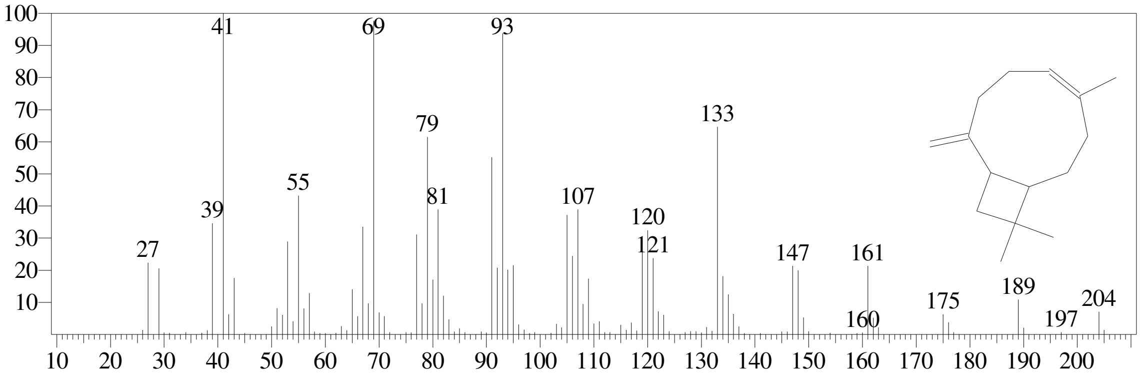

Hit#:2 Entry:24803 Library:NIST23s.lib
SI:95 Formula:C15H24 CAS:118-65-0 MolWeight:204 RetIndex:1448
CompName:Bicyclo[7.2.0]undec-4-ene, 4,11,11-trimethyl-8-methylene-,[1R-(1R*,4Z,9S*)]- $$ Isocaryophyllene $$ 4,11,

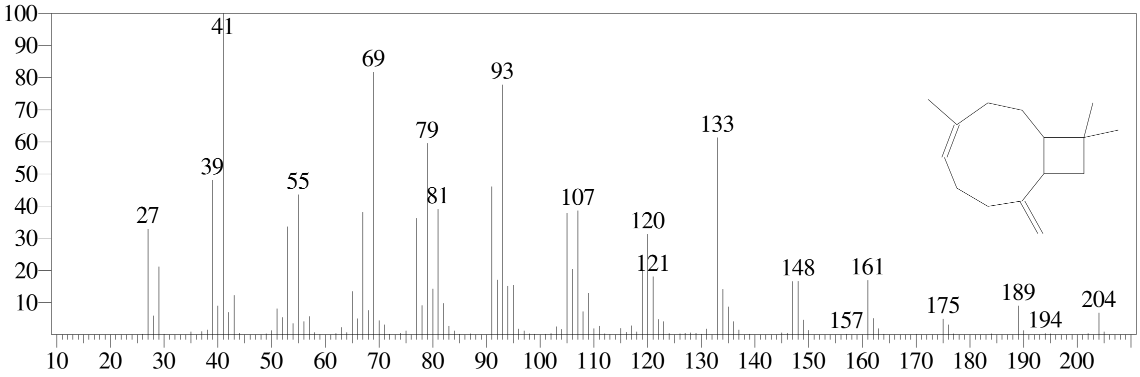

<< Target >>
Line#:13 R.Time:26.150(Scan#:2839) MassPeaks:56
RawMode:Averaged 26.142-26.158(2838-2840) BasePeak:41.00(30057)
BG Mode:None Group 1 - Event 1 Scan

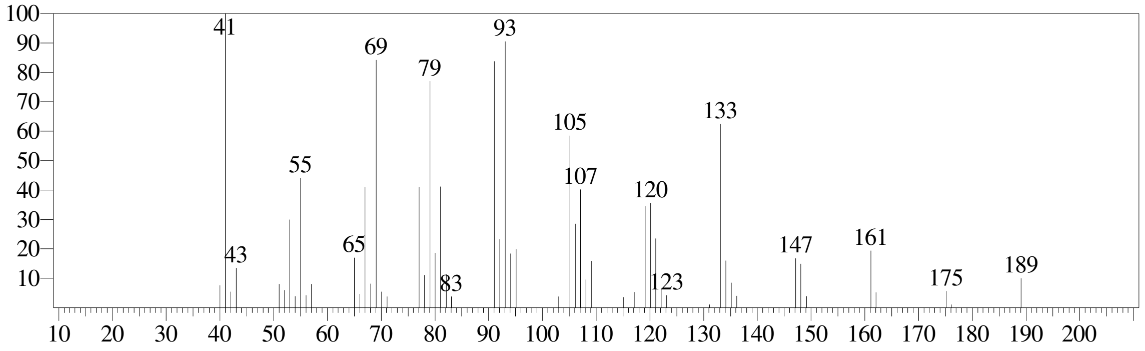

Hit#:3 Entry:24827 Library:NIST23s.lib
SI:95 Formula:C15H24 CAS:87-44-5 MolWeight:204 RetIndex:1448
CompName:Caryophyllene $$ Bicyclo[7.2.0]undec-4-ene, 4,11,11-trimethyl-8-methylene-, [1R-(1R*,4E,9S*)]- $$ Bicycl

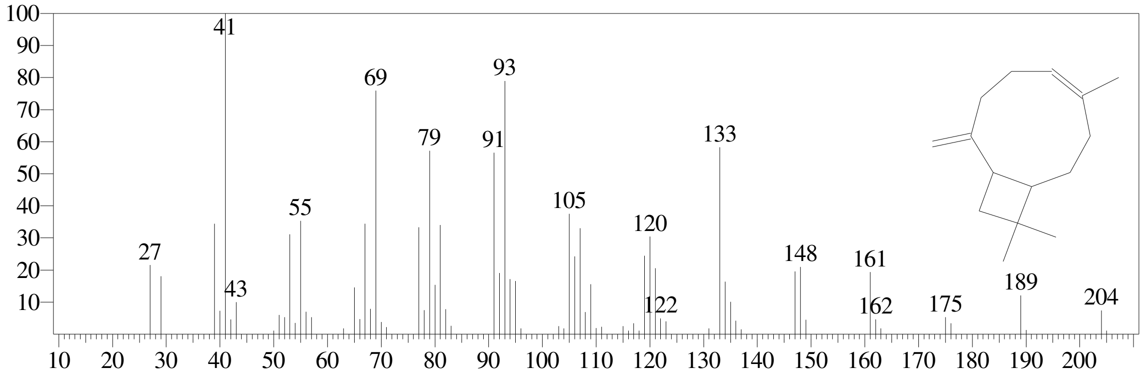

Hit#:4 Entry:62827 Library:NIST23-1.lib
SI:94 Formula:C15H24 CAS:87-44-5 MolWeight:204 RetIndex:1448
CompName:Caryophyllene $$ Bicyclo[7.2.0]undec-4-ene, 4,11,11-trimethyl-8-methylene-, [1R-(1R*,4E,9S*)]- $$ Bicycl

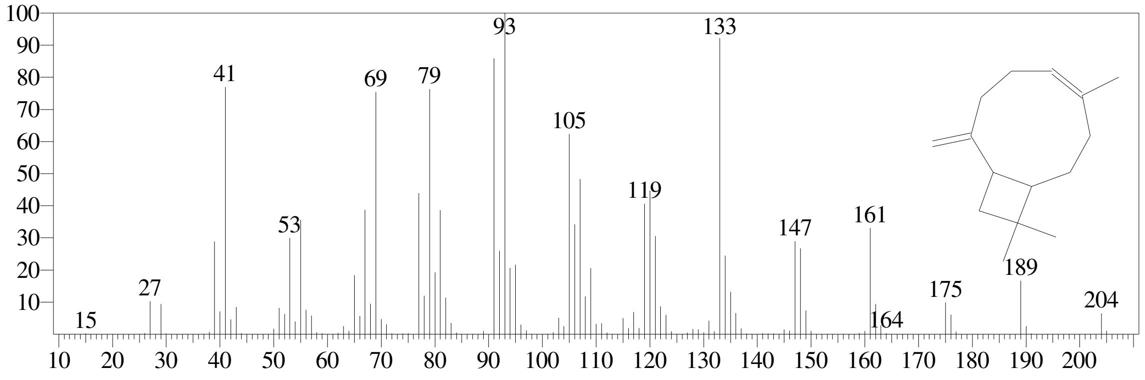

<< Target >>
Line#:13 R.Time:26.150(Scan#:2839) MassPeaks:56
RawMode:Averaged 26.142-26.158(2838-2840) BasePeak:41.00(30057)
BG Mode:None Group 1 - Event 1 Scan

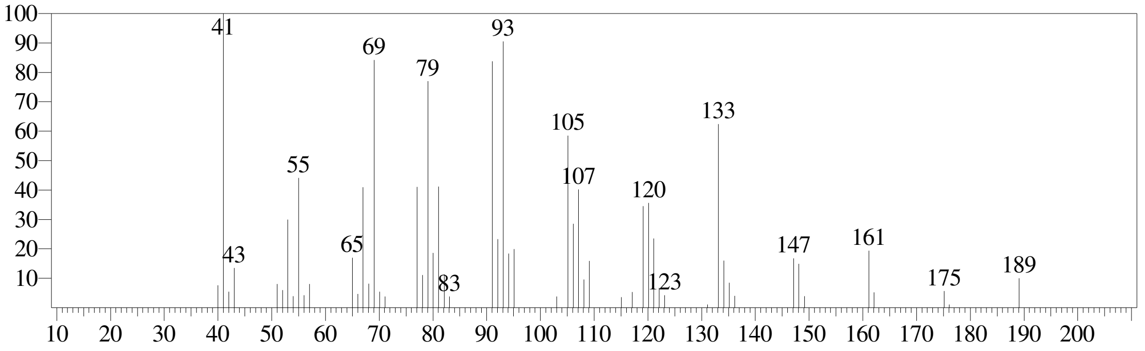

Hit#:5 Entry:24884 Library:NIST23s.lib
SI:93 Formula:C15H24 CAS:13877-93-5 MolWeight:204 RetIndex:1448
CompName:Bicyclo[7.2.0]undec-4-ene, 4,11,11-trimethyl-8-methylene- $$ Bicyclo[7.2.0]undec-4-ene, 4,11,11-trimethyl-

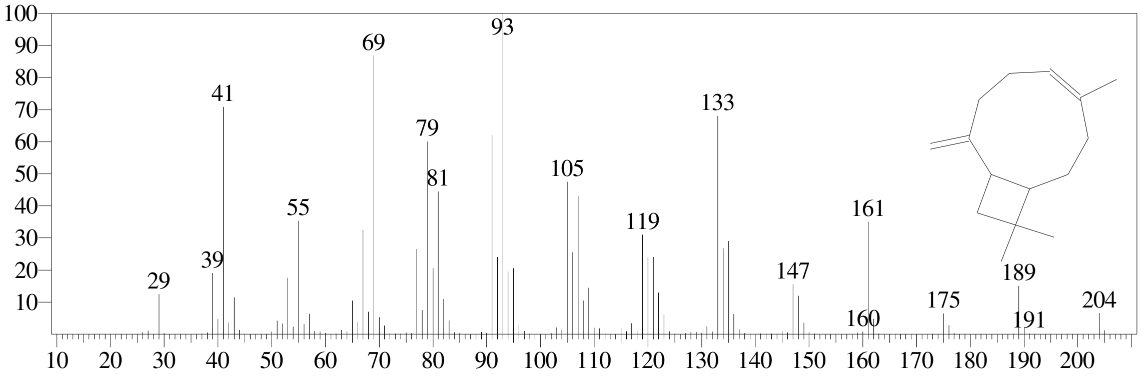

<< Target >>
Line#:14 R.Time:26.942(Scan#:2934) MassPeaks:13
RawMode:Averaged 26.933-26.950(2933-2935) BasePeak:91.05(2076)
BG Mode:None Group 1 - Event 1 Scan

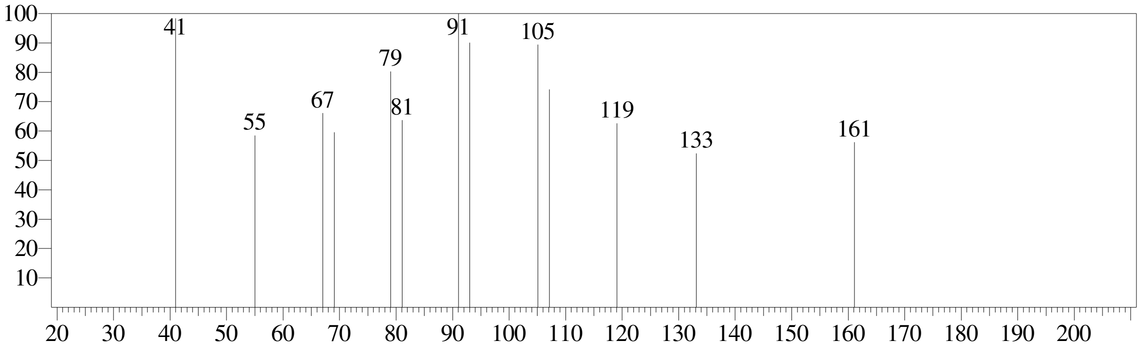

Hit#:1 Entry:24823 Library:NIST23s.lib
SI:76 Formula:C15H24 CAS:26560-14-5 MolWeight:204 RetIndex:1509
CompName:1,3,6,10-Dodecatetraene, 3,7,11-trimethyl-, (Z,E)- $$ (Z,E)-.alpha.-Farnesene $$ (3Z,6E)-3,7,11-Trimethyl-1.

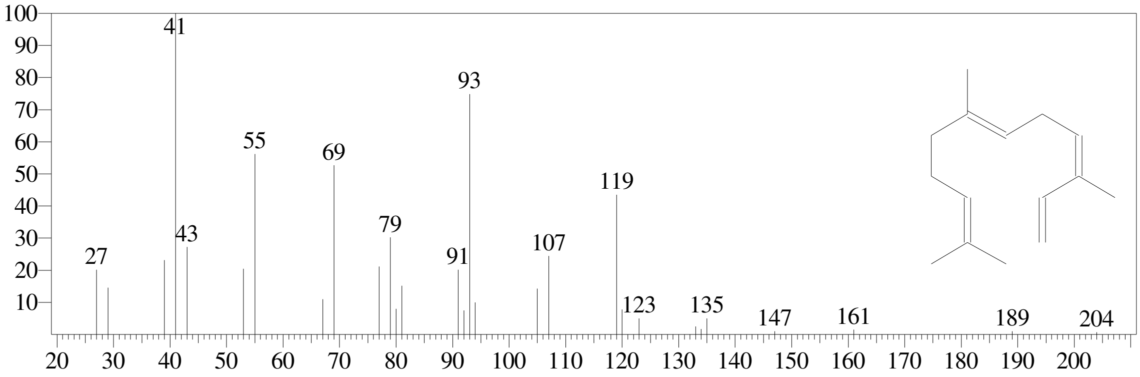

Hit#:2 Entry:62757 Library:NIST23-1.lib
SI:73 Formula:C15H24 CAS:28973-99-1 MolWeight:204 RetIndex:1509
CompName:(Z,Z)-.alpha.-Farnesene $$ (3Z,6Z)-3,7,11-Trimethyl-1,3,6,10-dodecatetraene # $$

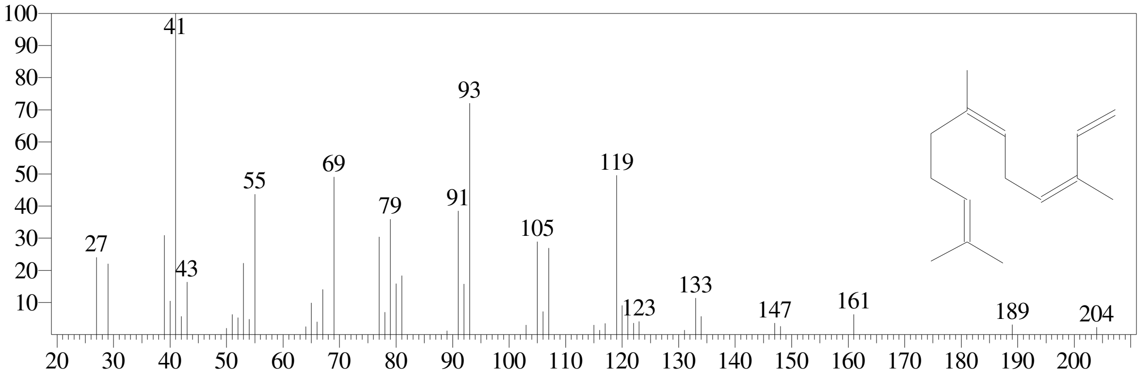

<< Target >>
Line#:14 R.Time:26.942(Scan#:2934) MassPeaks:13
RawMode:Averaged 26.933-26.950(2933-2935) BasePeak:91.05(2076)
BG Mode:None Group 1 - Event 1 Scan

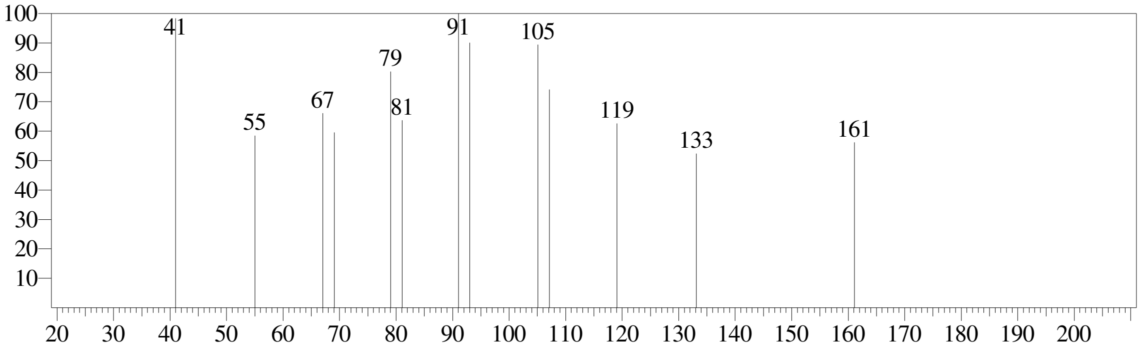

Hit#:3 Entry:62756 Library:NIST23-1.lib
SI:73 Formula:C15H24 CAS:26560-14-5 MolWeight:204 RetIndex:1509
CompName:1,3,6,10-Dodecatetraene, 3,7,11-trimethyl-, (Z,E)- $$ (Z,E)-.alpha.-Farnesene $$ (3Z,6E)-3,7,11-Trimethyl-1.

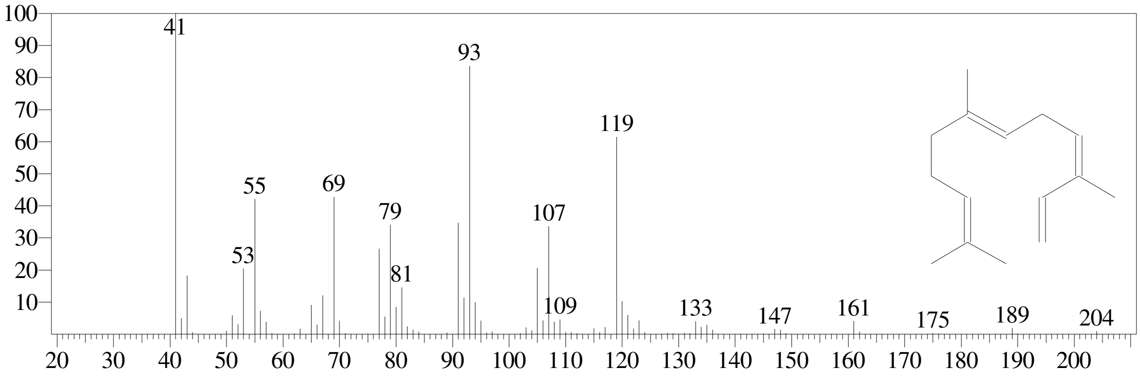

Hit#:4 Entry:24831 Library:NIST23s.lib
SI:73 Formula:C15H24 CAS:26560-14-5 MolWeight:204 RetIndex:1509
CompName:1,3,6,10-Dodecatetraene, 3,7,11-trimethyl-, (Z,E)- $$ (Z,E)-.alpha.-Farnesene $$ (3Z,6E)-3,7,11-Trimethyl-1.

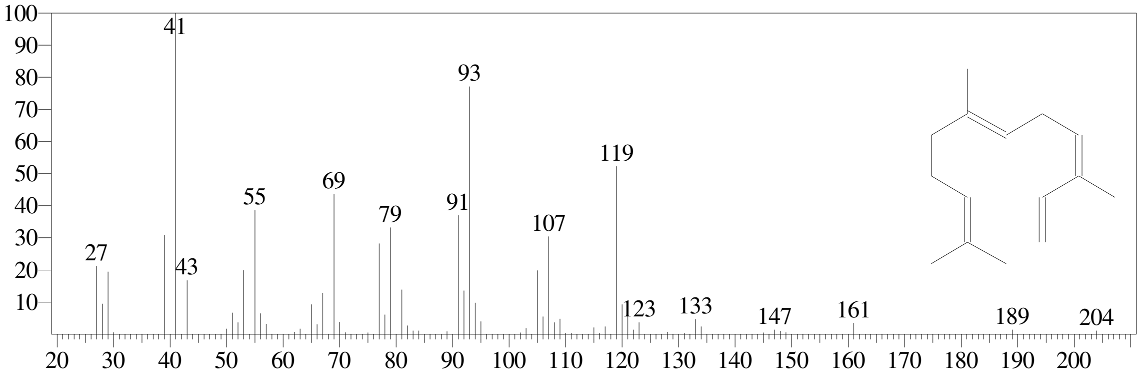

<< Target >>
Line#:14   R.Time:26.942(Scan#:2934) MassPeaks:13
RawMode:Averaged 26.933-26.950(2933-2935) BasePeak:91.05(2076)
BG Mode:None Group 1 - Event 1 Scan

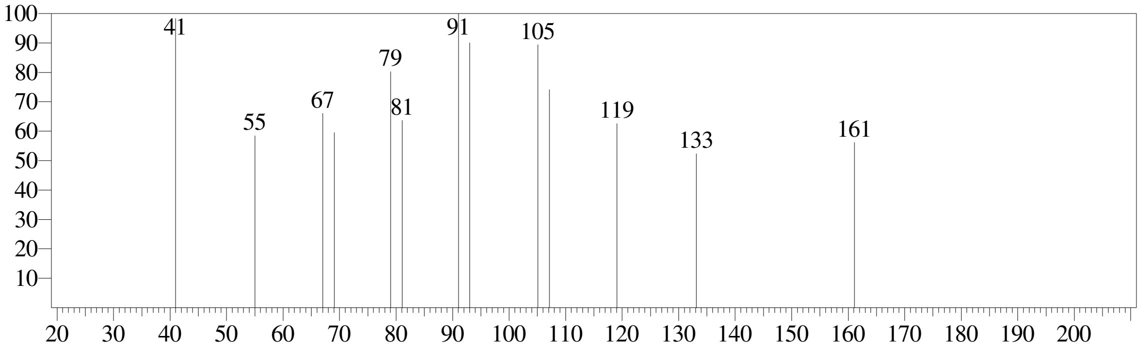

Hit#:5 Entry:24824 Library:NIST23s.lib
SI:73 Formula:C15H24 CAS:502-61-4 MolWeight:204 RetIndex:1509
CompName:.alpha.-Farnesene $$ 1,3,6,10-Dodecatetraene, 3,7,11-trimethyl-, (E,E)- $$ Farnesene $$ 2,6,10-Trimethyl-2,6

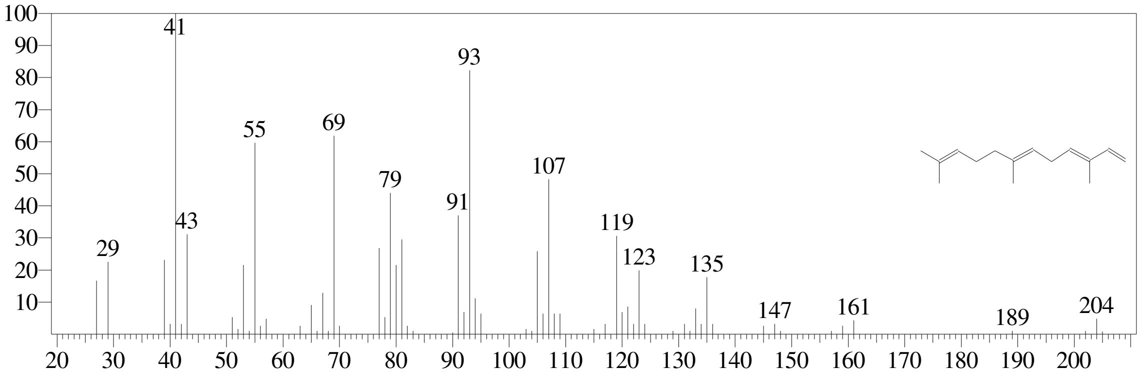

<< Target >>
Line#:15 R.Time:27.542(Scan#:3006) MassPeaks:19
RawMode:Averaged 27.533-27.550(3005-3007) BasePeak:93.05(11840)
BG Mode:Calc. from Peak Group 1 - Event 1 Scan

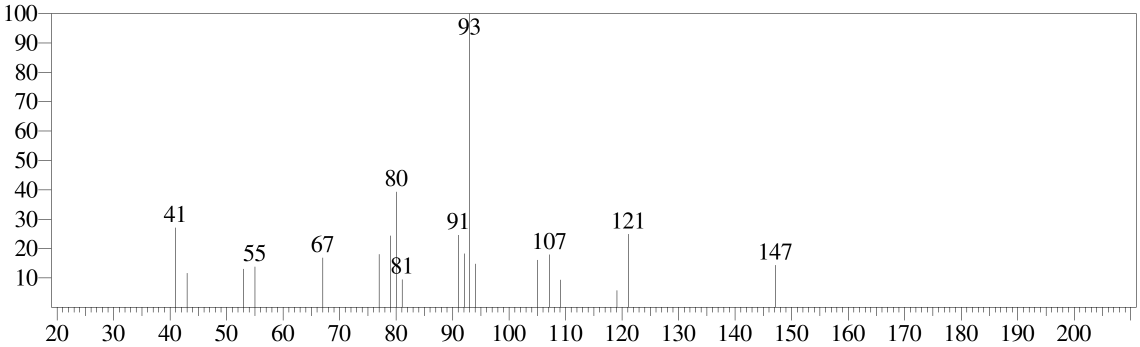

Hit#:1 Entry:24886 Library:NIST23s.lib
SI:87 Formula:C15H24 CAS:6753-98-6 MolWeight:204 RetIndex:1455
CompName:Humulene $$ .alpha.-Caryophyllene $$ 1,4,8-Cycloundecatriene, 2,6,6,9-tetramethyl-, (E,E,E)- $$ .alpha.-Hu

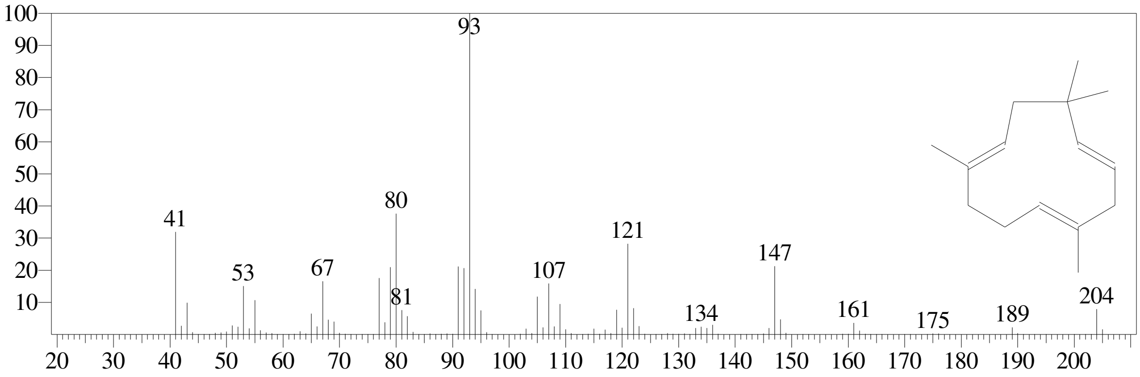

Hit#:2 Entry:62814 Library:NIST23-1.lib
SI:86 Formula:C15H24 CAS:6753-98-6 MolWeight:204 RetIndex:1455
CompName:Humulene $$ .alpha.-Caryophyllene $$ 1,4,8-Cycloundecatriene, 2,6,6,9-tetramethyl-, (E,E,E)- $$ .alpha.-Hu

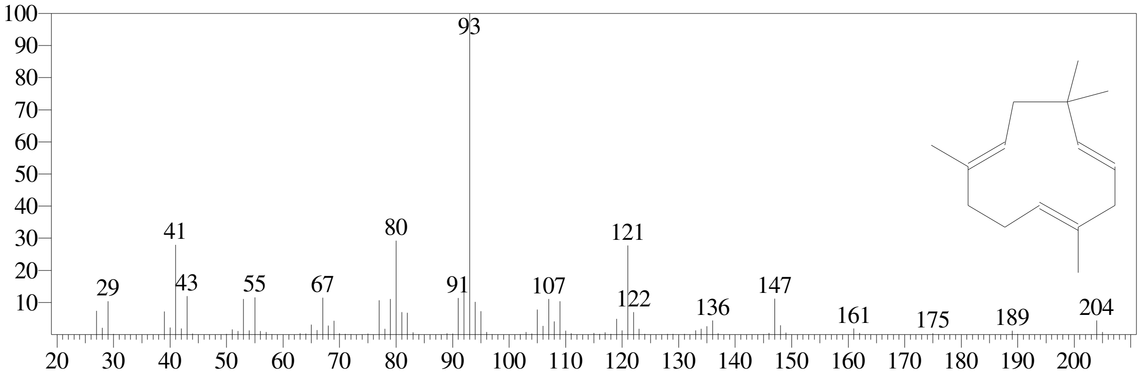

<< Target >>
Line#:15 R.Time:27.542(Scan#:3006) MassPeaks:19
RawMode:Averaged 27.533-27.550(3005-3007) BasePeak:93.05(11840)
BG Mode:Calc. from Peak Group 1 - Event 1 Scan

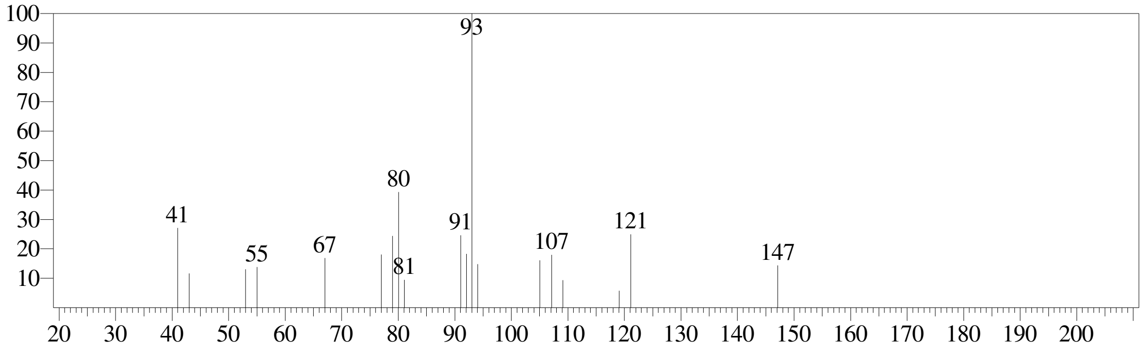

Hit#:3 Entry:8420 Library:NIST23s.lib
SI:86 Formula:C10H16 CAS:13877-91-3 MolWeight:136 RetIndex:1047
CompName:.beta.-Ocimene $$ 1,3,6-Octatriene, 3,7-dimethyl- $$ Ocimene $$ 3,7-Dimethyl-1,3,6-octatriene $$ beta-Ocir

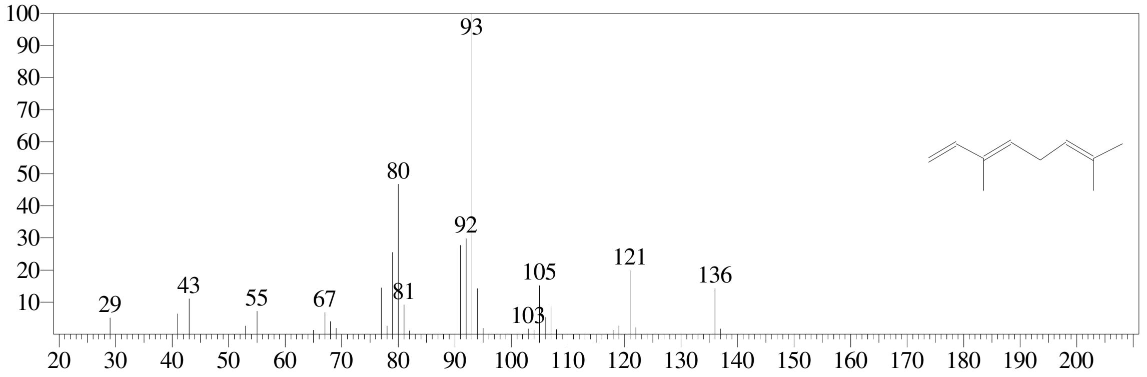

Hit#:4 Entry:24887 Library:NIST23s.lib
SI:86 Formula:C15H24 CAS:6753-98-6 MolWeight:204 RetIndex:1455
CompName:Humulene $$ .alpha.-Caryophyllene $$ 1,4,8-Cycloundecatriene, 2,6,6,9-tetramethyl-, (E,E,E)- $$ .alpha.-Hu

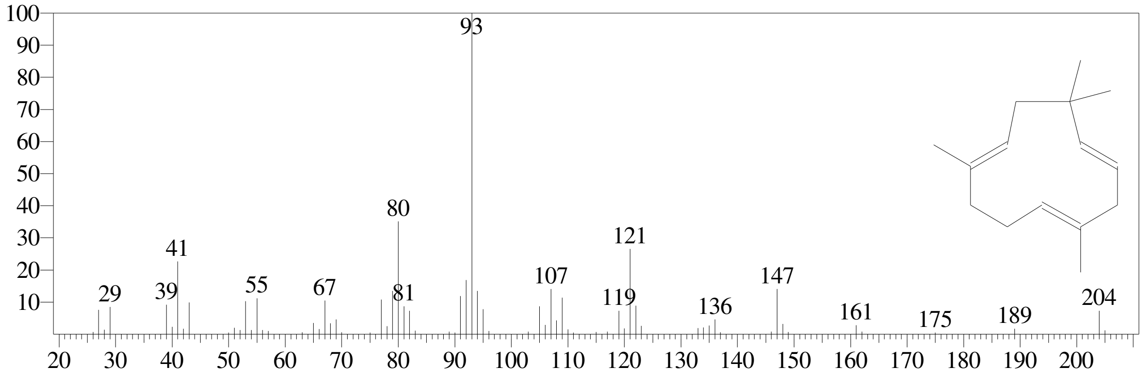

<< Target >>
Line#:15   R.Time:27.542(Scan#:3006) MassPeaks:19
RawMode:Averaged 27.533-27.550(3005-3007) BasePeak:93.05(11840)
BG Mode:Calc. from Peak Group 1 - Event 1 Scan

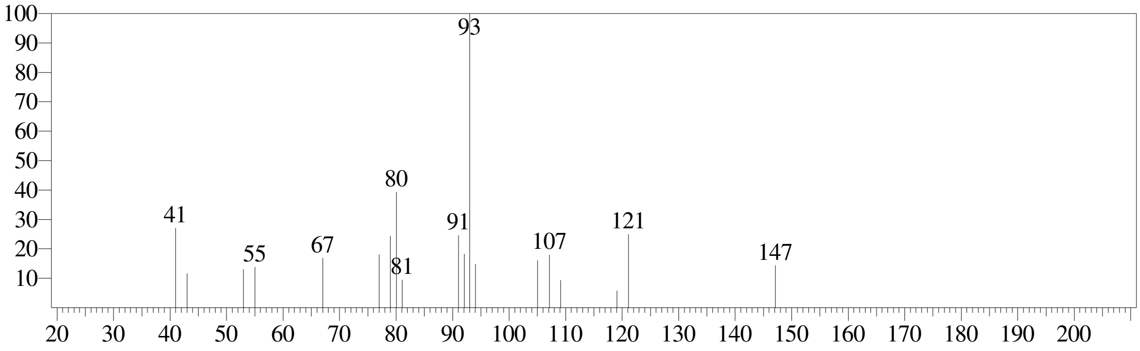

Hit#:5 Entry:24877 Library:NIST23s.lib
SI:86 Formula:C15H24 CAS:6753-98-6 MolWeight:204 RetIndex:1455
CompName:Humulene $$ .alpha.-Caryophyllene $$ 1,4,8-Cycloundecatriene, 2,6,6,9-tetramethyl-, (E,E,E)- $$ .alpha.-Hu

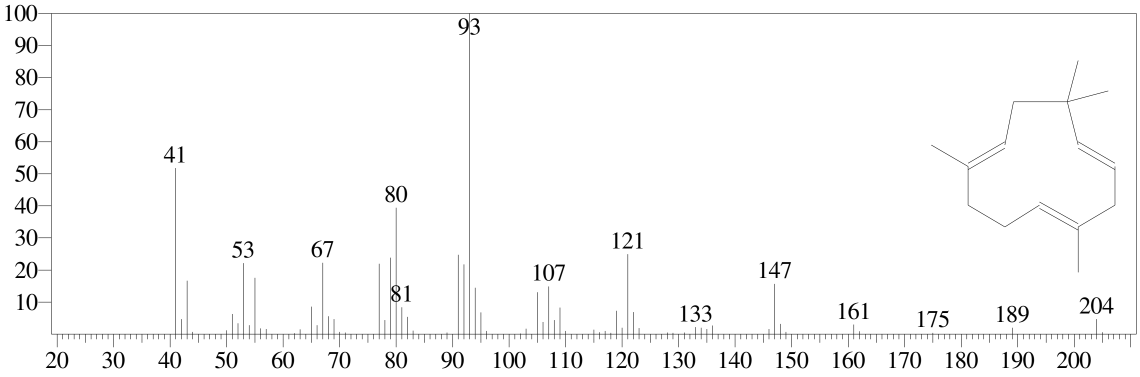

<< Target >>
Line#:16 R.Time:27.850(Scan#:3043) MassPeaks:14
RawMode:Averaged 27.842-27.858(3042-3044) BasePeak:91.05(2320)
BG Mode:None Group 1 - Event 1 Scan

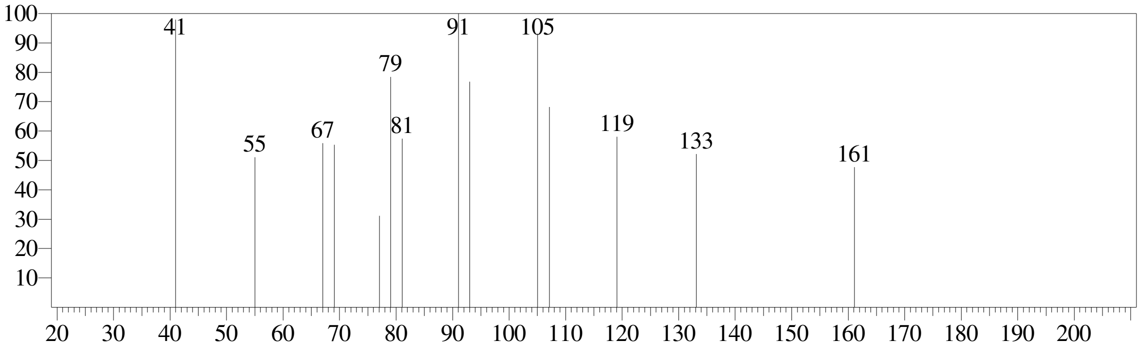

Hit#:1 Entry:24823 Library:NIST23s.lib
SI:78 Formula:C15H24 CAS:26560-14-5 MolWeight:204 RetIndex:1509
CompName:1,3,6,10-Dodecatetraene, 3,7,11-trimethyl-, (Z,E)- $$ (Z,E)-.alpha.-Farnesene $$ (3Z,6E)-3,7,11-Trimethyl-1,

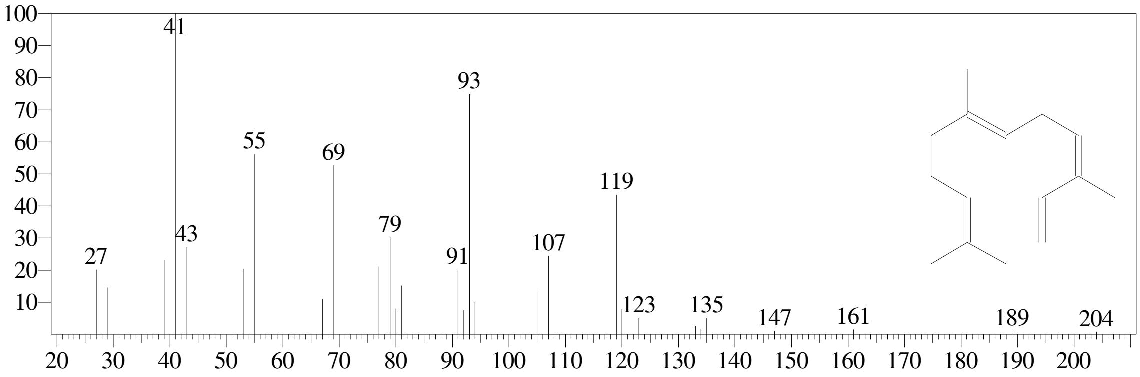

Hit#:2 Entry:62757 Library:NIST23-1.lib
SI:76 Formula:C15H24 CAS:28973-99-1 MolWeight:204 RetIndex:1509
CompName:(Z,Z)-.alpha.-Farnesene $$ (3Z,6Z)-3,7,11-Trimethyl-1,3,6,10-dodecatetraene # $$

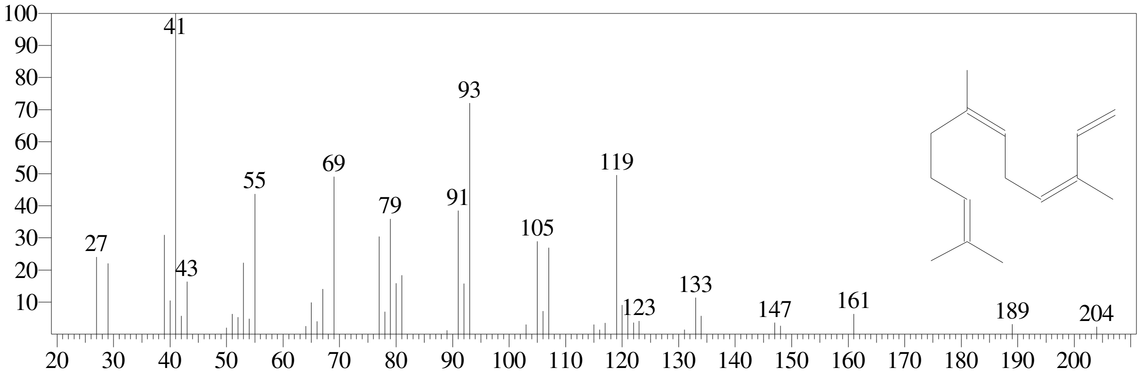

<< Target >>
Line#:16   R.Time:27.850(Scan#:3043) MassPeaks:14
RawMode:Averaged 27.842-27.858(3042-3044) BasePeak:91.05(2320)
BG Mode:None Group 1 - Event 1 Scan

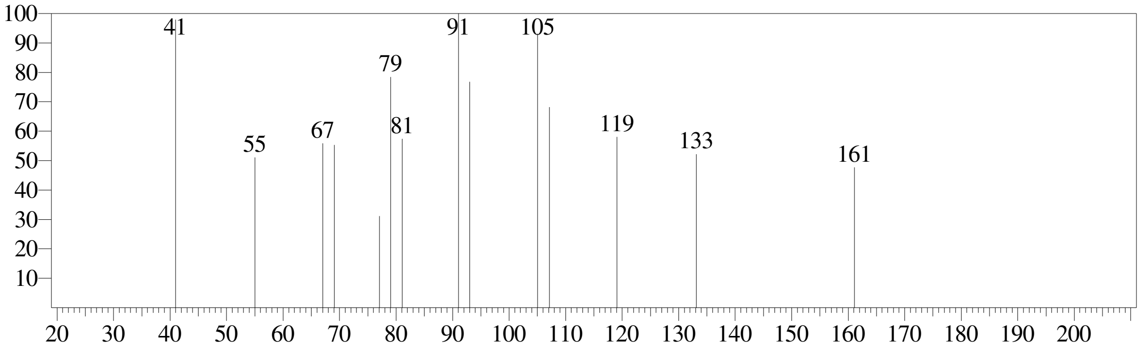

Hit#:3 Entry:24831 Library:NIST23s.lib
SI:76 Formula:C15H24 CAS:26560-14-5 MolWeight:204 RetIndex:1509
CompName:1,3,6,10-Dodecatetraene, 3,7,11-trimethyl-, (Z,E)- $$ (Z,E)-.alpha.-Farnesene $$ (3Z,6E)-3,7,11-Trimethyl-1.

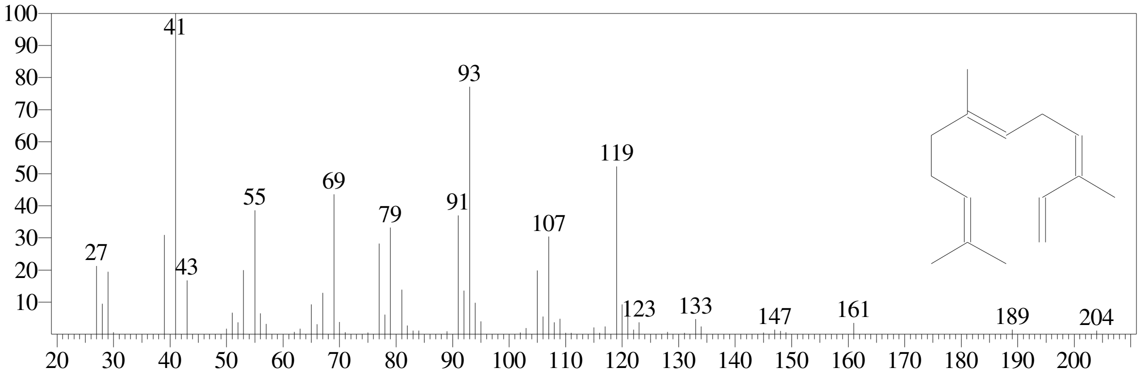

Hit#:4 Entry:62756 Library:NIST23-1.lib
SI:75 Formula:C15H24 CAS:26560-14-5 MolWeight:204 RetIndex:1509
CompName:1,3,6,10-Dodecatetraene, 3,7,11-trimethyl-, (Z,E)- $$ (Z,E)-.alpha.-Farnesene $$ (3Z,6E)-3,7,11-Trimethyl-1.

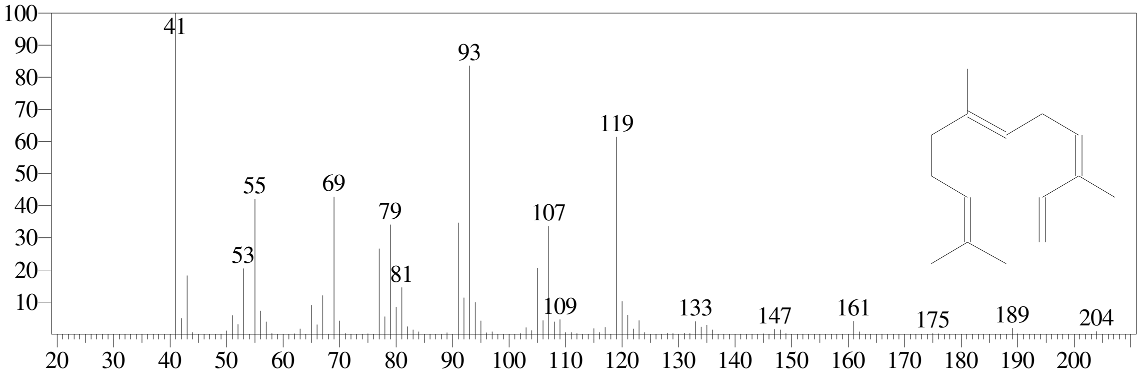

<< Target >>
Line#:16 R.Time:27.850(Scan#:3043) MassPeaks:14
RawMode:Averaged 27.842-27.858(3042-3044) BasePeak:91.05(2320)
BG Mode:None Group 1 - Event 1 Scan

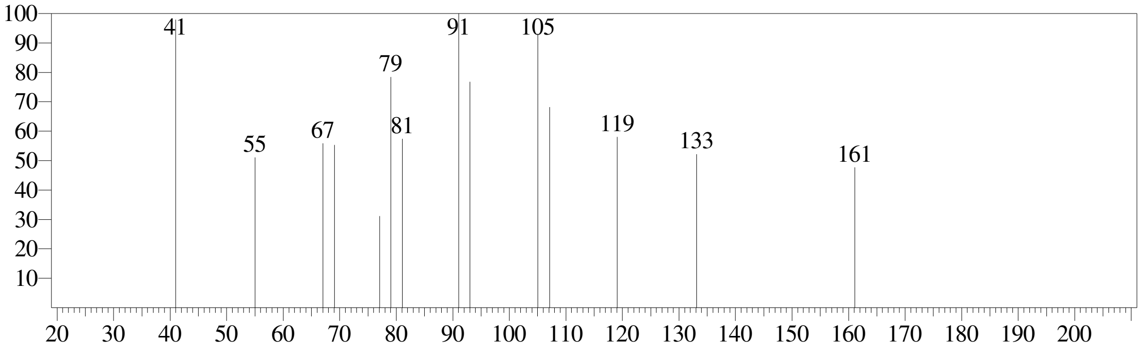

Hit#:5 Entry:25046 Library:NIST23s.lib
SI:75 Formula:C15H24 CAS:23986-74-5 MolWeight:204 RetIndex:1478
CompName:Germacrene D $$ (S,1Z,6Z)-8-Isopropyl-1-methyl-5-methylenecyclodeca-1,6-diene $$ D-Germacrene $$ 1(1

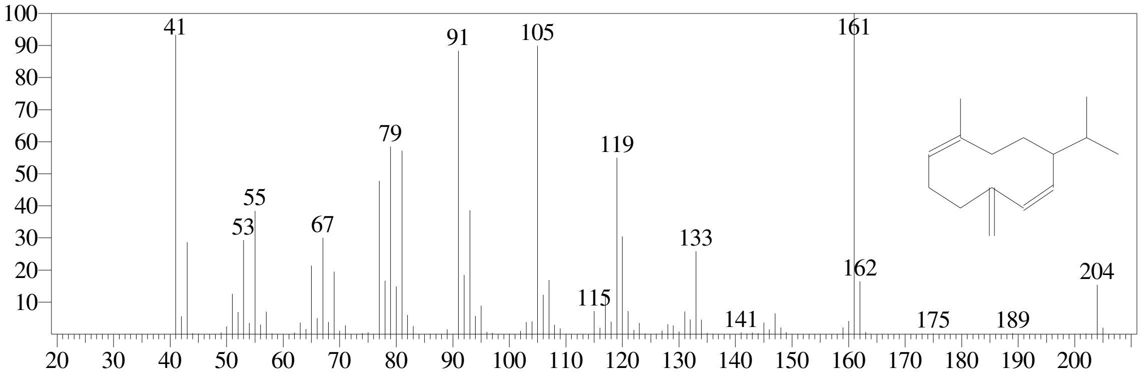

<< Target >>
Line#:17 R.Time:28.492(Scan#:3120) MassPeaks:10
RawMode:Averaged 28.483-28.500(3119-3121) BasePeak:105.05(2980)
BG Mode:None Group 1 - Event 1 Scan

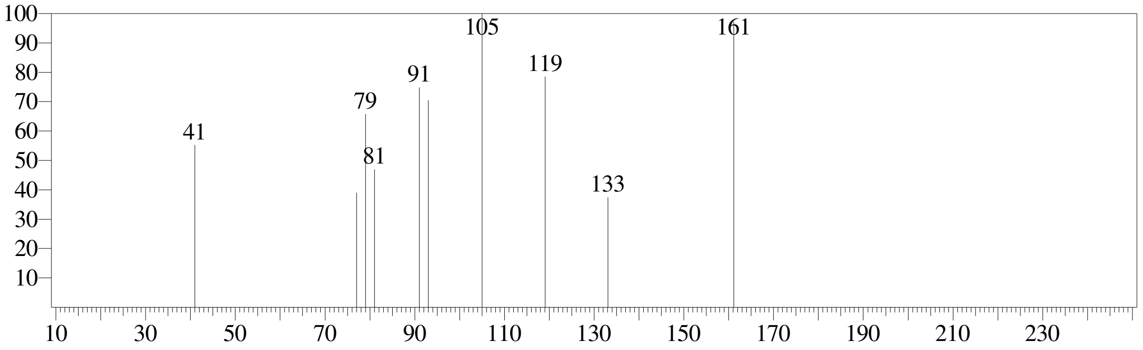

Hit#:1 Entry:31431 Library:NIST23s.lib
SI:74 Formula:C12H17Br CAS:57040-44-5 MolWeight:240 RetIndex:1581
CompName:Adamantane, 1-(2-bromoethenyl)- $$ 1-(2-Bromovinyl)-adamantane $$ 1-[(E)-2-Bromoethenyl]adamantane

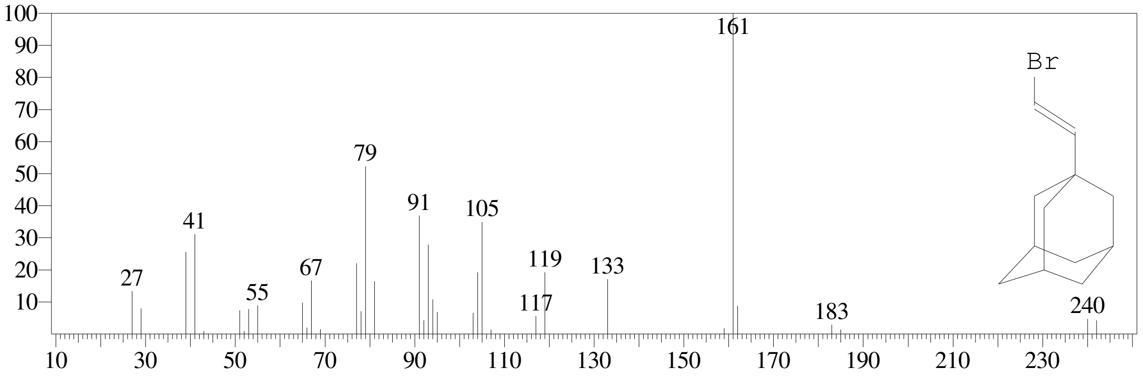

Hit#:2 Entry:25084 Library:NIST23s.lib
SI:74 Formula:C15H24 CAS:157374-44-2 MolWeight:204 RetIndex:1449
CompName:cis-muurola-3,5-diene

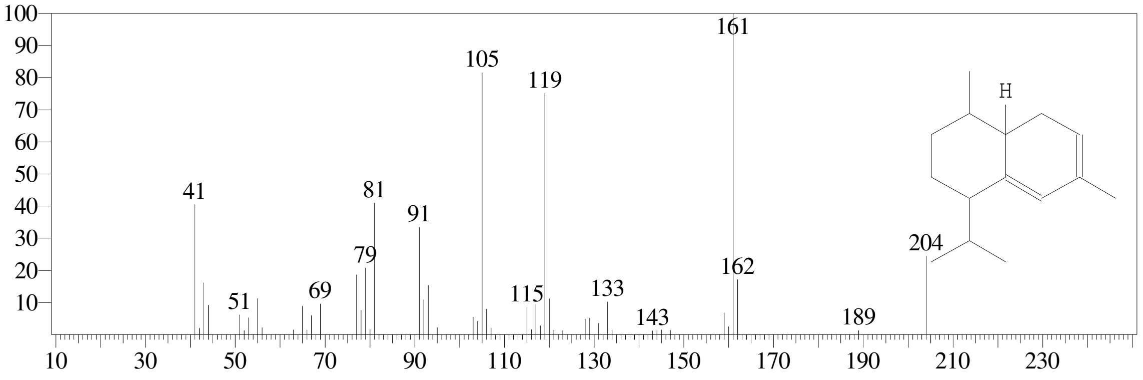

<< Target >>
Line#:17   R.Time:28.492(Scan#:3120) MassPeaks:10
RawMode:Averaged 28.483-28.500(3119-3121) BasePeak:105.05(2980)
BG Mode:None Group 1 - Event 1 Scan

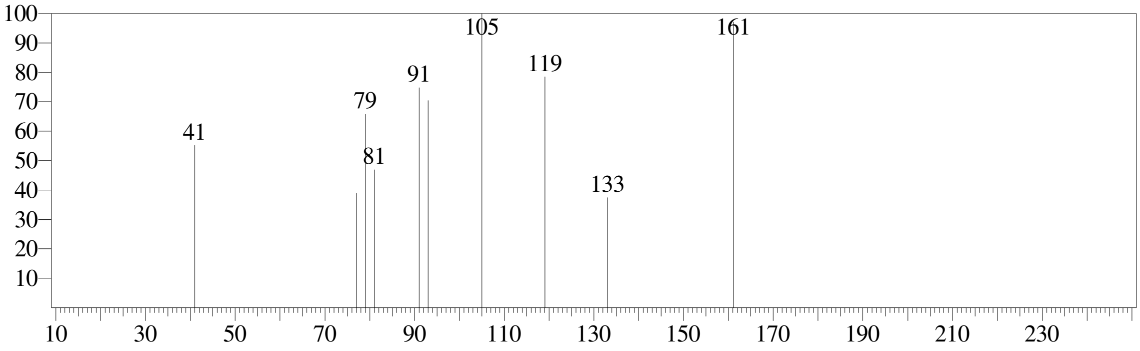

Hit#:3 Entry:25079 Library:NIST23s.lib
SI:74 Formula:C15H24 CAS:157477-72-0 MolWeight:204 RetIndex:1487
CompName:cis-Muurola-4(15),5-diene $$ (1S,4S,4aR)-1-Isopropyl-4-methyl-7-methylene-1,2,3,4,4a,5,6,7-octahydronapł

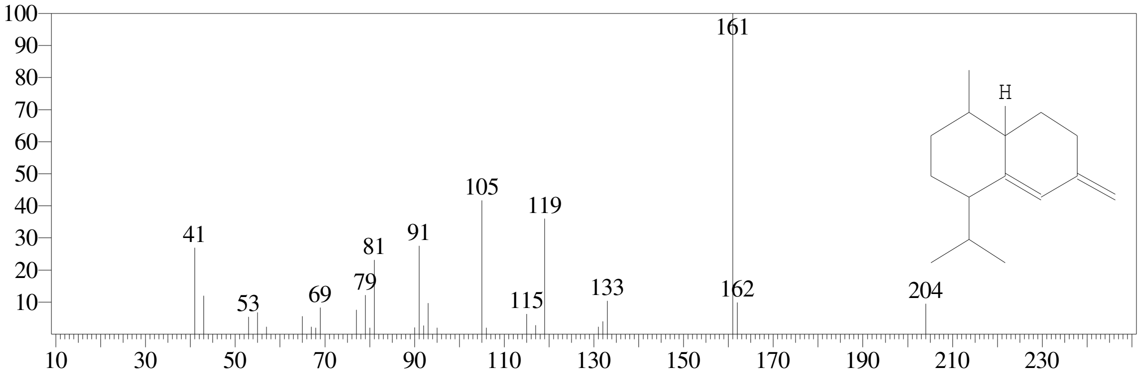

Hit#:4 Entry:25067 Library:NIST23s.lib
SI:74 Formula:C15H24 CAS:23986-74-5 MolWeight:204 RetIndex:1478
CompName:Germacrene D $$ (S,1Z,6Z)-8-Isopropyl-1-methyl-5-methylenecyclodeca-1,6-diene $$ D-Germacrene $$ 1(1

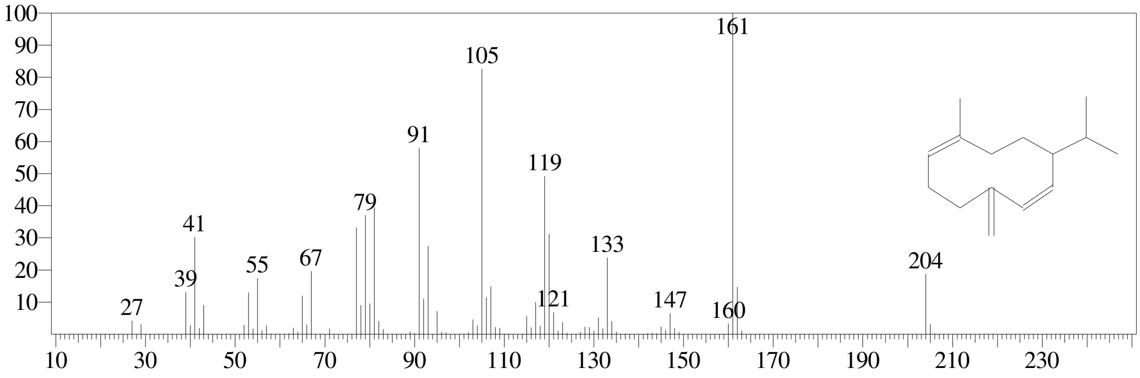

<< Target >>
Line#:17 R.Time:28.492(Scan#:3120) MassPeaks:10
RawMode:Averaged 28.483-28.500(3119-3121) BasePeak:105.05(2980)
BG Mode:None Group 1 - Event 1 Scan

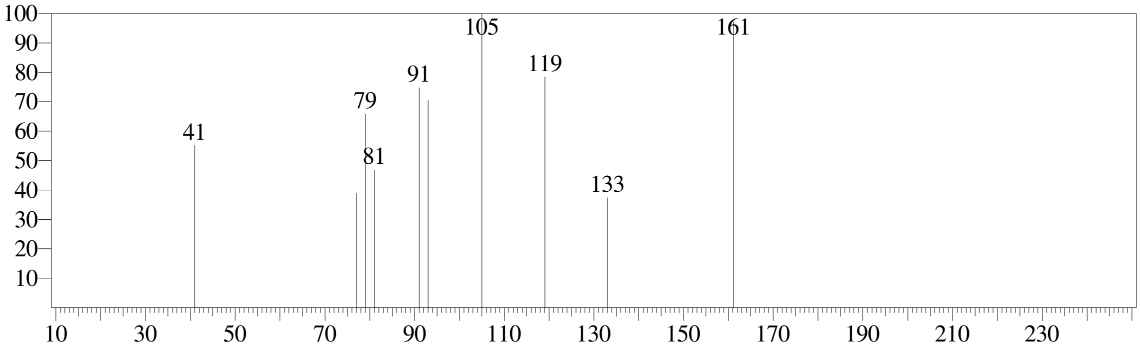

Hit#:5 Entry:62981 Library:NIST23-1.lib
SI:73 Formula:C15H24 CAS:23986-74-5 MolWeight:204 RetIndex:1478
CompName:Germacrene D $$ (S,1Z,6Z)-8-Isopropyl-1-methyl-5-methylenecyclodeca-1,6-diene $$ D-Germacrene $$ 1(1

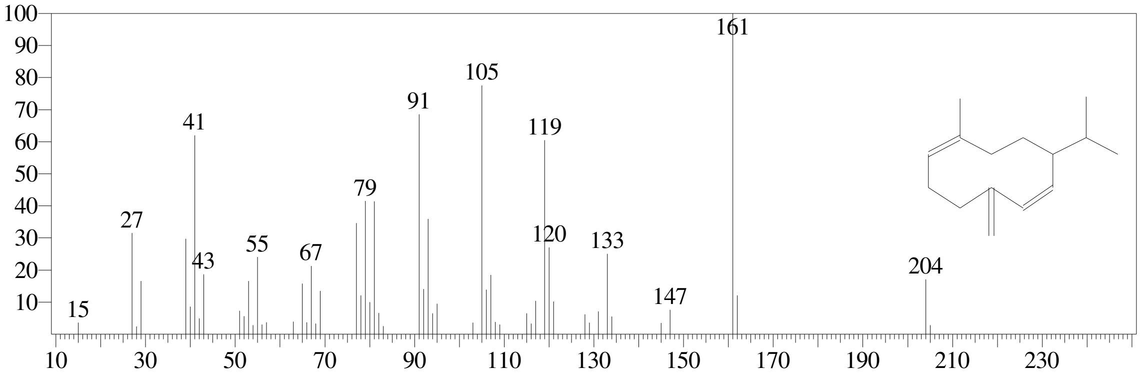

<< Target >>
Line#:18   R.Time:29.242(Scan#:3210) MassPeaks:24
RawMode:Averaged 29.233-29.250(3209-3211) BasePeak:105.05(4941)
BG Mode:None Group 1 - Event 1 Scan

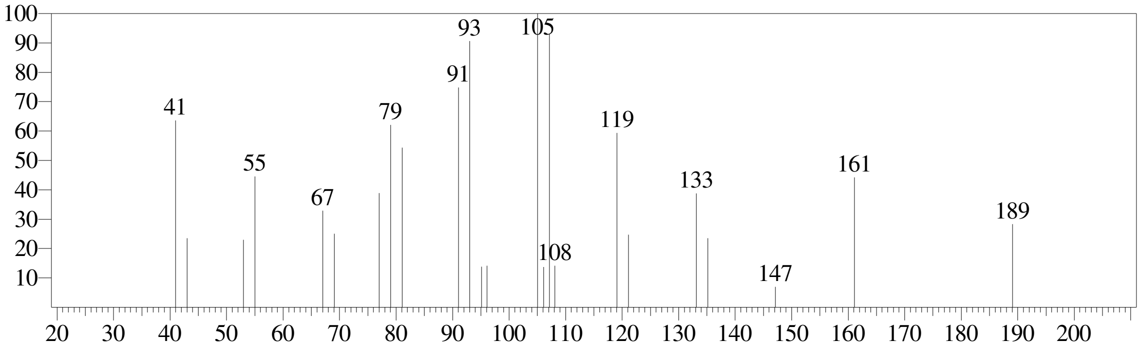

Hit#:1 Entry:24894 Library:NIST23s.lib
SI:85 Formula:C15H24 CAS:3691-11-0 MolWeight:204 RetIndex:1498
CompName:Azulene, 1,2,3,5,6,7,8,8a-octahydro-1,4-dimethyl-7-(1-methylethenyl)-, [1S-(1.alpha.,7.alpha.,8a.beta.)]- $$ (

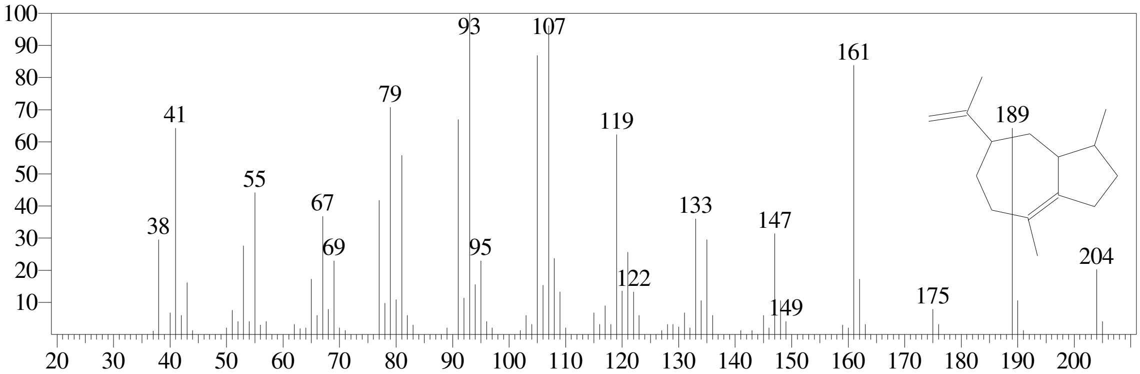

Hit#:2 Entry:24958 Library:NIST23s.lib
SI:83 Formula:C15H24 CAS:3691-11-0 MolWeight:204 RetIndex:1498
CompName:Azulene, 1,2,3,5,6,7,8,8a-octahydro-1,4-dimethyl-7-(1-methylethenyl)-, [1S-(1.alpha.,7.alpha.,8a.beta.)]- $$ (

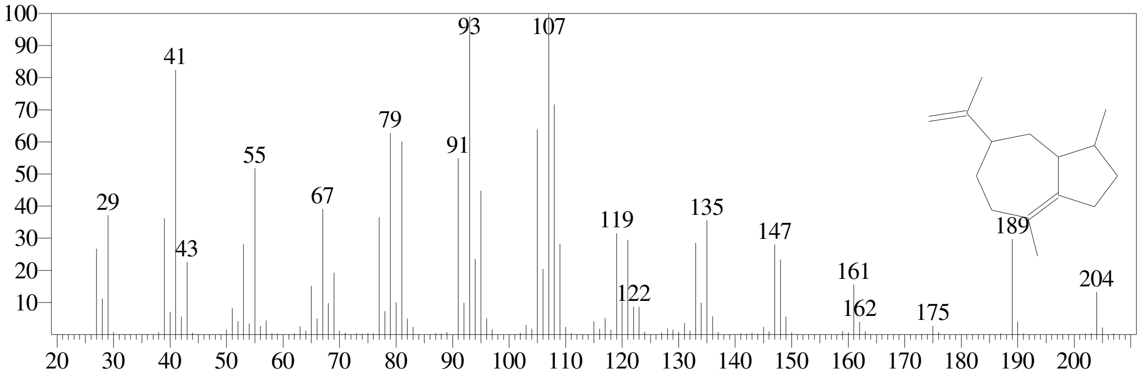

<< Target >>
Line#:18 R.Time:29.242(Scan#:3210) MassPeaks:24
RawMode:Averaged 29.233-29.250(3209-3211) BasePeak:105.05(4941)
BG Mode:None Group 1 - Event 1 Scan

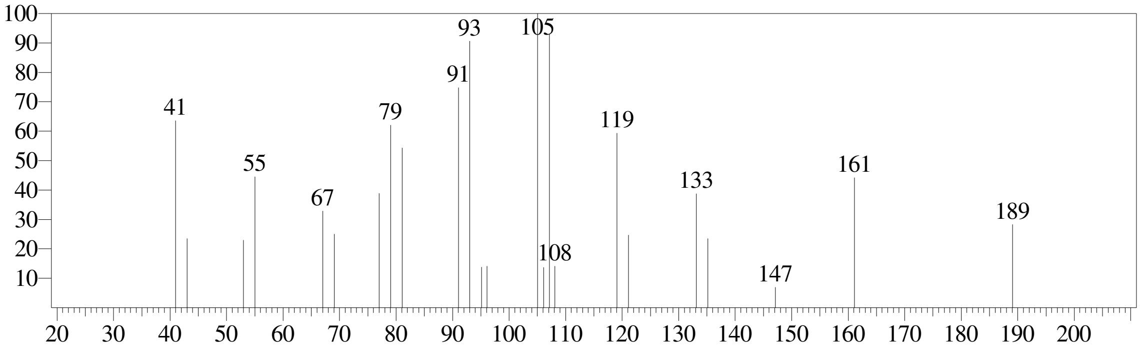

Hit#:3 Entry:24962 Library:NIST23s.lib
SI:82 Formula:C15H24 CAS:21747-46-6 MolWeight:204 RetIndex:1451
CompName:1H-Cycloprop[e]azulene, 1a,2,3,5,6,7,7a,7b-octahydro-1,1,4,7-tetramethyl-, [1aR-(1a.alpha.,7.alpha.,7a.beta.,

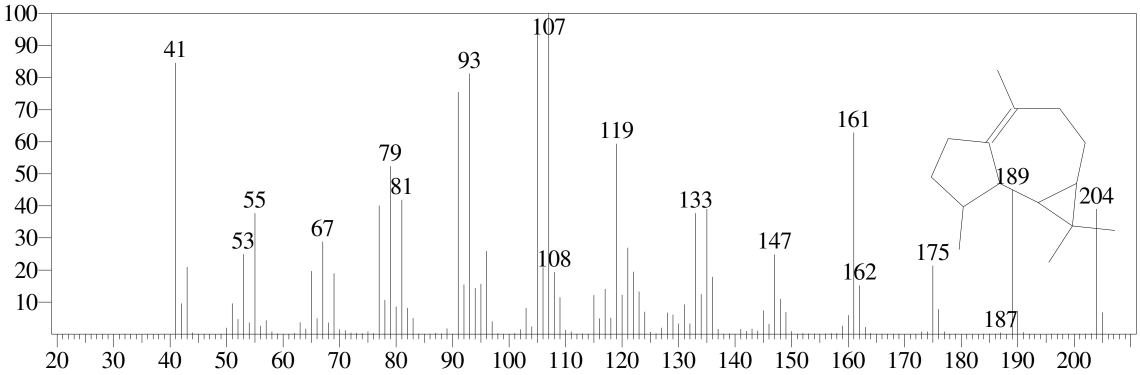

Hit#:4 Entry:62671 Library:NIST23-1.lib
SI:82 Formula:C13H20N2 CAS:0-00-0 MolWeight:204 RetIndex:1704
CompName:1,4-Methanocycloocta[d]pyridazine, 1,4,4a,5,6,9,10,10a-octahydro-11,11-dimethyl-, (1.alpha.,4.alpha.,4a.alpl

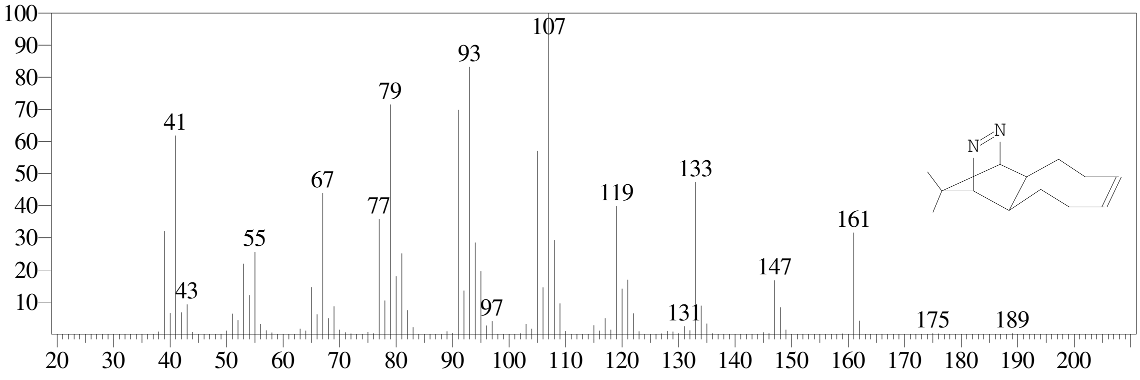

<< Target >>
Line#:18   R.Time:29.242(Scan#:3210) MassPeaks:24
RawMode:Averaged 29.233-29.250(3209-3211) BasePeak:105.05(4941)
BG Mode:None Group 1 - Event 1 Scan

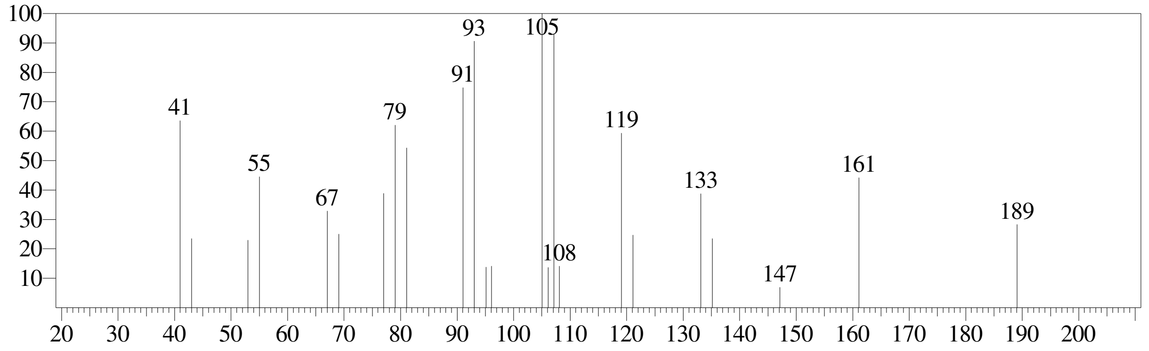

Hit#:5 Entry:24964 Library:NIST23s.lib
SI:82 Formula:C15H24 CAS:21747-46-6 MolWeight:204 RetIndex:1451
CompName:1H-Cycloprop[e]azulene, 1a,2,3,5,6,7,7a,7b-octahydro-1,1,4,7-tetramethyl-, [1aR-(1a.alpha.,7.alpha.,7a.beta.,

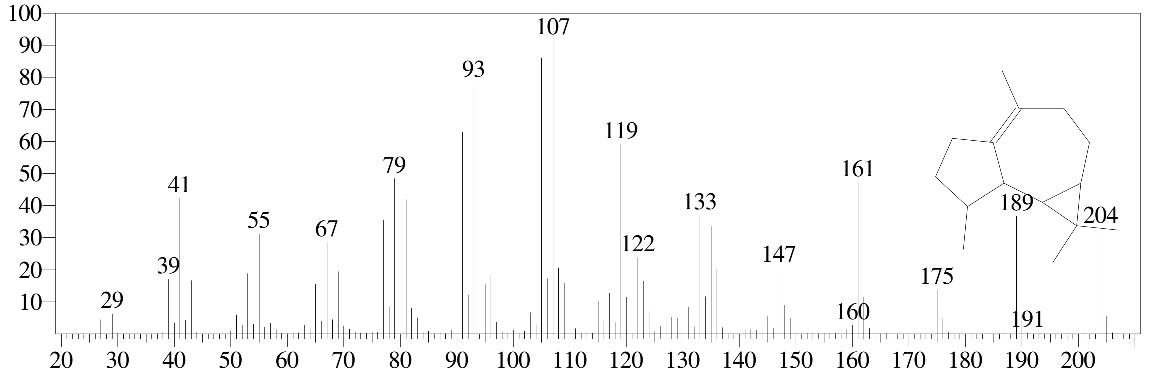

<< Target >>
Line#:19 R.Time:29.767(Scan#:3273) MassPeaks:34
RawMode:Averaged 29.758-29.775(3272-3274) BasePeak:69.05(18219)
BG Mode:None Group 1 - Event 1 Scan

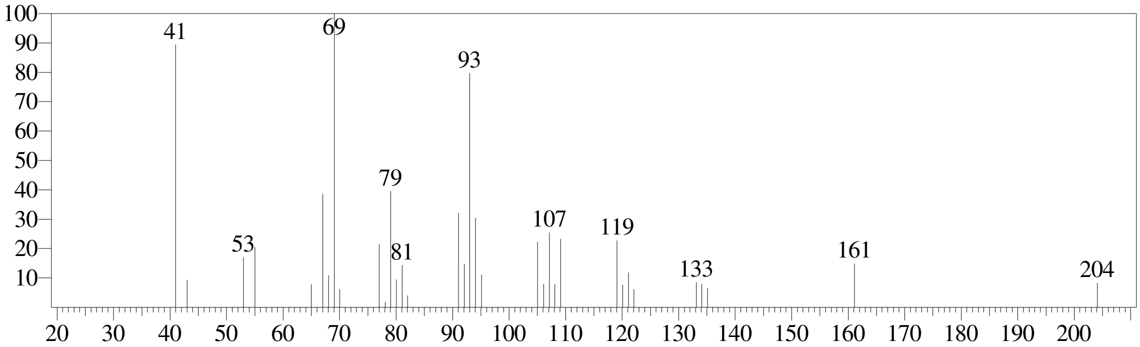

Hit#:1 Entry:24855 Library:NIST23s.lib
SI:94 Formula:C15H24 CAS:495-61-4 MolWeight:204 RetIndex:1502
CompName:.beta.-Bisabolene $$ Cyclohexene, 1-methyl-4-(5-methyl-1-methylene-4-hexenyl)-, (S)- $$ 1,5-Heptadiene, 6

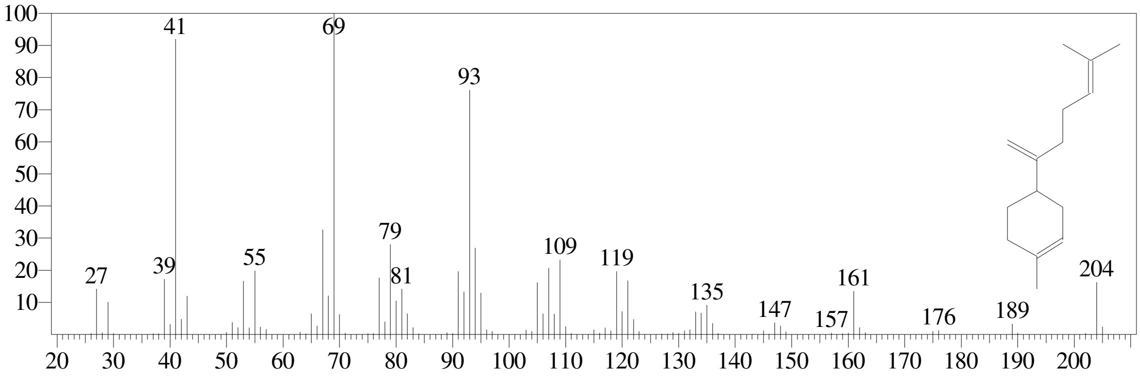

Hit#:2 Entry:24810 Library:NIST23s.lib
SI:93 Formula:C15H24 CAS:495-61-4 MolWeight:204 RetIndex:1502
CompName:.beta.-Bisabolene $$ Cyclohexene, 1-methyl-4-(5-methyl-1-methylene-4-hexenyl)-, (S)- $$ 1,5-Heptadiene, 6

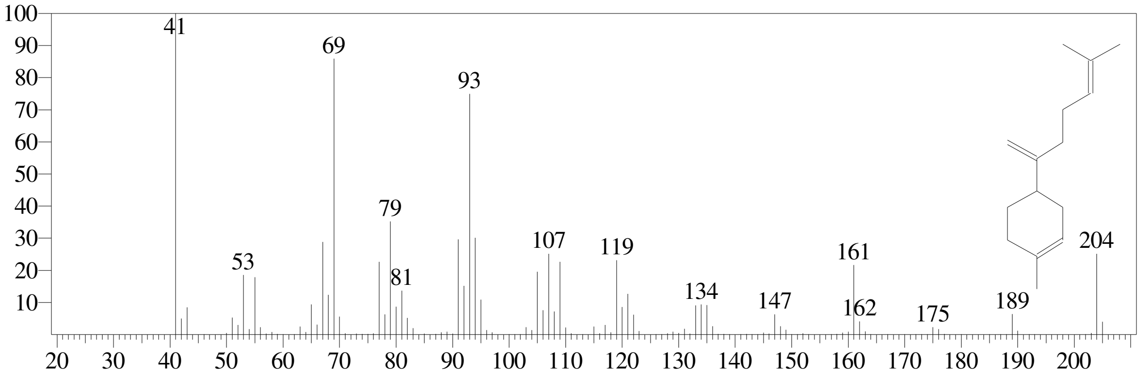

<< Target >>
Line#:19   R.Time:29.767(Scan#:3273) MassPeaks:34
RawMode:Averaged 29.758-29.775(3272-3274) BasePeak:69.05(18219)
BG Mode:None Group 1 - Event 1 Scan

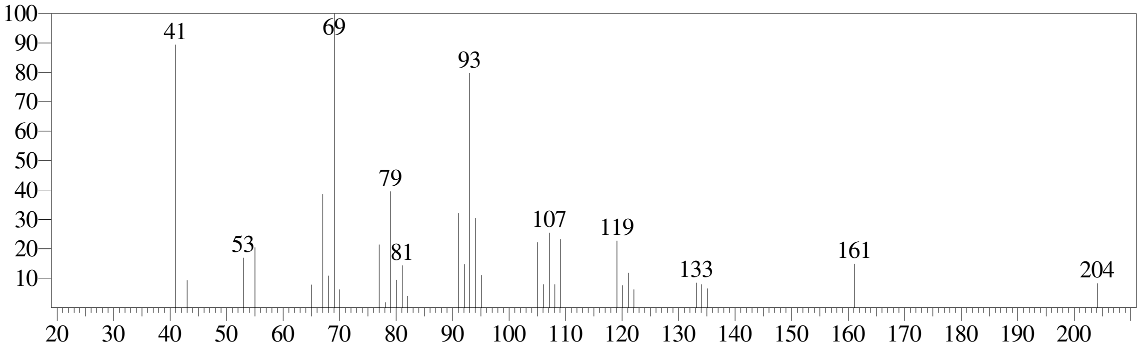

Hit#:3 Entry:24856 Library:NIST23s.lib
SI:93 Formula:C15H24 CAS:495-61-4 MolWeight:204 RetIndex:1502
CompName:.beta.-Bisabolene $$ Cyclohexene, 1-methyl-4-(5-methyl-1-methylene-4-hexenyl)-, (S)- $$ 1,5-Heptadiene, 6

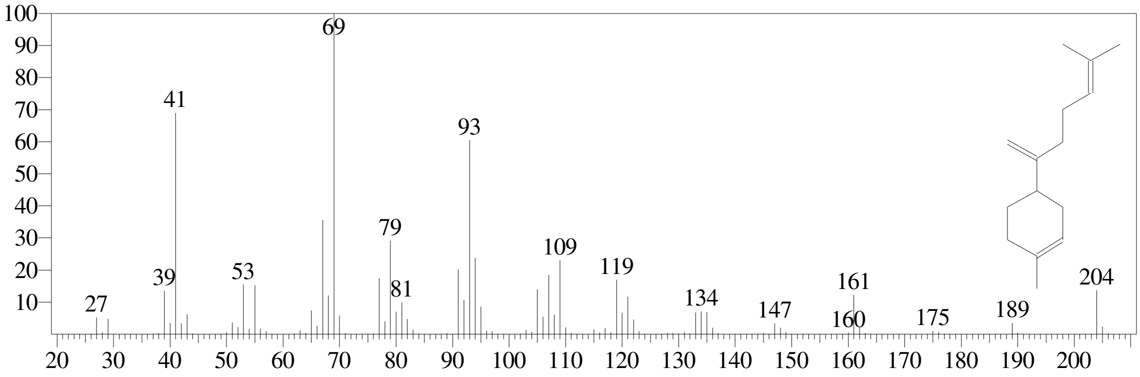

Hit#:4 Entry:62778 Library:NIST23-1.lib
SI:91 Formula:C15H24 CAS:495-61-4 MolWeight:204 RetIndex:1502
CompName:.beta.-Bisabolene $$ Cyclohexene, 1-methyl-4-(5-methyl-1-methylene-4-hexenyl)-, (S)- $$ 1,5-Heptadiene, 6

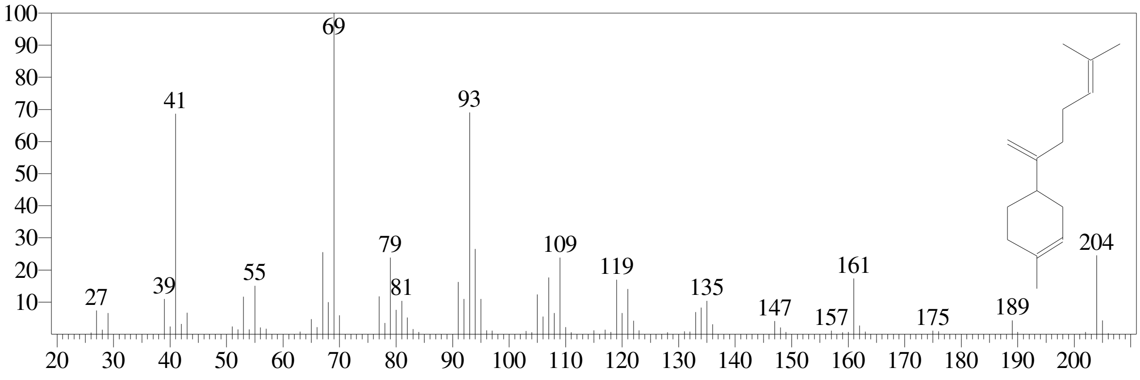

<< Target >>
Line#:19   R.Time:29.767(Scan#:3273) MassPeaks:34
RawMode:Averaged 29.758-29.775(3272-3274) BasePeak:69.05(18219)
BG Mode:None Group 1 - Event 1 Scan

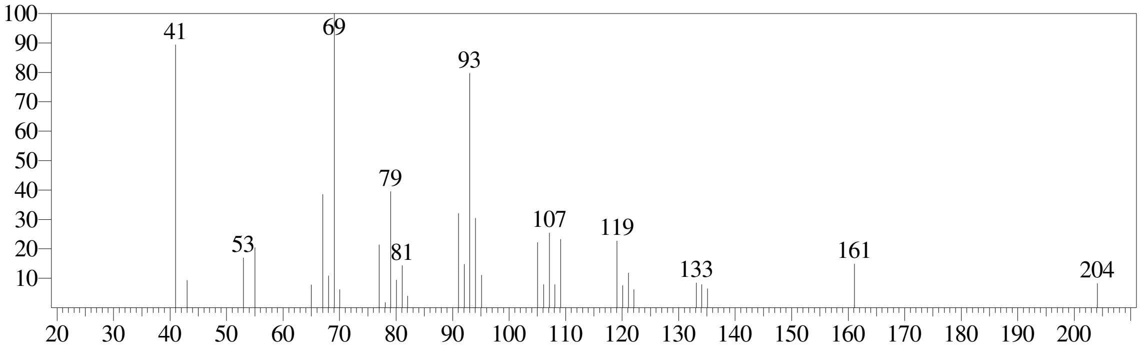

Hit#:5 Entry:24849 Library:NIST23s.lib
SI:91 Formula:C15H24 CAS:18794-84-8 MolWeight:204 RetIndex:1465
CompName:(E)-.beta.-Farnesene $$ 1,6,10-Dodecatriene, 7,11-dimethyl-3-methylene-, (E)- $$ 7,11-Dimethyl-3-methylen

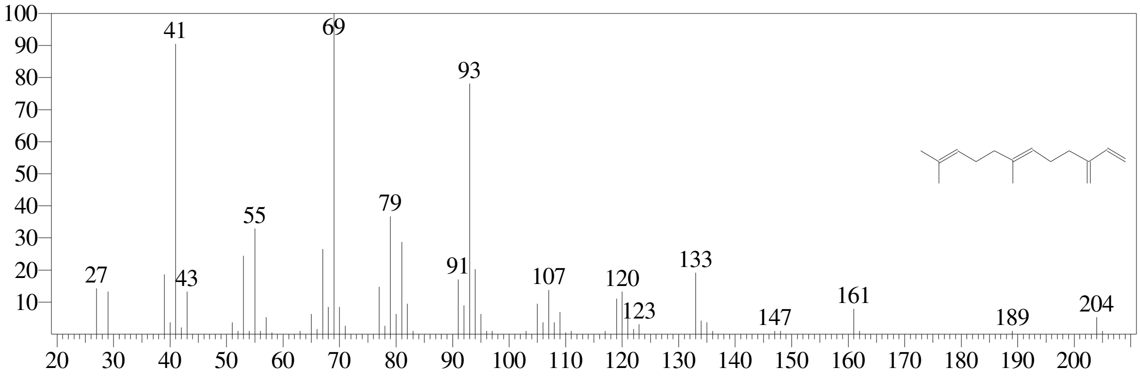

<< Target >>
Line#:20 R.Time:30.025(Scan#:3304) MassPeaks:24
RawMode:Averaged 30.017-30.033(3303-3305) BasePeak:43.00(5178)
BG Mode:None Group 1 - Event 1 Scan

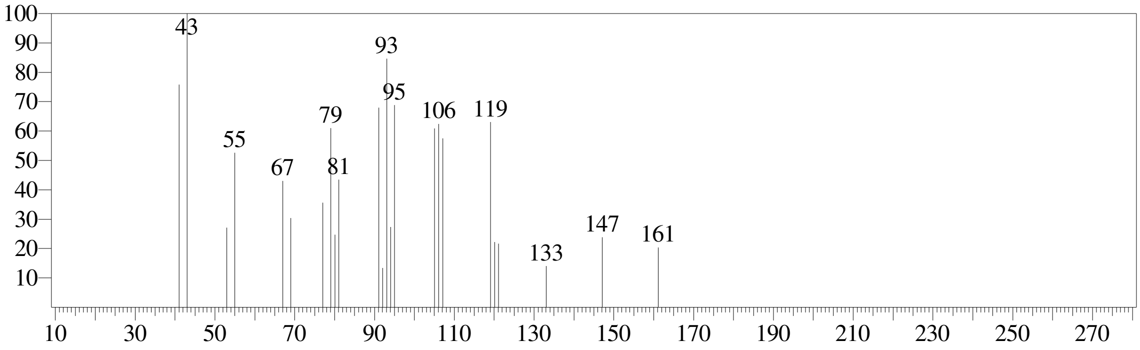

Hit#:1 Entry:62671 Library:NIST23-1.lib
SI:82 Formula:C13H20N2 CAS:0-00-0 MolWeight:204 RetIndex:1704
CompName:1,4-Methanocycloocta[d]pyridazine, 1,4,4a,5,6,9,10,10a-octahydro-11,11-dimethyl-, (1.alpha.,4.alpha.,4a.alph

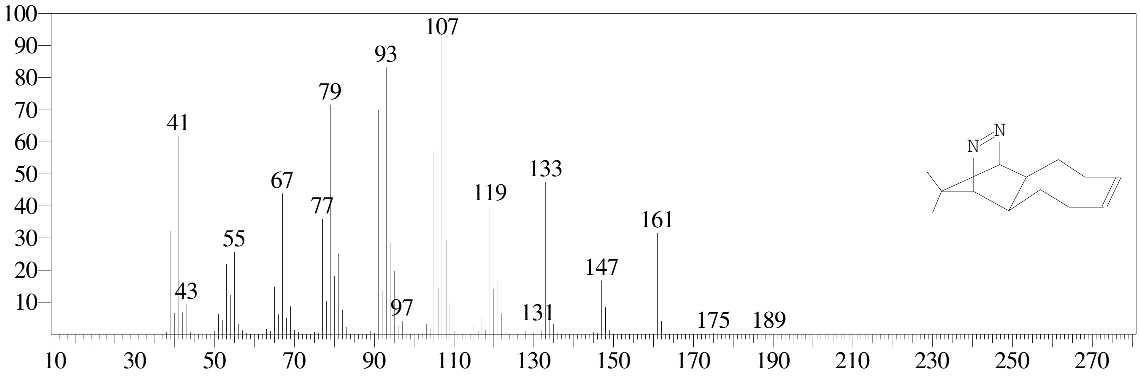

Hit#:2 Entry:62755 Library:NIST23-1.lib
SI:81 Formula:C15H24 CAS:85048-01-7 MolWeight:204 RetIndex:1436
CompName:Aromadendrane,dehydro-

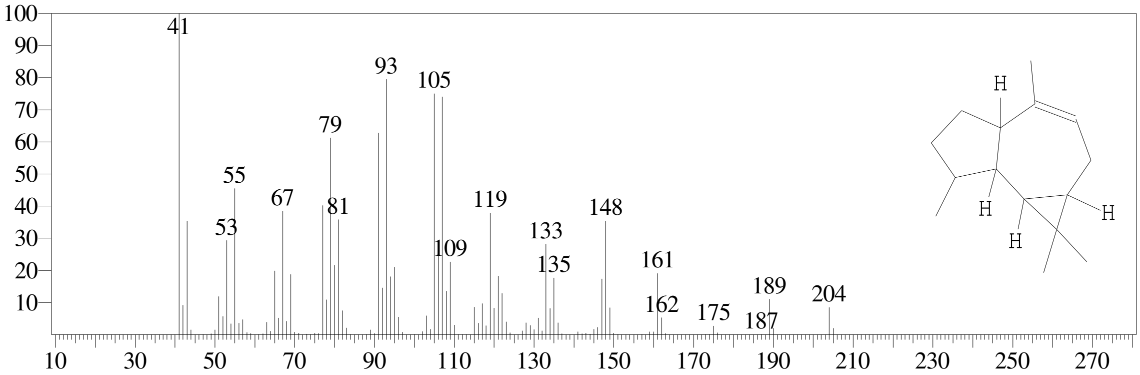

<< Target >>
Line#:20   R.Time:30.025(Scan#:3304) MassPeaks:24
RawMode:Averaged 30.017-30.033(3303-3305) BasePeak:43.00(5178)
BG Mode:None Group 1 - Event 1 Scan

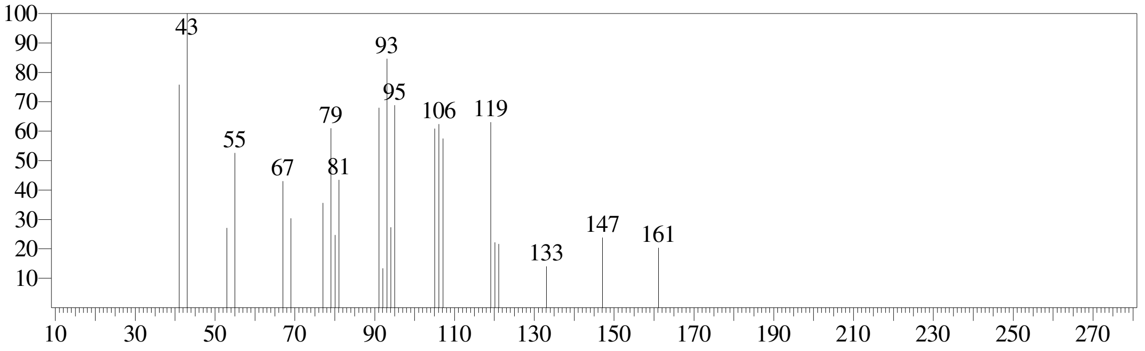

Hit#:3 Entry:149236 Library:NIST23-1.lib
SI:81 Formula:C20H34 CAS:28393-07-9 MolWeight:274 RetIndex:2186
CompName:Icosa-9,11-diyne

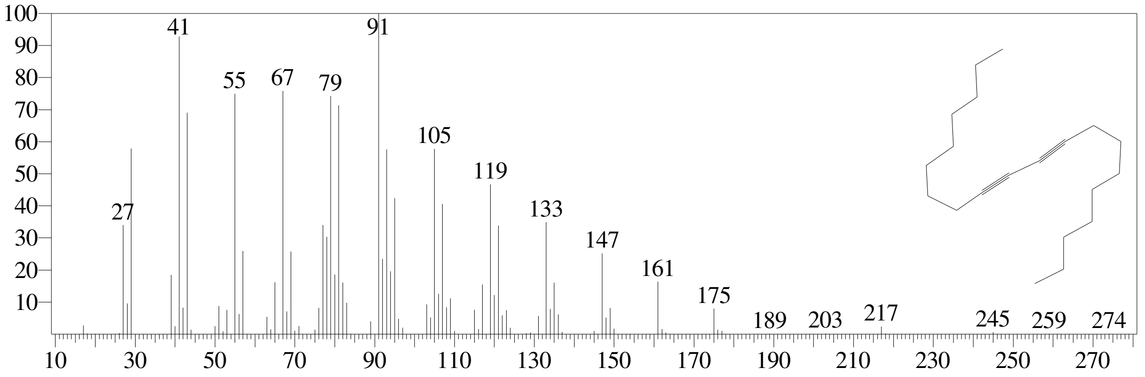

Hit#:4 Entry:62901 Library:NIST23-1.lib
SI:81 Formula:C15H24 CAS:622792-97-6 MolWeight:204 RetIndex:1530
CompName:Bisabolene,(E)-iso-gamma-

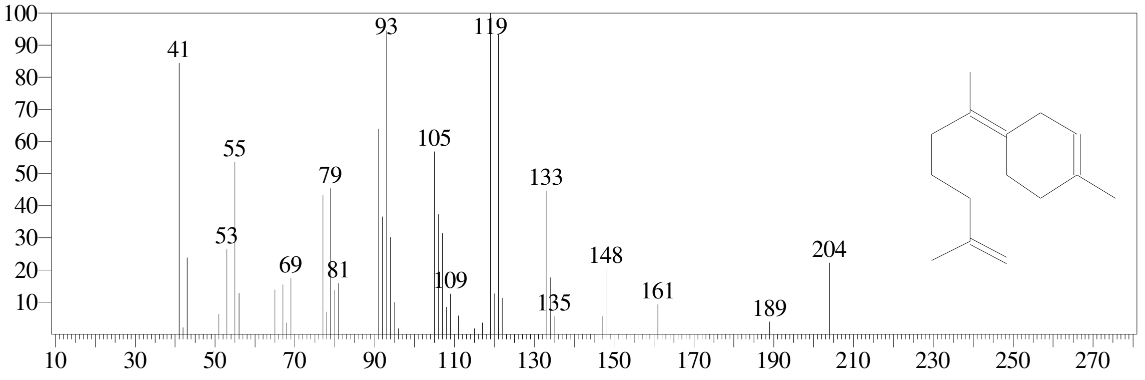

<< Target >>
Line#:20 R.Time:30.025(Scan#:3304) MassPeaks:24
RawMode:Averaged 30.017-30.033(3303-3305) BasePeak:43.00(5178)
BG Mode:None Group 1 - Event 1 Scan

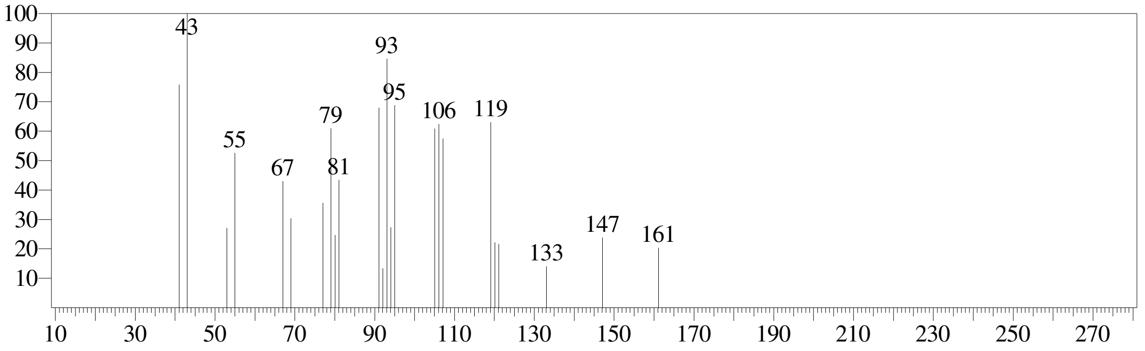

Hit#:5 Entry:24803 Library:NIST23s.lib
SI:81 Formula:C15H24 CAS:118-65-0 MolWeight:204 RetIndex:1448
CompName:Bicyclo[7.2.0]undec-4-ene, 4,11,11-trimethyl-8-methylene-,[1R-(1R*,4Z,9S*)]- $$ Isocaryophyllene $$ 4,11,

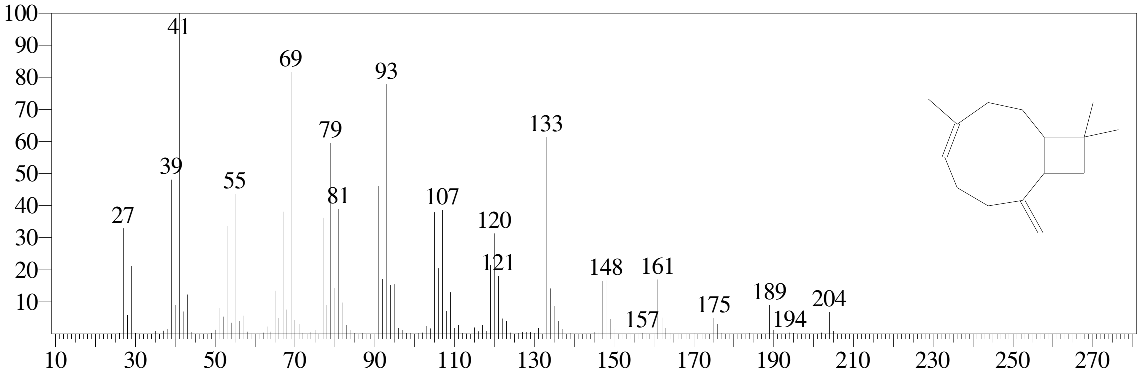

<< Target >>
Line#:21 R.Time:30.375(Scan#:3346) MassPeaks:21
RawMode:Averaged 30.367-30.383(3345-3347) BasePeak:119.10(6194)
BG Mode:Calc. from Peak Group 1 - Event 1 Scan

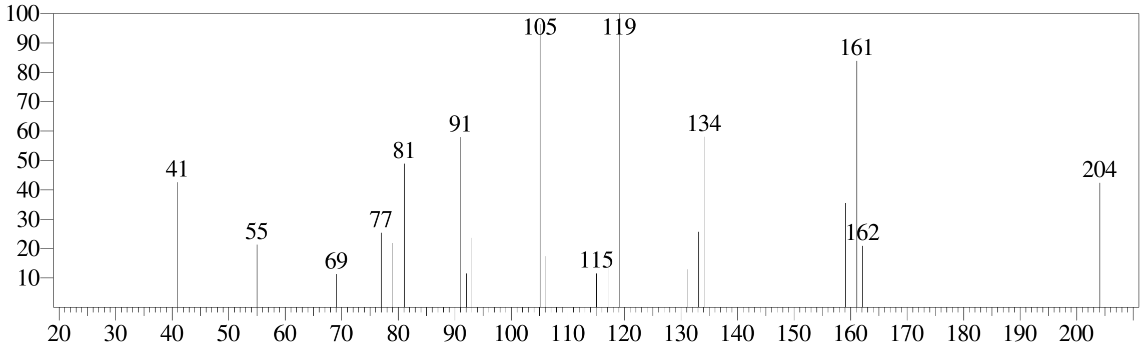

Hit#:1 Entry:25084 Library:NIST23s.lib
SI:82 Formula:C15H24 CAS:157374-44-2 MolWeight:204 RetIndex:1449
CompName:cis-muurola-3,5-diene

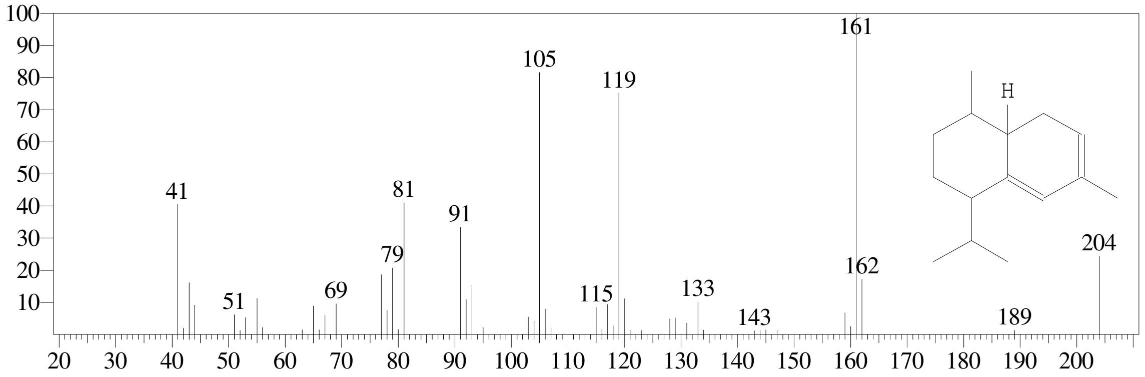

Hit#:2 Entry:24949 Library:NIST23s.lib
SI:82 Formula:C15H24 CAS:17699-14-8 MolWeight:204 RetIndex:1381
CompName:.alpha.-Cubebene $$ 1H-Cyclopenta[1,3]cyclopropa[1,2]benzene, 3a,3b,4,5,6,7-hexahydro-3,7-dimethyl-4-(1

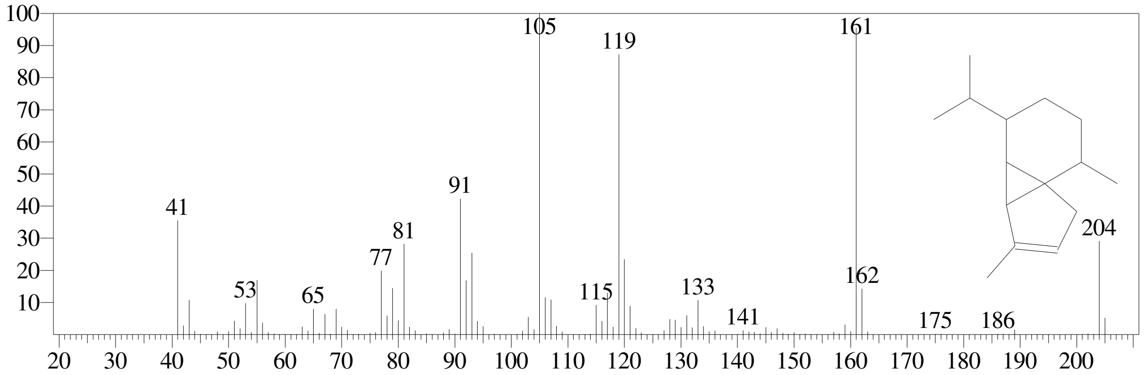

<< Target >>
Line#:21   R.Time:30.375(Scan#:3346) MassPeaks:21
RawMode:Averaged 30.367-30.383(3345-3347) BasePeak:119.10(6194)
BG Mode:Calc. from Peak Group 1 - Event 1 Scan

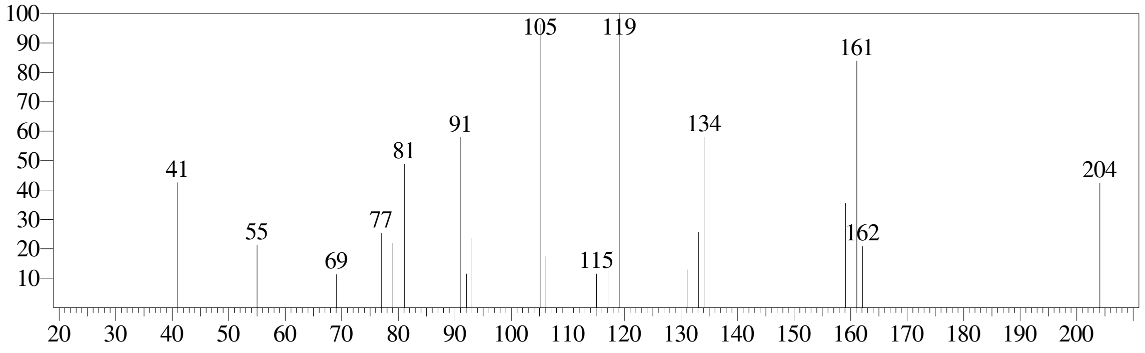

Hit#:3 Entry:25097 Library:NIST23s.lib
SI:81 Formula:C15H24 CAS:483-76-1 MolWeight:204 RetIndex:1526
CompName:Naphthalene, 1,2,3,5,6,8a-hexahydro-4,7-dimethyl-1-(1-methylethyl)-, (1S-cis)- $$ Cadina-1(10),4-diene $$.

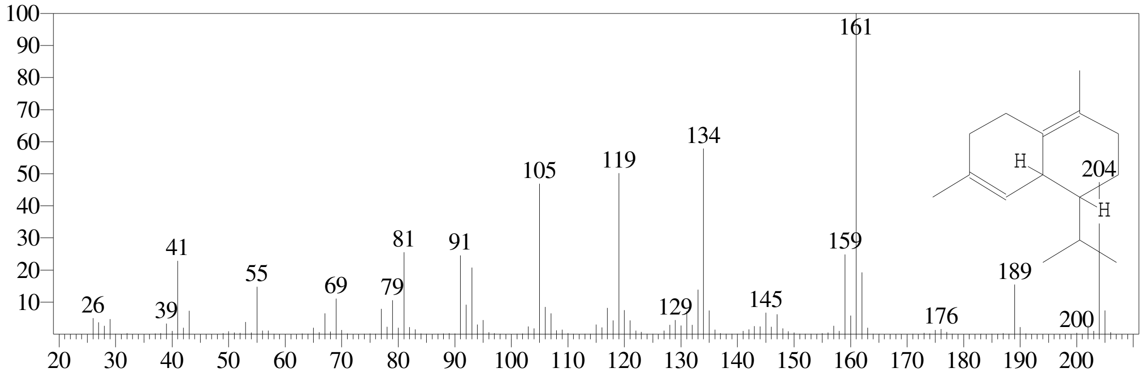

Hit#:4 Entry:62988 Library:NIST23-1.lib
SI:81 Formula:C15H24 CAS:17699-14-8 MolWeight:204 RetIndex:1381
CompName:.alpha.-Cubebene $$ 1H-Cyclopenta[1,3]cyclopropa[1,2]benzene, 3a,3b,4,5,6,7-hexahydro-3,7-dimethyl-4-(1

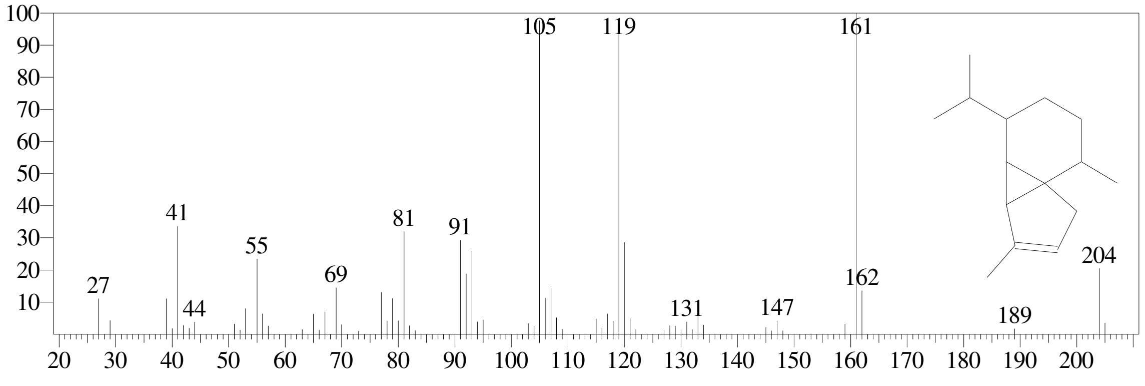

<< Target >>
Line#:21 R.Time:30.375(Scan#:3346) MassPeaks:21
RawMode:Averaged 30.367-30.383(3345-3347) BasePeak:119.10(6194)
BG Mode:Calc. from Peak Group 1 - Event 1 Scan

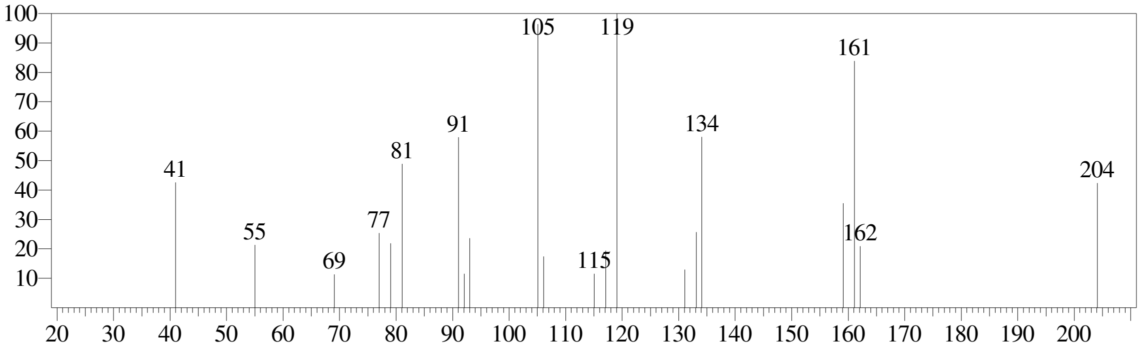

Hit#:5 Entry:24948 Library:NIST23s.lib
SI:81 Formula:C15H24 CAS:17699-14-8 MolWeight:204 RetIndex:1381
CompName:.alpha.-Cubebene $$ 1H-Cyclopenta[1,3]cyclopropa[1,2]benzene, 3a,3b,4,5,6,7-hexahydro-3,7-dimethyl-4-(1

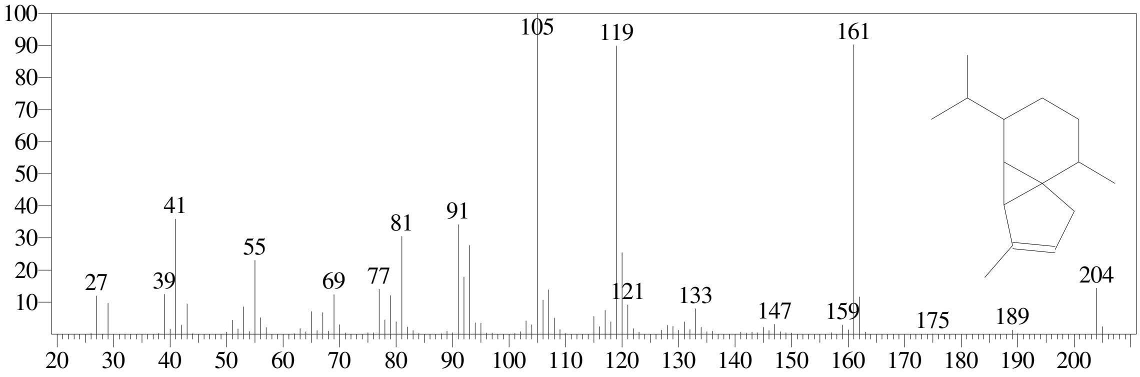

<< Target >>
Line#:22 R.Time:30.817(Scan#:3399) MassPeaks:17
RawMode:Averaged 30.808-30.825(3398-3400) BasePeak:105.05(3520)
BG Mode:None Group 1 - Event 1 Scan

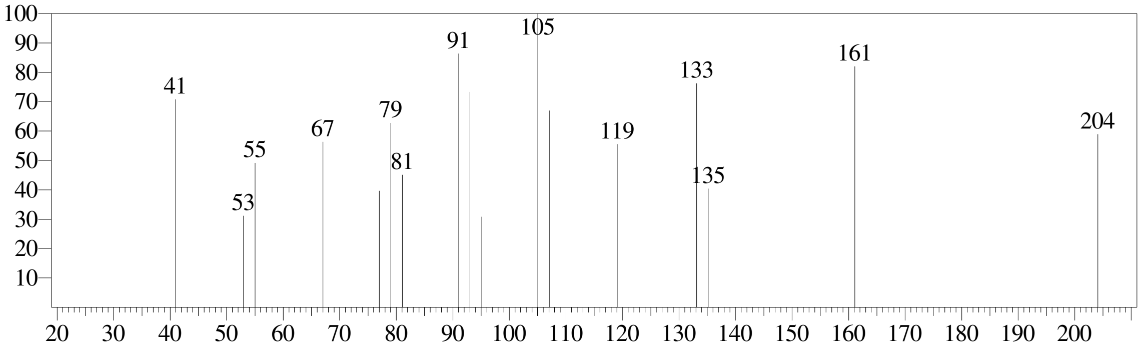

Hit#:1 Entry:25043 Library:NIST23s.lib
SI:78 Formula:C15H24 CAS:10219-75-7 MolWeight:204 RetIndex:1496
CompName:Naphthalene, 1,2,3,5,6,7,8,8a-octahydro-1,8a-dimethyl-7-(1-methylethenyl)-, [1S-(1.alpha.,7.alpha.,8a.alpha.)

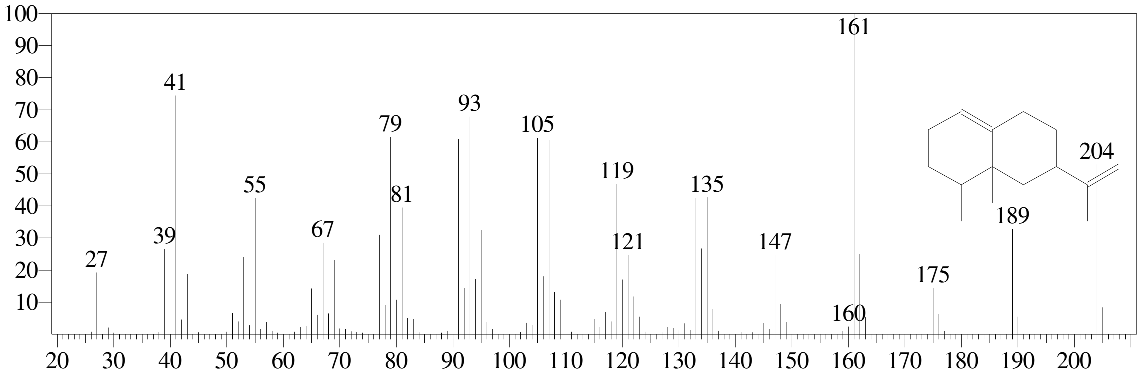

Hit#:2 Entry:25041 Library:NIST23s.lib
SI:78 Formula:C15H24 CAS:4630-07-3 MolWeight:204 RetIndex:1496
CompName:Naphthalene, 1,2,3,5,6,7,8,8a-octahydro-1,8a-dimethyl-7-(1-methylethenyl)-, [1R-(1.alpha.,7.beta.,8a.alpha.)]

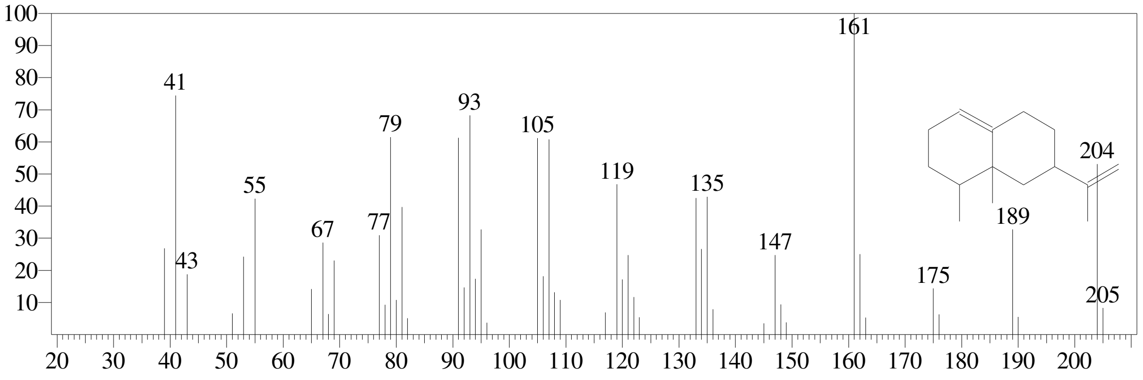

<< Target >>
Line#:22 R.Time:30.817(Scan#:3399) MassPeaks:17
RawMode:Averaged 30.808-30.825(3398-3400) BasePeak:105.05(3520)
BG Mode:None Group 1 - Event 1 Scan

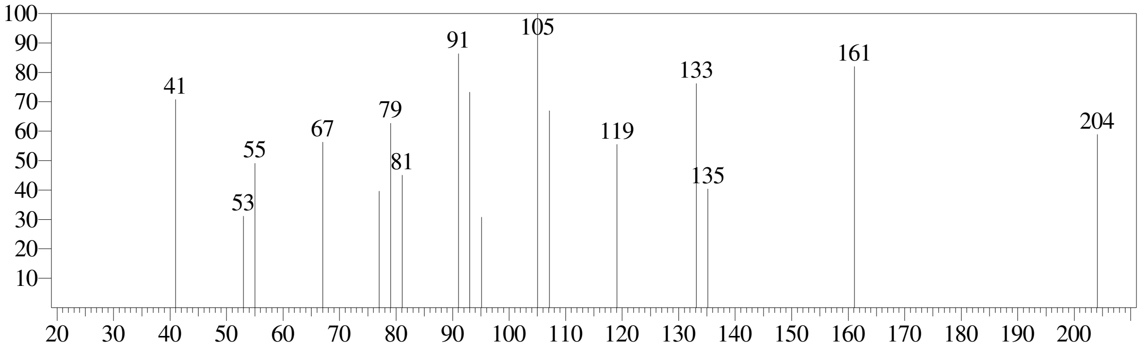

Hit#:3 Entry:24841 Library:NIST23s.lib
SI:78 Formula:C15H24 CAS:4630-07-3 MolWeight:204 RetIndex:1496
CompName:Naphthalene, 1,2,3,5,6,7,8,8a-octahydro-1,8a-dimethyl-7-(1-methylethenyl)-, [1R-(1.alpha.,7.beta.,8a.alpha.)]

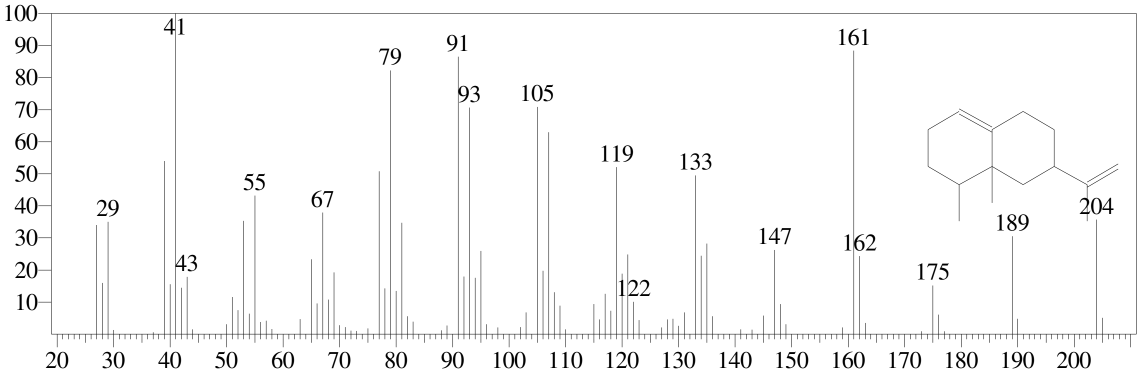

Hit#:4 Entry:62997 Library:NIST23-1.lib
SI:77 Formula:C15H24 CAS:58893-88-2 MolWeight:204 RetIndex:1527
CompName:(4aR,8aS)-4a-Methyl-1-methylene-7-(propan-2-ylidene)decahydronaphthalene $$ Naphthalene, decahydro-4a

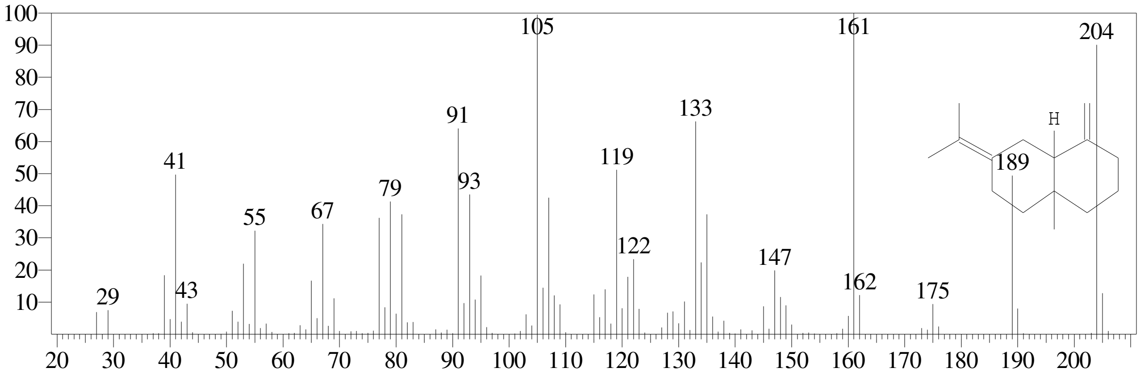

<< Target >>
Line#:22   R.Time:30.817(Scan#:3399) MassPeaks:17
RawMode:Averaged 30.808-30.825(3398-3400) BasePeak:105.05(3520)
BG Mode:None Group 1 - Event 1 Scan

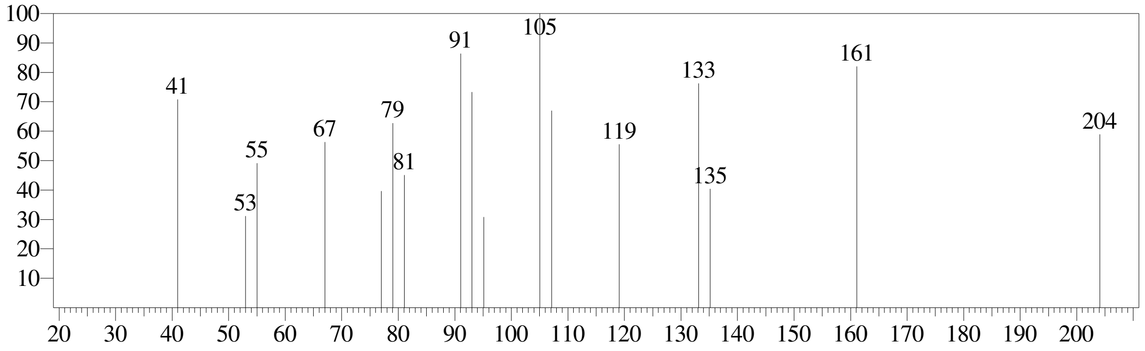

Hit#:5 Entry:62974 Library:NIST23-1.lib
SI:77 Formula:C15H24 CAS:24741-64-8 MolWeight:204 RetIndex:1496
CompName:(3R,4aS,5R)-4a,5-Dimethyl-3-(prop-1-en-2-yl)-1,2,3,4,4a,5,6,7-octahydronaphthalene $$ Naphthalene, 1,2,3,

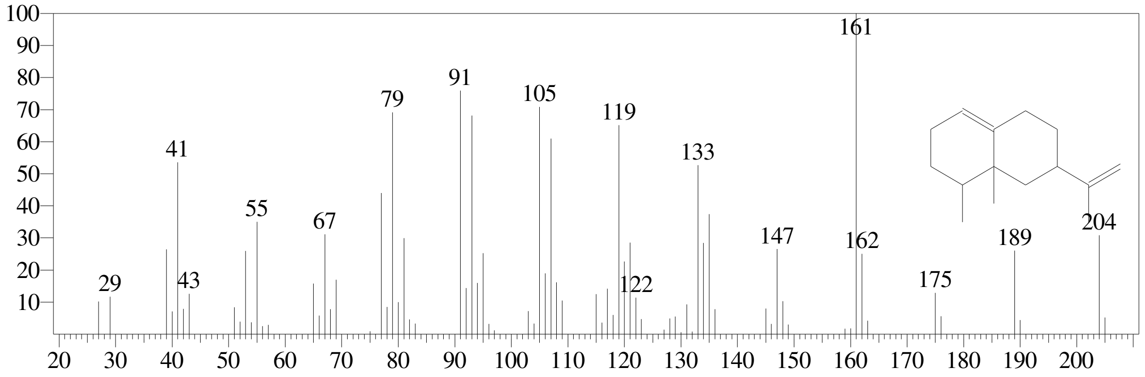

<< Target >>
Line#:23   R.Time:32.467(Scan#:3597)   MassPeaks:39
RawMode:Averaged 32.458-32.475(3596-3598) BasePeak:43.00(10333)
BG Mode:None Group 1 - Event 1 Scan

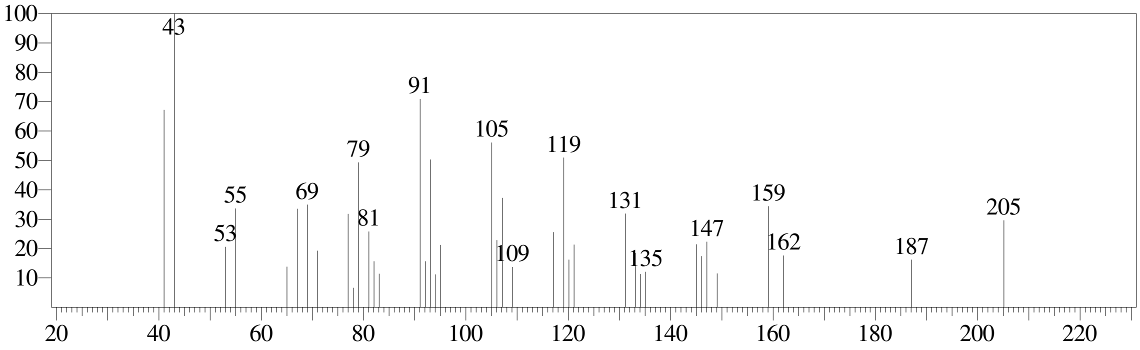

Hit#:1 Entry:28181 Library:NIST23s.lib
SI:94 Formula:C15H24O CAS:6750-60-3 MolWeight:220 RetIndex:1584
CompName:1H-Cycloprop[e]azulen-7-ol, decahydro-1,1,7-trimethyl-4-methylene-, [1ar-(1a.alpha.,4a.alpha.,7.beta.,7a.bet

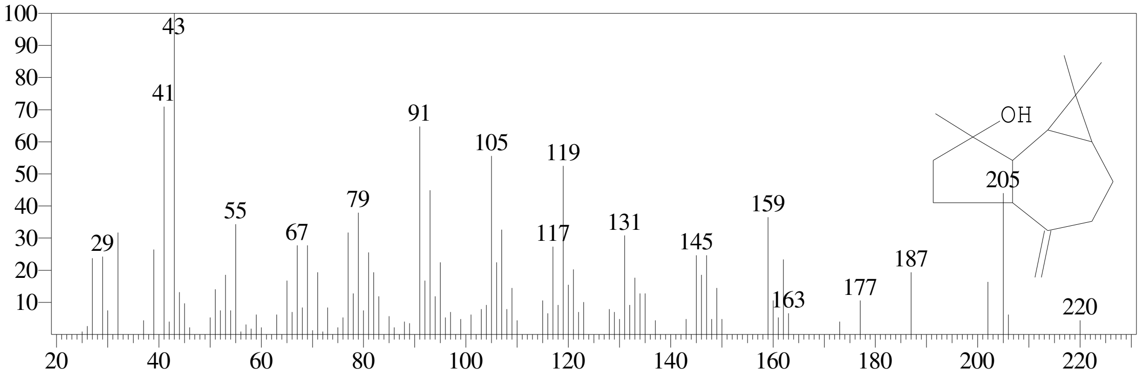

Hit#:2 Entry:80771 Library:NIST23-1.lib
SI:92 Formula:C15H24O CAS:77171-55-2 MolWeight:220 RetIndex:1584
CompName:(-)-Spathulenol $$ (1aS,4aS,7R,7aS,7bS)-1,1,7-Trimethyl-4-methylenedecahydro-1H-cyclopropa[e]azulen-7-

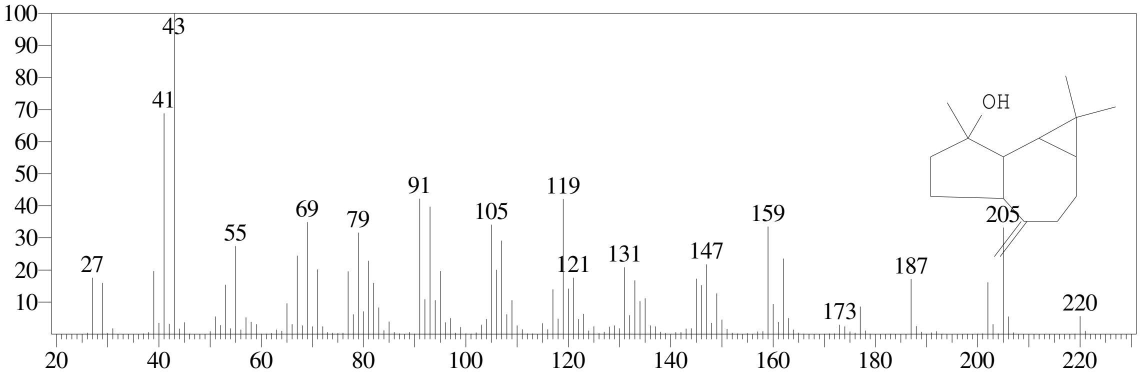

<< Target >>
Line#:23 R.Time:32.467(Scan#:3597) MassPeaks:39
RawMode:Averaged 32.458-32.475(3596-3598) BasePeak:43.00(10333)
BG Mode:None Group 1 - Event 1 Scan

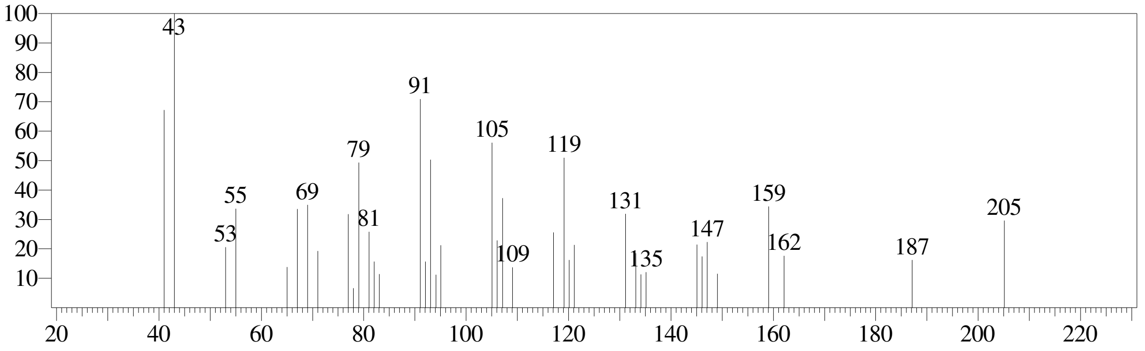

Hit#:3 Entry:80776 Library:NIST23-1.lib
SI:90 Formula:C15H24O CAS:6750-60-3 MolWeight:220 RetIndex:1584
CompName:1H-Cycloprop[e]azulen-7-ol, decahydro-1,1,7-trimethyl-4-methylene-, [1ar-(1a.alpha.,4a.alpha.,7.beta.,7a.bet

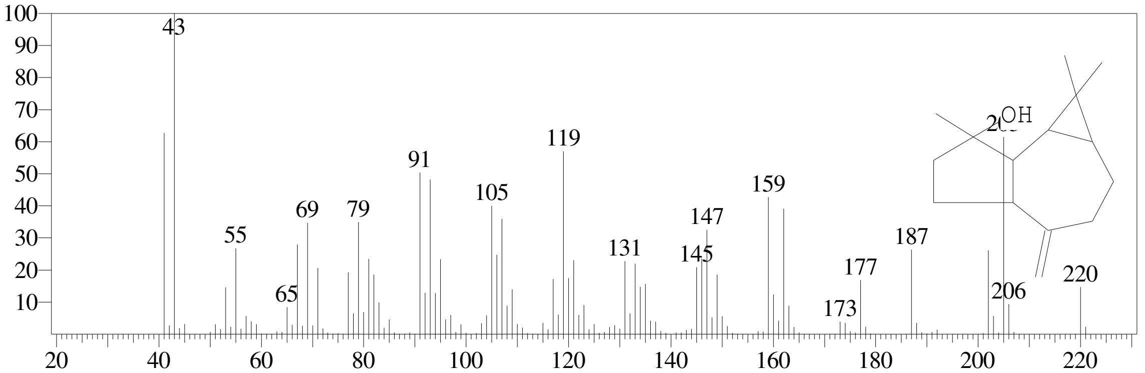

Hit#:4 Entry:28190 Library:NIST23s.lib
SI:90 Formula:C15H24O CAS:6750-60-3 MolWeight:220 RetIndex:1584
CompName:1H-Cycloprop[e]azulen-7-ol, decahydro-1,1,7-trimethyl-4-methylene-, [1ar-(1a.alpha.,4a.alpha.,7.beta.,7a.bet

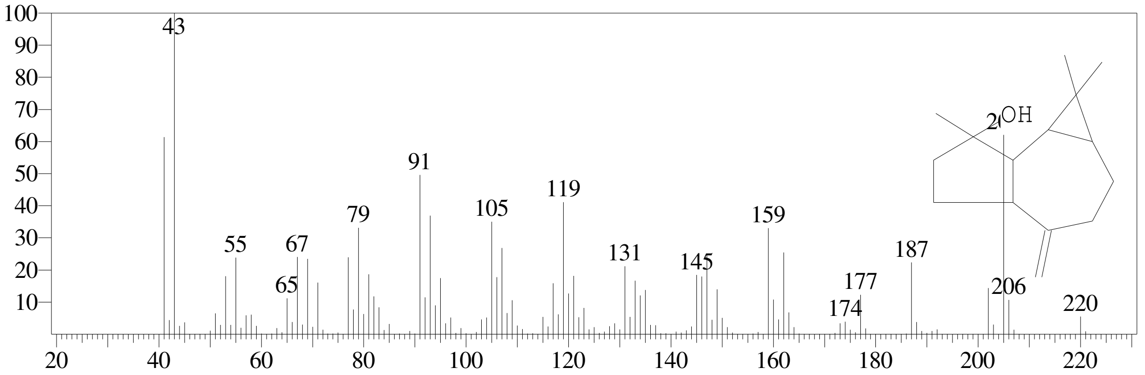

<< Target >>
Line#:23 R.Time:32.467(Scan#:3597) MassPeaks:39
RawMode:Averaged 32.458-32.475(3596-3598) BasePeak:43.00(10333)
BG Mode:None Group 1 - Event 1 Scan

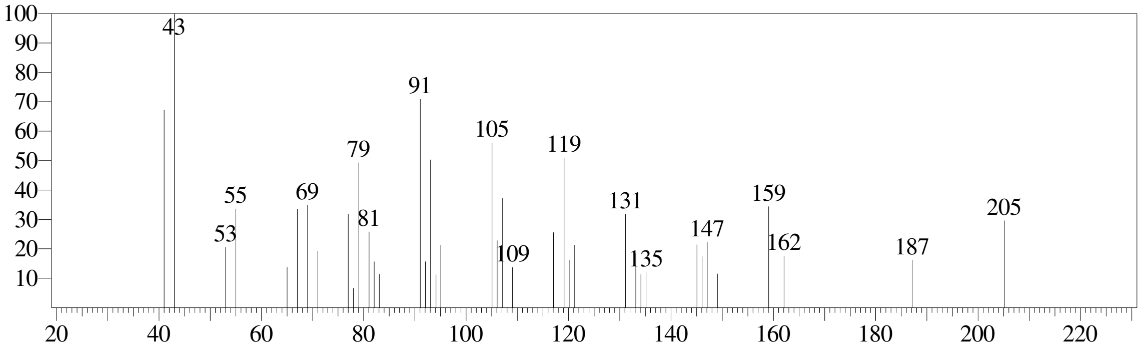

Hit#:5 Entry:28225 Library:NIST23s.lib
SI:85 Formula:C15H24O CAS:6750-60-3 MolWeight:220 RetIndex:1584
CompName:1H-Cycloprop[e]azulen-7-ol, decahydro-1,1,7-trimethyl-4-methylene-, [1ar-(1a.alpha.,4a.alpha.,7.beta.,7a.bet

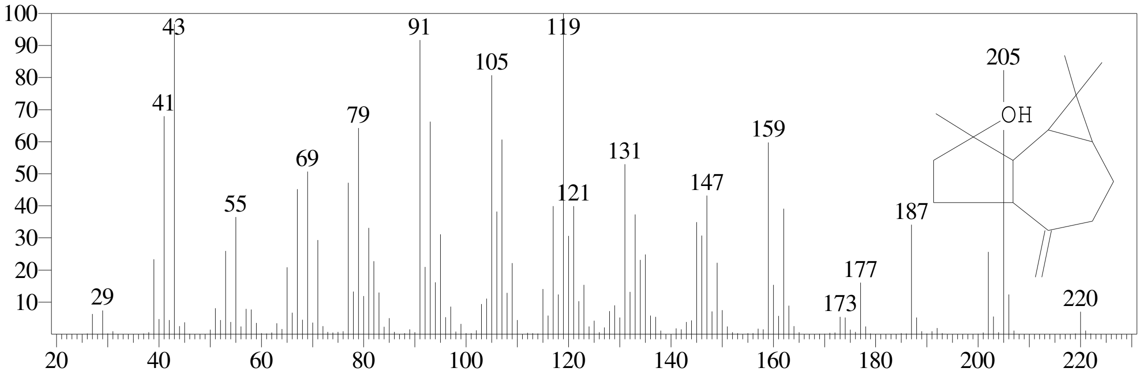

<< Target >>
Line#:24 R.Time:32.708(Scan#:3626) MassPeaks:17
RawMode:Averaged 32.700-32.717(3625-3627) BasePeak:43.00(3167)
BG Mode:Calc. from Peak Group 1 - Event 1 Scan

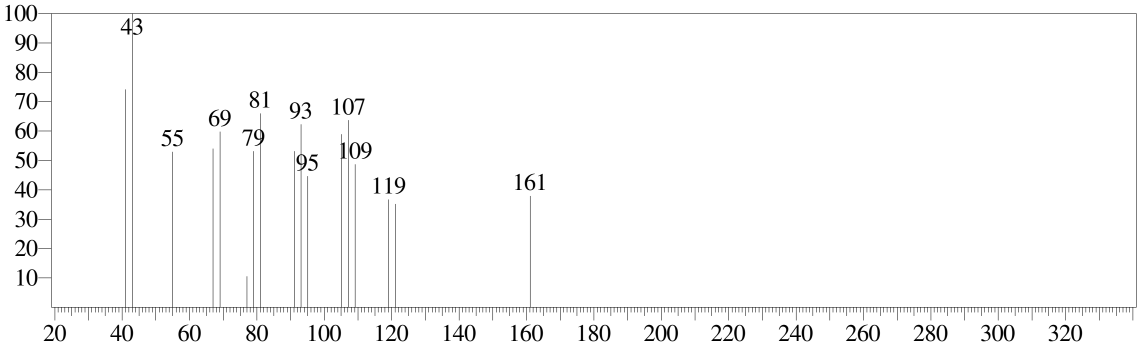

Hit#:1 Entry:135591 Library:NIST23-1.lib
SI:76 Formula:C17H28O2 CAS:25428-44-8 MolWeight:264 RetIndex:1786
CompName:Bisabolol acetate,alpha-

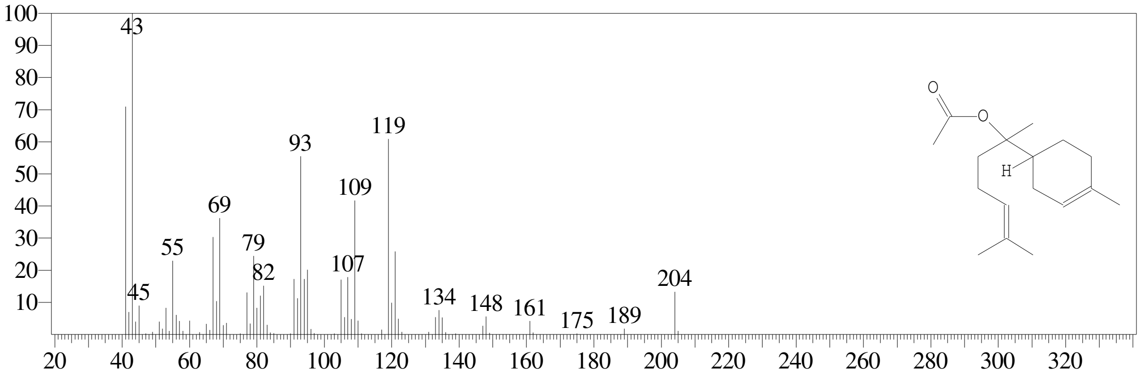

Hit#:2 Entry:83456 Library:NIST23-1.lib
SI:76 Formula:C15H26O CAS:515-69-5 MolWeight:222 RetIndex:1676
CompName:.alpha.-Bisabolol $$ 3-Cyclohexene-1-methanol, .alpha.,4-dimethyl-.alpha.-(4-methyl-3-penten-1-yl)-, (.alpha

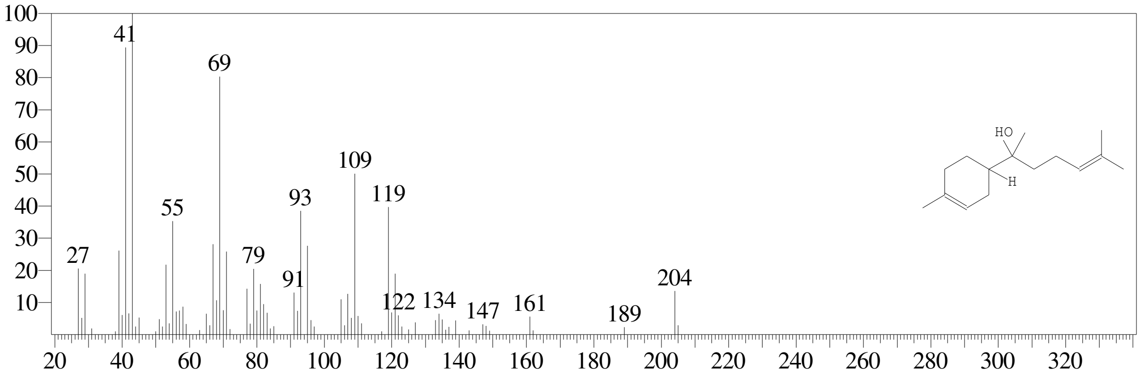

<< Target >>
Line#:24 R.Time:32.708(Scan#:3626) MassPeaks:17
RawMode:Averaged 32.700-32.717(3625-3627) BasePeak:43.00(3167)
BG Mode:Calc. from Peak Group 1 - Event 1 Scan

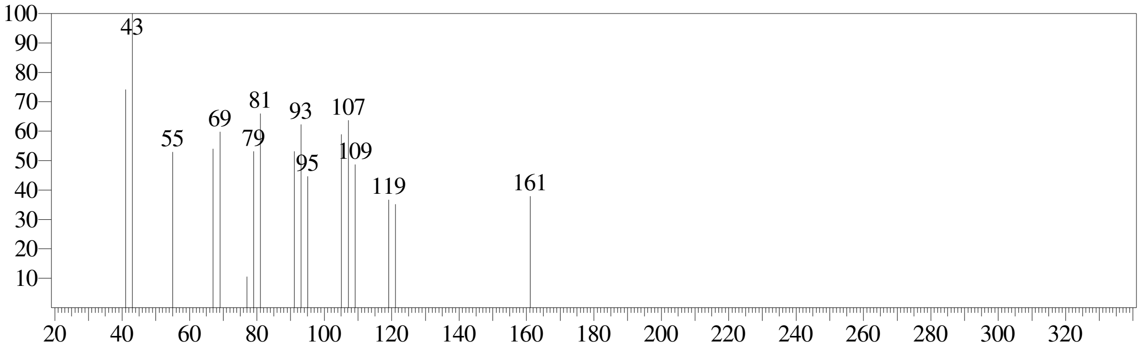

Hit#:3 Entry:24824 Library:NIST23s.lib
SI:76 Formula:C15H24 CAS:502-61-4 MolWeight:204 RetIndex:1509
CompName:.alpha.-Farnesene $$ 1,3,6,10-Dodecatetraene, 3,7,11-trimethyl-, (E,E)- $$ Farnesene $$ 2,6,10-Trimethyl-2,6

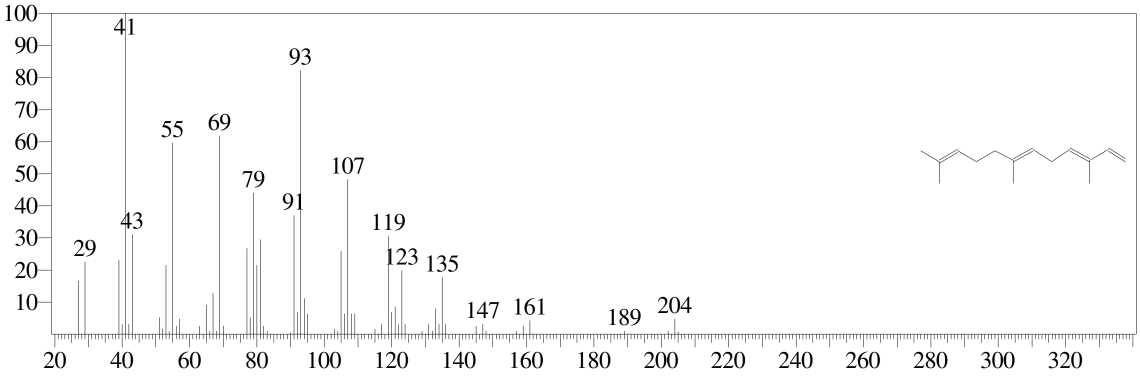

Hit#:4 Entry:135590 Library:NIST23-1.lib
SI:76 Formula:C17H28O2 CAS:256332-39-5 MolWeight:264 RetIndex:1786
CompName:Bisabolol acetate,epi-alpha-

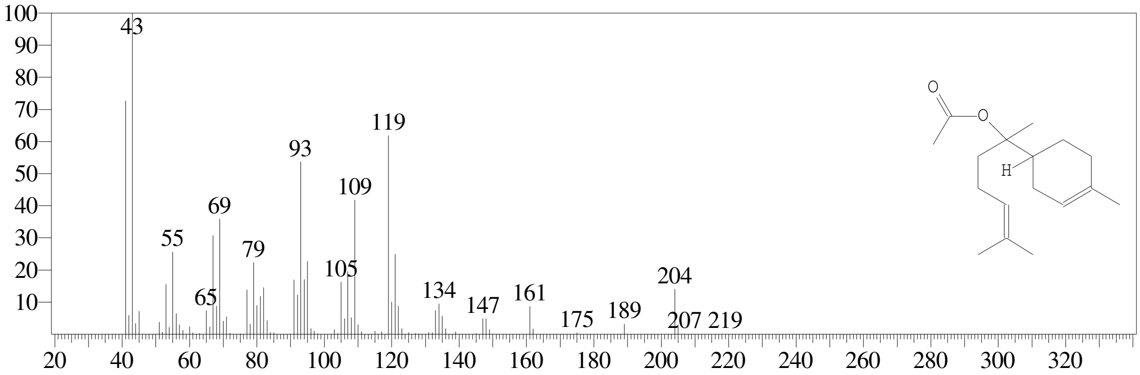

<< Target >>
Line#:24   R.Time:32.708(Scan#:3626) MassPeaks:17
RawMode:Averaged 32.700-32.717(3625-3627) BasePeak:43.00(3167)
BG Mode:Calc. from Peak Group 1 - Event 1 Scan

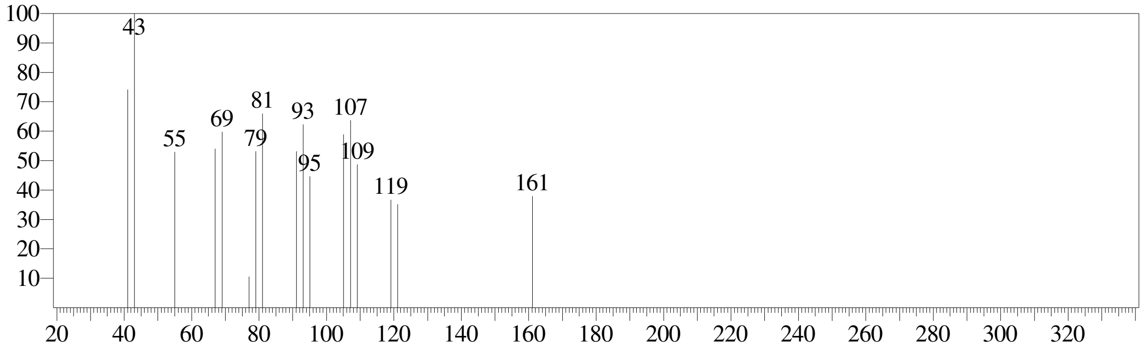

Hit#:5 Entry:226498 Library:NIST23-1.lib
SI:76 Formula:C22H36O2 CAS:41756-14-3 MolWeight:332 RetIndex:2234
CompName:8a(2H)-Phenanthrenol, 7-ethenyldodecahydro-1,1,4a,7-tetramethyl-, acetate, [4as-(4a.alpha.,4b.beta.,7.beta.,8

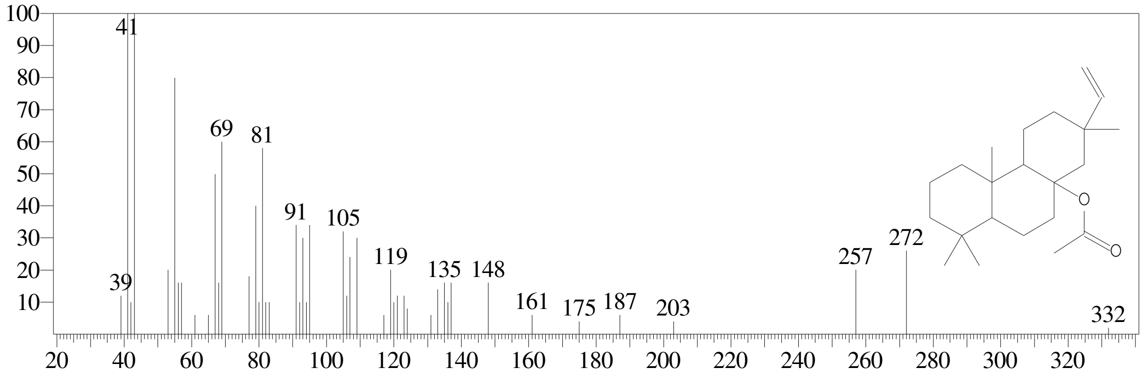

<< Target >>
Line#:25 R.Time:34.400(Scan#:3829) MassPeaks:18
RawMode:Averaged 34.392-34.408(3828-3830) BasePeak:119.10(2288)
BG Mode:Calc. from Peak Group 1 - Event 1 Scan

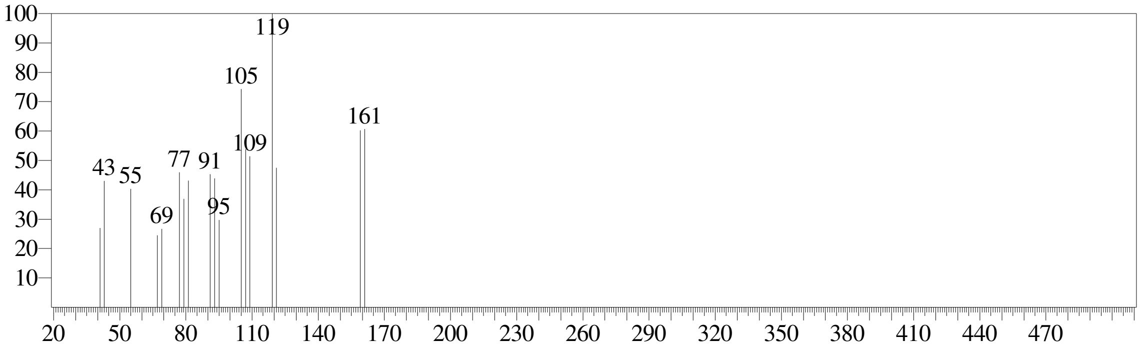

Hit#:1 Entry:95410 Library:NIST23-2.lib
SI:73 Formula:C41H52O5 CAS:0-00-0 MolWeight:624 RetIndex:4985
CompName:25-Dehydro-neotigogenin benzoate $$ (24Z)-26-(Benzoyloxy)furost-24-en-3-yl benzoate # $$

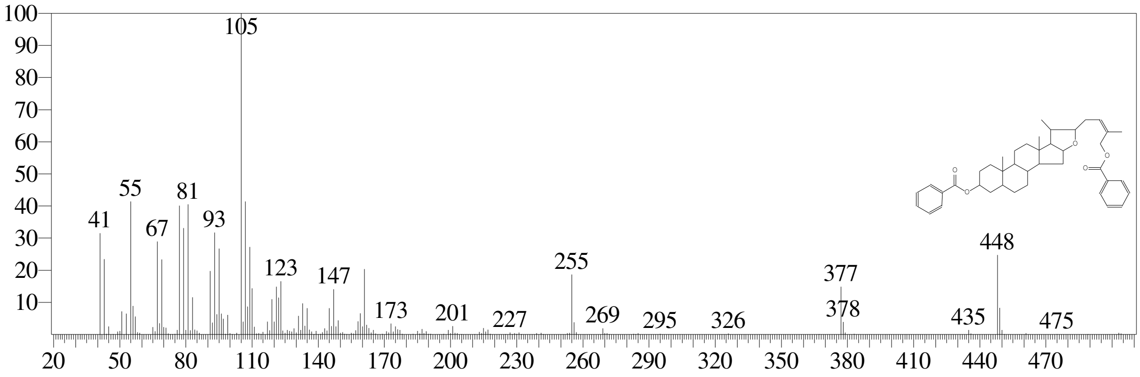

Hit#:2 Entry:146620 Library:NIST23-1.lib
SI:73 Formula:C20H32 CAS:0-00-0 MolWeight:272 RetIndex:1990
CompName:geranyl-.alpha.-terpinene

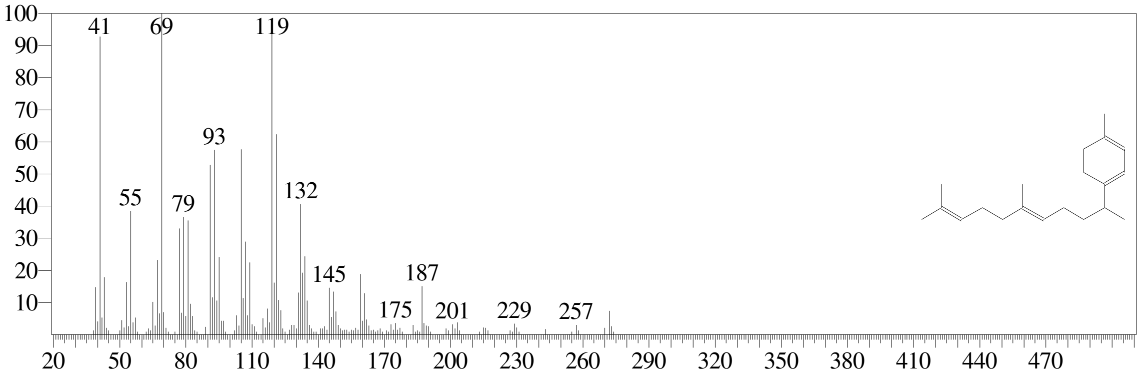

<< Target >>
Line#:25 R.Time:34.400(Scan#:3829) MassPeaks:18
RawMode:Averaged 34.392-34.408(3828-3830) BasePeak:119.10(2288)
BG Mode:Calc. from Peak Group 1 - Event 1 Scan

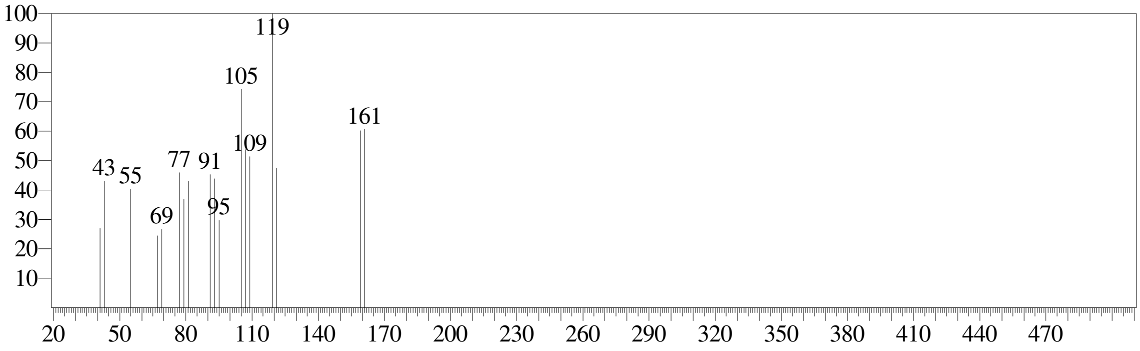

Hit#:3 Entry:62766 Library:NIST23-1.lib
SI:73 Formula:C15H24 CAS:0-00-0 MolWeight:204 RetIndex:1316
CompName:Bicyclo[4.1.0]-3-heptene, 2-isopropenyl-5-isopropyl-7,7-dimethyl- $$ 2-Isopropenyl-5-isopropyl-7,7-dimethy

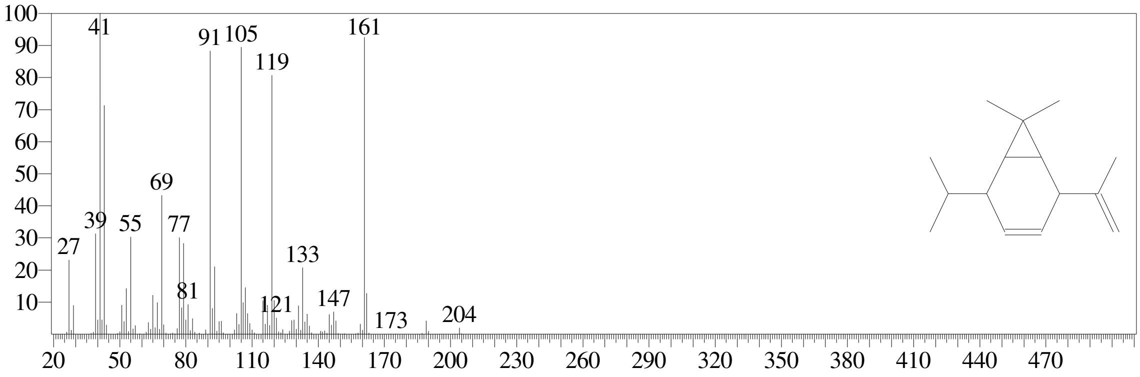

Hit#:4 Entry:83606 Library:NIST23-1.lib
SI:72 Formula:C15H26O CAS:38230-60-3 MolWeight:222 RetIndex:1562
CompName:(3R,3aR,3bR,4S,7R,7aR)-4-Isopropyl-3,7-dimethyloctahydro-1H-cyclopenta[1,3]cyclopropa[1,2]benzen-3-ol

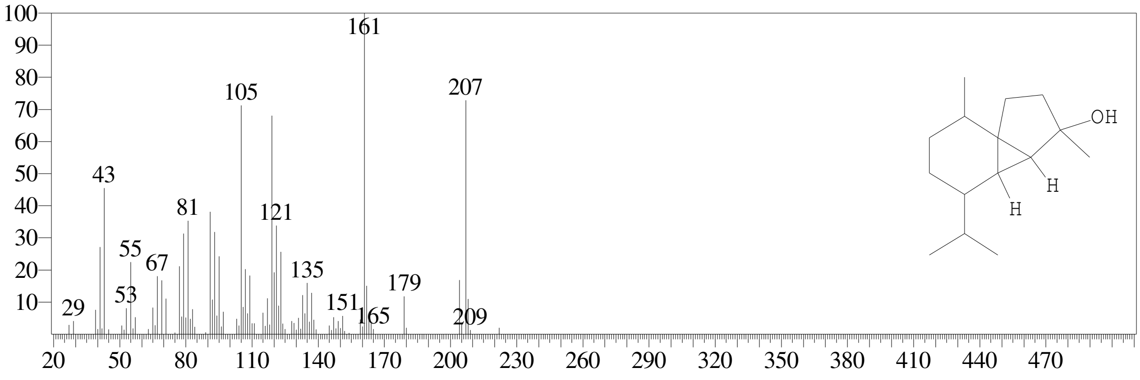

<< Target >>
Line#:25   R.Time:34.400(Scan#:3829) MassPeaks:18
RawMode:Averaged 34.392-34.408(3828-3830) BasePeak:119.10(2288)
BG Mode:Calc. from Peak Group 1 - Event 1 Scan

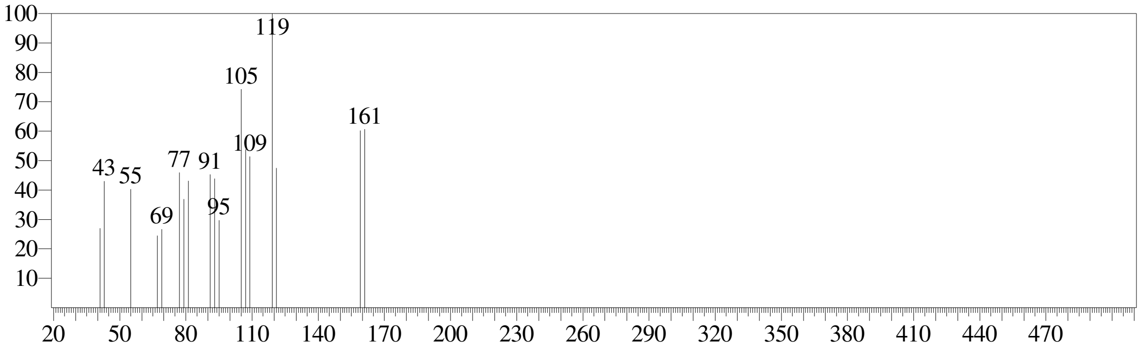

Hit#:5 Entry:128065 Library:NIST23-1.lib
SI:72 Formula:C18H26O CAS:17974-57-1 MolWeight:258 RetIndex:2119
CompName:(3E,5E,7E)-6-Methyl-8-(2,6,6-trimethyl-1-cyclohexenyl)-3,5,7-octatrien-2-one $$ (3E,5E,7E)-6-Methyl-8-(2,

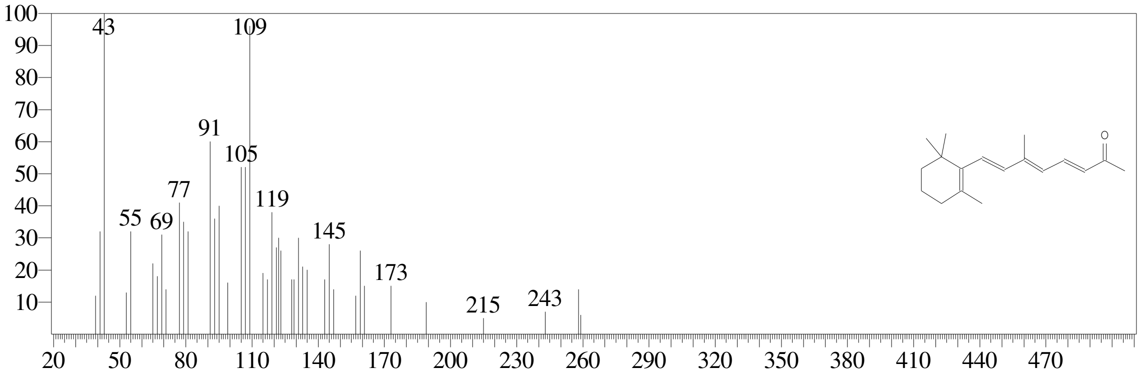

Supplement: Supplementary file 1 [file plants-15-01406-s001.zip › EBbb.pdf]
