# Supplementary material for: Unveiling Chemical Profile and Insecticidal Potential of Essential Oils from Leaves of Seven Eugenia L. Species (Myrtaceae)
Source: Plants (Basel). 2026 May 5;15(9):1406. doi: 10.3390/plants15091406 (PMC13165059; doi:10.3390/plants15091406)

UNIVERSIDADE FERAL RURAL DO RIO DE JANEIRO
INSTITUTO DE QUÍMICA

CGMS

Analyzed by: Cristiane Cardoso
Analyzed: 17/6/2025
Solicitante:Douglas
Sample Name: EI
Injection Volume: 1,0 uL      Solvente: Diclorometano
Data File: C:\GCMSsolution\Data\Project1\Douglas\2025\MLLENA\170625\EI.qgd
Method File: C:\GCMSsolution\Data\Project1\Douglas\Essencial Adams-Inj.qgm
EQUIPAMENTO: Modelo:GCMS-QP2010 Plus ( Shimadzu)
Coluna: VF-5m (30X0.25X0.25)

Chromatogram EI C:\GCMSsolution\Data\Project1\Douglas\2025\MLLENA\170625\EI.qgd

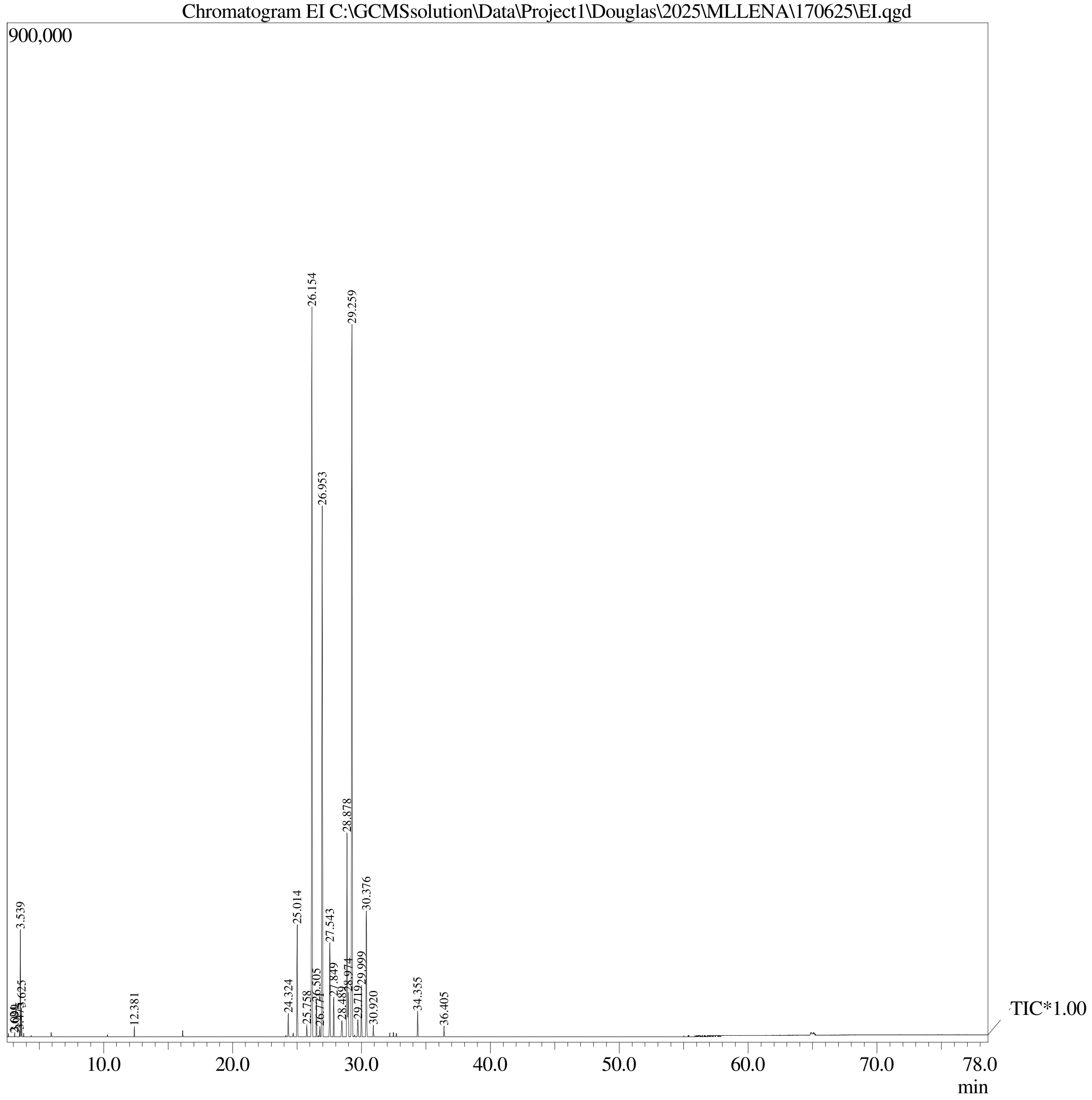

Library

<< Target >>
Line#:1   R.Time:2.617(Scan#:15)  MassPeaks:2
RawMode:Averaged 2.608-2.625(14-16) BasePeak:59.00(1553)
BG Mode:None  Group 1 - Event 1  Scan

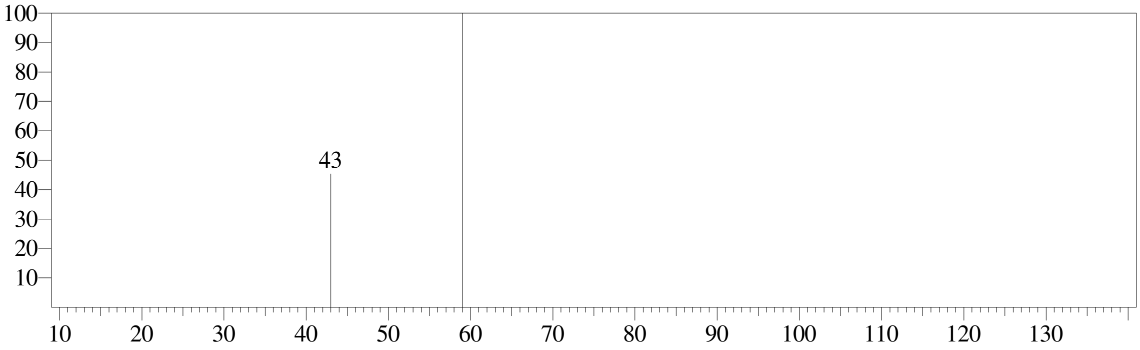


Hit#:1  Entry:2768  Library:NIST23s.lib
SI:93  Formula:C4H8O3  CAS:594-61-6  MolWeight:104  RetIndex:932
CompName:Propanoic acid, 2-hydroxy-2-methyl- $$ 2-Hydroxyisobutyric acid $$ Lactic acid, 2-methyl- $$ .alpha.-Hydro

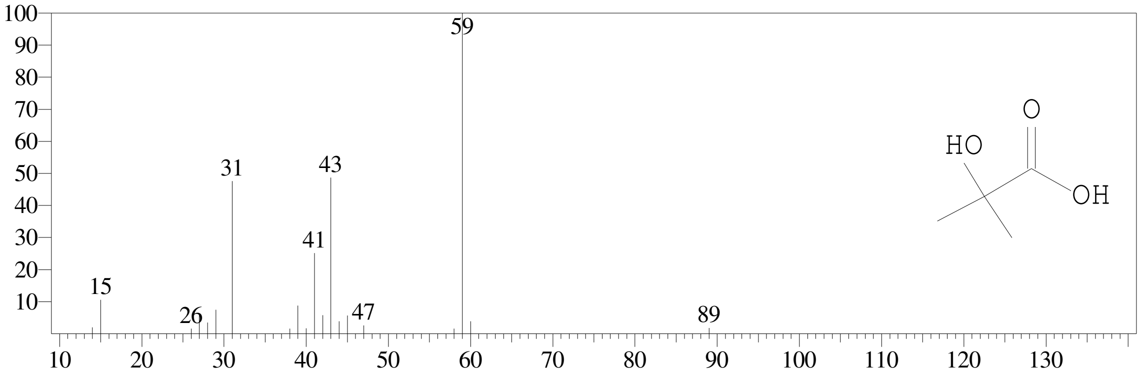


Hit#:2  Entry:2534  Library:NIST23s.lib
SI:93  Formula:C5H10O2  CAS:115-22-0  MolWeight:102  RetIndex:737
CompName:3-Hydroxy-3-methyl-2-butanone $$ 2-Butanone, 3-hydroxy-3-methyl- $$ Dimethylacetylcarbinol $$ 3-Hydro

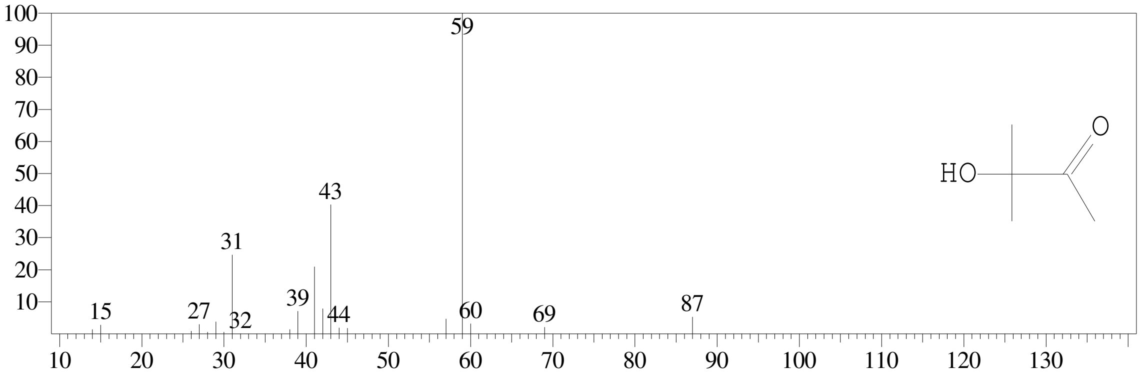

<< Target >>
Line#:1   R.Time:2.617(Scan#:15)  MassPeaks:2
RawMode:Averaged 2.608-2.625(14-16) BasePeak:59.00(1553)
BG Mode:None  Group 1 - Event 1  Scan

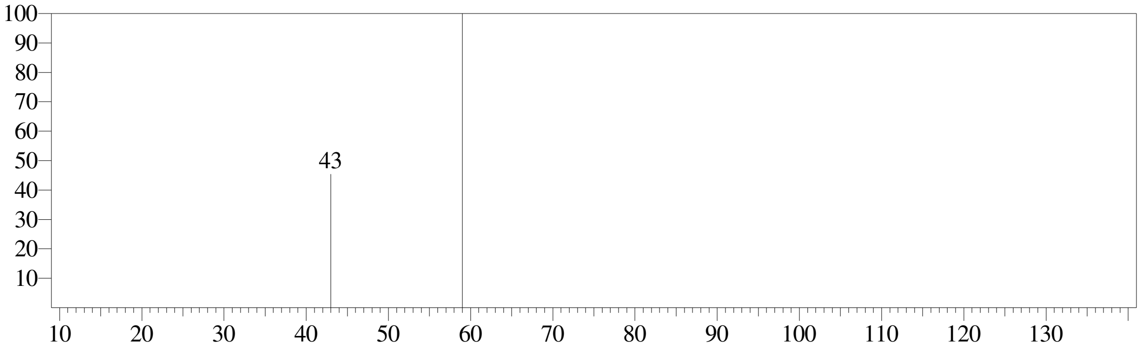


Hit#:3  Entry:2726  Library:NIST23-1.lib
SI:93  Formula:C4H8O3  CAS:594-61-6  MolWeight:104  RetIndex:932
CompName:Propanoic acid, 2-hydroxy-2-methyl- $$ 2-Hydroxyisobutyric acid $$ Lactic acid, 2-methyl- $$ .alpha.-Hydro

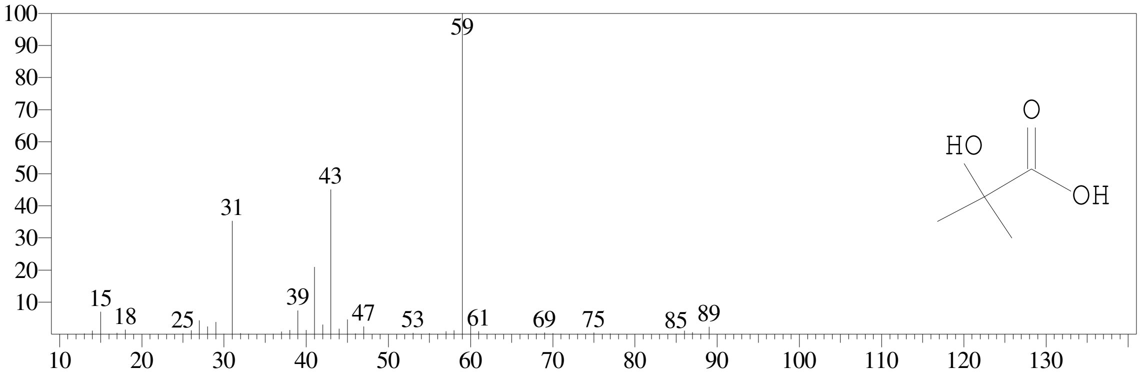


Hit#:4  Entry:9783  Library:NIST23-1.lib
SI:93  Formula:C6H12O3  CAS:70657-70-4  MolWeight:132  RetIndex:880
CompName:2-Methoxypropyl acetate $$ 1-Propanol, 2-methoxy-, 1-acetate $$ 1-Propanol, 2-methoxy-, acetate $$ 2-Meth

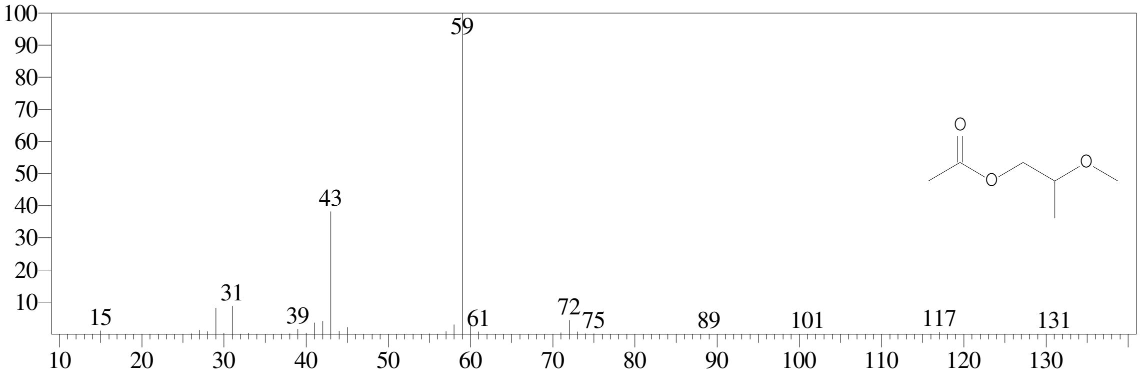

<< Target >>
Line#:1 R.Time:2.617(Scan#:15) MassPeaks:2
RawMode:Averaged 2.608-2.625(14-16) BasePeak:59.00(1553)
BG Mode:None Group 1 - Event 1 Scan

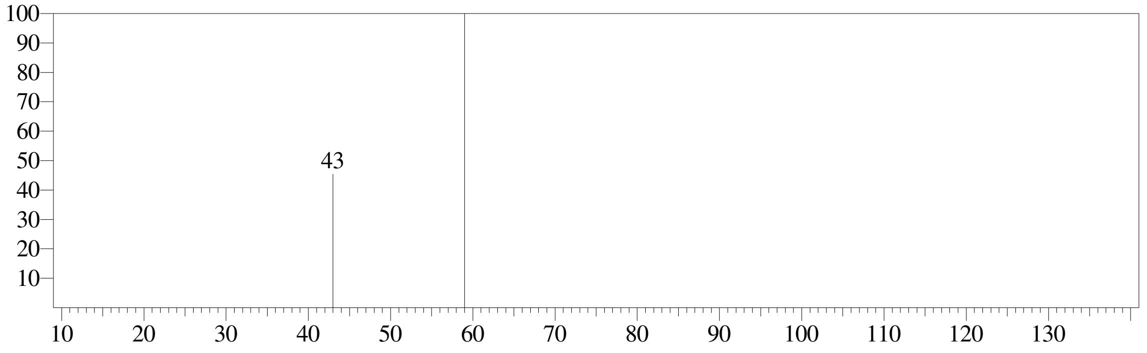


Hit#:5 Entry:4803 Library:NIST23s.lib
SI:92 Formula:C5H10O3 CAS:2110-78-3 MolWeight:118 RetIndex:769
CompName:Propanoic acid, 2-hydroxy-2-methyl-, methyl ester $$ Methyl .alpha.-hydroxyisobutyrate $$ Lactic acid, 2-me

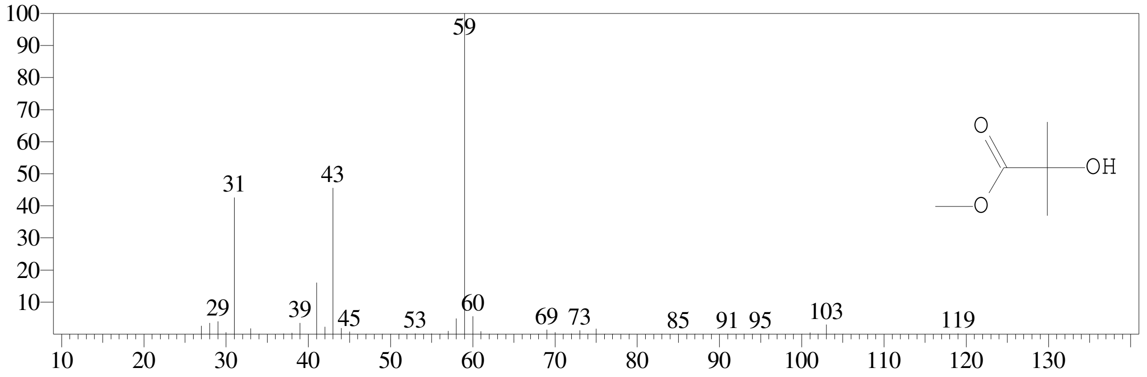

<< Target >>
Line#:2   R.Time:3.100(Scan#:73)  MassPeaks:2
RawMode:Averaged 3.092-3.108(72-74) BasePeak:43.00(1922)
BG Mode:None  Group 1 - Event 1  Scan

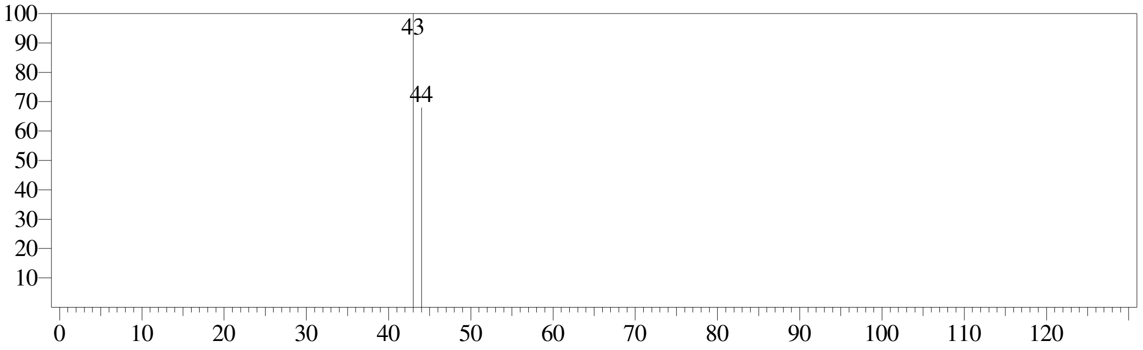


Hit#:1  Entry:1019  Library:NIST23s.lib
SI:95  Formula:C4H6O2  CAS:3068-88-0  MolWeight:86  RetIndex:724
CompName:2-Oxetanone, 4-methyl- $$ .beta.-Butyrolactone $$ .beta.-Butyrolakton $$ 3-Hydroxybutanoic acid, .beta.-lac

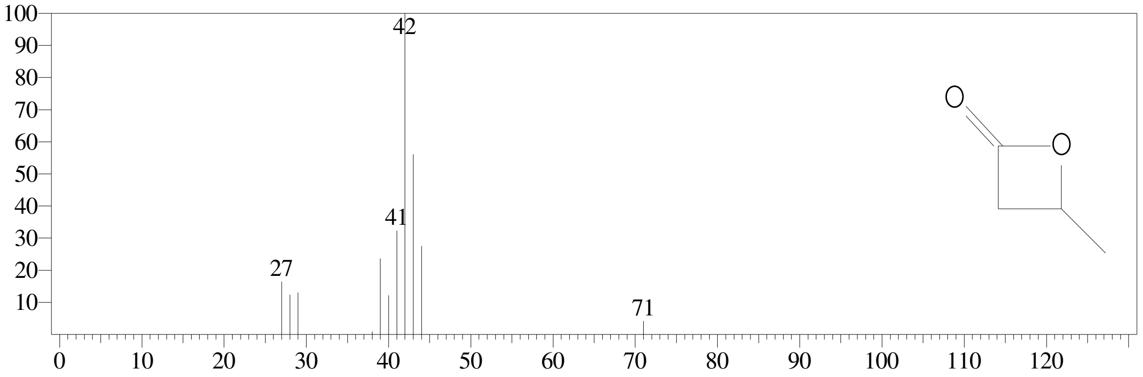


Hit#:2  Entry:21730  Library:NIST23-1.lib
SI:94  Formula:C4H4N4O3  CAS:0-00-0  MolWeight:156  RetIndex:1987
CompName:Pyrimidine-2,4(1H,3H)-dione, 5-amino-6-nitroso- $$ 5-Amino-6-nitroso-2,4(1H,3H)-pyrimidinedione  # $$

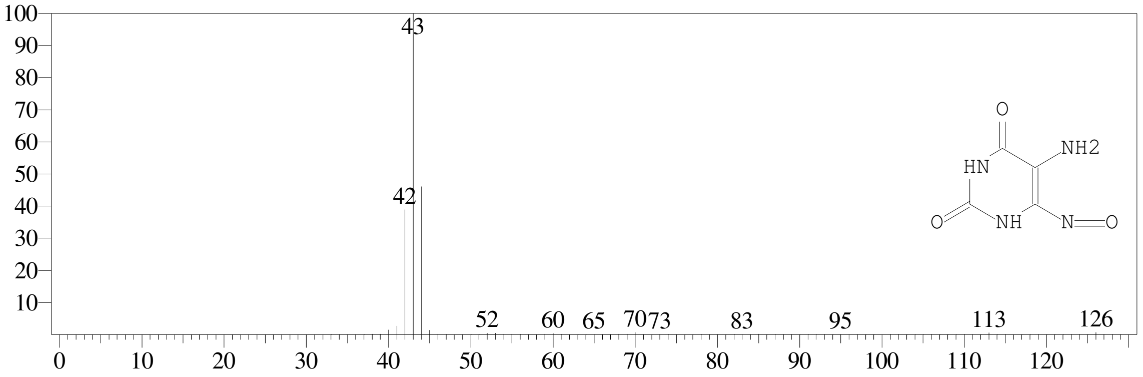

<< Target >>
Line#:2   R.Time:3.100(Scan#:73)  MassPeaks:2
RawMode:Averaged 3.092-3.108(72-74) BasePeak:43.00(1922)
BG Mode:None  Group 1 - Event 1  Scan

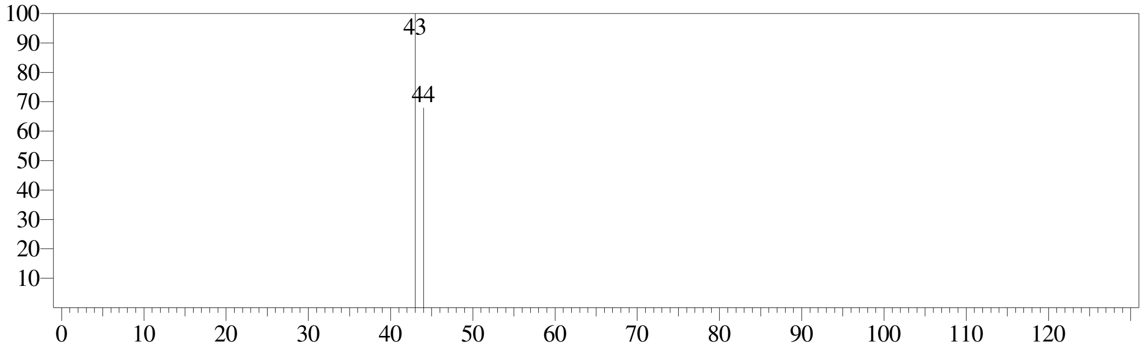


Hit#:3  Entry:45  Library:NIST23s.lib
SI:93  Formula:C3H8  CAS:74-98-6  MolWeight:44  RetIndex:293
CompName:Propane $$ n-Propane $$ Dimethylmethane $$ Freon 290 $$ Liquefied petroleum gas $$ LPG $$ Propyl hyd

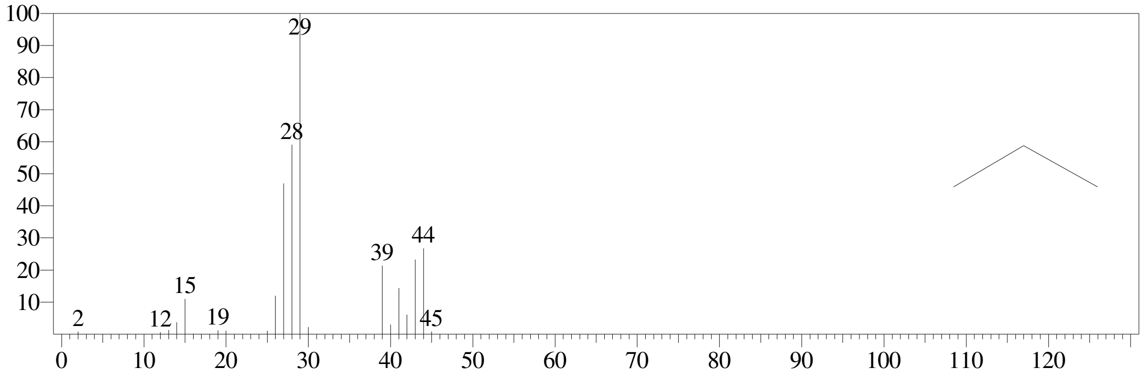


Hit#:4  Entry:48  Library:NIST23-1.lib
SI:92  Formula:C3H8  CAS:74-98-6  MolWeight:44  RetIndex:293
CompName:Propane $$ n-Propane $$ Dimethylmethane $$ Freon 290 $$ Liquefied petroleum gas $$ LPG $$ Propyl hyd

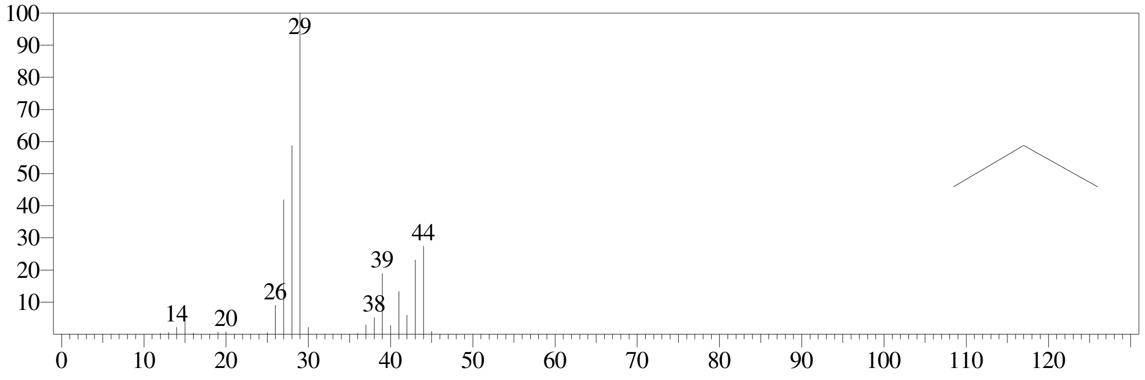

<< Target >>
Line#:2   R.Time:3.100(Scan#:73)  MassPeaks:2
RawMode:Averaged 3.092-3.108(72-74) BasePeak:43.00(1922)
BG Mode:None  Group 1 - Event 1  Scan

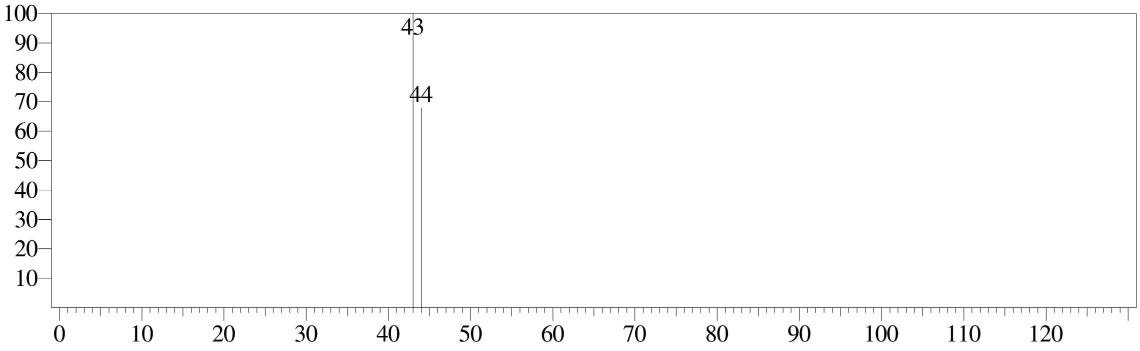


Hit#:5  Entry:44  Library:NIST23s.lib
SI:92  Formula:C3H8  CAS:74-98-6  MolWeight:44  RetIndex:293
CompName:Propane $$ n-Propane $$ Dimethylmethane $$ Freon 290 $$ Liquefied petroleum gas $$ LPG $$ Propyl hyd

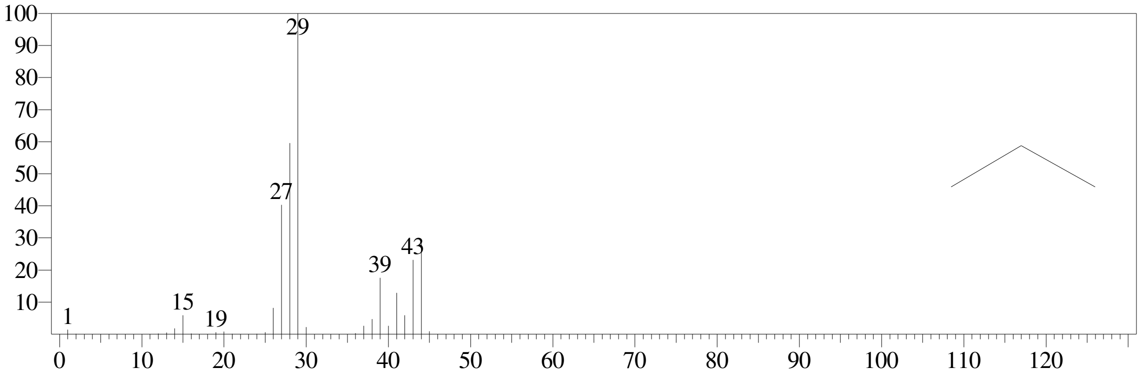

<< Target >>
Line#:3   R.Time:3.475(Scan#:118)  MassPeaks:2
RawMode:Averaged 3.467-3.483(117-119) BasePeak:59.05(3341)
BG Mode:None  Group 1 - Event 1  Scan

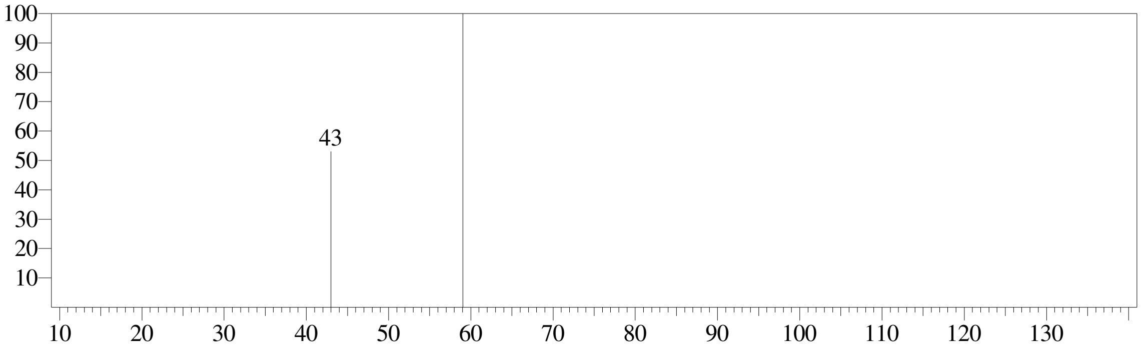


Hit#:1  Entry:2768  Library:NIST23s.lib
SI:93  Formula:C4H8O3  CAS:594-61-6  MolWeight:104  RetIndex:932
CompName:Propanoic acid, 2-hydroxy-2-methyl- $$ 2-Hydroxyisobutyric acid $$ Lactic acid, 2-methyl- $$ .alpha.-Hydro

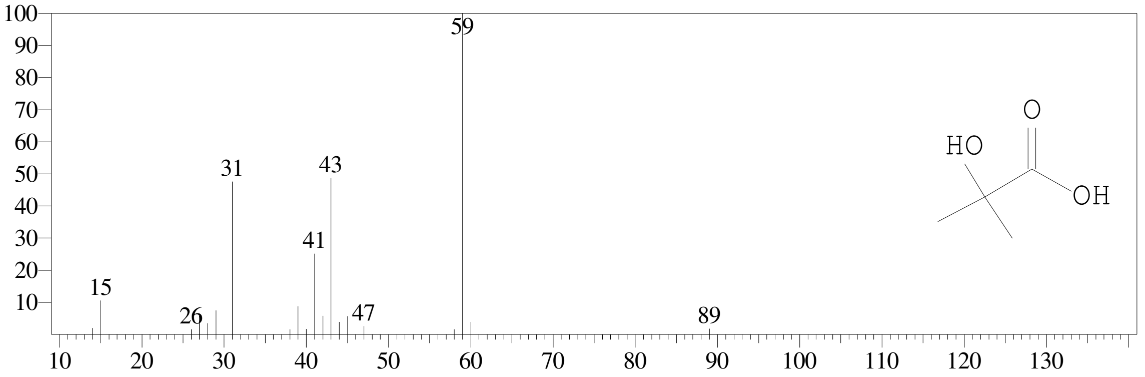


Hit#:2  Entry:152  Library:NIST23s.lib
SI:92  Formula:CH5N3  CAS:113-00-8  MolWeight:59  RetIndex:1188
CompName:Guanidine $$ Aminoformamidine $$ Aminomethanamidine $$ Carbamamidine $$ Carbamidine $$ Guanidin

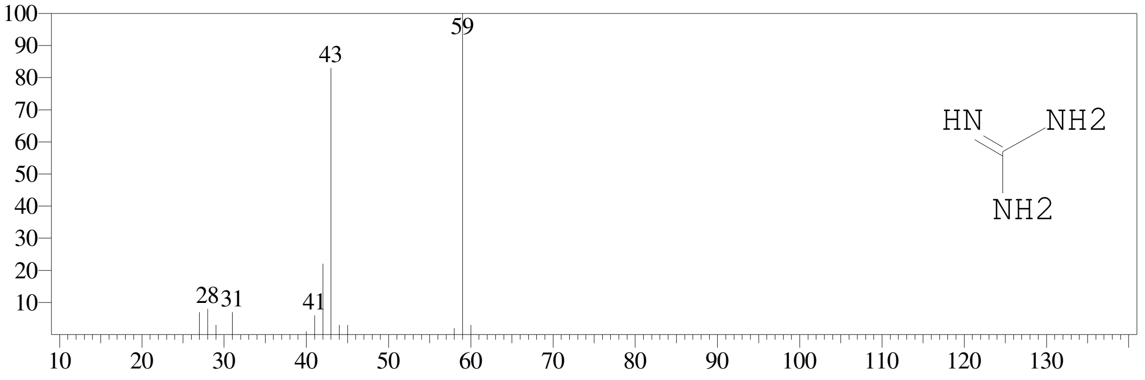

<< Target >>
Line#:3   R.Time:3.475(Scan#:118)  MassPeaks:2
RawMode:Averaged 3.467-3.483(117-119) BasePeak:59.05(3341)
BG Mode:None  Group 1 - Event 1  Scan

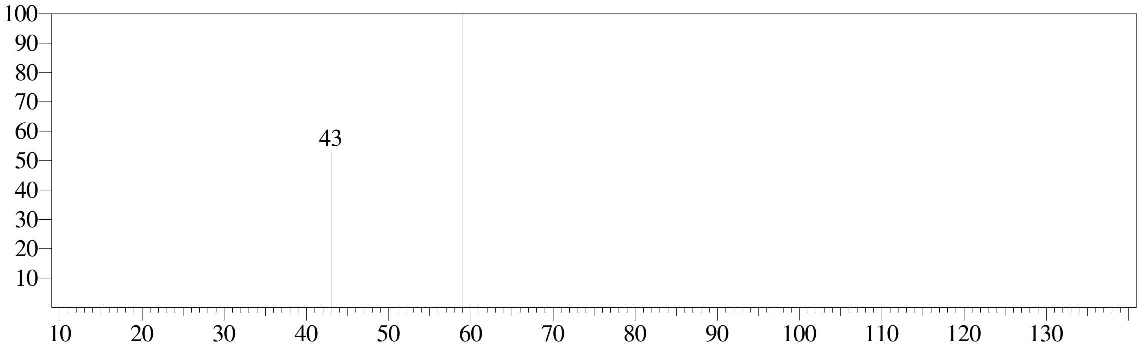


Hit#:3  Entry:2213  Library:NIST23-1.lib
SI:92  Formula:C6H12O  CAS:624-97-5  MolWeight:100  RetIndex:702
CompName:4-Pentene-2-ol, 2-methyl $$ CH2=CHCH2C(CH3)2OH $$ 1-Pentene-4-ol, 4-methyl $$ 4-Penten-2-ol, 2-met

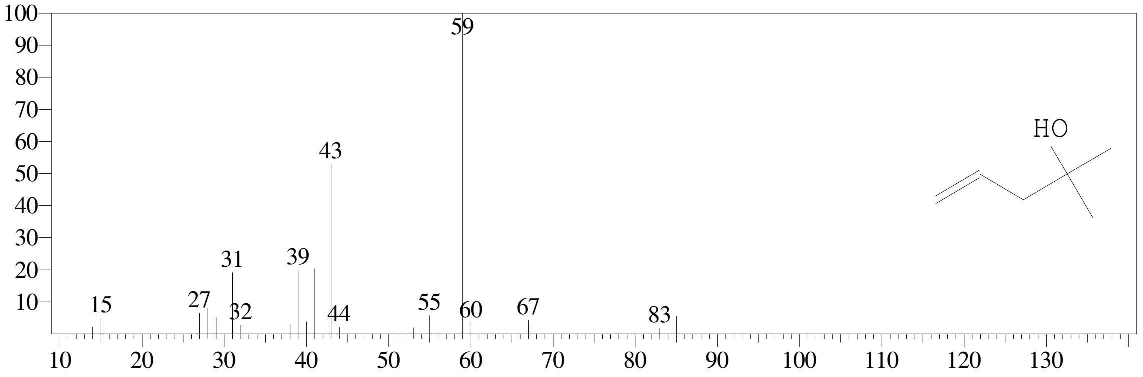


Hit#:4  Entry:9784  Library:NIST23-1.lib
SI:92  Formula:C6H12O3  CAS:80-55-7  MolWeight:132  RetIndex:843
CompName:Propanoic acid, 2-hydroxy-2-methyl-, ethyl ester $$ Lactic acid, 2-methyl-, ethyl ester $$ Ethyl .alpha.-hydro:

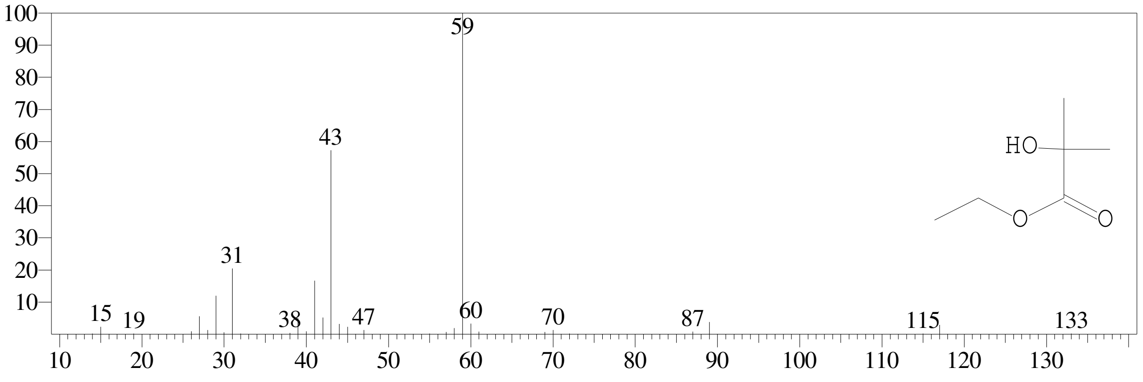

<< Target >>
Line#:3 R.Time:3.475(Scan#:118) MassPeaks:2
RawMode:Averaged 3.467-3.483(117-119) BasePeak:59.05(3341)
BG Mode:None Group 1 - Event 1 Scan

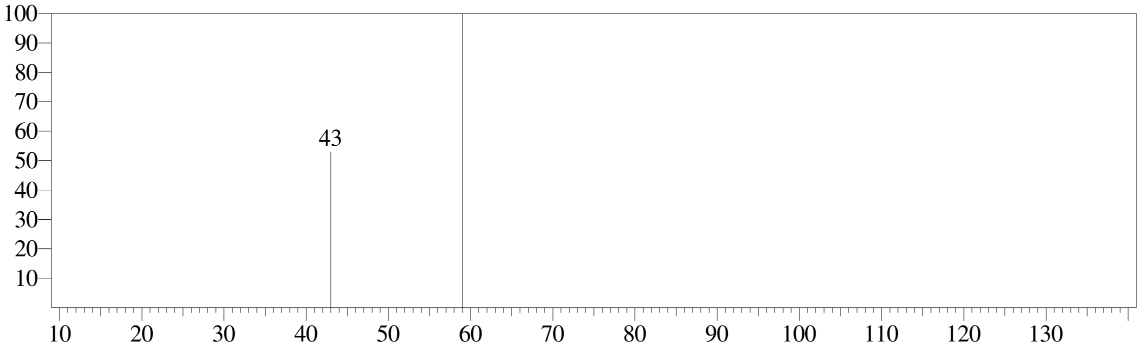


Hit#:5 Entry:121 Library:NIST23-1.lib
SI:92 Formula:CH5N3 CAS:113-00-8 MolWeight:59 RetIndex:1188
CompName:Guanidine $$ Aminoformamidine $$ Aminomethanamidine $$ Carbamamidine $$ Carbamidine $$ Guanidin

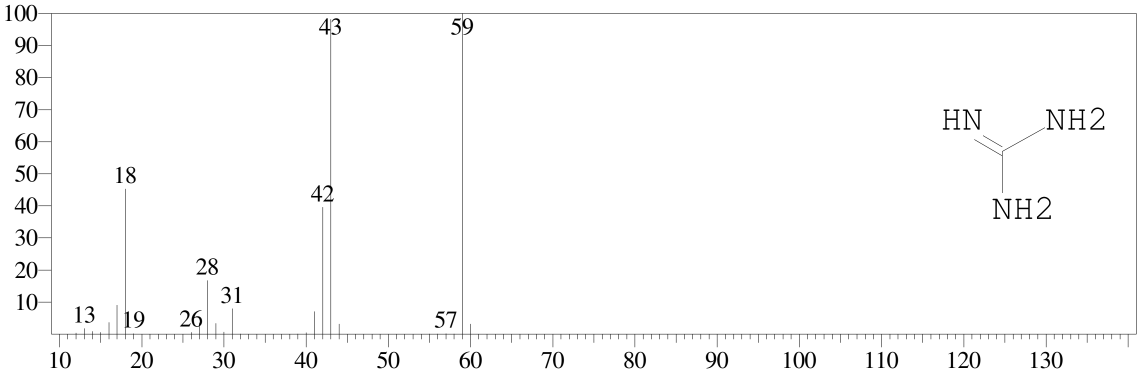

<< Target >>
Line#:4   R.Time:3.542(Scan#:126)  MassPeaks:11
RawMode:Averaged 3.533-3.550(125-127) BasePeak:45.00(51026)
BG Mode:None  Group 1 - Event 1  Scan

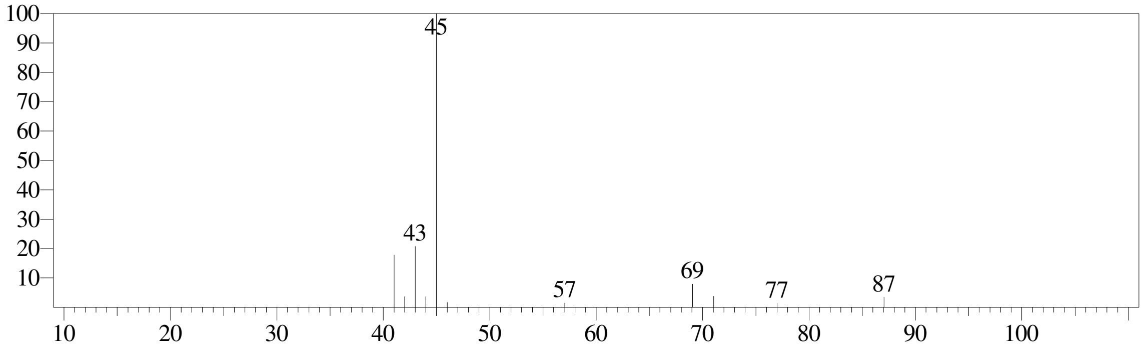


Hit#:1  Entry:2533  Library:NIST23-1.lib
SI:93  Formula:C6H14O  CAS:52019-78-0  MolWeight:102  RetIndex:791
CompName:2-Hexanol, (S)- $$ (S)-(+)-2-Hexanol $$ 2-Hexanol  # $$

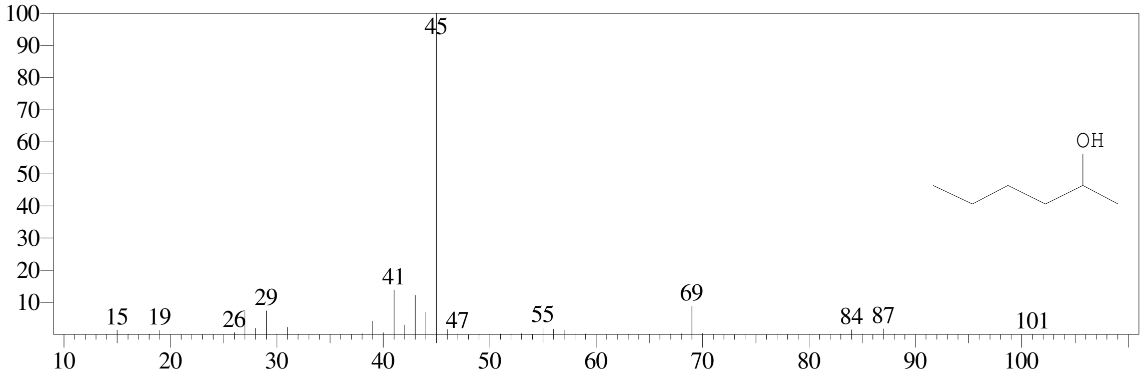


Hit#:2  Entry:2534  Library:NIST23-1.lib
SI:93  Formula:C6H14O  CAS:26549-24-6  MolWeight:102  RetIndex:791
CompName:2-Hexanol, (R)- $$ (R)-(-)-2-Hexanol $$ 2-Hexanol  # $$

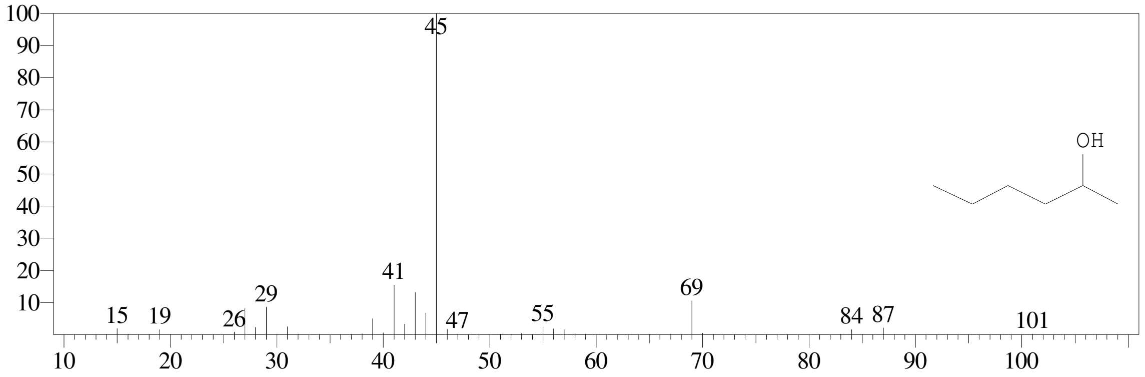

<< Target >>
Line#:4   R.Time:3.542(Scan#:126)  MassPeaks:11
RawMode:Averaged 3.533-3.550(125-127) BasePeak:45.00(51026)
BG Mode:None  Group 1 - Event 1  Scan

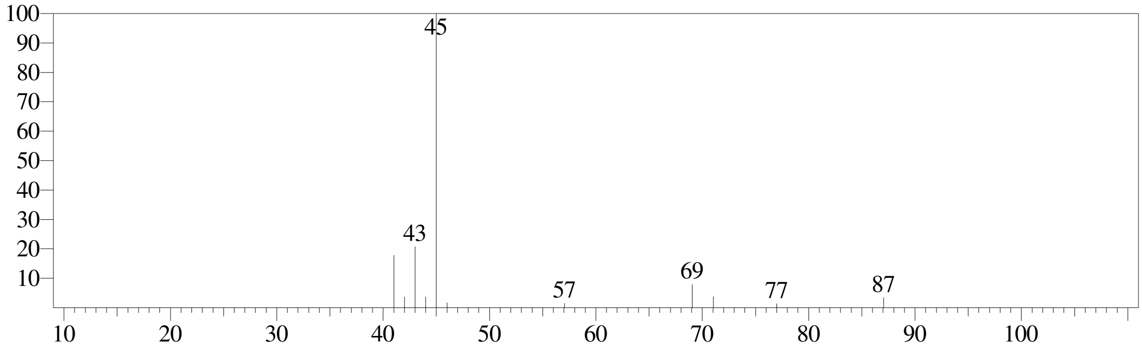


Hit#:3  Entry:2597  Library:NIST23s.lib
SI:93  Formula:C6H14O  CAS:108-11-2  MolWeight:102  RetIndex:752
CompName:2-Pentanol, 4-methyl- $$ Isobutylmethylcarbinol $$ Isobutylmethylmethanol $$ Methylisobutylcarbinol $$ M

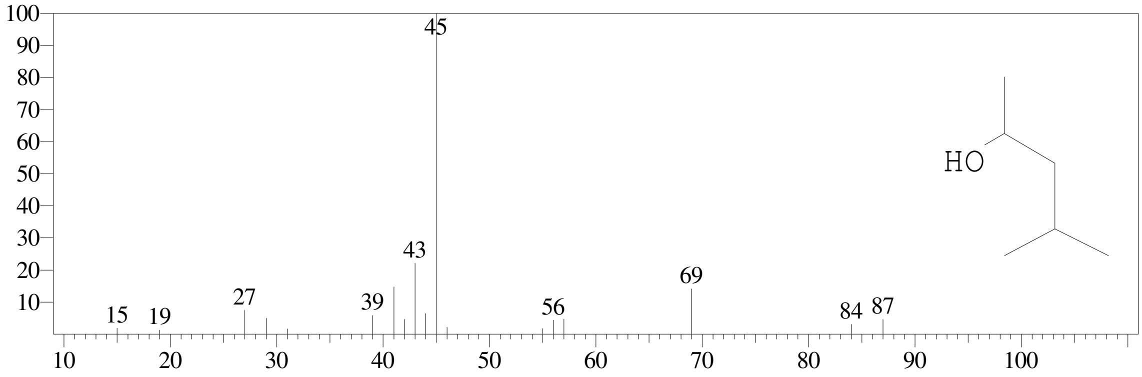


Hit#:4  Entry:1082  Library:NIST23s.lib
SI:93  Formula:C5H10O  CAS:625-31-0  MolWeight:86  RetIndex:658
CompName:4-Penten-2-ol $$ 1-Penten-4-ol $$ 4-Hydroxypent-1-ene $$ CH2=CHCH2CH(OH)CH3 $$

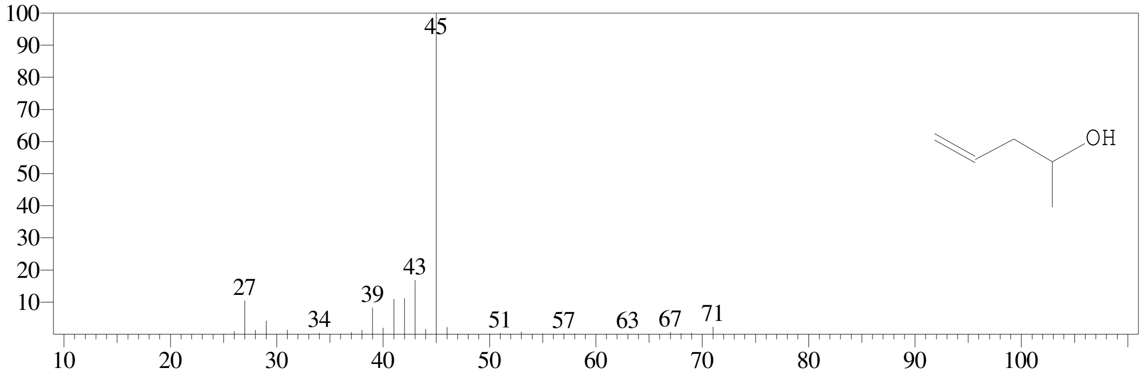

<< Target >>
Line#:4   R.Time:3.542(Scan#:126)  MassPeaks:11
RawMode:Averaged 3.533-3.550(125-127) BasePeak:45.00(51026)
BG Mode:None  Group 1 - Event 1  Scan

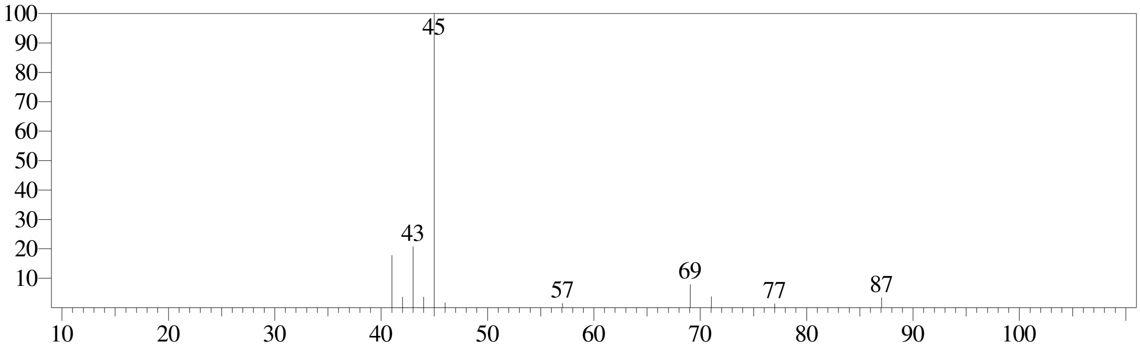


Hit#:5  Entry:2596  Library:NIST23s.lib
SI:92  Formula:C6H14O  CAS:626-93-7  MolWeight:102  RetIndex:791
CompName:2-Hexanol $$ n-C4H9CH(OH)CH3 $$ n-Butylmethylcarbinol $$ Hexanol-(2) $$ sec-Hexyl alcohol $$ n-Hex

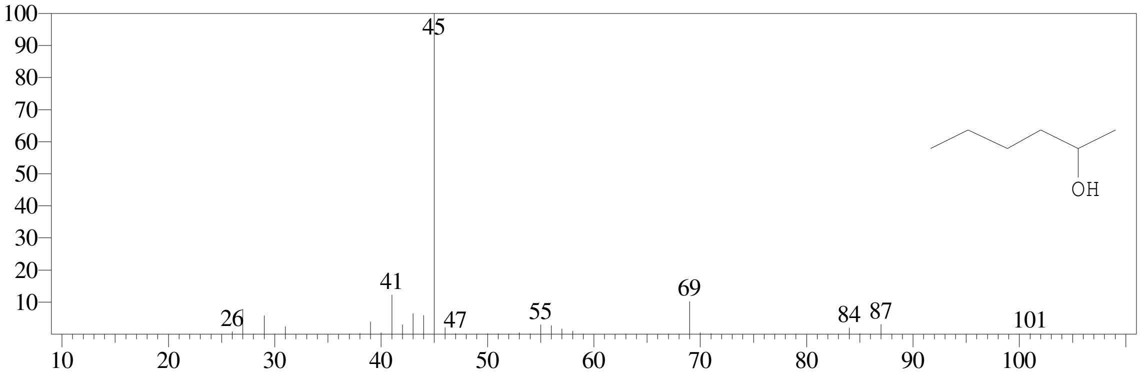

<< Target >>
Line#:5   R.Time:3.625(Scan#:136)  MassPeaks:5
RawMode:Averaged 3.617-3.633(135-137) BasePeak:59.00(12488)
BG Mode:None  Group 1 - Event 1  Scan

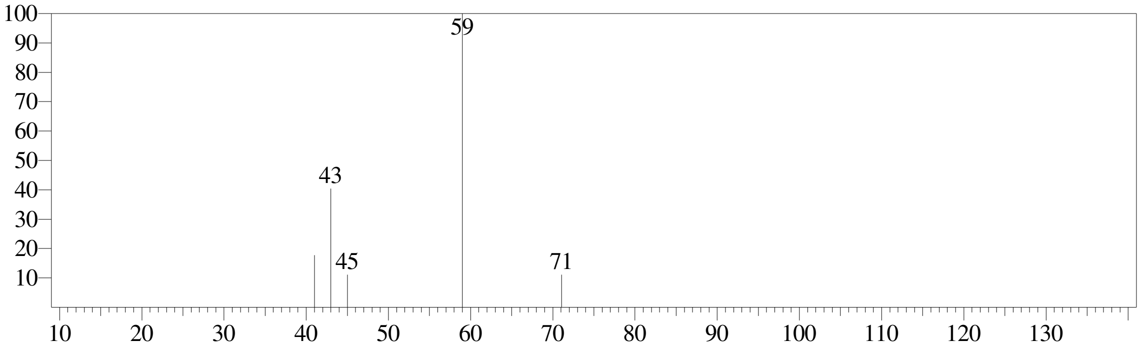


Hit#:1  Entry:2726  Library:NIST23-1.lib
SI:91  Formula:C4H8O3  CAS:594-61-6  MolWeight:104  RetIndex:932
CompName:Propanoic acid, 2-hydroxy-2-methyl- $$ 2-Hydroxyisobutyric acid $$ Lactic acid, 2-methyl- $$ .alpha.-Hydrc

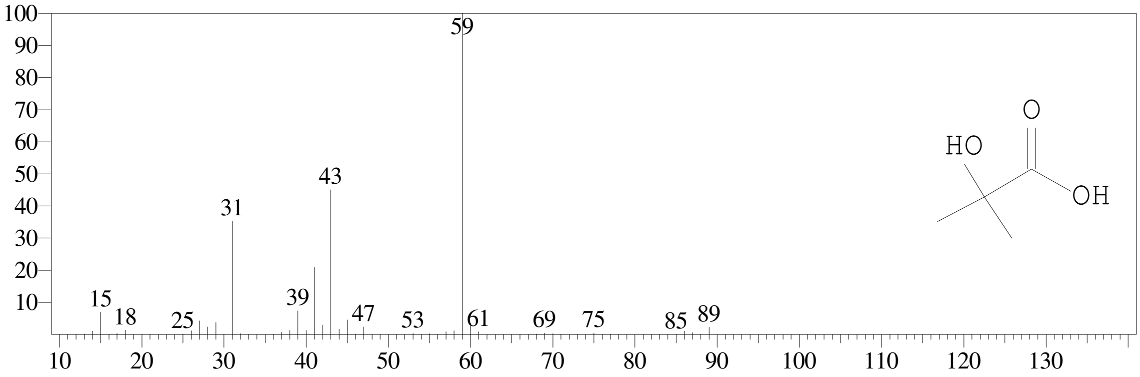


Hit#:2  Entry:2466  Library:NIST23-1.lib
SI:90  Formula:C5H10O2  CAS:115-22-0  MolWeight:102  RetIndex:737
CompName:3-Hydroxy-3-methyl-2-butanone $$ 2-Butanone, 3-hydroxy-3-methyl- $$ Dimethylacetylcarbinol $$ 3-Hydrc

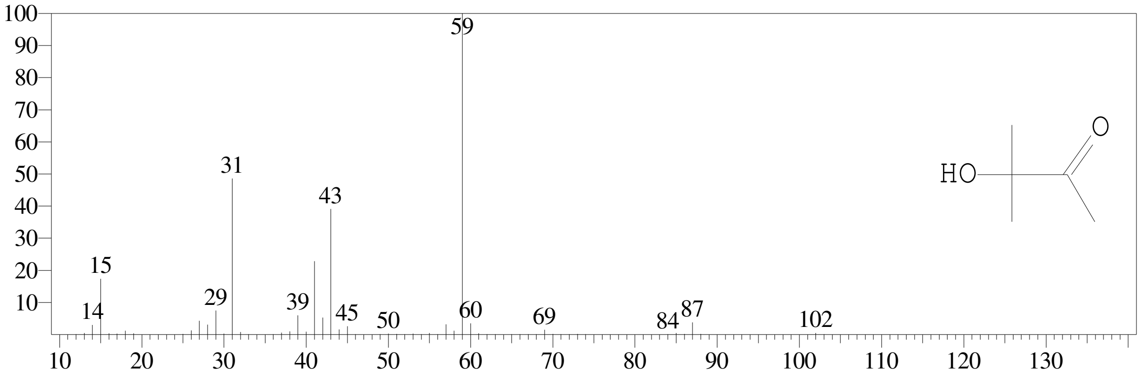

<< Target >>
Line#:5   R.Time:3.625(Scan#:136)  MassPeaks:5
RawMode:Averaged 3.617-3.633(135-137) BasePeak:59.00(12488)
BG Mode:None  Group 1 - Event 1  Scan

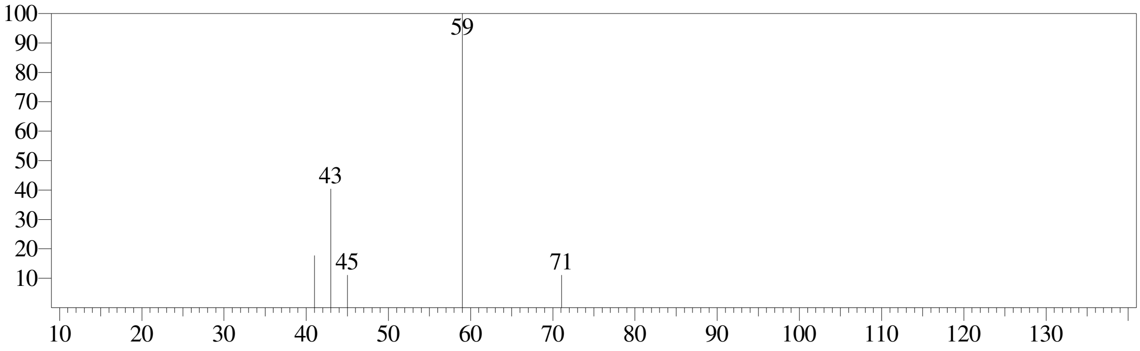


Hit#:3  Entry:2775  Library:NIST23-1.lib
SI:89  Formula:C5H12O2  CAS:5396-58-7  MolWeight:104  RetIndex:812
CompName:2-Methyl-2,3-butanediol $$ 2-Methylbutane-2,3-diol $$ 2,3-Dihydroxy-2-methylbutane $$

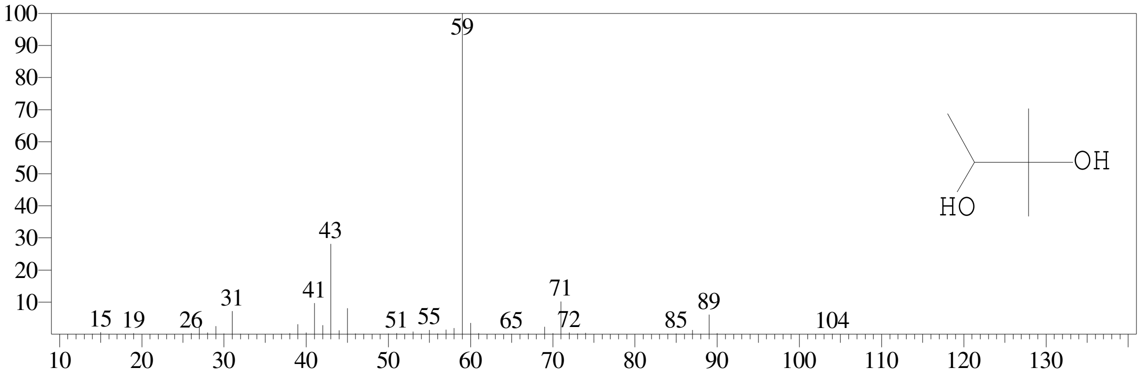


Hit#:4  Entry:9783  Library:NIST23-1.lib
SI:89  Formula:C6H12O3  CAS:70657-70-4  MolWeight:132  RetIndex:880
CompName:2-Methoxypropyl acetate $$ 1-Propanol, 2-methoxy-, 1-acetate $$ 1-Propanol, 2-methoxy-, acetate $$ 2-Meth

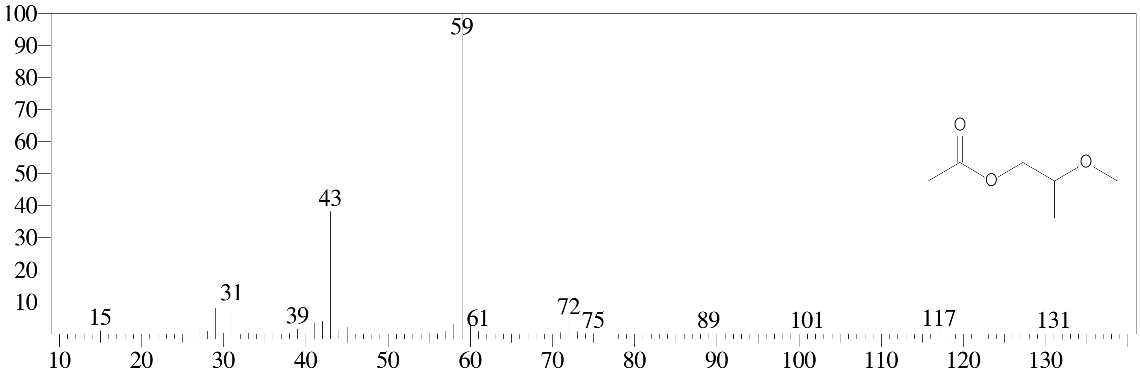

<< Target >>
Line#:5   R.Time:3.625(Scan#:136)  MassPeaks:5
RawMode:Averaged 3.617-3.633(135-137) BasePeak:59.00(12488)
BG Mode:None  Group 1 - Event 1  Scan

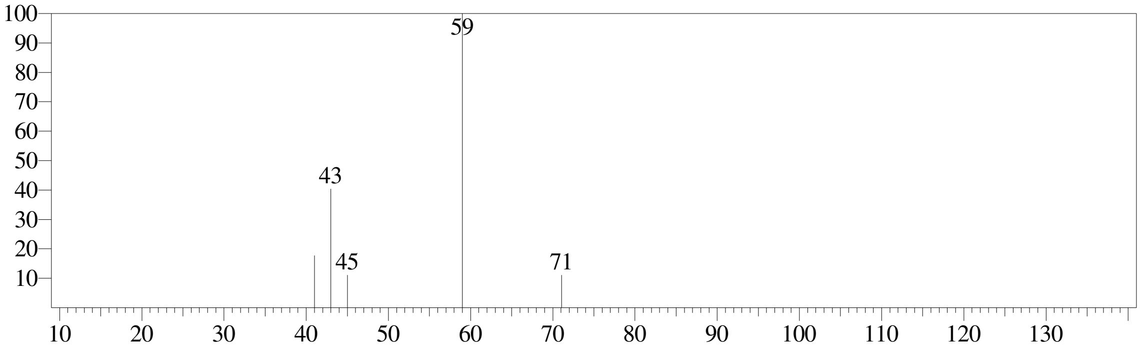


Hit#:5  Entry:2768  Library:NIST23s.lib
SI:89  Formula:C4H8O3  CAS:594-61-6  MolWeight:104  RetIndex:932
CompName:Propanoic acid, 2-hydroxy-2-methyl- $$ 2-Hydroxyisobutyric acid $$ Lactic acid, 2-methyl- $$ .alpha.-Hydrc

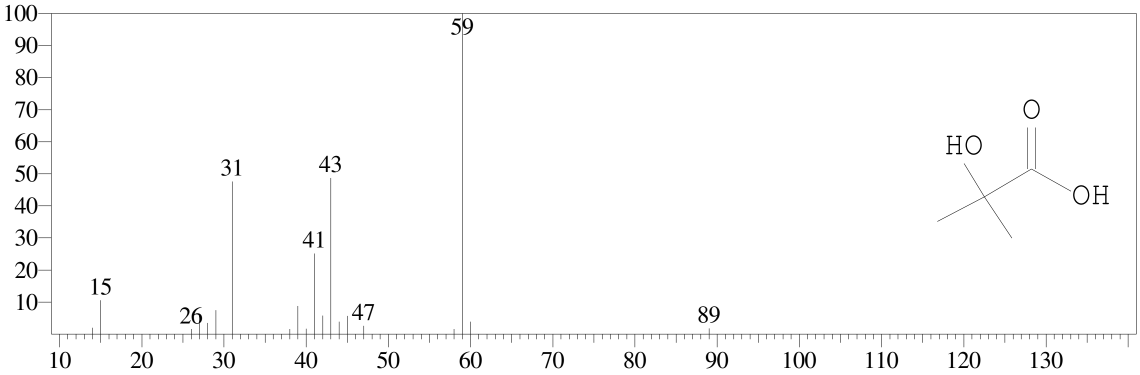

<< Target >>
Line#:6 R.Time:12.383(Scan#:1187) MassPeaks:6
RawMode:Averaged 12.375-12.392(1186-1188) BasePeak:71.05(1705)
BG Mode:None Group 1 - Event 1 Scan

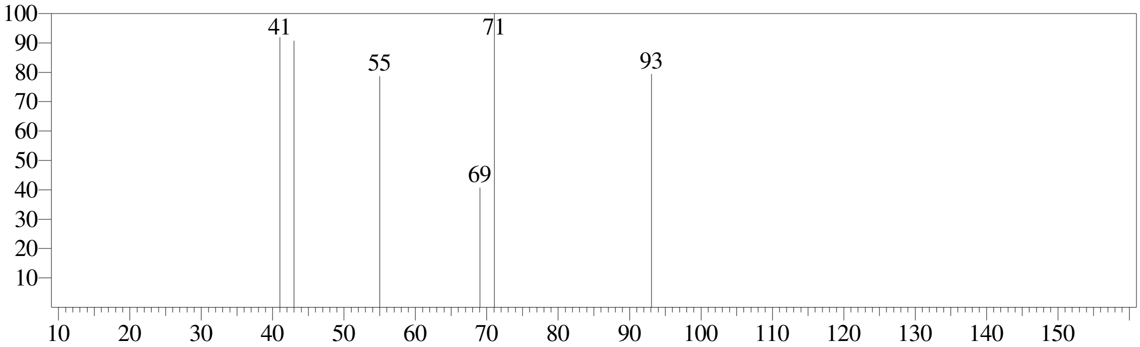


Hit#:1 Entry:17853 Library:NIST23-1.lib
SI:79 Formula:C5H11Br CAS:1809-10-5 MolWeight:150 RetIndex:827
CompName:Pentane, 3-bromo- $$ 3-Bromopentane $$ 3-Pentyl bromide $$

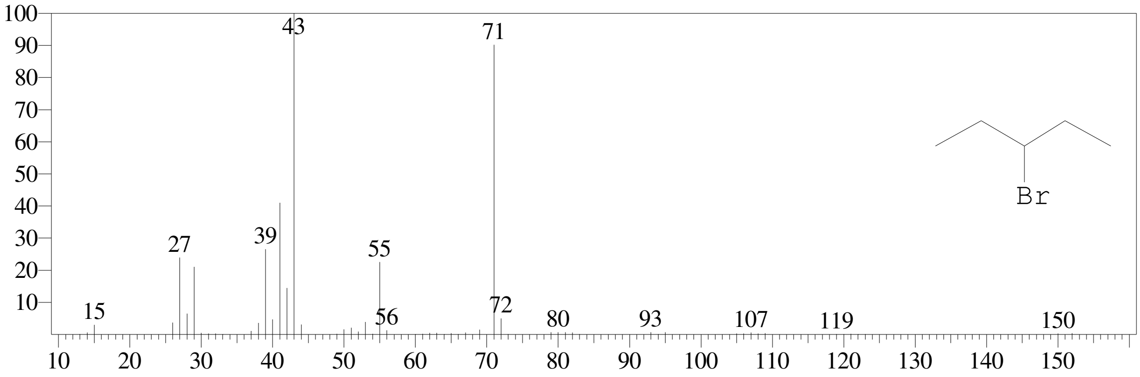


Hit#:2 Entry:11279 Library:NIST23s.lib
SI:79 Formula:C5H11Br CAS:1809-10-5 MolWeight:150 RetIndex:827
CompName:Pentane, 3-bromo- $$ 3-Bromopentane $$ 3-Pentyl bromide $$

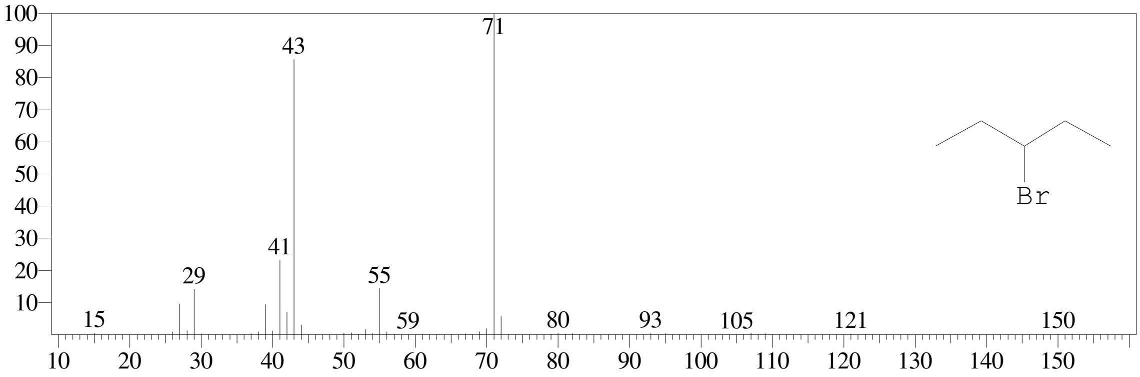

<< Target >>
Line#:6 R.Time:12.383(Scan#:1187) MassPeaks:6
RawMode:Averaged 12.375-12.392(1186-1188) BasePeak:71.05(1705)
BG Mode:None Group 1 - Event 1 Scan

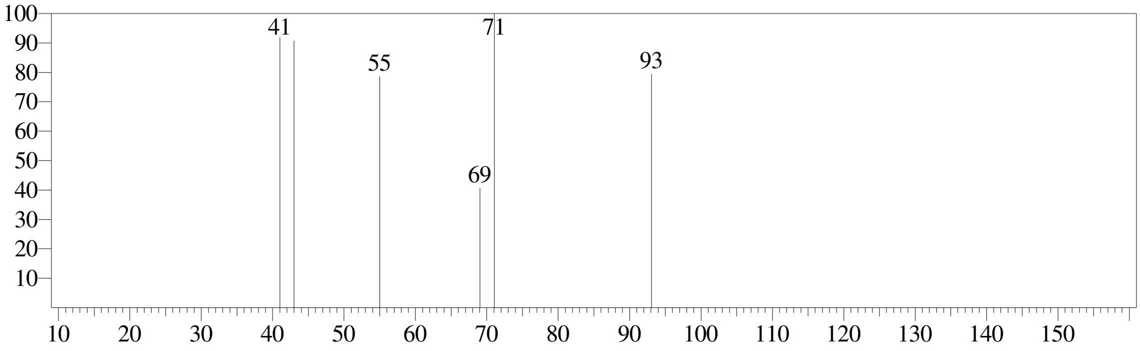


Hit#:3 Entry:11267 Library:NIST23s.lib
SI:78 Formula:C5H11Br CAS:1809-10-5 MolWeight:150 RetIndex:827
CompName:Pentane, 3-bromo- $$ 3-Bromopentane $$ 3-Pentyl bromide $$

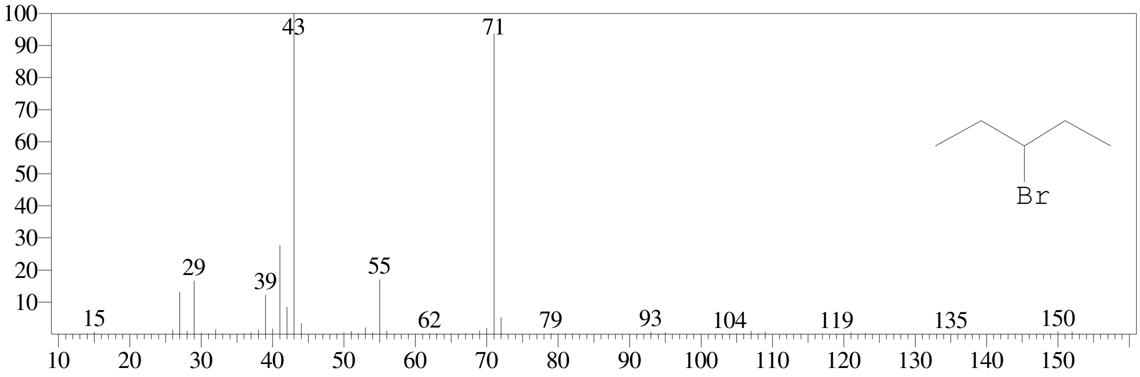


Hit#:4 Entry:11278 Library:NIST23s.lib
SI:78 Formula:C5H11Br CAS:110-53-2 MolWeight:150 RetIndex:850
CompName:Pentane, 1-bromo- $$ n-Amyl bromide $$ n-Pentyl bromide $$ Amyl bromide $$ Pentyl bromide $$ 1-Brom

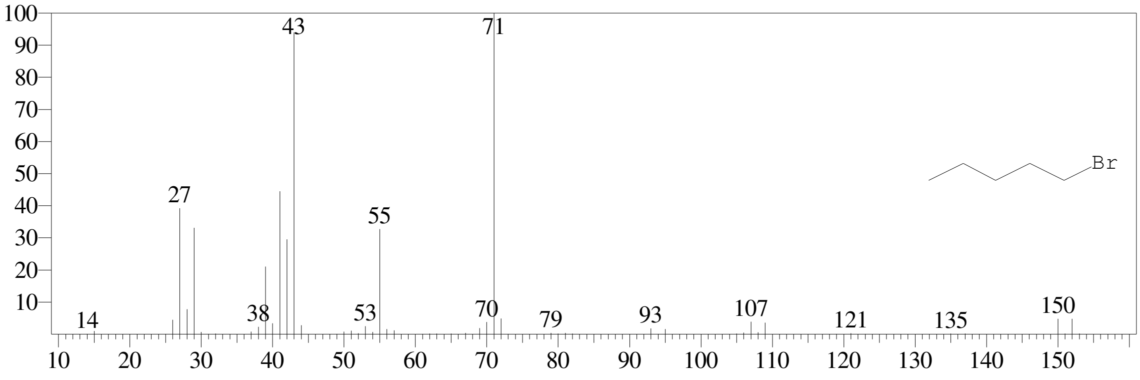

<< Target >>
Line#:6   R.Time:12.383(Scan#:1187)  MassPeaks:6
RawMode:Averaged 12.375-12.392(1186-1188) BasePeak:71.05(1705)
BG Mode:None  Group 1 - Event 1  Scan

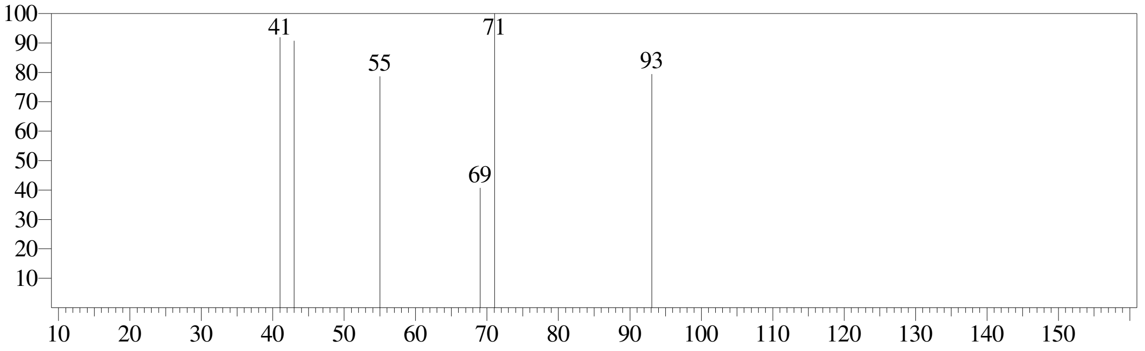


Hit#:5  Entry:11280  Library:NIST23s.lib
SI:78  Formula:C5H11Br  CAS:507-36-8  MolWeight:150  RetIndex:769
CompName:Butane, 2-bromo-2-methyl- $$ tert-Amyl bromide $$ tert-Pentyl bromide $$ 2-Bromo-2-methylbutane $$ 2-M

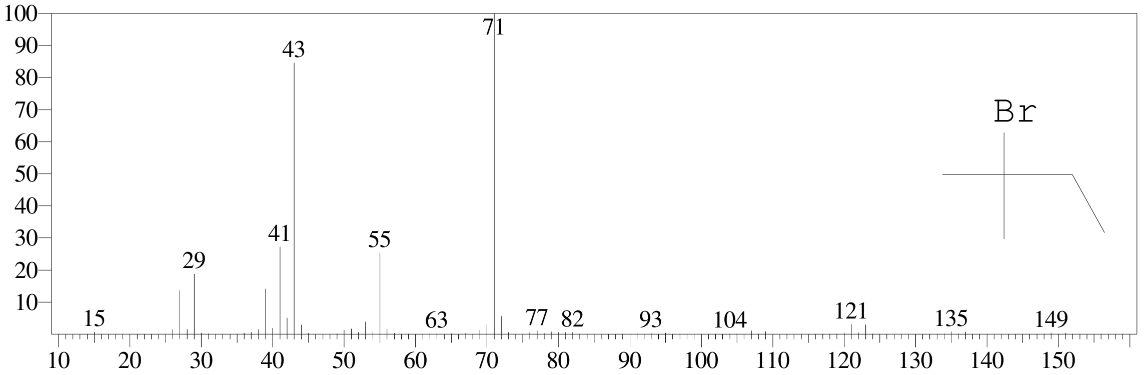

<< Target >>
Line#:7  R.Time:24.325(Scan#:2620)  MassPeaks:9
RawMode:Averaged 24.317-24.333(2619-2621) BasePeak:105.10(4333)
BG Mode:None  Group 1 - Event 1  Scan

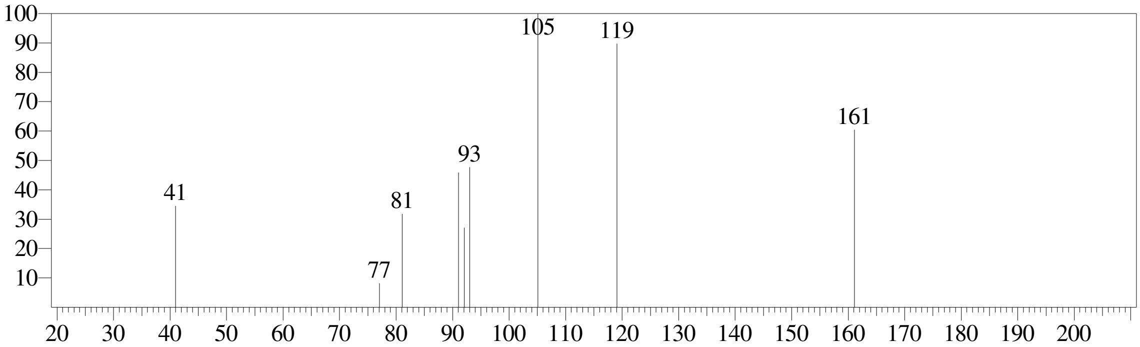


Hit#:1  Entry:24948  Library:NIST23s.lib
SI:74  Formula:C15H24  CAS:17699-14-8  MolWeight:204  RetIndex:1381
CompName:.alpha.-Cubebene $$ 1H-Cyclopenta[1,3]cyclopropa[1,2]benzene, 3a,3b,4,5,6,7-hexahydro-3,7-dimethyl-4-(1

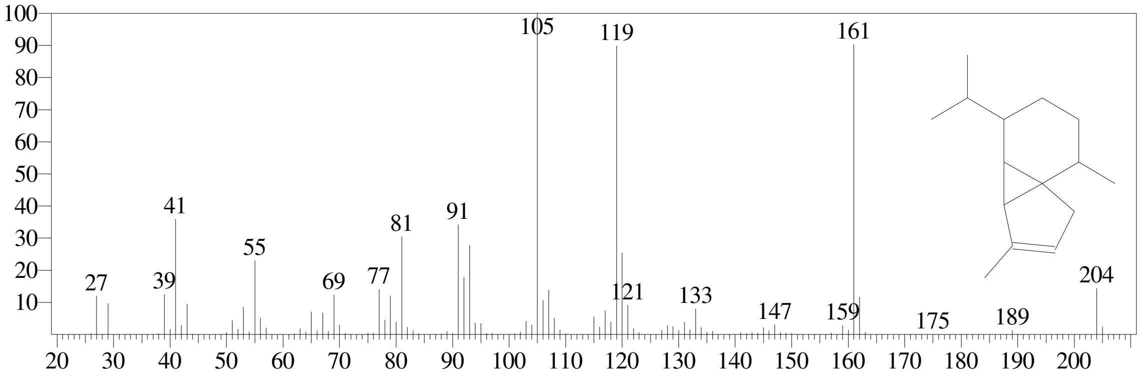


Hit#:2  Entry:62988  Library:NIST23-1.lib
SI:73  Formula:C15H24  CAS:17699-14-8  MolWeight:204  RetIndex:1381
CompName:.alpha.-Cubebene $$ 1H-Cyclopenta[1,3]cyclopropa[1,2]benzene, 3a,3b,4,5,6,7-hexahydro-3,7-dimethyl-4-(1

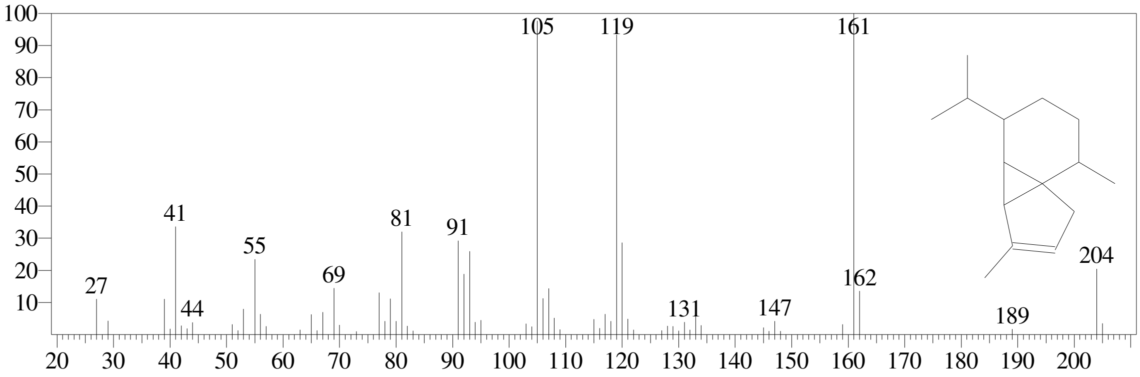

<< Target >>
Line#:7   R.Time:24.325(Scan#:2620)  MassPeaks:9
RawMode:Averaged 24.317-24.333(2619-2621) BasePeak:105.10(4333)
BG Mode:None  Group 1 - Event 1  Scan

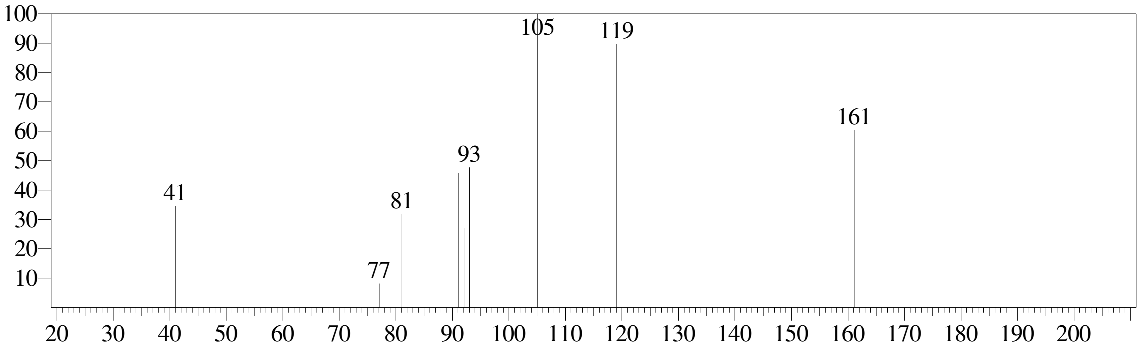


Hit#:3  Entry:25079  Library:NIST23s.lib
SI:72  Formula:C15H24  CAS:157477-72-0  MolWeight:204  RetIndex:1487
CompName:cis-Muurola-4(15),5-diene $$ (1S,4S,4aR)-1-Isopropyl-4-methyl-7-methylene-1,2,3,4,4a,5,6,7-octahydronapł

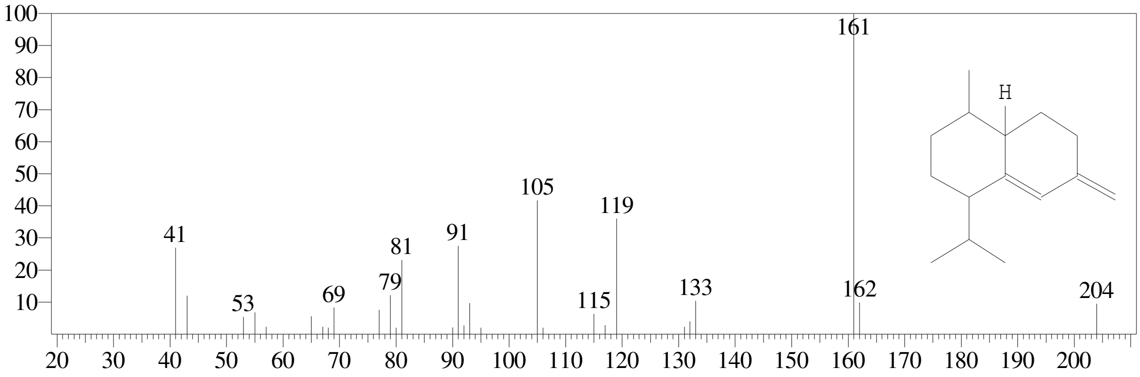


Hit#:4  Entry:24949  Library:NIST23s.lib
SI:72  Formula:C15H24  CAS:17699-14-8  MolWeight:204  RetIndex:1381
CompName:.alpha.-Cubebene $$ 1H-Cyclopenta[1,3]cyclopropa[1,2]benzene, 3a,3b,4,5,6,7-hexahydro-3,7-dimethyl-4-(1-

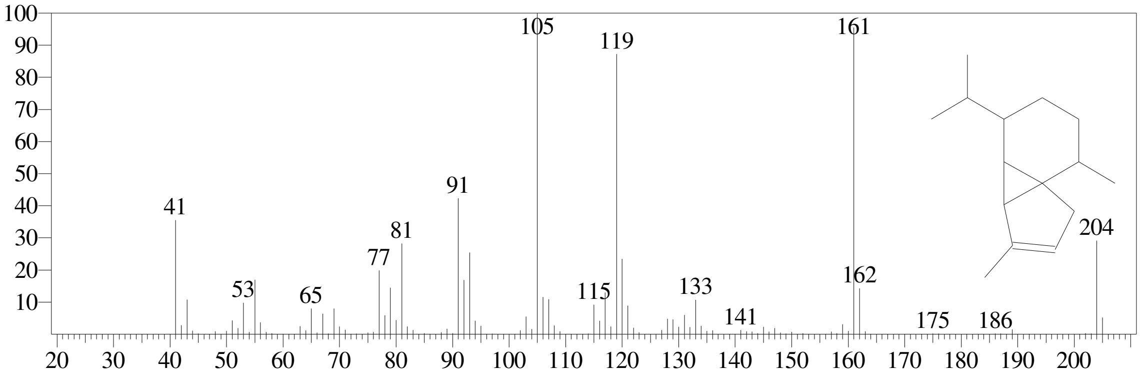

<< Target >>
Line#:7   R.Time:24.325(Scan#:2620)  MassPeaks:9
RawMode:Averaged 24.317-24.333(2619-2621) BasePeak:105.10(4333)
BG Mode:None  Group 1 - Event 1  Scan

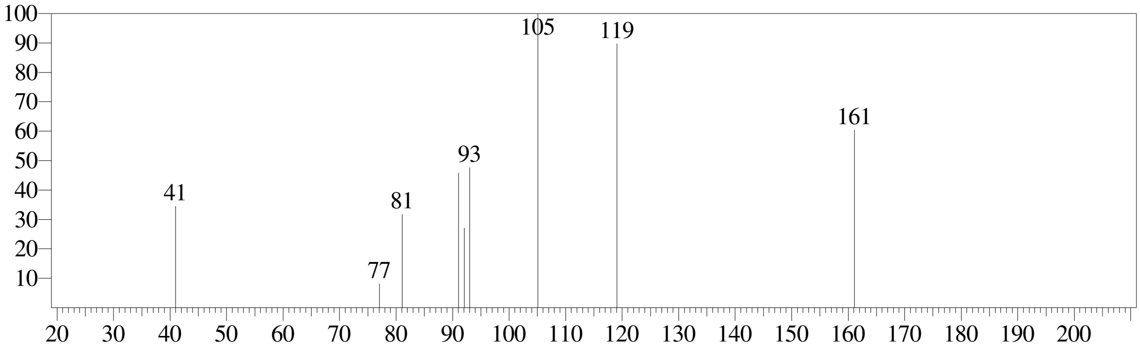


Hit#:5  Entry:25087  Library:NIST23s.lib
SI:71  Formula:C15H24  CAS:3856-25-5  MolWeight:204  RetIndex:1407
CompName:Copaene $$ Tricyclo[4.4.0.02,7]dec-3-ene, 1,3-dimethyl-8-(1-methylethyl)-, stereoisomer $$ Tricyclo[4.4.0.0

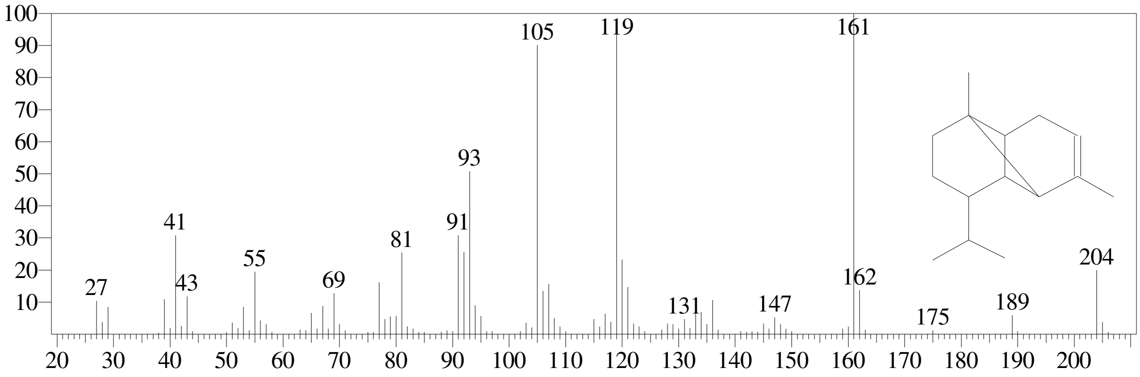

<< Target >>
Line#:8 R.Time:25.017(Scan#:2703) MassPeaks:30
RawMode:Averaged 25.008-25.025(2702-2704) BasePeak:93.05(8604)
BG Mode:None Group 1 - Event 1 Scan

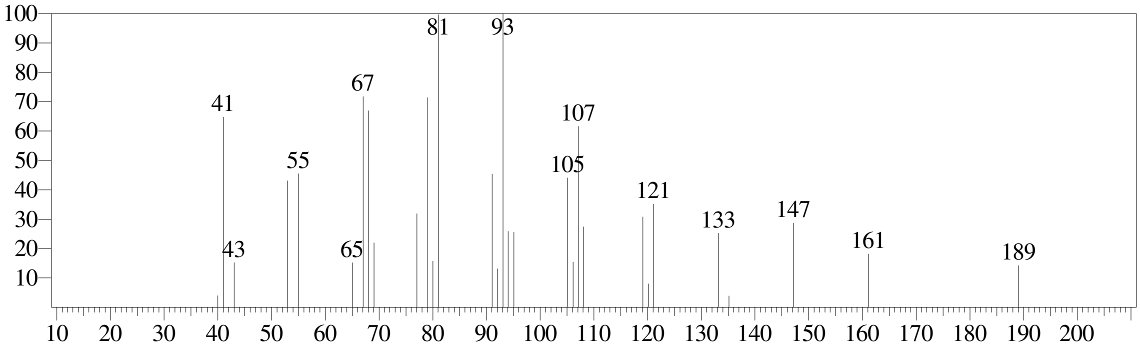


Hit#:1 Entry:24863 Library:NIST23s.lib
SI:92 Formula:C15H24 CAS:515-13-9 MolWeight:204 RetIndex:1398
CompName:Cyclohexane, 1-ethenyl-1-methyl-2,4-bis(1-methylethenyl)-, [1S-(1.alpha.,2.beta.,4.beta.)]- $$ Cyclohexane, 1

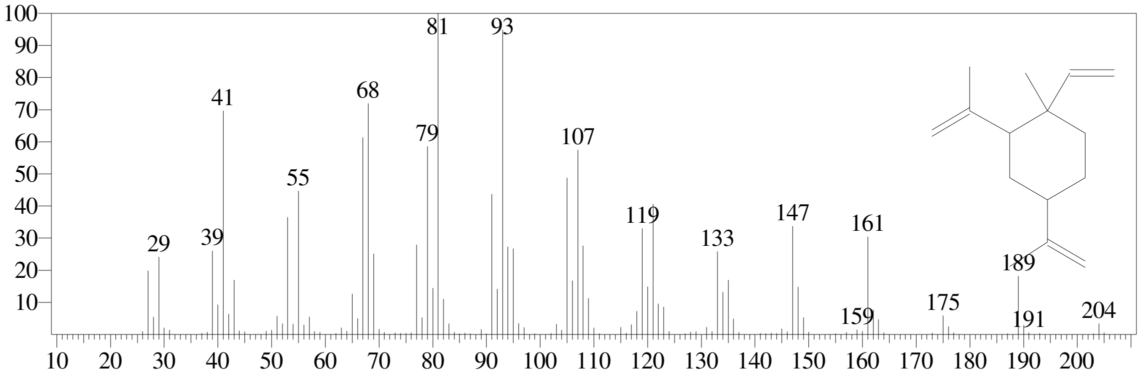


Hit#:2 Entry:24889 Library:NIST23s.lib
SI:90 Formula:C15H24 CAS:515-13-9 MolWeight:204 RetIndex:1398
CompName:Cyclohexane, 1-ethenyl-1-methyl-2,4-bis(1-methylethenyl)-, [1S-(1.alpha.,2.beta.,4.beta.)]- $$ Cyclohexane, 1

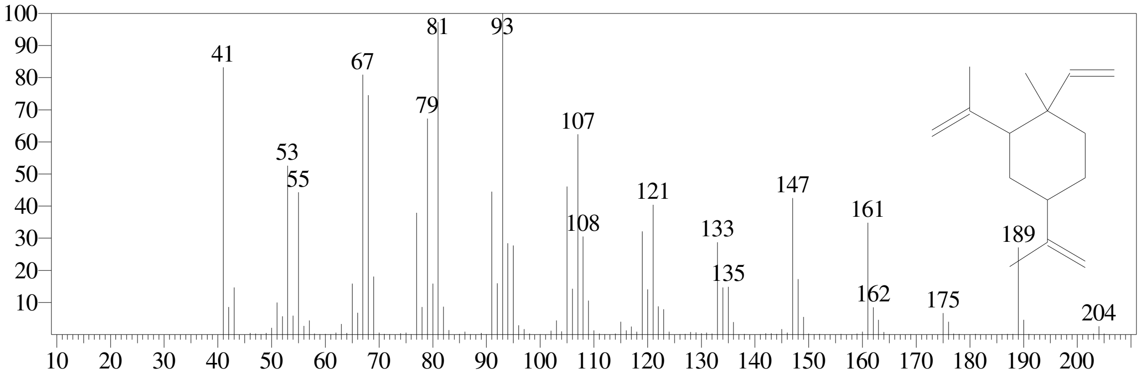

<< Target >>
Line#:8 R.Time:25.017(Scan#:2703) MassPeaks:30
RawMode:Averaged 25.008-25.025(2702-2704) BasePeak:93.05(8604)
BG Mode:None Group 1 - Event 1 Scan

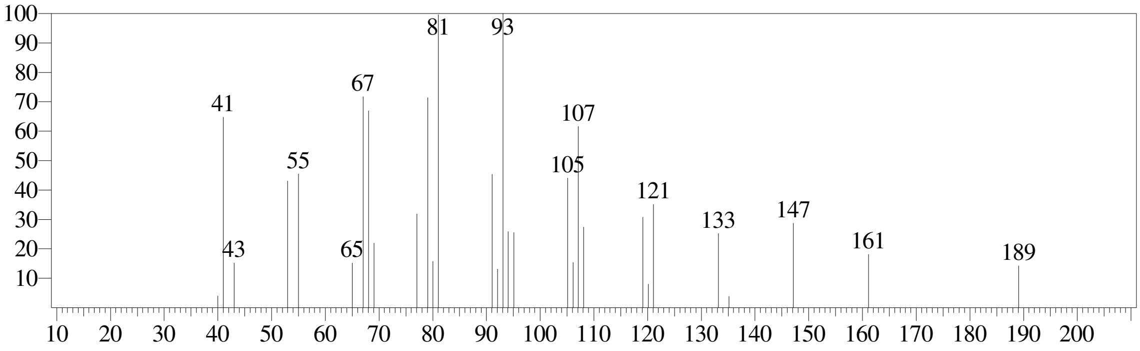

Hit#:3 Entry:62789 Library:NIST23-1.lib
SI:89 Formula:C15H24 CAS:515-13-9 MolWeight:204 RetIndex:1398
CompName:Cyclohexane, 1-ethenyl-1-methyl-2,4-bis(1-methylethenyl)-, [1S-(1.alpha.,2.beta.,4.beta.)]- $$ Cyclohexane,

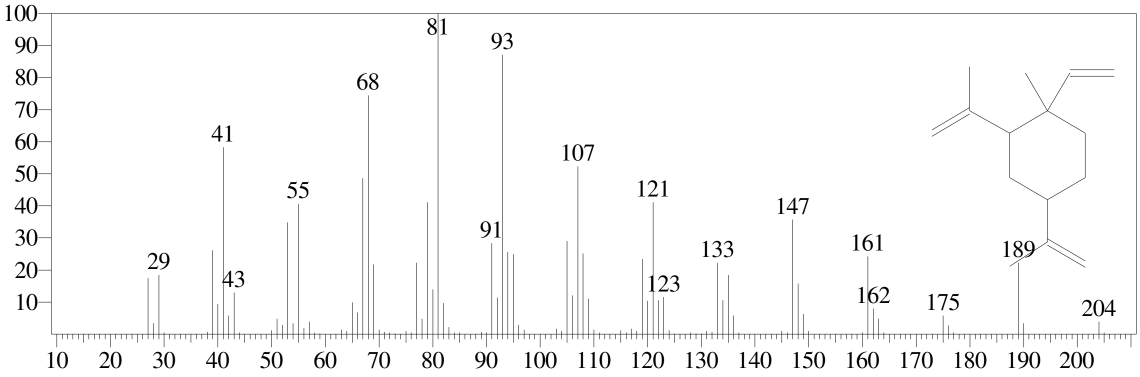

Hit#:4 Entry:62785 Library:NIST23-1.lib
SI:88 Formula:C15H24 CAS:110823-68-2 MolWeight:204 RetIndex:1398
CompName:Cyclohexane, 1-ethenyl-1-methyl-2,4-bis(1-methylethenyl)- $$ 2,4-Diisopropenyl-1-methyl-1-vinylcyclohexa

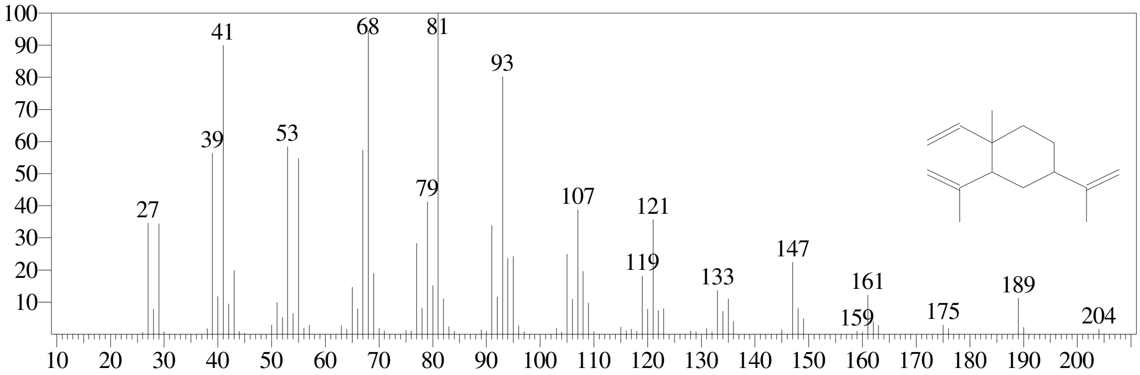

<< Target >>
Line#:8   R.Time:25.017(Scan#:2703)  MassPeaks:30
RawMode:Averaged 25.008-25.025(2702-2704) BasePeak:93.05(8604)
BG Mode:None  Group 1 - Event 1  Scan

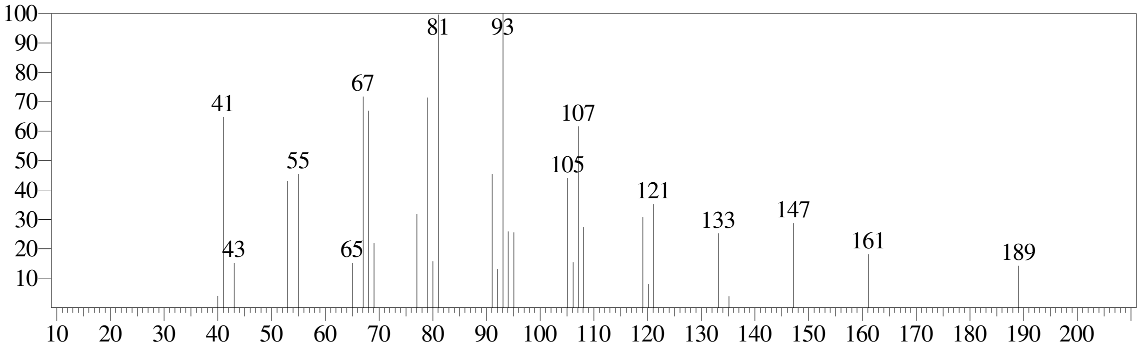


Hit#:5  Entry:24860  Library:NIST23s.lib
SI:88  Formula:C15H24  CAS:515-13-9  MolWeight:204  RetIndex:1398
CompName:Cyclohexane, 1-ethenyl-1-methyl-2,4-bis(1-methylethenyl)-, [1S-(1.alpha.,2.beta.,4.beta.)]- $$ Cyclohexane, [illegible]

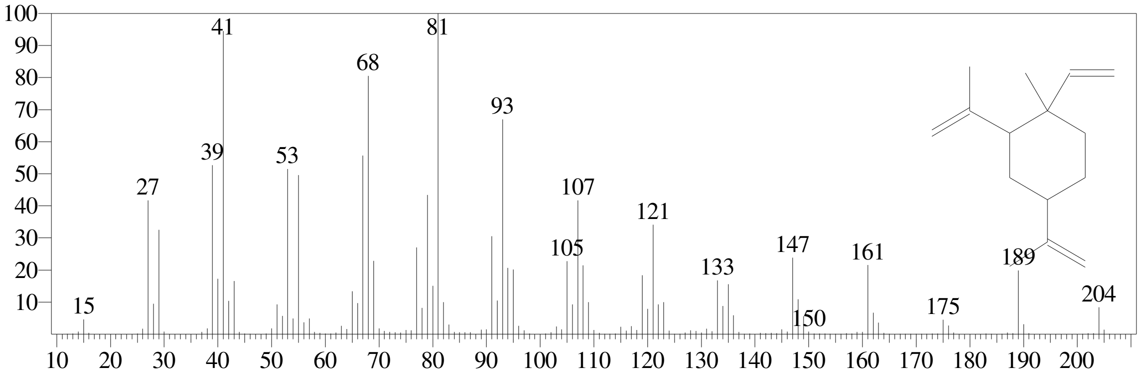

<< Target >>
Line#:9 R.Time:25.758(Scan#:2792) MassPeaks:8
RawMode:Averaged 25.750-25.767(2791-2793) BasePeak:105.10(2097)
BG Mode:None Group 1 - Event 1 Scan

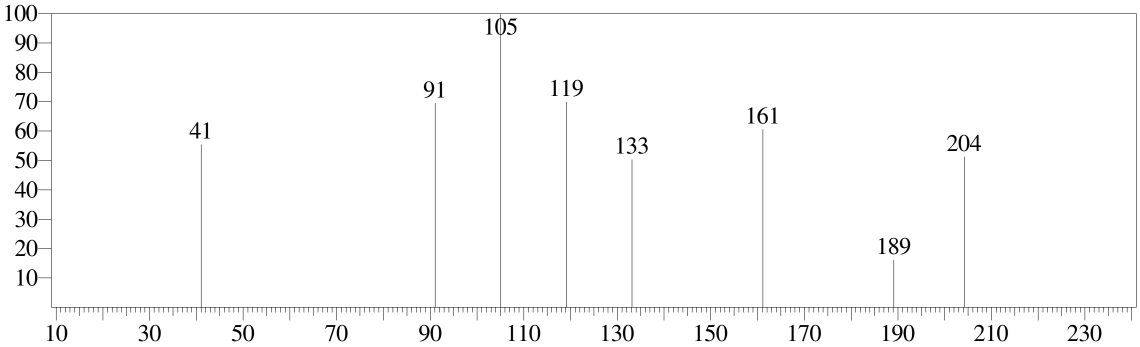


Hit#:1 Entry:62859 Library:NIST23-1.lib
SI:70 Formula:C15H24 CAS:95910-36-4 MolWeight:204 RetIndex:1396
CompName:isoledene $$ 1,1,4,7-Tetramethyl-1a,2,3,4,5,6,7,7b-octahydro-1H-cyclopropa[e]azulene # $$ (1aR,4R,7R,7bS

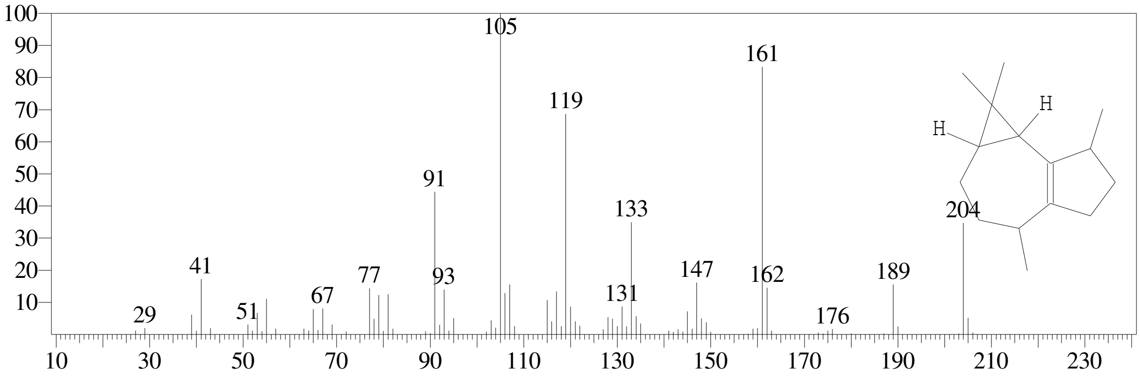


Hit#:2 Entry:62843 Library:NIST23-1.lib
SI:69 Formula:C15H24 CAS:54932-91-1 MolWeight:204 RetIndex:1442
CompName:Benzene, (1-methyl-1-propylpentyl)- $$ (1-Methyl-1-propylpentyl)benzene # $$

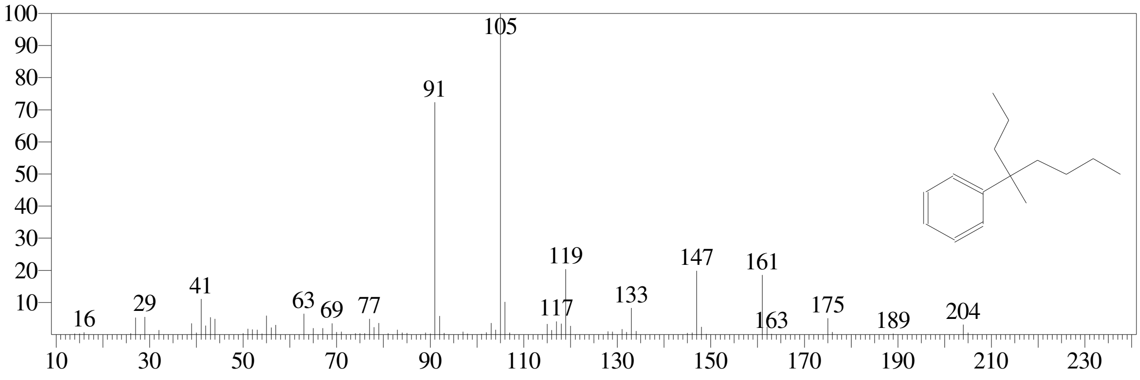

<< Target >>
Line#:9 R.Time:25.758(Scan#:2792) MassPeaks:8
RawMode:Averaged 25.750-25.767(2791-2793) BasePeak:105.10(2097)
BG Mode:None Group 1 - Event 1 Scan

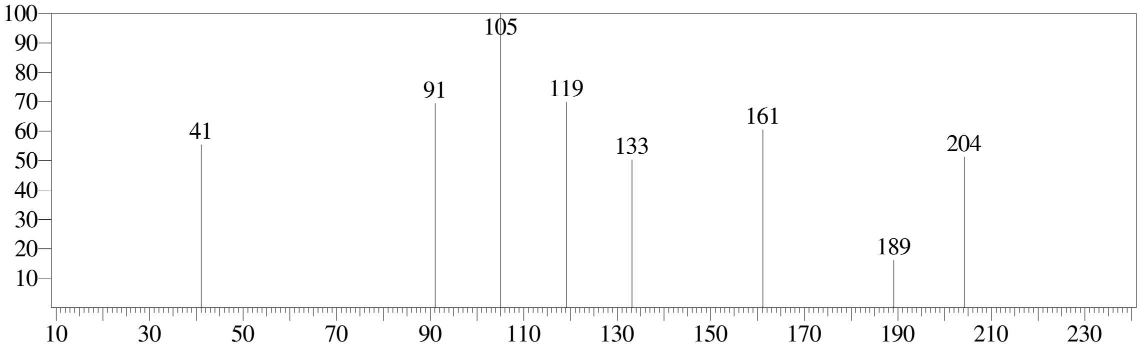


Hit#:3 Entry:102501 Library:NIST23-1.lib
SI:67 Formula:C15H23Cl CAS:64275-44-1 MolWeight:238 RetIndex:1839
CompName:5,10-Pentadecadiyne, 1-chloro- $$ 1-Chloro-5,10-pentadecadiyne # $$

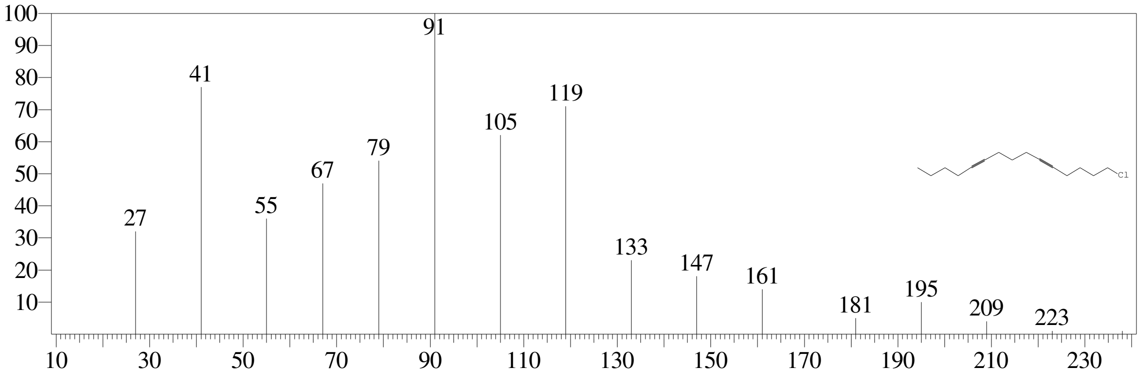


Hit#:4 Entry:62948 Library:NIST23-1.lib
SI:67 Formula:C15H24 CAS:60909-27-5 MolWeight:204 RetIndex:1464
CompName:Himachala-2,4-diene $$ 3,5,5,9-Tetramethyl-5,6,7,8,9,9a-hexahydro-1H-benzo[a]cycloheptene # $$

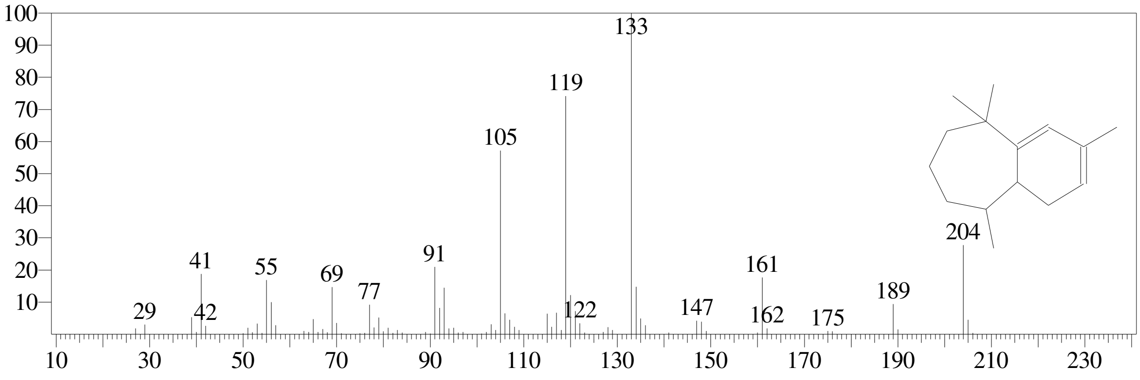

<< Target >>
Line#:9   R.Time:25.758(Scan#:2792)  MassPeaks:8
RawMode:Averaged 25.750-25.767(2791-2793) BasePeak:105.10(2097)
BG Mode:None  Group 1 - Event 1  Scan

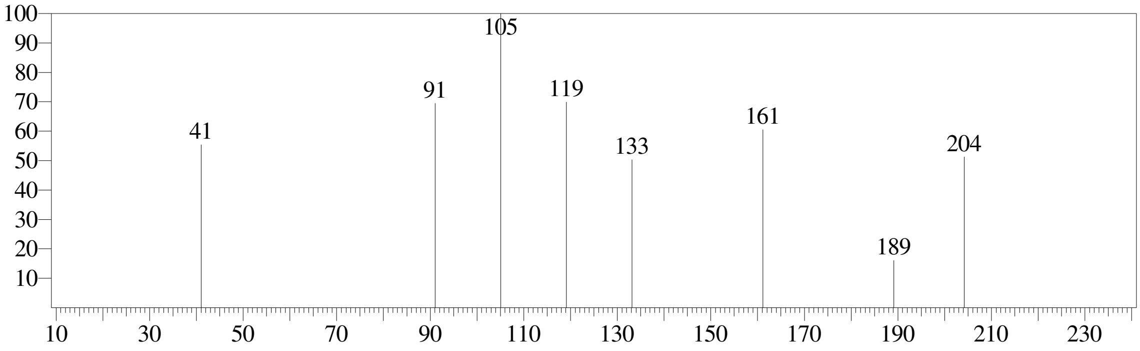


Hit#:5  Entry:25079  Library:NIST23s.lib
SI:66  Formula:C15H24  CAS:157477-72-0  MolWeight:204  RetIndex:1487
CompName:cis-Muurola-4(15),5-diene $$ (1S,4S,4aR)-1-Isopropyl-4-methyl-7-methylene-1,2,3,4,4a,5,6,7-octahydronaph

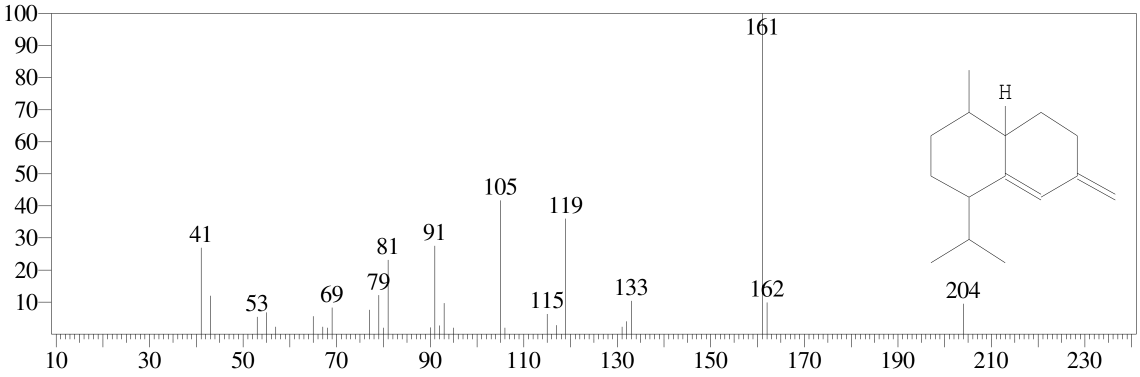

<< Target >>
Line#:10 R.Time:26.150(Scan#:2839) MassPeaks:60
RawMode:Averaged 26.142-26.158(2838-2840) BasePeak:41.00(47448)
BG Mode:None Group 1 - Event 1 Scan

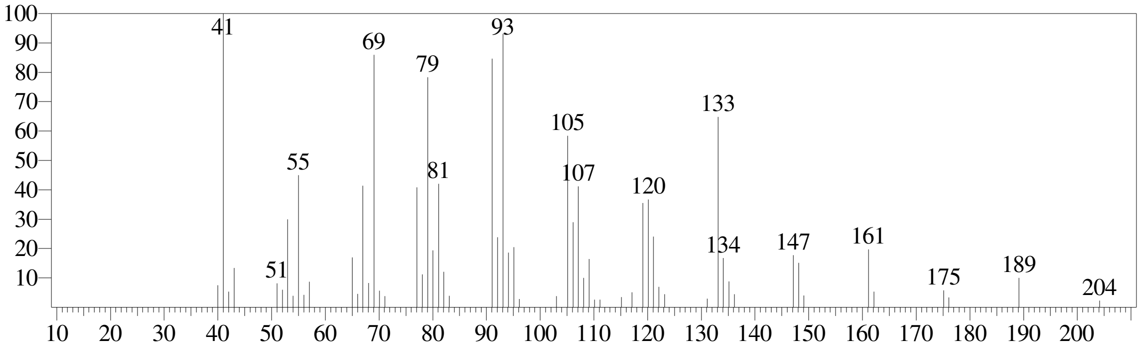


Hit#:1 Entry:24804 Library:NIST23s.lib
SI:95 Formula:C15H24 CAS:87-44-5 MolWeight:204 RetIndex:1448
CompName:Caryophyllene $$ Bicyclo[7.2.0]undec-4-ene, 4,11,11-trimethyl-8-methylene-, [1R-(1R*,4E,9S*)]- $$ Bicycl

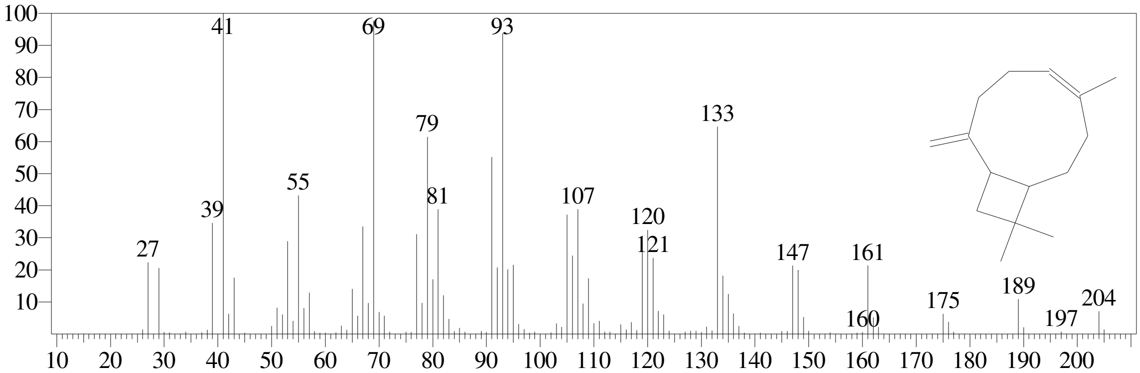


Hit#:2 Entry:62827 Library:NIST23-1.lib
SI:94 Formula:C15H24 CAS:87-44-5 MolWeight:204 RetIndex:1448
CompName:Caryophyllene $$ Bicyclo[7.2.0]undec-4-ene, 4,11,11-trimethyl-8-methylene-, [1R-(1R*,4E,9S*)]- $$ Bicycl

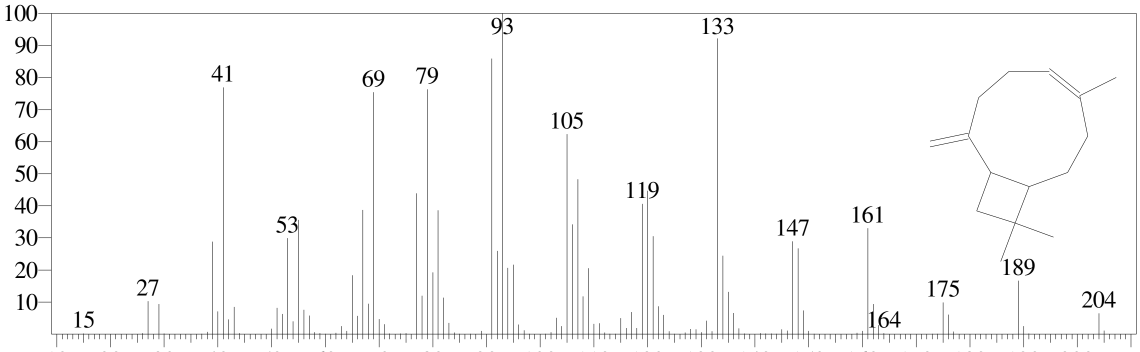

<< Target >>
Line#:10   R.Time:26.150(Scan#:2839)  MassPeaks:60
RawMode:Averaged 26.142-26.158(2838-2840) BasePeak:41.00(47448)
BG Mode:None  Group 1 - Event 1  Scan

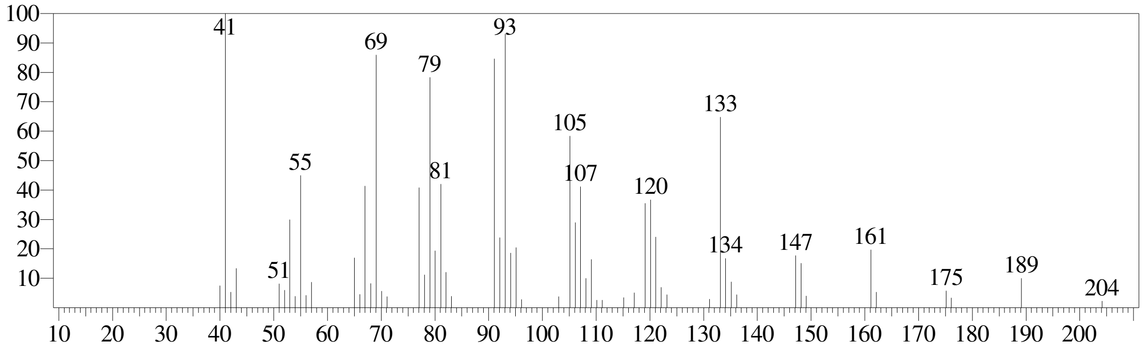


Hit#:3  Entry:24803  Library:NIST23s.lib
SI:94  Formula:C15H24  CAS:118-65-0  MolWeight:204  RetIndex:1448
CompName:Bicyclo[7.2.0]undec-4-ene, 4,11,11-trimethyl-8-methylene-,[1R-(1R*,4Z,9S*)]- $$ Isocaryophyllene $$ 4,11,

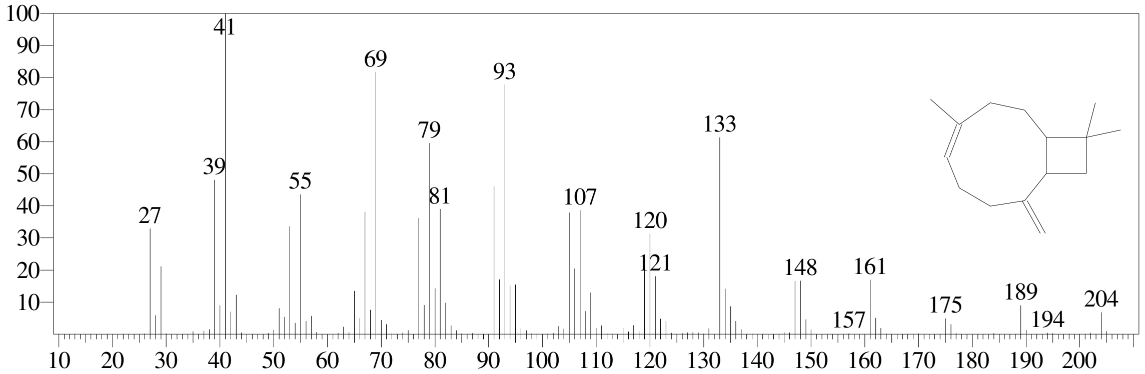


Hit#:4  Entry:24827  Library:NIST23s.lib
SI:94  Formula:C15H24  CAS:87-44-5  MolWeight:204  RetIndex:1448
CompName:Caryophyllene $$ Bicyclo[7.2.0]undec-4-ene, 4,11,11-trimethyl-8-methylene-, [1R-(1R*,4E,9S*)]- $$ Bicycl

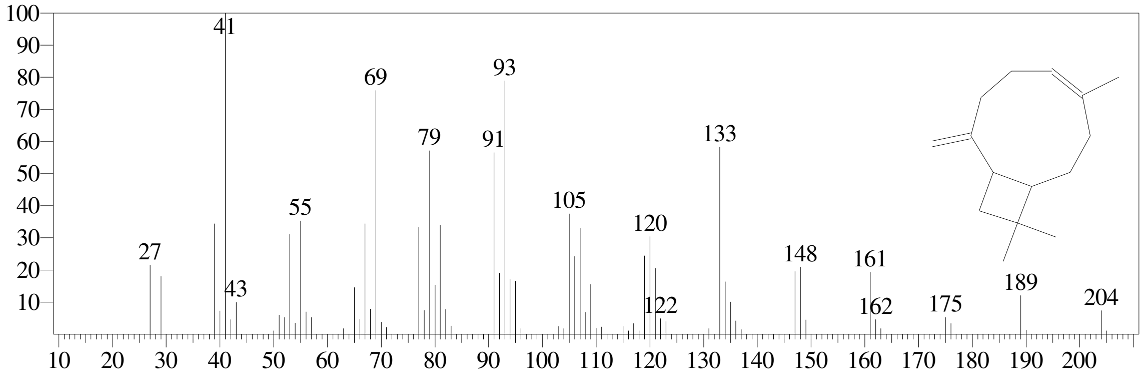

<< Target >>
Line#:10   R.Time:26.150(Scan#:2839)  MassPeaks:60
RawMode:Averaged 26.142-26.158(2838-2840) BasePeak:41.00(47448)
BG Mode:None  Group 1 - Event 1  Scan

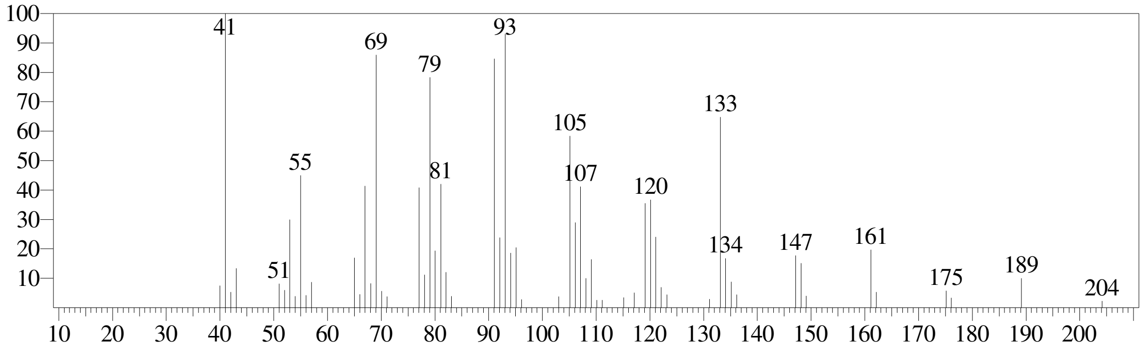


Hit#:5  Entry:62803  Library:NIST23-1.lib
SI:94  Formula:C15H24  CAS:13877-93-5  MolWeight:204  RetIndex:1448
CompName:Bicyclo[7.2.0]undec-4-ene, 4,11,11-trimethyl-8-methylene- $$ Bicyclo[7.2.0]undec-4-ene, 4,11,11-trimethyl-

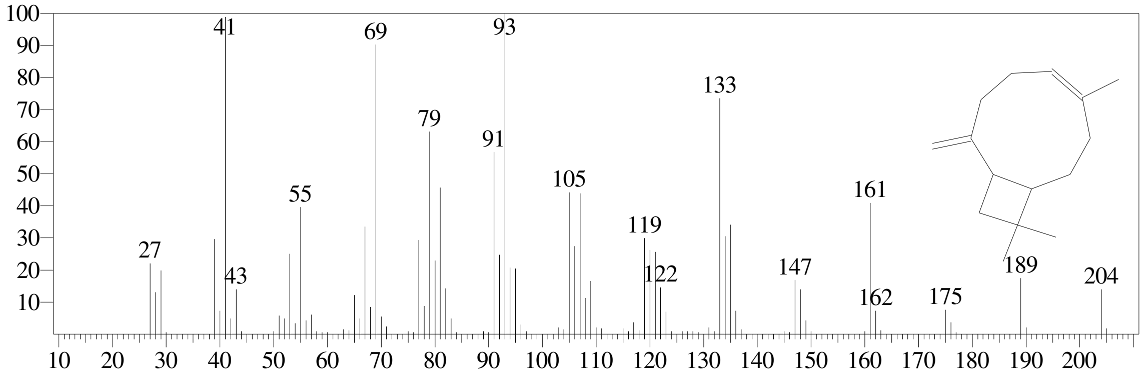

<< Target >>
Line#:11 R.Time:26.508(Scan#:2882) MassPeaks:15
RawMode:Averaged 26.500-26.517(2881-2883) BasePeak:91.05(2913)
BG Mode:None Group 1 - Event 1 Scan

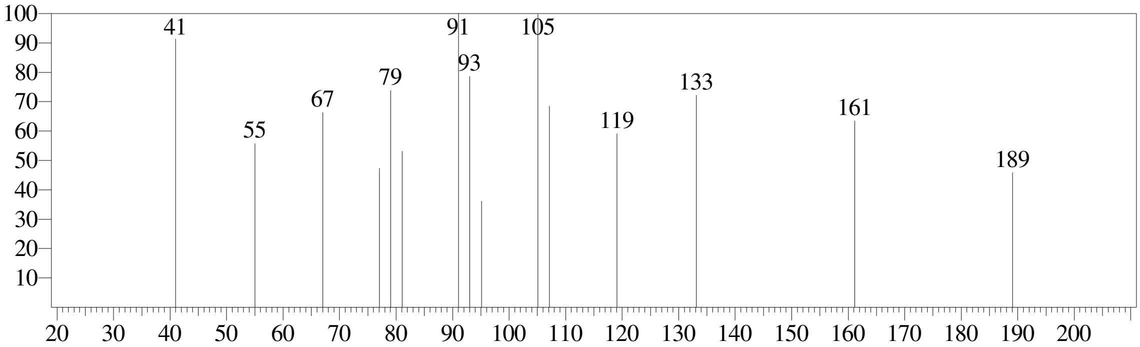


Hit#:1 Entry:24842 Library:NIST23s.lib
SI:76 Formula:C15H24 CAS:56684-97-0 MolWeight:204 RetIndex:1408
CompName:.beta.-Panasinsene $$ 2,2,4a-Trimethyl-8-methylenedecahydrocyclobuta[c]indene # $$

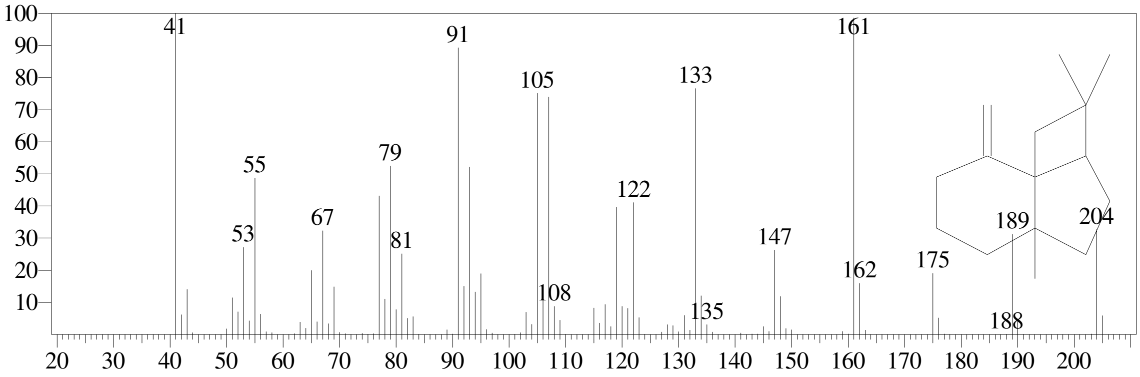


Hit#:2 Entry:24894 Library:NIST23s.lib
SI:76 Formula:C15H24 CAS:3691-11-0 MolWeight:204 RetIndex:1498
CompName:Azulene, 1,2,3,5,6,7,8,8a-octahydro-1,4-dimethyl-7-(1-methylethenyl)-, [1S-(1.alpha.,7.alpha.,8a.beta.)]- $$ (

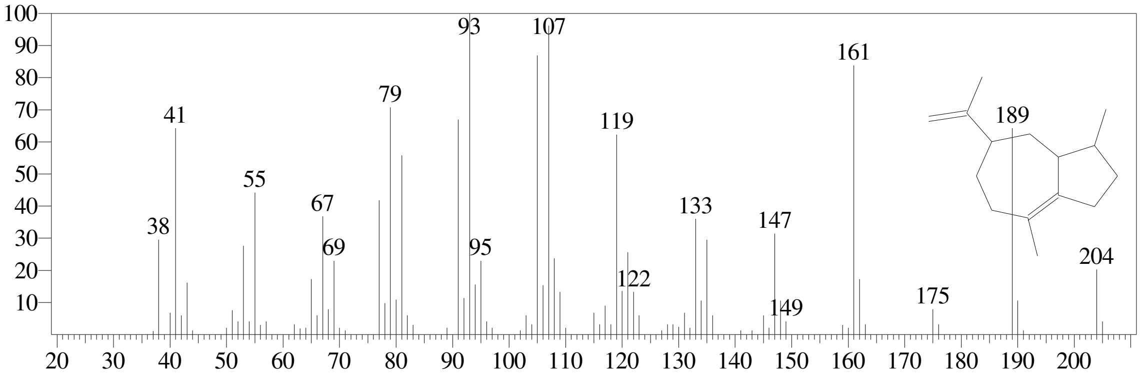

<< Target >>
Line#:11 R.Time:26.508(Scan#:2882) MassPeaks:15
RawMode:Averaged 26.500-26.517(2881-2883) BasePeak:91.05(2913)
BG Mode:None Group 1 - Event 1 Scan

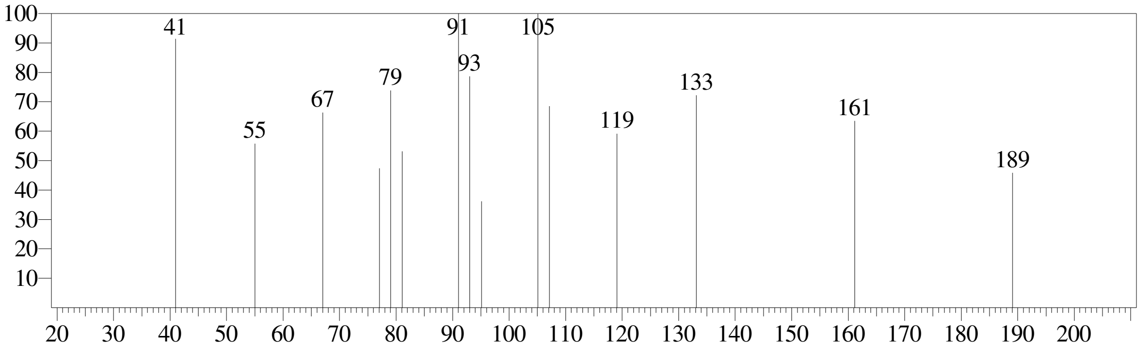


Hit#:3 Entry:24840 Library:NIST23s.lib
SI:76 Formula:C15H24 CAS:473-14-3 MolWeight:204 RetIndex:1509
CompName:Naphthalene, 2,3,4,4a,5,6-hexahydro-1,4a-dimethyl-7-(1-methylethyl)- $$ 6-Isopropyl-4,8a-dimethyl-1,2,3,7,

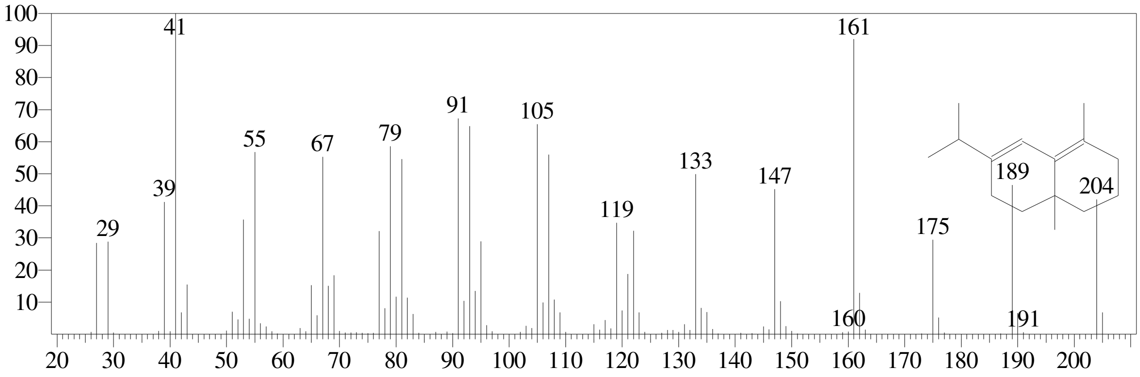


Hit#:4 Entry:24843 Library:NIST23s.lib
SI:76 Formula:C15H24 CAS:17066-67-0 MolWeight:204 RetIndex:1489
CompName:Naphthalene, decahydro-4a-methyl-1-methylene-7-(1-methylethenyl)-, [4aR-(4a.alpha.,7.alpha.,8a.beta.)]- $$

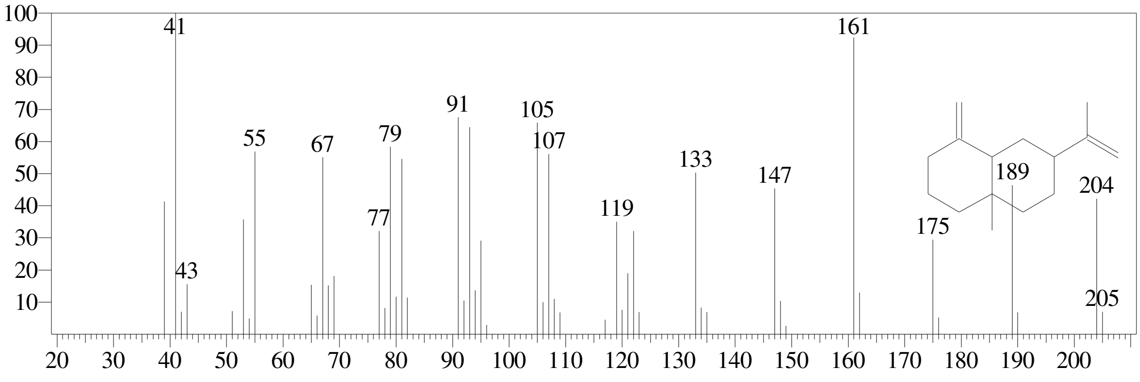

<< Target >>
Line#:11 R.Time:26.508(Scan#:2882) MassPeaks:15
RawMode:Averaged 26.500-26.517(2881-2883) BasePeak:91.05(2913)
BG Mode:None Group 1 - Event 1 Scan

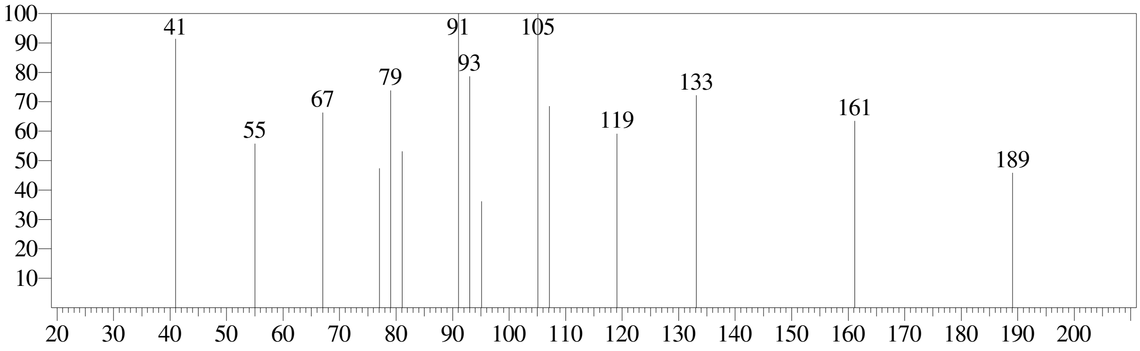


Hit#:5 Entry:24841 Library:NIST23s.lib
SI:74 Formula:C15H24 CAS:4630-07-3 MolWeight:204 RetIndex:1496
CompName:Naphthalene, 1,2,3,5,6,7,8,8a-octahydro-1,8a-dimethyl-7-(1-methylethenyl)-, [1R-(1.alpha.,7.beta.,8a.alpha.)]

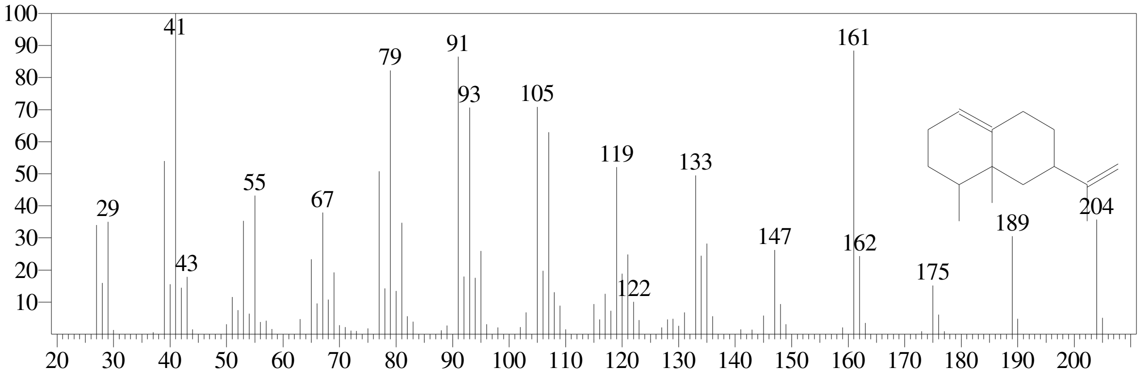

<< Target >>
Line#:12   R.Time:26.767(Scan#:2913)  MassPeaks:6
RawMode:Averaged 26.758-26.775(2912-2914) BasePeak:107.10(2263)
BG Mode:None  Group 1 - Event 1  Scan

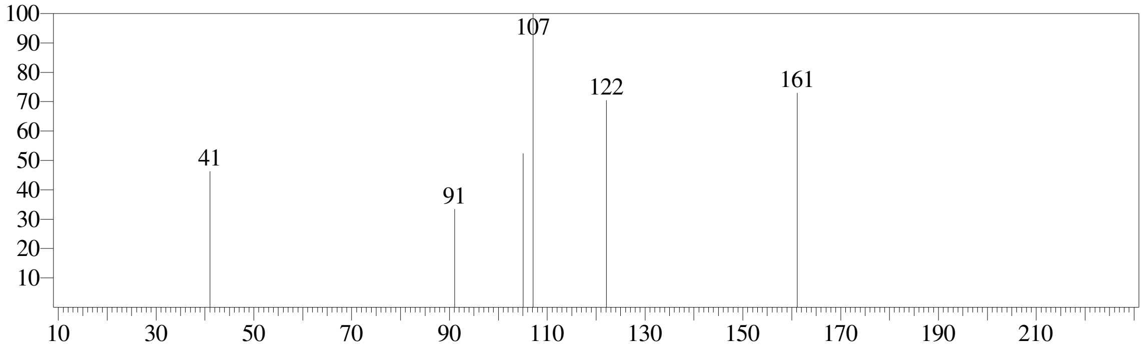


Hit#:1  Entry:25094  Library:NIST23s.lib
SI:71  Formula:C15H24  CAS:123123-37-5  MolWeight:204  RetIndex:1483
CompName:(2S,4aR,8aR)-4a,8-Dimethyl-2-(prop-1-en-2-yl)-1,2,3,4,4a,5,6,8a-octahydronaphthalene $$ Naphthalene, 1,2,

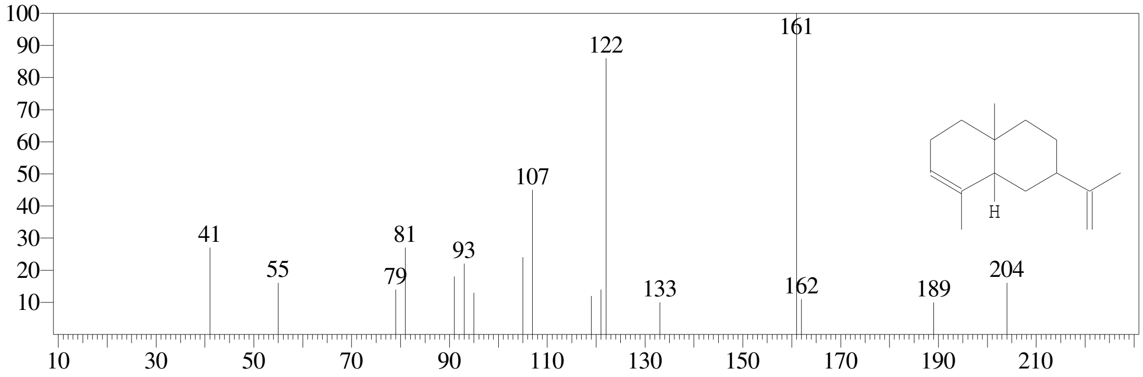


Hit#:2  Entry:28768  Library:NIST23s.lib
SI:68  Formula:C15H26O  CAS:123123-38-6  MolWeight:222  RetIndex:1626
CompName:7-epi-a-Eudesmol $$ 7-epi-.alpha.-eudesmol $$ 2-((2S,4aR,8aR)-4a,8-dimethyl-1,2,3,4,4a,5,6,8a-octahydrona

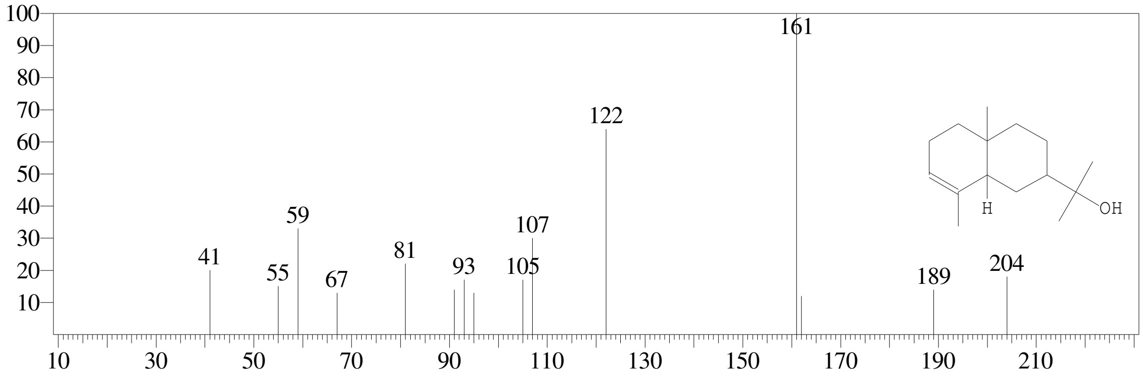

<< Target >>
Line#:12 R.Time:26.767(Scan#:2913) MassPeaks:6
RawMode:Averaged 26.758-26.775(2912-2914) BasePeak:107.10(2263)
BG Mode:None Group 1 - Event 1 Scan

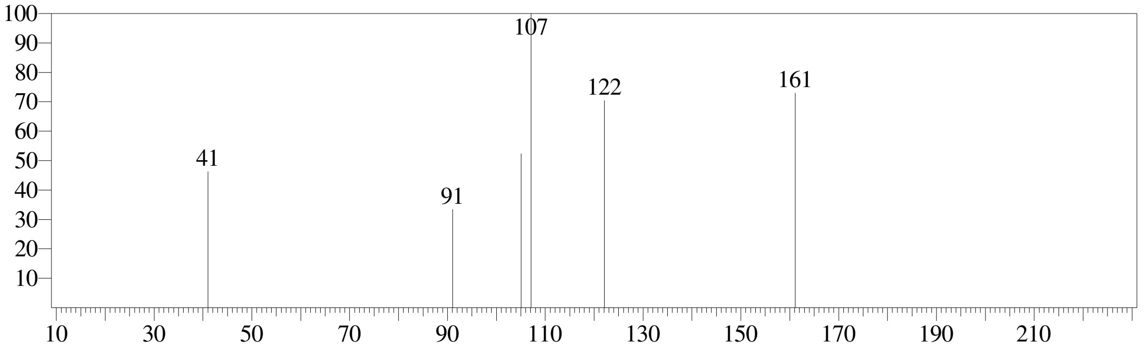


Hit#:3 Entry:62942 Library:NIST23-1.lib
SI:68 Formula:C15H24 CAS:56633-28-4 MolWeight:204 RetIndex:1410
CompName:(-)-.alpha.-Panasinsen $$ (2aR,4aS,8aR)-2,2,4a,8-Tetramethyl-1,2,2a,3,4,4a,5,6-octahydrocyclobuta[c]indene

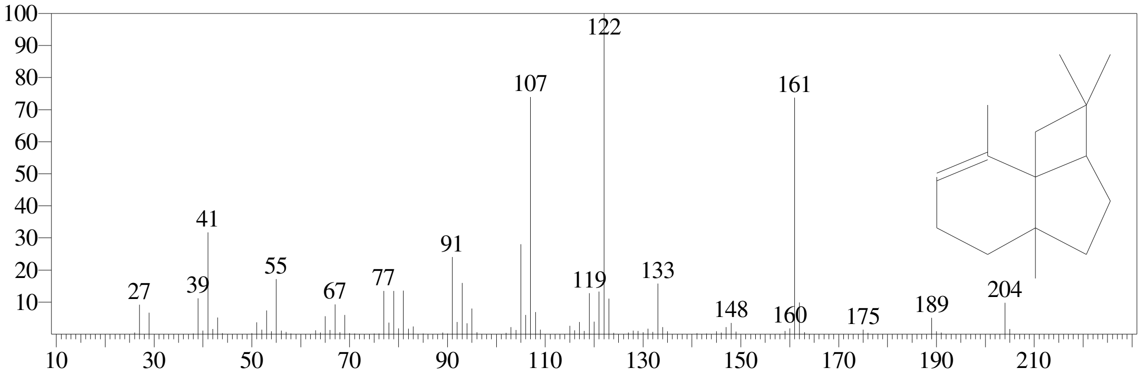


Hit#:4 Entry:27295 Library:NIST23-1.lib
SI:67 Formula:C10H12O2 CAS:3245-23-6 MolWeight:164 RetIndex:1272
CompName:4-Ethylphenyl acetate $$ Phenol, 4-ethyl-, acetate $$ 1-Acetoxy-4-ethylbenzene $$

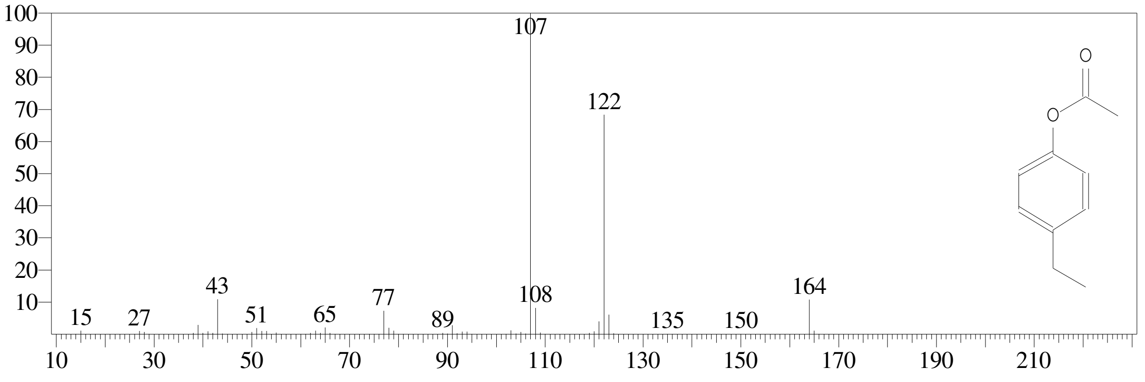

<< Target >>
Line#:12   R.Time:26.767(Scan#:2913)  MassPeaks:6
RawMode:Averaged 26.758-26.775(2912-2914) BasePeak:107.10(2263)
BG Mode:None  Group 1 - Event 1  Scan

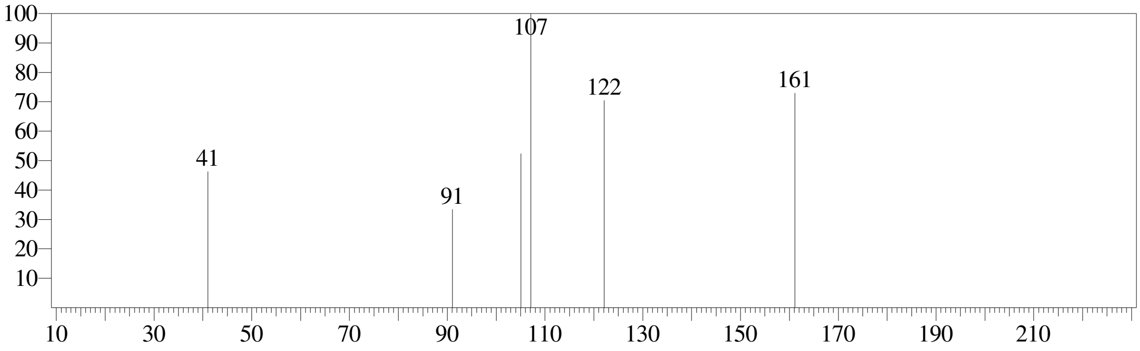


Hit#:5  Entry:63009  Library:NIST23-1.lib
SI:66  Formula:C15H24  CAS:123123-37-5  MolWeight:204  RetIndex:1483
CompName:(2S,4aR,8aR)-4a,8-Dimethyl-2-(prop-1-en-2-yl)-1,2,3,4,4a,5,6,8a-octahydronaphthalene $$ Naphthalene, 1,2,

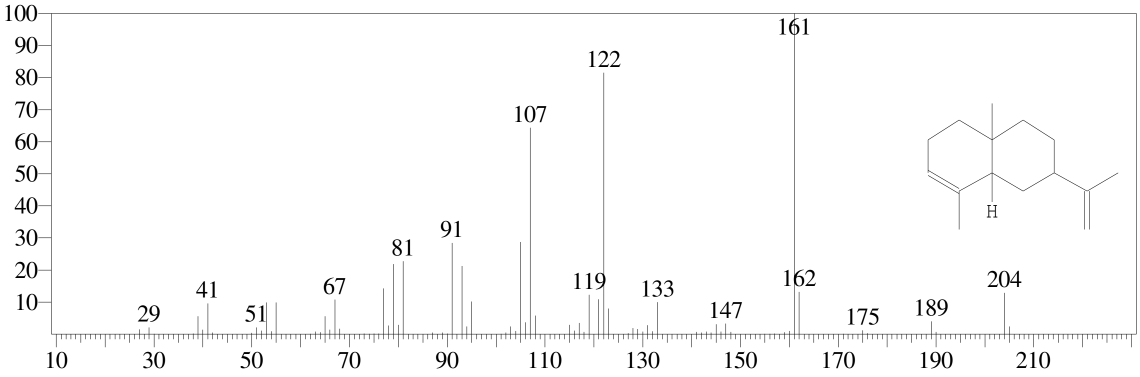

<< Target >>
Line#:13  R.Time:26.950(Scan#:2935)  MassPeaks:57
RawMode:Averaged 26.942-26.958(2934-2936) BasePeak:41.00(29600)
BG Mode:None  Group 1 - Event 1  Scan

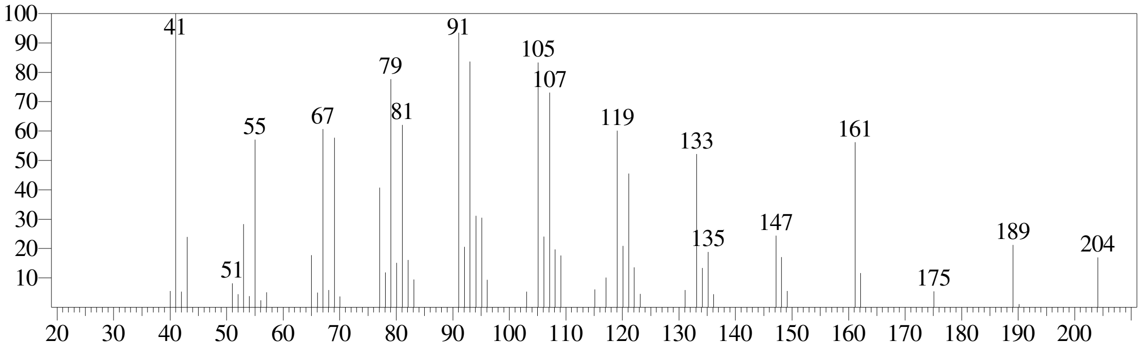


Hit#:1  Entry:24844  Library:NIST23s.lib
SI:94  Formula:C15H24  CAS:489-39-4  MolWeight:204  RetIndex:1424
CompName:Aromandendrene $$ 1H-Cycloprop[e]azulene, decahydro-1,1,7-trimethyl-4-methylene-, [1aR-(1a.alpha.,4a.al

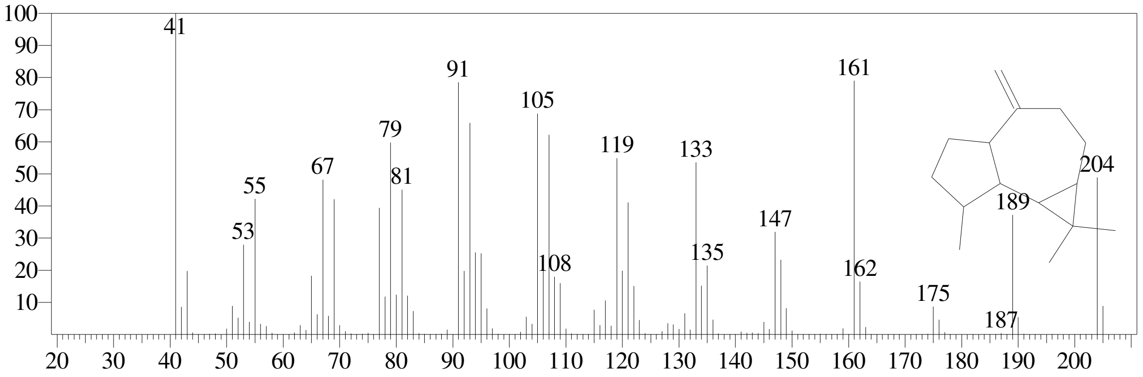


Hit#:2  Entry:24869  Library:NIST23s.lib
SI:94  Formula:C15H24  CAS:489-39-4  MolWeight:204  RetIndex:1424
CompName:Aromandendrene $$ 1H-Cycloprop[e]azulene, decahydro-1,1,7-trimethyl-4-methylene-, [1aR-(1a.alpha.,4a.al

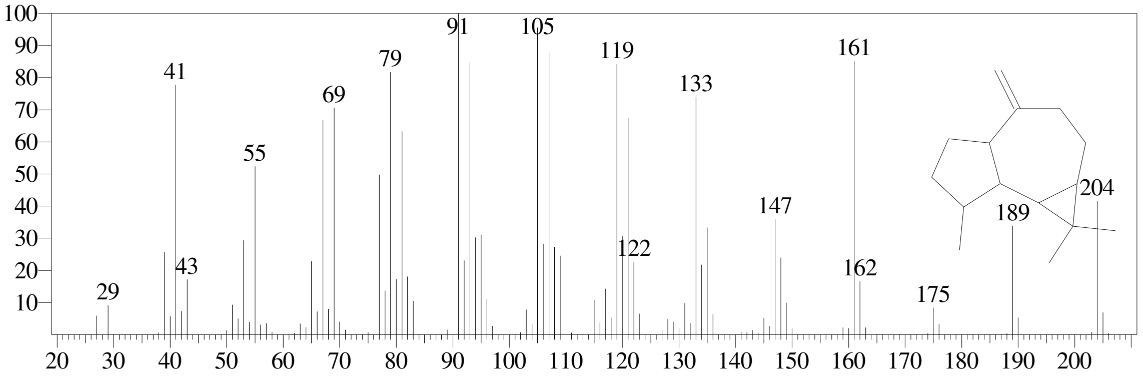

<< Target >>
Line#:13   R.Time:26.950(Scan#:2935)  MassPeaks:57
RawMode:Averaged 26.942-26.958(2934-2936) BasePeak:41.00(29600)
BG Mode:None  Group 1 - Event 1  Scan

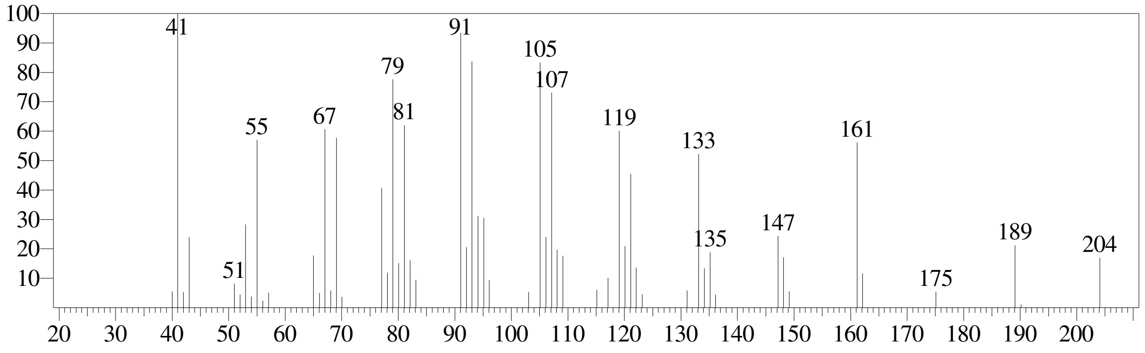


Hit#:3  Entry:62794  Library:NIST23-1.lib
SI:94  Formula:C15H24  CAS:68832-35-9  MolWeight:204  RetIndex:1450
CompName:(1R,9R,E)-4,11,11-Trimethyl-8-methylenebicyclo[7.2.0]undec-4-ene $$ Bicyclo[7.2.0]undec-4-ene, 4,11,11-t

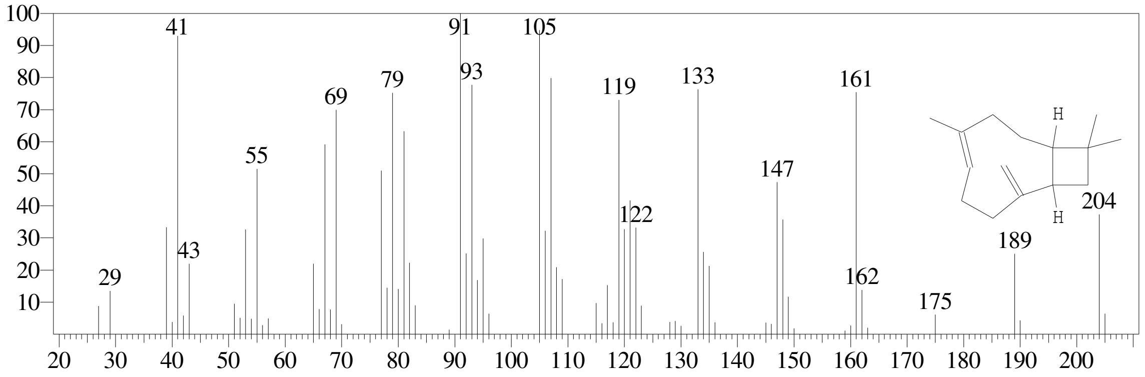


Hit#:4  Entry:62764  Library:NIST23-1.lib
SI:94  Formula:C15H24  CAS:489-39-4  MolWeight:204  RetIndex:1424
CompName:Aromandendrene $$ 1H-Cycloprop[e]azulene, decahydro-1,1,7-trimethyl-4-methylene-, [1aR-(1a.alpha.,4a.al

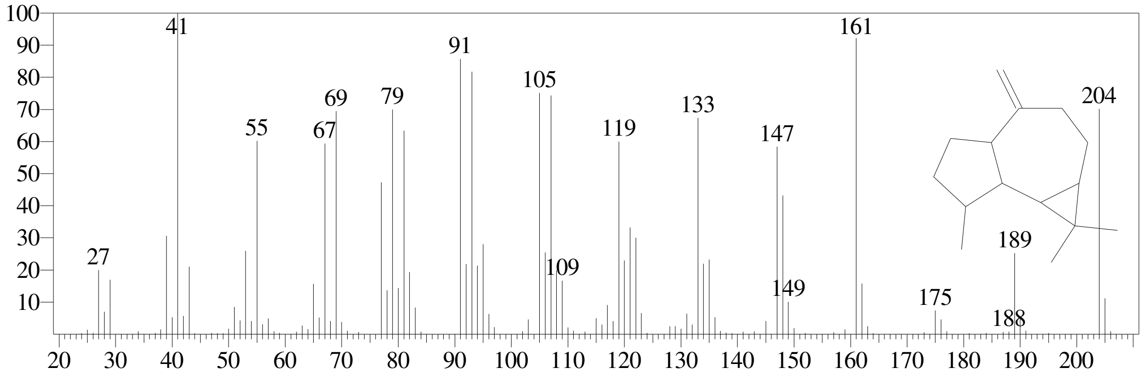

<< Target >>
Line#:13   R.Time:26.950(Scan#:2935)  MassPeaks:57
RawMode:Averaged 26.942-26.958(2934-2936) BasePeak:41.00(29600)
BG Mode:None  Group 1 - Event 1  Scan

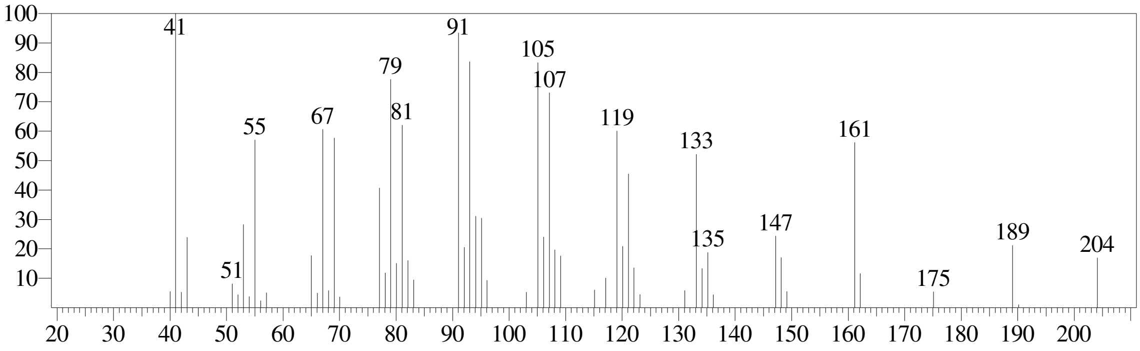


Hit#:5  Entry:24868  Library:NIST23s.lib
SI:93  Formula:C15H24  CAS:25246-27-9  MolWeight:204  RetIndex:1424
CompName:Alloaromadendrene $$ 1H-Cycloprop[e]azulene, decahydro-1,1,7-trimethyl-4-methylene-, [1aR-(1a.alpha.,4a

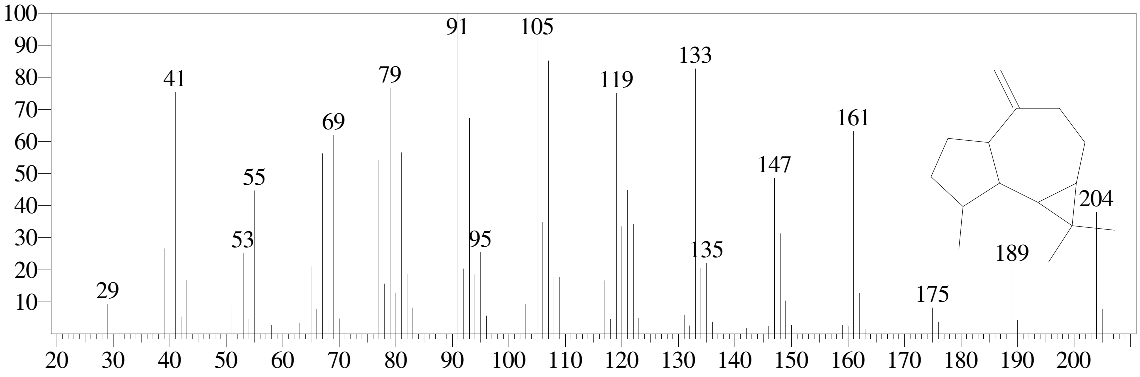

<< Target >>
Line#:14 R.Time:27.542(Scan#:3006) MassPeaks:23
RawMode:Averaged 27.533-27.550(3005-3007) BasePeak:93.10(19326)
BG Mode:None Group 1 - Event 1 Scan

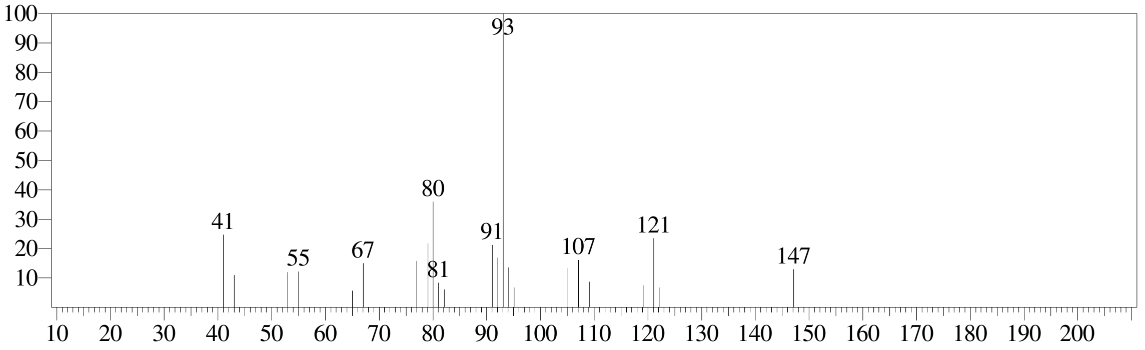


Hit#:1 Entry:24887 Library:NIST23s.lib
SI:90 Formula:C15H24 CAS:6753-98-6 MolWeight:204 RetIndex:1455
CompName:Humulene $$ .alpha.-Caryophyllene $$ 1,4,8-Cycloundecatriene, 2,6,6,9-tetramethyl-, (E,E,E)- $$ .alpha.-Hu

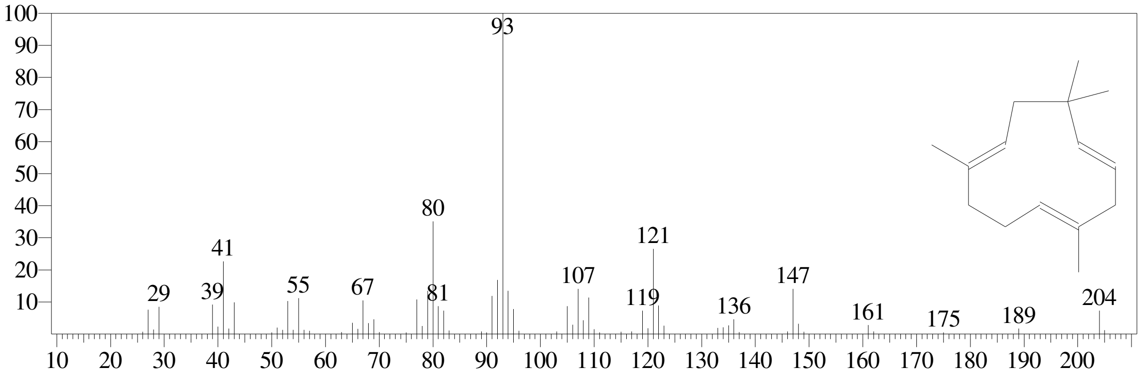


Hit#:2 Entry:62814 Library:NIST23-1.lib
SI:90 Formula:C15H24 CAS:6753-98-6 MolWeight:204 RetIndex:1455
CompName:Humulene $$ .alpha.-Caryophyllene $$ 1,4,8-Cycloundecatriene, 2,6,6,9-tetramethyl-, (E,E,E)- $$ .alpha.-Hu

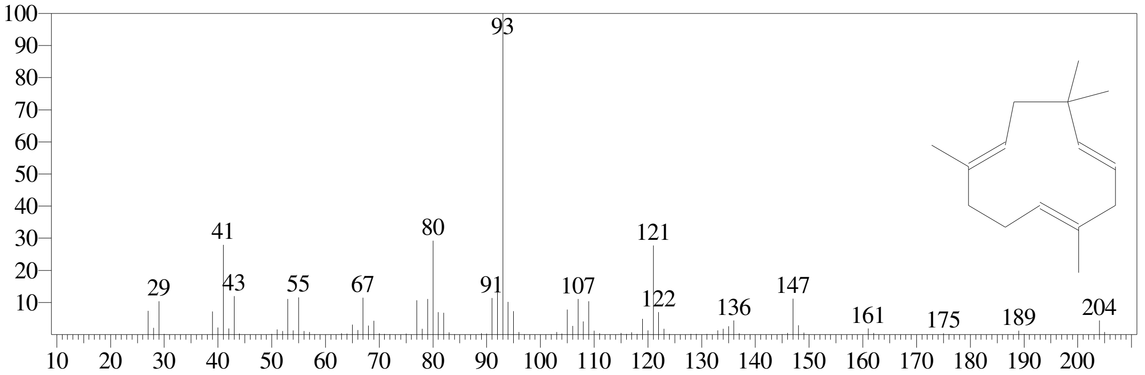

<< Target >>
Line#:14 R.Time:27.542(Scan#:3006) MassPeaks:23
RawMode:Averaged 27.533-27.550(3005-3007) BasePeak:93.10(19326)
BG Mode:None Group 1 - Event 1 Scan

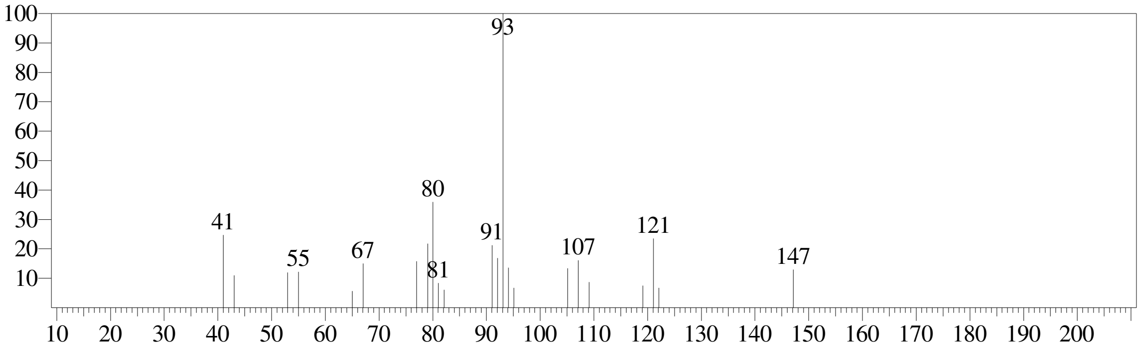

Hit#:3 Entry:24886 Library:NIST23s.lib
SI:90 Formula:C15H24 CAS:6753-98-6 MolWeight:204 RetIndex:1455
CompName:Humulene $$ .alpha.-Caryophyllene $$ 1,4,8-Cycloundecatriene, 2,6,6,9-tetramethyl-, (E,E,E)- $$ .alpha.-Hu

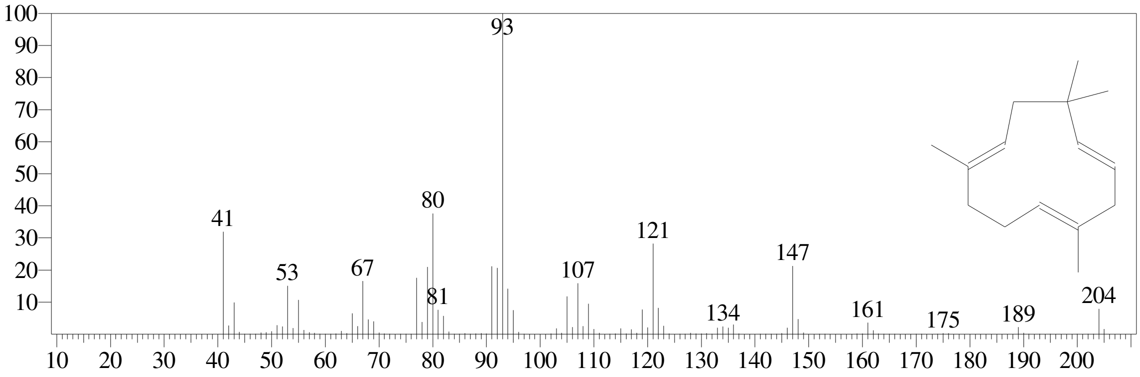

Hit#:4 Entry:24888 Library:NIST23s.lib
SI:89 Formula:C15H24 CAS:6753-98-6 MolWeight:204 RetIndex:1455
CompName:Humulene $$ .alpha.-Caryophyllene $$ 1,4,8-Cycloundecatriene, 2,6,6,9-tetramethyl-, (E,E,E)- $$ .alpha.-Hu

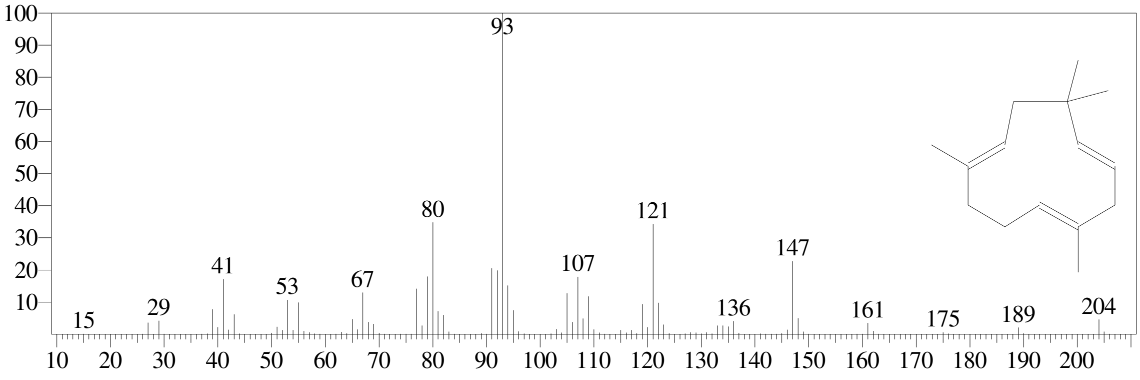

<< Target >>
Line#:14   R.Time:27.542(Scan#:3006)  MassPeaks:23
RawMode:Averaged 27.533-27.550(3005-3007) BasePeak:93.10(19326)
BG Mode:None  Group 1 - Event 1  Scan

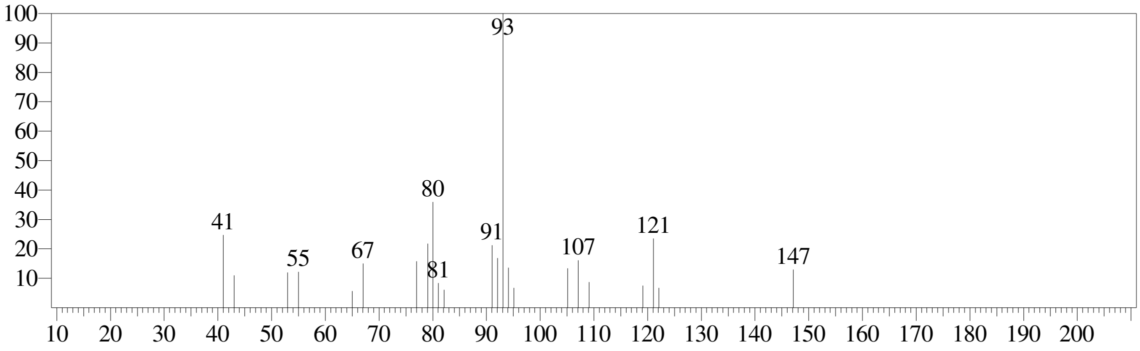


Hit#:5  Entry:24877  Library:NIST23s.lib
SI:88  Formula:C15H24  CAS:6753-98-6  MolWeight:204  RetIndex:1455
CompName:Humulene $$ .alpha.-Caryophyllene $$ 1,4,8-Cycloundecatriene, 2,6,6,9-tetramethyl-, (E,E,E)- $$ .alpha.-Hu

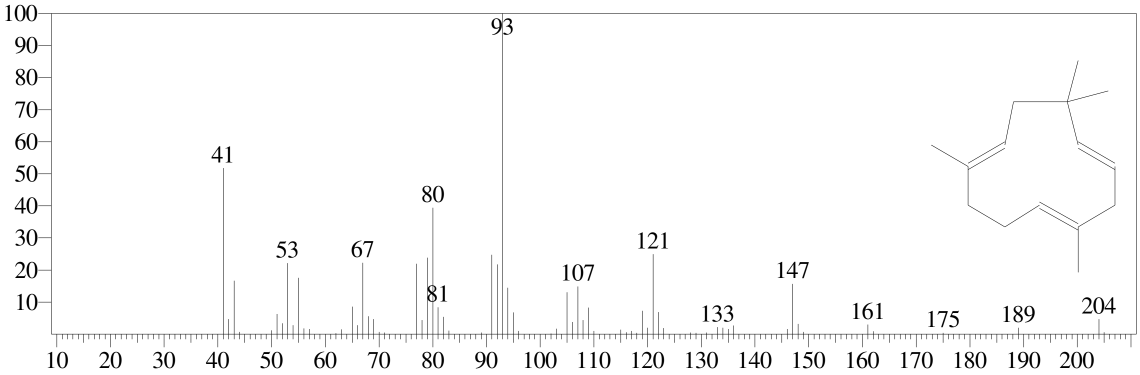

<< Target >>
Line#:15   R.Time:27.850(Scan#:3043)  MassPeaks:16
RawMode:Averaged 27.842-27.858(3042-3044) BasePeak:91.05(3415)
BG Mode:None  Group 1 - Event 1  Scan

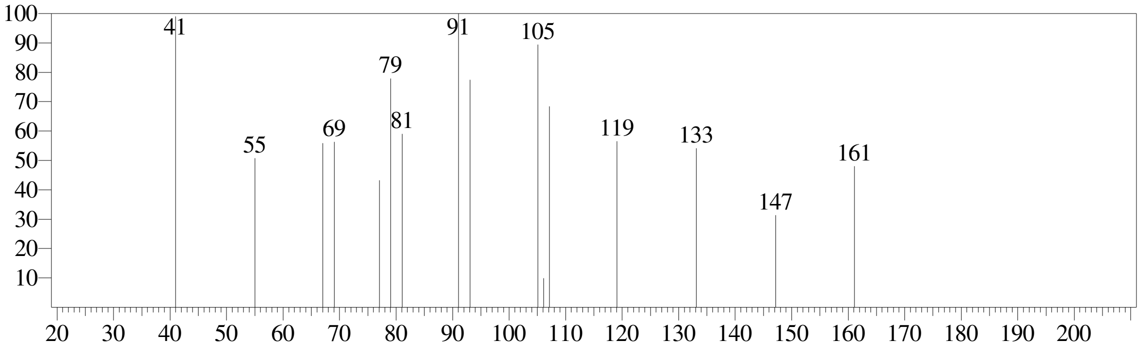


Hit#:1  Entry:62757  Library:NIST23-1.lib
SI:77  Formula:C15H24  CAS:28973-99-1  MolWeight:204  RetIndex:1509
CompName:(Z,Z)-.alpha.-Farnesene $$ (3Z,6Z)-3,7,11-Trimethyl-1,3,6,10-dodecatetraene  # $$

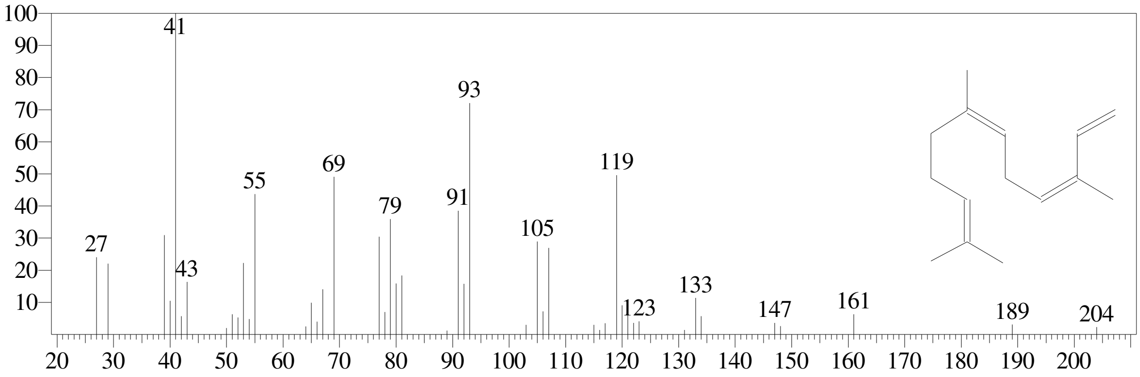


Hit#:2  Entry:24823  Library:NIST23s.lib
SI:77  Formula:C15H24  CAS:26560-14-5  MolWeight:204  RetIndex:1509
CompName:1,3,6,10-Dodecatetraene, 3,7,11-trimethyl-, (Z,E)- $$ (Z,E)-.alpha.-Farnesene $$ (3Z,6E)-3,7,11-Trimethyl-1.

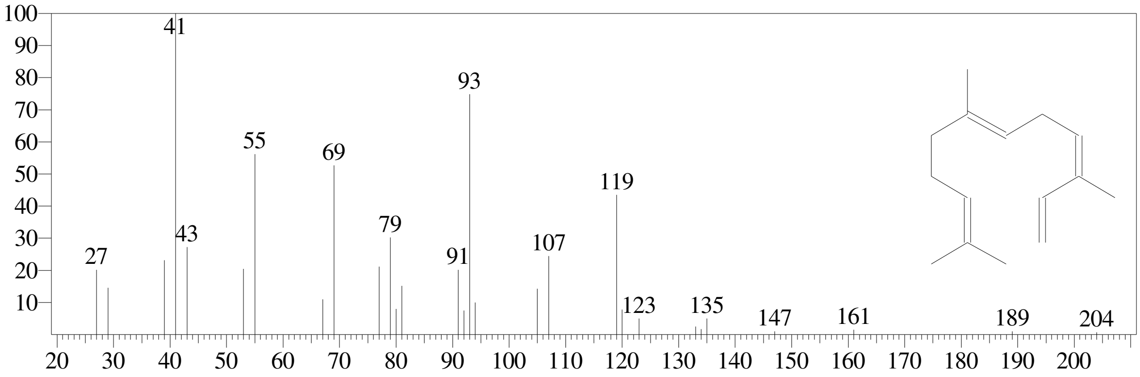

<< Target >>
Line#:15  R.Time:27.850(Scan#:3043)  MassPeaks:16
RawMode:Averaged 27.842-27.858(3042-3044) BasePeak:91.05(3415)
BG Mode:None  Group 1 - Event 1  Scan

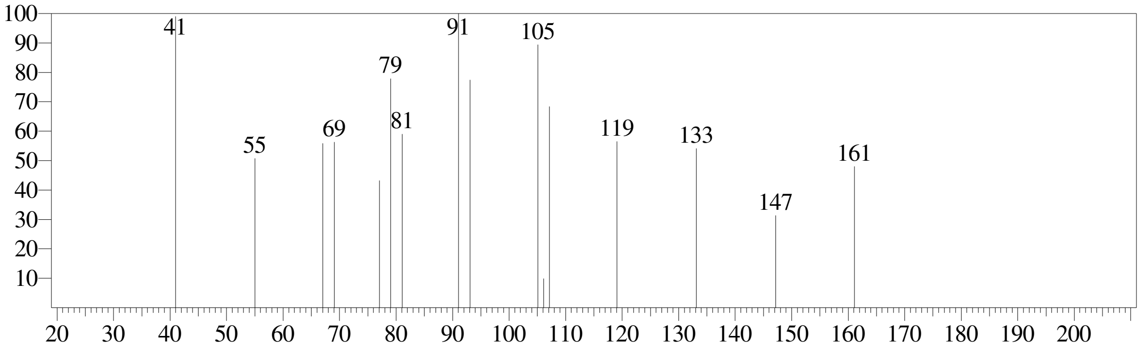


Hit#:3  Entry:62671  Library:NIST23-1.lib
SI:77  Formula:C13H20N2  CAS:0-00-0  MolWeight:204  RetIndex:1704
CompName:1,4-Methanocycloocta[d]pyridazine, 1,4,4a,5,6,9,10,10a-octahydro-11,11-dimethyl-, (1.alpha.,4.alpha.,4a.alpł

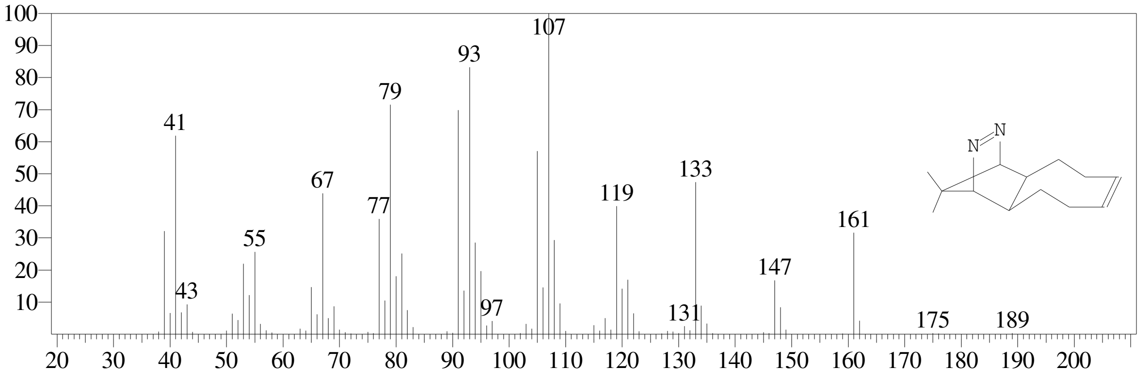


Hit#:4  Entry:25046  Library:NIST23s.lib
SI:76  Formula:C15H24  CAS:23986-74-5  MolWeight:204  RetIndex:1478
CompName:Germacrene D $$ (S,1Z,6Z)-8-Isopropyl-1-methyl-5-methylenecyclodeca-1,6-diene $$ D-Germacrene $$ 1(1

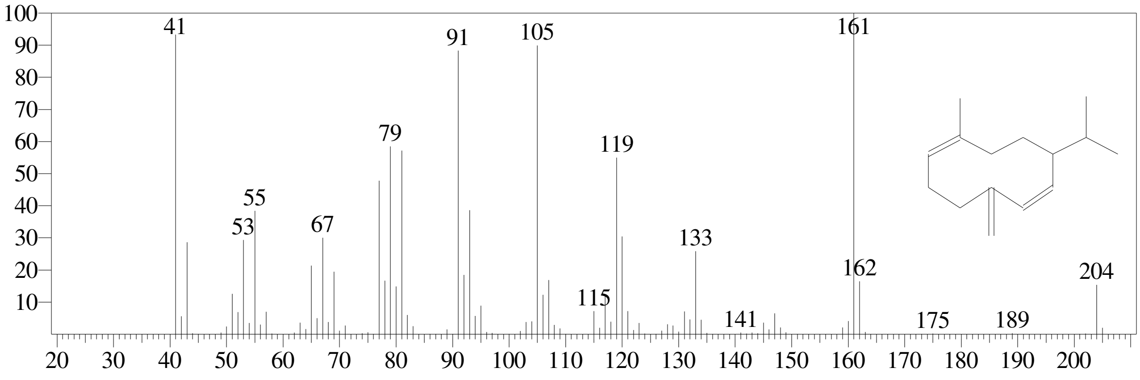

<< Target >>
Line#:15   R.Time:27.850(Scan#:3043)  MassPeaks:16
RawMode:Averaged 27.842-27.858(3042-3044) BasePeak:91.05(3415)
BG Mode:None  Group 1 - Event 1  Scan

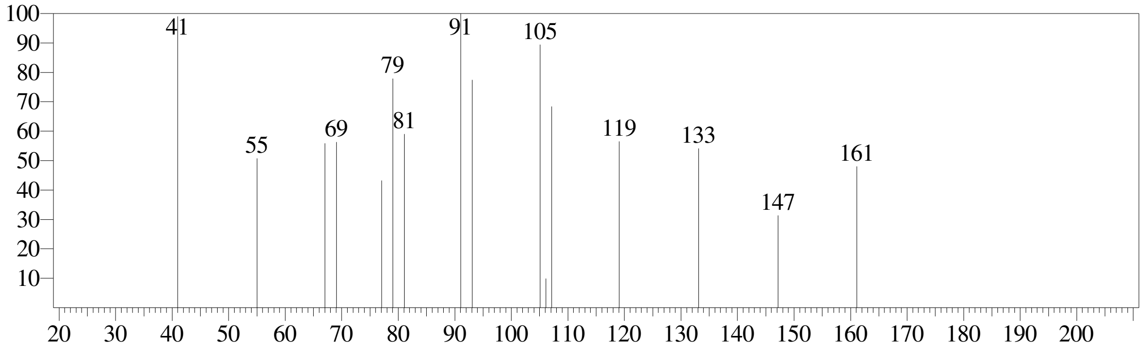


Hit#:5  Entry:24814  Library:NIST23s.lib
SI:76  Formula:C15H24  CAS:25246-27-9  MolWeight:204  RetIndex:1424
CompName:Alloaromadendrene $$ 1H-Cycloprop[e]azulene, decahydro-1,1,7-trimethyl-4-methylene-, [1aR-(1a.alpha.,4a

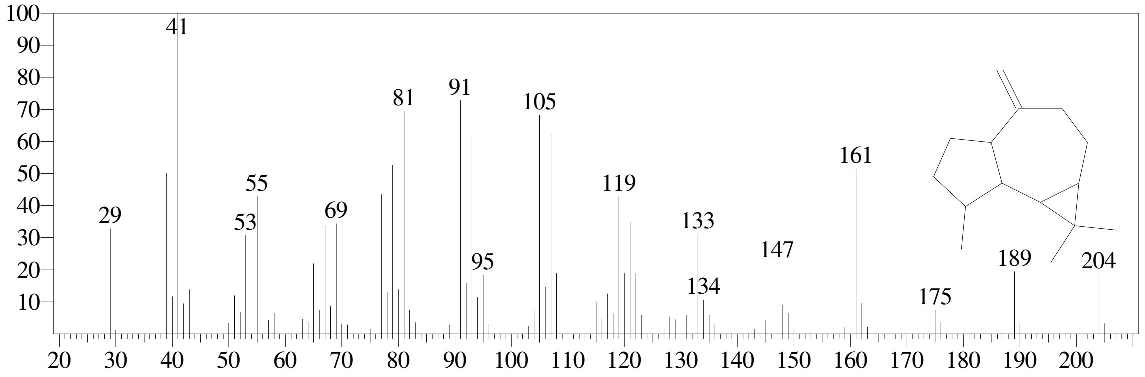

<< Target >>
Line#:16   R.Time:28.492(Scan#:3120)  MassPeaks:9
RawMode:Averaged 28.483-28.500(3119-3121) BasePeak:105.10(2171)
BG Mode:None  Group 1 - Event 1  Scan

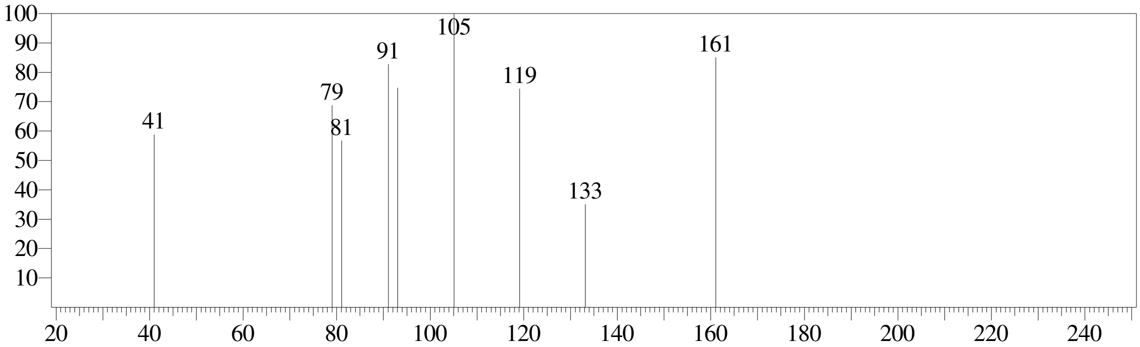


Hit#:1  Entry:25079  Library:NIST23s.lib
SI:72  Formula:C15H24  CAS:157477-72-0  MolWeight:204  RetIndex:1487
CompName:cis-Muurola-4(15),5-diene $$ (1S,4S,4aR)-1-Isopropyl-4-methyl-7-methylene-1,2,3,4,4a,5,6,7-octahydronaph

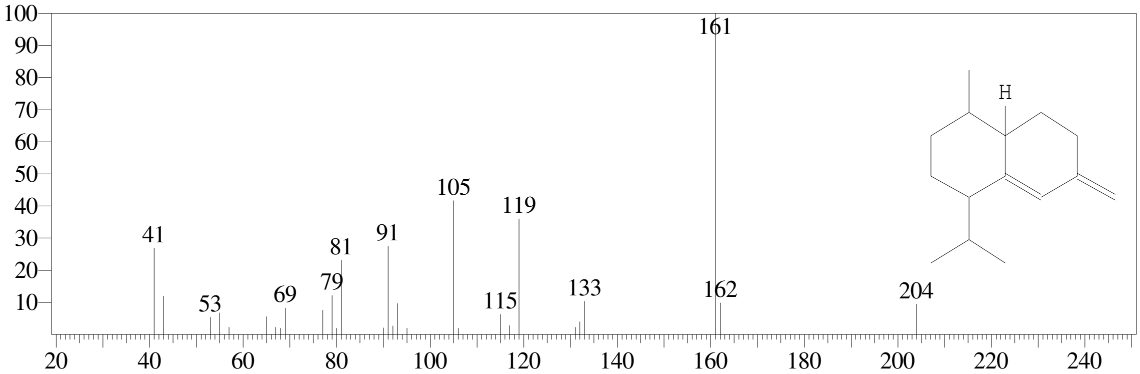


Hit#:2  Entry:25084  Library:NIST23s.lib
SI:71  Formula:C15H24  CAS:157374-44-2  MolWeight:204  RetIndex:1449
CompName:cis-muurola-3,5-diene

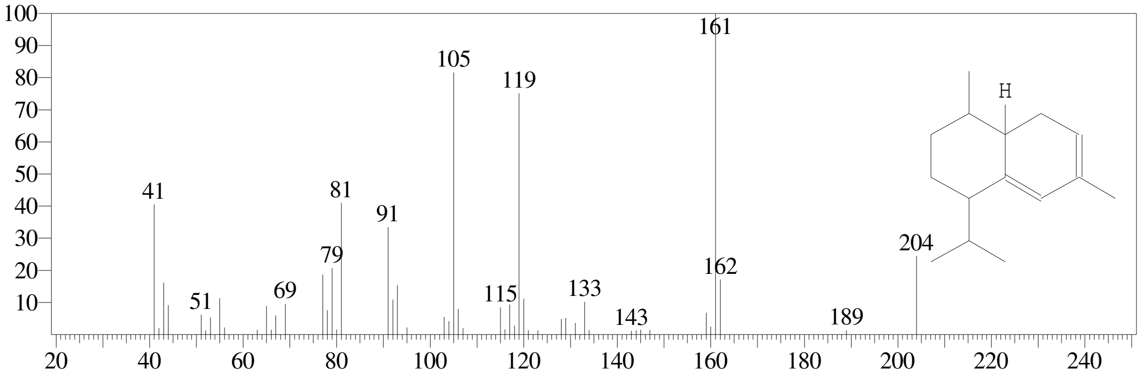

<< Target >>
Line#:16   R.Time:28.492(Scan#:3120)  MassPeaks:9
RawMode:Averaged 28.483-28.500(3119-3121) BasePeak:105.10(2171)
BG Mode:None  Group 1 - Event 1  Scan

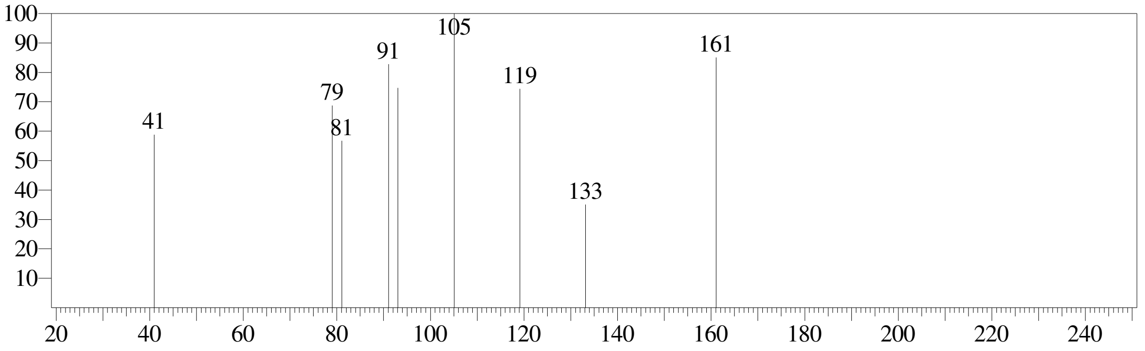


Hit#:3  Entry:31431  Library:NIST23s.lib
SI:71  Formula:C12H17Br  CAS:57040-44-5  MolWeight:240  RetIndex:1581
CompName:Adamantane, 1-(2-bromoethenyl)- $$ 1-(2-Bromovinyl)-adamantane $$ 1-[(E)-2-Bromoethenyl]adamantane

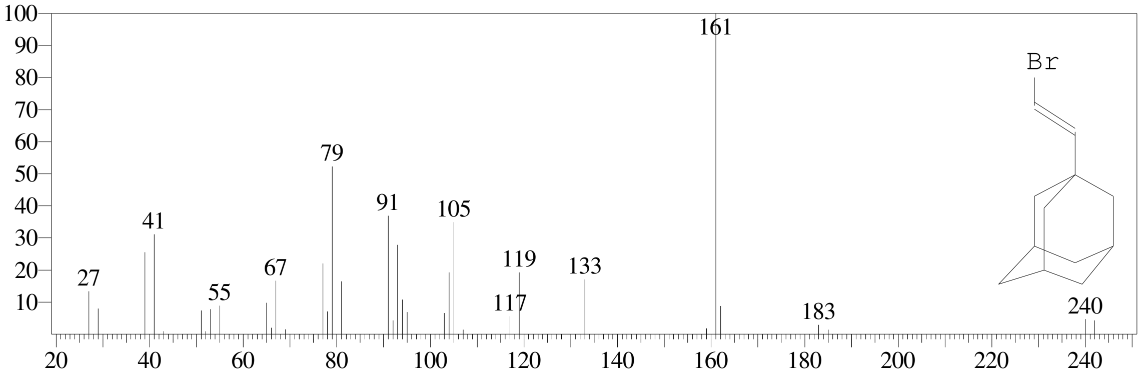


Hit#:4  Entry:102501  Library:NIST23-1.lib
SI:71  Formula:C15H23Cl  CAS:64275-44-1  MolWeight:238  RetIndex:1839
CompName:5,10-Pentadecadiyne, 1-chloro- $$ 1-Chloro-5,10-pentadecadiyne  # $$

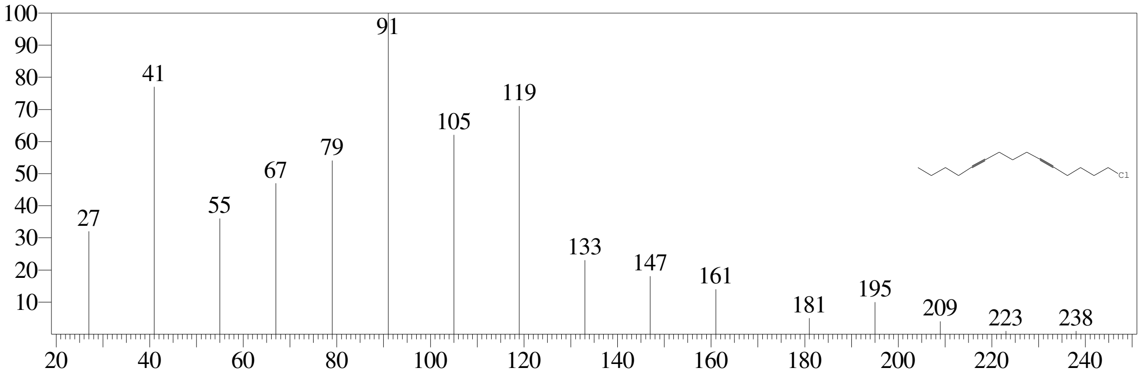

<< Target >>
Line#:16   R.Time:28.492(Scan#:3120)  MassPeaks:9
RawMode:Averaged 28.483-28.500(3119-3121) BasePeak:105.10(2171)
BG Mode:None  Group 1 - Event 1  Scan

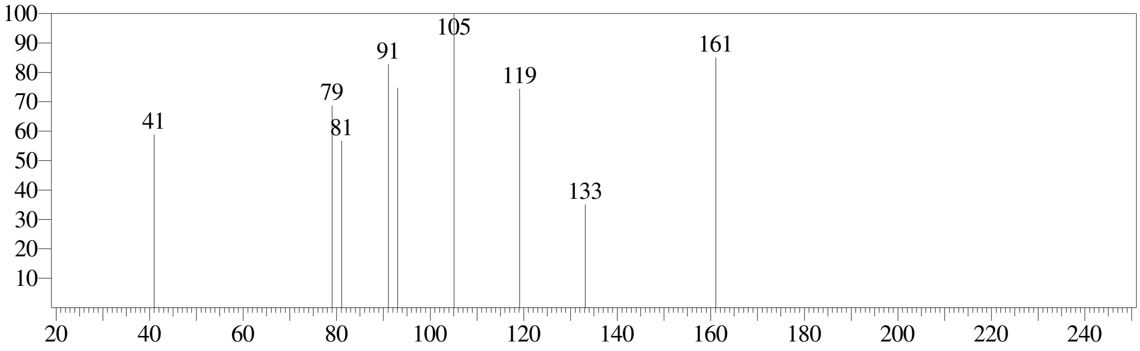


Hit#:5  Entry:62910  Library:NIST23-1.lib
SI:70  Formula:C15H24  CAS:3856-25-5  MolWeight:204  RetIndex:1407
CompName:Copaene $$ Tricyclo[4.4.0.02,7]dec-3-ene, 1,3-dimethyl-8-(1-methylethyl)-, stereoisomer $$ Tricyclo[4.4.0.0

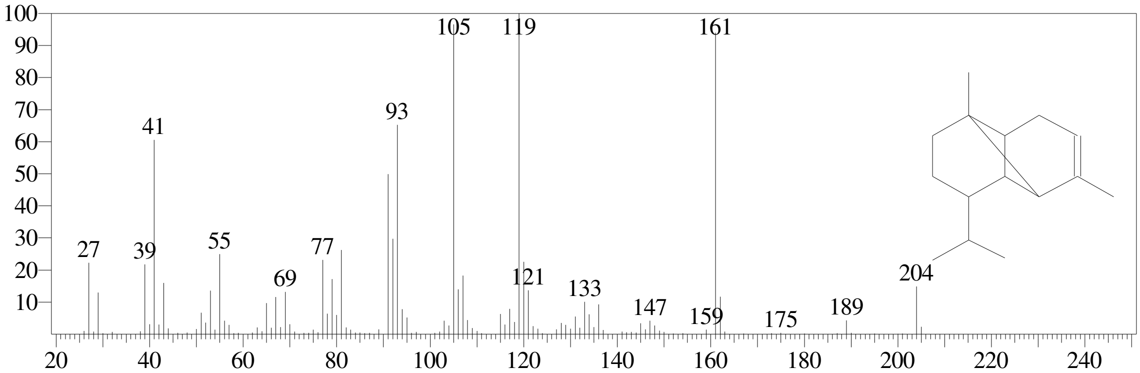

<< Target >>
Line#:17 R.Time:28.875(Scan#:3166) MassPeaks:40
RawMode:Averaged 28.867-28.883(3165-3167) BasePeak:93.05(10291)
BG Mode:Calc. from Peak Group 1 - Event 1 Scan

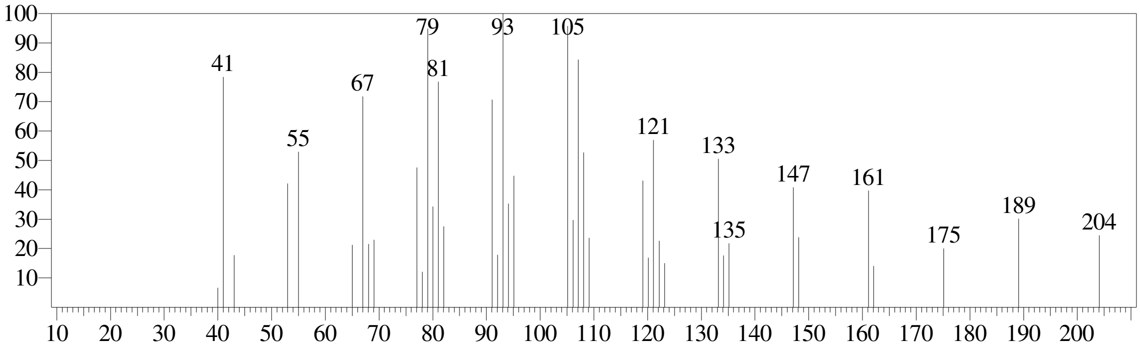


Hit#:1 Entry:24892 Library:NIST23s.lib
SI:95 Formula:C15H24 CAS:17066-67-0 MolWeight:204 RetIndex:1489
CompName:Naphthalene, decahydro-4a-methyl-1-methylene-7-(1-methylethenyl)-, [4aR-(4a.alpha.,7.alpha.,8a.beta.)]- $$

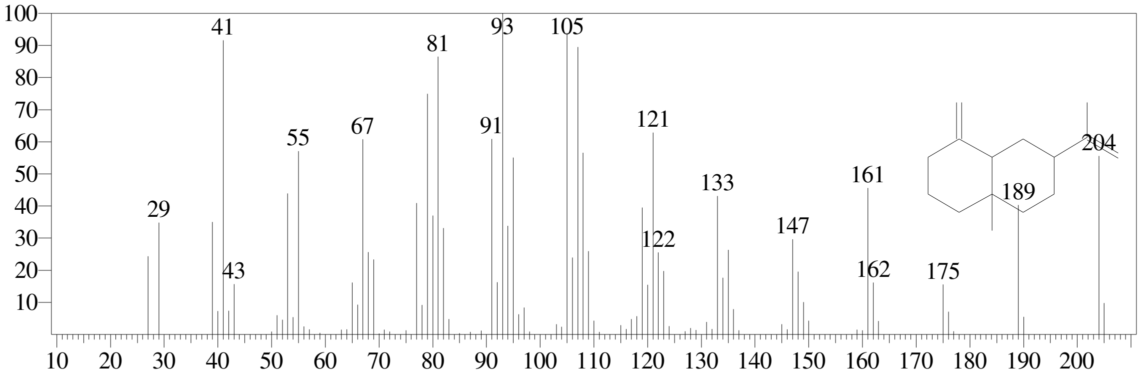


Hit#:2 Entry:24936 Library:NIST23s.lib
SI:94 Formula:C15H24 CAS:17066-67-0 MolWeight:204 RetIndex:1489
CompName:Naphthalene, decahydro-4a-methyl-1-methylene-7-(1-methylethenyl)-, [4aR-(4a.alpha.,7.alpha.,8a.beta.)]- $$

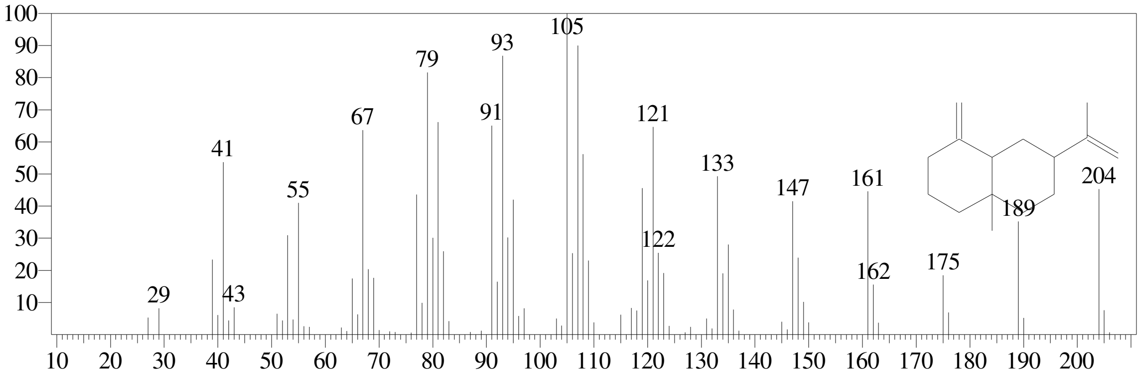

<< Target >>
Line#:17 R.Time:28.875(Scan#:3166) MassPeaks:40
RawMode:Averaged 28.867-28.883(3165-3167) BasePeak:93.05(10291)
BG Mode:Calc. from Peak Group 1 - Event 1 Scan

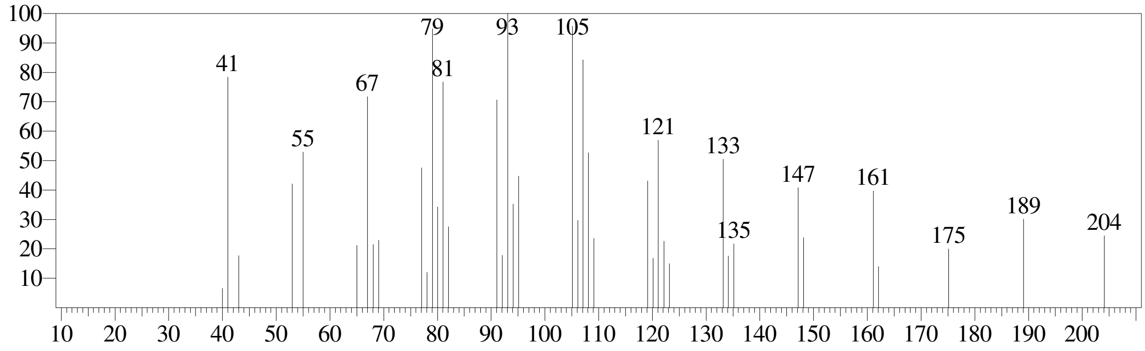

Hit#:3 Entry:62812 Library:NIST23-1.lib
SI:94 Formula:C15H24 CAS:0-00-0 MolWeight:204 RetIndex:1475
CompName:Bicyclo[5.3.0]decane, 2-methylene-5-(1-methylvinyl)-8-methyl- $$ 7-Isopropenyl-1-methyl-4-methylenedeca

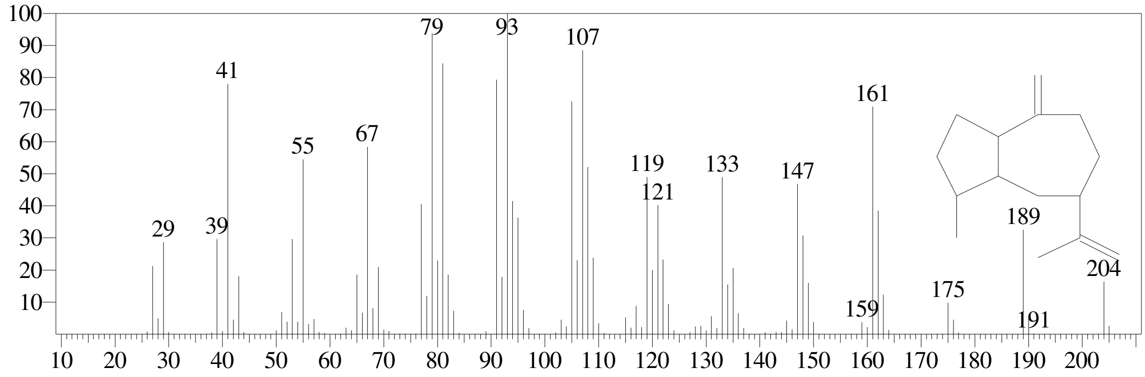

Hit#:4 Entry:62758 Library:NIST23-1.lib
SI:93 Formula:C15H24 CAS:17066-67-0 MolWeight:204 RetIndex:1489
CompName:Naphthalene, decahydro-4a-methyl-1-methylene-7-(1-methylethenyl)-, [4aR-(4a.alpha.,7.alpha.,8a.beta.)]- $$

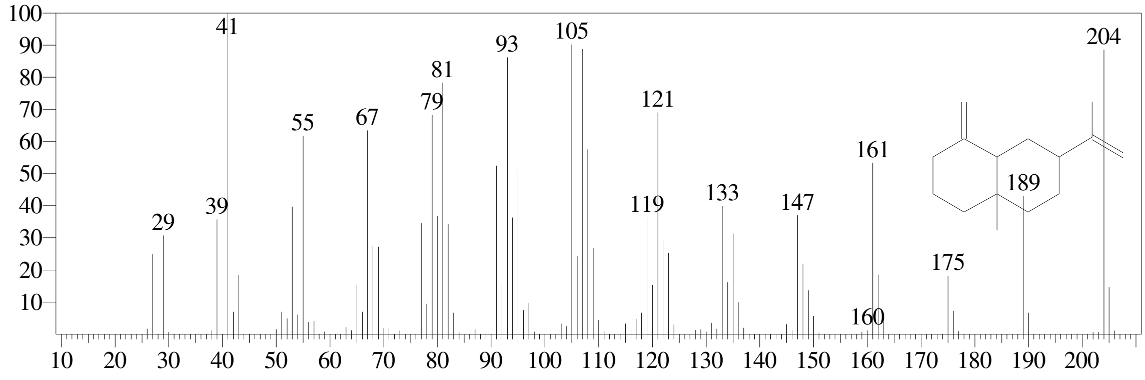

<< Target >>
Line#:17 R.Time:28.875(Scan#:3166) MassPeaks:40
RawMode:Averaged 28.867-28.883(3165-3167) BasePeak:93.05(10291)
BG Mode:Calc. from Peak Group 1 - Event 1 Scan

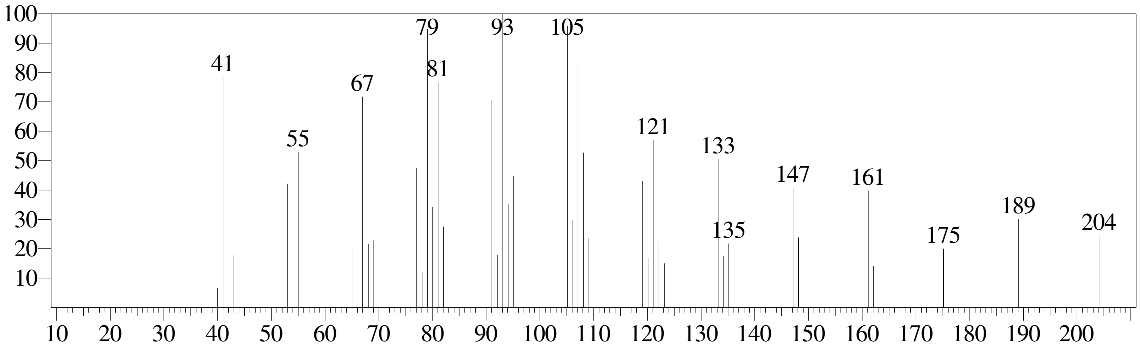


Hit#:5 Entry:62866 Library:NIST23-1.lib
SI:91 Formula:C15H24 CAS:10219-75-7 MolWeight:204 RetIndex:1496
CompName:Naphthalene, 1,2,3,5,6,7,8,8a-octahydro-1,8a-dimethyl-7-(1-methylethenyl)-, [1S-(1.alpha.,7.alpha.,8a.alpha.)

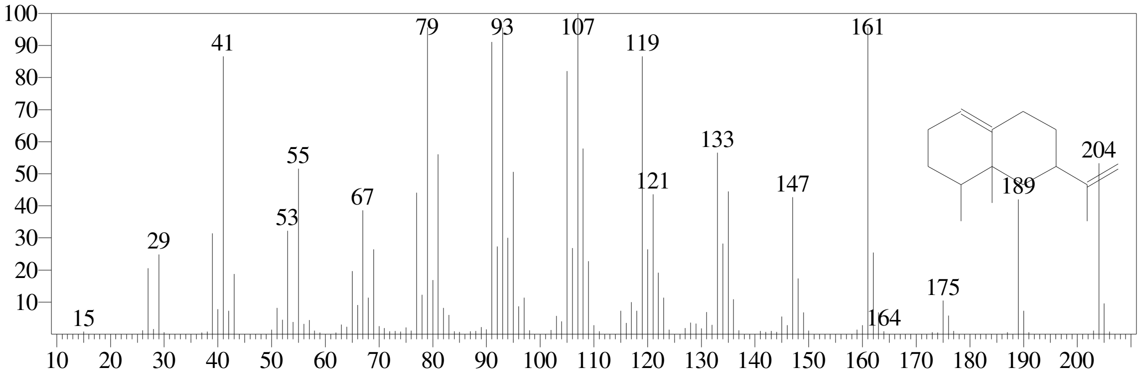

<< Target >>
Line#:18   R.Time:28.975(Scan#:3178)  MassPeaks:19
RawMode:Averaged 28.967-28.983(3177-3179) BasePeak:105.10(2631)
BG Mode:Calc. from Peak  Group 1 - Event 1  Scan

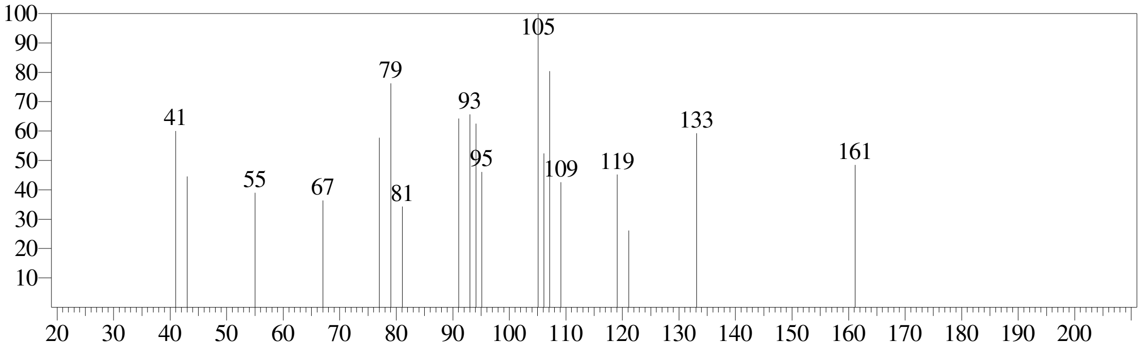


Hit#:1  Entry:62671  Library:NIST23-1.lib
SI:79  Formula:C13H20N2  CAS:0-00-0  MolWeight:204  RetIndex:1704
CompName:1,4-Methanocycloocta[d]pyridazine, 1,4,4a,5,6,9,10,10a-octahydro-11,11-dimethyl-, (1.alpha.,4.alpha.,4a.alph

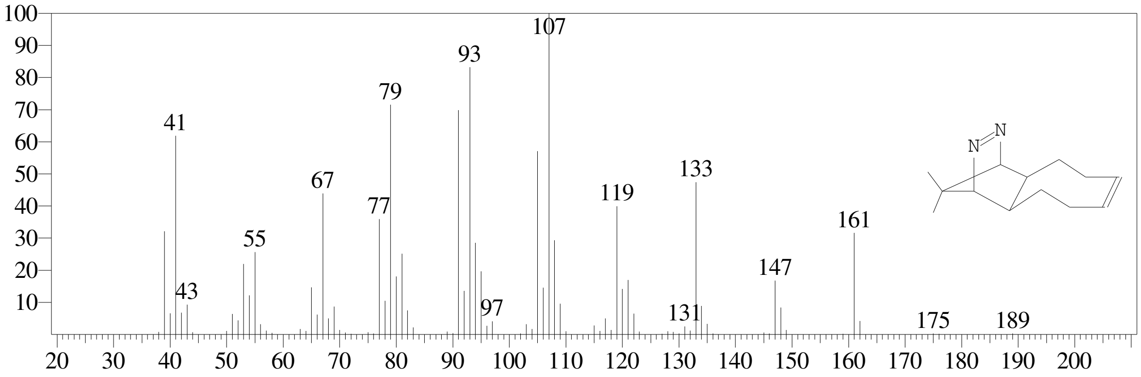


Hit#:2  Entry:37014  Library:NIST23-1.lib
SI:77  Formula:C13H20  CAS:0-00-0  MolWeight:176  RetIndex:1193
CompName:(+)-3-Carene, 2-.alpha.-isopropenyl- $$ 2-Isopropenyl-3,7,7-trimethylbicyclo[4.1.0]hept-3-ene  # $$

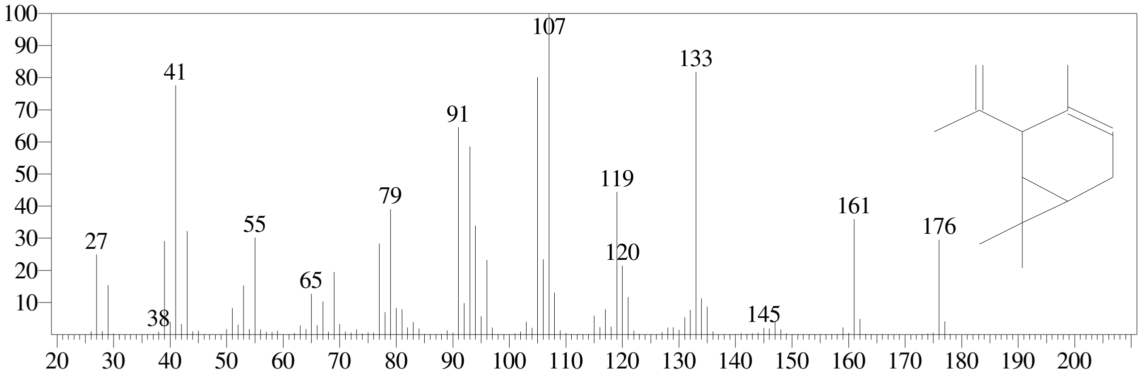

<< Target >>
Line#:18 R.Time:28.975(Scan#:3178) MassPeaks:19
RawMode:Averaged 28.967-28.983(3177-3179) BasePeak:105.10(2631)
BG Mode:Calc. from Peak Group 1 - Event 1 Scan

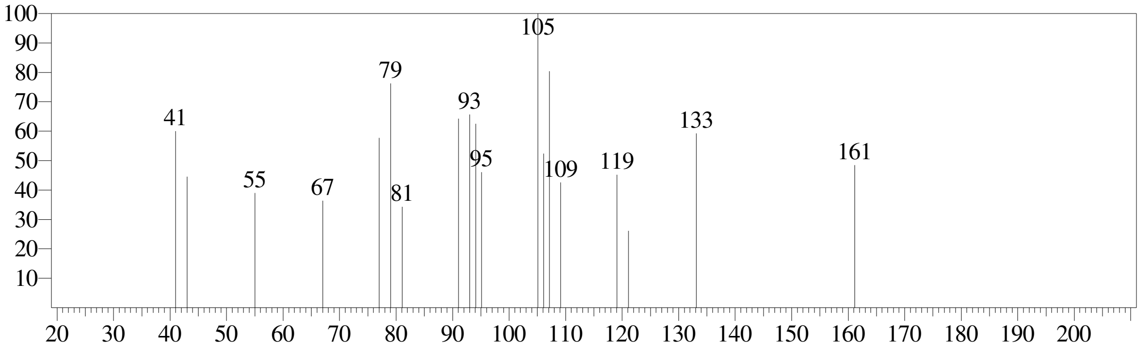


Hit#:3 Entry:62755 Library:NIST23-1.lib
SI:77 Formula:C15H24 CAS:85048-01-7 MolWeight:204 RetIndex:1436
CompName:Aromadendrane,dehydro-

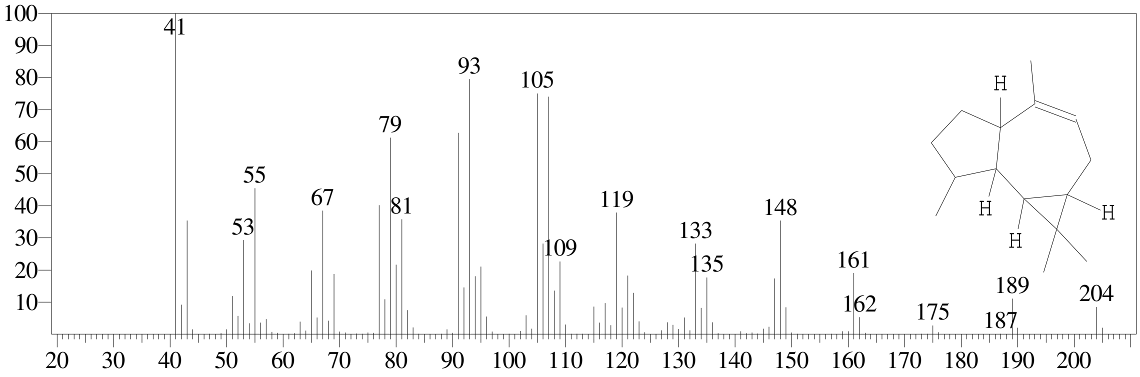


Hit#:4 Entry:24933 Library:NIST23s.lib
SI:77 Formula:C15H24 CAS:22469-52-9 MolWeight:204 RetIndex:1381
CompName:1,2,4-Metheno-1H-indene, octahydro-1,7a-dimethyl-5-(1-methylethyl)-, [1S-(1.alpha.,2.alpha.,3a.beta.,4.alpha

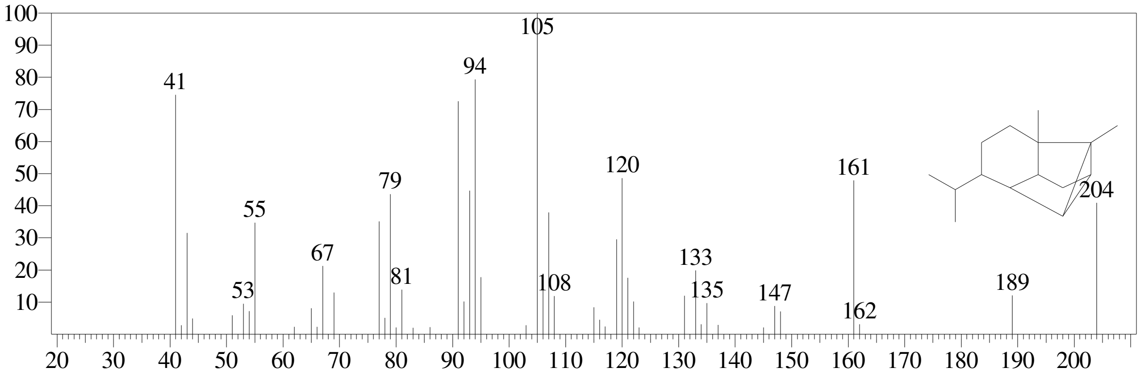

<< Target >>
Line#:18   R.Time:28.975(Scan#:3178)  MassPeaks:19
RawMode:Averaged 28.967-28.983(3177-3179) BasePeak:105.10(2631)
BG Mode:Calc. from Peak  Group 1 - Event 1  Scan

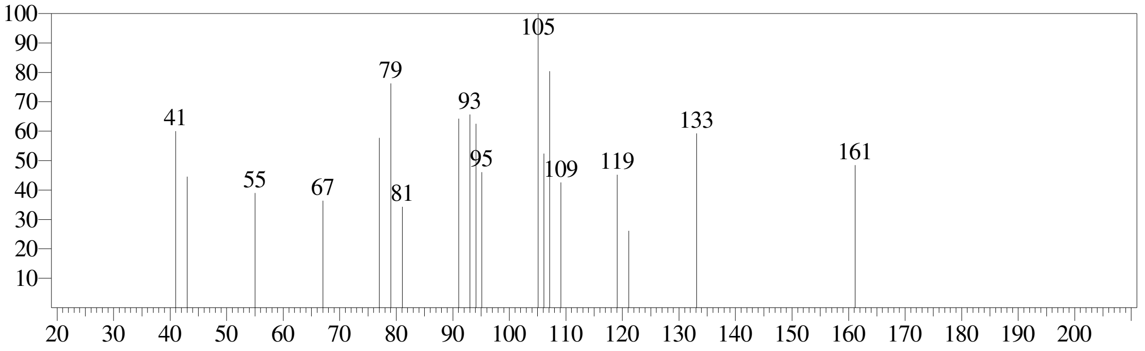


Hit#:5  Entry:24928  Library:NIST23s.lib
SI:77  Formula:C15H24  CAS:20085-19-2  MolWeight:204  RetIndex:1498
CompName:(1R,4aS,8aR)-1-Isopropyl-4,7-dimethyl-1,2,4a,5,6,8a-hexahydronaphthalene $$ .alpha.-Amorphene $$ Amor

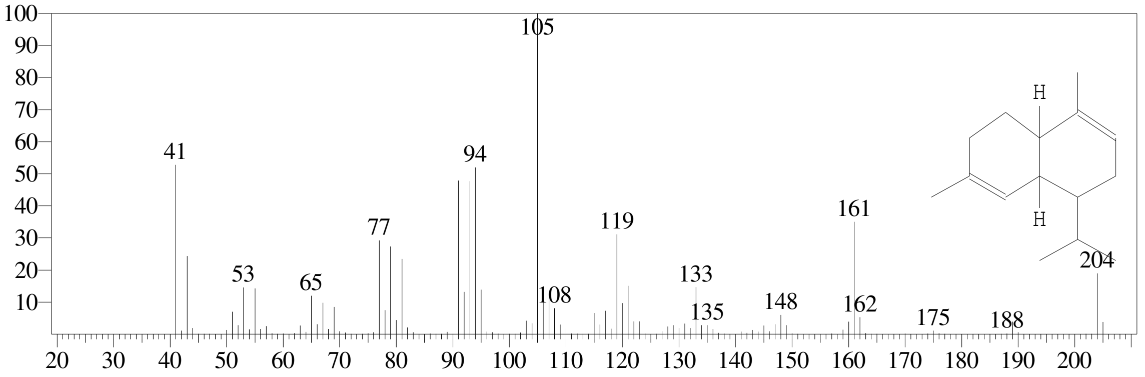

<< Target >>
Line#:19 R.Time:29.258(Scan#:3212) MassPeaks:68
RawMode:Averaged 29.250-29.267(3211-3213) BasePeak:107.10(47000)
BG Mode:None Group 1 - Event 1 Scan

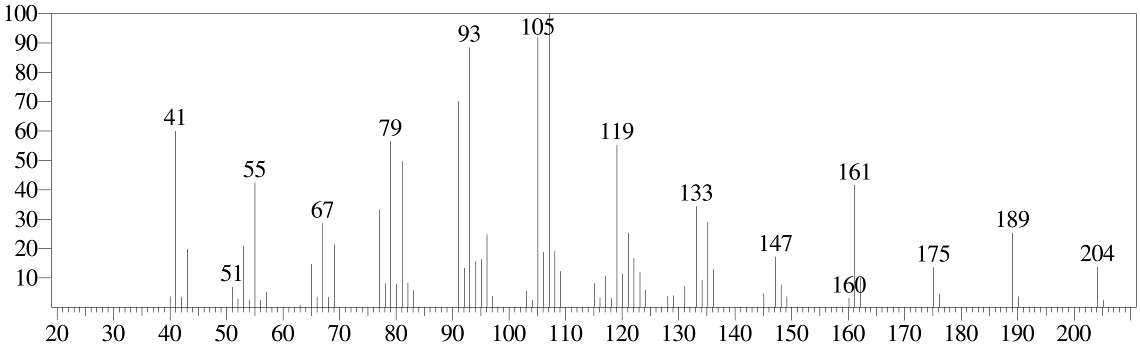


Hit#:1 Entry:24962 Library:NIST23s.lib
SI:95 Formula:C15H24 CAS:21747-46-6 MolWeight:204 RetIndex:1451
CompName:1H-Cycloprop[e]azulene, 1a,2,3,5,6,7,7a,7b-octahydro-1,1,4,7-tetramethyl-, [1aR-(1a.alpha.,7.alpha.,7a.beta.,

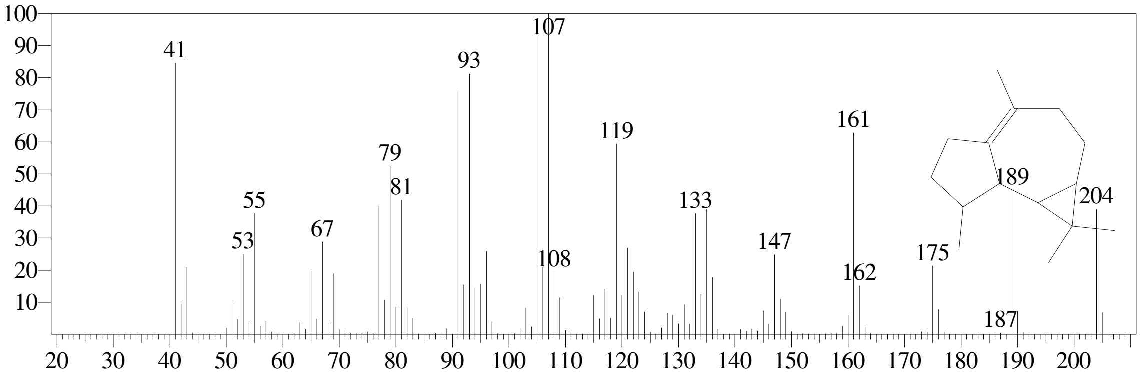


Hit#:2 Entry:24964 Library:NIST23s.lib
SI:93 Formula:C15H24 CAS:21747-46-6 MolWeight:204 RetIndex:1451
CompName:1H-Cycloprop[e]azulene, 1a,2,3,5,6,7,7a,7b-octahydro-1,1,4,7-tetramethyl-, [1aR-(1a.alpha.,7.alpha.,7a.beta.,

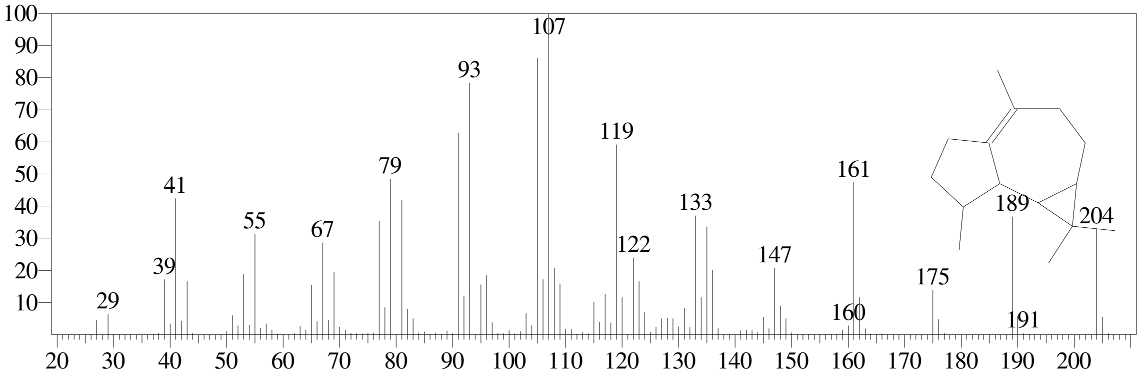

<< Target >>
Line#:19   R.Time:29.258(Scan#:3212)  MassPeaks:68
RawMode:Averaged 29.250-29.267(3211-3213) BasePeak:107.10(47000)
BG Mode:None  Group 1 - Event 1  Scan

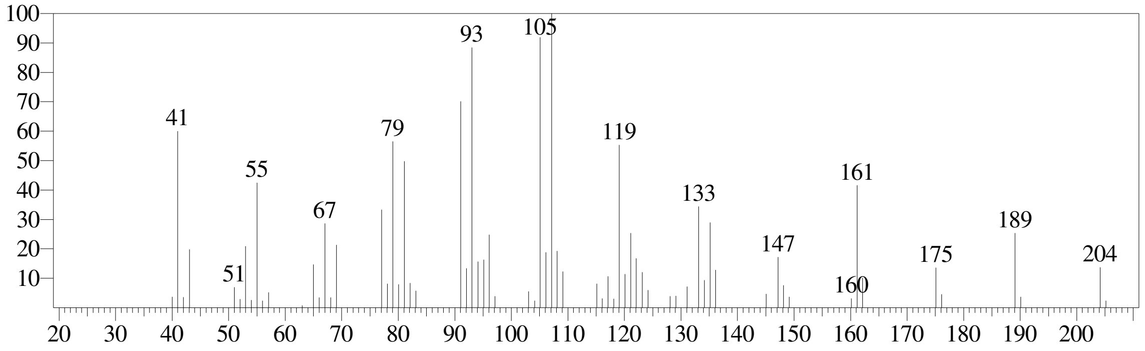


Hit#:3  Entry:62871  Library:NIST23-1.lib
SI:92  Formula:C15H24  CAS:21747-46-6  MolWeight:204  RetIndex:1451
CompName:1H-Cycloprop[e]azulene, 1a,2,3,5,6,7,7a,7b-octahydro-1,1,4,7-tetramethyl-, [1aR-(1a.alpha.,7.alpha.,7a.beta.,

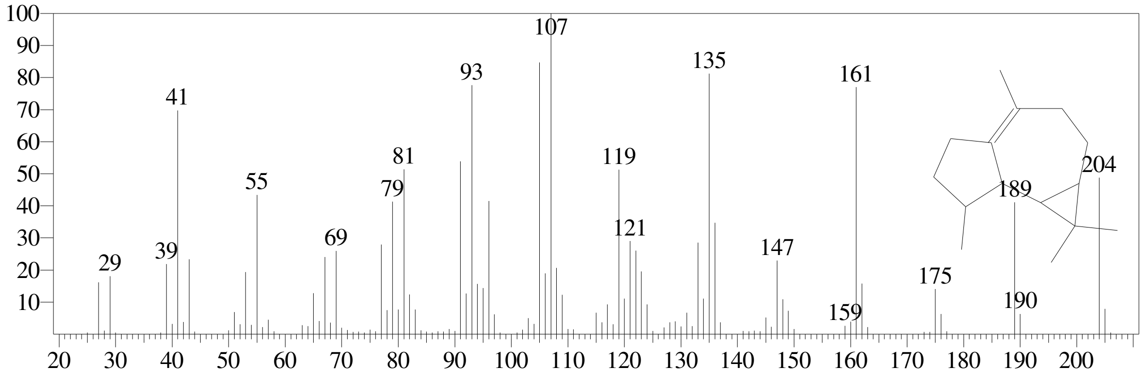


Hit#:4  Entry:24963  Library:NIST23s.lib
SI:92  Formula:C15H24  CAS:21747-46-6  MolWeight:204  RetIndex:1451
CompName:1H-Cycloprop[e]azulene, 1a,2,3,5,6,7,7a,7b-octahydro-1,1,4,7-tetramethyl-, [1aR-(1a.alpha.,7.alpha.,7a.beta.,

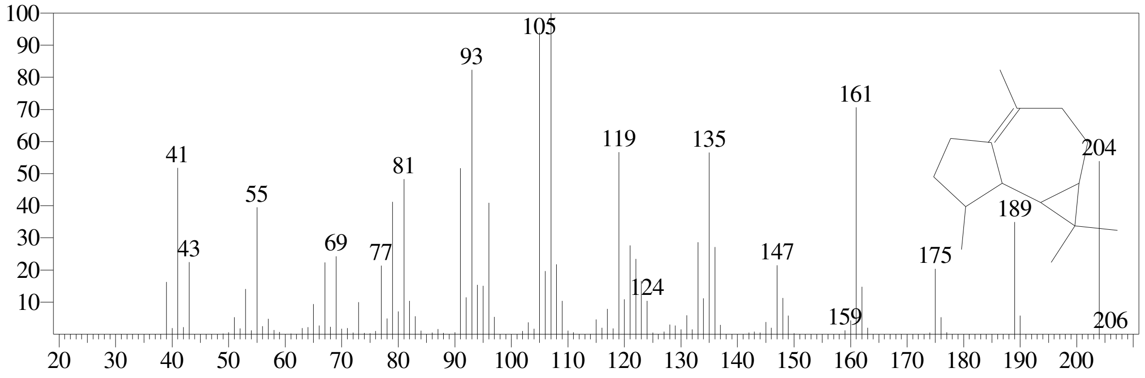

<< Target >>
Line#:19 R.Time:29.258(Scan#:3212) MassPeaks:68
RawMode:Averaged 29.250-29.267(3211-3213) BasePeak:107.10(47000)
BG Mode:None Group 1 - Event 1 Scan

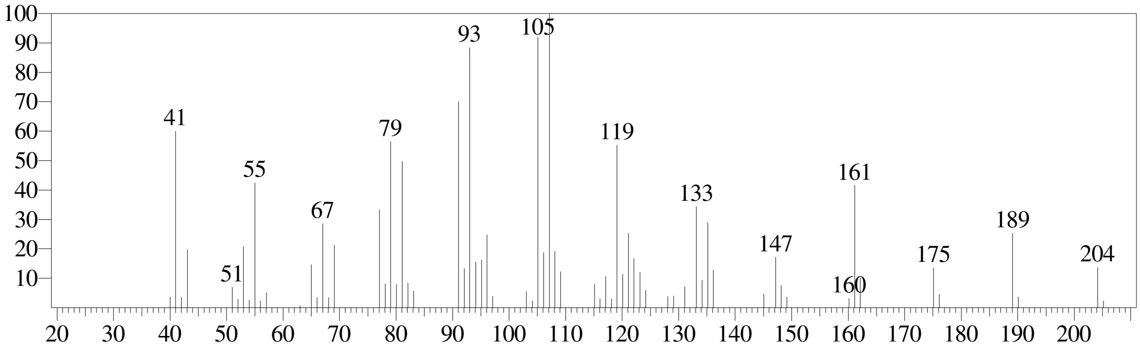


Hit#:5 Entry:24894 Library:NIST23s.lib
SI:92 Formula:C15H24 CAS:3691-11-0 MolWeight:204 RetIndex:1498
CompName:Azulene, 1,2,3,5,6,7,8,8a-octahydro-1,4-dimethyl-7-(1-methylethenyl)-, [1S-(1.alpha.,7.alpha.,8a.beta.)]- $$ (

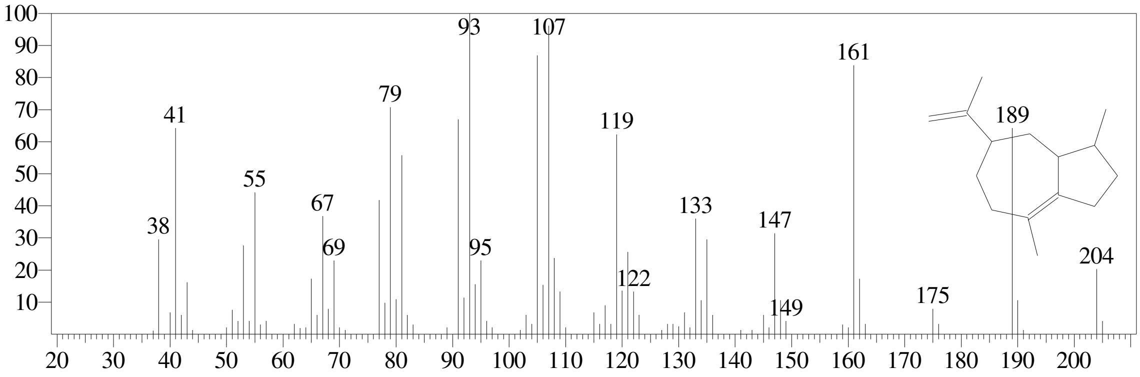

<< Target >>
Line#:20 R.Time:29.717(Scan#:3267) MassPeaks:10
RawMode:Averaged 29.708-29.725(3266-3268) BasePeak:105.10(2146)
BG Mode:None Group 1 - Event 1 Scan

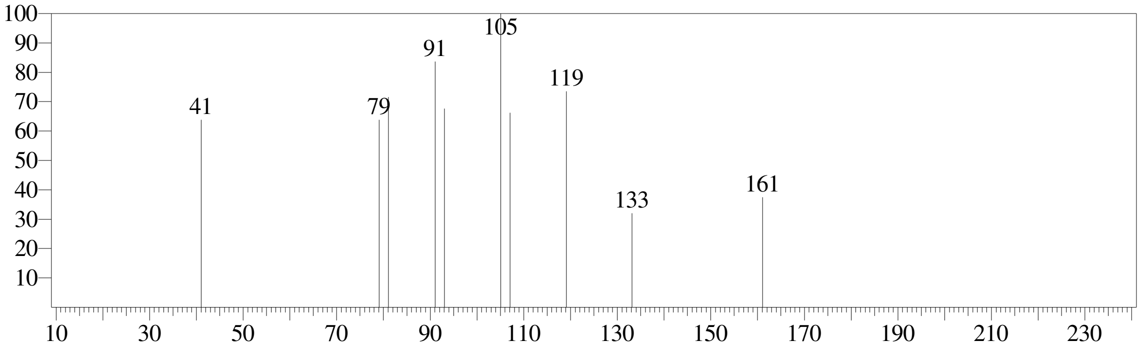


Hit#:1 Entry:62910 Library:NIST23-1.lib
SI:69 Formula:C15H24 CAS:3856-25-5 MolWeight:204 RetIndex:1407
CompName:Copaene $$ Tricyclo[4.4.0.02,7]dec-3-ene, 1,3-dimethyl-8-(1-methylethyl)-, stereoisomer $$ Tricyclo[4.4.0.0

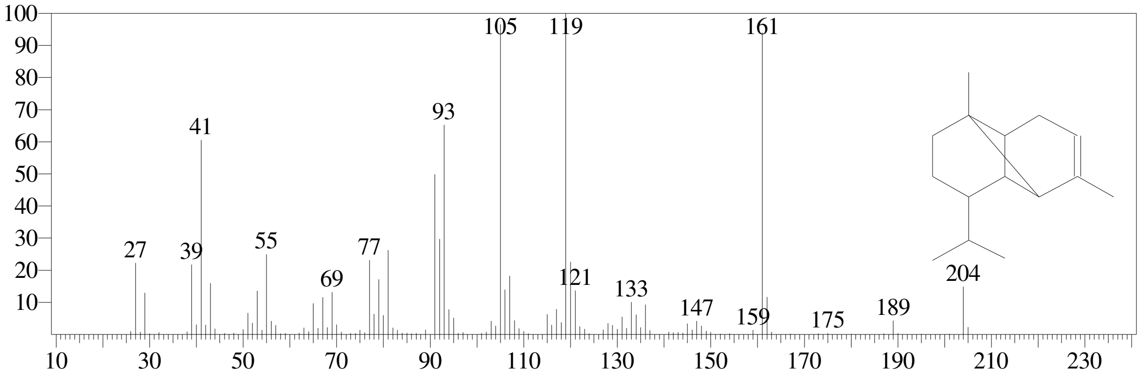


Hit#:2 Entry:62981 Library:NIST23-1.lib
SI:68 Formula:C15H24 CAS:23986-74-5 MolWeight:204 RetIndex:1478
CompName:Germacrene D $$ (S,1Z,6Z)-8-Isopropyl-1-methyl-5-methylenecyclodeca-1,6-diene $$ D-Germacrene $$ 1(1

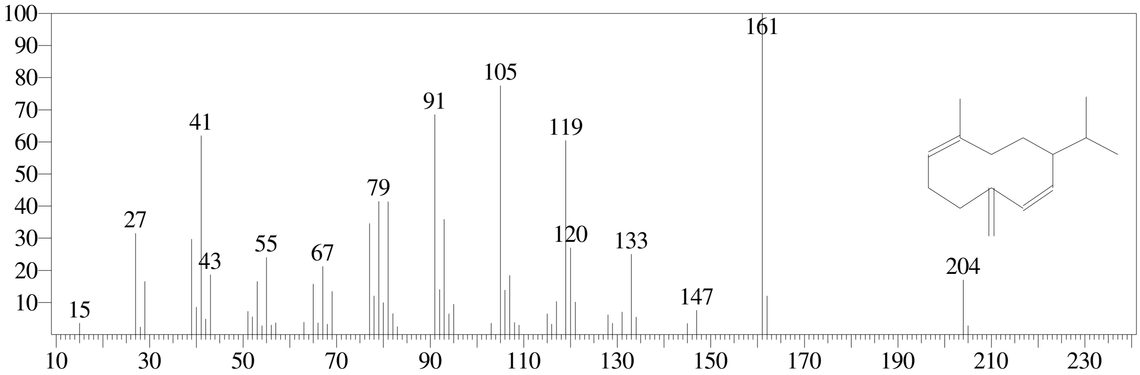

<< Target >>
Line#:20 R.Time:29.717(Scan#:3267) MassPeaks:10
RawMode:Averaged 29.708-29.725(3266-3268) BasePeak:105.10(2146)
BG Mode:None Group 1 - Event 1 Scan

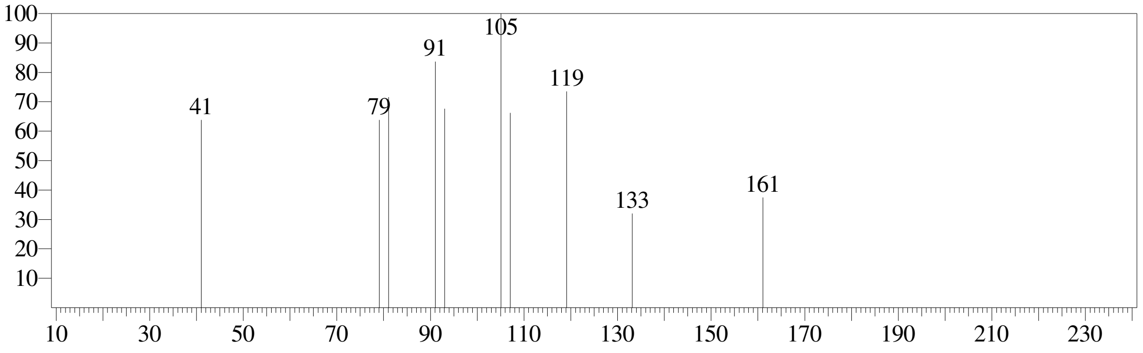


Hit#:3 Entry:24948 Library:NIST23s.lib
SI:68 Formula:C15H24 CAS:17699-14-8 MolWeight:204 RetIndex:1381
CompName:.alpha.-Cubebene $$ 1H-Cyclopenta[1,3]cyclopropa[1,2]benzene, 3a,3b,4,5,6,7-hexahydro-3,7-dimethyl-4-(1-

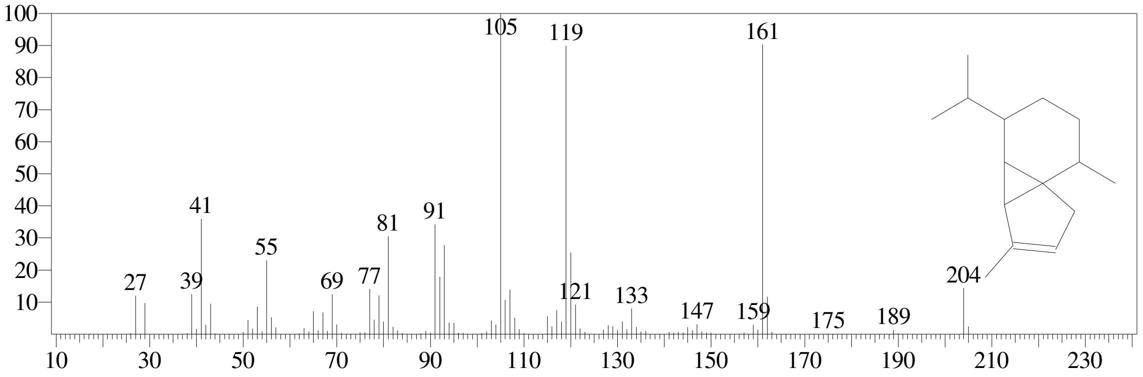


Hit#:4 Entry:102501 Library:NIST23-1.lib
SI:68 Formula:C15H23Cl CAS:64275-44-1 MolWeight:238 RetIndex:1839
CompName:5,10-Pentadecadiyne, 1-chloro- $$ 1-Chloro-5,10-pentadecadiyne # $$

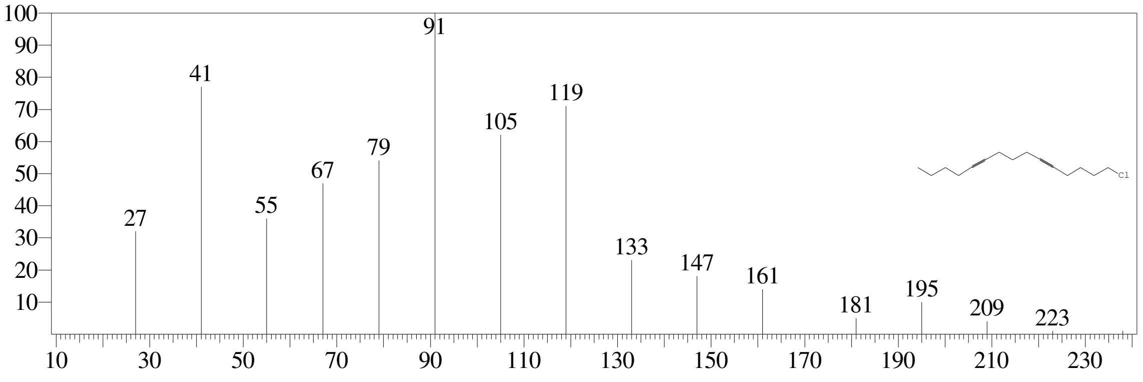

<< Target >>
Line#:20   R.Time:29.717(Scan#:3267)  MassPeaks:10
RawMode:Averaged 29.708-29.725(3266-3268) BasePeak:105.10(2146)
BG Mode:None  Group 1 - Event 1  Scan

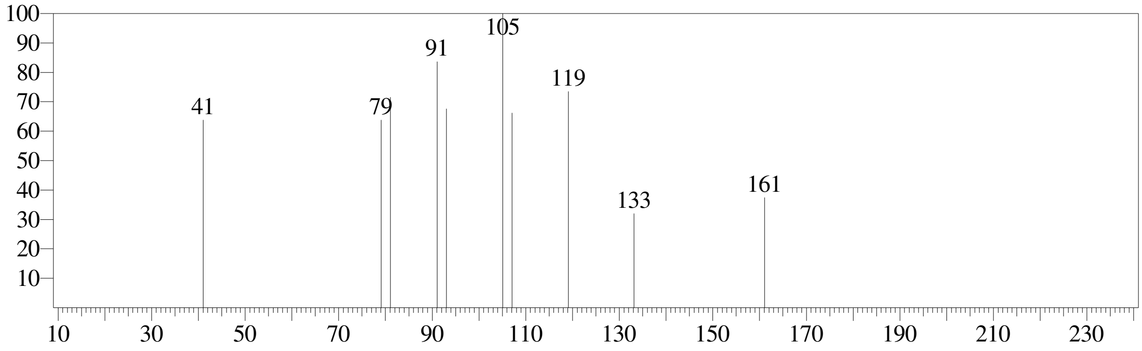


Hit#:5  Entry:25084  Library:NIST23s.lib
SI:68  Formula:C15H24  CAS:157374-44-2  MolWeight:204  RetIndex:1449
CompName:cis-muurola-3,5-diene

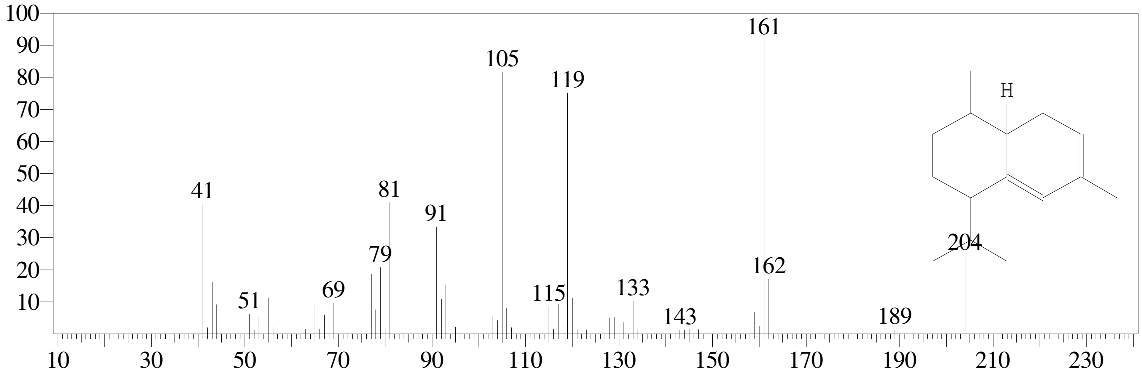

<< Target >>
Line#:21   R.Time:30.000(Scan#:3301)  MassPeaks:18
RawMode:Averaged 29.992-30.008(3300-3302) BasePeak:161.10(6161)
BG Mode:None  Group 1 - Event 1  Scan

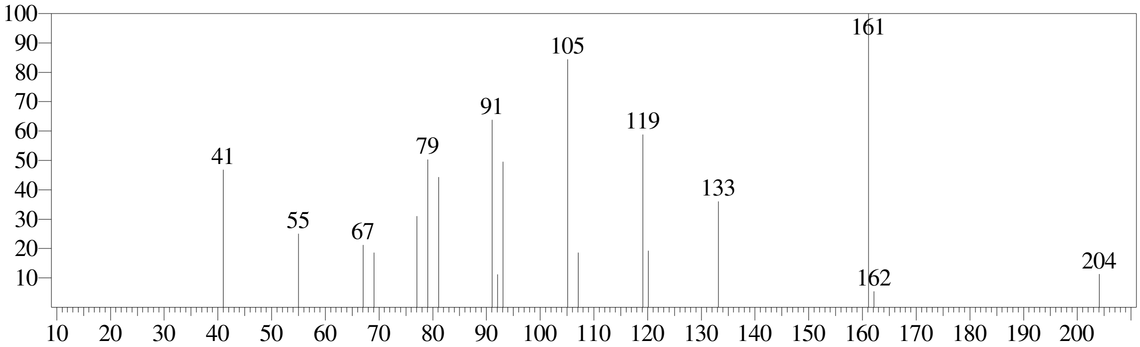


Hit#:1  Entry:62981  Library:NIST23-1.lib
SI:84  Formula:C15H24  CAS:23986-74-5  MolWeight:204  RetIndex:1478
CompName:Germacrene D $$ (S,1Z,6Z)-8-Isopropyl-1-methyl-5-methylenecyclodeca-1,6-diene $$ D-Germacrene $$ 1(1

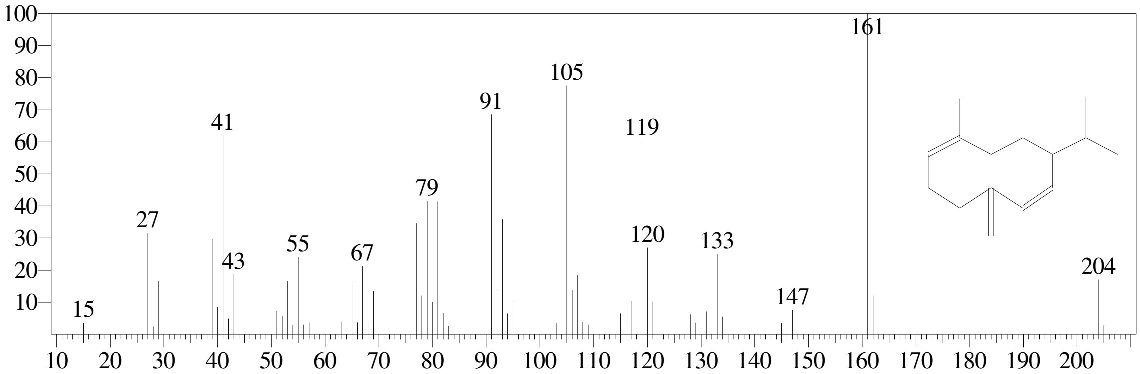


Hit#:2  Entry:25067  Library:NIST23s.lib
SI:84  Formula:C15H24  CAS:23986-74-5  MolWeight:204  RetIndex:1478
CompName:Germacrene D $$ (S,1Z,6Z)-8-Isopropyl-1-methyl-5-methylenecyclodeca-1,6-diene $$ D-Germacrene $$ 1(1

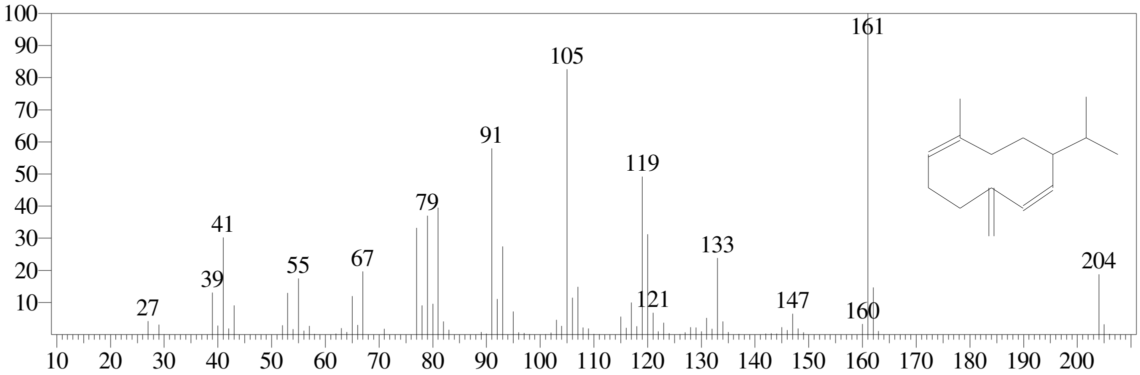

<< Target >>
Line#:21   R.Time:30.000(Scan#:3301)  MassPeaks:18
RawMode:Averaged 29.992-30.008(3300-3302) BasePeak:161.10(6161)
BG Mode:None  Group 1 - Event 1  Scan

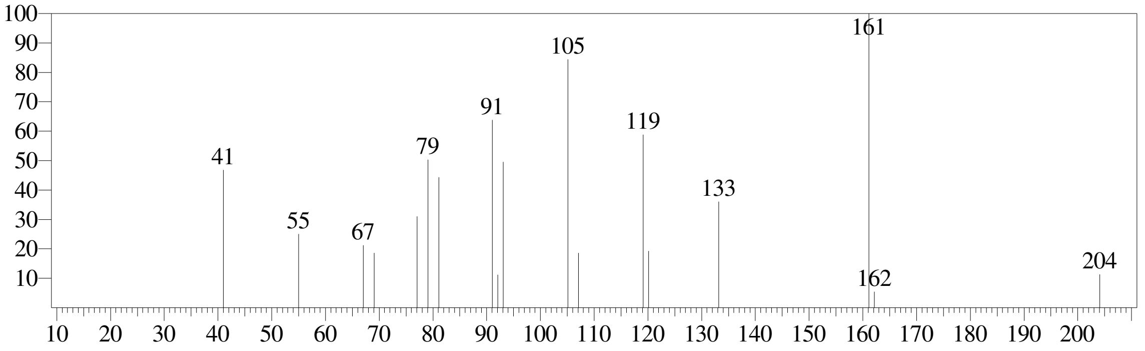


Hit#:3  Entry:25086  Library:NIST23s.lib
SI:82  Formula:C15H24  CAS:30021-74-0  MolWeight:204  RetIndex:1483
CompName:.gamma.-Muurolene $$ Naphthalene, 1,2,3,4,4a,5,6,8a-octahydro-7-methyl-4-methylene-1-(1-methylethyl)-, (

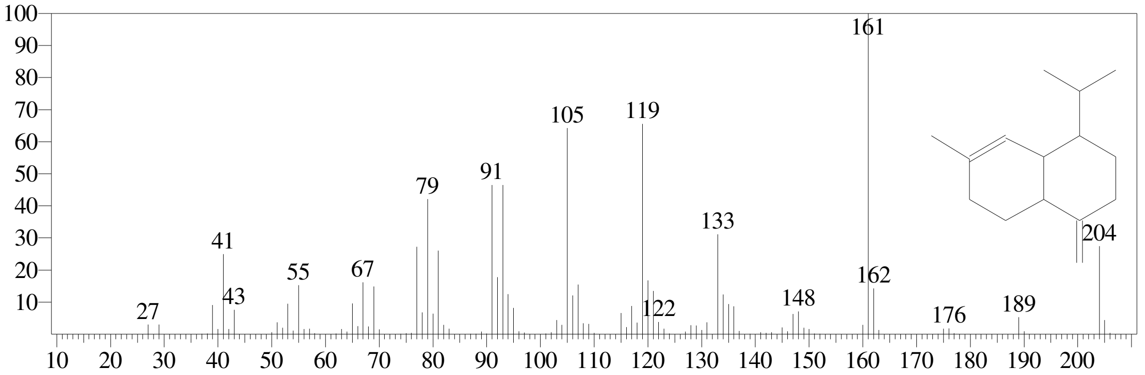


Hit#:4  Entry:25069  Library:NIST23s.lib
SI:82  Formula:C15H24  CAS:18252-44-3  MolWeight:204  RetIndex:1422
CompName:(1R,2S,6S,7S,8S)-8-Isopropyl-1-methyl-3-methylenetricyclo[4.4.0.02,7]decane-rel- $$ Tricyclo[4.4.0.02,7]de

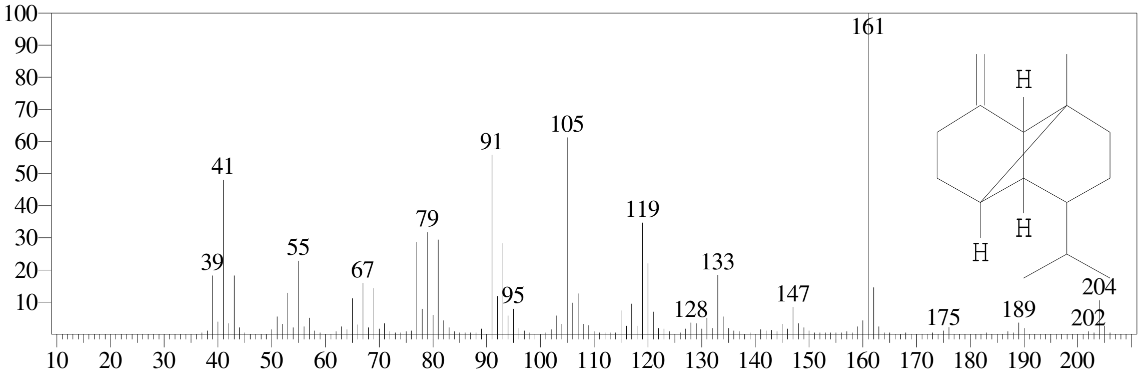

<< Target >>
Line#:21   R.Time:30.000(Scan#:3301)  MassPeaks:18
RawMode:Averaged 29.992-30.008(3300-3302) BasePeak:161.10(6161)
BG Mode:None  Group 1 - Event 1  Scan

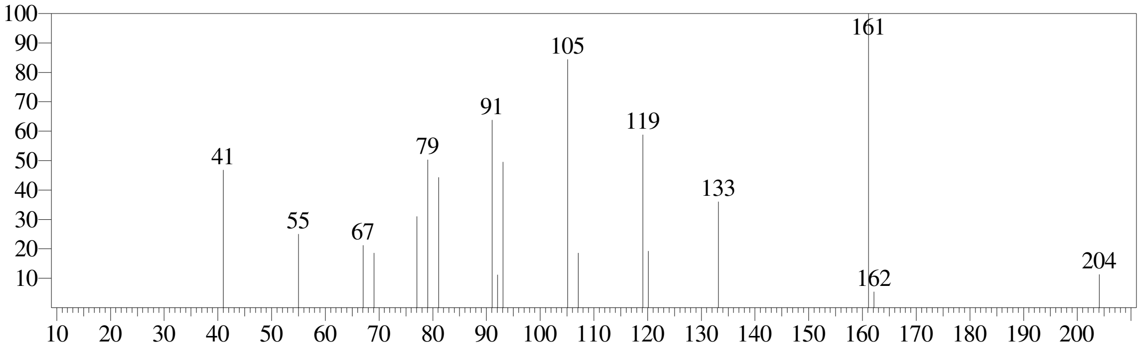


Hit#:5  Entry:62988  Library:NIST23-1.lib
SI:81  Formula:C15H24  CAS:17699-14-8  MolWeight:204  RetIndex:1381
CompName:.alpha.-Cubebene $$ 1H-Cyclopenta[1,3]cyclopropa[1,2]benzene, 3a,3b,4,5,6,7-hexahydro-3,7-dimethyl-4-(1

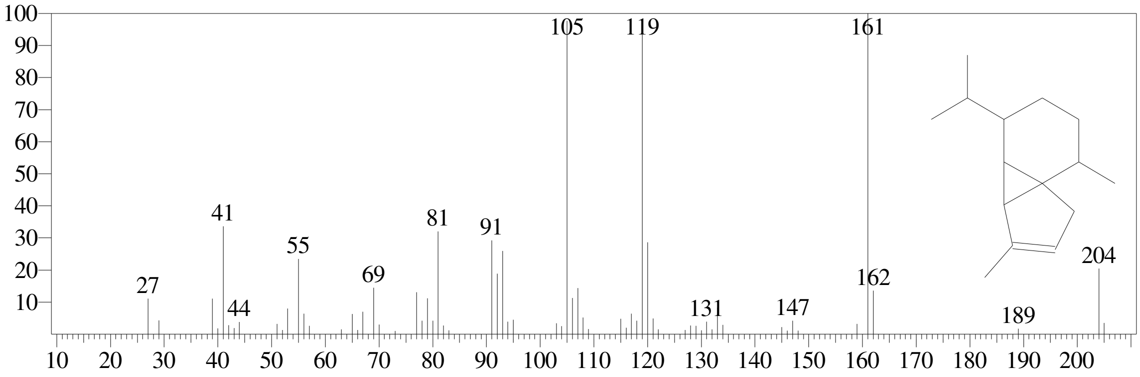

<< Target >>
Line#:22 R.Time:30.375(Scan#:3346) MassPeaks:31
RawMode:Averaged 30.367-30.383(3345-3347) BasePeak:119.10(12692)
BG Mode:None Group 1 - Event 1 Scan

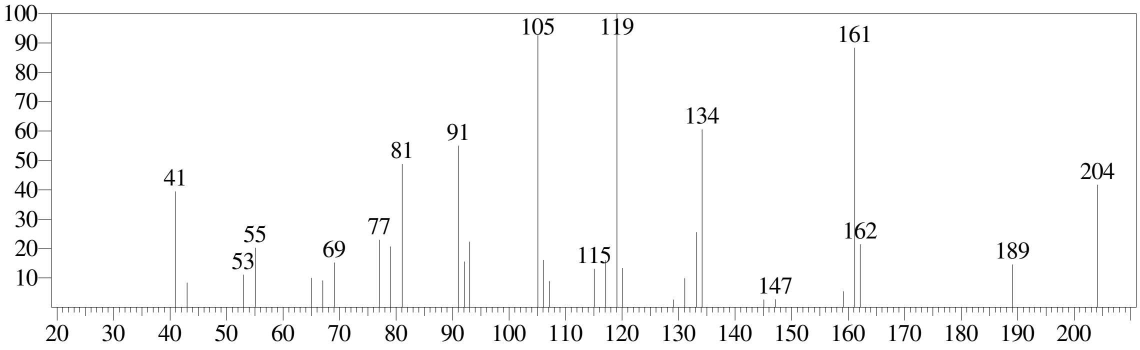


Hit#:1 Entry:63006 Library:NIST23-1.lib
SI:88 Formula:C15H24 CAS:16729-01-4 MolWeight:204 RetIndex:1526
CompName:1-Isopropyl-4,7-dimethyl-1,2,3,5,6,8a-hexahydronaphthalene $$ Cadina-1(10),4-diene $$

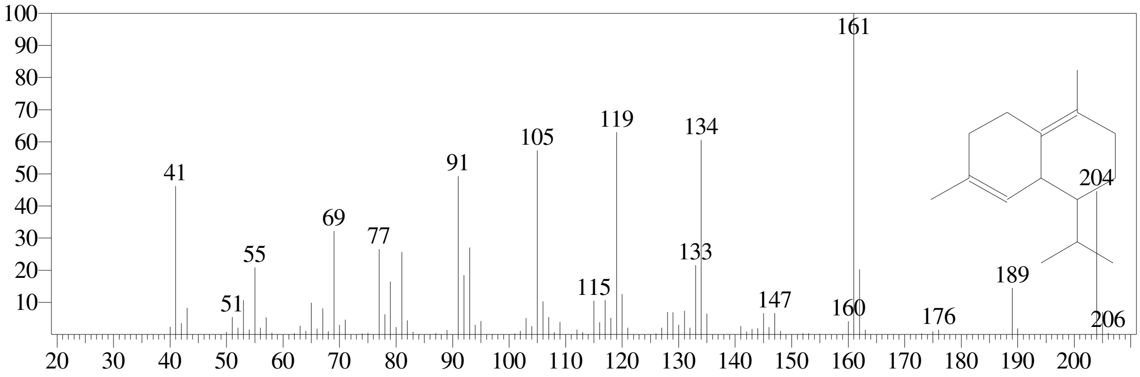


Hit#:2 Entry:25090 Library:NIST23s.lib
SI:88 Formula:C15H24 CAS:483-76-1 MolWeight:204 RetIndex:1526
CompName:Naphthalene, 1,2,3,5,6,8a-hexahydro-4,7-dimethyl-1-(1-methylethyl)-, (1S-cis)- $$ Cadina-1(10),4-diene $$ .

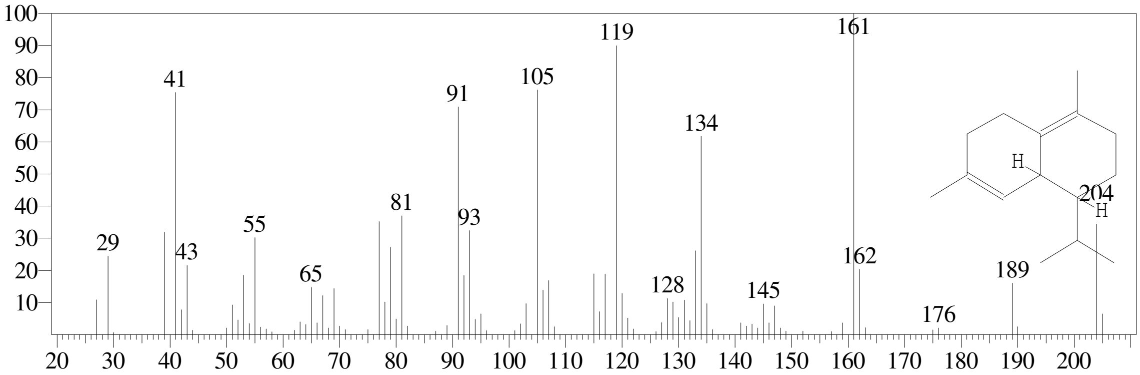

<< Target >>
Line#:22   R.Time:30.375(Scan#:3346)  MassPeaks:31
RawMode:Averaged 30.367-30.383(3345-3347) BasePeak:119.10(12692)
BG Mode:None  Group 1 - Event 1  Scan

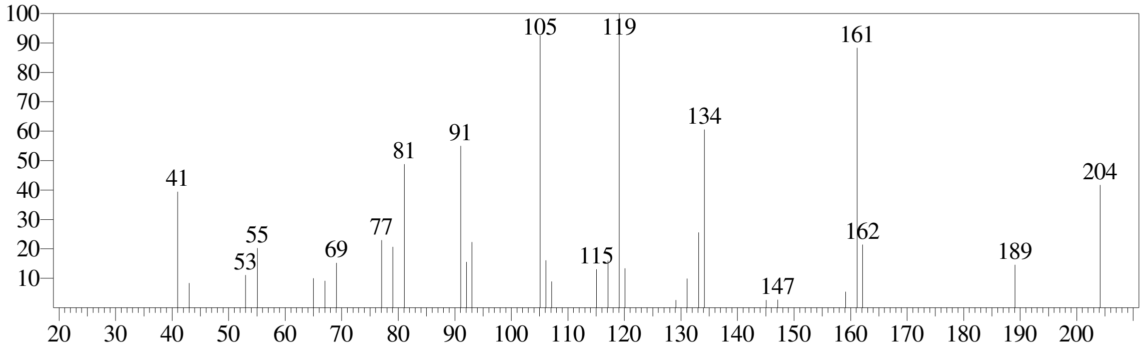


Hit#:3  Entry:63005  Library:NIST23-1.lib
SI:88  Formula:C15H24  CAS:189165-79-5  MolWeight:204  RetIndex:1526
CompName:Amorphene,delta-

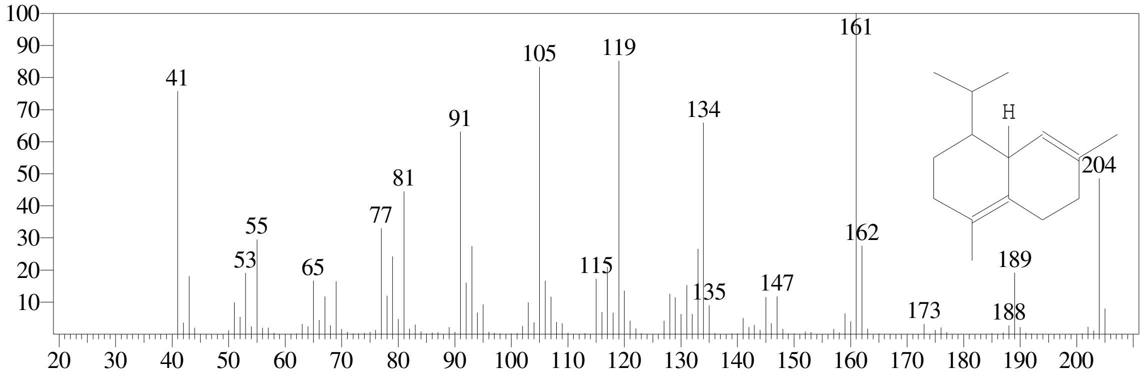


Hit#:4  Entry:24949  Library:NIST23s.lib
SI:87  Formula:C15H24  CAS:17699-14-8  MolWeight:204  RetIndex:1381
CompName:.alpha.-Cubebene $$ 1H-Cyclopenta[1,3]cyclopropa[1,2]benzene, 3a,3b,4,5,6,7-hexahydro-3,7-dimethyl-4-(1

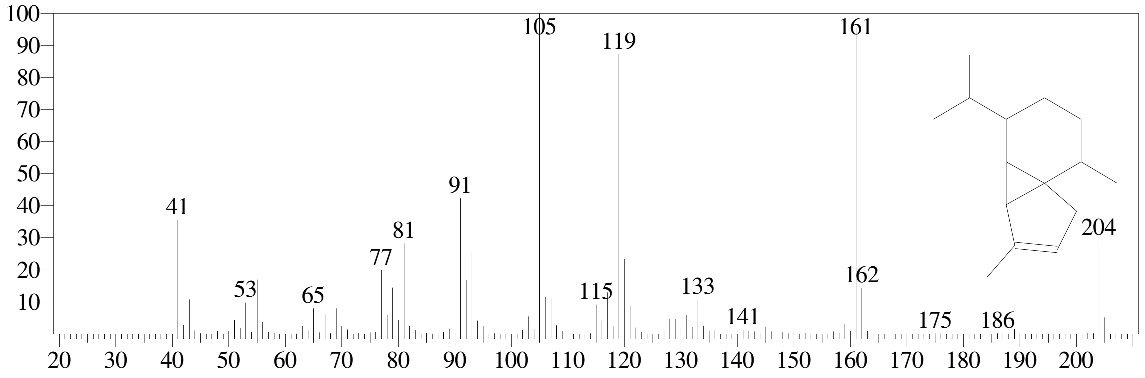

<< Target >>
Line#:22   R.Time:30.375(Scan#:3346)  MassPeaks:31
RawMode:Averaged 30.367-30.383(3345-3347) BasePeak:119.10(12692)
BG Mode:None  Group 1 - Event 1  Scan

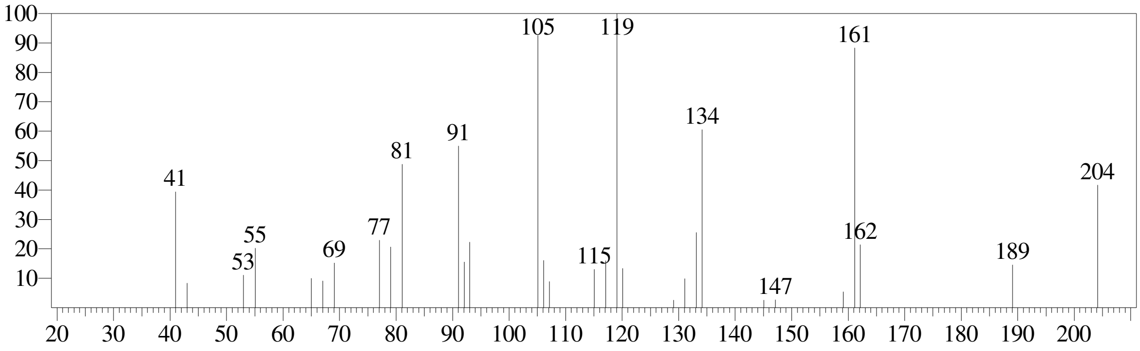


Hit#:5  Entry:25089  Library:NIST23s.lib
SI:87  Formula:C15H24  CAS:3856-25-5  MolWeight:204  RetIndex:1407
CompName:Copaene $$ Tricyclo[4.4.0.02,7]dec-3-ene, 1,3-dimethyl-8-(1-methylethyl)-, stereoisomer $$ Tricyclo[4.4.0.0

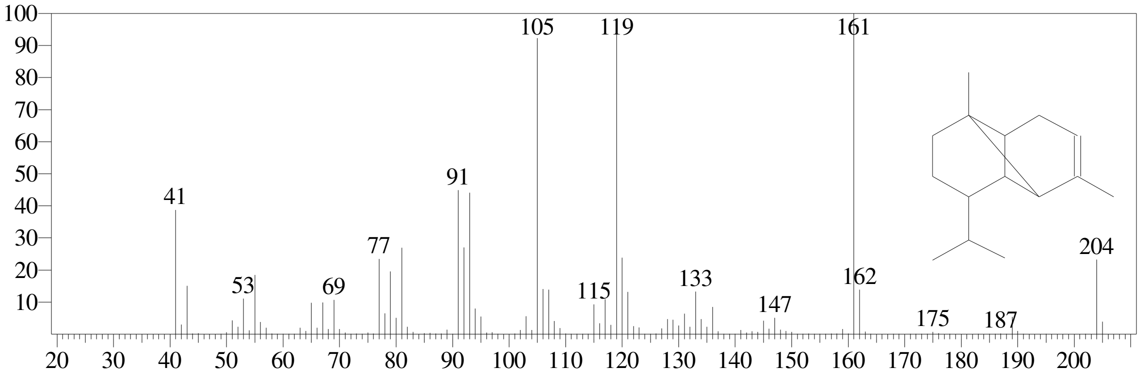

<< Target >>
Line#:23 R.Time:30.917(Scan#:3411) MassPeaks:6
RawMode:Averaged 30.908-30.925(3410-3412) BasePeak:105.10(4147)
BG Mode:None Group 1 - Event 1 Scan

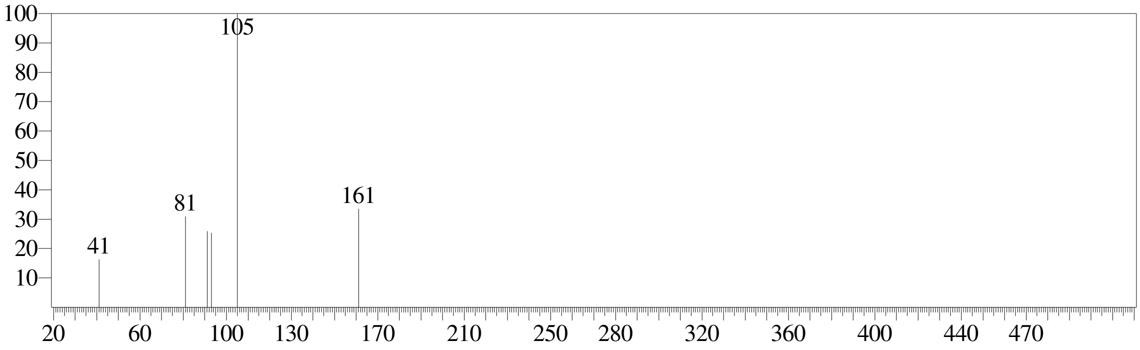


Hit#:1 Entry:226428 Library:NIST23-1.lib
SI:71 Formula:C21H32O3 CAS:0-00-0 MolWeight:332 RetIndex:2612
CompName:4-Oxo-4-phenylbutyric acid, undecyl ester

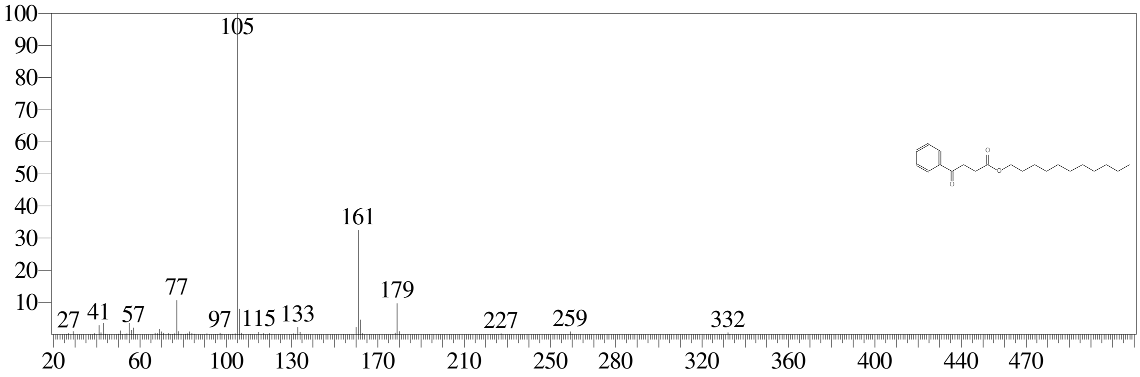


Hit#:2 Entry:86443 Library:NIST23-2.lib
SI:70 Formula:C34H36O4 CAS:0-00-0 MolWeight:508 RetIndex:4167
CompName:Cyclopenta[d]anthracene-8,11-diol, 3-isopropyl-1,2,3,3a,4,5,6,6a,7,12-decahydro-, dibenzoate $$ 8-(Benzoylo

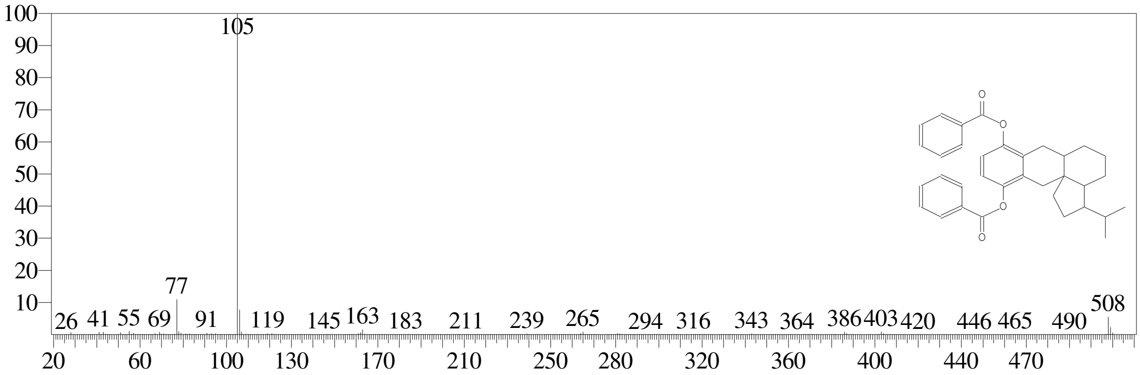

<< Target >>
Line#:23 R.Time:30.917(Scan#:3411) MassPeaks:6
RawMode:Averaged 30.908-30.925(3410-3412) BasePeak:105.10(4147)
BG Mode:None Group 1 - Event 1 Scan

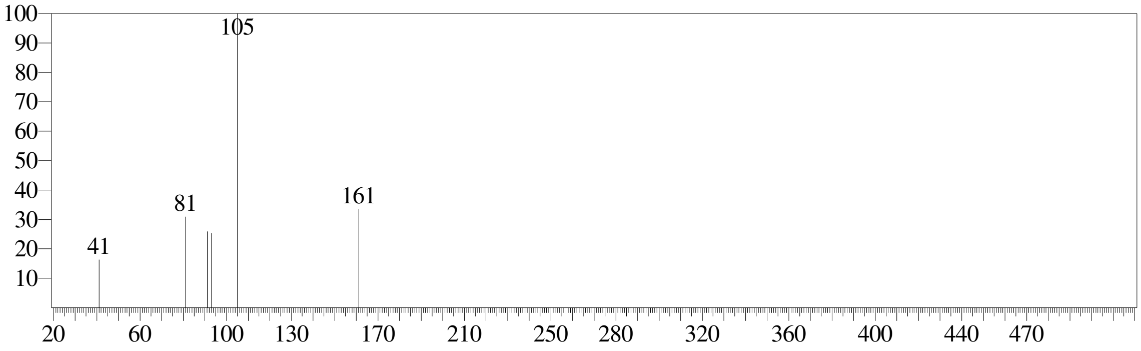


Hit#:3 Entry:10819 Library:NIST23-2.lib
SI:69 Formula:C23H36O3 CAS:0-00-0 MolWeight:360 RetIndex:2821
CompName:4-Oxo-4-phenylbutyric acid, tridecyl ester

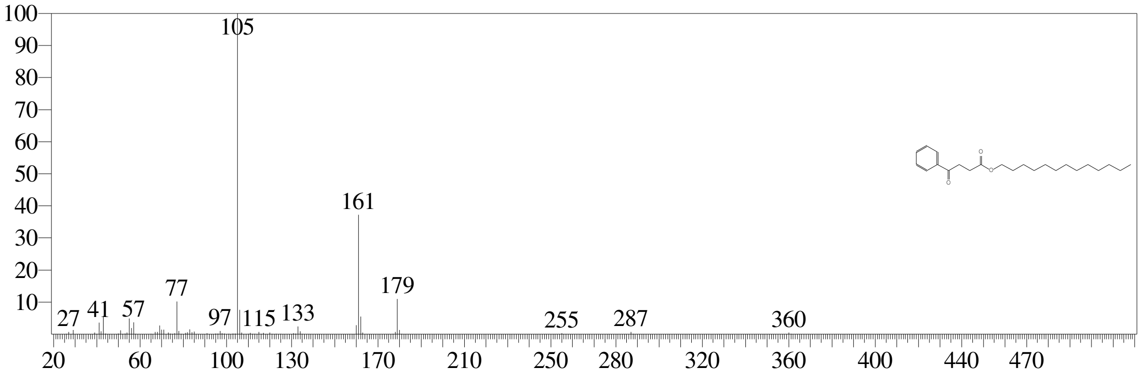


Hit#:4 Entry:243778 Library:NIST23-1.lib
SI:68 Formula:C22H34O3 CAS:0-00-0 MolWeight:346 RetIndex:2716
CompName:4-Oxo-4-phenylbutyric acid, dodecyl ester

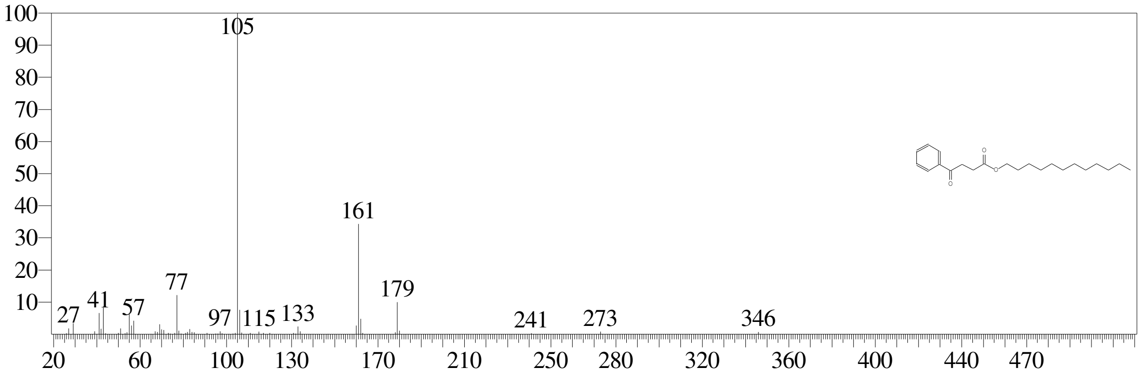

<< Target >>
Line#:23   R.Time:30.917(Scan#:3411)  MassPeaks:6
RawMode:Averaged 30.908-30.925(3410-3412) BasePeak:105.10(4147)
BG Mode:None  Group 1 - Event 1  Scan

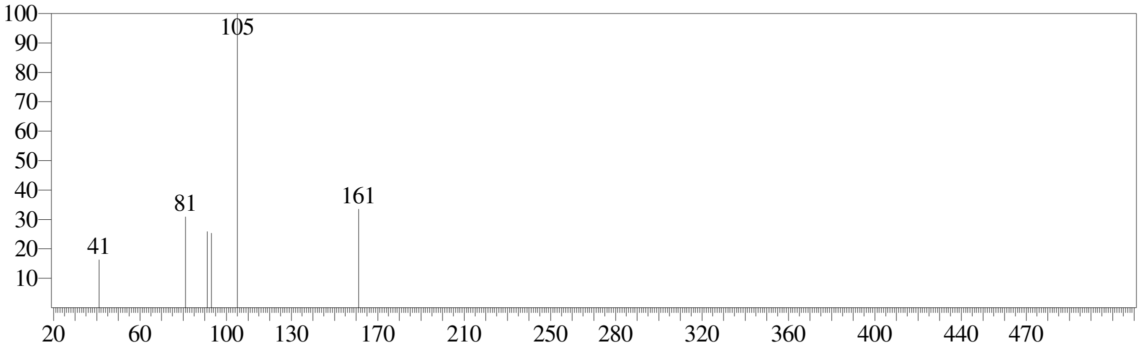


Hit#:5  Entry:78385  Library:NIST23-1.lib
SI:68  Formula:C15H22O  CAS:98088-51-8  MolWeight:218  RetIndex:1449
CompName:Benzene, [1-[[1-(1-methylethyl)-3-butenyl]oxy]ethyl]-, [S-(R*,R*)]- $$ (1-[(1-Isopropyl-3-butenyl)oxy]ethyl]

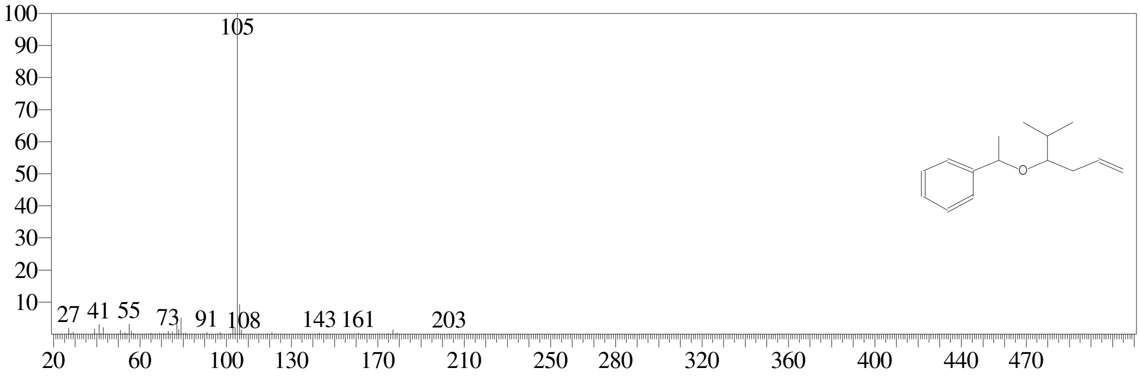

<< Target >>
Line#:24   R.Time:34.358(Scan#:3824)  MassPeaks:15
RawMode:Averaged 34.350-34.367(3823-3825) BasePeak:41.00(2130)
BG Mode:None  Group 1 - Event 1  Scan

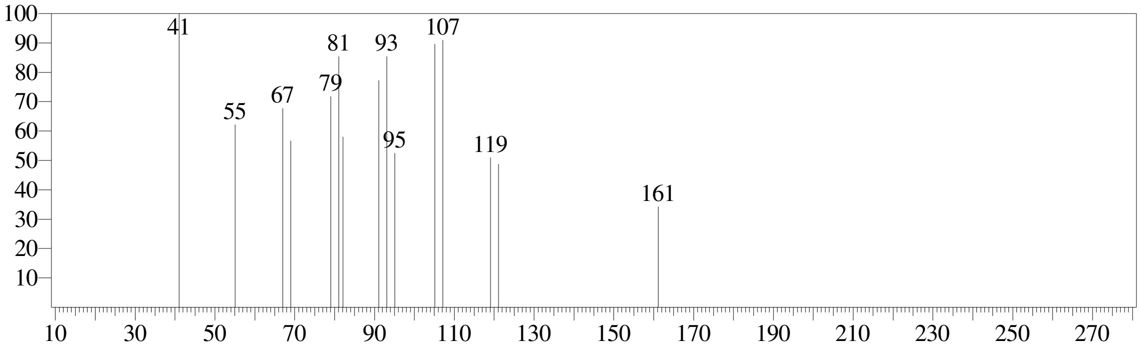


Hit#:1  Entry:128063  Library:NIST23-1.lib
SI:75  Formula:C18H26O  CAS:0-00-0  MolWeight:258  RetIndex:2011
CompName:1,3-Bis-(2-cyclopropyl,2-methylcyclopropyl)-but-2-en-1-one

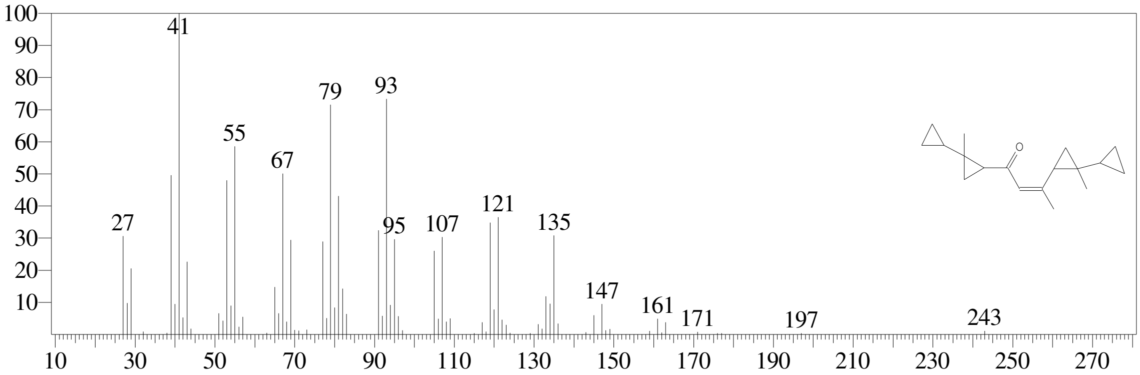


Hit#:2  Entry:62867  Library:NIST23-1.lib
SI:75  Formula:C15H24  CAS:0-00-0  MolWeight:204  RetIndex:1394
CompName:Cycloheptane, 4-methylene-1-methyl-2-(2-methyl-1-propen-1-yl)-1-vinyl- $$ 1-Methyl-4-methylene-2-(2-me

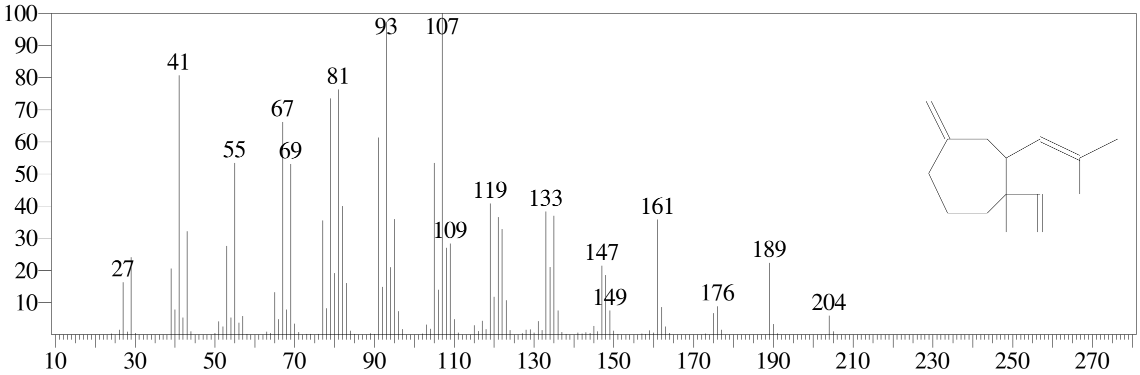

<< Target >>
Line#:24 R.Time:34.358(Scan#:3824) MassPeaks:15
RawMode:Averaged 34.350-34.367(3823-3825) BasePeak:41.00(2130)
BG Mode:None Group 1 - Event 1 Scan

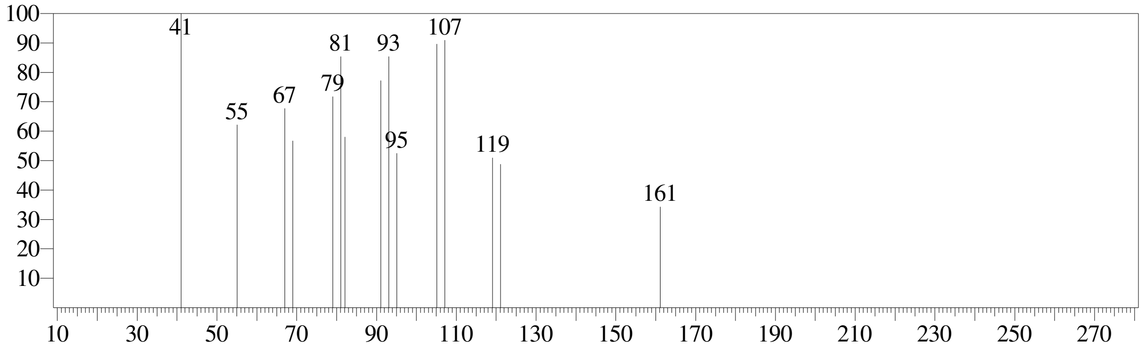


Hit#:3 Entry:48959 Library:NIST23-1.lib
SI:73 Formula:C14H22 CAS:62338-42-5 MolWeight:190 RetIndex:1338
CompName:Cyclobutene, 4,4-dimethyl-1-(2,7-octadienyl)- $$ 4,4-Dimethyl-1-[(2E)-2,7-octadienyl]-1-cyclobutene # $$

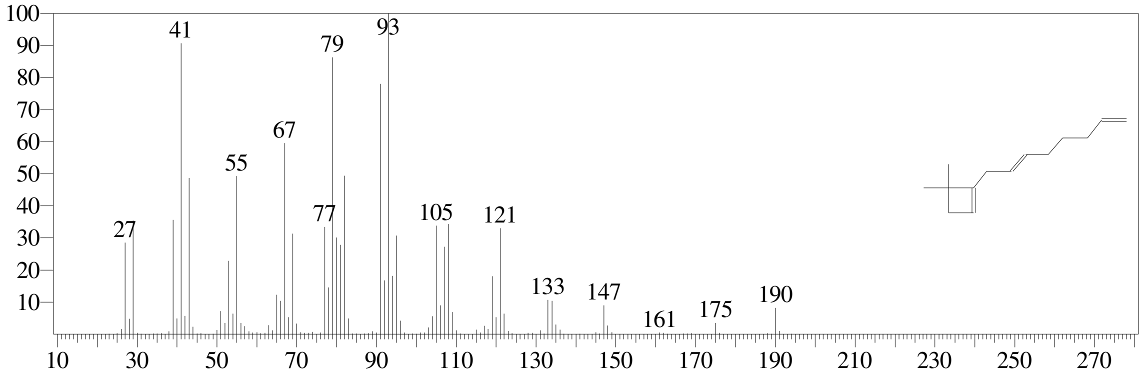


Hit#:4 Entry:149236 Library:NIST23-1.lib
SI:73 Formula:C20H34 CAS:28393-07-9 MolWeight:274 RetIndex:2186
CompName:Icosa-9,11-diyne

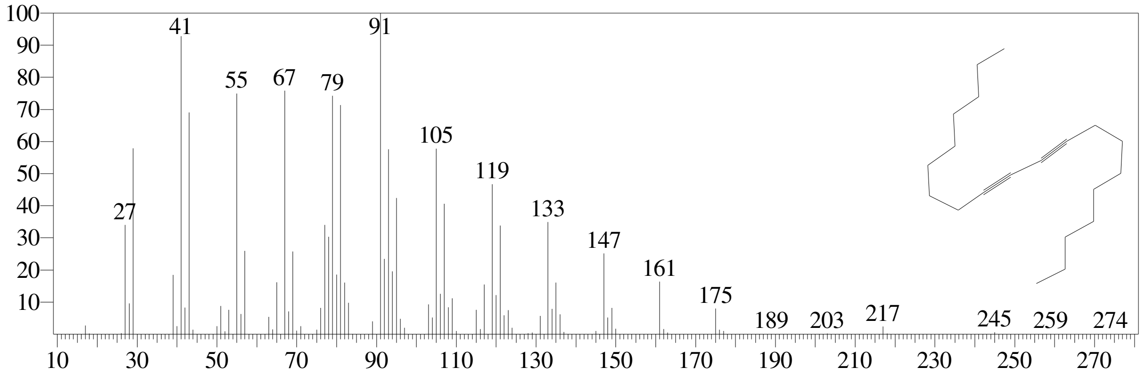

<< Target >>
Line#:24 R.Time:34.358(Scan#:3824) MassPeaks:15
RawMode:Averaged 34.350-34.367(3823-3825) BasePeak:41.00(2130)
BG Mode:None Group 1 - Event 1 Scan

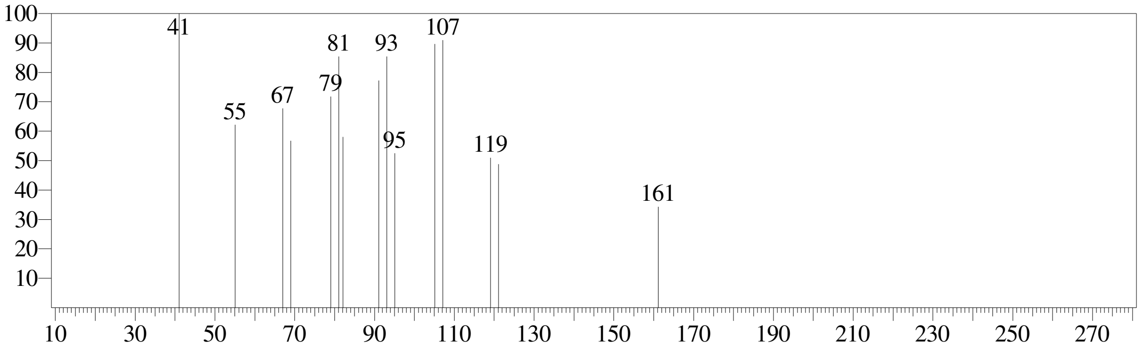


Hit#:5 Entry:24824 Library:NIST23s.lib
SI:73 Formula:C15H24 CAS:502-61-4 MolWeight:204 RetIndex:1509
CompName:.alpha.-Farnesene $$ 1,3,6,10-Dodecatetraene, 3,7,11-trimethyl-, (E,E)- $$ Farnesene $$ 2,6,10-Trimethyl-2,6

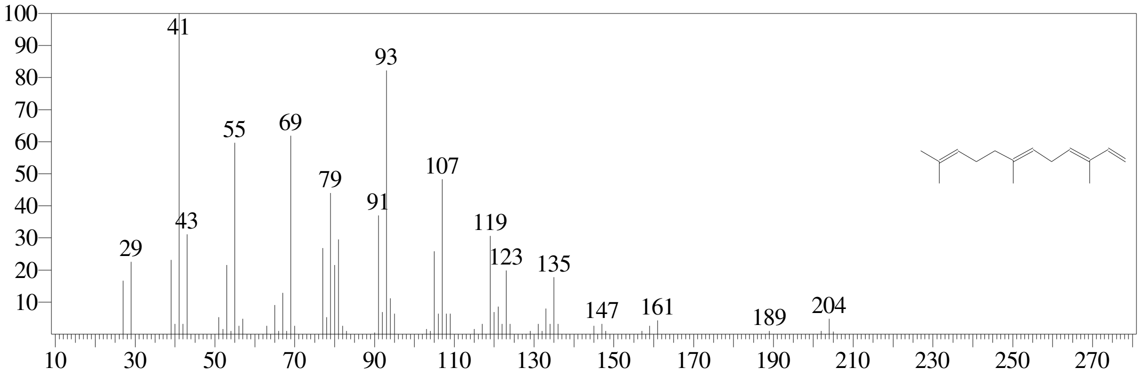

<< Target >>
Line#:25   R.Time:36.408(Scan#:4070)  MassPeaks:7
RawMode:Averaged 36.400-36.417(4069-4071) BasePeak:105.10(2052)
BG Mode:None  Group 1 - Event 1  Scan

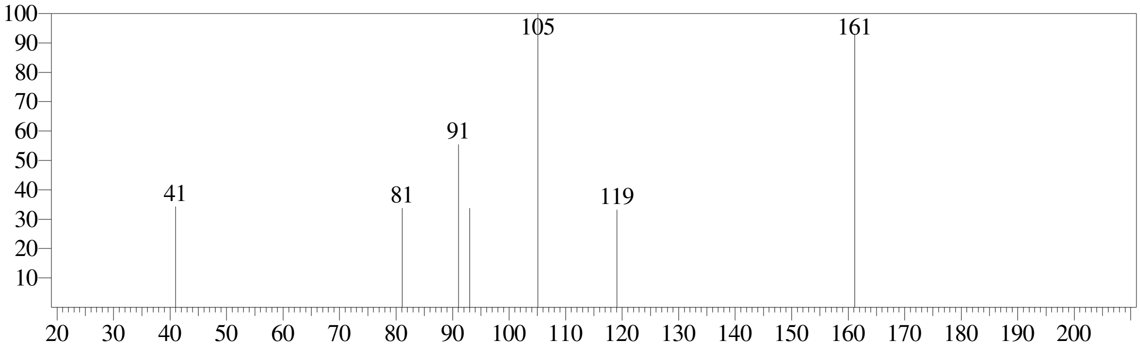


Hit#:1  Entry:25079  Library:NIST23s.lib
SI:75  Formula:C15H24  CAS:157477-72-0  MolWeight:204  RetIndex:1487
CompName:cis-Muurola-4(15),5-diene $$ (1S,4S,4aR)-1-Isopropyl-4-methyl-7-methylene-1,2,3,4,4a,5,6,7-octahydronaph

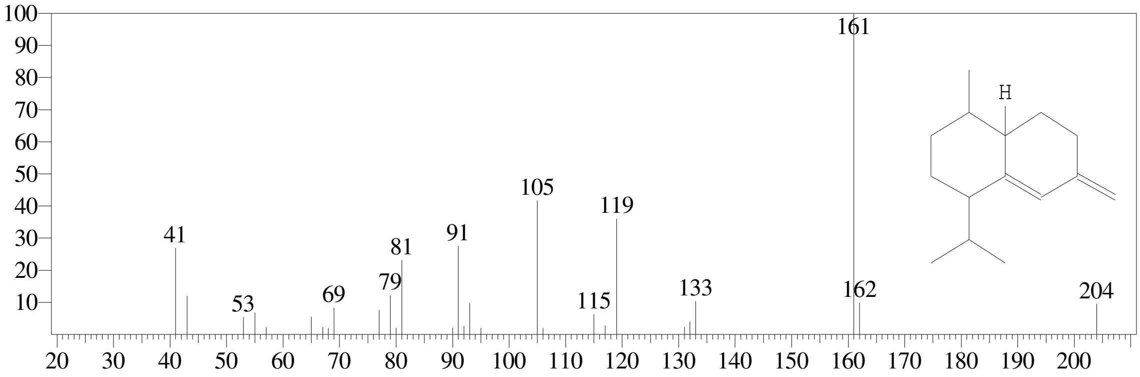


Hit#:2  Entry:25078  Library:NIST23s.lib
SI:70  Formula:C15H24  CAS:157477-72-0  MolWeight:204  RetIndex:1487
CompName:cis-Muurola-4(15),5-diene $$ (1S,4S,4aR)-1-Isopropyl-4-methyl-7-methylene-1,2,3,4,4a,5,6,7-octahydronaph

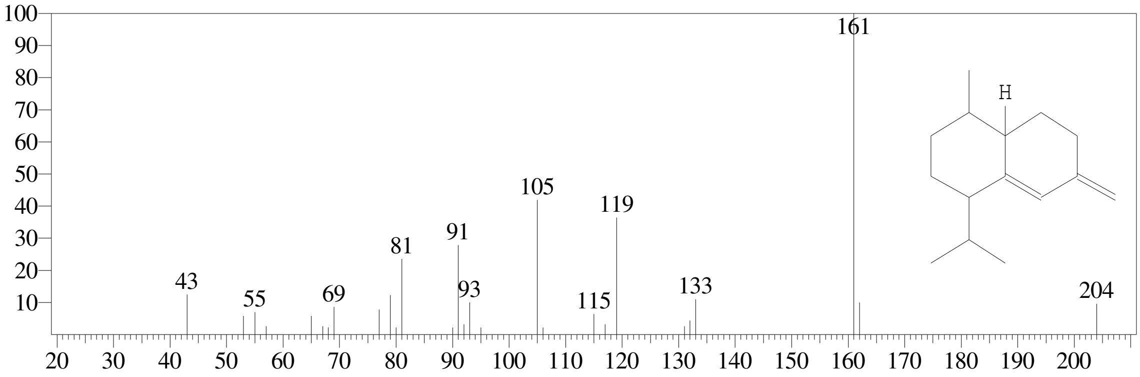

<< Target >>
Line#:25   R.Time:36.408(Scan#:4070)  MassPeaks:7
RawMode:Averaged 36.400-36.417(4069-4071) BasePeak:105.10(2052)
BG Mode:None  Group 1 - Event 1  Scan

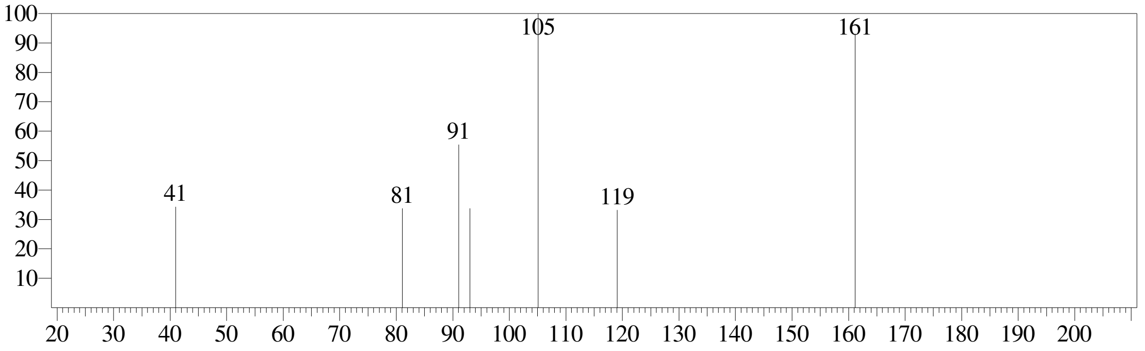


Hit#:3  Entry:62987  Library:NIST23-1.lib
SI:70  Formula:C15H24  CAS:267665-20-3  MolWeight:204  RetIndex:1465
CompName:(1S,4S,4aS)-1-Isopropyl-4,7-dimethyl-1,2,3,4,4a,5-hexahydronaphthalene $$ Naphthalene, 1,2,3,4,4a,5-hexah

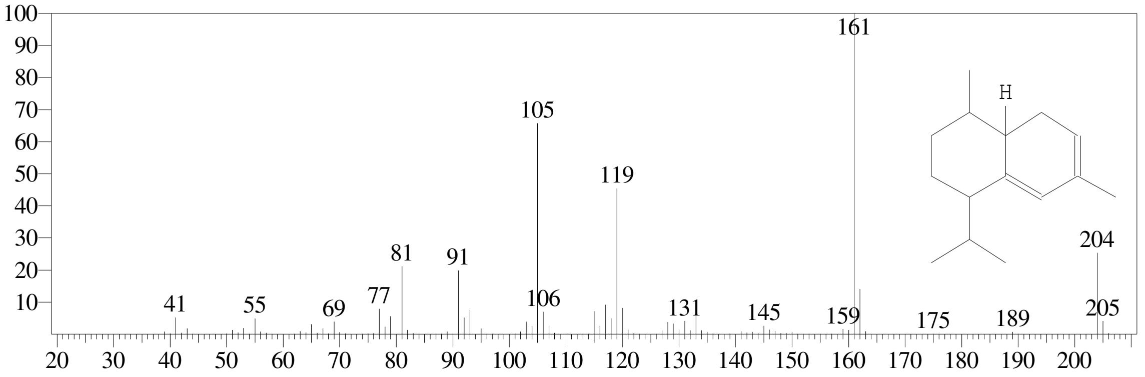


Hit#:4  Entry:24948  Library:NIST23s.lib
SI:69  Formula:C15H24  CAS:17699-14-8  MolWeight:204  RetIndex:1381
CompName:.alpha.-Cubebene $$ 1H-Cyclopenta[1,3]cyclopropa[1,2]benzene, 3a,3b,4,5,6,7-hexahydro-3,7-dimethyl-4-(1-

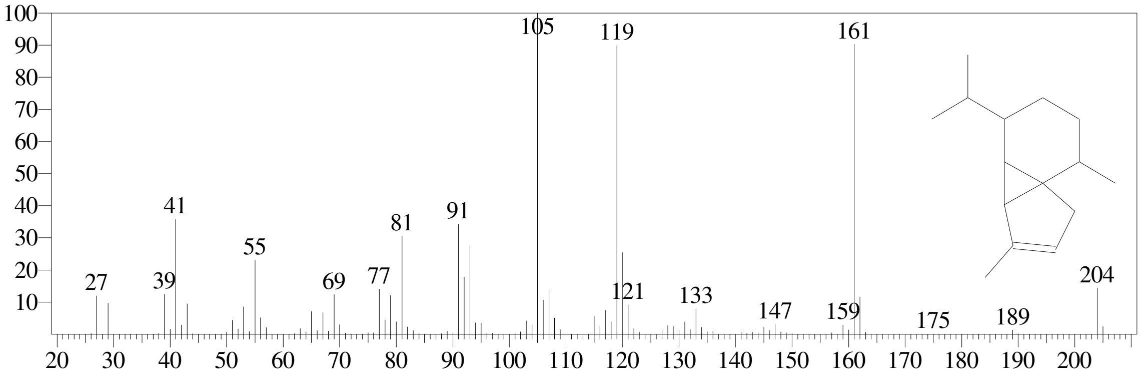

<< Target >>
Line#:25   R.Time:36.408(Scan#:4070)  MassPeaks:7
RawMode:Averaged 36.400-36.417(4069-4071) BasePeak:105.10(2052)
BG Mode:None  Group 1 - Event 1  Scan

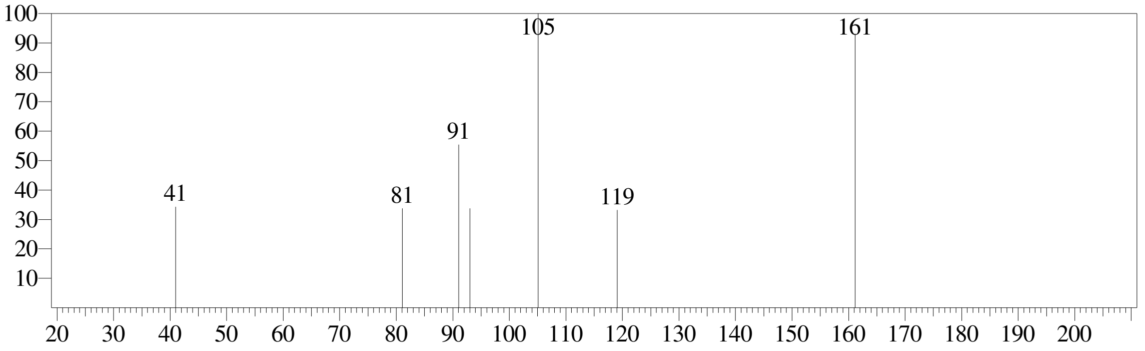


Hit#:5  Entry:121322  Library:NIST23-1.lib
SI:69  Formula:C17H19NO  CAS:0-00-0  MolWeight:253  RetIndex:2112
CompName:2-Phenylacetamide, N-(1-phenyl-2-propyl)- $$ N-(1-Methyl-2-phenylethyl)-2-phenylacetamide  # $$

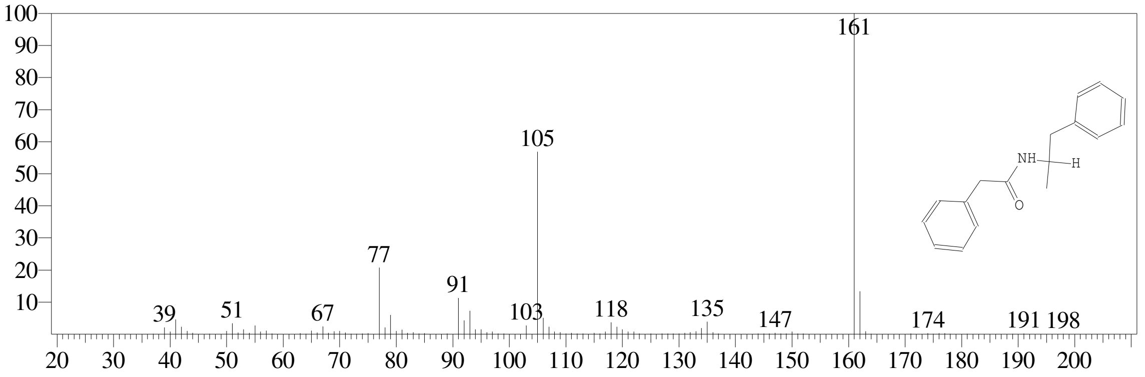

Supplement: Supplementary file 1 [file plants-15-01406-s001.zip › EI bb.pdf]
