# Supplementary material for: Unveiling Chemical Profile and Insecticidal Potential of Essential Oils from Leaves of Seven Eugenia L. Species (Myrtaceae)
Source: Plants (Basel). 2026 May 5;15(9):1406. doi: 10.3390/plants15091406 (PMC13165059; doi:10.3390/plants15091406)

CAM · UFRRJ
LabCrom
Laboratório de Cromatografia

UNIVERSIDADE FERAL RURAL DO RIO DE JANEIRO
INSTITUTO DE QUÍMICA

CENTRAL ANALÍTICA MULTIUSUÁRIO
INSTITUTO DE QUÍMICA · UFRRJ

CGMS

Analyzed by: Cristiane Cardoso
Analyzed: 17/6/2025
Solicitante:Douglas
Sample Name: EL
Injection Volume: 1,0 uL Solvente: Diclorometano
Data File: C:\GCMSsolution\Data\Project1\Douglas\2025\MLLENA\170625\EL.qgd
Method File: C:\GCMSsolution\Data\Project1\Douglas\Essencial Adams-Inj.qgm
EQUIPAMENTO: Modelo:GCMS-QP2010 Plus (Shimadzu)
Coluna: VF-5m (30X0.25X0.25)

Chromatogram EL C:\GCMSsolution\Data\Project1\Douglas\2025\MLLENA\170625\EL.qgd

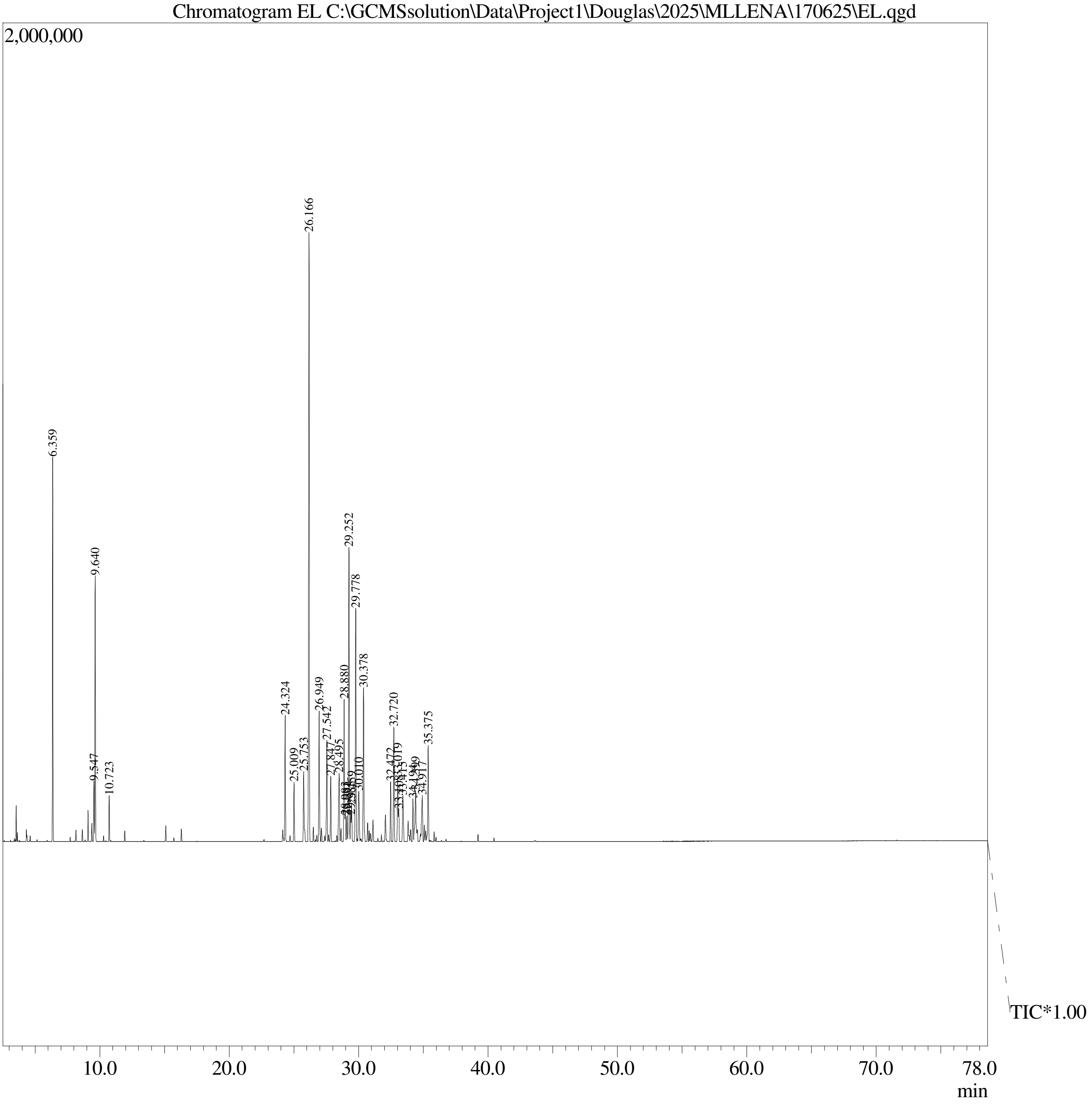

Library

<< Target >>
Line#:1   R.Time:6.358(Scan#:464) MassPeaks:44
RawMode:Averaged 6.350-6.367(463-465) BasePeak:93.05(217889)
BG Mode:None Group 1 - Event 1 Scan

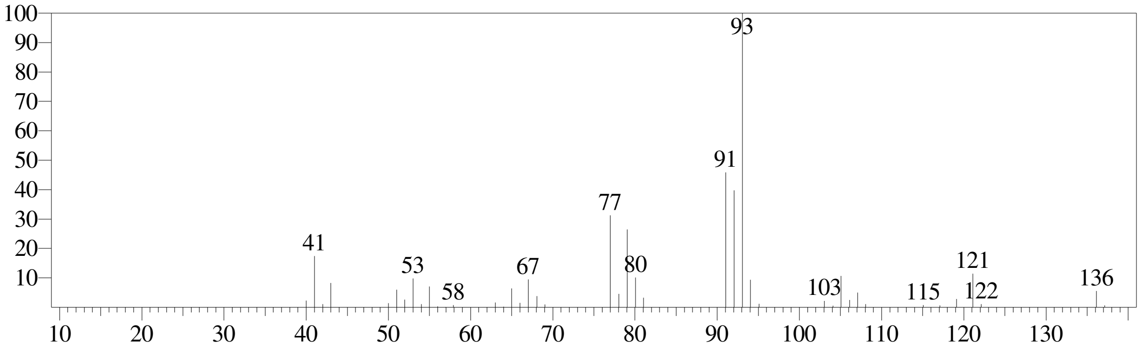

Hit#:1 Entry:8451 Library:NIST23s.lib
SI:97 Formula:C10H16 CAS:80-56-8 MolWeight:136 RetIndex:947
CompName:.alpha.-Pinene $$ Bicyclo[3.1.1]hept-2-ene, 2,6,6-trimethyl- $$ 2-Pinene $$ 2,6,6-Trimethylbicyclo[3.1.1]hep

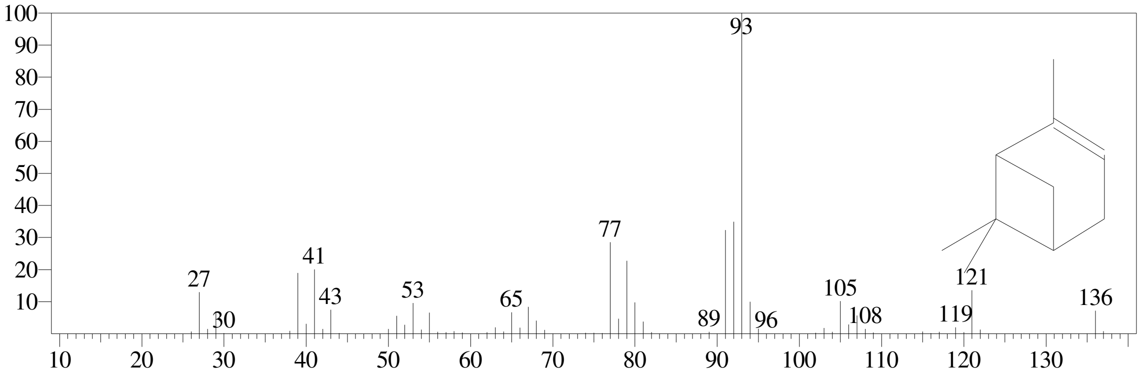

Hit#:2 Entry:8445 Library:NIST23s.lib
SI:97 Formula:C10H16 CAS:80-56-8 MolWeight:136 RetIndex:947
CompName:.alpha.-Pinene $$ Bicyclo[3.1.1]hept-2-ene, 2,6,6-trimethyl- $$ 2-Pinene $$ 2,6,6-Trimethylbicyclo[3.1.1]hep

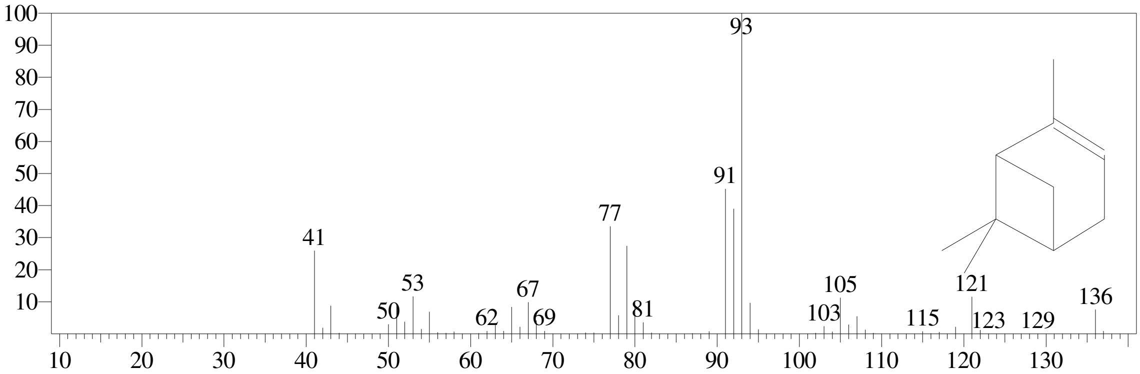

<< Target >>
Line#:1 R.Time:6.358(Scan#:464) MassPeaks:44
RawMode:Averaged 6.350-6.367(463-465) BasePeak:93.05(217889)
BG Mode:None Group 1 - Event 1 Scan

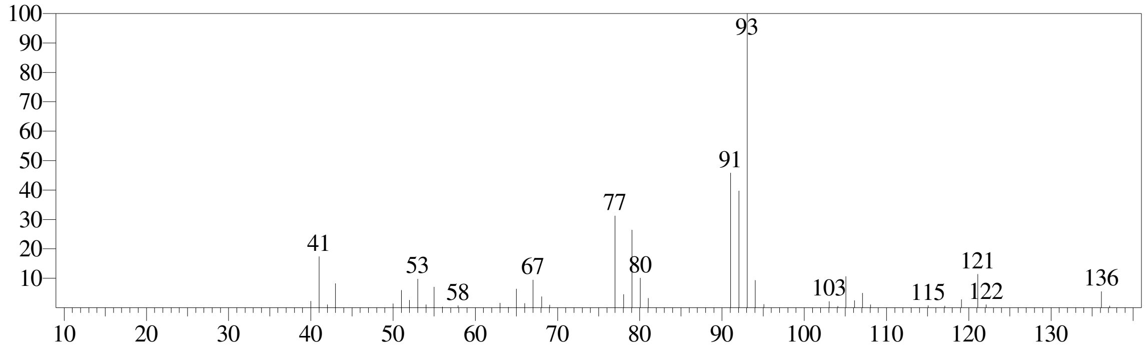

Hit#:3 Entry:11410 Library:NIST23-1.lib
SI:96 Formula:C10H16 CAS:80-56-8 MolWeight:136 RetIndex:947
CompName:.alpha.-Pinene $$ Bicyclo[3.1.1]hept-2-ene, 2,6,6-trimethyl- $$ 2-Pinene $$ 2,6,6-Trimethylbicyclo[3.1.1]hep

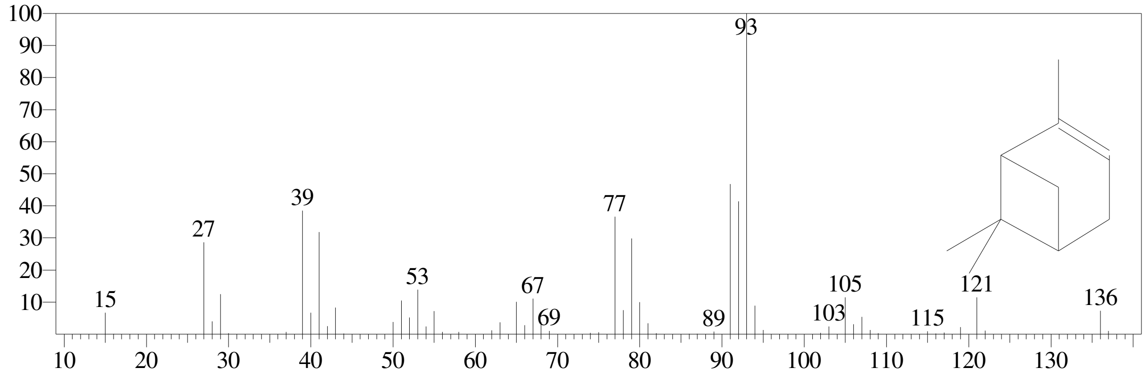

Hit#:4 Entry:8450 Library:NIST23s.lib
SI:96 Formula:C10H16 CAS:80-56-8 MolWeight:136 RetIndex:947
CompName:.alpha.-Pinene $$ Bicyclo[3.1.1]hept-2-ene, 2,6,6-trimethyl- $$ 2-Pinene $$ 2,6,6-Trimethylbicyclo[3.1.1]hep

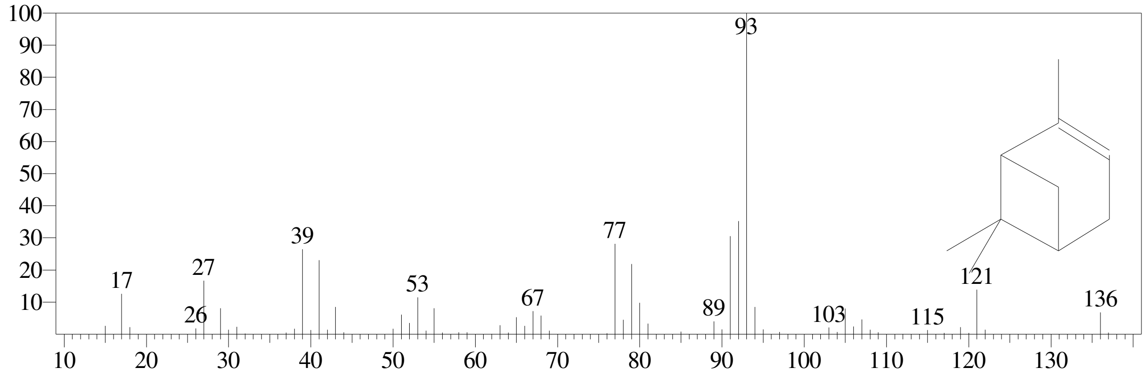

<< Target >>
Line#:1   R.Time:6.358(Scan#:464) MassPeaks:44
RawMode:Averaged 6.350-6.367(463-465) BasePeak:93.05(217889)
BG Mode:None Group 1 - Event 1 Scan

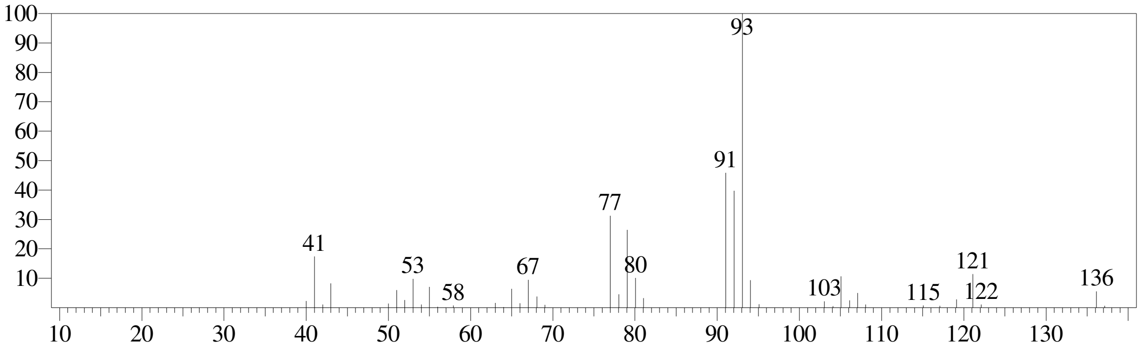

Hit#:5 Entry:8443 Library:NIST23s.lib
SI:95 Formula:C10H16 CAS:3779-61-1 MolWeight:136 RetIndex:1047
CompName:trans-.beta.-Ocimene $$ 1,3,6-Octatriene, 3,7-dimethyl-, (E)- $$ .beta.-trans-Ocimene $$ trans-3,7-Dimethyl-

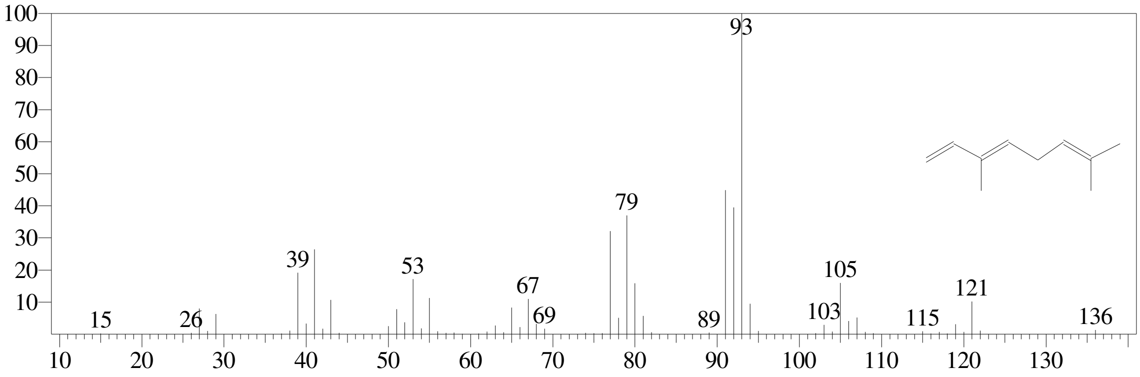

<< Target >>
Line#:2   R.Time:9.550(Scan#:847) MassPeaks:25
RawMode:Averaged 9.542-9.558(846-848) BasePeak:68.05(19726)
BG Mode:Calc. from Peak Group 1 - Event 1 Scan

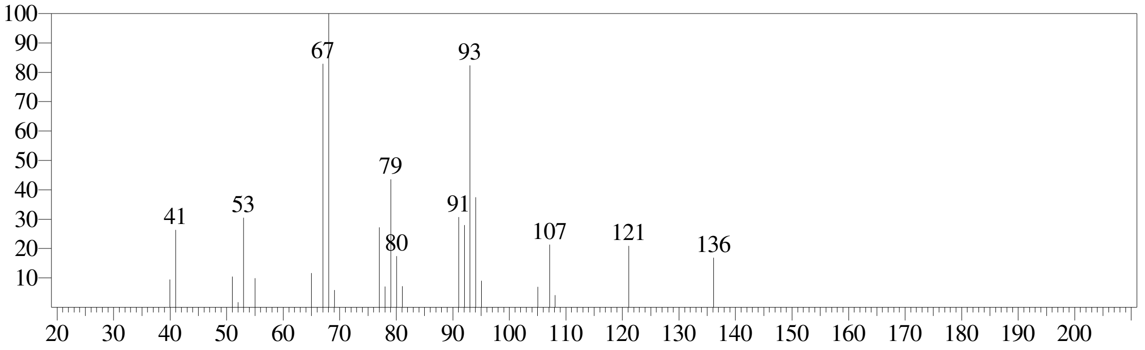

Hit#:1 Entry:8371 Library:NIST23s.lib
SI:93 Formula:C10H16 CAS:5989-27-5 MolWeight:136 RetIndex:1031
CompName:D-Limonene $$ Cyclohexene, 1-methyl-4-(1-methylethenyl)-, (R)- $$ p-Mentha-1,8-diene, (R)-(+)- $$ (+)-(R

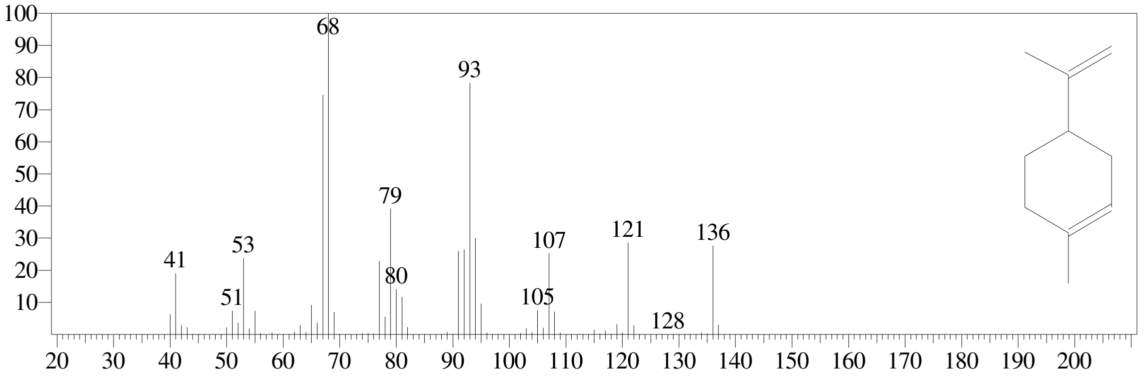

Hit#:2 Entry:11345 Library:NIST23-1.lib
SI:91 Formula:C10H16 CAS:19465-02-2 MolWeight:136 RetIndex:968
CompName:Cyclobutane, 1,2-bis(1-methylethenyl)-, trans- $$ 1,2-Diisopropenylcyclobutane # $$

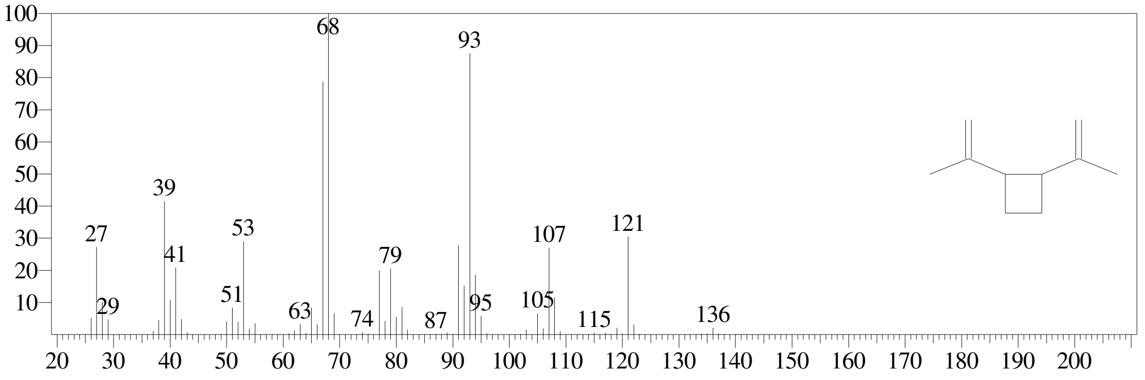

<< Target >>
Line#:2   R.Time:9.550(Scan#:847) MassPeaks:25
RawMode:Averaged 9.542-9.558(846-848) BasePeak:68.05(19726)
BG Mode:Calc. from Peak Group 1 - Event 1 Scan

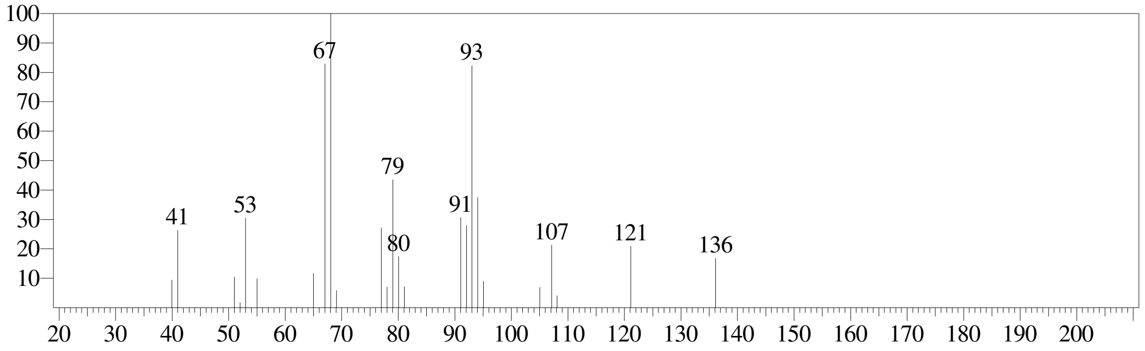

Hit#:3 Entry:11340 Library:NIST23-1.lib
SI:91 Formula:C10H16 CAS:138-86-3 MolWeight:136 RetIndex:1031
CompName:Limonene $$ Cyclohexene, 1-methyl-4-(1-methylethenyl)- $$ p-Mentha-1,8-diene $$ .alpha.-Limonene $$ C

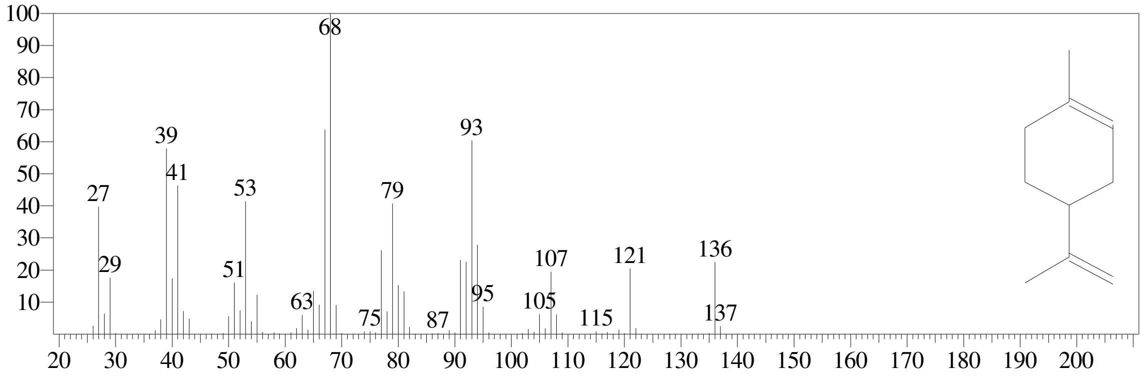

Hit#:4 Entry:8365 Library:NIST23s.lib
SI:90 Formula:C10H16 CAS:5989-54-8 MolWeight:136 RetIndex:1031
CompName:Cyclohexene, 1-methyl-4-(1-methylethenyl)-, (S)- $$ p-Mentha-1,8-diene, (S)-(-)- $$ (-)-Limonene $$ L-Lim

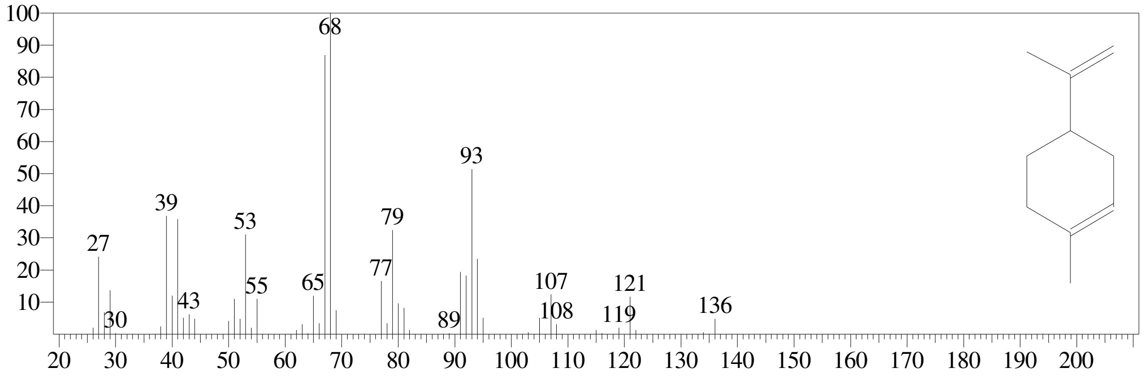

<< Target >>
Line#:2   R.Time:9.550(Scan#:847) MassPeaks:25
RawMode:Averaged 9.542-9.558(846-848) BasePeak:68.05(19726)
BG Mode:Calc. from Peak Group 1 - Event 1 Scan

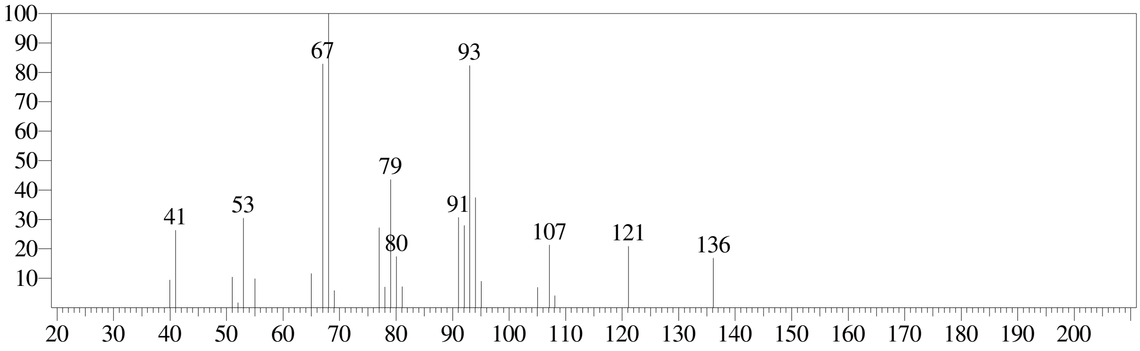

Hit#:5 Entry:8364 Library:NIST23s.lib
SI:90 Formula:C10H16 CAS:138-86-3 MolWeight:136 RetIndex:1031
CompName:Limonene $$ Cyclohexene, 1-methyl-4-(1-methylethenyl)- $$ p-Mentha-1,8-diene $$ .alpha.-Limonene $$ C

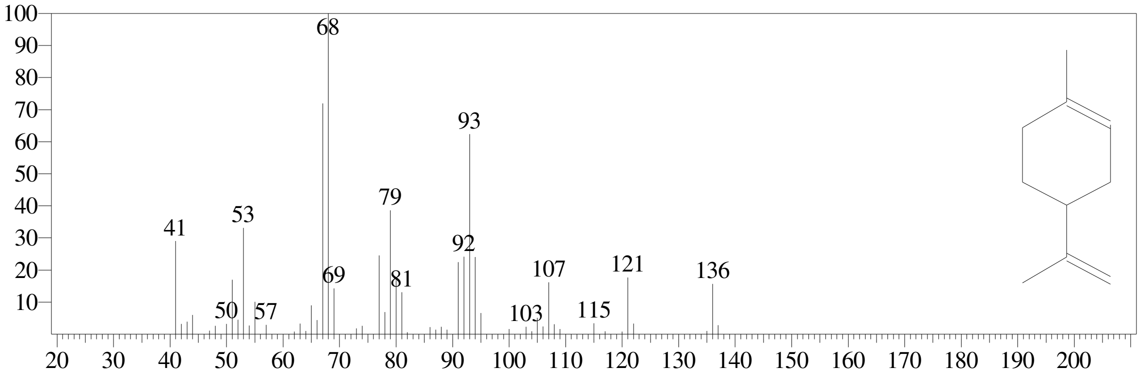

<< Target >>
Line#:3   R.Time:9.642(Scan#:858) MassPeaks:54
RawMode:Averaged 9.633-9.650(857-859) BasePeak:43.00(85586)
BG Mode:Calc. from Peak Group 1 - Event 1 Scan

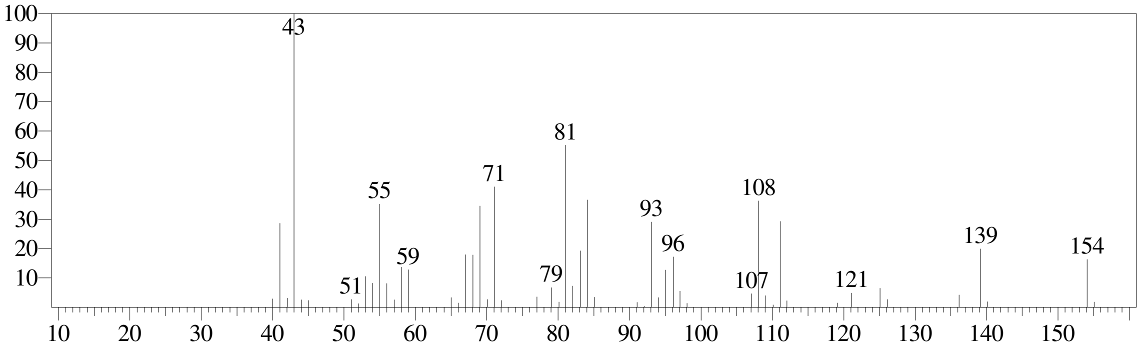

Hit#:1 Entry:12890 Library:NIST23s.lib
SI:95 Formula:C10H18O CAS:470-82-6 MolWeight:154 RetIndex:1028
CompName:Eucalyptol $$ Cineole $$ 2-Oxabicyclo[2.2.2]octane, 1,3,3-trimethyl- $$ p-Menthane, 1,8-epoxy- $$ p-Cineo

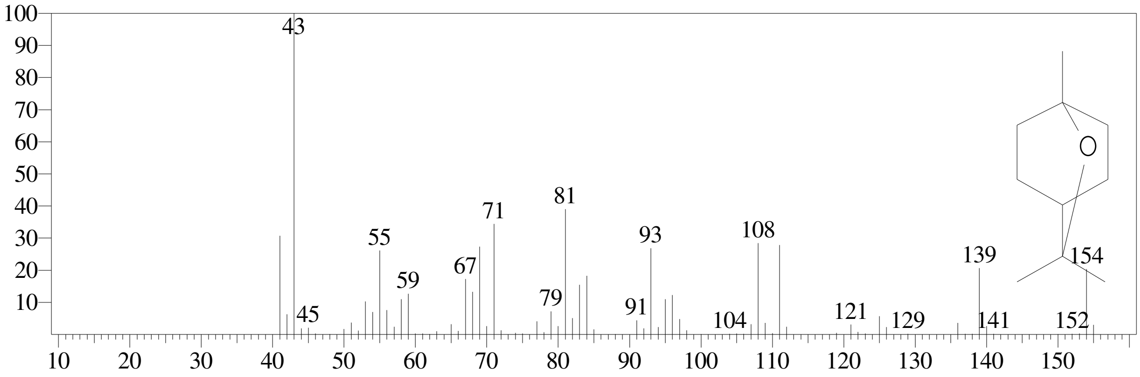

Hit#:2 Entry:12891 Library:NIST23s.lib
SI:94 Formula:C10H18O CAS:470-82-6 MolWeight:154 RetIndex:1028
CompName:Eucalyptol $$ Cineole $$ 2-Oxabicyclo[2.2.2]octane, 1,3,3-trimethyl- $$ p-Menthane, 1,8-epoxy- $$ p-Cineo

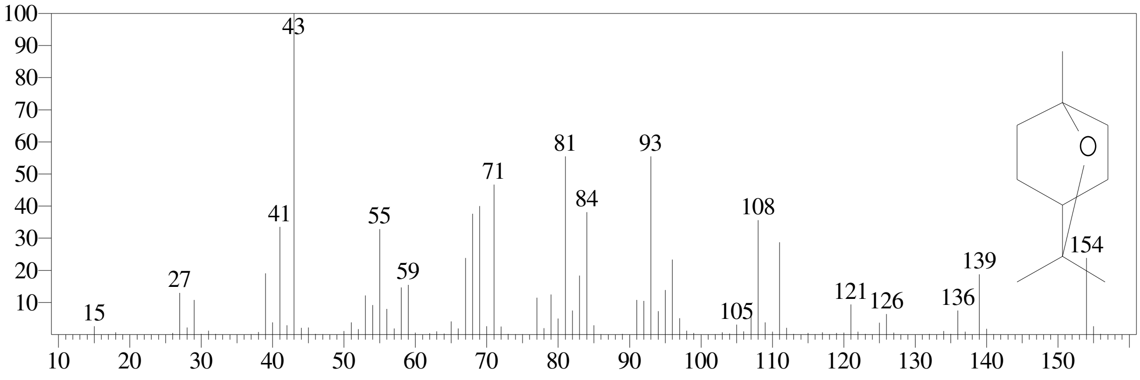

<< Target >>
Line#:3   R.Time:9.642(Scan#:858) MassPeaks:54
RawMode:Averaged 9.633-9.650(857-859) BasePeak:43.00(85586)
BG Mode:Calc. from Peak Group 1 - Event 1 Scan

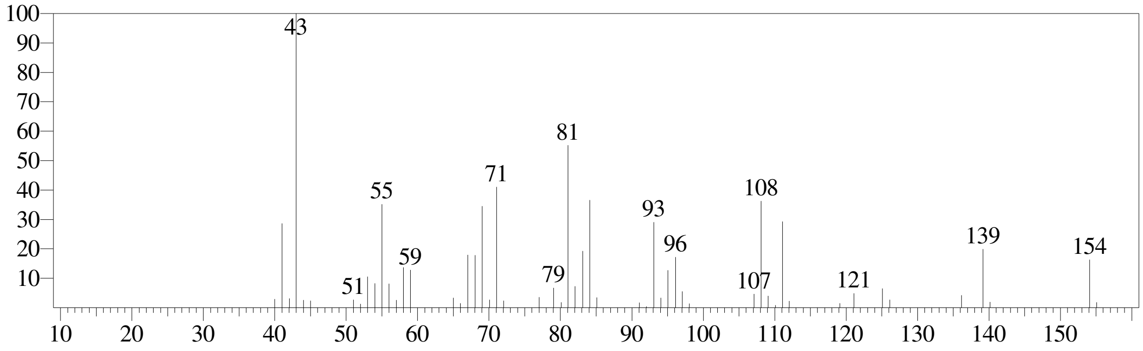

Hit#:3 Entry:20951 Library:NIST23-1.lib
SI:93 Formula:C10H18O CAS:470-82-6 MolWeight:154 RetIndex:1028
CompName:Eucalyptol $$ Cineole $$ 2-Oxabicyclo[2.2.2]octane, 1,3,3-trimethyl- $$ p-Menthane, 1,8-epoxy- $$ p-Cineo

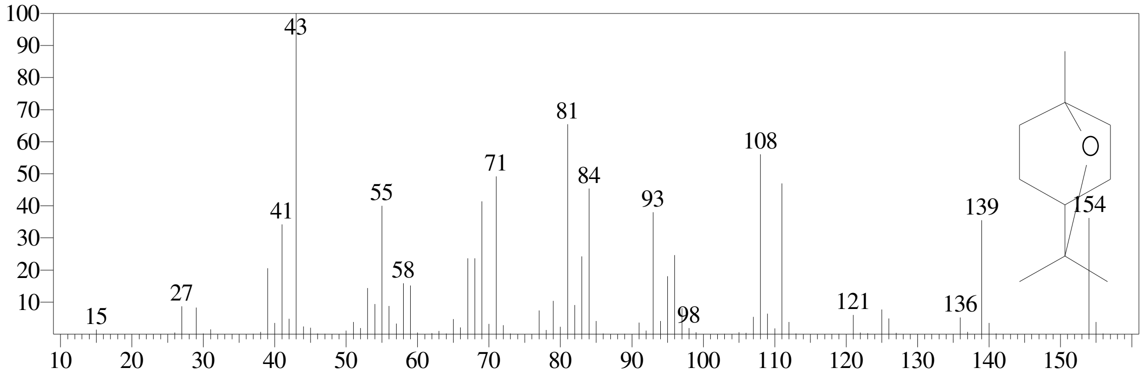

Hit#:4 Entry:12889 Library:NIST23s.lib
SI:93 Formula:C10H18O CAS:470-82-6 MolWeight:154 RetIndex:1028
CompName:Eucalyptol $$ Cineole $$ 2-Oxabicyclo[2.2.2]octane, 1,3,3-trimethyl- $$ p-Menthane, 1,8-epoxy- $$ p-Cineo

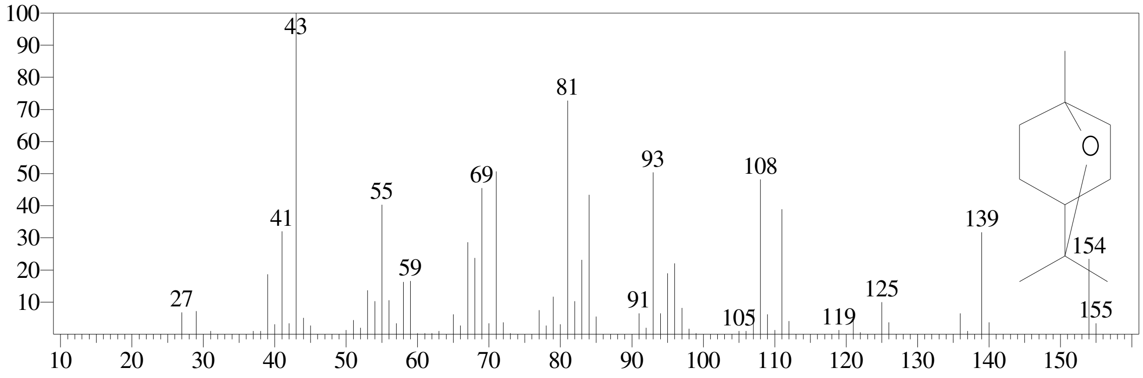

<< Target >>
Line#:3 R.Time:9.642(Scan#:858) MassPeaks:54
RawMode:Averaged 9.633-9.650(857-859) BasePeak:43.00(85586)
BG Mode:Calc. from Peak Group 1 - Event 1 Scan

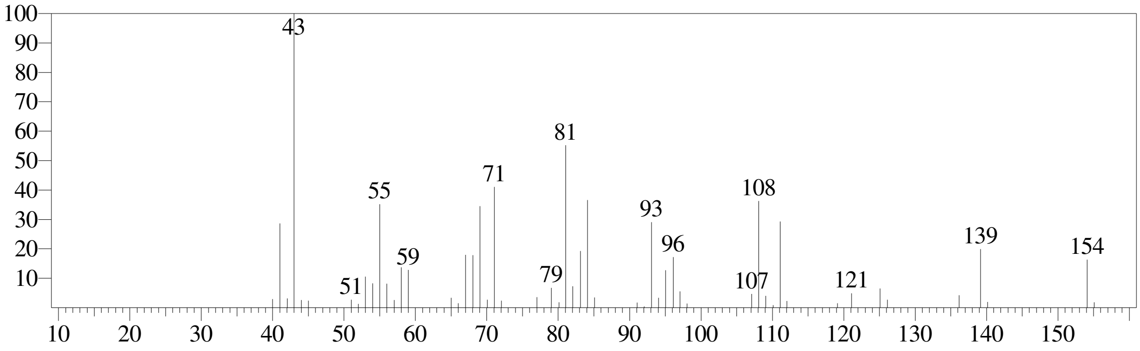

Hit#:5 Entry:12893 Library:NIST23s.lib
SI:93 Formula:C10H18O CAS:470-82-6 MolWeight:154 RetIndex:1028
CompName:Eucalyptol $$ Cineole $$ 2-Oxabicyclo[2.2.2]octane, 1,3,3-trimethyl- $$ p-Menthane, 1,8-epoxy- $$ p-Cineo

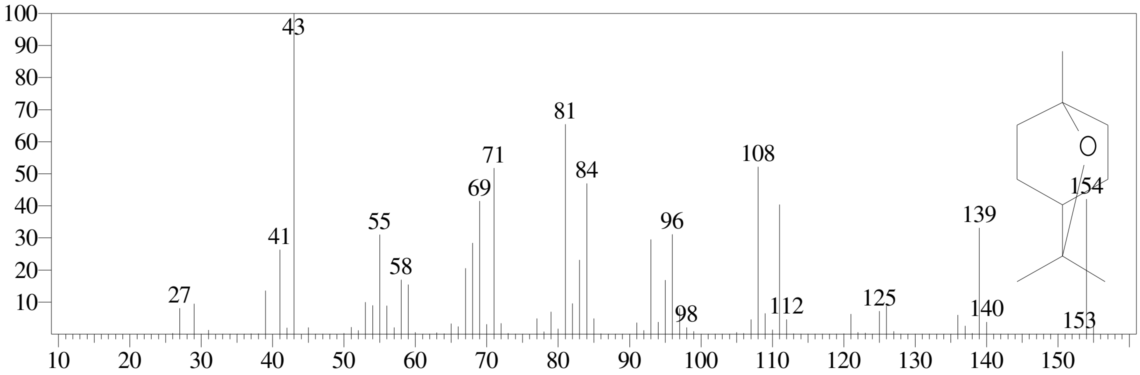

<< Target >>
Line#:4 R.Time:10.725(Scan#:988) MassPeaks:19
RawMode:Averaged 10.717-10.733(987-989) BasePeak:93.05(24039)
BG Mode:Calc. from Peak Group 1 - Event 1 Scan

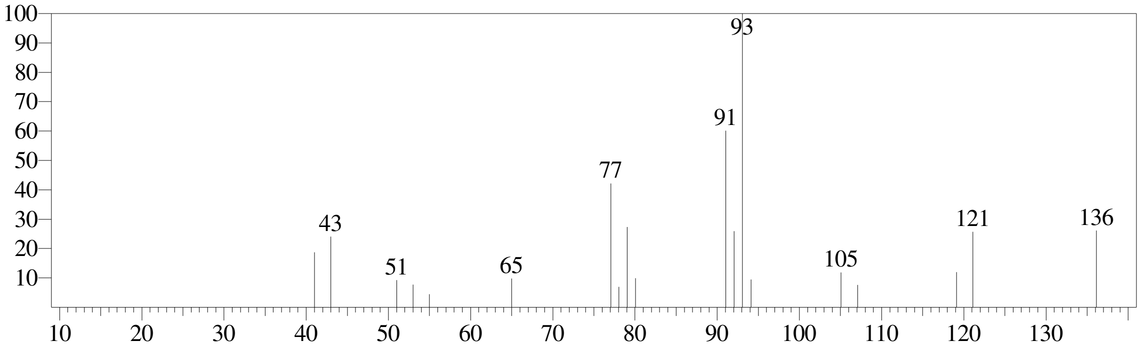

Hit#:1 Entry:8426 Library:NIST23s.lib
SI:93 Formula:C10H16 CAS:99-85-4 MolWeight:136 RetIndex:1038
CompName:.gamma.-Terpinene $$ 1,4-Cyclohexadiene, 1-methyl-4-(1-methylethyl)- $$ .gamma.-Terpinen $$ p-Mentha-

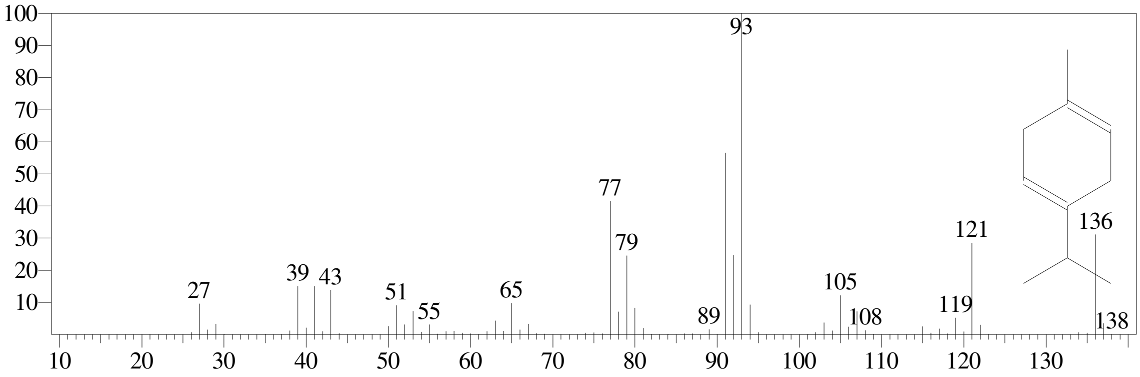

Hit#:2 Entry:8427 Library:NIST23s.lib
SI:92 Formula:C10H16 CAS:99-85-4 MolWeight:136 RetIndex:1038
CompName:.gamma.-Terpinene $$ 1,4-Cyclohexadiene, 1-methyl-4-(1-methylethyl)- $$ .gamma.-Terpinen $$ p-Mentha-

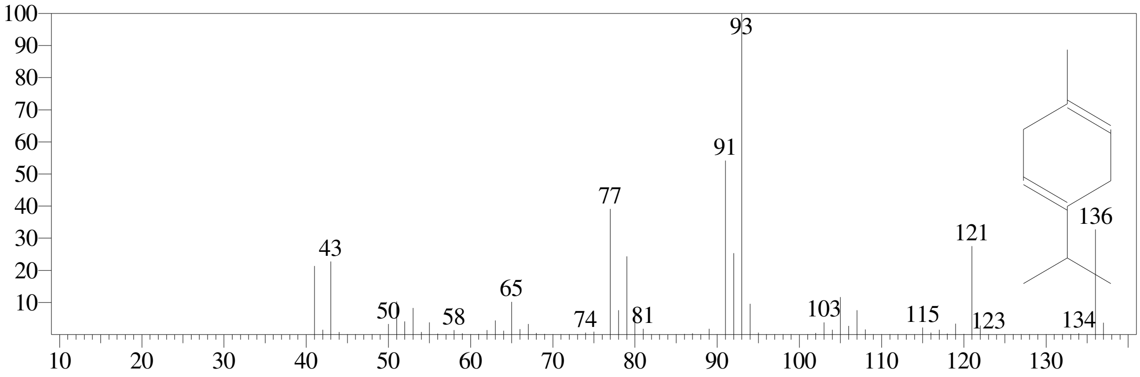

<< Target >>
Line#:4 R.Time:10.725(Scan#:988) MassPeaks:19
RawMode:Averaged 10.717-10.733(987-989) BasePeak:93.05(24039)
BG Mode:Calc. from Peak Group 1 - Event 1 Scan

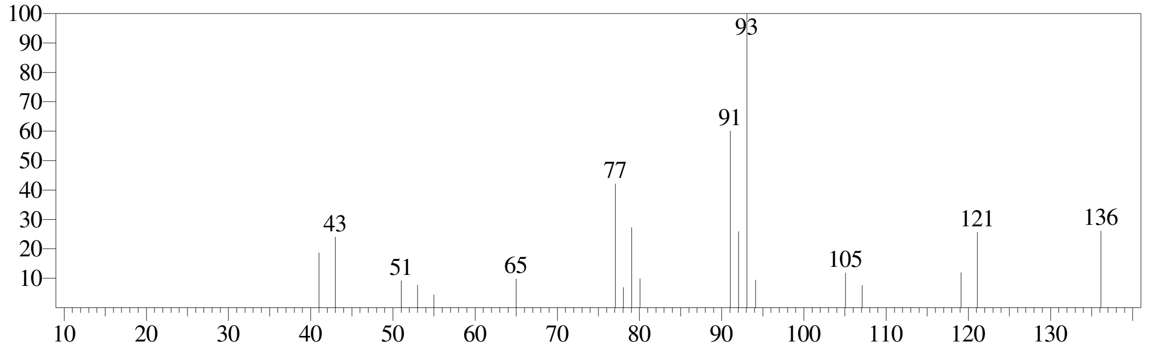

Hit#:3 Entry:8424 Library:NIST23s.lib
SI:91 Formula:C10H16 CAS:99-85-4 MolWeight:136 RetIndex:1038
CompName:.gamma.-Terpinene $$ 1,4-Cyclohexadiene, 1-methyl-4-(1-methylethyl)- $$ .gamma.-Terpinen $$ p-Mentha-

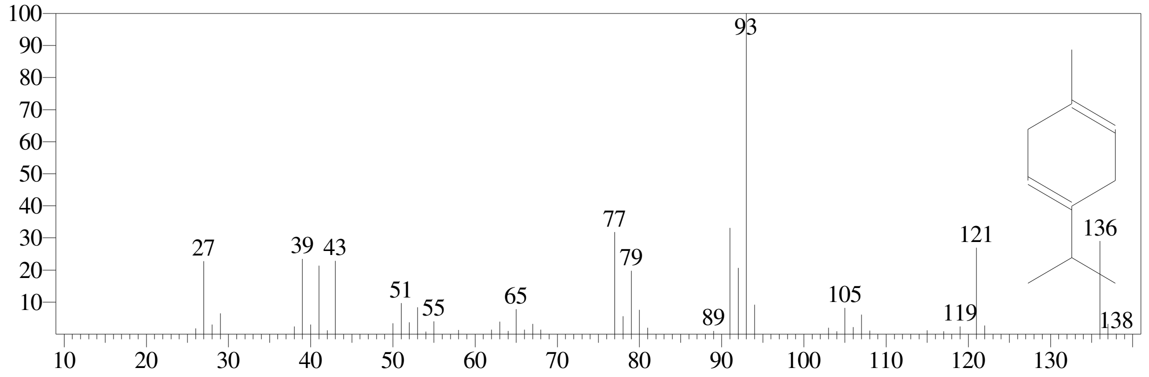

Hit#:4 Entry:8425 Library:NIST23s.lib
SI:90 Formula:C10H16 CAS:99-85-4 MolWeight:136 RetIndex:1038
CompName:.gamma.-Terpinene $$ 1,4-Cyclohexadiene, 1-methyl-4-(1-methylethyl)- $$ .gamma.-Terpinen $$ p-Mentha-

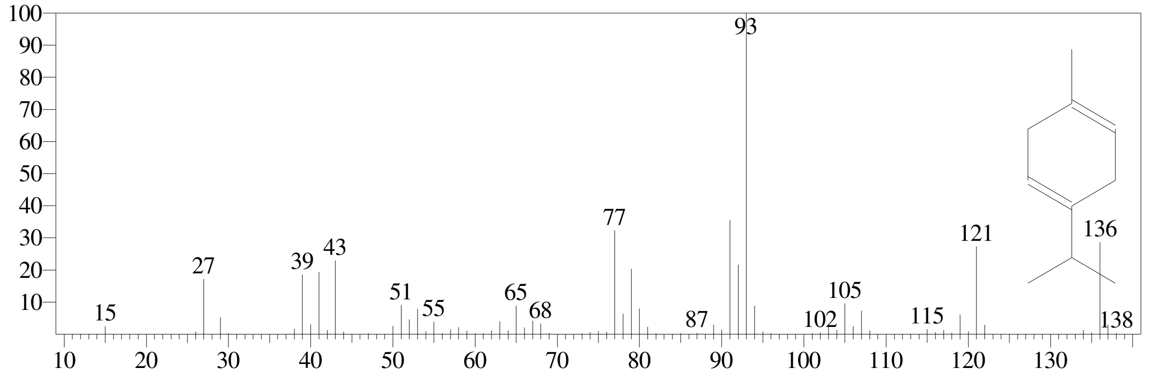

<< Target >>
Line#:4   R.Time:10.725(Scan#:988) MassPeaks:19
RawMode:Averaged 10.717-10.733(987-989) BasePeak:93.05(24039)
BG Mode:Calc. from Peak Group 1 - Event 1 Scan

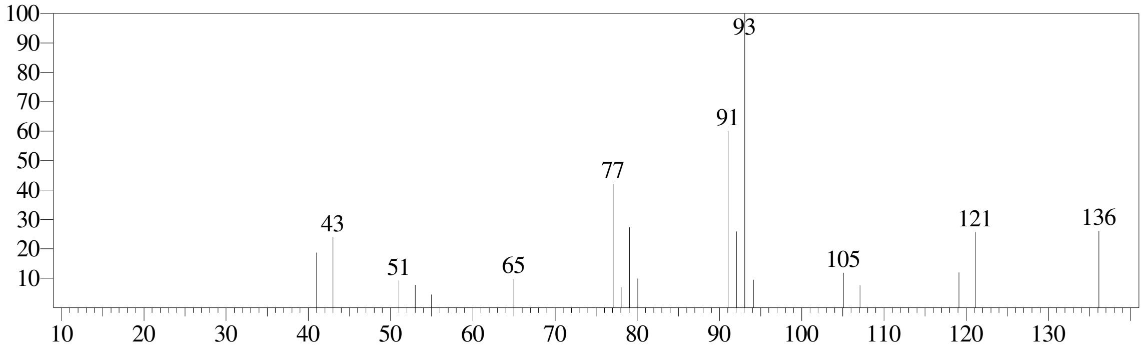

Hit#:5 Entry:8446 Library:NIST23s.lib
SI:90 Formula:C10H16 CAS:99-85-4 MolWeight:136 RetIndex:1038
CompName:.gamma.-Terpinene $$ 1,4-Cyclohexadiene, 1-methyl-4-(1-methylethyl)- $$ .gamma.-Terpinen $$ p-Mentha-

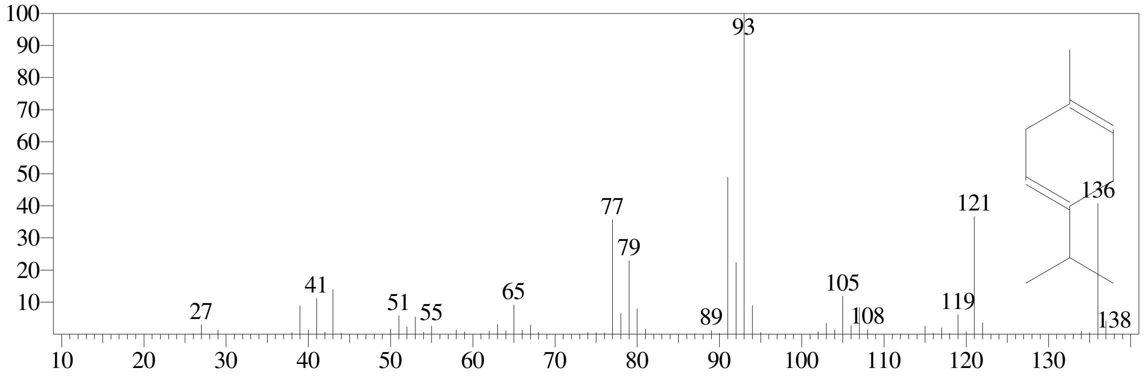

<< Target >>
Line#:5 R.Time:24.325(Scan#:2620) MassPeaks:46
RawMode:Averaged 24.317-24.333(2619-2621) BasePeak:105.05(38815)
BG Mode:Calc. from Peak Group 1 - Event 1 Scan

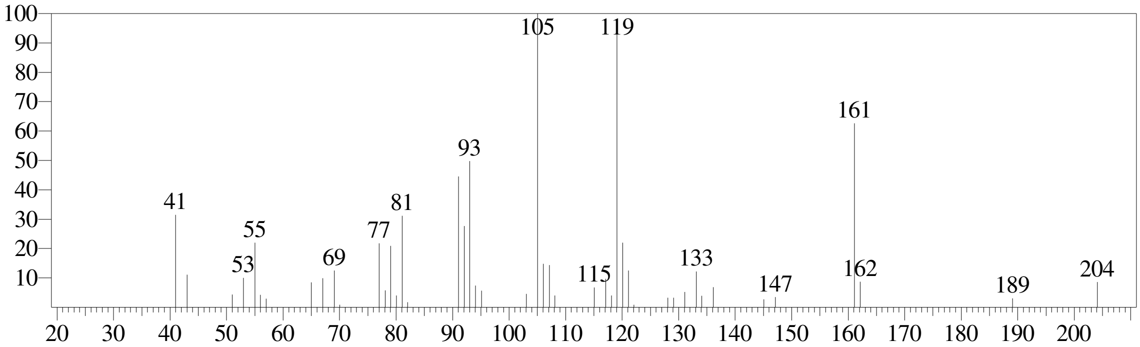

Hit#:1 Entry:25089 Library:NIST23s.lib
SI:95 Formula:C15H24 CAS:3856-25-5 MolWeight:204 RetIndex:1407
CompName:Copaene $$ Tricyclo[4.4.0.02,7]dec-3-ene, 1,3-dimethyl-8-(1-methylethyl)-, stereoisomer $$ Tricyclo[4.4.0.0

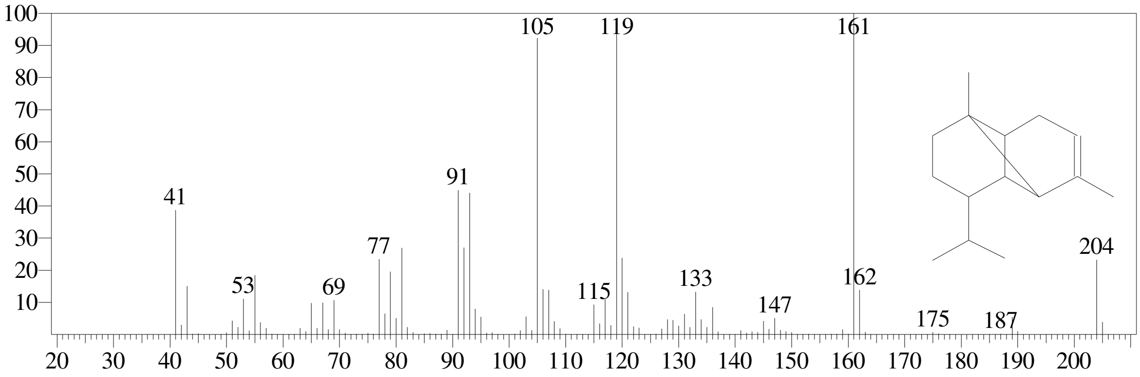

Hit#:2 Entry:62910 Library:NIST23-1.lib
SI:94 Formula:C15H24 CAS:3856-25-5 MolWeight:204 RetIndex:1407
CompName:Copaene $$ Tricyclo[4.4.0.02,7]dec-3-ene, 1,3-dimethyl-8-(1-methylethyl)-, stereoisomer $$ Tricyclo[4.4.0.0

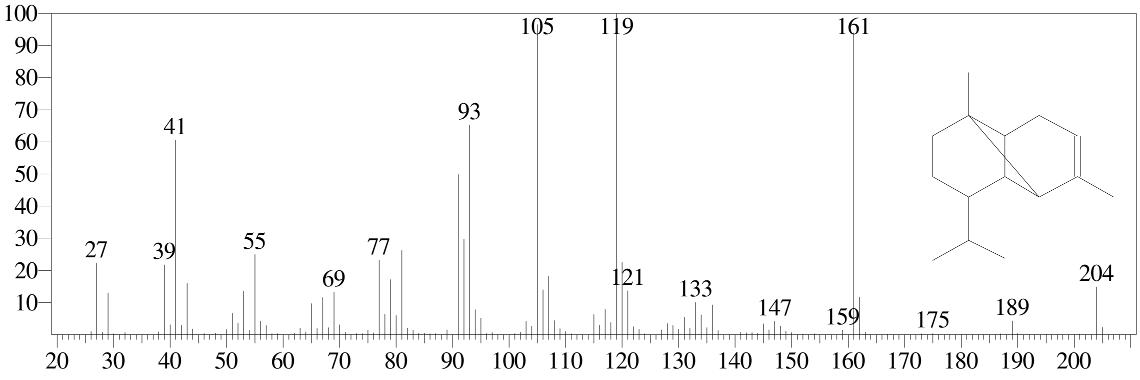

<< Target >>
Line#:5 R.Time:24.325(Scan#:2620) MassPeaks:46
RawMode:Averaged 24.317-24.333(2619-2621) BasePeak:105.05(38815)
BG Mode:Calc. from Peak Group 1 - Event 1 Scan

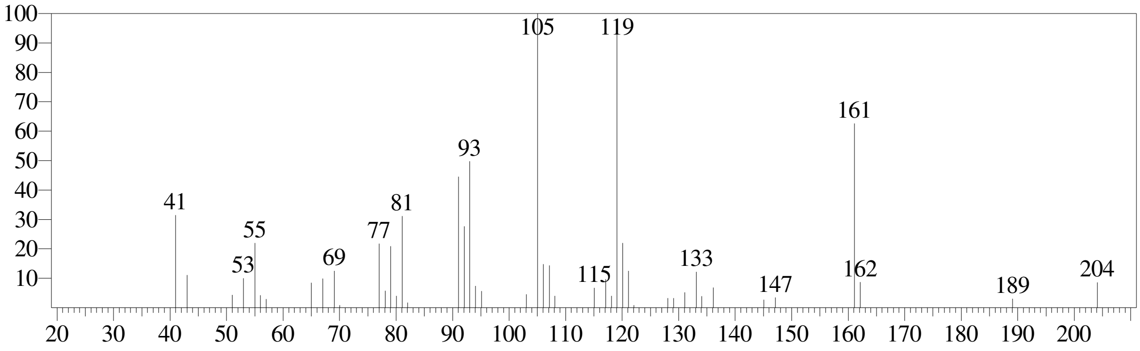

Hit#:3 Entry:25087 Library:NIST23s.lib
SI:94 Formula:C15H24 CAS:3856-25-5 MolWeight:204 RetIndex:1407
CompName:Copaene $$ Tricyclo[4.4.0.02,7]dec-3-ene, 1,3-dimethyl-8-(1-methylethyl)-, stereoisomer $$ Tricyclo[4.4.0.0

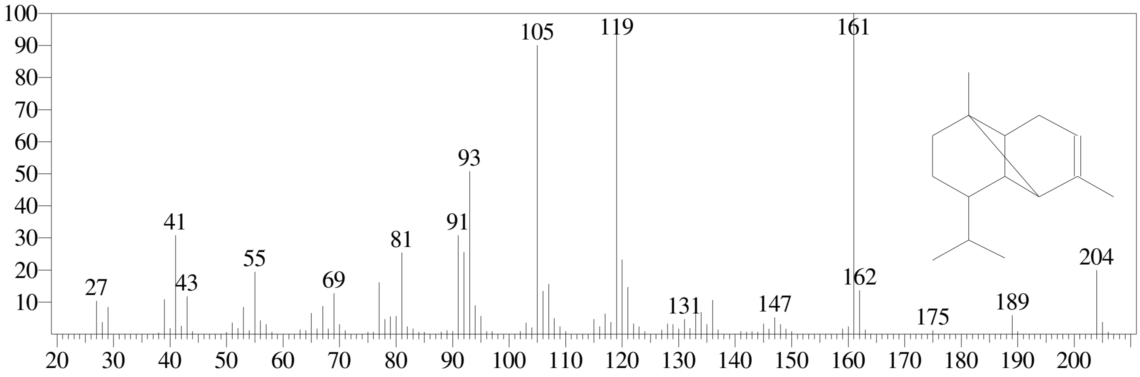

Hit#:4 Entry:24948 Library:NIST23s.lib
SI:93 Formula:C15H24 CAS:17699-14-8 MolWeight:204 RetIndex:1381
CompName:.alpha.-Cubebene $$ 1H-Cyclopenta[1,3]cyclopropa[1,2]benzene, 3a,3b,4,5,6,7-hexahydro-3,7-dimethyl-4-(1-

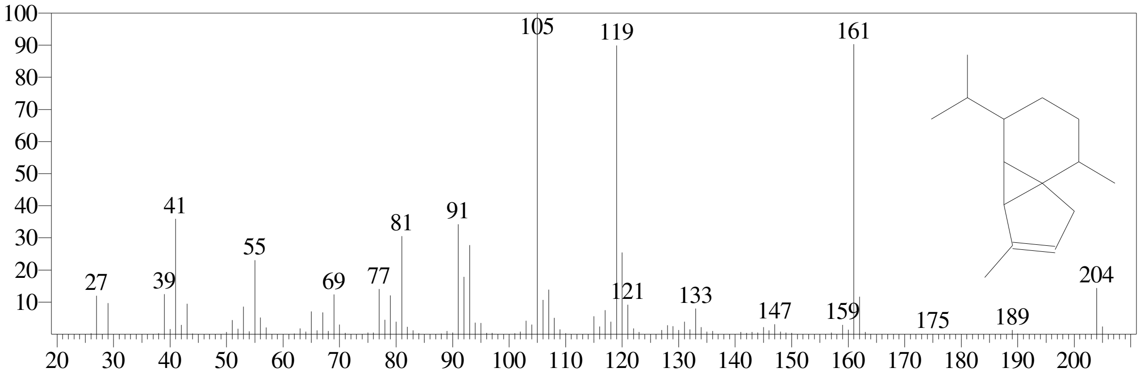

<< Target >>
Line#:5   R.Time:24.325(Scan#:2620) MassPeaks:46
RawMode:Averaged 24.317-24.333(2619-2621) BasePeak:105.05(38815)
BG Mode:Calc. from Peak Group 1 - Event 1 Scan

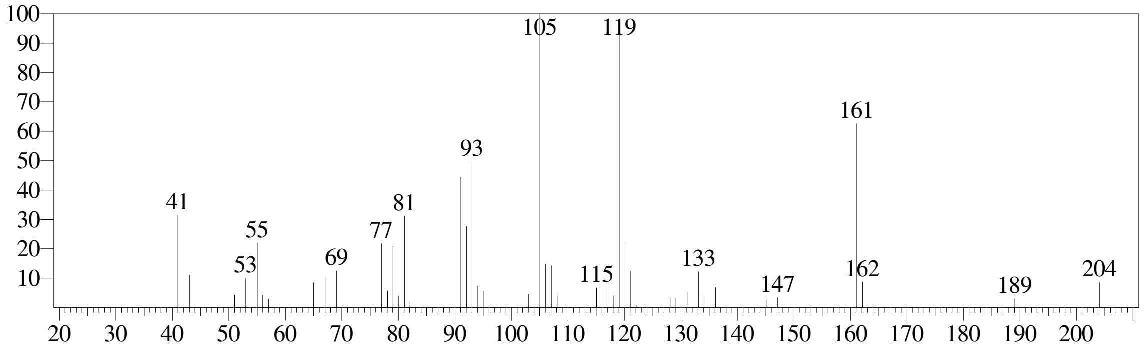

Hit#:5 Entry:25081 Library:NIST23s.lib
SI:93 Formula:C15H24 CAS:3856-25-5 MolWeight:204 RetIndex:1407
CompName:Copaene $$ Tricyclo[4.4.0.02,7]dec-3-ene, 1,3-dimethyl-8-(1-methylethyl)-, stereoisomer $$ Tricyclo[4.4.0.0

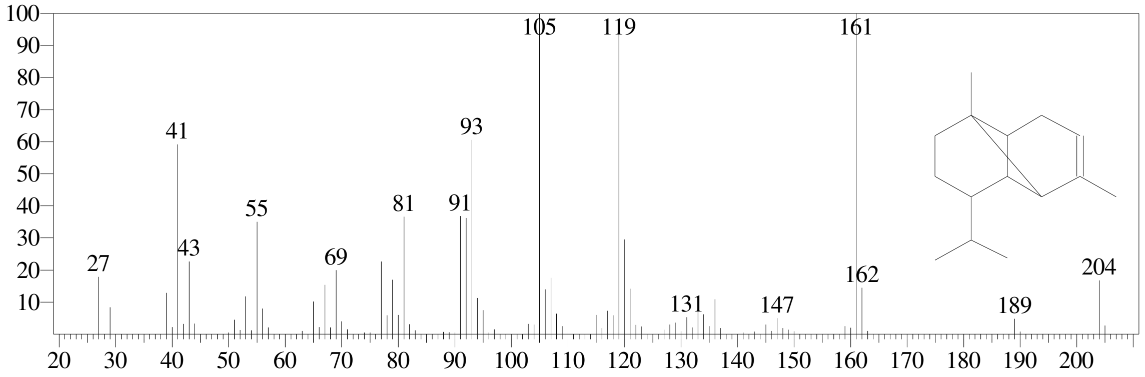

<< Target >>
Line#:6 R.Time:25.008(Scan#:2702) MassPeaks:35
RawMode:Averaged 25.000-25.017(2701-2703) BasePeak:93.05(12045)
BG Mode:None Group 1 - Event 1 Scan

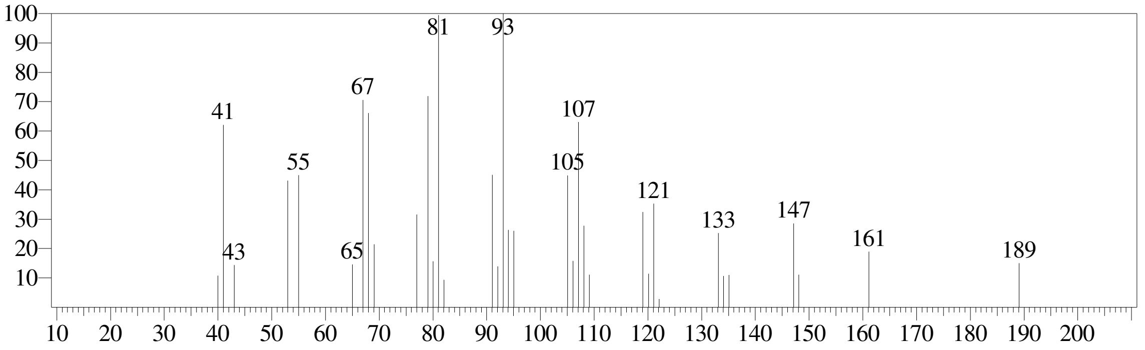

Hit#:1 Entry:24863 Library:NIST23s.lib
SI:95 Formula:C15H24 CAS:515-13-9 MolWeight:204 RetIndex:1398
CompName:Cyclohexane, 1-ethenyl-1-methyl-2,4-bis(1-methylethenyl)-, [1S-(1.alpha.,2.beta.,4.beta.)]- $$ Cyclohexane, [illegible]

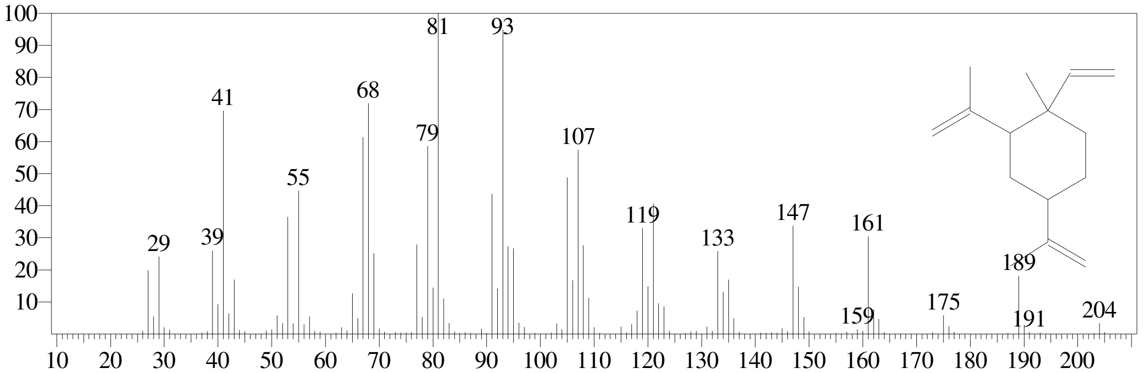

Hit#:2 Entry:24889 Library:NIST23s.lib
SI:93 Formula:C15H24 CAS:515-13-9 MolWeight:204 RetIndex:1398
CompName:Cyclohexane, 1-ethenyl-1-methyl-2,4-bis(1-methylethenyl)-, [1S-(1.alpha.,2.beta.,4.beta.)]- $$ Cyclohexane, [illegible]

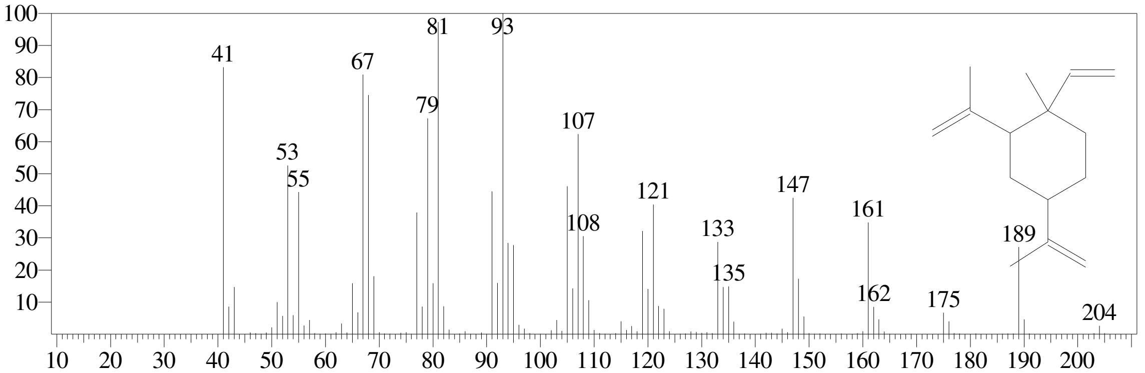

<< Target >>
Line#:6 R.Time:25.008(Scan#:2702) MassPeaks:35
RawMode:Averaged 25.000-25.017(2701-2703) BasePeak:93.05(12045)
BG Mode:None Group 1 - Event 1 Scan

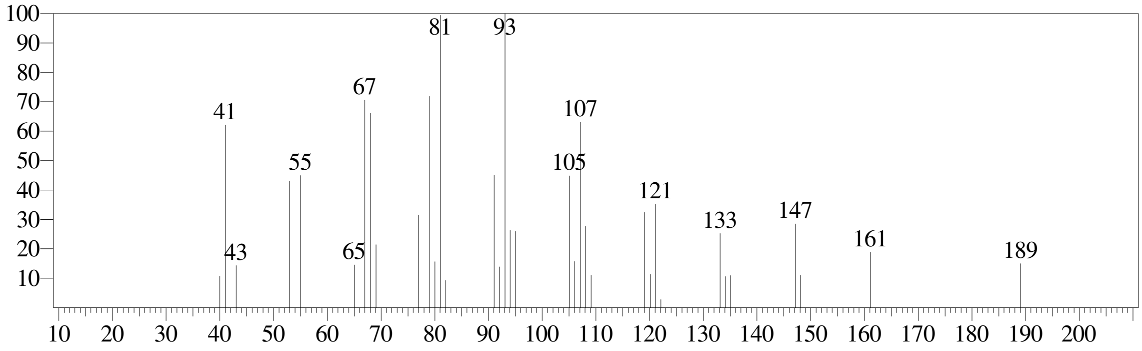

Hit#:3 Entry:62789 Library:NIST23-1.lib
SI:92 Formula:C15H24 CAS:515-13-9 MolWeight:204 RetIndex:1398
CompName:Cyclohexane, 1-ethenyl-1-methyl-2,4-bis(1-methylethenyl)-, [1S-(1.alpha.,2.beta.,4.beta.)]- $$ Cyclohexane, [illegible]

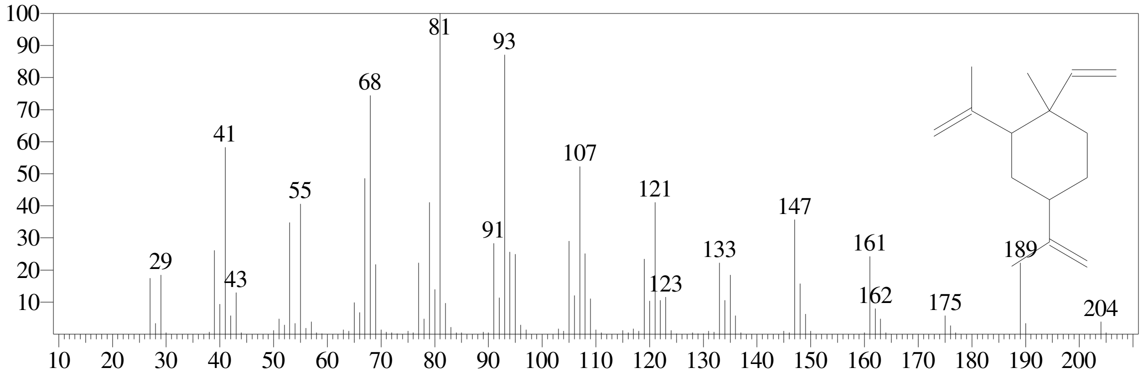

Hit#:4 Entry:62785 Library:NIST23-1.lib
SI:90 Formula:C15H24 CAS:110823-68-2 MolWeight:204 RetIndex:1398
CompName:Cyclohexane, 1-ethenyl-1-methyl-2,4-bis(1-methylethenyl)- $$ 2,4-Diisopropenyl-1-methyl-1-vinylcyclohexa[illegible]

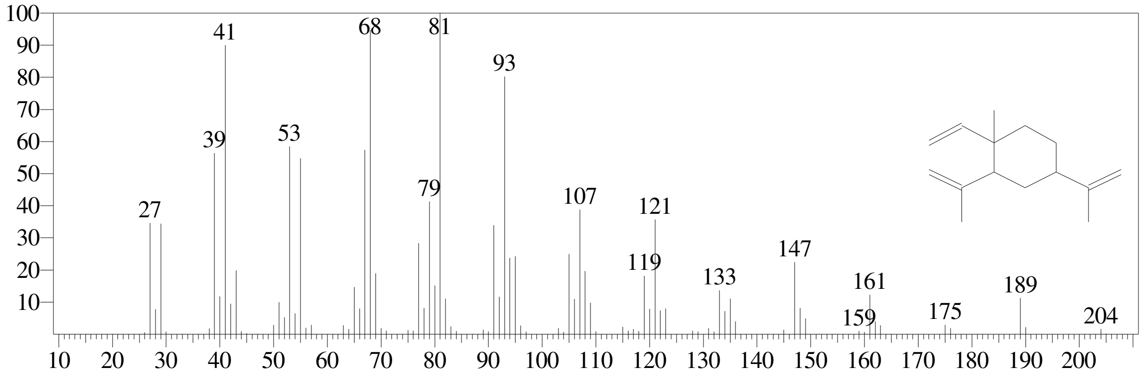

<< Target >>
Line#:6   R.Time:25.008(Scan#:2702) MassPeaks:35
RawMode:Averaged 25.000-25.017(2701-2703) BasePeak:93.05(12045)
BG Mode:None Group 1 - Event 1 Scan

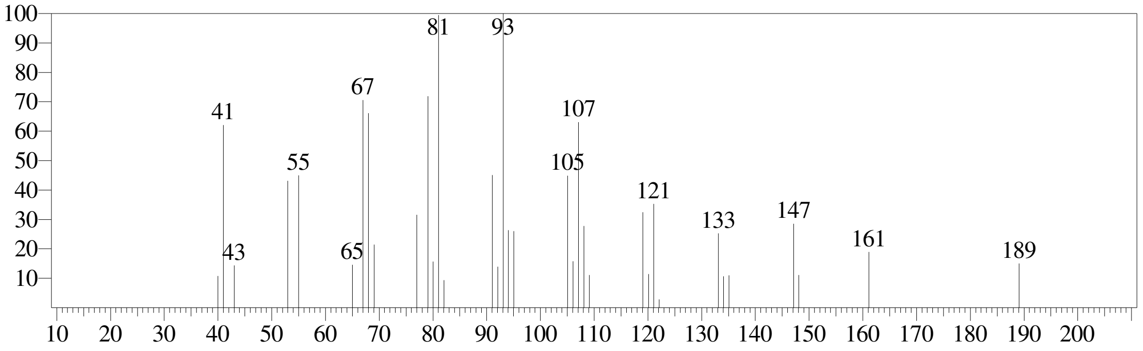

Hit#:5 Entry:24860 Library:NIST23s.lib
SI:90 Formula:C15H24 CAS:515-13-9 MolWeight:204 RetIndex:1398
CompName:Cyclohexane, 1-ethenyl-1-methyl-2,4-bis(1-methylethenyl)-, [1S-(1.alpha.,2.beta.,4.beta.)]- $$ Cyclohexane, 1

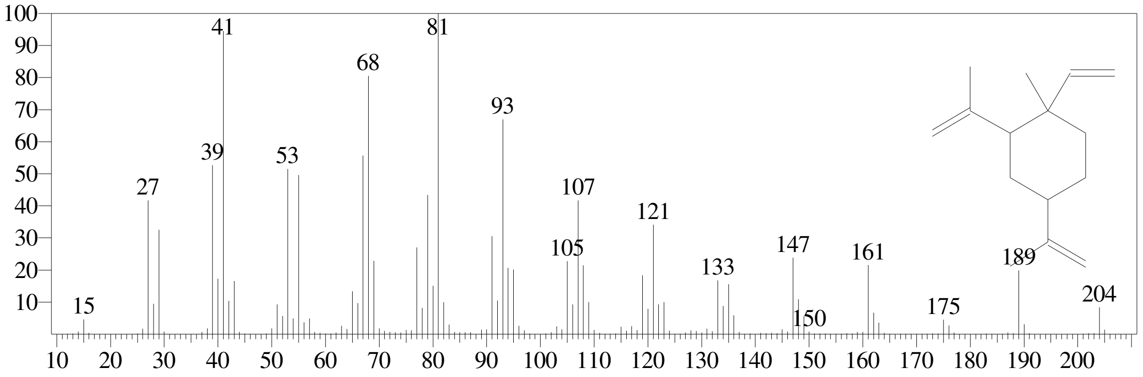

<< Target >>
Line#:7   R.Time:25.750(Scan#:2791) MassPeaks:42
RawMode:Averaged 25.742-25.758(2790-2792) BasePeak:105.05(14104)
BG Mode:Calc. from Peak Group 1 - Event 1 Scan

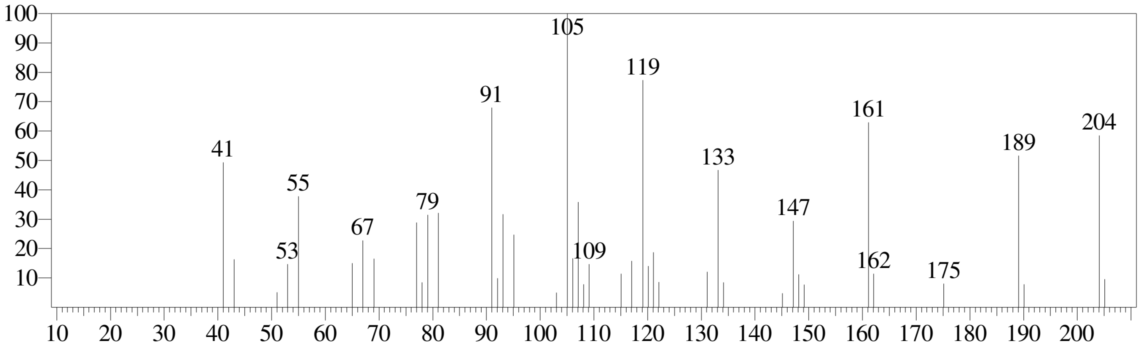

Hit#:1 Entry:63051 Library:NIST23-1.lib
SI:93 Formula:C15H24 CAS:489-40-7 MolWeight:204 RetIndex:1410
CompName:1H-Cycloprop[e]azulene, 1a,2,3,4,4a,5,6,7b-octahydro-1,1,4,7-tetramethyl-, [1aR-(1a.alpha.,4.alpha.,4a.beta.,

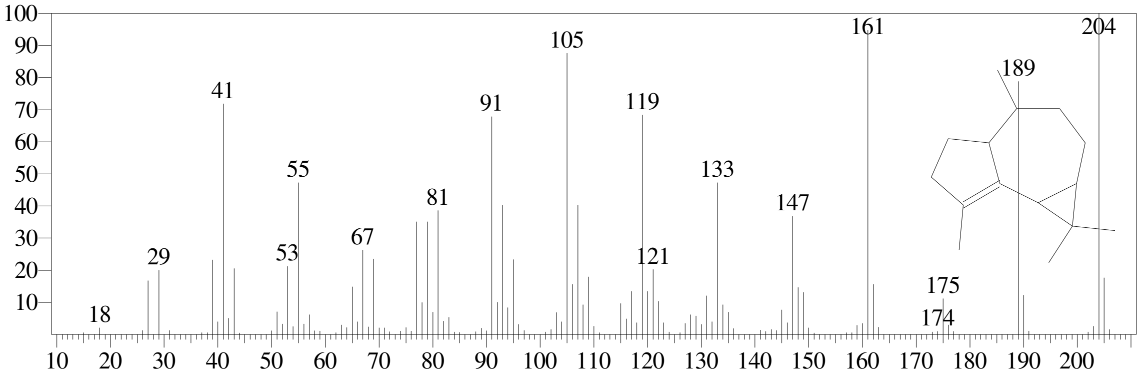

Hit#:2 Entry:62840 Library:NIST23-1.lib
SI:92 Formula:C15H24 CAS:53863-54-0 MolWeight:204 RetIndex:1494
CompName:Guaiene,trans-beta-

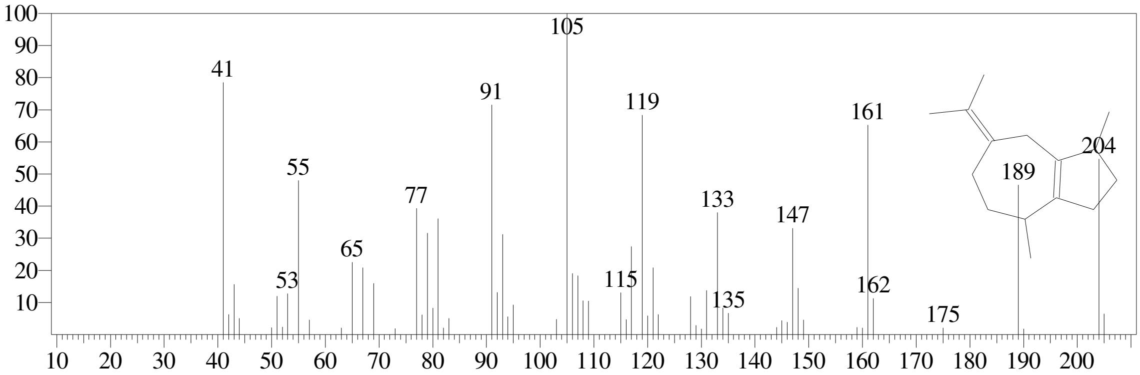

<< Target >>
Line#:7   R.Time:25.750(Scan#:2791) MassPeaks:42
RawMode:Averaged 25.742-25.758(2790-2792) BasePeak:105.05(14104)
BG Mode:Calc. from Peak Group 1 - Event 1 Scan

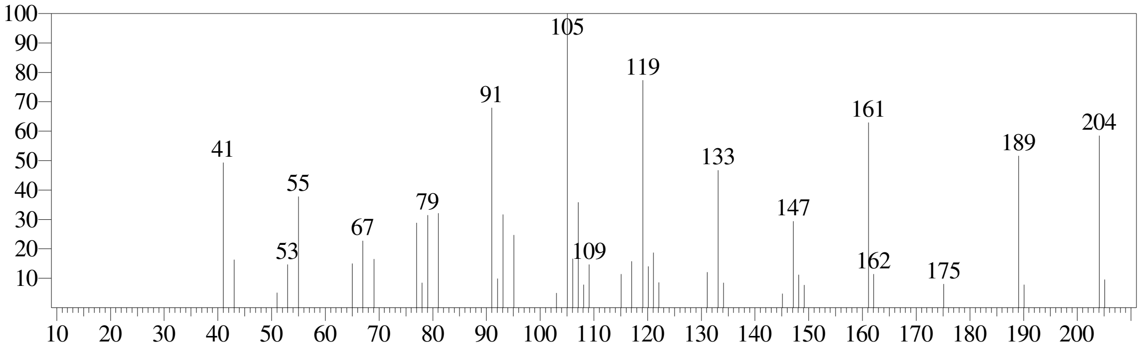

Hit#:3 Entry:25141 Library:NIST23s.lib
SI:92 Formula:C15H24 CAS:489-40-7 MolWeight:204 RetIndex:1410
CompName:1H-Cycloprop[e]azulene, 1a,2,3,4,4a,5,6,7b-octahydro-1,1,4,7-tetramethyl-, [1aR-(1a.alpha.,4.alpha.,4a.beta.,

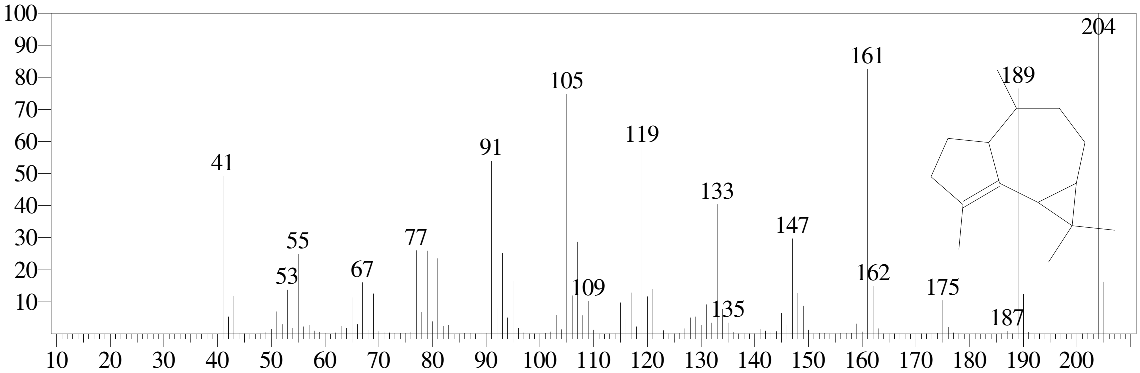

Hit#:4 Entry:25061 Library:NIST23s.lib
SI:91 Formula:C15H24 CAS:88-84-6 MolWeight:204 RetIndex:1494
CompName:.beta.-Guaiene $$ Azulene, 1,2,3,4,5,6,7,8-octahydro-1,4-dimethyl-7-(1-methylethylidene)-, (1S-cis)- $$ Guai

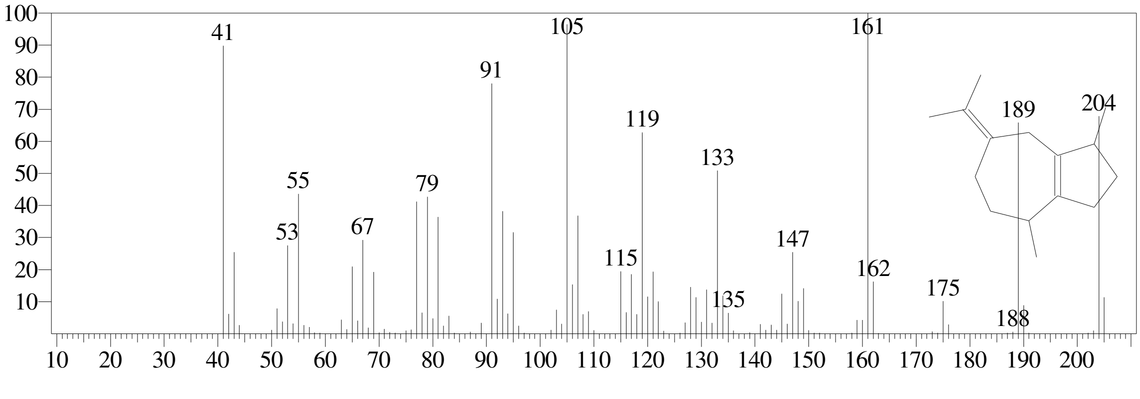

<< Target >>
Line#:7 R.Time:25.750(Scan#:2791) MassPeaks:42
RawMode:Averaged 25.742-25.758(2790-2792) BasePeak:105.05(14104)
BG Mode:Calc. from Peak Group 1 - Event 1 Scan

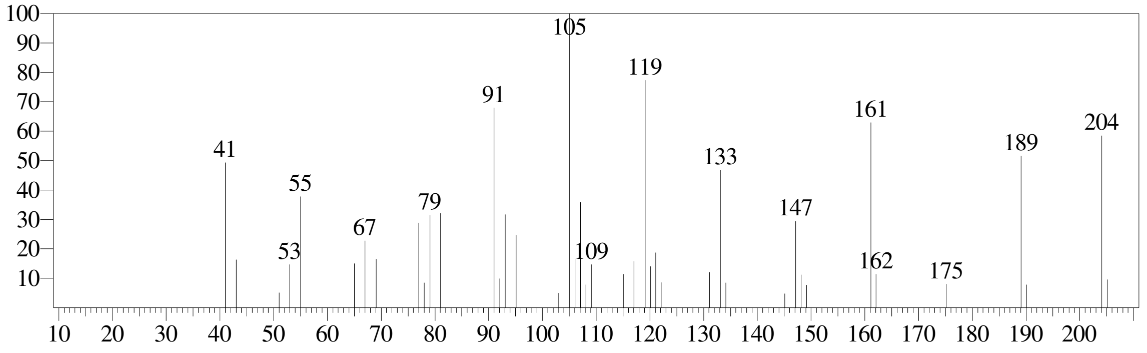

Hit#:5 Entry:24835 Library:NIST23s.lib
SI:91 Formula:C15H24 CAS:489-40-7 MolWeight:204 RetIndex:1410
CompName:1H-Cycloprop[e]azulene, 1a,2,3,4,4a,5,6,7b-octahydro-1,1,4,7-tetramethyl-, [1aR-(1a.alpha.,4.alpha.,4a.beta.,

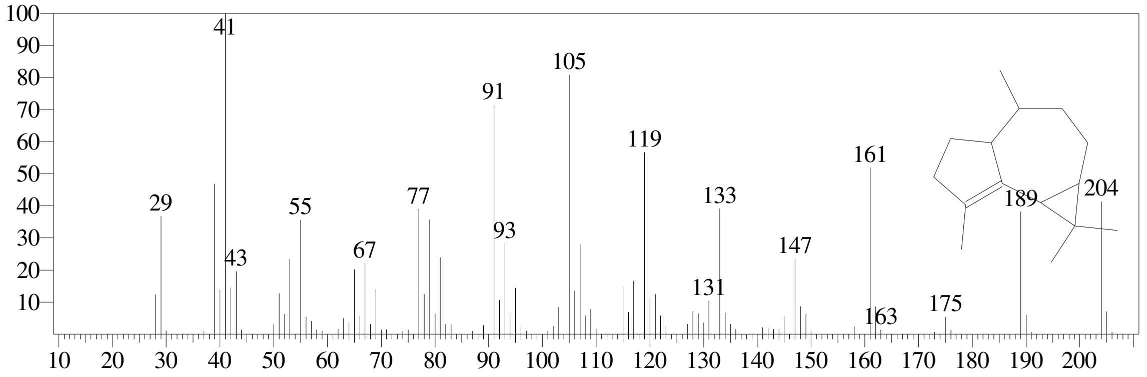

<< Target >>
Line#:8 R.Time:26.167(Scan#:2841) MassPeaks:74
RawMode:Averaged 26.158-26.175(2840-2842) BasePeak:93.05(106676)
BG Mode:None Group 1 - Event 1 Scan

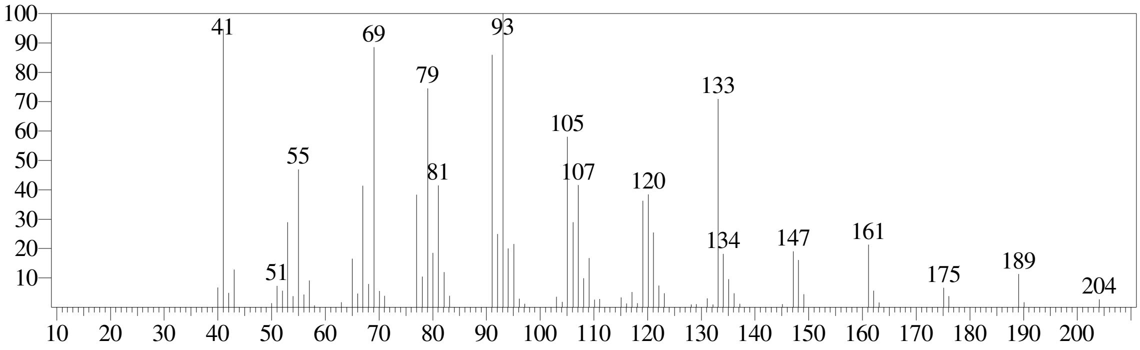

Hit#:1 Entry:24804 Library:NIST23s.lib
SI:95 Formula:C15H24 CAS:87-44-5 MolWeight:204 RetIndex:1448
CompName:Caryophyllene $$ Bicyclo[7.2.0]undec-4-ene, 4,11,11-trimethyl-8-methylene-, [1R-(1R*,4E,9S*)]- $$ Bicycl

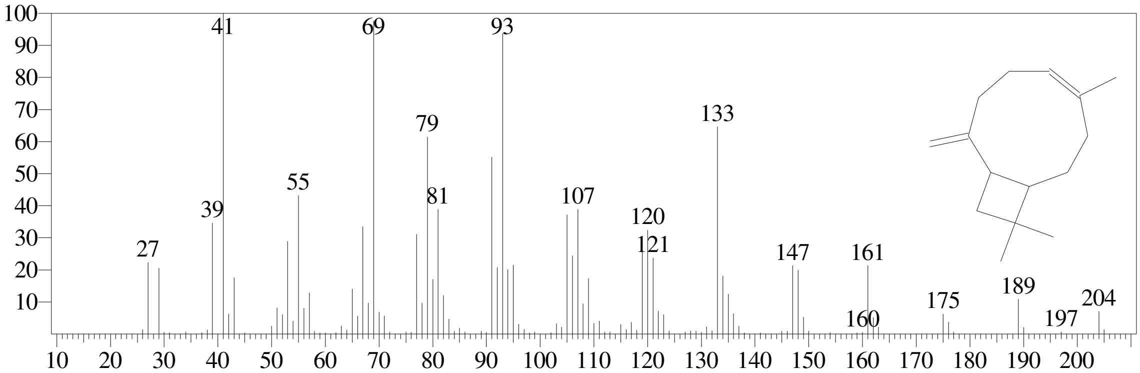

Hit#:2 Entry:62827 Library:NIST23-1.lib
SI:95 Formula:C15H24 CAS:87-44-5 MolWeight:204 RetIndex:1448
CompName:Caryophyllene $$ Bicyclo[7.2.0]undec-4-ene, 4,11,11-trimethyl-8-methylene-, [1R-(1R*,4E,9S*)]- $$ Bicycl

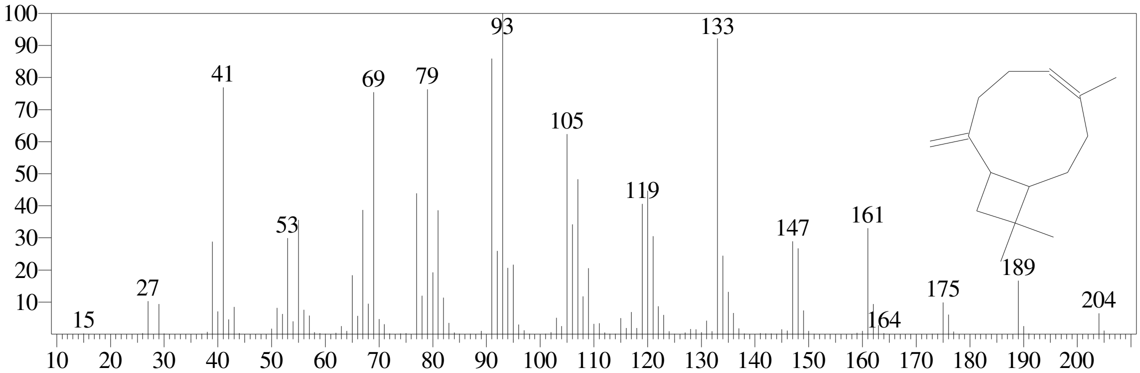

<< Target >>
Line#:8 R.Time:26.167(Scan#:2841) MassPeaks:74
RawMode:Averaged 26.158-26.175(2840-2842) BasePeak:93.05(106676)
BG Mode:None Group 1 - Event 1 Scan

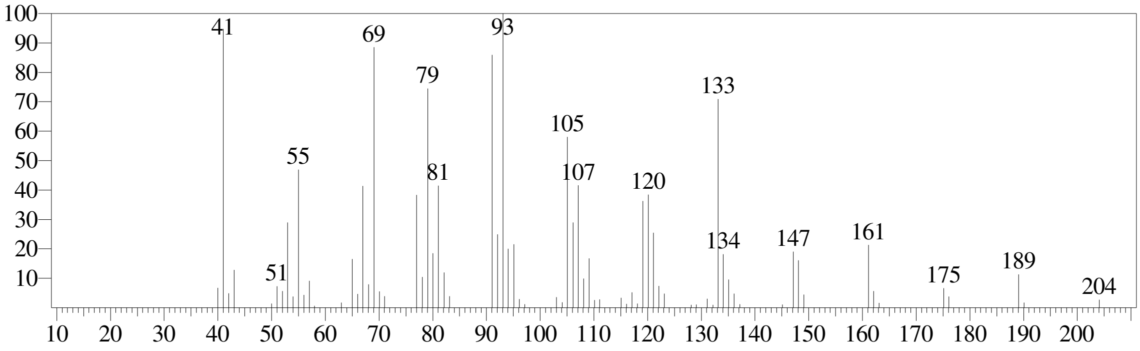

Hit#:3 Entry:24884 Library:NIST23s.lib
SI:94 Formula:C15H24 CAS:13877-93-5 MolWeight:204 RetIndex:1448
CompName:Bicyclo[7.2.0]undec-4-ene, 4,11,11-trimethyl-8-methylene- $$ Bicyclo[7.2.0]undec-4-ene, 4,11,11-trimethyl-

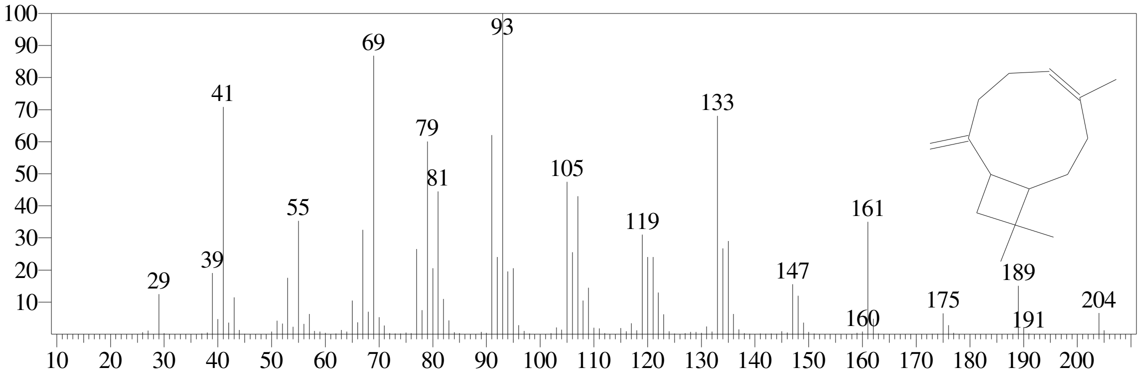

Hit#:4 Entry:62803 Library:NIST23-1.lib
SI:94 Formula:C15H24 CAS:13877-93-5 MolWeight:204 RetIndex:1448
CompName:Bicyclo[7.2.0]undec-4-ene, 4,11,11-trimethyl-8-methylene- $$ Bicyclo[7.2.0]undec-4-ene, 4,11,11-trimethyl-

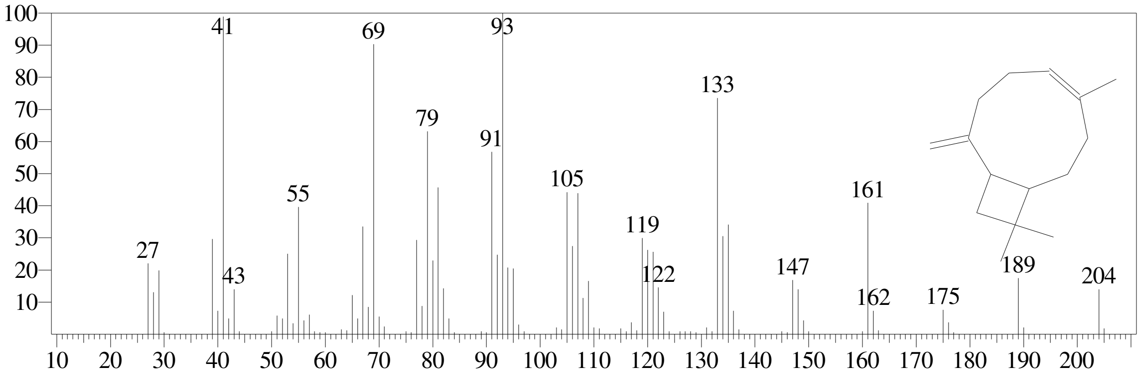

<< Target >>
Line#:8   R.Time:26.167(Scan#:2841) MassPeaks:74
RawMode:Averaged 26.158-26.175(2840-2842) BasePeak:93.05(106676)
BG Mode:None Group 1 - Event 1 Scan

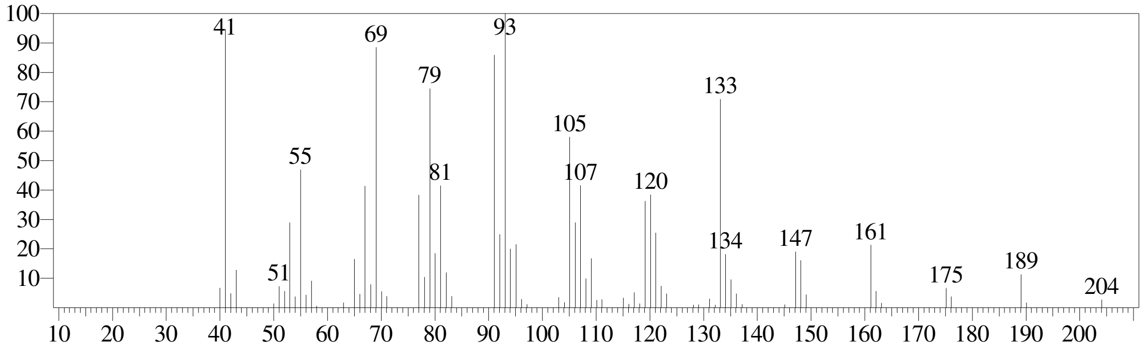

Hit#:5 Entry:24803 Library:NIST23s.lib
SI:94 Formula:C15H24 CAS:118-65-0 MolWeight:204 RetIndex:1448
CompName:Bicyclo[7.2.0]undec-4-ene, 4,11,11-trimethyl-8-methylene-,[1R-(1R*,4Z,9S*)]- $$ Isocaryophyllene $$ 4,11,

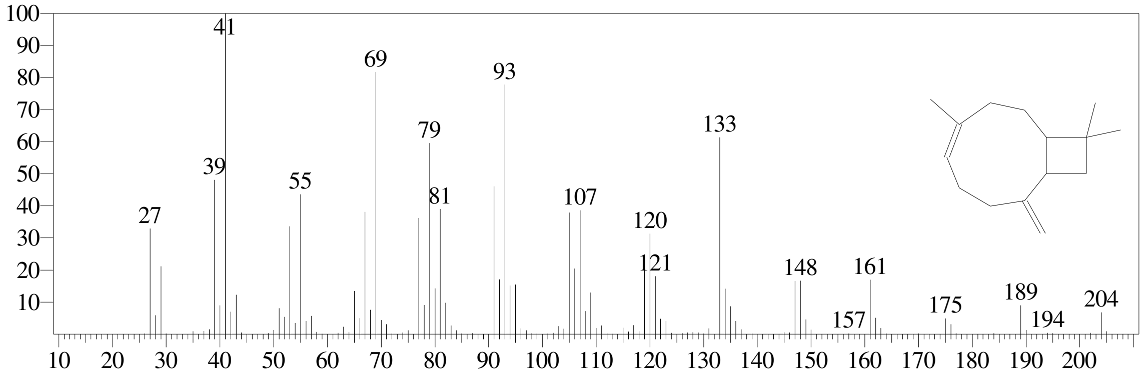

<< Target >>
Line#:9 R.Time:26.950(Scan#:2935) MassPeaks:50
RawMode:Averaged 26.942-26.958(2934-2936) BasePeak:41.00(19624)
BG Mode:Calc. from Peak Group 1 - Event 1 Scan

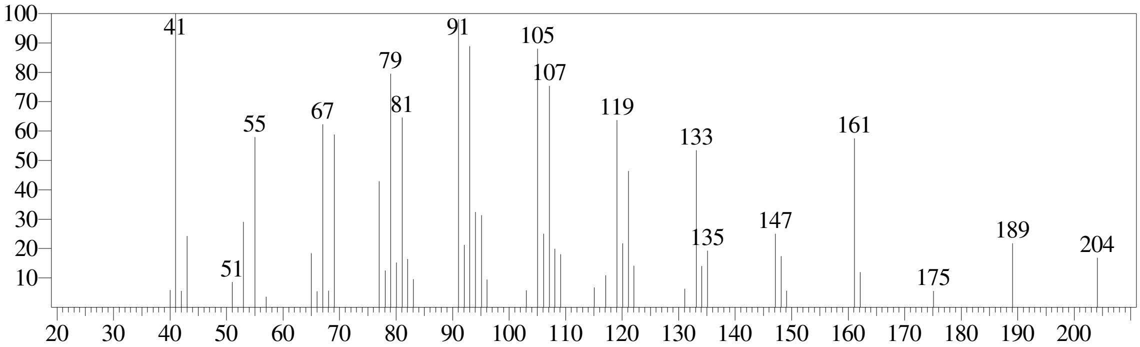

Hit#:1 Entry:24869 Library:NIST23s.lib
SI:95 Formula:C15H24 CAS:489-39-4 MolWeight:204 RetIndex:1424
CompName:Aromandendrene $$ 1H-Cycloprop[e]azulene, decahydro-1,1,7-trimethyl-4-methylene-, [1aR-(1a.alpha.,4a.al

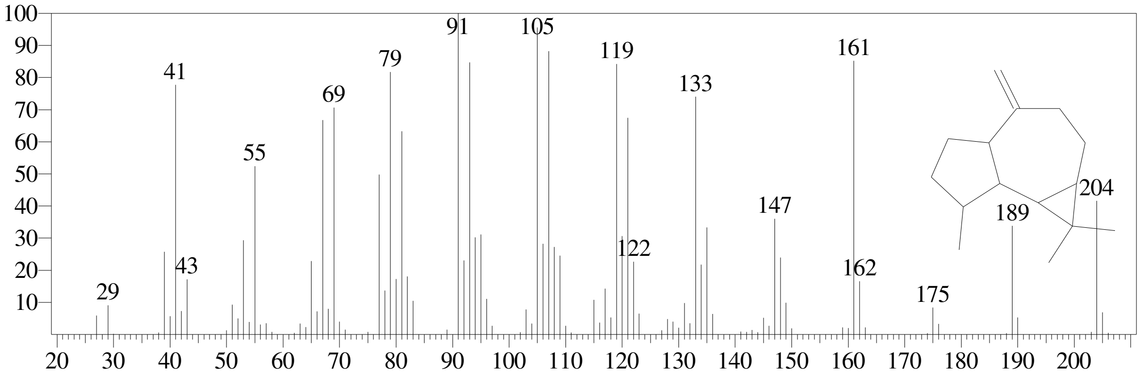

Hit#:2 Entry:62794 Library:NIST23-1.lib
SI:94 Formula:C15H24 CAS:68832-35-9 MolWeight:204 RetIndex:1450
CompName:(1R,9R,E)-4,11,11-Trimethyl-8-methylenebicyclo[7.2.0]undec-4-ene $$ Bicyclo[7.2.0]undec-4-ene, 4,11,11-t

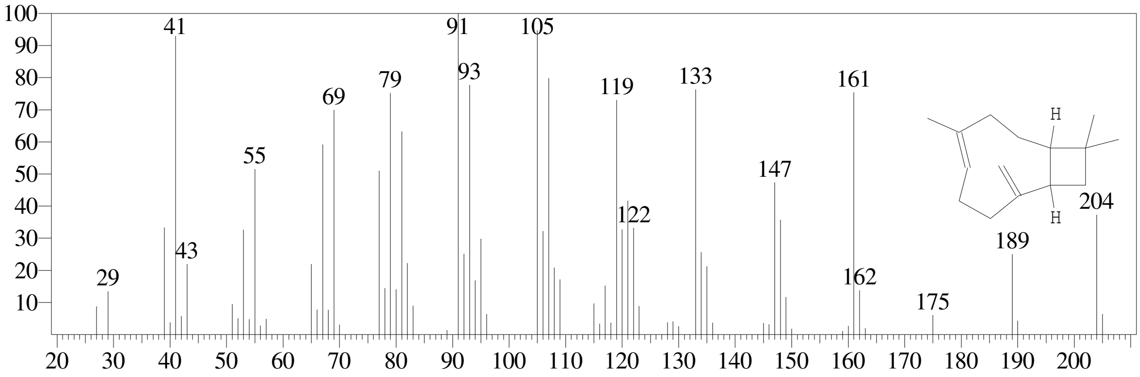

<< Target >>
Line#:9   R.Time:26.950(Scan#:2935) MassPeaks:50
RawMode:Averaged 26.942-26.958(2934-2936) BasePeak:41.00(19624)
BG Mode:Calc. from Peak Group 1 - Event 1 Scan

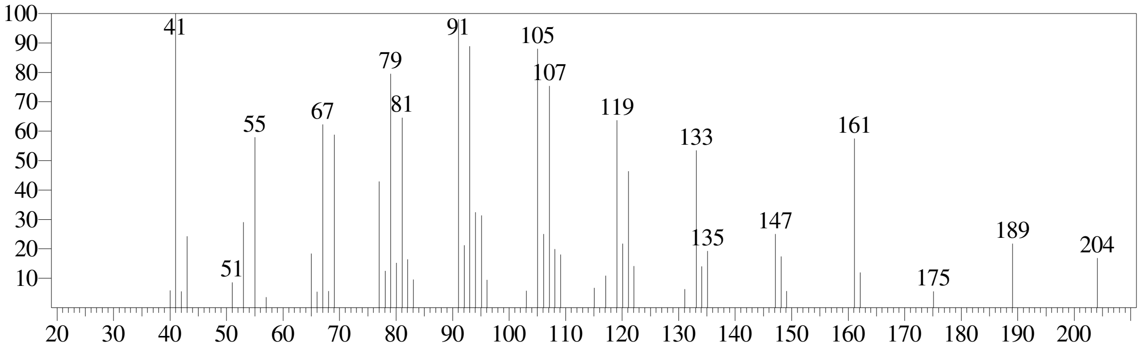

Hit#:3 Entry:24844 Library:NIST23s.lib
SI:94 Formula:C15H24 CAS:489-39-4 MolWeight:204 RetIndex:1424
CompName:Aromandendrene $$ 1H-Cycloprop[e]azulene, decahydro-1,1,7-trimethyl-4-methylene-, [1aR-(1a.alpha.,4a.al

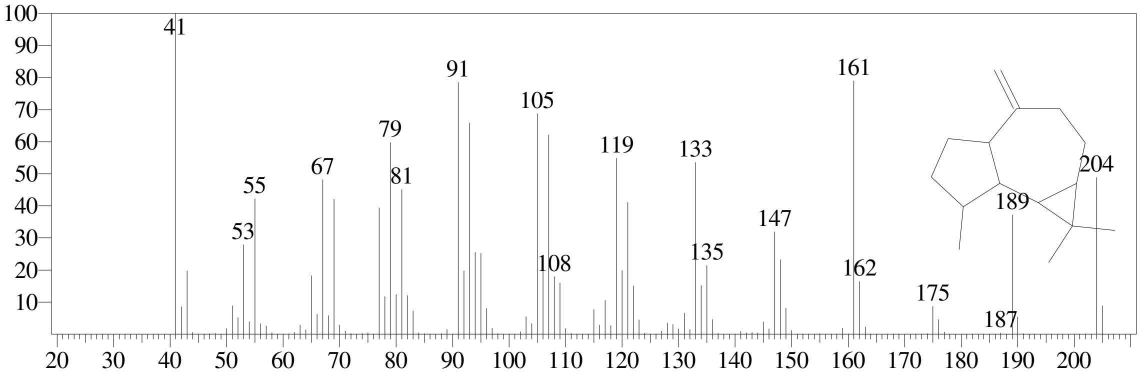

Hit#:4 Entry:24868 Library:NIST23s.lib
SI:94 Formula:C15H24 CAS:25246-27-9 MolWeight:204 RetIndex:1424
CompName:Alloaromadendrene $$ 1H-Cycloprop[e]azulene, decahydro-1,1,7-trimethyl-4-methylene-, [1aR-(1a.alpha.,4a

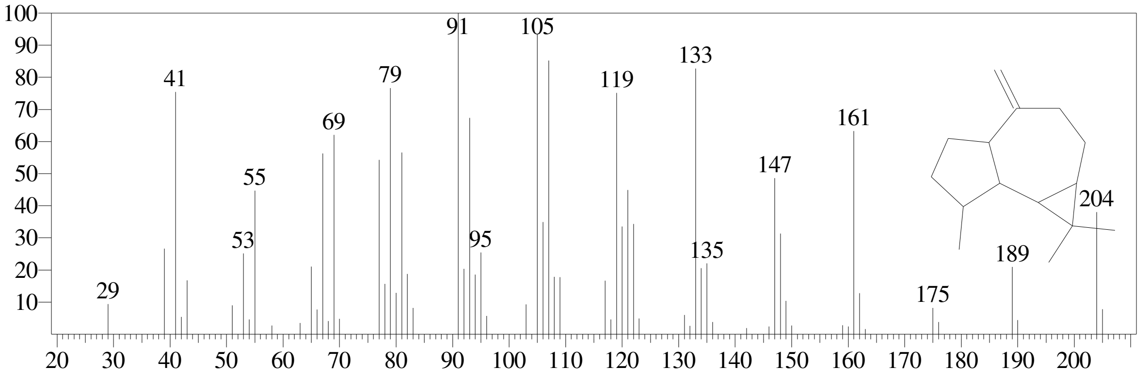

<< Target >>
Line#:9 R.Time:26.950(Scan#:2935) MassPeaks:50
RawMode:Averaged 26.942-26.958(2934-2936) BasePeak:41.00(19624)
BG Mode:Calc. from Peak Group 1 - Event 1 Scan

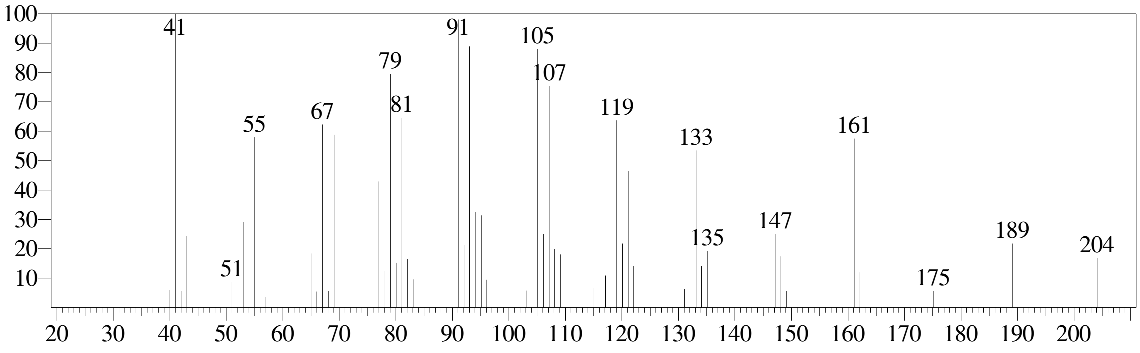

Hit#:5 Entry:62764 Library:NIST23-1.lib
SI:94 Formula:C15H24 CAS:489-39-4 MolWeight:204 RetIndex:1424
CompName:Aromandendrene $$ 1H-Cycloprop[e]azulene, decahydro-1,1,7-trimethyl-4-methylene-, [1aR-(1a.alpha.,4a.al

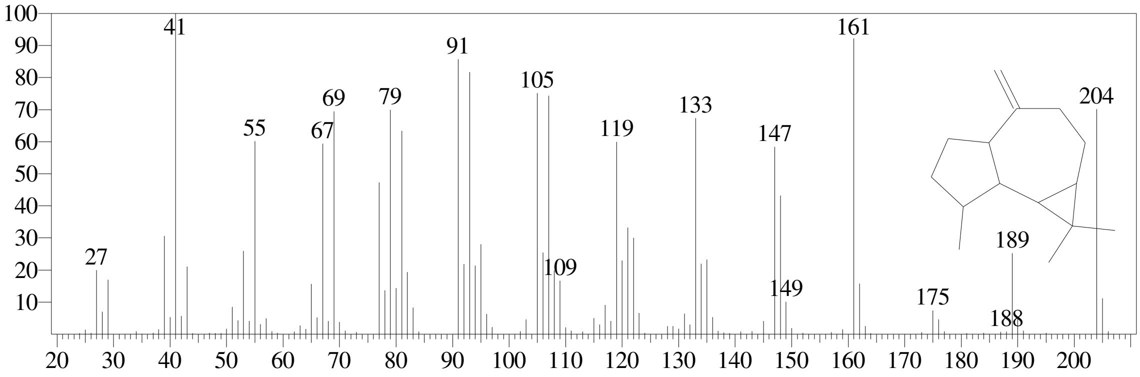

<< Target >>
Line#:10 R.Time:27.542(Scan#:3006) MassPeaks:38
RawMode:Averaged 27.533-27.550(3005-3007) BasePeak:93.05(49972)
BG Mode:Calc. from Peak Group 1 - Event 1 Scan

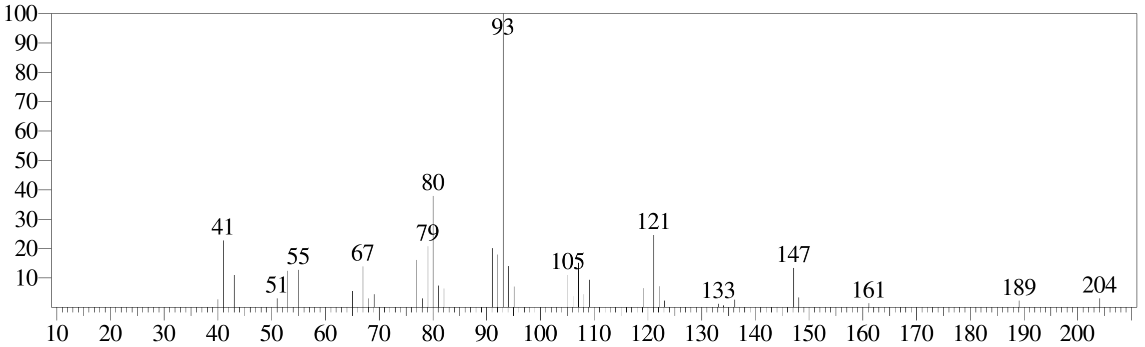

Hit#:1 Entry:24887 Library:NIST23s.lib
SI:95 Formula:C15H24 CAS:6753-98-6 MolWeight:204 RetIndex:1455
CompName:Humulene $$ .alpha.-Caryophyllene $$ 1,4,8-Cycloundecatriene, 2,6,6,9-tetramethyl-, (E,E,E)- $$ .alpha.-Hu

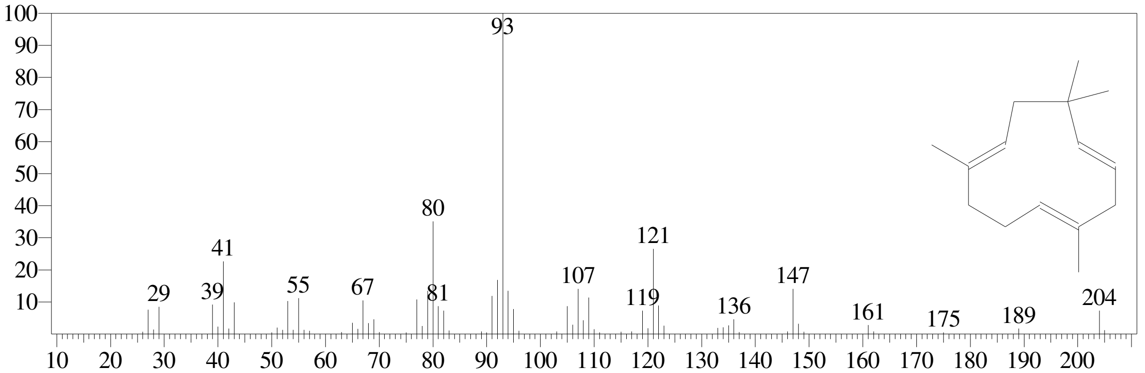

Hit#:2 Entry:24888 Library:NIST23s.lib
SI:94 Formula:C15H24 CAS:6753-98-6 MolWeight:204 RetIndex:1455
CompName:Humulene $$ .alpha.-Caryophyllene $$ 1,4,8-Cycloundecatriene, 2,6,6,9-tetramethyl-, (E,E,E)- $$ .alpha.-Hu

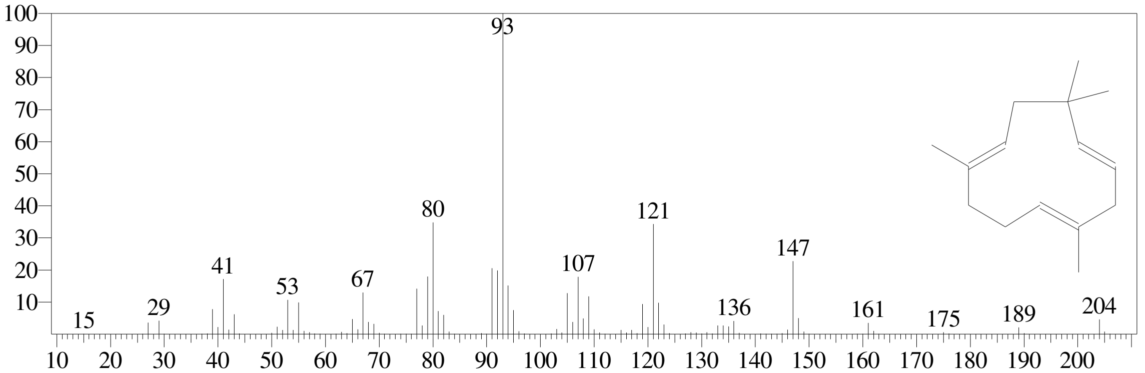

<< Target >>
Line#:10   R.Time:27.542(Scan#:3006) MassPeaks:38
RawMode:Averaged 27.533-27.550(3005-3007) BasePeak:93.05(49972)
BG Mode:Calc. from Peak Group 1 - Event 1 Scan

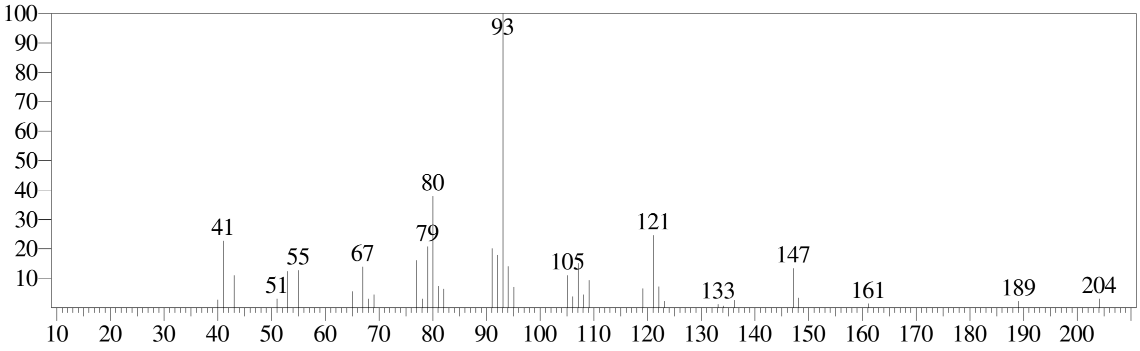

Hit#:3 Entry:62814 Library:NIST23-1.lib
SI:94 Formula:C15H24 CAS:6753-98-6 MolWeight:204 RetIndex:1455
CompName:Humulene $$ .alpha.-Caryophyllene $$ 1,4,8-Cycloundecatriene, 2,6,6,9-tetramethyl-, (E,E,E)- $$ .alpha.-Hu

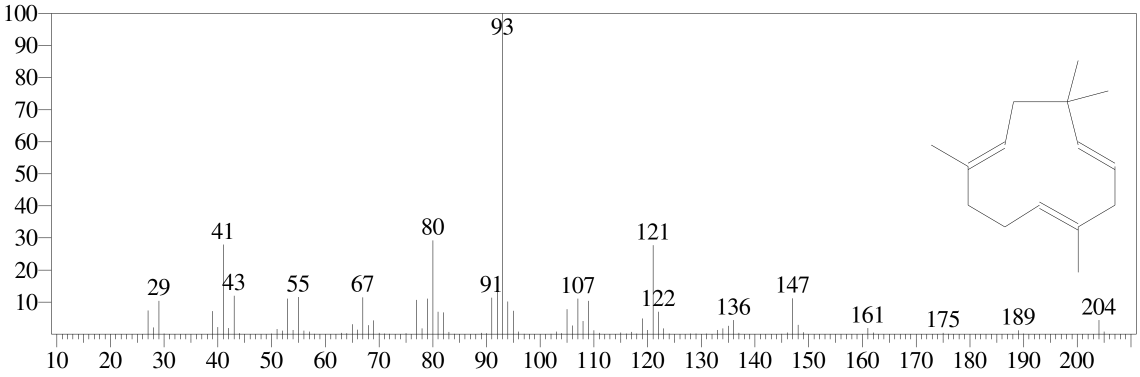

Hit#:4 Entry:24886 Library:NIST23s.lib
SI:94 Formula:C15H24 CAS:6753-98-6 MolWeight:204 RetIndex:1455
CompName:Humulene $$ .alpha.-Caryophyllene $$ 1,4,8-Cycloundecatriene, 2,6,6,9-tetramethyl-, (E,E,E)- $$ .alpha.-Hu

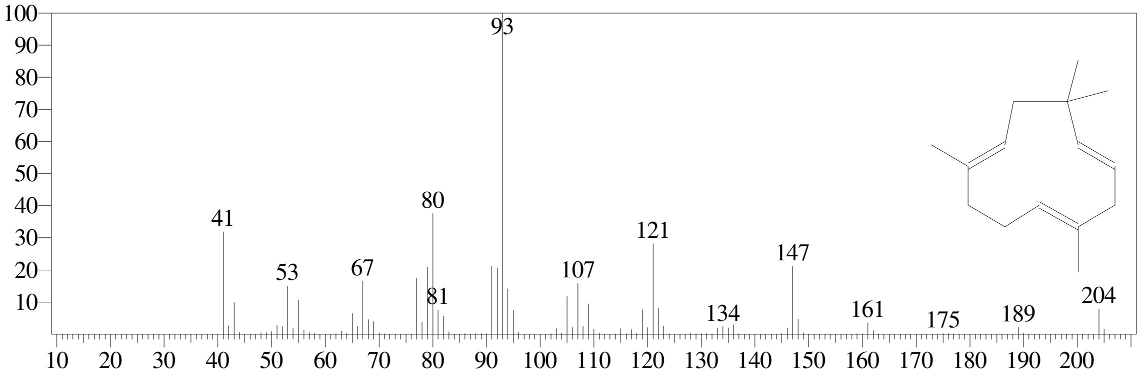

<< Target >>
Line#:10 R.Time:27.542(Scan#:3006) MassPeaks:38
RawMode:Averaged 27.533-27.550(3005-3007) BasePeak:93.05(49972)
BG Mode:Calc. from Peak Group 1 - Event 1 Scan

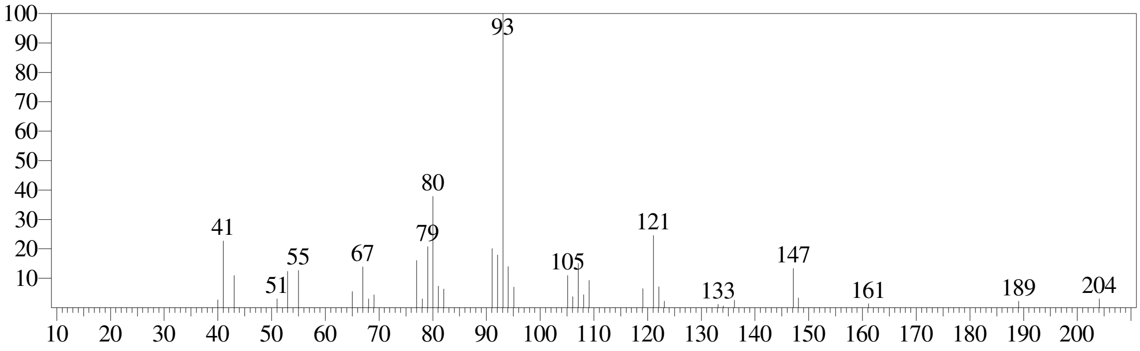

Hit#:5 Entry:62816 Library:NIST23-1.lib
SI:92 Formula:C15H24 CAS:0-00-0 MolWeight:204 RetIndex:1463
CompName:1,4,7,-Cycloundecatriene, 1,5,9,9-tetramethyl-, Z,Z,Z- $$ 1,5,9,9-Tetramethyl-1,4,7-cycloundecatriene # $$

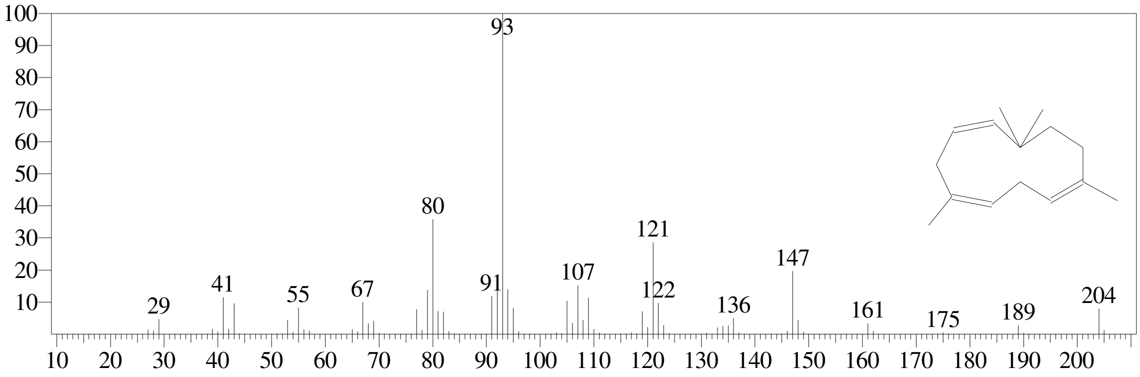

<< Target >>
Line#:11 R.Time:27.850(Scan#:3043) MassPeaks:38
RawMode:Averaged 27.842-27.858(3042-3044) BasePeak:91.05(10385)
BG Mode:Calc. from Peak Group 1 - Event 1 Scan

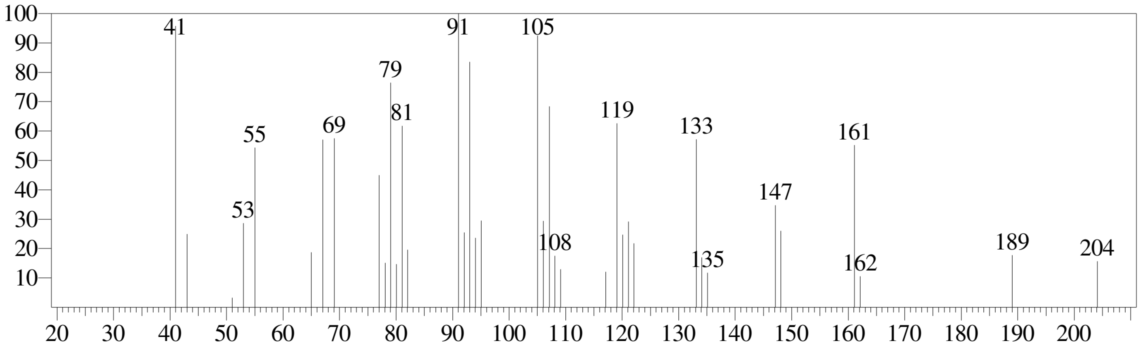

Hit#:1 Entry:62794 Library:NIST23-1.lib
SI:94 Formula:C15H24 CAS:68832-35-9 MolWeight:204 RetIndex:1450
CompName:(1R,9R,E)-4,11,11-Trimethyl-8-methylenebicyclo[7.2.0]undec-4-ene $$ Bicyclo[7.2.0]undec-4-ene, 4,11,11-t

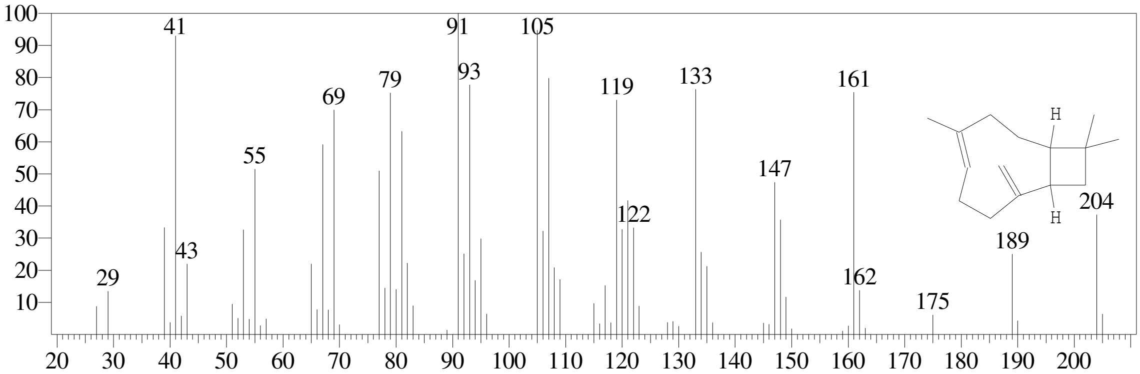

Hit#:2 Entry:62764 Library:NIST23-1.lib
SI:93 Formula:C15H24 CAS:489-39-4 MolWeight:204 RetIndex:1424
CompName:Aromandendrene $$ 1H-Cycloprop[e]azulene, decahydro-1,1,7-trimethyl-4-methylene-, [1aR-(1a.alpha.,4a.al

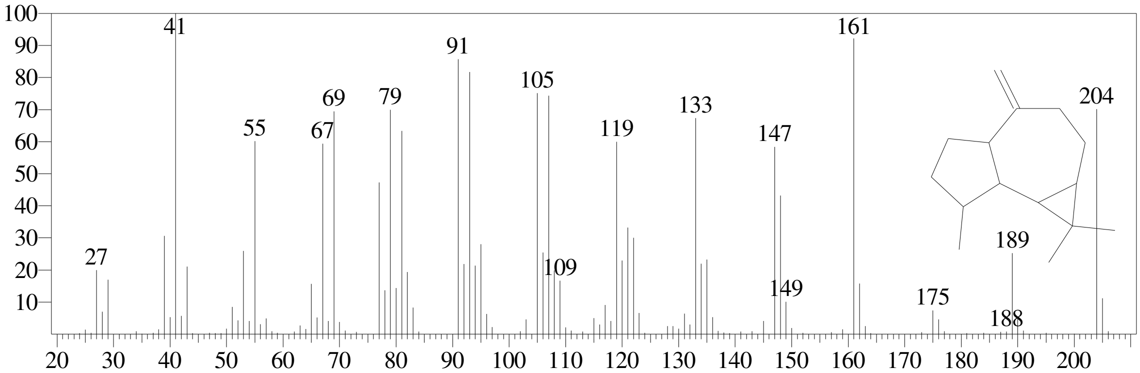

<< Target >>
Line#:11 R.Time:27.850(Scan#:3043) MassPeaks:38
RawMode:Averaged 27.842-27.858(3042-3044) BasePeak:91.05(10385)
BG Mode:Calc. from Peak Group 1 - Event 1 Scan

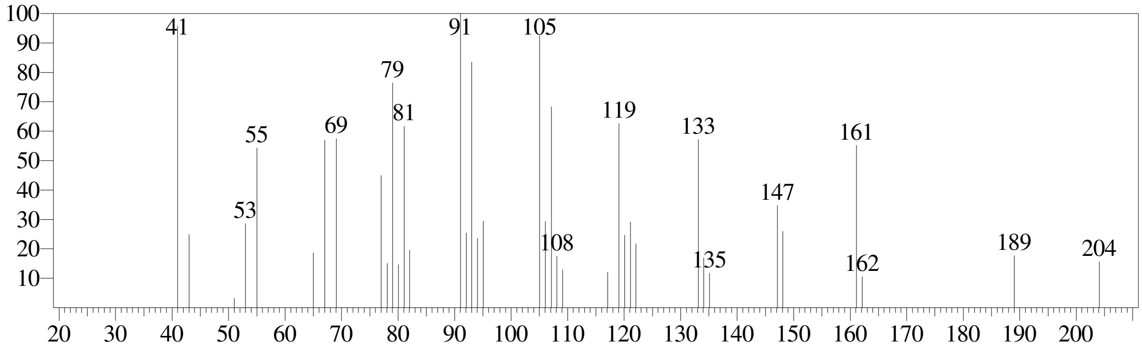

Hit#:3 Entry:24868 Library:NIST23s.lib
SI:93 Formula:C15H24 CAS:25246-27-9 MolWeight:204 RetIndex:1424
CompName:Alloaromadendrene $$ 1H-Cycloprop[e]azulene, decahydro-1,1,7-trimethyl-4-methylene-, [1aR-(1a.alpha.,4a

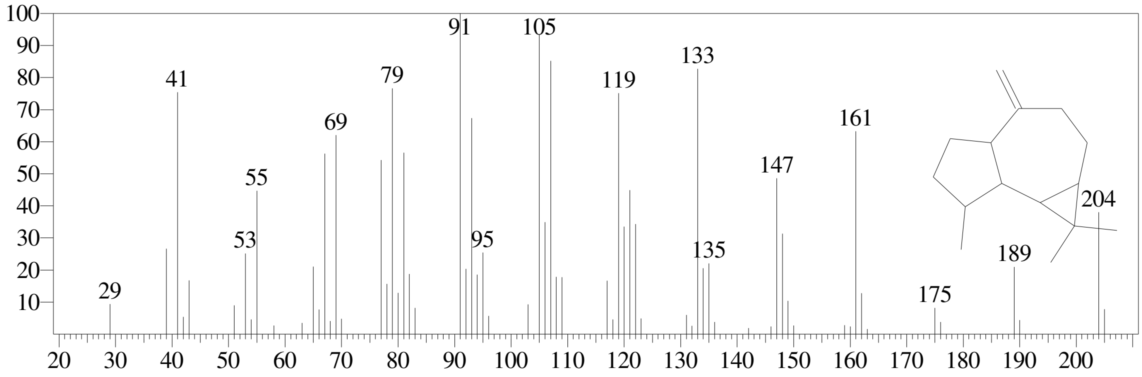

Hit#:4 Entry:24930 Library:NIST23s.lib
SI:93 Formula:C15H24 CAS:25246-27-9 MolWeight:204 RetIndex:1424
CompName:Alloaromadendrene $$ 1H-Cycloprop[e]azulene, decahydro-1,1,7-trimethyl-4-methylene-, [1aR-(1a.alpha.,4a

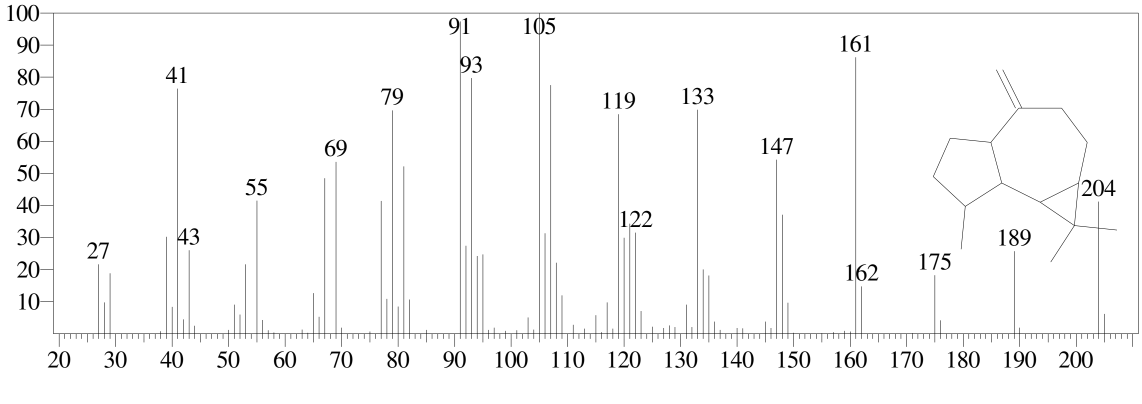

<< Target >>
Line#:11   R.Time:27.850(Scan#:3043) MassPeaks:38
RawMode:Averaged 27.842-27.858(3042-3044) BasePeak:91.05(10385)
BG Mode:Calc. from Peak Group 1 - Event 1 Scan

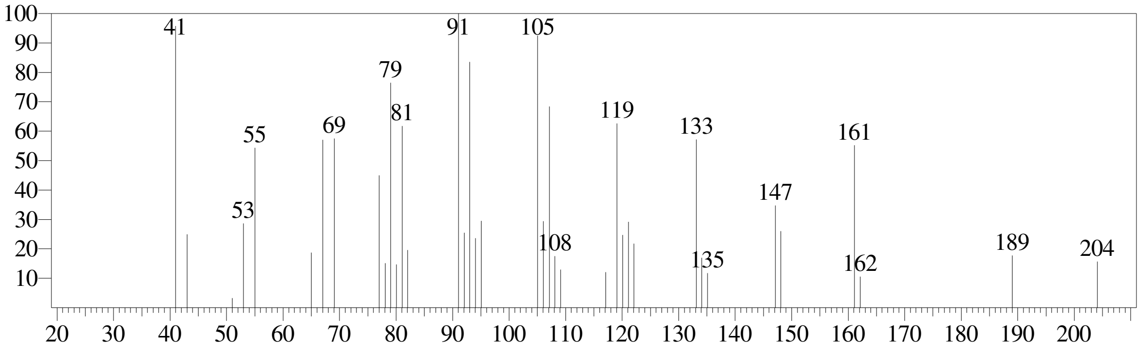

Hit#:5 Entry:62966 Library:NIST23-1.lib
SI:93 Formula:C15H24 CAS:25246-27-9 MolWeight:204 RetIndex:1424
CompName:Alloaromadendrene $$ 1H-Cycloprop[e]azulene, decahydro-1,1,7-trimethyl-4-methylene-, [1aR-(1a.alpha.,4a

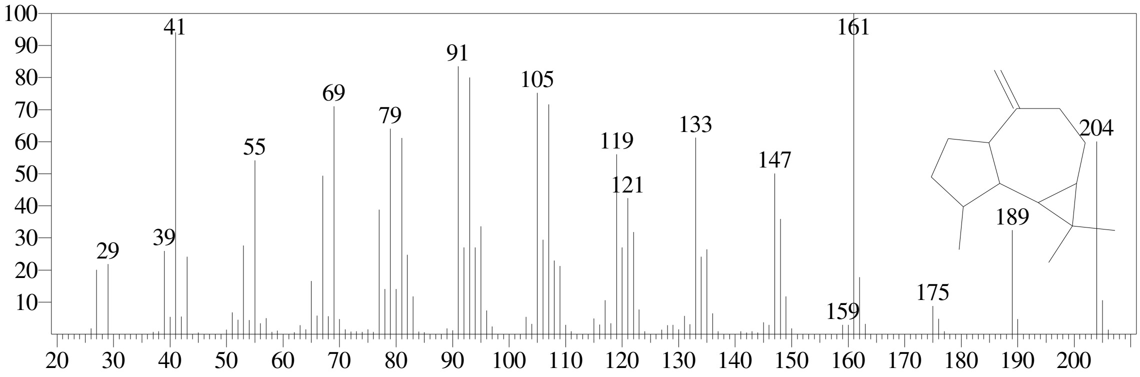

<< Target >>
Line#:12 R.Time:28.492(Scan#:3120) MassPeaks:38
RawMode:Averaged 28.483-28.500(3119-3121) BasePeak:161.10(14704)
BG Mode:Calc. from Peak Group 1 - Event 1 Scan

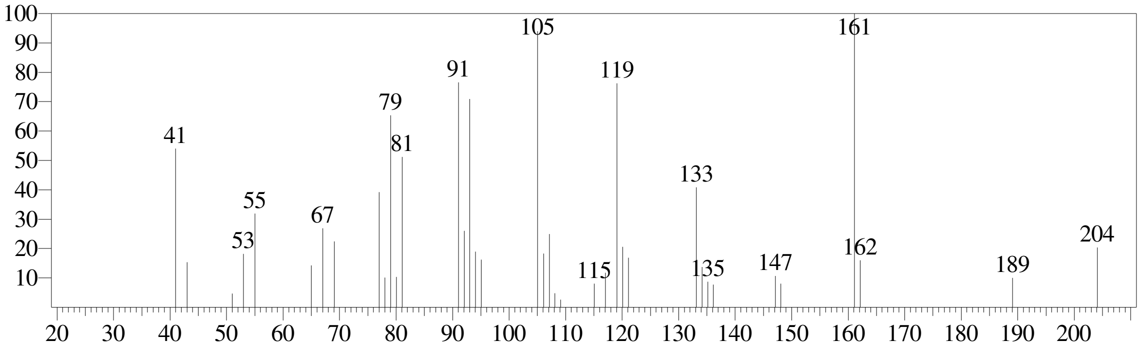

Hit#:1 Entry:25071 Library:NIST23s.lib
SI:93 Formula:C15H24 CAS:483-75-0 MolWeight:204 RetIndex:1500
CompName:Naphthalene, 1,2,4a,5,6,8a-hexahydro-4,7-dimethyl-1-(1-methylethyl)- $$ 1-Isopropyl-4,7-dimethyl-1,2,4a,5,

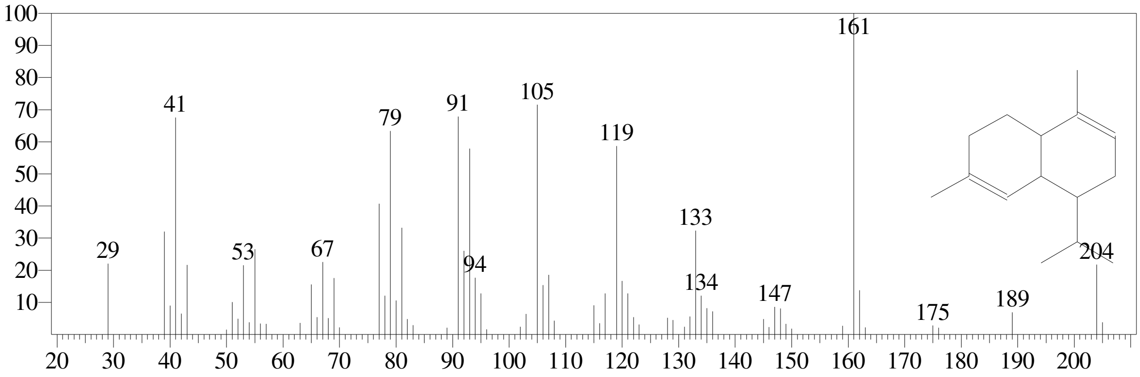

Hit#:2 Entry:62975 Library:NIST23-1.lib
SI:93 Formula:C15H24 CAS:317819-80-0 MolWeight:204 RetIndex:1478
CompName:(S,1Z,6Z)-8-Isopropyl-1-methyl-5-methylenecyclodeca-1,6-diene $$ 1,6-Cyclodecadiene, 1-methyl-5-methyl

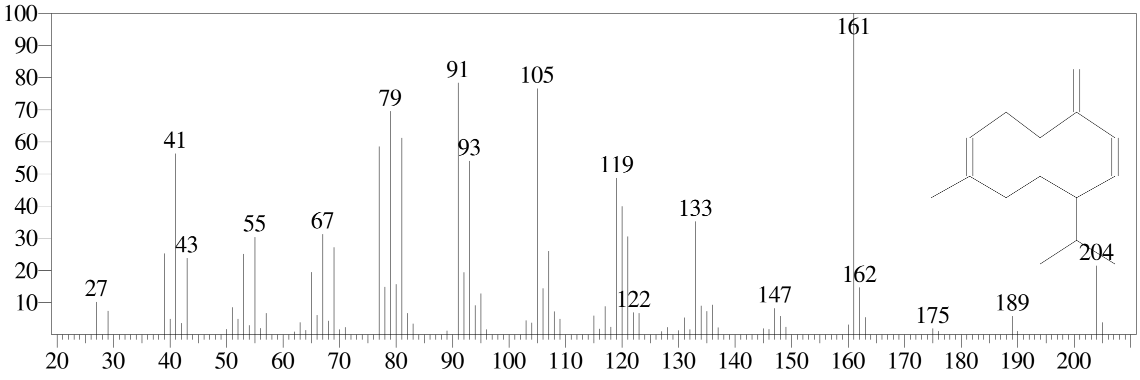

<< Target >>
Line#:12   R.Time:28.492(Scan#:3120) MassPeaks:38
RawMode:Averaged 28.483-28.500(3119-3121) BasePeak:161.10(14704)
BG Mode:Calc. from Peak Group 1 - Event 1 Scan

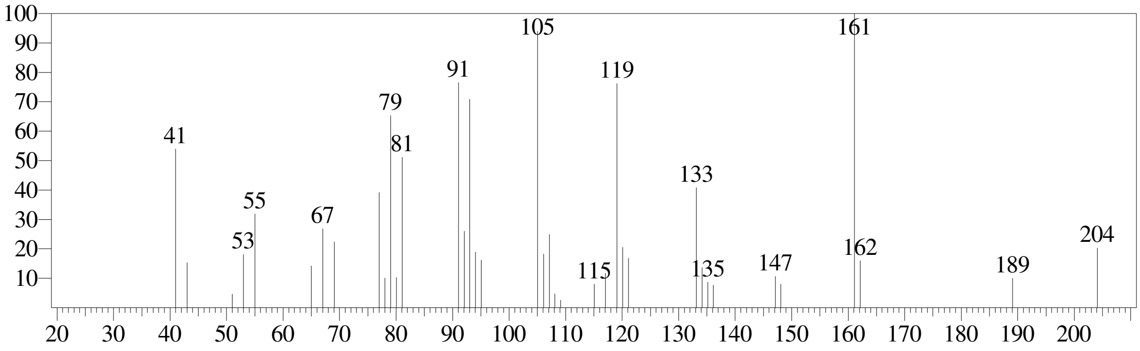

Hit#:3 Entry:25048 Library:NIST23s.lib
SI:92 Formula:C15H24 CAS:6980-46-7 MolWeight:204 RetIndex:1483
CompName:(1S,4aR,8aS)-1-Isopropyl-7-methyl-4-methylene-1,2,3,4,4a,5,6,8a-octahydronaphthalene $$ .gamma.-Amorp

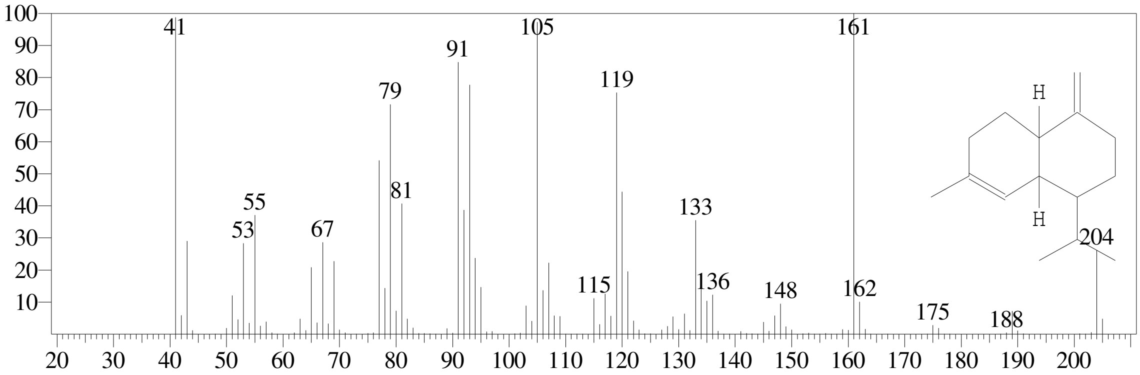

Hit#:4 Entry:25047 Library:NIST23s.lib
SI:92 Formula:C15H24 CAS:30021-74-0 MolWeight:204 RetIndex:1483
CompName:.gamma.-Muurolene $$ Naphthalene, 1,2,3,4,4a,5,6,8a-octahydro-7-methyl-4-methylene-1-(1-methylethyl)-, (

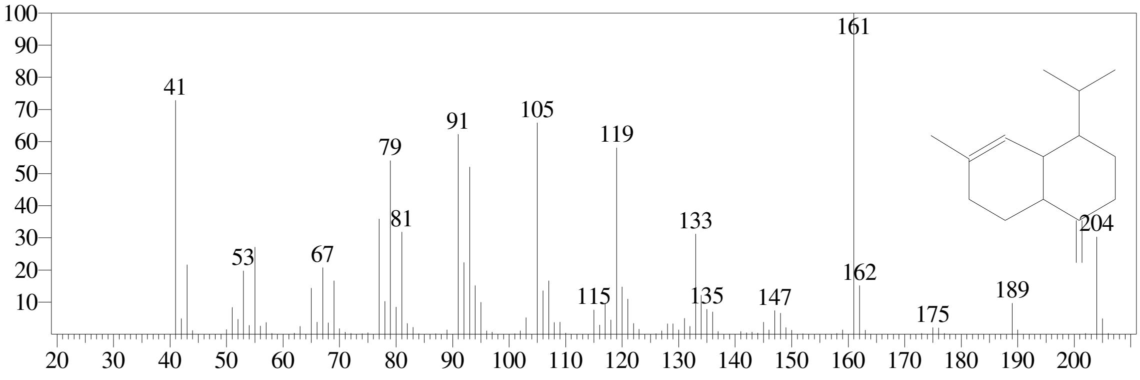

<< Target >>
Line#:12 R.Time:28.492(Scan#:3120) MassPeaks:38
RawMode:Averaged 28.483-28.500(3119-3121) BasePeak:161.10(14704)
BG Mode:Calc. from Peak Group 1 - Event 1 Scan

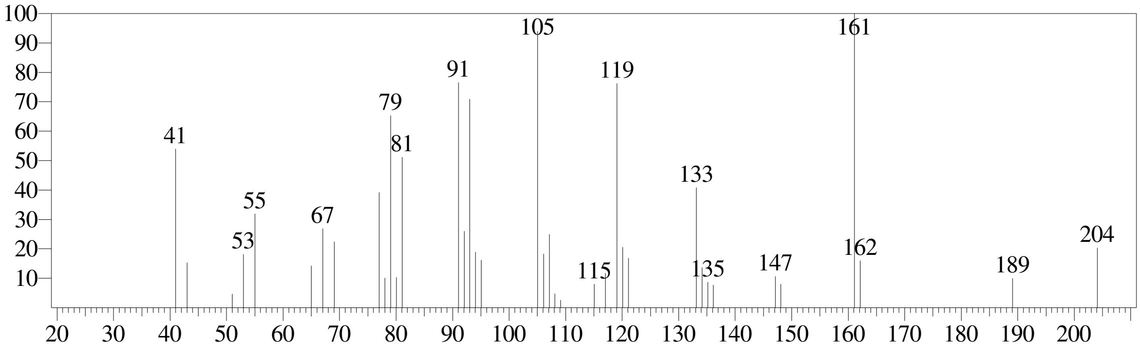

Hit#:5 Entry:62974 Library:NIST23-1.lib
SI:91 Formula:C15H24 CAS:24741-64-8 MolWeight:204 RetIndex:1496
CompName:(3R,4aS,5R)-4a,5-Dimethyl-3-(prop-1-en-2-yl)-1,2,3,4,4a,5,6,7-octahydronaphthalene $$ Naphthalene, 1,2,3,[illegible]

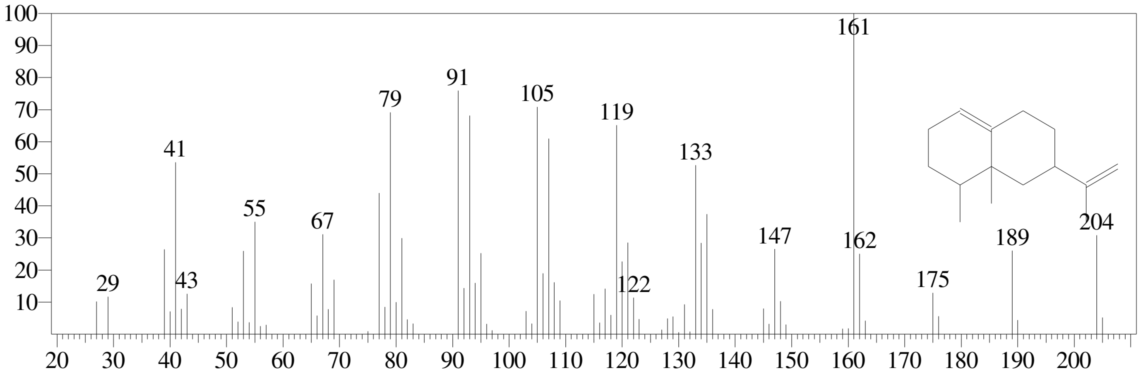

<< Target >>
Line#:13 R.Time:28.883(Scan#:3167) MassPeaks:56
RawMode:Averaged 28.875-28.892(3166-3168) BasePeak:93.05(18173)
BG Mode:Calc. from Peak Group 1 - Event 1 Scan

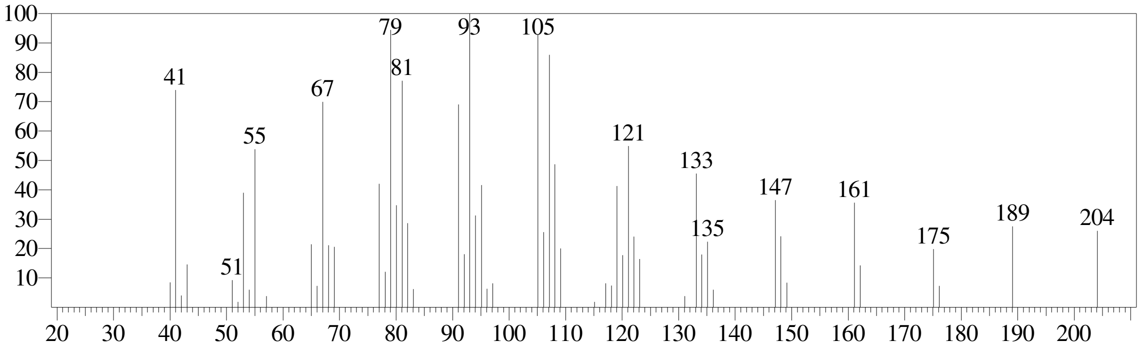

Hit#:1 Entry:24936 Library:NIST23s.lib
SI:96 Formula:C15H24 CAS:17066-67-0 MolWeight:204 RetIndex:1489
CompName:Naphthalene, decahydro-4a-methyl-1-methylene-7-(1-methylethenyl)-, [4aR-(4a.alpha.,7.alpha.,8a.beta.)]- $$

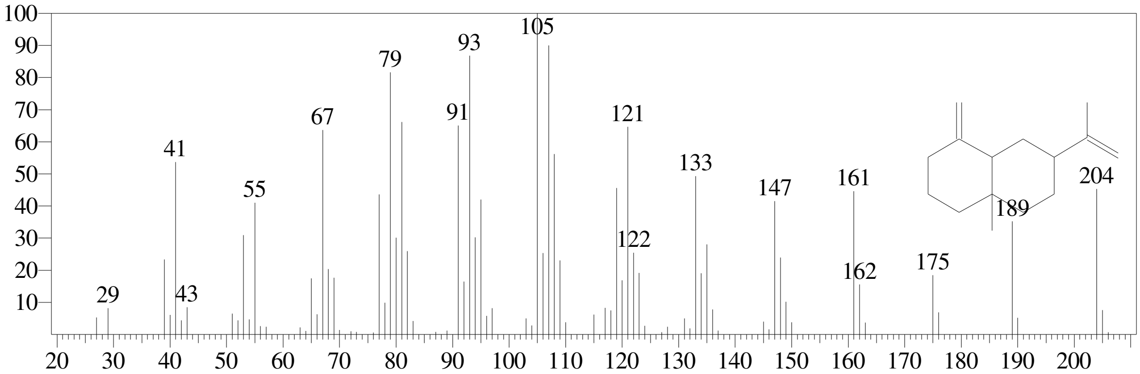

Hit#:2 Entry:24892 Library:NIST23s.lib
SI:95 Formula:C15H24 CAS:17066-67-0 MolWeight:204 RetIndex:1489
CompName:Naphthalene, decahydro-4a-methyl-1-methylene-7-(1-methylethenyl)-, [4aR-(4a.alpha.,7.alpha.,8a.beta.)]- $$

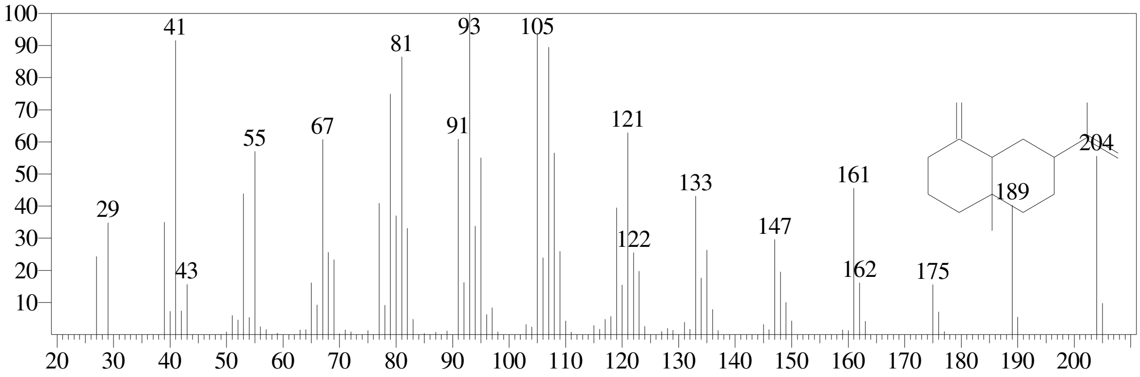

<< Target >>
Line#:13 R.Time:28.883(Scan#:3167) MassPeaks:56
RawMode:Averaged 28.875-28.892(3166-3168) BasePeak:93.05(18173)
BG Mode:Calc. from Peak Group 1 - Event 1 Scan

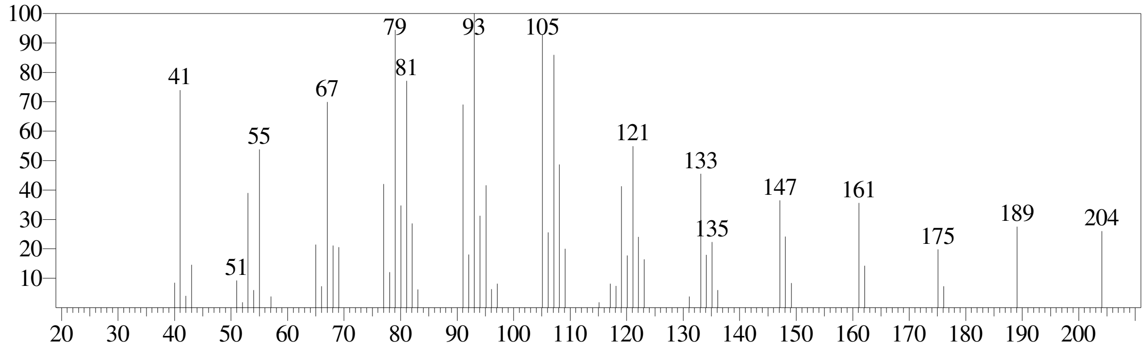

Hit#:3 Entry:62812 Library:NIST23-1.lib
SI:94 Formula:C15H24 CAS:0-00-0 MolWeight:204 RetIndex:1475
CompName:Bicyclo[5.3.0]decane, 2-methylene-5-(1-methylvinyl)-8-methyl- $$ 7-Isopropenyl-1-methyl-4-methylenedeca

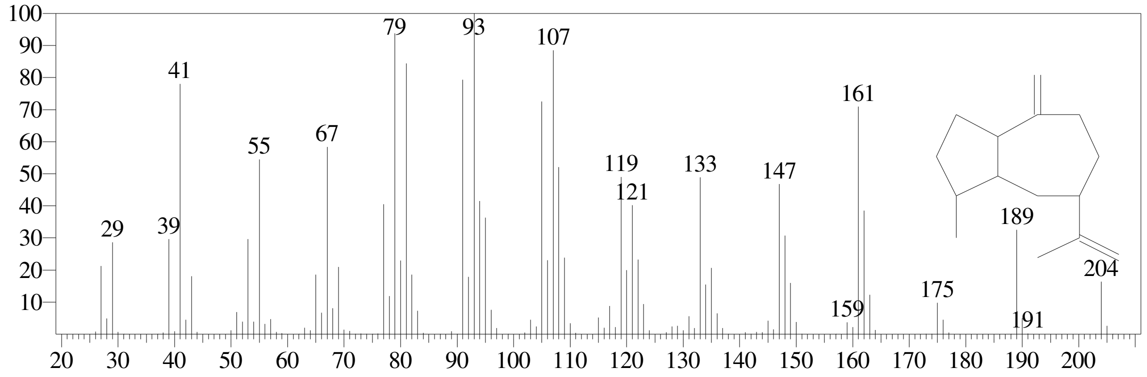

Hit#:4 Entry:62758 Library:NIST23-1.lib
SI:93 Formula:C15H24 CAS:17066-67-0 MolWeight:204 RetIndex:1489
CompName:Naphthalene, decahydro-4a-methyl-1-methylene-7-(1-methylethenyl)-, [4aR-(4a.alpha.,7.alpha.,8a.beta.)]- $$

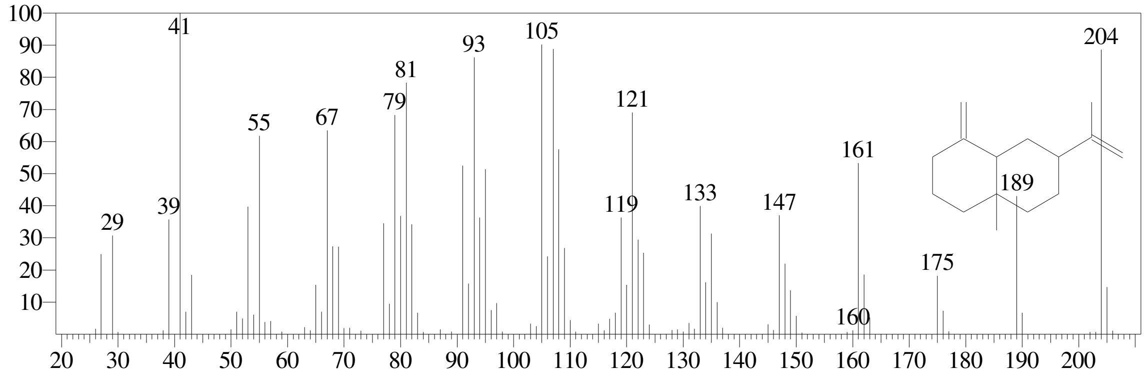

<< Target >>
Line#:13 R.Time:28.883(Scan#:3167) MassPeaks:56
RawMode:Averaged 28.875-28.892(3166-3168) BasePeak:93.05(18173)
BG Mode:Calc. from Peak Group 1 - Event 1 Scan

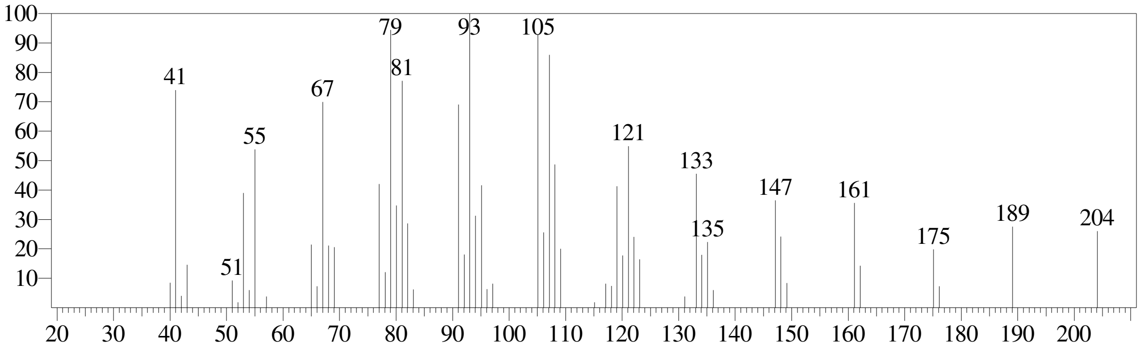

Hit#:5 Entry:24955 Library:NIST23s.lib
SI:93 Formula:C15H24 CAS:17066-67-0 MolWeight:204 RetIndex:1489
CompName:Naphthalene, decahydro-4a-methyl-1-methylene-7-(1-methylethenyl)-, [4aR-(4a.alpha.,7.alpha.,8a.beta.)]- $$

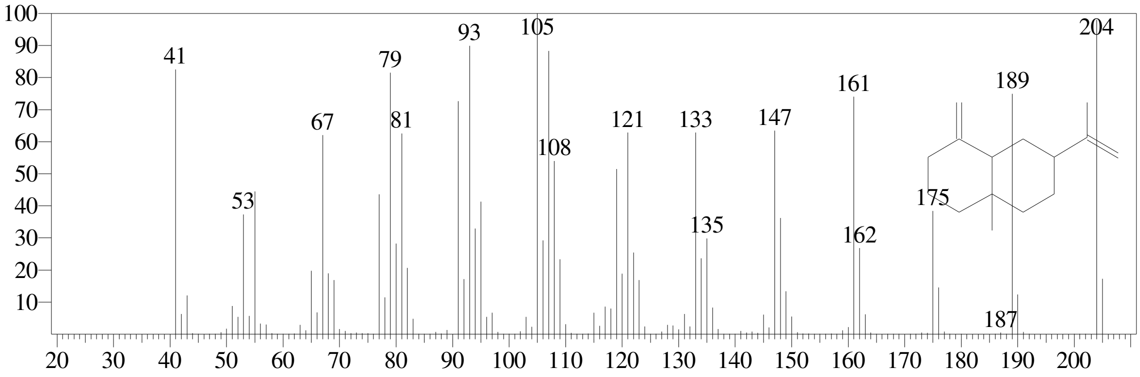

<< Target >>
Line#:14 R.Time:28.983(Scan#:3179) MassPeaks:26
RawMode:Averaged 28.975-28.992(3178-3180) BasePeak:105.05(3189)
BG Mode:Calc. from Peak Group 1 - Event 1 Scan

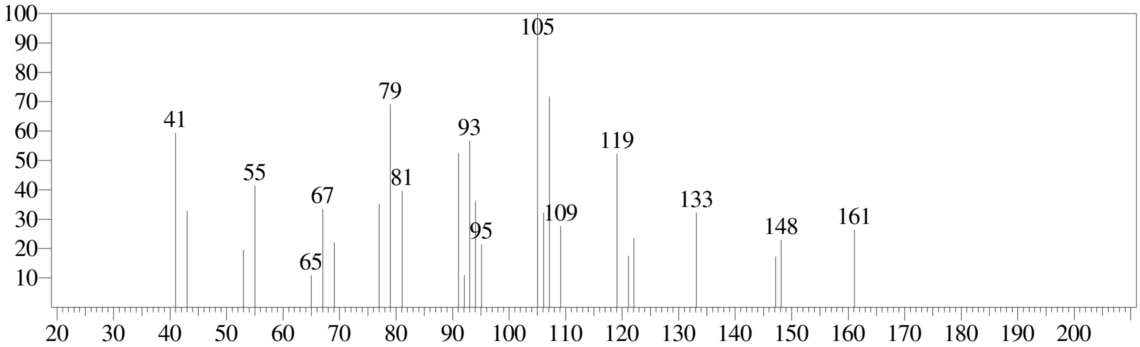

Hit#:1 Entry:62755 Library:NIST23-1.lib
SI:85 Formula:C15H24 CAS:85048-01-7 MolWeight:204 RetIndex:1436
CompName:Aromadendrane,dehydro-

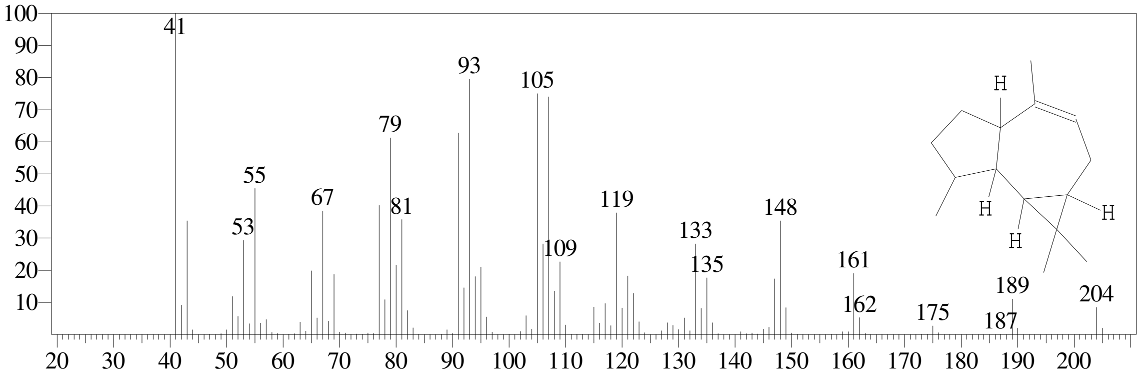

Hit#:2 Entry:62671 Library:NIST23-1.lib
SI:84 Formula:C13H20N2 CAS:0-00-0 MolWeight:204 RetIndex:1704
CompName:1,4-Methanocycloocta[d]pyridazine, 1,4,4a,5,6,9,10,10a-octahydro-11,11-dimethyl-, (1.alpha.,4.alpha.,4a.alpl

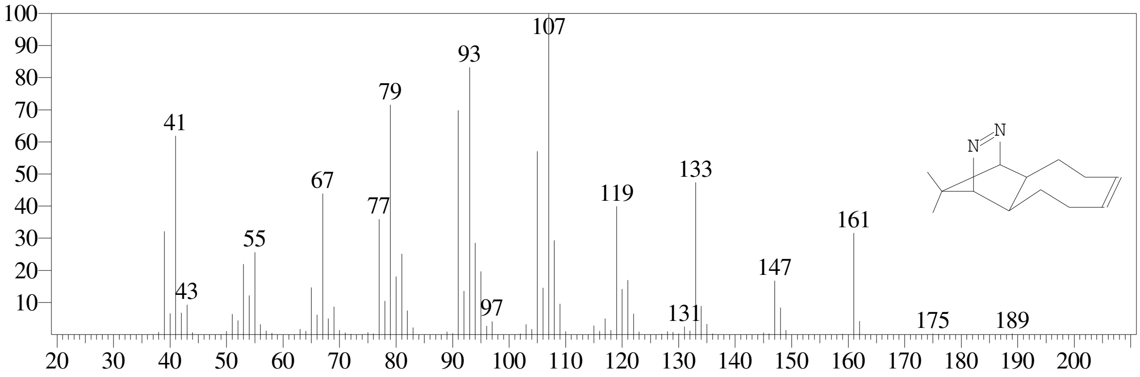

<< Target >>
Line#:14   R.Time:28.983(Scan#:3179) MassPeaks:26
RawMode:Averaged 28.975-28.992(3178-3180) BasePeak:105.05(3189)
BG Mode:Calc. from Peak Group 1 - Event 1 Scan

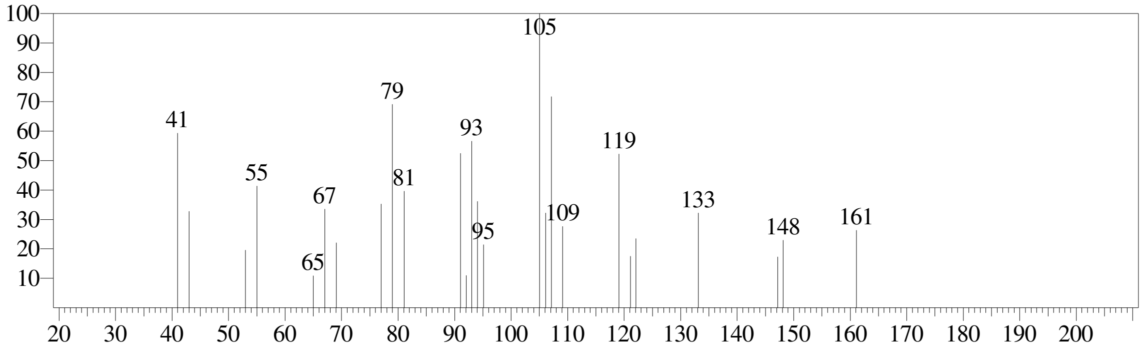

Hit#:3 Entry:24813 Library:NIST23s.lib
SI:83 Formula:C15H24 CAS:489-39-4 MolWeight:204 RetIndex:1424
CompName:Aromandendrene $$ 1H-Cycloprop[e]azulene, decahydro-1,1,7-trimethyl-4-methylene-, [1aR-(1a.alpha.,4a.al

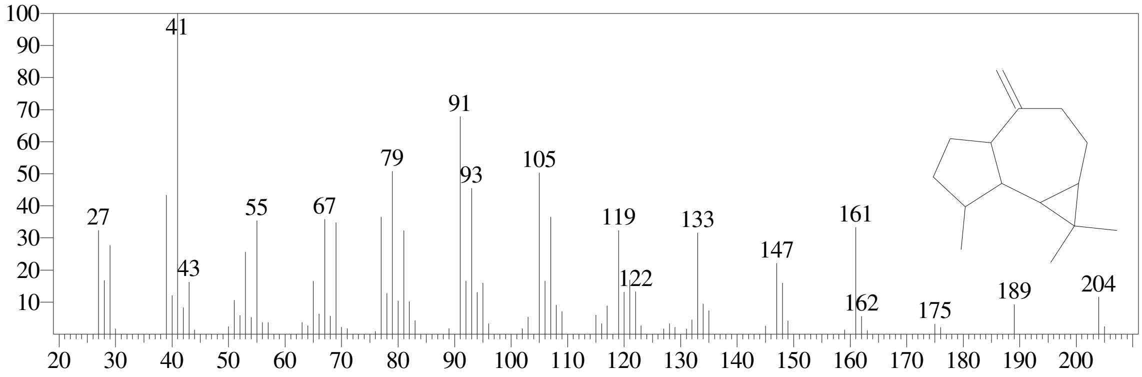

Hit#:4 Entry:62848 Library:NIST23-1.lib
SI:82 Formula:C15H24 CAS:3691-12-1 MolWeight:204 RetIndex:1454
CompName:.alpha.-Guaiene $$ Azulene, 1,2,3,4,5,6,7,8-octahydro-1,4-dimethyl-7-(1-methylethenyl)-, [1S-(1.alpha.,4.alp

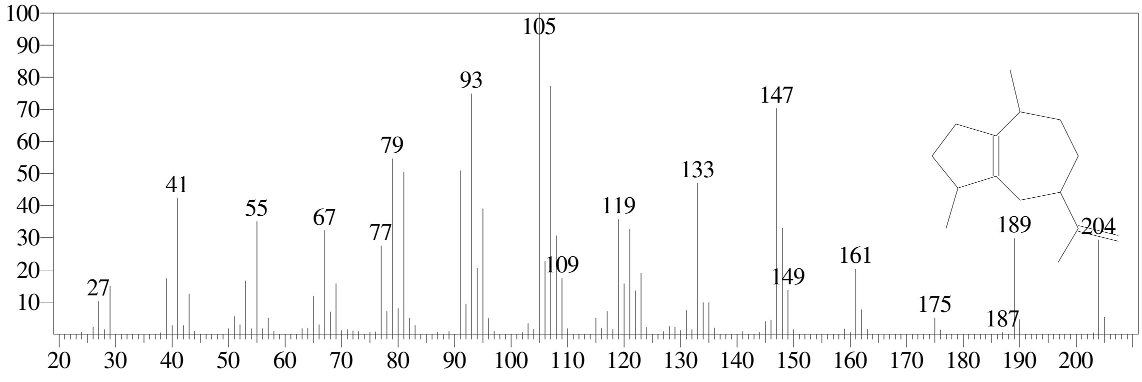

<< Target >>
Line#:14   R.Time:28.983(Scan#:3179) MassPeaks:26
RawMode:Averaged 28.975-28.992(3178-3180) BasePeak:105.05(3189)
BG Mode:Calc. from Peak Group 1 - Event 1 Scan

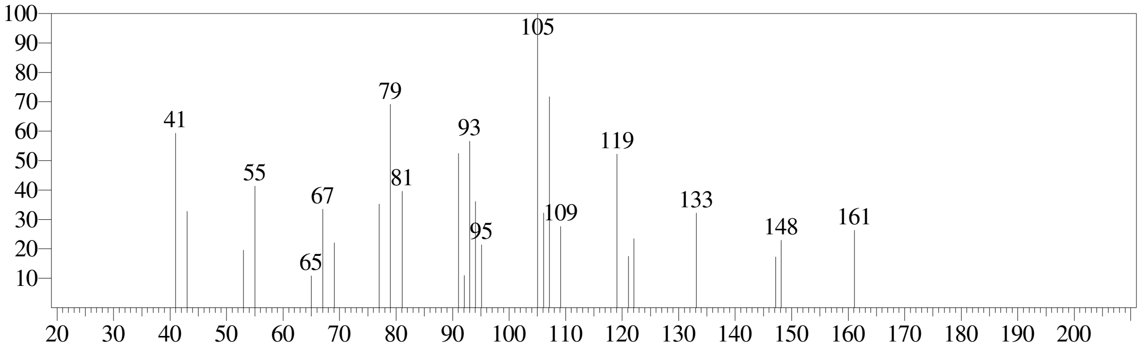

Hit#:5 Entry:62901 Library:NIST23-1.lib
SI:82 Formula:C15H24 CAS:622792-97-6 MolWeight:204 RetIndex:1530
CompName:Bisabolene,(E)-iso-gamma-

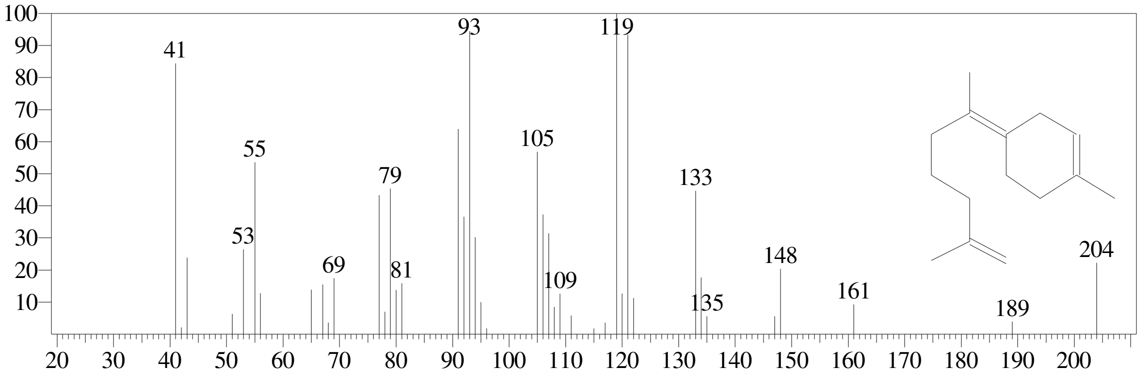

<< Target >>
Line#:15 R.Time:29.092(Scan#:3192) MassPeaks:23
RawMode:Averaged 29.083-29.100(3191-3193) BasePeak:161.10(5681)
BG Mode:Calc. from Peak Group 1 - Event 1 Scan

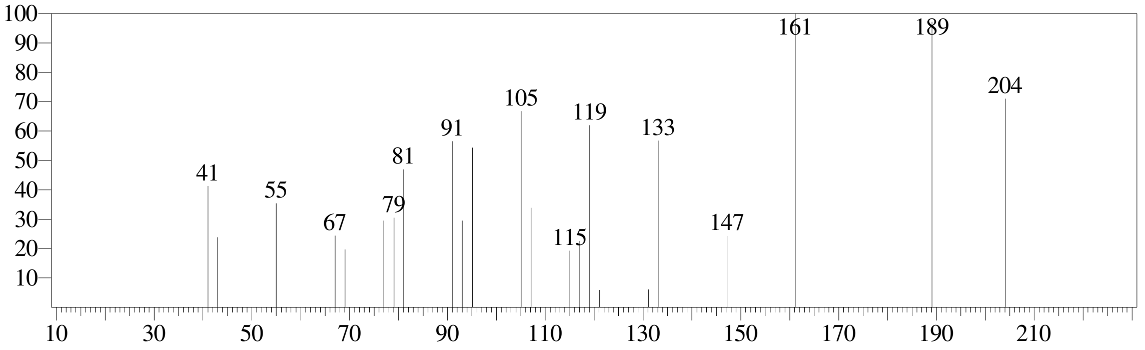

Hit#:1 Entry:83610 Library:NIST23-1.lib
SI:85 Formula:C15H26O CAS:98683-12-6 MolWeight:222 RetIndex:1640
CompName:2-(4a,8-Dimethyl-2,3,4,5,6,7-hexahydro-1H-naphthalen-2-yl)propan-2-ol

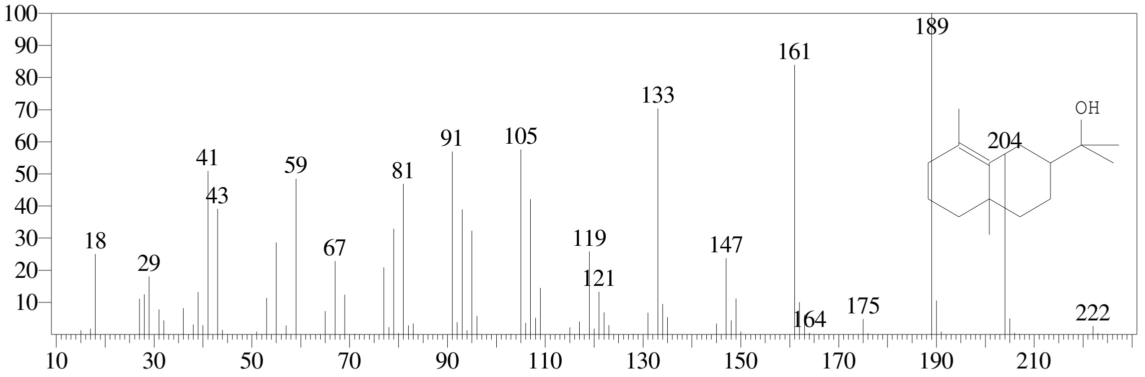

Hit#:2 Entry:25130 Library:NIST23s.lib
SI:84 Formula:C15H24 CAS:473-14-3 MolWeight:204 RetIndex:1509
CompName:Naphthalene, 2,3,4,4a,5,6-hexahydro-1,4a-dimethyl-7-(1-methylethyl)- $$ 6-Isopropyl-4,8a-dimethyl-1,2,3,7,

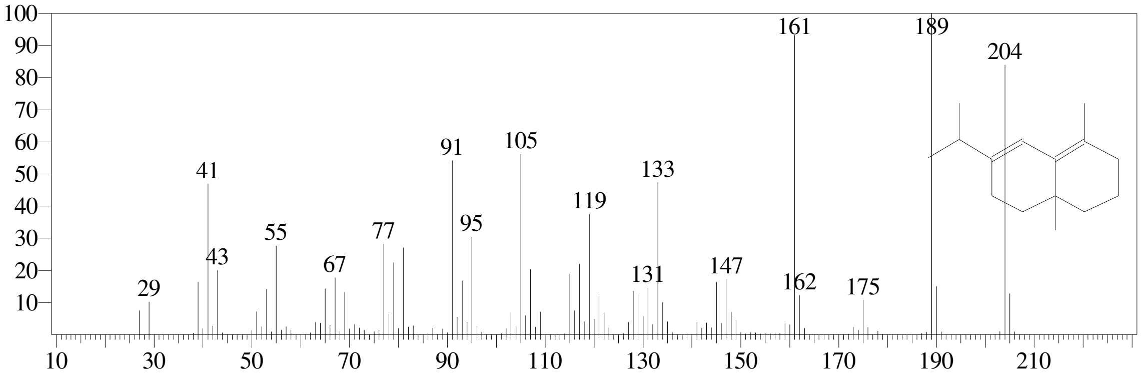

<< Target >>
Line#:15   R.Time:29.092(Scan#:3192) MassPeaks:23
RawMode:Averaged 29.083-29.100(3191-3193) BasePeak:161.10(5681)
BG Mode:Calc. from Peak Group 1 - Event 1 Scan

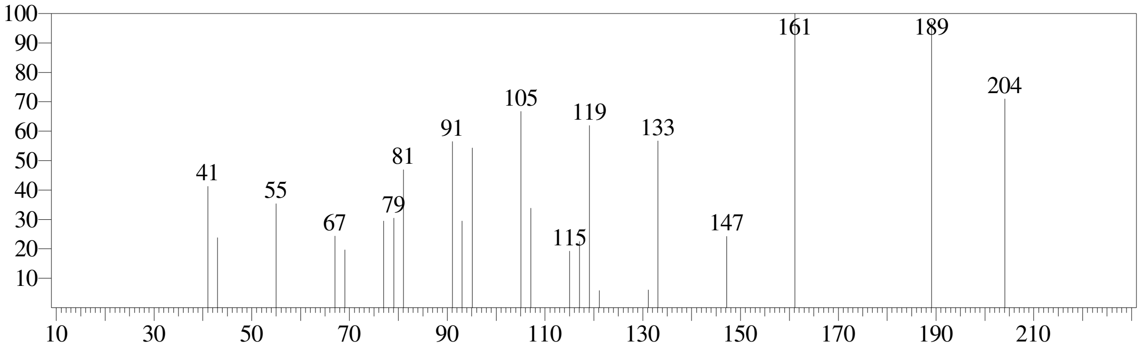

Hit#:3 Entry:63012 Library:NIST23-1.lib
SI:84 Formula:C15H24 CAS:28624-28-4 MolWeight:204 RetIndex:1505
CompName:Selinene, delta-

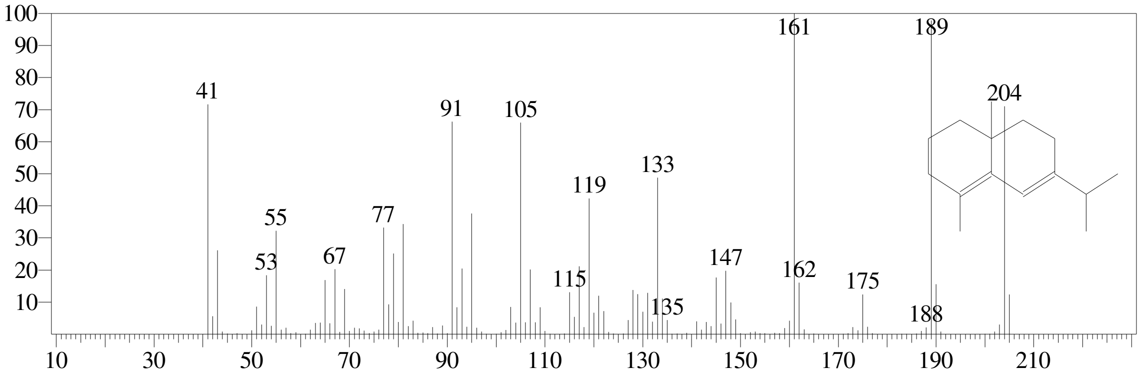

Hit#:4 Entry:63042 Library:NIST23-1.lib
SI:84 Formula:C15H24 CAS:489-29-2 MolWeight:204 RetIndex:1410
CompName:1H-Cyclopropa[a]naphthalene, 1a,2,3,3a,4,5,6,7b-octahydro-1,1,3a,7-tetramethyl-, [1aR-(1a.alpha.,3a.alpha.,7

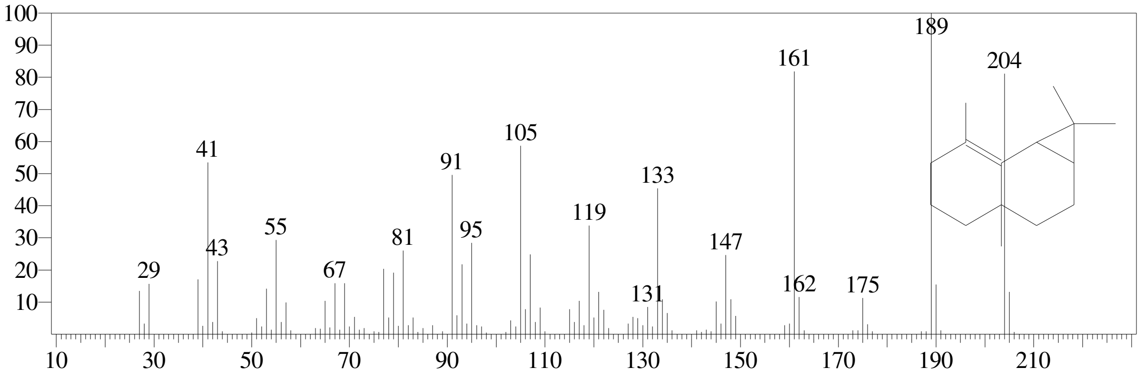

<< Target >>
Line#:15   R.Time:29.092(Scan#:3192) MassPeaks:23
RawMode:Averaged 29.083-29.100(3191-3193) BasePeak:161.10(5681)
BG Mode:Calc. from Peak Group 1 - Event 1 Scan

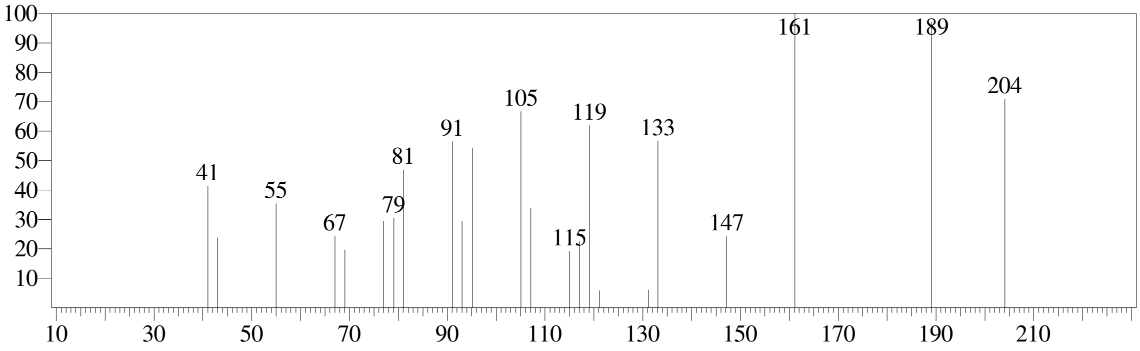

Hit#:5 Entry:62985 Library:NIST23-1.lib
SI:84 Formula:C15H24 CAS:14029-18-6 MolWeight:204 RetIndex:1471
CompName:Sibirene

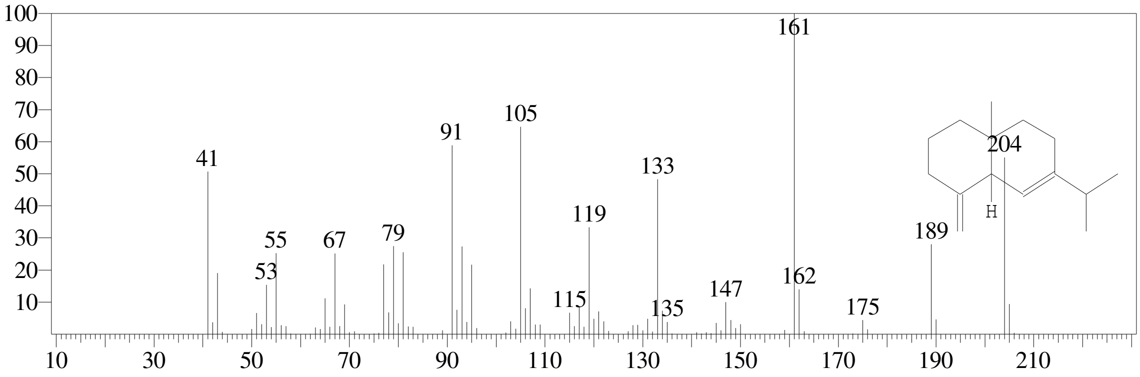

<< Target >>
Line#:16   R.Time:29.250(Scan#:3211) MassPeaks:71
RawMode:Averaged 29.242-29.258(3210-3212) BasePeak:93.05(46881)
BG Mode:Calc. from Peak Group 1 - Event 1 Scan

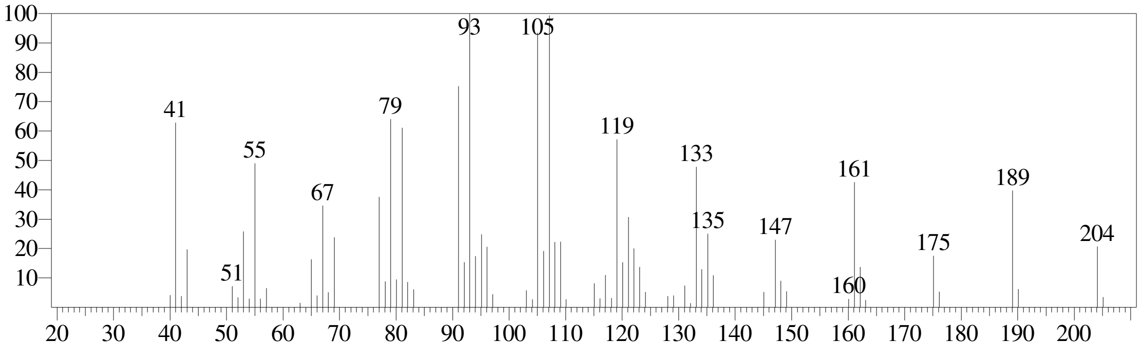

Hit#:1 Entry:24962 Library:NIST23s.lib
SI:94 Formula:C15H24 CAS:21747-46-6 MolWeight:204 RetIndex:1451
CompName:1H-Cycloprop[e]azulene, 1a,2,3,5,6,7,7a,7b-octahydro-1,1,4,7-tetramethyl-, [1aR-(1a.alpha.,7.alpha.,7a.beta.,

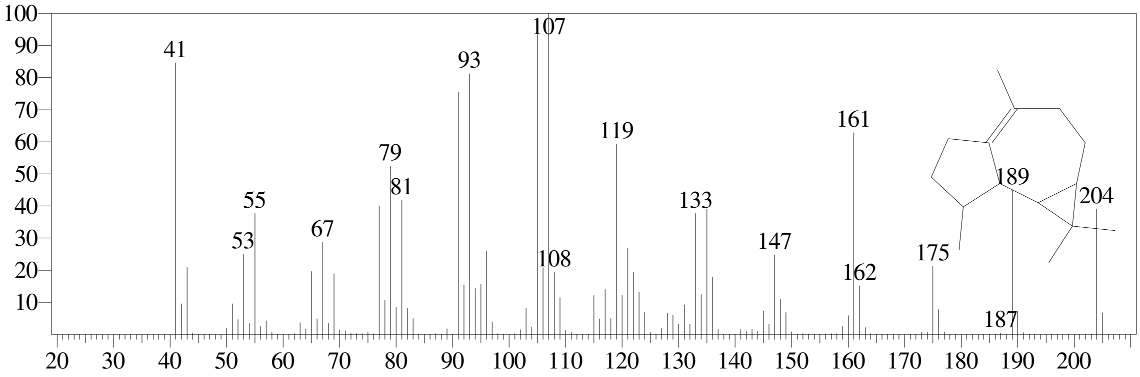

Hit#:2 Entry:24957 Library:NIST23s.lib
SI:93 Formula:C15H24 CAS:21747-46-6 MolWeight:204 RetIndex:1451
CompName:1H-Cycloprop[e]azulene, 1a,2,3,5,6,7,7a,7b-octahydro-1,1,4,7-tetramethyl-, [1aR-(1a.alpha.,7.alpha.,7a.beta.,

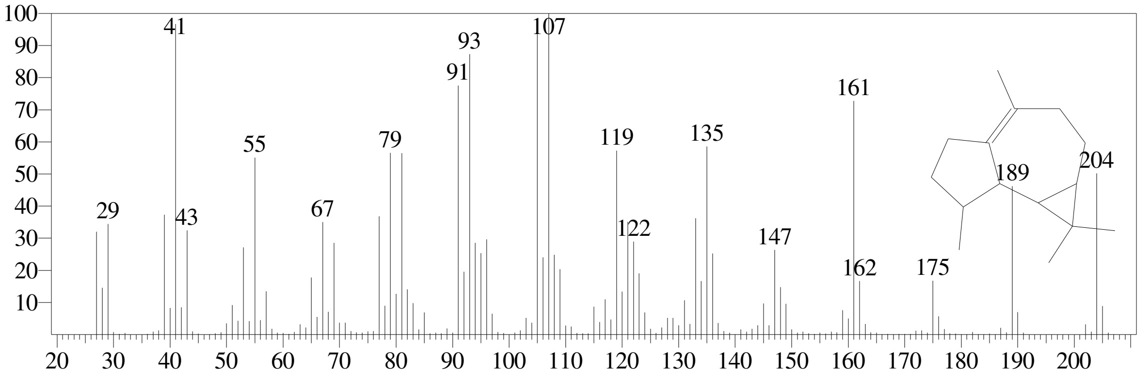

<< Target >>
Line#:16 R.Time:29.250(Scan#:3211) MassPeaks:71
RawMode:Averaged 29.242-29.258(3210-3212) BasePeak:93.05(46881)
BG Mode:Calc. from Peak Group 1 - Event 1 Scan

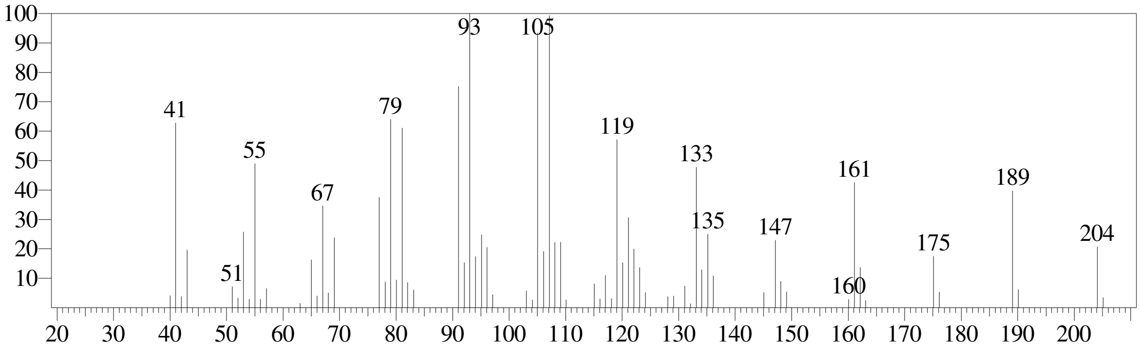

Hit#:3 Entry:24894 Library:NIST23s.lib
SI:93 Formula:C15H24 CAS:3691-11-0 MolWeight:204 RetIndex:1498
CompName:Azulene, 1,2,3,5,6,7,8,8a-octahydro-1,4-dimethyl-7-(1-methylethenyl)-, [1S-(1.alpha.,7.alpha.,8a.beta.)]- $$ (

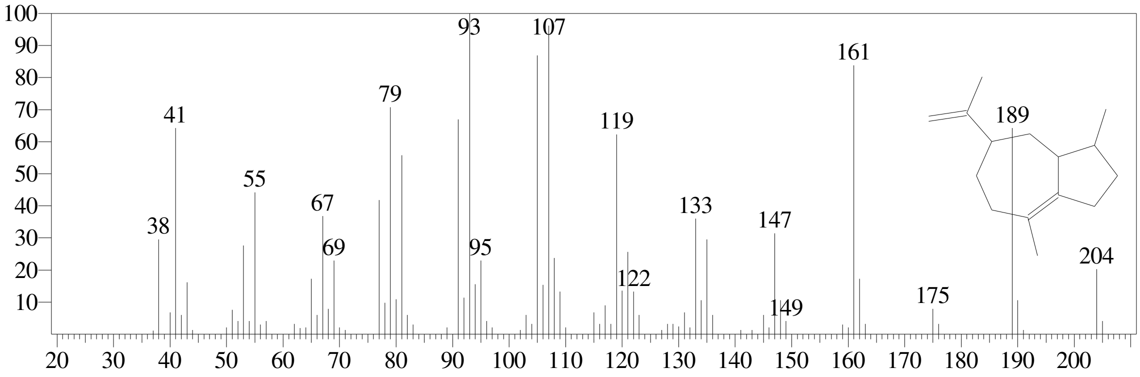

Hit#:4 Entry:24964 Library:NIST23s.lib
SI:92 Formula:C15H24 CAS:21747-46-6 MolWeight:204 RetIndex:1451
CompName:1H-Cycloprop[e]azulene, 1a,2,3,5,6,7,7a,7b-octahydro-1,1,4,7-tetramethyl-, [1aR-(1a.alpha.,7.alpha.,7a.beta.,

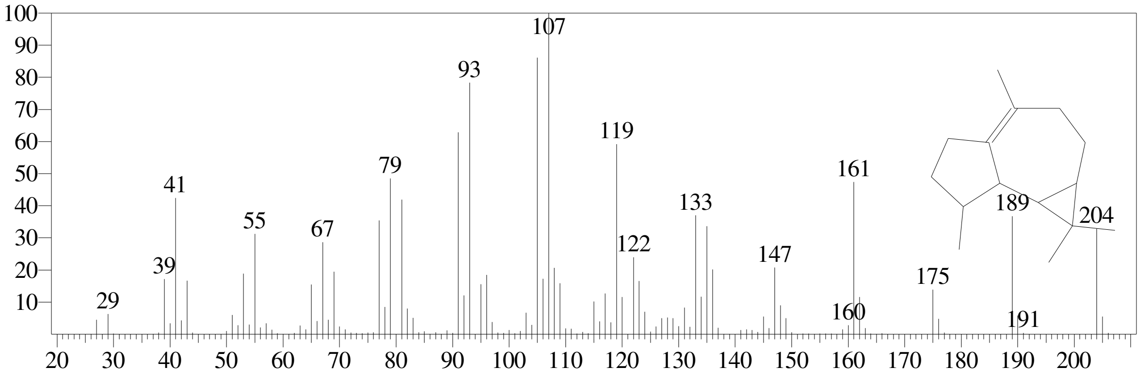

<< Target >>
Line#:16   R.Time:29.250(Scan#:3211) MassPeaks:71
RawMode:Averaged 29.242-29.258(3210-3212) BasePeak:93.05(46881)
BG Mode:Calc. from Peak Group 1 - Event 1 Scan

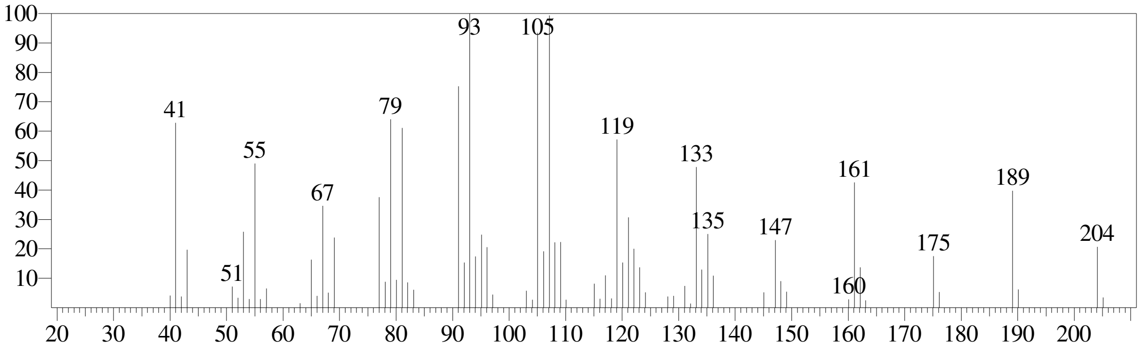

Hit#:5 Entry:62871 Library:NIST23-1.lib
SI:91 Formula:C15H24 CAS:21747-46-6 MolWeight:204 RetIndex:1451
CompName:1H-Cycloprop[e]azulene, 1a,2,3,5,6,7,7a,7b-octahydro-1,1,4,7-tetramethyl-, [1aR-(1a.alpha.,7.alpha.,7a.beta.,

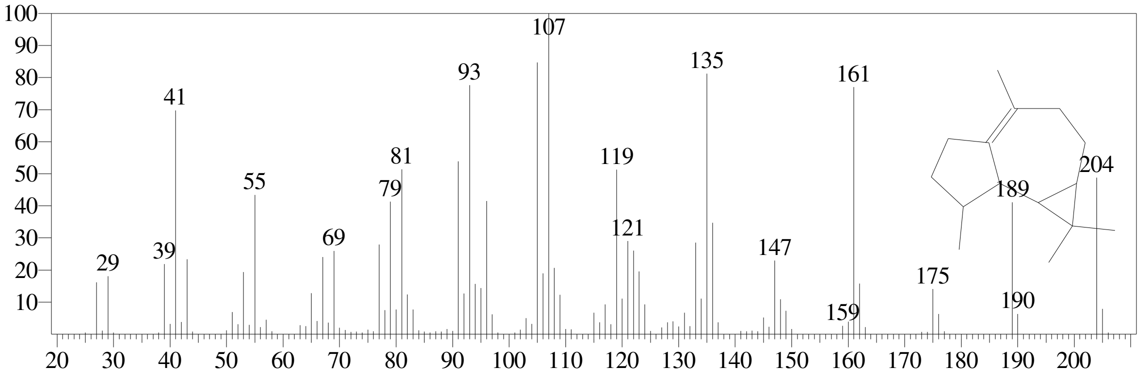

<< Target >>
Line#:17 R.Time:29.383(Scan#:3227) MassPeaks:21
RawMode:Averaged 29.375-29.392(3226-3228) BasePeak:161.10(5508)
BG Mode:Calc. from Peak Group 1 - Event 1 Scan

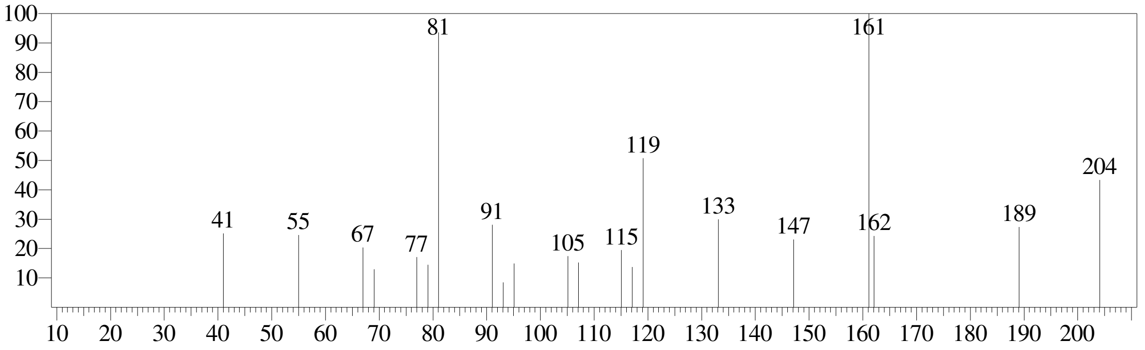

Hit#:1 Entry:62972 Library:NIST23-1.lib
SI:82 Formula:C15H24 CAS:41929-05-9 MolWeight:204 RetIndex:1525
CompName:Zonarene $$ (1R,8aR)-4-Isopropyl-1,6-dimethyl-1,2,3,7,8,8a-hexahydronaphthalene $$ Naphthalene, 1,2,3,7,

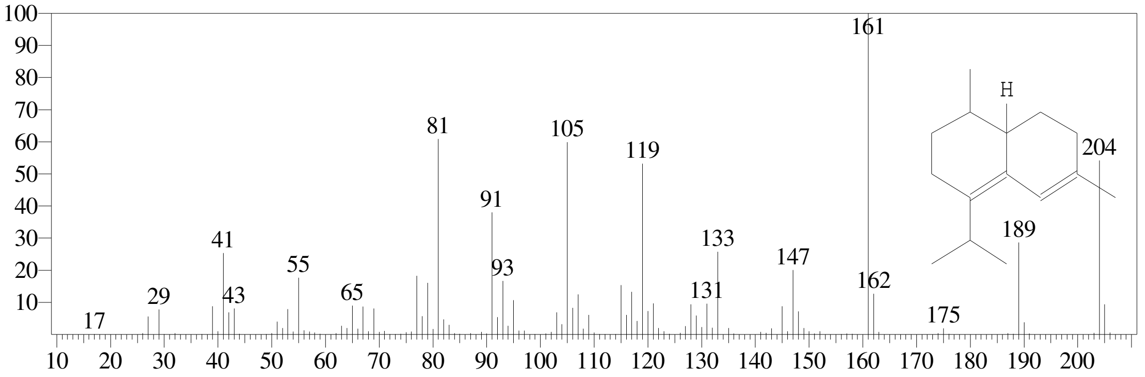

Hit#:2 Entry:62967 Library:NIST23-1.lib
SI:82 Formula:C15H24 CAS:0-00-0 MolWeight:204 RetIndex:1432
CompName:.beta.-GURJUNENE

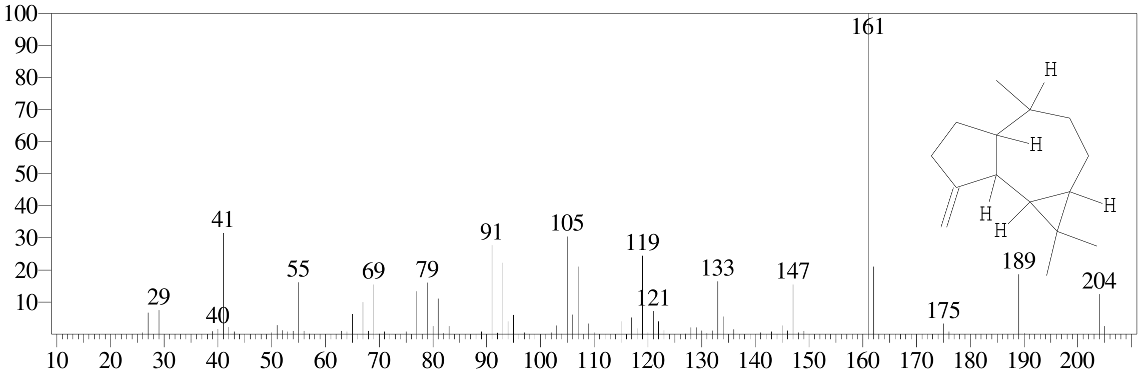

<< Target >>
Line#:17   R.Time:29.383(Scan#:3227) MassPeaks:21
RawMode:Averaged 29.375-29.392(3226-3228) BasePeak:161.10(5508)
BG Mode:Calc. from Peak Group 1 - Event 1 Scan

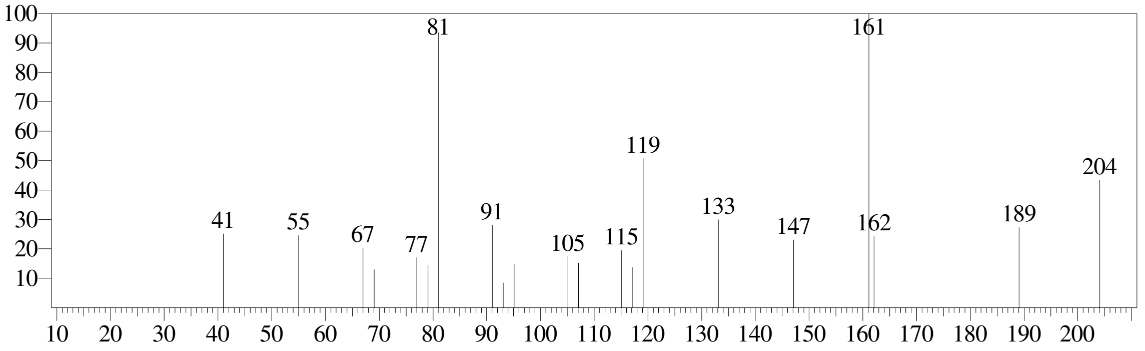

Hit#:3 Entry:25051 Library:NIST23s.lib
SI:81 Formula:C15H24 CAS:41702-63-0 MolWeight:204 RetIndex:1506
CompName:Epizonarene $$ 4-Isopropyl-1,6-dimethyl-1,2,3,7,8,8a-hexahydronaphthalene # $$

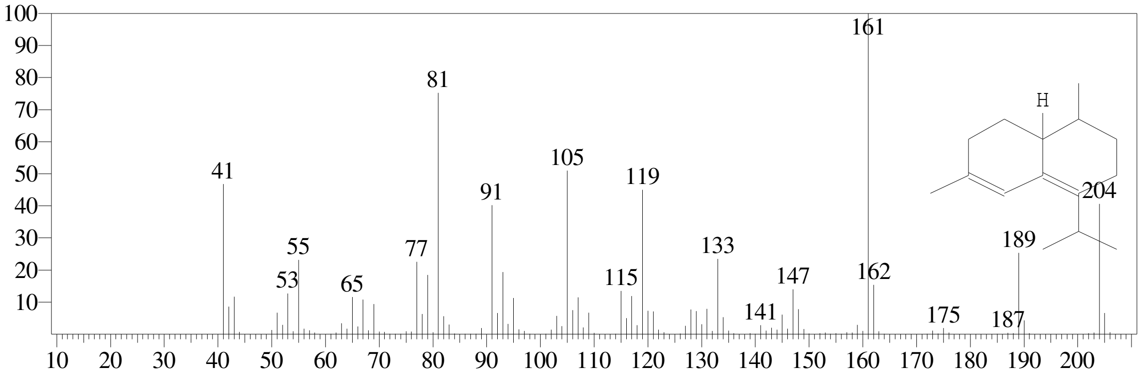

Hit#:4 Entry:25109 Library:NIST23s.lib
SI:80 Formula:C15H24 CAS:41929-05-9 MolWeight:204 RetIndex:1525
CompName:Zonarene $$ (1R,8aR)-4-Isopropyl-1,6-dimethyl-1,2,3,7,8,8a-hexahydronaphthalene $$ Naphthalene, 1,2,3,7,

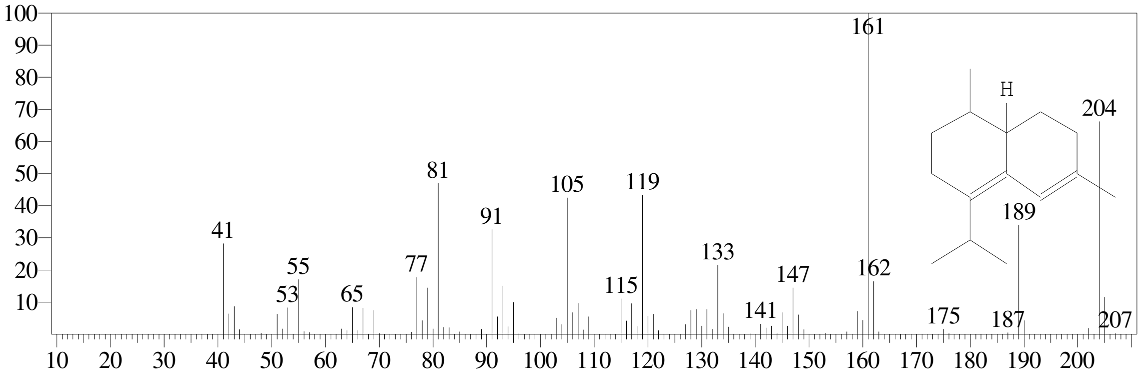

<< Target >>
Line#:17 R.Time:29.383(Scan#:3227) MassPeaks:21
RawMode:Averaged 29.375-29.392(3226-3228) BasePeak:161.10(5508)
BG Mode:Calc. from Peak Group 1 - Event 1 Scan

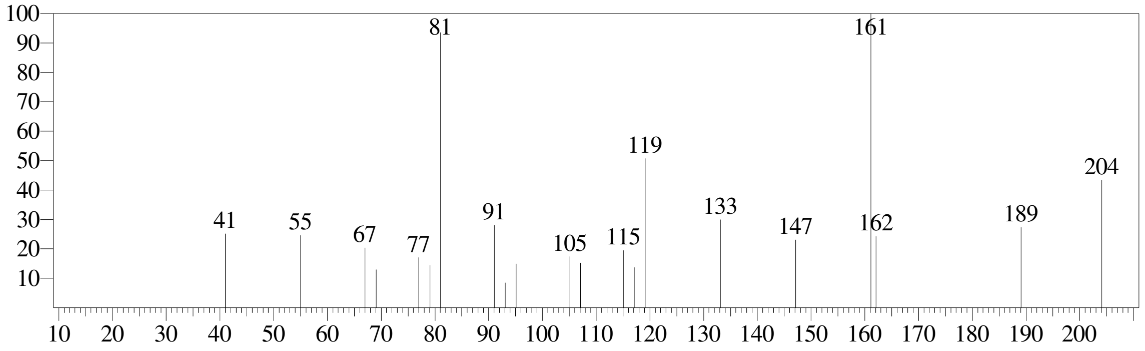

Hit#:5 Entry:25063 Library:NIST23s.lib
SI:80 Formula:C15H24 CAS:17334-55-3 MolWeight:204 RetIndex:1446
CompName:1H-Cyclopropa[a]naphthalene, 1a,2,3,5,6,7,7a,7b-octahydro-1,1,7,7a-tetramethyl-, [1aR-(1a.alpha.,7.alpha.,7a

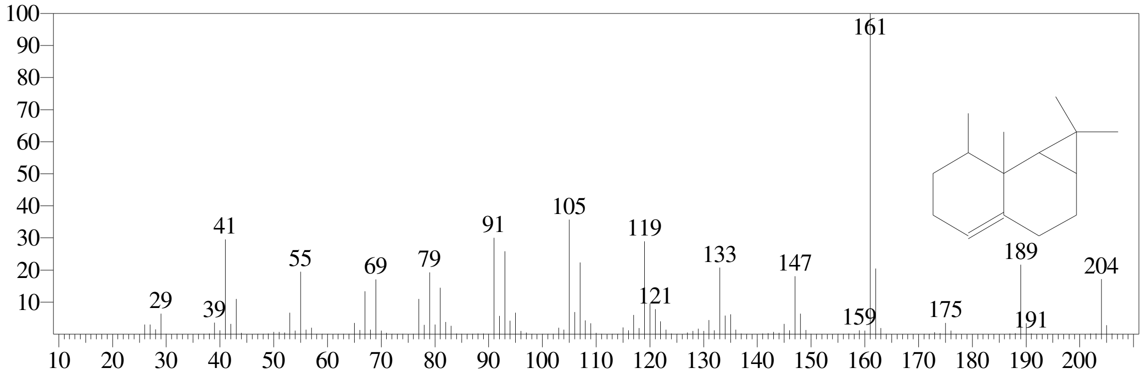

<< Target >>
Line#:18 R.Time:29.458(Scan#:3236) MassPeaks:27
RawMode:Averaged 29.450-29.467(3235-3237) BasePeak:105.05(11134)
BG Mode:Calc. from Peak Group 1 - Event 1 Scan

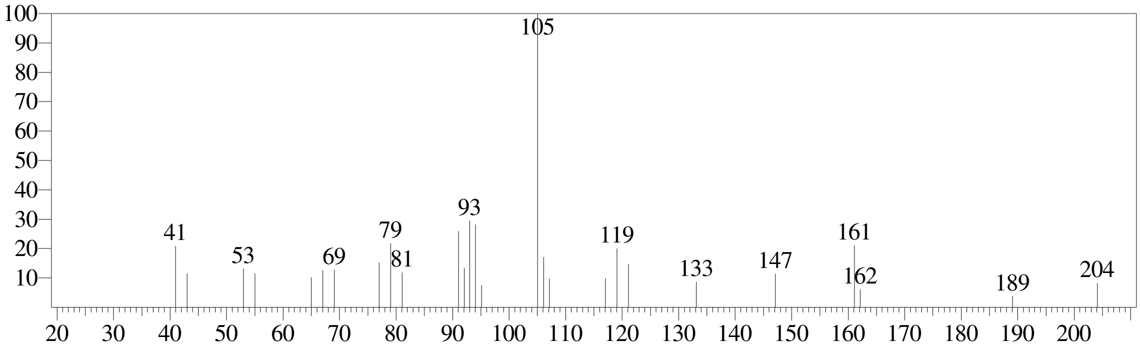

Hit#:1 Entry:62856 Library:NIST23-1.lib
SI:87 Formula:C15H24 CAS:10208-80-7 MolWeight:204 RetIndex:1500
CompName:.alpha.-Muurolene $$ Naphthalene, 1,2,4a,5,6,8a-hexahydro-4,7-dimethyl-1-(1-methylethyl)-, (1S,4aS,8aR)- $

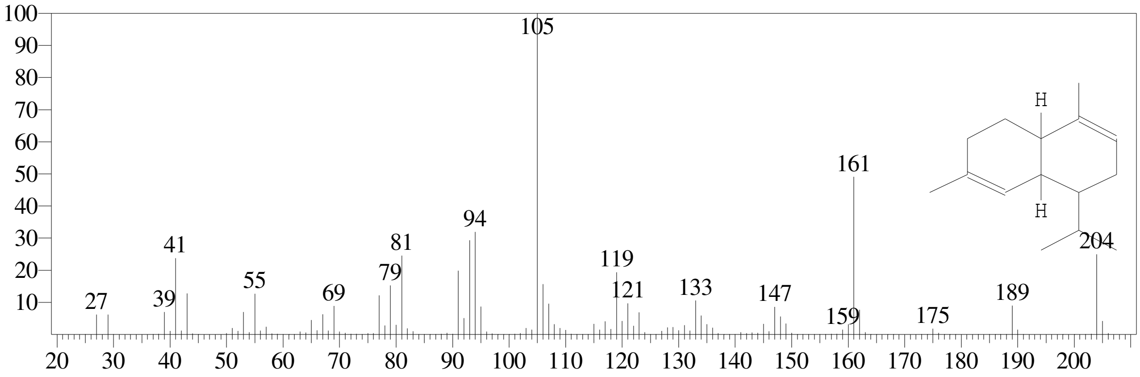

Hit#:2 Entry:62857 Library:NIST23-1.lib
SI:86 Formula:C15H24 CAS:483-75-0 MolWeight:204 RetIndex:1500
CompName:Naphthalene, 1,2,4a,5,6,8a-hexahydro-4,7-dimethyl-1-(1-methylethyl)- $$ 1-Isopropyl-4,7-dimethyl-1,2,4a,5,

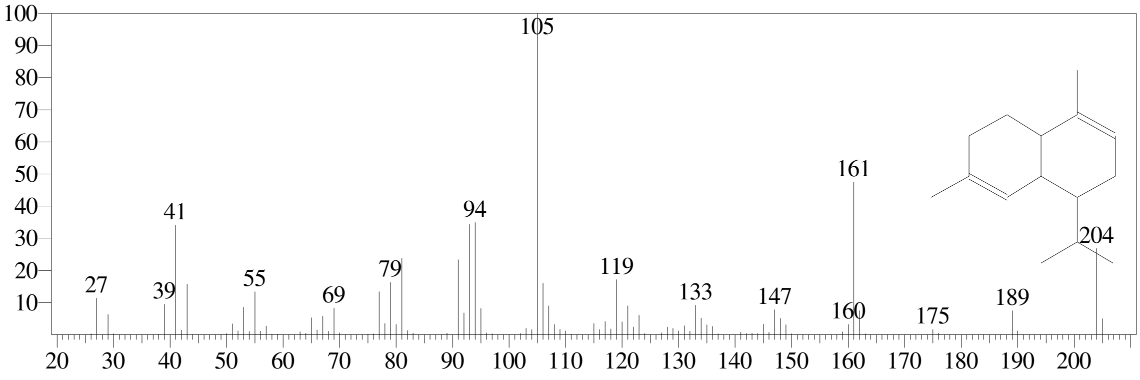

<< Target >>
Line#:18   R.Time:29.458(Scan#:3236) MassPeaks:27
RawMode:Averaged 29.450-29.467(3235-3237) BasePeak:105.05(11134)
BG Mode:Calc. from Peak Group 1 - Event 1 Scan

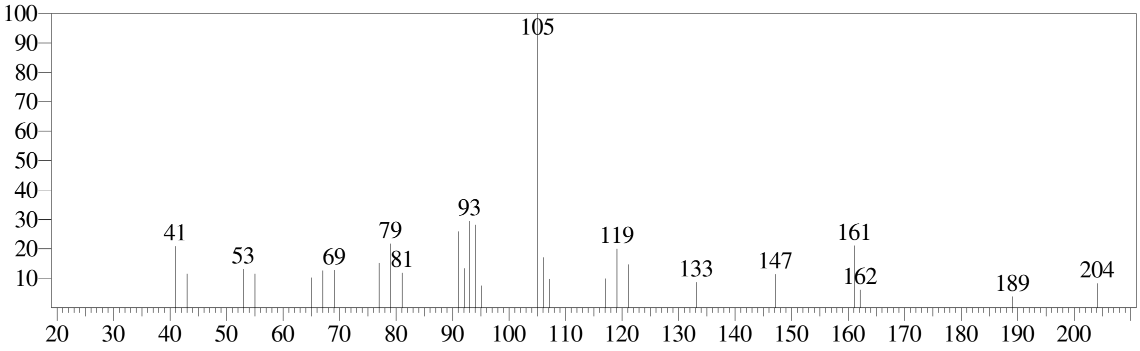

Hit#:3 Entry:24947 Library:NIST23s.lib
SI:86 Formula:C15H24 CAS:31983-22-9 MolWeight:204 RetIndex:1498
CompName:Naphthalene, 1,2,4a,5,6,8a-hexahydro-4,7-dimethyl-1-(1-methylethyl)-, (1.alpha.,4a.alpha.,8a.alpha.)- $$ [1.a

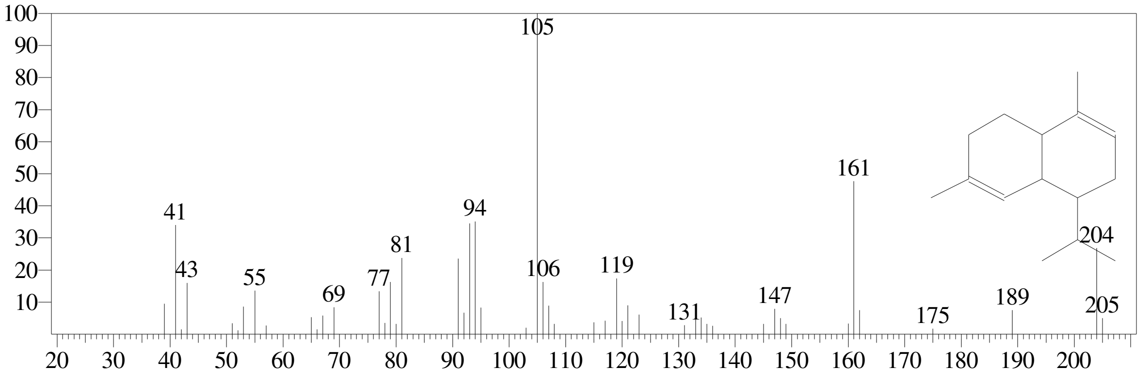

Hit#:4 Entry:24946 Library:NIST23s.lib
SI:85 Formula:C15H24 CAS:10208-80-7 MolWeight:204 RetIndex:1500
CompName:.alpha.-Muurolene $$ Naphthalene, 1,2,4a,5,6,8a-hexahydro-4,7-dimethyl-1-(1-methylethyl)-, (1S,4aS,8aR)- $

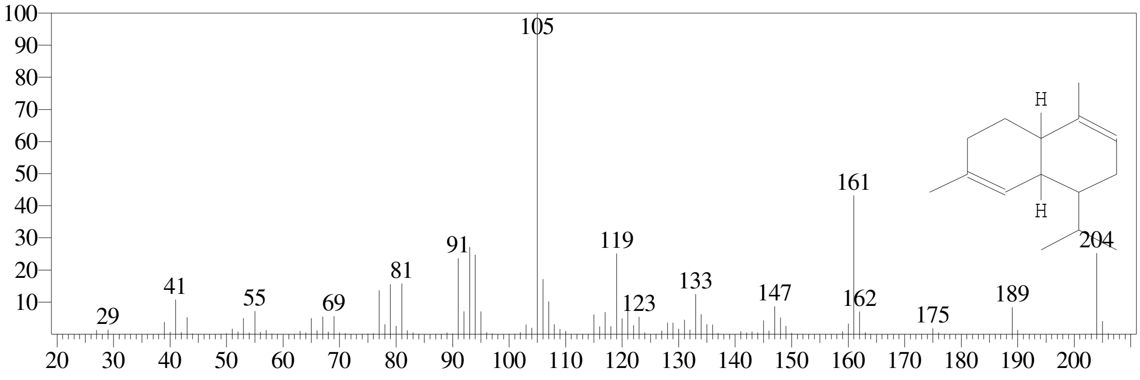

<< Target >>
Line#:18   R.Time:29.458(Scan#:3236) MassPeaks:27
RawMode:Averaged 29.450-29.467(3235-3237) BasePeak:105.05(11134)
BG Mode:Calc. from Peak Group 1 - Event 1 Scan

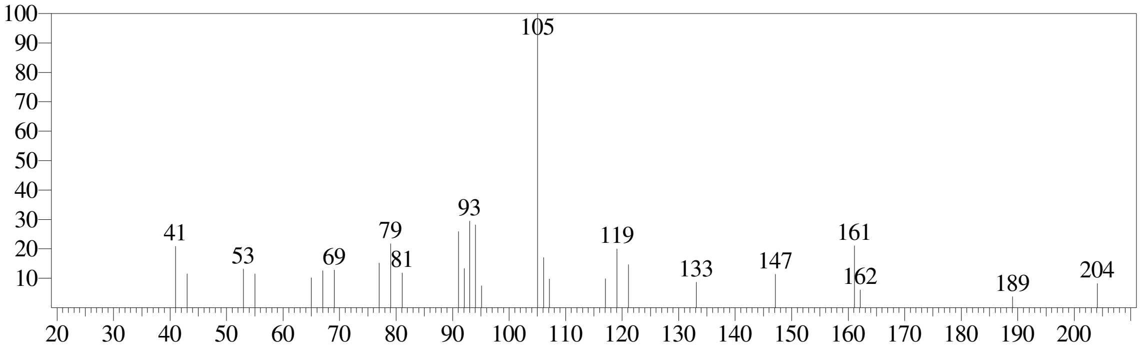

Hit#:5 Entry:24926 Library:NIST23s.lib
SI:84 Formula:C15H24 CAS:54868-40-5 MolWeight:204 RetIndex:1489
CompName:(4S,4aR,6R)-4,4a-Dimethyl-6-(prop-1-en-2-yl)-1,2,3,4,4a,5,6,7-octahydronaphthalene $$ Naphthalene, 1,2,3,

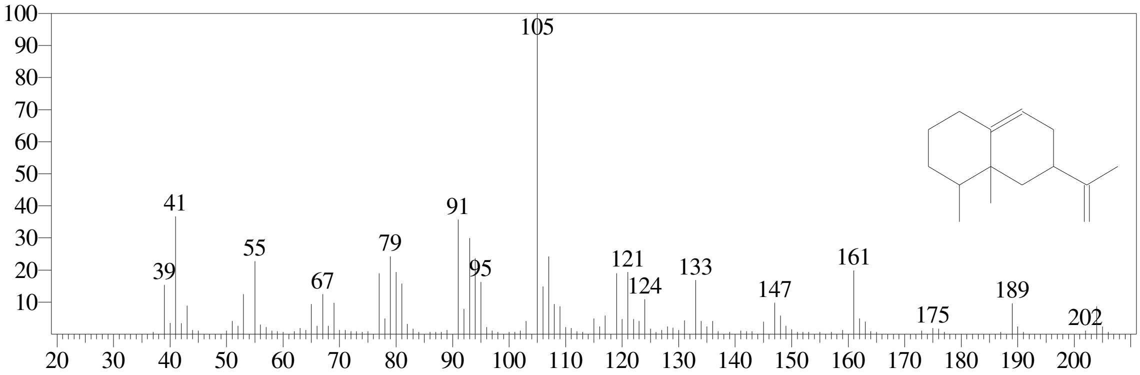

<< Target >>
Line#:19 R.Time:29.775(Scan#:3274) MassPeaks:54
RawMode:Averaged 29.767-29.783(3273-3275) BasePeak:69.05(69826)
BG Mode:Calc. from Peak Group 1 - Event 1 Scan

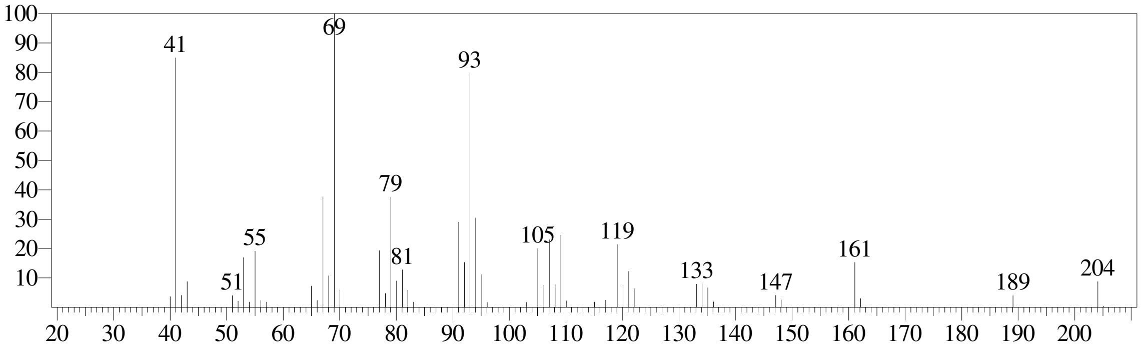

Hit#:1 Entry:24855 Library:NIST23s.lib
SI:96 Formula:C15H24 CAS:495-61-4 MolWeight:204 RetIndex:1502
CompName:.beta.-Bisabolene $$ Cyclohexene, 1-methyl-4-(5-methyl-1-methylene-4-hexenyl)-, (S)- $$ 1,5-Heptadiene, 6

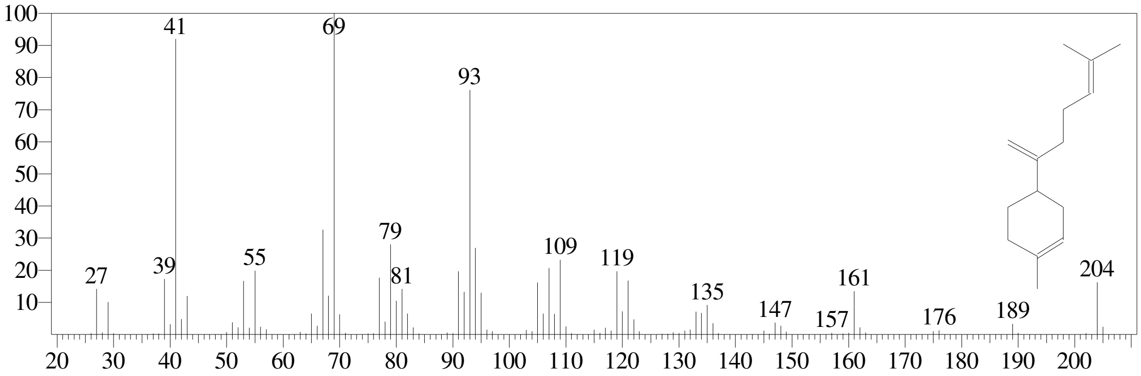

Hit#:2 Entry:24810 Library:NIST23s.lib
SI:95 Formula:C15H24 CAS:495-61-4 MolWeight:204 RetIndex:1502
CompName:.beta.-Bisabolene $$ Cyclohexene, 1-methyl-4-(5-methyl-1-methylene-4-hexenyl)-, (S)- $$ 1,5-Heptadiene, 6

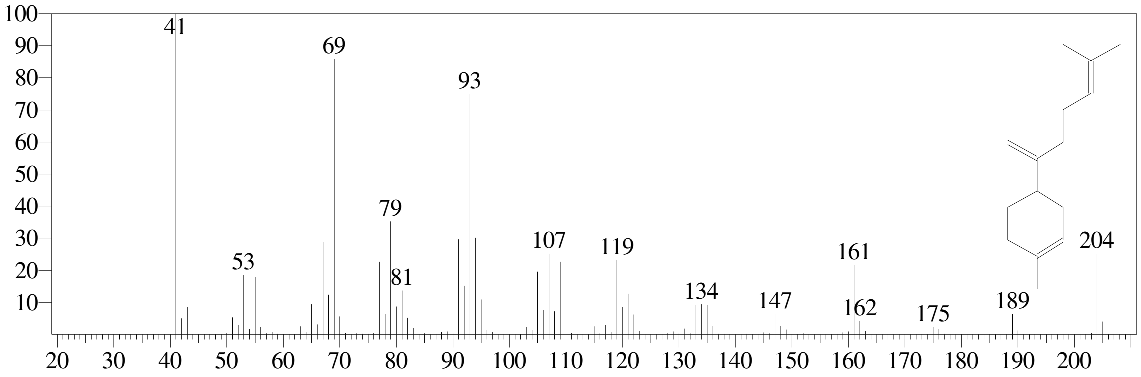

<< Target >>
Line#:19   R.Time:29.775(Scan#:3274) MassPeaks:54
RawMode:Averaged 29.767-29.783(3273-3275) BasePeak:69.05(69826)
BG Mode:Calc. from Peak Group 1 - Event 1 Scan

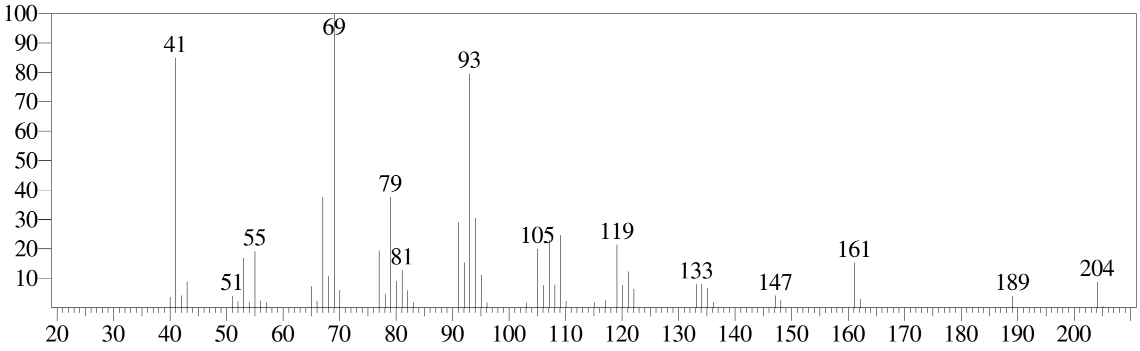

Hit#:3 Entry:24856 Library:NIST23s.lib
SI:95 Formula:C15H24 CAS:495-61-4 MolWeight:204 RetIndex:1502
CompName:.beta.-Bisabolene $$ Cyclohexene, 1-methyl-4-(5-methyl-1-methylene-4-hexenyl)-, (S)- $$ 1,5-Heptadiene, 6

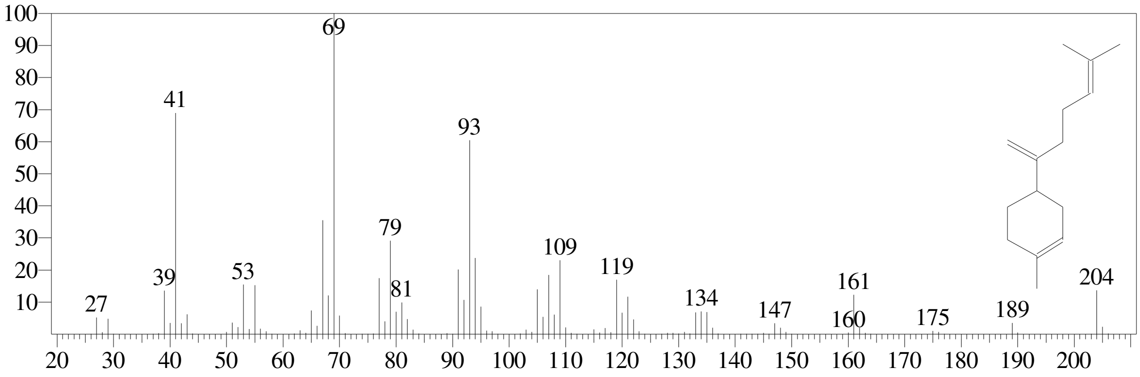

Hit#:4 Entry:62778 Library:NIST23-1.lib
SI:94 Formula:C15H24 CAS:495-61-4 MolWeight:204 RetIndex:1502
CompName:.beta.-Bisabolene $$ Cyclohexene, 1-methyl-4-(5-methyl-1-methylene-4-hexenyl)-, (S)- $$ 1,5-Heptadiene, 6

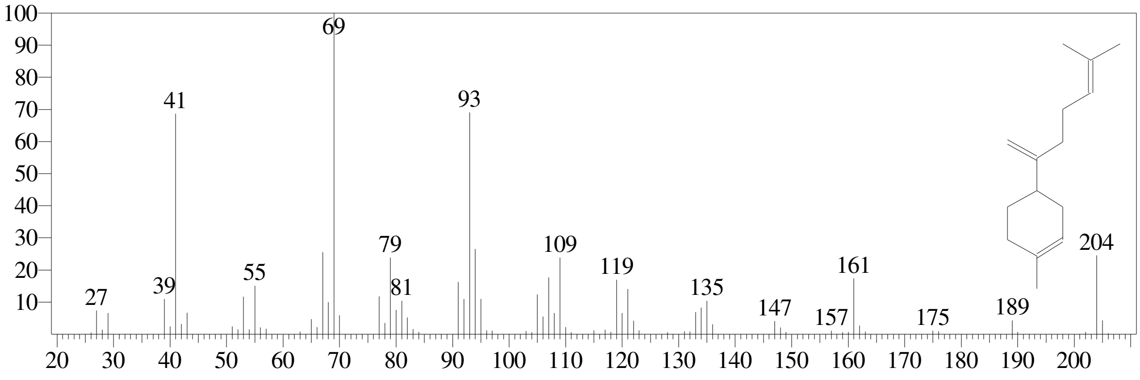

<< Target >>
Line#:19   R.Time:29.775(Scan#:3274) MassPeaks:54
RawMode:Averaged 29.767-29.783(3273-3275) BasePeak:69.05(69826)
BG Mode:Calc. from Peak Group 1 - Event 1 Scan

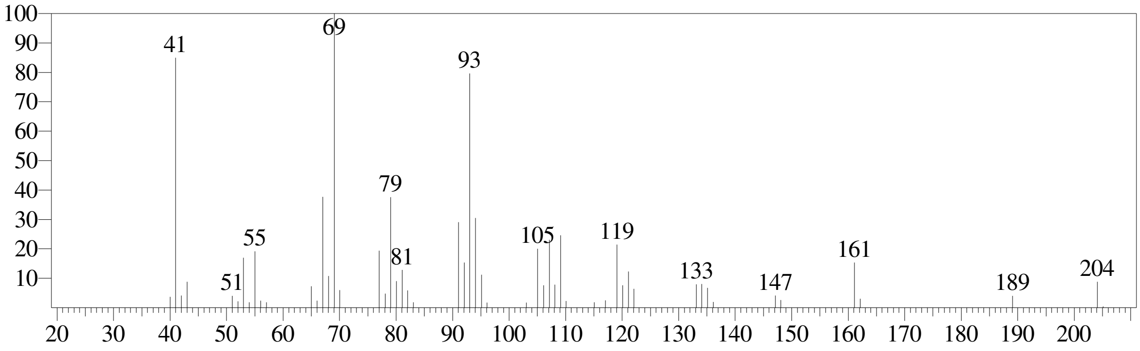

Hit#:5 Entry:24809 Library:NIST23s.lib
SI:91 Formula:C15H24 CAS:495-61-4 MolWeight:204 RetIndex:1502
CompName:.beta.-Bisabolene $$ Cyclohexene, 1-methyl-4-(5-methyl-1-methylene-4-hexenyl)-, (S)- $$ 1,5-Heptadiene, 6

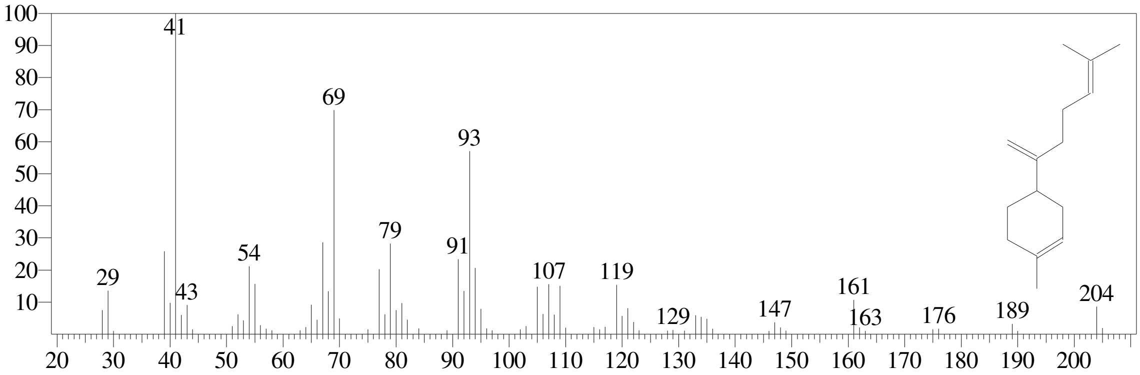

<< Target >>
Line#:20 R.Time:30.008(Scan#:3302) MassPeaks:31
RawMode:Averaged 30.000-30.017(3301-3303) BasePeak:161.10(10070)
BG Mode:Calc. from Peak Group 1 - Event 1 Scan

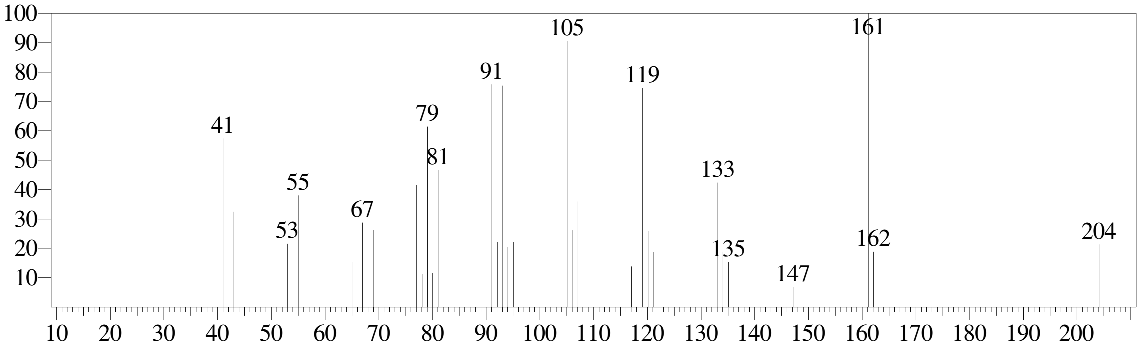

Hit#:1 Entry:62975 Library:NIST23-1.lib
SI:90 Formula:C15H24 CAS:317819-80-0 MolWeight:204 RetIndex:1478
CompName:(S,1Z,6Z)-8-Isopropyl-1-methyl-5-methylenecyclodeca-1,6-diene $$ 1,6-Cyclodecadiene, 1-methyl-5-methyl

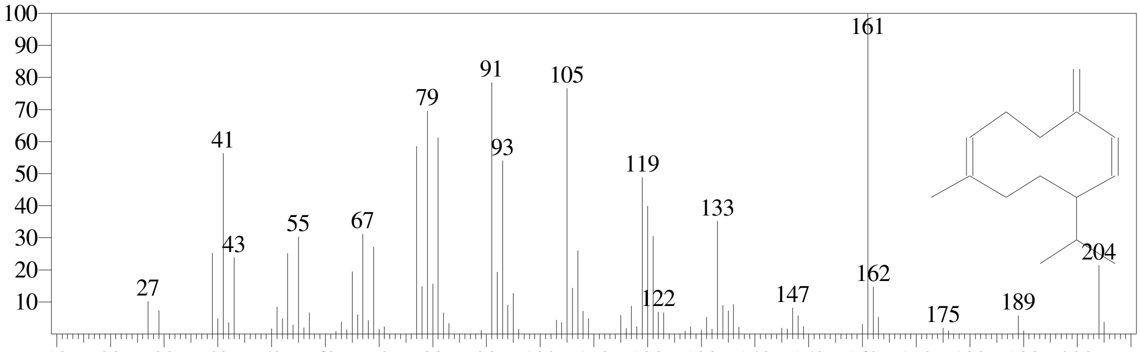

Hit#:2 Entry:25071 Library:NIST23s.lib
SI:90 Formula:C15H24 CAS:483-75-0 MolWeight:204 RetIndex:1500
CompName:Naphthalene, 1,2,4a,5,6,8a-hexahydro-4,7-dimethyl-1-(1-methylethyl)- $$ 1-Isopropyl-4,7-dimethyl-1,2,4a,5,

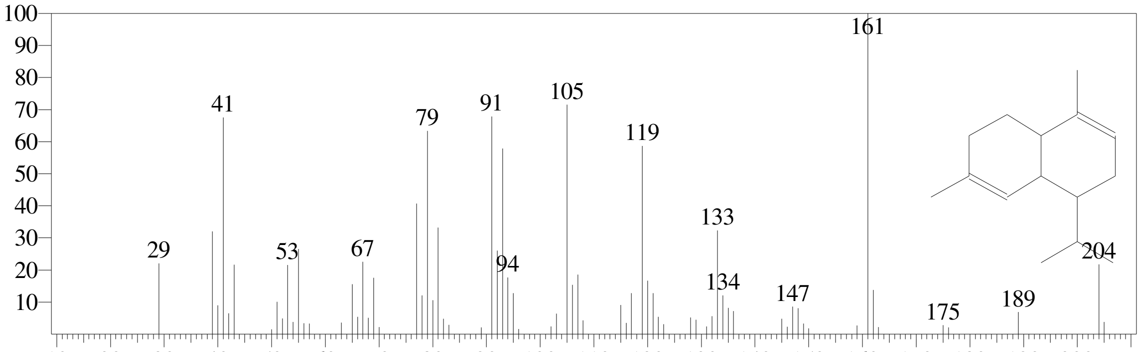

<< Target >>
Line#:20 R.Time:30.008(Scan#:3302) MassPeaks:31
RawMode:Averaged 30.000-30.017(3301-3303) BasePeak:161.10(10070)
BG Mode:Calc. from Peak Group 1 - Event 1 Scan

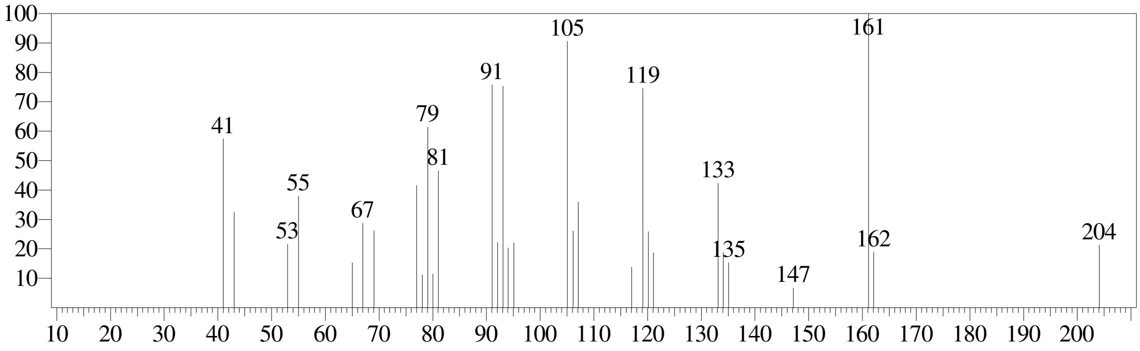

Hit#:3 Entry:25048 Library:NIST23s.lib
SI:90 Formula:C15H24 CAS:6980-46-7 MolWeight:204 RetIndex:1483
CompName:(1S,4aR,8aS)-1-Isopropyl-7-methyl-4-methylene-1,2,3,4,4a,5,6,8a-octahydronaphthalene $$ .gamma.-Amorp

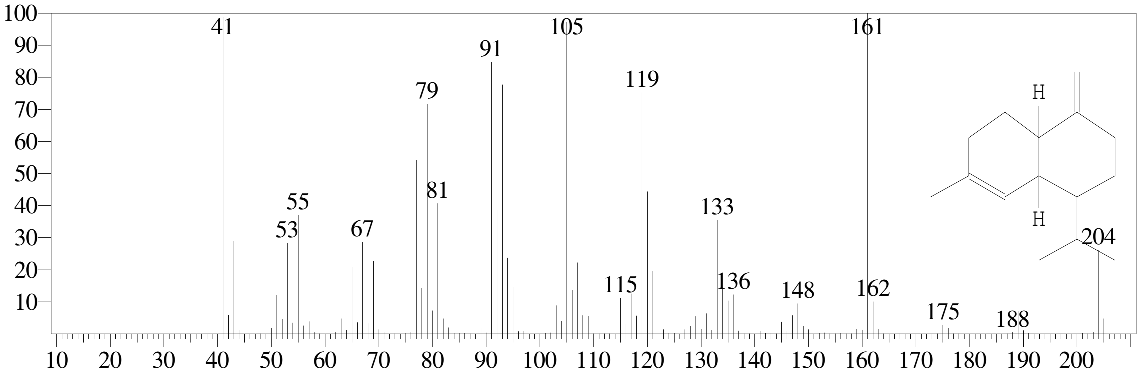

Hit#:4 Entry:25047 Library:NIST23s.lib
SI:89 Formula:C15H24 CAS:30021-74-0 MolWeight:204 RetIndex:1483
CompName:.gamma.-Muurolene $$ Naphthalene, 1,2,3,4,4a,5,6,8a-octahydro-7-methyl-4-methylene-1-(1-methylethyl)-, (

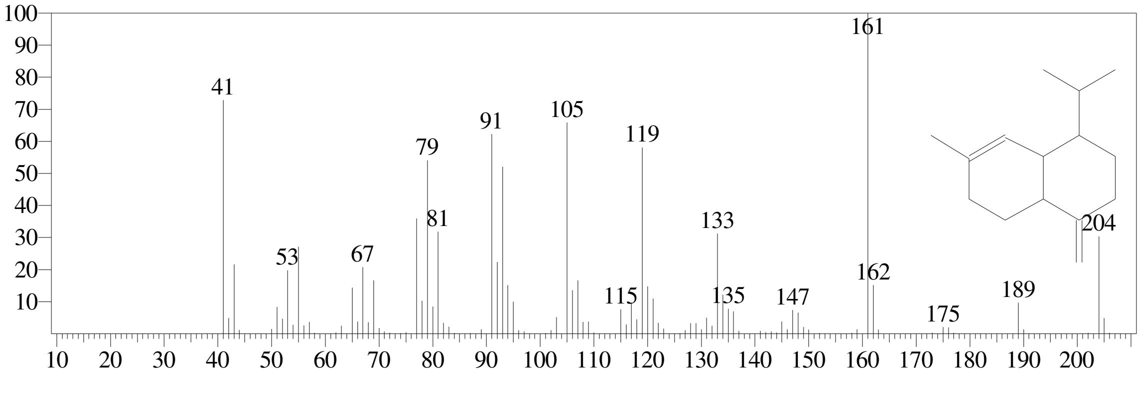

<< Target >>
Line#:20   R.Time:30.008(Scan#:3302) MassPeaks:31
RawMode:Averaged 30.000-30.017(3301-3303) BasePeak:161.10(10070)
BG Mode:Calc. from Peak Group 1 - Event 1 Scan

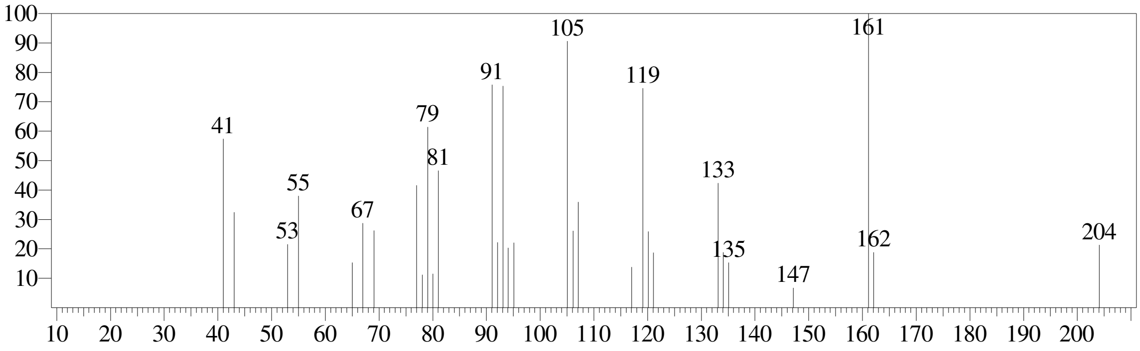

Hit#:5 Entry:62981 Library:NIST23-1.lib
SI:89 Formula:C15H24 CAS:23986-74-5 MolWeight:204 RetIndex:1478
CompName:Germacrene D $$ (S,1Z,6Z)-8-Isopropyl-1-methyl-5-methylenecyclodeca-1,6-diene $$ D-Germacrene $$ 1(1

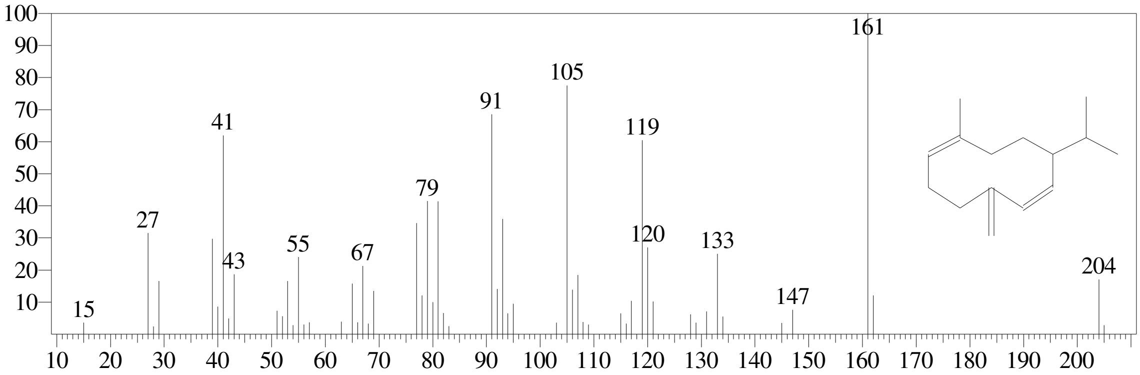

<< Target >>
Line#:21 R.Time:30.375(Scan#:3346) MassPeaks:52
RawMode:Averaged 30.367-30.383(3345-3347) BasePeak:119.10(35507)
BG Mode:Calc. from Peak Group 1 - Event 1 Scan

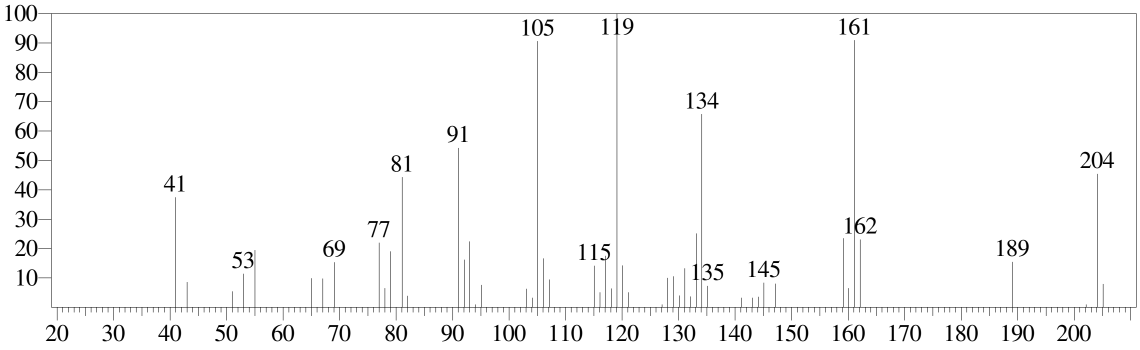

Hit#:1 Entry:63005 Library:NIST23-1.lib
SI:93 Formula:C15H24 CAS:189165-79-5 MolWeight:204 RetIndex:1526
CompName:Amorphene,delta-

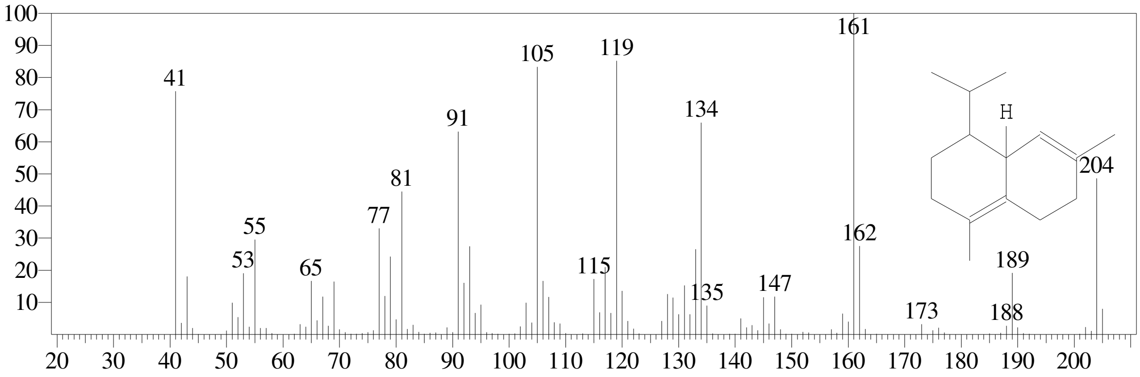

Hit#:2 Entry:25112 Library:NIST23s.lib
SI:92 Formula:C15H24 CAS:483-76-1 MolWeight:204 RetIndex:1526
CompName:Naphthalene, 1,2,3,5,6,8a-hexahydro-4,7-dimethyl-1-(1-methylethyl)-, (1S-cis)- $$ Cadina-1(10),4-diene $$.

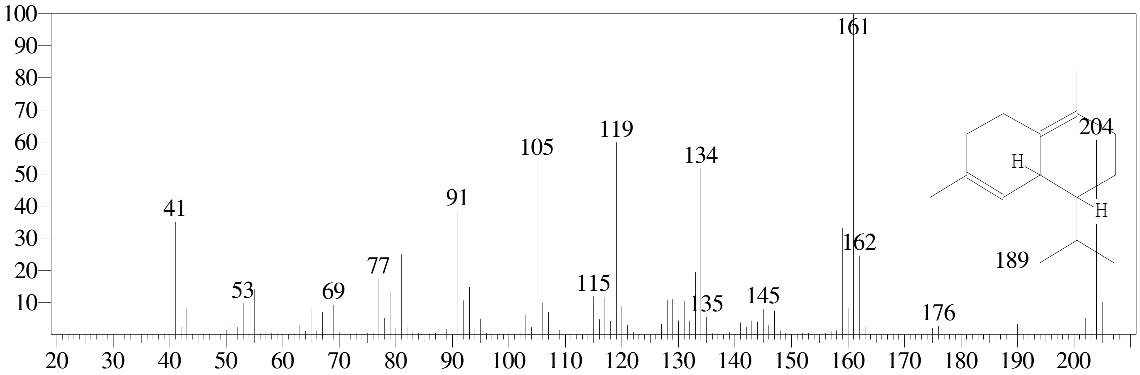

<< Target >>
Line#:21   R.Time:30.375(Scan#:3346) MassPeaks:52
RawMode:Averaged 30.367-30.383(3345-3347) BasePeak:119.10(35507)
BG Mode:Calc. from Peak Group 1 - Event 1 Scan

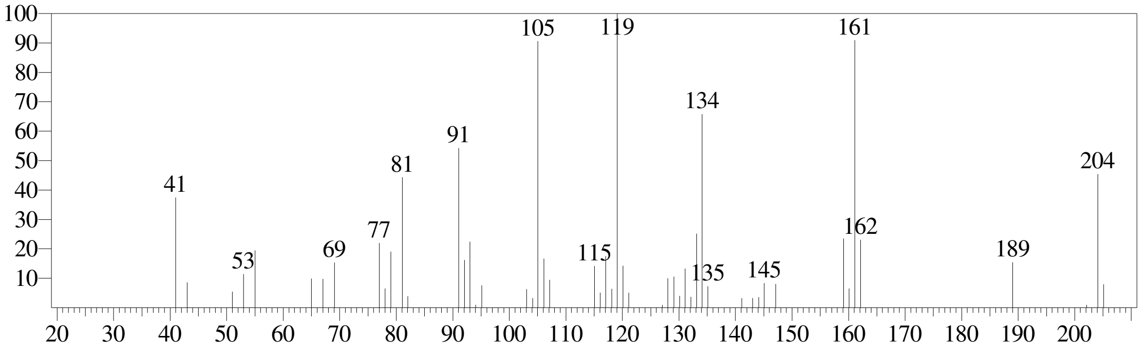

Hit#:3 Entry:63006 Library:NIST23-1.lib
SI:91 Formula:C15H24 CAS:16729-01-4 MolWeight:204 RetIndex:1526
CompName:1-Isopropyl-4,7-dimethyl-1,2,3,5,6,8a-hexahydronaphthalene $$ Cadina-1(10),4-diene $$

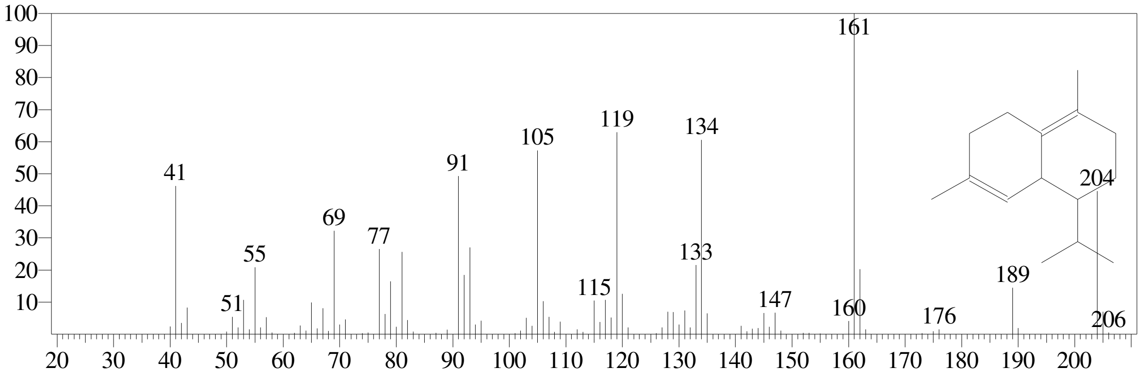

Hit#:4 Entry:25090 Library:NIST23s.lib
SI:90 Formula:C15H24 CAS:483-76-1 MolWeight:204 RetIndex:1526
CompName:Naphthalene, 1,2,3,5,6,8a-hexahydro-4,7-dimethyl-1-(1-methylethyl)-, (1S-cis)- $$ Cadina-1(10),4-diene $$.

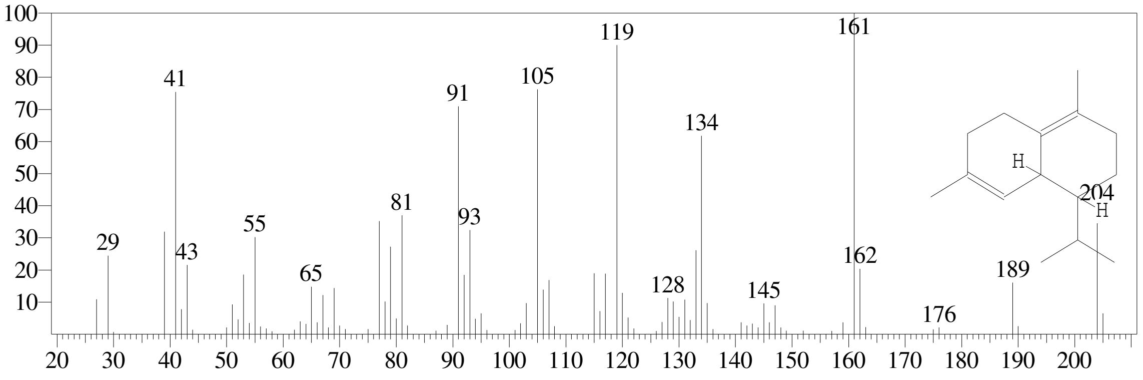

<< Target >>
Line#:21   R.Time:30.375(Scan#:3346) MassPeaks:52
RawMode:Averaged 30.367-30.383(3345-3347) BasePeak:119.10(35507)
BG Mode:Calc. from Peak Group 1 - Event 1 Scan

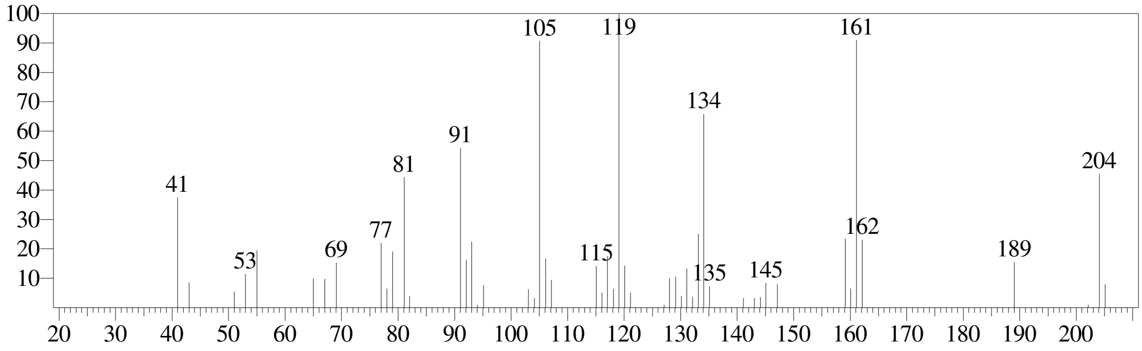

Hit#:5 Entry:24949 Library:NIST23s.lib
SI:88 Formula:C15H24 CAS:17699-14-8 MolWeight:204 RetIndex:1381
CompName:.alpha.-Cubebene $$ 1H-Cyclopenta[1,3]cyclopropa[1,2]benzene, 3a,3b,4,5,6,7-hexahydro-3,7-dimethyl-4-(1

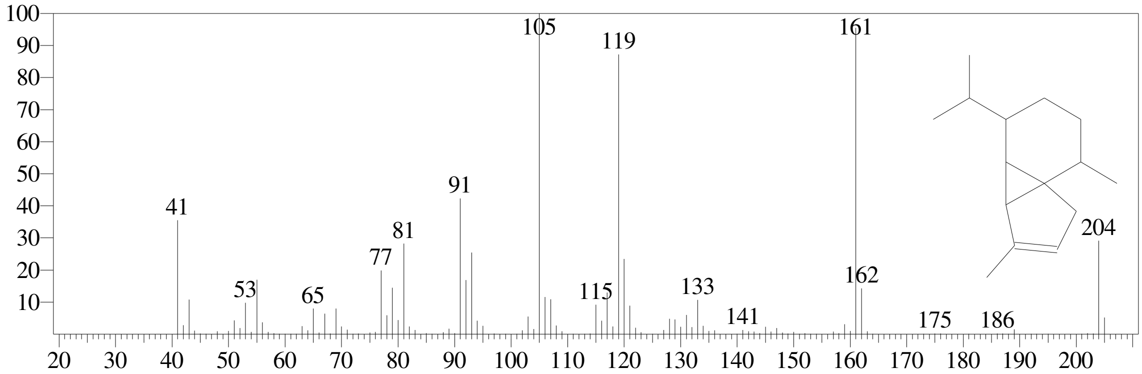

<< Target >>
Line#:22   R.Time:32.475(Scan#:3598) MassPeaks:43
RawMode:Averaged 32.467-32.483(3597-3599) BasePeak:43.00(10983)
BG Mode:Calc. from Peak Group 1 - Event 1 Scan

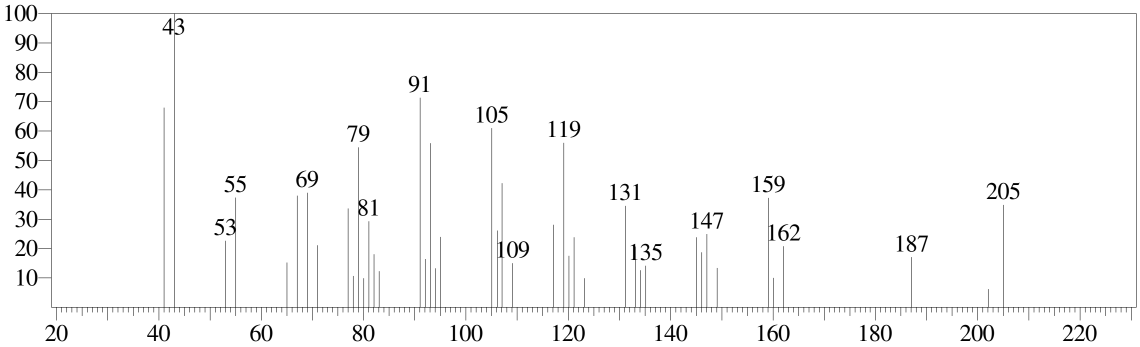

Hit#:1 Entry:28181 Library:NIST23s.lib
SI:95 Formula:C15H24O CAS:6750-60-3 MolWeight:220 RetIndex:1584
CompName:1H-Cycloprop[e]azulen-7-ol, decahydro-1,1,7-trimethyl-4-methylene-, [1ar-(1a.alpha.,4a.alpha.,7.beta.,7a.bet

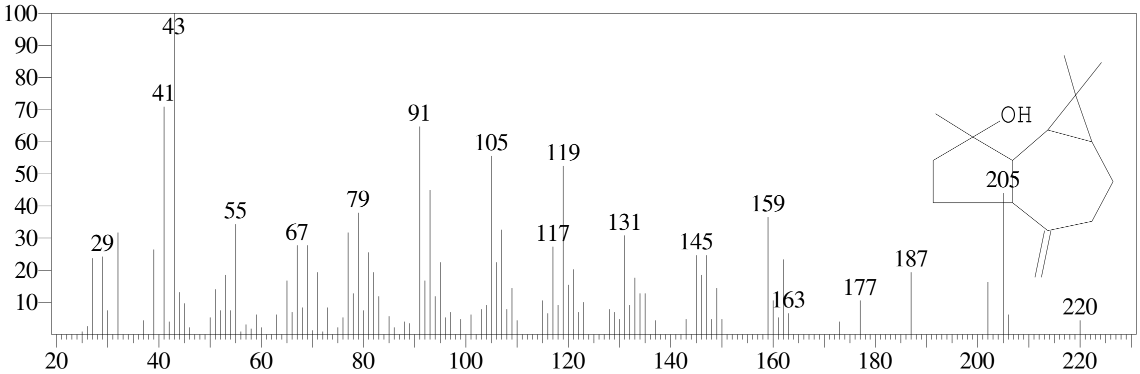

Hit#:2 Entry:80771 Library:NIST23-1.lib
SI:92 Formula:C15H24O CAS:77171-55-2 MolWeight:220 RetIndex:1584
CompName:(-)-Spathulenol $$ (1aS,4aS,7R,7aS,7bS)-1,1,7-Trimethyl-4-methylenedecahydro-1H-cyclopropa[e]azulen-7-

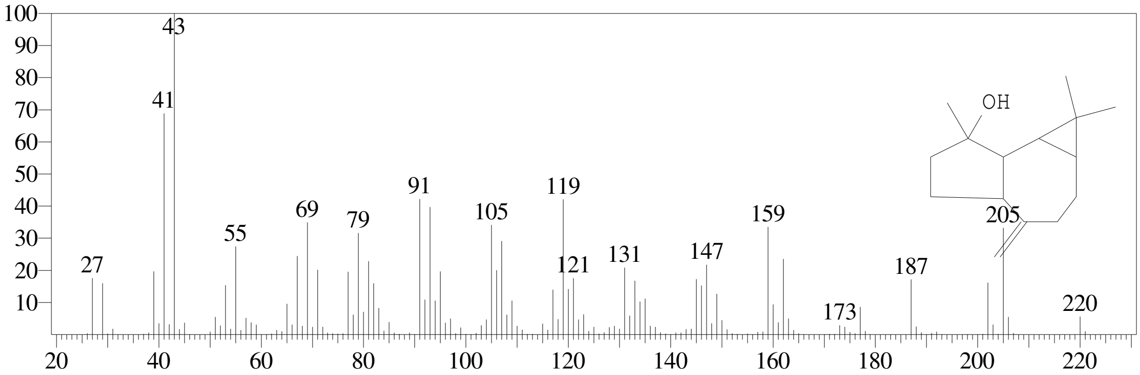

<< Target >>
Line#:22 R.Time:32.475(Scan#:3598) MassPeaks:43
RawMode:Averaged 32.467-32.483(3597-3599) BasePeak:43.00(10983)
BG Mode:Calc. from Peak Group 1 - Event 1 Scan

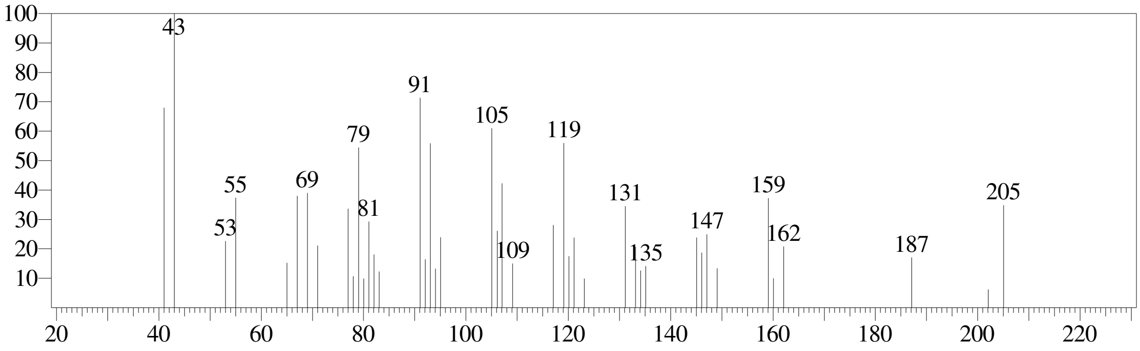

Hit#:3 Entry:80776 Library:NIST23-1.lib
SI:91 Formula:C15H24O CAS:6750-60-3 MolWeight:220 RetIndex:1584
CompName:1H-Cycloprop[e]azulen-7-ol, decahydro-1,1,7-trimethyl-4-methylene-, [1ar-(1a.alpha.,4a.alpha.,7.beta.,7a.bet

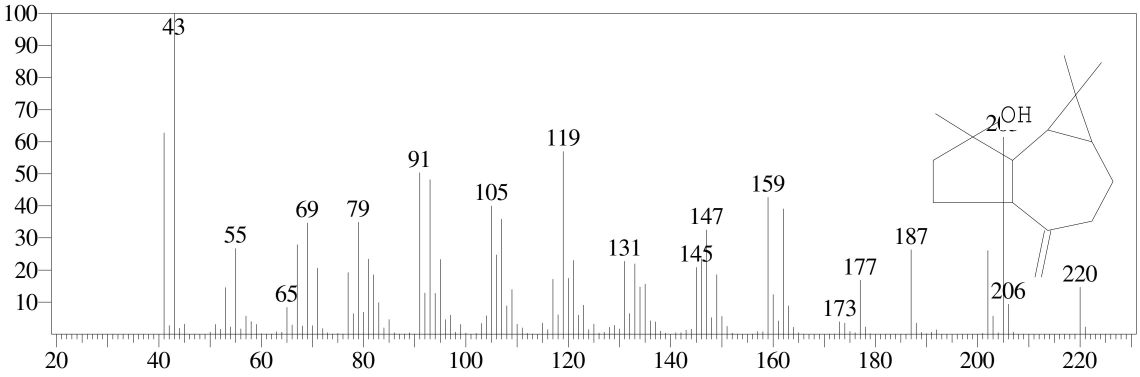

Hit#:4 Entry:28190 Library:NIST23s.lib
SI:90 Formula:C15H24O CAS:6750-60-3 MolWeight:220 RetIndex:1584
CompName:1H-Cycloprop[e]azulen-7-ol, decahydro-1,1,7-trimethyl-4-methylene-, [1ar-(1a.alpha.,4a.alpha.,7.beta.,7a.bet

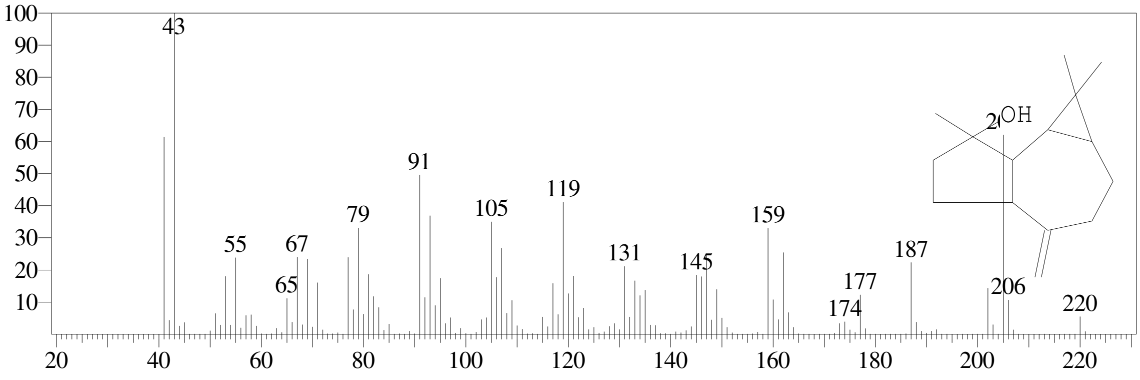

<< Target >>
Line#:22   R.Time:32.475(Scan#:3598) MassPeaks:43
RawMode:Averaged 32.467-32.483(3597-3599) BasePeak:43.00(10983)
BG Mode:Calc. from Peak Group 1 - Event 1 Scan

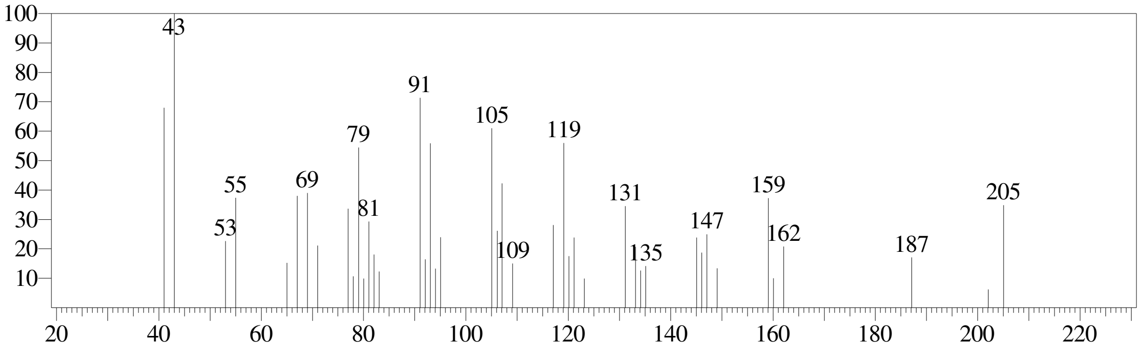

Hit#:5 Entry:28225 Library:NIST23s.lib
SI:89 Formula:C15H24O CAS:6750-60-3 MolWeight:220 RetIndex:1584
CompName:1H-Cycloprop[e]azulen-7-ol, decahydro-1,1,7-trimethyl-4-methylene-, [1ar-(1a.alpha.,4a.alpha.,7.beta.,7a.bet

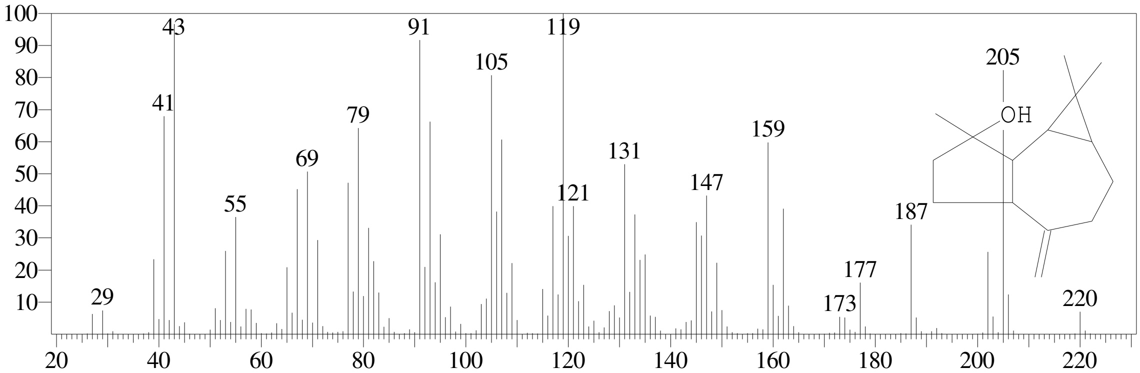

<< Target >>
Line#:23 R.Time:32.717(Scan#:3627) MassPeaks:54
RawMode:Averaged 32.708-32.725(3626-3628) BasePeak:43.00(22986)
BG Mode:None Group 1 - Event 1 Scan

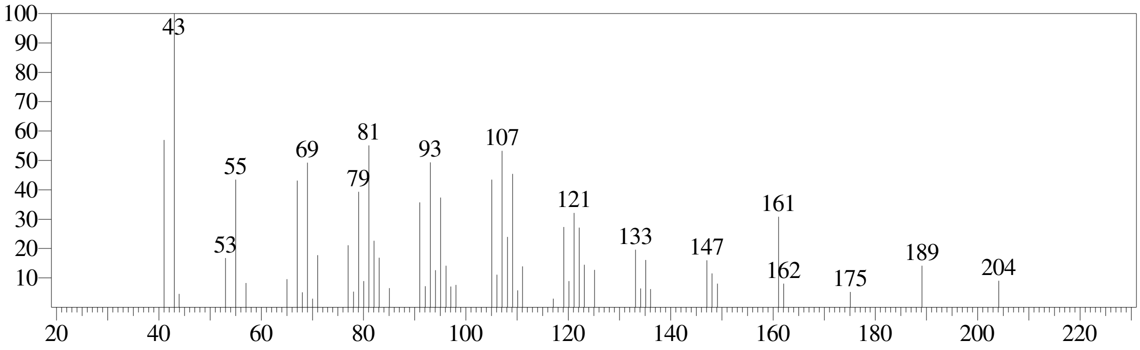

Hit#:1 Entry:83457 Library:NIST23-1.lib
SI:93 Formula:C15H26O CAS:51371-47-2 MolWeight:222 RetIndex:1588
CompName:Globulol $$ 1,1,4,7-Tetramethyldecahydro-1H-cyclopropa[e]azulen-4-ol, (1a.alpha.,4.alpha.,4a.alpha.,7.alpha

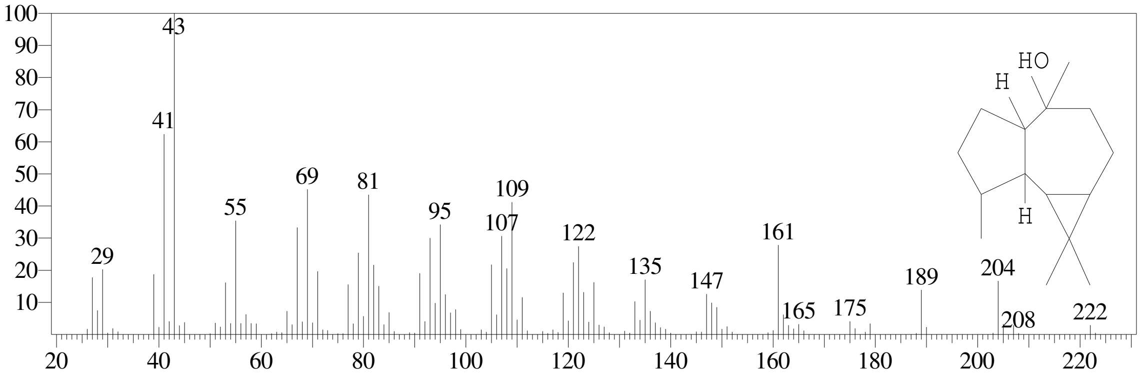

Hit#:2 Entry:28653 Library:NIST23s.lib
SI:92 Formula:C15H26O CAS:88728-58-9 MolWeight:222 RetIndex:1588
CompName:(1aR,4S,4aR,7R,7aS,7bS)-1,1,4,7-Tetramethyldecahydro-1H-cyclopropa[e]azulen-4-ol $$ Epiglobulol $$ epi

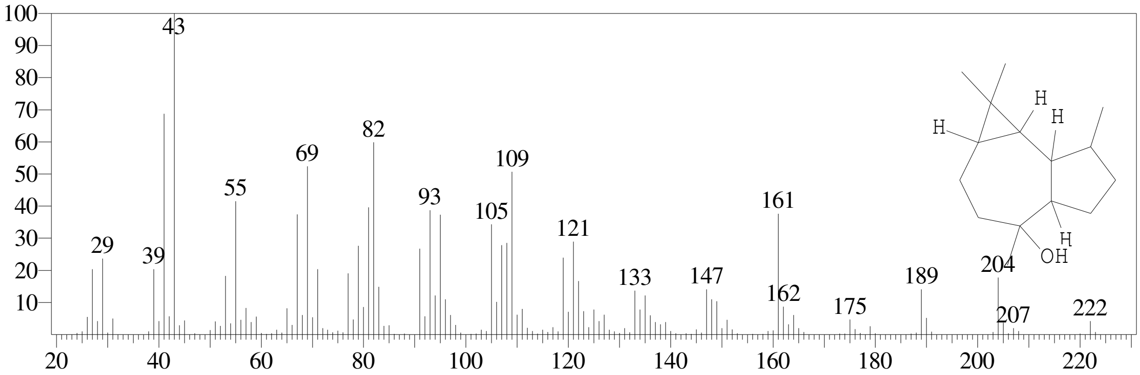

<< Target >>
Line#:23   R.Time:32.717(Scan#:3627) MassPeaks:54
RawMode:Averaged 32.708-32.725(3626-3628) BasePeak:43.00(22986)
BG Mode:None Group 1 - Event 1 Scan

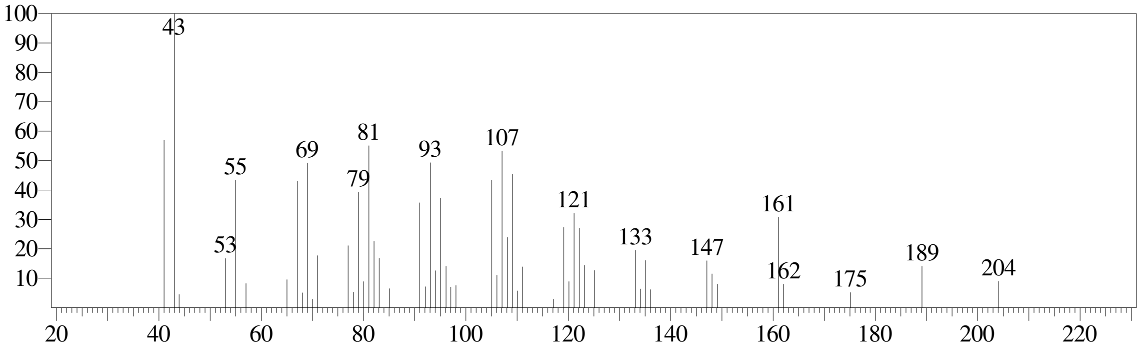

Hit#:3 Entry:28656 Library:NIST23s.lib
SI:91 Formula:C15H26O CAS:552-02-3 MolWeight:222 RetIndex:1588
CompName:1H-Cycloprop[e]azulen-4-ol, decahydro-1,1,4,7-tetramethyl-, [1aR-(1a.alpha.,4.beta.,4a.beta.,7.alpha.,7a.beta

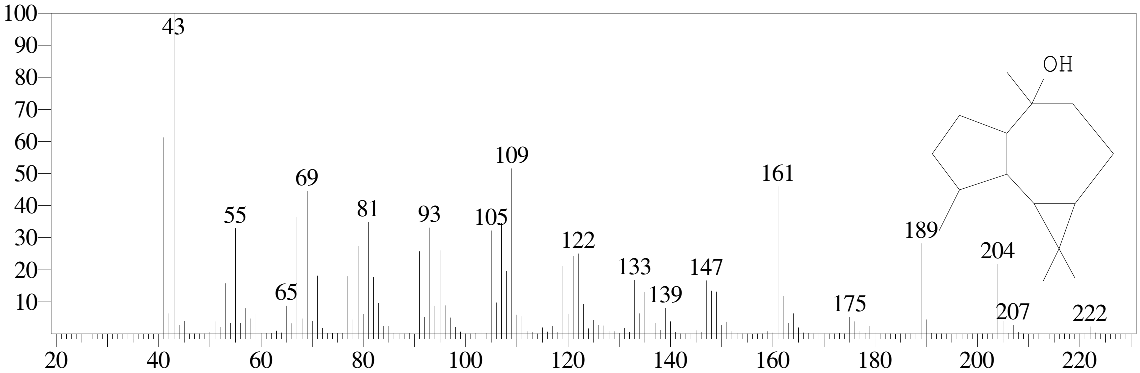

Hit#:4 Entry:28666 Library:NIST23s.lib
SI:91 Formula:C15H26O CAS:489-41-8 MolWeight:222 RetIndex:1588
CompName:(-)-Globulol $$ (1aR,4R,4aR,7R,7aS,7bS)-1,1,4,7-Tetramethyldecahydro-1H-cyclopropa[e]azulen-4-ol $$ Gl

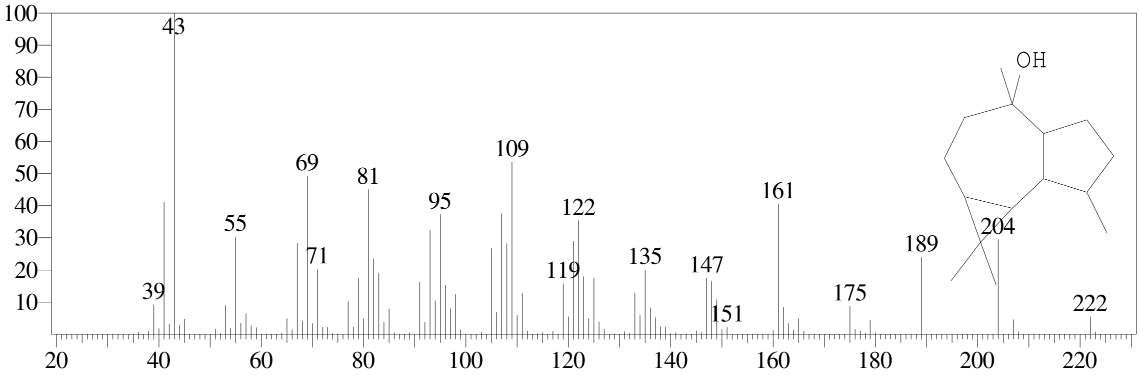

<< Target >>
Line#:23 R.Time:32.717(Scan#:3627) MassPeaks:54
RawMode:Averaged 32.708-32.725(3626-3628) BasePeak:43.00(22986)
BG Mode:None Group 1 - Event 1 Scan

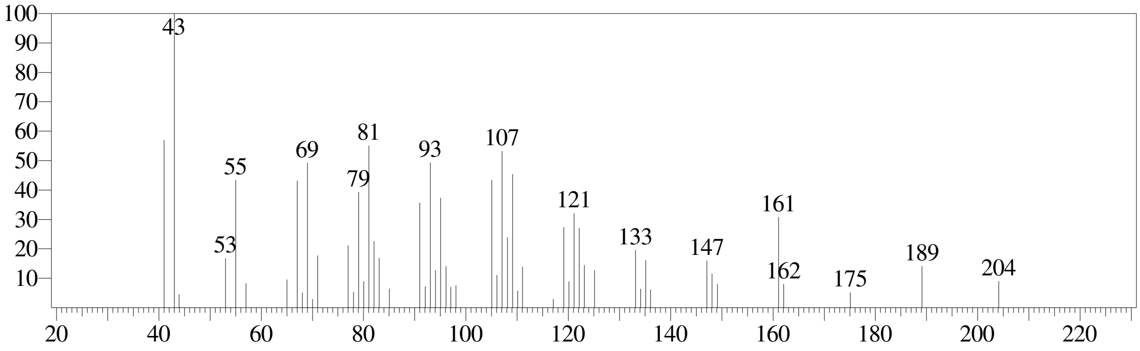

Hit#:5 Entry:28650 Library:NIST23s.lib
SI:91 Formula:C15H26O CAS:21698-41-9 MolWeight:222 RetIndex:1641
CompName:1,4-Dimethyl-7-(prop-1-en-2-yl)decahydroazulen-4-ol $$ Pogostole $$ trans-Guai-11-en-10-ol $$

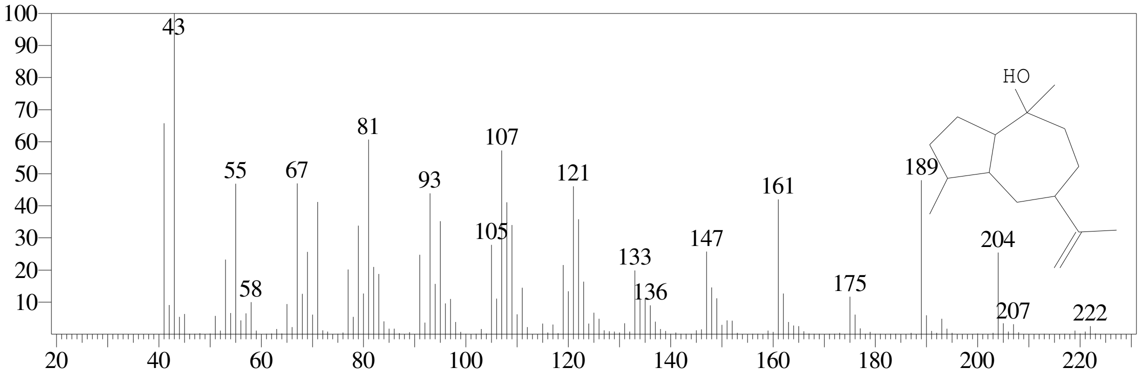

<< Target >>
Line#:24   R.Time:33.017(Scan#:3663) MassPeaks:41
RawMode:Averaged 33.008-33.025(3662-3664) BasePeak:43.00(9889)
BG Mode:Calc. from Peak Group 1 - Event 1 Scan

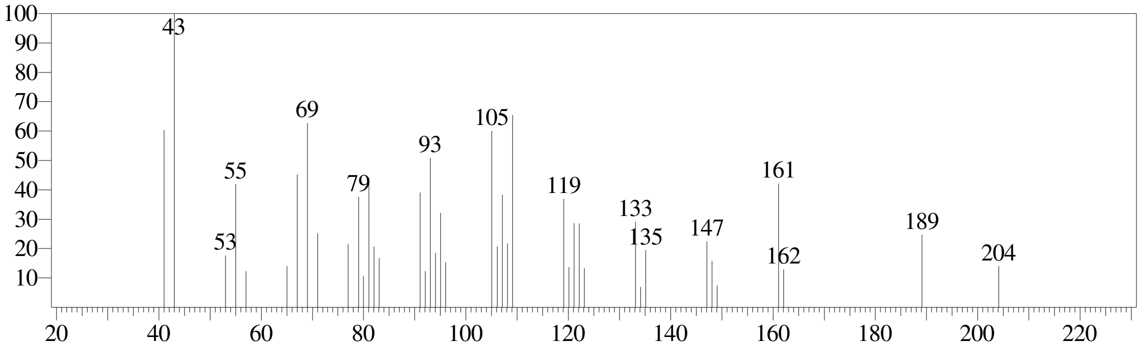

Hit#:1 Entry:28656 Library:NIST23s.lib
SI:91 Formula:C15H26O CAS:552-02-3 MolWeight:222 RetIndex:1588
CompName:1H-Cycloprop[e]azulen-4-ol, decahydro-1,1,4,7-tetramethyl-, [1aR-(1a.alpha.,4.beta.,4a.beta.,7.alpha.,7a.beta

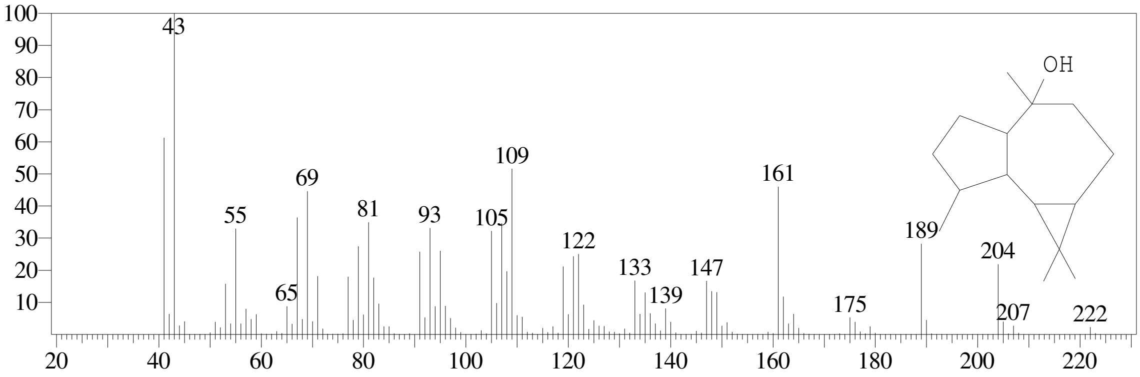

Hit#:2 Entry:83550 Library:NIST23-1.lib
SI:91 Formula:C15H26O CAS:88728-58-9 MolWeight:222 RetIndex:1588
CompName:(1aR,4S,4aR,7R,7aS,7bS)-1,1,4,7-Tetramethyldecahydro-1H-cyclopropa[e]azulen-4-ol $$ Epiglobulol $$ epi

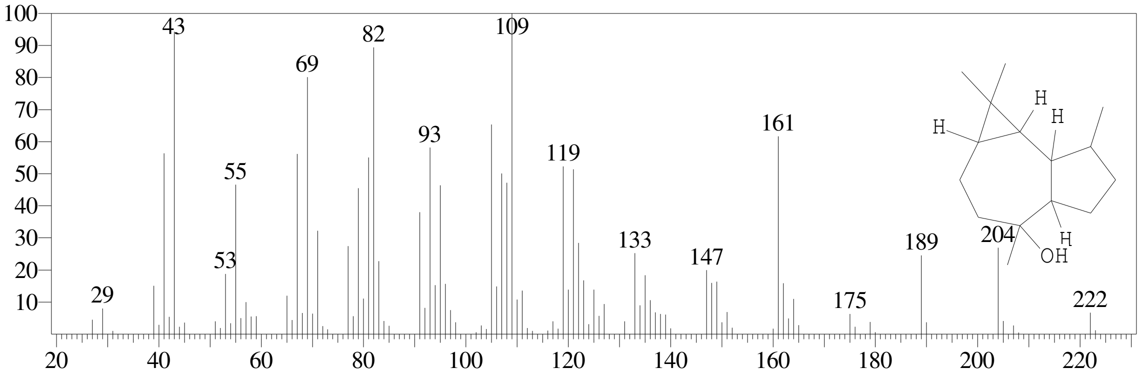

<< Target >>
Line#:24   R.Time:33.017(Scan#:3663) MassPeaks:41
RawMode:Averaged 33.008-33.025(3662-3664) BasePeak:43.00(9889)
BG Mode:Calc. from Peak Group 1 - Event 1 Scan

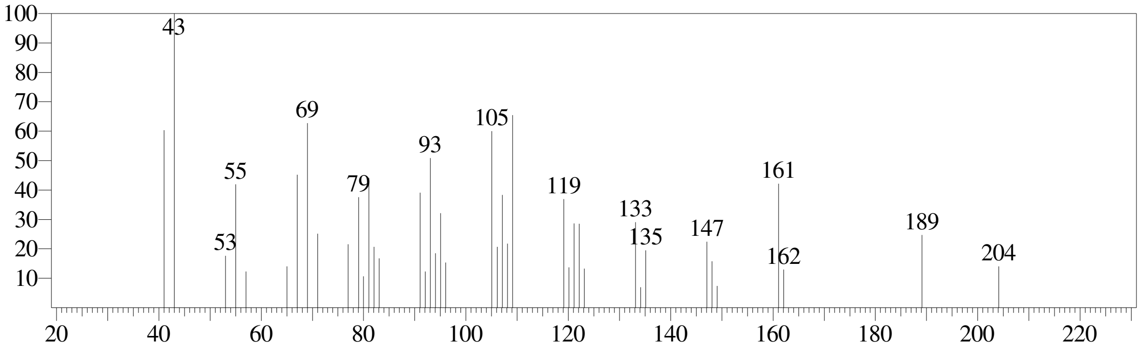

Hit#:3 Entry:28653 Library:NIST23s.lib
SI:91 Formula:C15H26O CAS:88728-58-9 MolWeight:222 RetIndex:1588
CompName:(1aR,4S,4aR,7R,7aS,7bS)-1,1,4,7-Tetramethyldecahydro-1H-cyclopropa[e]azulen-4-ol $$ Epiglobulol $$ epi

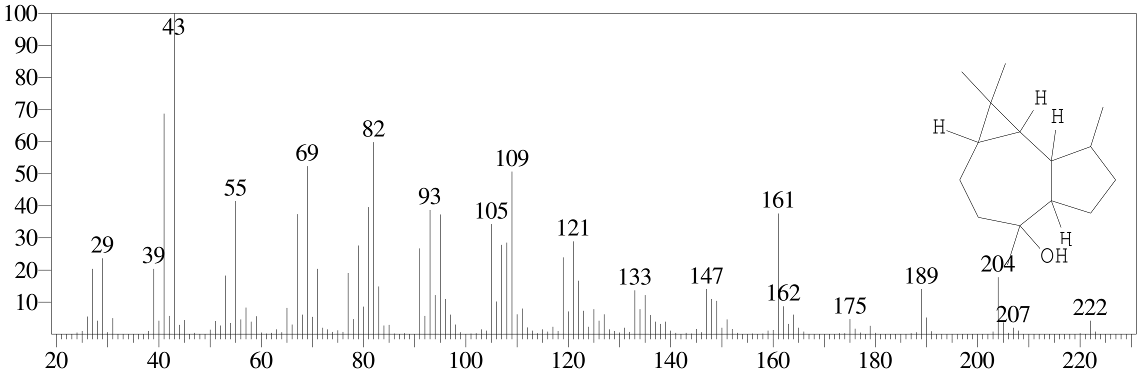

Hit#:4 Entry:83549 Library:NIST23-1.lib
SI:90 Formula:C15H26O CAS:552-02-3 MolWeight:222 RetIndex:1588
CompName:1H-Cycloprop[e]azulen-4-ol, decahydro-1,1,4,7-tetramethyl-, [1aR-(1a.alpha.,4.beta.,4a.beta.,7.alpha.,7a.beta

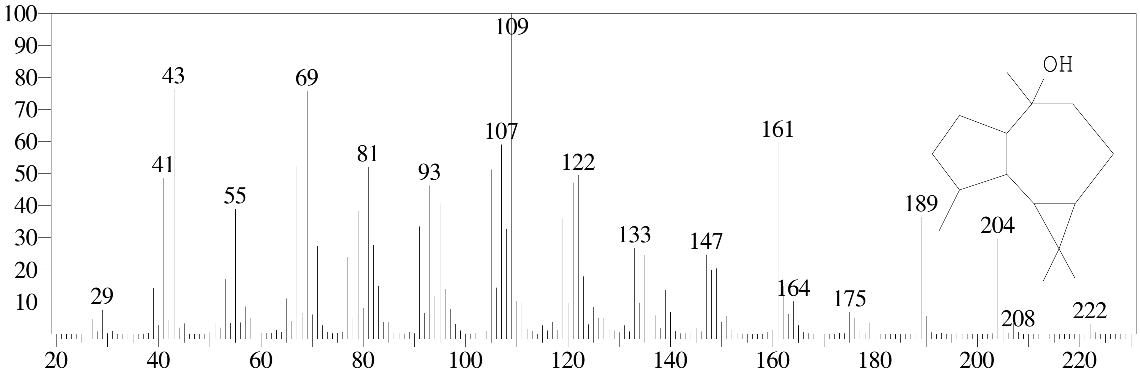

<< Target >>
Line#:24   R.Time:33.017(Scan#:3663) MassPeaks:41
RawMode:Averaged 33.008-33.025(3662-3664) BasePeak:43.00(9889)
BG Mode:Calc. from Peak Group 1 - Event 1 Scan

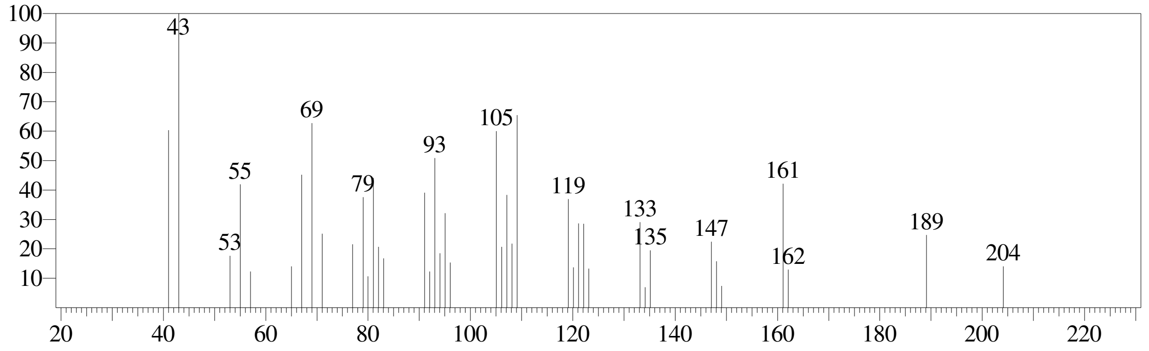

Hit#:5 Entry:28629 Library:NIST23s.lib
SI:90 Formula:C15H26O CAS:552-02-3 MolWeight:222 RetIndex:1588
CompName:1H-Cycloprop[e]azulen-4-ol, decahydro-1,1,4,7-tetramethyl-, [1aR-(1a.alpha.,4.beta.,4a.beta.,7.alpha.,7a.beta

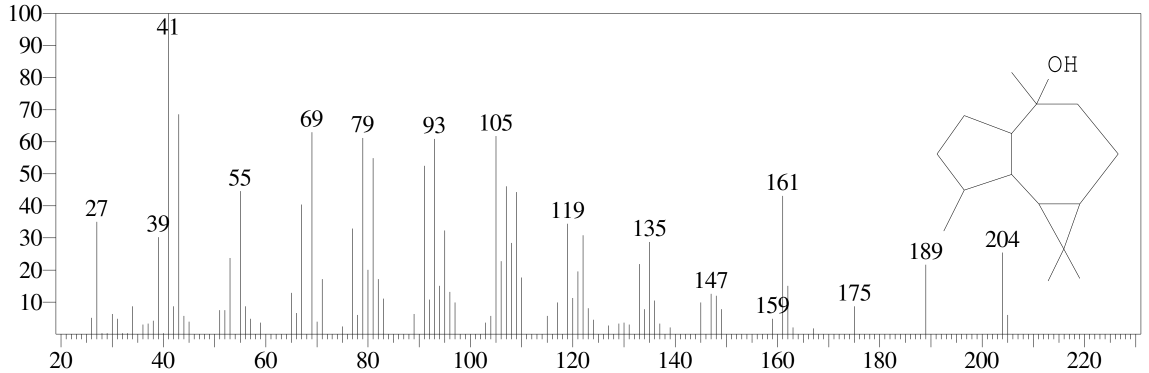

<< Target >>
Line#:25 R.Time:33.108(Scan#:3674) MassPeaks:24
RawMode:Averaged 33.100-33.117(3673-3675) BasePeak:107.10(7670)
BG Mode:Calc. from Peak Group 1 - Event 1 Scan

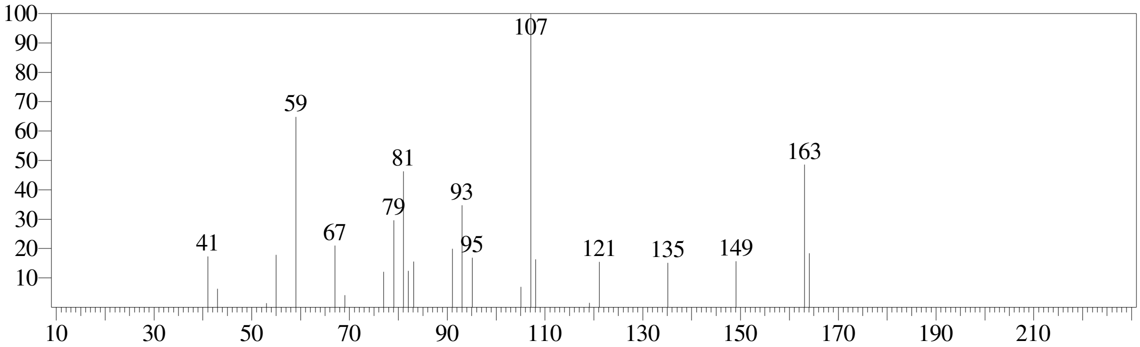

Hit#:1 Entry:83547 Library:NIST23-1.lib
SI:86 Formula:C15H26O CAS:220766-71-2 MolWeight:222 RetIndex:1595
CompName:2-((3R,3aR,3bS,4R,7R,7aS)-3,7-Dimethyloctahydro-1H-cyclopenta[1,3]cyclopropa[1,2]benzen-4-yl)propan-

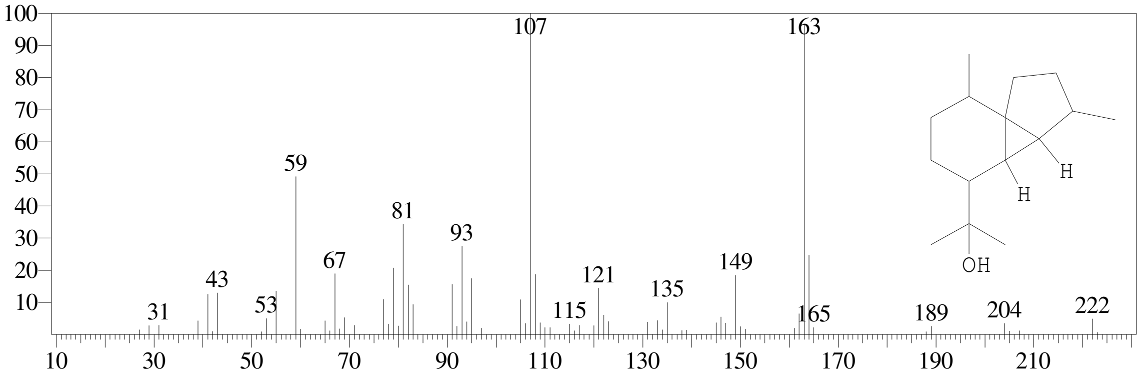

Hit#:2 Entry:28774 Library:NIST23s.lib
SI:85 Formula:C15H26O CAS:220766-71-2 MolWeight:222 RetIndex:1595
CompName:2-((3R,3aR,3bS,4R,7R,7aS)-3,7-Dimethyloctahydro-1H-cyclopenta[1,3]cyclopropa[1,2]benzen-4-yl)propan-

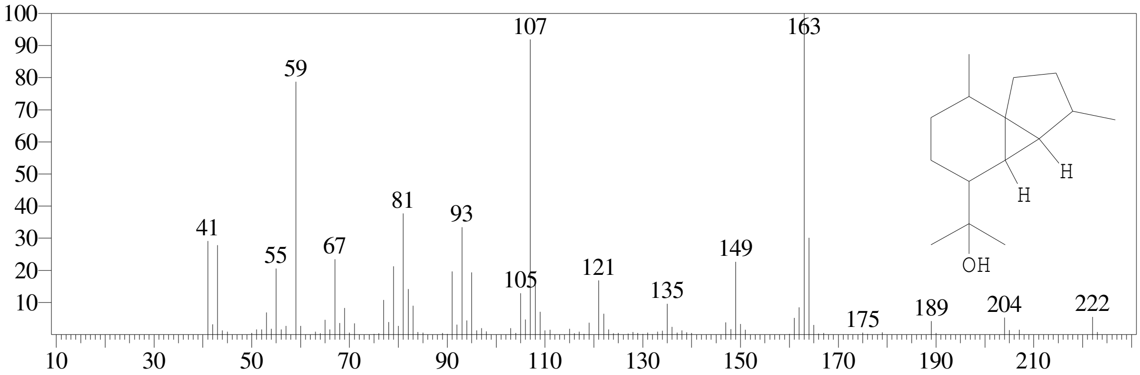

<< Target >>
Line#:25   R.Time:33.108(Scan#:3674) MassPeaks:24
RawMode:Averaged 33.100-33.117(3673-3675) BasePeak:107.10(7670)
BG Mode:Calc. from Peak Group 1 - Event 1 Scan

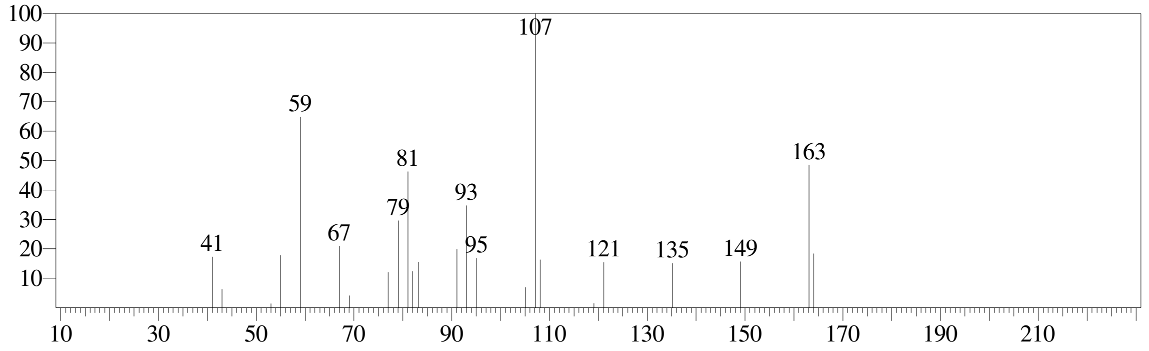

Hit#:3 Entry:83484 Library:NIST23-1.lib
SI:76 Formula:C15H26O CAS:60441-29-4 MolWeight:222 RetIndex:1600
CompName:2,7-Cyclodecadiene-1-methanol, .alpha.,.alpha.,4,8-tetramethyl- $$ .alpha.,.alpha.,4,8-Tetramethyl-2,7-cyclod

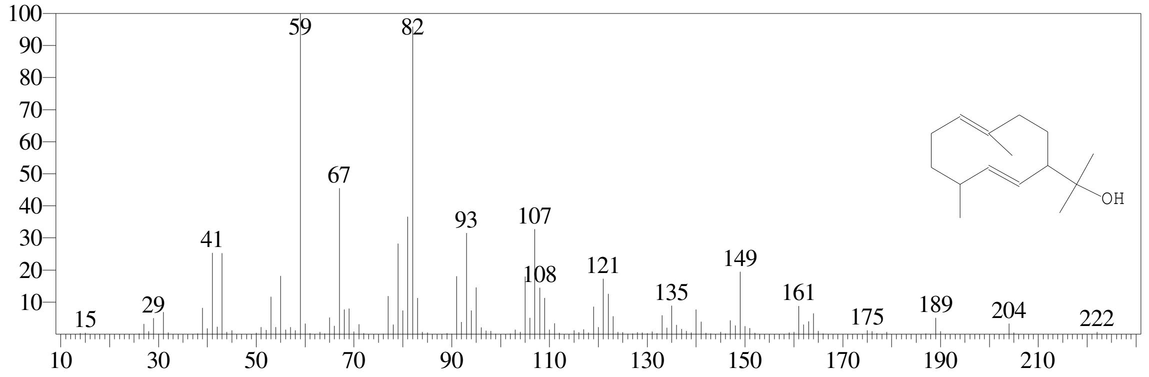

Hit#:4 Entry:81843 Library:NIST23-1.lib
SI:75 Formula:C13H19NS CAS:136860-49-6 MolWeight:221 RetIndex:1731
CompName:Adamantane, 1-isothiocyanato-3,5-dimethyl- $$ 1-Isothiocyanato-3,5-dimethyladamantane # $$

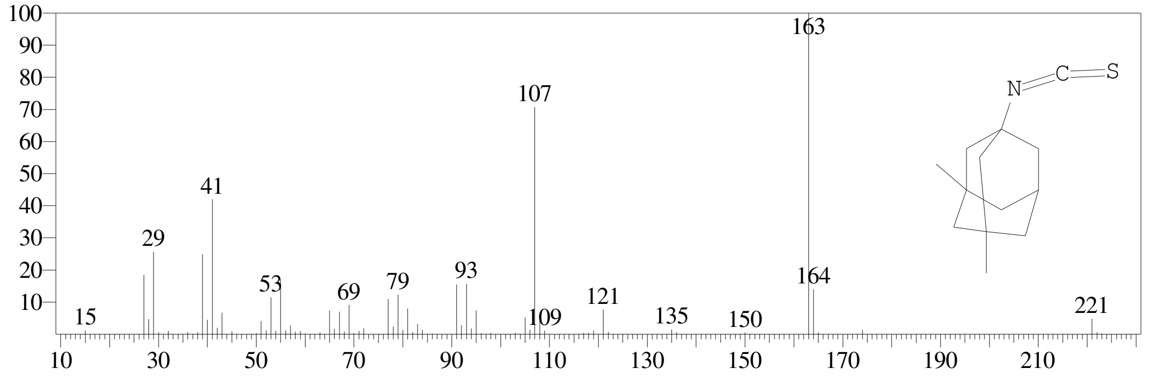

<< Target >>
Line#:25   R.Time:33.108(Scan#:3674) MassPeaks:24
RawMode:Averaged 33.100-33.117(3673-3675) BasePeak:107.10(7670)
BG Mode:Calc. from Peak Group 1 - Event 1 Scan

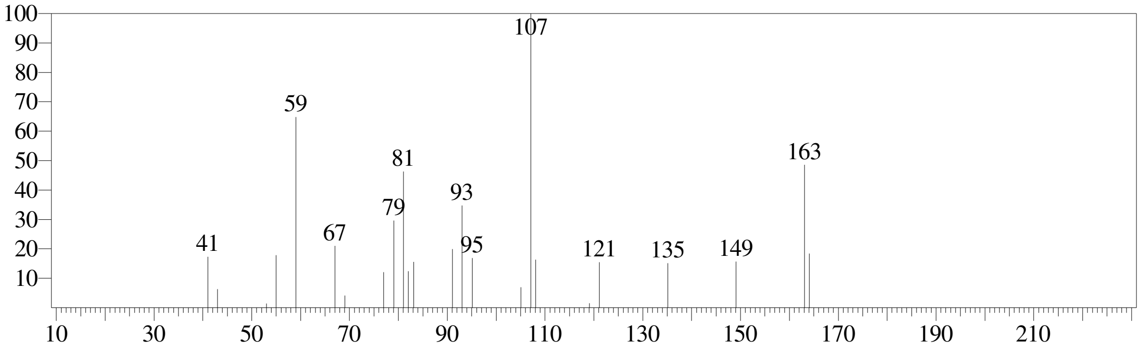

Hit#:5 Entry:83490 Library:NIST23-1.lib
SI:75 Formula:C15H26O CAS:63891-61-2 MolWeight:222 RetIndex:1602
CompName:2-Naphthalenemethanol, 2,3,4,4a,5,6,7,8-octahydro-.alpha.,.alpha.,4a,8-tetramethyl-, [2R-(2.alpha.,4a.beta.,8.

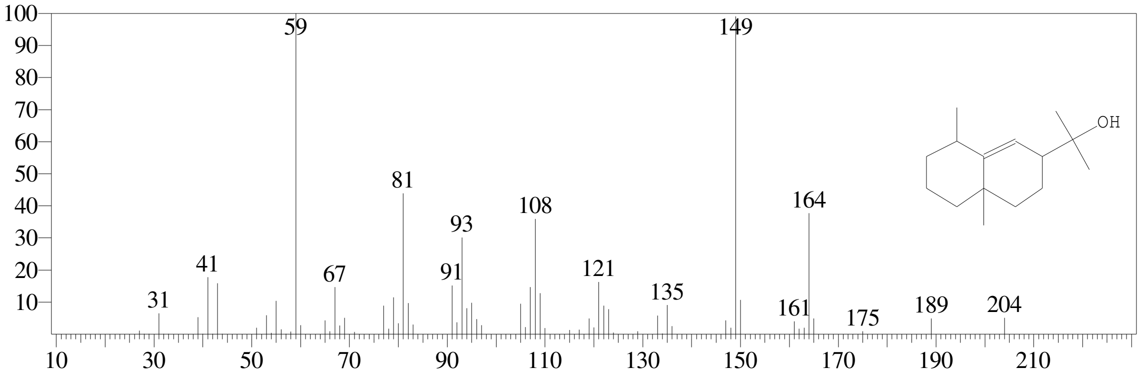

<< Target >>
Line#:26 R.Time:33.417(Scan#:3711) MassPeaks:31
RawMode:Averaged 33.408-33.425(3710-3712) BasePeak:59.00(12271)
BG Mode:Calc. from Peak Group 1 - Event 1 Scan

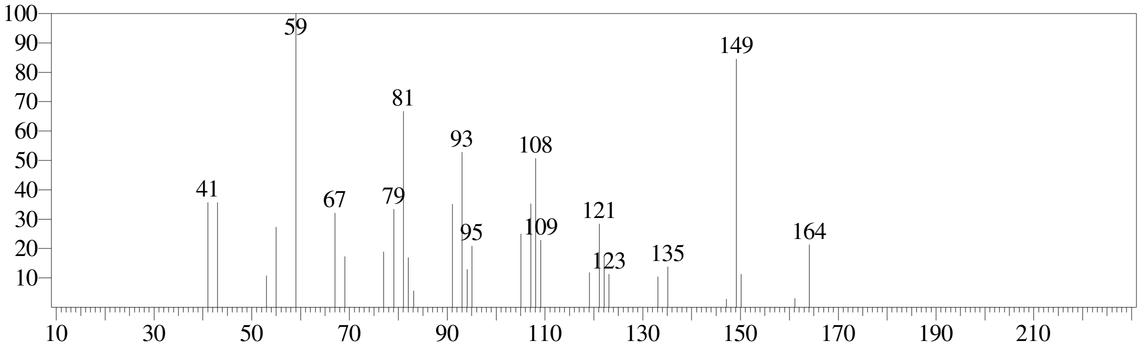

Hit#:1 Entry:28743 Library:NIST23s.lib
SI:88 Formula:C15H26O CAS:63891-61-2 MolWeight:222 RetIndex:1602
CompName:2-Naphthalenemethanol, 2,3,4,4a,5,6,7,8-octahydro-.alpha.,.alpha.,4a,8-tetramethyl-, [2R-(2.alpha.,4a.beta.,8.

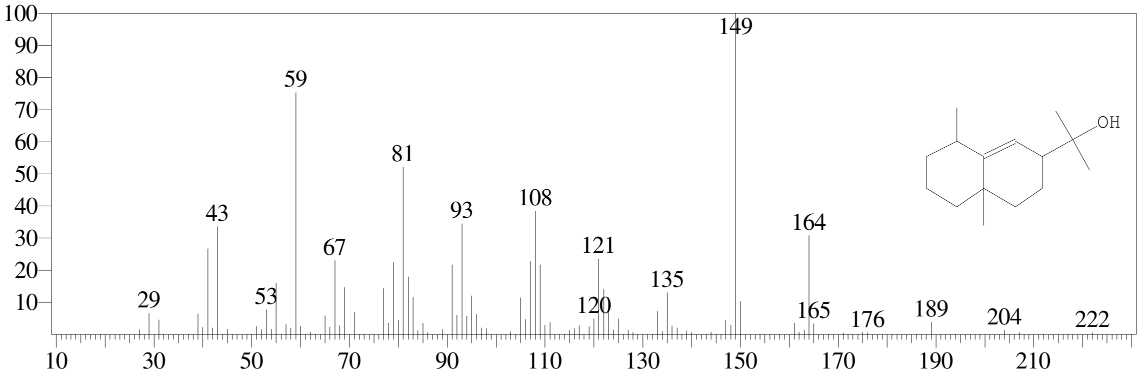

Hit#:2 Entry:28744 Library:NIST23s.lib
SI:87 Formula:C15H26O CAS:63891-61-2 MolWeight:222 RetIndex:1602
CompName:2-Naphthalenemethanol, 2,3,4,4a,5,6,7,8-octahydro-.alpha.,.alpha.,4a,8-tetramethyl-, [2R-(2.alpha.,4a.beta.,8.

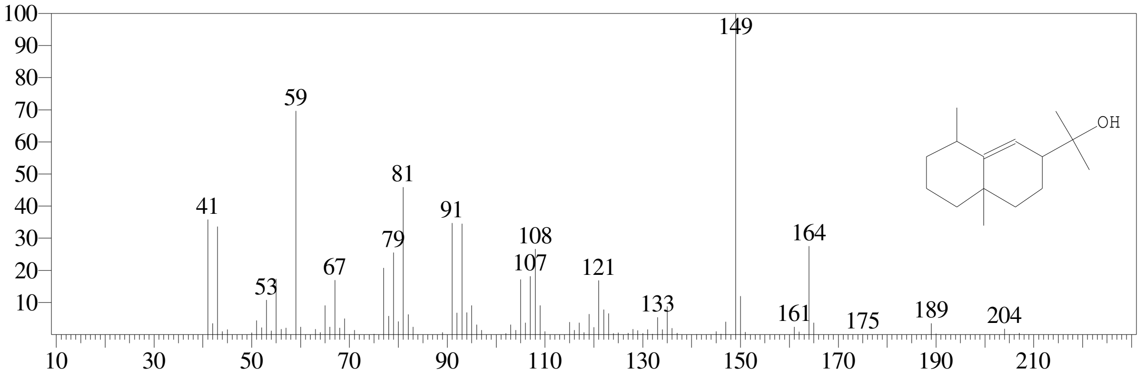

<< Target >>
Line#:26 R.Time:33.417(Scan#:3711) MassPeaks:31
RawMode:Averaged 33.408-33.425(3710-3712) BasePeak:59.00(12271)
BG Mode:Calc. from Peak Group 1 - Event 1 Scan

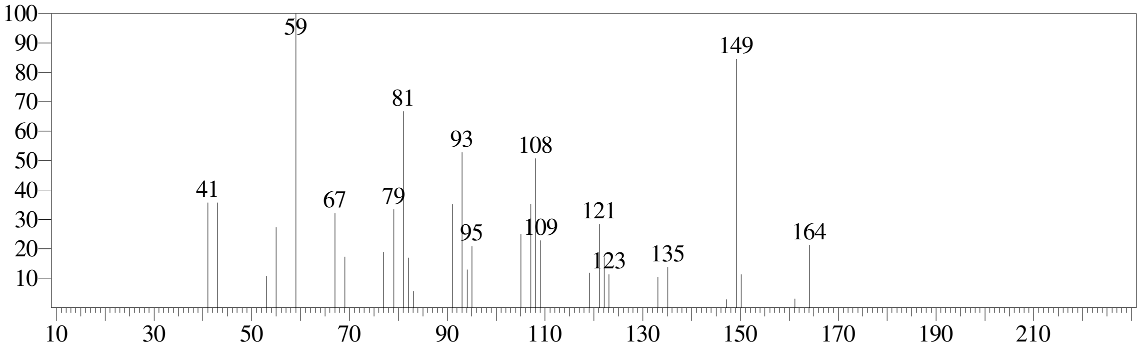

Hit#:3 Entry:83491 Library:NIST23-1.lib
SI:87 Formula:C15H26O CAS:94373-04-3 MolWeight:222 RetIndex:1644
CompName:2-(4a,8-Dimethyl-2,3,4,5,6,8a-hexahydro-1H-naphthalen-2-yl)propan-2-ol

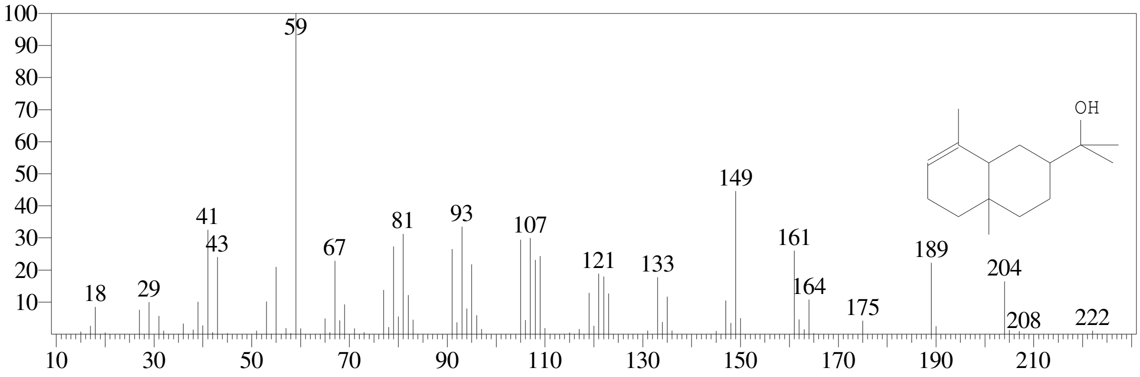

Hit#:4 Entry:83490 Library:NIST23-1.lib
SI:85 Formula:C15H26O CAS:63891-61-2 MolWeight:222 RetIndex:1602
CompName:2-Naphthalenemethanol, 2,3,4,4a,5,6,7,8-octahydro-.alpha.,.alpha.,4a,8-tetramethyl-, [2R-(2.alpha.,4a.beta.,8.

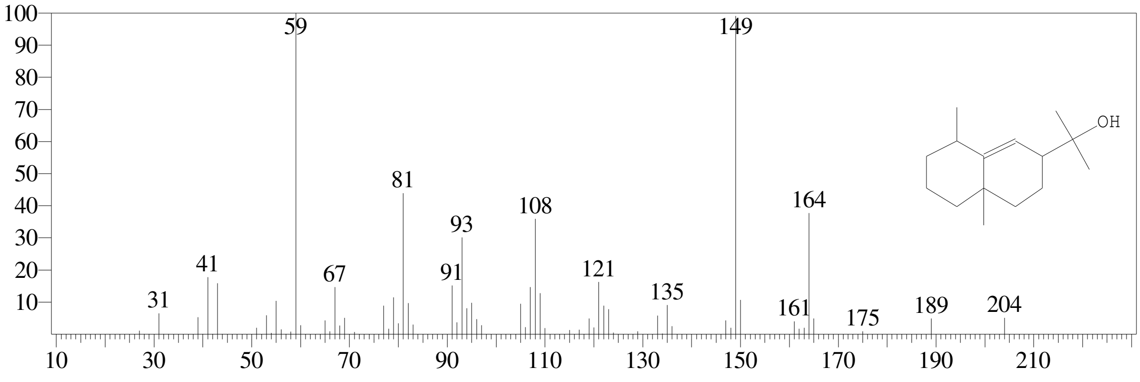

<< Target >>
Line#:26   R.Time:33.417(Scan#:3711) MassPeaks:31
RawMode:Averaged 33.408-33.425(3710-3712) BasePeak:59.00(12271)
BG Mode:Calc. from Peak Group 1 - Event 1 Scan

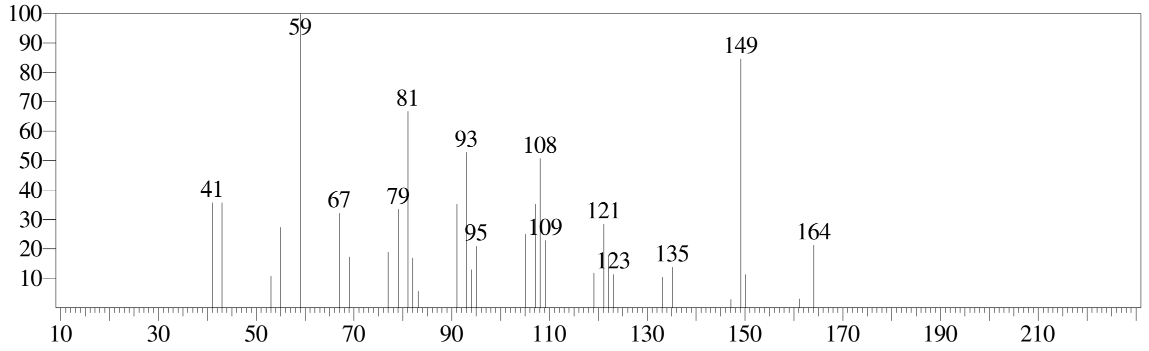

Hit#:5 Entry:28688 Library:NIST23s.lib
SI:85 Formula:C15H26O CAS:473-15-4 MolWeight:222 RetIndex:1652
CompName:2-Naphthalenemethanol, decahydro-.alpha.,.alpha.,4a-trimethyl-8-methylene-, [2R-(2.alpha.,4a.alpha.,8a.beta.

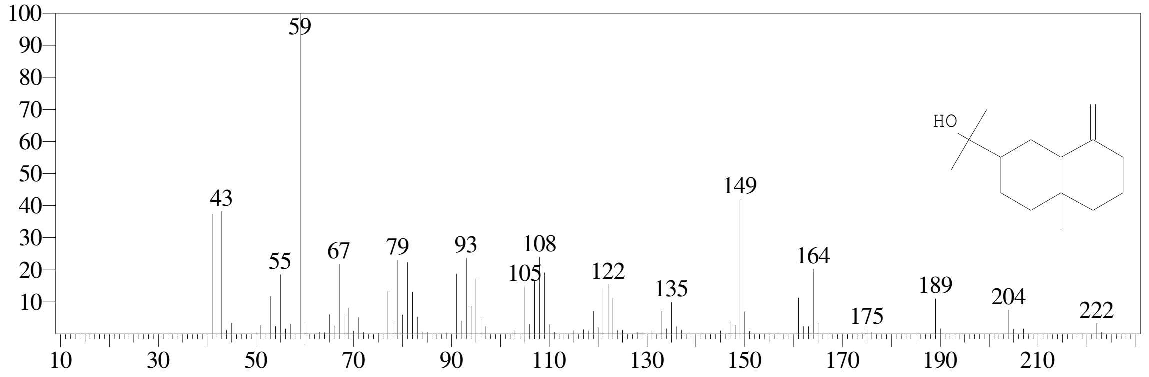

<< Target >>
Line#:27 R.Time:34.192(Scan#:3804) MassPeaks:27
RawMode:Averaged 34.183-34.200(3803-3805) BasePeak:59.00(12384)
BG Mode:Calc. from Peak Group 1 - Event 1 Scan

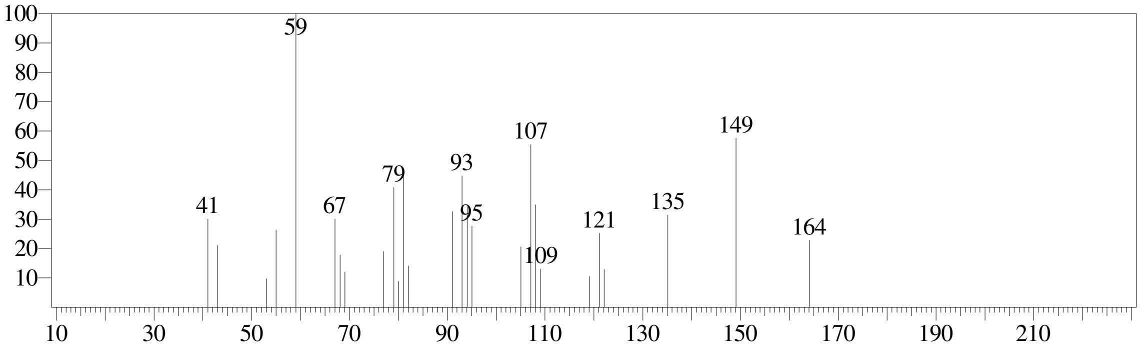

Hit#:1 Entry:83491 Library:NIST23-1.lib
SI:83 Formula:C15H26O CAS:94373-04-3 MolWeight:222 RetIndex:1644
CompName:2-(4a,8-Dimethyl-2,3,4,5,6,8a-hexahydro-1H-naphthalen-2-yl)propan-2-ol

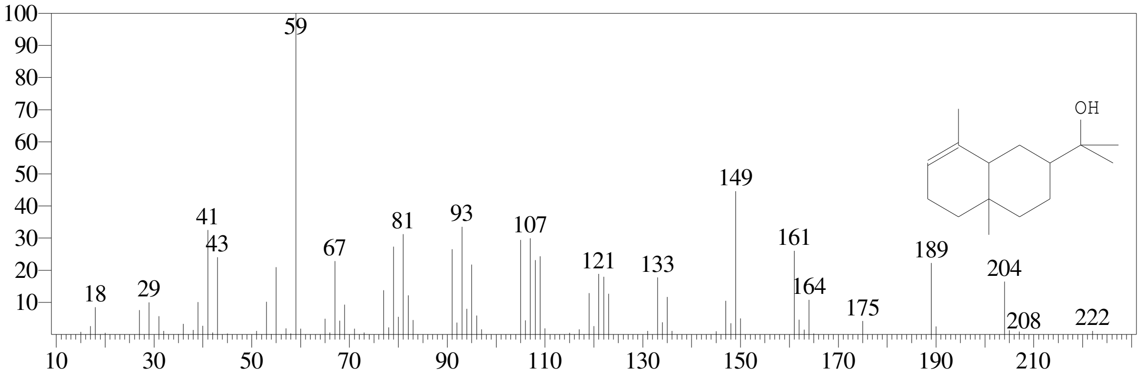

Hit#:2 Entry:28744 Library:NIST23s.lib
SI:83 Formula:C15H26O CAS:63891-61-2 MolWeight:222 RetIndex:1602
CompName:2-Naphthalenemethanol, 2,3,4,4a,5,6,7,8-octahydro-.alpha.,.alpha.,4a,8-tetramethyl-, [2R-(2.alpha.,4a.beta.,8.

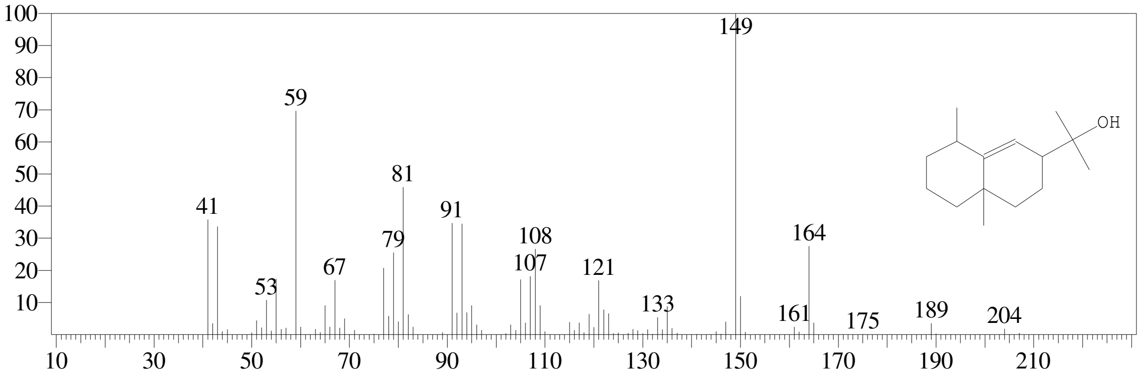

<< Target >>
Line#:27   R.Time:34.192(Scan#:3804) MassPeaks:27
RawMode:Averaged 34.183-34.200(3803-3805) BasePeak:59.00(12384)
BG Mode:Calc. from Peak Group 1 - Event 1 Scan

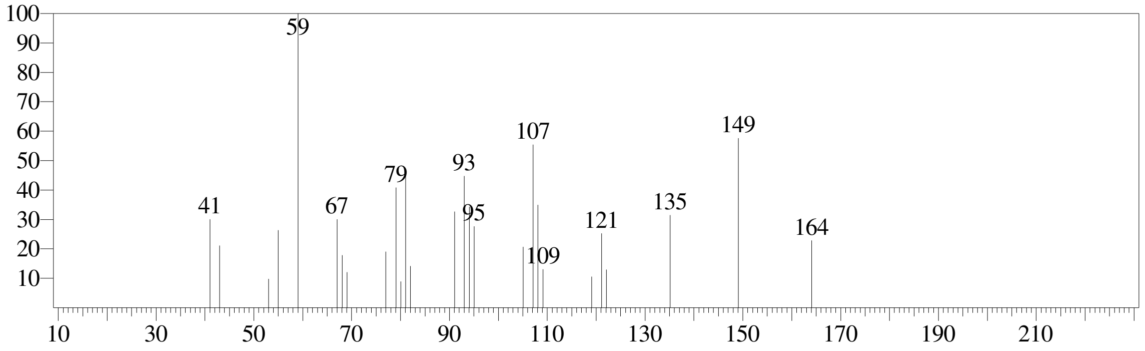

Hit#:3 Entry:83484 Library:NIST23-1.lib
SI:82 Formula:C15H26O CAS:60441-29-4 MolWeight:222 RetIndex:1600
CompName:2,7-Cyclodecadiene-1-methanol, .alpha.,.alpha.,4,8-tetramethyl- $$ .alpha.,.alpha.,4,8-Tetramethyl-2,7-cyclod

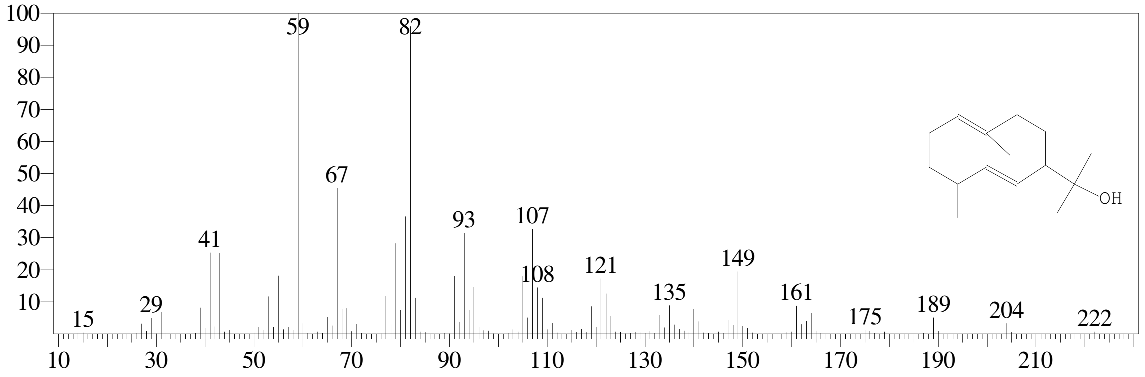

Hit#:4 Entry:28688 Library:NIST23s.lib
SI:82 Formula:C15H26O CAS:473-15-4 MolWeight:222 RetIndex:1652
CompName:2-Naphthalenemethanol, decahydro-.alpha.,.alpha.,4a-trimethyl-8-methylene-, [2R-(2.alpha.,4a.alpha.,8a.beta.

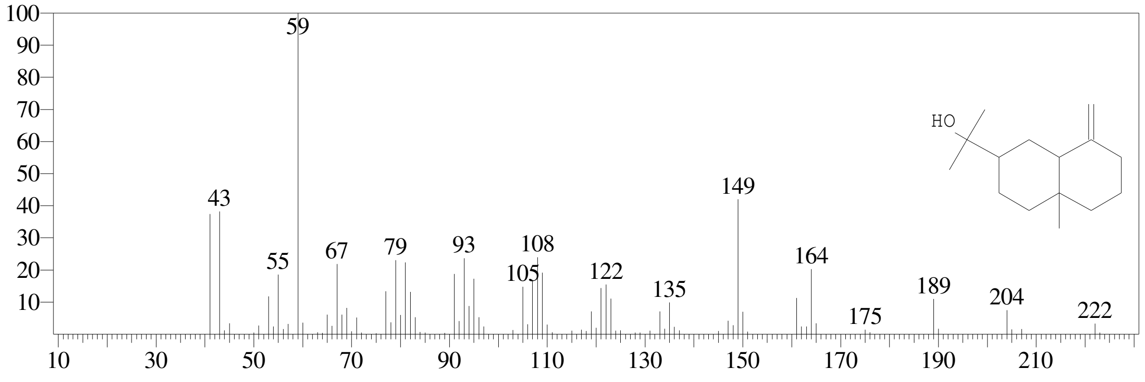

<< Target >>
Line#:27   R.Time:34.192(Scan#:3804) MassPeaks:27
RawMode:Averaged 34.183-34.200(3803-3805) BasePeak:59.00(12384)
BG Mode:Calc. from Peak Group 1 - Event 1 Scan

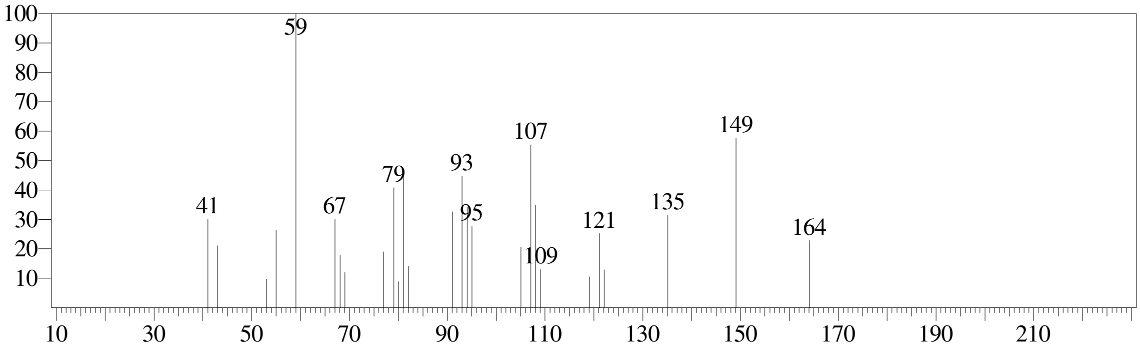

Hit#:5 Entry:83490 Library:NIST23-1.lib
SI:82 Formula:C15H26O CAS:63891-61-2 MolWeight:222 RetIndex:1602
CompName:2-Naphthalenemethanol, 2,3,4,4a,5,6,7,8-octahydro-.alpha.,.alpha.,4a,8-tetramethyl-, [2R-(2.alpha.,4a.beta.,8.

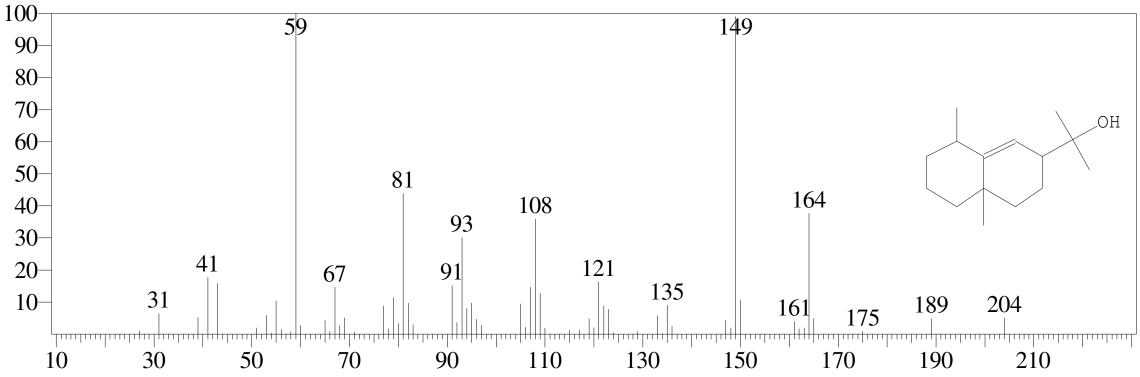

<< Target >>
Line#:28 R.Time:34.400(Scan#:3829) MassPeaks:41
RawMode:Averaged 34.392-34.408(3828-3830) BasePeak:119.10(10875)
BG Mode:Calc. from Peak Group 1 - Event 1 Scan

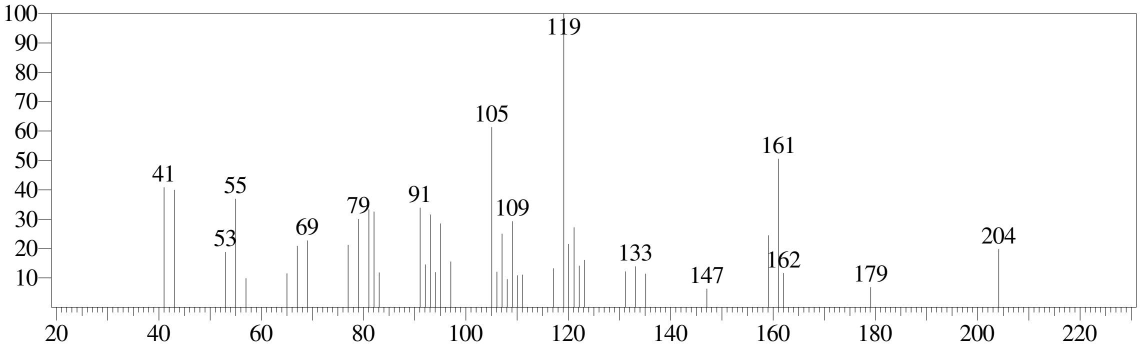

Hit#:1 Entry:28736 Library:NIST23s.lib
SI:88 Formula:C15H26O CAS:19912-67-5 MolWeight:222 RetIndex:1629
CompName:4a(2H)-Naphthalenol, 1,3,4,5,6,8a-hexahydro-4,7-dimethyl-1-(1-methylethyl)-, (1S,4R,4aS,8aR)- $$ Epicube

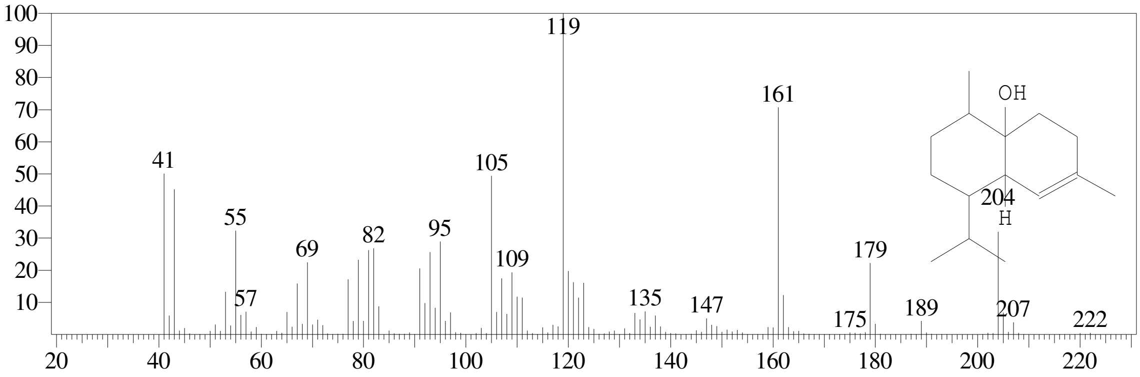

Hit#:2 Entry:83573 Library:NIST23-1.lib
SI:88 Formula:C15H26O CAS:19912-67-5 MolWeight:222 RetIndex:1629
CompName:4a(2H)-Naphthalenol, 1,3,4,5,6,8a-hexahydro-4,7-dimethyl-1-(1-methylethyl)-, (1S,4R,4aS,8aR)- $$ Epicube

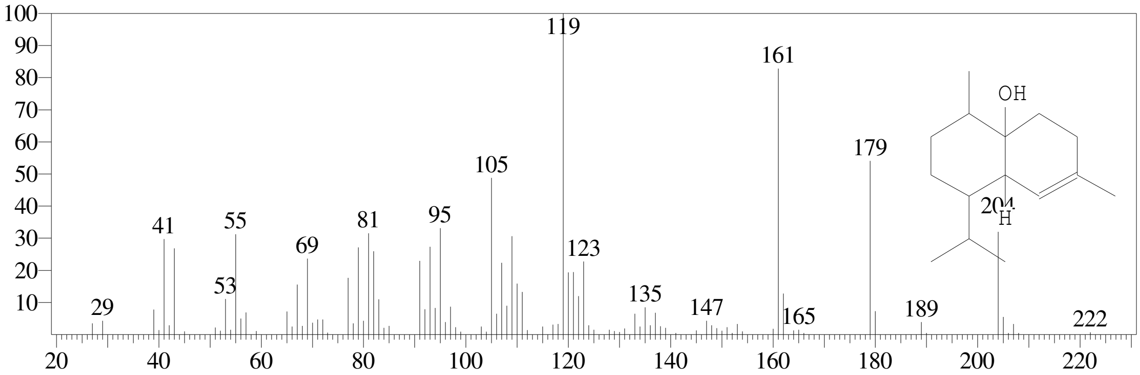

<< Target >>
Line#:28   R.Time:34.400(Scan#:3829) MassPeaks:41
RawMode:Averaged 34.392-34.408(3828-3830) BasePeak:119.10(10875)
BG Mode:Calc. from Peak Group 1 - Event 1 Scan

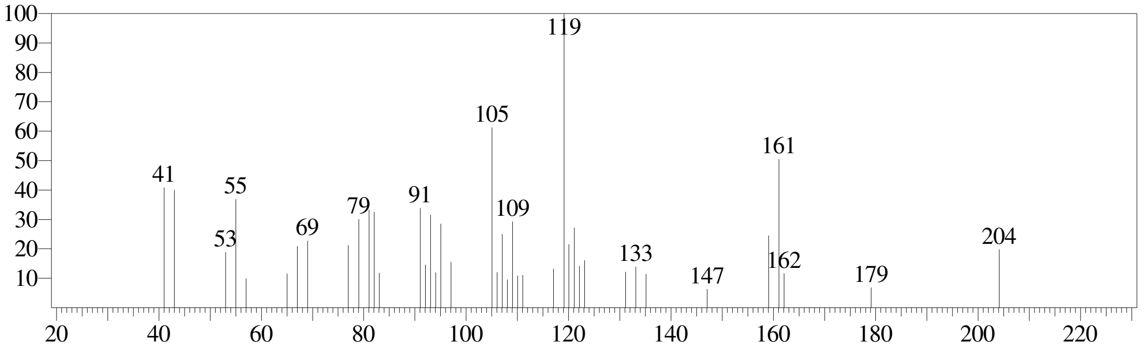

Hit#:3 Entry:28766 Library:NIST23s.lib
SI:87 Formula:C15H26O CAS:73365-77-2 MolWeight:222 RetIndex:1630
CompName:4a(2H)-Naphthalenol, 1,3,4,5,6,8a-hexahydro-4,7-dimethyl-1-(1-methylethyl)-, (1S,4S,4aS,8aR)- $$ Di-epi-1

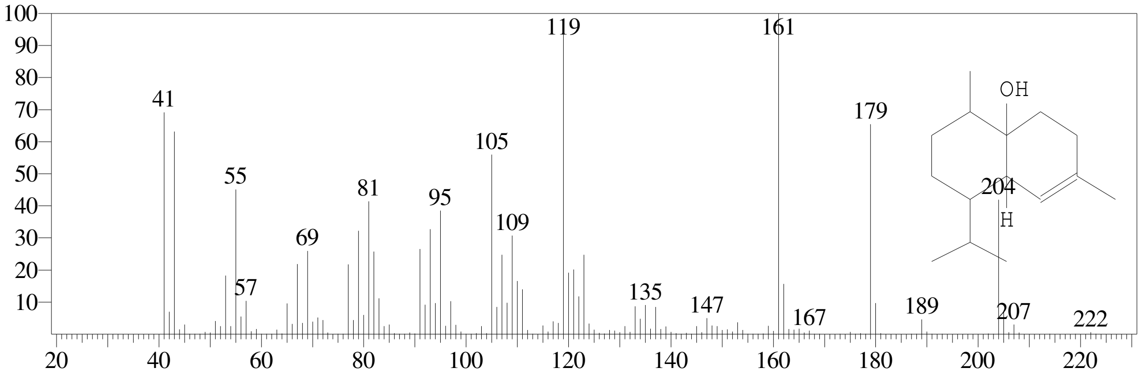

Hit#:4 Entry:28764 Library:NIST23s.lib
SI:85 Formula:C15H26O CAS:23445-02-5 MolWeight:222 RetIndex:1562
CompName:(3S,3aR,3bR,4S,7R,7aR)-4-Isopropyl-3,7-dimethyloctahydro-1H-cyclopenta[1,3]cyclopropa[1,2]benzen-3-ol

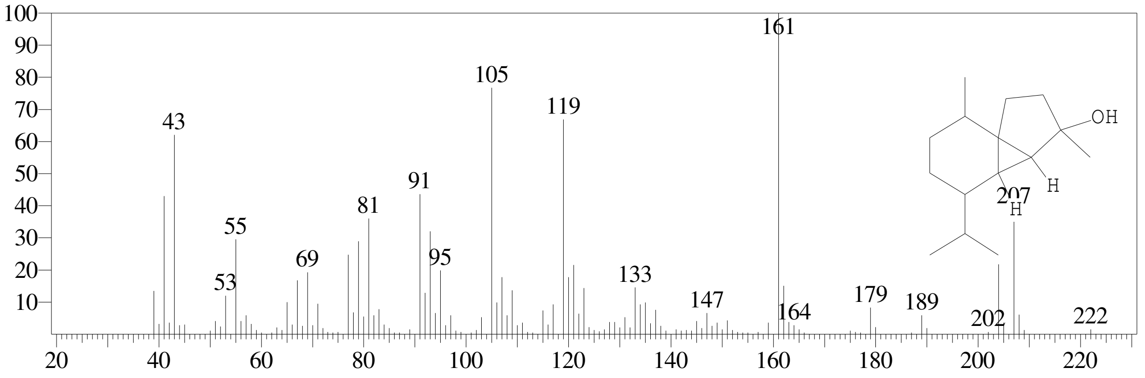

<< Target >>
Line#:28   R.Time:34.400(Scan#:3829) MassPeaks:41
RawMode:Averaged 34.392-34.408(3828-3830) BasePeak:119.10(10875)
BG Mode:Calc. from Peak Group 1 - Event 1 Scan

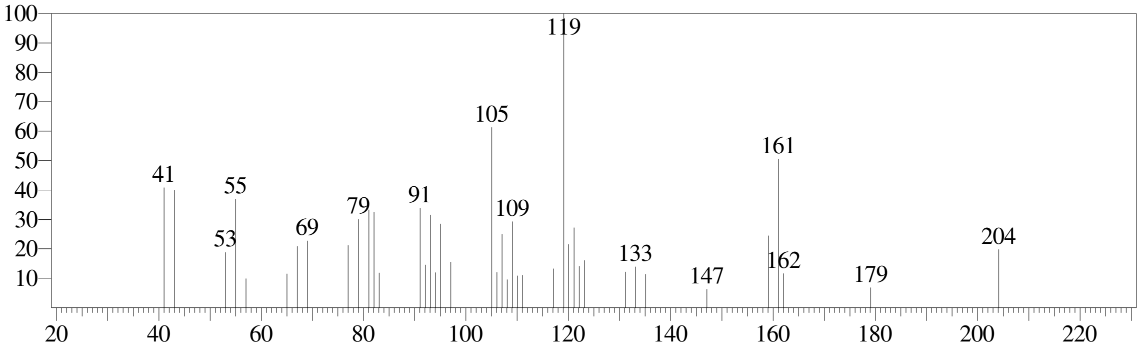

Hit#:5 Entry:28762 Library:NIST23s.lib
SI:84 Formula:C15H26O CAS:38230-60-3 MolWeight:222 RetIndex:1562
CompName:(3R,3aR,3bR,4S,7R,7aR)-4-Isopropyl-3,7-dimethyloctahydro-1H-cyclopenta[1,3]cyclopropa[1,2]benzen-3-ol

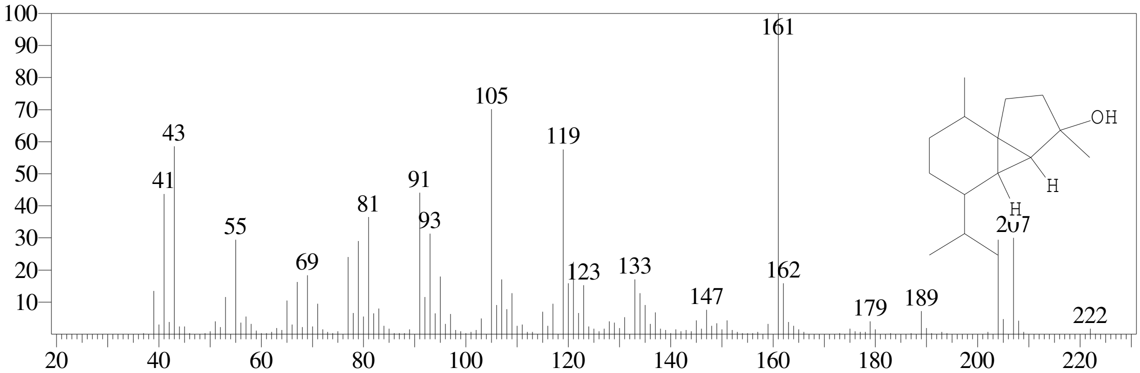

<< Target >>
Line#:29 R.Time:34.917(Scan#:3891) MassPeaks:33
RawMode:Averaged 34.908-34.925(3890-3892) BasePeak:95.05(9354)
BG Mode:Calc. from Peak Group 1 - Event 1 Scan

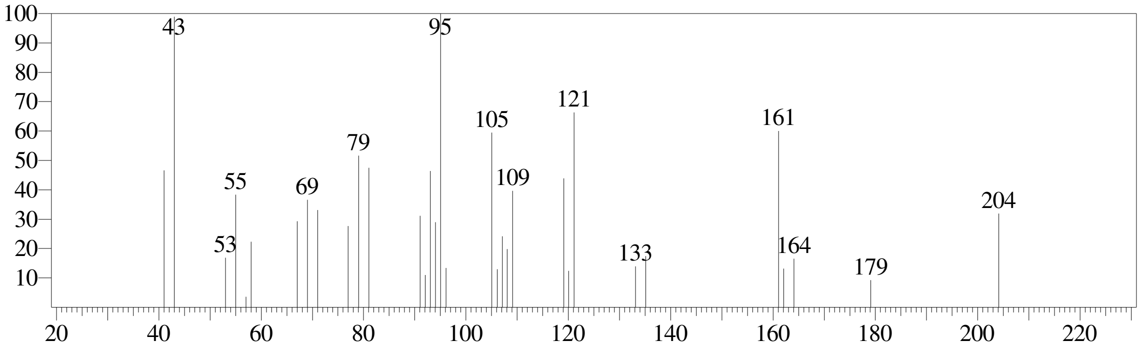

Hit#:1 Entry:83541 Library:NIST23-1.lib
SI:89 Formula:C15H26O CAS:19912-62-0 MolWeight:222 RetIndex:1635
CompName:.tau.-Muurolol $$ 4-Isopropyl-1,6-dimethyl-1,2,3,4,4a,7,8,8a-octahydro-1-naphthalenol-, [1S-(1.alpha.,4.alpha

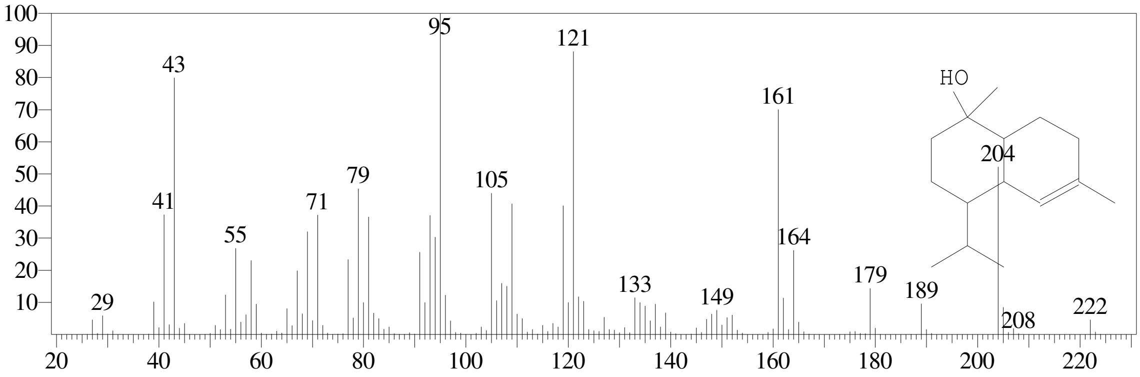

Hit#:2 Entry:28752 Library:NIST23s.lib
SI:88 Formula:C15H26O CAS:5937-11-1 MolWeight:222 RetIndex:1637
CompName:.tau.-Cadinol $$ 4-Isopropyl-1,6-dimethyl-1,2,3,4,4a,7,8,8a-octahydro-1-naphthalenol-, (1S-(1alpha,4alpha,4a

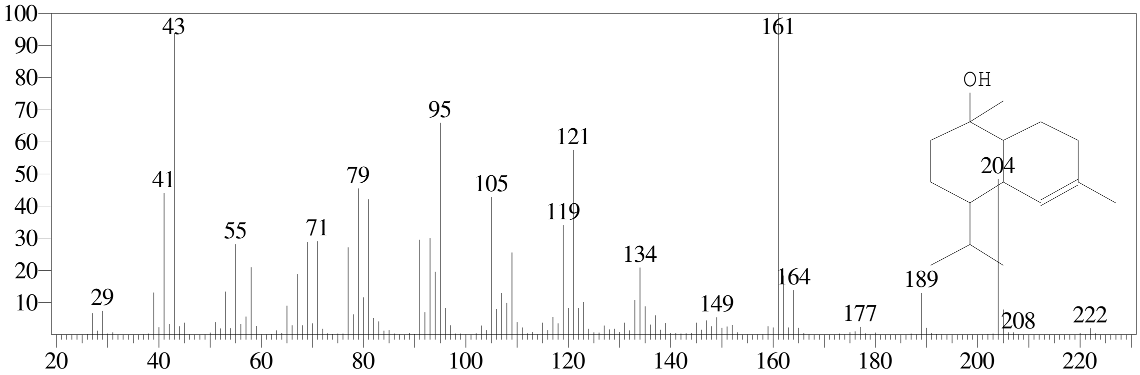

<< Target >>
Line#:29 R.Time:34.917(Scan#:3891) MassPeaks:33
RawMode:Averaged 34.908-34.925(3890-3892) BasePeak:95.05(9354)
BG Mode:Calc. from Peak Group 1 - Event 1 Scan

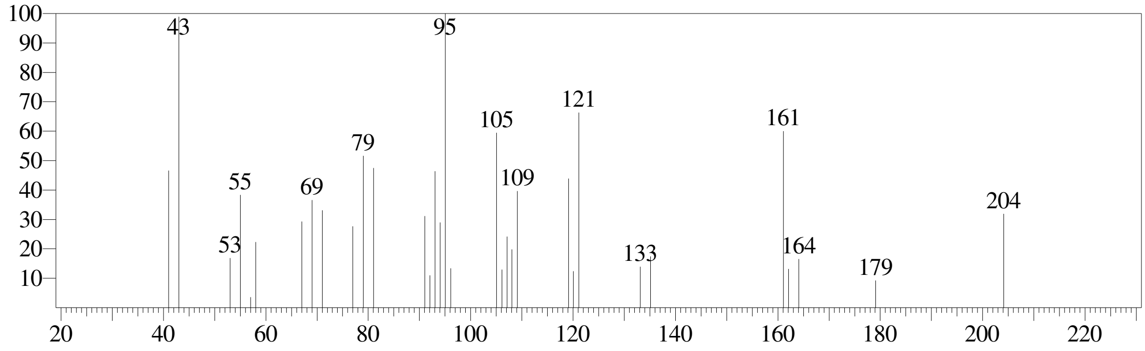

Hit#:3 Entry:83540 Library:NIST23-1.lib
SI:87 Formula:C15H26O CAS:481-34-5 MolWeight:222 RetIndex:1637
CompName:.alpha.-Cadinol $$ 4-Isopropyl-1,6-dimethyl-1,2,3,4,4a,7,8,8a-octahydro-1-naphthalenol # $$ (1R,4S,4aR,8al

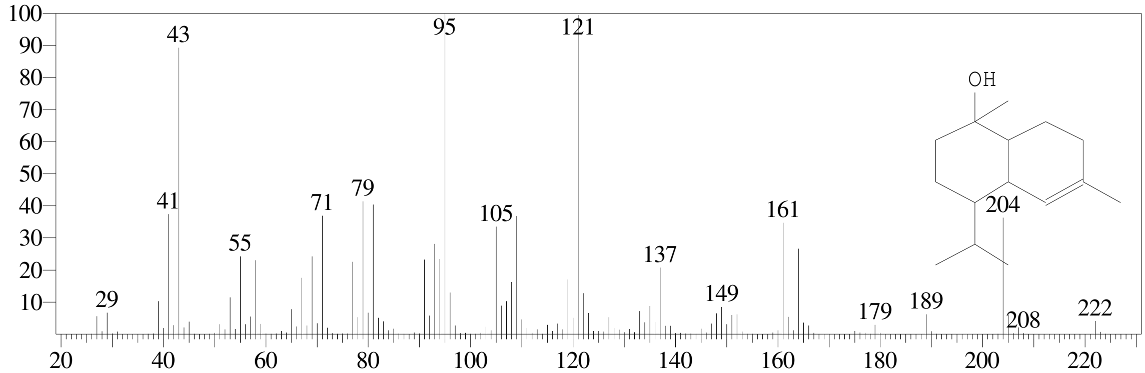

Hit#:4 Entry:28663 Library:NIST23s.lib
SI:87 Formula:C15H26O CAS:481-34-5 MolWeight:222 RetIndex:1637
CompName:.alpha.-Cadinol $$ 4-Isopropyl-1,6-dimethyl-1,2,3,4,4a,7,8,8a-octahydro-1-naphthalenol # $$ (1R,4S,4aR,8al

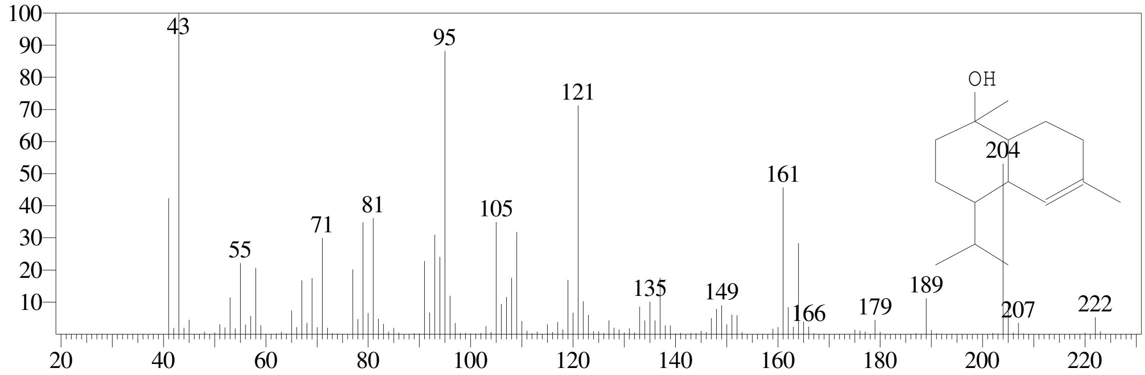

<< Target >>
Line#:29 R.Time:34.917(Scan#:3891) MassPeaks:33
RawMode:Averaged 34.908-34.925(3890-3892) BasePeak:95.05(9354)
BG Mode:Calc. from Peak Group 1 - Event 1 Scan

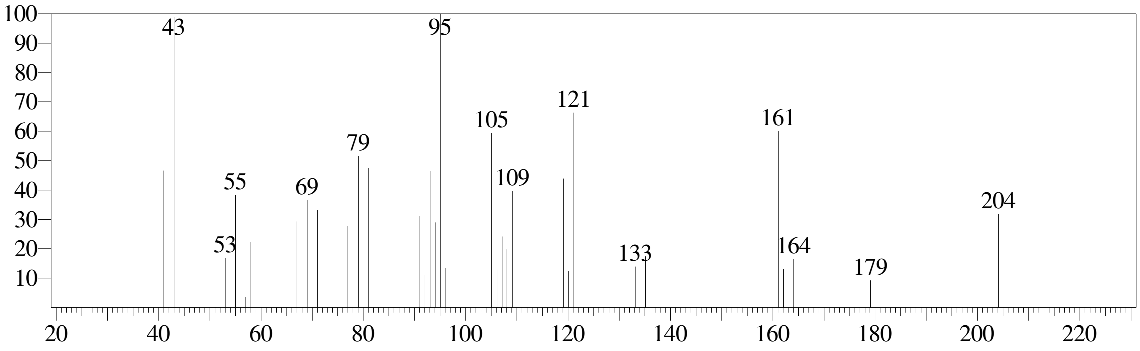

Hit#:5 Entry:28664 Library:NIST23s.lib
SI:86 Formula:C15H26O CAS:19912-62-0 MolWeight:222 RetIndex:1635
CompName:.tau.-Muurolol $$ 4-Isopropyl-1,6-dimethyl-1,2,3,4,4a,7,8,8a-octahydro-1-naphthalenol-, [1S-(1.alpha.,4.alpha

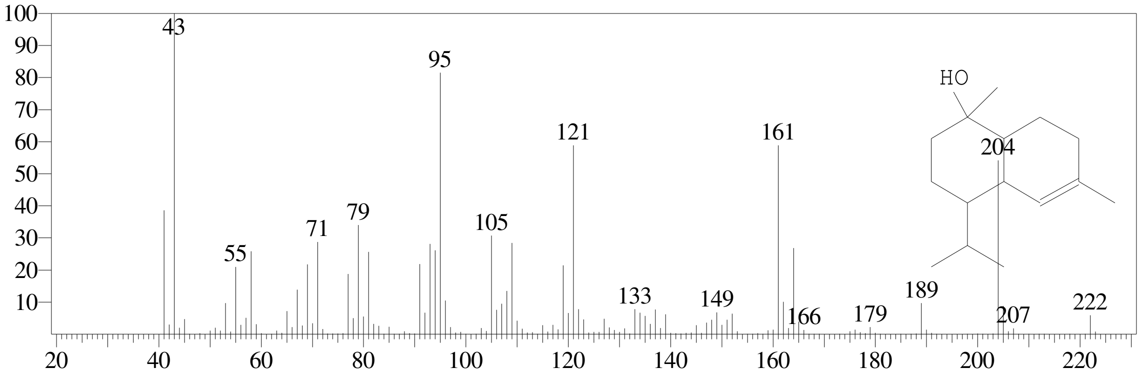

<< Target >>
Line#:30 R.Time:35.375(Scan#:3946) MassPeaks:50
RawMode:Averaged 35.367-35.383(3945-3947) BasePeak:43.00(18189)
BG Mode:Calc. from Peak Group 1 - Event 1 Scan

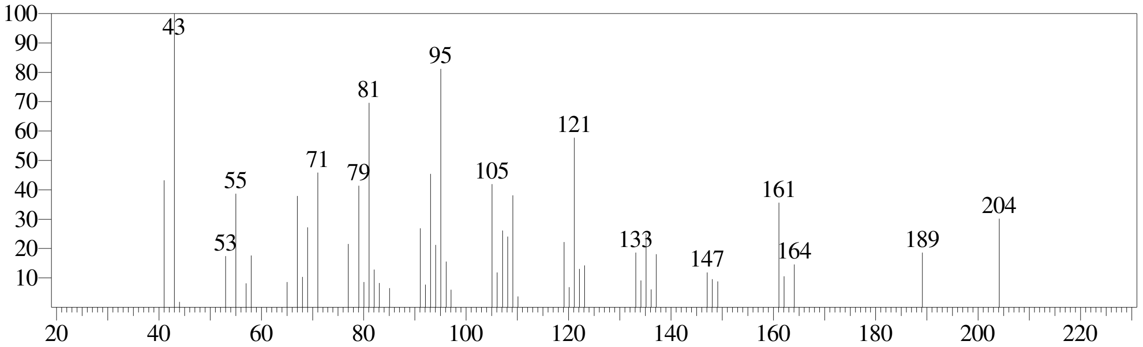

Hit#:1 Entry:28651 Library:NIST23s.lib
SI:89 Formula:C15H26O CAS:5945-72-2 MolWeight:222 RetIndex:1655
CompName:Neointermedeol $$ (1S,4aR,7R,8aR)-1,4a-Dimethyl-7-(prop-1-en-2-yl)decahydronaphthalen-1-ol $$ 1-Napht

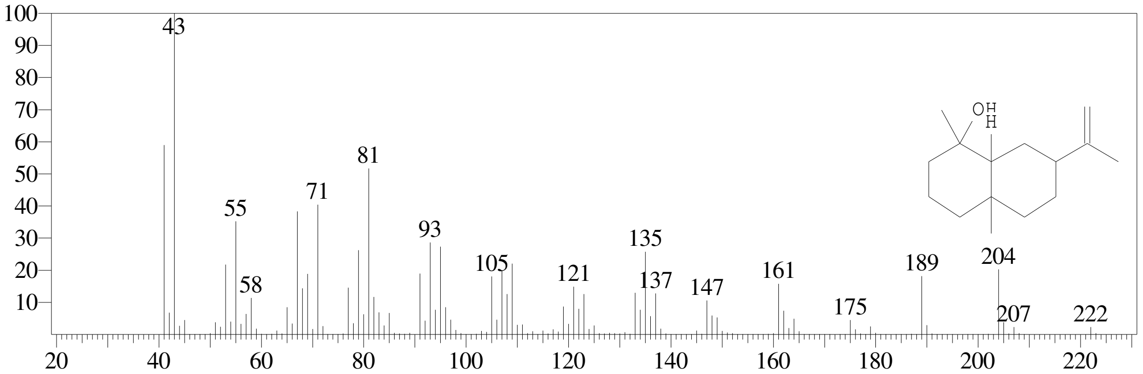

Hit#:2 Entry:28663 Library:NIST23s.lib
SI:89 Formula:C15H26O CAS:481-34-5 MolWeight:222 RetIndex:1637
CompName:.alpha.-Cadinol $$ 4-Isopropyl-1,6-dimethyl-1,2,3,4,4a,7,8,8a-octahydro-1-naphthalenol # $$ (1R,4S,4aR,8al

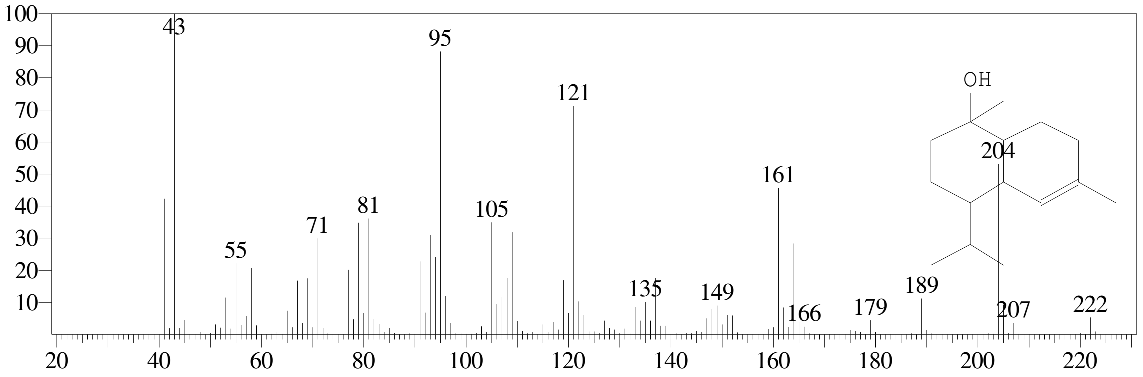

<< Target >>
Line#:30   R.Time:35.375(Scan#:3946) MassPeaks:50
RawMode:Averaged 35.367-35.383(3945-3947) BasePeak:43.00(18189)
BG Mode:Calc. from Peak Group 1 - Event 1 Scan

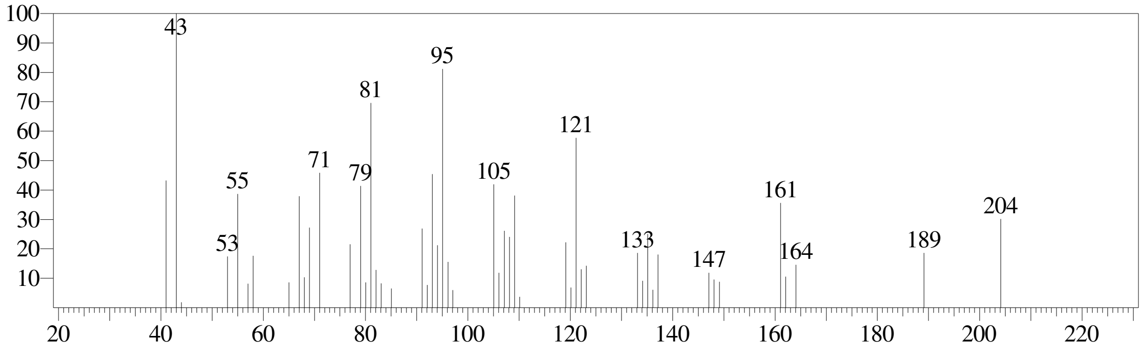

Hit#:3 Entry:83465 Library:NIST23-1.lib
SI:89 Formula:C15H26O CAS:16641-47-7 MolWeight:222 RetIndex:1655
CompName:Selin-11-en-4-alpha-ol

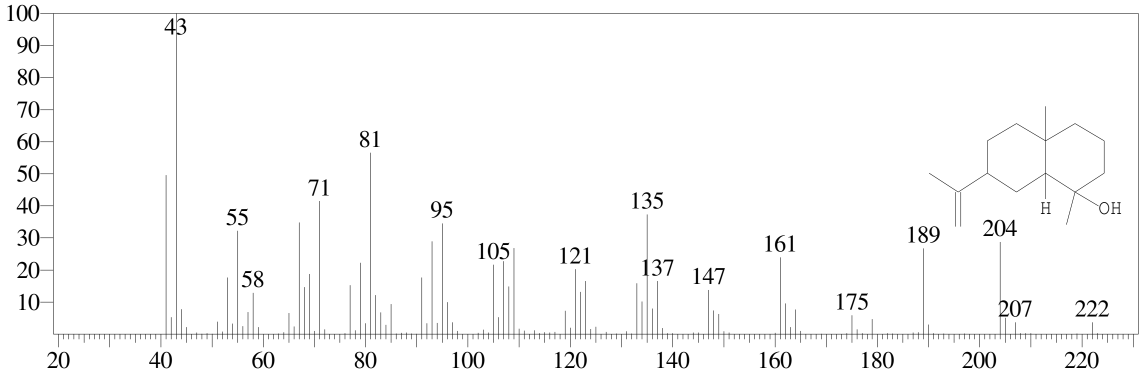

Hit#:4 Entry:28752 Library:NIST23s.lib
SI:89 Formula:C15H26O CAS:5937-11-1 MolWeight:222 RetIndex:1637
CompName:.tau.-Cadinol $$ 4-Isopropyl-1,6-dimethyl-1,2,3,4,4a,7,8,8a-octahydro-1-naphthalenol-, (1S-(1alpha,4alpha,4a

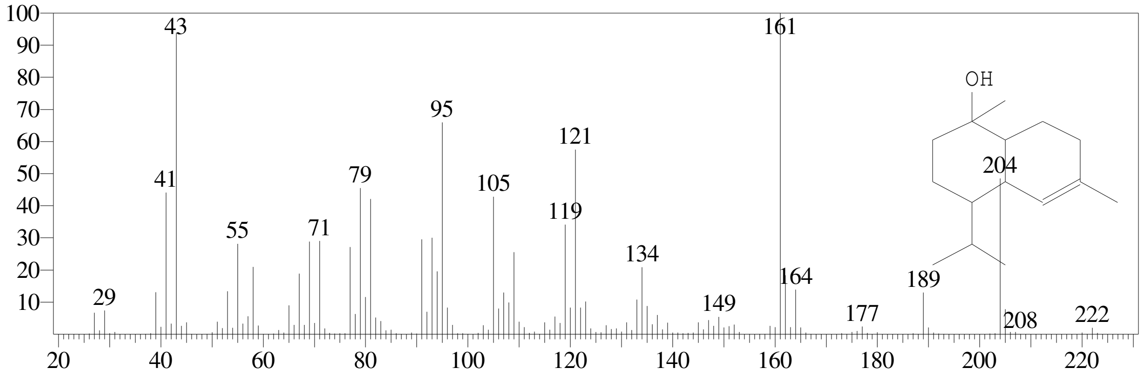

<< Target >>
Line#:30   R.Time:35.375(Scan#:3946) MassPeaks:50
RawMode:Averaged 35.367-35.383(3945-3947) BasePeak:43.00(18189)
BG Mode:Calc. from Peak Group 1 - Event 1 Scan

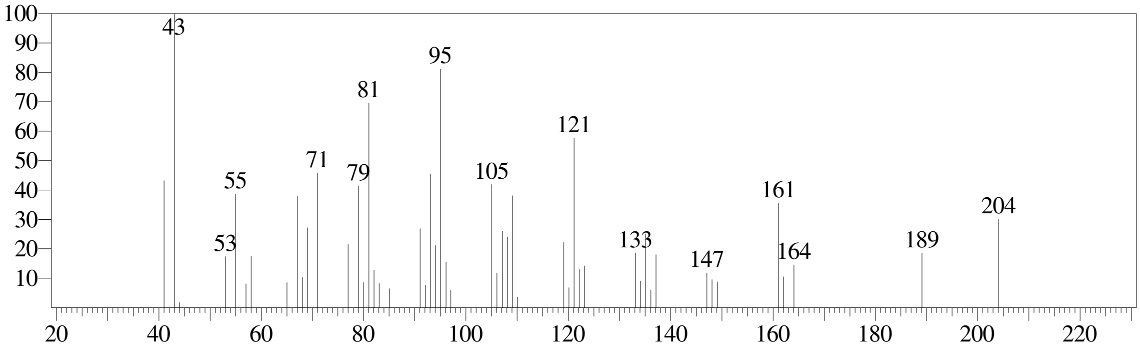

Hit#:5 Entry:83541 Library:NIST23-1.lib
SI:89 Formula:C15H26O CAS:19912-62-0 MolWeight:222 RetIndex:1635
CompName:.tau.-Muurolol $$ 4-Isopropyl-1,6-dimethyl-1,2,3,4,4a,7,8,8a-octahydro-1-naphthalenol-, [1S-(1.alpha.,4.alph

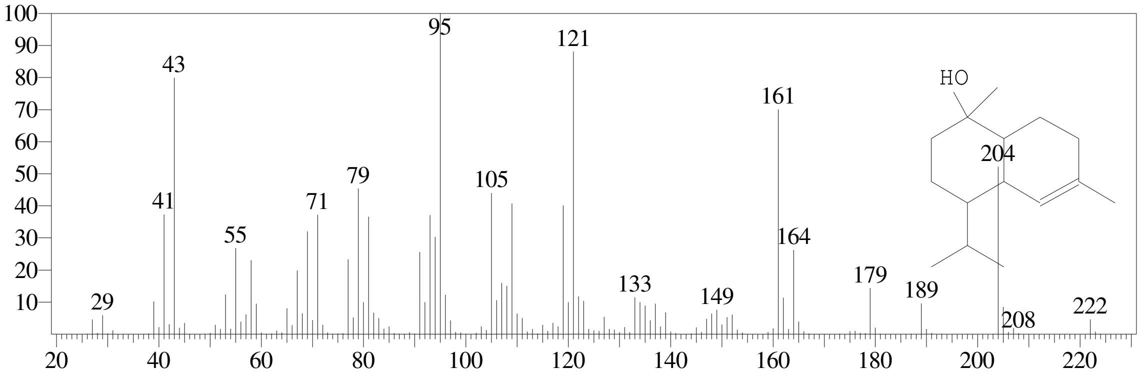

Supplement: Supplementary file 1 [file plants-15-01406-s001.zip › ELbb.pdf]
