# Supplementary material for: Unveiling Chemical Profile and Insecticidal Potential of Essential Oils from Leaves of Seven Eugenia L. Species (Myrtaceae)
Source: Plants (Basel). 2026 May 5;15(9):1406. doi: 10.3390/plants15091406 (PMC13165059; doi:10.3390/plants15091406)

UNIVERSIDADE FERAL RURAL DO RIO DE JANEIRO
INSTITUTO DE QUÍMICA

LabCrom — Laboratório de Cromatografia — CAM · UFRRJ

CENTRAL ANALÍTICA MULTIUSUÁRIO
INSTITUTO DE QUÍMICA · UFRRJ

CGMS

Analyzed by: Cristiane Cardoso
Analyzed: 17/6/2025
Solicitante:Douglas
Sample Name: EM
Injection Volume: 1,0 uL   Solvente: Diclorometano
Data File: C:\GCMSsolution\Data\Project1\Douglas\2025\MLLENA\170625\EM.qgd
Method File: C:\GCMSsolution\Data\Project1\Douglas\Essencial Adams-Inj.qgm
EQUIPAMENTO: Modelo:GCMS-QP2010 Plus (Shimadzu)
Coluna: VF-5m (30X0.25X0.25)

Chromatogram EM C:\GCMSsolution\Data\Project1\Douglas\2025\MLLENA\170625\EM.qgd

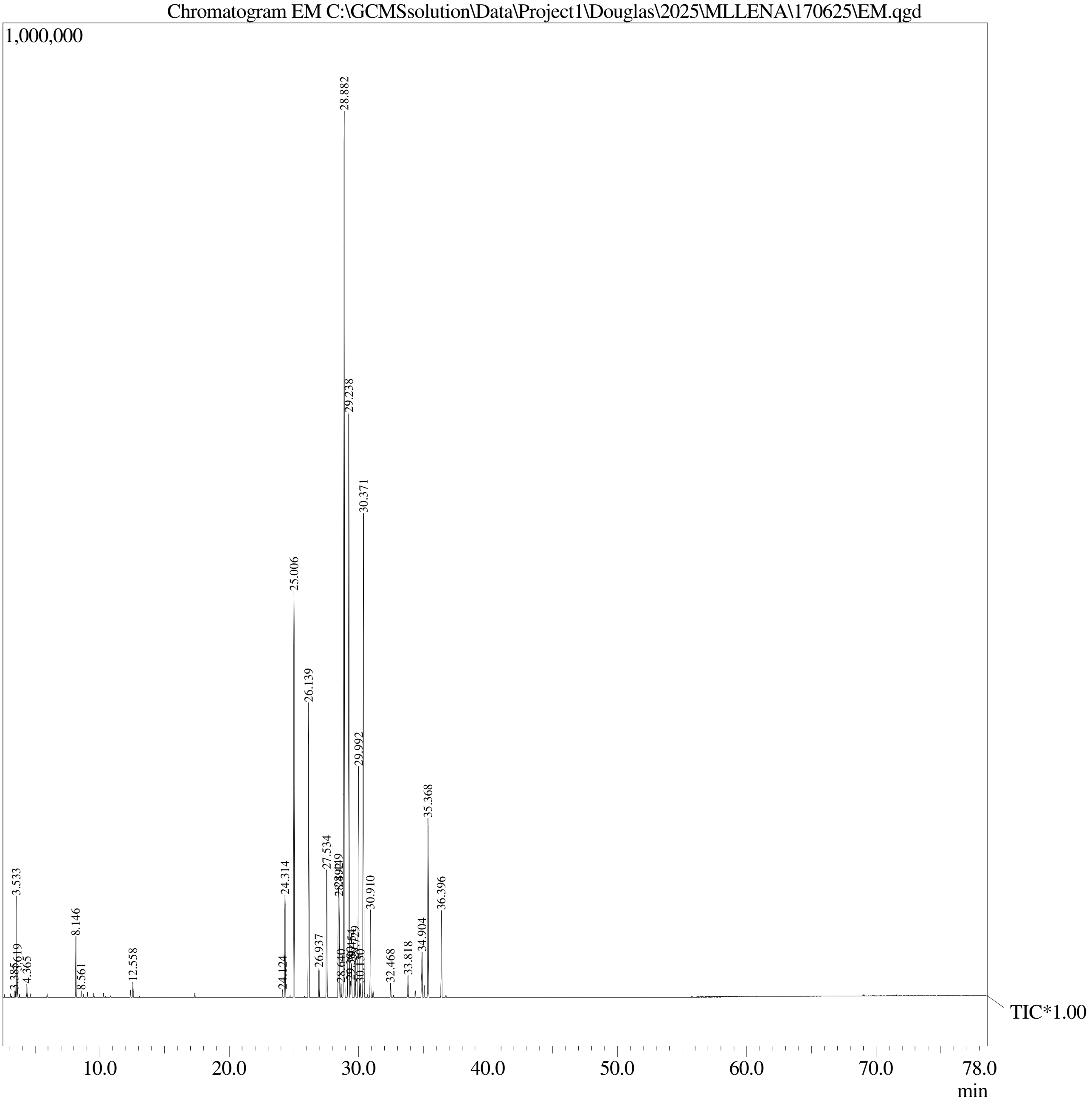

Library

<< Target >>
Line#:1   R.Time:3.383(Scan#:107) MassPeaks:5
RawMode:Averaged 3.375-3.392(106-108) BasePeak:44.00(1668)
BG Mode:None Group 1 - Event 1 Scan

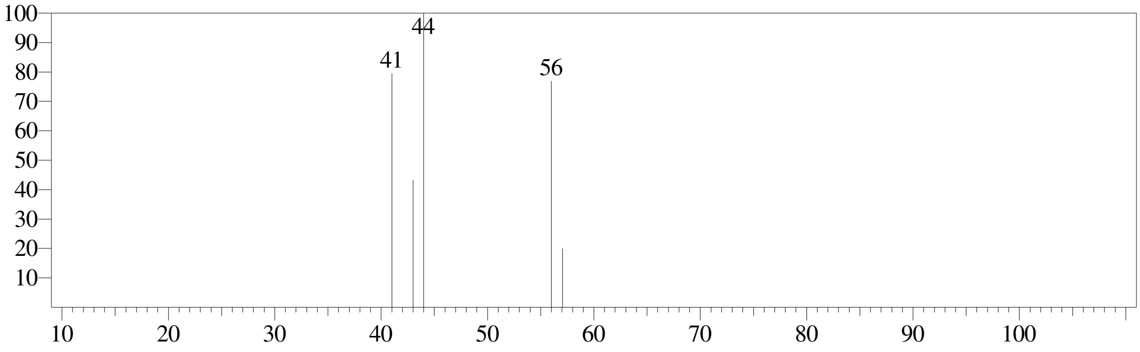

Hit#:1 Entry:2258 Library:NIST23s.lib
SI:85 Formula:C6H12O CAS:66-25-1 MolWeight:100 RetIndex:804
CompName:Hexanal $$ n-Caproaldehyde $$ n-Hexanal $$ Caproaldehyde $$ Caproic aldehyde $$ Capronaldehyde $$ He

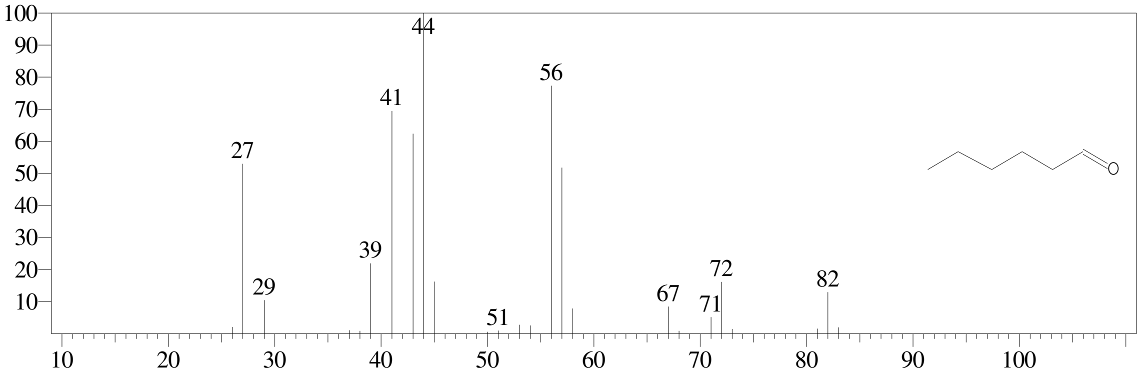

Hit#:2 Entry:2255 Library:NIST23s.lib
SI:83 Formula:C6H12O CAS:66-25-1 MolWeight:100 RetIndex:804
CompName:Hexanal $$ n-Caproaldehyde $$ n-Hexanal $$ Caproaldehyde $$ Caproic aldehyde $$ Capronaldehyde $$ He

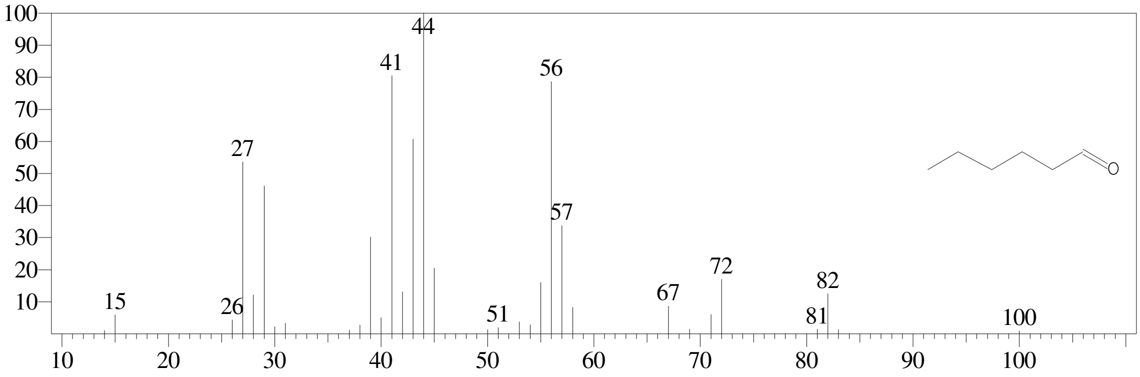

<< Target >>
Line#:1   R.Time:3.383(Scan#:107) MassPeaks:5
RawMode:Averaged 3.375-3.392(106-108) BasePeak:44.00(1668)
BG Mode:None Group 1 - Event 1 Scan

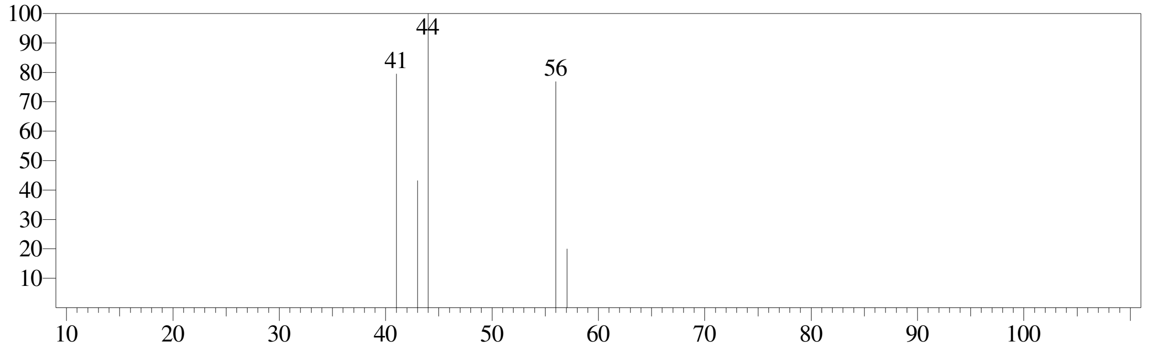

Hit#:3 Entry:2168 Library:NIST23-1.lib
SI:82 Formula:C6H12O CAS:66-25-1 MolWeight:100 RetIndex:804
CompName:Hexanal $$ n-Caproaldehyde $$ n-Hexanal $$ Caproaldehyde $$ Caproic aldehyde $$ Capronaldehyde $$ He

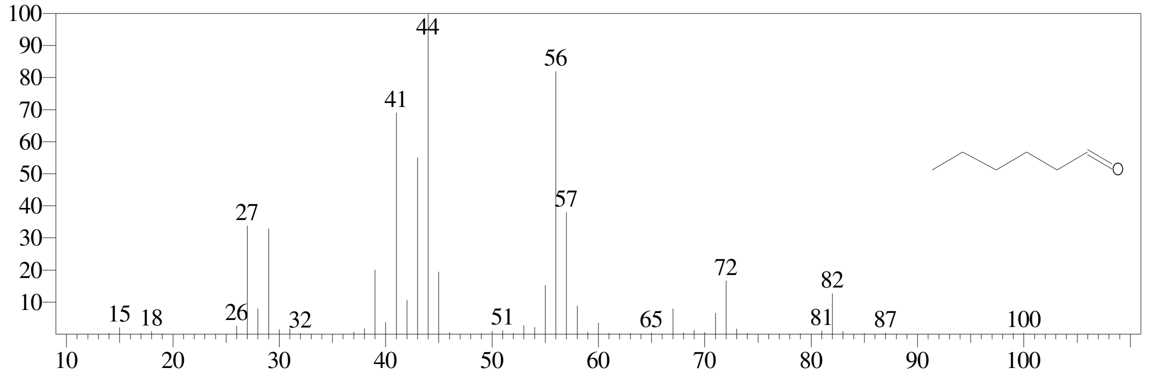

Hit#:4 Entry:2257 Library:NIST23s.lib
SI:81 Formula:C6H12O CAS:66-25-1 MolWeight:100 RetIndex:804
CompName:Hexanal $$ n-Caproaldehyde $$ n-Hexanal $$ Caproaldehyde $$ Caproic aldehyde $$ Capronaldehyde $$ He

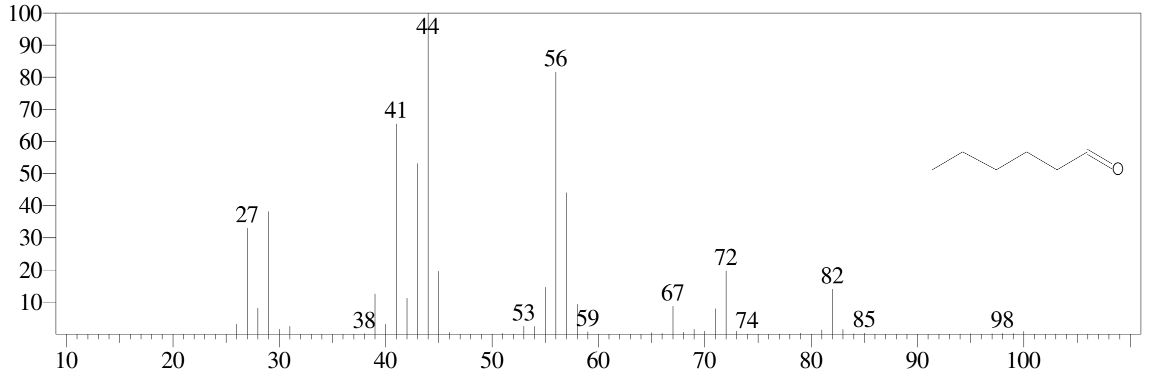

<< Target >>
Line#:1   R.Time:3.383(Scan#:107) MassPeaks:5
RawMode:Averaged 3.375-3.392(106-108) BasePeak:44.00(1668)
BG Mode:None Group 1 - Event 1 Scan

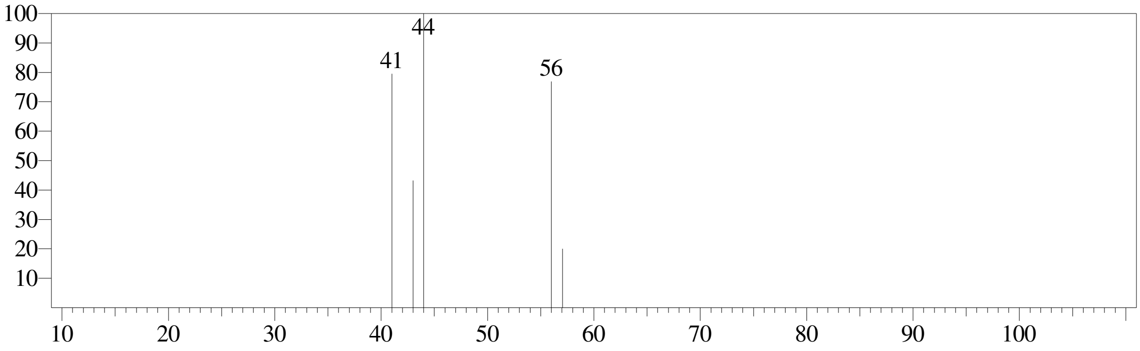

Hit#:5 Entry:2256 Library:NIST23s.lib
SI:80 Formula:C6H12O CAS:66-25-1 MolWeight:100 RetIndex:804
CompName:Hexanal $$ n-Caproaldehyde $$ n-Hexanal $$ Caproaldehyde $$ Caproic aldehyde $$ Capronaldehyde $$ He

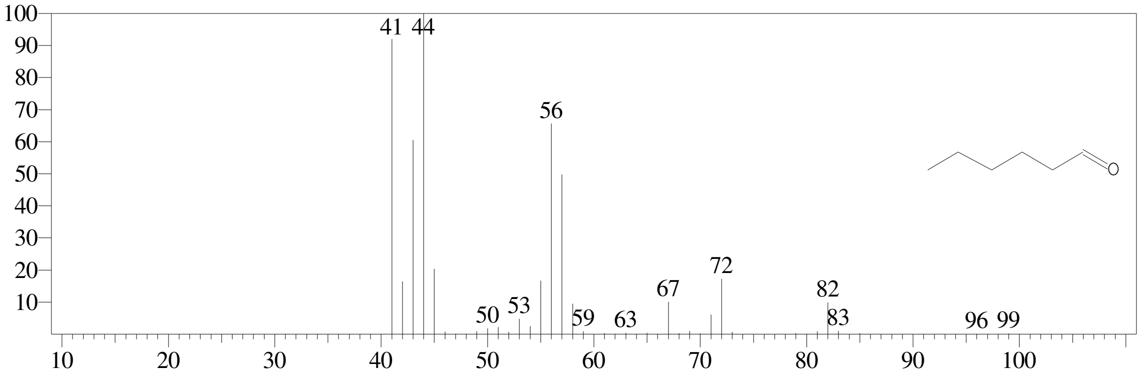

<< Target >>
Line#:2   R.Time:3.533(Scan#:125) MassPeaks:12
RawMode:Averaged 3.525-3.542(124-126) BasePeak:45.00(53521)
BG Mode:Calc. from Peak Group 1 - Event 1 Scan

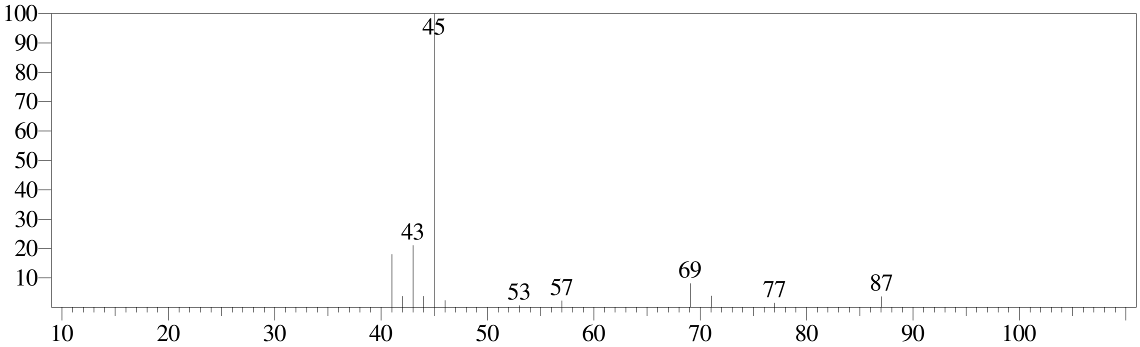

Hit#:1 Entry:2533 Library:NIST23-1.lib
SI:93 Formula:C6H14O CAS:52019-78-0 MolWeight:102 RetIndex:791
CompName:2-Hexanol, (S)- $$ (S)-(+)-2-Hexanol $$ 2-Hexanol # $$

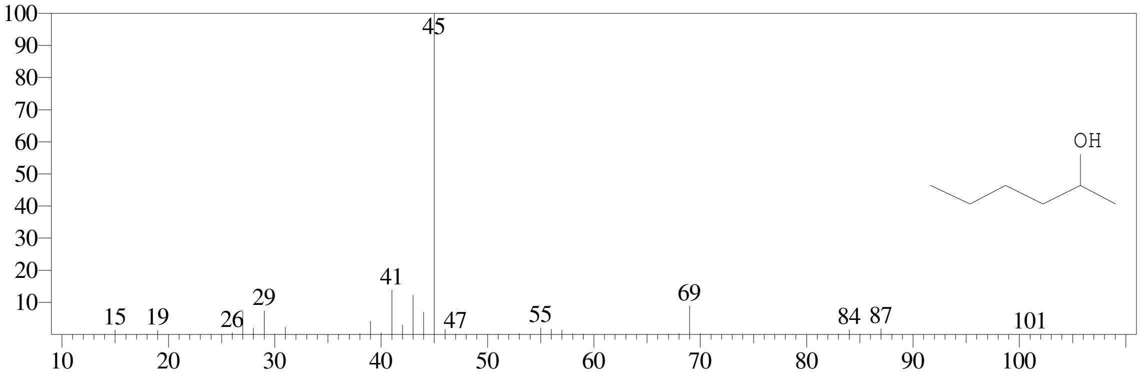

Hit#:2 Entry:2597 Library:NIST23s.lib
SI:93 Formula:C6H14O CAS:108-11-2 MolWeight:102 RetIndex:752
CompName:2-Pentanol, 4-methyl- $$ Isobutylmethylcarbinol $$ Isobutylmethylmethanol $$ Methylisobutylcarbinol $$ M

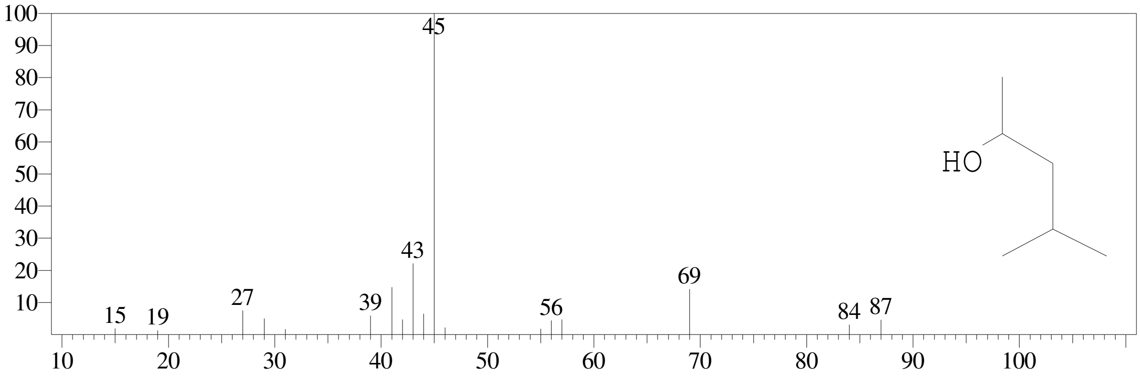

<< Target >>
Line#:2 R.Time:3.533(Scan#:125) MassPeaks:12
RawMode:Averaged 3.525-3.542(124-126) BasePeak:45.00(53521)
BG Mode:Calc. from Peak Group 1 - Event 1 Scan

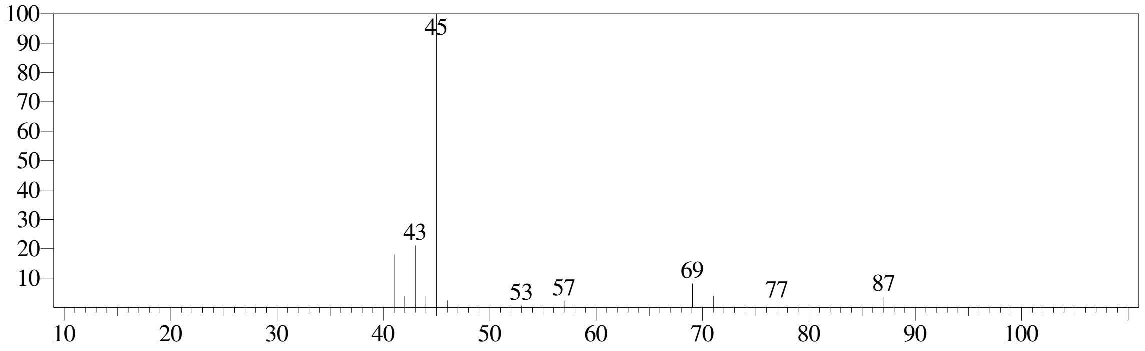

Hit#:3 Entry:2534 Library:NIST23-1.lib
SI:93 Formula:C6H14O CAS:26549-24-6 MolWeight:102 RetIndex:791
CompName:2-Hexanol, (R)- $$ (R)-(-)-2-Hexanol $$ 2-Hexanol # $$

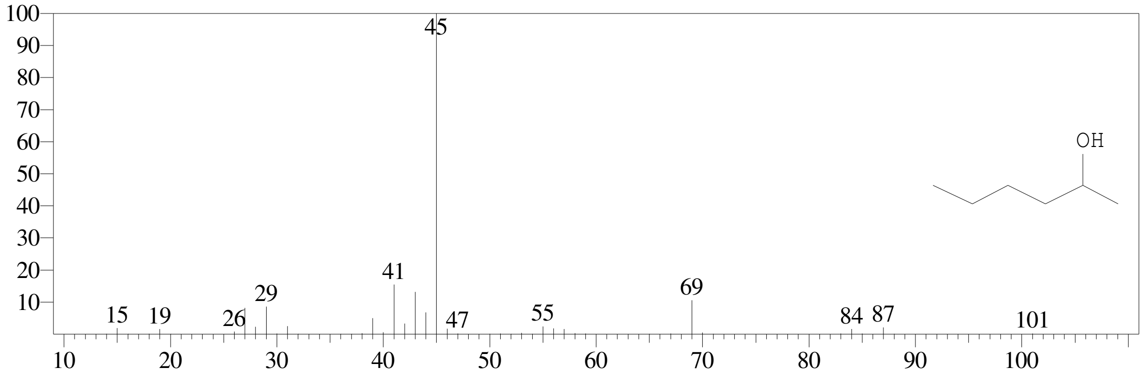

Hit#:4 Entry:1082 Library:NIST23s.lib
SI:93 Formula:C5H10O CAS:625-31-0 MolWeight:86 RetIndex:658
CompName:4-Penten-2-ol $$ 1-Penten-4-ol $$ 4-Hydroxypent-1-ene $$ CH2=CHCH2CH(OH)CH3 $$

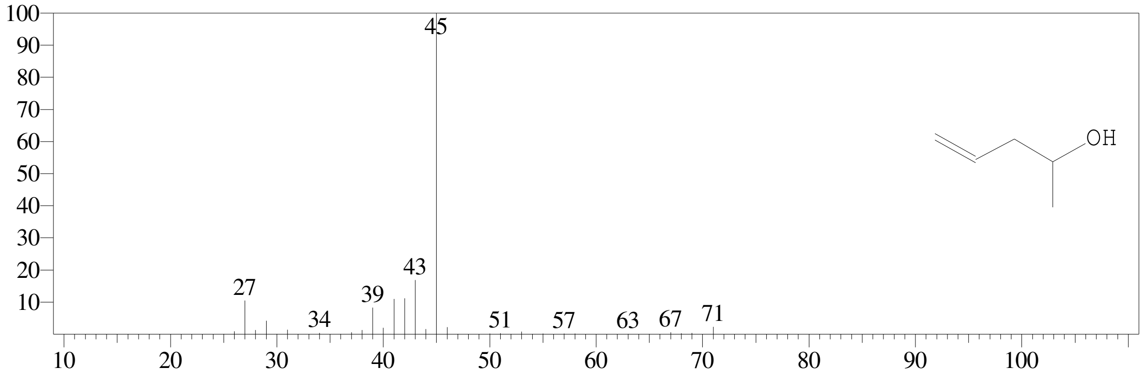

<< Target >>
Line#:2 R.Time:3.533(Scan#:125) MassPeaks:12
RawMode:Averaged 3.525-3.542(124-126) BasePeak:45.00(53521)
BG Mode:Calc. from Peak Group 1 - Event 1 Scan

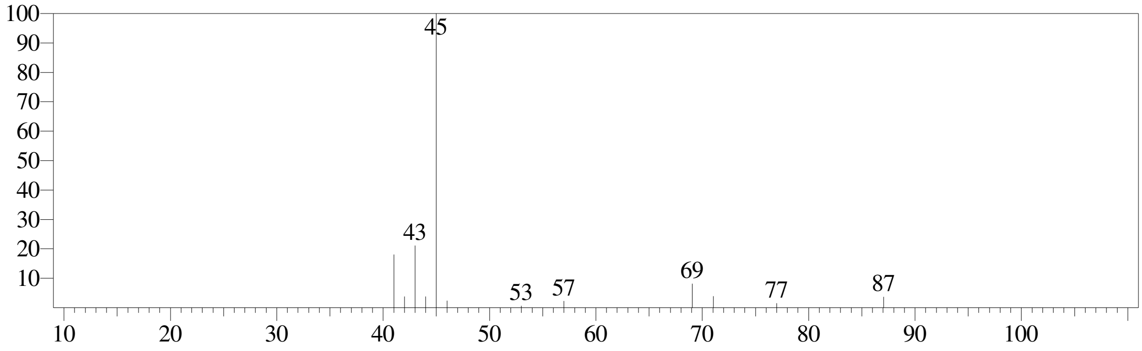

Hit#:5 Entry:897 Library:NIST23-1.lib
SI:92 Formula:C5H10O CAS:625-31-0 MolWeight:86 RetIndex:658
CompName:4-Penten-2-ol $$ 1-Penten-4-ol $$ 4-Hydroxypent-1-ene $$ CH2=CHCH2CH(OH)CH3 $$

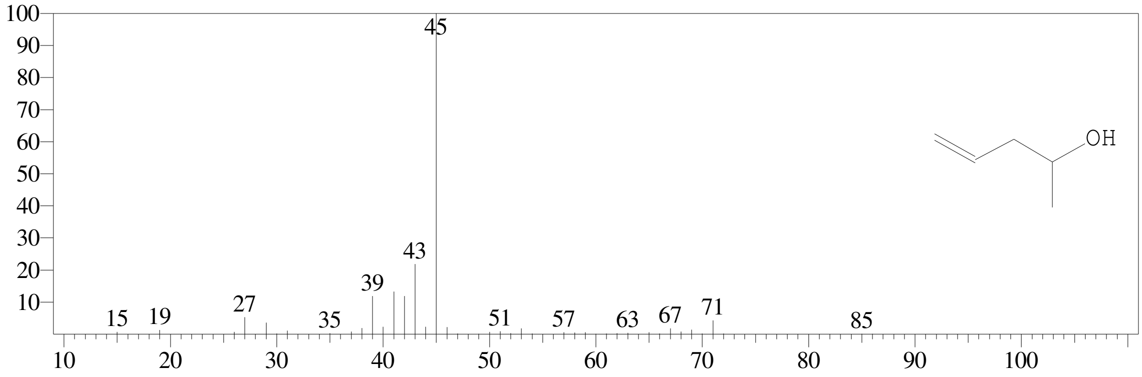

<< Target >>
Line#:3   R.Time:3.617(Scan#:135) MassPeaks:5
RawMode:Averaged 3.608-3.625(134-136) BasePeak:59.00(13161)
BG Mode:None Group 1 - Event 1 Scan

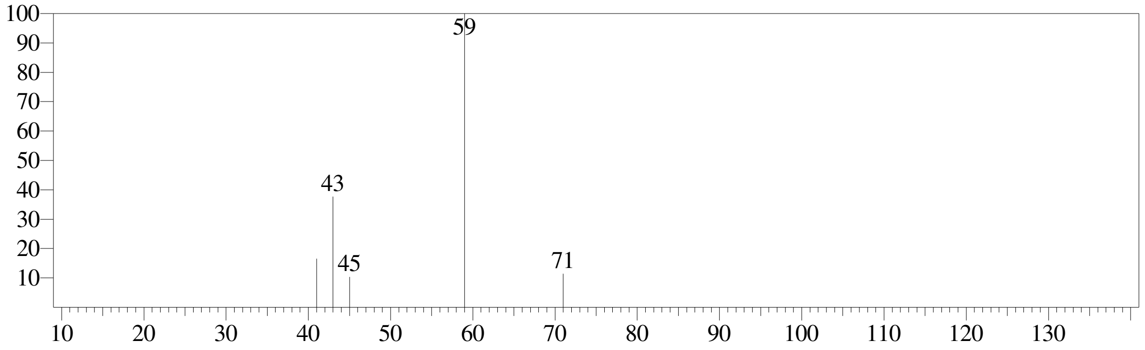

Hit#:1 Entry:2726 Library:NIST23-1.lib
SI:90 Formula:C4H8O3 CAS:594-61-6 MolWeight:104 RetIndex:932
CompName:Propanoic acid, 2-hydroxy-2-methyl- $$ 2-Hydroxyisobutyric acid $$ Lactic acid, 2-methyl- $$ .alpha.-Hydrc

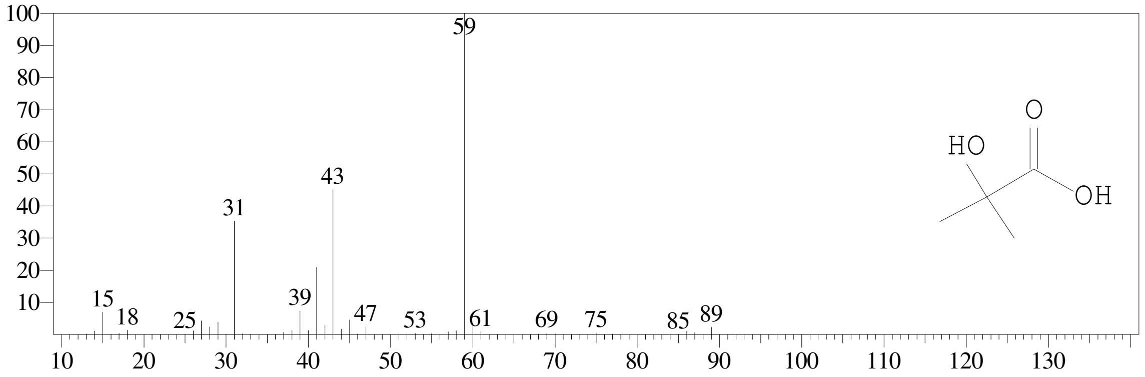

Hit#:2 Entry:2466 Library:NIST23-1.lib
SI:90 Formula:C5H10O2 CAS:115-22-0 MolWeight:102 RetIndex:737
CompName:3-Hydroxy-3-methyl-2-butanone $$ 2-Butanone, 3-hydroxy-3-methyl- $$ Dimethylacetylcarbinol $$ 3-Hydrc

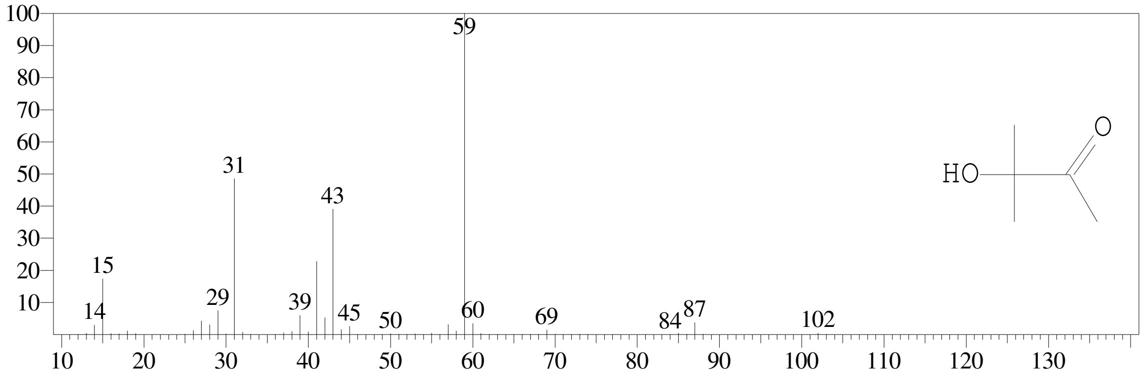

<< Target >>
Line#:3   R.Time:3.617(Scan#:135) MassPeaks:5
RawMode:Averaged 3.608-3.625(134-136) BasePeak:59.00(13161)
BG Mode:None Group 1 - Event 1 Scan

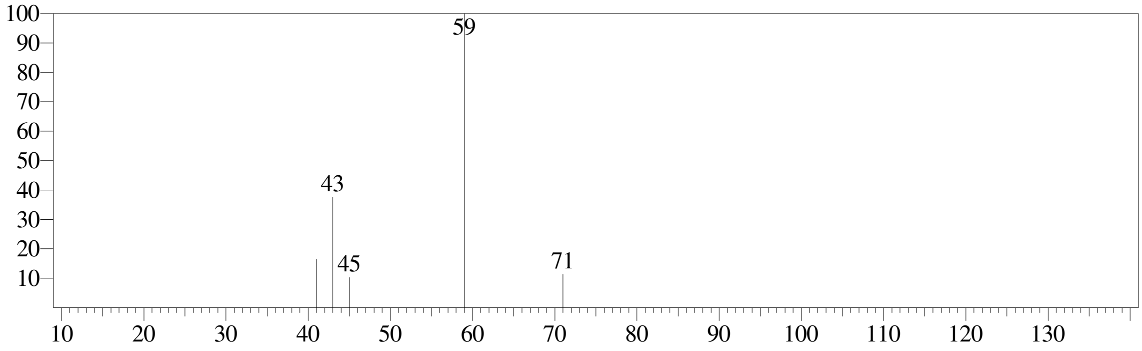

Hit#:3 Entry:2775 Library:NIST23-1.lib
SI:90 Formula:C5H12O2 CAS:5396-58-7 MolWeight:104 RetIndex:812
CompName:2-Methyl-2,3-butanediol $$ 2-Methylbutane-2,3-diol $$ 2,3-Dihydroxy-2-methylbutane $$

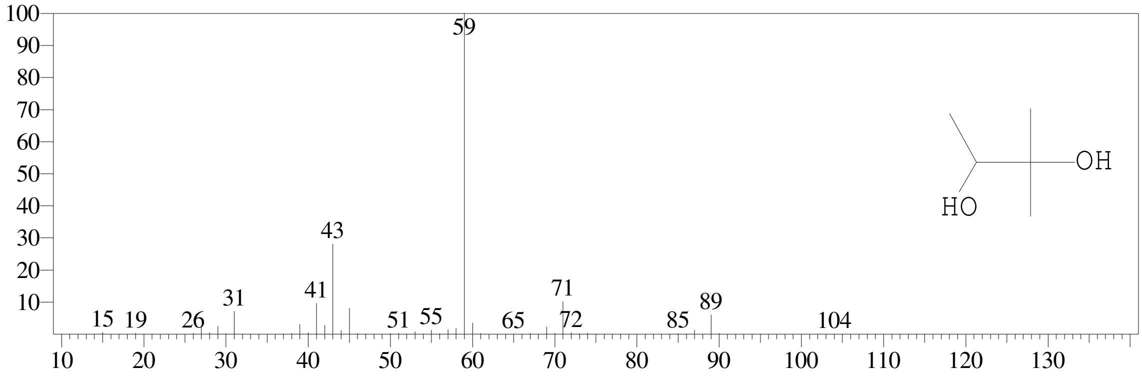

Hit#:4 Entry:9783 Library:NIST23-1.lib
SI:89 Formula:C6H12O3 CAS:70657-70-4 MolWeight:132 RetIndex:880
CompName:2-Methoxypropyl acetate $$ 1-Propanol, 2-methoxy-, 1-acetate $$ 1-Propanol, 2-methoxy-, acetate $$ 2-Meth

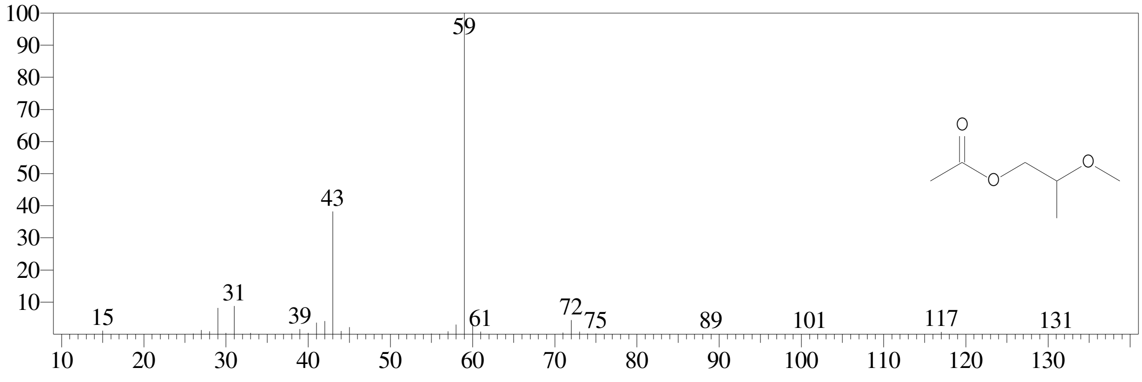

<< Target >>
Line#:3   R.Time:3.617(Scan#:135) MassPeaks:5
RawMode:Averaged 3.608-3.625(134-136) BasePeak:59.00(13161)
BG Mode:None Group 1 - Event 1 Scan

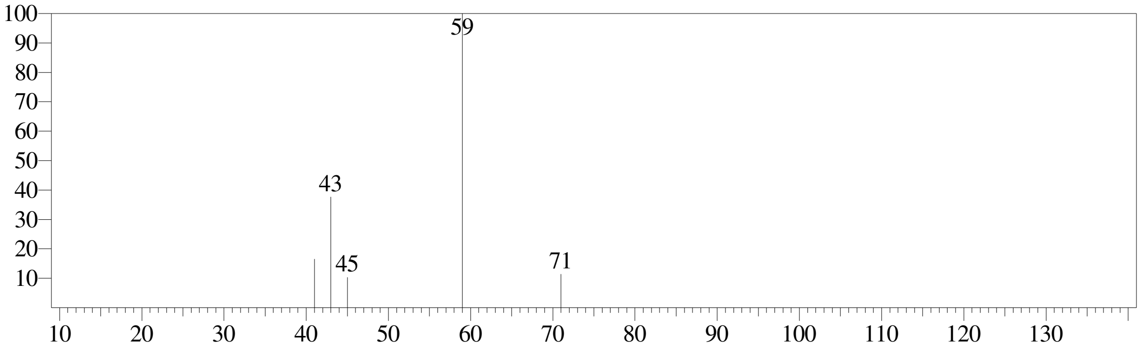

Hit#:5 Entry:583 Library:NIST23s.lib
SI:89 Formula:C4H10O CAS:598-53-8 MolWeight:74 RetIndex:471
CompName:Propane, 2-methoxy- $$ Ether, isopropyl methyl $$ Isopropyl methyl ether $$ Isopryl $$ Methyl isopropyl etl

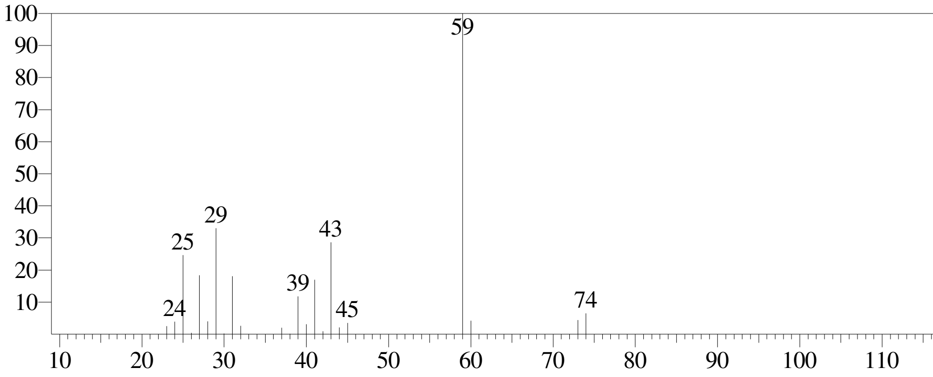

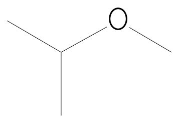

<< Target >>
Line#:4 R.Time:4.367(Scan#:225) MassPeaks:6
RawMode:Averaged 4.358-4.375(224-226) BasePeak:41.00(3407)
BG Mode:Calc. from Peak Group 1 - Event 1 Scan

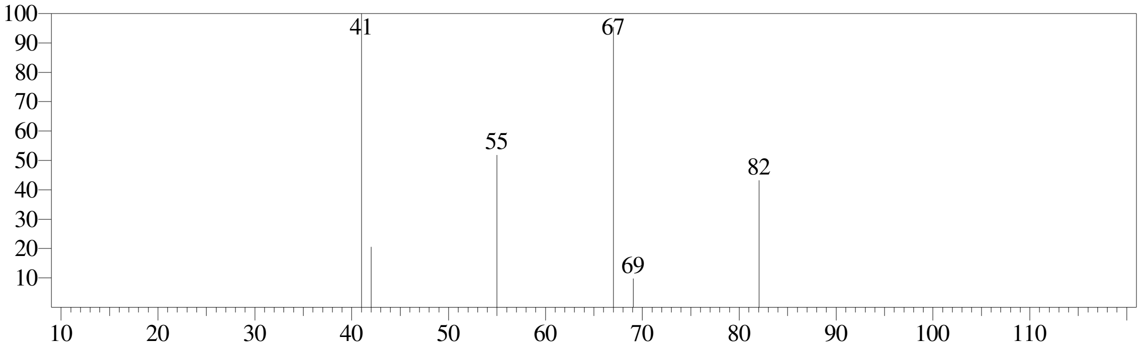

Hit#:1 Entry:6482 Library:NIST23s.lib
SI:83 Formula:C7H12O2 CAS:33467-73-1 MolWeight:128 RetIndex:931
CompName:3-Hexen-1-ol, formate, (Z)- $$ cis-.beta.-Hexenyl formate $$ cis-3-Hexenyl formate $$ cis-3-Hexen-1-ol forr

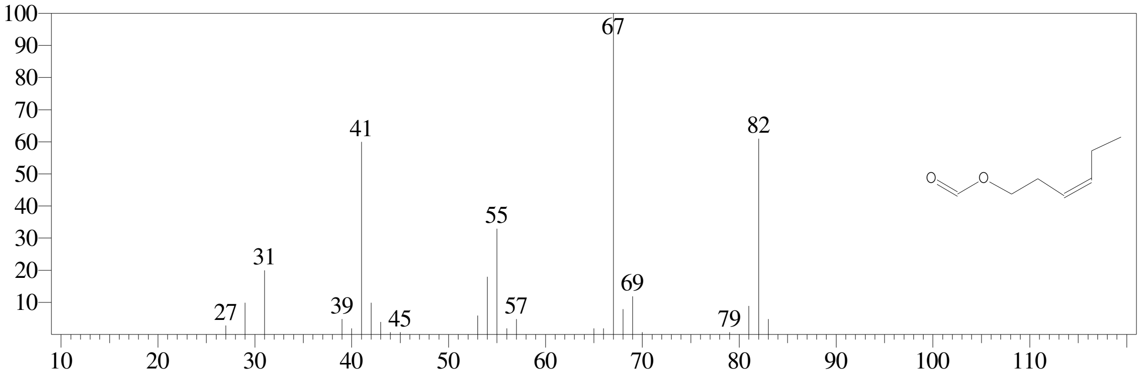

Hit#:2 Entry:2218 Library:NIST23s.lib
SI:83 Formula:C6H12O CAS:544-12-7 MolWeight:100 RetIndex:857
CompName:3-Hexen-1-ol $$ 3-Hexen-1-ol (c,t) $$ 3-Hexenol $$ Hex-3-en-1-ol $$

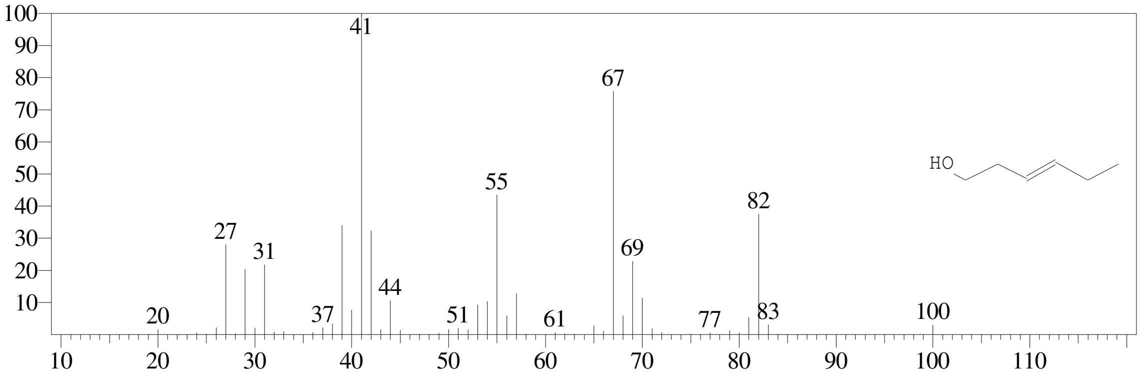

<< Target >>
Line#:4   R.Time:4.367(Scan#:225) MassPeaks:6
RawMode:Averaged 4.358-4.375(224-226) BasePeak:41.00(3407)
BG Mode:Calc. from Peak Group 1 - Event 1 Scan

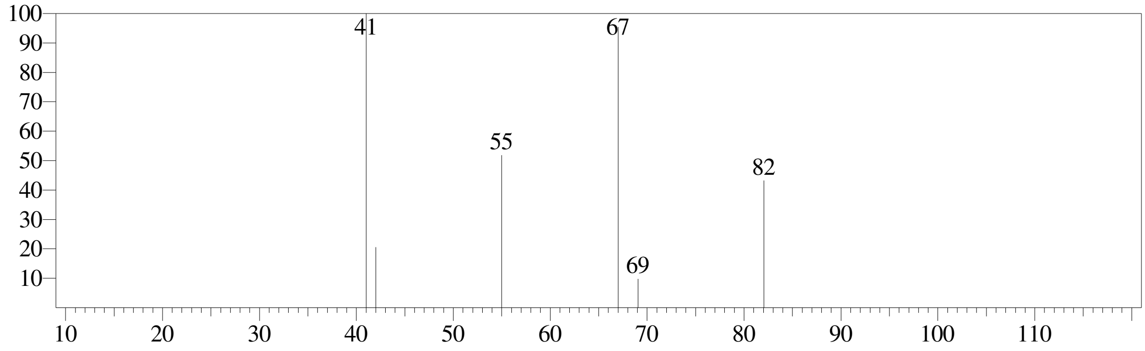

Hit#:3 Entry:2217 Library:NIST23s.lib
SI:82 Formula:C6H12O CAS:544-12-7 MolWeight:100 RetIndex:857
CompName:3-Hexen-1-ol $$ 3-Hexen-1-ol (c,t) $$ 3-Hexenol $$ Hex-3-en-1-ol $$

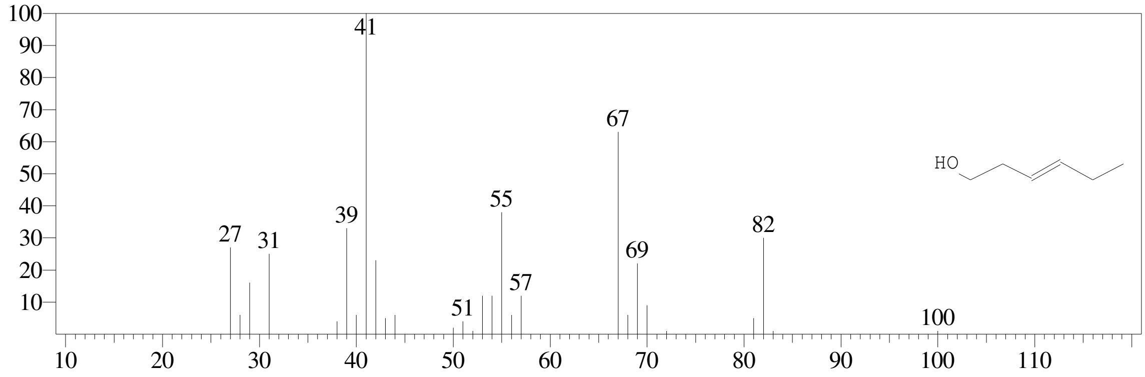

Hit#:4 Entry:2219 Library:NIST23s.lib
SI:82 Formula:C6H12O CAS:544-12-7 MolWeight:100 RetIndex:857
CompName:3-Hexen-1-ol $$ 3-Hexen-1-ol (c,t) $$ 3-Hexenol $$ Hex-3-en-1-ol $$

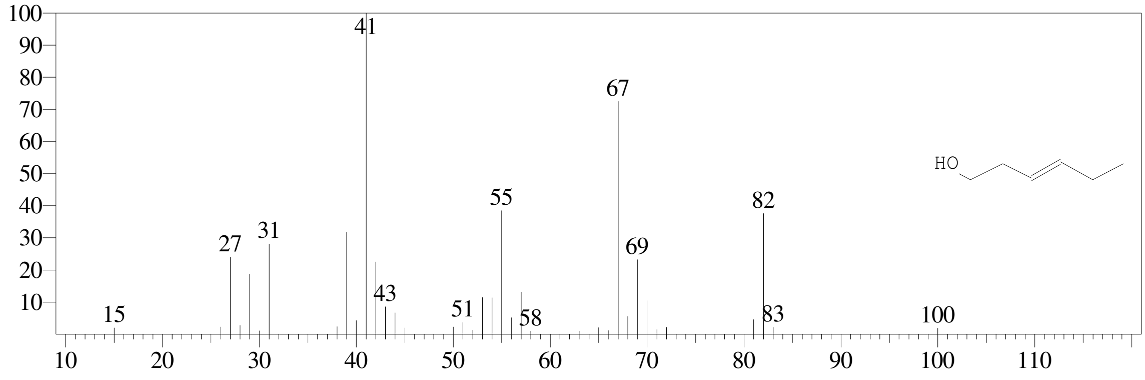

<< Target >>
Line#:4 R.Time:4.367(Scan#:225) MassPeaks:6
RawMode:Averaged 4.358-4.375(224-226) BasePeak:41.00(3407)
BG Mode:Calc. from Peak Group 1 - Event 1 Scan

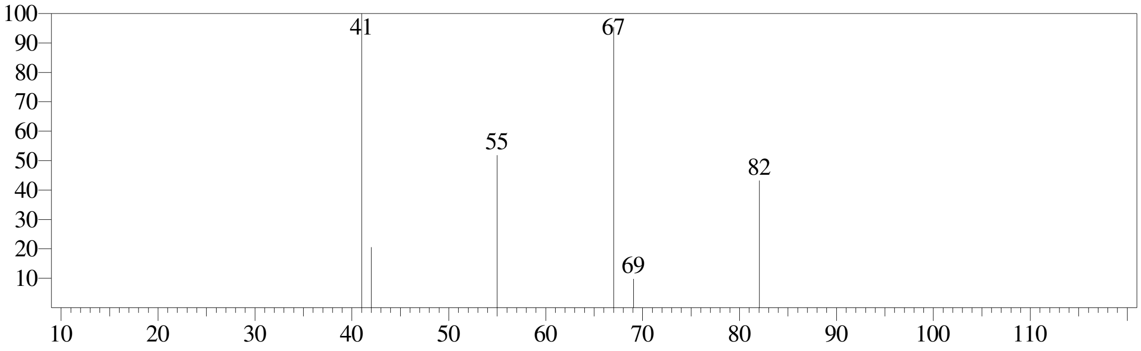

Hit#:5 Entry:6479 Library:NIST23s.lib
SI:82 Formula:C7H12O2 CAS:33467-73-1 MolWeight:128 RetIndex:931
CompName:3-Hexen-1-ol, formate, (Z)- $$ cis-.beta.-Hexenyl formate $$ cis-3-Hexenyl formate $$ cis-3-Hexen-1-ol forr

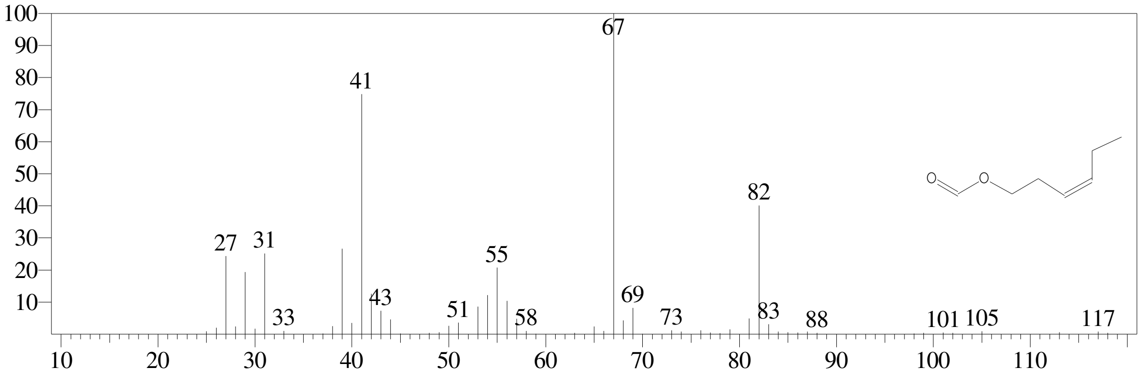

<< Target >>
Line#:5   R.Time:8.150(Scan#:679) MassPeaks:12
RawMode:Averaged 8.142-8.158(678-680) BasePeak:41.00(15911)
BG Mode:None Group 1 - Event 1 Scan

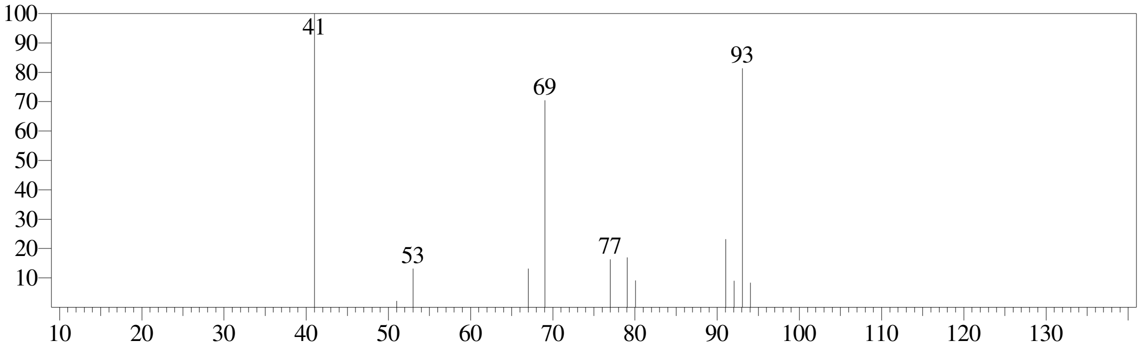

Hit#:1 Entry:8395 Library:NIST23s.lib
SI:89 Formula:C10H16 CAS:123-35-3 MolWeight:136 RetIndex:993
CompName:.beta.-Myrcene $$ 1,6-Octadiene, 7-methyl-3-methylene- $$ Myrcene $$ 7-Methyl-3-methylene-1,6-octadien

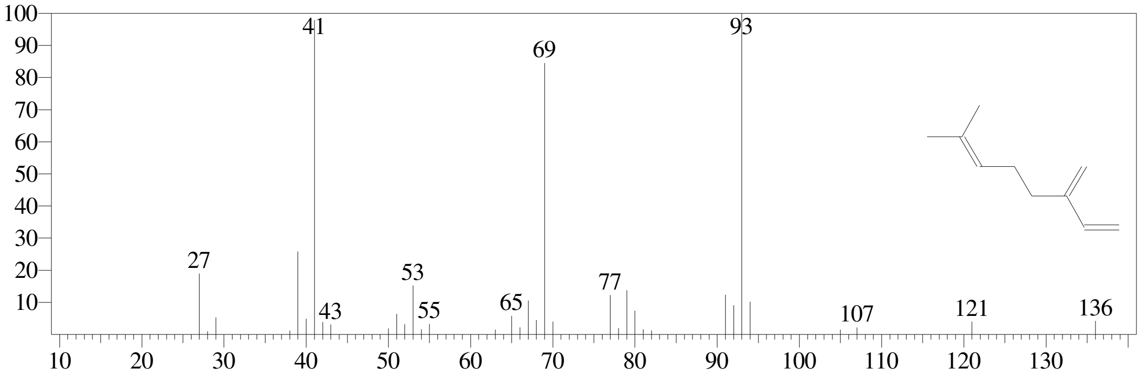

Hit#:2 Entry:8355 Library:NIST23s.lib
SI:89 Formula:C10H16 CAS:123-35-3 MolWeight:136 RetIndex:993
CompName:.beta.-Myrcene $$ 1,6-Octadiene, 7-methyl-3-methylene- $$ Myrcene $$ 7-Methyl-3-methylene-1,6-octadien

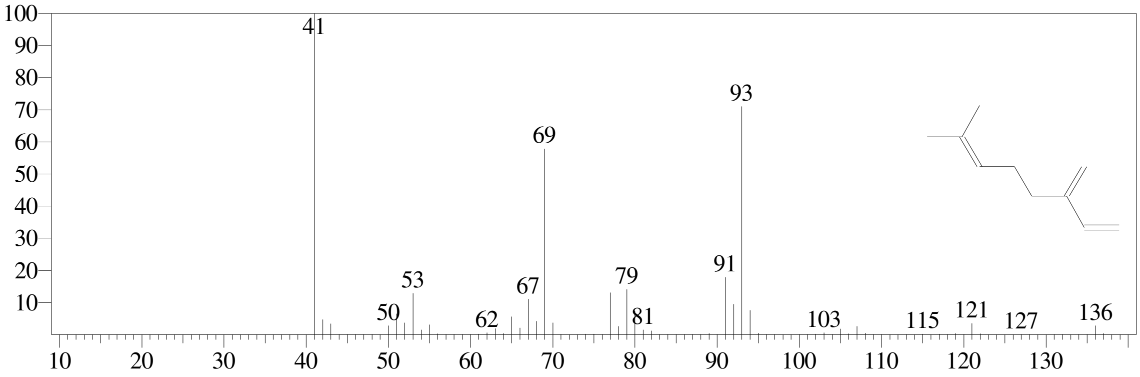

<< Target >>
Line#:5   R.Time:8.150(Scan#:679) MassPeaks:12
RawMode:Averaged 8.142-8.158(678-680) BasePeak:41.00(15911)
BG Mode:None Group 1 - Event 1 Scan

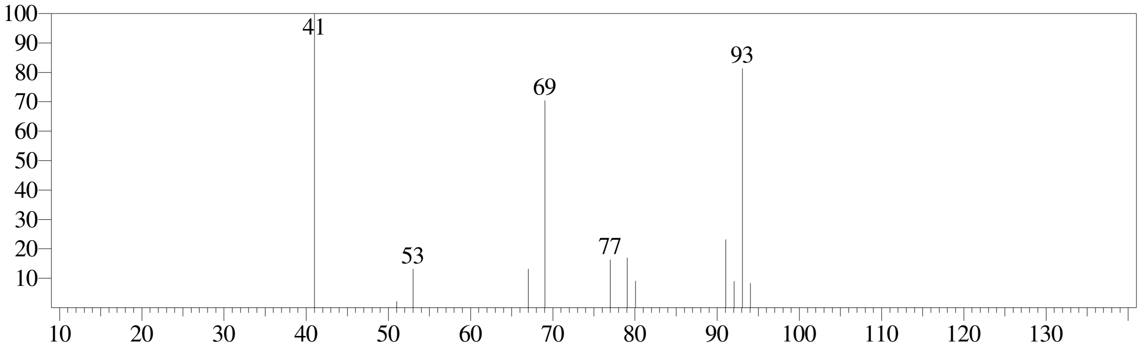

Hit#:3 Entry:11317 Library:NIST23-1.lib
SI:88 Formula:C10H16 CAS:123-35-3 MolWeight:136 RetIndex:993
CompName:.beta.-Myrcene $$ 1,6-Octadiene, 7-methyl-3-methylene- $$ Myrcene $$ 7-Methyl-3-methylene-1,6-octadien

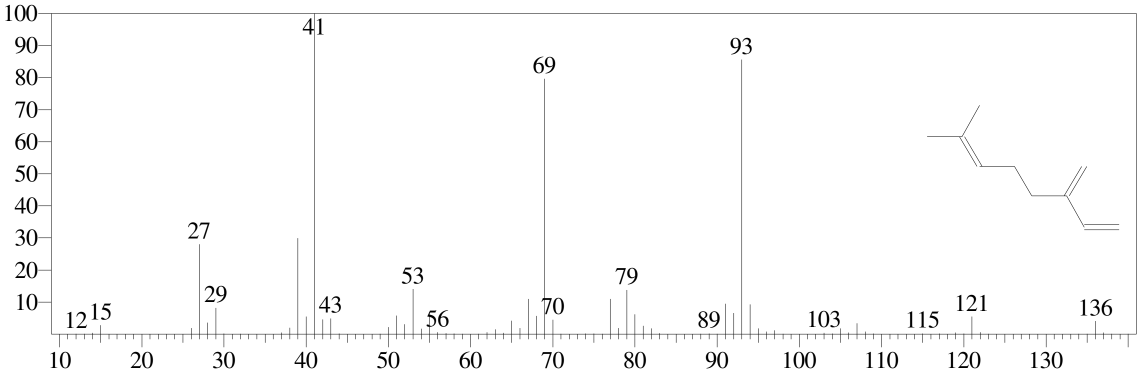

Hit#:4 Entry:8406 Library:NIST23s.lib
SI:88 Formula:C10H16 CAS:123-35-3 MolWeight:136 RetIndex:993
CompName:.beta.-Myrcene $$ 1,6-Octadiene, 7-methyl-3-methylene- $$ Myrcene $$ 7-Methyl-3-methylene-1,6-octadien

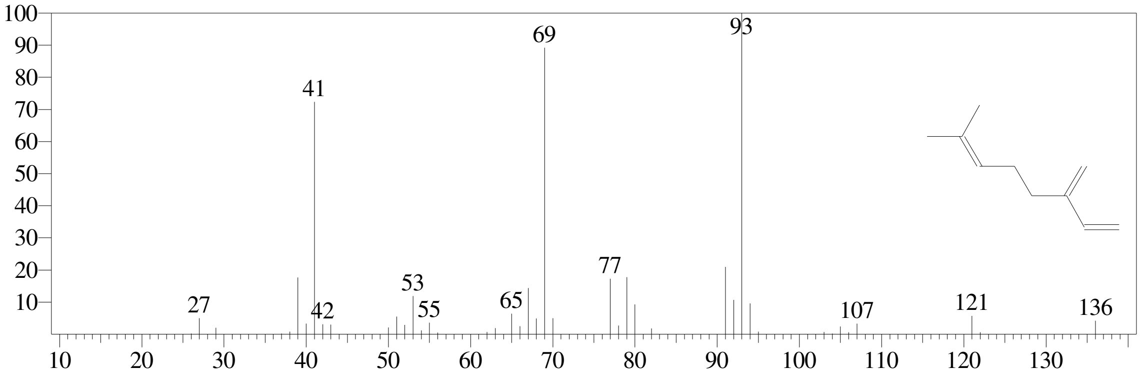

<< Target >>
Line#:5   R.Time:8.150(Scan#:679) MassPeaks:12
RawMode:Averaged 8.142-8.158(678-680) BasePeak:41.00(15911)
BG Mode:None Group 1 - Event 1 Scan

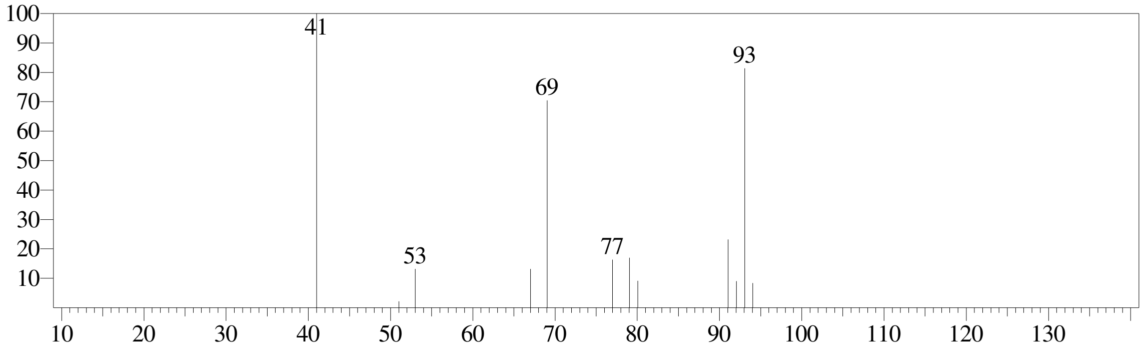

Hit#:5 Entry:8354 Library:NIST23s.lib
SI:86 Formula:C10H16 CAS:123-35-3 MolWeight:136 RetIndex:993
CompName:.beta.-Myrcene $$ 1,6-Octadiene, 7-methyl-3-methylene- $$ Myrcene $$ 7-Methyl-3-methylene-1,6-octadien

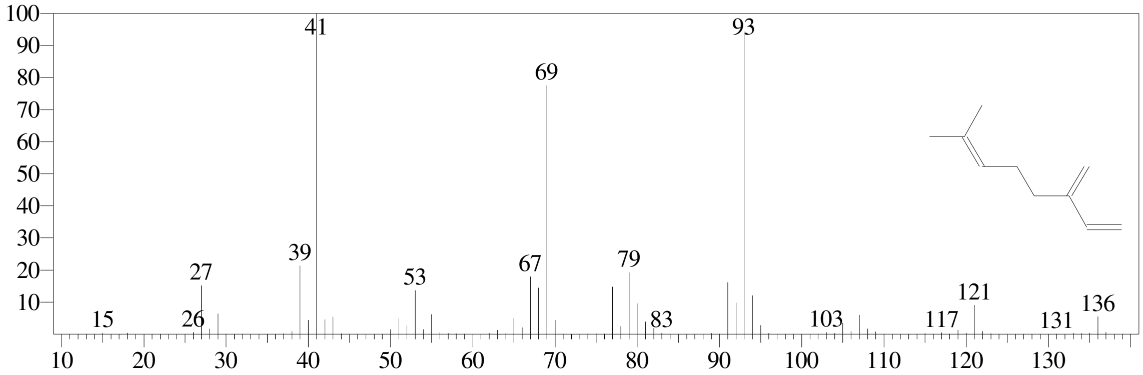

<< Target >>
Line#:6   R.Time:8.558(Scan#:728) MassPeaks:4
RawMode:Averaged 8.550-8.567(727-729) BasePeak:57.00(2633)
BG Mode:None Group 1 - Event 1 Scan

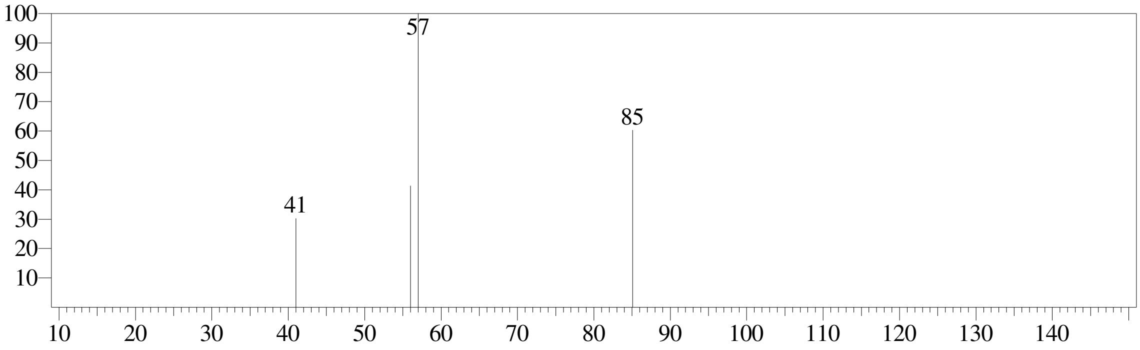

Hit#:1 Entry:45171 Library:NIST23-1.lib
SI:87 Formula:C10H18O3 CAS:1538-75-6 MolWeight:186 RetIndex:1045
CompName:2,2-Dimethylpropanoic anhydride $$ Trimethylacetic anhydride $$ Pivalic anhydride $$ Pivalic anydride $$ I

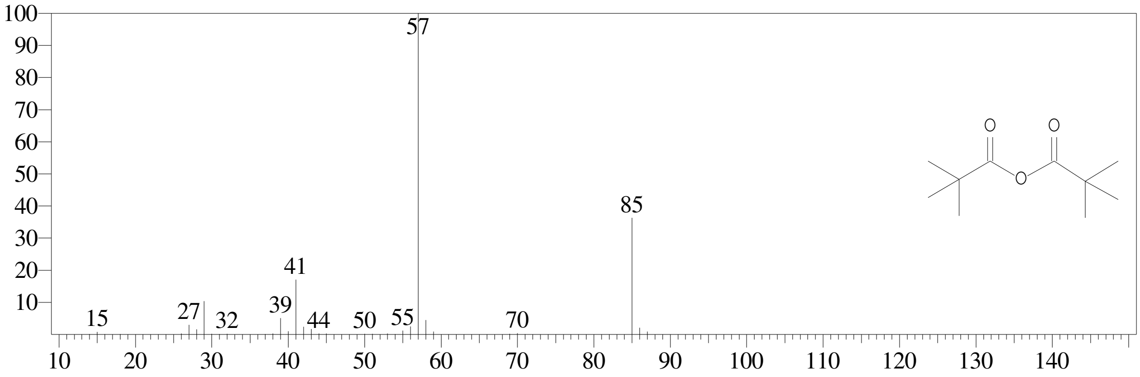

Hit#:2 Entry:9810 Library:NIST23s.lib
SI:86 Formula:C9H18O CAS:19078-97-8 MolWeight:142 RetIndex:968
CompName:2,2-Dimethyl-3-heptanone

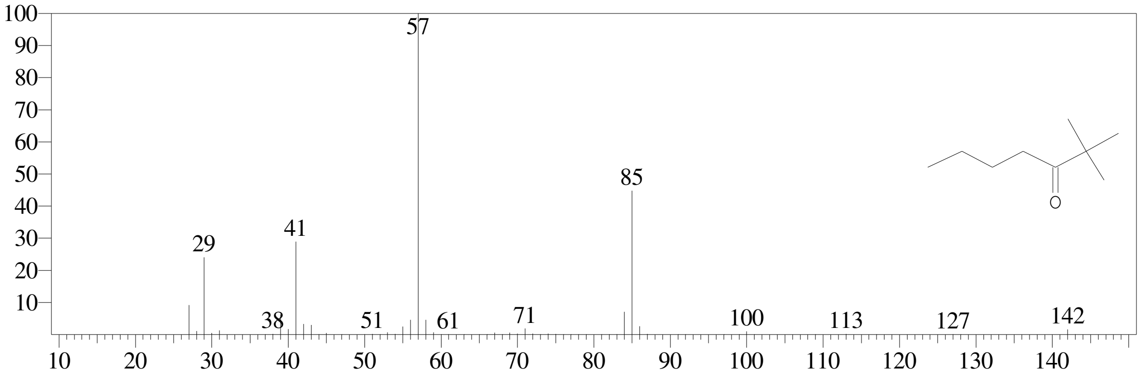

<< Target >>
Line#:6   R.Time:8.558(Scan#:728) MassPeaks:4
RawMode:Averaged 8.550-8.567(727-729) BasePeak:57.00(2633)
BG Mode:None Group 1 - Event 1 Scan

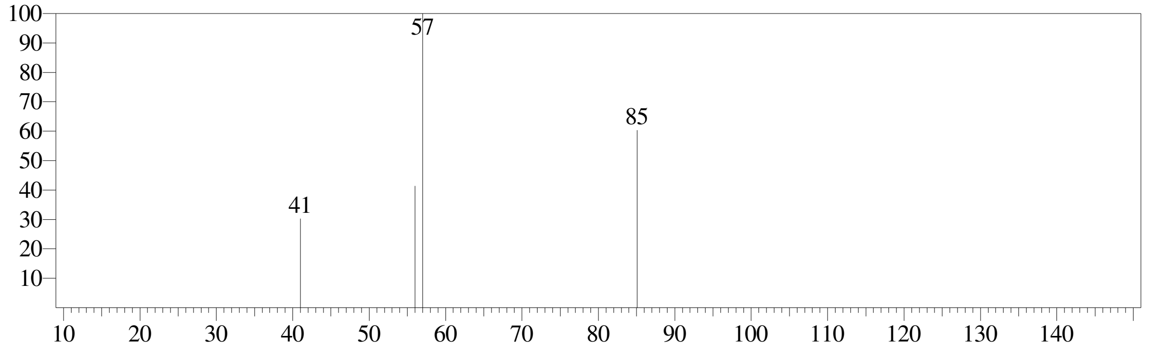

Hit#:3 Entry:13874 Library:NIST23s.lib
SI:85 Formula:C9H18O2 CAS:2445-67-2 MolWeight:158 RetIndex:1002
CompName:Butanoic acid, 2-methyl-, 2-methylpropyl ester $$ Butyric acid, 2-methyl-, isobutyl ester $$ Isobutyl 2-methy

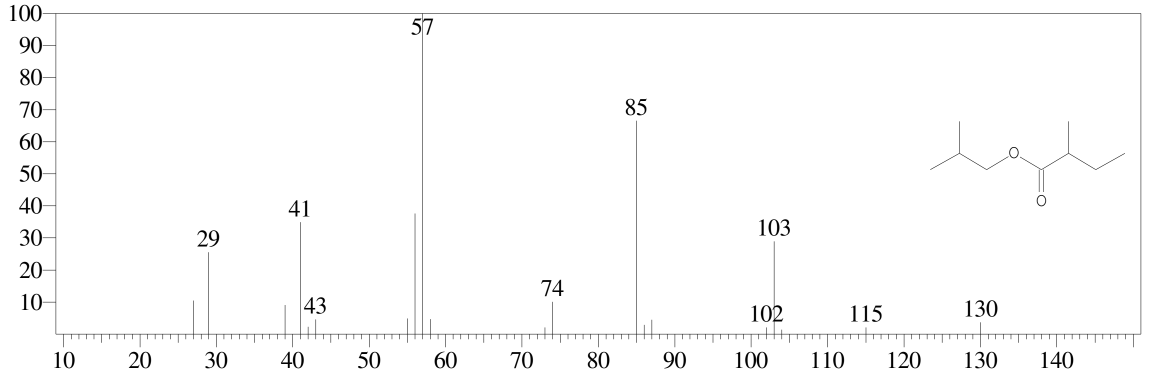

Hit#:4 Entry:14391 Library:NIST23-1.lib
SI:85 Formula:C9H18O CAS:14705-50-1 MolWeight:142 RetIndex:921
CompName:t-Butyl isobutyl ketone $$ 2,2,5-Trimethyl-3-hexanone # $$

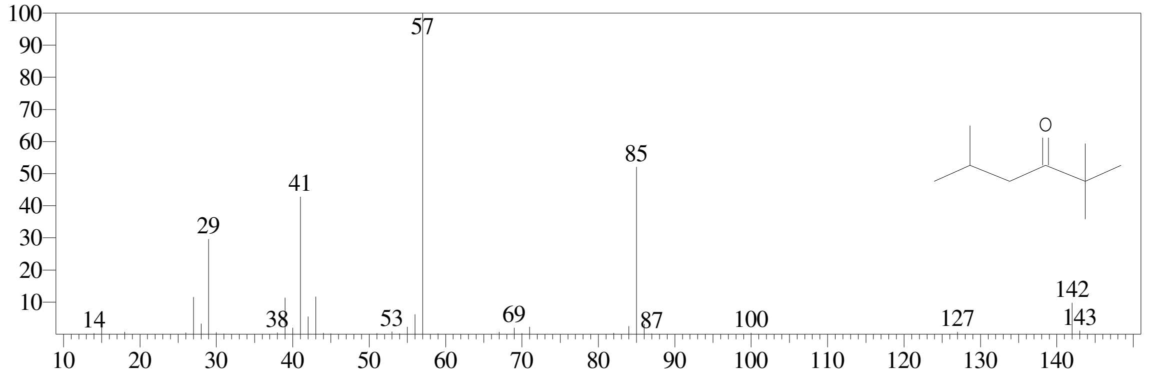

<< Target >>
Line#:6   R.Time:8.558(Scan#:728) MassPeaks:4
RawMode:Averaged 8.550-8.567(727-729) BasePeak:57.00(2633)
BG Mode:None Group 1 - Event 1 Scan

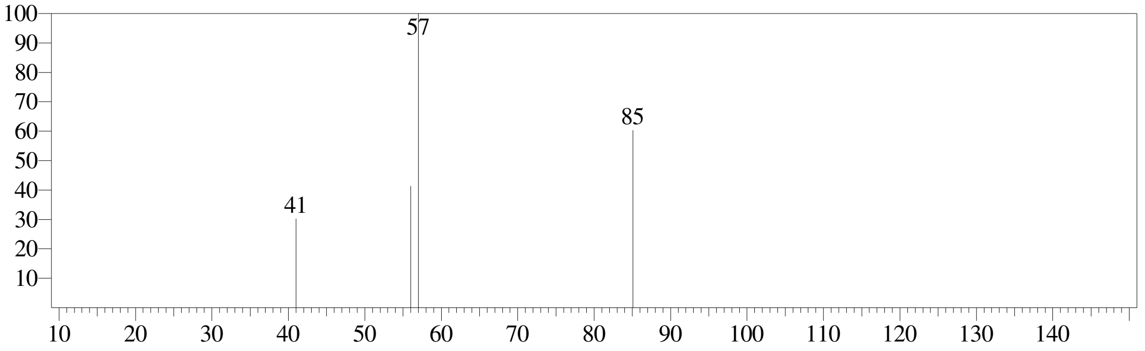

Hit#:5 Entry:13863 Library:NIST23s.lib
SI:84 Formula:C9H18O2 CAS:5129-38-4 MolWeight:158 RetIndex:931
CompName:Pivalic acid, 2-methylpropyl ester $$ iso-Butyl pivalate $$ Propanoic acid, 2,2-dimethyl, 2-methylpropyl este

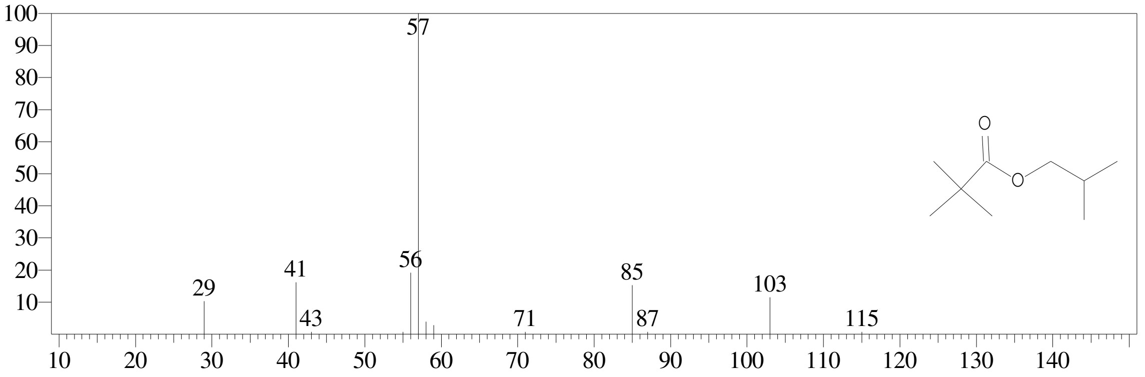

<< Target >>
Line#:7 R.Time:12.558(Scan#:1208) MassPeaks:6
RawMode:Averaged 12.550-12.567(1207-1209) BasePeak:57.05(3909)
BG Mode:None Group 1 - Event 1 Scan

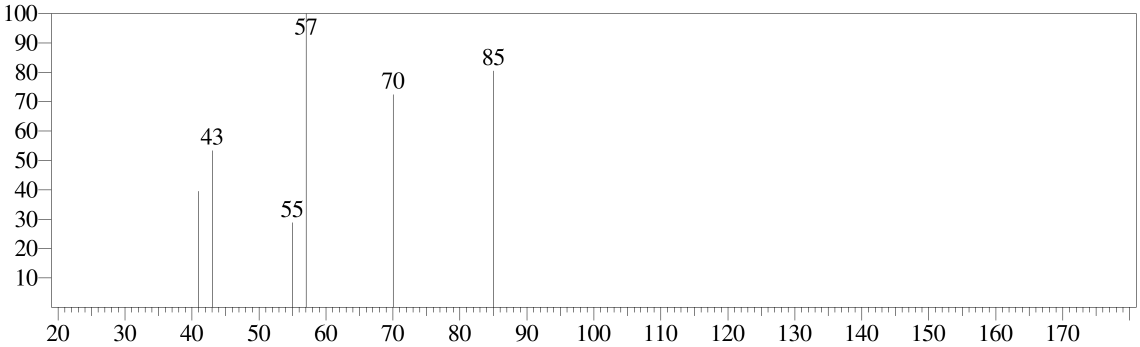

Hit#:1 Entry:17365 Library:NIST23s.lib
SI:86 Formula:C10H20O2 CAS:2445-78-5 MolWeight:172 RetIndex:1102
CompName:Butanoic acid, 2-methyl-, 2-methylbutyl ester $$ Butyric acid, 2-methyl-, 2-methylbutyl ester $$ 2-Methylbut

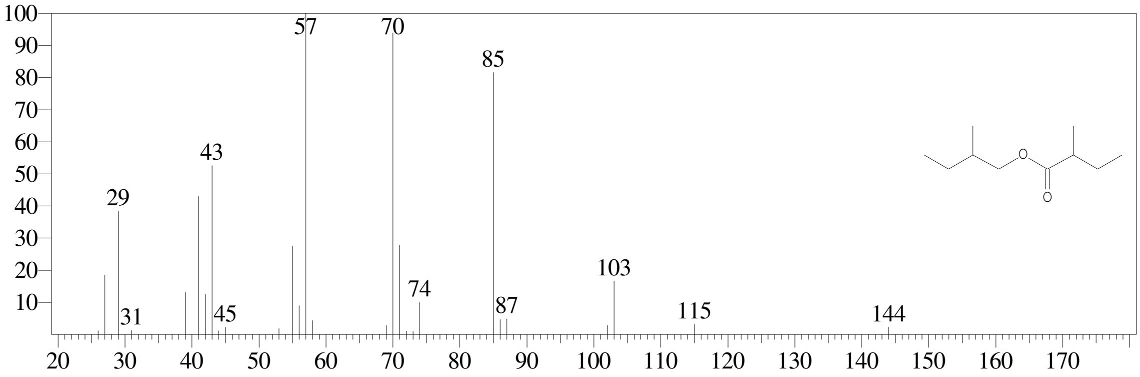

Hit#:2 Entry:17364 Library:NIST23s.lib
SI:84 Formula:C10H20O2 CAS:2445-78-5 MolWeight:172 RetIndex:1102
CompName:Butanoic acid, 2-methyl-, 2-methylbutyl ester $$ Butyric acid, 2-methyl-, 2-methylbutyl ester $$ 2-Methylbut

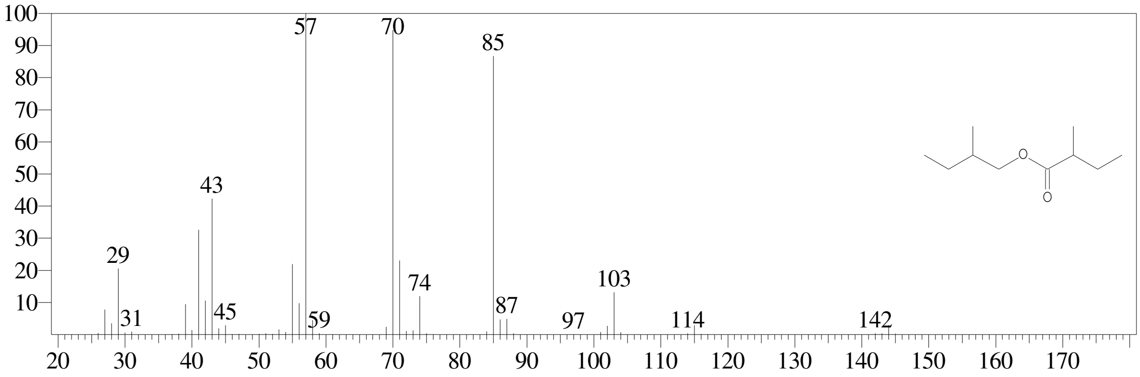

<< Target >>
Line#:7   R.Time:12.558(Scan#:1208) MassPeaks:6
RawMode:Averaged 12.550-12.567(1207-1209) BasePeak:57.05(3909)
BG Mode:None Group 1 - Event 1 Scan

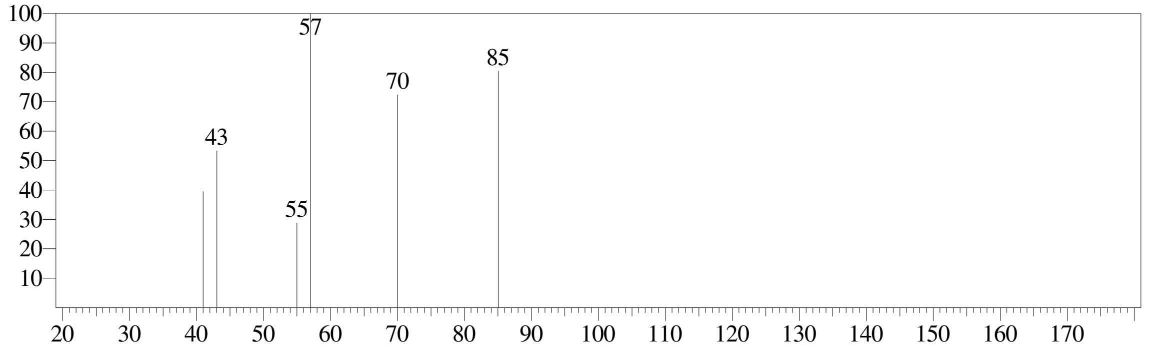

Hit#:3 Entry:17368 Library:NIST23s.lib
SI:84 Formula:C10H20O2 CAS:2445-78-5 MolWeight:172 RetIndex:1102
CompName:Butanoic acid, 2-methyl-, 2-methylbutyl ester $$ Butyric acid, 2-methyl-, 2-methylbutyl ester $$ 2-Methylbut

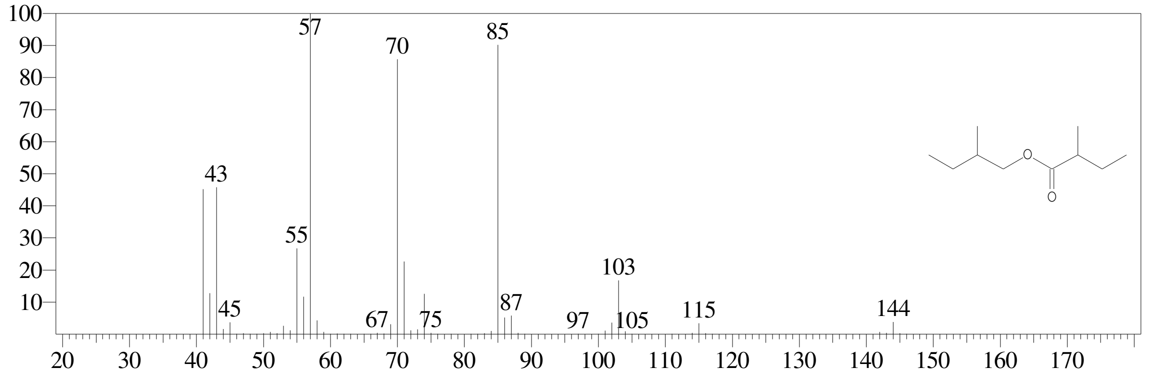

Hit#:4 Entry:5111 Library:NIST23-1.lib
SI:83 Formula:C6H12O2 CAS:68113-55-3 MolWeight:116 RetIndex:923
CompName:1-Hydroxy-4-methylpentan-2-one $$ 2-Pentanone, 1-hydroxy-4-methyl- $$

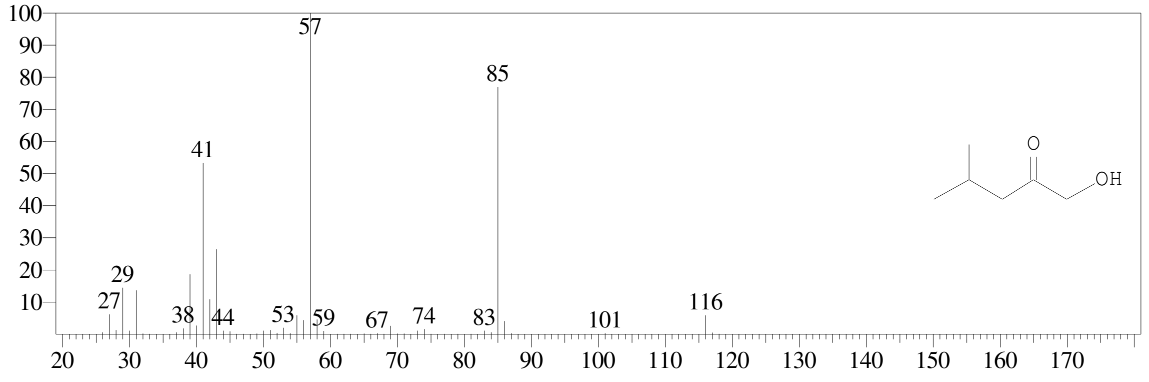

<< Target >>
Line#:7   R.Time:12.558(Scan#:1208) MassPeaks:6
RawMode:Averaged 12.550-12.567(1207-1209) BasePeak:57.05(3909)
BG Mode:None Group 1 - Event 1 Scan

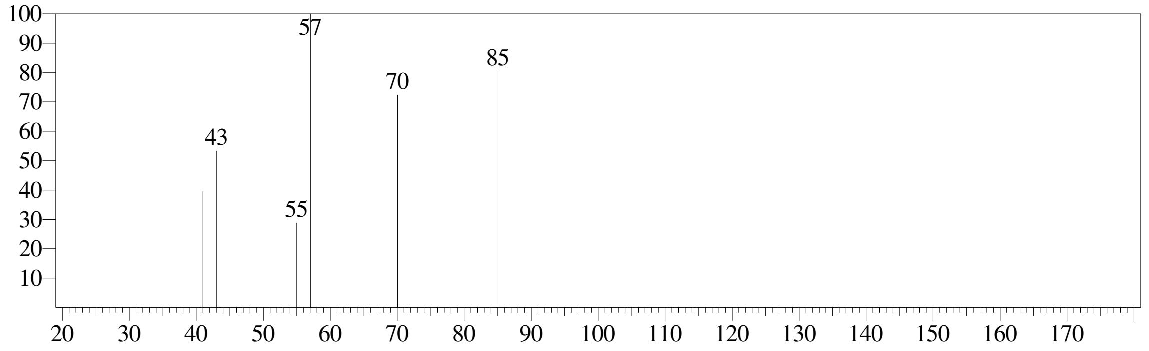

Hit#:5 Entry:33902 Library:NIST23-1.lib
SI:82 Formula:C10H20O2 CAS:2445-78-5 MolWeight:172 RetIndex:1102
CompName:Butanoic acid, 2-methyl-, 2-methylbutyl ester $$ Butyric acid, 2-methyl-, 2-methylbutyl ester $$ 2-Methylbut

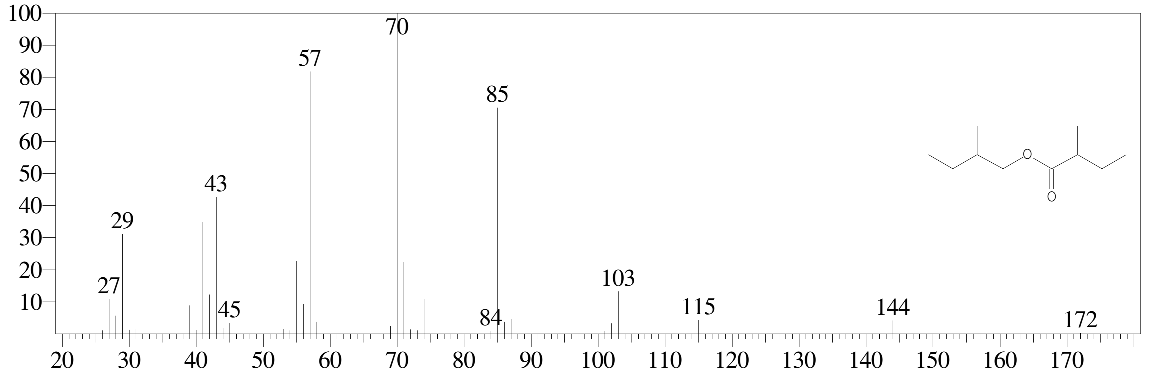

<< Target >>
Line#:8 R.Time:24.125(Scan#:2596) MassPeaks:5
RawMode:Averaged 24.117-24.133(2595-2597) BasePeak:105.05(2097)
BG Mode:None Group 1 - Event 1 Scan

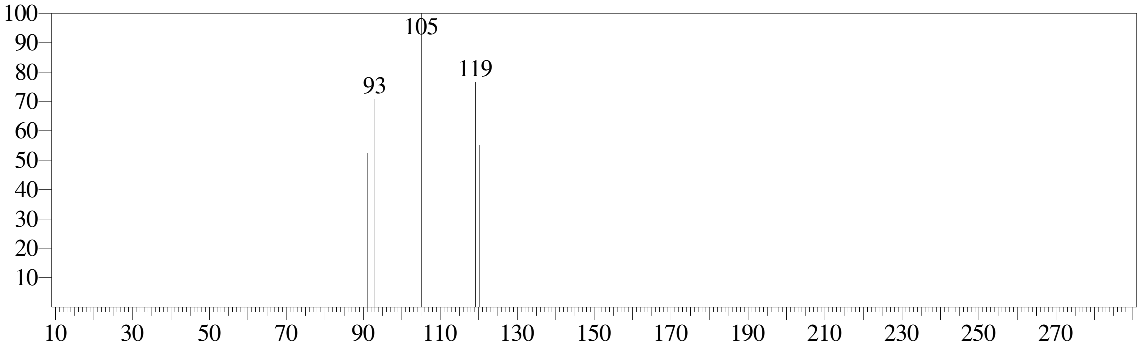

Hit#:1 Entry:160510 Library:NIST23-1.lib
SI:88 Formula:C16H13NO4 CAS:0-00-0 MolWeight:283 RetIndex:2883
CompName:2-Methyl-2-phenyl-5-(1,4-dihydropyridin-4-ylidene)-1,3-dioxan-4,6-dione $$ 2-Methyl-2-phenyl-5-(4(1H)-py

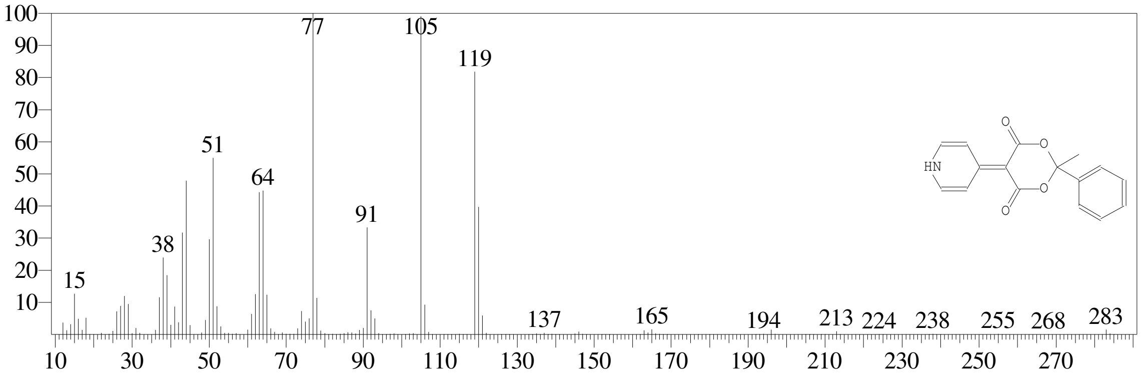

Hit#:2 Entry:5871 Library:NIST23-1.lib
SI:86 Formula:C4H12O2Si CAS:0-00-0 MolWeight:120 RetIndex:635
CompName:Ethoxy(methoxy)methylsilane

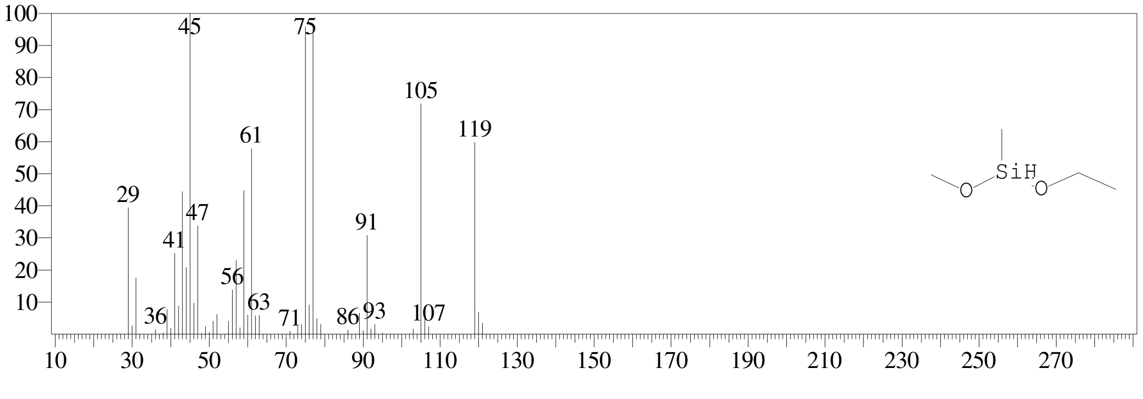

<< Target >>
Line#:8 R.Time:24.125(Scan#:2596) MassPeaks:5
RawMode:Averaged 24.117-24.133(2595-2597) BasePeak:105.05(2097)
BG Mode:None Group 1 - Event 1 Scan

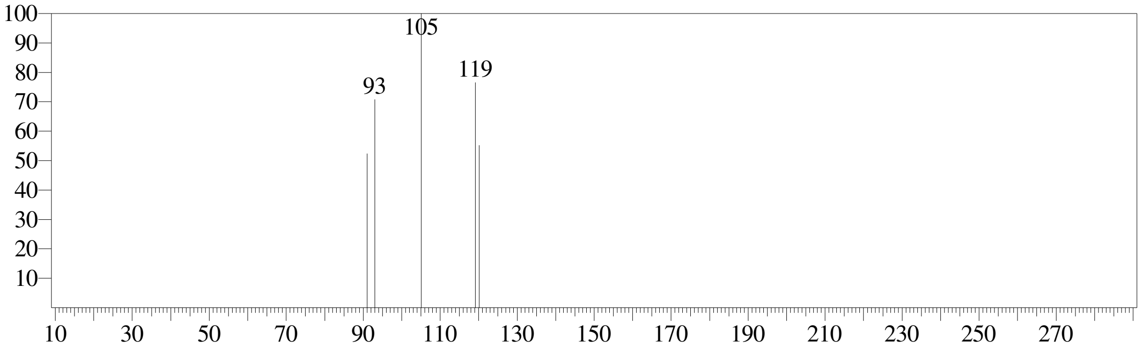

Hit#:3 Entry:5950 Library:NIST23-1.lib
SI:82 Formula:C7H8N2 CAS:13017-50-0 MolWeight:120 RetIndex:1112
CompName:Propanedinitrile, (1-methylpropylidene)- $$ 2-(1-Methylpropylidene)malononitrile # $$

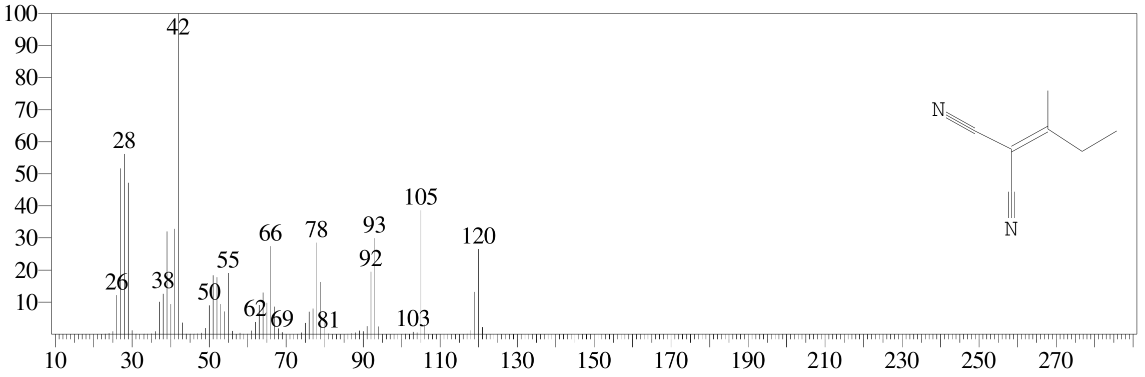

Hit#:4 Entry:5948 Library:NIST23-1.lib
SI:81 Formula:C7H8N2 CAS:6904-10-5 MolWeight:120 RetIndex:1035
CompName:1,1-Cyclopropanedicarbonitrile, 2,3-dimethyl-, trans- $$ 2,3-Dimethyl-1,1-cyclopropanedicarbonitrile-, trans-

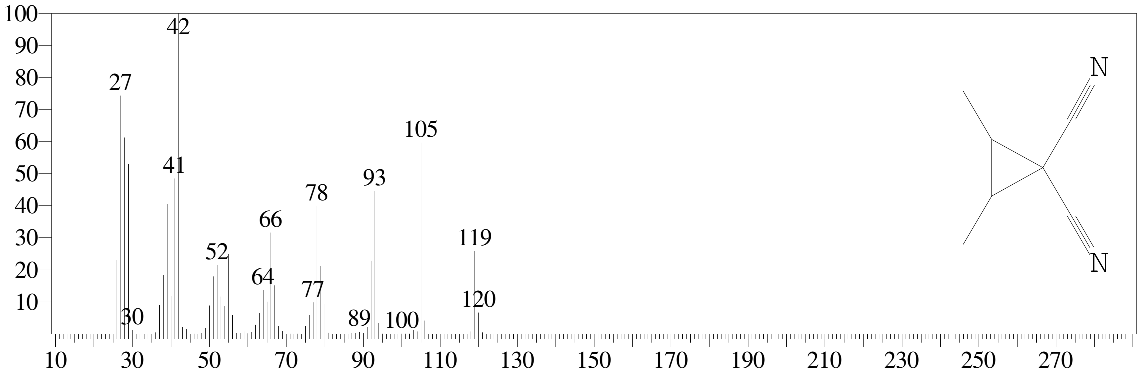

<< Target >>
Line#:8   R.Time:24.125(Scan#:2596) MassPeaks:5
RawMode:Averaged 24.117-24.133(2595-2597) BasePeak:105.05(2097)
BG Mode:None Group 1 - Event 1 Scan

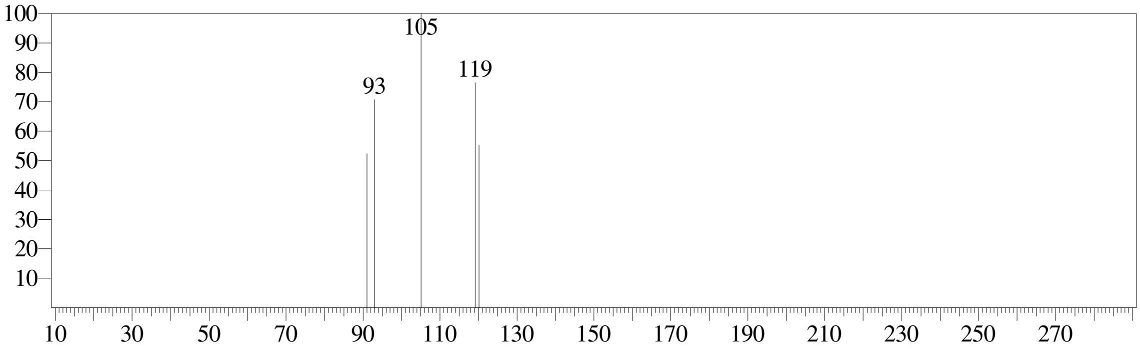

Hit#:5 Entry:67274 Library:NIST23-1.lib
SI:81 Formula:C13H20O2 CAS:3457-61-2 MolWeight:208 RetIndex:1283
CompName:Peroxide, 1,1-dimethylethyl 1-methyl-1-phenylethyl $$ tert-Butyl .alpha.,.alpha.-dimethylbenzyl peroxide $$

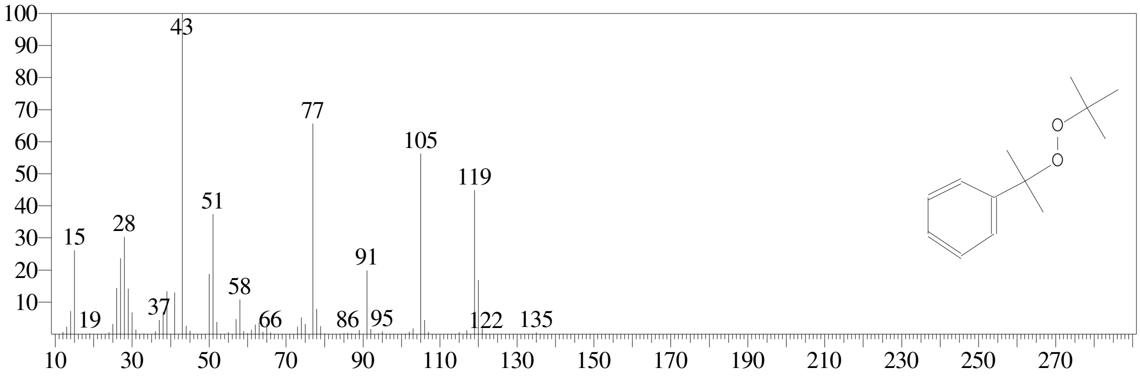

<< Target >>
Line#:9 R.Time:24.317(Scan#:2619) MassPeaks:27
RawMode:Averaged 24.308-24.325(2618-2620) BasePeak:105.05(15273)
BG Mode:None Group 1 - Event 1 Scan

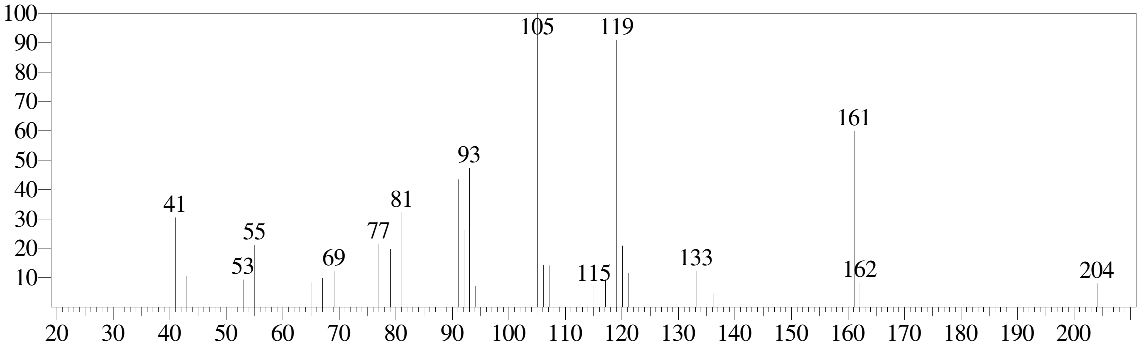

Hit#:1 Entry:24948 Library:NIST23s.lib
SI:90 Formula:C15H24 CAS:17699-14-8 MolWeight:204 RetIndex:1381
CompName:.alpha.-Cubebene $$ 1H-Cyclopenta[1,3]cyclopropa[1,2]benzene, 3a,3b,4,5,6,7-hexahydro-3,7-dimethyl-4-(1-

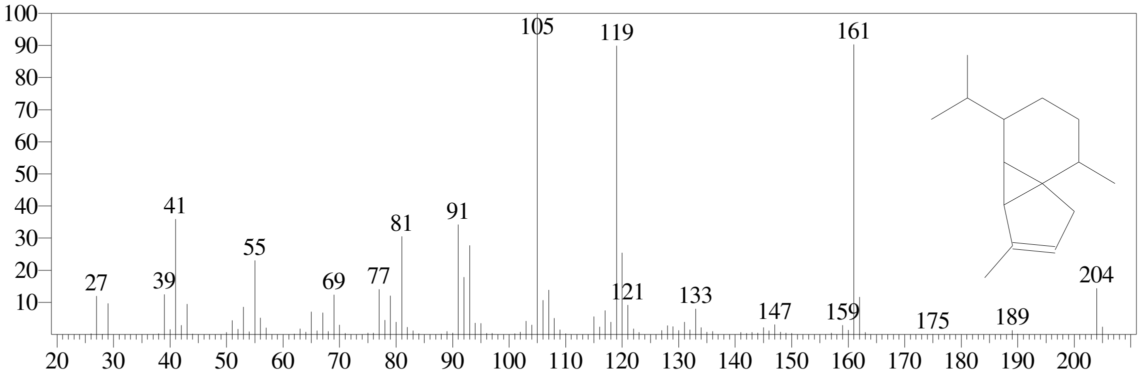

Hit#:2 Entry:25089 Library:NIST23s.lib
SI:89 Formula:C15H24 CAS:3856-25-5 MolWeight:204 RetIndex:1407
CompName:Copaene $$ Tricyclo[4.4.0.02,7]dec-3-ene, 1,3-dimethyl-8-(1-methylethyl)-, stereoisomer $$ Tricyclo[4.4.0.0

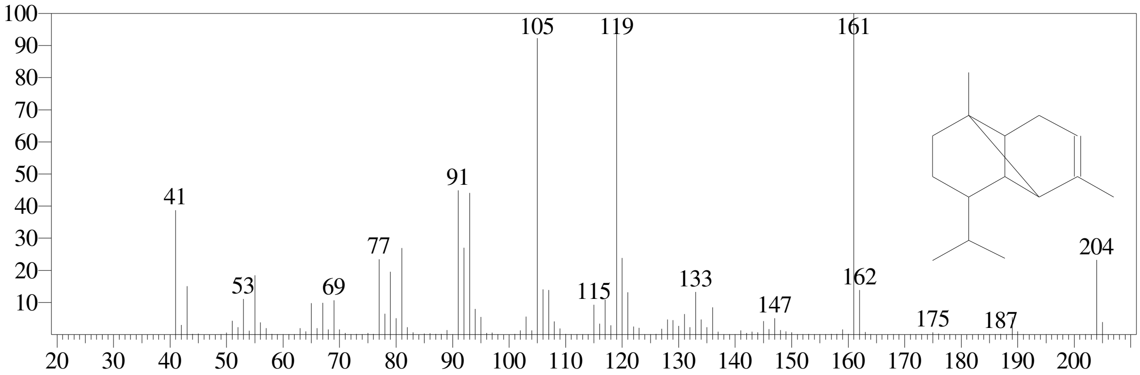

<< Target >>
Line#:9   R.Time:24.317(Scan#:2619) MassPeaks:27
RawMode:Averaged 24.308-24.325(2618-2620) BasePeak:105.05(15273)
BG Mode:None Group 1 - Event 1 Scan

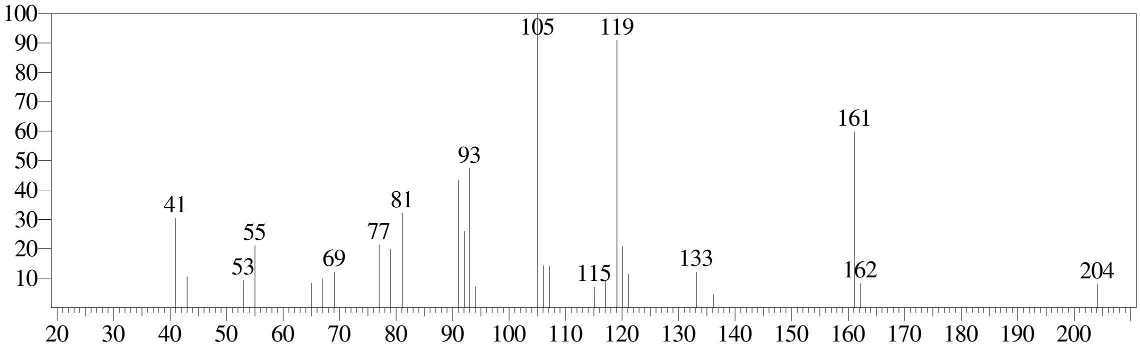

Hit#:3 Entry:62910 Library:NIST23-1.lib
SI:89 Formula:C15H24 CAS:3856-25-5 MolWeight:204 RetIndex:1407
CompName:Copaene $$ Tricyclo[4.4.0.02,7]dec-3-ene, 1,3-dimethyl-8-(1-methylethyl)-, stereoisomer $$ Tricyclo[4.4.0.0

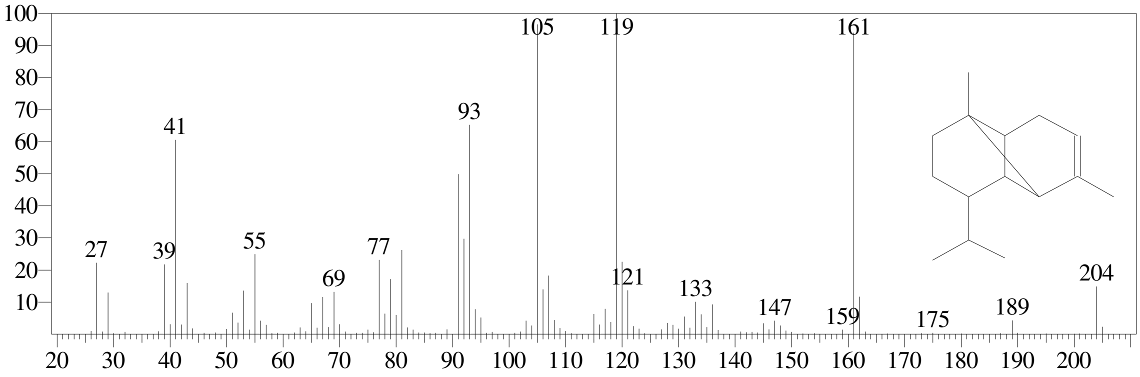

Hit#:4 Entry:25087 Library:NIST23s.lib
SI:89 Formula:C15H24 CAS:3856-25-5 MolWeight:204 RetIndex:1407
CompName:Copaene $$ Tricyclo[4.4.0.02,7]dec-3-ene, 1,3-dimethyl-8-(1-methylethyl)-, stereoisomer $$ Tricyclo[4.4.0.0

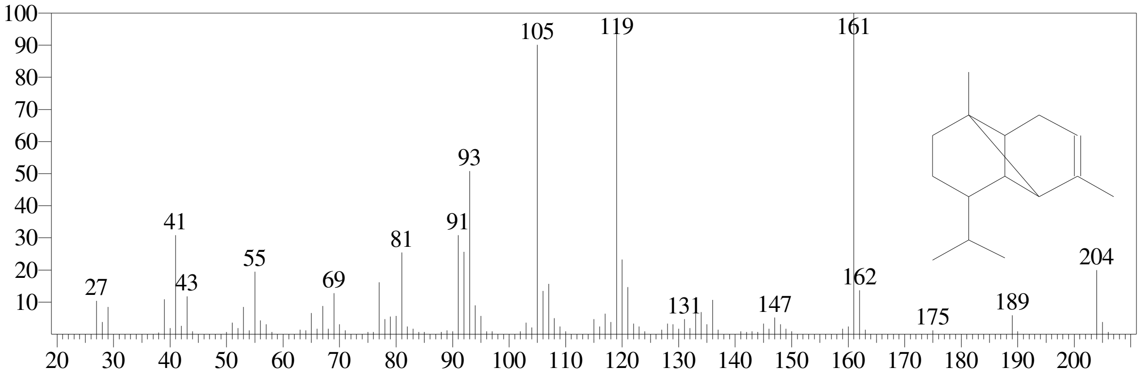

<< Target >>
Line#:9 R.Time:24.317(Scan#:2619) MassPeaks:27
RawMode:Averaged 24.308-24.325(2618-2620) BasePeak:105.05(15273)
BG Mode:None Group 1 - Event 1 Scan

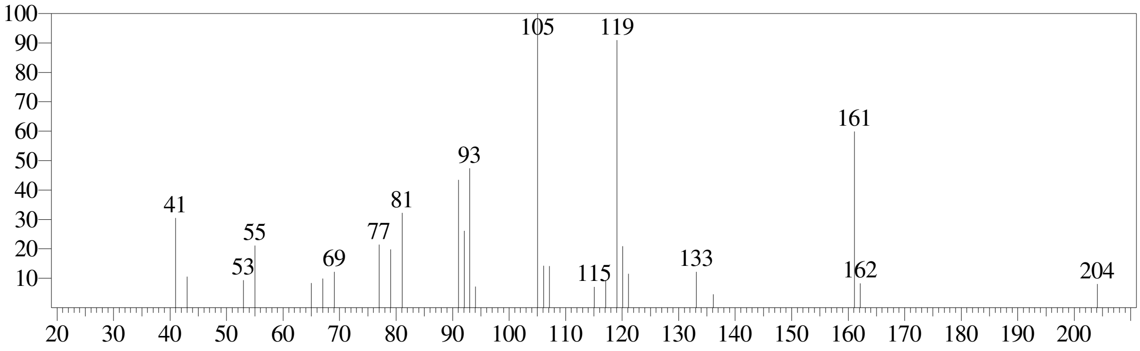

Hit#:5 Entry:24949 Library:NIST23s.lib
SI:88 Formula:C15H24 CAS:17699-14-8 MolWeight:204 RetIndex:1381
CompName:.alpha.-Cubebene $$ 1H-Cyclopenta[1,3]cyclopropa[1,2]benzene, 3a,3b,4,5,6,7-hexahydro-3,7-dimethyl-4-(1-

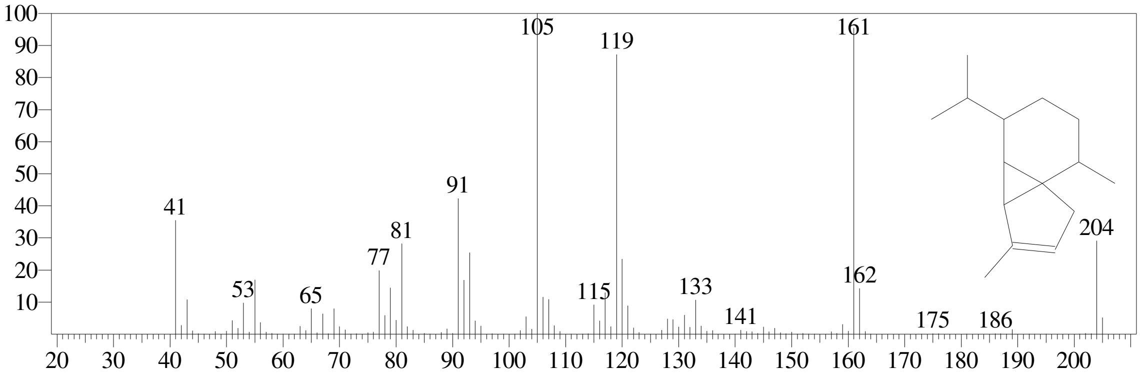

<< Target >>
Line#:10   R.Time:25.008(Scan#:2702) MassPeaks:48
RawMode:Averaged 25.000-25.017(2701-2703) BasePeak:81.05(33396)
BG Mode:None Group 1 - Event 1 Scan

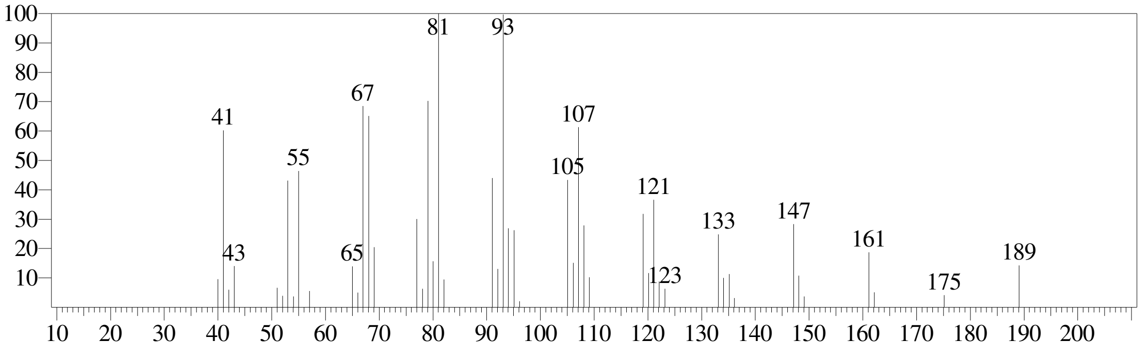

Hit#:1 Entry:24863 Library:NIST23s.lib
SI:97 Formula:C15H24 CAS:515-13-9 MolWeight:204 RetIndex:1398
CompName:Cyclohexane, 1-ethenyl-1-methyl-2,4-bis(1-methylethenyl)-, [1S-(1.alpha.,2.beta.,4.beta.)]- $$ Cyclohexane,

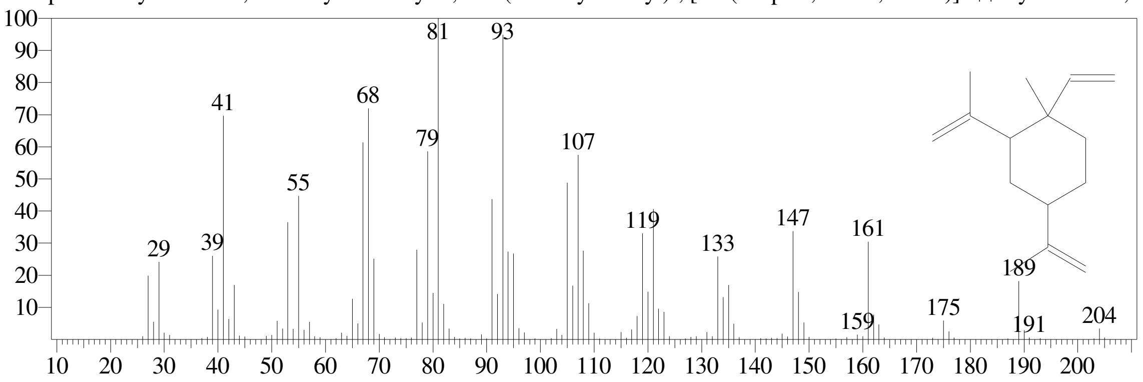

Hit#:2 Entry:24889 Library:NIST23s.lib
SI:95 Formula:C15H24 CAS:515-13-9 MolWeight:204 RetIndex:1398
CompName:Cyclohexane, 1-ethenyl-1-methyl-2,4-bis(1-methylethenyl)-, [1S-(1.alpha.,2.beta.,4.beta.)]- $$ Cyclohexane,

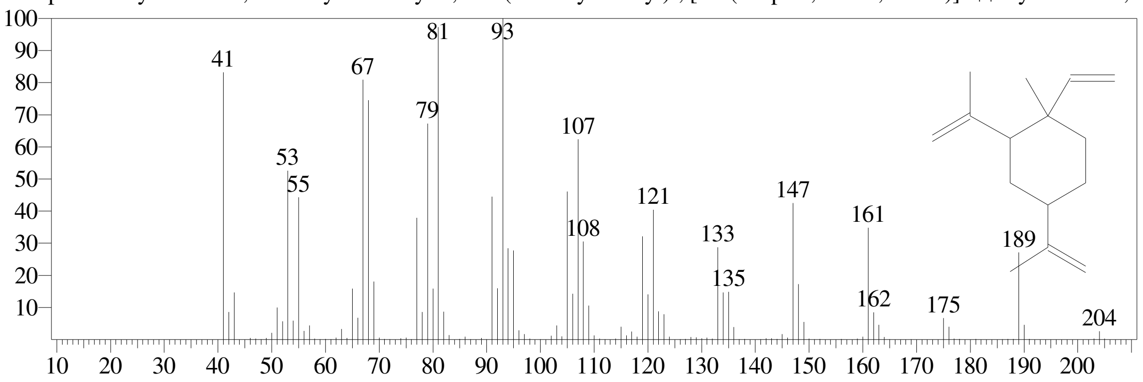

<< Target >>
Line#:10   R.Time:25.008(Scan#:2702) MassPeaks:48
RawMode:Averaged 25.000-25.017(2701-2703) BasePeak:81.05(33396)
BG Mode:None Group 1 - Event 1 Scan

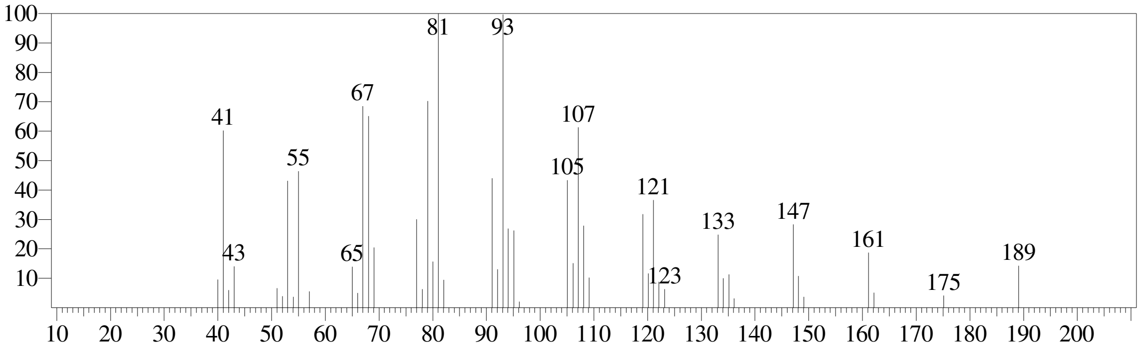

Hit#:3 Entry:62789 Library:NIST23-1.lib
SI:94 Formula:C15H24 CAS:515-13-9 MolWeight:204 RetIndex:1398
CompName:Cyclohexane, 1-ethenyl-1-methyl-2,4-bis(1-methylethenyl)-, [1S-(1.alpha.,2.beta.,4.beta.)]- $$ Cyclohexane,

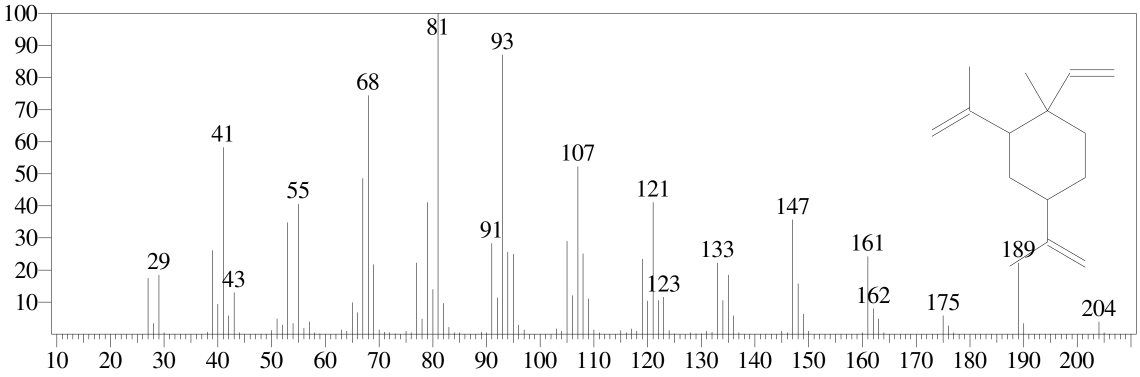

Hit#:4 Entry:62785 Library:NIST23-1.lib
SI:93 Formula:C15H24 CAS:110823-68-2 MolWeight:204 RetIndex:1398
CompName:Cyclohexane, 1-ethenyl-1-methyl-2,4-bis(1-methylethenyl)- $$ 2,4-Diisopropenyl-1-methyl-1-vinylcyclohexa

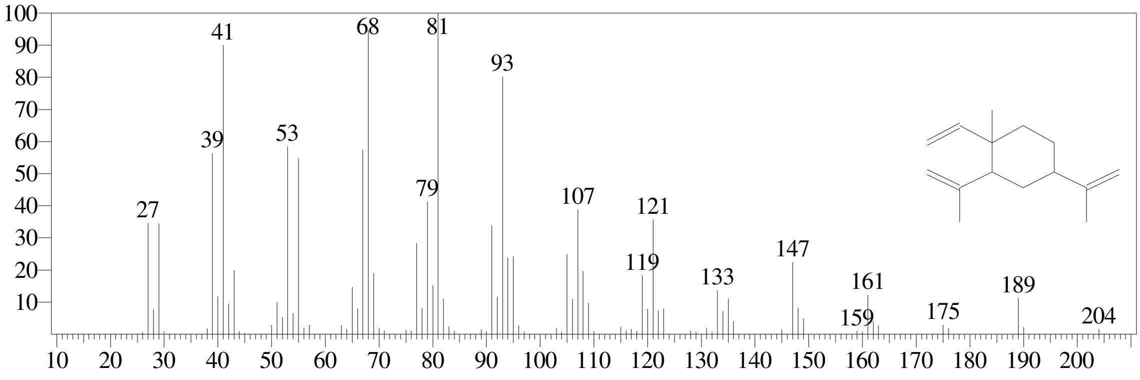

<< Target >>
Line#:10   R.Time:25.008(Scan#:2702) MassPeaks:48
RawMode:Averaged 25.000-25.017(2701-2703) BasePeak:81.05(33396)
BG Mode:None Group 1 - Event 1 Scan

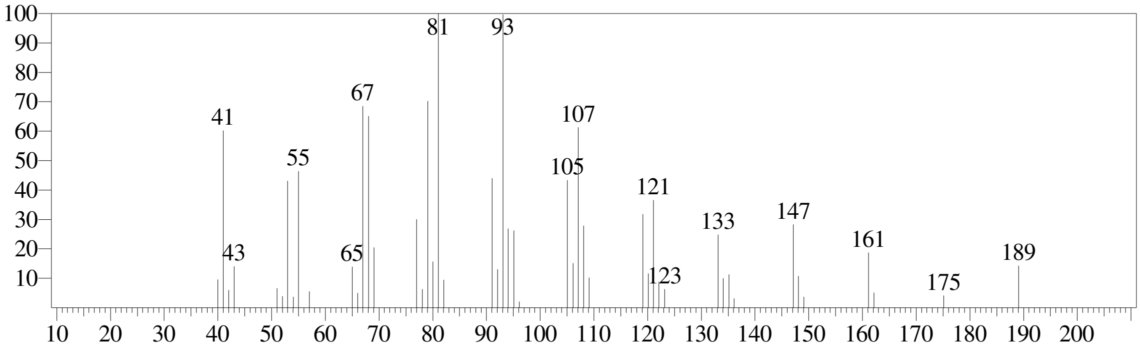

Hit#:5 Entry:24860 Library:NIST23s.lib
SI:93 Formula:C15H24 CAS:515-13-9 MolWeight:204 RetIndex:1398
CompName:Cyclohexane, 1-ethenyl-1-methyl-2,4-bis(1-methylethenyl)-, [1S-(1.alpha.,2.beta.,4.beta.)]- $$ Cyclohexane,

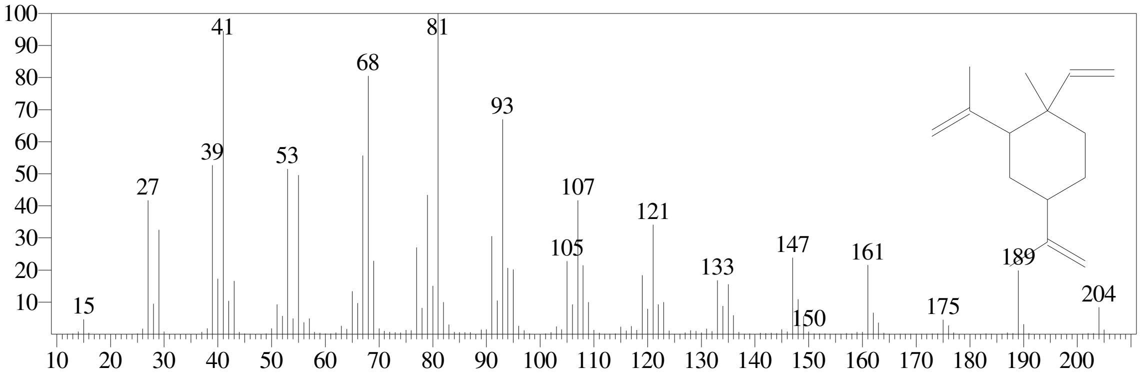

<< Target >>
Line#:11   R.Time:26.142(Scan#:2838) MassPeaks:49
RawMode:Averaged 26.133-26.150(2837-2839) BasePeak:41.00(22608)
BG Mode:None Group 1 - Event 1 Scan

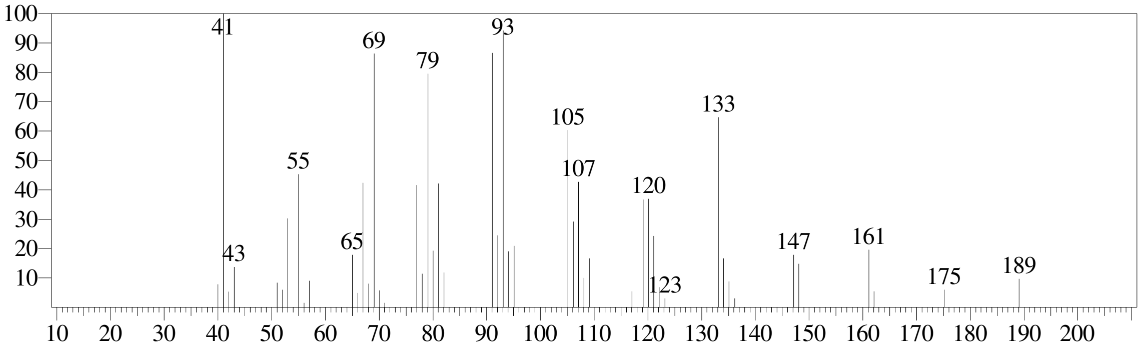

Hit#:1 Entry:24804 Library:NIST23s.lib
SI:95 Formula:C15H24 CAS:87-44-5 MolWeight:204 RetIndex:1448
CompName:Caryophyllene $$ Bicyclo[7.2.0]undec-4-ene, 4,11,11-trimethyl-8-methylene-, [1R-(1R*,4E,9S*)]- $$ Bicycl

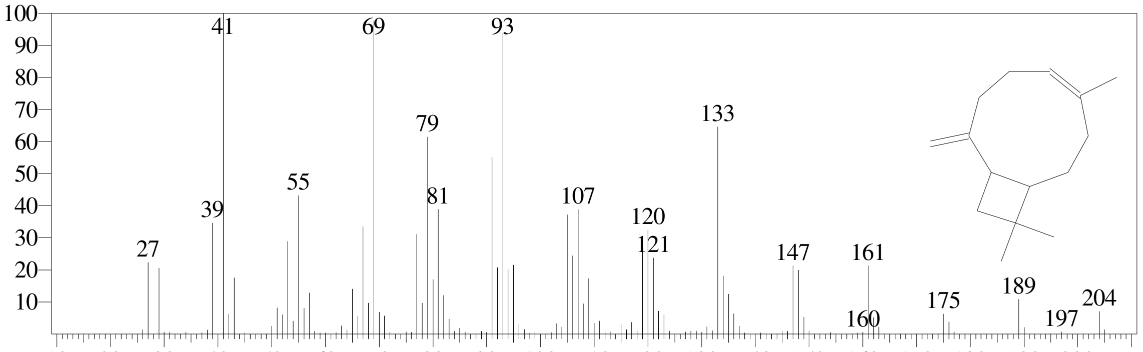

Hit#:2 Entry:62827 Library:NIST23-1.lib
SI:94 Formula:C15H24 CAS:87-44-5 MolWeight:204 RetIndex:1448
CompName:Caryophyllene $$ Bicyclo[7.2.0]undec-4-ene, 4,11,11-trimethyl-8-methylene-, [1R-(1R*,4E,9S*)]- $$ Bicycl

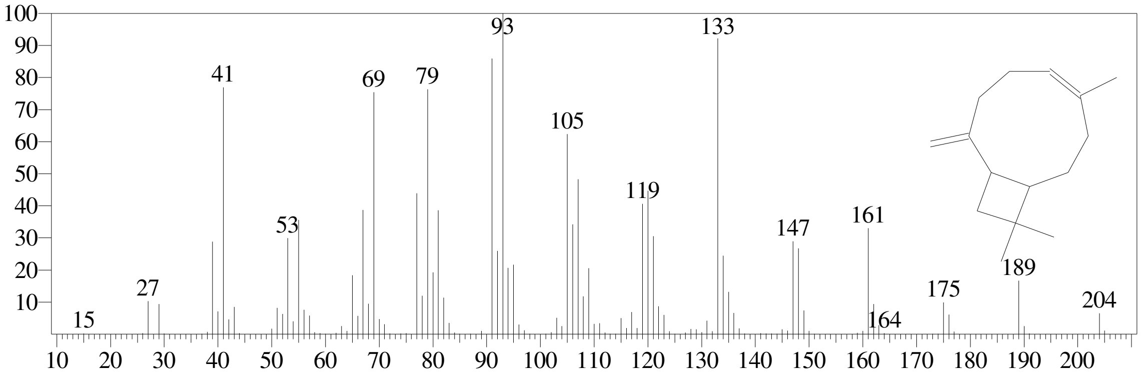

<< Target >>
Line#:11   R.Time:26.142(Scan#:2838) MassPeaks:49
RawMode:Averaged 26.133-26.150(2837-2839) BasePeak:41.00(22608)
BG Mode:None Group 1 - Event 1 Scan

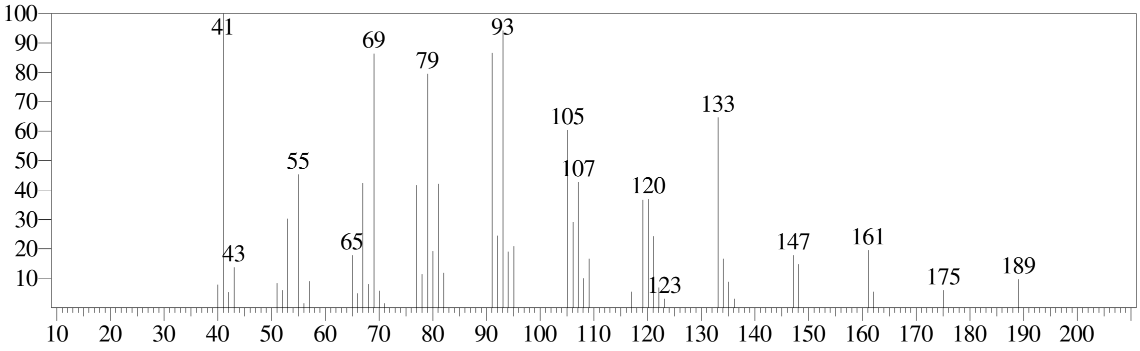

Hit#:3 Entry:24803 Library:NIST23s.lib
SI:94 Formula:C15H24 CAS:118-65-0 MolWeight:204 RetIndex:1448
CompName:Bicyclo[7.2.0]undec-4-ene, 4,11,11-trimethyl-8-methylene-,[1R-(1R*,4Z,9S*)]- $$ Isocaryophyllene $$ 4,11,

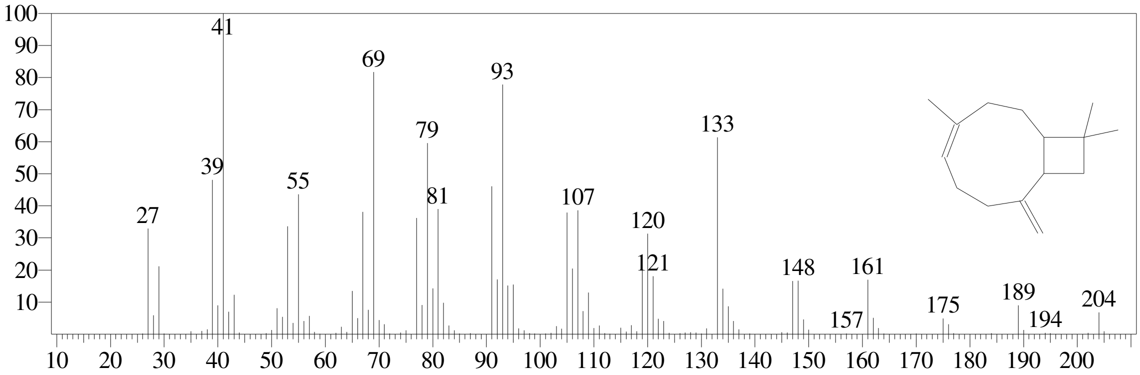

Hit#:4 Entry:24827 Library:NIST23s.lib
SI:94 Formula:C15H24 CAS:87-44-5 MolWeight:204 RetIndex:1448
CompName:Caryophyllene $$ Bicyclo[7.2.0]undec-4-ene, 4,11,11-trimethyl-8-methylene-, [1R-(1R*,4E,9S*)]- $$ Bicycl

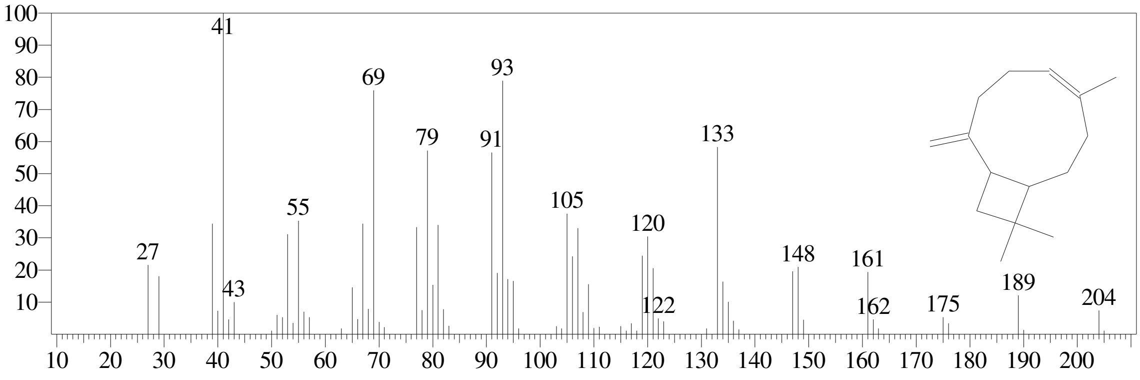

<< Target >>
Line#:11   R.Time:26.142(Scan#:2838) MassPeaks:49
RawMode:Averaged 26.133-26.150(2837-2839) BasePeak:41.00(22608)
BG Mode:None Group 1 - Event 1 Scan

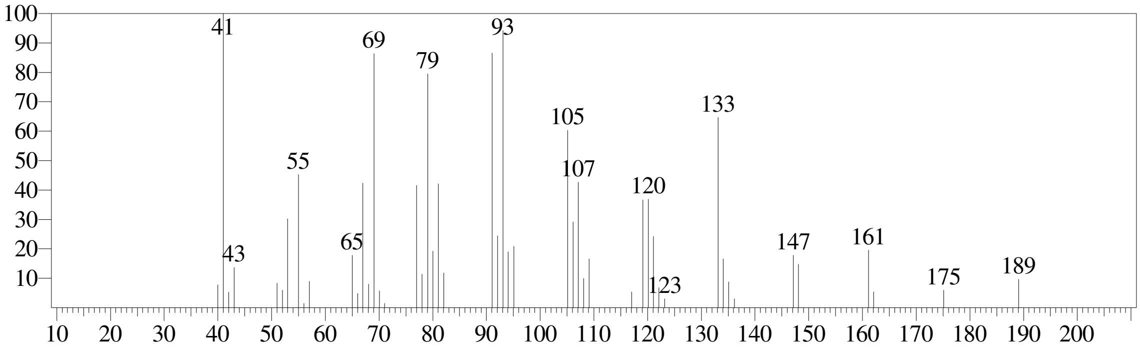

Hit#:5 Entry:62803 Library:NIST23-1.lib
SI:94 Formula:C15H24 CAS:13877-93-5 MolWeight:204 RetIndex:1448
CompName:Bicyclo[7.2.0]undec-4-ene, 4,11,11-trimethyl-8-methylene- $$ Bicyclo[7.2.0]undec-4-ene, 4,11,11-trimethyl-

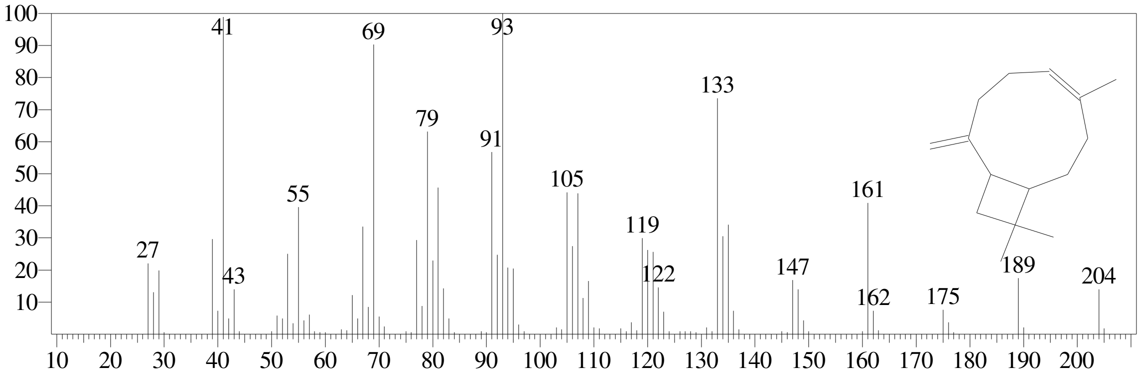

<< Target >>
Line#:12 R.Time:26.933(Scan#:2933) MassPeaks:15
RawMode:Averaged 26.925-26.942(2932-2934) BasePeak:41.00(2782)
BG Mode:None Group 1 - Event 1 Scan

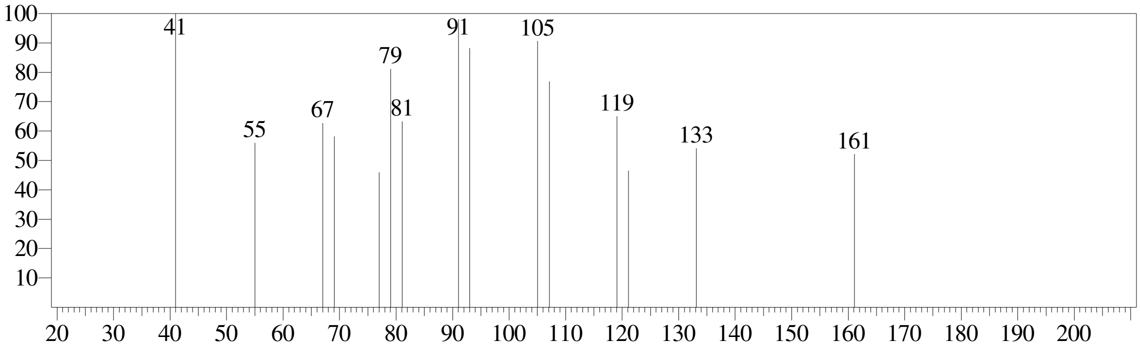

Hit#:1 Entry:24814 Library:NIST23s.lib
SI:76 Formula:C15H24 CAS:25246-27-9 MolWeight:204 RetIndex:1424
CompName:Alloaromadendrene $$ 1H-Cycloprop[e]azulene, decahydro-1,1,7-trimethyl-4-methylene-, [1aR-(1a.alpha.,4a

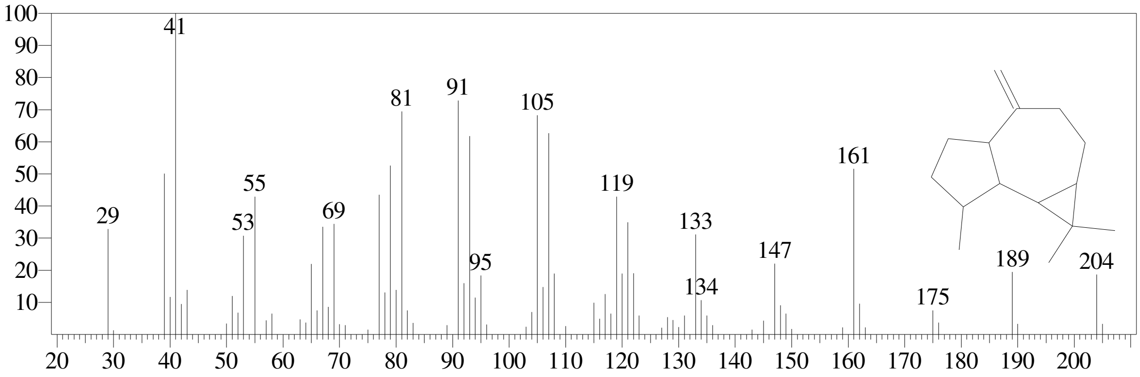

Hit#:2 Entry:62671 Library:NIST23-1.lib
SI:76 Formula:C13H20N2 CAS:0-00-0 MolWeight:204 RetIndex:1704
CompName:1,4-Methanocycloocta[d]pyridazine, 1,4,4a,5,6,9,10,10a-octahydro-11,11-dimethyl-, (1.alpha.,4.alpha.,4a.alpl

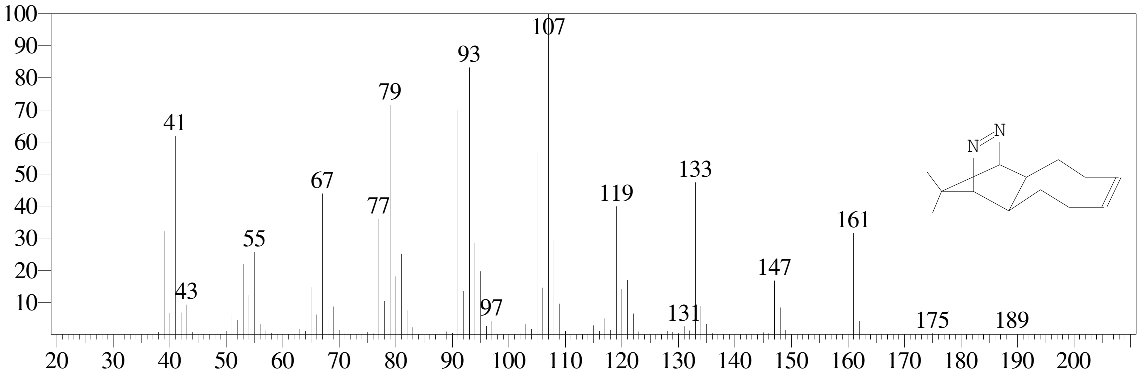

<< Target >>
Line#:12   R.Time:26.933(Scan#:2933) MassPeaks:15
RawMode:Averaged 26.925-26.942(2932-2934) BasePeak:41.00(2782)
BG Mode:None Group 1 - Event 1 Scan

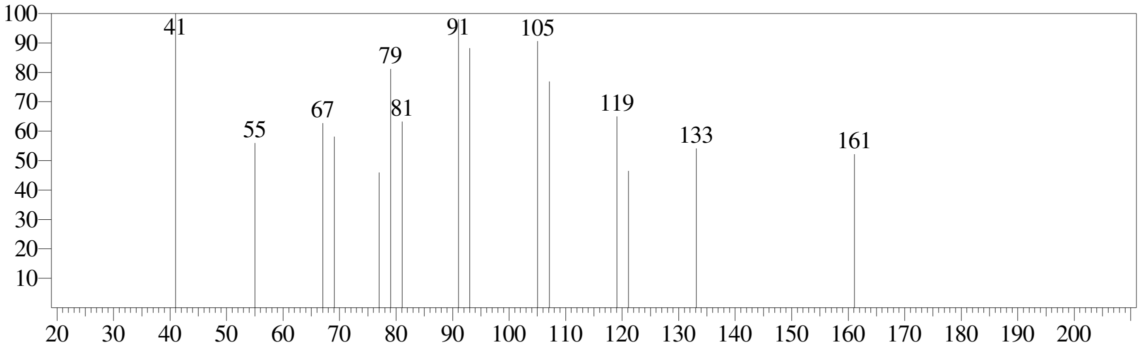

Hit#:3 Entry:62757 Library:NIST23-1.lib
SI:76 Formula:C15H24 CAS:28973-99-1 MolWeight:204 RetIndex:1509
CompName:(Z,Z)-.alpha.-Farnesene $$ (3Z,6Z)-3,7,11-Trimethyl-1,3,6,10-dodecatetraene # $$

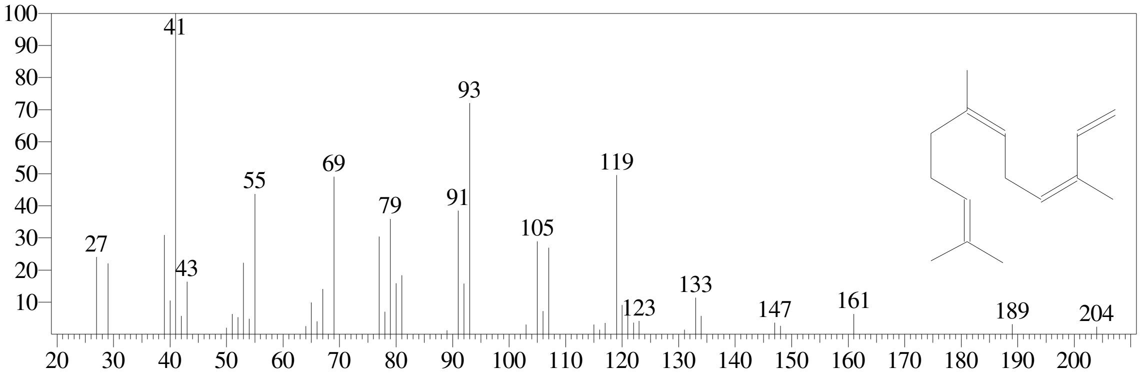

Hit#:4 Entry:25046 Library:NIST23s.lib
SI:75 Formula:C15H24 CAS:23986-74-5 MolWeight:204 RetIndex:1478
CompName:Germacrene D $$ (S,1Z,6Z)-8-Isopropyl-1-methyl-5-methylenecyclodeca-1,6-diene $$ D-Germacrene $$ 1(1

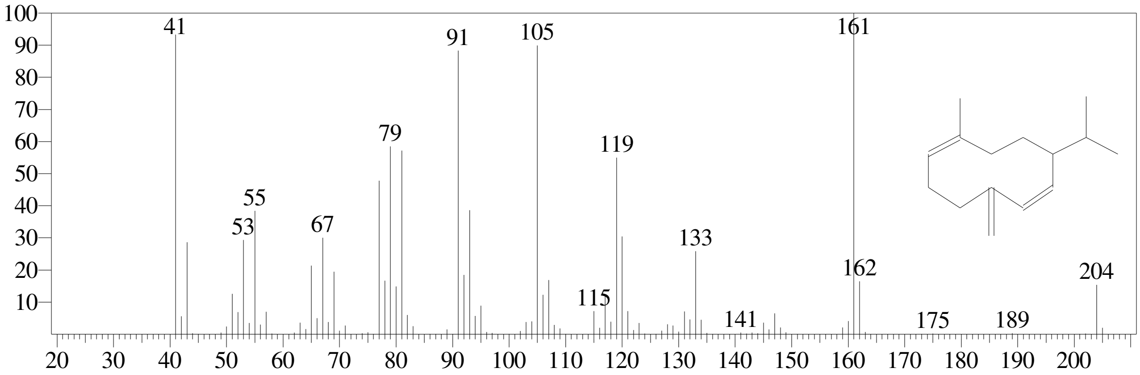

<< Target >>
Line#:12   R.Time:26.933(Scan#:2933) MassPeaks:15
RawMode:Averaged 26.925-26.942(2932-2934) BasePeak:41.00(2782)
BG Mode:None Group 1 - Event 1 Scan

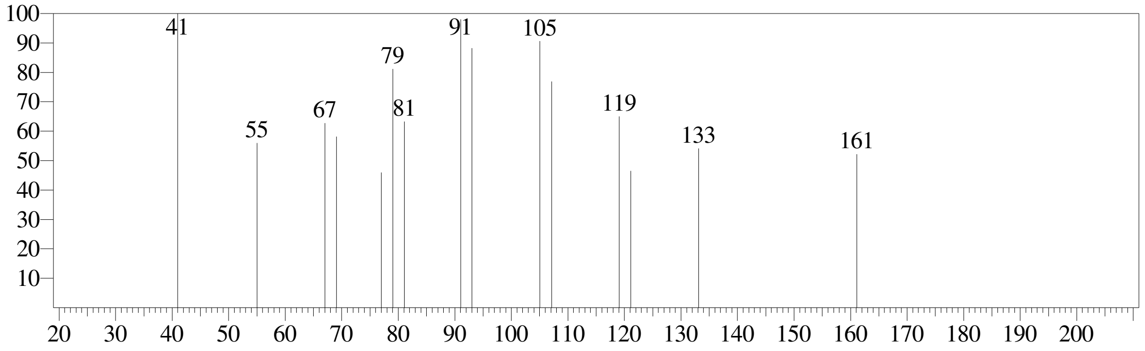

Hit#:5 Entry:24831 Library:NIST23s.lib
SI:75 Formula:C15H24 CAS:26560-14-5 MolWeight:204 RetIndex:1509
CompName:1,3,6,10-Dodecatetraene, 3,7,11-trimethyl-, (Z,E)- $$ (Z,E)-.alpha.-Farnesene $$ (3Z,6E)-3,7,11-Trimethyl-1.

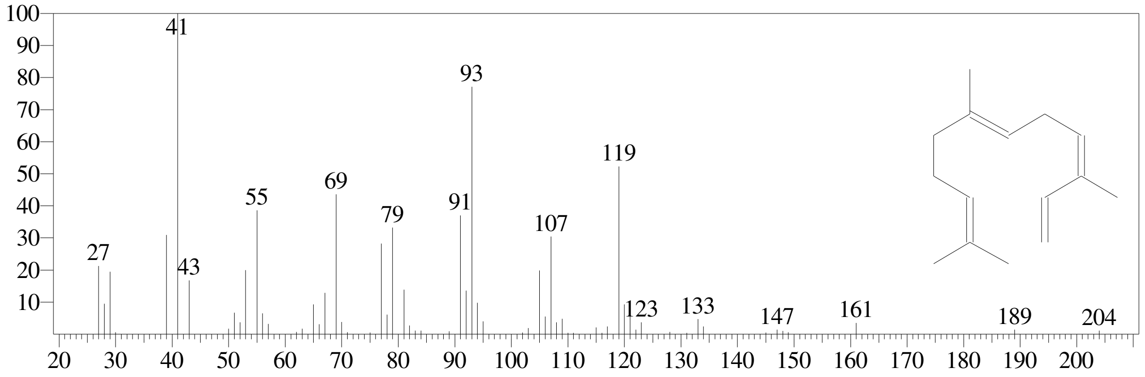

<< Target >>
Line#:13   R.Time:27.533(Scan#:3005) MassPeaks:26
RawMode:Averaged 27.525-27.542(3004-3006) BasePeak:93.05(29701)
BG Mode:None Group 1 - Event 1 Scan

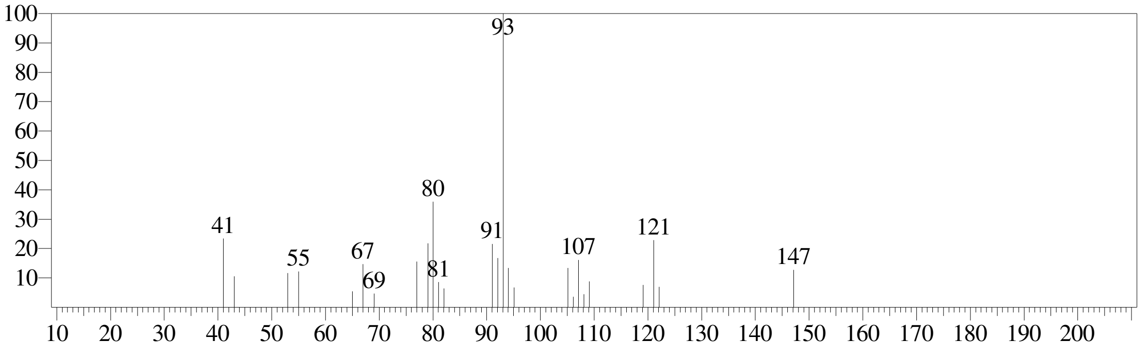

Hit#:1 Entry:24887 Library:NIST23s.lib
SI:92 Formula:C15H24 CAS:6753-98-6 MolWeight:204 RetIndex:1455
CompName:Humulene $$ .alpha.-Caryophyllene $$ 1,4,8-Cycloundecatriene, 2,6,6,9-tetramethyl-, (E,E,E)- $$ .alpha.-Hu

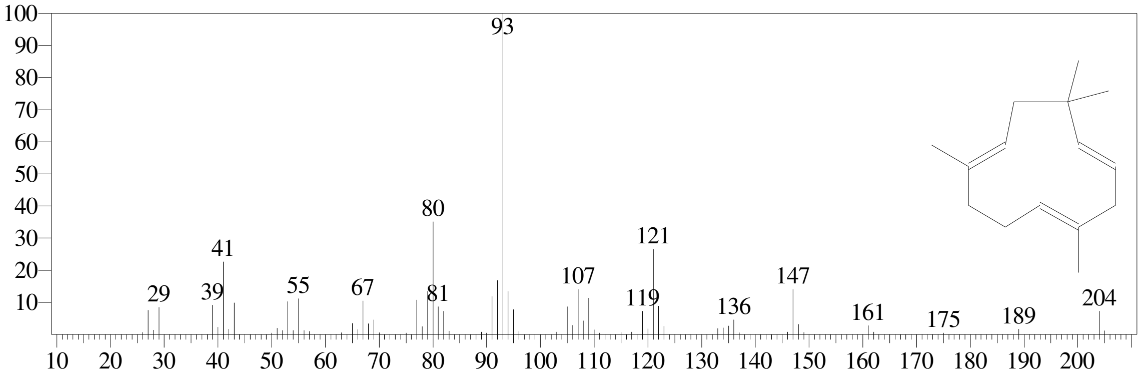

Hit#:2 Entry:62814 Library:NIST23-1.lib
SI:91 Formula:C15H24 CAS:6753-98-6 MolWeight:204 RetIndex:1455
CompName:Humulene $$ .alpha.-Caryophyllene $$ 1,4,8-Cycloundecatriene, 2,6,6,9-tetramethyl-, (E,E,E)- $$ .alpha.-Hu

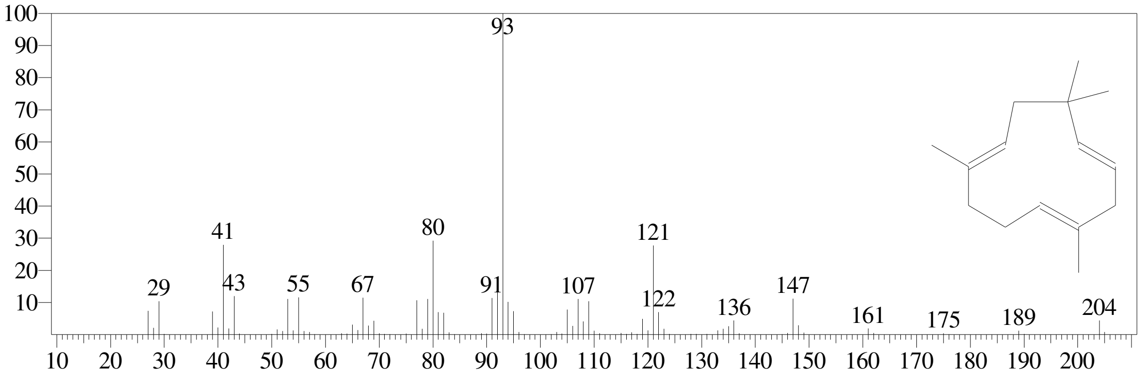

<< Target >>
Line#:13 R.Time:27.533(Scan#:3005) MassPeaks:26
RawMode:Averaged 27.525-27.542(3004-3006) BasePeak:93.05(29701)
BG Mode:None Group 1 - Event 1 Scan

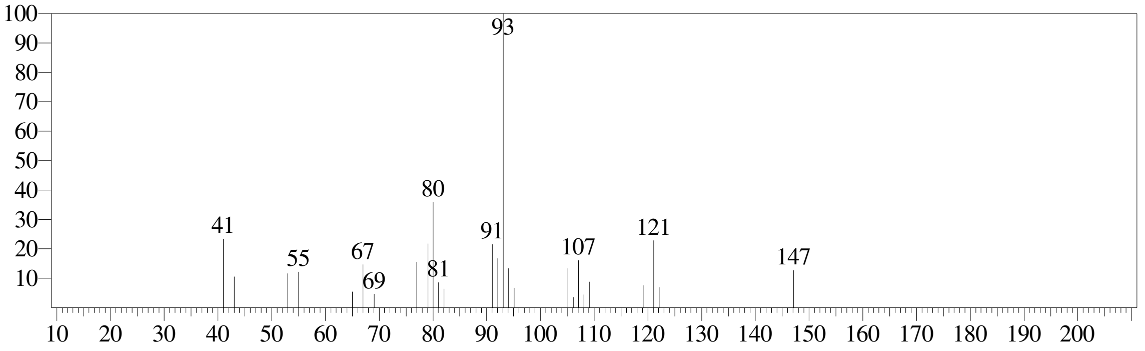

Hit#:3 Entry:24888 Library:NIST23s.lib
SI:91 Formula:C15H24 CAS:6753-98-6 MolWeight:204 RetIndex:1455
CompName:Humulene $$ .alpha.-Caryophyllene $$ 1,4,8-Cycloundecatriene, 2,6,6,9-tetramethyl-, (E,E,E)- $$ .alpha.-Hu

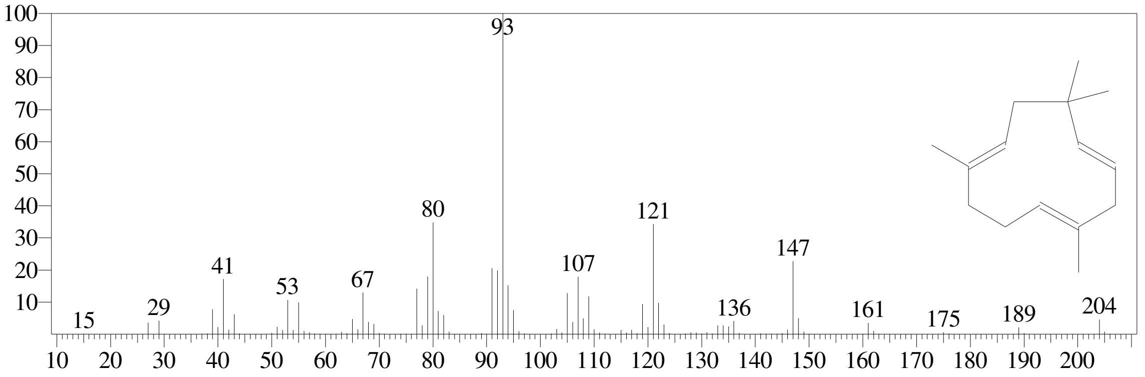

Hit#:4 Entry:24886 Library:NIST23s.lib
SI:90 Formula:C15H24 CAS:6753-98-6 MolWeight:204 RetIndex:1455
CompName:Humulene $$ .alpha.-Caryophyllene $$ 1,4,8-Cycloundecatriene, 2,6,6,9-tetramethyl-, (E,E,E)- $$ .alpha.-Hu

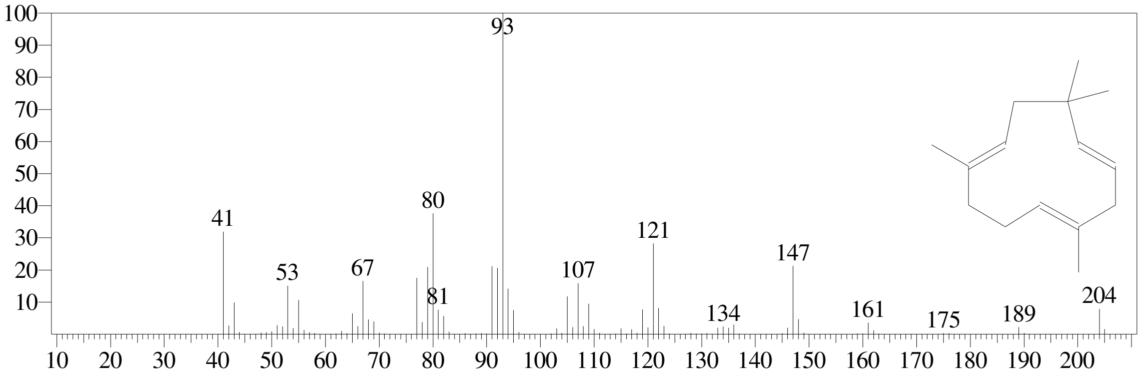

<< Target >>
Line#:13 R.Time:27.533(Scan#:3005) MassPeaks:26
RawMode:Averaged 27.525-27.542(3004-3006) BasePeak:93.05(29701)
BG Mode:None Group 1 - Event 1 Scan

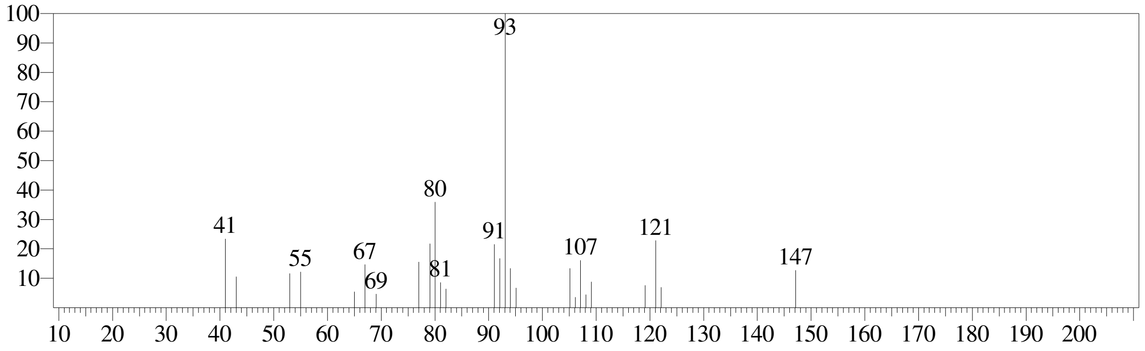

Hit#:5 Entry:62816 Library:NIST23-1.lib
SI:89 Formula:C15H24 CAS:0-00-0 MolWeight:204 RetIndex:1463
CompName:1,4,7,-Cycloundecatriene, 1,5,9,9-tetramethyl-, Z,Z,Z- $$ 1,5,9,9-Tetramethyl-1,4,7-cycloundecatriene # $$

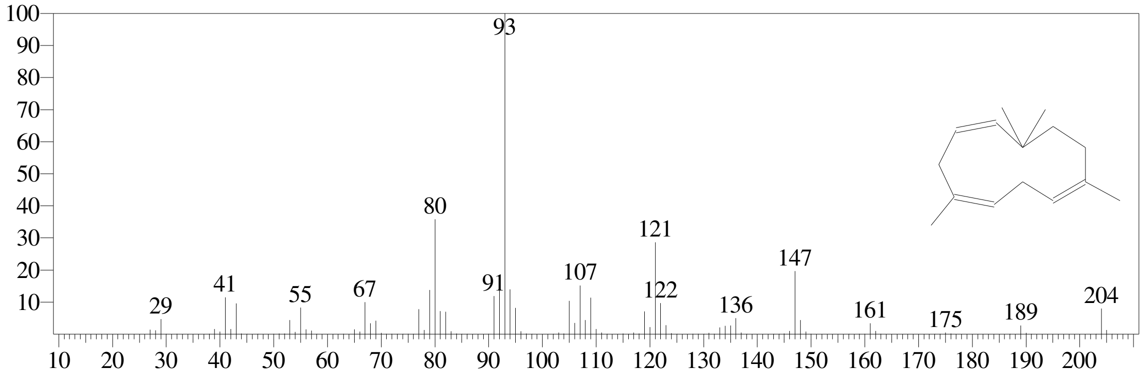

<< Target >>
Line#:14 R.Time:28.450(Scan#:3115) MassPeaks:28
RawMode:Averaged 28.442-28.458(3114-3116) BasePeak:189.10(5819)
BG Mode:Calc. from Peak Group 1 - Event 1 Scan

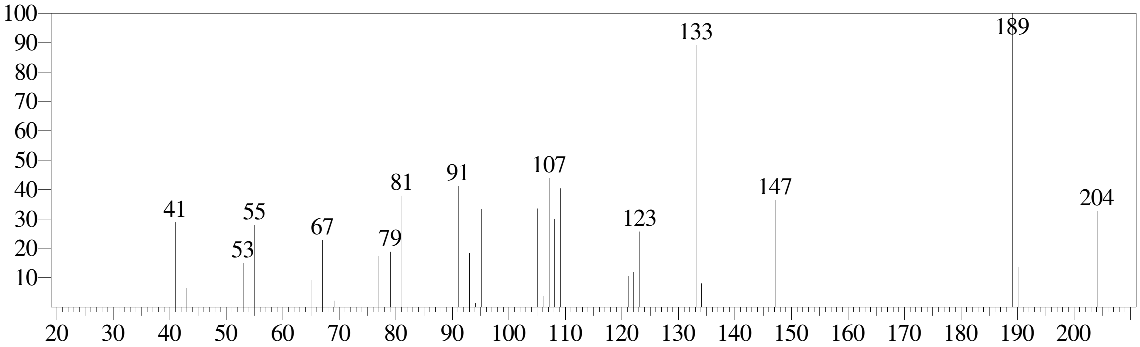

Hit#:1 Entry:63039 Library:NIST23-1.lib
SI:86 Formula:C15H24 CAS:103827-22-1 MolWeight:204 RetIndex:1489
CompName:4a,8-Dimethyl-2-(prop-1-en-2-yl)-1,2,3,4,4a,5,6,7-octahydronaphthalene $$ Naphthalene, 1,2,3,4,4a,5,6,7-oct

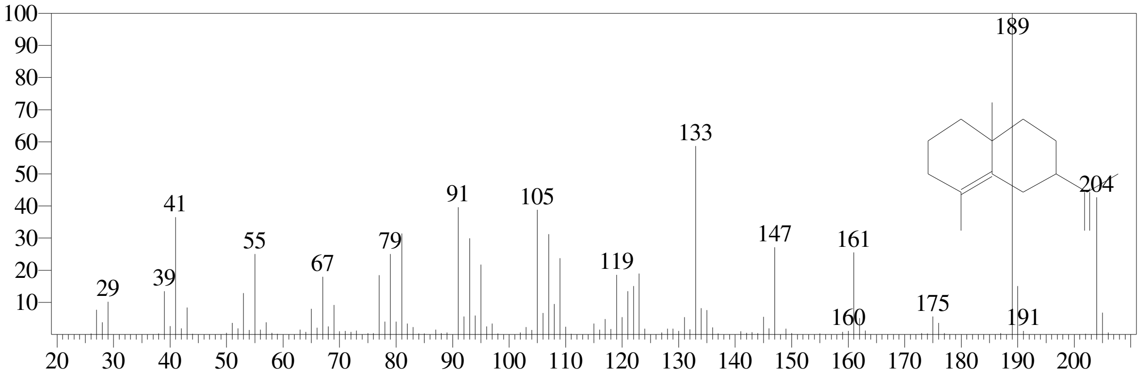

Hit#:2 Entry:25125 Library:NIST23s.lib
SI:86 Formula:C15H24 CAS:103827-22-1 MolWeight:204 RetIndex:1489
CompName:4a,8-Dimethyl-2-(prop-1-en-2-yl)-1,2,3,4,4a,5,6,7-octahydronaphthalene $$ Naphthalene, 1,2,3,4,4a,5,6,7-oct

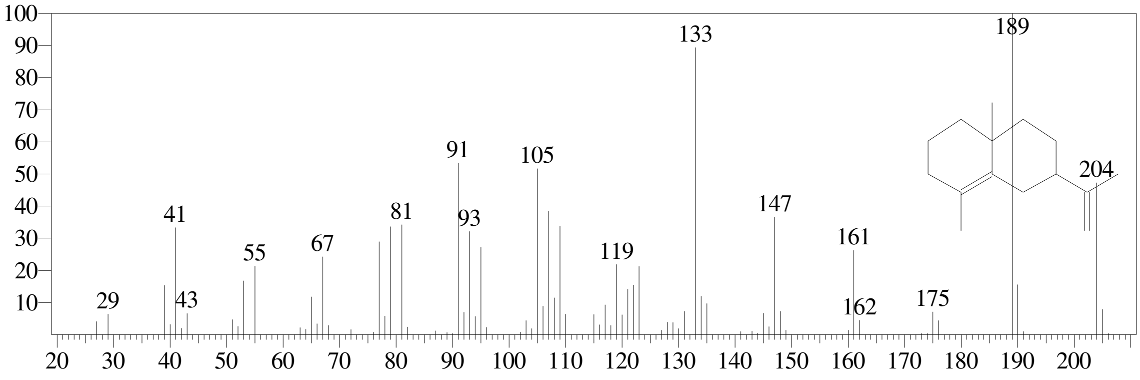

<< Target >>
Line#:14 R.Time:28.450(Scan#:3115) MassPeaks:28
RawMode:Averaged 28.442-28.458(3114-3116) BasePeak:189.10(5819)
BG Mode:Calc. from Peak Group 1 - Event 1 Scan

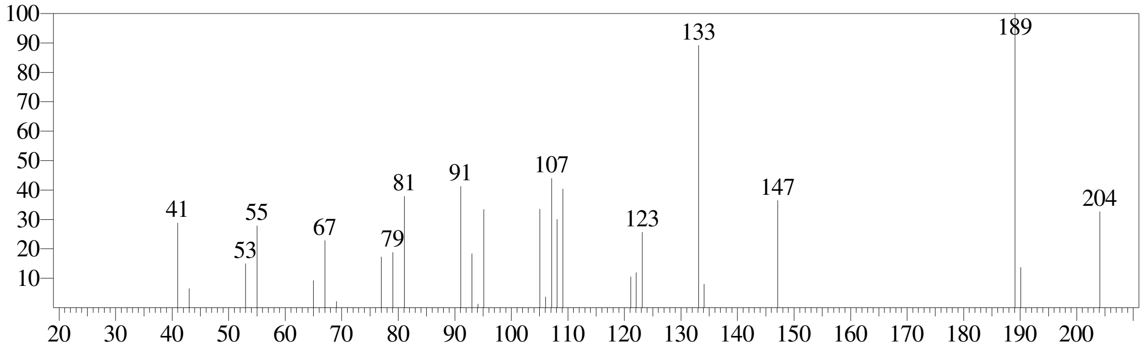

Hit#:3 Entry:63040 Library:NIST23-1.lib
SI:81 Formula:C15H24 CAS:515-17-3 MolWeight:204 RetIndex:1527
CompName:Naphthalene, decahydro-4a-methyl-1-methylene-7-(1-methylethylidene)-, (4aR-trans)- $$ Eudesma-4(14),7(1

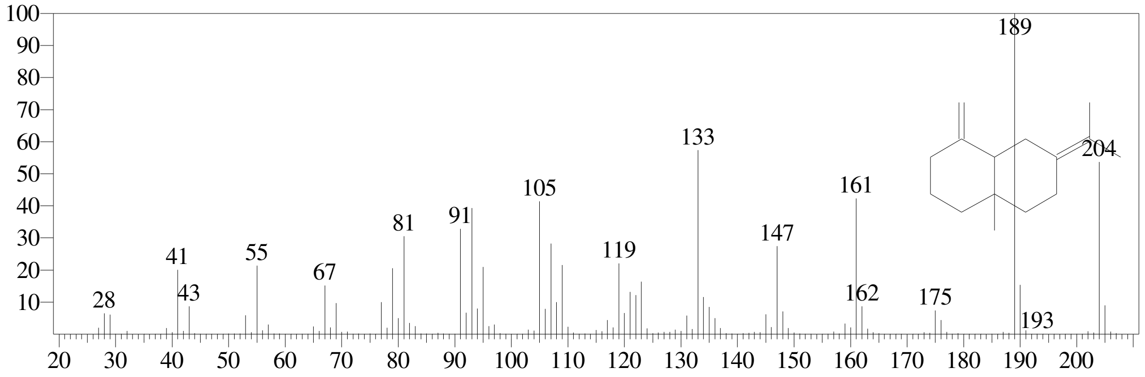

Hit#:4 Entry:25133 Library:NIST23s.lib
SI:81 Formula:C15H24 CAS:473-13-2 MolWeight:204 RetIndex:1484
CompName:Naphthalene, 1,2,3,4,4a,5,6,8a-octahydro-4a,8-dimethyl-2-(1-methylethenyl)-, [2R-(2.alpha.,4a.alpha.,8a.beta

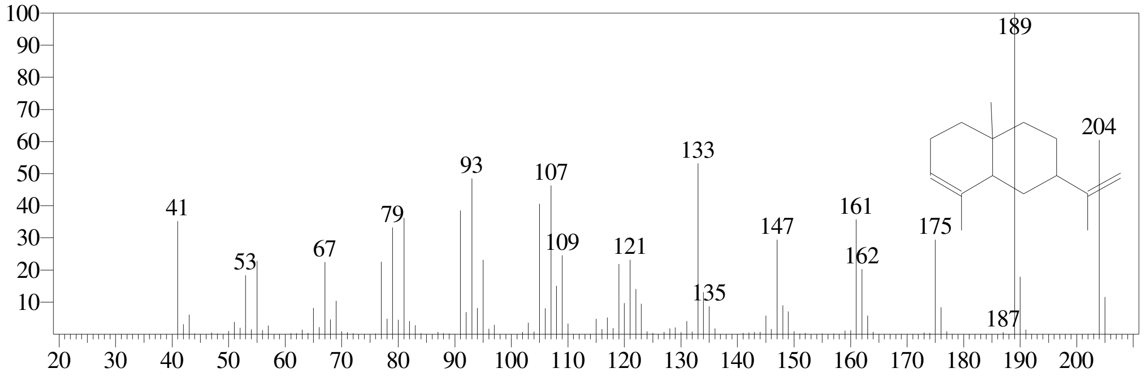

<< Target >>
Line#:14   R.Time:28.450(Scan#:3115) MassPeaks:28
RawMode:Averaged 28.442-28.458(3114-3116) BasePeak:189.10(5819)
BG Mode:Calc. from Peak Group 1 - Event 1 Scan

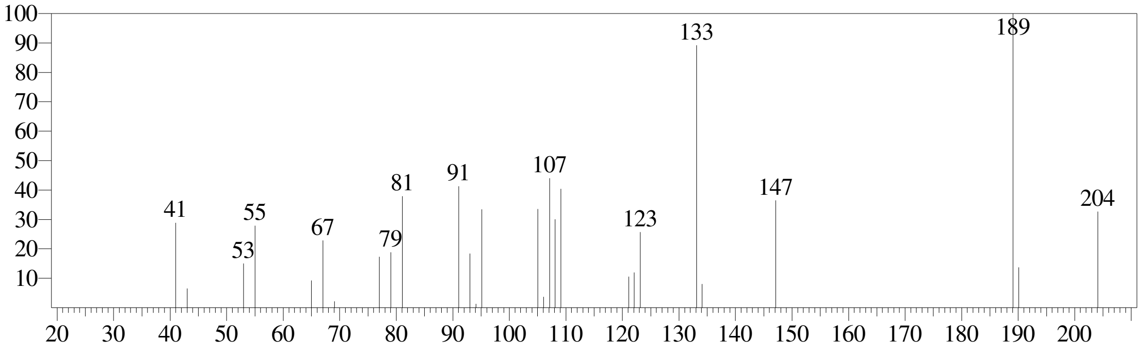

Hit#:5 Entry:63033 Library:NIST23-1.lib
SI:80 Formula:C15H24 CAS:207297-57-2 MolWeight:204 RetIndex:1483
CompName:2-Isopropenyl-4a,8-dimethyl-1,2,3,4,4a,5,6,8a-octahydronaphthalene

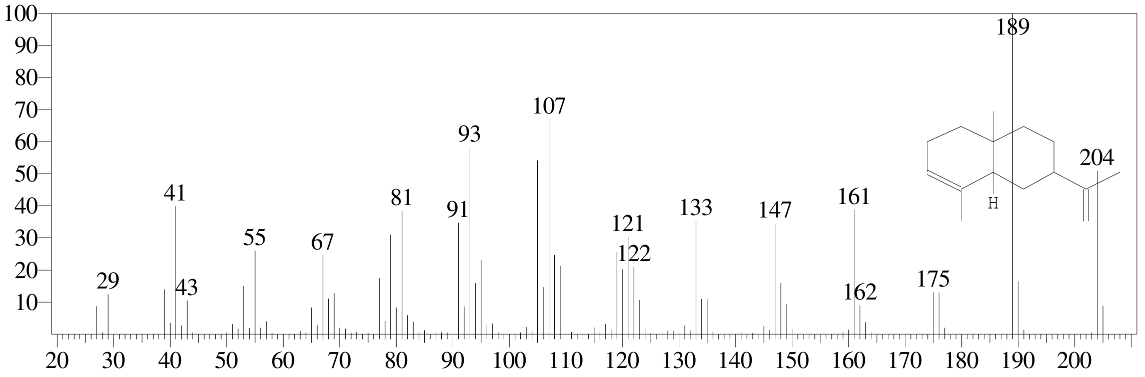

<< Target >>
Line#:15 R.Time:28.492(Scan#:3120) MassPeaks:28
RawMode:Averaged 28.483-28.500(3119-3121) BasePeak:161.10(1556)
BG Mode:Calc. from Peak Group 1 - Event 1 Scan

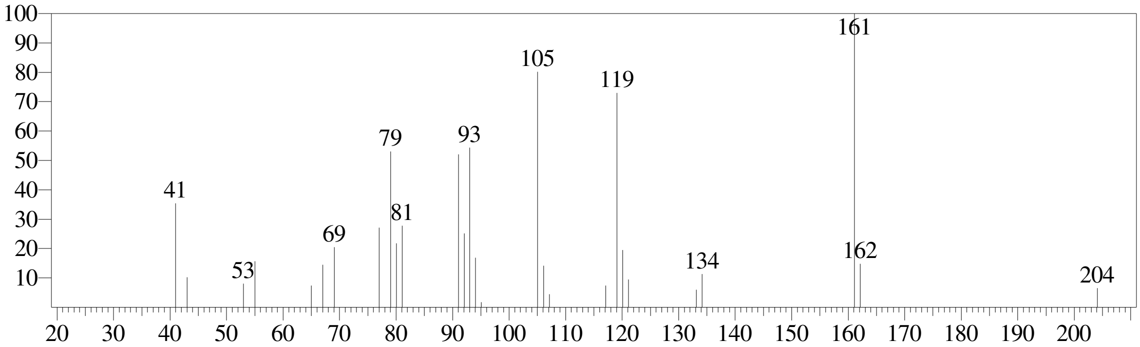

Hit#:1 Entry:25086 Library:NIST23s.lib
SI:88 Formula:C15H24 CAS:30021-74-0 MolWeight:204 RetIndex:1483
CompName:.gamma.-Muurolene $$ Naphthalene, 1,2,3,4,4a,5,6,8a-octahydro-7-methyl-4-methylene-1-(1-methylethyl)-, (

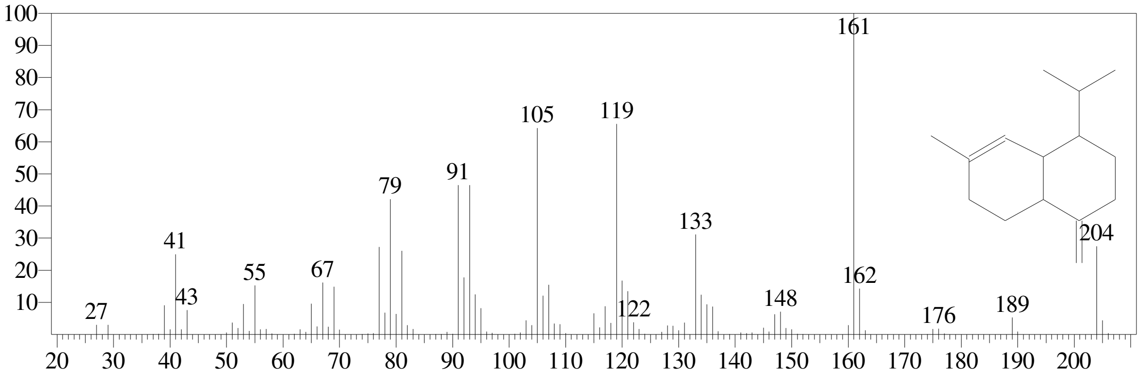

Hit#:2 Entry:62990 Library:NIST23-1.lib
SI:88 Formula:C15H24 CAS:6980-46-7 MolWeight:204 RetIndex:1483
CompName:(1S,4aR,8aS)-1-Isopropyl-7-methyl-4-methylene-1,2,3,4,4a,5,6,8a-octahydronaphthalene $$ .gamma.-Amorp

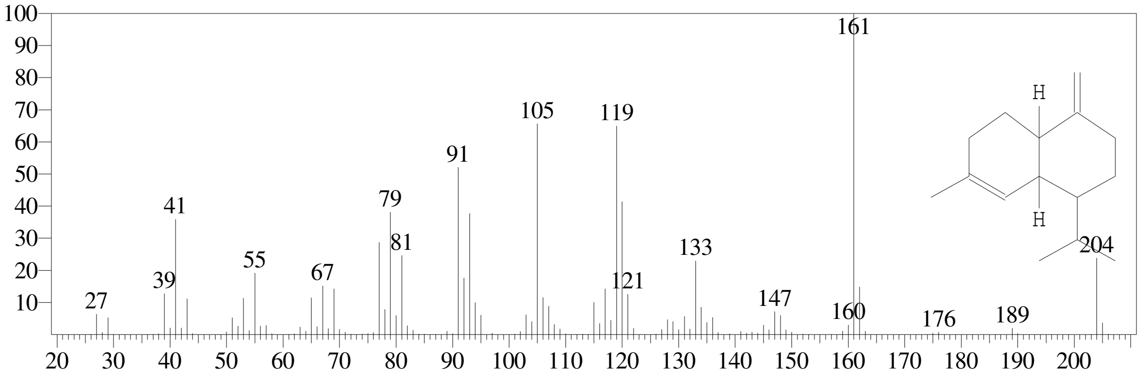

<< Target >>
Line#:15   R.Time:28.492(Scan#:3120) MassPeaks:28
RawMode:Averaged 28.483-28.500(3119-3121) BasePeak:161.10(1556)
BG Mode:Calc. from Peak Group 1 - Event 1 Scan

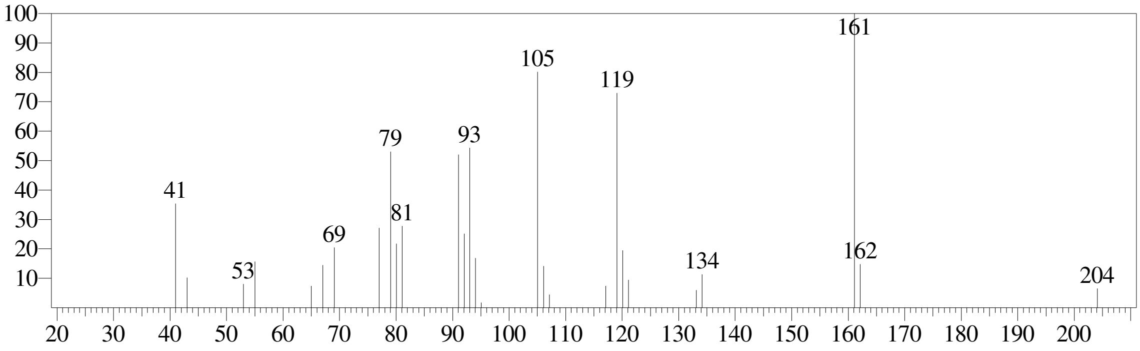

Hit#:3 Entry:25087 Library:NIST23s.lib
SI:88 Formula:C15H24 CAS:3856-25-5 MolWeight:204 RetIndex:1407
CompName:Copaene $$ Tricyclo[4.4.0.02,7]dec-3-ene, 1,3-dimethyl-8-(1-methylethyl)-, stereoisomer $$ Tricyclo[4.4.0.0

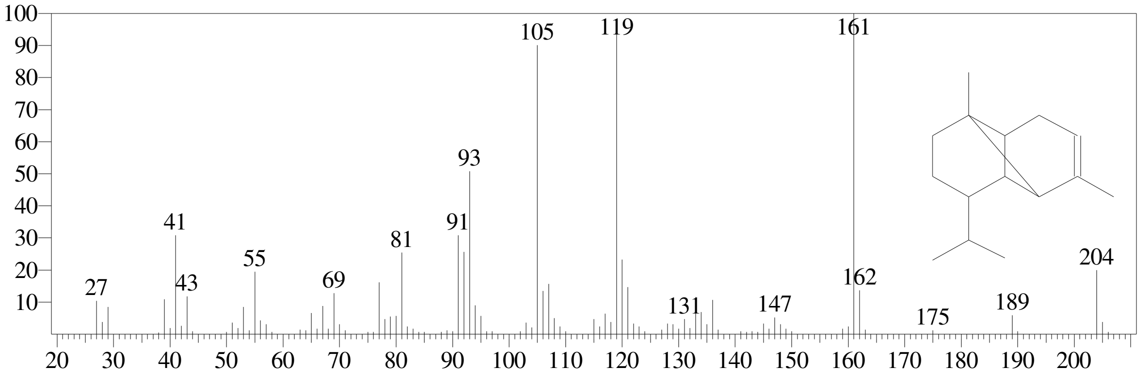

Hit#:4 Entry:62910 Library:NIST23-1.lib
SI:88 Formula:C15H24 CAS:3856-25-5 MolWeight:204 RetIndex:1407
CompName:Copaene $$ Tricyclo[4.4.0.02,7]dec-3-ene, 1,3-dimethyl-8-(1-methylethyl)-, stereoisomer $$ Tricyclo[4.4.0.0

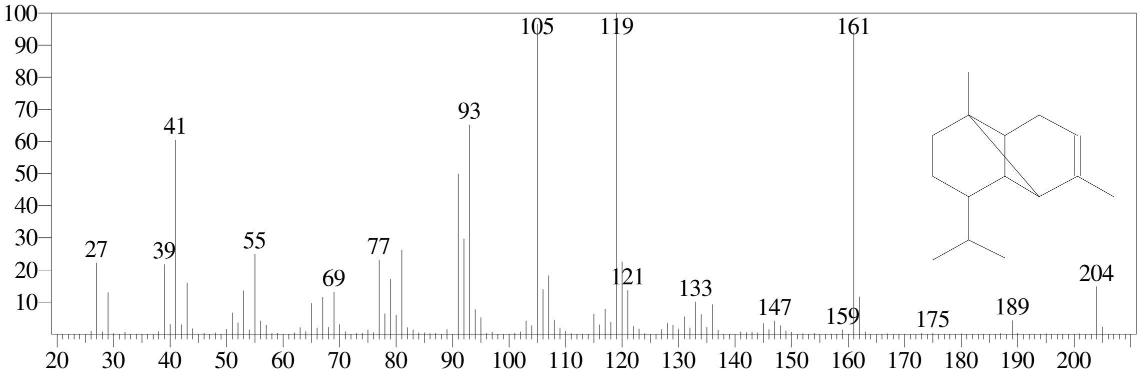

<< Target >>
Line#:15 R.Time:28.492(Scan#:3120) MassPeaks:28
RawMode:Averaged 28.483-28.500(3119-3121) BasePeak:161.10(1556)
BG Mode:Calc. from Peak Group 1 - Event 1 Scan

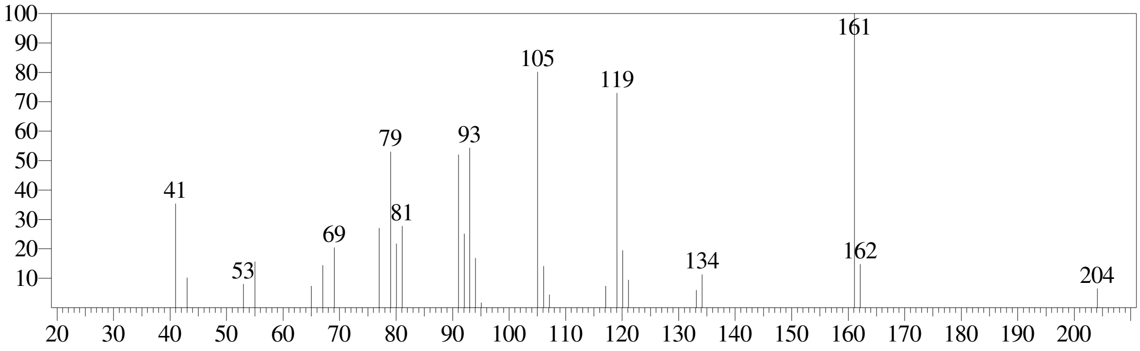

Hit#:5 Entry:25089 Library:NIST23s.lib
SI:87 Formula:C15H24 CAS:3856-25-5 MolWeight:204 RetIndex:1407
CompName:Copaene $$ Tricyclo[4.4.0.02,7]dec-3-ene, 1,3-dimethyl-8-(1-methylethyl)-, stereoisomer $$ Tricyclo[4.4.0.0

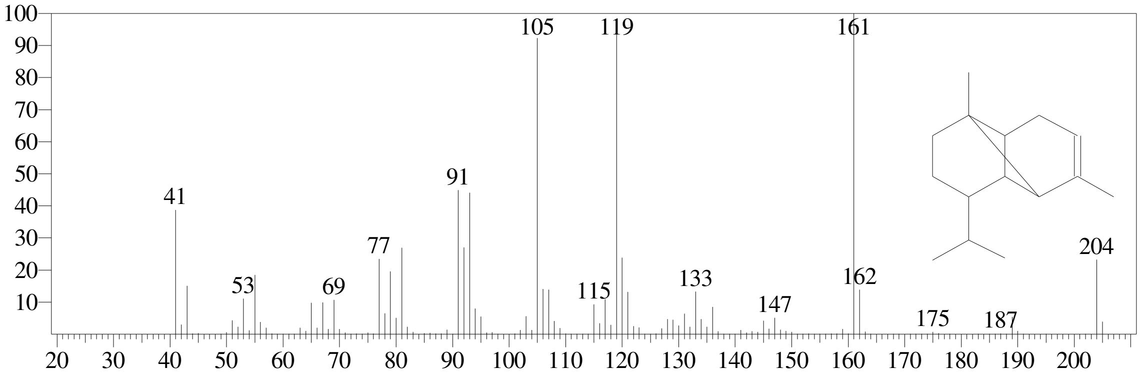

<< Target >>
Line#:16 R.Time:28.642(Scan#:3138) MassPeaks:8
RawMode:Averaged 28.633-28.650(3137-3139) BasePeak:105.05(3273)
BG Mode:Calc. from Peak Group 1 - Event 1 Scan

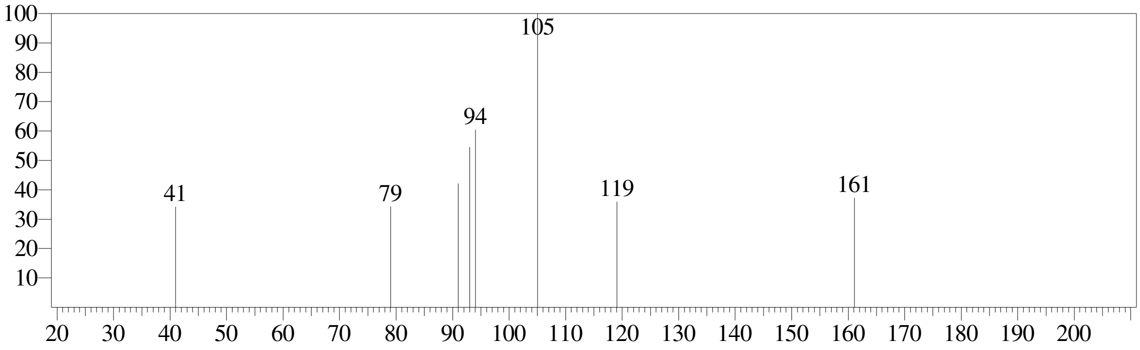

Hit#:1 Entry:24935 Library:NIST23s.lib
SI:66 Formula:C15H24 CAS:483-75-0 MolWeight:204 RetIndex:1500
CompName:Naphthalene, 1,2,4a,5,6,8a-hexahydro-4,7-dimethyl-1-(1-methylethyl)- $$ 1-Isopropyl-4,7-dimethyl-1,2,4a,5,

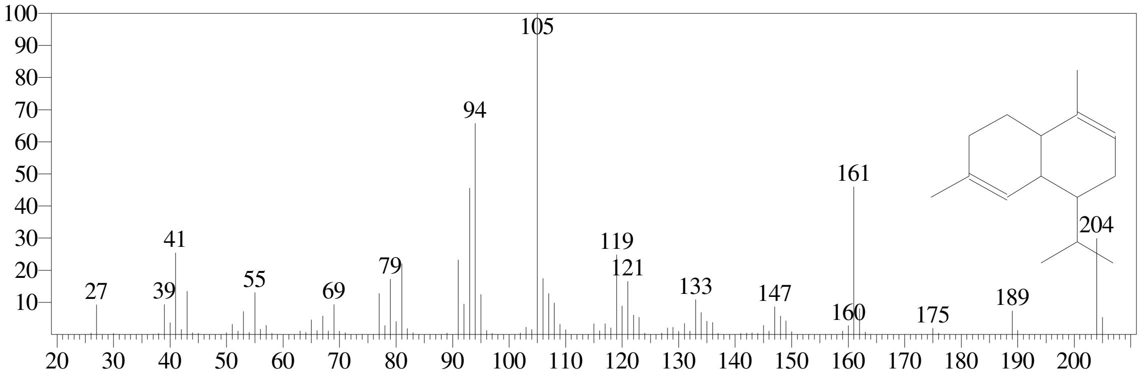

Hit#:2 Entry:24934 Library:NIST23s.lib
SI:66 Formula:C15H24 CAS:483-75-0 MolWeight:204 RetIndex:1500
CompName:Naphthalene, 1,2,4a,5,6,8a-hexahydro-4,7-dimethyl-1-(1-methylethyl)- $$ 1-Isopropyl-4,7-dimethyl-1,2,4a,5,

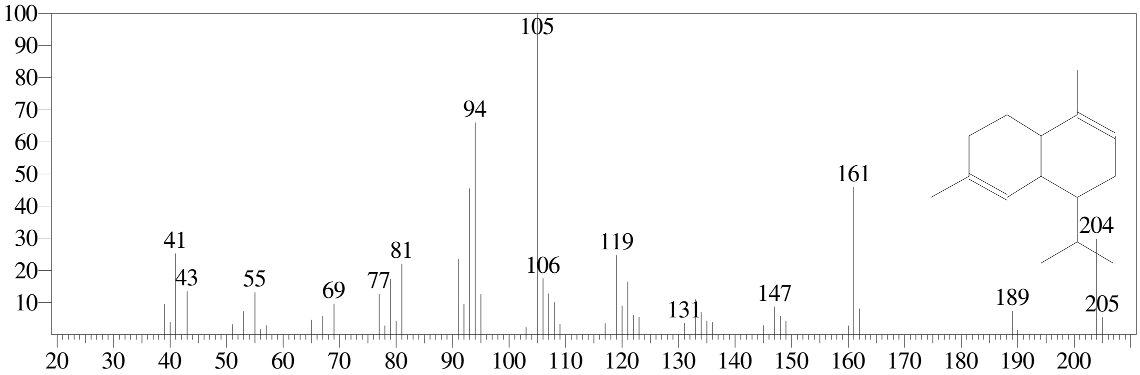

<< Target >>
Line#:16   R.Time:28.642(Scan#:3138) MassPeaks:8
RawMode:Averaged 28.633-28.650(3137-3139) BasePeak:105.05(3273)
BG Mode:Calc. from Peak Group 1 - Event 1 Scan

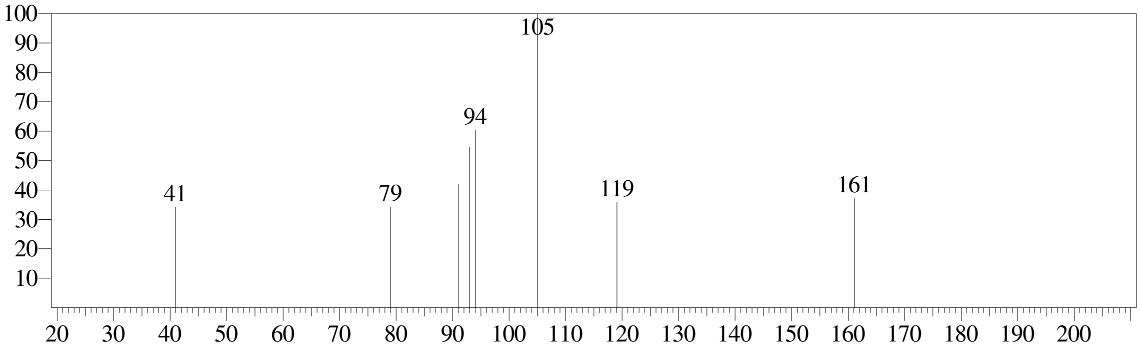

Hit#:3 Entry:24928 Library:NIST23s.lib
SI:66 Formula:C15H24 CAS:20085-19-2 MolWeight:204 RetIndex:1498
CompName:(1R,4aS,8aR)-1-Isopropyl-4,7-dimethyl-1,2,4a,5,6,8a-hexahydronaphthalene $$ .alpha.-Amorphene $$ Amor

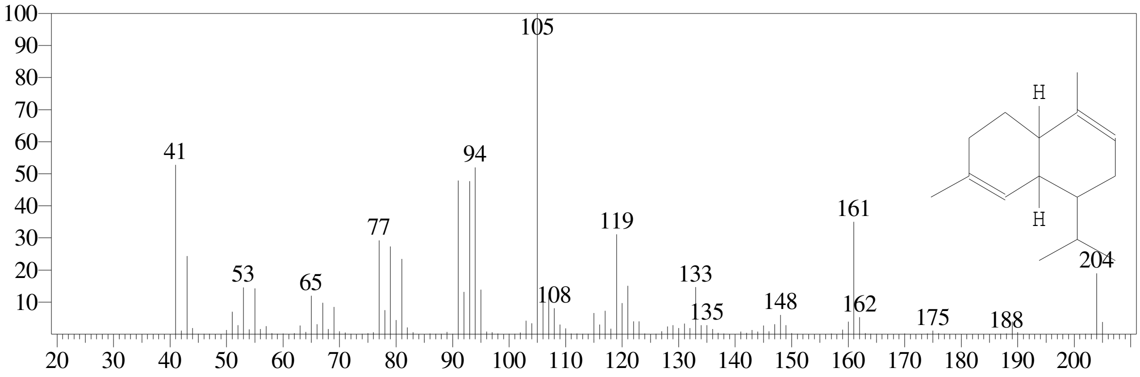

Hit#:4 Entry:62846 Library:NIST23-1.lib
SI:66 Formula:C15H24 CAS:20085-19-2 MolWeight:204 RetIndex:1498
CompName:(1R,4aS,8aR)-1-Isopropyl-4,7-dimethyl-1,2,4a,5,6,8a-hexahydronaphthalene $$ .alpha.-Amorphene $$ Amor

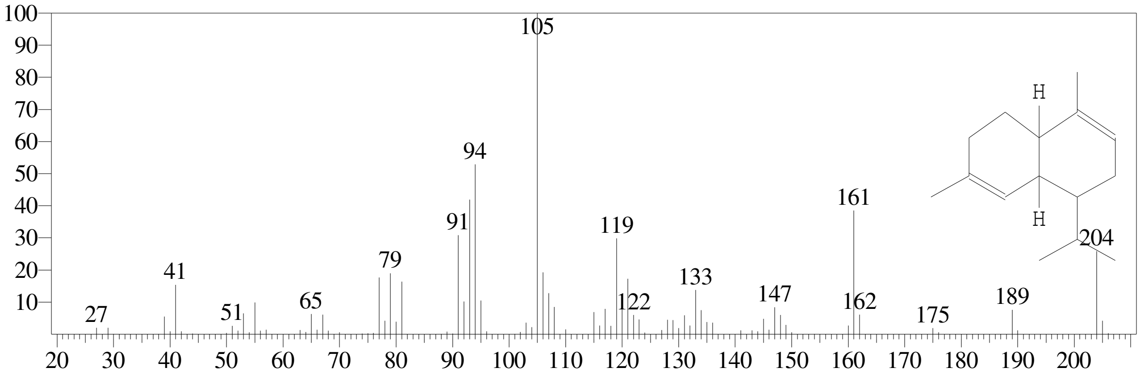

<< Target >>
Line#:16   R.Time:28.642(Scan#:3138) MassPeaks:8
RawMode:Averaged 28.633-28.650(3137-3139) BasePeak:105.05(3273)
BG Mode:Calc. from Peak Group 1 - Event 1 Scan

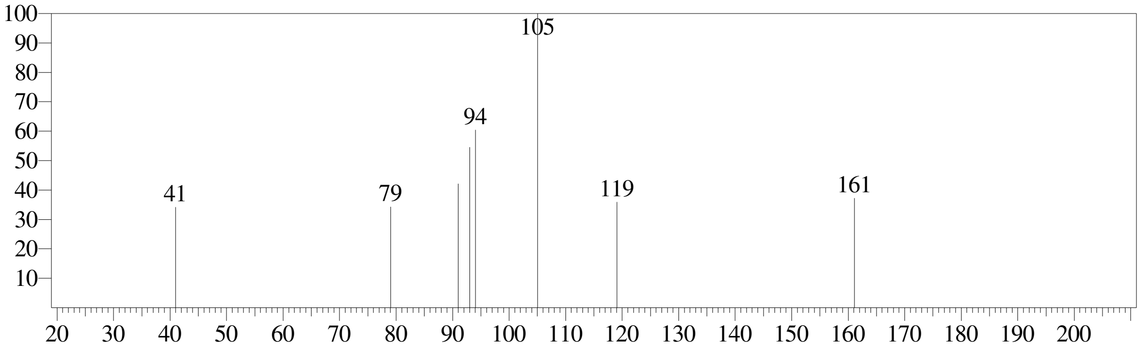

Hit#:5 Entry:62857 Library:NIST23-1.lib
SI:66 Formula:C15H24 CAS:483-75-0 MolWeight:204 RetIndex:1500
CompName:Naphthalene, 1,2,4a,5,6,8a-hexahydro-4,7-dimethyl-1-(1-methylethyl)- $$ 1-Isopropyl-4,7-dimethyl-1,2,4a,5,

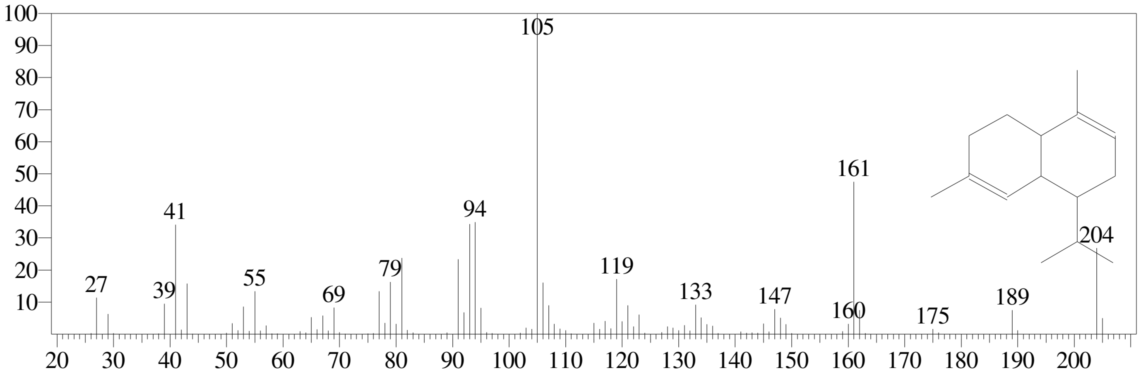

<< Target >>
Line#:17 R.Time:28.883(Scan#:3167) MassPeaks:67
RawMode:Averaged 28.875-28.892(3166-3168) BasePeak:93.05(53832)
BG Mode:None Group 1 - Event 1 Scan

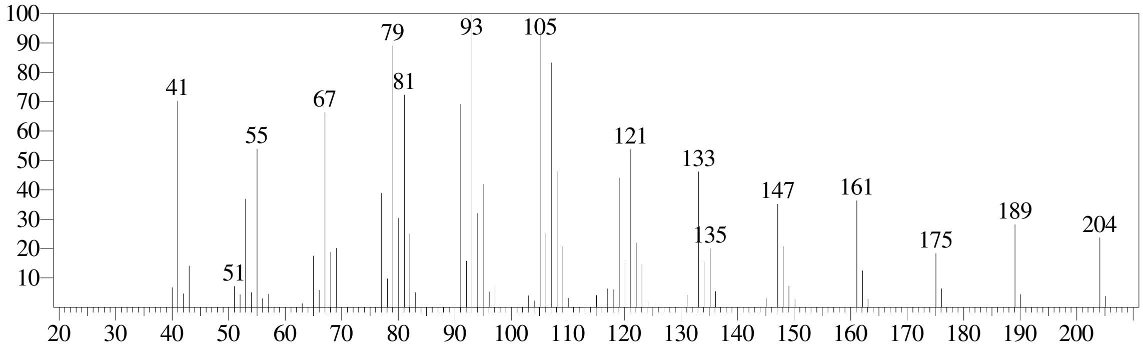

Hit#:1 Entry:24936 Library:NIST23s.lib
SI:96 Formula:C15H24 CAS:17066-67-0 MolWeight:204 RetIndex:1489
CompName:Naphthalene, decahydro-4a-methyl-1-methylene-7-(1-methylethenyl)-, [4aR-(4a.alpha.,7.alpha.,8a.beta.)]- $$

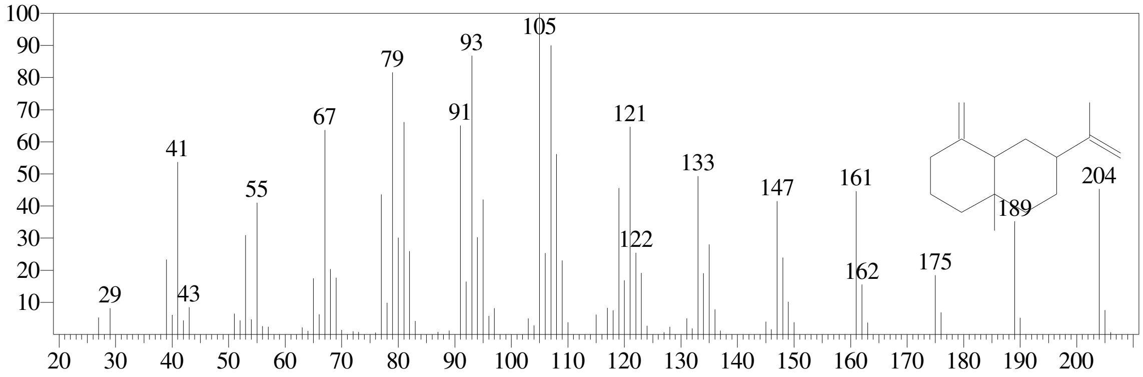

Hit#:2 Entry:24892 Library:NIST23s.lib
SI:95 Formula:C15H24 CAS:17066-67-0 MolWeight:204 RetIndex:1489
CompName:Naphthalene, decahydro-4a-methyl-1-methylene-7-(1-methylethenyl)-, [4aR-(4a.alpha.,7.alpha.,8a.beta.)]- $$

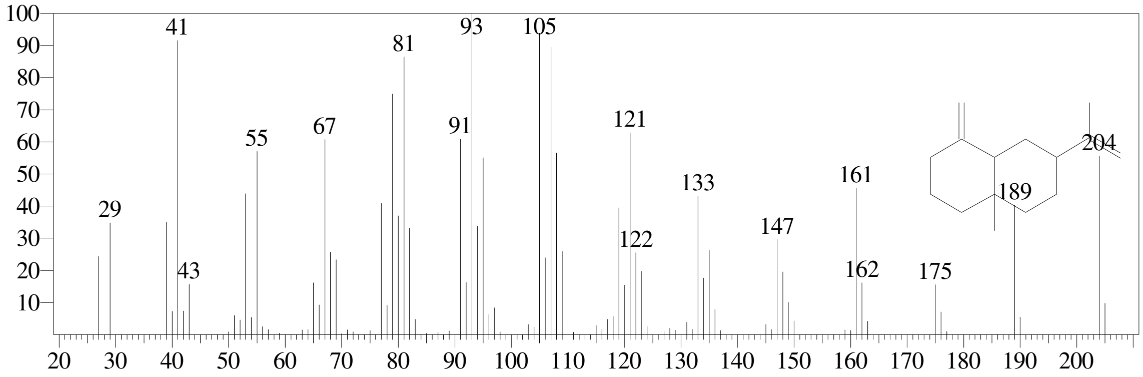

<< Target >>
Line#:17 R.Time:28.883(Scan#:3167) MassPeaks:67
RawMode:Averaged 28.875-28.892(3166-3168) BasePeak:93.05(53832)
BG Mode:None Group 1 - Event 1 Scan

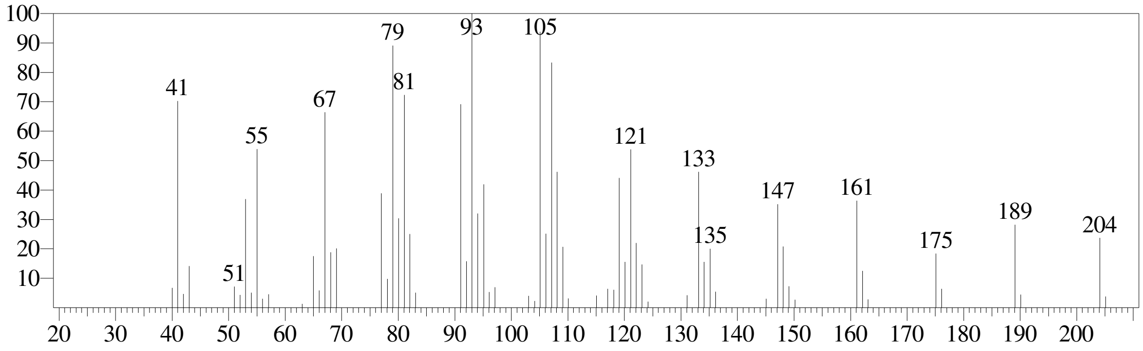

Hit#:3 Entry:62812 Library:NIST23-1.lib
SI:94 Formula:C15H24 CAS:0-00-0 MolWeight:204 RetIndex:1475
CompName:Bicyclo[5.3.0]decane, 2-methylene-5-(1-methylvinyl)-8-methyl- $$ 7-Isopropenyl-1-methyl-4-methylenedeca

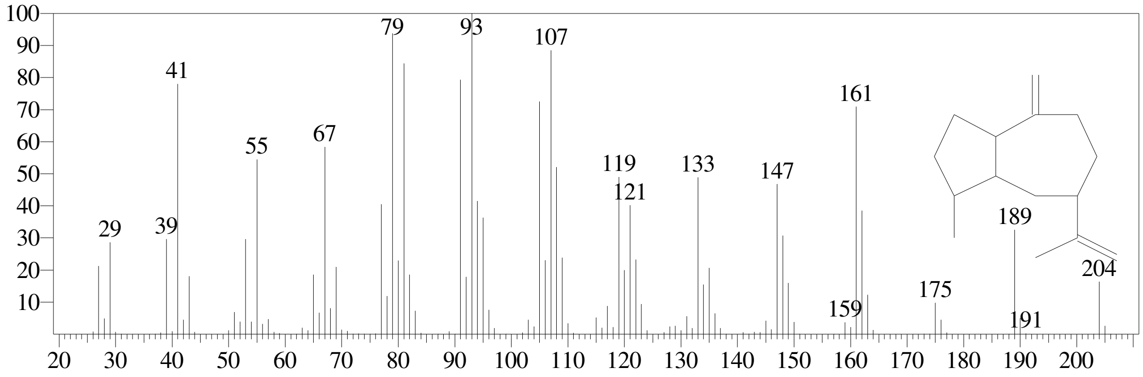

Hit#:4 Entry:62758 Library:NIST23-1.lib
SI:93 Formula:C15H24 CAS:17066-67-0 MolWeight:204 RetIndex:1489
CompName:Naphthalene, decahydro-4a-methyl-1-methylene-7-(1-methylethenyl)-, [4aR-(4a.alpha.,7.alpha.,8a.beta.)]- $$

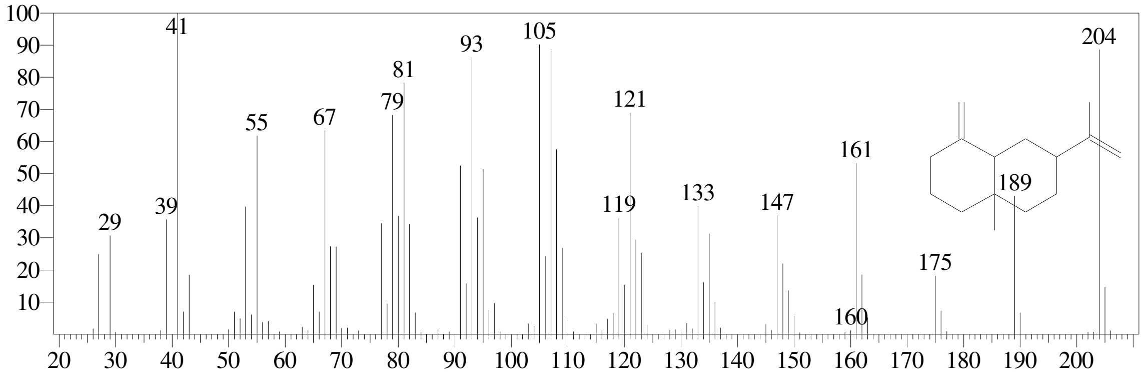

<< Target >>
Line#:17   R.Time:28.883(Scan#:3167) MassPeaks:67
RawMode:Averaged 28.875-28.892(3166-3168) BasePeak:93.05(53832)
BG Mode:None Group 1 - Event 1 Scan

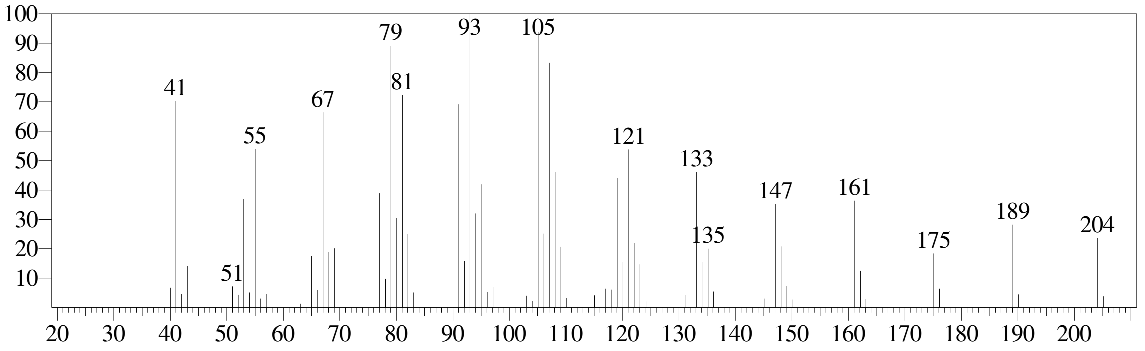

Hit#:5 Entry:24955 Library:NIST23s.lib
SI:92 Formula:C15H24 CAS:17066-67-0 MolWeight:204 RetIndex:1489
CompName:Naphthalene, decahydro-4a-methyl-1-methylene-7-(1-methylethenyl)-, [4aR-(4a.alpha.,7.alpha.,8a.beta.)]- $$

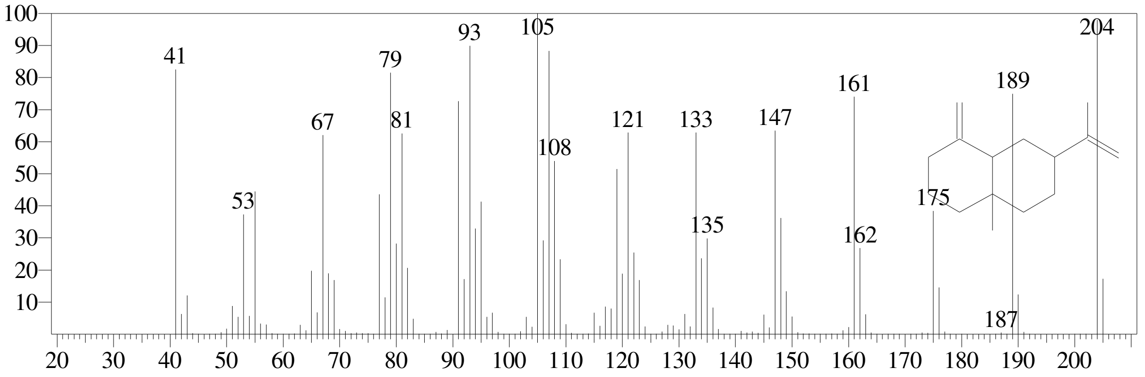

<< Target >>
Line#:18   R.Time:29.242(Scan#:3210) MassPeaks:65
RawMode:Averaged 29.233-29.250(3209-3211) BasePeak:93.05(37636)
BG Mode:None Group 1 - Event 1 Scan

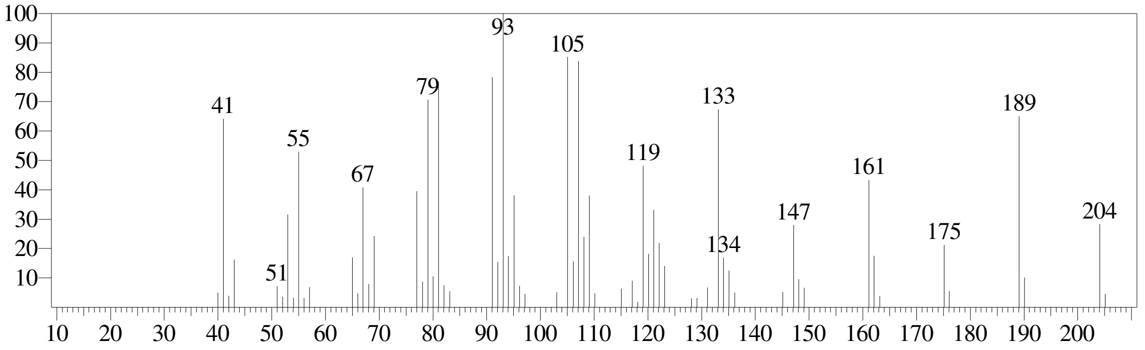

Hit#:1 Entry:24894 Library:NIST23s.lib
SI:93 Formula:C15H24 CAS:3691-11-0 MolWeight:204 RetIndex:1498
CompName:Azulene, 1,2,3,5,6,7,8,8a-octahydro-1,4-dimethyl-7-(1-methylethenyl)-, [1S-(1.alpha.,7.alpha.,8a.beta.)]- $$ (

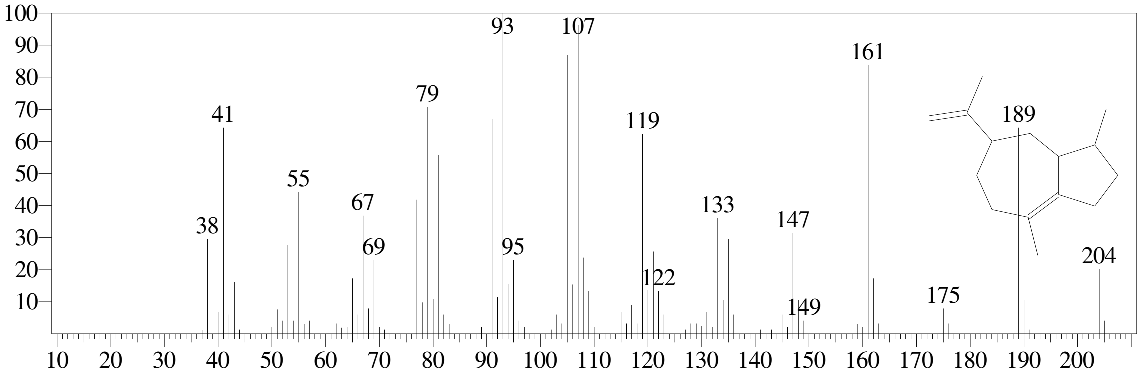

Hit#:2 Entry:24925 Library:NIST23s.lib
SI:92 Formula:C15H24 CAS:3691-12-1 MolWeight:204 RetIndex:1454
CompName:.alpha.-Guaiene $$ Azulene, 1,2,3,4,5,6,7,8-octahydro-1,4-dimethyl-7-(1-methylethenyl)-, [1S-(1.alpha.,4.alp

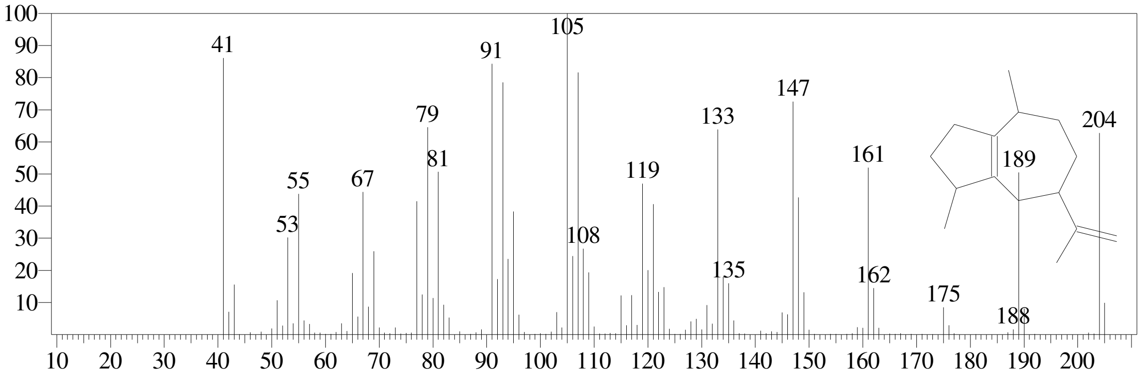

<< Target >>
Line#:18 R.Time:29.242(Scan#:3210) MassPeaks:65
RawMode:Averaged 29.233-29.250(3209-3211) BasePeak:93.05(37636)
BG Mode:None Group 1 - Event 1 Scan

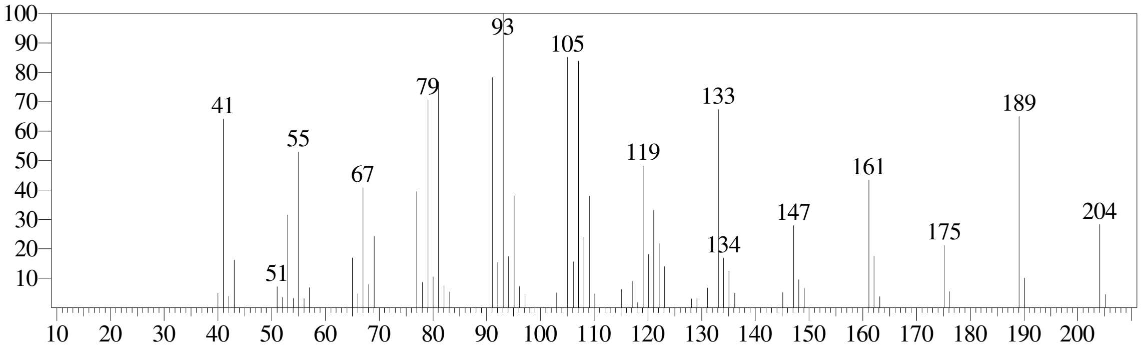

Hit#:3 Entry:24937 Library:NIST23s.lib
SI:92 Formula:C15H24 CAS:3691-12-1 MolWeight:204 RetIndex:1454
CompName:.alpha.-Guaiene $$ Azulene, 1,2,3,4,5,6,7,8-octahydro-1,4-dimethyl-7-(1-methylethenyl)-, [1S-(1.alpha.,4.alp

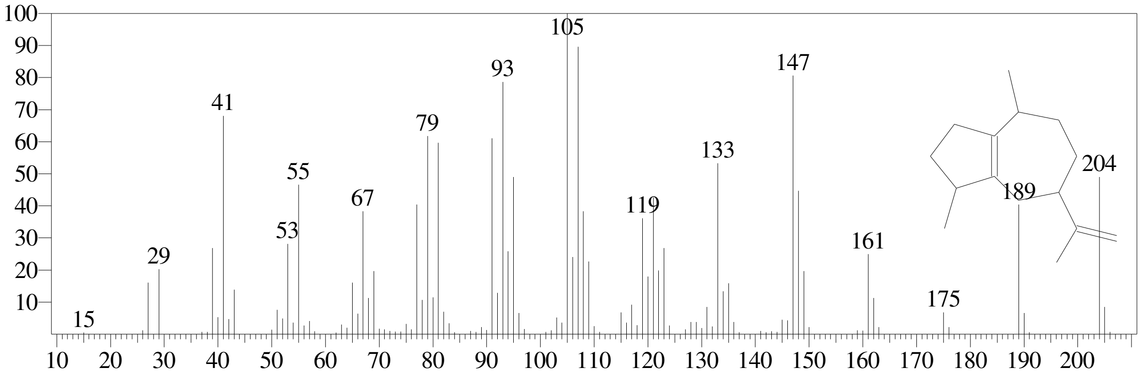

Hit#:4 Entry:62812 Library:NIST23-1.lib
SI:92 Formula:C15H24 CAS:0-00-0 MolWeight:204 RetIndex:1475
CompName:Bicyclo[5.3.0]decane, 2-methylene-5-(1-methylvinyl)-8-methyl- $$ 7-Isopropenyl-1-methyl-4-methylenedeca

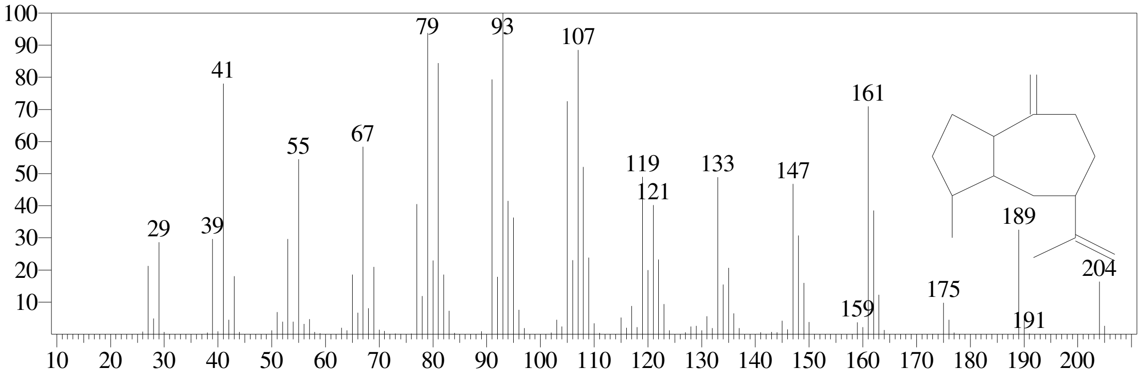

<< Target >>
Line#:18   R.Time:29.242(Scan#:3210) MassPeaks:65
RawMode:Averaged 29.233-29.250(3209-3211) BasePeak:93.05(37636)
BG Mode:None Group 1 - Event 1 Scan

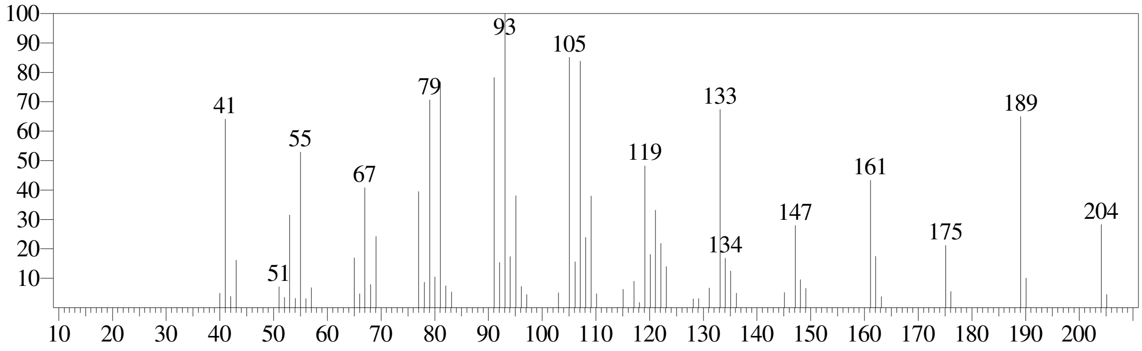

Hit#:5 Entry:63031 Library:NIST23-1.lib
SI:91 Formula:C15H24 CAS:18431-82-8 MolWeight:204 RetIndex:1477
CompName:Spiro[5.5]undec-2-ene, 3,7,7-trimethyl-11-methylene-, (-)- $$ .beta.-Chamigrene $$ Chamigren $$ Chamigre

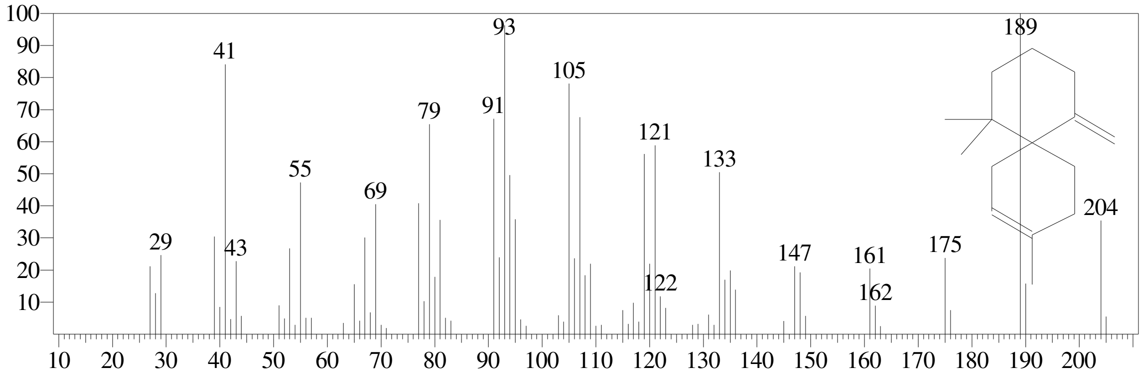

<< Target >>
Line#:19 R.Time:29.383(Scan#:3227) MassPeaks:8
RawMode:Averaged 29.375-29.392(3226-3228) BasePeak:161.10(1806)
BG Mode:Calc. from Peak Group 1 - Event 1 Scan

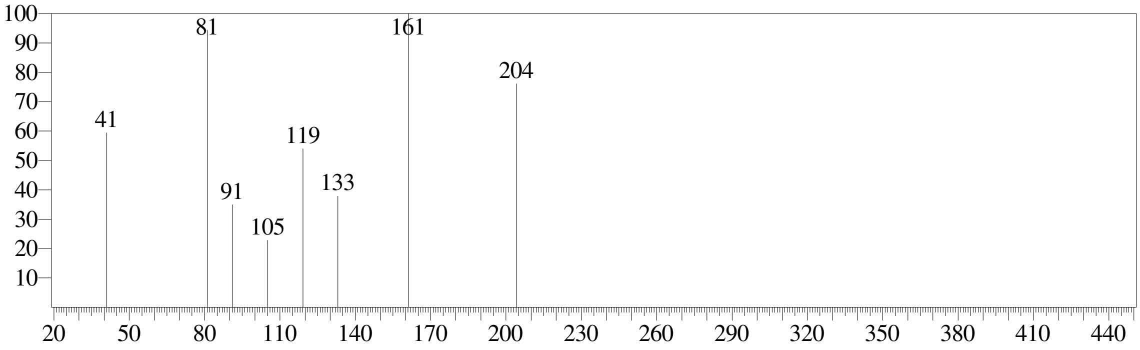

Hit#:1 Entry:25079 Library:NIST23s.lib
SI:73 Formula:C15H24 CAS:157477-72-0 MolWeight:204 RetIndex:1487
CompName:cis-Muurola-4(15),5-diene $$ (1S,4S,4aR)-1-Isopropyl-4-methyl-7-methylene-1,2,3,4,4a,5,6,7-octahydronaph

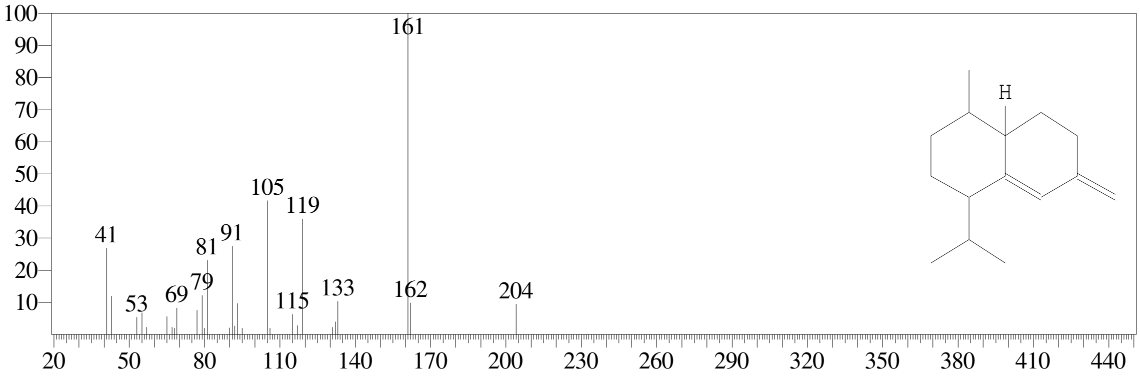

Hit#:2 Entry:69566 Library:NIST23-2.lib
SI:69 Formula:C28H47NO3 CAS:0-00-0 MolWeight:445 RetIndex:3421
CompName:l-Alanine, N-(4-butylbenzoyl)-, tetradecyl ester

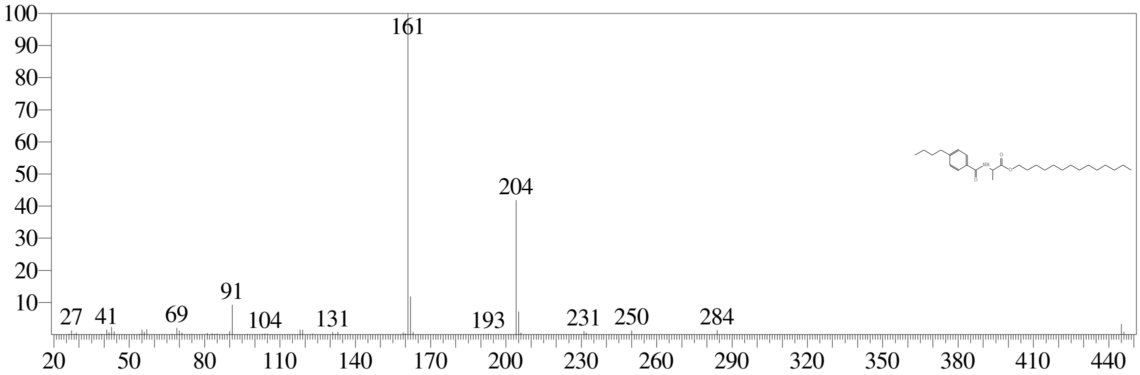

<< Target >>
Line#:19 R.Time:29.383(Scan#:3227) MassPeaks:8
RawMode:Averaged 29.375-29.392(3226-3228) BasePeak:161.10(1806)
BG Mode:Calc. from Peak Group 1 - Event 1 Scan

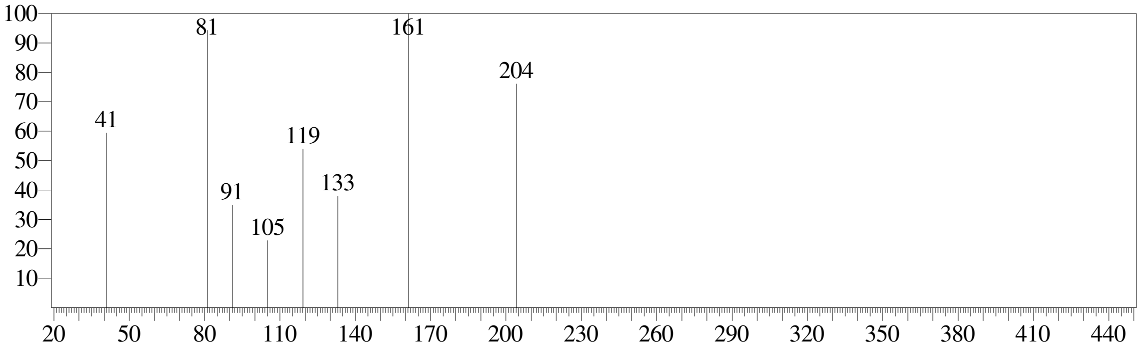

Hit#:3 Entry:62987 Library:NIST23-1.lib
SI:69 Formula:C15H24 CAS:267665-20-3 MolWeight:204 RetIndex:1465
CompName:(1S,4S,4aS)-1-Isopropyl-4,7-dimethyl-1,2,3,4,4a,5-hexahydronaphthalene $$ Naphthalene, 1,2,3,4,4a,5-hexah

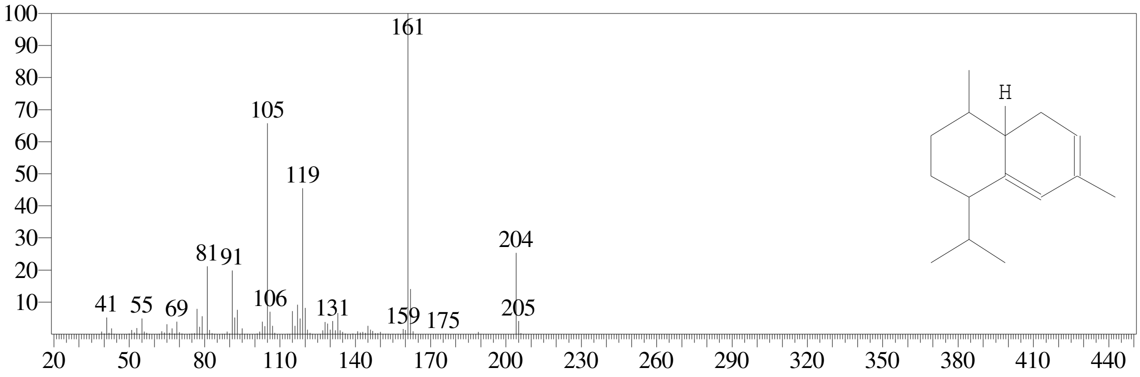

Hit#:4 Entry:62992 Library:NIST23-1.lib
SI:68 Formula:C15H24 CAS:54274-73-6 MolWeight:204 RetIndex:1487
CompName:(+)-epi-Bicyclosesquiphellandrene $$ 1-Isopropyl-4-methyl-7-methylene-1,2,3,4,4a,5,6,7-octahydronaphthale

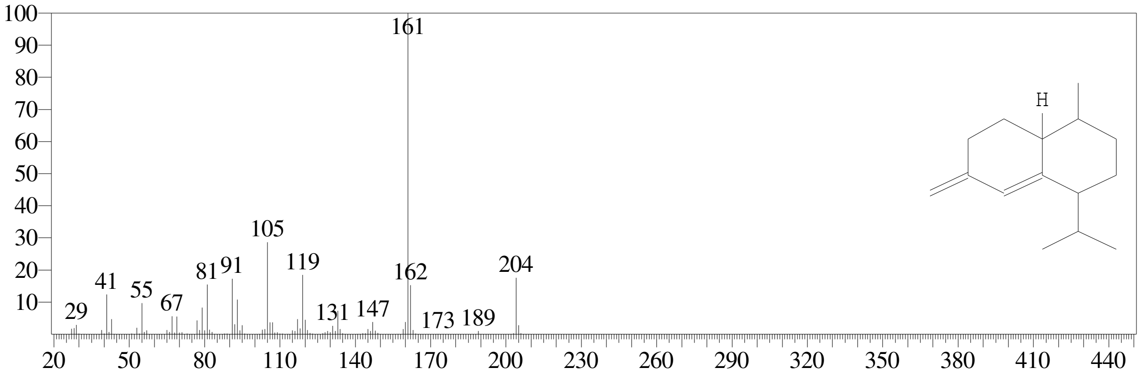

<< Target >>
Line#:19   R.Time:29.383(Scan#:3227) MassPeaks:8
RawMode:Averaged 29.375-29.392(3226-3228) BasePeak:161.10(1806)
BG Mode:Calc. from Peak Group 1 - Event 1 Scan

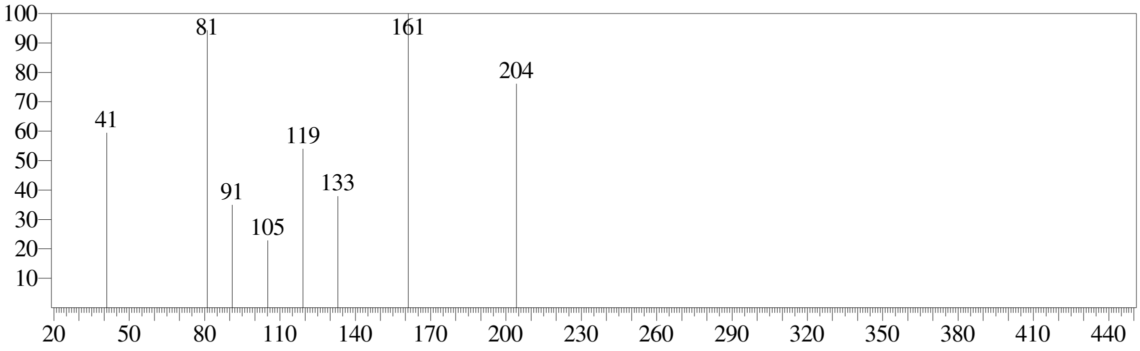

Hit#:5 Entry:227428 Library:NIST23-1.lib
SI:68 Formula:C20H31NO3 CAS:0-00-0 MolWeight:333 RetIndex:2566
CompName:l-Alanine, N-(4-butylbenzoyl)-, isohexyl ester

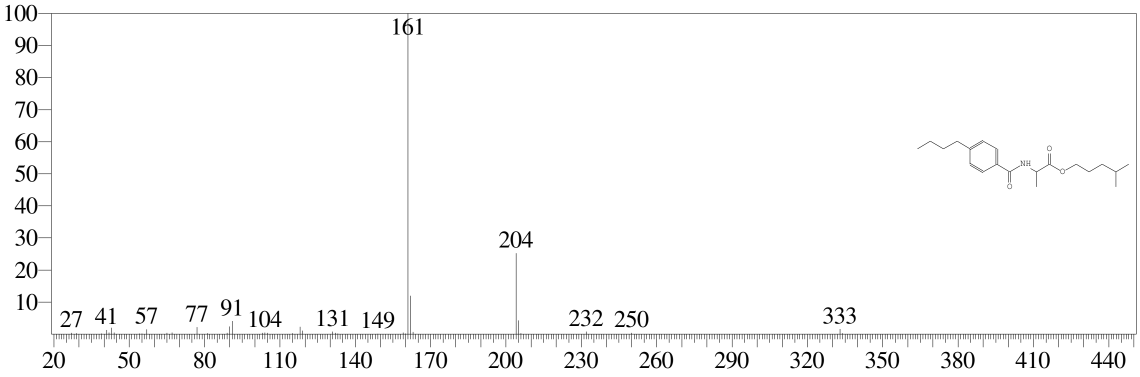

<< Target >>
Line#:20 R.Time:29.450(Scan#:3235) MassPeaks:15
RawMode:Averaged 29.442-29.458(3234-3236) BasePeak:105.05(6485)
BG Mode:Calc. from Peak Group 1 - Event 1 Scan

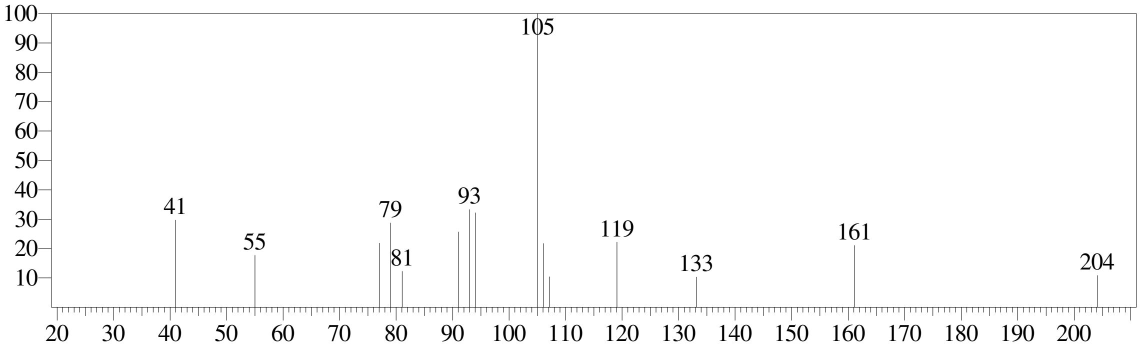

Hit#:1 Entry:62857 Library:NIST23-1.lib
SI:78 Formula:C15H24 CAS:483-75-0 MolWeight:204 RetIndex:1500
CompName:Naphthalene, 1,2,4a,5,6,8a-hexahydro-4,7-dimethyl-1-(1-methylethyl)- $$ 1-Isopropyl-4,7-dimethyl-1,2,4a,5,

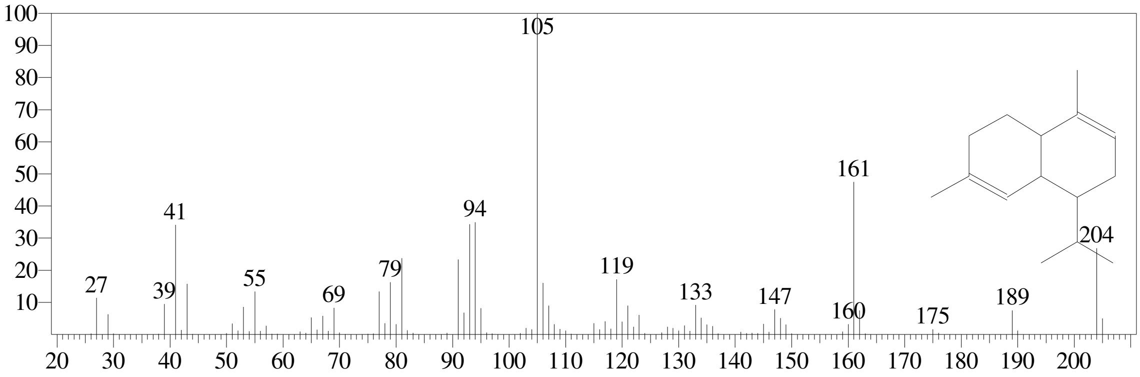

Hit#:2 Entry:24947 Library:NIST23s.lib
SI:78 Formula:C15H24 CAS:31983-22-9 MolWeight:204 RetIndex:1498
CompName:Naphthalene, 1,2,4a,5,6,8a-hexahydro-4,7-dimethyl-1-(1-methylethyl)-, (1.alpha.,4a.alpha.,8a.alpha.)- $$ [1.a

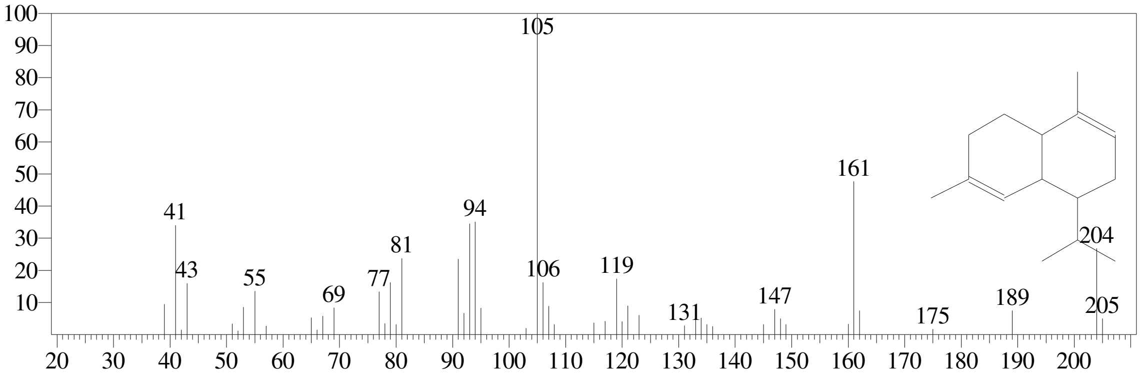

<< Target >>
Line#:20   R.Time:29.450(Scan#:3235) MassPeaks:15
RawMode:Averaged 29.442-29.458(3234-3236) BasePeak:105.05(6485)
BG Mode:Calc. from Peak Group 1 - Event 1 Scan

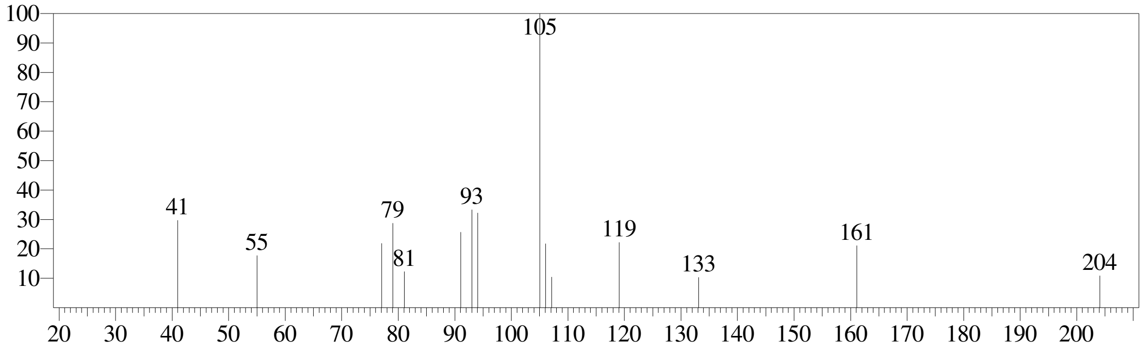

Hit#:3 Entry:62856 Library:NIST23-1.lib
SI:78 Formula:C15H24 CAS:10208-80-7 MolWeight:204 RetIndex:1500
CompName:.alpha.-Muurolene $$ Naphthalene, 1,2,4a,5,6,8a-hexahydro-4,7-dimethyl-1-(1-methylethyl)-, (1S,4aS,8aR)-

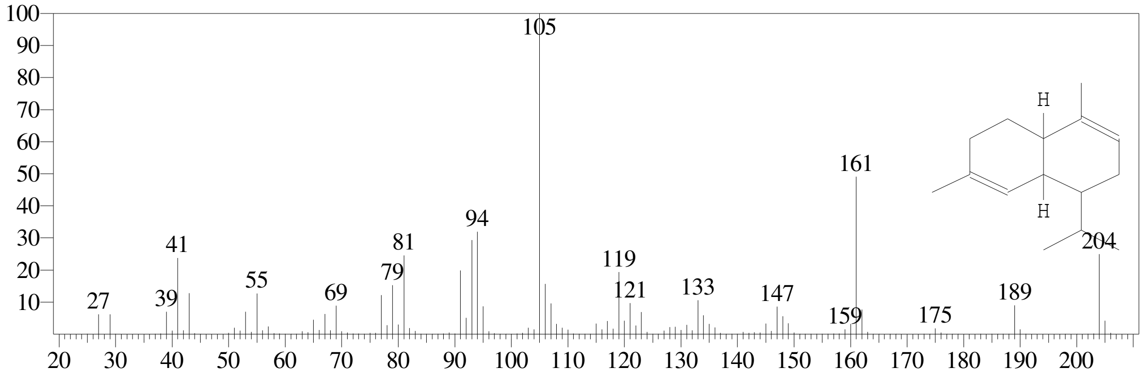

Hit#:4 Entry:24946 Library:NIST23s.lib
SI:76 Formula:C15H24 CAS:10208-80-7 MolWeight:204 RetIndex:1500
CompName:.alpha.-Muurolene $$ Naphthalene, 1,2,4a,5,6,8a-hexahydro-4,7-dimethyl-1-(1-methylethyl)-, (1S,4aS,8aR)-

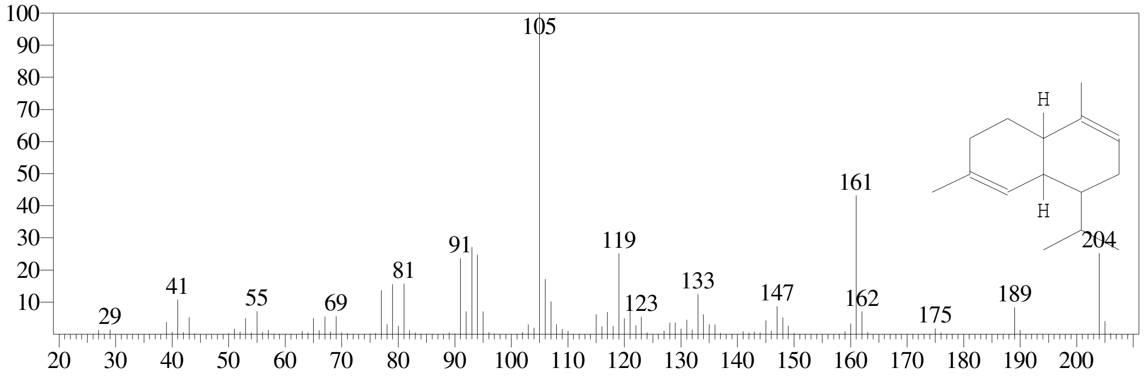

<< Target >>
Line#:20   R.Time:29.450(Scan#:3235) MassPeaks:15
RawMode:Averaged 29.442-29.458(3234-3236) BasePeak:105.05(6485)
BG Mode:Calc. from Peak Group 1 - Event 1 Scan

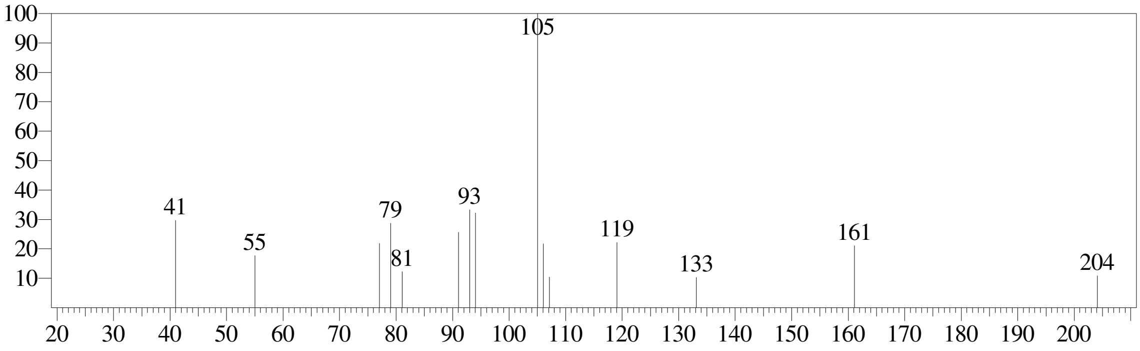

Hit#:5 Entry:62846 Library:NIST23-1.lib
SI:75 Formula:C15H24 CAS:20085-19-2 MolWeight:204 RetIndex:1498
CompName:(1R,4aS,8aR)-1-Isopropyl-4,7-dimethyl-1,2,4a,5,6,8a-hexahydronaphthalene $$ .alpha.-Amorphene $$ Amor

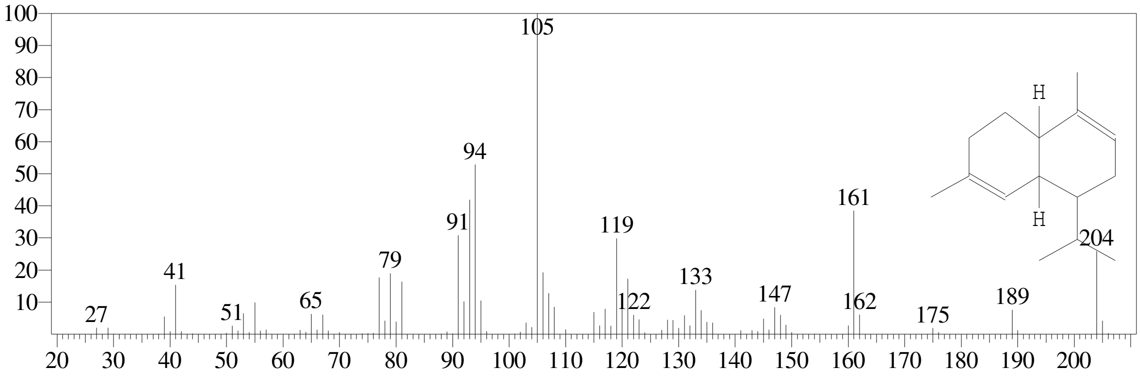

<< Target >>
Line#:21 R.Time:29.733(Scan#:3269) MassPeaks:16
RawMode:Averaged 29.725-29.742(3268-3270) BasePeak:105.05(4724)
BG Mode:None Group 1 - Event 1 Scan

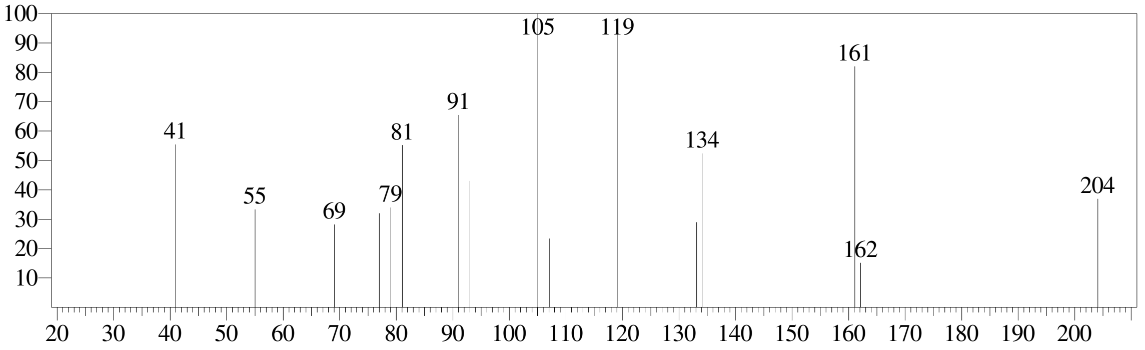

Hit#:1 Entry:62988 Library:NIST23-1.lib
SI:79 Formula:C15H24 CAS:17699-14-8 MolWeight:204 RetIndex:1381
CompName:.alpha.-Cubebene $$ 1H-Cyclopenta[1,3]cyclopropa[1,2]benzene, 3a,3b,4,5,6,7-hexahydro-3,7-dimethyl-4-(1-

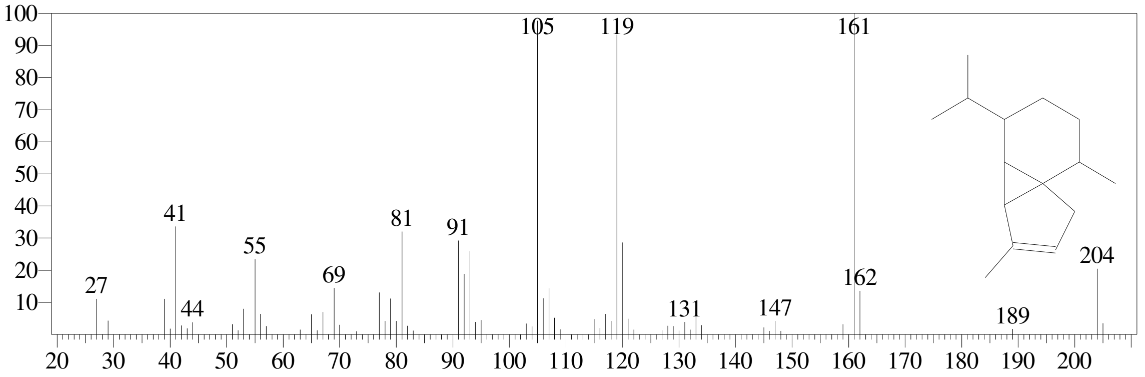

Hit#:2 Entry:25090 Library:NIST23s.lib
SI:79 Formula:C15H24 CAS:483-76-1 MolWeight:204 RetIndex:1526
CompName:Naphthalene, 1,2,3,5,6,8a-hexahydro-4,7-dimethyl-1-(1-methylethyl)-, (1S-cis)- $$ Cadina-1(10),4-diene $$.

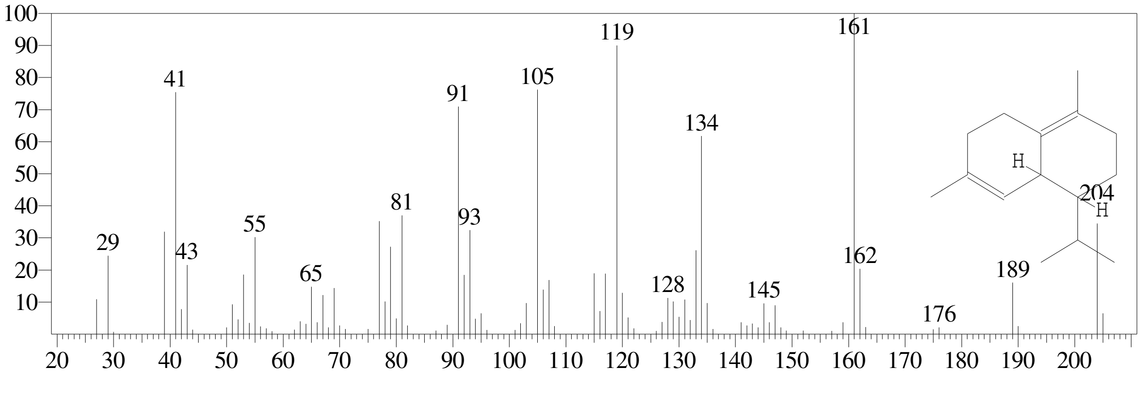

<< Target >>
Line#:21   R.Time:29.733(Scan#:3269) MassPeaks:16
RawMode:Averaged 29.725-29.742(3268-3270) BasePeak:105.05(4724)
BG Mode:None Group 1 - Event 1 Scan

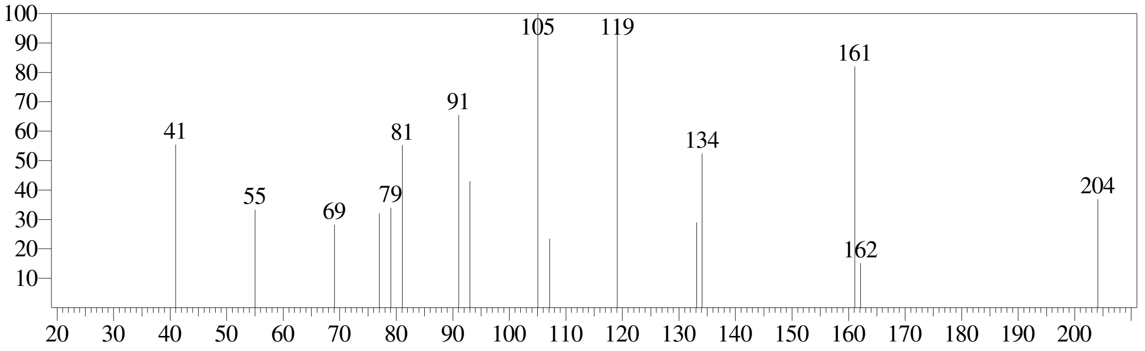

Hit#:3 Entry:24948 Library:NIST23s.lib
SI:78 Formula:C15H24 CAS:17699-14-8 MolWeight:204 RetIndex:1381
CompName:.alpha.-Cubebene $$ 1H-Cyclopenta[1,3]cyclopropa[1,2]benzene, 3a,3b,4,5,6,7-hexahydro-3,7-dimethyl-4-(1

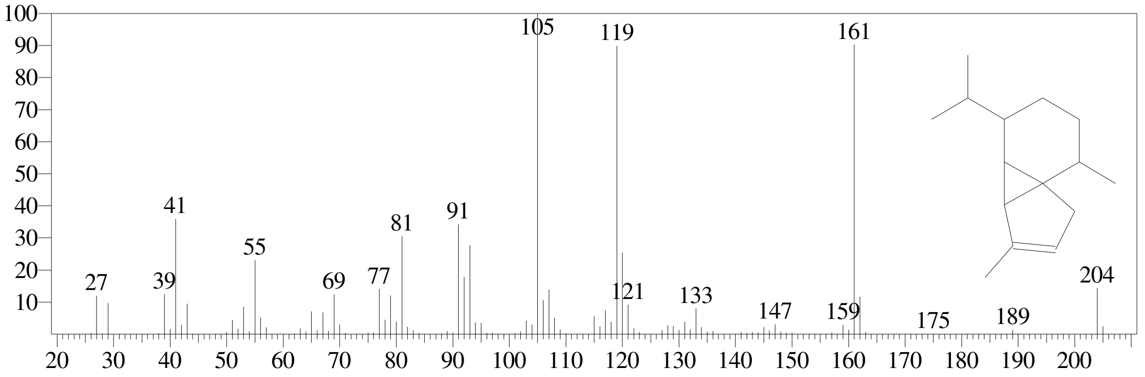

Hit#:4 Entry:62910 Library:NIST23-1.lib
SI:78 Formula:C15H24 CAS:3856-25-5 MolWeight:204 RetIndex:1407
CompName:Copaene $$ Tricyclo[4.4.0.02,7]dec-3-ene, 1,3-dimethyl-8-(1-methylethyl)-, stereoisomer $$ Tricyclo[4.4.0.0

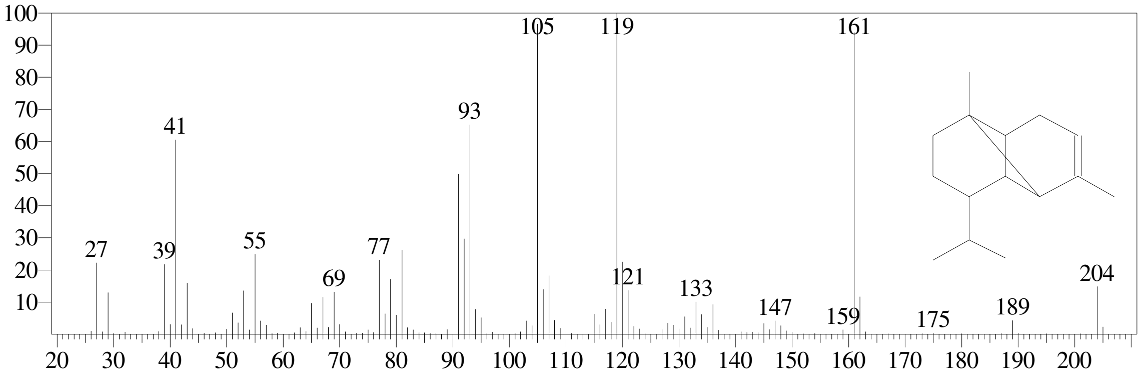

<< Target >>
Line#:21   R.Time:29.733(Scan#:3269) MassPeaks:16
RawMode:Averaged 29.725-29.742(3268-3270) BasePeak:105.05(4724)
BG Mode:None Group 1 - Event 1 Scan

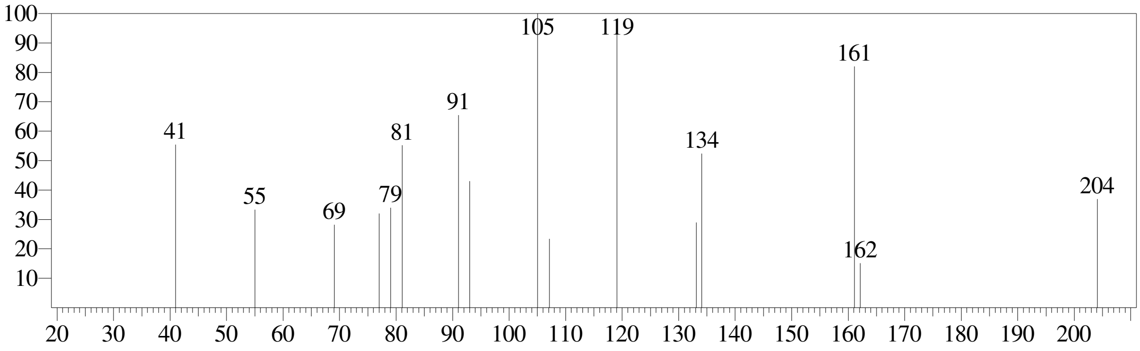

Hit#:5 Entry:63006 Library:NIST23-1.lib
SI:78 Formula:C15H24 CAS:16729-01-4 MolWeight:204 RetIndex:1526
CompName:1-Isopropyl-4,7-dimethyl-1,2,3,5,6,8a-hexahydronaphthalene $$ Cadina-1(10),4-diene $$

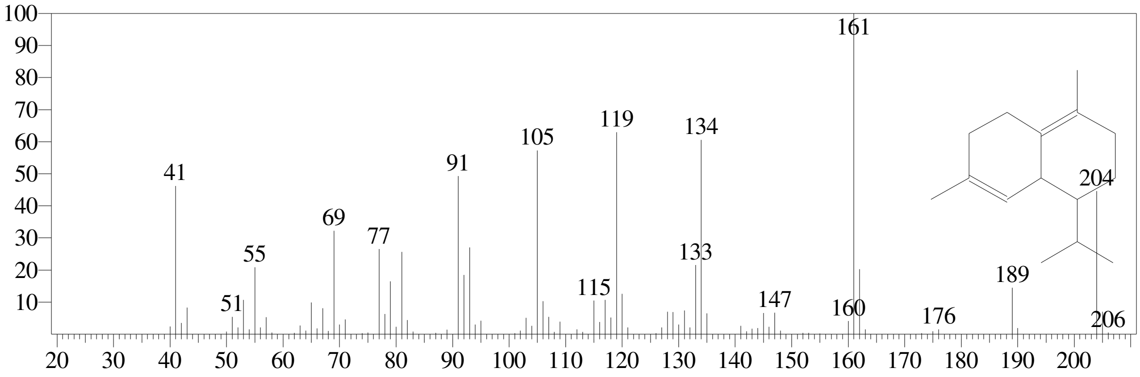

<< Target >>
Line#:22   R.Time:29.992(Scan#:3300) MassPeaks:41
RawMode:Averaged 29.983-30.000(3299-3301) BasePeak:161.10(25480)
BG Mode:None Group 1 - Event 1 Scan

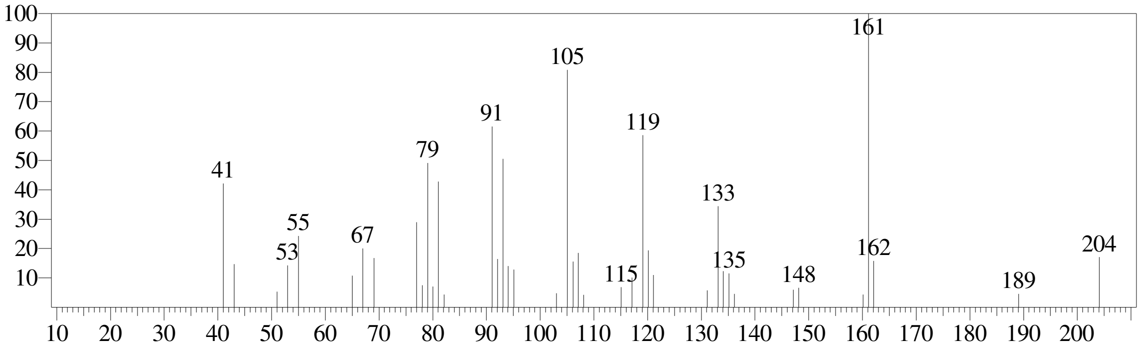

Hit#:1 Entry:25086 Library:NIST23s.lib
SI:94 Formula:C15H24 CAS:30021-74-0 MolWeight:204 RetIndex:1483
CompName:.gamma.-Muurolene $$ Naphthalene, 1,2,3,4,4a,5,6,8a-octahydro-7-methyl-4-methylene-1-(1-methylethyl)-, (

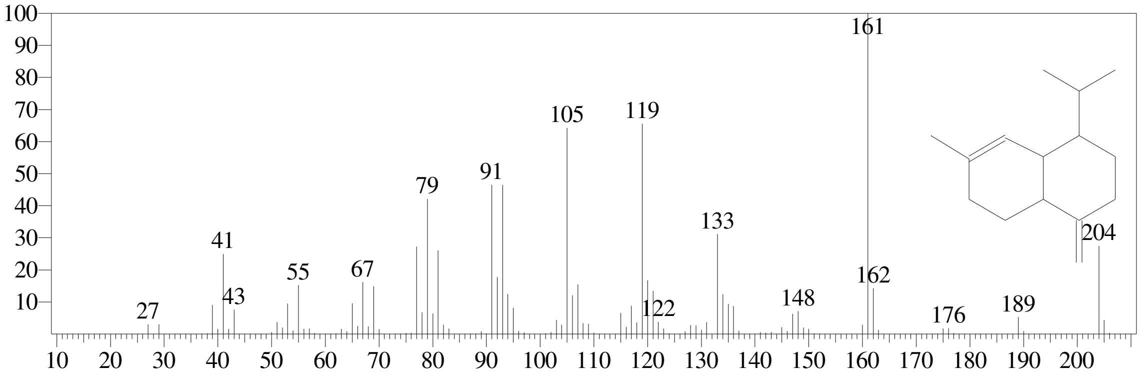

Hit#:2 Entry:25047 Library:NIST23s.lib
SI:94 Formula:C15H24 CAS:30021-74-0 MolWeight:204 RetIndex:1483
CompName:.gamma.-Muurolene $$ Naphthalene, 1,2,3,4,4a,5,6,8a-octahydro-7-methyl-4-methylene-1-(1-methylethyl)-, (

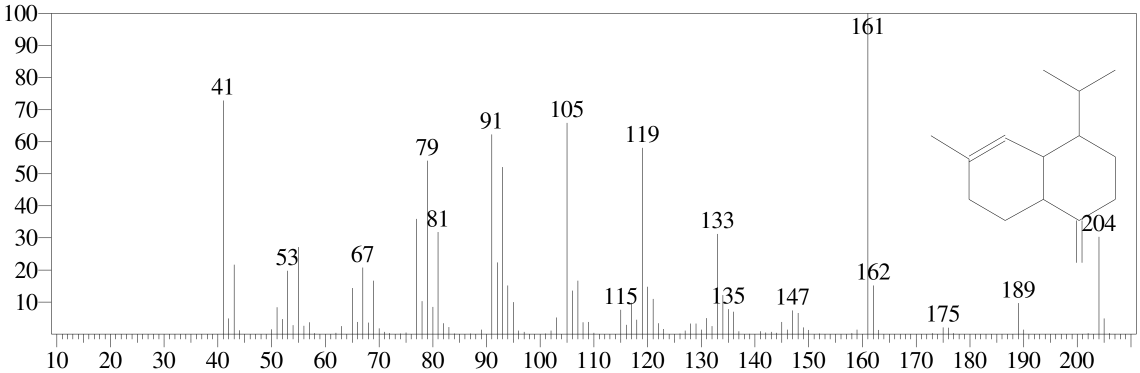

<< Target >>
Line#:22   R.Time:29.992(Scan#:3300) MassPeaks:41
RawMode:Averaged 29.983-30.000(3299-3301) BasePeak:161.10(25480)
BG Mode:None Group 1 - Event 1 Scan

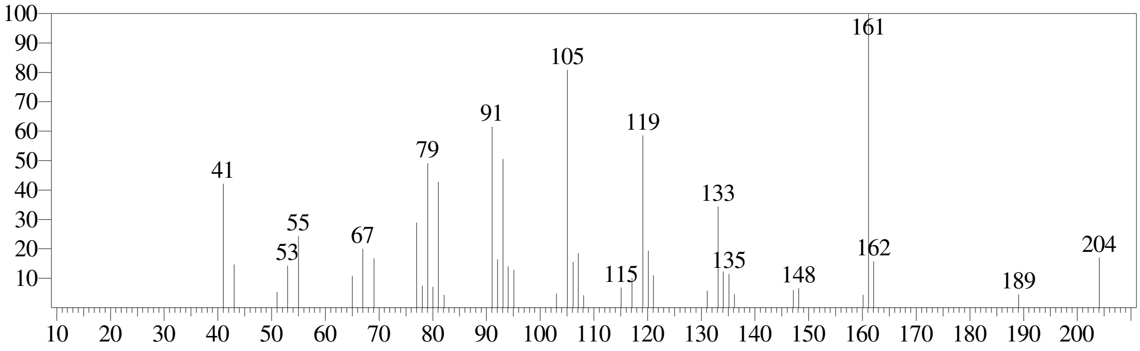

Hit#:3 Entry:25071 Library:NIST23s.lib
SI:93 Formula:C15H24 CAS:483-75-0 MolWeight:204 RetIndex:1500
CompName:Naphthalene, 1,2,4a,5,6,8a-hexahydro-4,7-dimethyl-1-(1-methylethyl)- $$ 1-Isopropyl-4,7-dimethyl-1,2,4a,5,

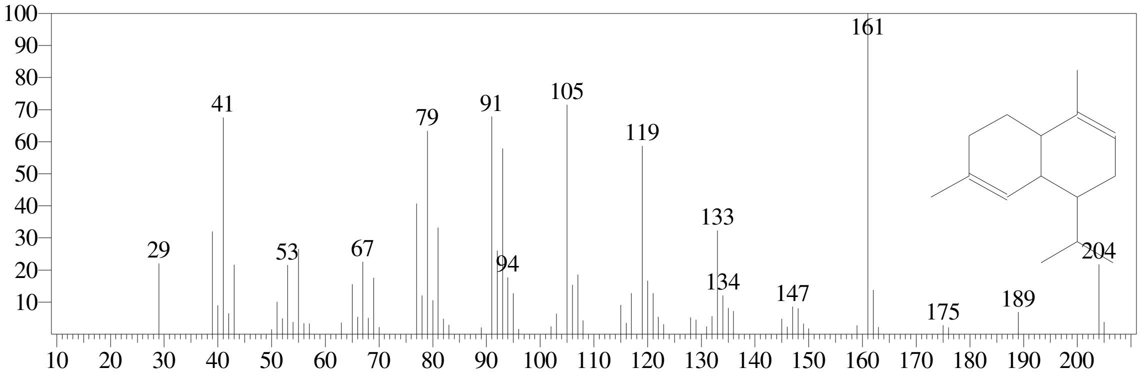

Hit#:4 Entry:62981 Library:NIST23-1.lib
SI:92 Formula:C15H24 CAS:23986-74-5 MolWeight:204 RetIndex:1478
CompName:Germacrene D $$ (S,1Z,6Z)-8-Isopropyl-1-methyl-5-methylenecyclodeca-1,6-diene $$ D-Germacrene $$ 1(1

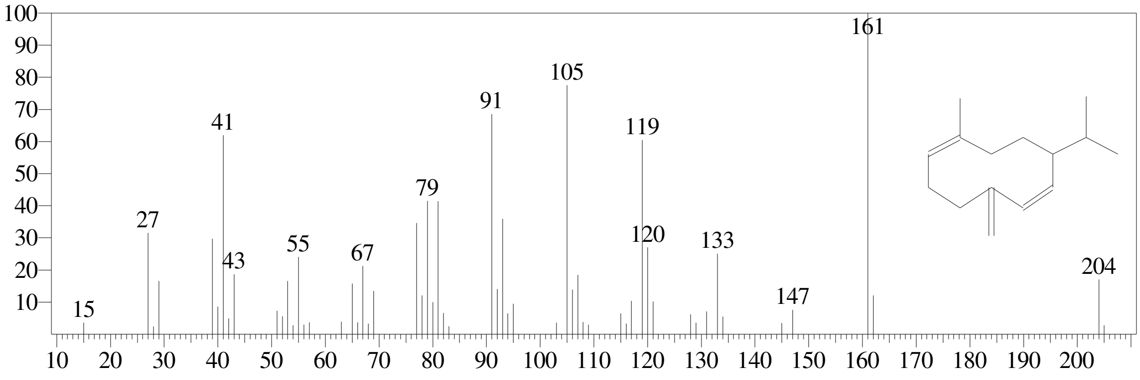

<< Target >>
Line#:22   R.Time:29.992(Scan#:3300) MassPeaks:41
RawMode:Averaged 29.983-30.000(3299-3301) BasePeak:161.10(25480)
BG Mode:None Group 1 - Event 1 Scan

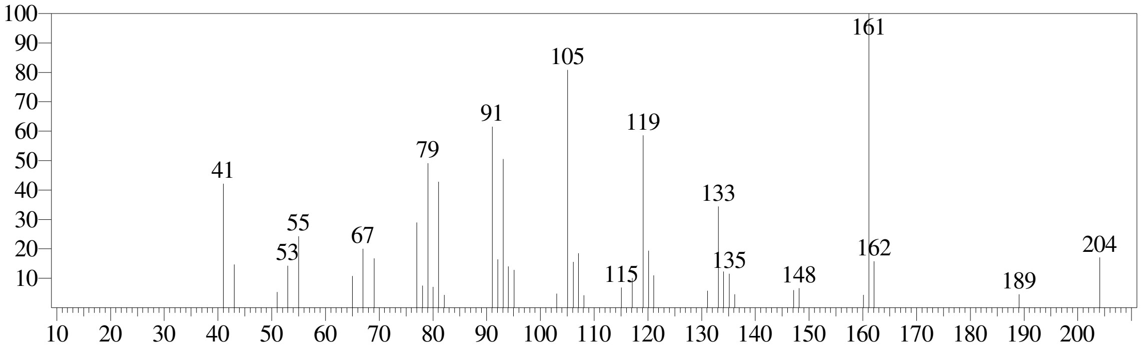

Hit#:5 Entry:25058 Library:NIST23s.lib
SI:92 Formula:C15H24 CAS:30021-74-0 MolWeight:204 RetIndex:1483
CompName:.gamma.-Muurolene $$ Naphthalene, 1,2,3,4,4a,5,6,8a-octahydro-7-methyl-4-methylene-1-(1-methylethyl)-, (

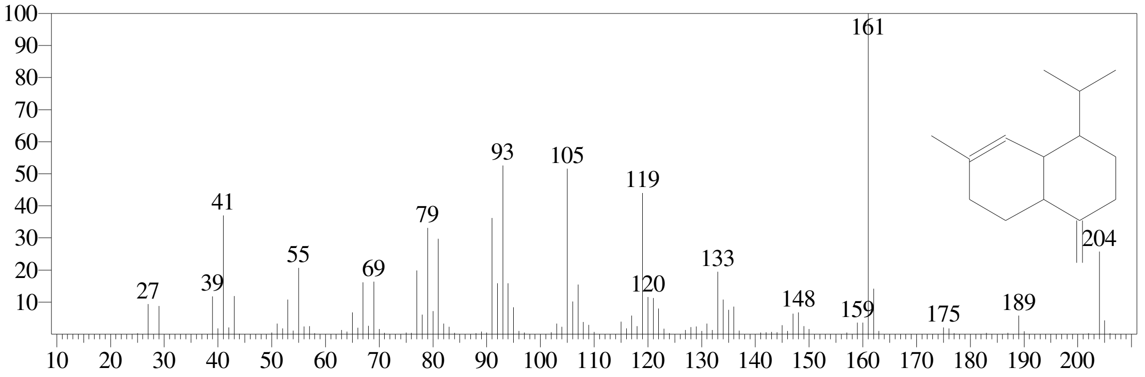

<< Target >>
Line#:23 R.Time:30.133(Scan#:3317) MassPeaks:9
RawMode:Averaged 30.125-30.142(3316-3318) BasePeak:107.10(1847)
BG Mode:Calc. from Peak Group 1 - Event 1 Scan

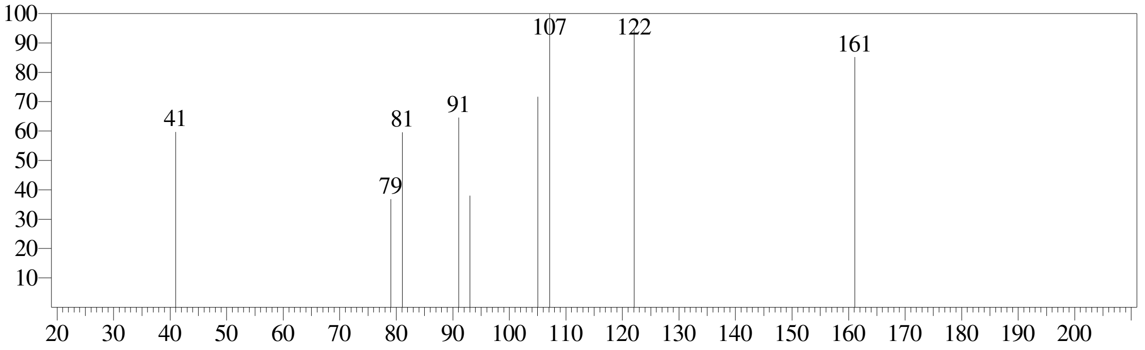

Hit#:1 Entry:25094 Library:NIST23s.lib
SI:78 Formula:C15H24 CAS:123123-37-5 MolWeight:204 RetIndex:1483
CompName:(2S,4aR,8aR)-4a,8-Dimethyl-2-(prop-1-en-2-yl)-1,2,3,4,4a,5,6,8a-octahydronaphthalene $$ Naphthalene, 1,2,

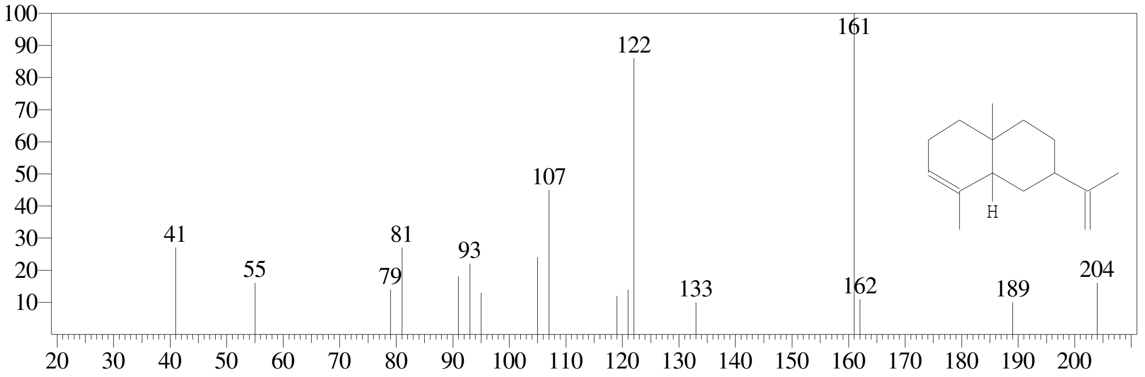

Hit#:2 Entry:63009 Library:NIST23-1.lib
SI:73 Formula:C15H24 CAS:123123-37-5 MolWeight:204 RetIndex:1483
CompName:(2S,4aR,8aR)-4a,8-Dimethyl-2-(prop-1-en-2-yl)-1,2,3,4,4a,5,6,8a-octahydronaphthalene $$ Naphthalene, 1,2,

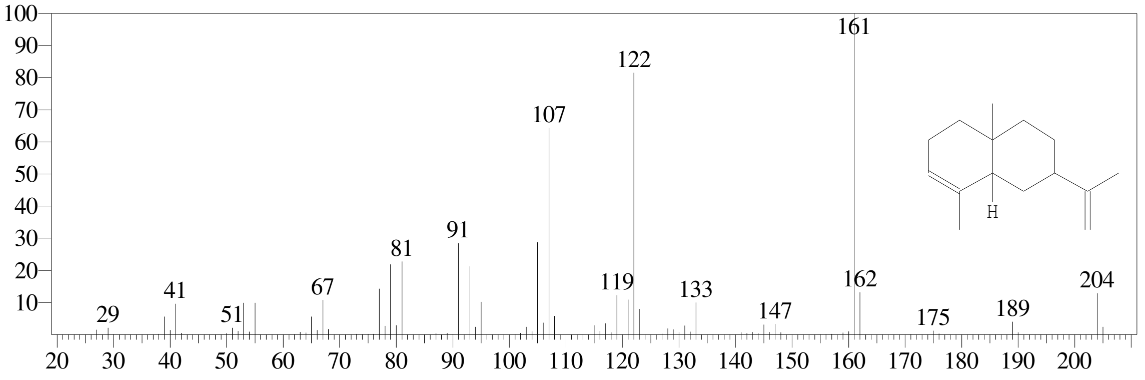

<< Target >>
Line#:23   R.Time:30.133(Scan#:3317) MassPeaks:9
RawMode:Averaged 30.125-30.142(3316-3318) BasePeak:107.10(1847)
BG Mode:Calc. from Peak Group 1 - Event 1 Scan

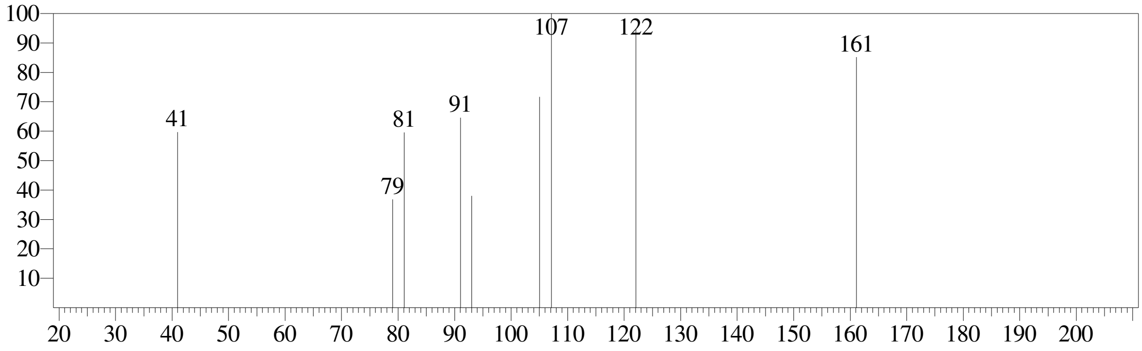

Hit#:3 Entry:62942 Library:NIST23-1.lib
SI:72 Formula:C15H24 CAS:56633-28-4 MolWeight:204 RetIndex:1410
CompName:(-)-.alpha.-Panasinsen $$ (2aR,4aS,8aR)-2,2,4a,8-Tetramethyl-1,2,2a,3,4,4a,5,6-octahydrocyclobuta[c]indene

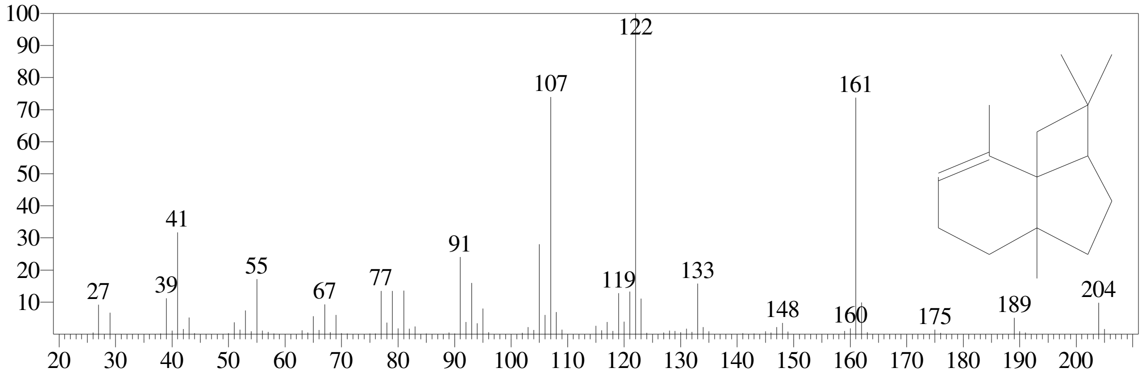

Hit#:4 Entry:25093 Library:NIST23s.lib
SI:70 Formula:C15H24 CAS:6813-21-4 MolWeight:204 RetIndex:1522
CompName:Selina-3,7(11)-diene $$ Naphthalene, 1,2,3,4,4a,5,6,8a-octahydro-4a,8-dimethyl-2-(1-methylethylidene)-, (4a

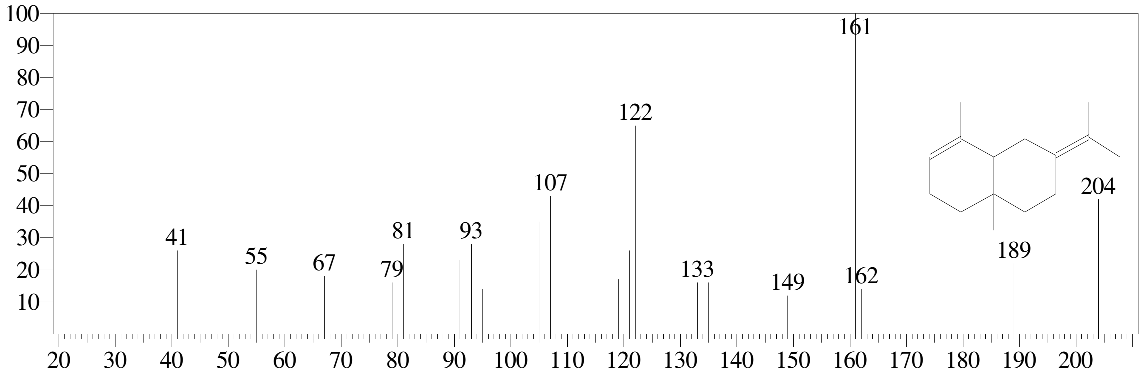

<< Target >>
Line#:23   R.Time:30.133(Scan#:3317) MassPeaks:9
RawMode:Averaged 30.125-30.142(3316-3318) BasePeak:107.10(1847)
BG Mode:Calc. from Peak Group 1 - Event 1 Scan

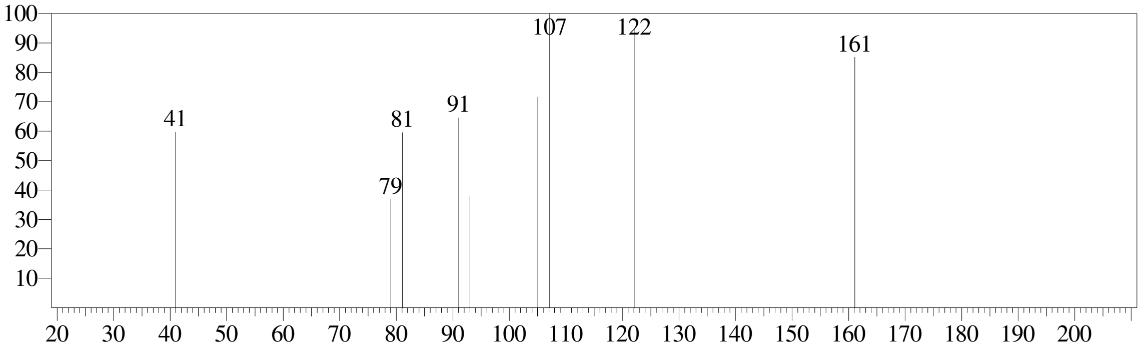

Hit#:5 Entry:25028 Library:NIST23s.lib
SI:69 Formula:C15H24 CAS:56633-28-4 MolWeight:204 RetIndex:1410
CompName:(-)-.alpha.-Panasinsen $$ (2aR,4aS,8aR)-2,2,4a,8-Tetramethyl-1,2,2a,3,4,4a,5,6-octahydrocyclobuta[c]indene

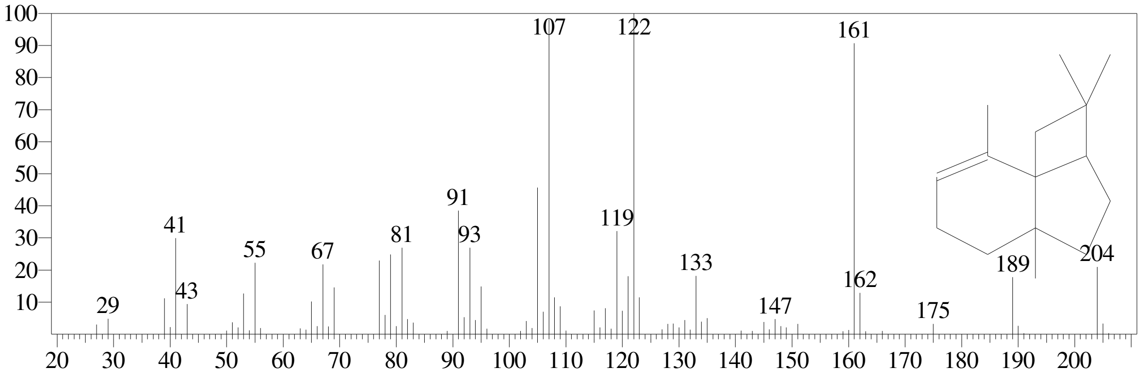

<< Target >>
Line#:24 R.Time:30.375(Scan#:3346) MassPeaks:58
RawMode:Averaged 30.367-30.383(3345-3347) BasePeak:119.10(50485)
BG Mode:None Group 1 - Event 1 Scan

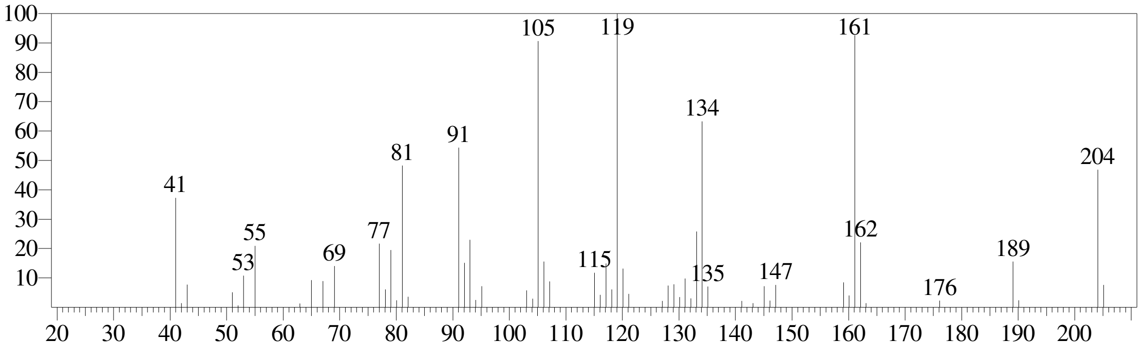

Hit#:1 Entry:63005 Library:NIST23-1.lib
SI:93 Formula:C15H24 CAS:189165-79-5 MolWeight:204 RetIndex:1526
CompName:Amorphene,delta-

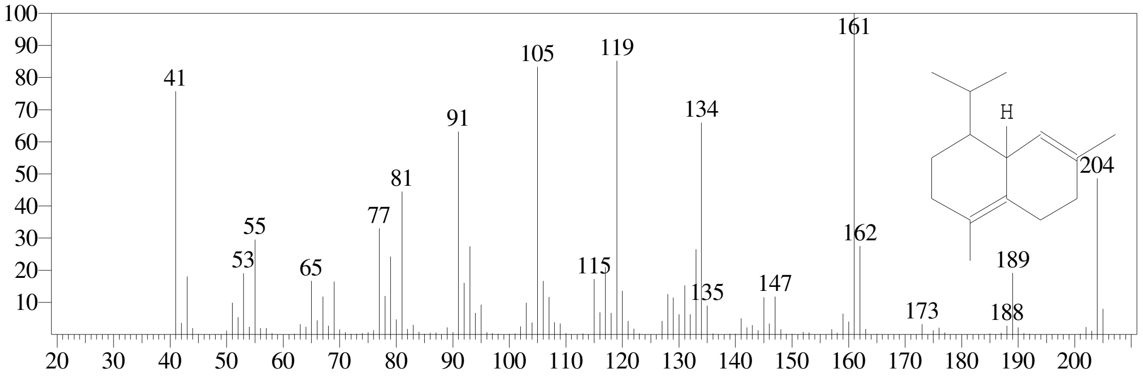

Hit#:2 Entry:63006 Library:NIST23-1.lib
SI:92 Formula:C15H24 CAS:16729-01-4 MolWeight:204 RetIndex:1526
CompName:1-Isopropyl-4,7-dimethyl-1,2,3,5,6,8a-hexahydronaphthalene $$ Cadina-1(10),4-diene $$

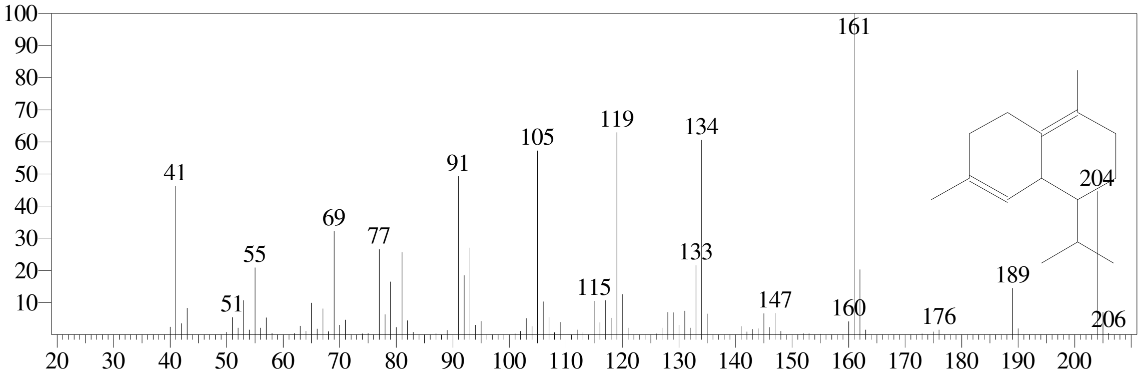

<< Target >>
Line#:24   R.Time:30.375(Scan#:3346) MassPeaks:58
RawMode:Averaged 30.367-30.383(3345-3347) BasePeak:119.10(50485)
BG Mode:None Group 1 - Event 1 Scan

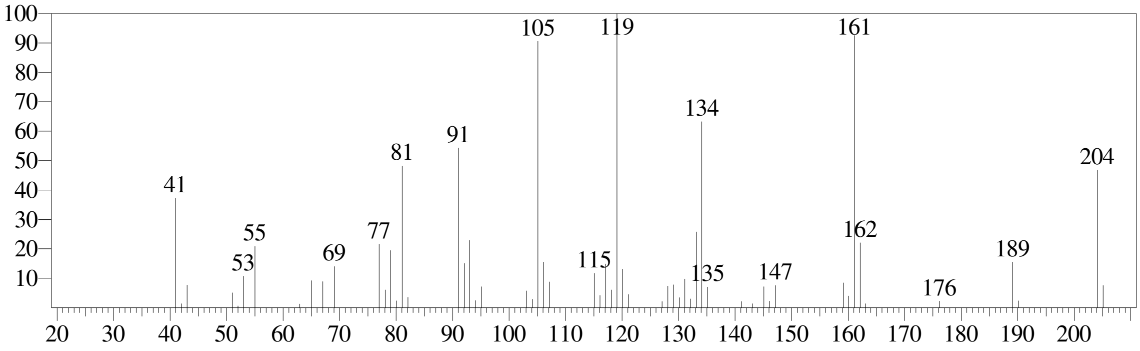

Hit#:3 Entry:25090 Library:NIST23s.lib
SI:92 Formula:C15H24 CAS:483-76-1 MolWeight:204 RetIndex:1526
CompName:Naphthalene, 1,2,3,5,6,8a-hexahydro-4,7-dimethyl-1-(1-methylethyl)-, (1S-cis)- $$ Cadina-1(10),4-diene $$.

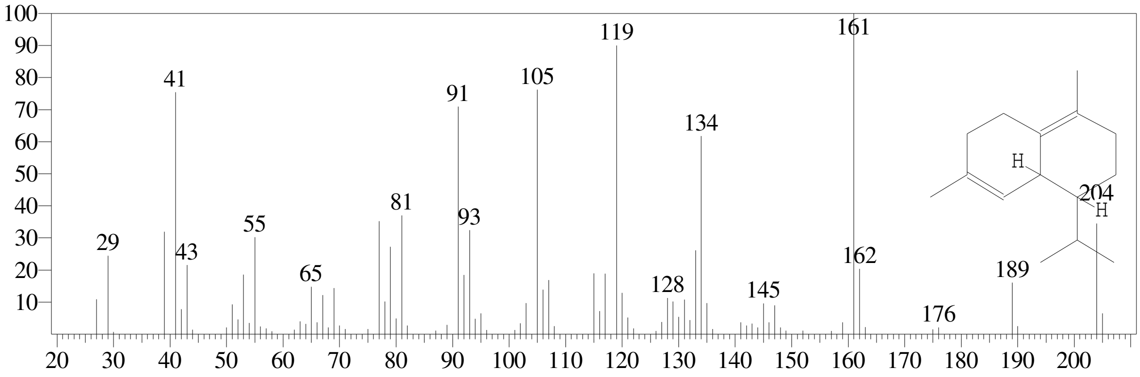

Hit#:4 Entry:25112 Library:NIST23s.lib
SI:90 Formula:C15H24 CAS:483-76-1 MolWeight:204 RetIndex:1526
CompName:Naphthalene, 1,2,3,5,6,8a-hexahydro-4,7-dimethyl-1-(1-methylethyl)-, (1S-cis)- $$ Cadina-1(10),4-diene $$.

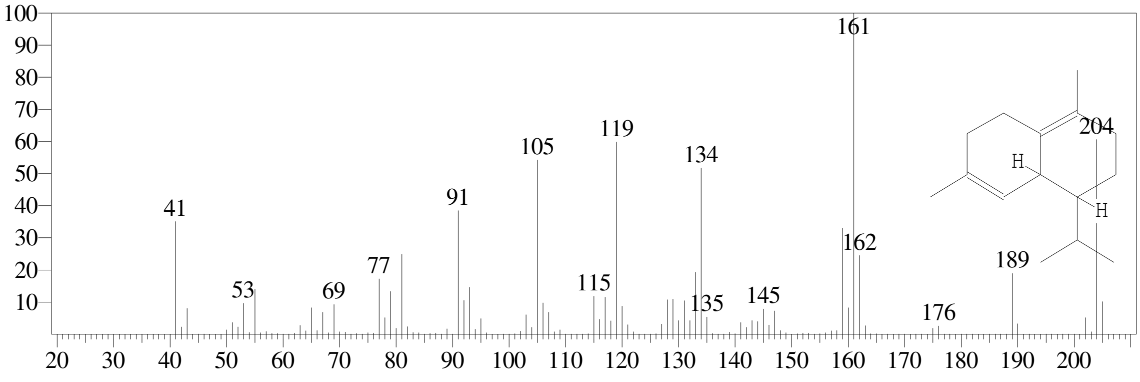

<< Target >>
Line#:24   R.Time:30.375(Scan#:3346) MassPeaks:58
RawMode:Averaged 30.367-30.383(3345-3347) BasePeak:119.10(50485)
BG Mode:None Group 1 - Event 1 Scan

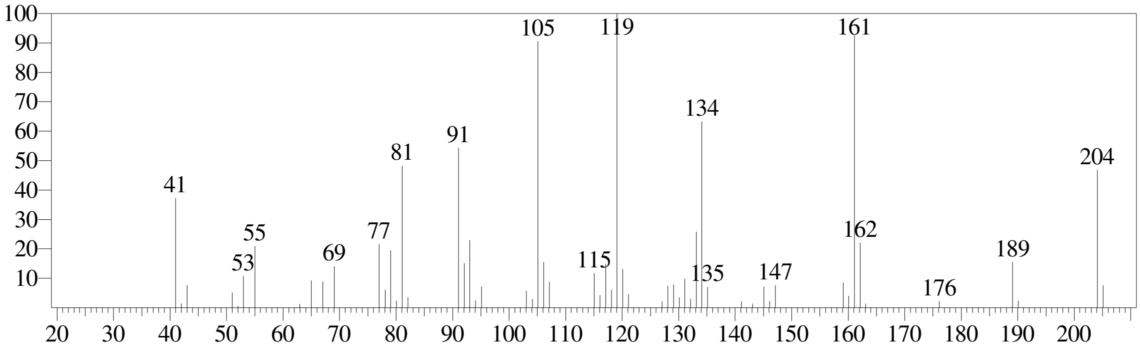

Hit#:5 Entry:24949 Library:NIST23s.lib
SI:90 Formula:C15H24 CAS:17699-14-8 MolWeight:204 RetIndex:1381
CompName:.alpha.-Cubebene $$ 1H-Cyclopenta[1,3]cyclopropa[1,2]benzene, 3a,3b,4,5,6,7-hexahydro-3,7-dimethyl-4-(1

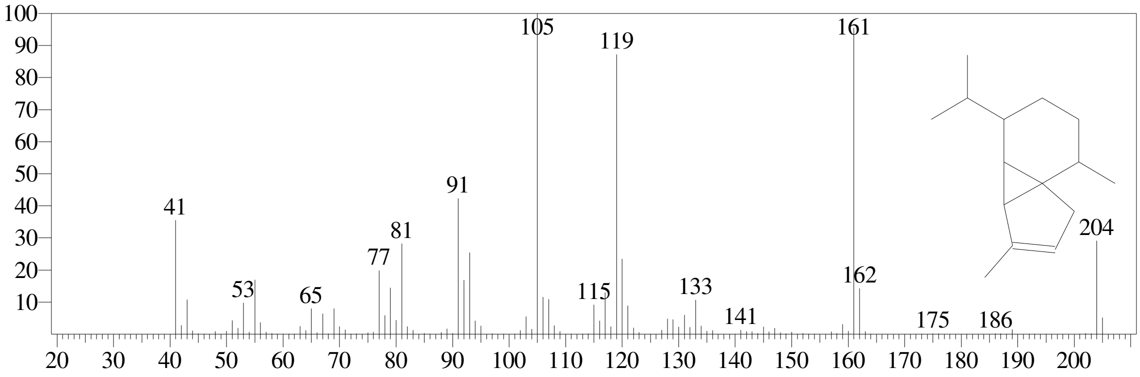

<< Target >>
Line#:25 R.Time:30.908(Scan#:3410) MassPeaks:30
RawMode:Averaged 30.900-30.917(3409-3411) BasePeak:105.10(17815)
BG Mode:Calc. from Peak Group 1 - Event 1 Scan

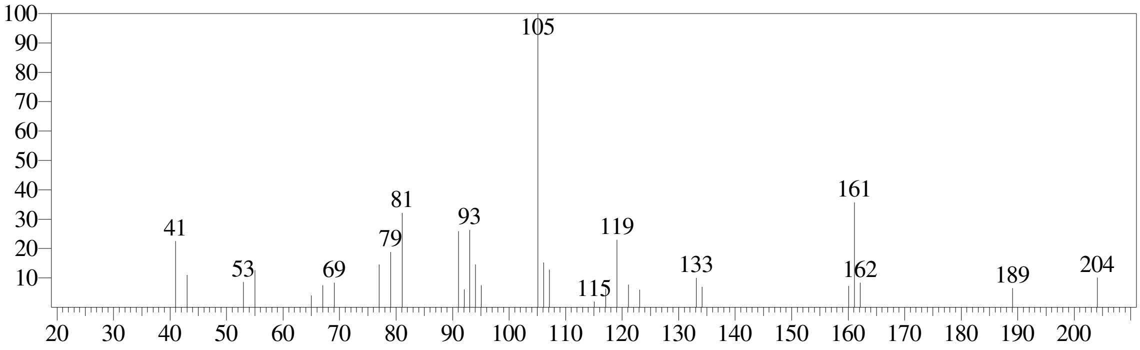

Hit#:1 Entry:62856 Library:NIST23-1.lib
SI:90 Formula:C15H24 CAS:10208-80-7 MolWeight:204 RetIndex:1500
CompName:.alpha.-Muurolene $$ Naphthalene, 1,2,4a,5,6,8a-hexahydro-4,7-dimethyl-1-(1-methylethyl)-, (1S,4aS,8aR)-

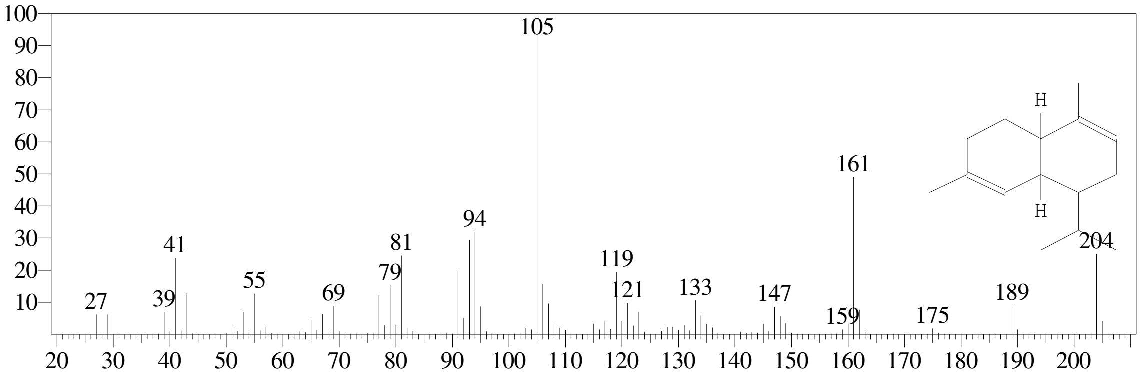

Hit#:2 Entry:24945 Library:NIST23s.lib
SI:89 Formula:C15H24 CAS:24406-05-1 MolWeight:204 RetIndex:1499
CompName:Naphthalene, 1,2,4a,5,6,8a-hexahydro-4,7-dimethyl-1-(1-methylethyl)-, [1S-(1.alpha.,4a.beta.,8a.alpha.)]- $$

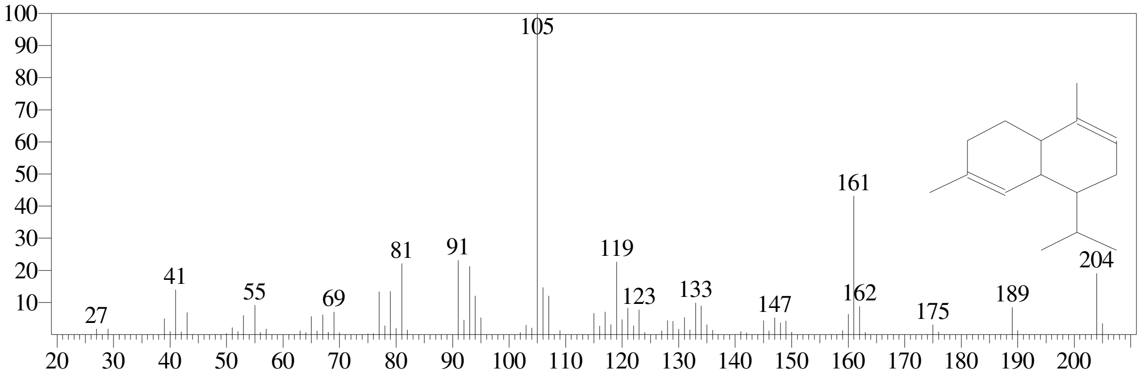

<< Target >>
Line#:25 R.Time:30.908(Scan#:3410) MassPeaks:30
RawMode:Averaged 30.900-30.917(3409-3411) BasePeak:105.10(17815)
BG Mode:Calc. from Peak Group 1 - Event 1 Scan

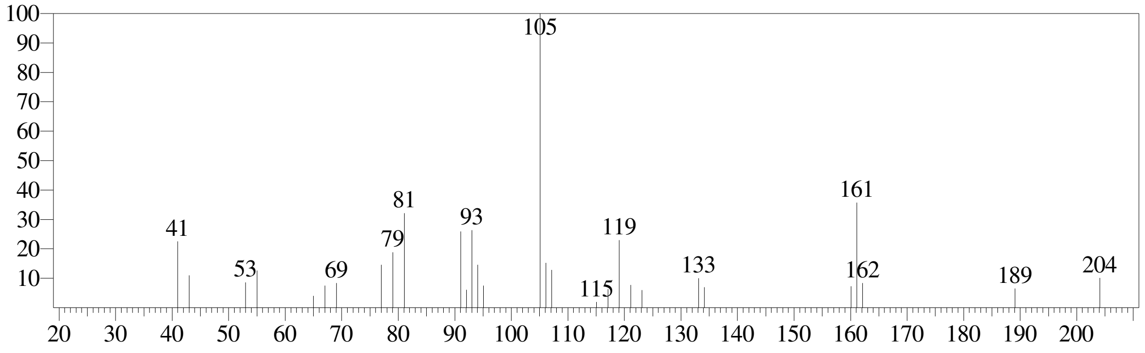

Hit#:3 Entry:62857 Library:NIST23-1.lib
SI:89 Formula:C15H24 CAS:483-75-0 MolWeight:204 RetIndex:1500
CompName:Naphthalene, 1,2,4a,5,6,8a-hexahydro-4,7-dimethyl-1-(1-methylethyl)- $$ 1-Isopropyl-4,7-dimethyl-1,2,4a,5,

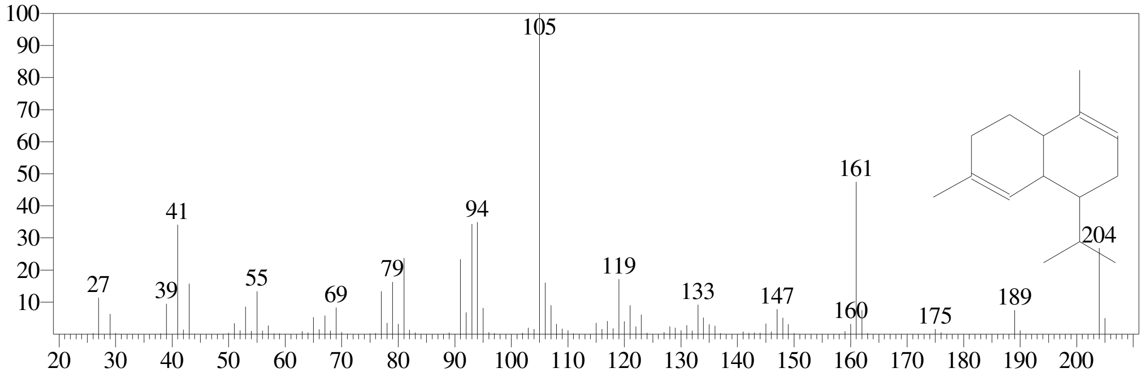

Hit#:4 Entry:24947 Library:NIST23s.lib
SI:89 Formula:C15H24 CAS:31983-22-9 MolWeight:204 RetIndex:1498
CompName:Naphthalene, 1,2,4a,5,6,8a-hexahydro-4,7-dimethyl-1-(1-methylethyl)-, (1.alpha.,4a.alpha.,8a.alpha.)- $$ [1.a

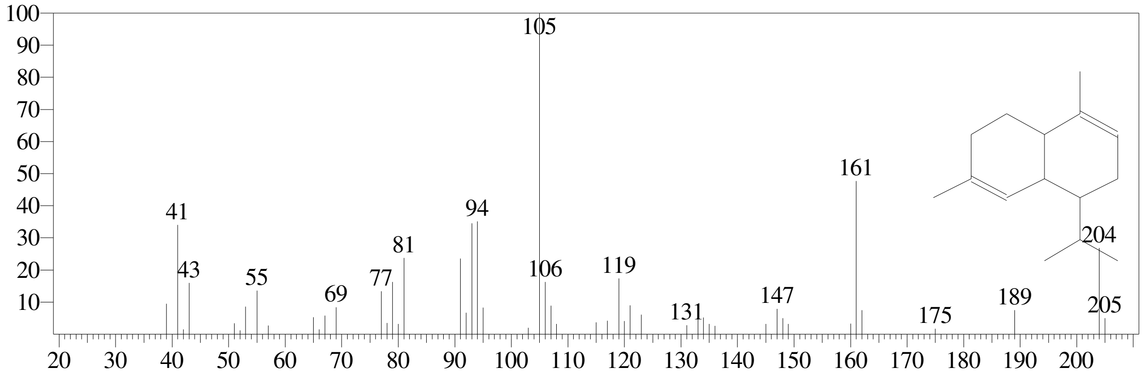

<< Target >>
Line#:25 R.Time:30.908(Scan#:3410) MassPeaks:30
RawMode:Averaged 30.900-30.917(3409-3411) BasePeak:105.10(17815)
BG Mode:Calc. from Peak Group 1 - Event 1 Scan

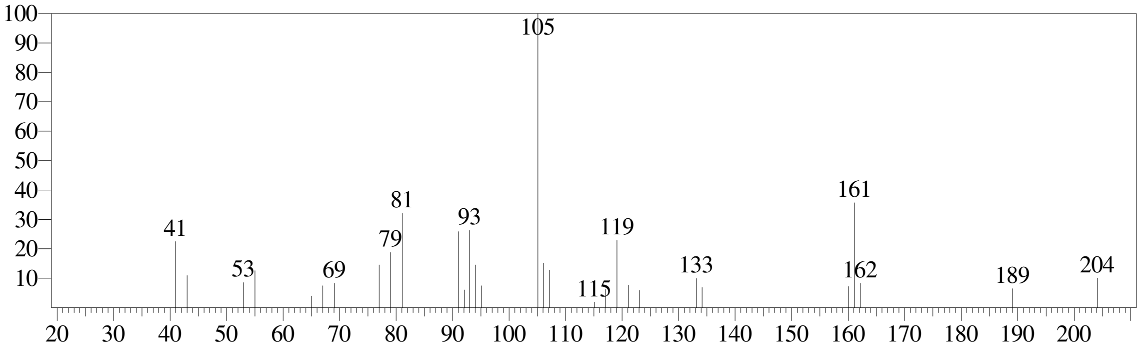

Hit#:5 Entry:62854 Library:NIST23-1.lib
SI:88 Formula:C15H24 CAS:17627-24-6 MolWeight:204 RetIndex:1500
CompName:Naphthalene, 1,2,4a,5,6,8a-hexahydro-4,7-dimethyl-1-(1-methylethyl)-,[1R-(1.alpha.,4a.alpha.,8a.alpha.)]- $$

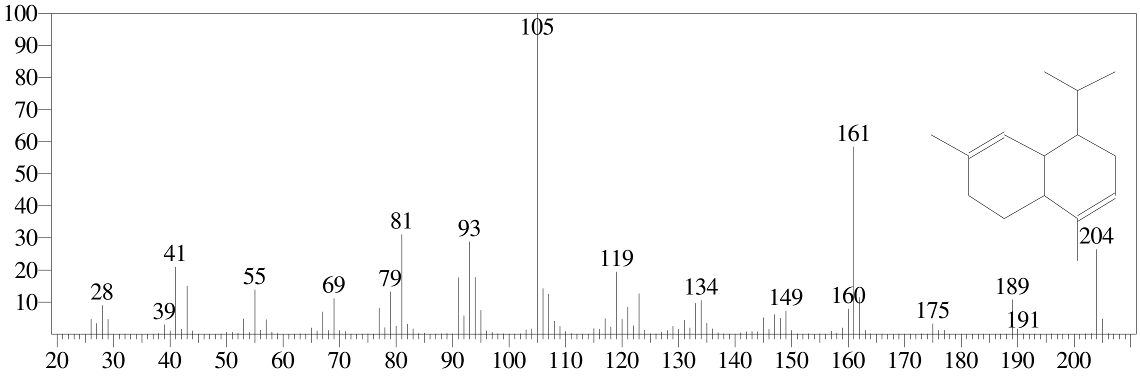

<< Target >>
Line#:26 R.Time:32.467(Scan#:3597) MassPeaks:9
RawMode:Averaged 32.458-32.475(3596-3598) BasePeak:43.00(2224)
BG Mode:None Group 1 - Event 1 Scan

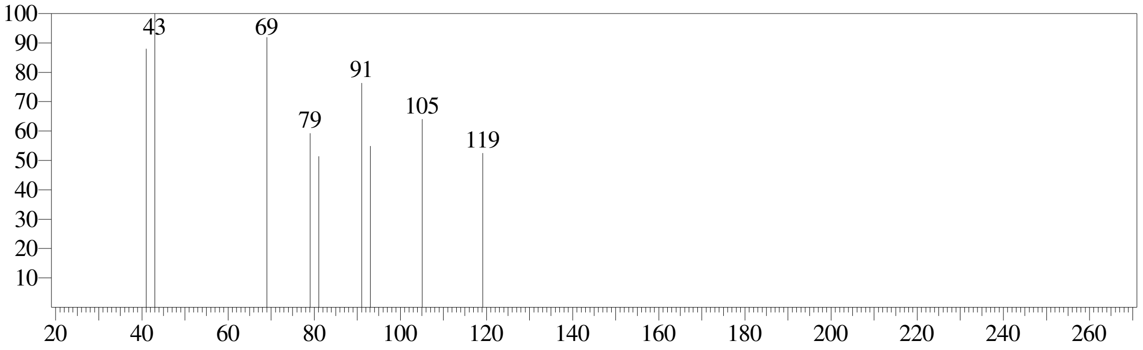

Hit#:1 Entry:23042 Library:NIST23s.lib
SI:71 Formula:C12H20O2 CAS:20777-39-3 MolWeight:196 RetIndex:1297
CompName:4-Hexen-1-ol, 5-methyl-2-(1-methylethenyl)-, acetate, (R)- $$ 4-Hexen-1-ol, 5-methyl-2-(1-methylethenyl)-,

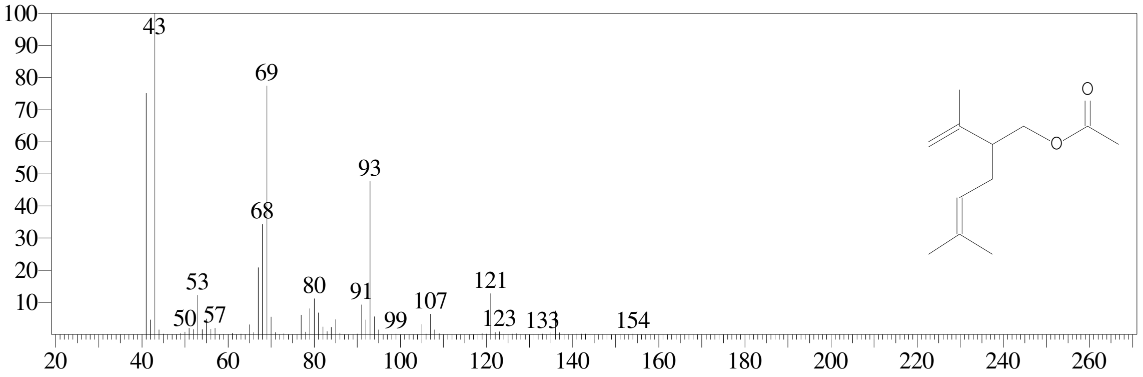

Hit#:2 Entry:24823 Library:NIST23s.lib
SI:71 Formula:C15H24 CAS:26560-14-5 MolWeight:204 RetIndex:1509
CompName:1,3,6,10-Dodecatetraene, 3,7,11-trimethyl-, (Z,E)- $$ (Z,E)-.alpha.-Farnesene $$ (3Z,6E)-3,7,11-Trimethyl-1.

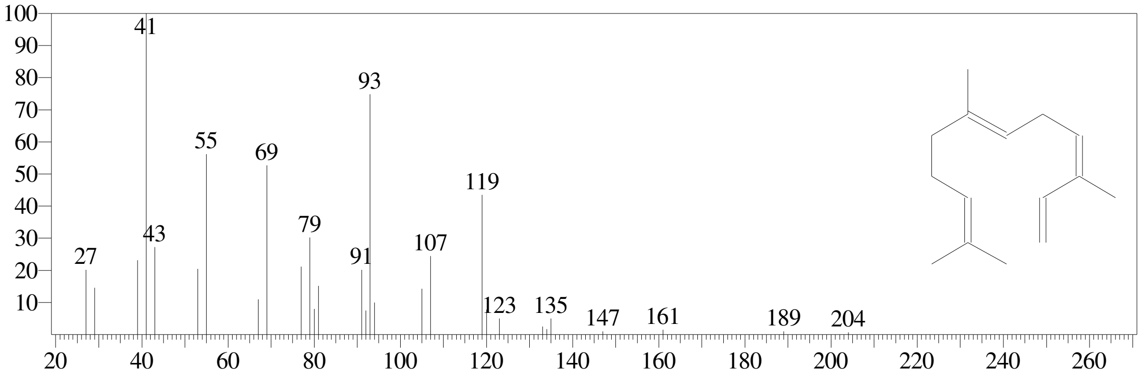

<< Target >>
Line#:26   R.Time:32.467(Scan#:3597) MassPeaks:9
RawMode:Averaged 32.458-32.475(3596-3598) BasePeak:43.00(2224)
BG Mode:None Group 1 - Event 1 Scan

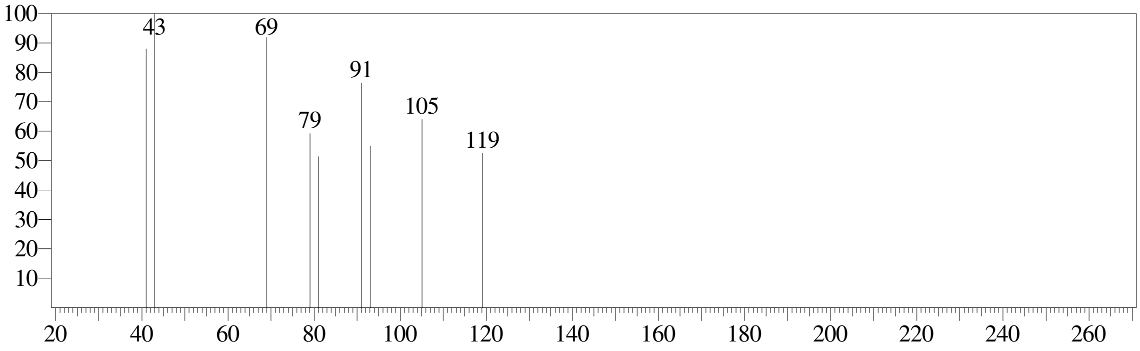

Hit#:3 Entry:135613 Library:NIST23-1.lib
SI:71 Formula:C17H28O2 CAS:122346-20-7 MolWeight:264 RetIndex:1749
CompName:Sesquilavandulyl acetate,E-

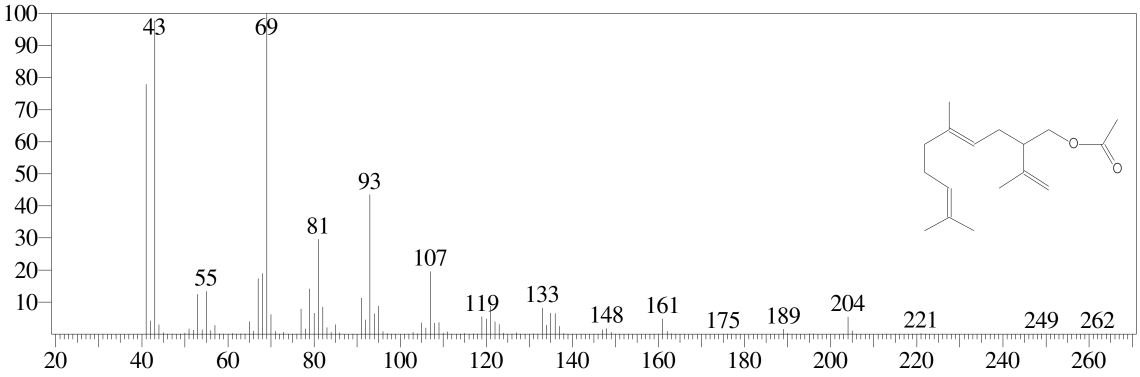

Hit#:4 Entry:24850 Library:NIST23s.lib
SI:69 Formula:C15H24 CAS:18794-84-8 MolWeight:204 RetIndex:1465
CompName:(E)-.beta.-Farnesene $$ 1,6,10-Dodecatriene, 7,11-dimethyl-3-methylene-, (E)- $$ 7,11-Dimethyl-3-methylen

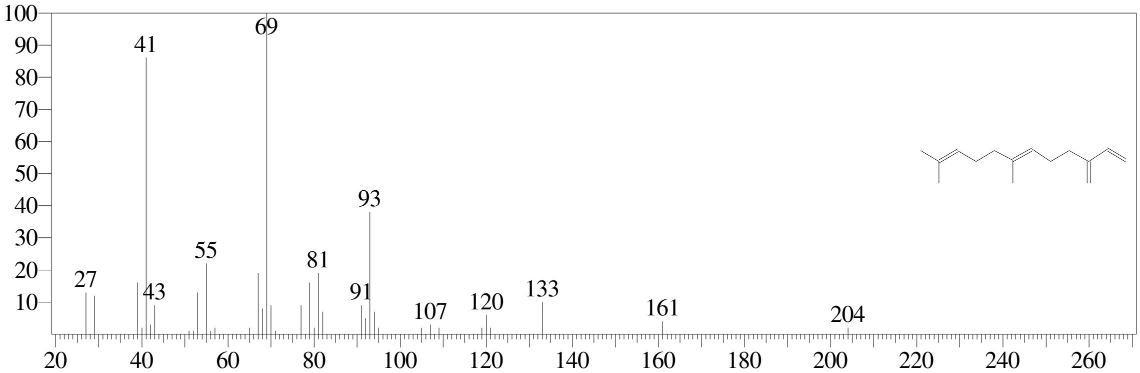

<< Target >>
Line#:26   R.Time:32.467(Scan#:3597) MassPeaks:9
RawMode:Averaged 32.458-32.475(3596-3598) BasePeak:43.00(2224)
BG Mode:None Group 1 - Event 1 Scan

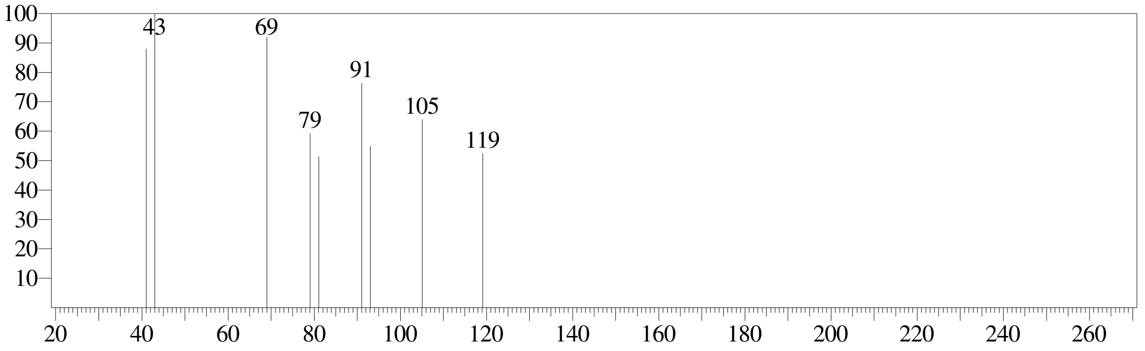

Hit#:5 Entry:80788 Library:NIST23-1.lib
SI:69 Formula:C15H24O CAS:2387-68-0 MolWeight:220 RetIndex:1558
CompName:6,10-Dodecadien-1-yn-3-ol, 3,7,11-trimethyl- $$ Deca-4,8-dienol, 1-ethynyl-1,5,9-trimethyl- $$ (6E)-3,7,11-

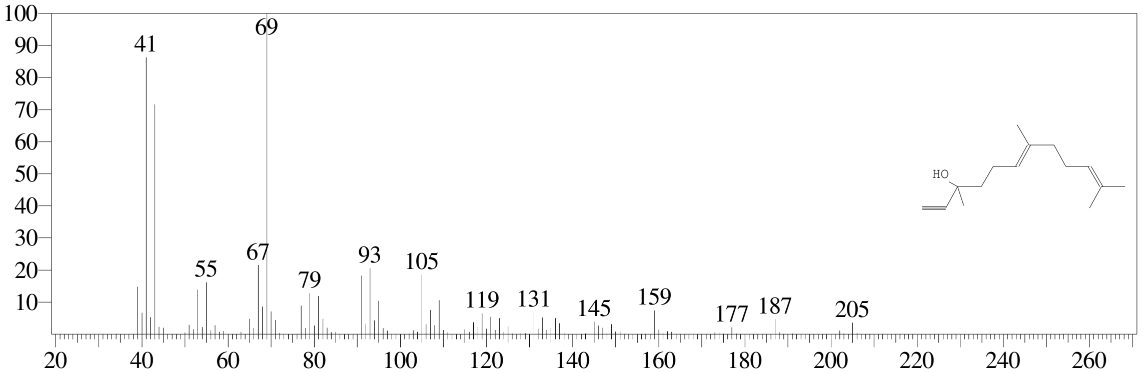

<< Target >>
Line#:27 R.Time:33.817(Scan#:3759) MassPeaks:15
RawMode:Averaged 33.808-33.825(3758-3760) BasePeak:43.00(2872)
BG Mode:Calc. from Peak Group 1 - Event 1 Scan

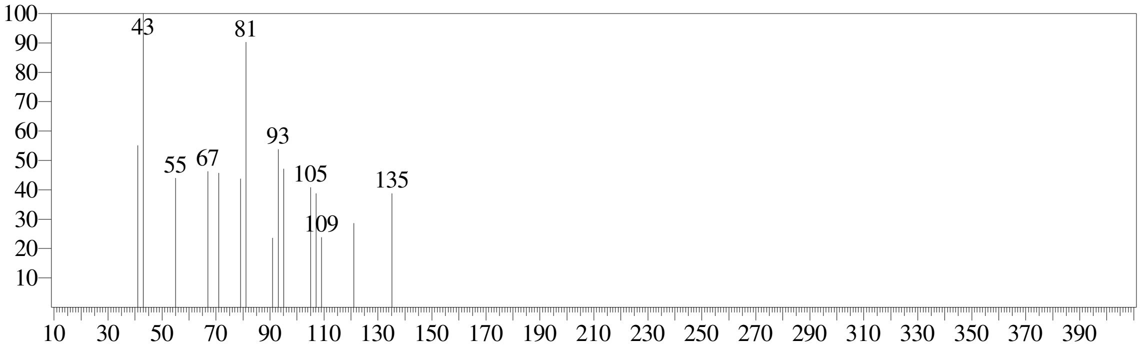

Hit#:1 Entry:46852 Library:NIST23-2.lib
SI:73 Formula:C20H35BrO3 CAS:115346-29-7 MolWeight:402 RetIndex:2941
CompName:1,2-Pentanediol, 5-(6-bromodecahydro-2-hydroxy-2,5,5a,8a-tetramethyl-1-naphthalenyl)-3-methylene- $$ 3-[

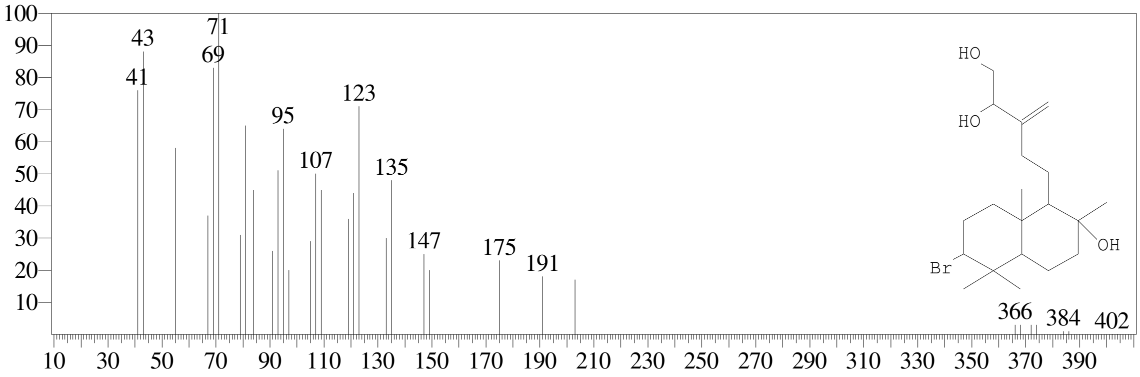

Hit#:2 Entry:223847 Library:NIST23-1.lib
SI:73 Formula:C21H30OS CAS:0-00-0 MolWeight:330 RetIndex:2502
CompName:Oxirane, 2,2-dimethyl-3-[3,7-dimethyl-9-(phenylthio)-3,7-nonadienyl] $$ 3-[(3E,7E)-3,7-Dimethyl-9-(phenyl

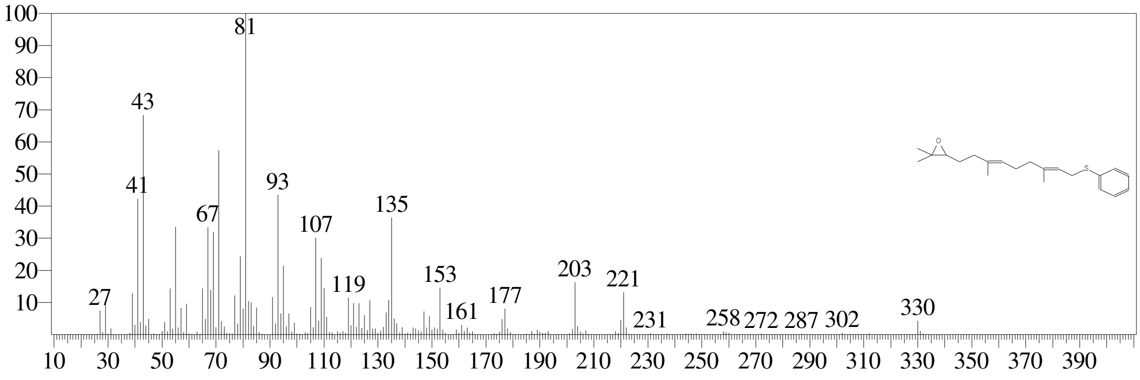

<< Target >>
Line#:27   R.Time:33.817(Scan#:3759) MassPeaks:15
RawMode:Averaged 33.808-33.825(3758-3760) BasePeak:43.00(2872)
BG Mode:Calc. from Peak Group 1 - Event 1 Scan

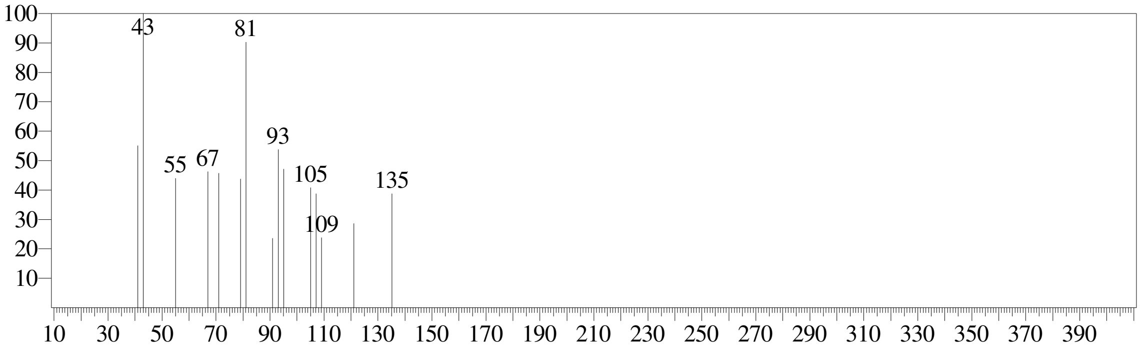

Hit#:3 Entry:85807 Library:NIST23-1.lib
SI:73 Formula:C14H24O2 CAS:0-00-0 MolWeight:224 RetIndex:1464
CompName:(+)-Dihydrocarveol, 2-methylpropionate

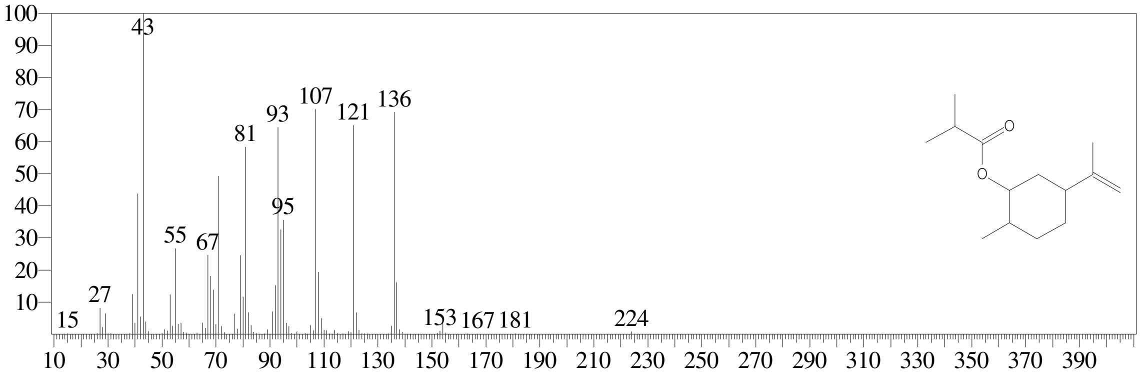

Hit#:4 Entry:243764 Library:NIST23-1.lib
SI:72 Formula:C22H34O3 CAS:55902-84-6 MolWeight:346 RetIndex:2698
CompName:Kauran-18-al, 17-(acetyloxy)-, (4.beta.)- $$ 18-Oxokauran-17-yl acetate # $$

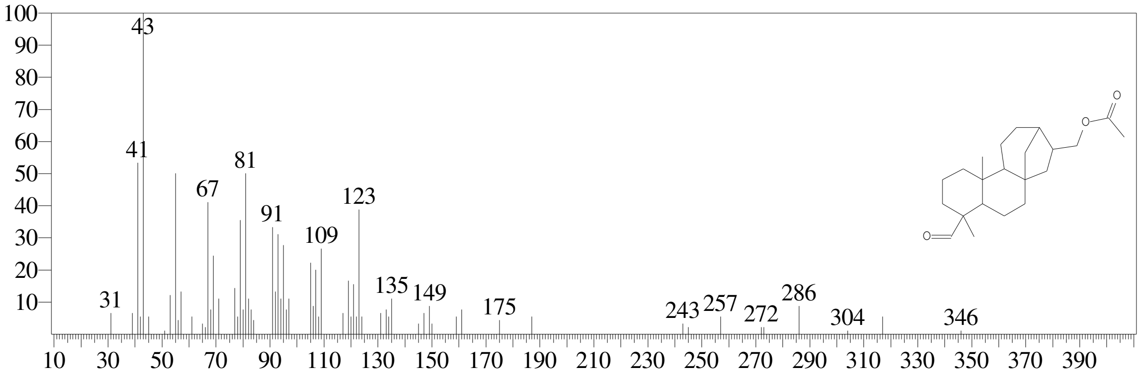

<< Target >>
Line#:27 R.Time:33.817(Scan#:3759) MassPeaks:15
RawMode:Averaged 33.808-33.825(3758-3760) BasePeak:43.00(2872)
BG Mode:Calc. from Peak Group 1 - Event 1 Scan

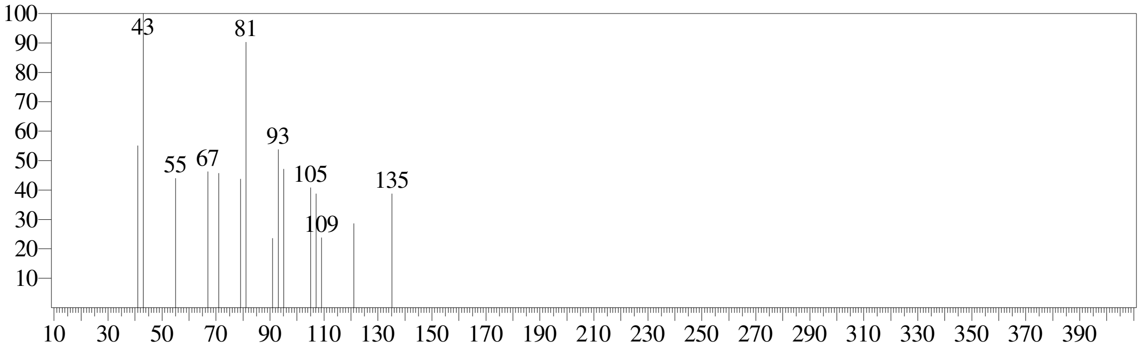

Hit#:5 Entry:28651 Library:NIST23s.lib
SI:72 Formula:C15H26O CAS:5945-72-2 MolWeight:222 RetIndex:1655
CompName:Neointermedeol $$ (1S,4aR,7R,8aR)-1,4a-Dimethyl-7-(prop-1-en-2-yl)decahydronaphthalen-1-ol $$ 1-Napht

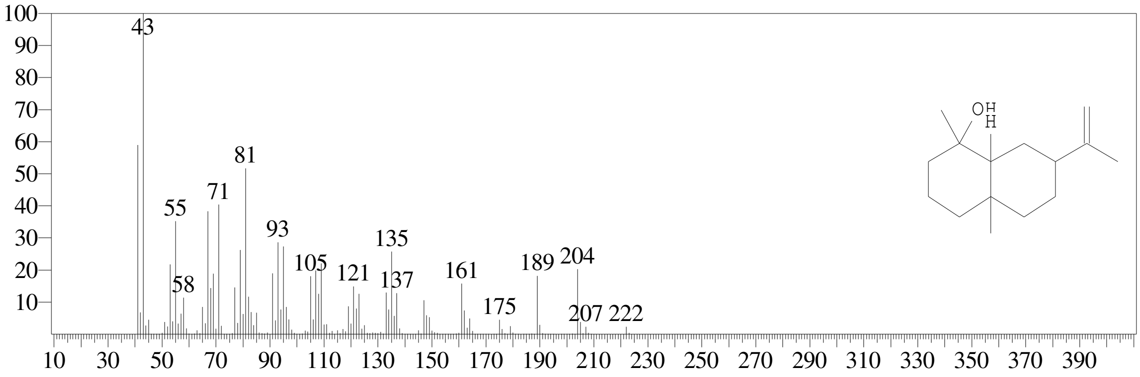

<< Target >>
Line#:28 R.Time:34.908(Scan#:3890) MassPeaks:20
RawMode:Averaged 34.900-34.917(3889-3891) BasePeak:43.00(5072)
BG Mode:None Group 1 - Event 1 Scan

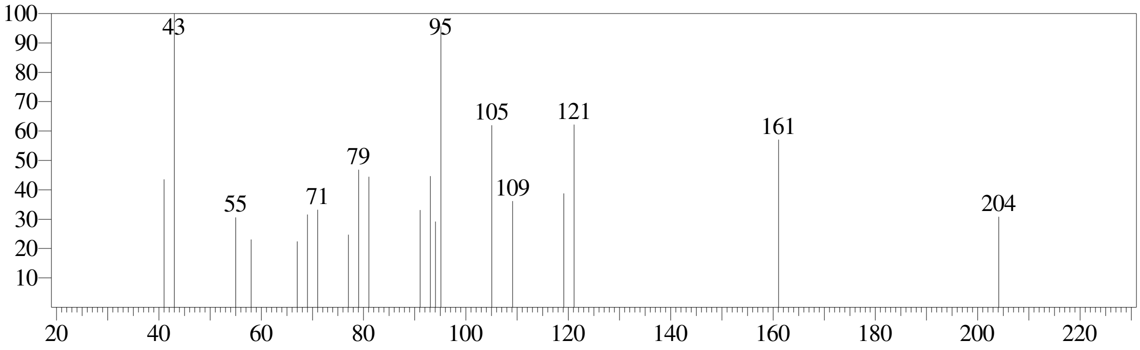

Hit#:1 Entry:28752 Library:NIST23s.lib
SI:83 Formula:C15H26O CAS:5937-11-1 MolWeight:222 RetIndex:1637
CompName:.tau.-Cadinol $$ 4-Isopropyl-1,6-dimethyl-1,2,3,4,4a,7,8,8a-octahydro-1-naphthalenol-, (1S-(1alpha,4alpha,4a

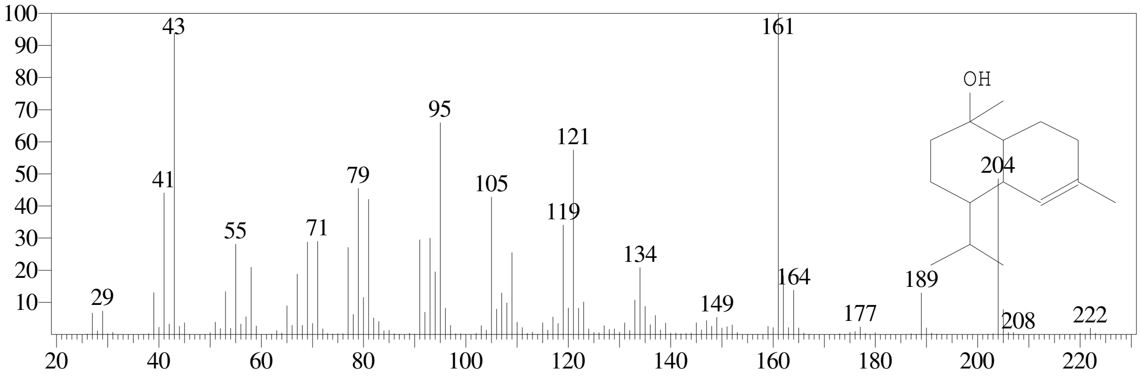

Hit#:2 Entry:83540 Library:NIST23-1.lib
SI:82 Formula:C15H26O CAS:481-34-5 MolWeight:222 RetIndex:1637
CompName:.alpha.-Cadinol $$ 4-Isopropyl-1,6-dimethyl-1,2,3,4,4a,7,8,8a-octahydro-1-naphthalenol # $$ (1R,4S,4aR,8al

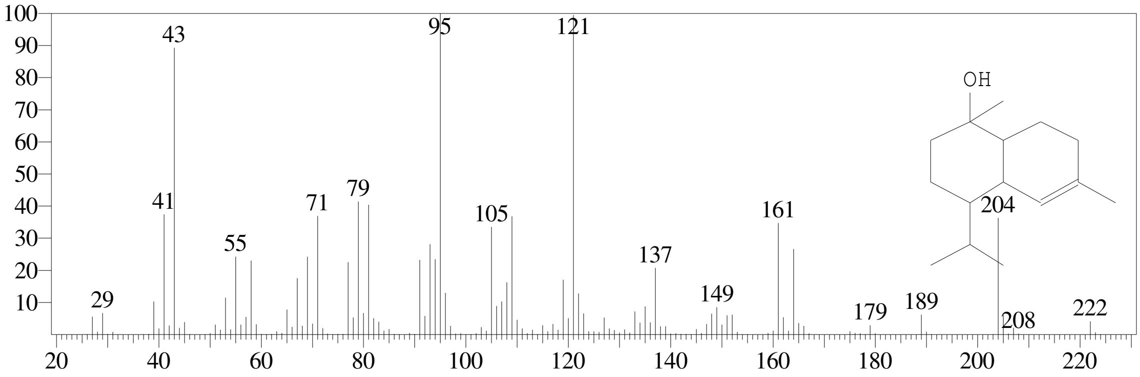

<< Target >>
Line#:28 R.Time:34.908(Scan#:3890) MassPeaks:20
RawMode:Averaged 34.900-34.917(3889-3891) BasePeak:43.00(5072)
BG Mode:None Group 1 - Event 1 Scan

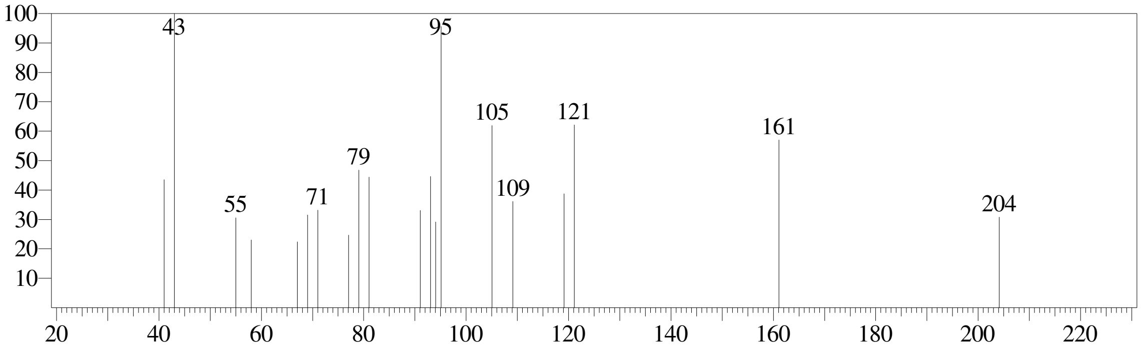

Hit#:3 Entry:83541 Library:NIST23-1.lib
SI:82 Formula:C15H26O CAS:19912-62-0 MolWeight:222 RetIndex:1635
CompName:.tau.-Muurolol $$ 4-Isopropyl-1,6-dimethyl-1,2,3,4,4a,7,8,8a-octahydro-1-naphthalenol-, [1S-(1.alpha.,4.alph

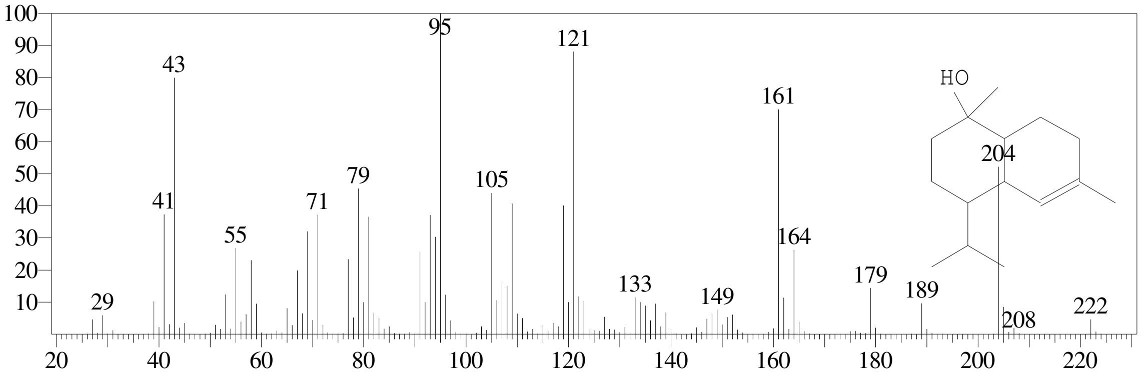

Hit#:4 Entry:28664 Library:NIST23s.lib
SI:82 Formula:C15H26O CAS:19912-62-0 MolWeight:222 RetIndex:1635
CompName:.tau.-Muurolol $$ 4-Isopropyl-1,6-dimethyl-1,2,3,4,4a,7,8,8a-octahydro-1-naphthalenol-, [1S-(1.alpha.,4.alph

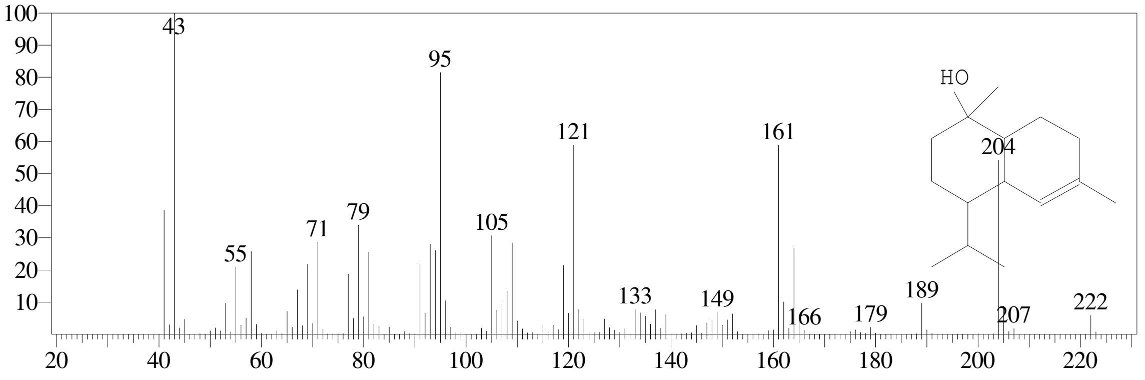

<< Target >>
Line#:28 R.Time:34.908(Scan#:3890) MassPeaks:20
RawMode:Averaged 34.900-34.917(3889-3891) BasePeak:43.00(5072)
BG Mode:None Group 1 - Event 1 Scan

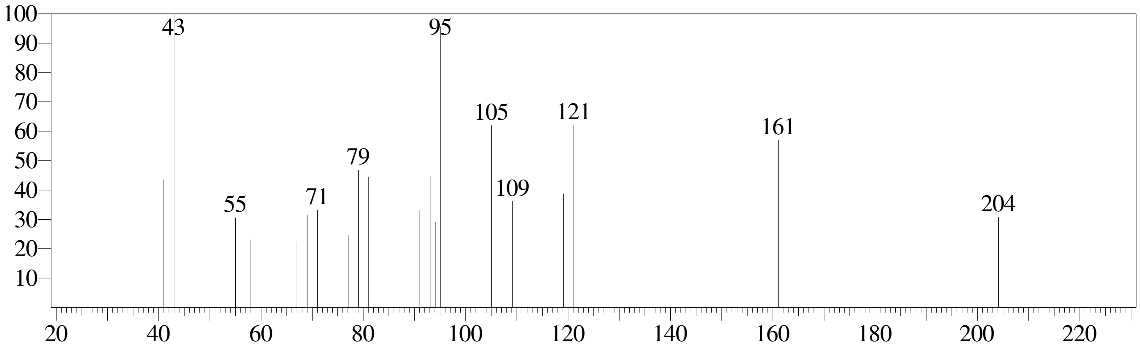

Hit#:5 Entry:28754 Library:NIST23s.lib
SI:81 Formula:C15H26O CAS:19435-97-3 MolWeight:222 RetIndex:1637
CompName:1-Naphthalenol, 1,2,3,4,4a,7,8,8a-octahydro-1,6-dimethyl-4-(1-methylethyl)-, [1R-(1.alpha.,4.beta.,4a.beta.,8a

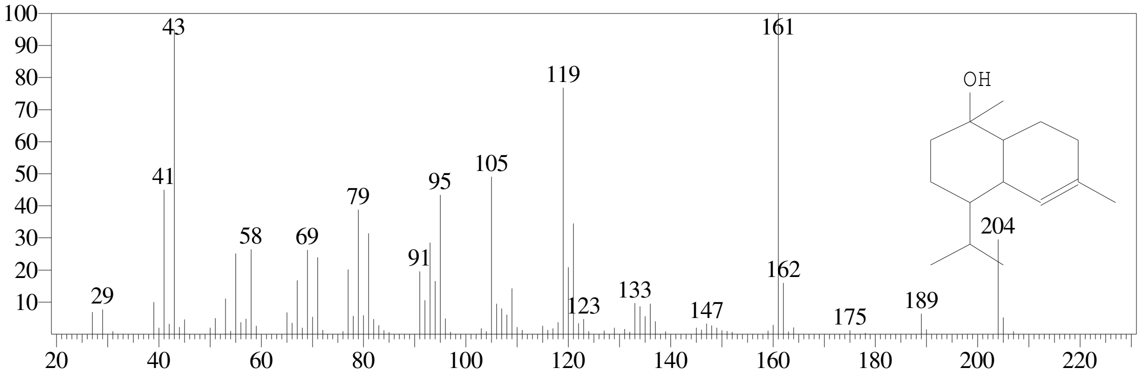

<< Target >>
Line#:29 R.Time:35.367(Scan#:3945) MassPeaks:47
RawMode:Averaged 35.358-35.375(3944-3946) BasePeak:43.00(14550)
BG Mode:None Group 1 - Event 1 Scan

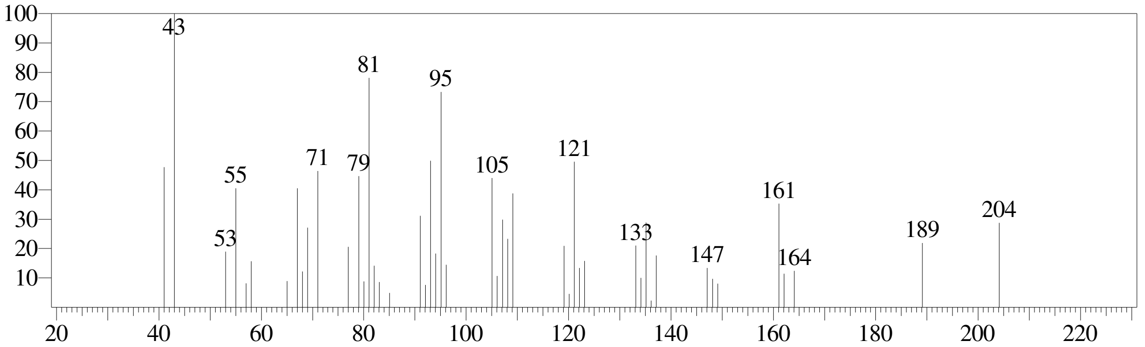

Hit#:1 Entry:28651 Library:NIST23s.lib
SI:89 Formula:C15H26O CAS:5945-72-2 MolWeight:222 RetIndex:1655
CompName:Neointermedeol $$ (1S,4aR,7R,8aR)-1,4a-Dimethyl-7-(prop-1-en-2-yl)decahydronaphthalen-1-ol $$ 1-Napht

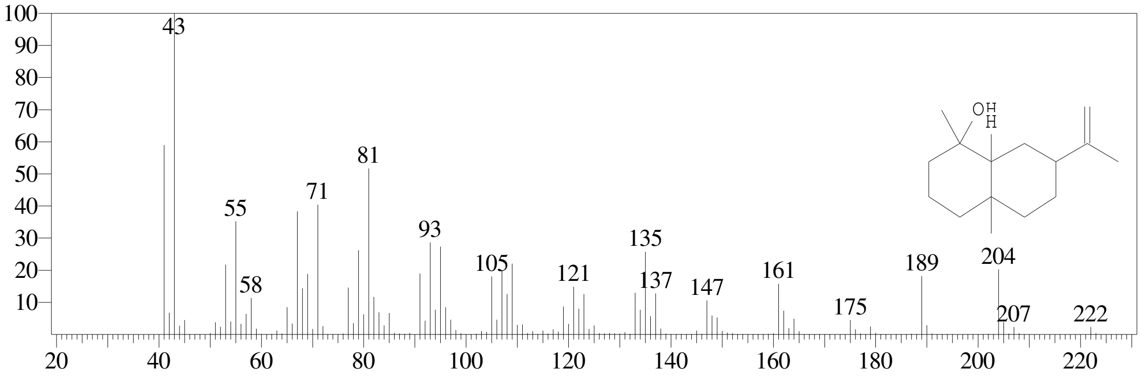

Hit#:2 Entry:83465 Library:NIST23-1.lib
SI:89 Formula:C15H26O CAS:16641-47-7 MolWeight:222 RetIndex:1655
CompName:Selin-11-en-4-alpha-ol

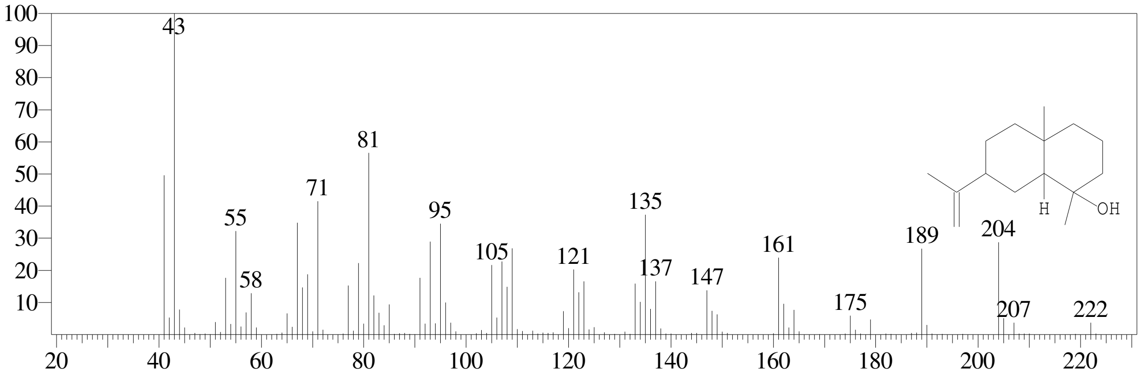

<< Target >>
Line#:29   R.Time:35.367(Scan#:3945) MassPeaks:47
RawMode:Averaged 35.358-35.375(3944-3946) BasePeak:43.00(14550)
BG Mode:None Group 1 - Event 1 Scan

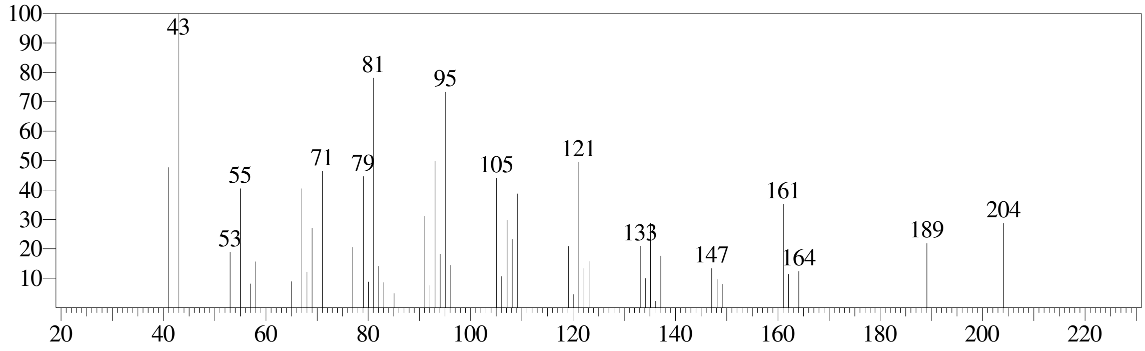

Hit#:3 Entry:83524 Library:NIST23-1.lib
SI:89 Formula:C15H26O CAS:5945-72-2 MolWeight:222 RetIndex:1655
CompName:Neointermedeol $$ (1S,4aR,7R,8aR)-1,4a-Dimethyl-7-(prop-1-en-2-yl)decahydronaphthalen-1-ol $$ 1-Napht

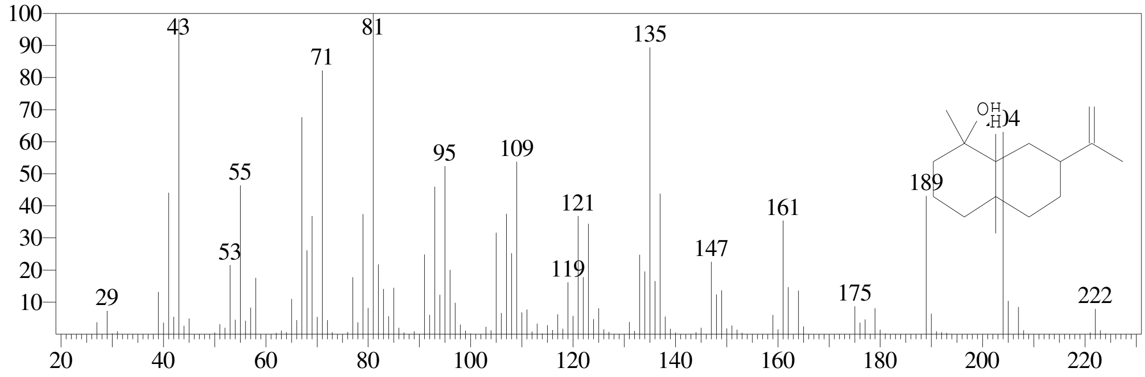

Hit#:4 Entry:28650 Library:NIST23s.lib
SI:89 Formula:C15H26O CAS:21698-41-9 MolWeight:222 RetIndex:1641
CompName:1,4-Dimethyl-7-(prop-1-en-2-yl)decahydroazulen-4-ol $$ Pogostole $$ trans-Guai-11-en-10-ol $$

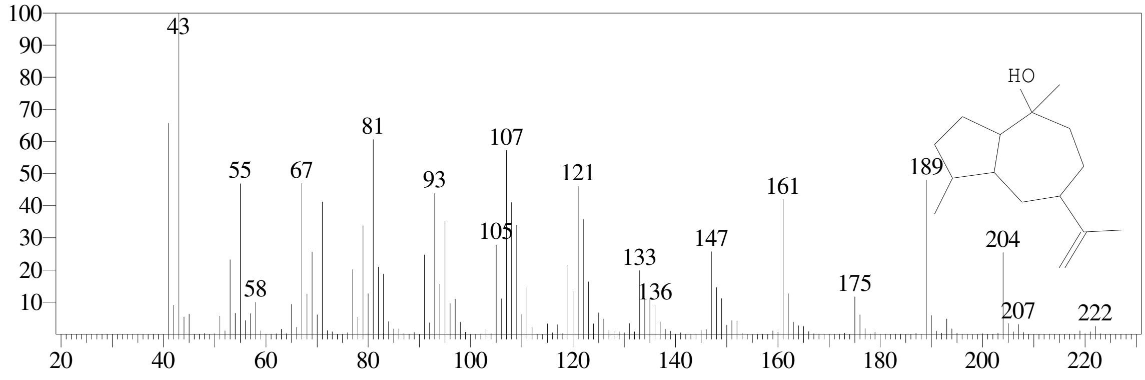

<< Target >>
Line#:29 R.Time:35.367(Scan#:3945) MassPeaks:47
RawMode:Averaged 35.358-35.375(3944-3946) BasePeak:43.00(14550)
BG Mode:None Group 1 - Event 1 Scan

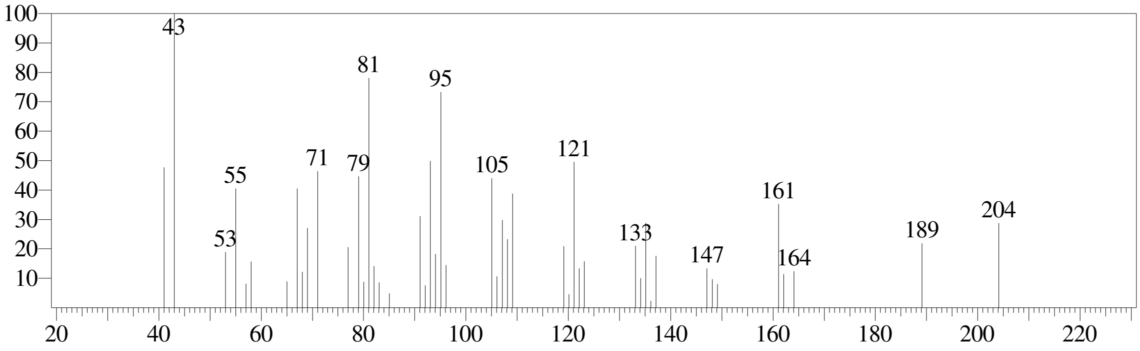

Hit#:5 Entry:28752 Library:NIST23s.lib
SI:89 Formula:C15H26O CAS:5937-11-1 MolWeight:222 RetIndex:1637
CompName:.tau.-Cadinol $$ 4-Isopropyl-1,6-dimethyl-1,2,3,4,4a,7,8,8a-octahydro-1-naphthalenol-, (1S-(1alpha,4alpha,4a

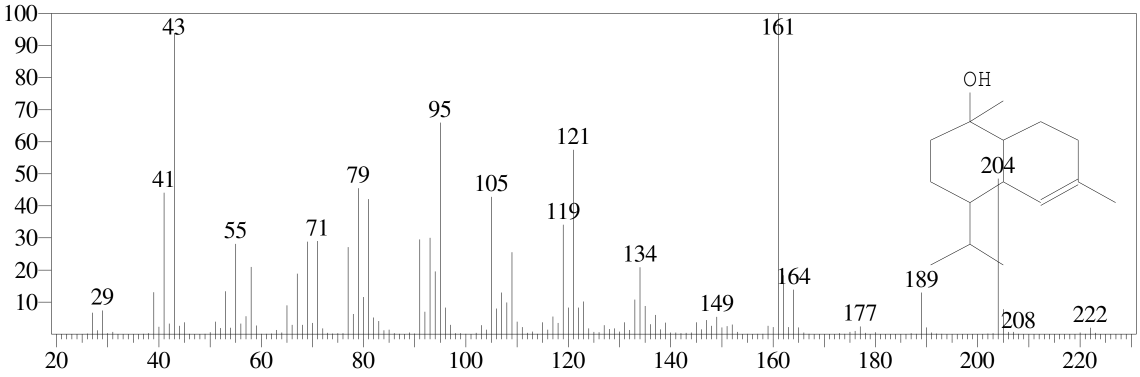

<< Target >>
Line#:30 R.Time:36.400(Scan#:4069) MassPeaks:31
RawMode:Averaged 36.392-36.408(4068-4070) BasePeak:161.10(9639)
BG Mode:None Group 1 - Event 1 Scan

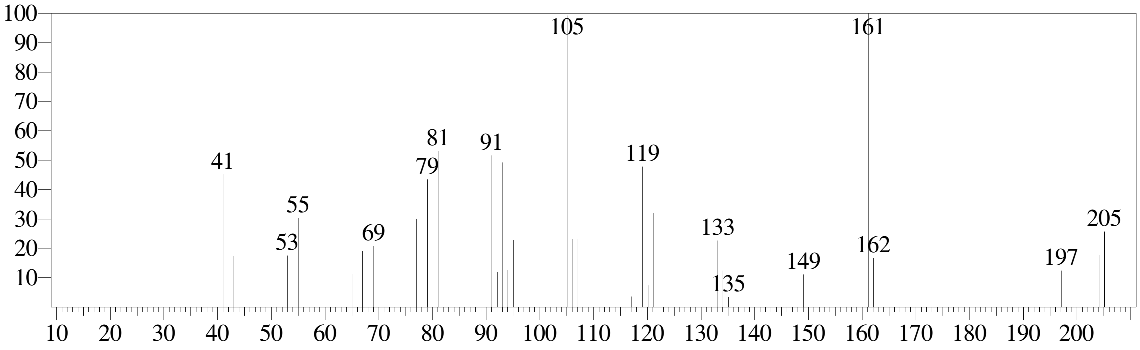

Hit#:1 Entry:25086 Library:NIST23s.lib
SI:86 Formula:C15H24 CAS:30021-74-0 MolWeight:204 RetIndex:1483
CompName:.gamma.-Muurolene $$ Naphthalene, 1,2,3,4,4a,5,6,8a-octahydro-7-methyl-4-methylene-1-(1-methylethyl)-, (

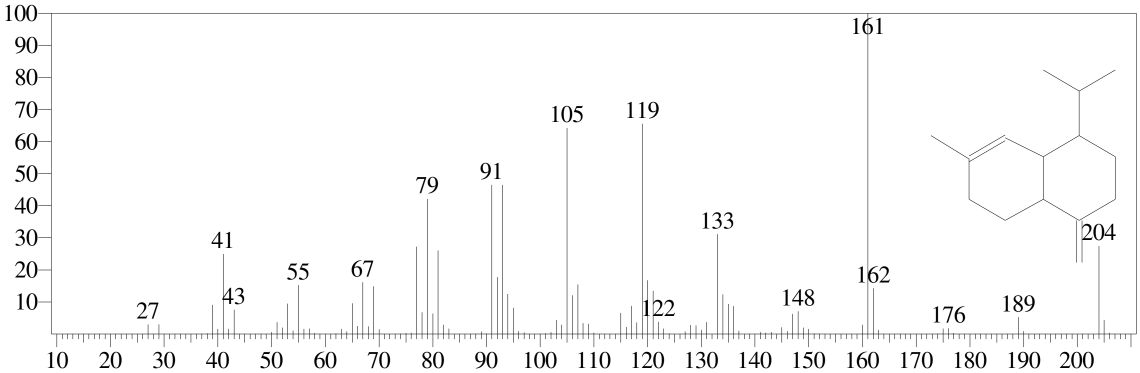

Hit#:2 Entry:25058 Library:NIST23s.lib
SI:85 Formula:C15H24 CAS:30021-74-0 MolWeight:204 RetIndex:1483
CompName:.gamma.-Muurolene $$ Naphthalene, 1,2,3,4,4a,5,6,8a-octahydro-7-methyl-4-methylene-1-(1-methylethyl)-, (

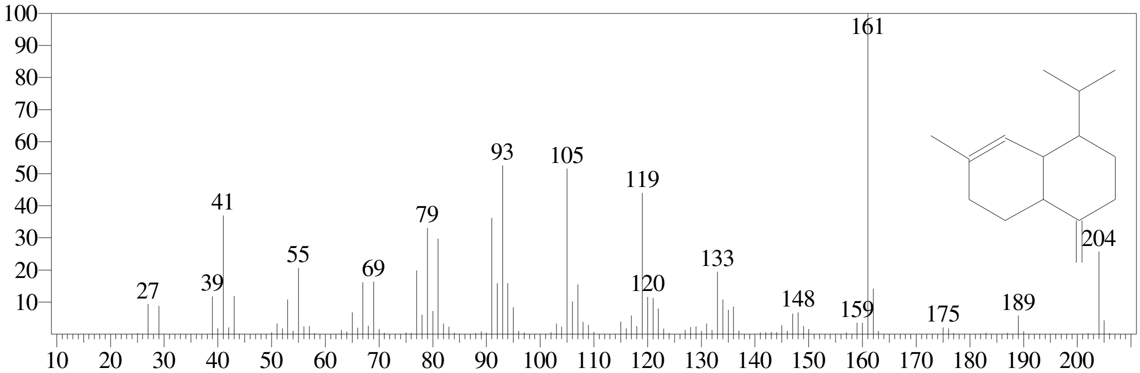

<< Target >>
Line#:30 R.Time:36.400(Scan#:4069) MassPeaks:31
RawMode:Averaged 36.392-36.408(4068-4070) BasePeak:161.10(9639)
BG Mode:None Group 1 - Event 1 Scan

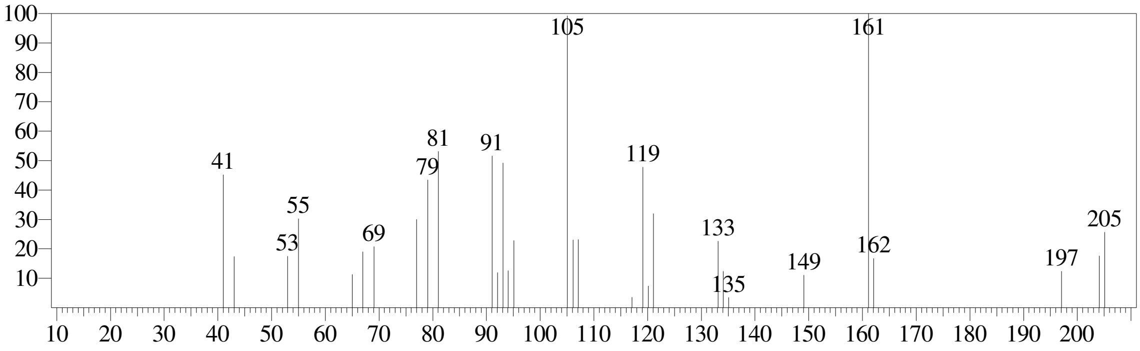

Hit#:3 Entry:25047 Library:NIST23s.lib
SI:85 Formula:C15H24 CAS:30021-74-0 MolWeight:204 RetIndex:1483
CompName:.gamma.-Muurolene $$ Naphthalene, 1,2,3,4,4a,5,6,8a-octahydro-7-methyl-4-methylene-1-(1-methylethyl)-, (

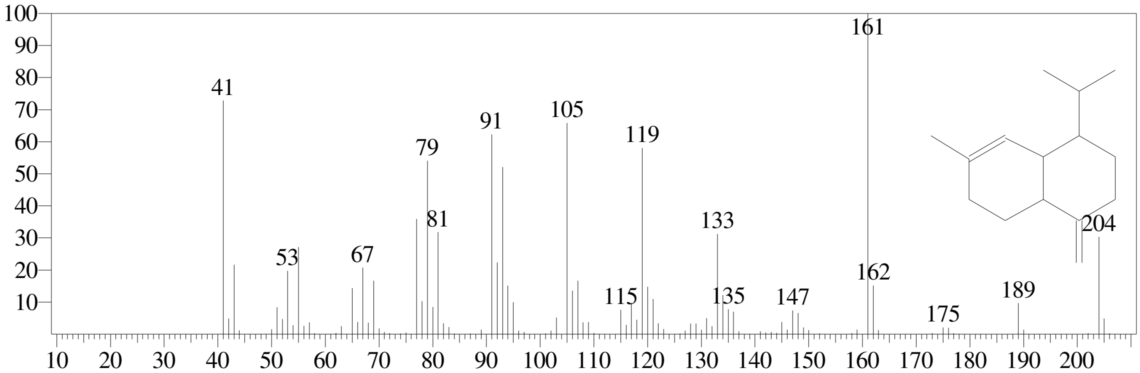

Hit#:4 Entry:62981 Library:NIST23-1.lib
SI:85 Formula:C15H24 CAS:23986-74-5 MolWeight:204 RetIndex:1478
CompName:Germacrene D $$ (S,1Z,6Z)-8-Isopropyl-1-methyl-5-methylenecyclodeca-1,6-diene $$ D-Germacrene $$ 1(1

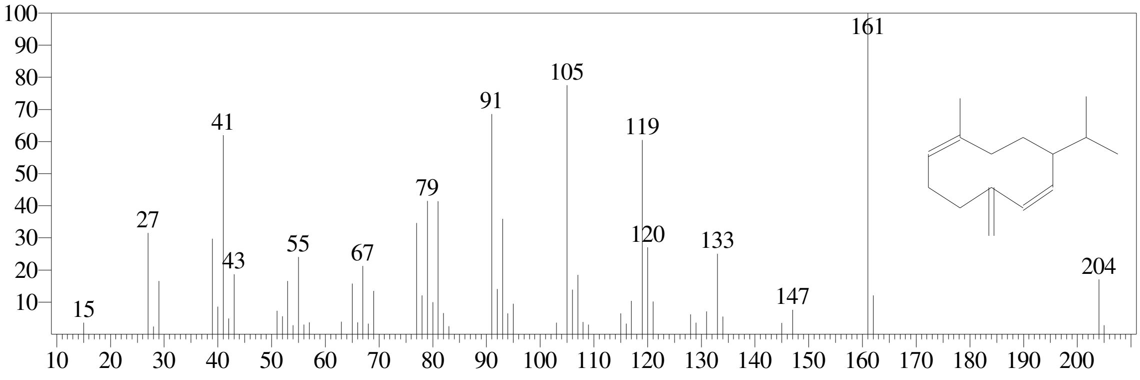

<< Target >>
Line#:30   R.Time:36.400(Scan#:4069) MassPeaks:31
RawMode:Averaged 36.392-36.408(4068-4070) BasePeak:161.10(9639)
BG Mode:None Group 1 - Event 1 Scan

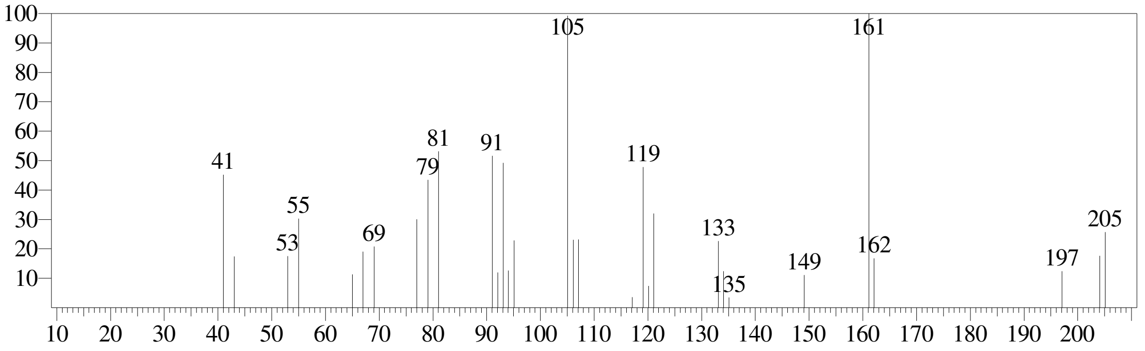

Hit#:5 Entry:25083 Library:NIST23s.lib
SI:85 Formula:C15H24 CAS:95910-36-4 MolWeight:204 RetIndex:1396
CompName:isoledene $$ 1,1,4,7-Tetramethyl-1a,2,3,4,5,6,7,7b-octahydro-1H-cyclopropa[e]azulene # $$ (1aR,4R,7R,7b[illegible]

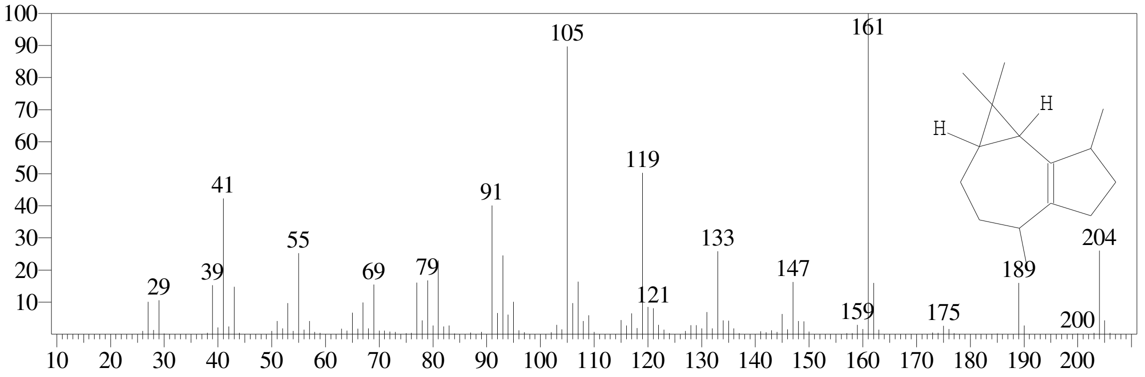

Supplement: Supplementary file 1 [file plants-15-01406-s001.zip › EMbb.pdf]
