# Supplementary material for: Unveiling Chemical Profile and Insecticidal Potential of Essential Oils from Leaves of Seven Eugenia L. Species (Myrtaceae)
Source: Plants (Basel). 2026 May 5;15(9):1406. doi: 10.3390/plants15091406 (PMC13165059; doi:10.3390/plants15091406)

LabCrom
Laboratório de Cromatografia

**UNIVERSIDADE FERAL RURAL DO RIO DE JANEIRO**
**INSTITUTO DE QUÍMICA**

CENTRAL ANALÍTICA MULTIUSUÁRIO
INSTITUTO DE QUÍMICA · UFRRJ

CGMS

Analyzed by: Cristiane Cardoso
Analyzed: 17/6/2025
Solicitante:Douglas
Sample Name: EN
Injection Volume: 1,0 uL      Solvente: Diclorometano
Data File: C:\GCMSsolution\Data\Project1\Douglas\2025\MLLENA\170625\EN.qgd
Method File: C:\GCMSsolution\Data\Project1\Douglas\Essencial Adams-Inj.qgm
EQUIPAMENTO: Modelo:GCMS-QP2010 Plus ( Shimadzu)
Coluna: VF-5m (30X0.25X0.25)

Chromatogram EN C:\GCMSsolution\Data\Project1\Douglas\2025\MLLENA\170625\EN.qgd

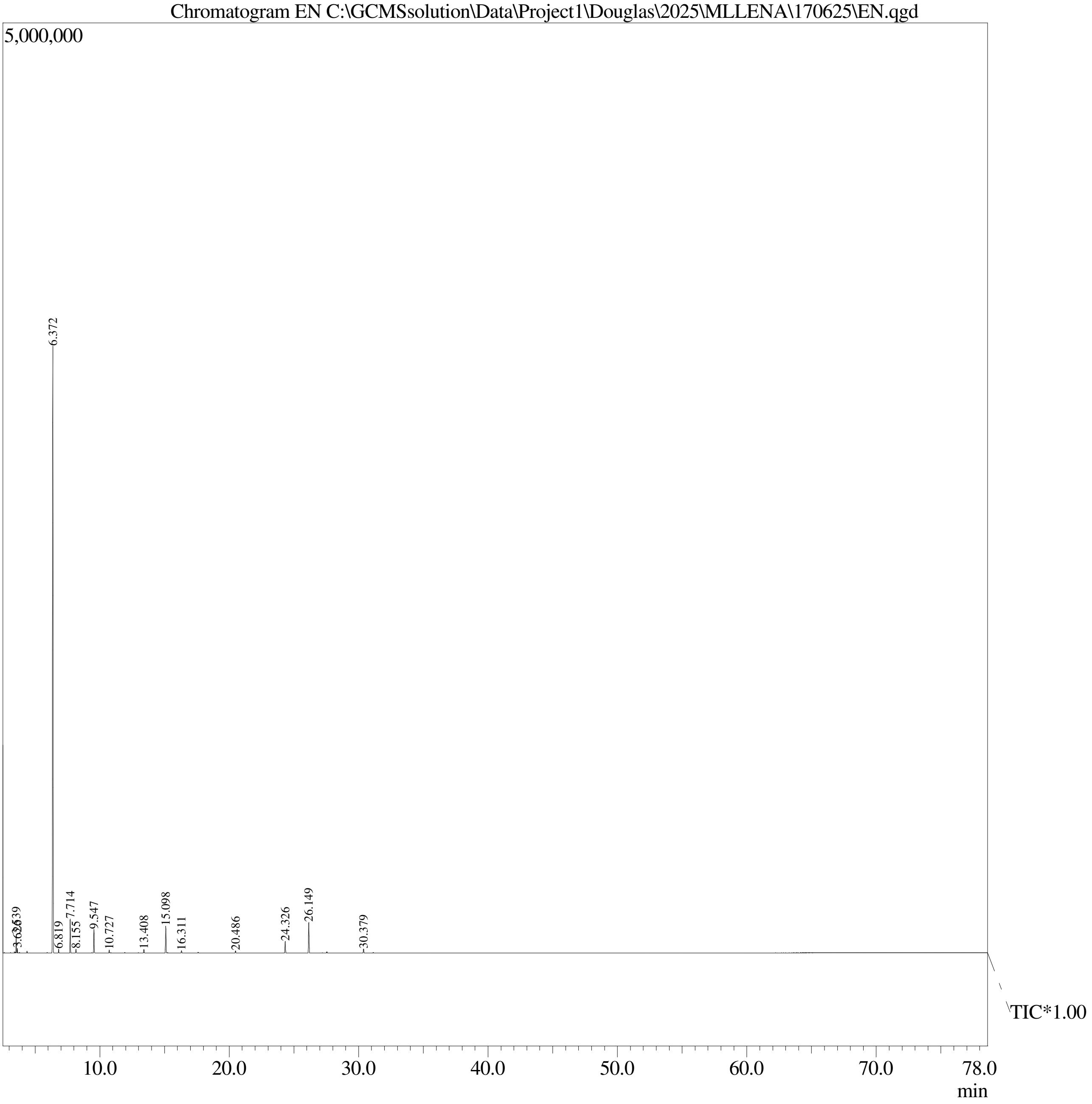

Library

<< Target >>
Line#:1   R.Time:3.542(Scan#:126)  MassPeaks:11
RawMode:Averaged 3.533-3.550(125-127) BasePeak:45.00(52560)
BG Mode:None  Group 1 - Event 1  Scan

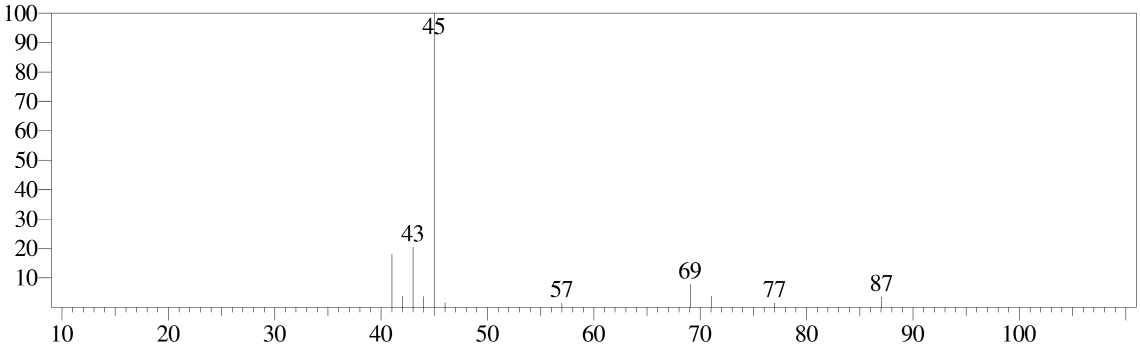


Hit#:1  Entry:2533  Library:NIST23-1.lib
SI:93  Formula:C6H14O  CAS:52019-78-0  MolWeight:102  RetIndex:791
CompName:2-Hexanol, (S)- $$ (S)-(+)-2-Hexanol $$ 2-Hexanol  # $$

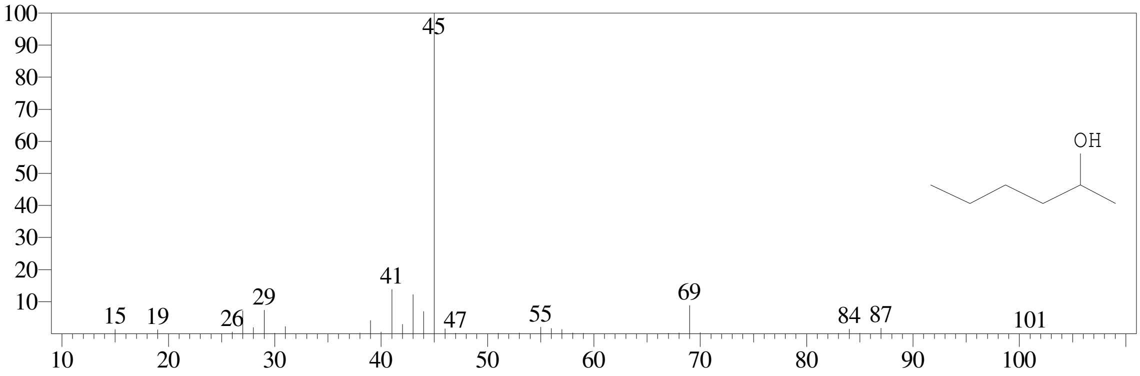


Hit#:2  Entry:2534  Library:NIST23-1.lib
SI:93  Formula:C6H14O  CAS:26549-24-6  MolWeight:102  RetIndex:791
CompName:2-Hexanol, (R)- $$ (R)-(-)-2-Hexanol $$ 2-Hexanol  # $$

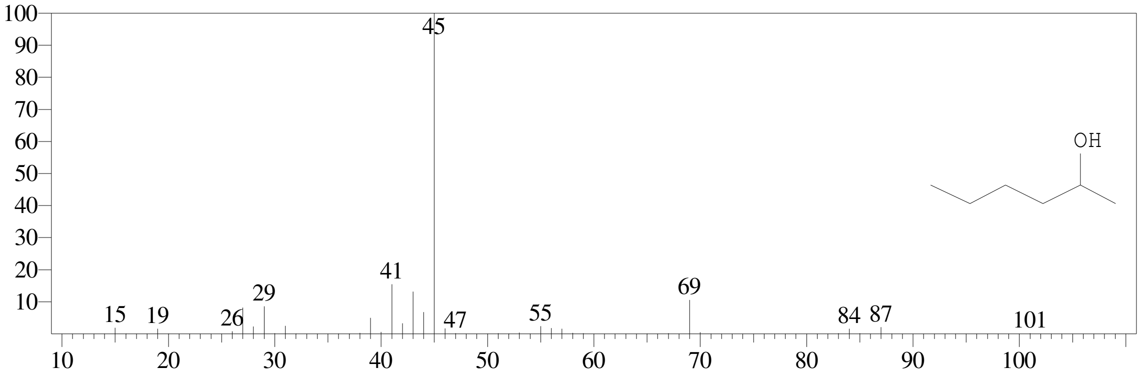

<< Target >>
Line#:1   R.Time:3.542(Scan#:126)  MassPeaks:11
RawMode:Averaged 3.533-3.550(125-127) BasePeak:45.00(52560)
BG Mode:None  Group 1 - Event 1  Scan

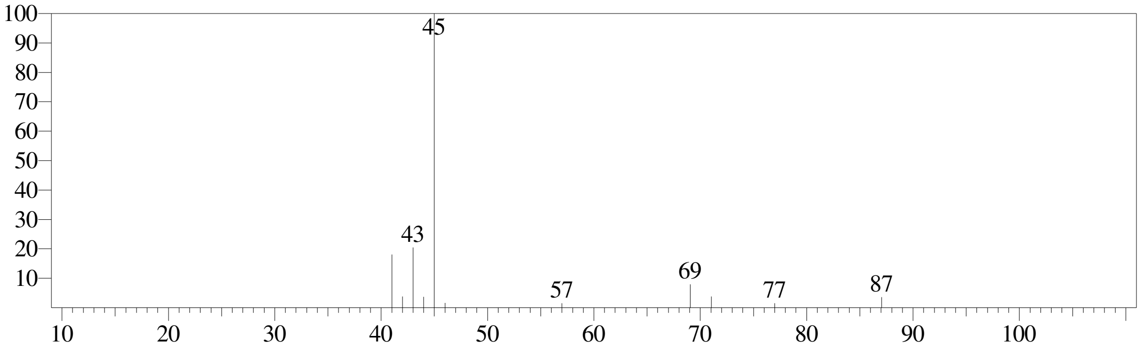


Hit#:3  Entry:2597  Library:NIST23s.lib
SI:93  Formula:C6H14O  CAS:108-11-2  MolWeight:102  RetIndex:752
CompName:2-Pentanol, 4-methyl- $$ Isobutylmethylcarbinol $$ Isobutylmethylmethanol $$ Methylisobutylcarbinol $$ M

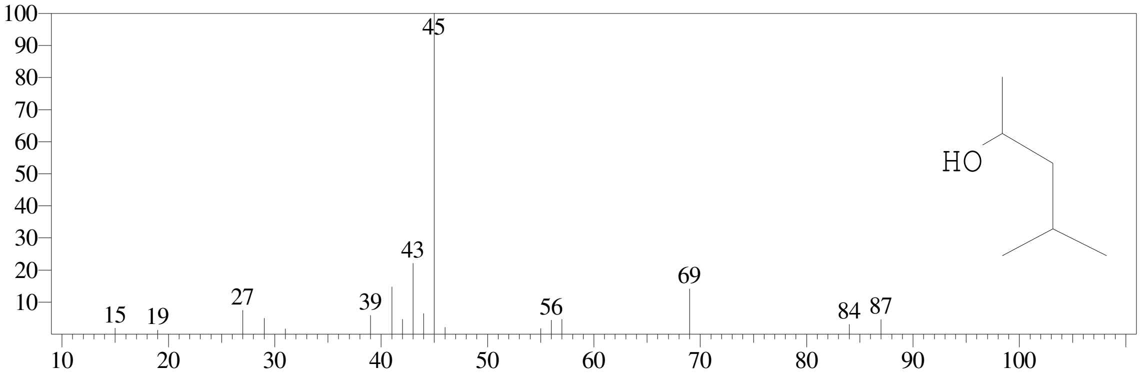


Hit#:4  Entry:1082  Library:NIST23s.lib
SI:92  Formula:C5H10O  CAS:625-31-0  MolWeight:86  RetIndex:658
CompName:4-Penten-2-ol $$ 1-Penten-4-ol $$ 4-Hydroxypent-1-ene $$ CH2=CHCH2CH(OH)CH3 $$

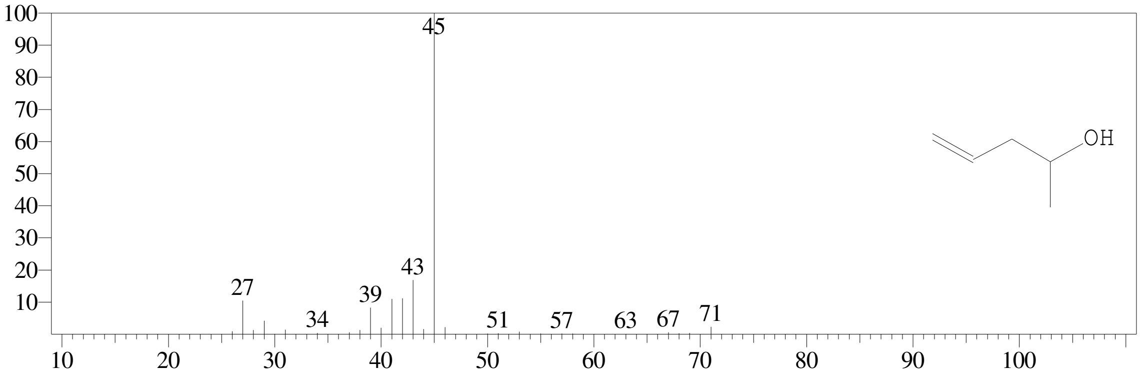

<< Target >>
Line#:1 R.Time:3.542(Scan#:126) MassPeaks:11
RawMode:Averaged 3.533-3.550(125-127) BasePeak:45.00(52560)
BG Mode:None Group 1 - Event 1 Scan

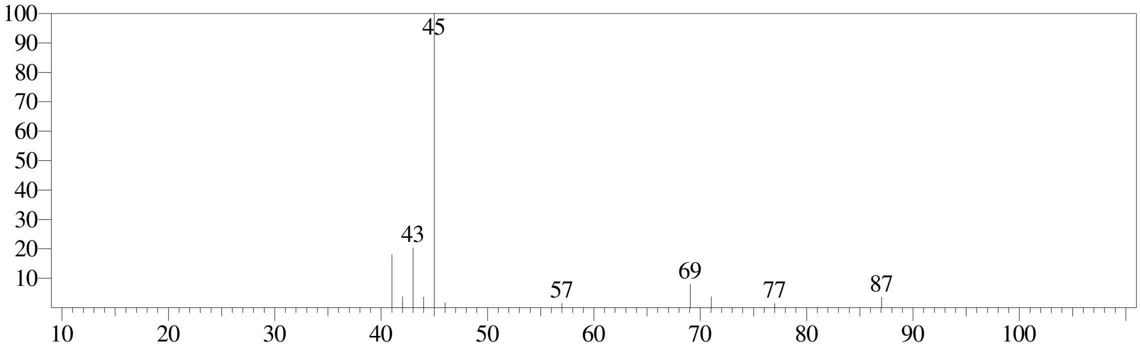


Hit#:5 Entry:2596 Library:NIST23s.lib
SI:92 Formula:C6H14O CAS:626-93-7 MolWeight:102 RetIndex:791
CompName:2-Hexanol $$ n-C4H9CH(OH)CH3 $$ n-Butylmethylcarbinol $$ Hexanol-(2) $$ sec-Hexyl alcohol $$ n-He

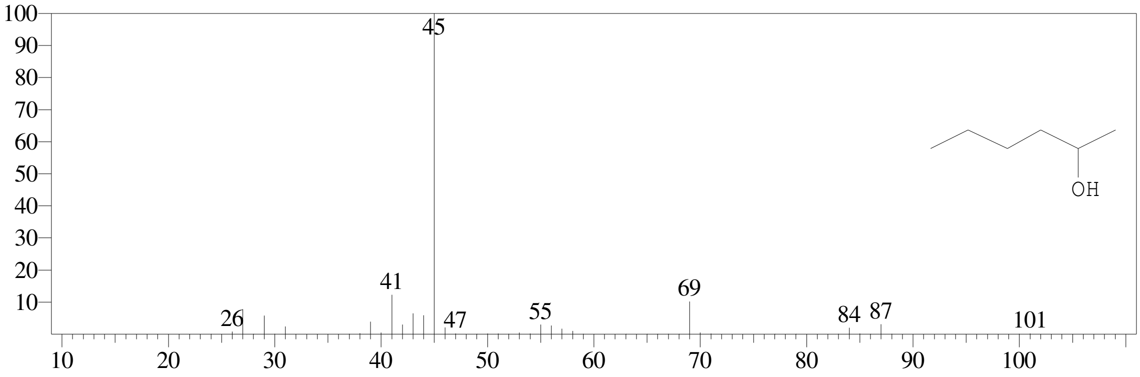

<< Target >>
Line#:2   R.Time:3.625(Scan#:136)  MassPeaks:5
RawMode:Averaged 3.617-3.633(135-137) BasePeak:59.00(12701)
BG Mode:None  Group 1 - Event 1  Scan

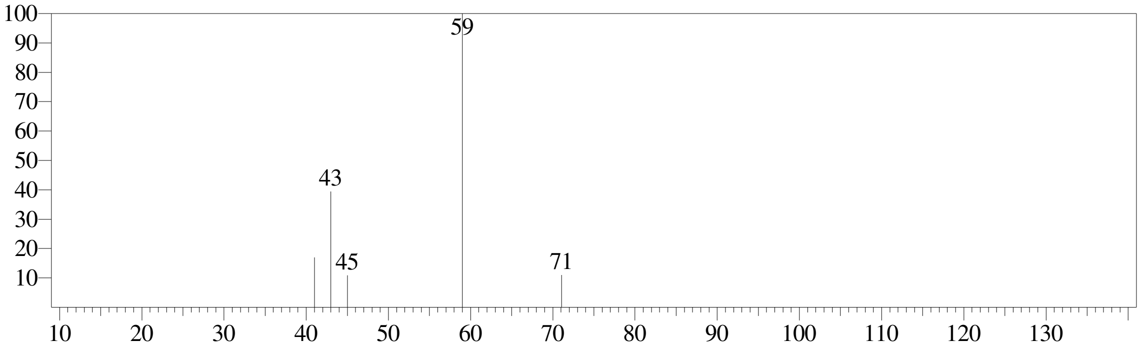


Hit#:1  Entry:2726  Library:NIST23-1.lib
SI:91  Formula:C4H8O3  CAS:594-61-6  MolWeight:104  RetIndex:932
CompName:Propanoic acid, 2-hydroxy-2-methyl- $$ 2-Hydroxyisobutyric acid $$ Lactic acid, 2-methyl- $$ .alpha.-Hydro

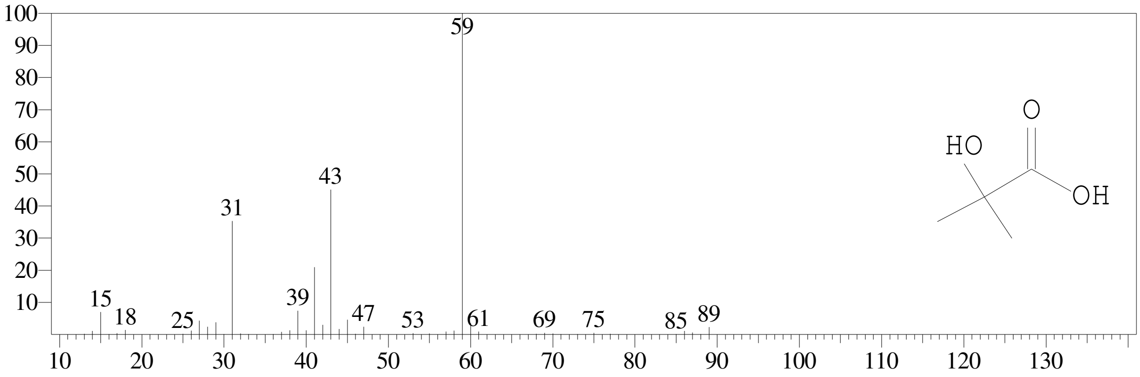


Hit#:2  Entry:2466  Library:NIST23-1.lib
SI:90  Formula:C5H10O2  CAS:115-22-0  MolWeight:102  RetIndex:737
CompName:3-Hydroxy-3-methyl-2-butanone $$ 2-Butanone, 3-hydroxy-3-methyl- $$ Dimethylacetylcarbinol $$ 3-Hydro

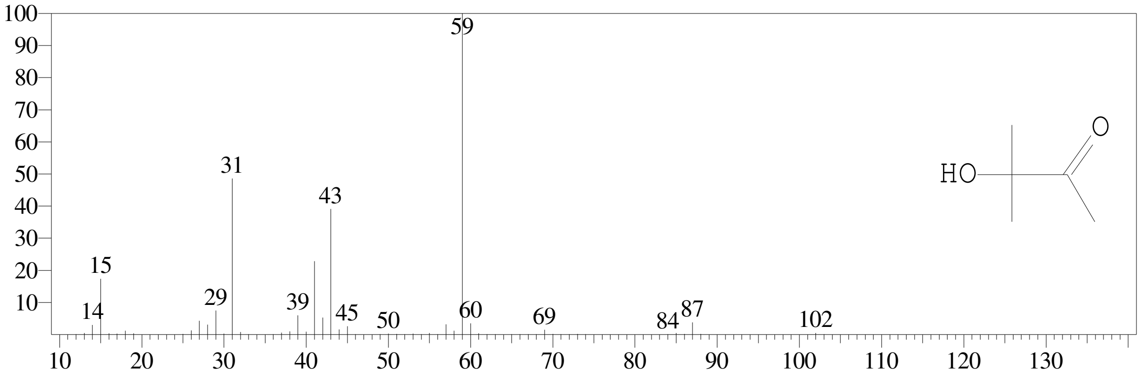

<< Target >>
Line#:2   R.Time:3.625(Scan#:136)  MassPeaks:5
RawMode:Averaged 3.617-3.633(135-137) BasePeak:59.00(12701)
BG Mode:None  Group 1 - Event 1  Scan

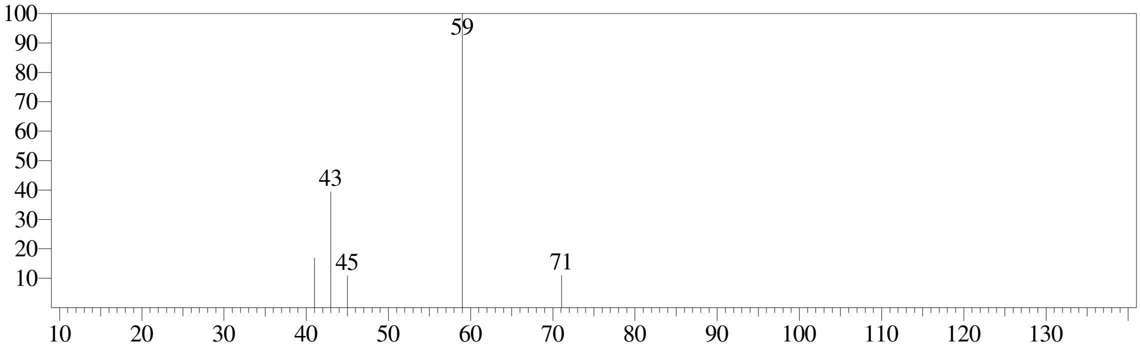


Hit#:3  Entry:2775  Library:NIST23-1.lib
SI:89  Formula:C5H12O2  CAS:5396-58-7  MolWeight:104  RetIndex:812
CompName:2-Methyl-2,3-butanediol $$ 2-Methylbutane-2,3-diol $$ 2,3-Dihydroxy-2-methylbutane $$

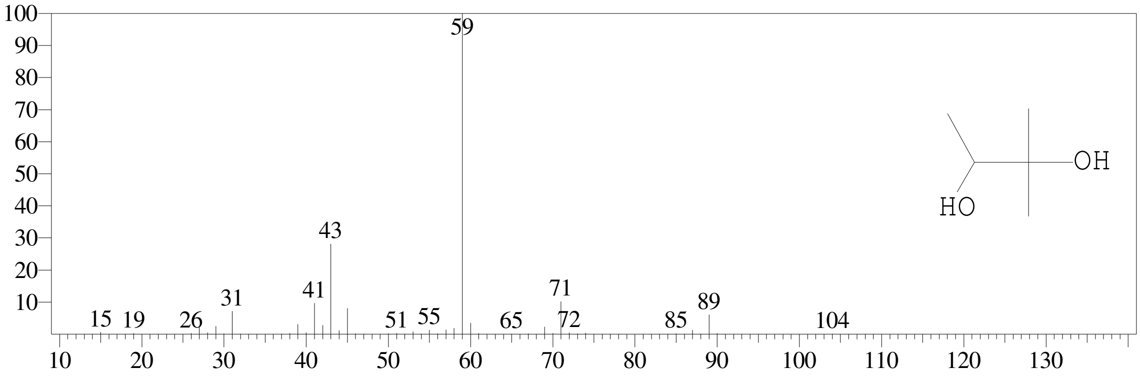


Hit#:4  Entry:9783  Library:NIST23-1.lib
SI:89  Formula:C6H12O3  CAS:70657-70-4  MolWeight:132  RetIndex:880
CompName:2-Methoxypropyl acetate $$ 1-Propanol, 2-methoxy-, 1-acetate $$ 1-Propanol, 2-methoxy-, acetate $$ 2-Meth

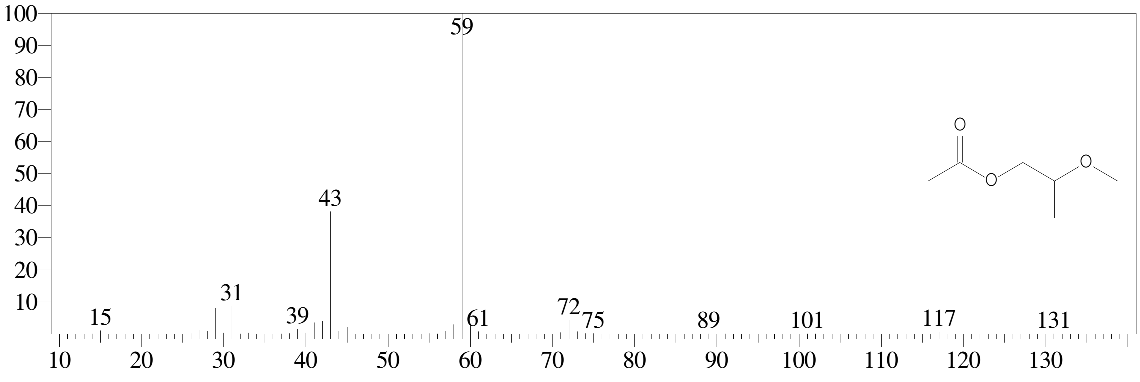

<< Target >>
Line#:2   R.Time:3.625(Scan#:136)  MassPeaks:5
RawMode:Averaged 3.617-3.633(135-137) BasePeak:59.00(12701)
BG Mode:None  Group 1 - Event 1  Scan

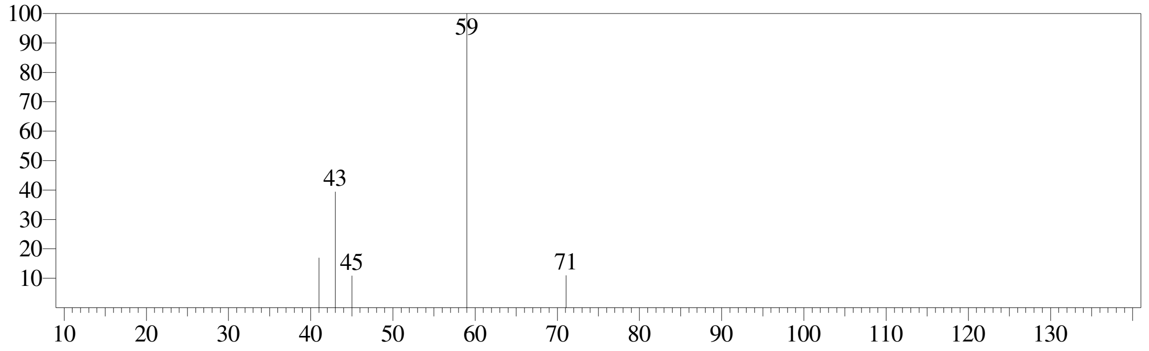


Hit#:5  Entry:583  Library:NIST23s.lib
SI:89  Formula:C4H10O  CAS:598-53-8  MolWeight:74  RetIndex:471
CompName:Propane, 2-methoxy- $$ Ether, isopropyl methyl $$ Isopropyl methyl ether $$ Isopryl $$ Methyl isopropyl et

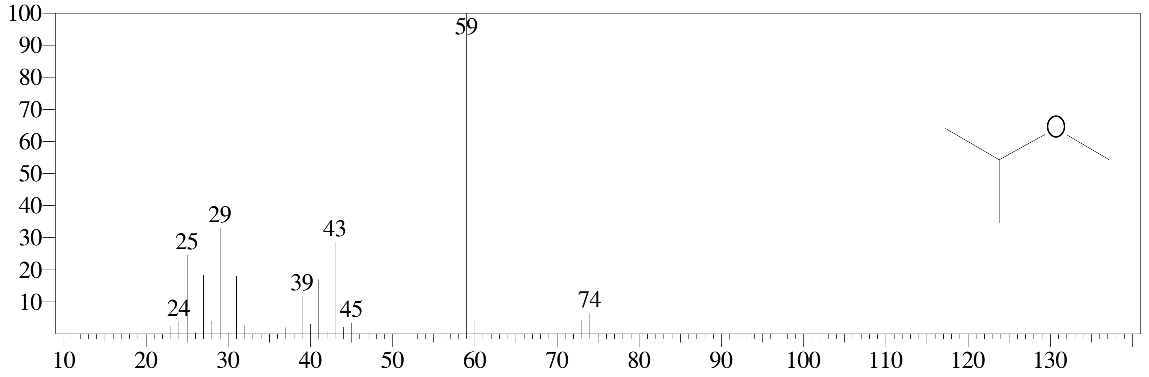

<< Target >>
Line#:3   R.Time:6.375(Scan#:466)  MassPeaks:60
RawMode:Averaged 6.367-6.383(465-467) BasePeak:93.10(747919)
BG Mode:None  Group 1 - Event 1  Scan

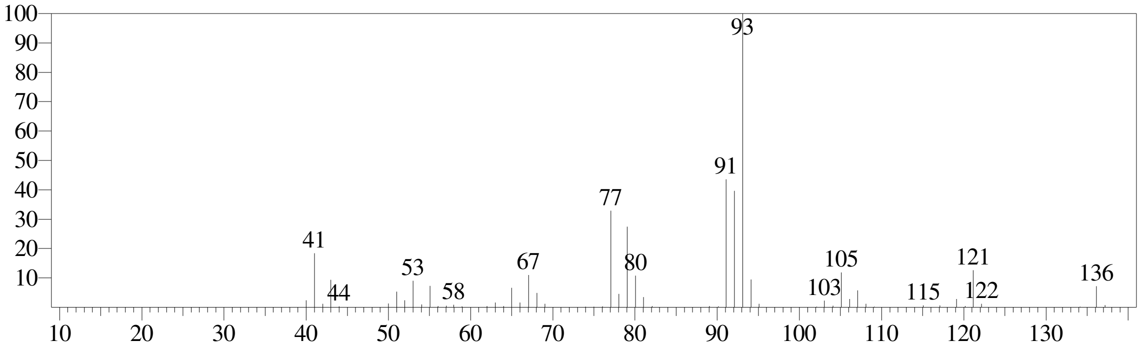


Hit#:1  Entry:8445  Library:NIST23s.lib
SI:97  Formula:C10H16  CAS:80-56-8  MolWeight:136  RetIndex:947
CompName:.alpha.-Pinene $$ Bicyclo[3.1.1]hept-2-ene, 2,6,6-trimethyl- $$ 2-Pinene $$ 2,6,6-Trimethylbicyclo[3.1.1]hep

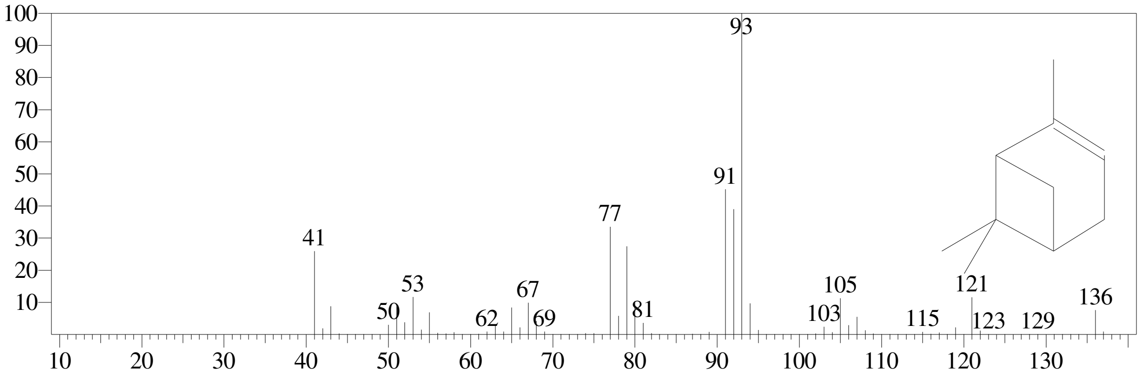


Hit#:2  Entry:8451  Library:NIST23s.lib
SI:97  Formula:C10H16  CAS:80-56-8  MolWeight:136  RetIndex:947
CompName:.alpha.-Pinene $$ Bicyclo[3.1.1]hept-2-ene, 2,6,6-trimethyl- $$ 2-Pinene $$ 2,6,6-Trimethylbicyclo[3.1.1]hep

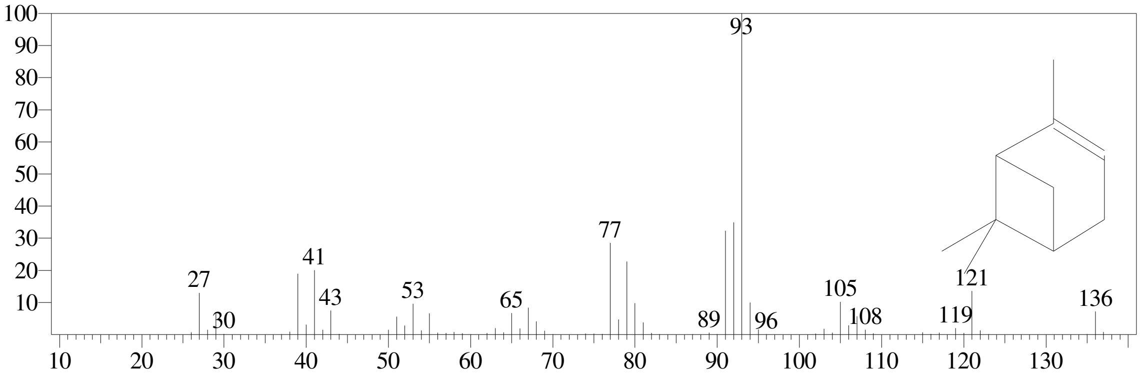

<< Target >>
Line#:3 R.Time:6.375(Scan#:466) MassPeaks:60
RawMode:Averaged 6.367-6.383(465-467) BasePeak:93.10(747919)
BG Mode:None Group 1 - Event 1 Scan

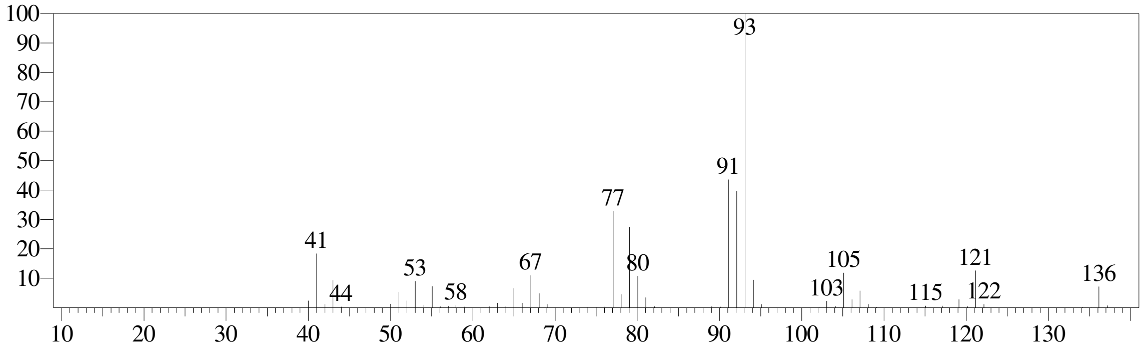


Hit#:3 Entry:11410 Library:NIST23-1.lib
SI:96 Formula:C10H16 CAS:80-56-8 MolWeight:136 RetIndex:947
CompName:.alpha.-Pinene $$ Bicyclo[3.1.1]hept-2-ene, 2,6,6-trimethyl- $$ 2-Pinene $$ 2,6,6-Trimethylbicyclo[3.1.1]hep

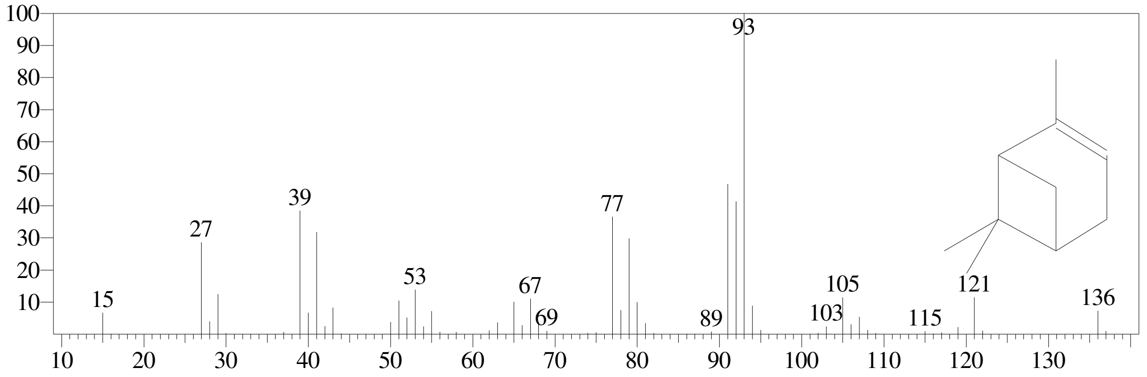


Hit#:4 Entry:8443 Library:NIST23s.lib
SI:96 Formula:C10H16 CAS:3779-61-1 MolWeight:136 RetIndex:1047
CompName:trans-.beta.-Ocimene $$ 1,3,6-Octatriene, 3,7-dimethyl-, (E)- $$ .beta.-trans-Ocimene $$ trans-3,7-Dimethyl-

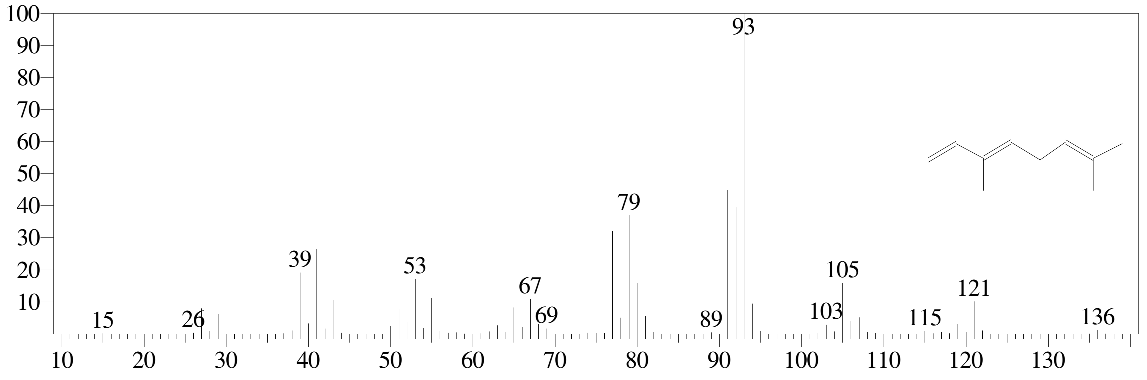

<< Target >>
Line#:3   R.Time:6.375(Scan#:466)  MassPeaks:60
RawMode:Averaged 6.367-6.383(465-467) BasePeak:93.10(747919)
BG Mode:None  Group 1 - Event 1  Scan

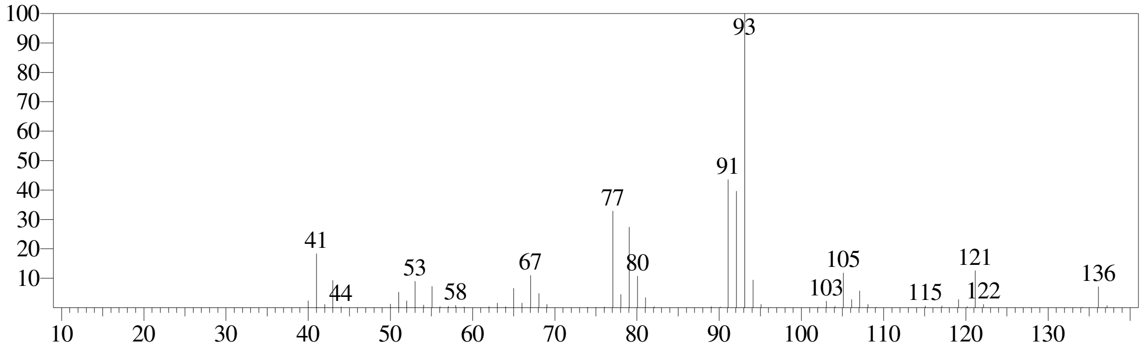


Hit#:5  Entry:8450  Library:NIST23s.lib
SI:95  Formula:C10H16  CAS:80-56-8  MolWeight:136  RetIndex:947
CompName:.alpha.-Pinene $$ Bicyclo[3.1.1]hept-2-ene, 2,6,6-trimethyl- $$ 2-Pinene $$ 2,6,6-Trimethylbicyclo[3.1.1]hep

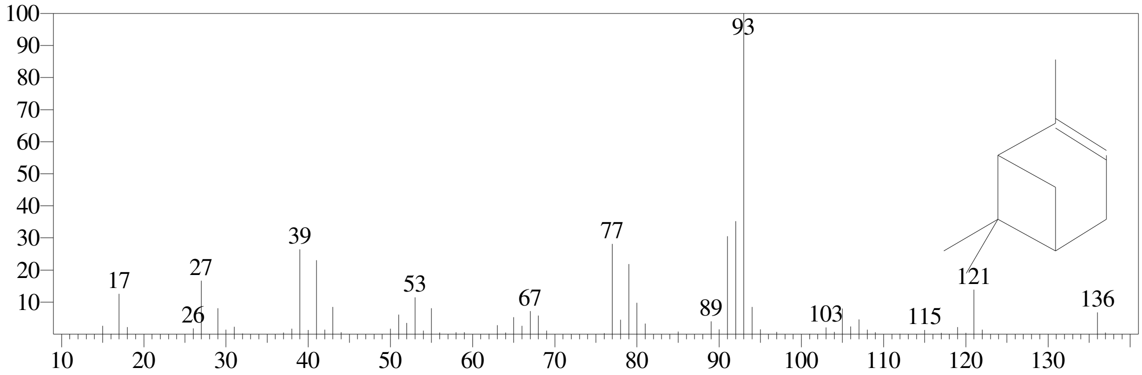

<< Target >>
Line#:4   R.Time:6.817(Scan#:519)  MassPeaks:9
RawMode:Averaged 6.808-6.825(518-520) BasePeak:93.05(4558)
BG Mode:Calc. from Peak  Group 1 - Event 1  Scan

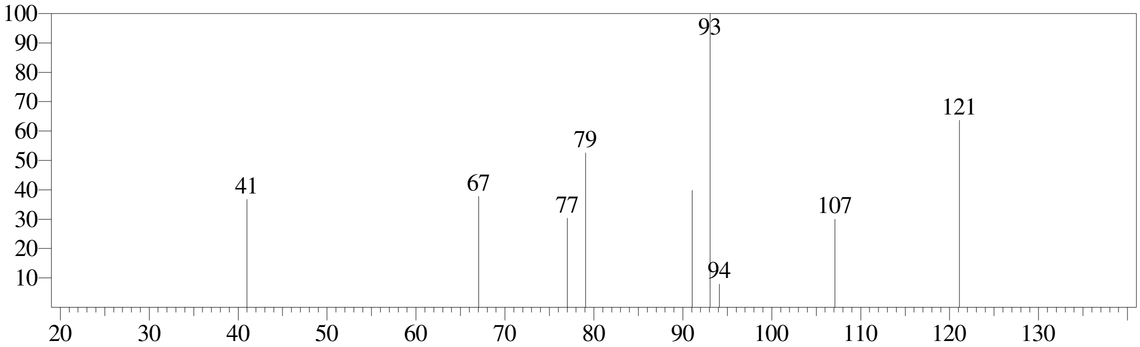


Hit#:1  Entry:11418  Library:NIST23-1.lib
SI:77  Formula:C10H16  CAS:79-92-5  MolWeight:136  RetIndex:969
CompName:Camphene $$ Bicyclo[2.2.1]heptane, 2,2-dimethyl-3-methylene- $$ 2,2-Dimethyl-3-methylenebicyclo[2.2.1]l

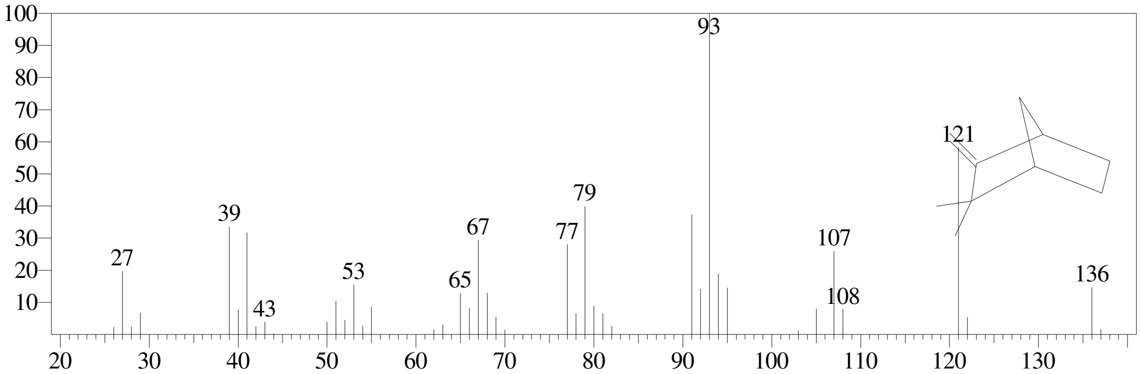


Hit#:2  Entry:8454  Library:NIST23s.lib
SI:76  Formula:C10H16  CAS:79-92-5  MolWeight:136  RetIndex:969
CompName:Camphene $$ Bicyclo[2.2.1]heptane, 2,2-dimethyl-3-methylene- $$ 2,2-Dimethyl-3-methylenebicyclo[2.2.1]l

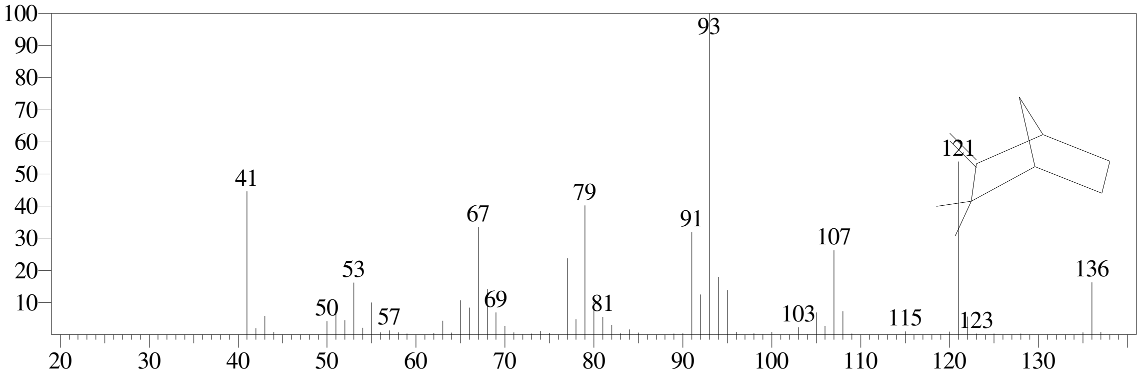

<< Target >>
Line#:4   R.Time:6.817(Scan#:519)  MassPeaks:9
RawMode:Averaged 6.808-6.825(518-520) BasePeak:93.05(4558)
BG Mode:Calc. from Peak  Group 1 - Event 1  Scan

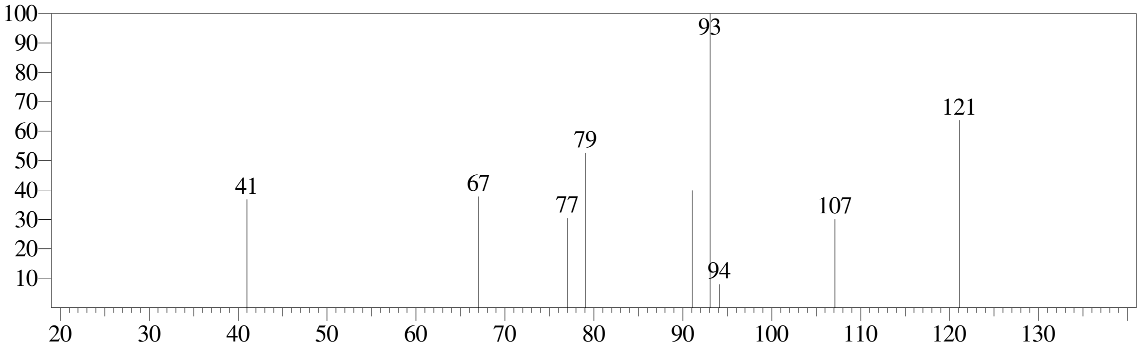


Hit#:3  Entry:8453  Library:NIST23s.lib
SI:76  Formula:C10H16  CAS:79-92-5  MolWeight:136  RetIndex:969
CompName:Camphene $$ Bicyclo[2.2.1]heptane, 2,2-dimethyl-3-methylene- $$ 2,2-Dimethyl-3-methylenebicyclo[2.2.1]

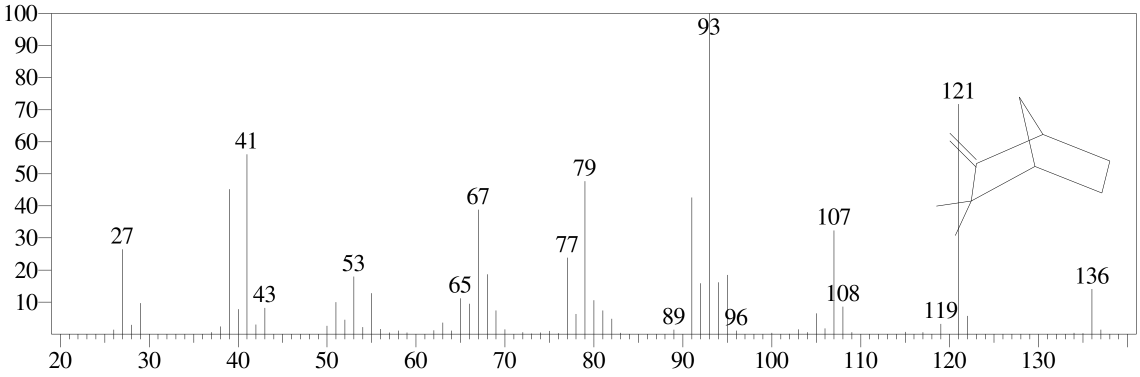


Hit#:4  Entry:8417  Library:NIST23s.lib
SI:76  Formula:C10H16  CAS:471-84-1  MolWeight:136  RetIndex:954
CompName:Bicyclo[2.2.1]heptane, 7,7-dimethyl-2-methylene- $$ Norbornane, 7,7-dimethyl-2-methylene- $$ .alpha.-Fen

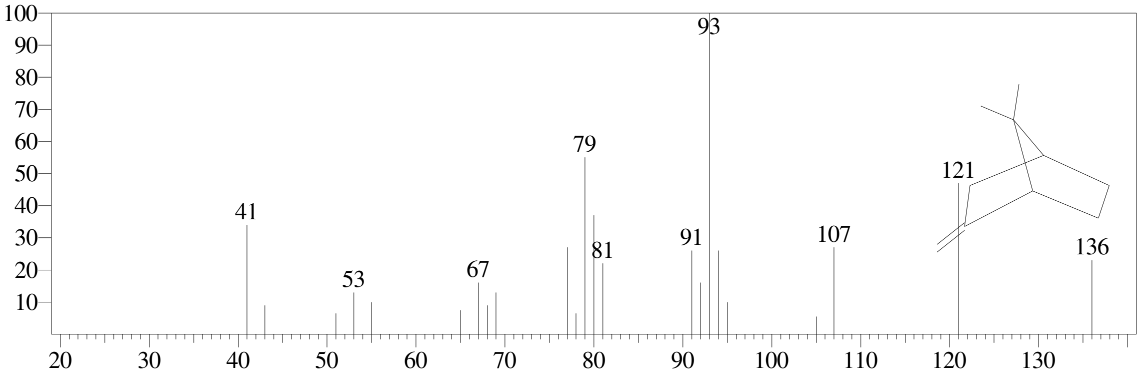

<< Target >>
Line#:4   R.Time:6.817(Scan#:519)  MassPeaks:9
RawMode:Averaged 6.808-6.825(518-520) BasePeak:93.05(4558)
BG Mode:Calc. from Peak  Group 1 - Event 1  Scan

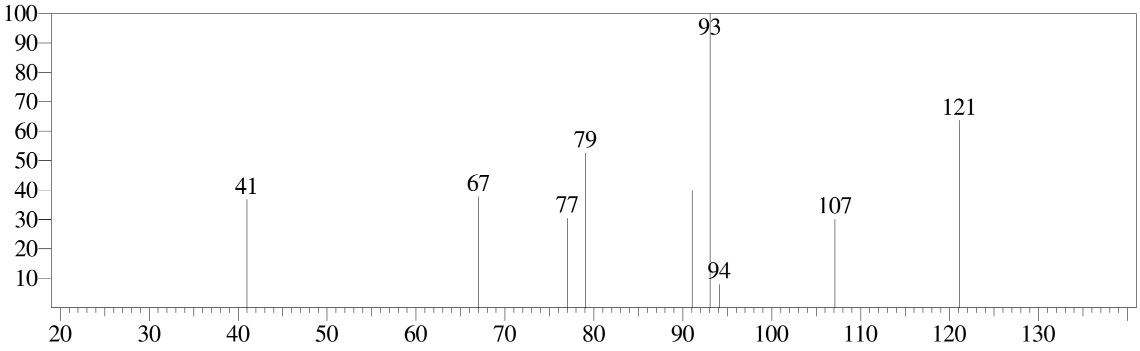


Hit#:5  Entry:11389  Library:NIST23-1.lib
SI:75  Formula:C10H16  CAS:99-84-3  MolWeight:136  RetIndex:1024
CompName:Cyclohexene, 4-methylene-1-(1-methylethyl)- $$ p-Mentha-1(7),3-diene $$ .beta.-Terpinen $$ .beta.-Terpine

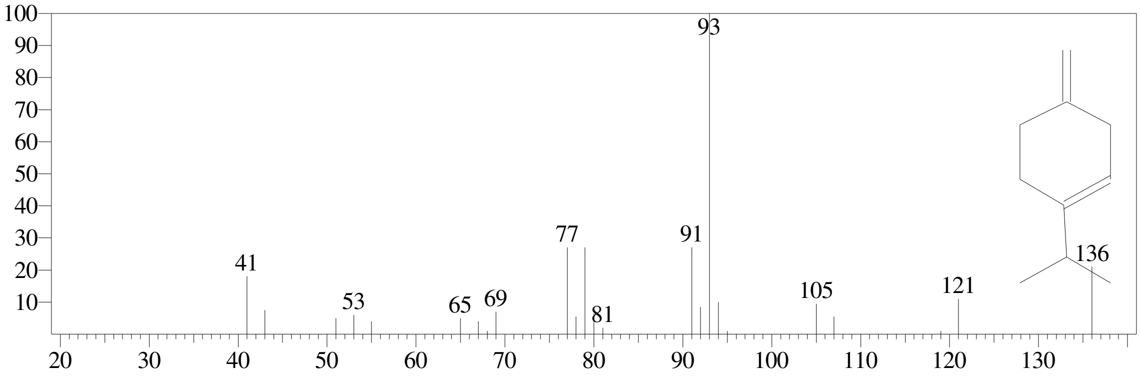

<< Target >>
Line#:5 R.Time:7.717(Scan#:627) MassPeaks:25
RawMode:Averaged 7.708-7.725(626-628) BasePeak:93.05(41926)
BG Mode:None Group 1 - Event 1 Scan

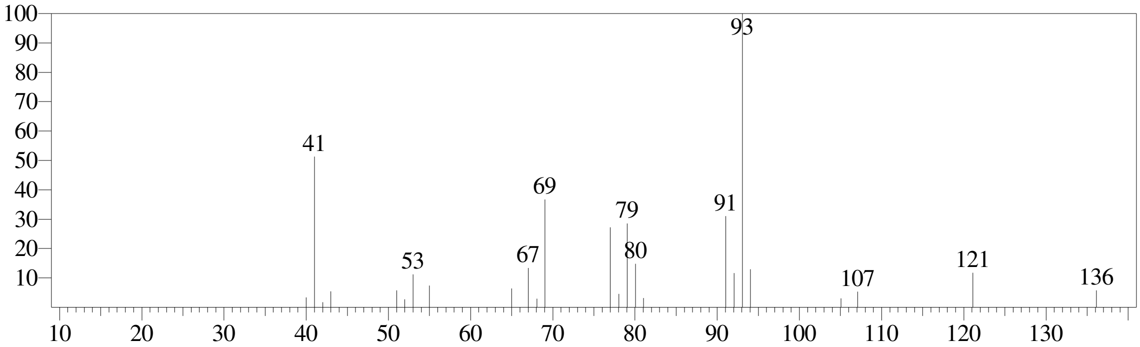


Hit#:1 Entry:8389 Library:NIST23s.lib
SI:95 Formula:C10H16 CAS:18172-67-3 MolWeight:136 RetIndex:978
CompName:Bicyclo[3.1.1]heptane, 6,6-dimethyl-2-methylene-, (1S)- $$ 2(10)-Pinene, (1S,5S)-(-)- $$ (-)-.beta.-Pinene $$

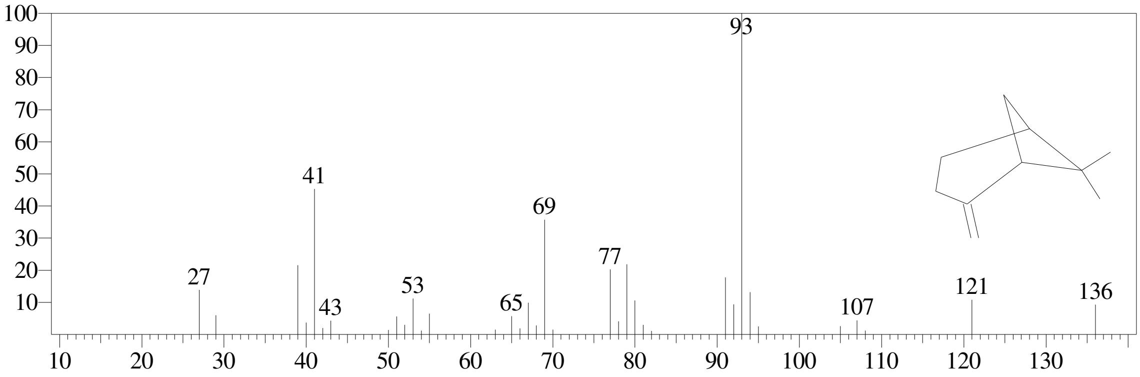


Hit#:2 Entry:11378 Library:NIST23-1.lib
SI:95 Formula:C10H16 CAS:127-91-3 MolWeight:136 RetIndex:978
CompName:.beta.-Pinene $$ Bicyclo[3.1.1]heptane, 6,6-dimethyl-2-methylene- $$ 2(10)-Pinene $$ Nopinen $$ Nopinene

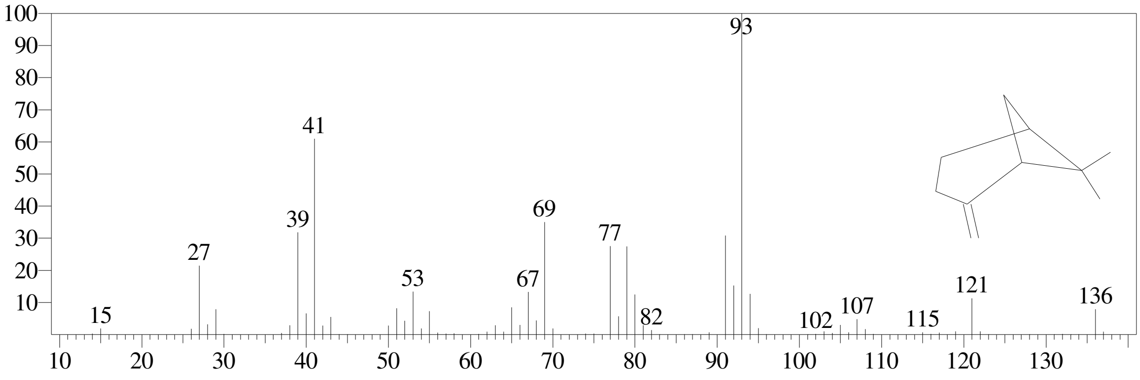

<< Target >>
Line#:5   R.Time:7.717(Scan#:627)  MassPeaks:25
RawMode:Averaged 7.708-7.725(626-628) BasePeak:93.05(41926)
BG Mode:None  Group 1 - Event 1  Scan

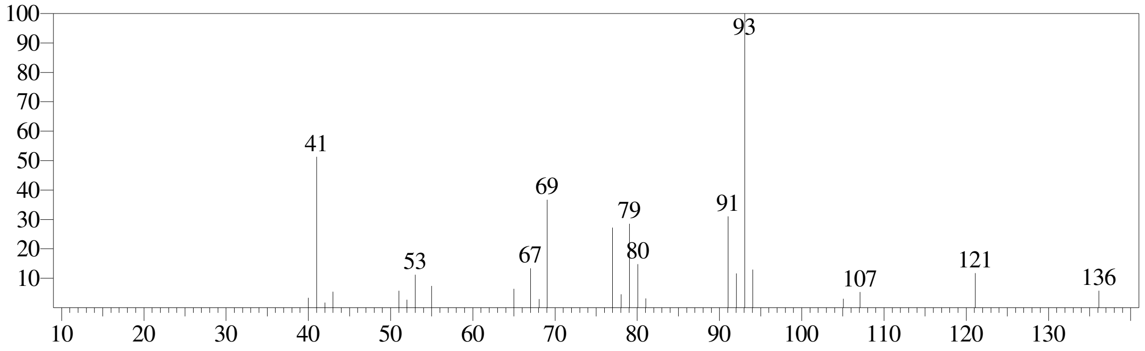


Hit#:3  Entry:8393  Library:NIST23s.lib
SI:95  Formula:C10H16  CAS:127-91-3  MolWeight:136  RetIndex:978
CompName:.beta.-Pinene $$ Bicyclo[3.1.1]heptane, 6,6-dimethyl-2-methylene- $$ 2(10)-Pinene $$ Nopinen $$ Nopinene

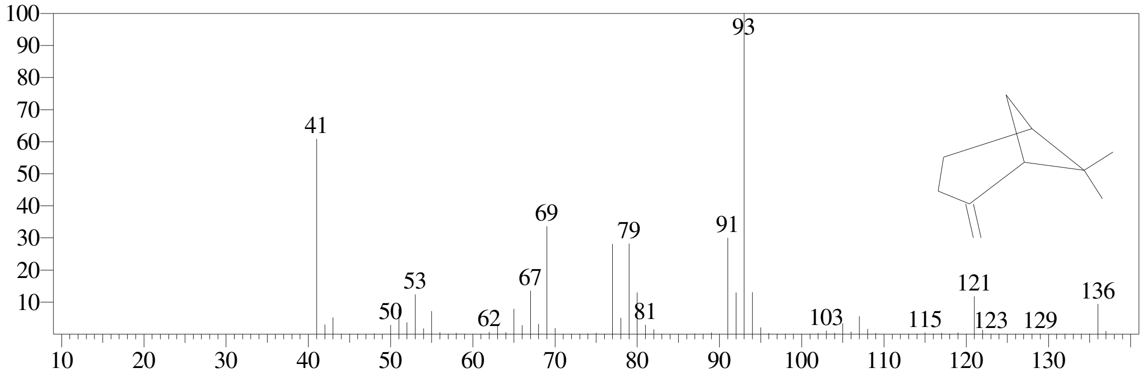


Hit#:4  Entry:8401  Library:NIST23s.lib
SI:94  Formula:C10H16  CAS:18172-67-3  MolWeight:136  RetIndex:978
CompName:Bicyclo[3.1.1]heptane, 6,6-dimethyl-2-methylene-, (1S)- $$ 2(10)-Pinene, (1S,5S)-(-)- $$ (-)-.beta.-Pinene $$

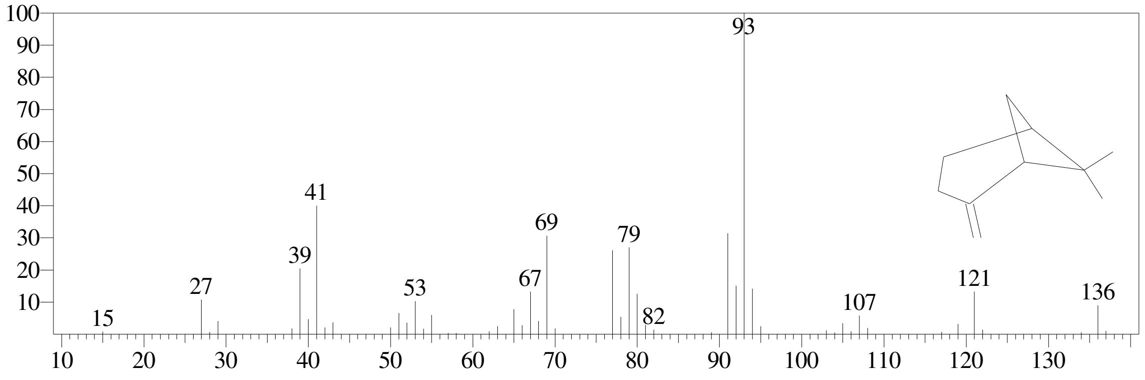

<< Target >>
Line#:5   R.Time:7.717(Scan#:627)  MassPeaks:25
RawMode:Averaged 7.708-7.725(626-628) BasePeak:93.05(41926)
BG Mode:None  Group 1 - Event 1  Scan

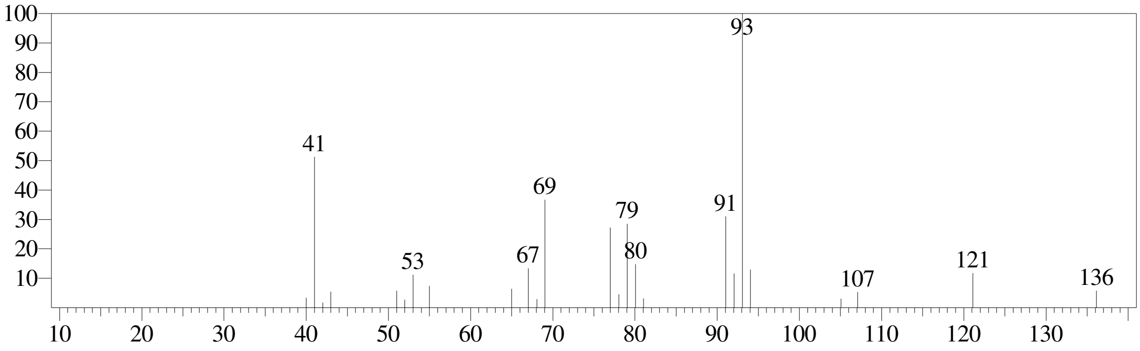


Hit#:5  Entry:8392  Library:NIST23s.lib
SI:94  Formula:C10H16  CAS:127-91-3  MolWeight:136  RetIndex:978
CompName:.beta.-Pinene $$ Bicyclo[3.1.1]heptane, 6,6-dimethyl-2-methylene- $$ 2(10)-Pinene $$ Nopinen $$ Nopinene

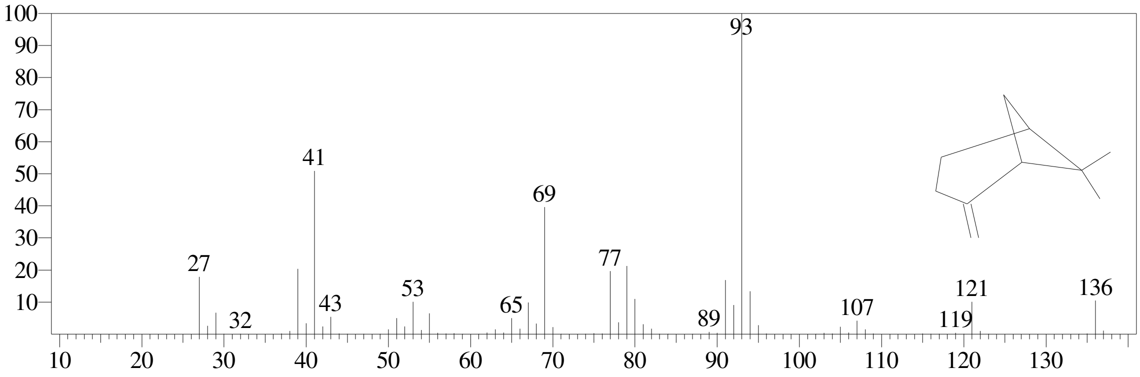

<< Target >>
Line#:6 R.Time:8.158(Scan#:680) MassPeaks:6
RawMode:Averaged 8.150-8.167(679-681) BasePeak:41.00(6361)
BG Mode:None Group 1 - Event 1 Scan

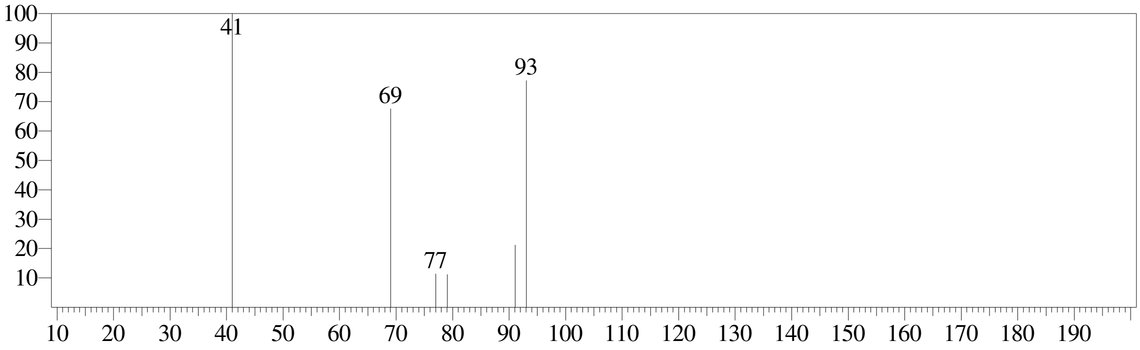


Hit#:1 Entry:58400 Library:NIST23-1.lib
SI:81 Formula:C10H16S2 CAS:73188-23-5 MolWeight:200 RetIndex:1622
CompName:4-(4-Methylpent-3-enyl)-3,6-dihydro-1,2-dithiin $$ 4-(4-Methyl-3-pentenyl)-3,6-dihydro-1,2-dithiine # $$

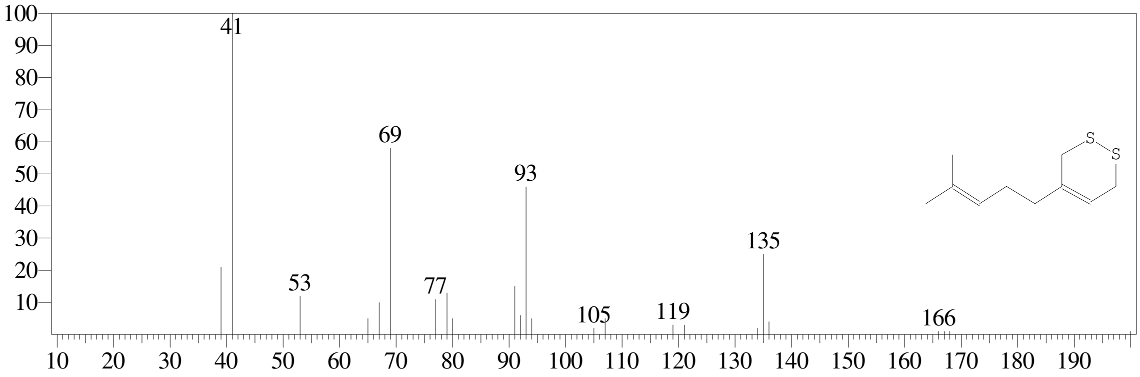


Hit#:2 Entry:8355 Library:NIST23s.lib
SI:81 Formula:C10H16 CAS:123-35-3 MolWeight:136 RetIndex:993
CompName:.beta.-Myrcene $$ 1,6-Octadiene, 7-methyl-3-methylene- $$ Myrcene $$ 7-Methyl-3-methylene-1,6-octadien

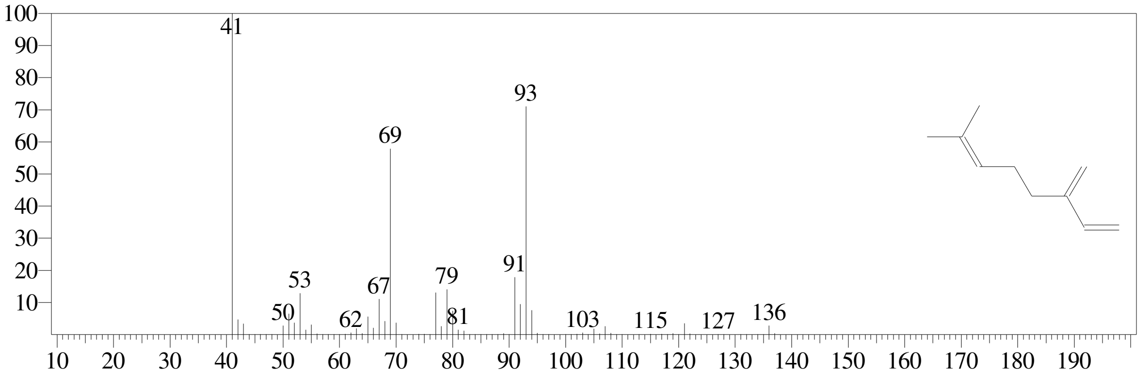

<< Target >>
Line#:6 R.Time:8.158(Scan#:680) MassPeaks:6
RawMode:Averaged 8.150-8.167(679-681) BasePeak:41.00(6361)
BG Mode:None Group 1 - Event 1 Scan

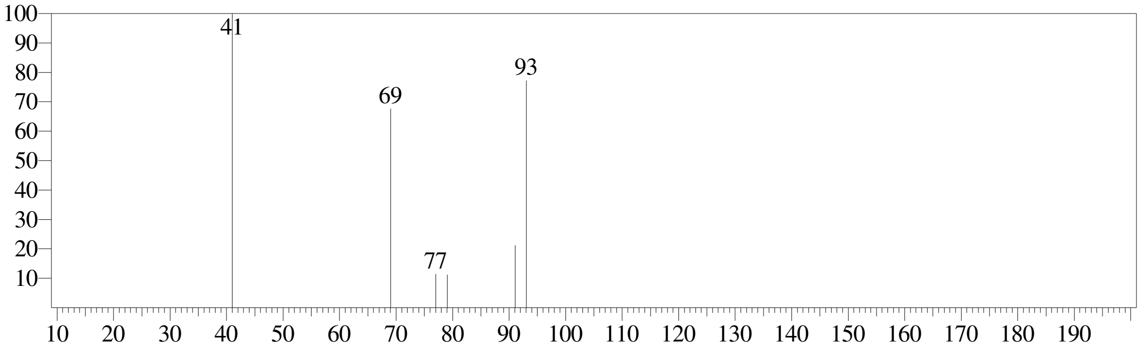


Hit#:3 Entry:8395 Library:NIST23s.lib
SI:81 Formula:C10H16 CAS:123-35-3 MolWeight:136 RetIndex:993
CompName:.beta.-Myrcene $$ 1,6-Octadiene, 7-methyl-3-methylene- $$ Myrcene $$ 7-Methyl-3-methylene-1,6-octadien

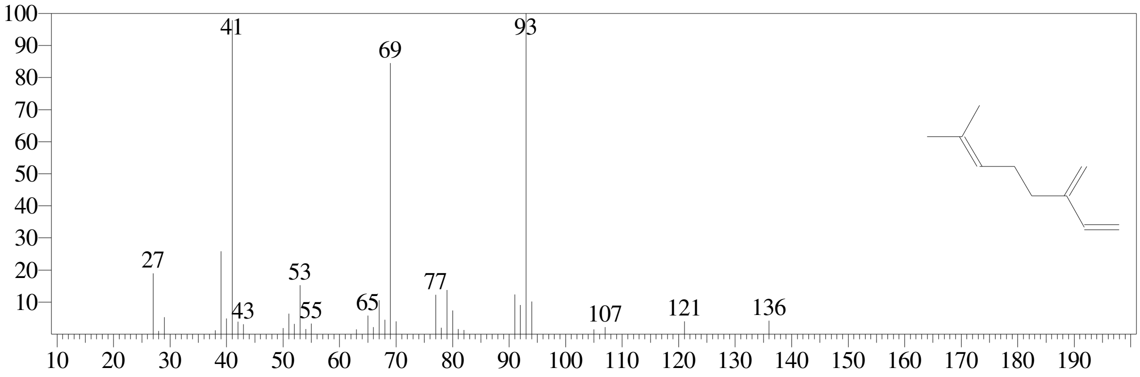


Hit#:4 Entry:11317 Library:NIST23-1.lib
SI:80 Formula:C10H16 CAS:123-35-3 MolWeight:136 RetIndex:993
CompName:.beta.-Myrcene $$ 1,6-Octadiene, 7-methyl-3-methylene- $$ Myrcene $$ 7-Methyl-3-methylene-1,6-octadien

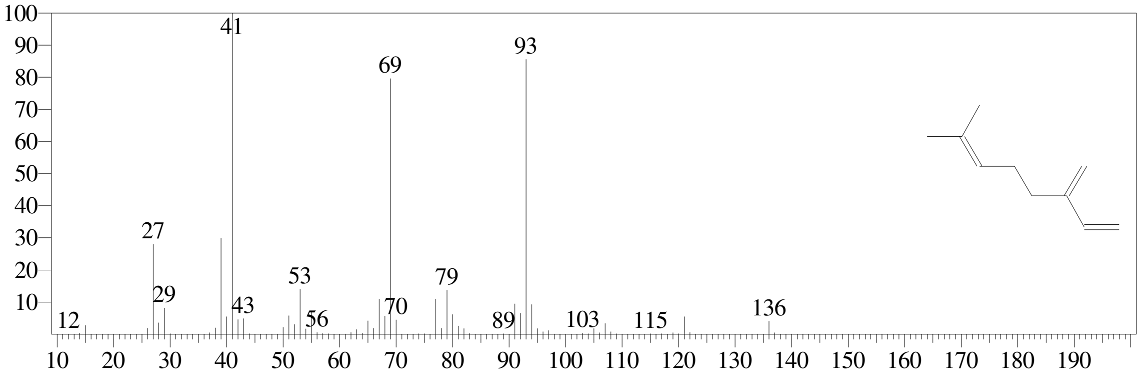

<< Target >>
Line#:6 R.Time:8.158(Scan#:680) MassPeaks:6
RawMode:Averaged 8.150-8.167(679-681) BasePeak:41.00(6361)
BG Mode:None Group 1 - Event 1 Scan

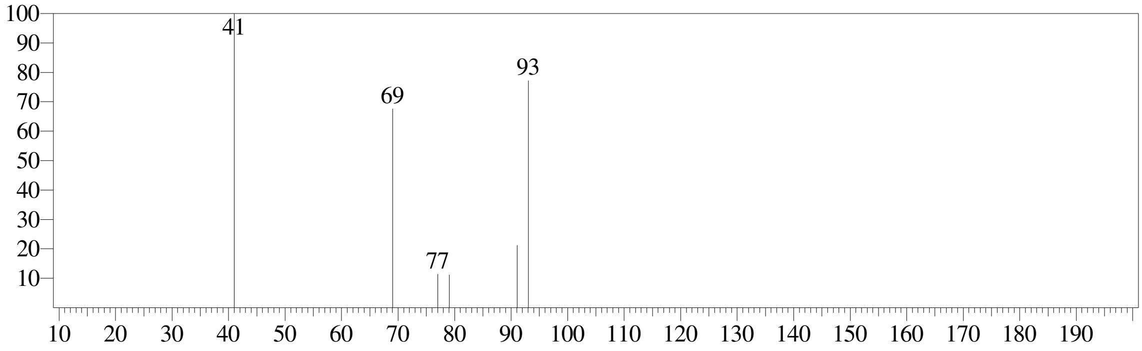


Hit#:5 Entry:25391 Library:NIST23-1.lib
SI:78 Formula:C10H11NO CAS:6580-95-6 MolWeight:161 RetIndex:1471
CompName:Ethanone, 1-cyclopropyl-2-(4-pyridinyl)- $$ Ketone, cyclopropyl 4-pyridylmethyl $$ Cyclopropyl 4-picolyl k

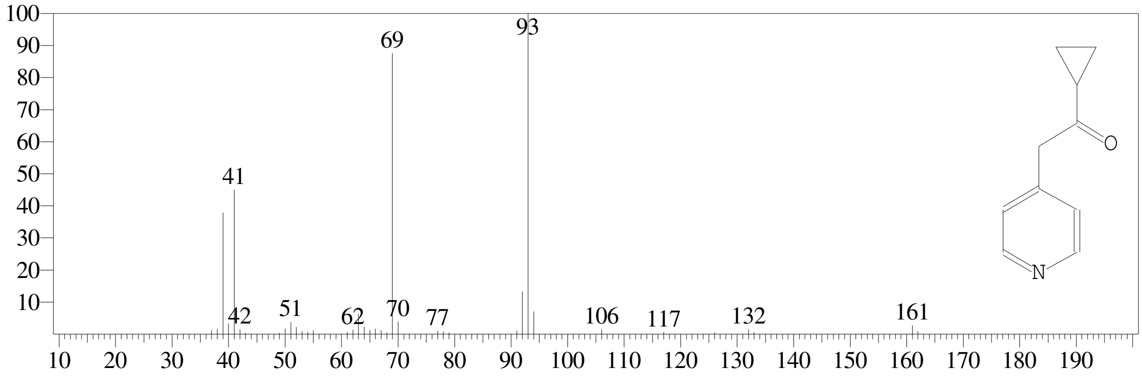

<< Target >>
Line#:7 R.Time:9.550(Scan#:847) MassPeaks:24
RawMode:Averaged 9.542-9.558(846-848) BasePeak:68.05(20589)
BG Mode:None Group 1 - Event 1 Scan

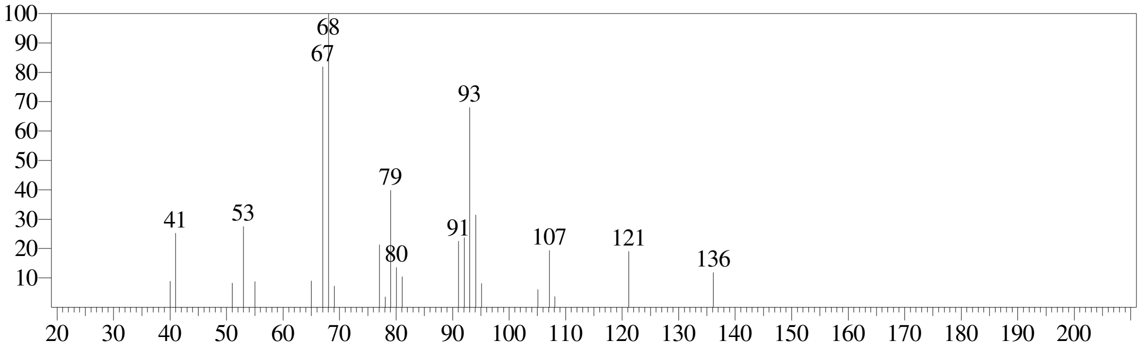


Hit#:1 Entry:8371 Library:NIST23s.lib
SI:93 Formula:C10H16 CAS:5989-27-5 MolWeight:136 RetIndex:1031
CompName:D-Limonene $$ Cyclohexene, 1-methyl-4-(1-methylethenyl)-, (R)- $$ p-Mentha-1,8-diene, (R)-(+)- $$ (+)-(R

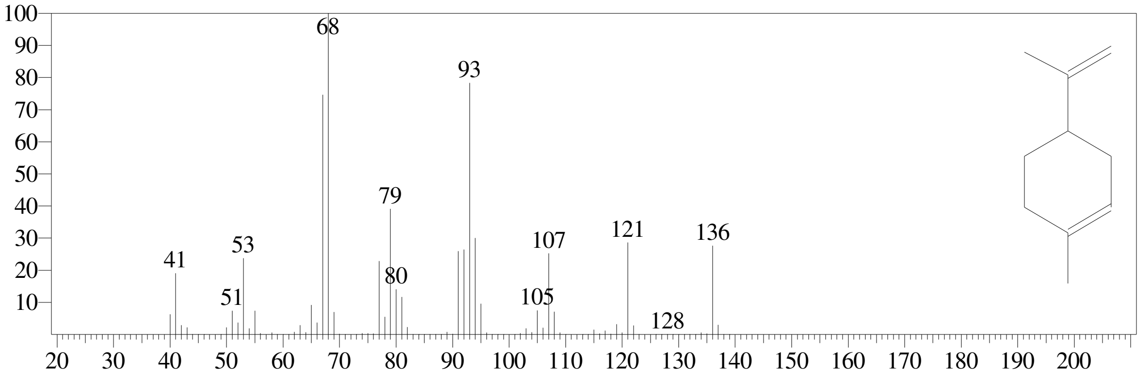


Hit#:2 Entry:8365 Library:NIST23s.lib
SI:92 Formula:C10H16 CAS:5989-54-8 MolWeight:136 RetIndex:1031
CompName:Cyclohexene, 1-methyl-4-(1-methylethenyl)-, (S)- $$ p-Mentha-1,8-diene, (S)-(-)- $$ (-)-Limonene $$ L-Lim

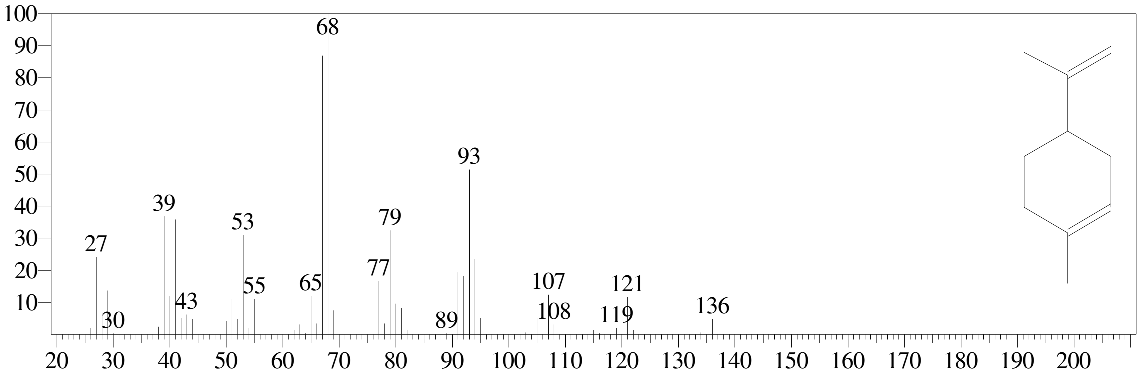

<< Target >>
Line#:7   R.Time:9.550(Scan#:847)  MassPeaks:24
RawMode:Averaged 9.542-9.558(846-848) BasePeak:68.05(20589)
BG Mode:None  Group 1 - Event 1  Scan

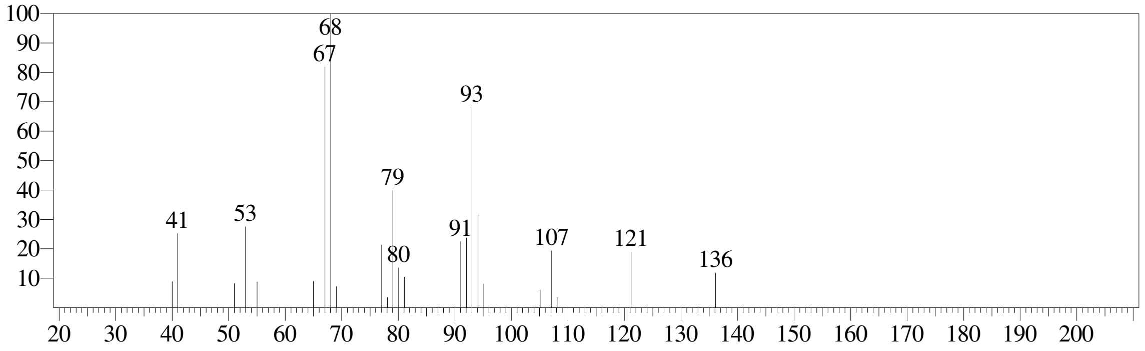


Hit#:3  Entry:11345  Library:NIST23-1.lib
SI:91  Formula:C10H16  CAS:19465-02-2  MolWeight:136  RetIndex:968
CompName:Cyclobutane, 1,2-bis(1-methylethenyl)-, trans- $$ 1,2-Diisopropenylcyclobutane  # $$

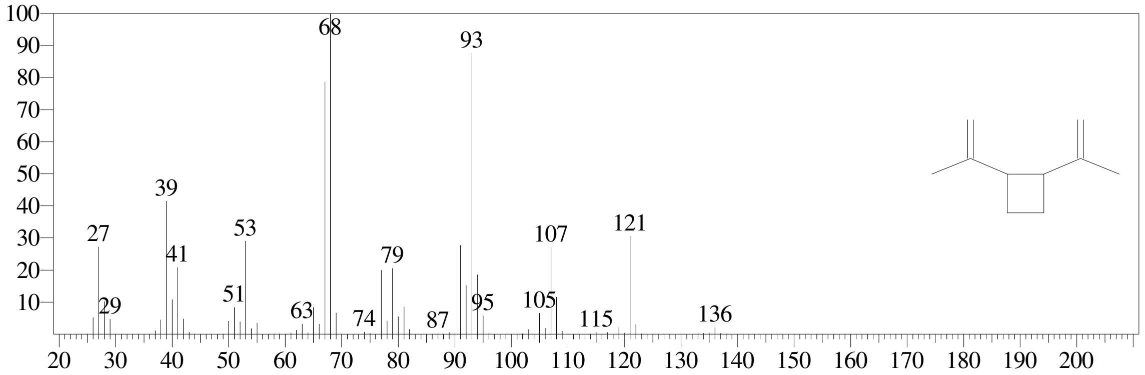


Hit#:4  Entry:8364  Library:NIST23s.lib
SI:91  Formula:C10H16  CAS:138-86-3  MolWeight:136  RetIndex:1031
CompName:Limonene $$ Cyclohexene, 1-methyl-4-(1-methylethenyl)- $$ p-Mentha-1,8-diene $$ .alpha.-Limonene $$ C

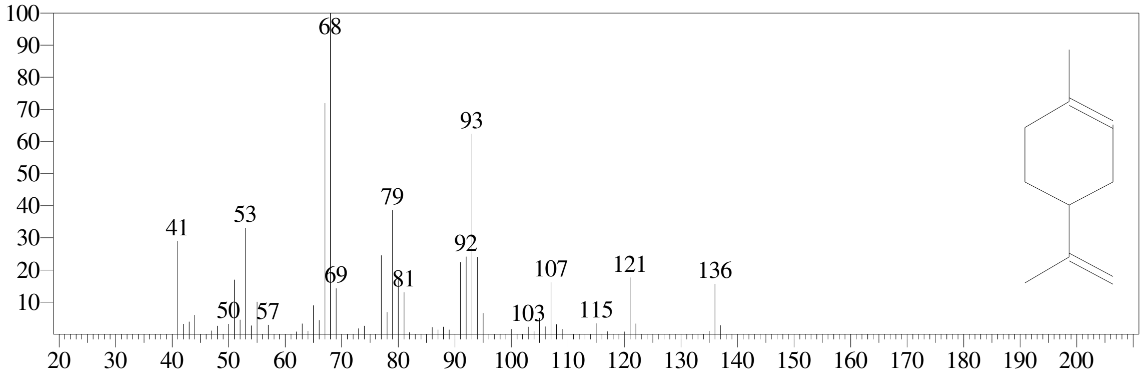

<< Target >>
Line#:7 R.Time:9.550(Scan#:847) MassPeaks:24
RawMode:Averaged 9.542-9.558(846-848) BasePeak:68.05(20589)
BG Mode:None Group 1 - Event 1 Scan

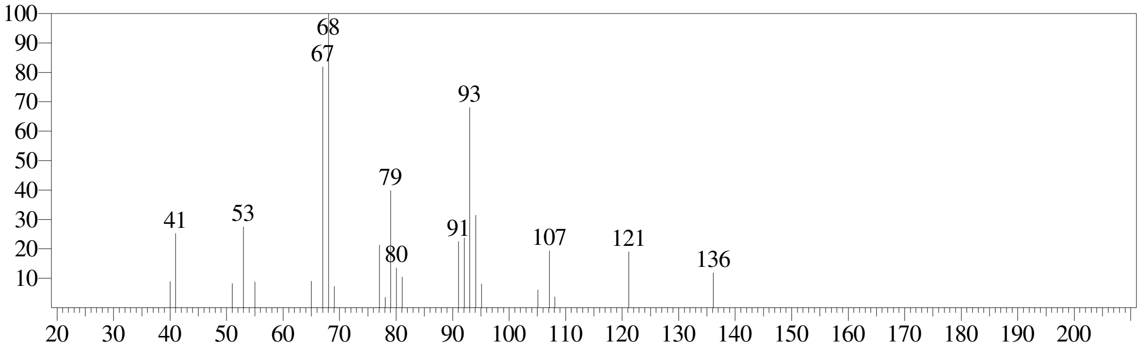

Hit#:5 Entry:11340 Library:NIST23-1.lib
SI:90 Formula:C10H16 CAS:138-86-3 MolWeight:136 RetIndex:1031
CompName:Limonene $$ Cyclohexene, 1-methyl-4-(1-methylethenyl)- $$ p-Mentha-1,8-diene $$ .alpha.-Limonene $$ C

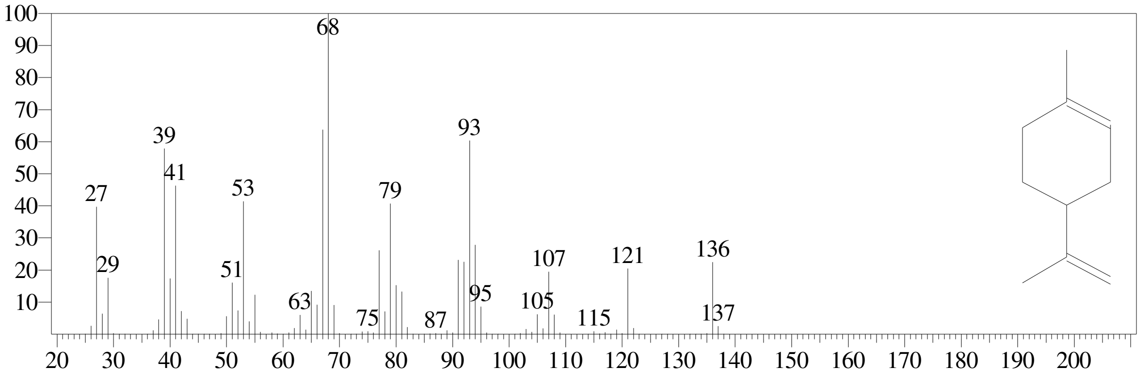

<< Target >>
Line#:8 R.Time:10.725(Scan#:988) MassPeaks:8
RawMode:Averaged 10.717-10.733(987-989) BasePeak:93.05(4464)
BG Mode:None Group 1 - Event 1 Scan

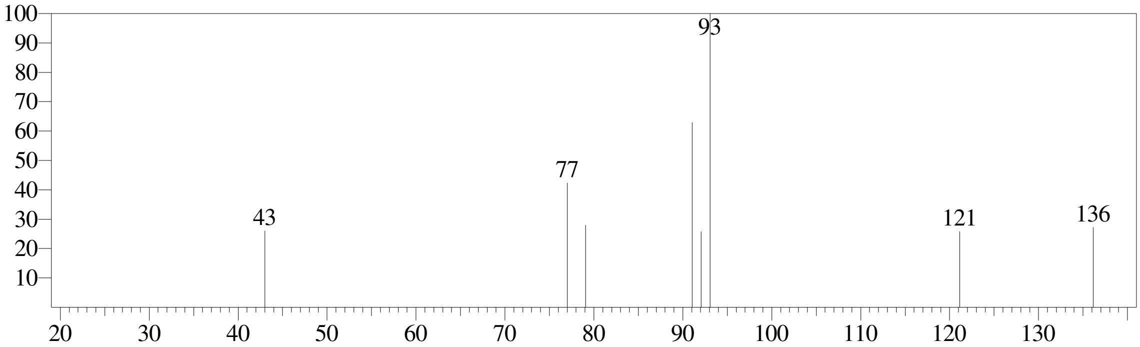


Hit#:1 Entry:8437 Library:NIST23s.lib
SI:84 Formula:C10H16 CAS:99-83-2 MolWeight:136 RetIndex:1017
CompName:.alpha.-Phellandrene $$ 1,3-Cyclohexadiene, 2-methyl-5-(1-methylethyl)- $$ .alpha.-Fellandrene $$ p-Mentha

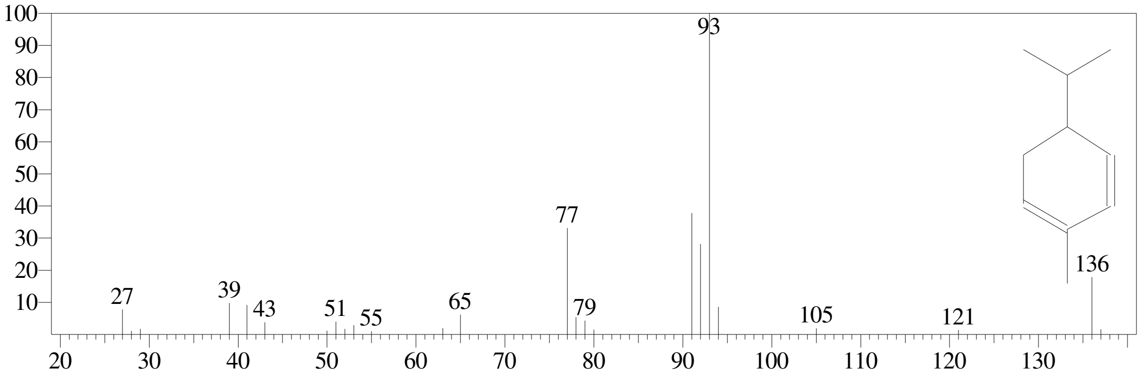


Hit#:2 Entry:8438 Library:NIST23s.lib
SI:83 Formula:C10H16 CAS:99-83-2 MolWeight:136 RetIndex:1017
CompName:.alpha.-Phellandrene $$ 1,3-Cyclohexadiene, 2-methyl-5-(1-methylethyl)- $$ .alpha.-Fellandrene $$ p-Mentha

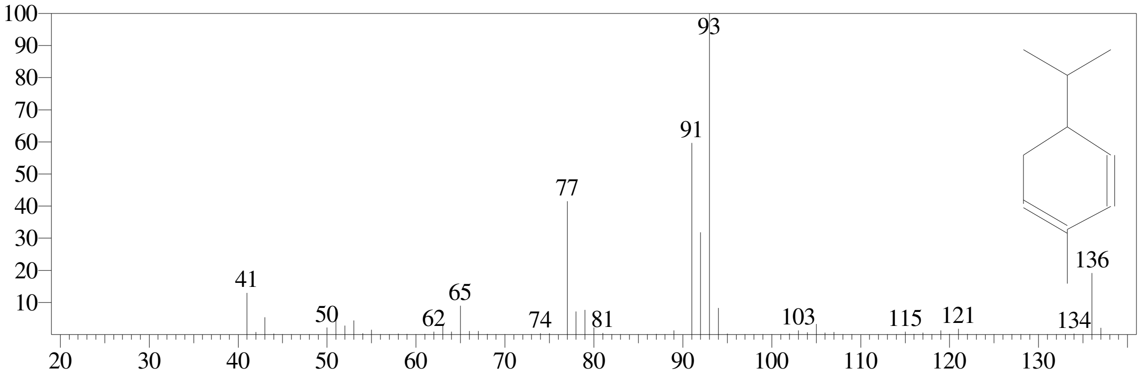

<< Target >>
Line#:8 R.Time:10.725(Scan#:988) MassPeaks:8
RawMode:Averaged 10.717-10.733(987-989) BasePeak:93.05(4464)
BG Mode:None Group 1 - Event 1 Scan

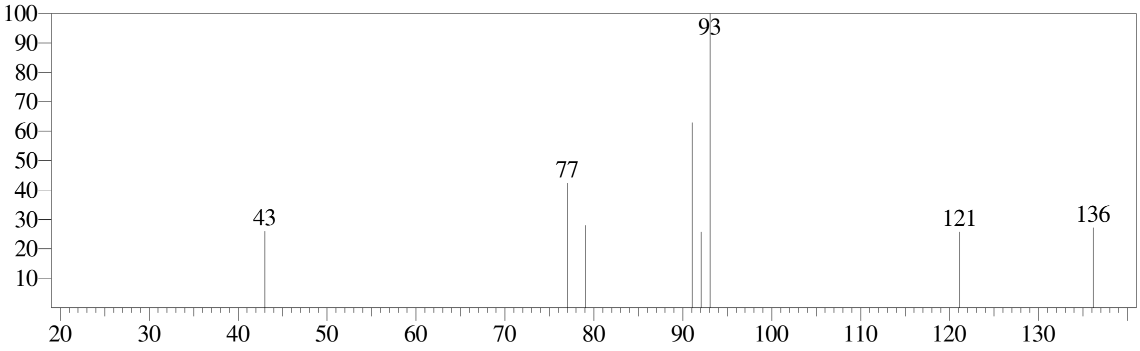


Hit#:3 Entry:11406 Library:NIST23-1.lib
SI:82 Formula:C10H16 CAS:2867-05-2 MolWeight:136 RetIndex:939
CompName:Bicyclo[3.1.0]hex-2-ene, 2-methyl-5-(1-methylethyl)- $$ 3-Thujene $$ .alpha.-Thujene $$ Thujene, .alpha.- $

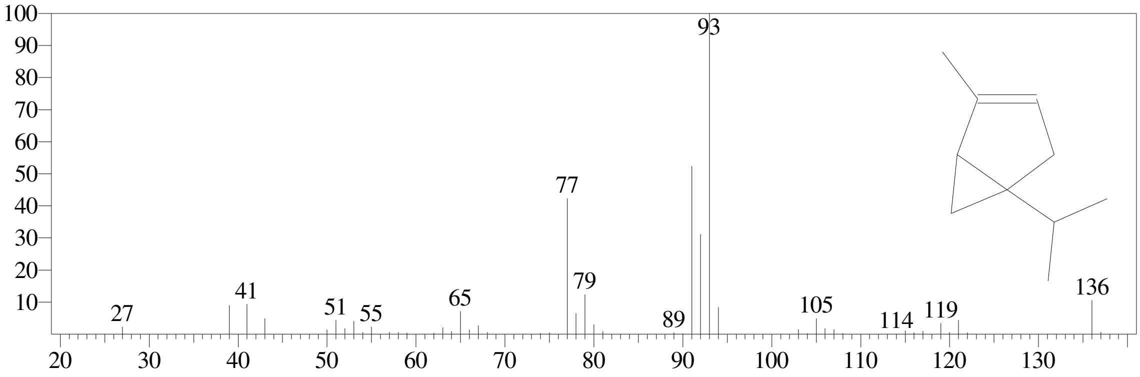


Hit#:4 Entry:8426 Library:NIST23s.lib
SI:82 Formula:C10H16 CAS:99-85-4 MolWeight:136 RetIndex:1038
CompName:.gamma.-Terpinene $$ 1,4-Cyclohexadiene, 1-methyl-4-(1-methylethyl)- $$ .gamma.-Terpinen $$ p-Mentha-

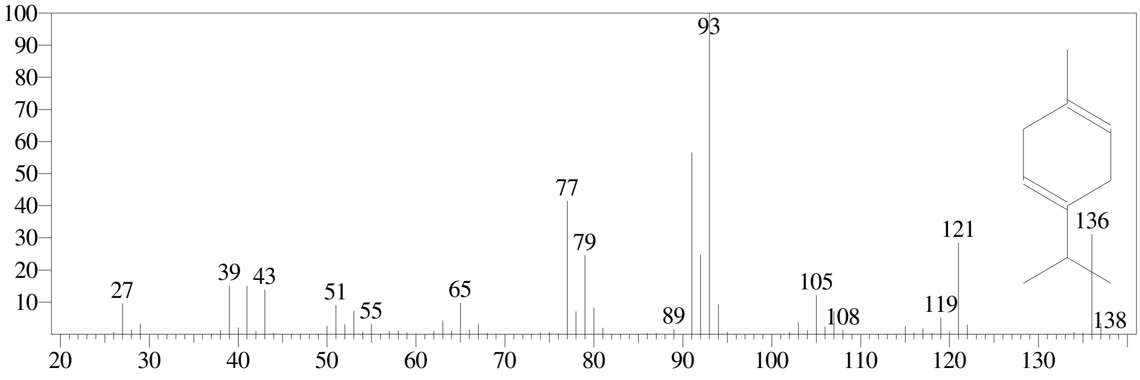

<< Target >>
Line#:8 R.Time:10.725(Scan#:988) MassPeaks:8
RawMode:Averaged 10.717-10.733(987-989) BasePeak:93.05(4464)
BG Mode:None Group 1 - Event 1 Scan

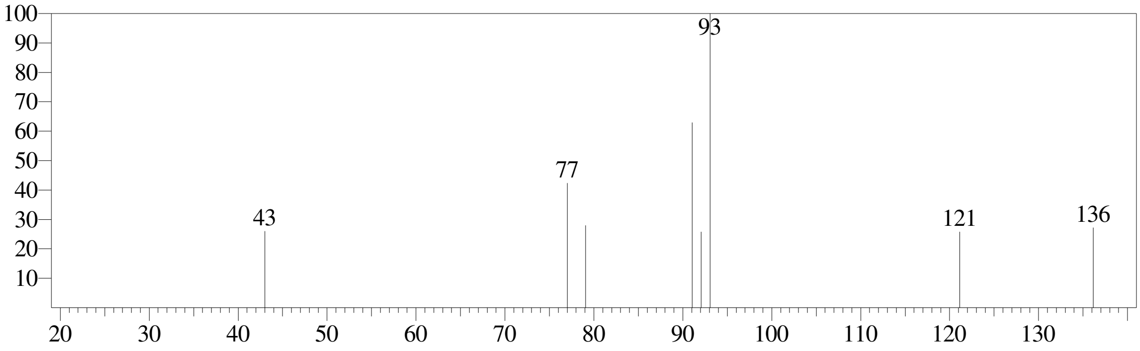


Hit#:5 Entry:8434 Library:NIST23s.lib
SI:82 Formula:C10H16 CAS:99-83-2 MolWeight:136 RetIndex:1017
CompName:.alpha.-Phellandrene $$ 1,3-Cyclohexadiene, 2-methyl-5-(1-methylethyl)- $$ .alpha.-Fellandrene $$ p-Mentha

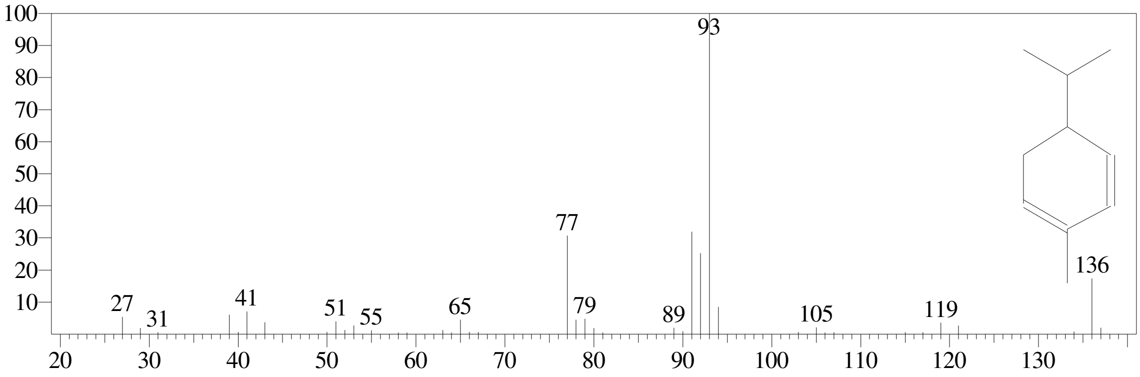

<< Target >>
Line#:9   R.Time:13.408(Scan#:1310)  MassPeaks:8
RawMode:Averaged 13.400-13.417(1309-1311) BasePeak:81.05(8635)
BG Mode:None  Group 1 - Event 1  Scan

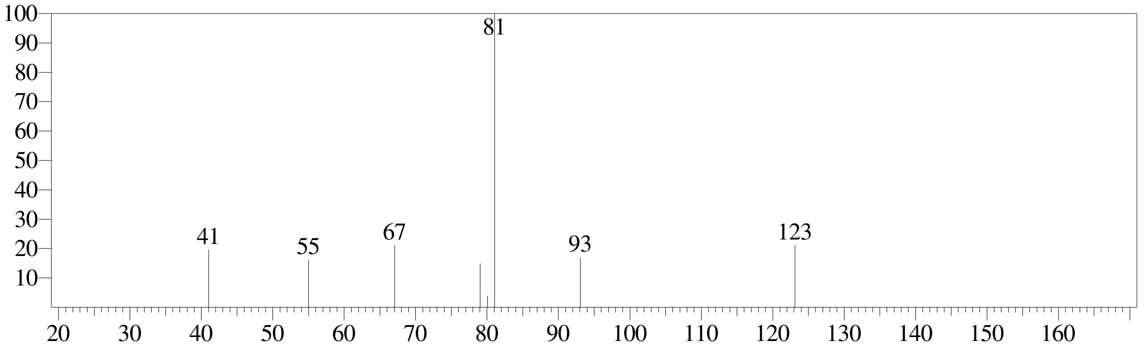


Hit#:1  Entry:27659  Library:NIST23-1.lib
SI:78  Formula:C12H20  CAS:83489-22-9  MolWeight:164  RetIndex:1174
CompName:1,E-4,Z-8-Dodecatriene $$ (4E,8Z)-1,4,8-Dodecatriene  # $$

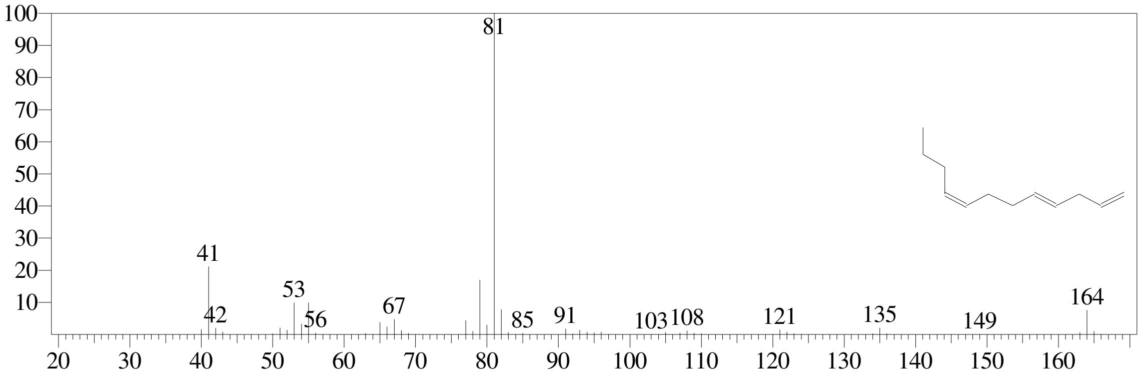


Hit#:2  Entry:6395  Library:NIST23-1.lib
SI:76  Formula:C9H14  CAS:15232-95-8  MolWeight:122  RetIndex:921
CompName:Cyclohexene,3-(2-propenyl)- $$ 3-Allylcyclohexene $$ 3-Allyl-1-cyclohexene  # $$ 3-Allylcyclohexene-1 $$

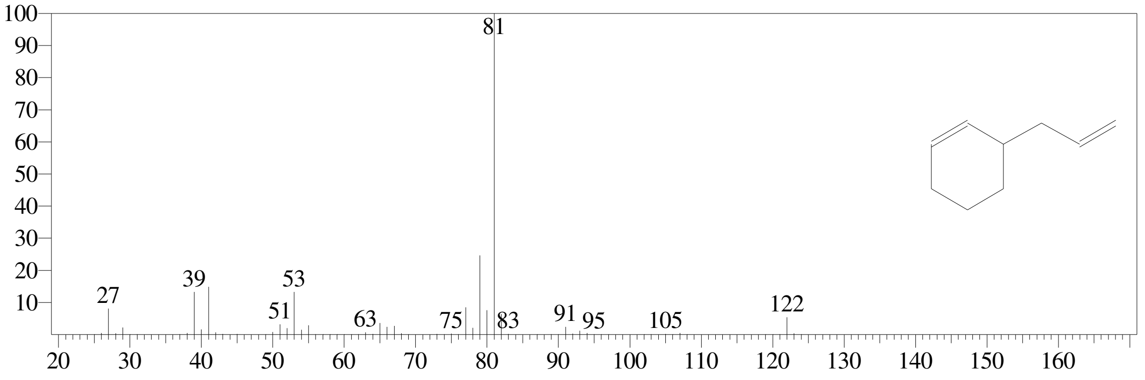

<< Target >>
Line#:9 R.Time:13.408(Scan#:1310) MassPeaks:8
RawMode:Averaged 13.400-13.417(1309-1311) BasePeak:81.05(8635)
BG Mode:None Group 1 - Event 1 Scan

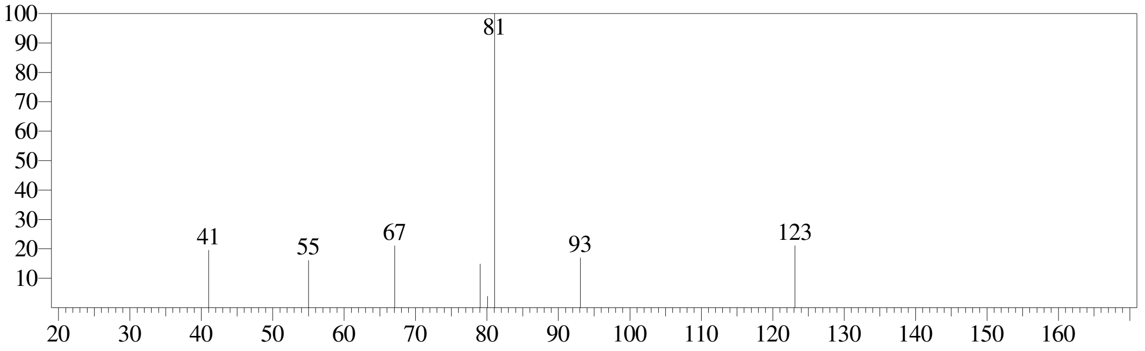


Hit#:3 Entry:5518 Library:NIST23s.lib
SI:76 Formula:C9H14 CAS:15232-95-8 MolWeight:122 RetIndex:921
CompName:Cyclohexene,3-(2-propenyl)- $$ 3-Allylcyclohexene $$ 3-Allyl-1-cyclohexene # $$ 3-Allylcyclohexene-1 $$

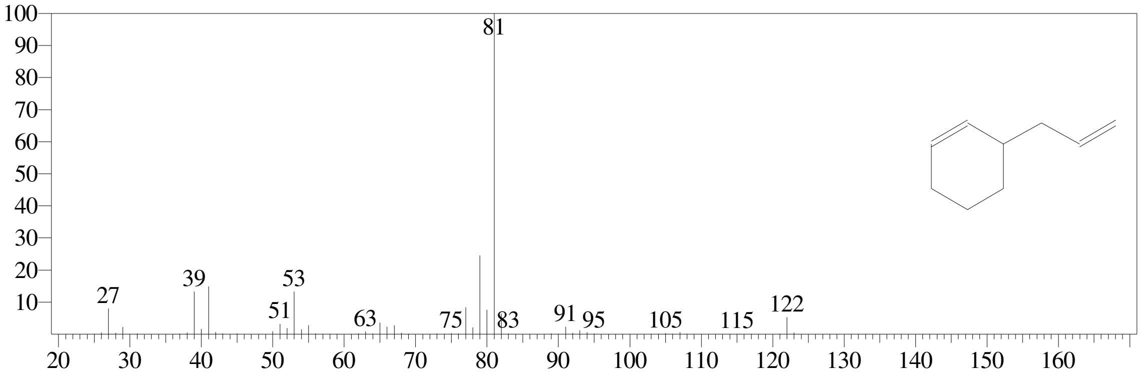


Hit#:4 Entry:19383 Library:NIST23-1.lib
SI:76 Formula:C9H12O2 CAS:31681-26-2 MolWeight:152 RetIndex:1119
CompName:2-Furanacetaldehyde, .alpha.-propyl- $$ 2-(2-Furyl)pentanal # $$

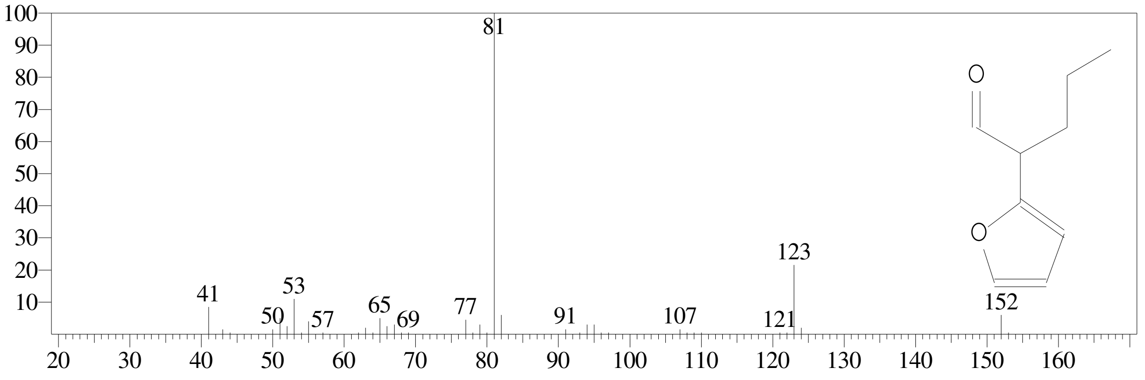

<< Target >>
Line#:9 R.Time:13.408(Scan#:1310) MassPeaks:8
RawMode:Averaged 13.400-13.417(1309-1311) BasePeak:81.05(8635)
BG Mode:None Group 1 - Event 1 Scan

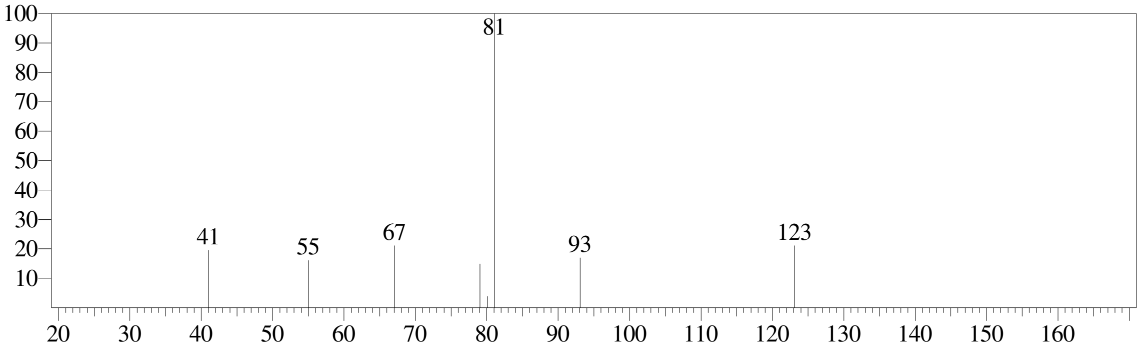


Hit#:5 Entry:1562 Library:NIST23-1.lib
SI:75 Formula:C7H12 CAS:17065-18-8 MolWeight:96 RetIndex:675
CompName:Bicyclo[2.1.0]pentane, 1,4-dimethyl- $$ 1,4-Dimethylbicyclo[2.1.0]pentane # $$

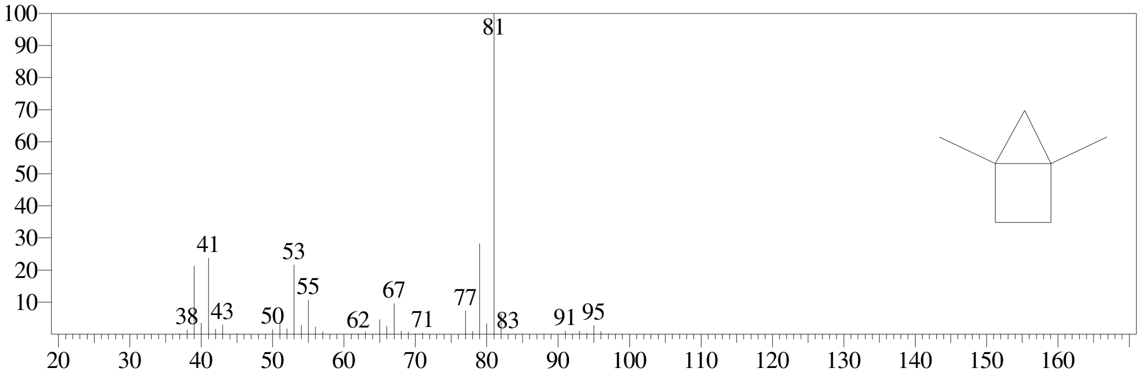

<< Target >>
Line#:10 R.Time:15.100(Scan#:1513) MassPeaks:29
RawMode:Averaged 15.092-15.108(1512-1514) BasePeak:95.10(31978)
BG Mode:None Group 1 - Event 1 Scan

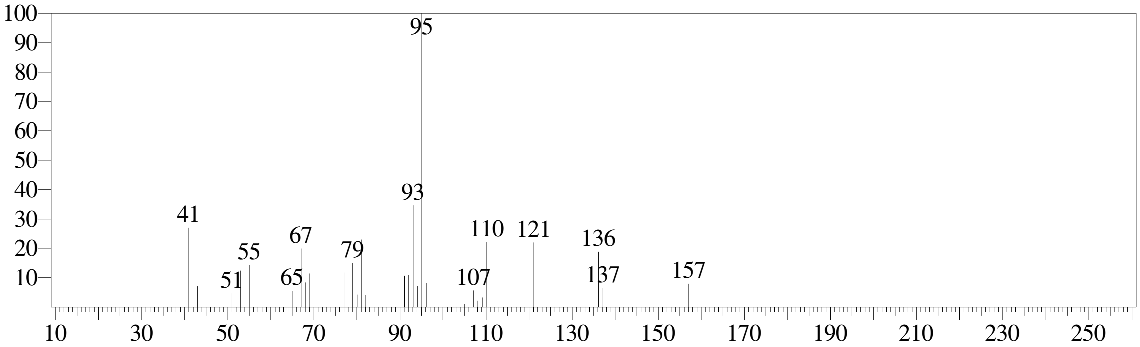


Hit#:1 Entry:33826 Library:NIST23-1.lib
SI:91 Formula:C10H17Cl CAS:30462-53-4 MolWeight:172 RetIndex:1165
CompName:Bicyclo[2.2.1]heptane, 2-chloro-1,7,7-trimethyl-, (1R-endo)- $$ 2-Chloro-1,7,7-trimethylbicyclo[2.2.1]heptar

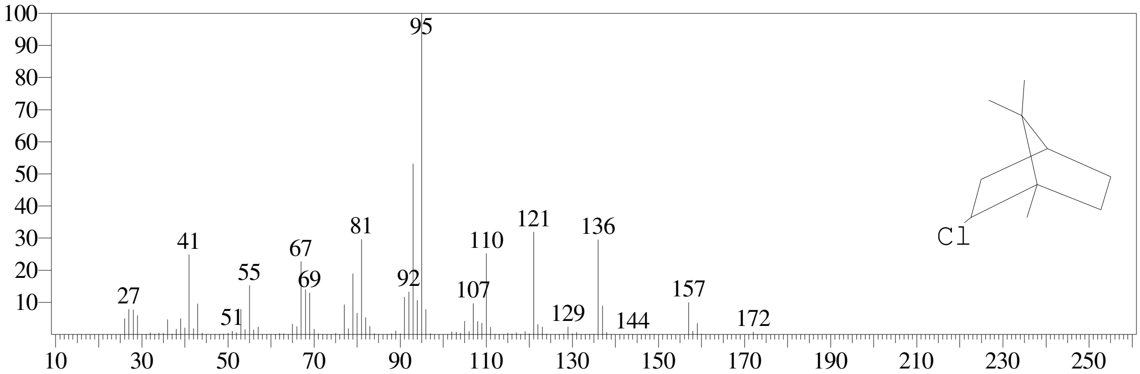


Hit#:2 Entry:33825 Library:NIST23-1.lib
SI:90 Formula:C10H17Cl CAS:464-41-5 MolWeight:172 RetIndex:1165
CompName:Bornyl chloride $$ Bicyclo[2.2.1]heptane, 2-chloro-1,7,7-trimethyl-, endo- $$ Bornane, 2-chloro-, endo- $$ T

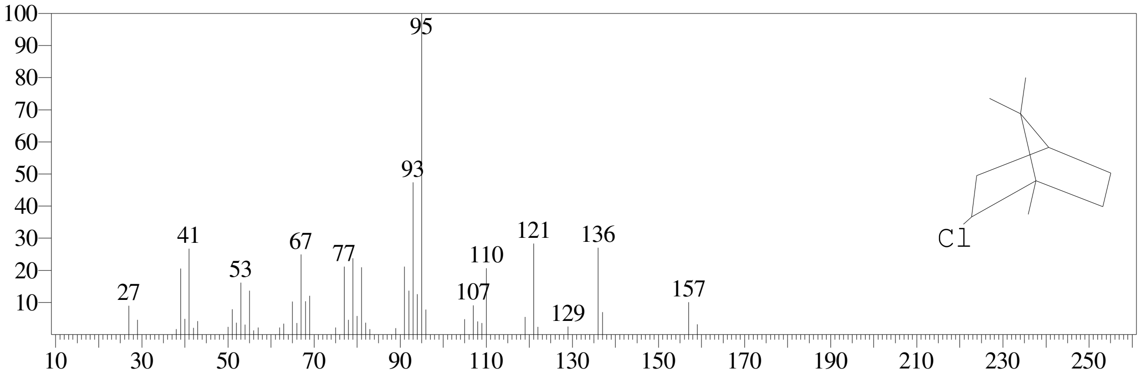

<< Target >>
Line#:10 R.Time:15.100(Scan#:1513) MassPeaks:29
RawMode:Averaged 15.092-15.108(1512-1514) BasePeak:95.10(31978)
BG Mode:None Group 1 - Event 1 Scan

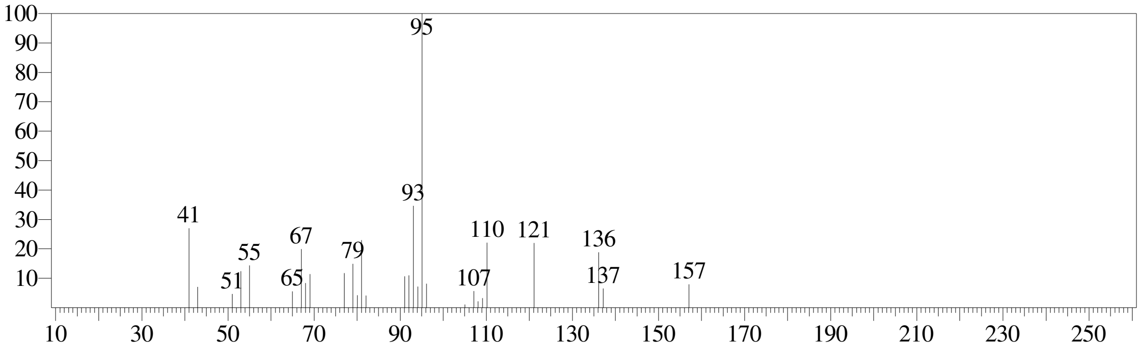


Hit#:3 Entry:32803 Library:NIST23s.lib
SI:89 Formula:C12H17F3O2 CAS:28587-55-5 MolWeight:250 RetIndex:1141
CompName:Borneol, trifluoroacetate (ester) $$ 1,7,7-Trimethylbicyclo[2.2.1]hept-2-yl trifluoroacetate # $$

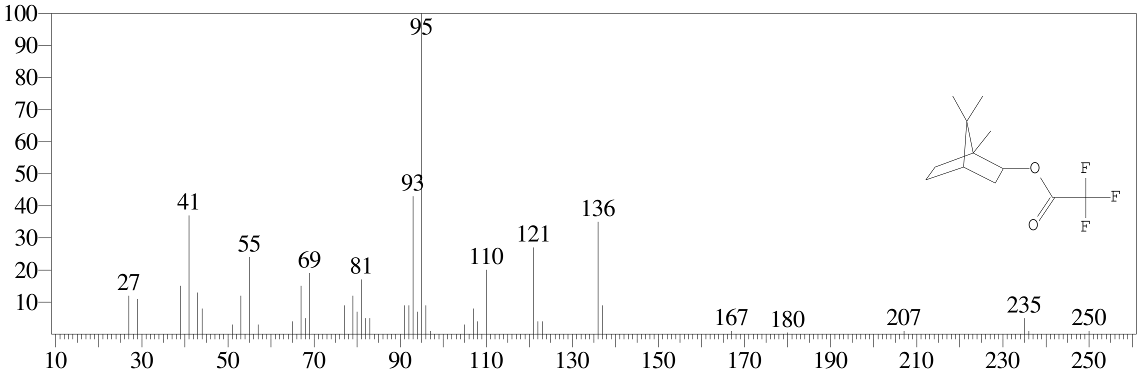


Hit#:4 Entry:116904 Library:NIST23-1.lib
SI:89 Formula:C12H17F3O2 CAS:28587-55-5 MolWeight:250 RetIndex:1141
CompName:Borneol, trifluoroacetate (ester) $$ 1,7,7-Trimethylbicyclo[2.2.1]hept-2-yl trifluoroacetate # $$

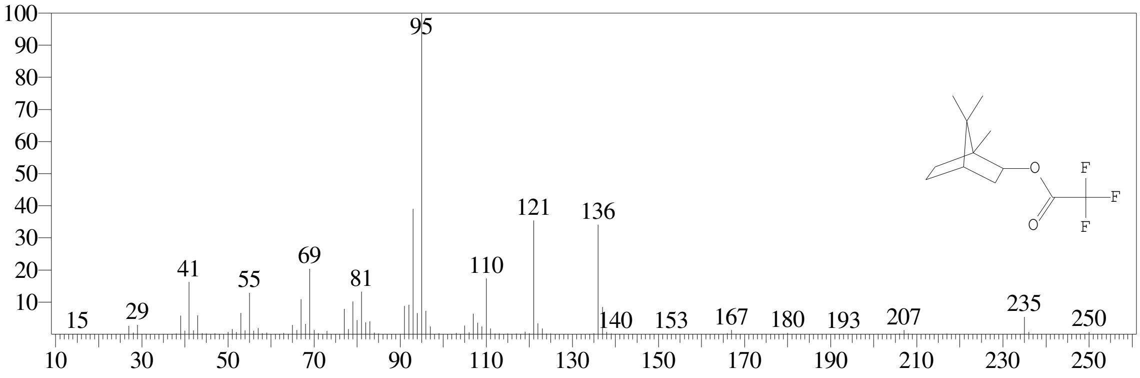

<< Target >>
Line#:10   R.Time:15.100(Scan#:1513)  MassPeaks:29
RawMode:Averaged 15.092-15.108(1512-1514) BasePeak:95.10(31978)
BG Mode:None  Group 1 - Event 1  Scan

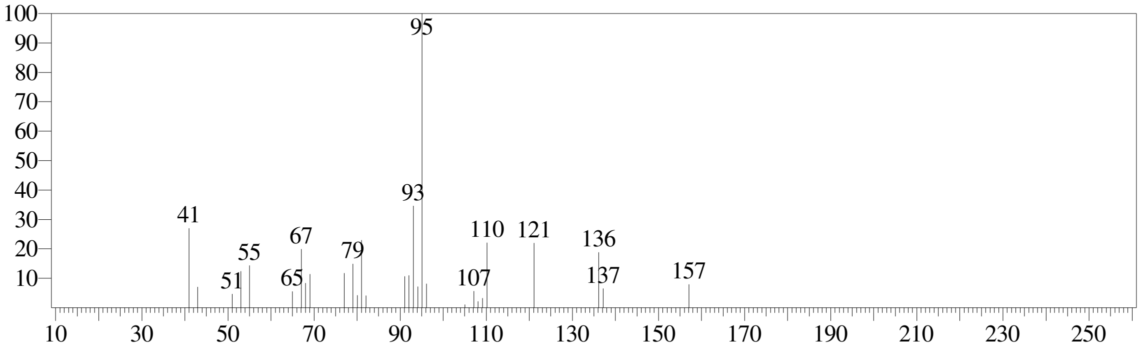


Hit#:5  Entry:19801  Library:NIST23s.lib
SI:87  Formula:C11H18O2  CAS:7492-41-3  MolWeight:182  RetIndex:1240
CompName:Bicyclo[2.2.1]heptan-2-ol, 1,7,7-trimethyl-, formate, endo- $$ Borneol, formate $$ Bornyl formate $$ 1,7,7-T

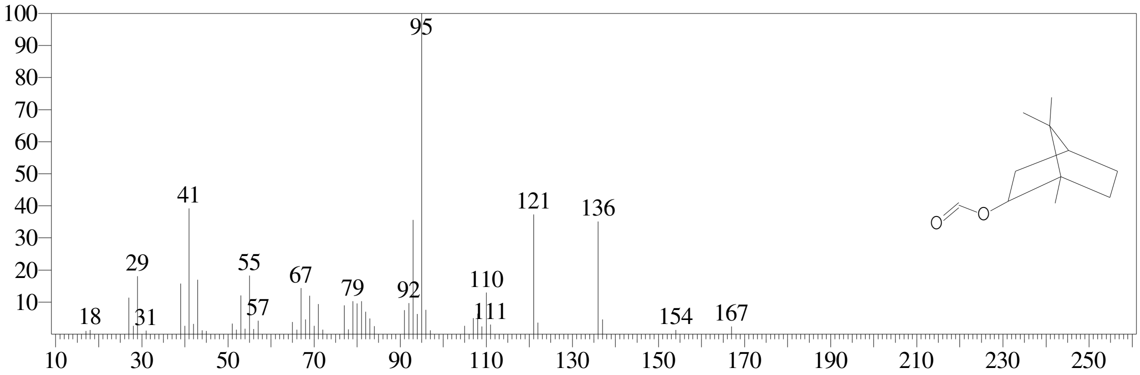

<< Target >>
Line#:11 R.Time:16.308(Scan#:1658) MassPeaks:7
RawMode:Averaged 16.300-16.317(1657-1659) BasePeak:59.05(3912)
BG Mode:None Group 1 - Event 1 Scan

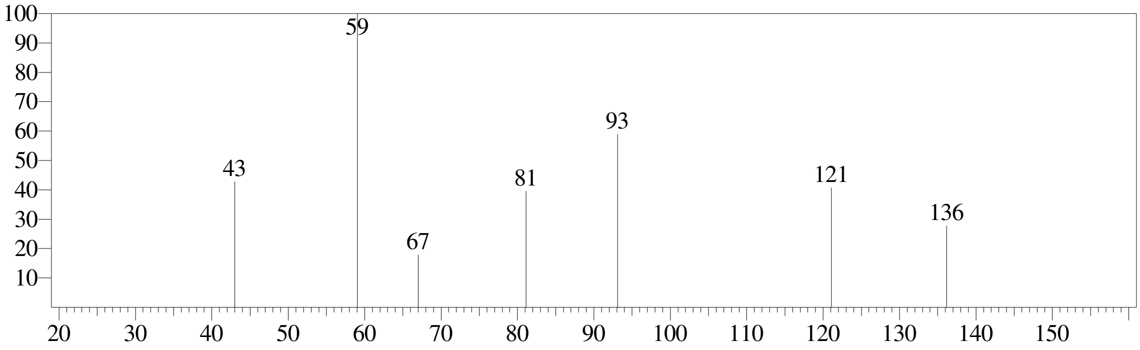


Hit#:1 Entry:20999 Library:NIST23-1.lib
SI:74 Formula:C10H18O CAS:7785-53-7 MolWeight:154 RetIndex:1183
CompName:3-Cyclohexene-1-methanol, .alpha.,.alpha.,4-trimethyl-, (R)- $$ 3-Cyclohexene-1-methanol, .alpha.,.alpha.,4-

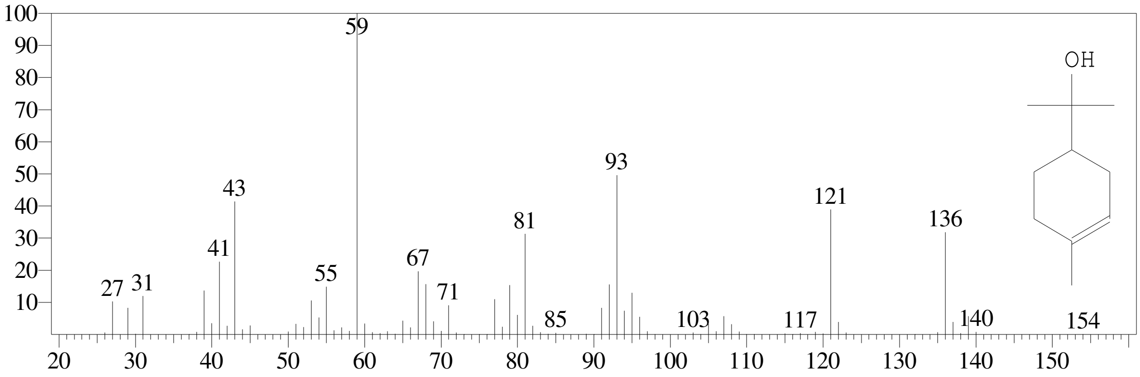


Hit#:2 Entry:12921 Library:NIST23s.lib
SI:74 Formula:C10H18O CAS:98-55-5 MolWeight:154 RetIndex:1183
CompName:.alpha.-Terpineol $$ 3-Cyclohexene-1-methanol, .alpha.,.alpha.4-trimethyl- $$ p-Menth-1-en-8-ol $$ Terpine

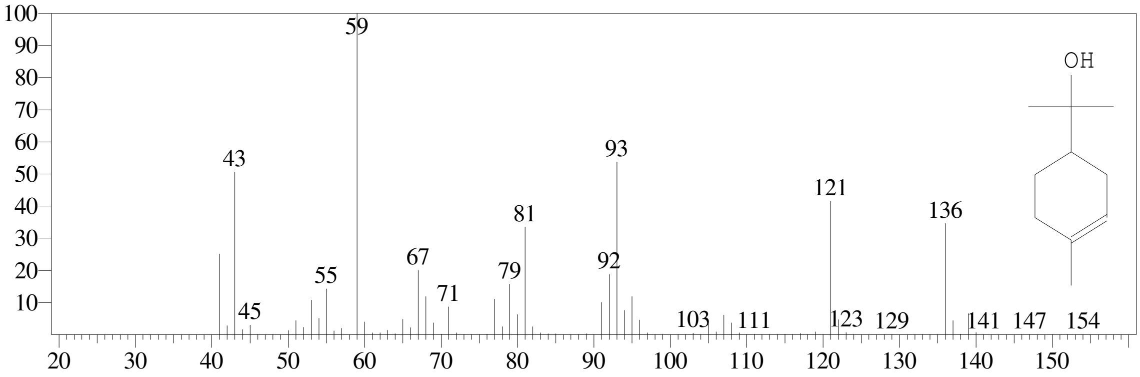

<< Target >>
Line#:11 R.Time:16.308(Scan#:1658) MassPeaks:7
RawMode:Averaged 16.300-16.317(1657-1659) BasePeak:59.05(3912)
BG Mode:None Group 1 - Event 1 Scan

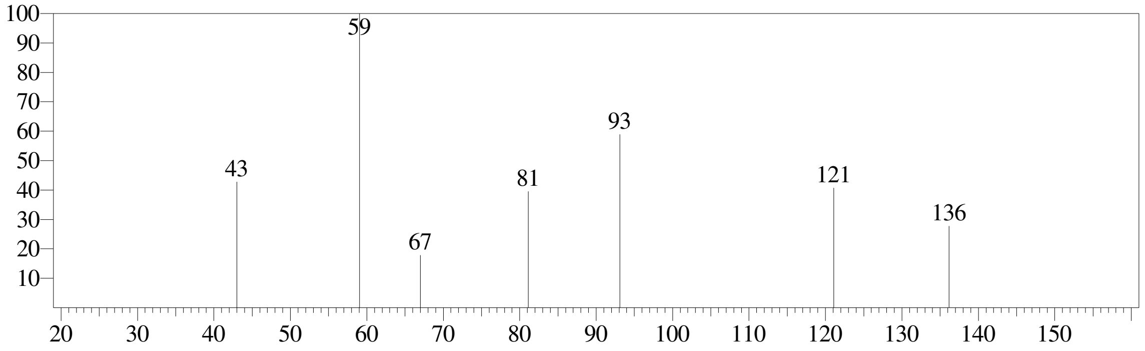


Hit#:3 Entry:20996 Library:NIST23-1.lib
SI:74 Formula:C10H18O CAS:7299-42-5 MolWeight:154 RetIndex:1166
CompName:Cyclohexanemethanol, .alpha.,.alpha.-dimethyl-4-methylene- $$ .delta.-Terpineol $$ .alpha.,.alpha.-Dimethyl

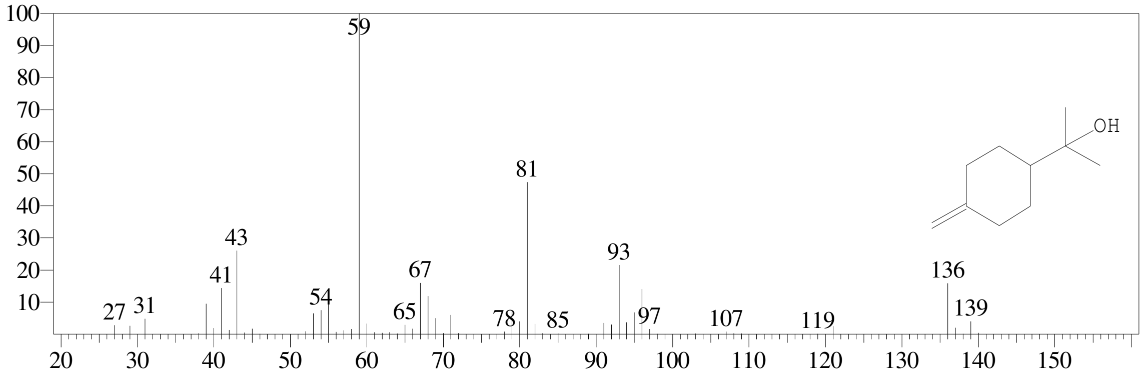


Hit#:4 Entry:12922 Library:NIST23s.lib
SI:73 Formula:C10H18O CAS:10482-56-1 MolWeight:154 RetIndex:1183
CompName:L-.alpha.-Terpineol $$ 3-Cyclohexene-1-methanol, .alpha.,.alpha.,4-trimethyl-, (S)- $$ p-Menth-1-en-8-ol, (S

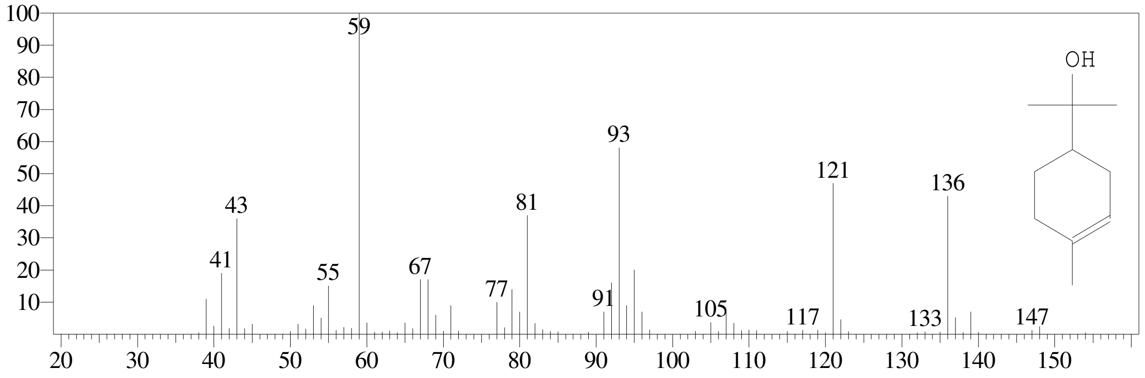

<< Target >>
Line#:11 R.Time:16.308(Scan#:1658) MassPeaks:7
RawMode:Averaged 16.300-16.317(1657-1659) BasePeak:59.05(3912)
BG Mode:None Group 1 - Event 1 Scan

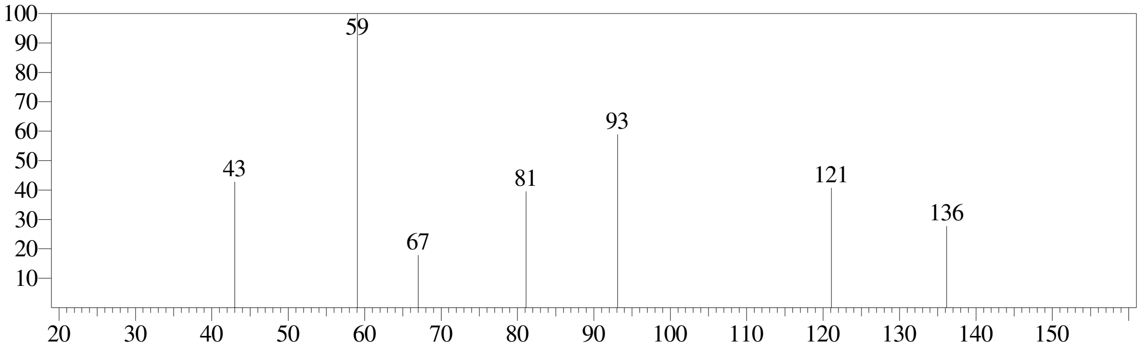


Hit#:5 Entry:21001 Library:NIST23-1.lib
SI:73 Formula:C10H18O CAS:10482-56-1 MolWeight:154 RetIndex:1183
CompName:L-.alpha.-Terpineol $$ 3-Cyclohexene-1-methanol, .alpha.,.alpha.,4-trimethyl-, (S)- $$ p-Menth-1-en-8-ol, (S

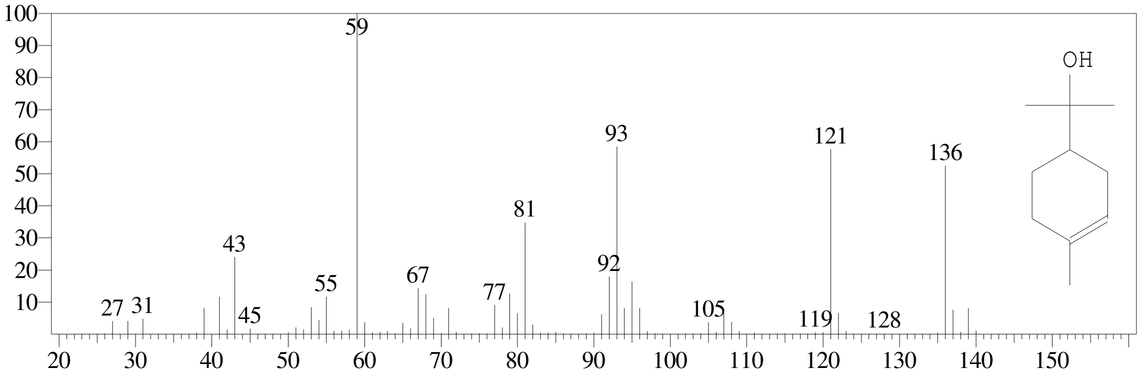

<< Target >>
Line#:12 R.Time:20.483(Scan#:2159) MassPeaks:6
RawMode:Averaged 20.475-20.492(2158-2160) BasePeak:95.10(3486)
BG Mode:None Group 1 - Event 1 Scan

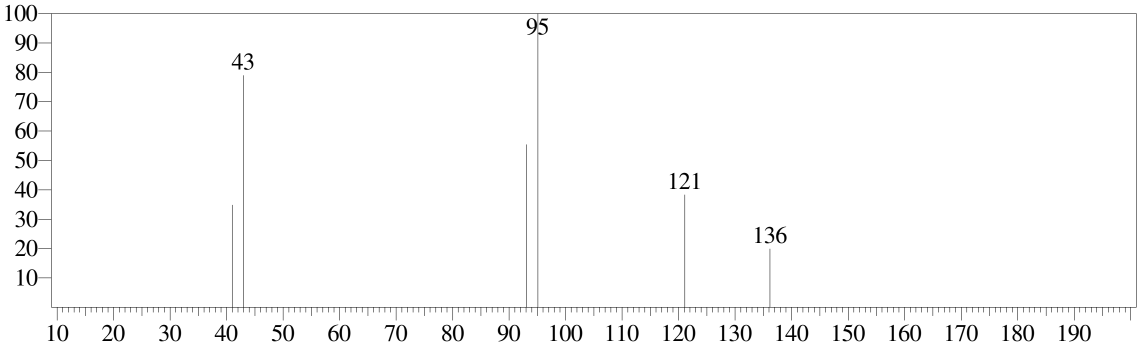


Hit#:1 Entry:23098 Library:NIST23s.lib
SI:70 Formula:C12H20O2 CAS:76-49-3 MolWeight:196 RetIndex:1288
CompName:Bornyl acetate $$ Bicyclo[2.2.1]heptan-2-ol, 1,7,7-trimethyl-, acetate, endo- $$ Borneol, acetate $$ Bornyl ac

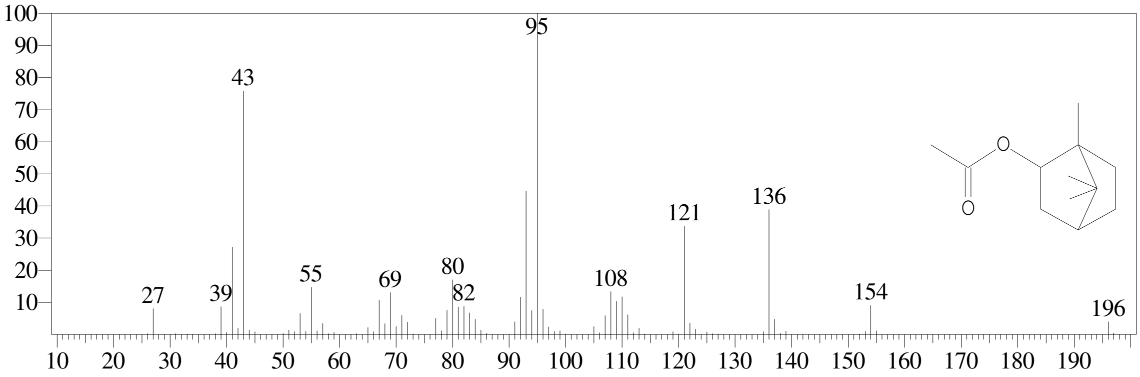


Hit#:2 Entry:54896 Library:NIST23-1.lib
SI:69 Formula:C12H20O2 CAS:5655-61-8 MolWeight:196 RetIndex:1288
CompName:Bicyclo[2.2.1]heptan-2-ol, 1,7,7-trimethyl-, acetate, (1S-endo)- $$ Borneol, acetate, (1S,2R,4S)-(-)- $$ (-)-Bo

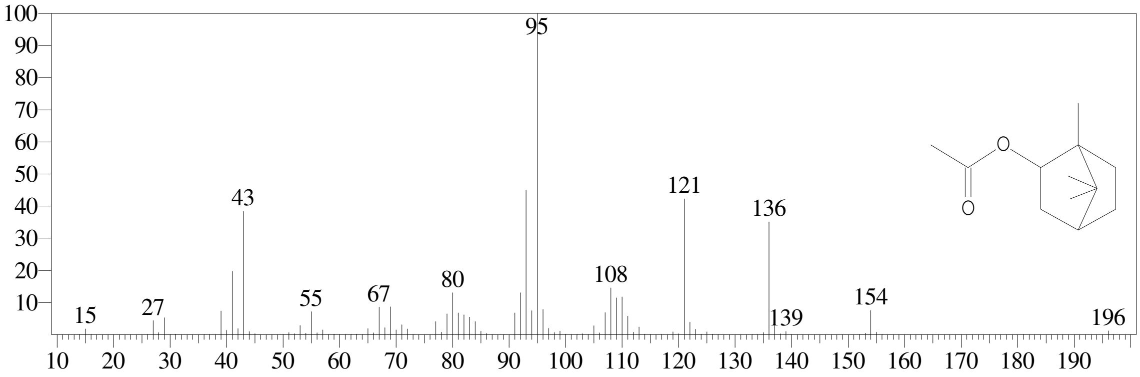

<< Target >>
Line#:12   R.Time:20.483(Scan#:2159)  MassPeaks:6
RawMode:Averaged 20.475-20.492(2158-2160) BasePeak:95.10(3486)
BG Mode:None  Group 1 - Event 1  Scan

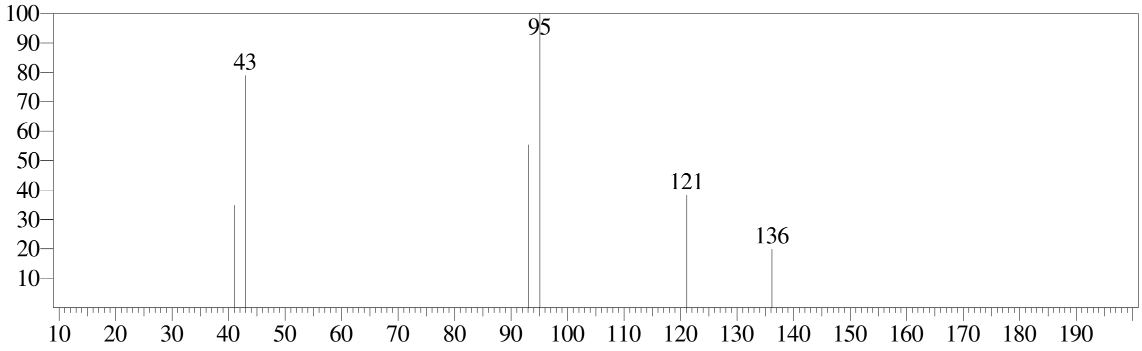


Hit#:3  Entry:11520  Library:NIST23-1.lib
SI:69  Formula:C6H7N3O  CAS:45810-14-8  MolWeight:137  RetIndex:1505
CompName:5-Aminopyrimidine, N-acetyl- $$ Acetamide, N-5-pyrimidinyl- $$ Pyrimidine, 5-amino-, Ac derivative $$

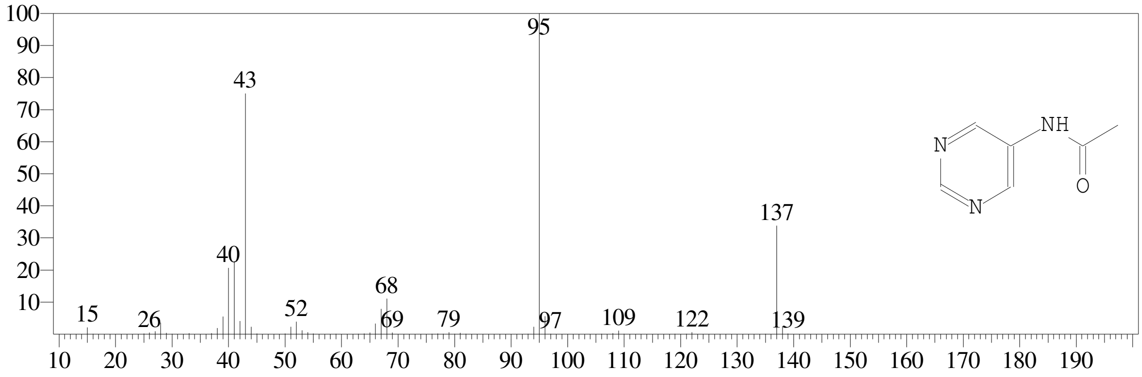


Hit#:4  Entry:5286  Library:NIST23s.lib
SI:69  Formula:C3H7Br  CAS:75-26-3  MolWeight:122  RetIndex:574
CompName:Propane, 2-bromo- $$ Isopropyl bromide $$ 2-Bromopropane $$ iso-C3H7Br $$ sec-Propyl bromide $$ UN

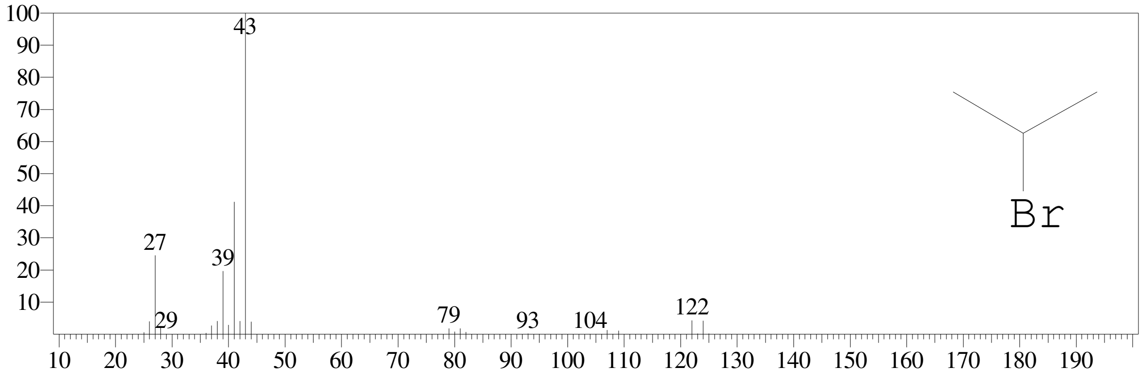

<< Target >>
Line#:12   R.Time:20.483(Scan#:2159)  MassPeaks:6
RawMode:Averaged 20.475-20.492(2158-2160) BasePeak:95.10(3486)
BG Mode:None  Group 1 - Event 1  Scan

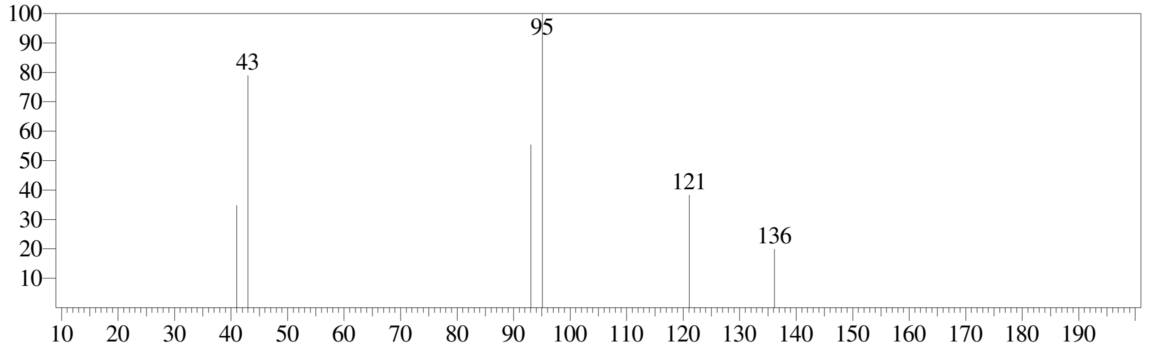


Hit#:5  Entry:27267  Library:NIST23-1.lib
SI:68  Formula:C10H12O2  CAS:59204-74-9  MolWeight:164  RetIndex:1293
CompName:Egomaketone $$ 3-Penten-1-one, 1-(3-furanyl)-4-methyl- $$ 3-Penten-1-one, 1-(3-furyl)-4-methyl- $$

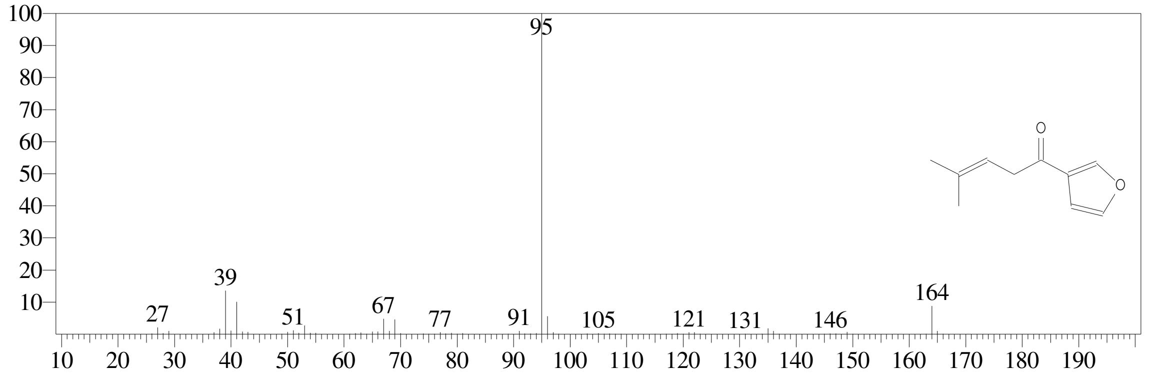

<< Target >>
Line#:13  R.Time:24.325(Scan#:2620)  MassPeaks:20
RawMode:Averaged 24.317-24.333(2619-2621) BasePeak:105.10(9754)
BG Mode:None  Group 1 - Event 1  Scan

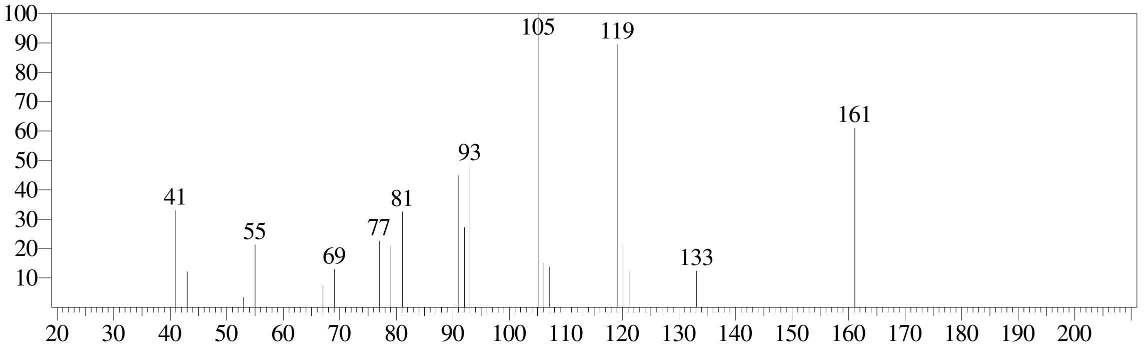


Hit#:1  Entry:24948  Library:NIST23s.lib
SI:86  Formula:C15H24  CAS:17699-14-8  MolWeight:204  RetIndex:1381
CompName:.alpha.-Cubebene $$ 1H-Cyclopenta[1,3]cyclopropa[1,2]benzene, 3a,3b,4,5,6,7-hexahydro-3,7-dimethyl-4-(1

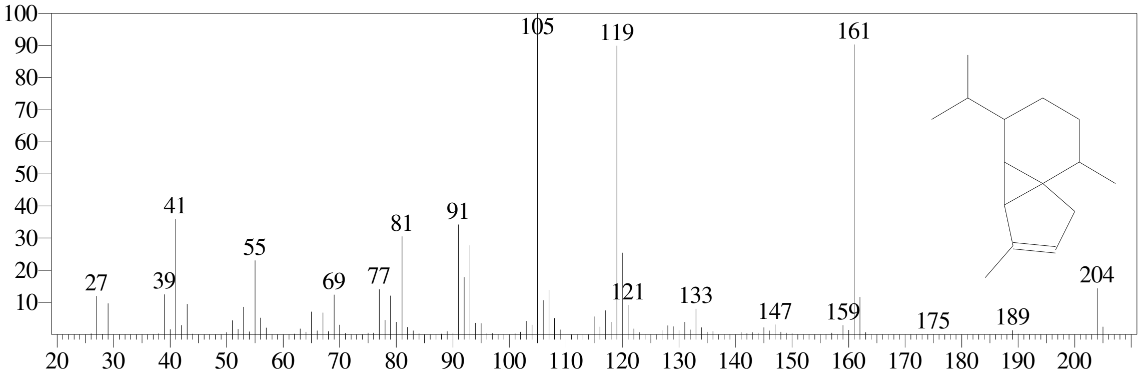


Hit#:2  Entry:62910  Library:NIST23-1.lib
SI:85  Formula:C15H24  CAS:3856-25-5  MolWeight:204  RetIndex:1407
CompName:Copaene $$ Tricyclo[4.4.0.02,7]dec-3-ene, 1,3-dimethyl-8-(1-methylethyl)-, stereoisomer $$ Tricyclo[4.4.0.0

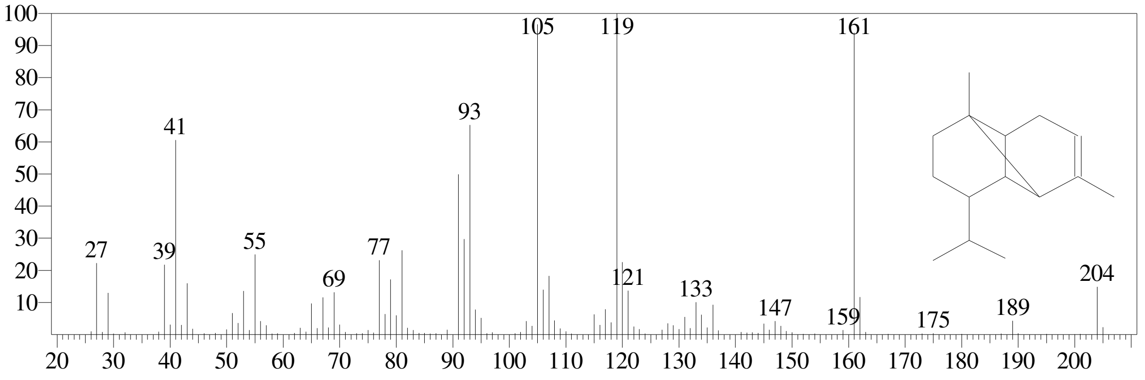

<< Target >>
Line#:13   R.Time:24.325(Scan#:2620)  MassPeaks:20
RawMode:Averaged 24.317-24.333(2619-2621) BasePeak:105.10(9754)
BG Mode:None  Group 1 - Event 1  Scan

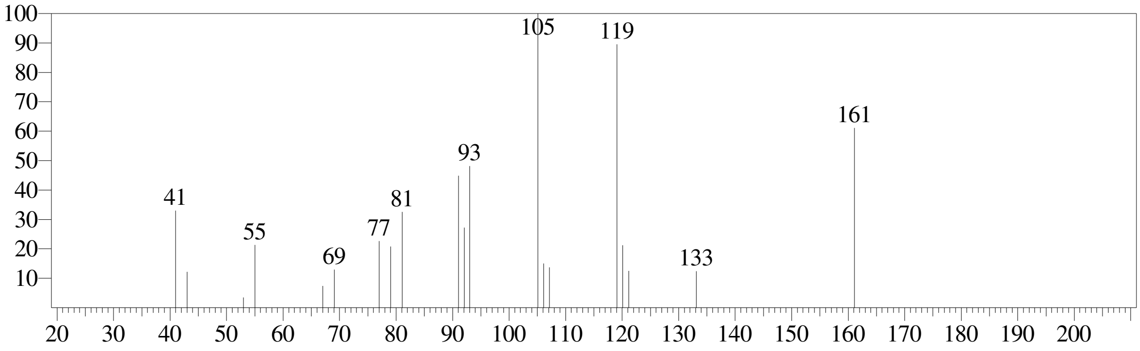


Hit#:3  Entry:25087  Library:NIST23s.lib
SI:85  Formula:C15H24  CAS:3856-25-5  MolWeight:204  RetIndex:1407
CompName:Copaene $$ Tricyclo[4.4.0.02,7]dec-3-ene, 1,3-dimethyl-8-(1-methylethyl)-, stereoisomer $$ Tricyclo[4.4.0.0

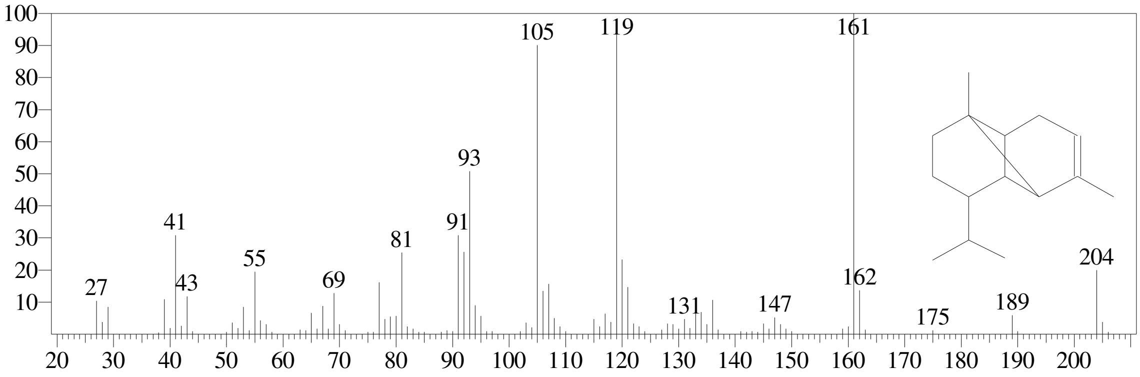


Hit#:4  Entry:25089  Library:NIST23s.lib
SI:84  Formula:C15H24  CAS:3856-25-5  MolWeight:204  RetIndex:1407
CompName:Copaene $$ Tricyclo[4.4.0.02,7]dec-3-ene, 1,3-dimethyl-8-(1-methylethyl)-, stereoisomer $$ Tricyclo[4.4.0.0

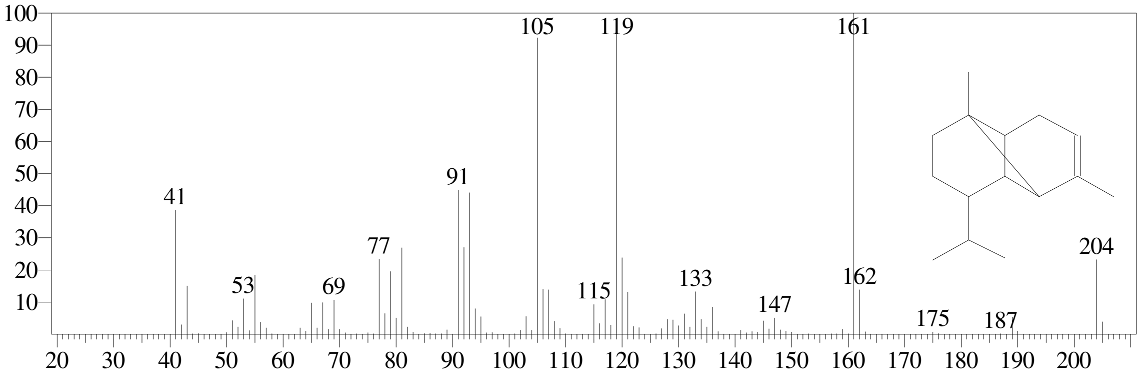

<< Target >>
Line#:13   R.Time:24.325(Scan#:2620)  MassPeaks:20
RawMode:Averaged 24.317-24.333(2619-2621) BasePeak:105.10(9754)
BG Mode:None  Group 1 - Event 1  Scan

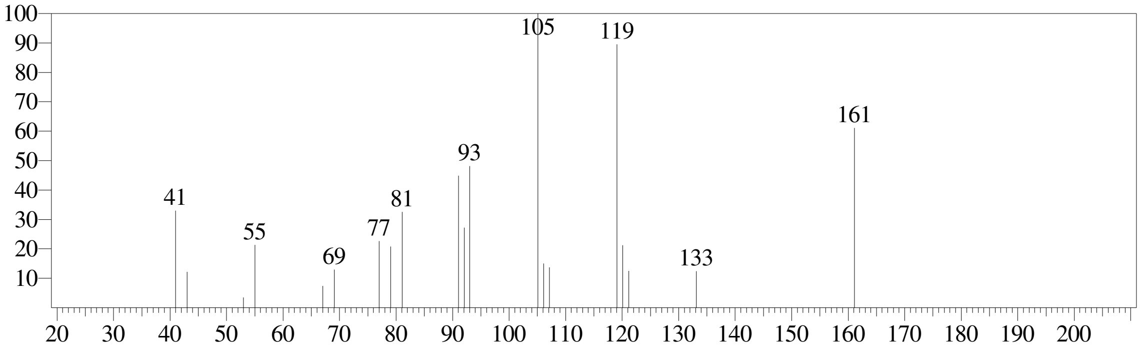


Hit#:5  Entry:25077  Library:NIST23s.lib
SI:84  Formula:C15H24  CAS:17699-14-8  MolWeight:204  RetIndex:1381
CompName:.alpha.-Cubebene $$ 1H-Cyclopenta[1,3]cyclopropa[1,2]benzene, 3a,3b,4,5,6,7-hexahydro-3,7-dimethyl-4-(1

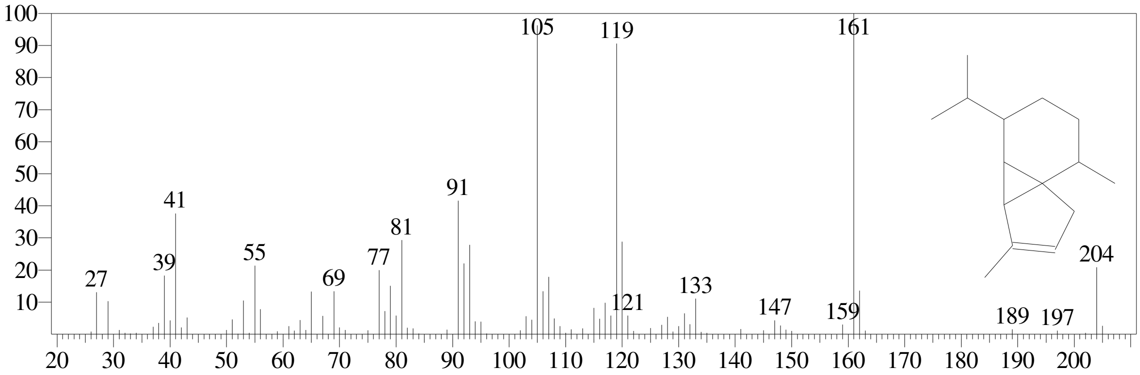

<< Target >>
Line#:14 R.Time:26.150(Scan#:2839) MassPeaks:37
RawMode:Averaged 26.142-26.158(2838-2840) BasePeak:41.00(13624)
BG Mode:None Group 1 - Event 1 Scan

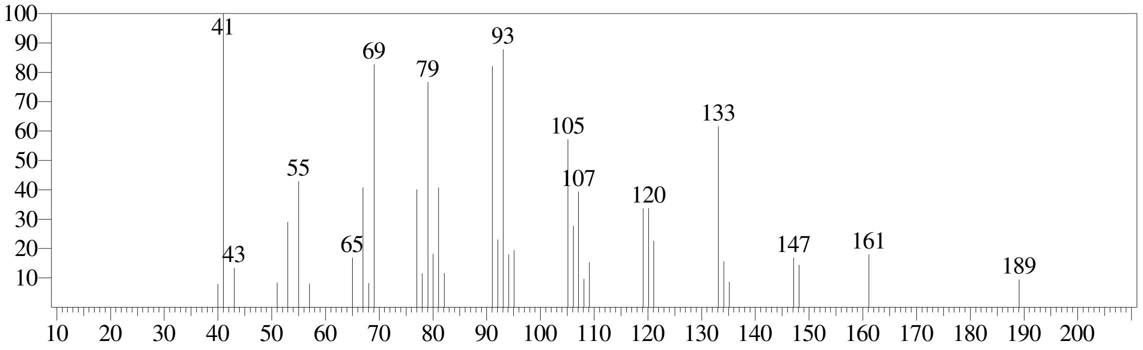


Hit#:1 Entry:24803 Library:NIST23s.lib
SI:94 Formula:C15H24 CAS:118-65-0 MolWeight:204 RetIndex:1448
CompName:Bicyclo[7.2.0]undec-4-ene, 4,11,11-trimethyl-8-methylene-,[1R-(1R*,4Z,9S*)]- $$ Isocaryophyllene $$ 4,11,

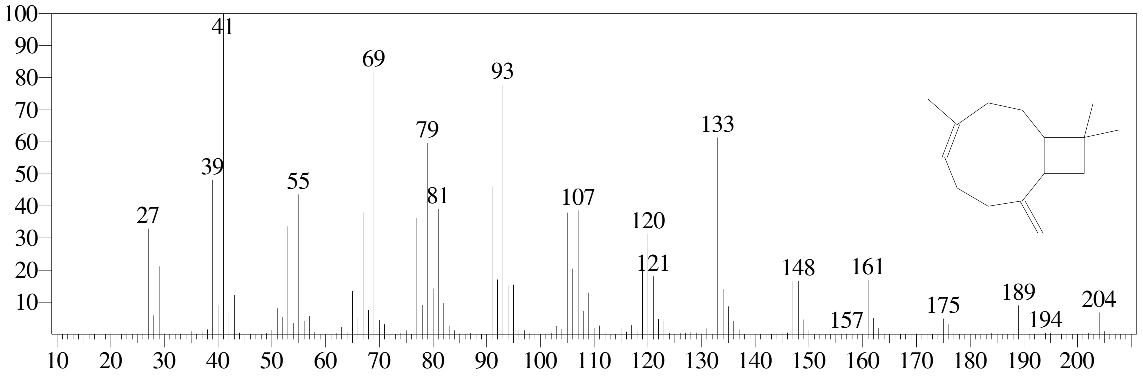


Hit#:2 Entry:24827 Library:NIST23s.lib
SI:94 Formula:C15H24 CAS:87-44-5 MolWeight:204 RetIndex:1448
CompName:Caryophyllene $$ Bicyclo[7.2.0]undec-4-ene, 4,11,11-trimethyl-8-methylene-, [1R-(1R*,4E,9S*)]- $$ Bicycl

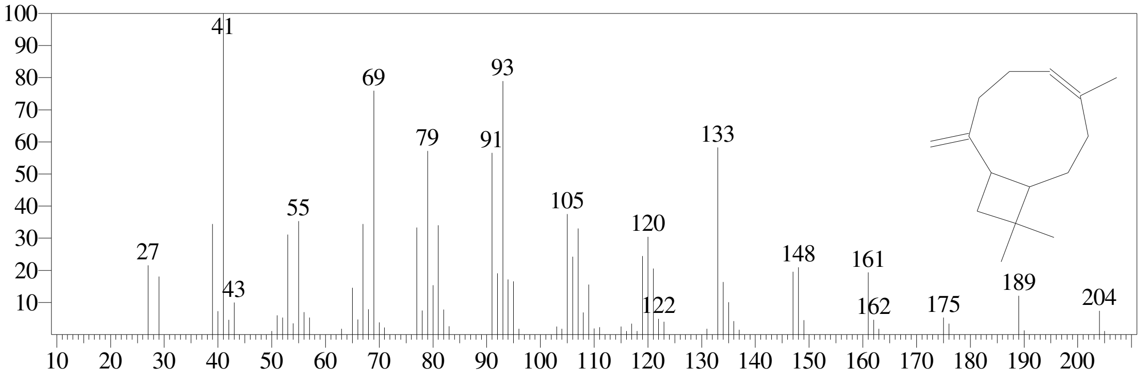

<< Target >>
Line#:14   R.Time:26.150(Scan#:2839)  MassPeaks:37
RawMode:Averaged 26.142-26.158(2838-2840) BasePeak:41.00(13624)
BG Mode:None  Group 1 - Event 1  Scan

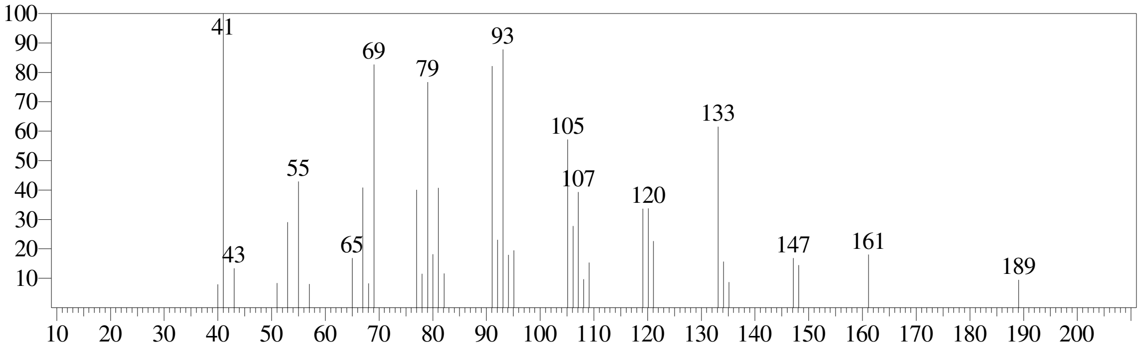


Hit#:3  Entry:24804  Library:NIST23s.lib
SI:93  Formula:C15H24  CAS:87-44-5  MolWeight:204  RetIndex:1448
CompName:Caryophyllene $$ Bicyclo[7.2.0]undec-4-ene, 4,11,11-trimethyl-8-methylene-, [1R-(1R*,4E,9S*)]- $$ Bicycl

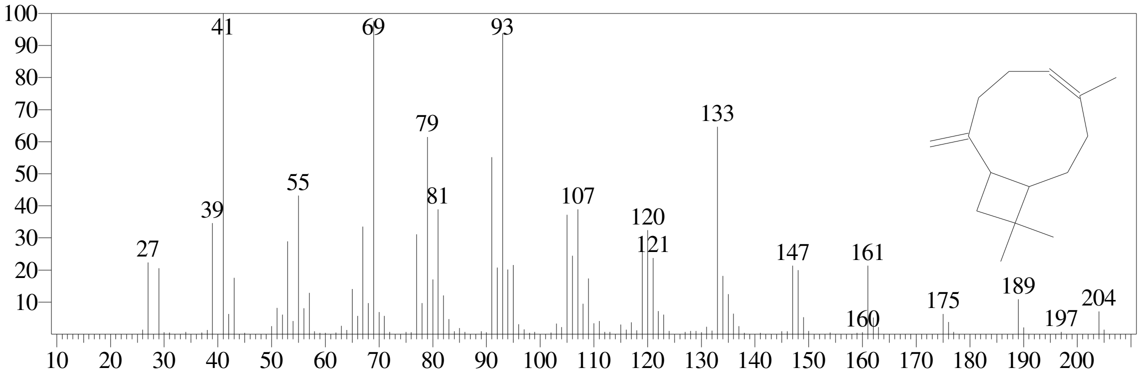


Hit#:4  Entry:62803  Library:NIST23-1.lib
SI:92  Formula:C15H24  CAS:13877-93-5  MolWeight:204  RetIndex:1448
CompName:Bicyclo[7.2.0]undec-4-ene, 4,11,11-trimethyl-8-methylene- $$ Bicyclo[7.2.0]undec-4-ene, 4,11,11-trimethyl-

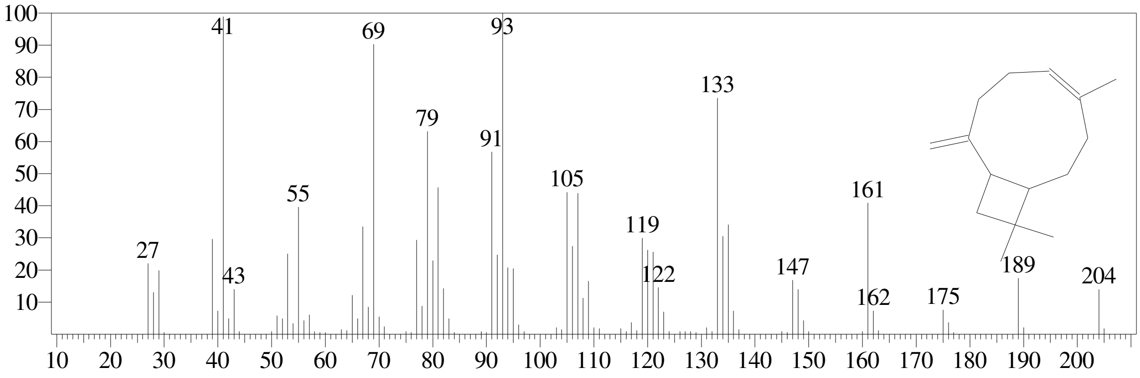

<< Target >>
Line#:14   R.Time:26.150(Scan#:2839)  MassPeaks:37
RawMode:Averaged 26.142-26.158(2838-2840) BasePeak:41.00(13624)
BG Mode:None  Group 1 - Event 1  Scan

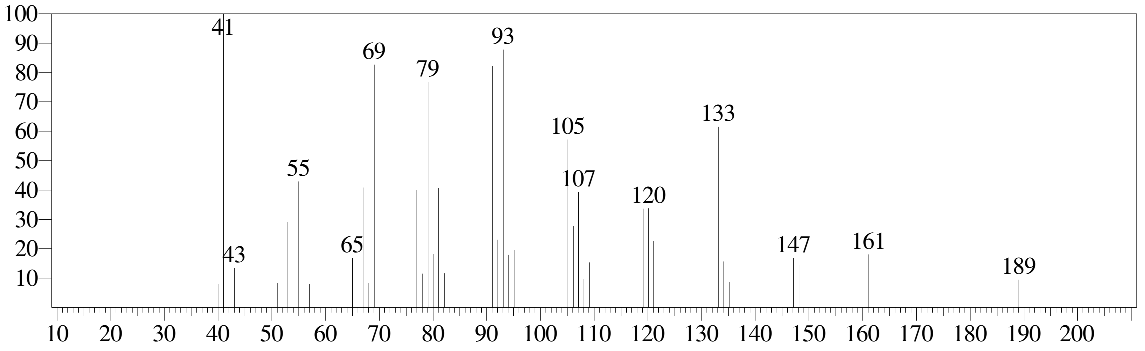


Hit#:5  Entry:62827  Library:NIST23-1.lib
SI:92  Formula:C15H24  CAS:87-44-5  MolWeight:204  RetIndex:1448
CompName:Caryophyllene $$ Bicyclo[7.2.0]undec-4-ene, 4,11,11-trimethyl-8-methylene-, [1R-(1R*,4E,9S*)]- $$ Bicycl

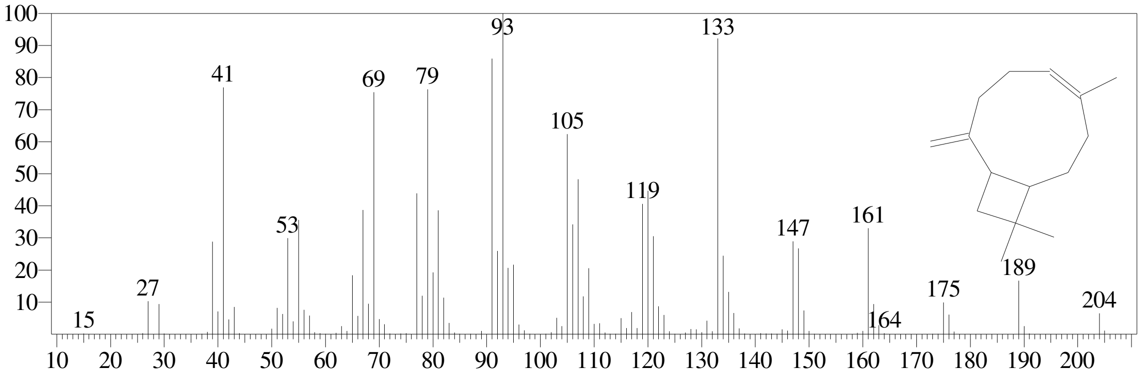

<< Target >>
Line#:15 R.Time:30.375(Scan#:3346) MassPeaks:9
RawMode:Averaged 30.367-30.383(3345-3347) BasePeak:119.10(3840)
BG Mode:None Group 1 - Event 1 Scan

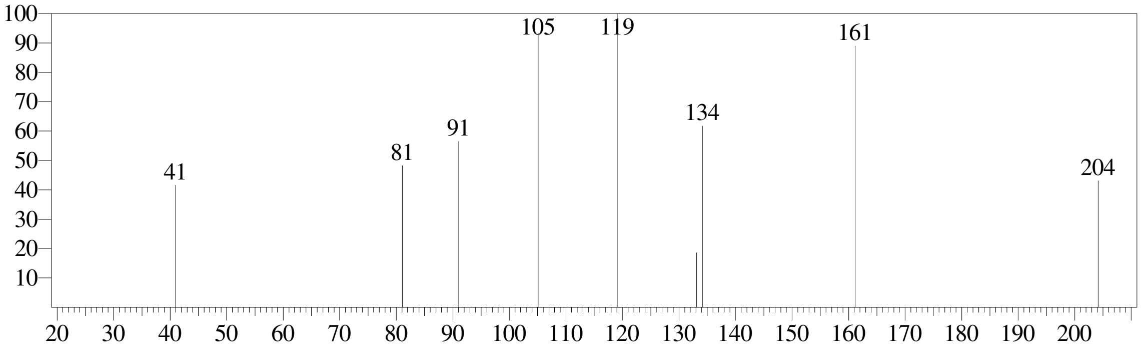


Hit#:1 Entry:25084 Library:NIST23s.lib
SI:72 Formula:C15H24 CAS:157374-44-2 MolWeight:204 RetIndex:1449
CompName:cis-muurola-3,5-diene

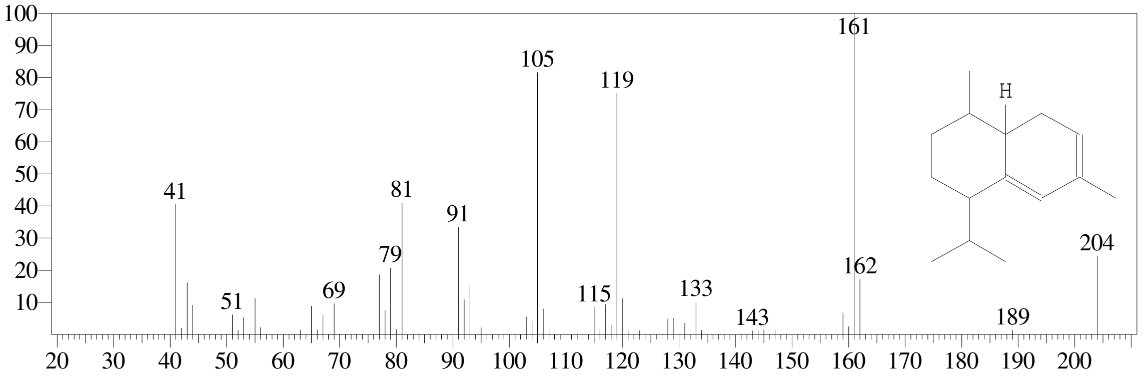


Hit#:2 Entry:63011 Library:NIST23-1.lib
SI:71 Formula:C15H24 CAS:483-76-1 MolWeight:204 RetIndex:1526
CompName:Naphthalene, 1,2,3,5,6,8a-hexahydro-4,7-dimethyl-1-(1-methylethyl)-, (1S-cis)- $$ Cadina-1(10),4-diene $$ .

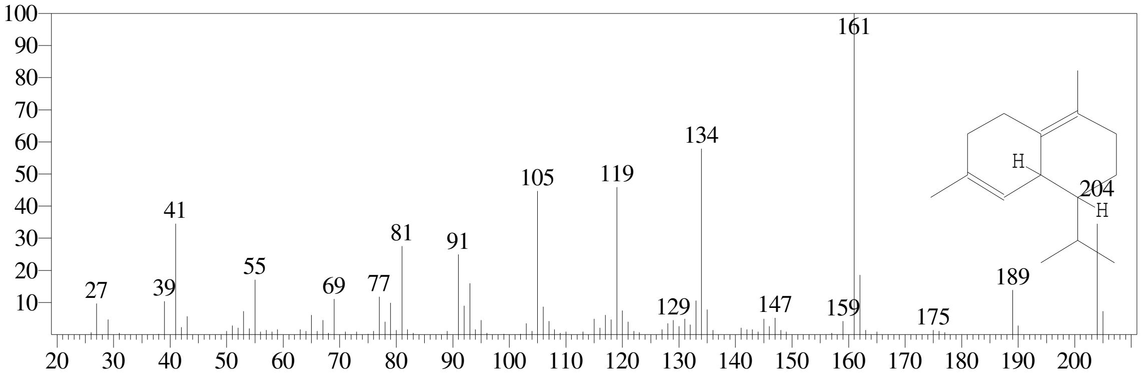

<< Target >>
Line#:15   R.Time:30.375(Scan#:3346)  MassPeaks:9
RawMode:Averaged 30.367-30.383(3345-3347) BasePeak:119.10(3840)
BG Mode:None  Group 1 - Event 1  Scan

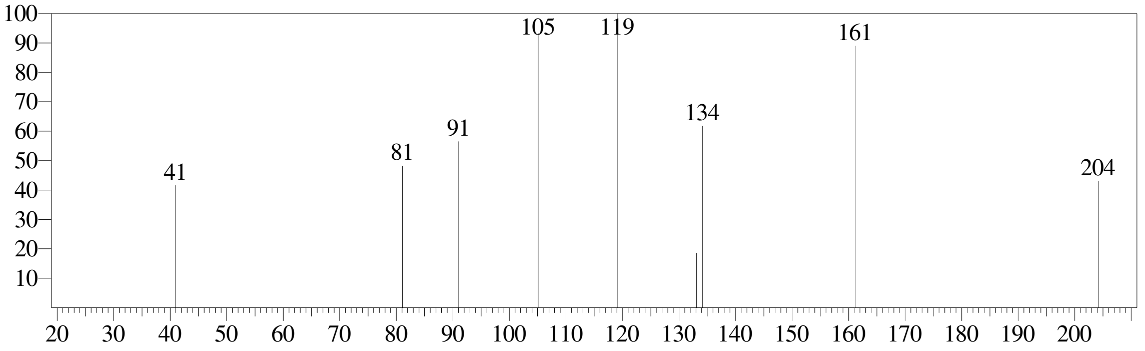


Hit#:3  Entry:62987  Library:NIST23-1.lib
SI:71  Formula:C15H24  CAS:267665-20-3  MolWeight:204  RetIndex:1465
CompName:(1S,4S,4aS)-1-Isopropyl-4,7-dimethyl-1,2,3,4,4a,5-hexahydronaphthalene $$ Naphthalene, 1,2,3,4,4a,5-hexah

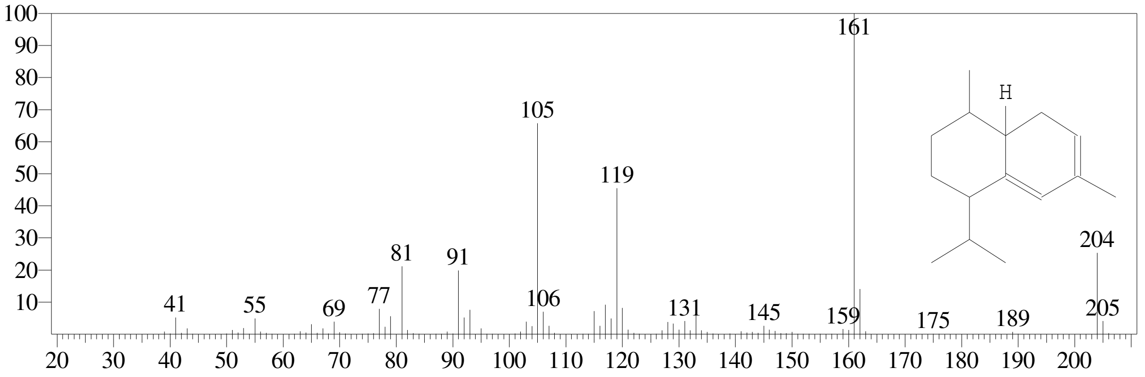


Hit#:4  Entry:62988  Library:NIST23-1.lib
SI:70  Formula:C15H24  CAS:17699-14-8  MolWeight:204  RetIndex:1381
CompName:.alpha.-Cubebene $$ 1H-Cyclopenta[1,3]cyclopropa[1,2]benzene, 3a,3b,4,5,6,7-hexahydro-3,7-dimethyl-4-(1-

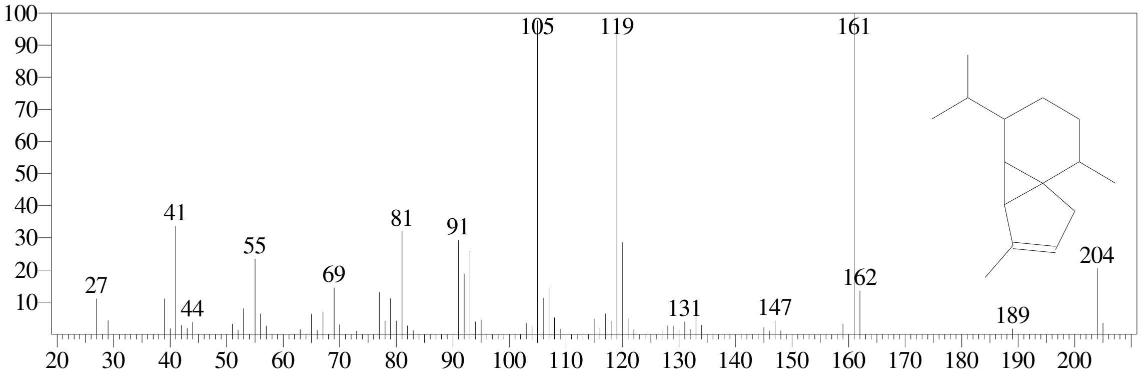

<< Target >>
Line#:15 R.Time:30.375(Scan#:3346) MassPeaks:9
RawMode:Averaged 30.367-30.383(3345-3347) BasePeak:119.10(3840)
BG Mode:None Group 1 - Event 1 Scan

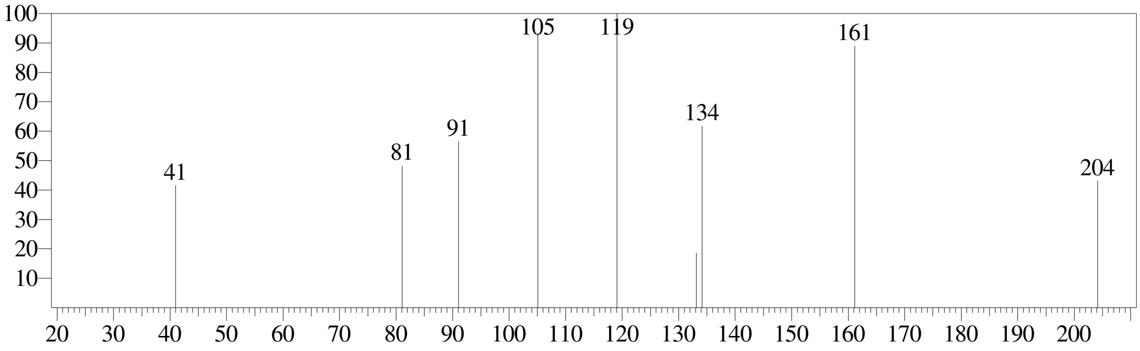


Hit#:5 Entry:25098 Library:NIST23s.lib
SI:70 Formula:C15H24 CAS:483-76-1 MolWeight:204 RetIndex:1526
CompName:Naphthalene, 1,2,3,5,6,8a-hexahydro-4,7-dimethyl-1-(1-methylethyl)-, (1S-cis)- $$ Cadina-1(10),4-diene $$ .

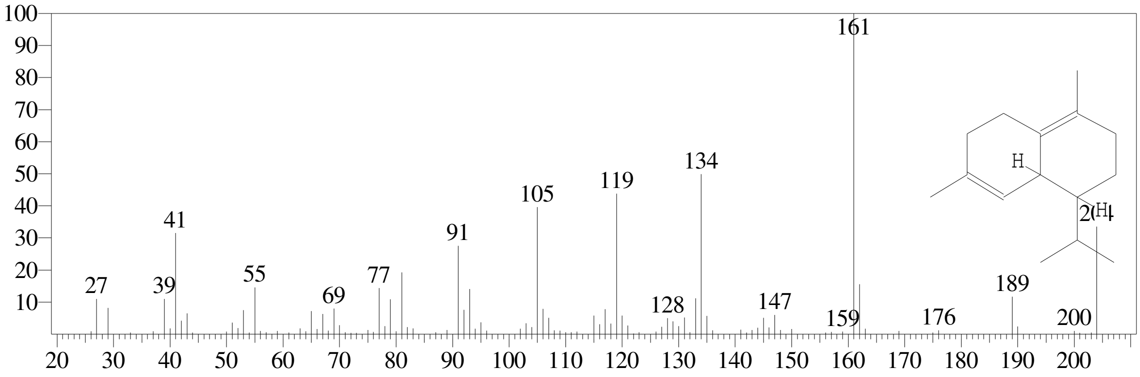

Supplement: Supplementary file 1 [file plants-15-01406-s001.zip › EN bb.pdf]
