# Supplementary material for: Unveiling Chemical Profile and Insecticidal Potential of Essential Oils from Leaves of Seven Eugenia L. Species (Myrtaceae)
Source: Plants (Basel). 2026 May 5;15(9):1406. doi: 10.3390/plants15091406 (PMC13165059; doi:10.3390/plants15091406)

UNIVERSIDADE FERAL RURAL DO RIO DE JANEIRO
INSTITUTO DE QUÍMICA

CGMS

Analyzed by: Cristiane Cardoso
Analyzed: 17/6/2025
Solicitante:Douglas
Sample Name: EP
Injection Volume: 1,0 uL   Solvente: Diclorometano
Data File: C:\GCMSsolution\Data\Project1\Douglas\2025\MLLENA\170625\EP.qgd
Method File: C:\GCMSsolution\Data\Project1\Douglas\Essencial Adams-Inj.qgm
EQUIPAMENTO: Modelo:GCMS-QP2010 Plus (Shimadzu)
Coluna: VF-5m (30X0.25X0.25)

Chromatogram EP C:\GCMSsolution\Data\Project1\Douglas\2025\MLLENA\170625\EP.qgd

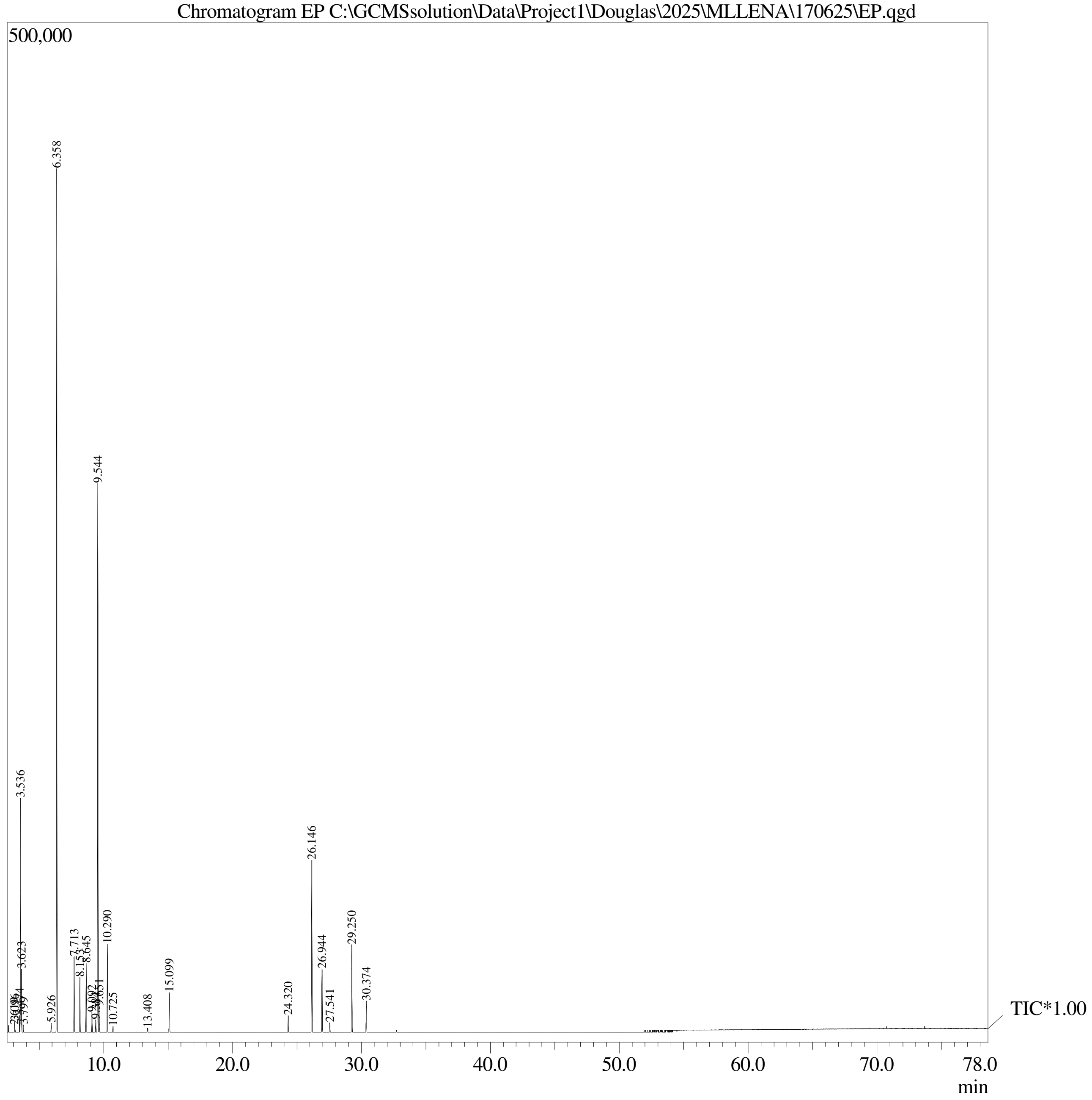

Library

<< Target >>
Line#:1   R.Time:2.617(Scan#:15) MassPeaks:2
RawMode:Averaged 2.608-2.625(14-16) BasePeak:59.00(1999)
BG Mode:None Group 1 - Event 1 Scan

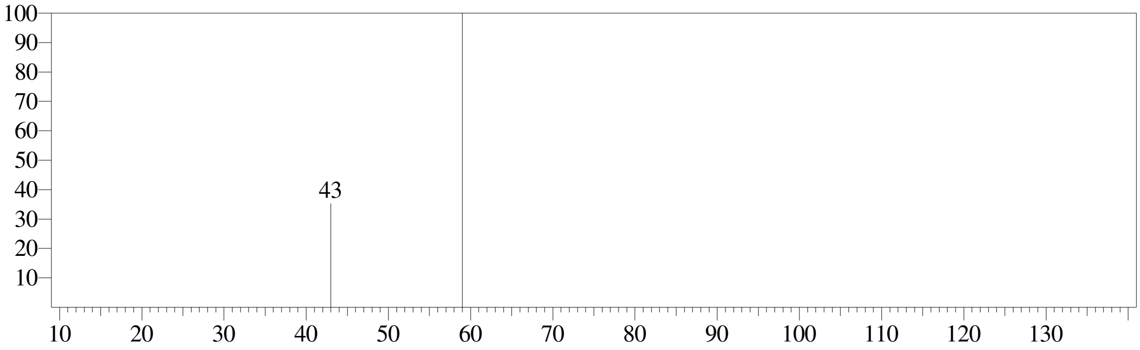

Hit#:1 Entry:5543 Library:NIST23-1.lib
SI:93 Formula:C5H10O3 CAS:2110-78-3 MolWeight:118 RetIndex:769
CompName:Propanoic acid, 2-hydroxy-2-methyl-, methyl ester $$ Methyl .alpha.-hydroxyisobutyrate $$ Lactic acid, 2-me

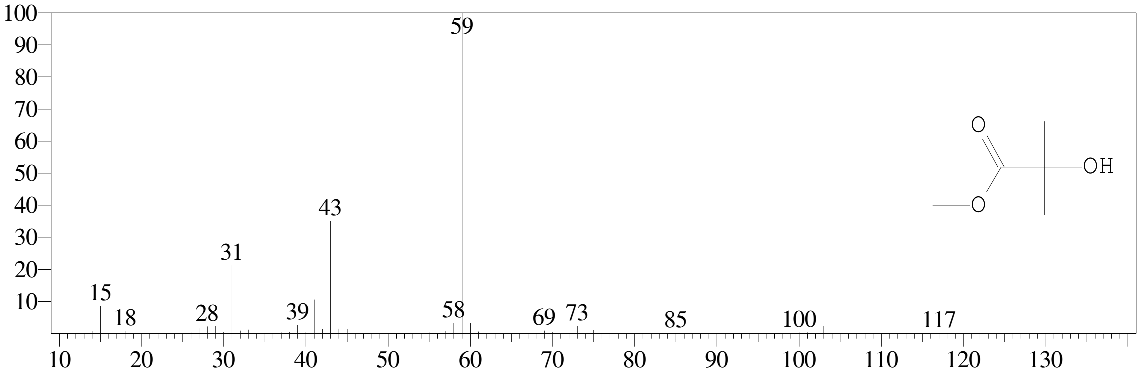

Hit#:2 Entry:9783 Library:NIST23-1.lib
SI:93 Formula:C6H12O3 CAS:70657-70-4 MolWeight:132 RetIndex:880
CompName:2-Methoxypropyl acetate $$ 1-Propanol, 2-methoxy-, 1-acetate $$ 1-Propanol, 2-methoxy-, acetate $$ 2-Meth

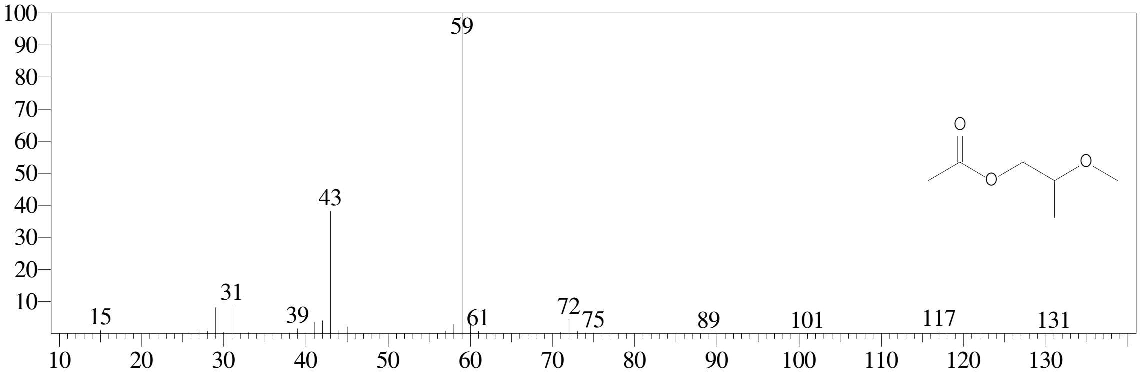

<< Target >>
Line#:1   R.Time:2.617(Scan#:15) MassPeaks:2
RawMode:Averaged 2.608-2.625(14-16) BasePeak:59.00(1999)
BG Mode:None Group 1 - Event 1 Scan

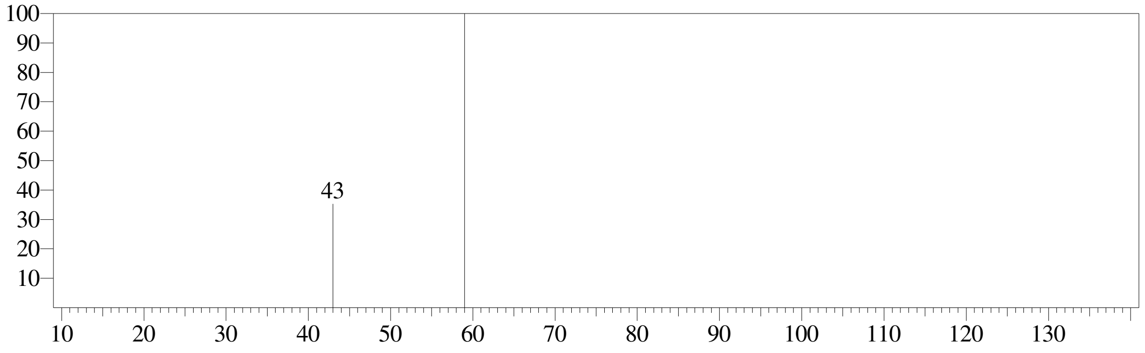

Hit#:3 Entry:4802 Library:NIST23s.lib
SI:93 Formula:C5H10O3 CAS:2110-78-3 MolWeight:118 RetIndex:769
CompName:Propanoic acid, 2-hydroxy-2-methyl-, methyl ester $$ Methyl .alpha.-hydroxyisobutyrate $$ Lactic acid, 2-m[illegible]

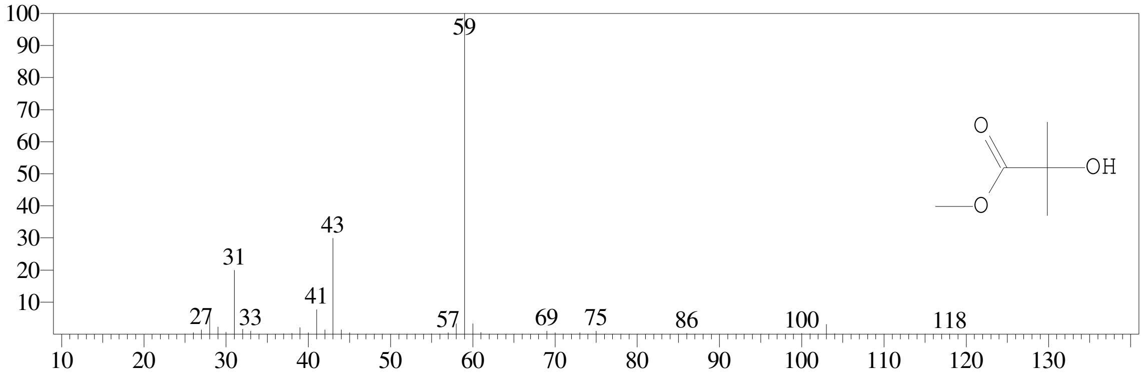

Hit#:4 Entry:2534 Library:NIST23s.lib
SI:93 Formula:C5H10O2 CAS:115-22-0 MolWeight:102 RetIndex:737
CompName:3-Hydroxy-3-methyl-2-butanone $$ 2-Butanone, 3-hydroxy-3-methyl- $$ Dimethylacetylcarbinol $$ 3-Hydr[illegible]

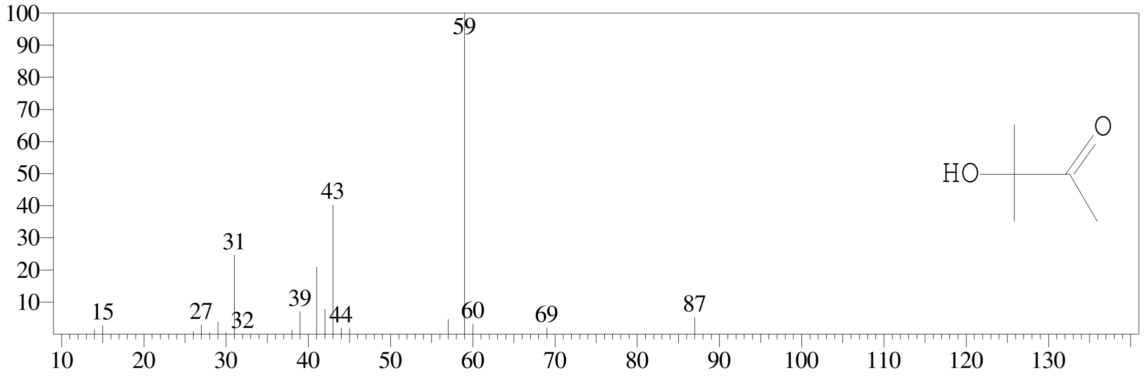

<< Target >>
Line#:1   R.Time:2.617(Scan#:15) MassPeaks:2
RawMode:Averaged 2.608-2.625(14-16) BasePeak:59.00(1999)
BG Mode:None Group 1 - Event 1 Scan

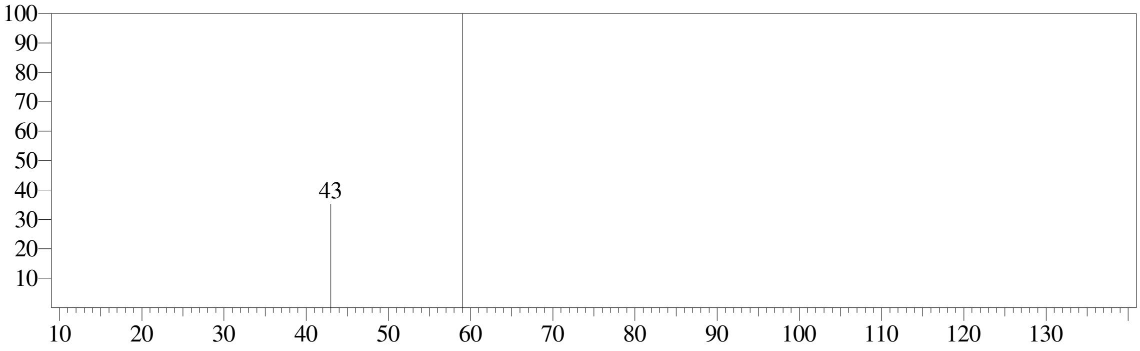

Hit#:5 Entry:7391 Library:NIST23s.lib
SI:93 Formula:C6H12O3 CAS:80-55-7 MolWeight:132 RetIndex:843
CompName:Propanoic acid, 2-hydroxy-2-methyl-, ethyl ester $$ Lactic acid, 2-methyl-, ethyl ester $$ Ethyl .alpha.-hydro:

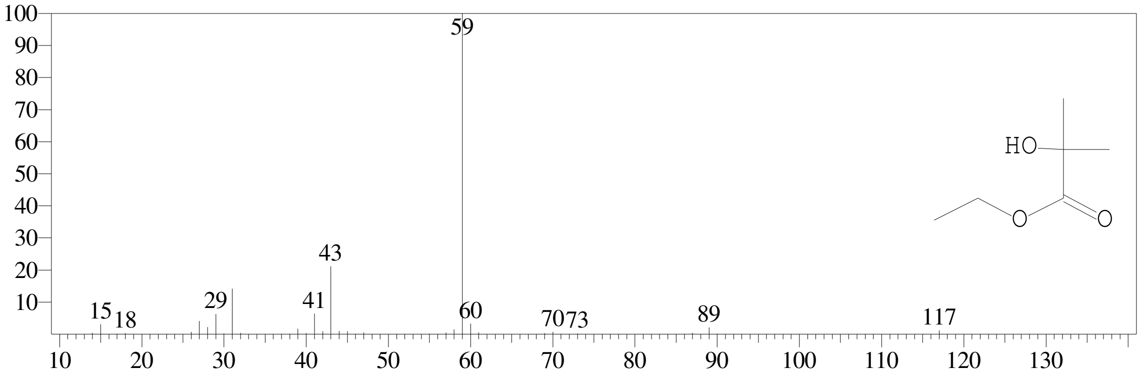

<< Target >>
Line#:2   R.Time:3.100(Scan#:73) MassPeaks:3
RawMode:Averaged 3.092-3.108(72-74) BasePeak:43.00(2292)
BG Mode:None Group 1 - Event 1 Scan

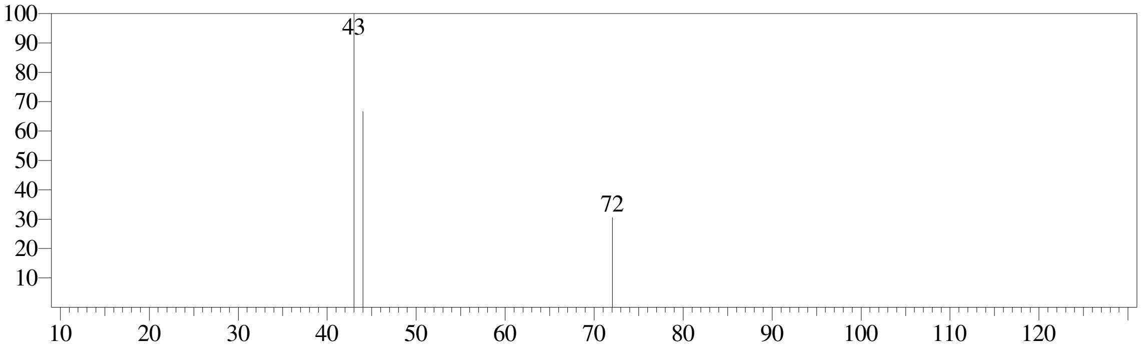

Hit#:1 Entry:437 Library:NIST23s.lib
SI:91 Formula:C4H8O CAS:109-92-2 MolWeight:72 RetIndex:481
CompName:Ethene, ethoxy- $$ Vinamar $$ Ether, ethyl vinyl $$ Ethoxyethene $$ Ethoxyethylene $$ Ethyl vinyl ether $$

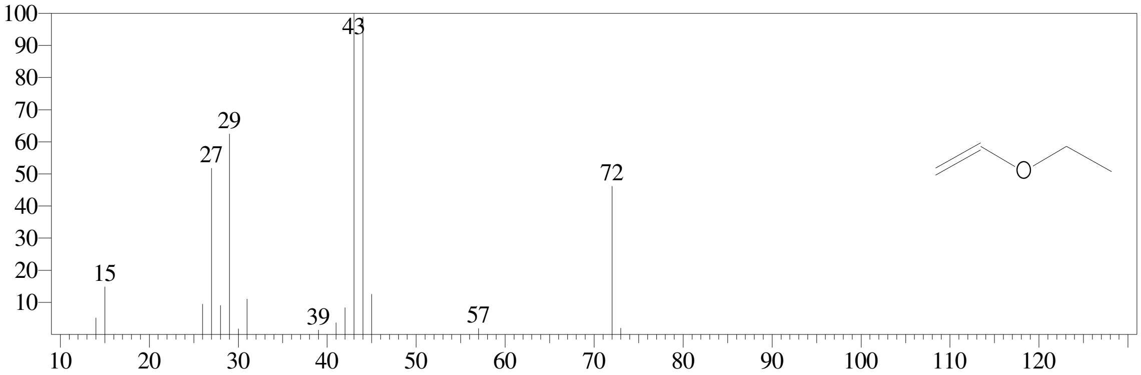

Hit#:2 Entry:21730 Library:NIST23-1.lib
SI:90 Formula:C4H4N4O3 CAS:0-00-0 MolWeight:156 RetIndex:1987
CompName:Pyrimidine-2,4(1H,3H)-dione, 5-amino-6-nitroso- $$ 5-Amino-6-nitroso-2,4(1H,3H)-pyrimidinedione # $$

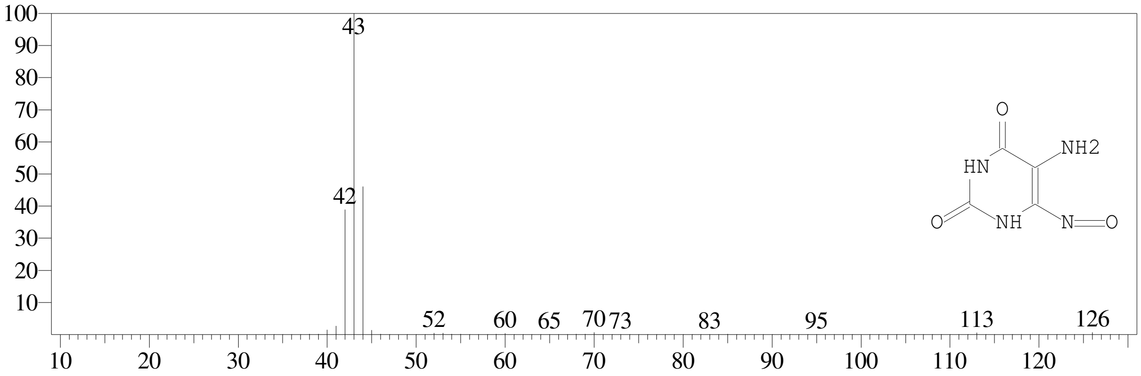

<< Target >>
Line#:2   R.Time:3.100(Scan#:73) MassPeaks:3
RawMode:Averaged 3.092-3.108(72-74) BasePeak:43.00(2292)
BG Mode:None Group 1 - Event 1 Scan

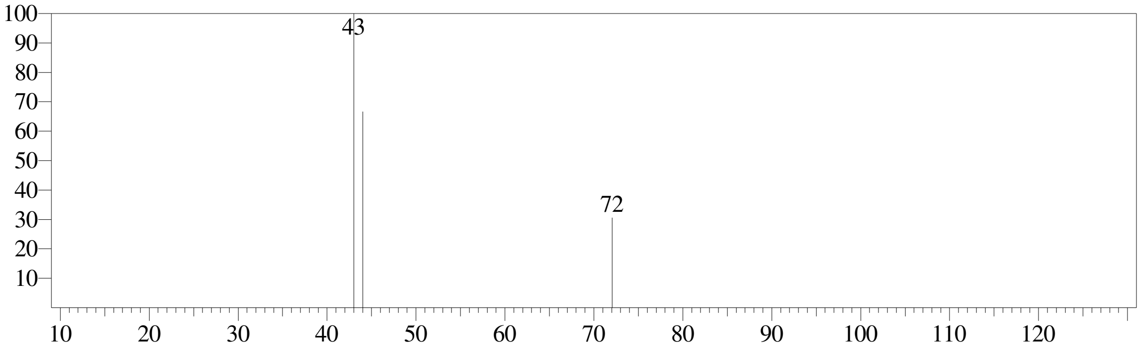

Hit#:3 Entry:334 Library:NIST23-1.lib
SI:90 Formula:C4H8O CAS:109-92-2 MolWeight:72 RetIndex:481
CompName:Ethene, ethoxy- $$ Vinamar $$ Ether, ethyl vinyl $$ Ethoxyethene $$ Ethoxyethylene $$ Ethyl vinyl ether $$

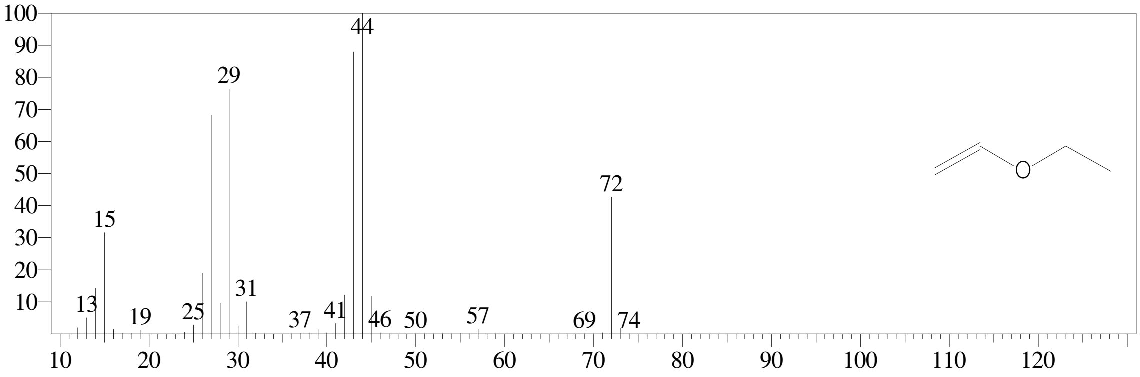

Hit#:4 Entry:310 Library:NIST23-1.lib
SI:90 Formula:C3H4O2 CAS:692-45-5 MolWeight:72 RetIndex:471
CompName:Formic acid, ethenyl ester $$ Formic acid, vinyl ester $$ Vinyl formate $$ Vinyl methanoate $$ Vinylester ky

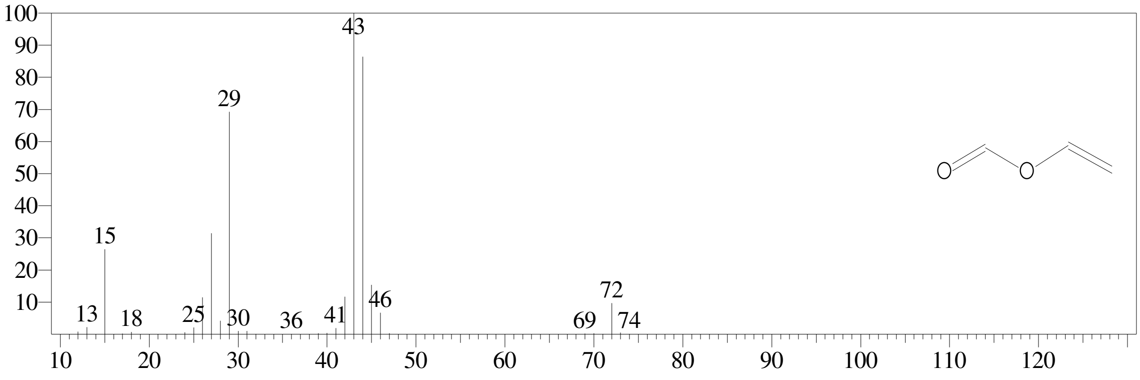

<< Target >>
Line#:2 R.Time:3.100(Scan#:73) MassPeaks:3
RawMode:Averaged 3.092-3.108(72-74) BasePeak:43.00(2292)
BG Mode:None Group 1 - Event 1 Scan

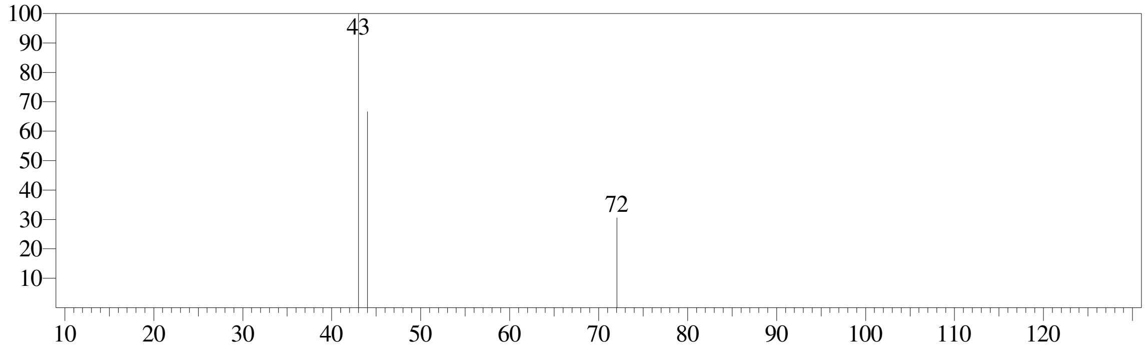

Hit#:5 Entry:456 Library:NIST23s.lib
SI:88 Formula:C4H8O CAS:109-92-2 MolWeight:72 RetIndex:481
CompName:Ethene, ethoxy- $$ Vinamar $$ Ether, ethyl vinyl $$ Ethoxyethene $$ Ethoxyethylene $$ Ethyl vinyl ether $$

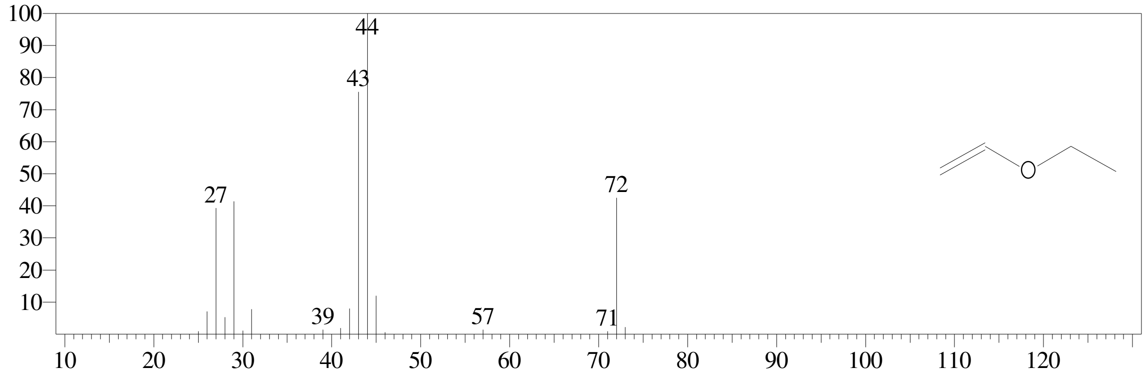

<< Target >>
Line#:3 R.Time:3.475(Scan#:118) MassPeaks:3
RawMode:Averaged 3.467-3.483(117-119) BasePeak:59.00(4215)
BG Mode:None Group 1 - Event 1 Scan

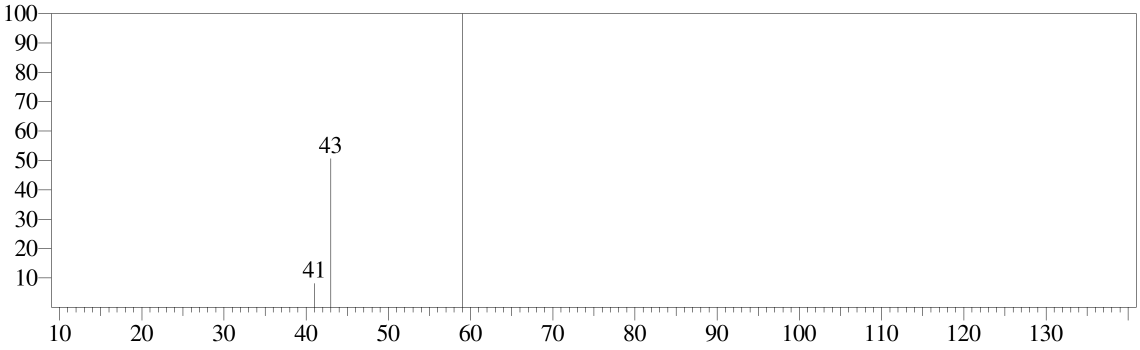

Hit#:1 Entry:2213 Library:NIST23-1.lib
SI:91 Formula:C6H12O CAS:624-97-5 MolWeight:100 RetIndex:702
CompName:4-Pentene-2-ol, 2-methyl $$ CH2=CHCH2C(CH3)2OH $$ 1-Pentene-4-ol, 4-methyl $$ 4-Penten-2-ol, 2-met

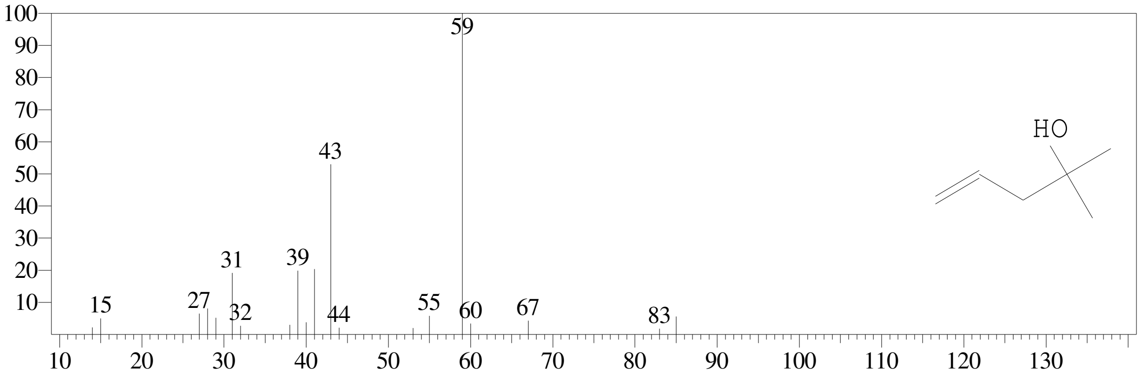

Hit#:2 Entry:5543 Library:NIST23-1.lib
SI:91 Formula:C5H10O3 CAS:2110-78-3 MolWeight:118 RetIndex:769
CompName:Propanoic acid, 2-hydroxy-2-methyl-, methyl ester $$ Methyl .alpha.-hydroxyisobutyrate $$ Lactic acid, 2-me

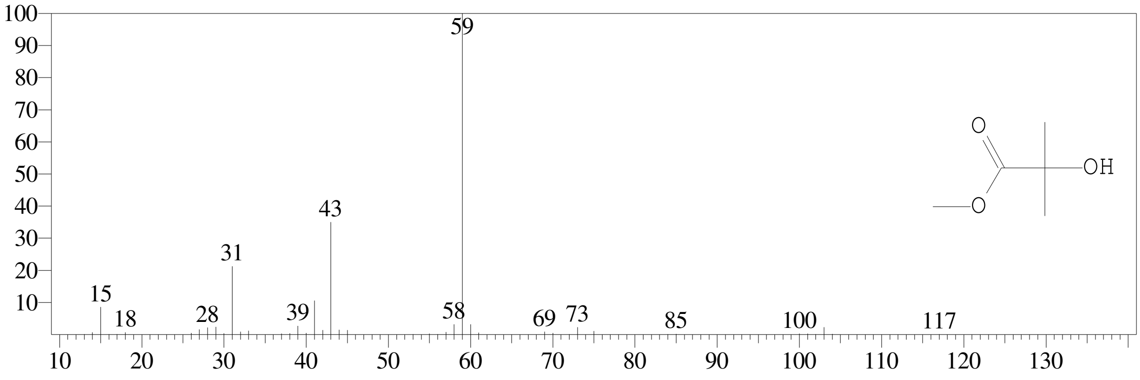

<< Target >>
Line#:3   R.Time:3.475(Scan#:118) MassPeaks:3
RawMode:Averaged 3.467-3.483(117-119) BasePeak:59.00(4215)
BG Mode:None Group 1 - Event 1 Scan

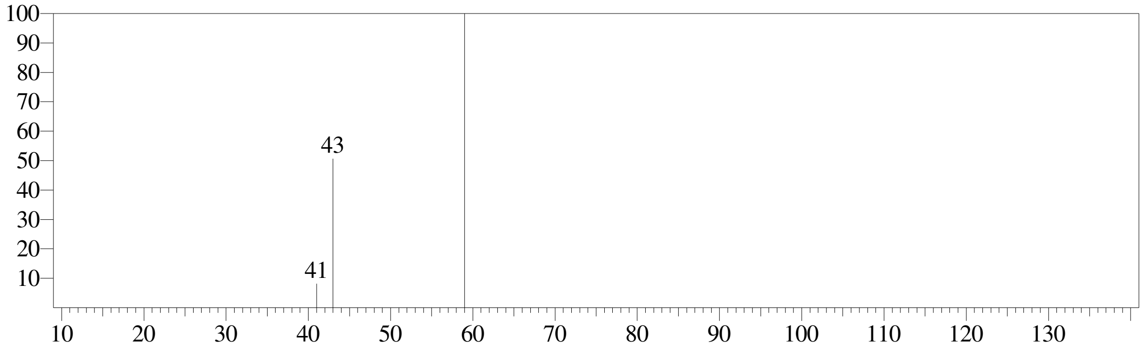

Hit#:3 Entry:4802 Library:NIST23s.lib
SI:90 Formula:C5H10O3 CAS:2110-78-3 MolWeight:118 RetIndex:769
CompName:Propanoic acid, 2-hydroxy-2-methyl-, methyl ester $$ Methyl .alpha.-hydroxyisobutyrate $$ Lactic acid, 2-me

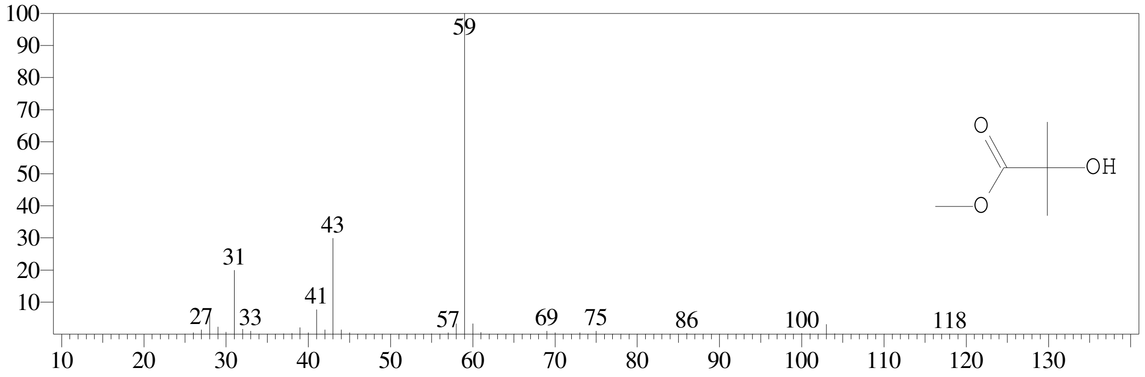

Hit#:4 Entry:9783 Library:NIST23-1.lib
SI:90 Formula:C6H12O3 CAS:70657-70-4 MolWeight:132 RetIndex:880
CompName:2-Methoxypropyl acetate $$ 1-Propanol, 2-methoxy-, 1-acetate $$ 1-Propanol, 2-methoxy-, acetate $$ 2-Meth

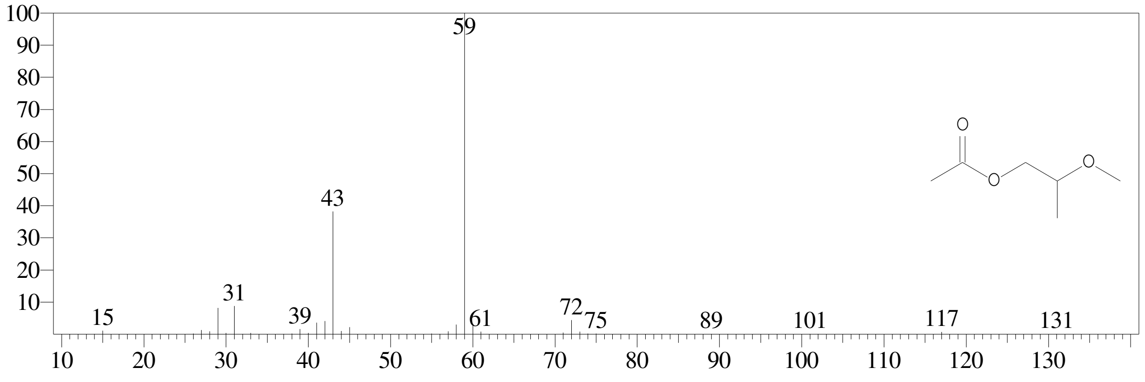

<< Target >>
Line#:3 R.Time:3.475(Scan#:118) MassPeaks:3
RawMode:Averaged 3.467-3.483(117-119) BasePeak:59.00(4215)
BG Mode:None Group 1 - Event 1 Scan

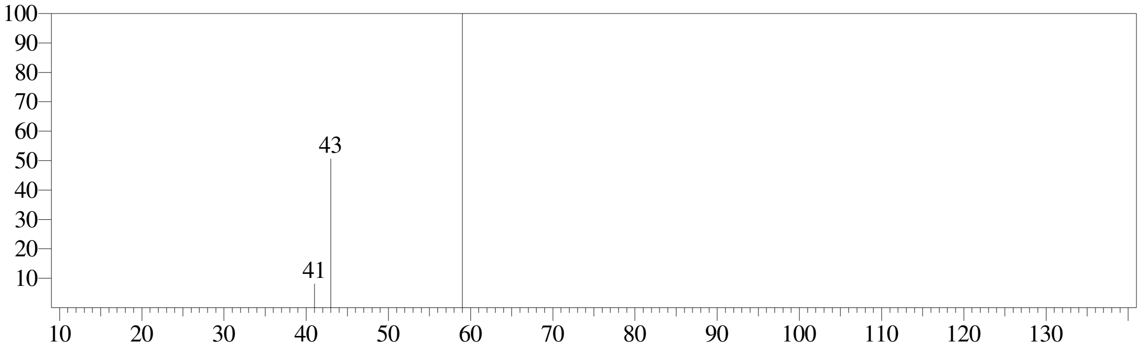

Hit#:5 Entry:2768 Library:NIST23s.lib
SI:90 Formula:C4H8O3 CAS:594-61-6 MolWeight:104 RetIndex:932
CompName:Propanoic acid, 2-hydroxy-2-methyl- $$ 2-Hydroxyisobutyric acid $$ Lactic acid, 2-methyl- $$ .alpha.-Hydrc

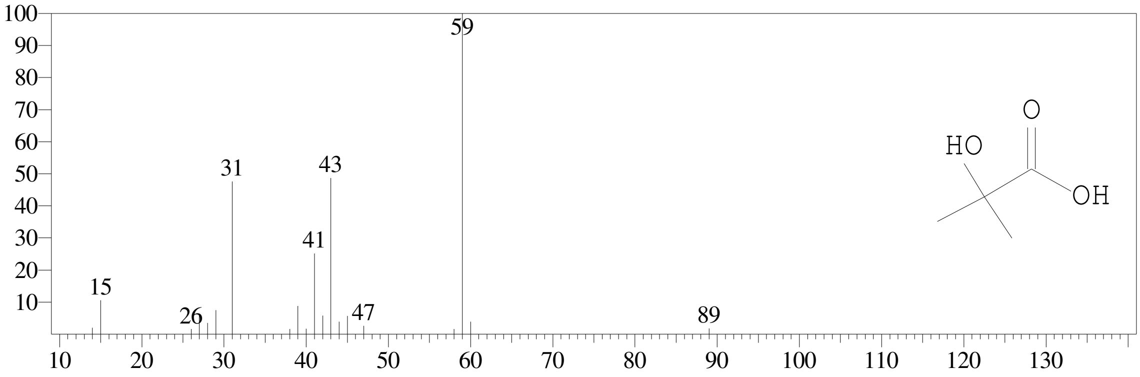

<< Target >>
Line#:4 R.Time:3.533(Scan#:125) MassPeaks:13
RawMode:Averaged 3.525-3.542(124-126) BasePeak:45.00(60902)
BG Mode:None Group 1 - Event 1 Scan

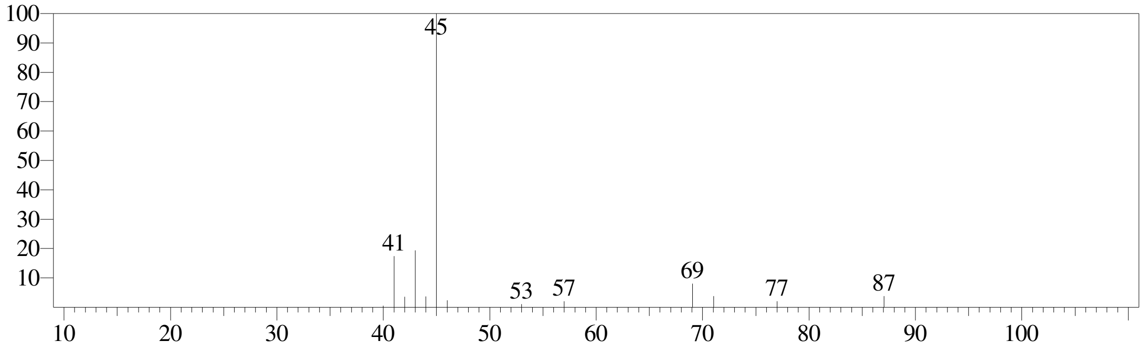

Hit#:1 Entry:2534 Library:NIST23-1.lib
SI:93 Formula:C6H14O CAS:26549-24-6 MolWeight:102 RetIndex:791
CompName:2-Hexanol, (R)- $$ (R)-(-)-2-Hexanol $$ 2-Hexanol # $$

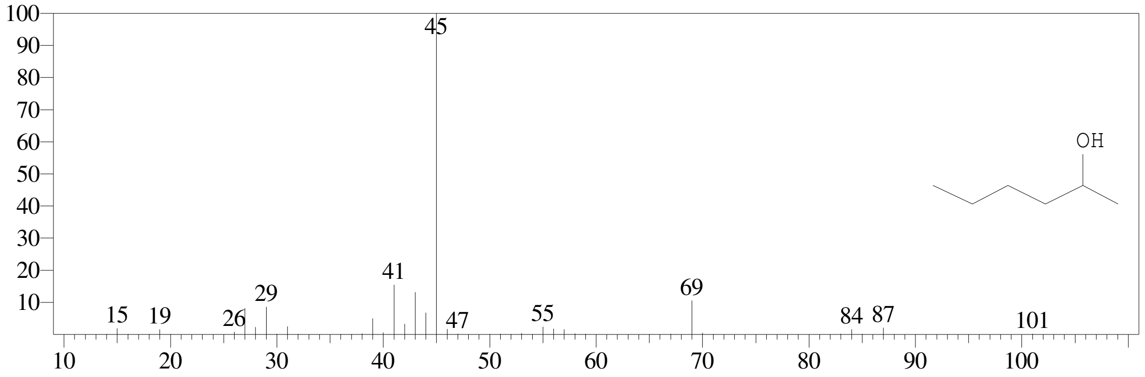

Hit#:2 Entry:2533 Library:NIST23-1.lib
SI:93 Formula:C6H14O CAS:52019-78-0 MolWeight:102 RetIndex:791
CompName:2-Hexanol, (S)- $$ (S)-(+)-2-Hexanol $$ 2-Hexanol # $$

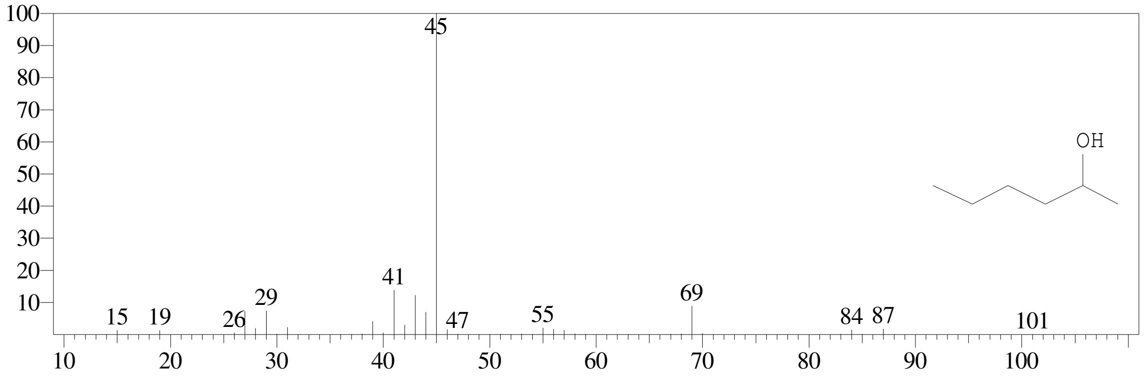

<< Target >>
Line#:4   R.Time:3.533(Scan#:125) MassPeaks:13
RawMode:Averaged 3.525-3.542(124-126) BasePeak:45.00(60902)
BG Mode:None Group 1 - Event 1 Scan

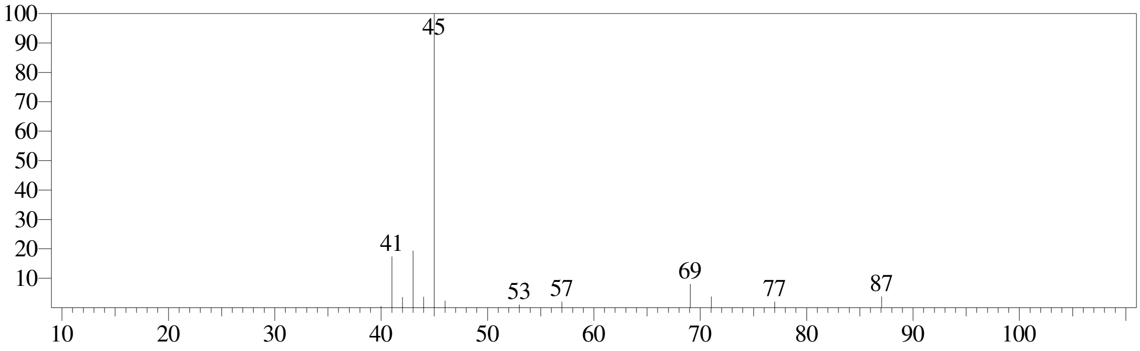

Hit#:3 Entry:1082 Library:NIST23s.lib
SI:93 Formula:C5H10O CAS:625-31-0 MolWeight:86 RetIndex:658
CompName:4-Penten-2-ol $$ 1-Penten-4-ol $$ 4-Hydroxypent-1-ene $$ CH2=CHCH2CH(OH)CH3 $$

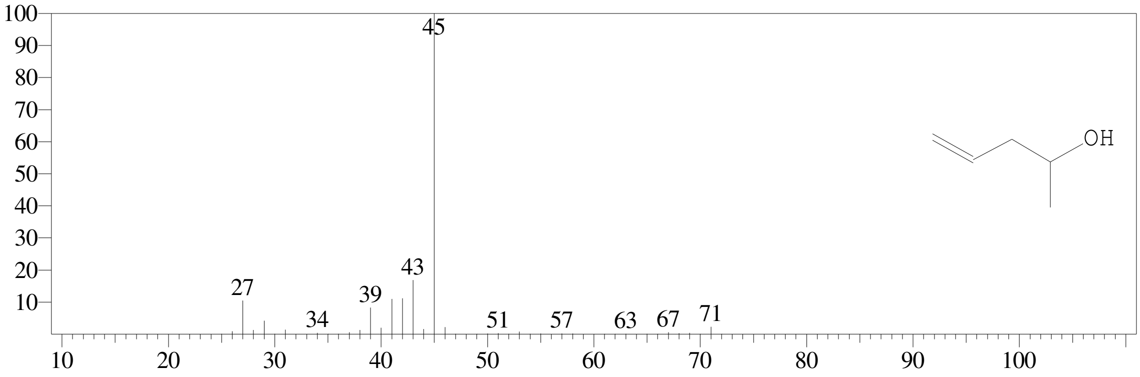

Hit#:4 Entry:2597 Library:NIST23s.lib
SI:93 Formula:C6H14O CAS:108-11-2 MolWeight:102 RetIndex:752
CompName:2-Pentanol, 4-methyl- $$ Isobutylmethylcarbinol $$ Isobutylmethylmethanol $$ Methylisobutylcarbinol $$ M

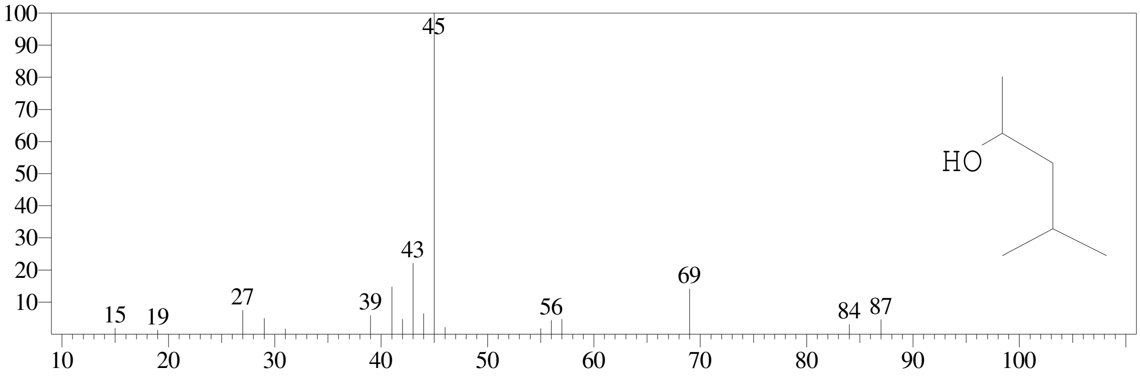

<< Target >>
Line#:4   R.Time:3.533(Scan#:125) MassPeaks:13
RawMode:Averaged 3.525-3.542(124-126) BasePeak:45.00(60902)
BG Mode:None Group 1 - Event 1 Scan

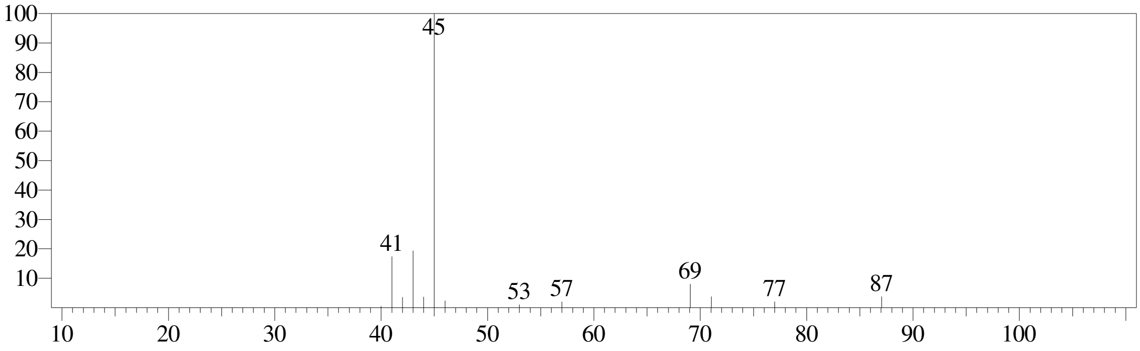

Hit#:5 Entry:2596 Library:NIST23s.lib
SI:92 Formula:C6H14O CAS:626-93-7 MolWeight:102 RetIndex:791
CompName:2-Hexanol $$ n-C4H9CH(OH)CH3 $$ n-Butylmethylcarbinol $$ Hexanol-(2) $$ sec-Hexyl alcohol $$ n-He[illegible]

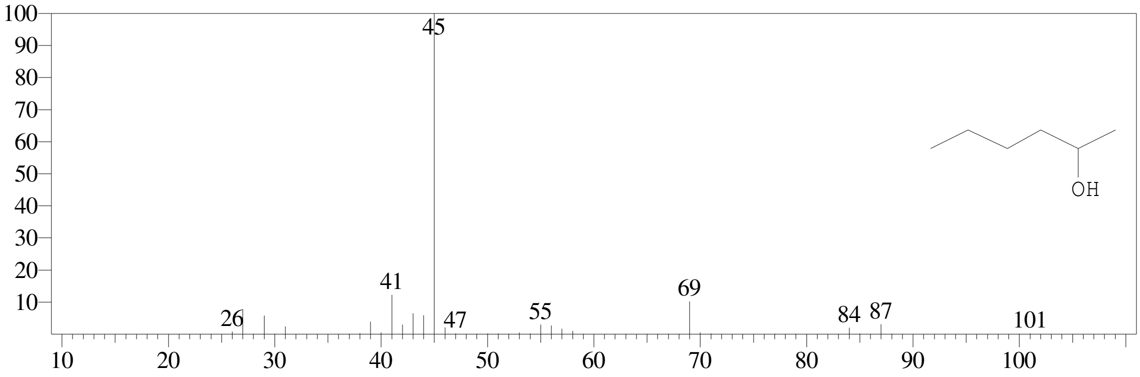

<< Target >>
Line#:5 R.Time:3.625(Scan#:136) MassPeaks:5
RawMode:Averaged 3.617-3.633(135-137) BasePeak:59.00(16188)
BG Mode:None Group 1 - Event 1 Scan

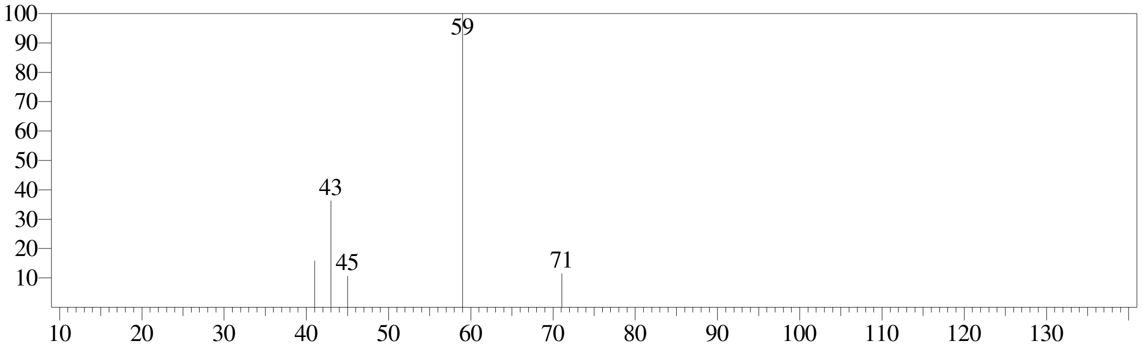

Hit#:1 Entry:2726 Library:NIST23-1.lib
SI:90 Formula:C4H8O3 CAS:594-61-6 MolWeight:104 RetIndex:932
CompName:Propanoic acid, 2-hydroxy-2-methyl- $$ 2-Hydroxyisobutyric acid $$ Lactic acid, 2-methyl- $$ .alpha.-Hydro

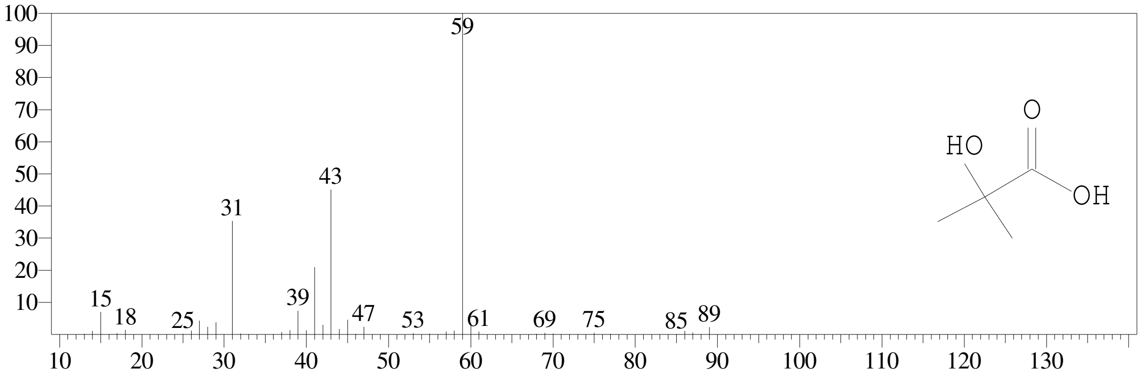

Hit#:2 Entry:2775 Library:NIST23-1.lib
SI:90 Formula:C5H12O2 CAS:5396-58-7 MolWeight:104 RetIndex:812
CompName:2-Methyl-2,3-butanediol $$ 2-Methylbutane-2,3-diol $$ 2,3-Dihydroxy-2-methylbutane $$

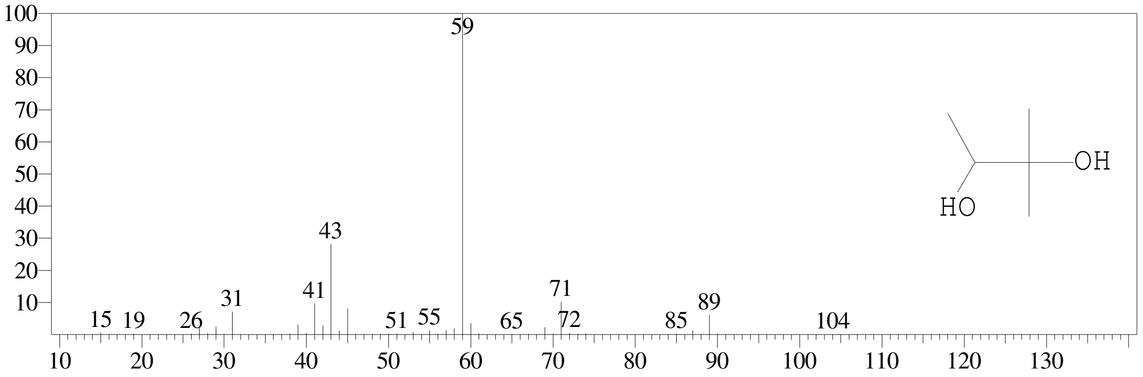

<< Target >>
Line#:5   R.Time:3.625(Scan#:136) MassPeaks:5
RawMode:Averaged 3.617-3.633(135-137) BasePeak:59.00(16188)
BG Mode:None Group 1 - Event 1 Scan

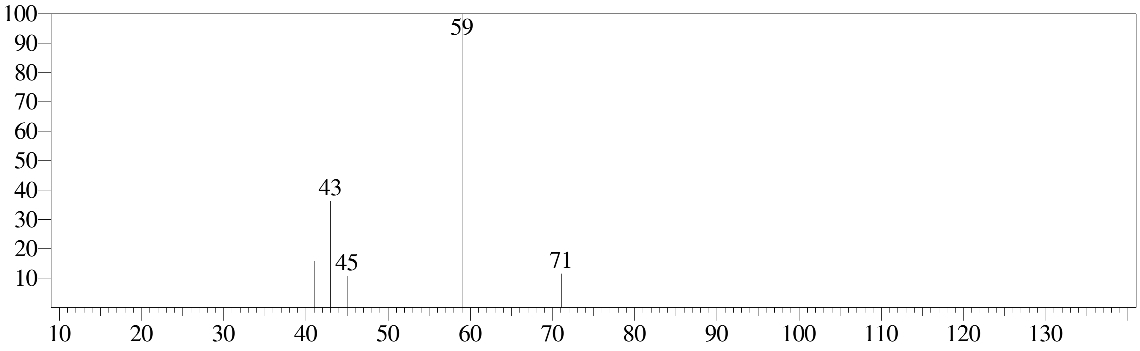

Hit#:3 Entry:2466 Library:NIST23-1.lib
SI:89 Formula:C5H10O2 CAS:115-22-0 MolWeight:102 RetIndex:737
CompName:3-Hydroxy-3-methyl-2-butanone $$ 2-Butanone, 3-hydroxy-3-methyl- $$ Dimethylacetylcarbinol $$ 3-Hydrc

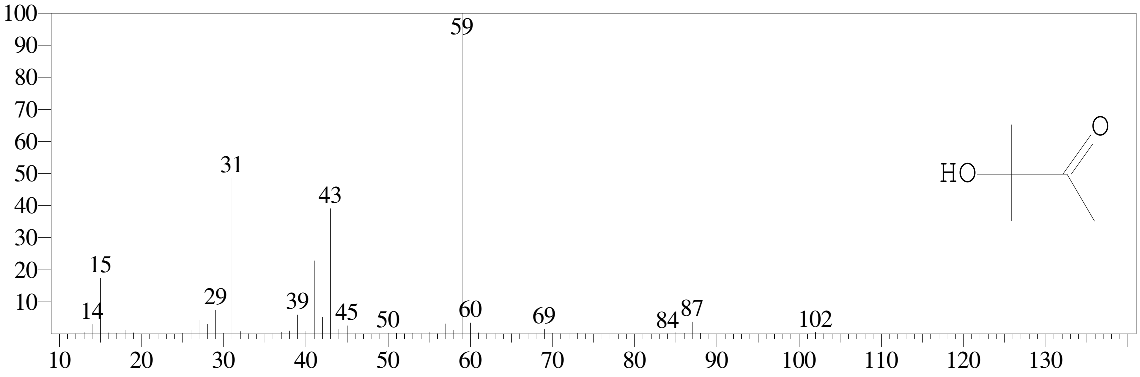

Hit#:4 Entry:583 Library:NIST23s.lib
SI:89 Formula:C4H10O CAS:598-53-8 MolWeight:74 RetIndex:471
CompName:Propane, 2-methoxy- $$ Ether, isopropyl methyl $$ Isopropyl methyl ether $$ Isopryl $$ Methyl isopropyl etl

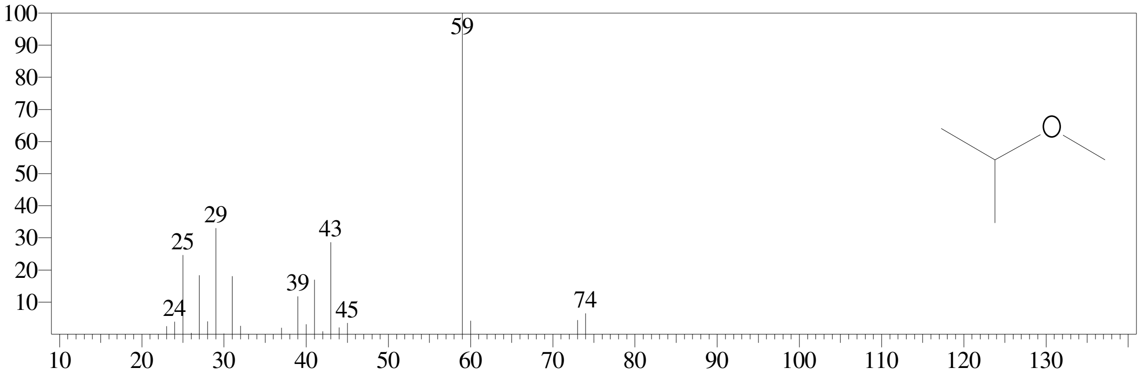

<< Target >>
Line#:5 R.Time:3.625(Scan#:136) MassPeaks:5
RawMode:Averaged 3.617-3.633(135-137) BasePeak:59.00(16188)
BG Mode:None Group 1 - Event 1 Scan

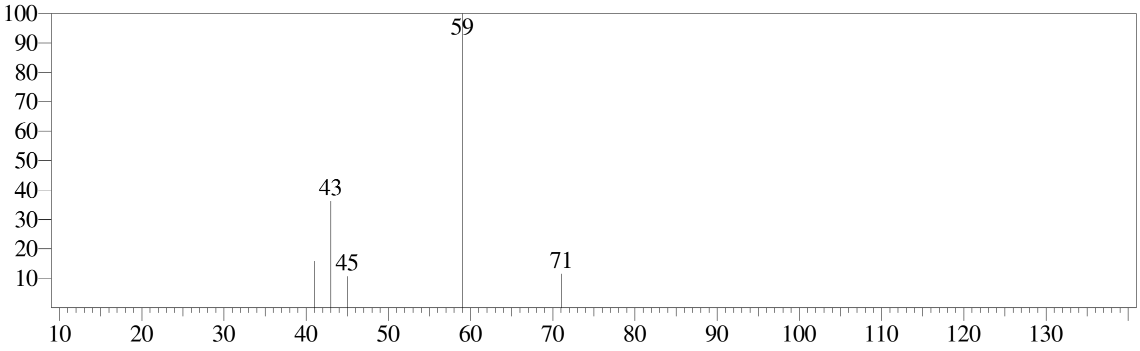

Hit#:5 Entry:9783 Library:NIST23-1.lib
SI:89 Formula:C6H12O3 CAS:70657-70-4 MolWeight:132 RetIndex:880
CompName:2-Methoxypropyl acetate $$ 1-Propanol, 2-methoxy-, 1-acetate $$ 1-Propanol, 2-methoxy-, acetate $$ 2-Meth

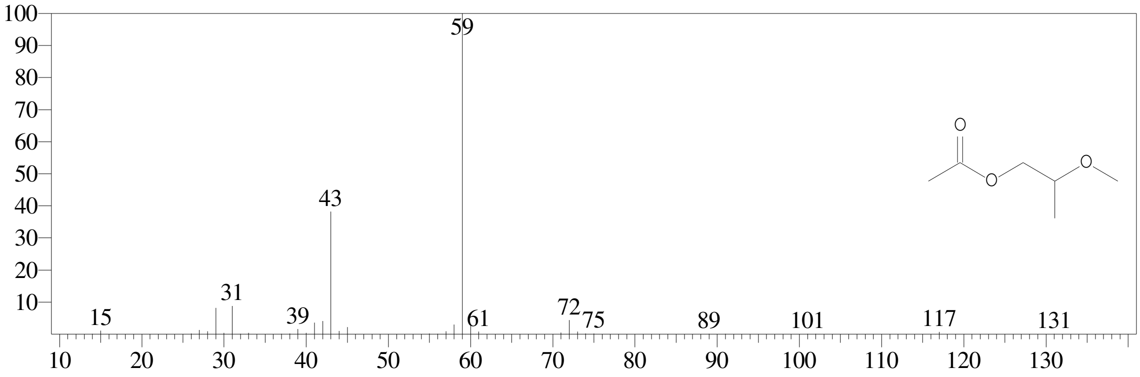

<< Target >>
Line#:6 R.Time:3.800(Scan#:157) MassPeaks:2
RawMode:Averaged 3.792-3.808(156-158) BasePeak:77.00(1902)
BG Mode:None Group 1 - Event 1 Scan

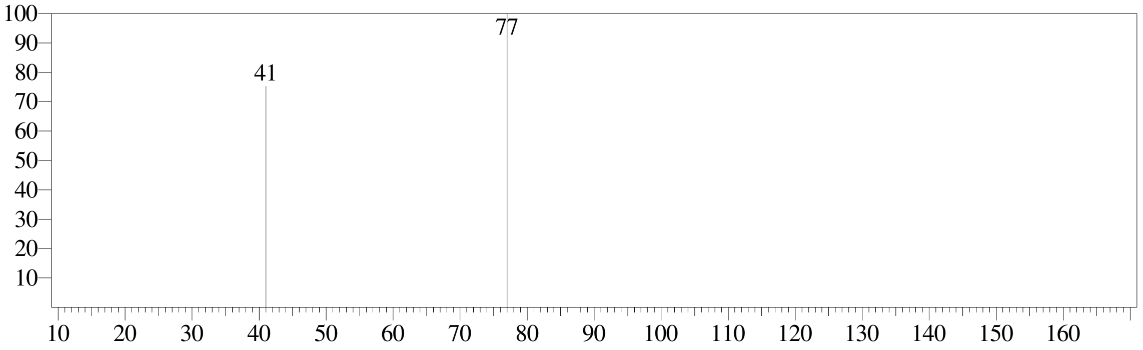

Hit#:1 Entry:21721 Library:NIST23-1.lib
SI:80 Formula:C3H6BrCl CAS:3017-95-6 MolWeight:156 RetIndex:776
CompName:Propane, 2-bromo-1-chloro- $$ 1-Chloro-2-bromopropane $$ 2-Bromo-1-chloropropane $$

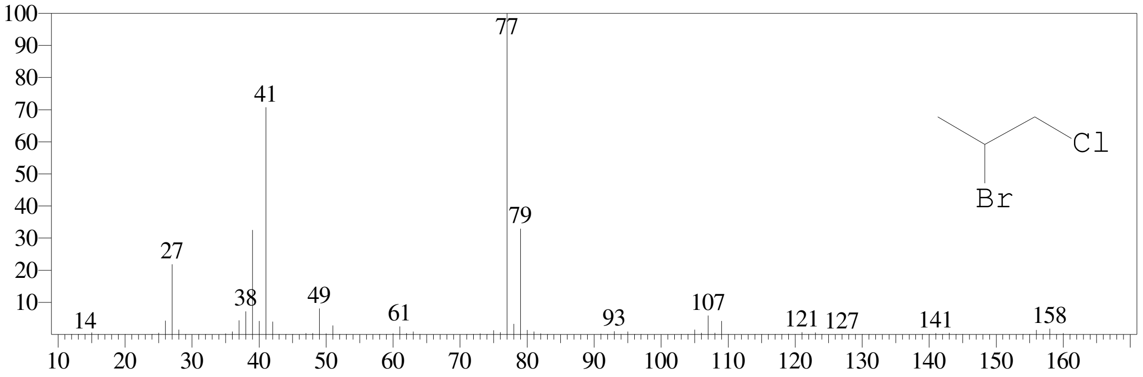

Hit#:2 Entry:13201 Library:NIST23s.lib
SI:76 Formula:C3H6BrCl CAS:3017-95-6 MolWeight:156 RetIndex:776
CompName:Propane, 2-bromo-1-chloro- $$ 1-Chloro-2-bromopropane $$ 2-Bromo-1-chloropropane $$

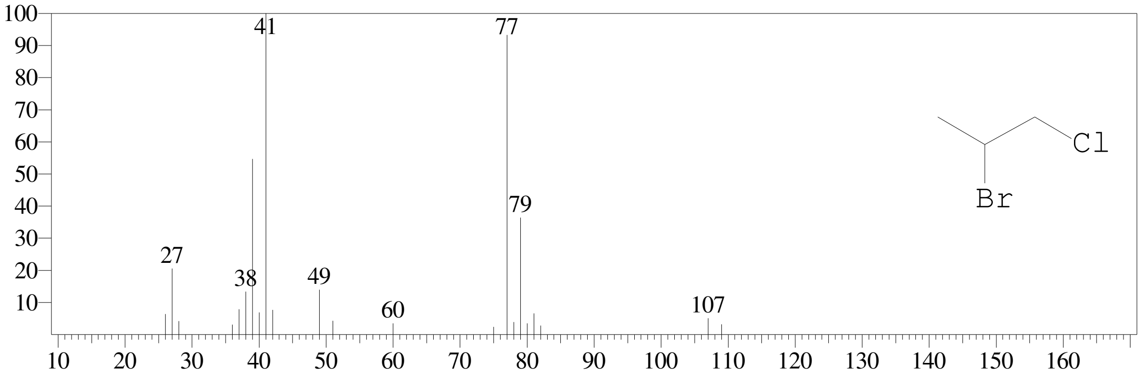

<< Target >>
Line#:6 R.Time:3.800(Scan#:157) MassPeaks:2
RawMode:Averaged 3.792-3.808(156-158) BasePeak:77.00(1902)
BG Mode:None Group 1 - Event 1 Scan

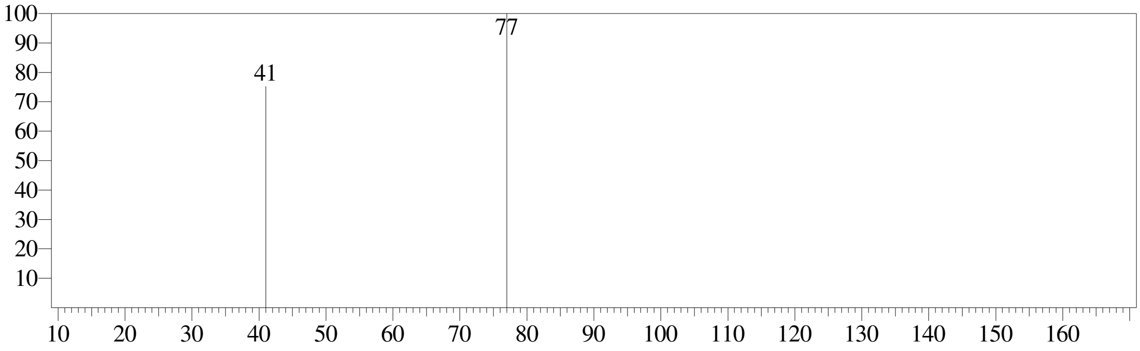

Hit#:3 Entry:13204 Library:NIST23s.lib
SI:75 Formula:C3H6BrCl CAS:3017-95-6 MolWeight:156 RetIndex:776
CompName:Propane, 2-bromo-1-chloro- $$ 1-Chloro-2-bromopropane $$ 2-Bromo-1-chloropropane $$

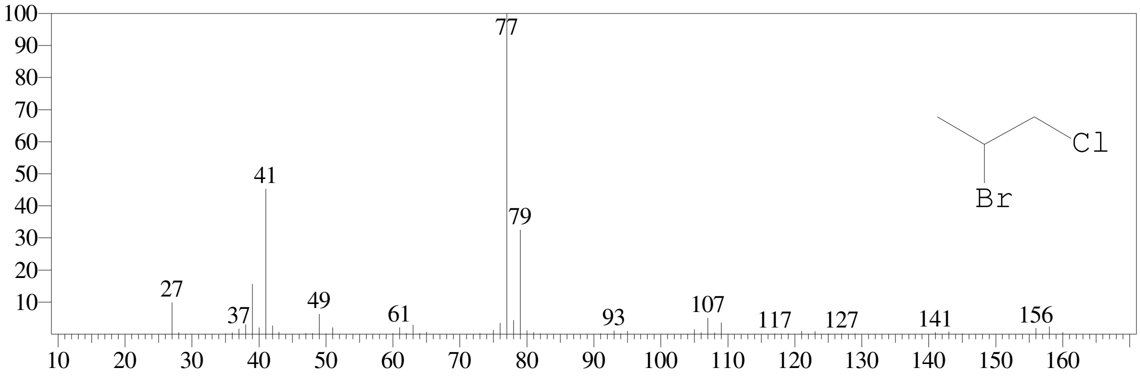

Hit#:4 Entry:3526 Library:NIST23s.lib
SI:73 Formula:C3H6Cl2 CAS:594-20-7 MolWeight:112 RetIndex:608
CompName:Propane, 2,2-dichloro- $$ Dimethyldichloromethane $$ 2,2-Dichloropropane $$

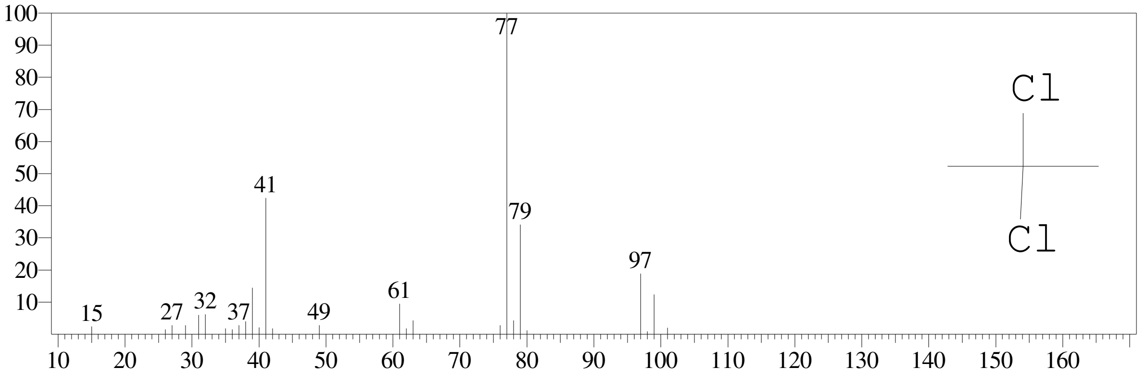

<< Target >>
Line#:6   R.Time:3.800(Scan#:157) MassPeaks:2
RawMode:Averaged 3.792-3.808(156-158) BasePeak:77.00(1902)
BG Mode:None Group 1 - Event 1 Scan

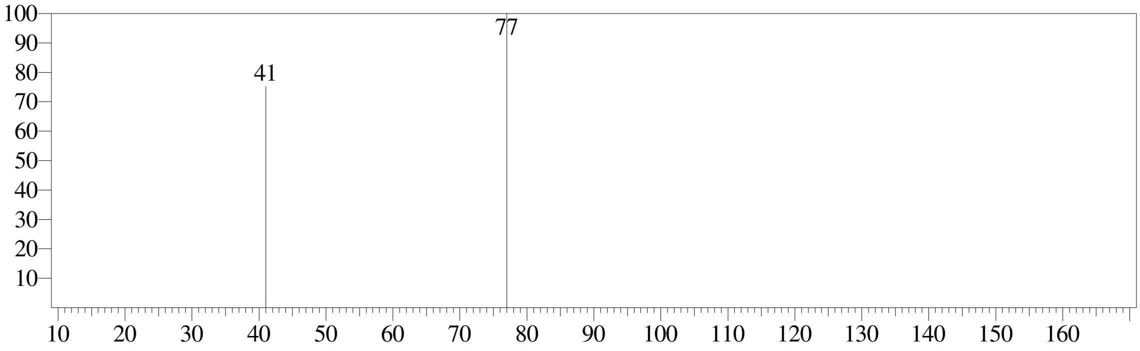

Hit#:5 Entry:3716 Library:NIST23-1.lib
SI:72 Formula:C3H6Cl2 CAS:78-99-9 MolWeight:112 RetIndex:679
CompName:Propane, 1,1-dichloro- $$ Propylidene chloride $$ 1,1-Dichloropropane $$

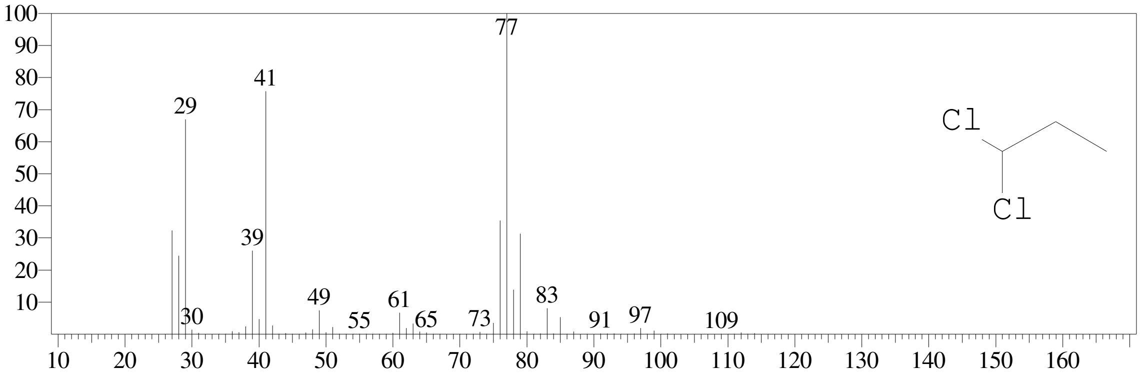

<< Target >>
Line#:7 R.Time:5.925(Scan#:412) MassPeaks:2
RawMode:Averaged 5.917-5.933(411-413) BasePeak:78.95(2827)
BG Mode:None Group 1 - Event 1 Scan

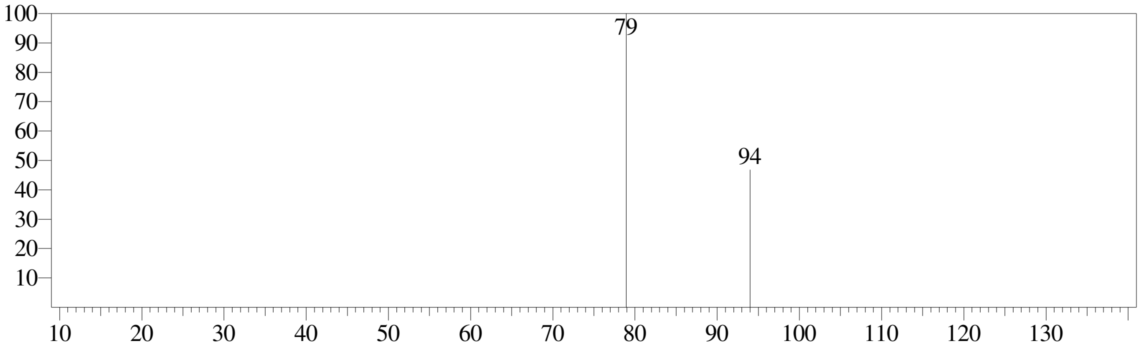

Hit#:1 Entry:1582 Library:NIST23s.lib
SI:96 Formula:C2H6O2S CAS:67-71-0 MolWeight:94 RetIndex:900
CompName:Dimethyl sulfone $$ Methane, sulfonylbis- $$ MSM $$ Methyl sulfone $$ Methylsulfonylmethane $$ Dimetl

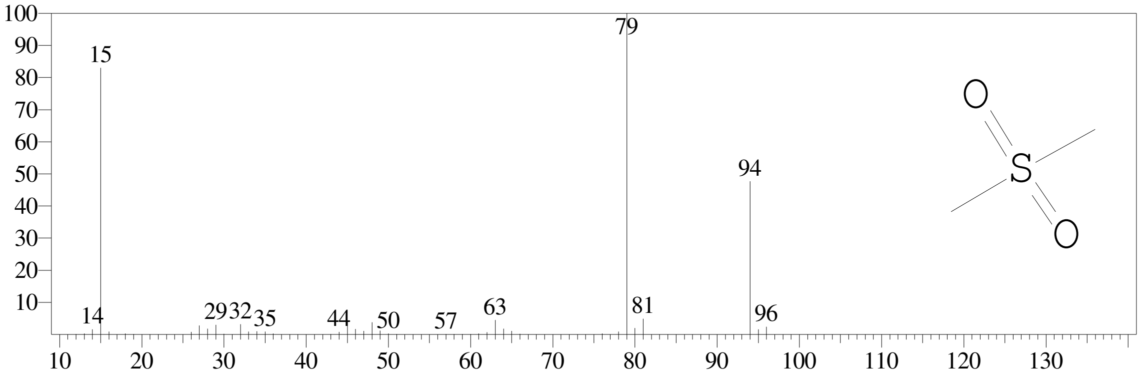

Hit#:2 Entry:1584 Library:NIST23s.lib
SI:95 Formula:C2H6O2S CAS:67-71-0 MolWeight:94 RetIndex:900
CompName:Dimethyl sulfone $$ Methane, sulfonylbis- $$ MSM $$ Methyl sulfone $$ Methylsulfonylmethane $$ Dimetl

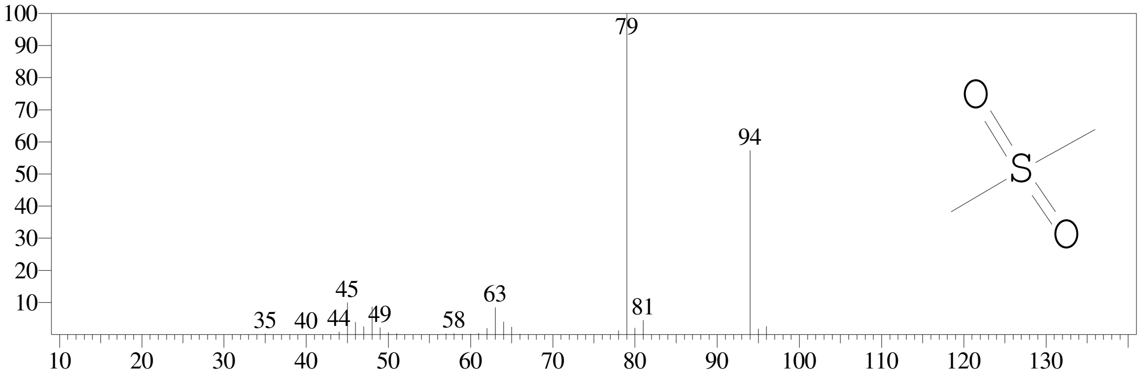

<< Target >>
Line#:7 R.Time:5.925(Scan#:412) MassPeaks:2
RawMode:Averaged 5.917-5.933(411-413) BasePeak:78.95(2827)
BG Mode:None Group 1 - Event 1 Scan

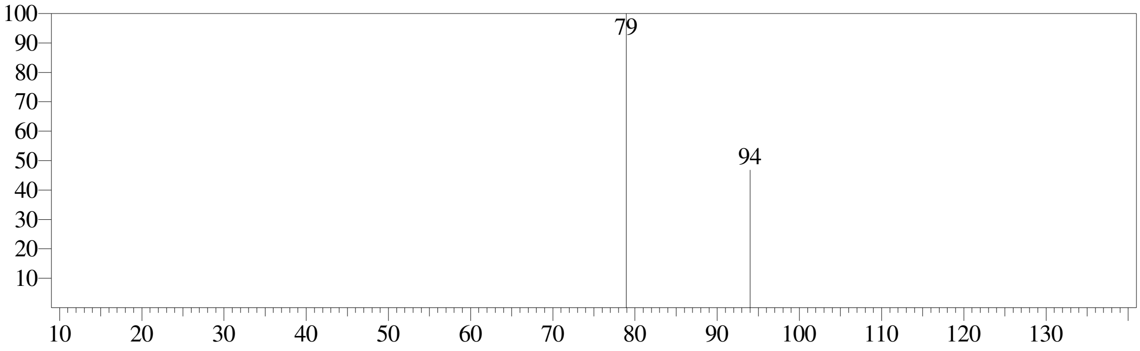

Hit#:3 Entry:1310 Library:NIST23-1.lib
SI:94 Formula:C2H6O2S CAS:67-71-0 MolWeight:94 RetIndex:900
CompName:Dimethyl sulfone $$ Methane, sulfonylbis- $$ MSM $$ Methyl sulfone $$ Methylsulfonylmethane $$ Dimetl

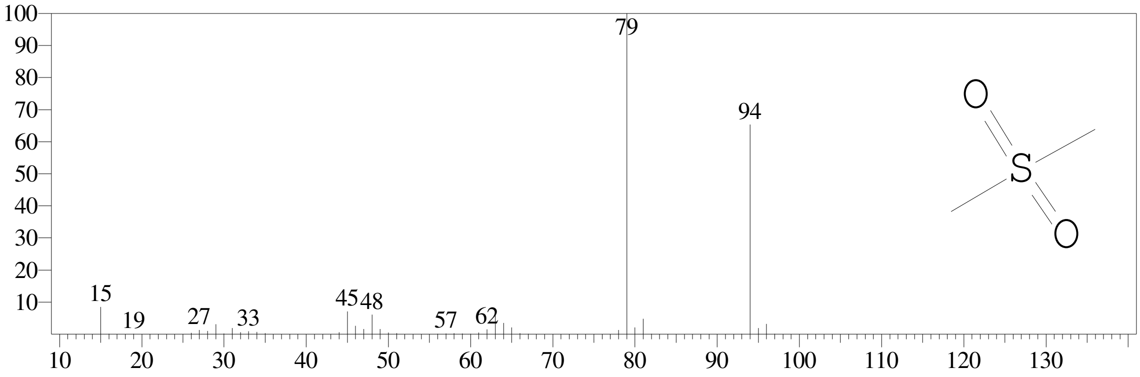

Hit#:4 Entry:11736 Library:NIST23-1.lib
SI:93 Formula:C3H6O4S CAS:2516-97-4 MolWeight:138 RetIndex:1309
CompName:Methanesulfonylacetic acid

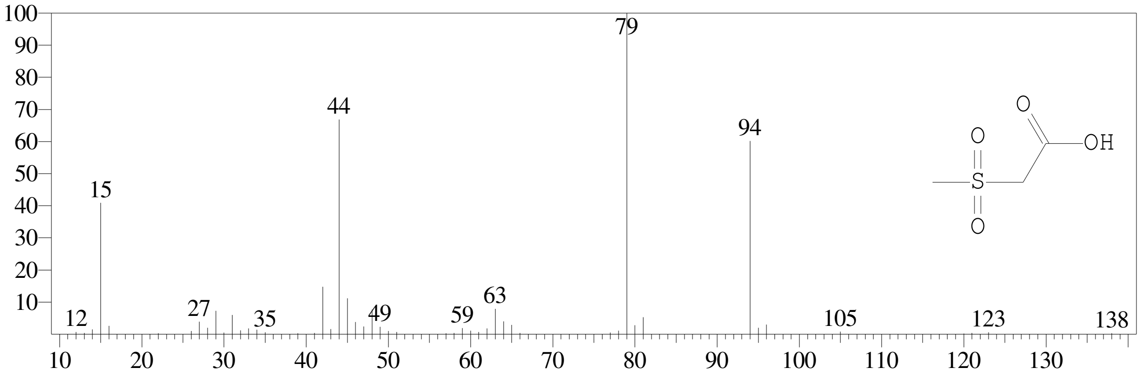

<< Target >>
Line#:7   R.Time:5.925(Scan#:412) MassPeaks:2
RawMode:Averaged 5.917-5.933(411-413) BasePeak:78.95(2827)
BG Mode:None Group 1 - Event 1 Scan

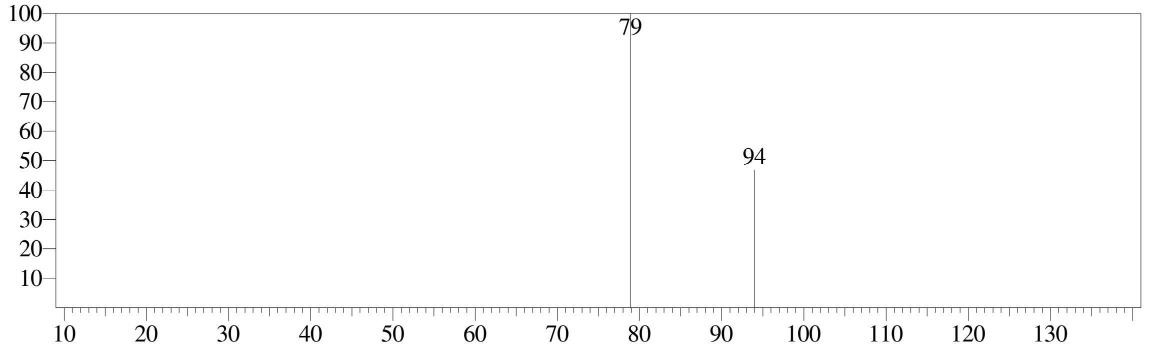

Hit#:5 Entry:1585 Library:NIST23s.lib
SI:92 Formula:C2H6O2S CAS:67-71-0 MolWeight:94 RetIndex:900
CompName:Dimethyl sulfone $$ Methane, sulfonylbis- $$ MSM $$ Methyl sulfone $$ Methylsulfonylmethane $$ Dimetl

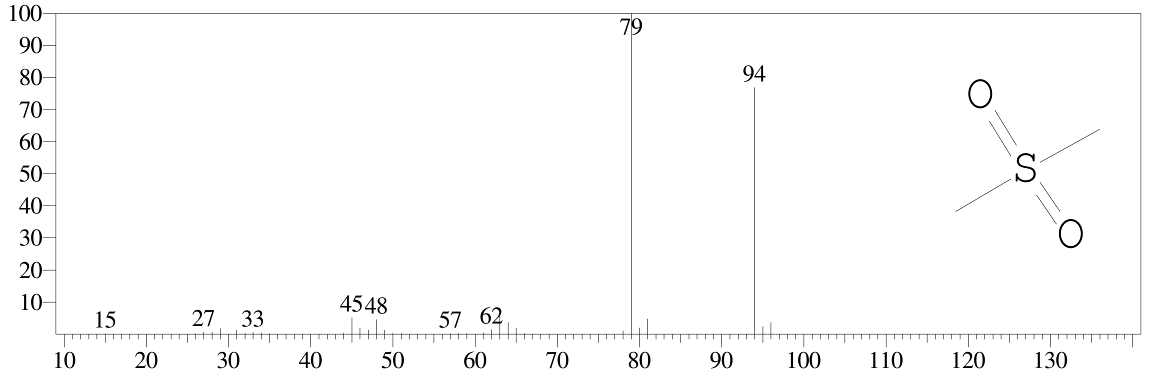

<< Target >>
Line#:8   R.Time:6.358(Scan#:464) MassPeaks:35
RawMode:Averaged 6.350-6.367(463-465) BasePeak:93.05(99604)
BG Mode:None Group 1 - Event 1 Scan

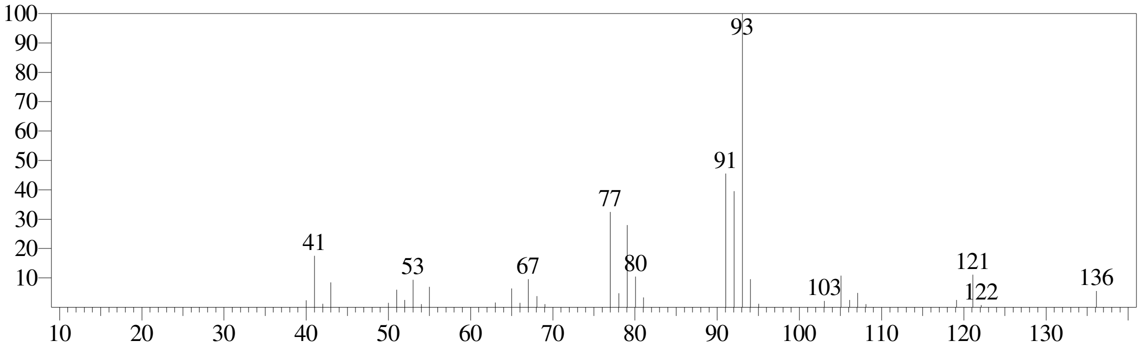

Hit#:1 Entry:8445 Library:NIST23s.lib
SI:97 Formula:C10H16 CAS:80-56-8 MolWeight:136 RetIndex:947
CompName:.alpha.-Pinene $$ Bicyclo[3.1.1]hept-2-ene, 2,6,6-trimethyl- $$ 2-Pinene $$ 2,6,6-Trimethylbicyclo[3.1.1]hep

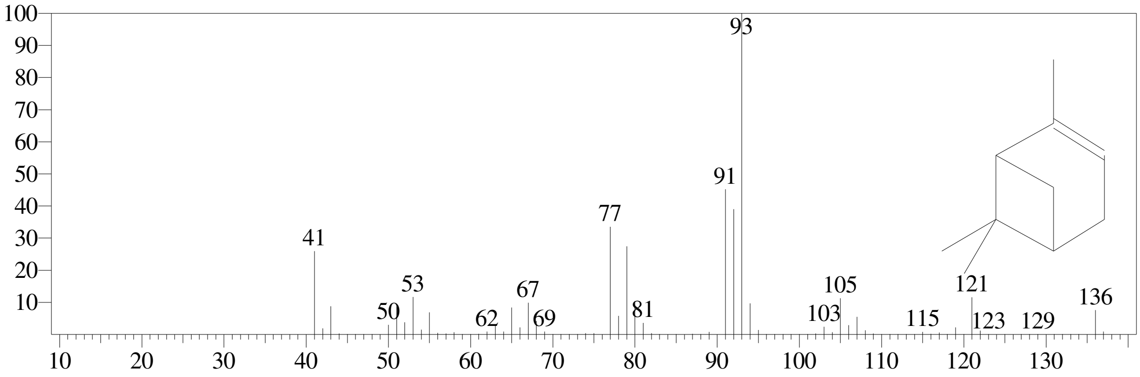

Hit#:2 Entry:8451 Library:NIST23s.lib
SI:96 Formula:C10H16 CAS:80-56-8 MolWeight:136 RetIndex:947
CompName:.alpha.-Pinene $$ Bicyclo[3.1.1]hept-2-ene, 2,6,6-trimethyl- $$ 2-Pinene $$ 2,6,6-Trimethylbicyclo[3.1.1]hep

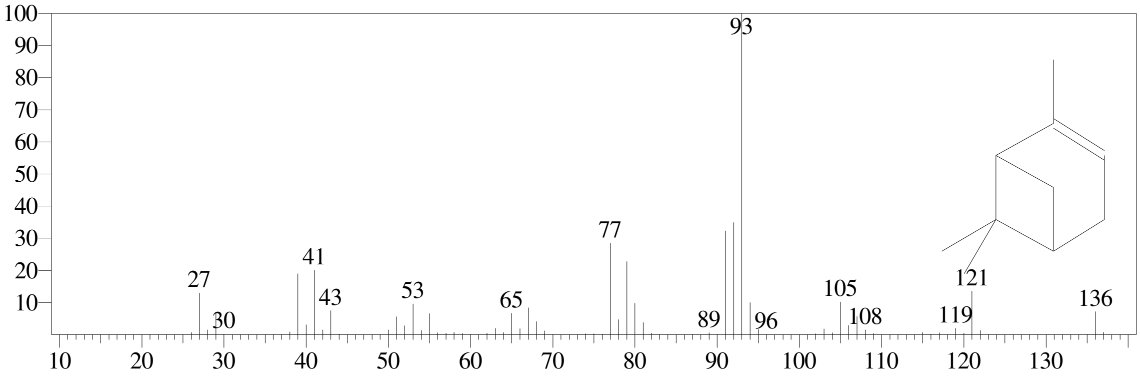

<< Target >>
Line#:8   R.Time:6.358(Scan#:464) MassPeaks:35
RawMode:Averaged 6.350-6.367(463-465) BasePeak:93.05(99604)
BG Mode:None Group 1 - Event 1 Scan

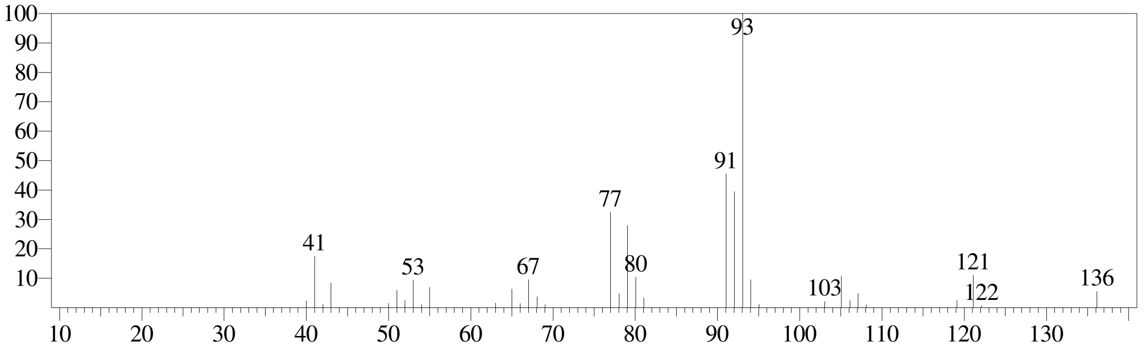

Hit#:3 Entry:11410 Library:NIST23-1.lib
SI:96 Formula:C10H16 CAS:80-56-8 MolWeight:136 RetIndex:947
CompName:.alpha.-Pinene $$ Bicyclo[3.1.1]hept-2-ene, 2,6,6-trimethyl- $$ 2-Pinene $$ 2,6,6-Trimethylbicyclo[3.1.1]hep

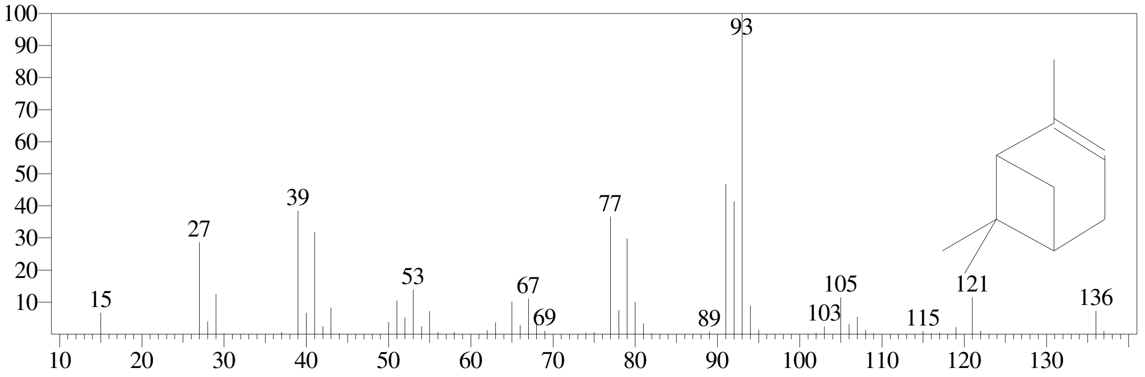

Hit#:4 Entry:8443 Library:NIST23s.lib
SI:95 Formula:C10H16 CAS:3779-61-1 MolWeight:136 RetIndex:1047
CompName:trans-.beta.-Ocimene $$ 1,3,6-Octatriene, 3,7-dimethyl-, (E)- $$ .beta.-trans-Ocimene $$ trans-3,7-Dimethyl-

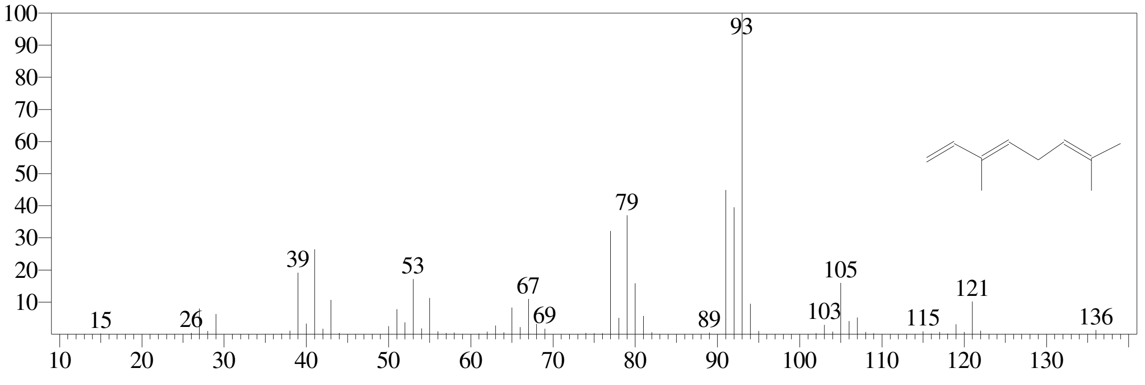

<< Target >>
Line#:8   R.Time:6.358(Scan#:464) MassPeaks:35
RawMode:Averaged 6.350-6.367(463-465) BasePeak:93.05(99604)
BG Mode:None Group 1 - Event 1 Scan

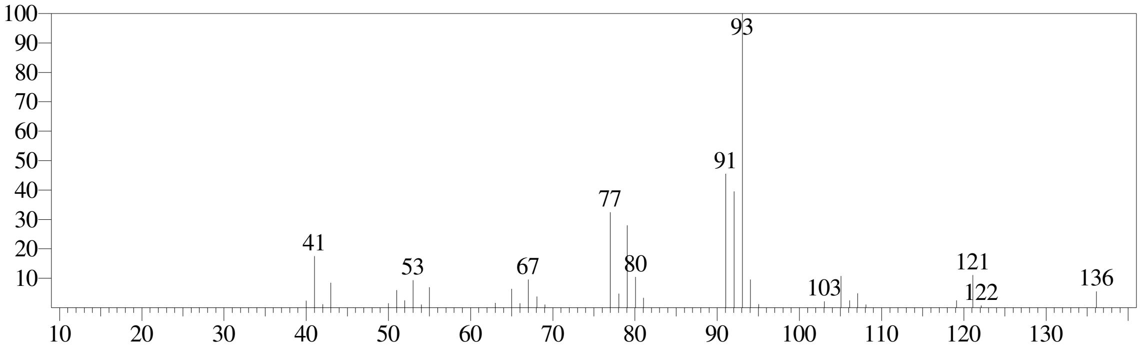

Hit#:5 Entry:8450 Library:NIST23s.lib
SI:95 Formula:C10H16 CAS:80-56-8 MolWeight:136 RetIndex:947
CompName:.alpha.-Pinene $$ Bicyclo[3.1.1]hept-2-ene, 2,6,6-trimethyl- $$ 2-Pinene $$ 2,6,6-Trimethylbicyclo[3.1.1]hep

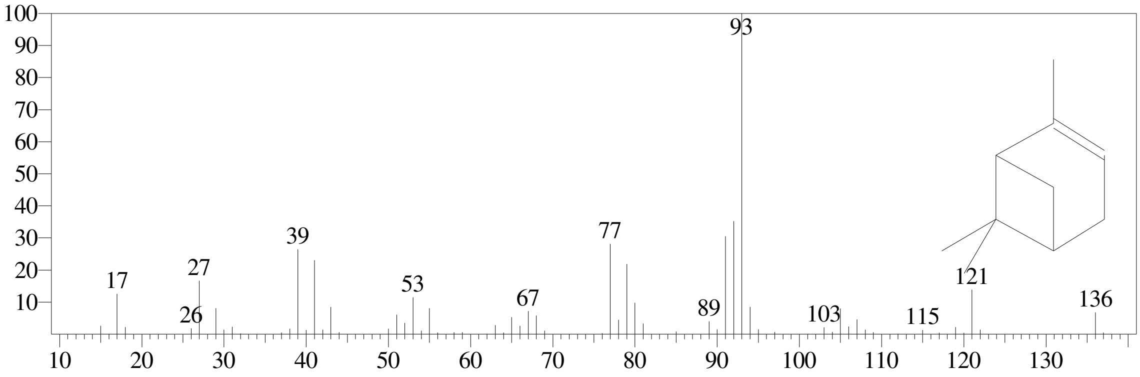

<< Target >>
Line#:9 R.Time:7.717(Scan#:627) MassPeaks:12
RawMode:Averaged 7.708-7.725(626-628) BasePeak:93.05(10062)
BG Mode:None Group 1 - Event 1 Scan

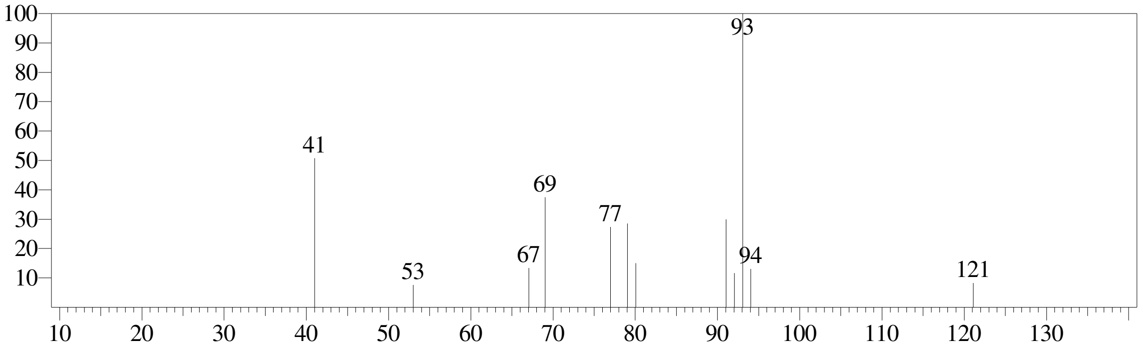

Hit#:1 Entry:8389 Library:NIST23s.lib
SI:87 Formula:C10H16 CAS:18172-67-3 MolWeight:136 RetIndex:978
CompName:Bicyclo[3.1.1]heptane, 6,6-dimethyl-2-methylene-, (1S)- $$ 2(10)-Pinene, (1S,5S)-(-)- $$ (-)-.beta.-Pinene $$

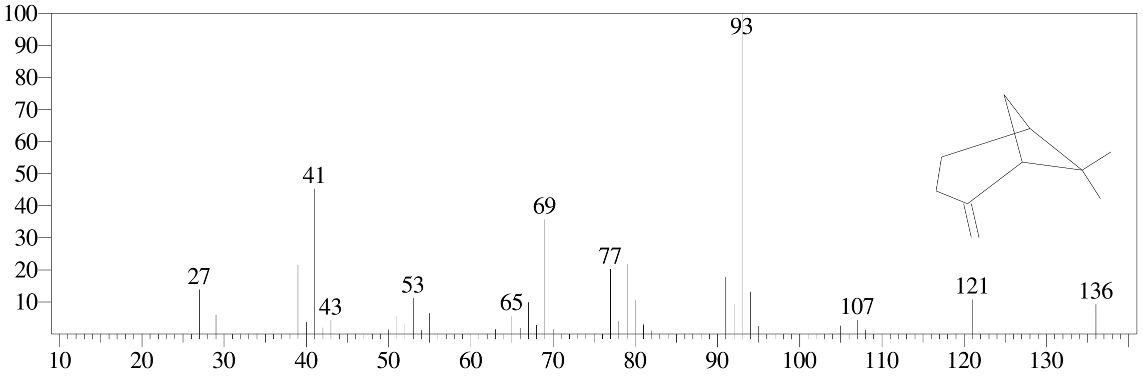

Hit#:2 Entry:8392 Library:NIST23s.lib
SI:87 Formula:C10H16 CAS:127-91-3 MolWeight:136 RetIndex:978
CompName:.beta.-Pinene $$ Bicyclo[3.1.1]heptane, 6,6-dimethyl-2-methylene- $$ 2(10)-Pinene $$ Nopinen $$ Nopinene

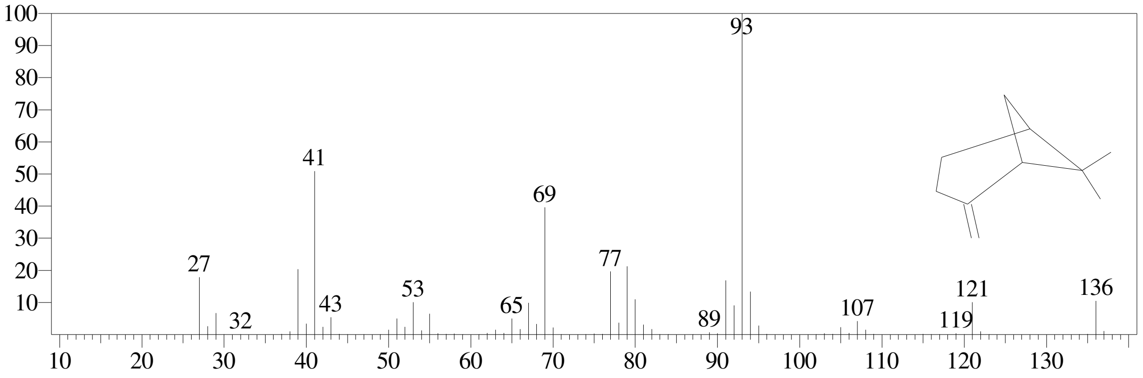

<< Target >>
Line#:9 R.Time:7.717(Scan#:627) MassPeaks:12
RawMode:Averaged 7.708-7.725(626-628) BasePeak:93.05(10062)
BG Mode:None Group 1 - Event 1 Scan

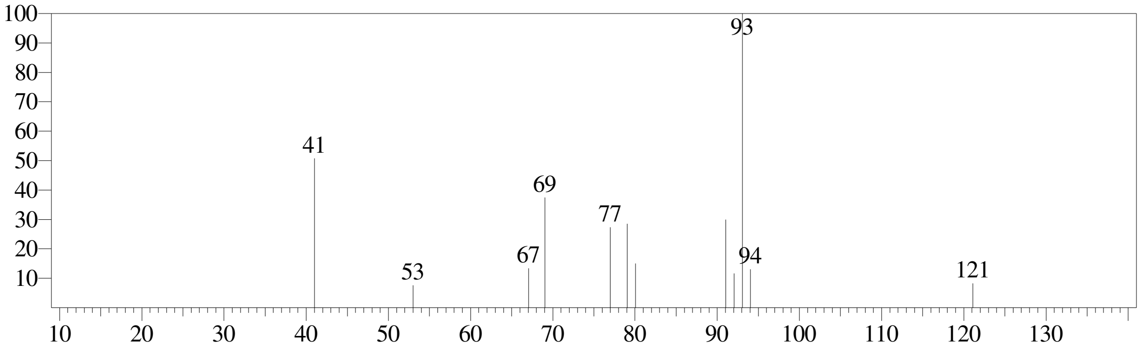

Hit#:3 Entry:8393 Library:NIST23s.lib
SI:87 Formula:C10H16 CAS:127-91-3 MolWeight:136 RetIndex:978
CompName:.beta.-Pinene $$ Bicyclo[3.1.1]heptane, 6,6-dimethyl-2-methylene- $$ 2(10)-Pinene $$ Nopinen $$ Nopinene

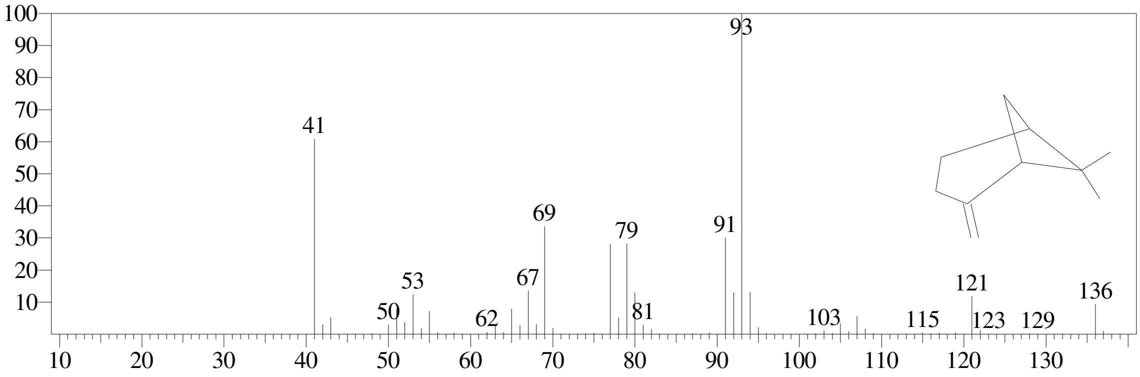

Hit#:4 Entry:11378 Library:NIST23-1.lib
SI:87 Formula:C10H16 CAS:127-91-3 MolWeight:136 RetIndex:978
CompName:.beta.-Pinene $$ Bicyclo[3.1.1]heptane, 6,6-dimethyl-2-methylene- $$ 2(10)-Pinene $$ Nopinen $$ Nopinene

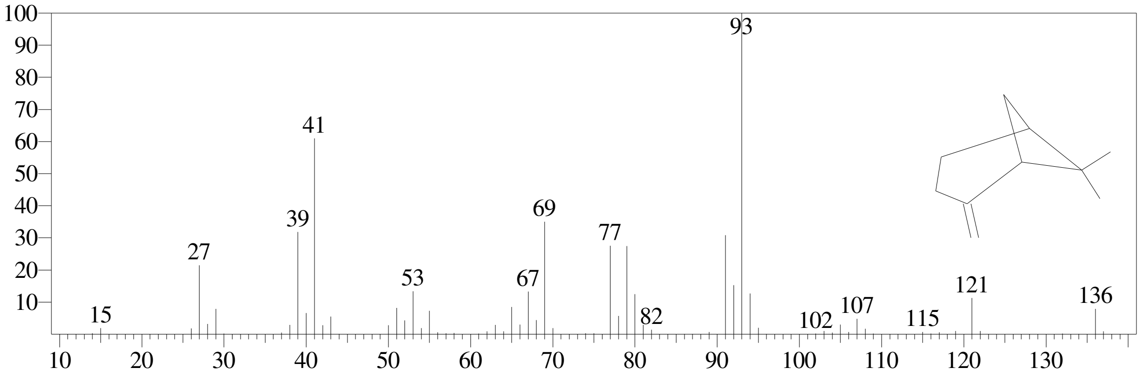

<< Target >>
Line#:9 R.Time:7.717(Scan#:627) MassPeaks:12
RawMode:Averaged 7.708-7.725(626-628) BasePeak:93.05(10062)
BG Mode:None Group 1 - Event 1 Scan

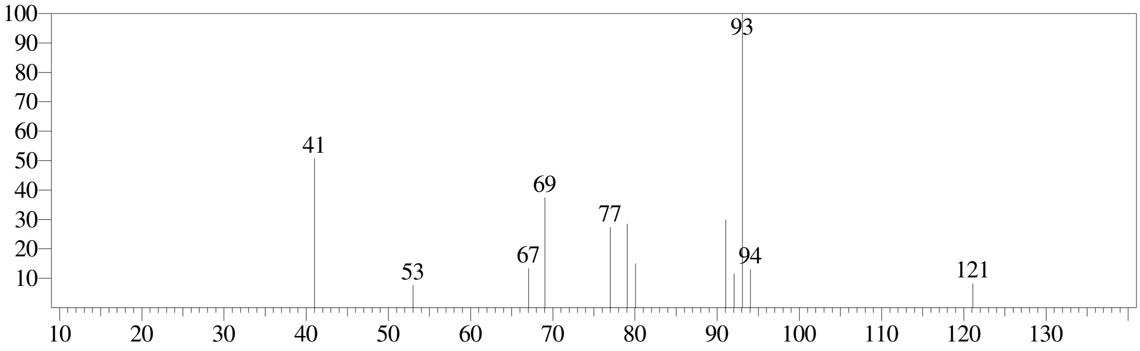

Hit#:5 Entry:11377 Library:NIST23-1.lib
SI:86 Formula:C10H16 CAS:18172-67-3 MolWeight:136 RetIndex:978
CompName:Bicyclo[3.1.1]heptane, 6,6-dimethyl-2-methylene-, (1S)- $$ 2(10)-Pinene, (1S,5S)-(-)- $$ (-)-.beta.-Pinene $$

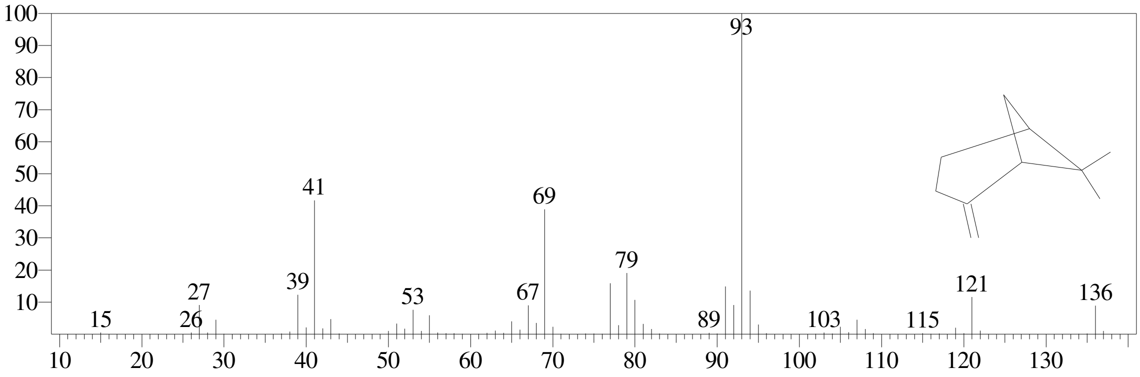

<< Target >>
Line#:10   R.Time:8.150(Scan#:679) MassPeaks:8
RawMode:Averaged 8.142-8.158(678-680) BasePeak:41.00(7760)
BG Mode:None Group 1 - Event 1 Scan

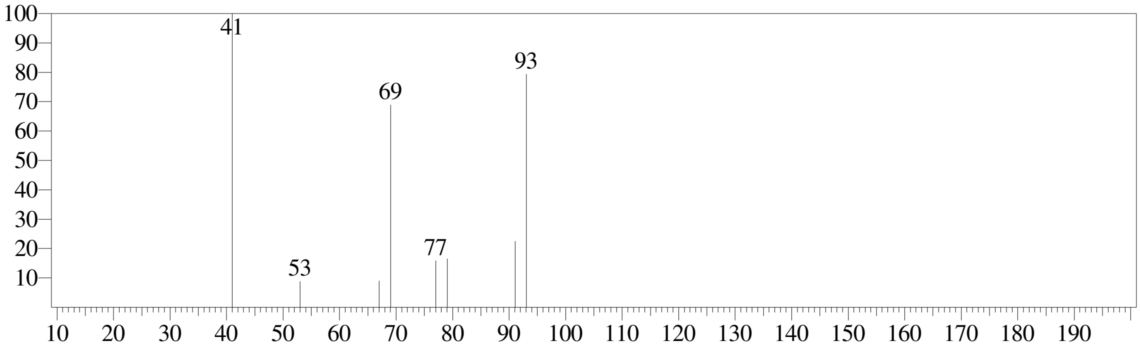

Hit#:1 Entry:8355 Library:NIST23s.lib
SI:85 Formula:C10H16 CAS:123-35-3 MolWeight:136 RetIndex:993
CompName:.beta.-Myrcene $$ 1,6-Octadiene, 7-methyl-3-methylene- $$ Myrcene $$ 7-Methyl-3-methylene-1,6-octadien

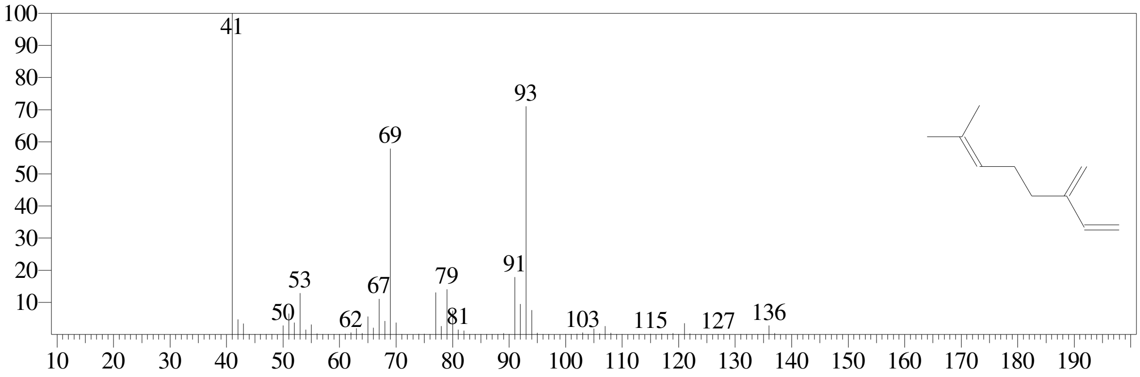

Hit#:2 Entry:8395 Library:NIST23s.lib
SI:85 Formula:C10H16 CAS:123-35-3 MolWeight:136 RetIndex:993
CompName:.beta.-Myrcene $$ 1,6-Octadiene, 7-methyl-3-methylene- $$ Myrcene $$ 7-Methyl-3-methylene-1,6-octadien

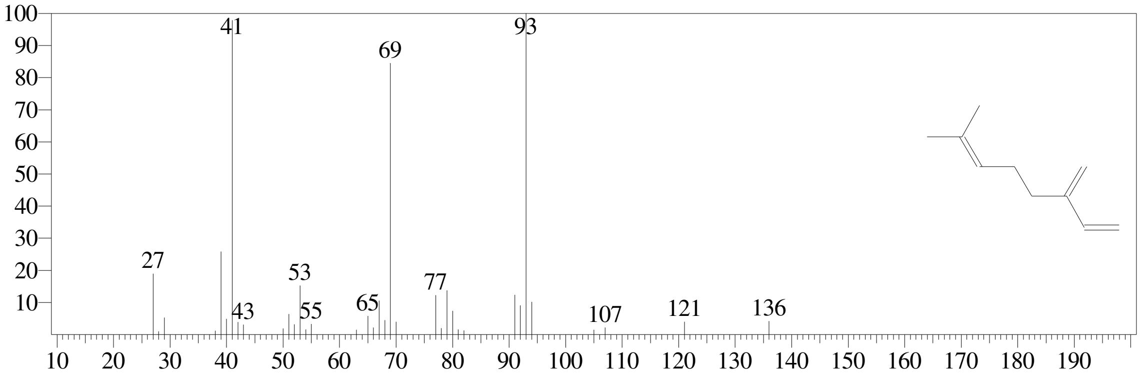

<< Target >>
Line#:10   R.Time:8.150(Scan#:679) MassPeaks:8
RawMode:Averaged 8.142-8.158(678-680) BasePeak:41.00(7760)
BG Mode:None Group 1 - Event 1 Scan

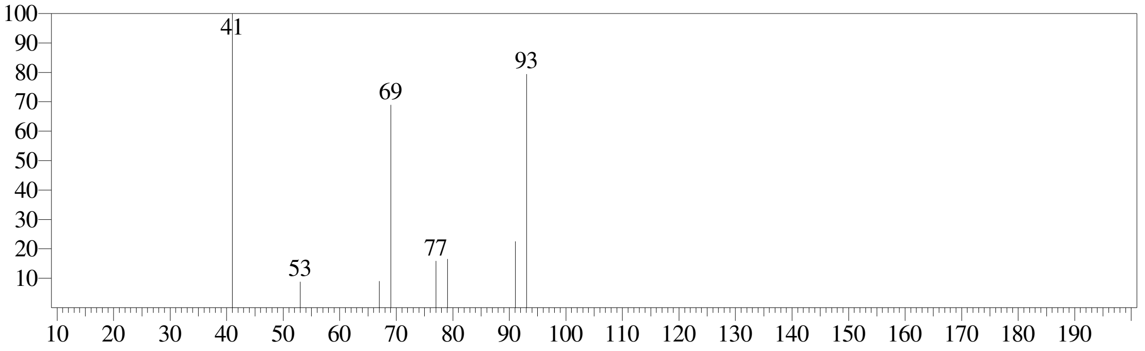

Hit#:3 Entry:58400 Library:NIST23-1.lib
SI:84 Formula:C10H16S2 CAS:73188-23-5 MolWeight:200 RetIndex:1622
CompName:4-(4-Methylpent-3-enyl)-3,6-dihydro-1,2-dithiin $$ 4-(4-Methyl-3-pentenyl)-3,6-dihydro-1,2-dithiine # $$

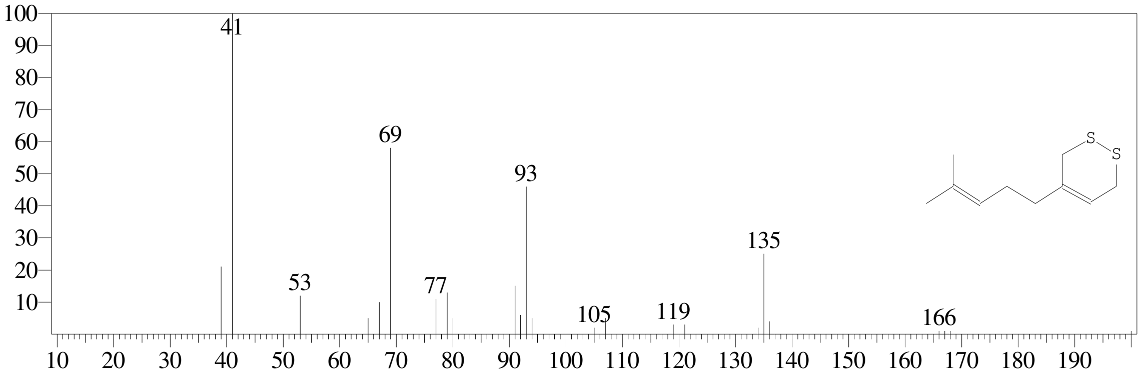

Hit#:4 Entry:11317 Library:NIST23-1.lib
SI:84 Formula:C10H16 CAS:123-35-3 MolWeight:136 RetIndex:993
CompName:.beta.-Myrcene $$ 1,6-Octadiene, 7-methyl-3-methylene- $$ Myrcene $$ 7-Methyl-3-methylene-1,6-octadien

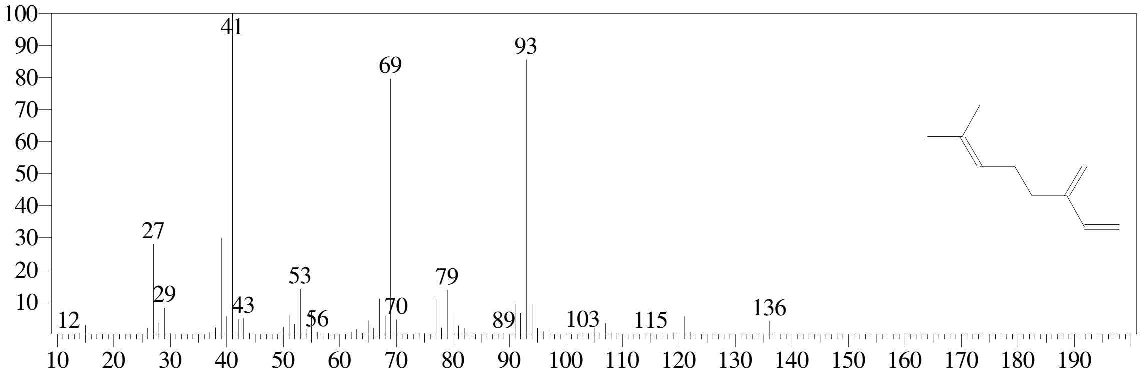

<< Target >>
Line#:10   R.Time:8.150(Scan#:679) MassPeaks:8
RawMode:Averaged 8.142-8.158(678-680) BasePeak:41.00(7760)
BG Mode:None Group 1 - Event 1 Scan

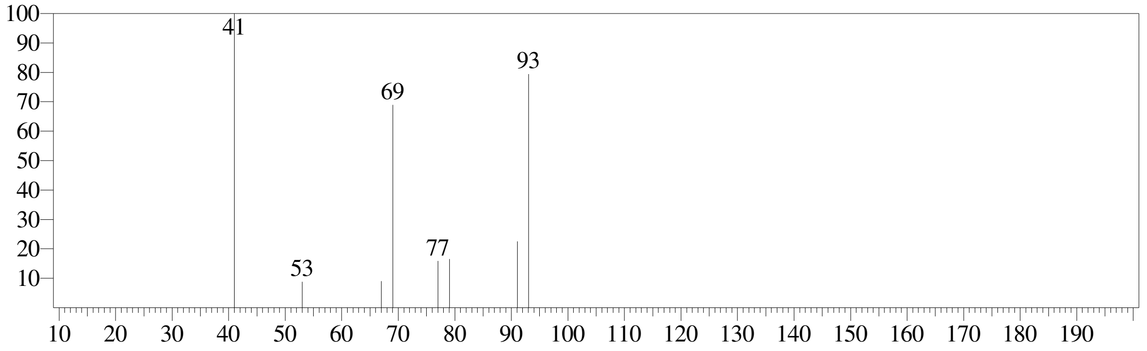

Hit#:5 Entry:8406 Library:NIST23s.lib
SI:82 Formula:C10H16 CAS:123-35-3 MolWeight:136 RetIndex:993
CompName:.beta.-Myrcene $$ 1,6-Octadiene, 7-methyl-3-methylene- $$ Myrcene $$ 7-Methyl-3-methylene-1,6-octadien[illegible]

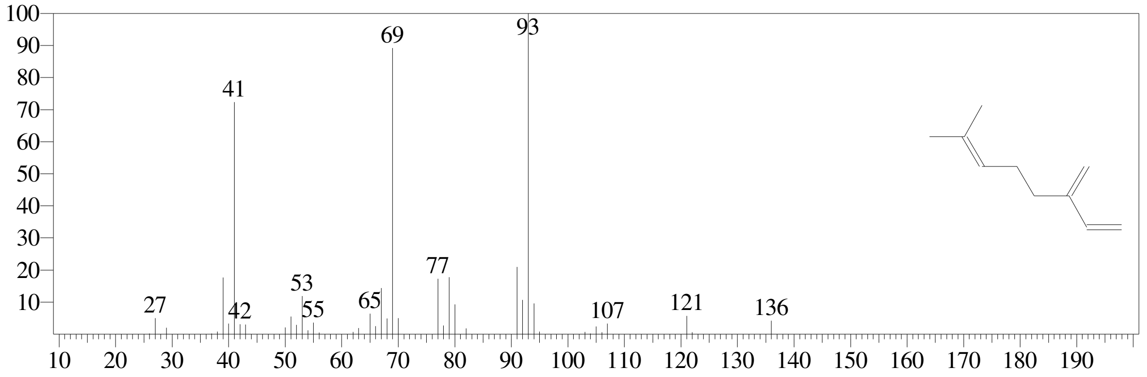

<< Target >>
Line#:11 R.Time:8.642(Scan#:738) MassPeaks:9
RawMode:Averaged 8.633-8.650(737-739) BasePeak:93.10(11255)
BG Mode:None Group 1 - Event 1 Scan

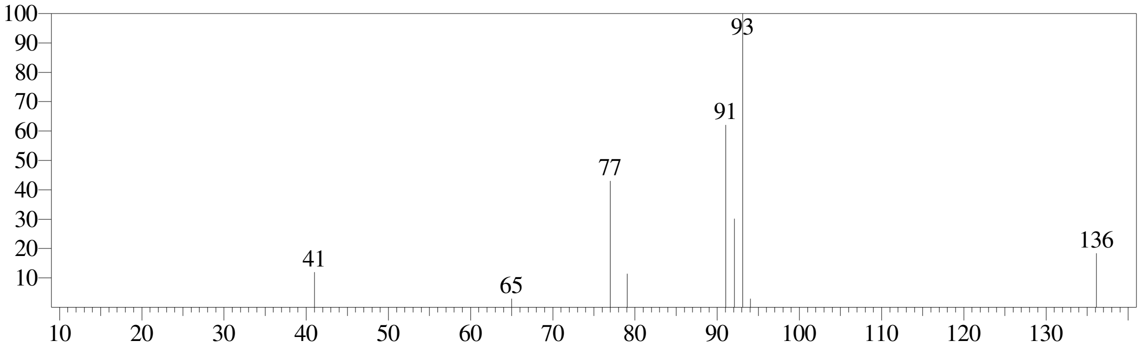

Hit#:1 Entry:8437 Library:NIST23s.lib
SI:90 Formula:C10H16 CAS:99-83-2 MolWeight:136 RetIndex:1017
CompName:.alpha.-Phellandrene $$ 1,3-Cyclohexadiene, 2-methyl-5-(1-methylethyl)- $$ .alpha.-Fellandrene $$ p-Mentha

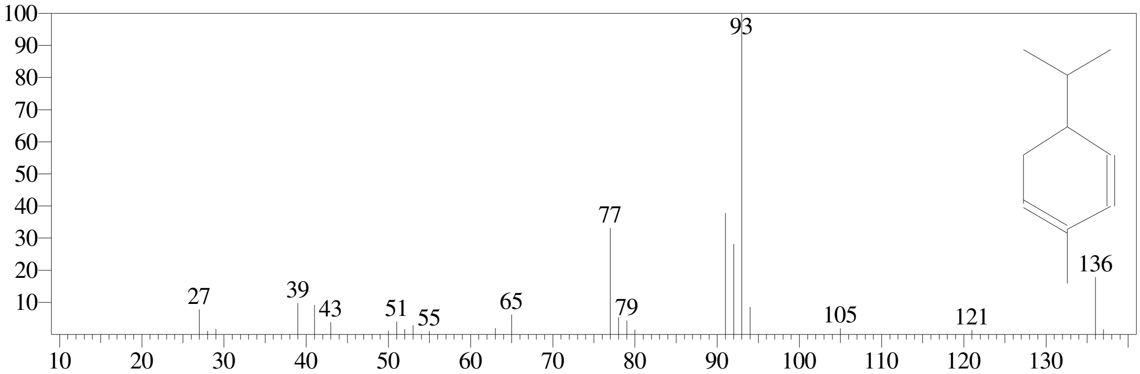

Hit#:2 Entry:8438 Library:NIST23s.lib
SI:88 Formula:C10H16 CAS:99-83-2 MolWeight:136 RetIndex:1017
CompName:.alpha.-Phellandrene $$ 1,3-Cyclohexadiene, 2-methyl-5-(1-methylethyl)- $$ .alpha.-Fellandrene $$ p-Mentha

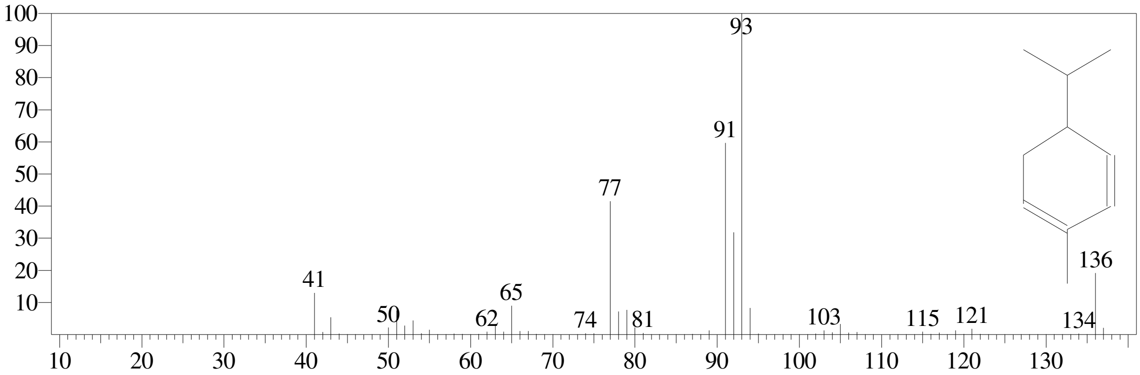

<< Target >>
Line#:11   R.Time:8.642(Scan#:738) MassPeaks:9
RawMode:Averaged 8.633-8.650(737-739) BasePeak:93.10(11255)
BG Mode:None Group 1 - Event 1 Scan

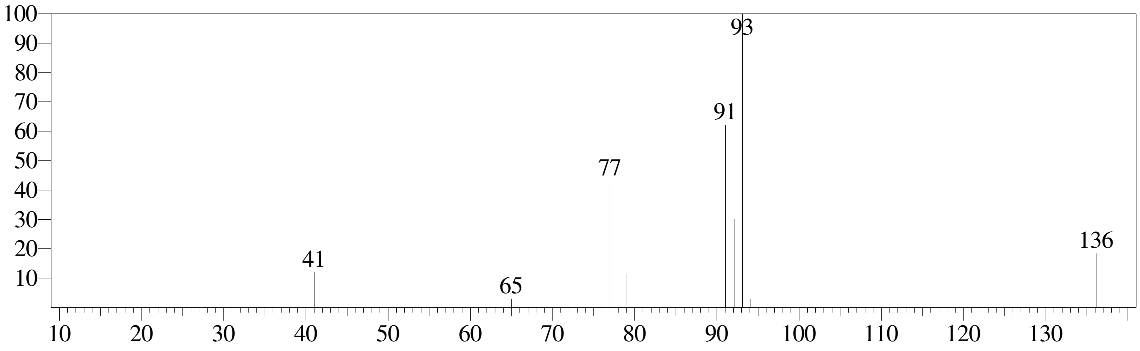

Hit#:3 Entry:11406 Library:NIST23-1.lib
SI:87 Formula:C10H16 CAS:2867-05-2 MolWeight:136 RetIndex:939
CompName:Bicyclo[3.1.0]hex-2-ene, 2-methyl-5-(1-methylethyl)- $$ 3-Thujene $$ .alpha.-Thujene $$ Thujene, .alpha.- $

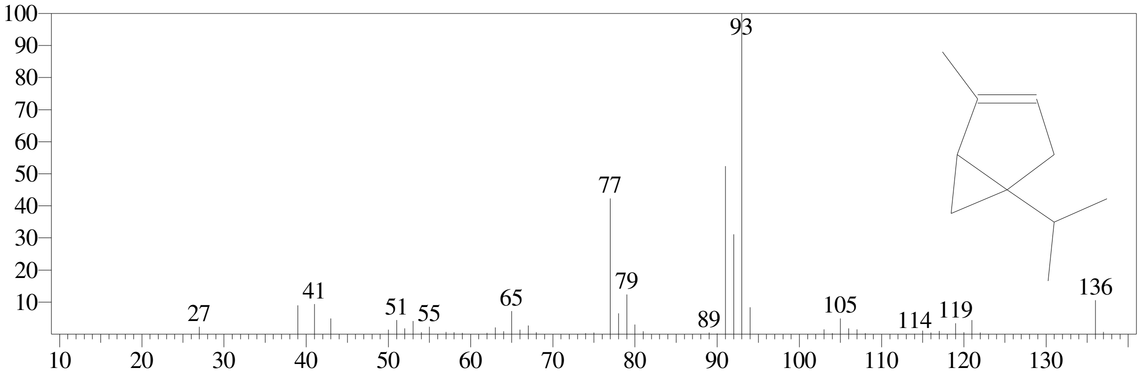

Hit#:4 Entry:8435 Library:NIST23s.lib
SI:87 Formula:C10H16 CAS:99-83-2 MolWeight:136 RetIndex:1017
CompName:.alpha.-Phellandrene $$ 1,3-Cyclohexadiene, 2-methyl-5-(1-methylethyl)- $$ .alpha.-Fellandrene $$ p-Menth

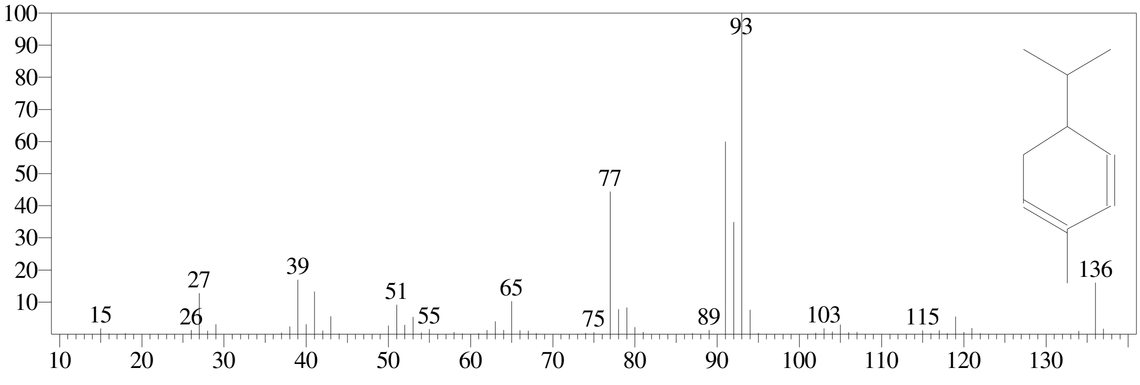

<< Target >>
Line#:11 R.Time:8.642(Scan#:738) MassPeaks:9
RawMode:Averaged 8.633-8.650(737-739) BasePeak:93.10(11255)
BG Mode:None Group 1 - Event 1 Scan

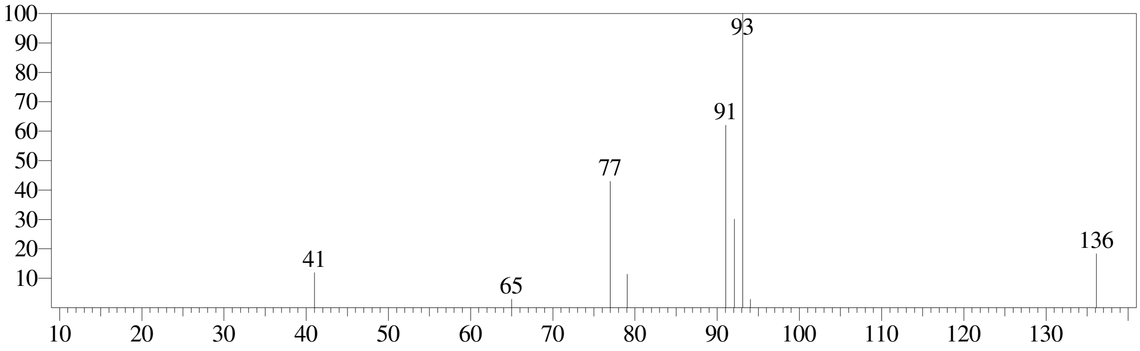

Hit#:5 Entry:8434 Library:NIST23s.lib
SI:86 Formula:C10H16 CAS:99-83-2 MolWeight:136 RetIndex:1017
CompName:.alpha.-Phellandrene $$ 1,3-Cyclohexadiene, 2-methyl-5-(1-methylethyl)- $$ .alpha.-Fellandrene $$ p-Mentha

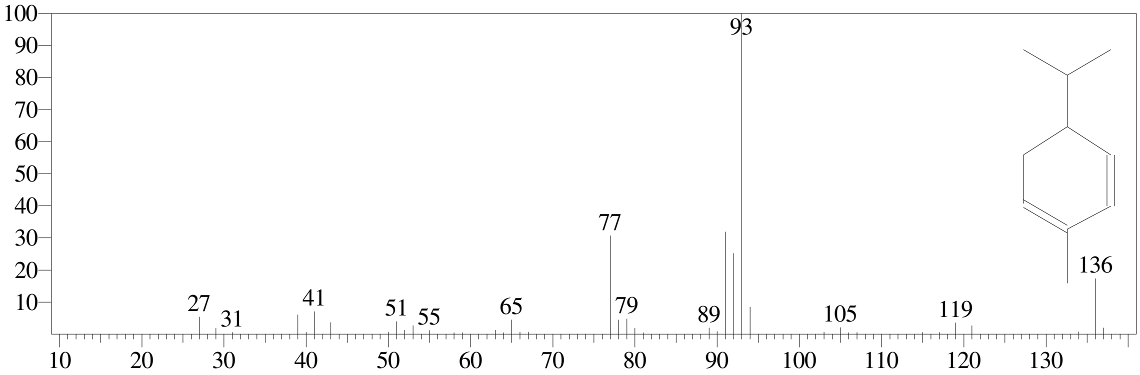

<< Target >>
Line#:12 R.Time:9.092(Scan#:792) MassPeaks:6
RawMode:Averaged 9.083-9.100(791-793) BasePeak:93.05(2466)
BG Mode:None Group 1 - Event 1 Scan

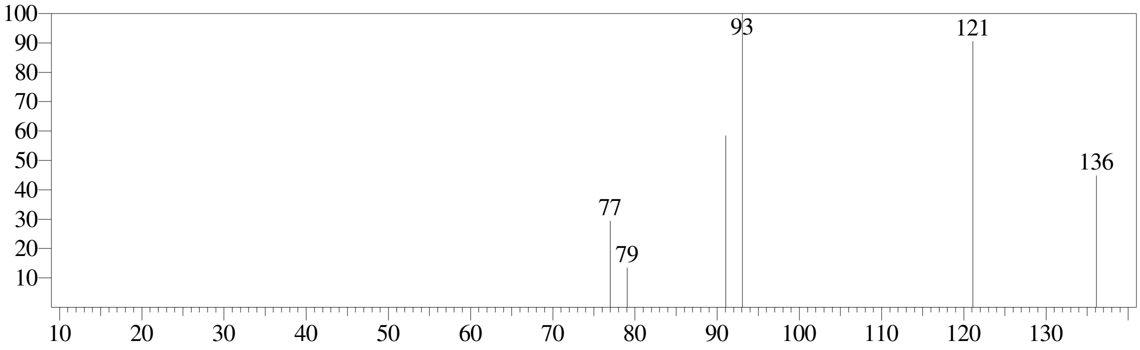

Hit#:1 Entry:8308 Library:NIST23s.lib
SI:90 Formula:C9H12O CAS:536-50-5 MolWeight:136 RetIndex:1149
CompName:Benzenemethanol, .alpha.,4-dimethyl- $$ Benzyl alcohol, p,.alpha.-dimethyl- $$ p-Tolylmethylcarbinol $$ p,.

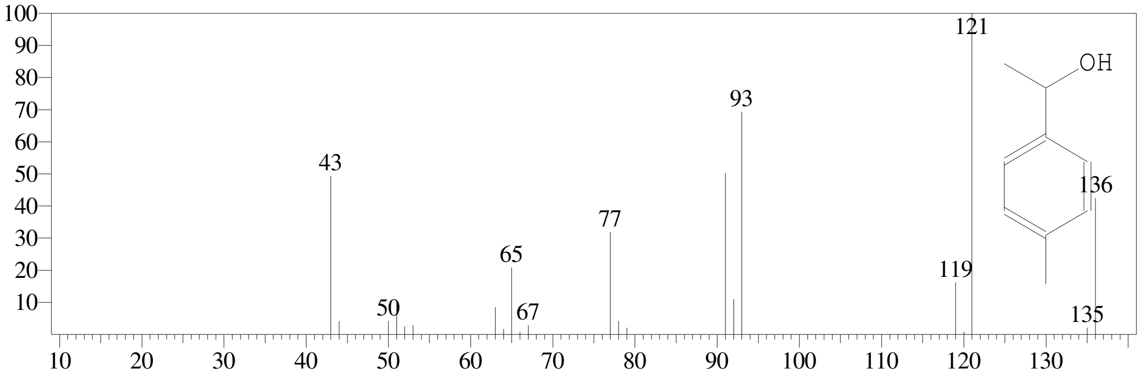

Hit#:2 Entry:11237 Library:NIST23-1.lib
SI:90 Formula:C9H12O CAS:29765-85-3 MolWeight:136 RetIndex:1116
CompName:2-Cyclopenten-1-one, 2,3,5-trimethyl-4-methylene- $$ 2,3,5-Trimethyl-4-methylene-2-cyclopenten-1-one # $

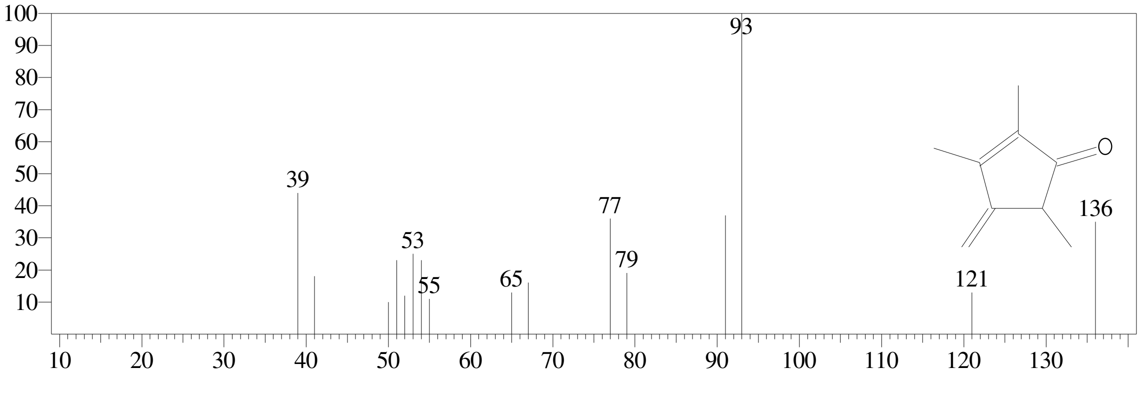

<< Target >>
Line#:12 R.Time:9.092(Scan#:792) MassPeaks:6
RawMode:Averaged 9.083-9.100(791-793) BasePeak:93.05(2466)
BG Mode:None Group 1 - Event 1 Scan

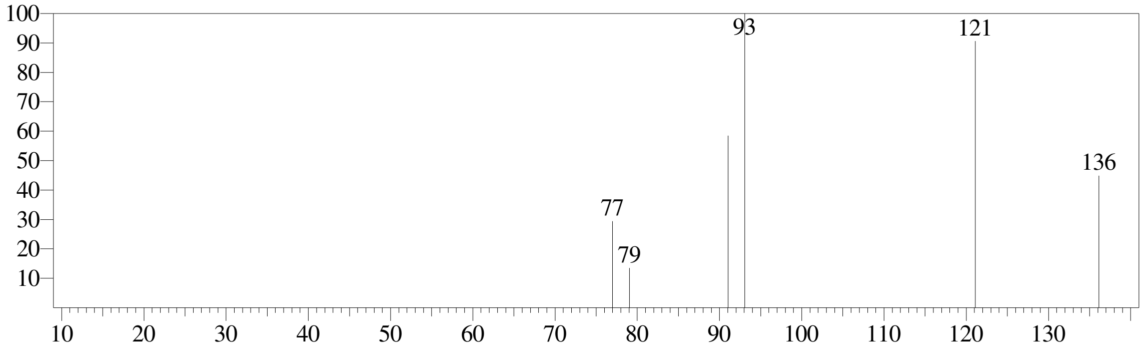

Hit#:3 Entry:8310 Library:NIST23s.lib
SI:86 Formula:C9H12O CAS:536-50-5 MolWeight:136 RetIndex:1149
CompName:Benzenemethanol, .alpha.,4-dimethyl- $$ Benzyl alcohol, p,.alpha.-dimethyl- $$ p-Tolylmethylcarbinol $$ p,.

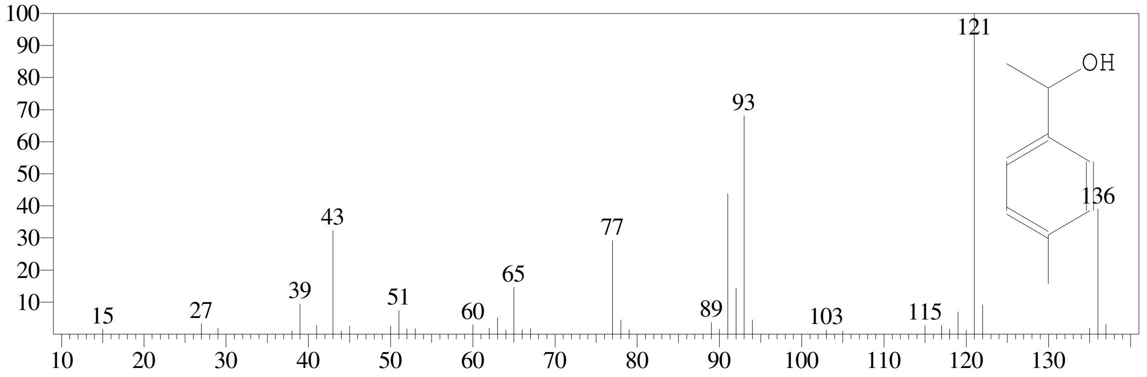

Hit#:4 Entry:11238 Library:NIST23-1.lib
SI:84 Formula:C9H12O CAS:0-00-0 MolWeight:136 RetIndex:1149
CompName:Methyl m-tolyl carbinol

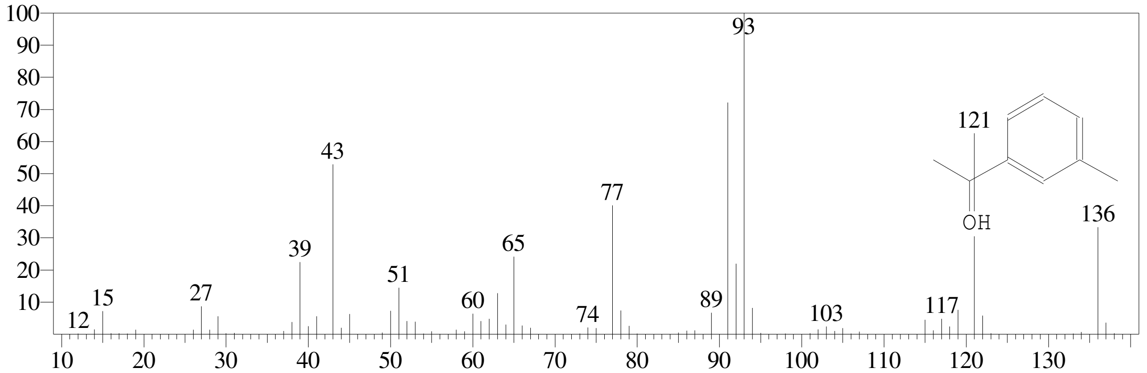

<< Target >>
Line#:12 R.Time:9.092(Scan#:792) MassPeaks:6
RawMode:Averaged 9.083-9.100(791-793) BasePeak:93.05(2466)
BG Mode:None Group 1 - Event 1 Scan

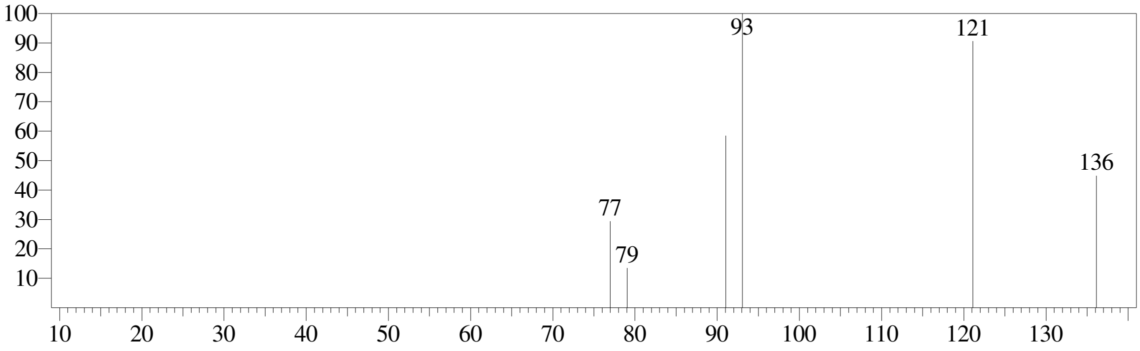

Hit#:5 Entry:11428 Library:NIST23-1.lib
SI:83 Formula:C10H16 CAS:29050-33-7 MolWeight:136 RetIndex:970
CompName:(+)-4-Carene $$ 4,7,7-Trimethylbicyclo[4.1.0]hept-2-ene # $$

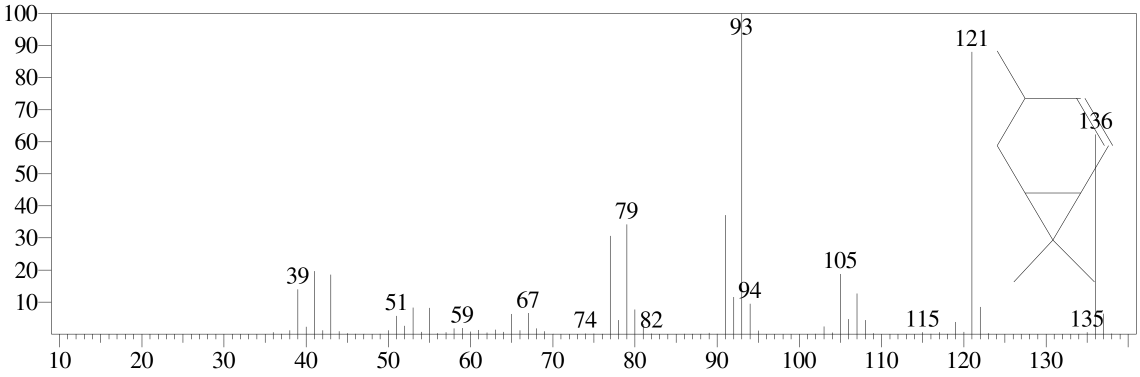

<< Target >>
Line#:13 R.Time:9.383(Scan#:827) MassPeaks:3
RawMode:Averaged 9.375-9.392(826-828) BasePeak:119.10(3604)
BG Mode:None Group 1 - Event 1 Scan

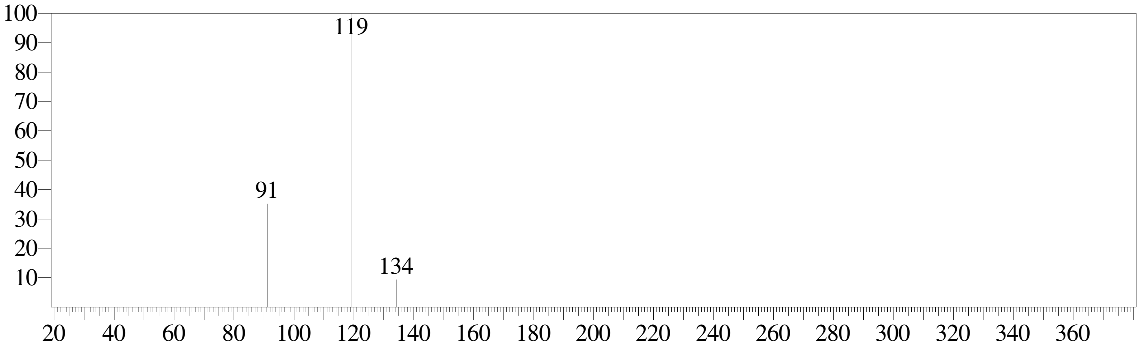

Hit#:1 Entry:19298 Library:NIST23-2.lib
SI:94 Formula:C19H15NO3S2 CAS:299929-13-8 MolWeight:369 RetIndex:3155
CompName:3-(4-Methylbenzoyl)-2-thioxo-4-thiazolyl 4-methylbenzoate $$ 3-(4-Methylbenzoyl)-2-thioxo-2,3-dihydro-1,

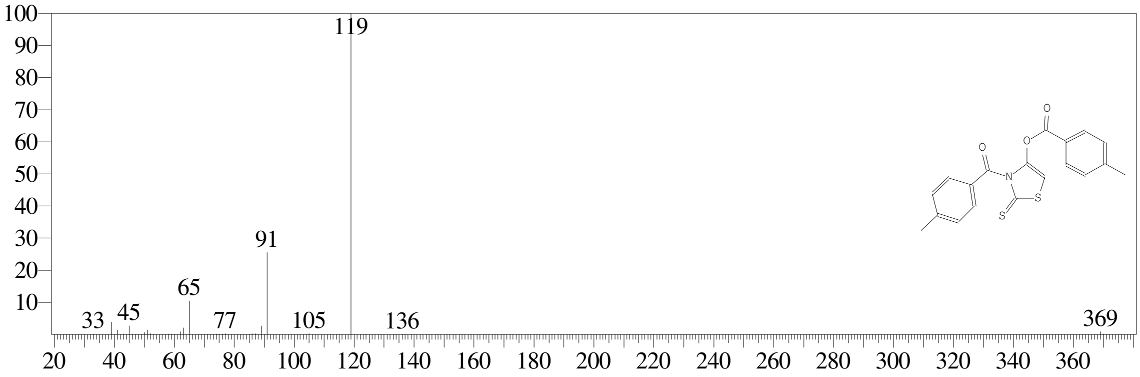

Hit#:2 Entry:95888 Library:NIST23-1.lib
SI:93 Formula:C12H11NO4 CAS:83039-57-0 MolWeight:233 RetIndex:2066
CompName:2,5-Pyrrolidinedione, 1-[(4-methylbenzoyl)oxy]-

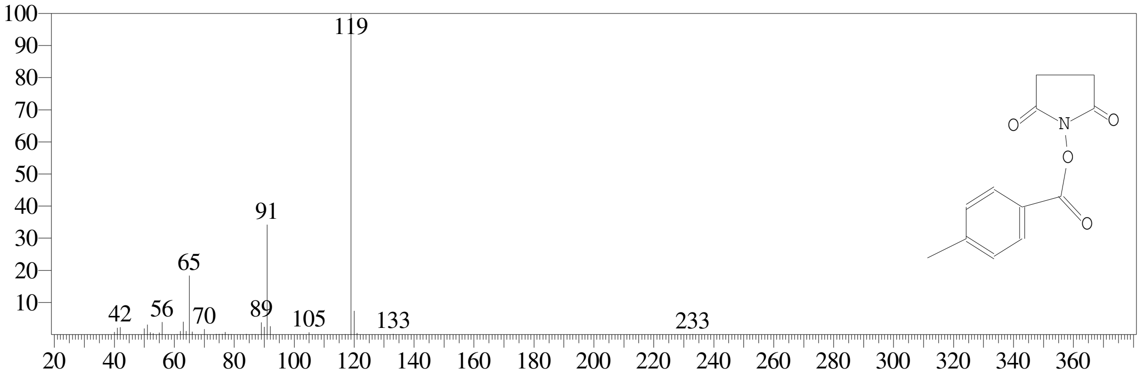

<< Target >>
Line#:13   R.Time:9.383(Scan#:827) MassPeaks:3
RawMode:Averaged 9.375-9.392(826-828) BasePeak:119.10(3604)
BG Mode:None Group 1 - Event 1 Scan

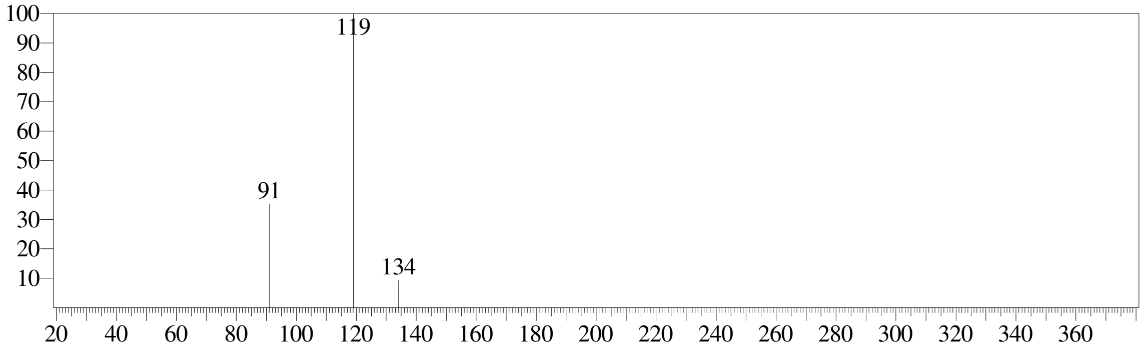

Hit#:3 Entry:234314 Library:NIST23-1.lib
SI:92 Formula:C18H13NO2S2 CAS:0-00-0 MolWeight:339 RetIndex:3102
CompName:5-Benzylidene-3-(p-toluoyl)rhodanine $$ (5Z)-5-Benzylidene-3-(4-methylbenzoyl)-2-thioxo-1,3-thiazolidin-4

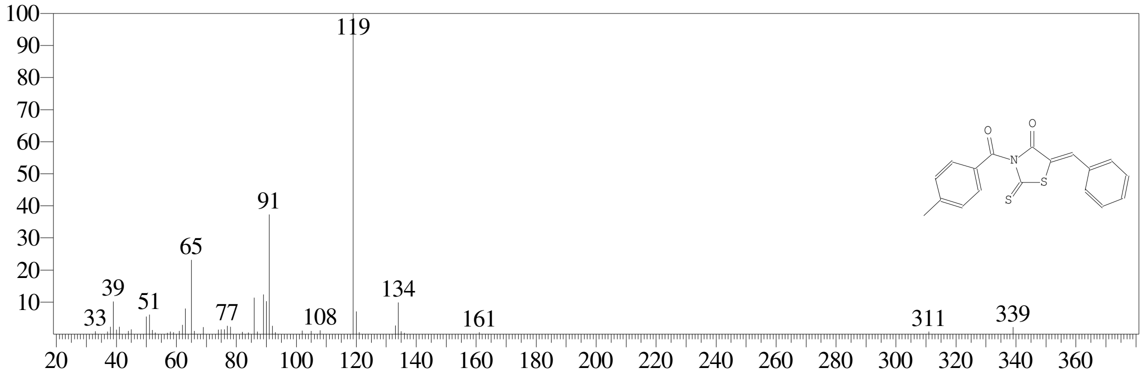

Hit#:4 Entry:220317 Library:NIST23-1.lib
SI:92 Formula:C16H19F3N2O2 CAS:303133-47-3 MolWeight:328 RetIndex:1948
CompName:2-Pyrazolin-5-ol, 5-tert-butyl-3-trifluoromethyl-1-(3-methylbenzoyl)- $$ 5-tert-Butyl-1-(3-methylbenzoyl)-3-

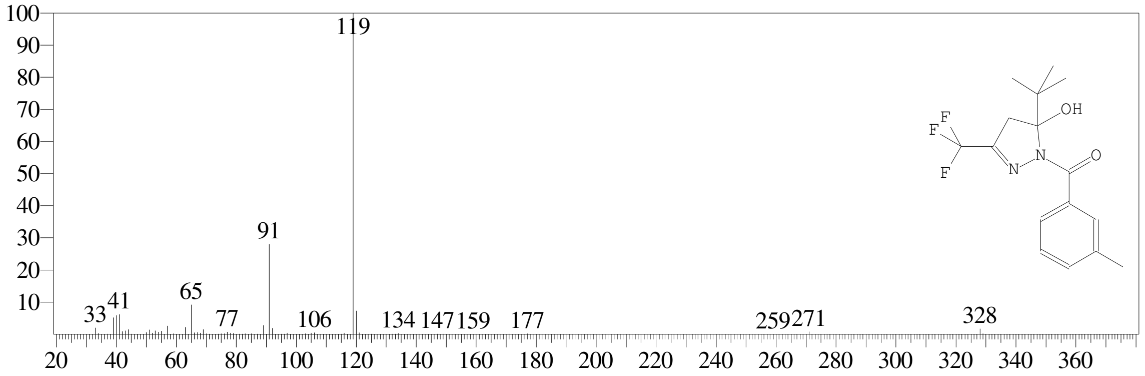

<< Target >>
Line#:13   R.Time:9.383(Scan#:827) MassPeaks:3
RawMode:Averaged 9.375-9.392(826-828) BasePeak:119.10(3604)
BG Mode:None Group 1 - Event 1 Scan

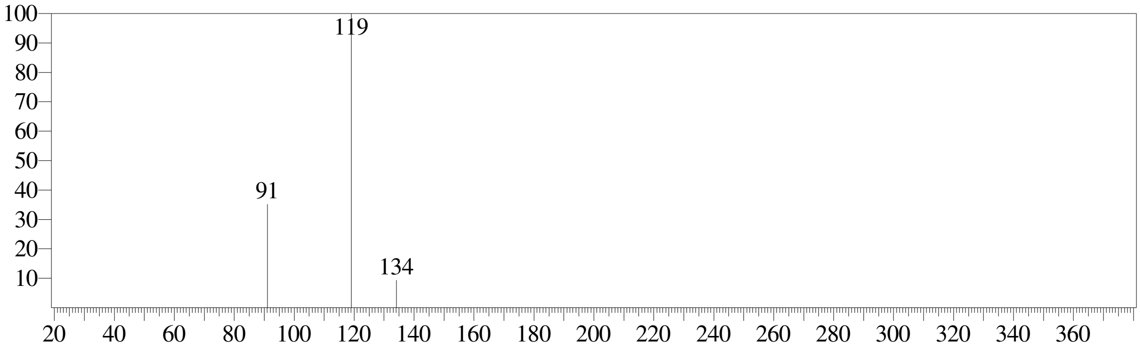

Hit#:5 Entry:101107 Library:NIST23-1.lib
SI:92 Formula:C15H11NO2 CAS:0-00-0 MolWeight:237 RetIndex:2054
CompName:m-Toluic acid, 4-cyanophenyl ester

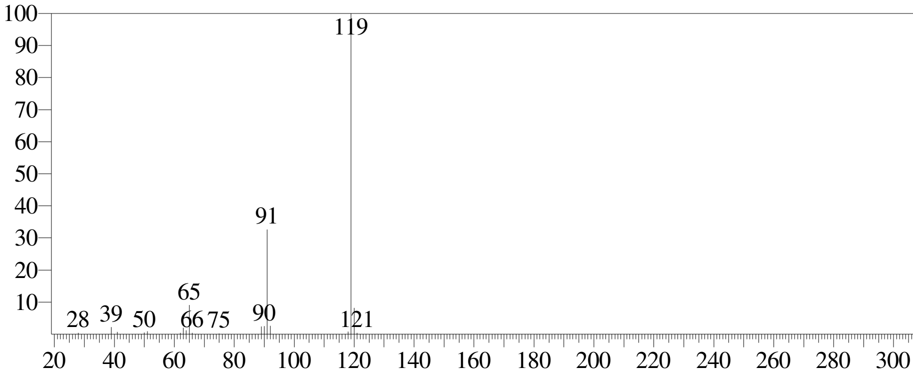

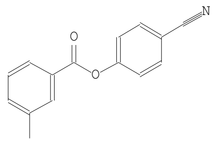

<< Target >>
Line#:14   R.Time:9.542(Scan#:846) MassPeaks:29
RawMode:Averaged 9.533-9.550(845-847) BasePeak:68.05(42489)
BG Mode:None Group 1 - Event 1 Scan

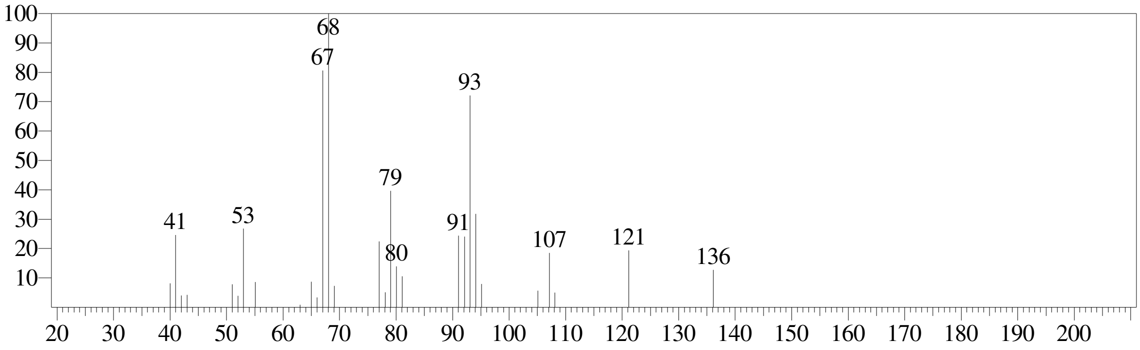

Hit#:1 Entry:8371 Library:NIST23s.lib
SI:95 Formula:C10H16 CAS:5989-27-5 MolWeight:136 RetIndex:1031
CompName:D-Limonene $$ Cyclohexene, 1-methyl-4-(1-methylethenyl)-, (R)- $$ p-Mentha-1,8-diene, (R)-(+)- $$ (+)-(R

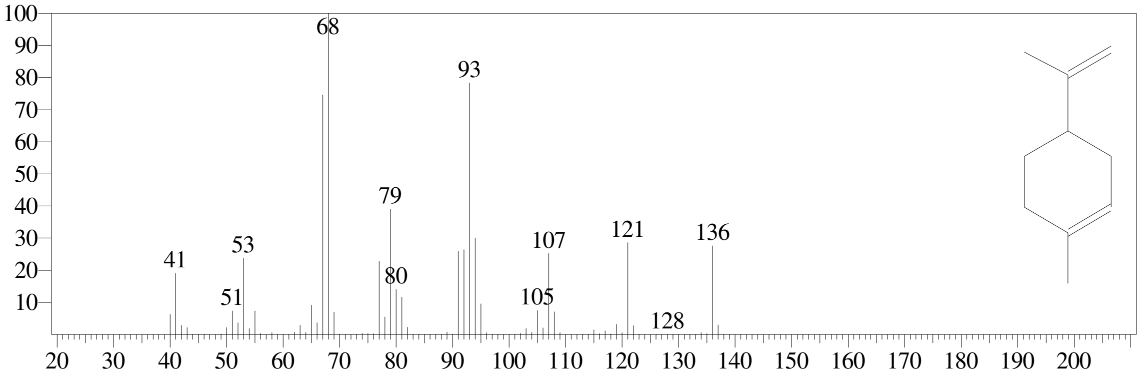

Hit#:2 Entry:8365 Library:NIST23s.lib
SI:93 Formula:C10H16 CAS:5989-54-8 MolWeight:136 RetIndex:1031
CompName:Cyclohexene, 1-methyl-4-(1-methylethenyl)-, (S)- $$ p-Mentha-1,8-diene, (S)-(-)- $$ (-)-Limonene $$ L-Lim

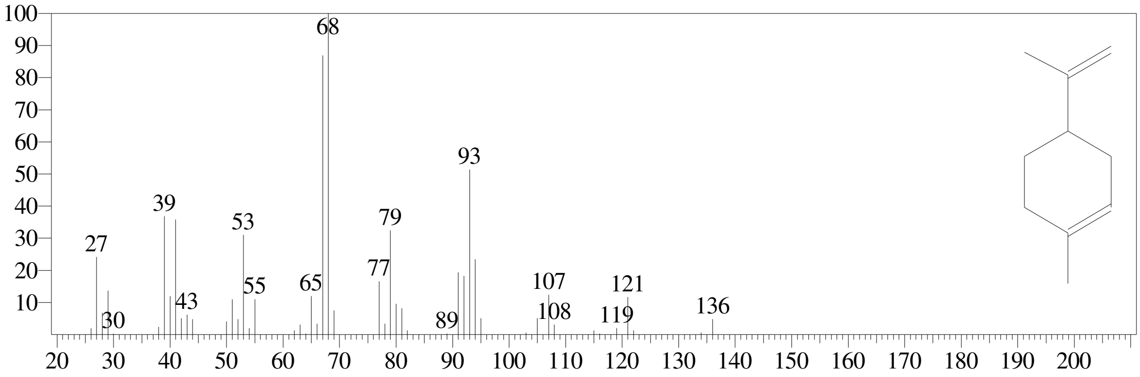

<< Target >>
Line#:14   R.Time:9.542(Scan#:846) MassPeaks:29
RawMode:Averaged 9.533-9.550(845-847) BasePeak:68.05(42489)
BG Mode:None Group 1 - Event 1 Scan

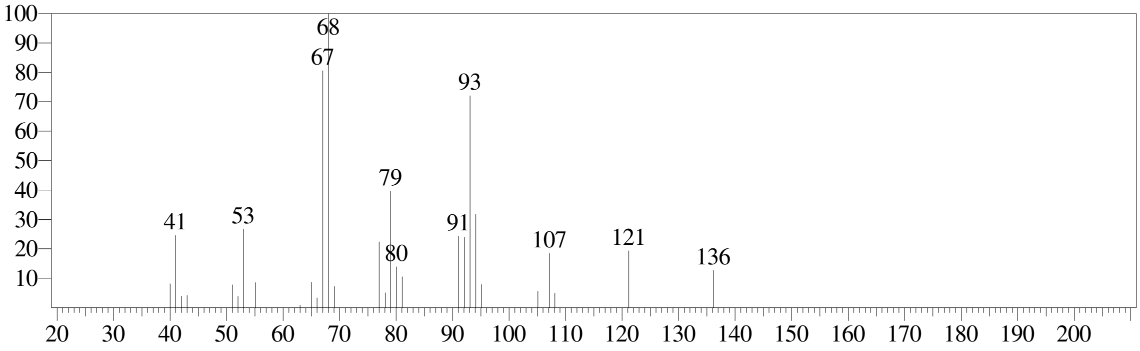

Hit#:3 Entry:11340 Library:NIST23-1.lib
SI:92 Formula:C10H16 CAS:138-86-3 MolWeight:136 RetIndex:1031
CompName:Limonene $$ Cyclohexene, 1-methyl-4-(1-methylethenyl)- $$ p-Mentha-1,8-diene $$ .alpha.-Limonene $$ C

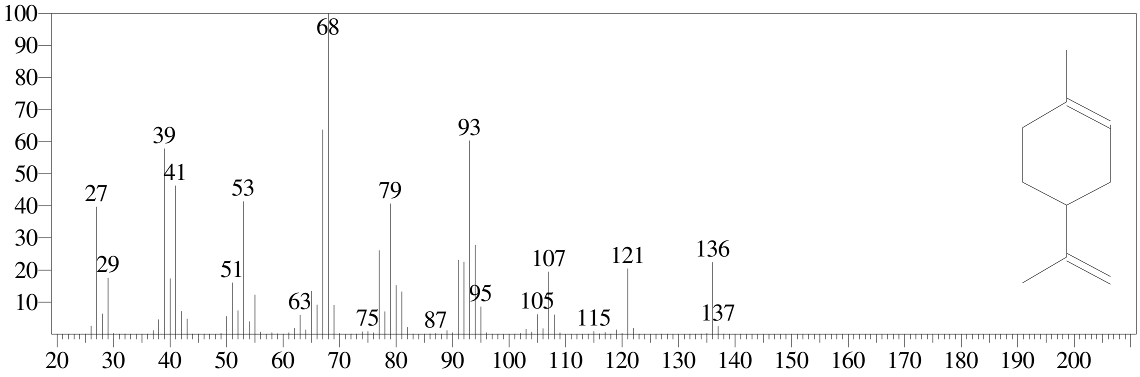

Hit#:4 Entry:11345 Library:NIST23-1.lib
SI:92 Formula:C10H16 CAS:19465-02-2 MolWeight:136 RetIndex:968
CompName:Cyclobutane, 1,2-bis(1-methylethenyl)-, trans- $$ 1,2-Diisopropenylcyclobutane # $$

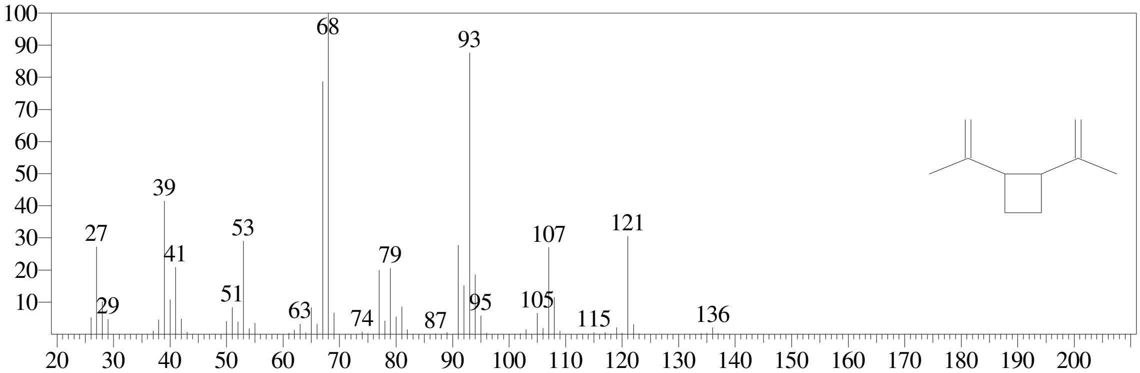

<< Target >>
Line#:14 R.Time:9.542(Scan#:846) MassPeaks:29
RawMode:Averaged 9.533-9.550(845-847) BasePeak:68.05(42489)
BG Mode:None Group 1 - Event 1 Scan

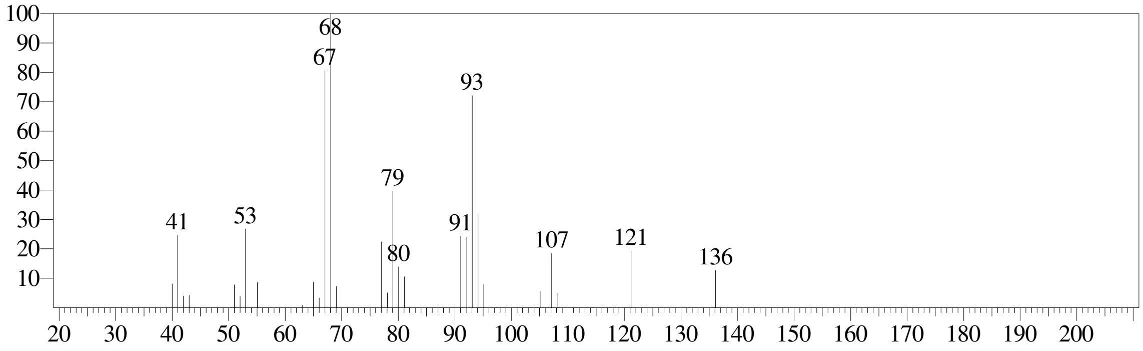

Hit#:5 Entry:8364 Library:NIST23s.lib
SI:92 Formula:C10H16 CAS:138-86-3 MolWeight:136 RetIndex:1031
CompName:Limonene $$ Cyclohexene, 1-methyl-4-(1-methylethenyl)- $$ p-Mentha-1,8-diene $$ .alpha.-Limonene $$ C[illegible]

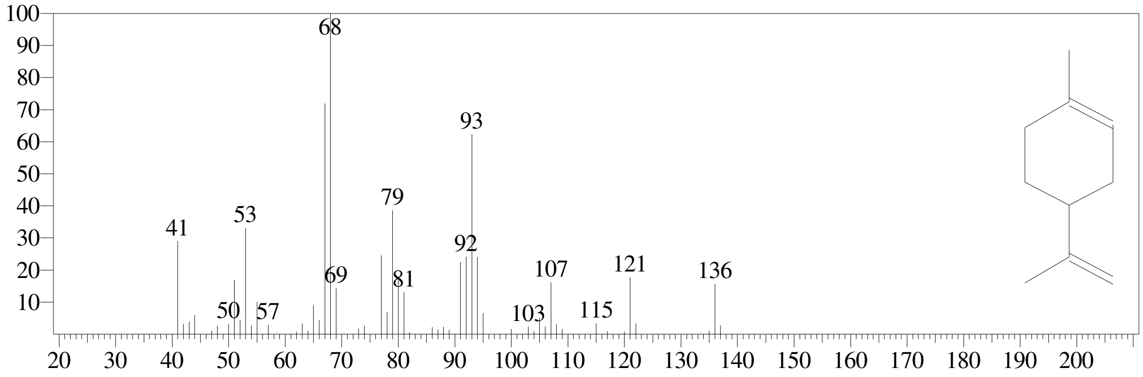

<< Target >>
Line#:15 R.Time:9.650(Scan#:859) MassPeaks:9
RawMode:Averaged 9.642-9.658(858-860) BasePeak:43.00(3122)
BG Mode:None Group 1 - Event 1 Scan

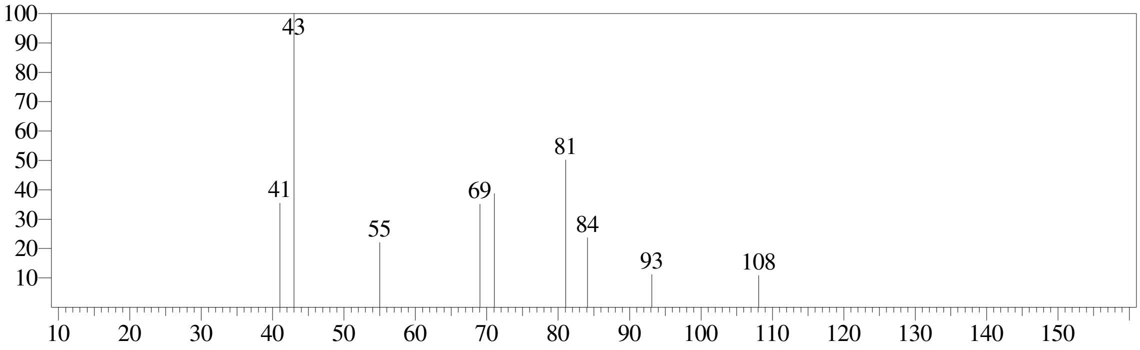

Hit#:1 Entry:17853 Library:NIST23-1.lib
SI:77 Formula:C5H11Br CAS:1809-10-5 MolWeight:150 RetIndex:827
CompName:Pentane, 3-bromo- $$ 3-Bromopentane $$ 3-Pentyl bromide $$

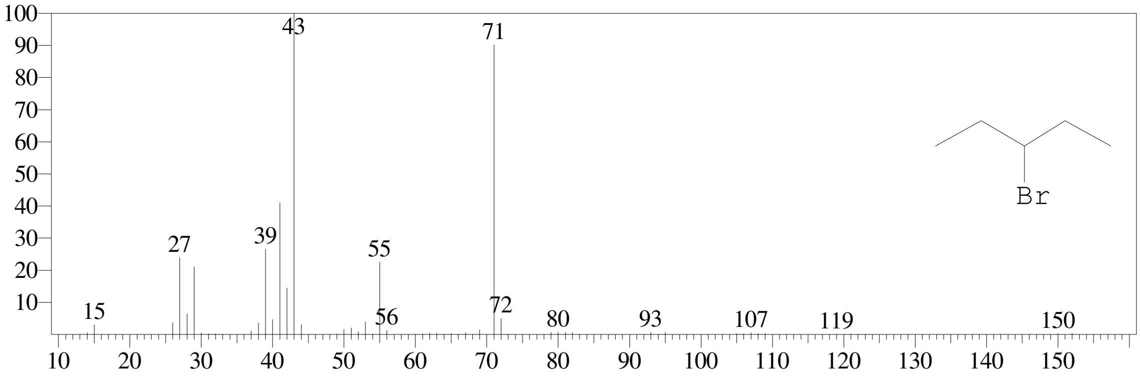

Hit#:2 Entry:5315 Library:NIST23-1.lib
SI:77 Formula:C5H11NO2 CAS:4609-89-6 MolWeight:117 RetIndex:861
CompName:Pentane, 2-nitro- $$ Pentane, 2-nitro $$ 2-Nitropentane # $$

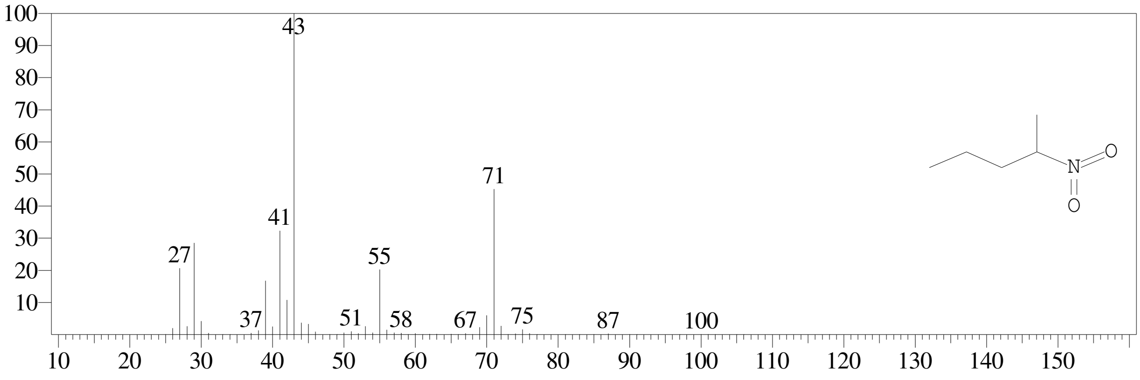

<< Target >>
Line#:15 R.Time:9.650(Scan#:859) MassPeaks:9
RawMode:Averaged 9.642-9.658(858-860) BasePeak:43.00(3122)
BG Mode:None Group 1 - Event 1 Scan

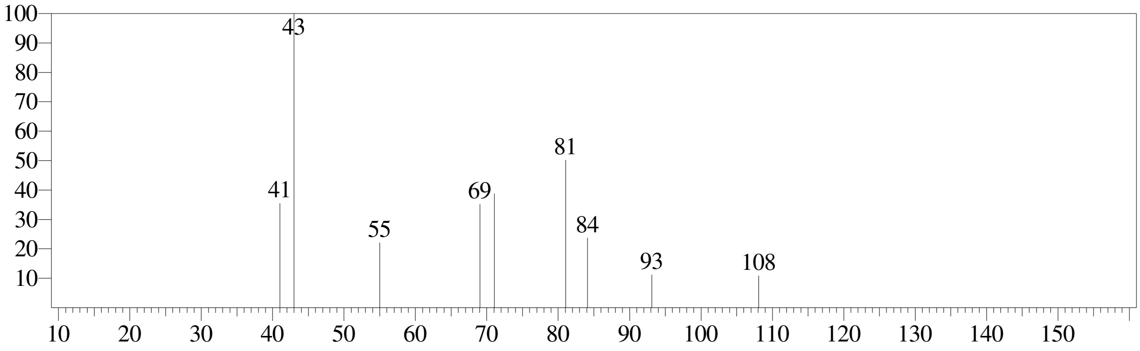

Hit#:3 Entry:3741 Library:NIST23s.lib
SI:76 Formula:C8H16 CAS:16106-59-5 MolWeight:112 RetIndex:745
CompName:1-Hexene, 4,5-dimethyl- $$ 4,5-Dimethyl-1-hexene $$ 4,5-Dimethylhex-1-ene $$

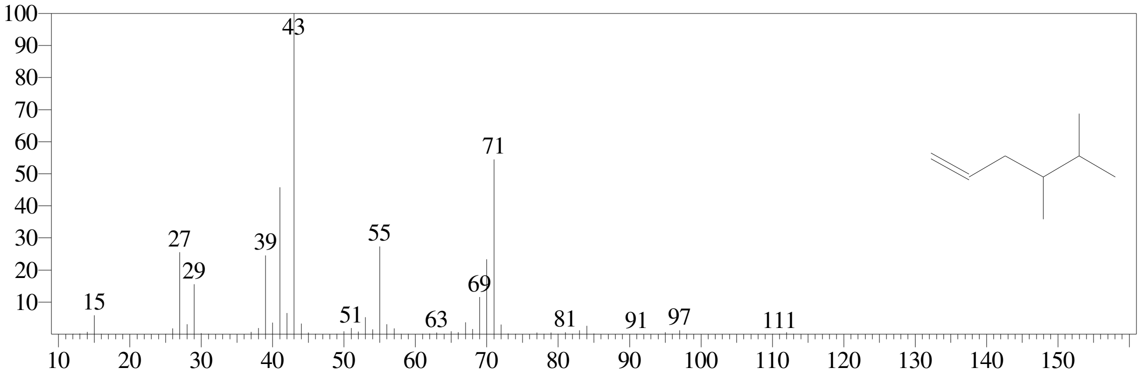

Hit#:4 Entry:2531 Library:NIST23-1.lib
SI:76 Formula:C6H14O CAS:105-30-6 MolWeight:102 RetIndex:827
CompName:1-Pentanol, 2-methyl- $$ 2-Methyl-1-pentanol $$ Isohexyl alcohol $$ 2-Methylpentanol-1 $$ M.I.B.C. $$ 2-M

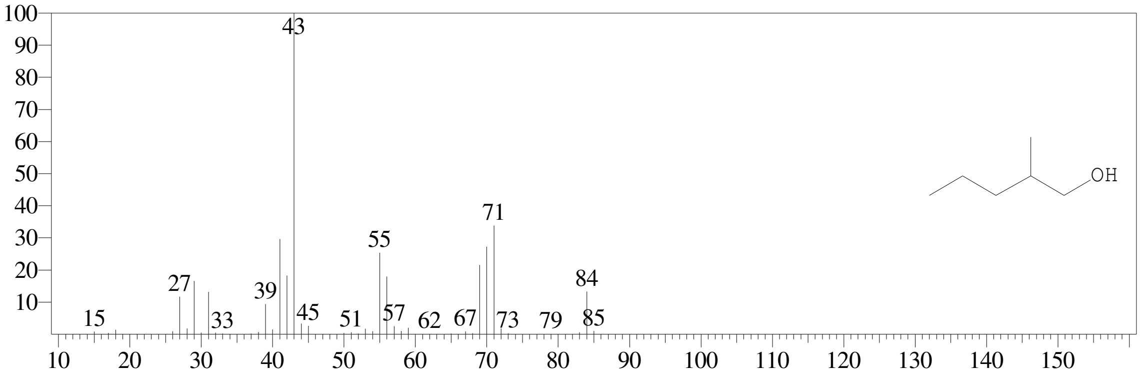

<< Target >>
Line#:15   R.Time:9.650(Scan#:859) MassPeaks:9
RawMode:Averaged 9.642-9.658(858-860) BasePeak:43.00(3122)
BG Mode:None Group 1 - Event 1 Scan

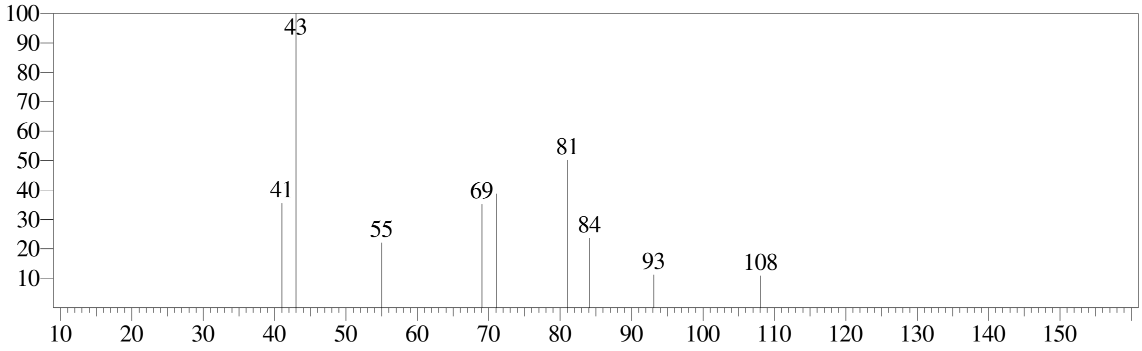

Hit#:5 Entry:11267 Library:NIST23s.lib
SI:76 Formula:C5H11Br CAS:1809-10-5 MolWeight:150 RetIndex:827
CompName:Pentane, 3-bromo- $$ 3-Bromopentane $$ 3-Pentyl bromide $$

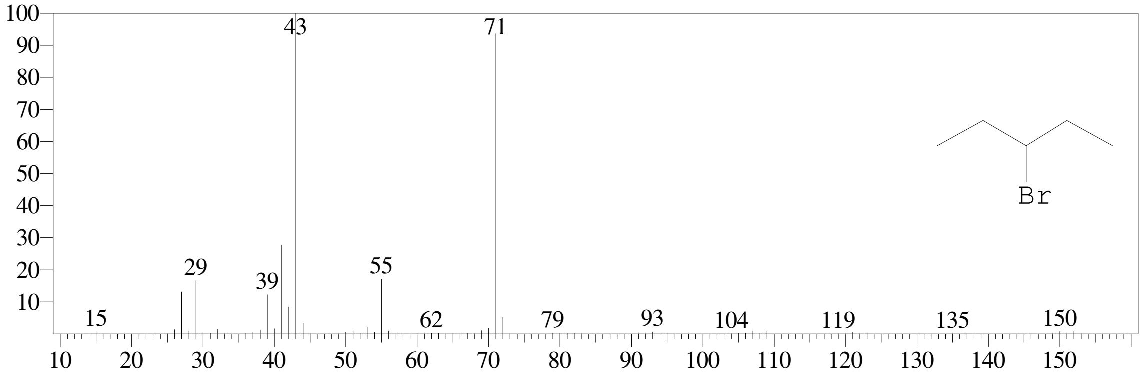

<< Target >>
Line#:16 R.Time:10.292(Scan#:936) MassPeaks:14
RawMode:Averaged 10.283-10.300(935-937) BasePeak:93.10(8676)
BG Mode:None Group 1 - Event 1 Scan

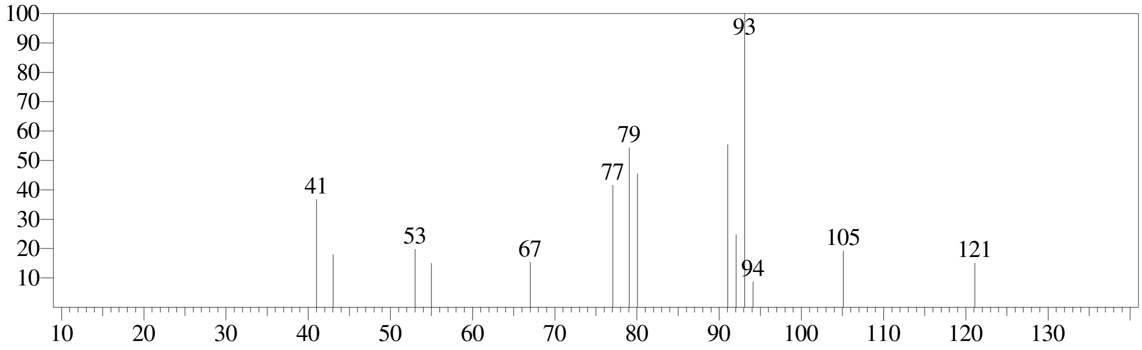

Hit#:1 Entry:8440 Library:NIST23s.lib
SI:88 Formula:C10H16 CAS:3338-55-4 MolWeight:136 RetIndex:1047
CompName:1,3,6-Octatriene, 3,7-dimethyl-, (Z)- $$ .beta.-cis-Ocimene $$ cis-.beta.-Ocimene $$ cis-3,7-Dimethyl-1,3,6-o

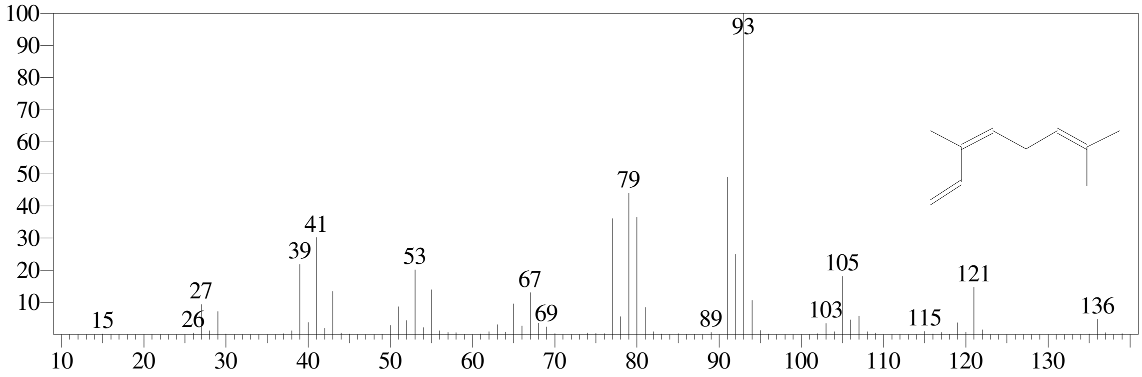

Hit#:2 Entry:11408 Library:NIST23-1.lib
SI:88 Formula:C10H16 CAS:13877-91-3 MolWeight:136 RetIndex:1047
CompName:.beta.-Ocimene $$ 1,3,6-Octatriene, 3,7-dimethyl- $$ Ocimene $$ 3,7-Dimethyl-1,3,6-octatriene $$ beta-Ocir

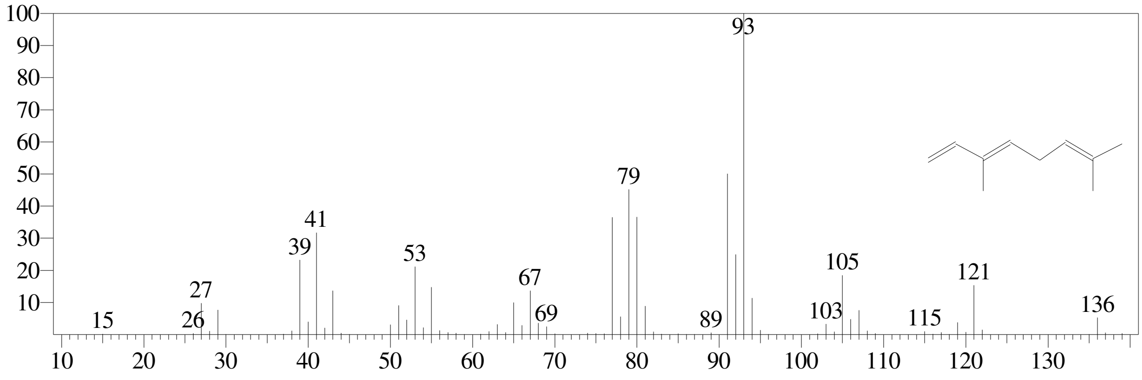

<< Target >>
Line#:16   R.Time:10.292(Scan#:936) MassPeaks:14
RawMode:Averaged 10.283-10.300(935-937) BasePeak:93.10(8676)
BG Mode:None Group 1 - Event 1 Scan

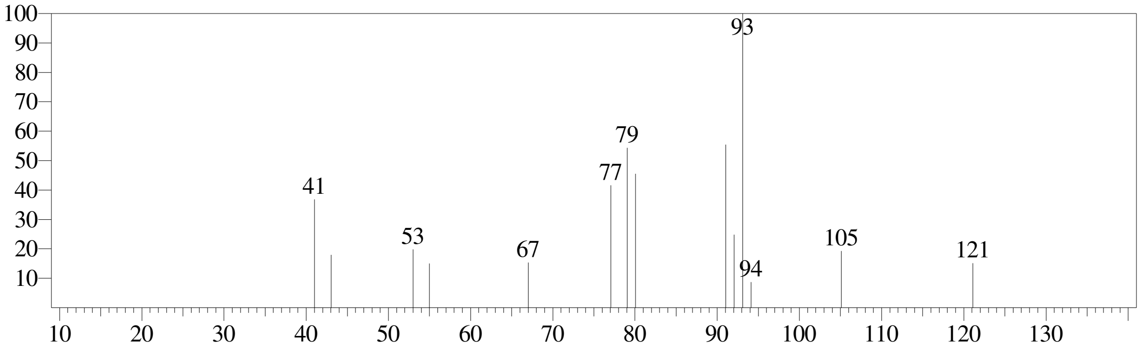

Hit#:3 Entry:8439 Library:NIST23s.lib
SI:87 Formula:C10H16 CAS:3779-61-1 MolWeight:136 RetIndex:1047
CompName:trans-.beta.-Ocimene $$ 1,3,6-Octatriene, 3,7-dimethyl-, (E)- $$ .beta.-trans-Ocimene $$ trans-3,7-Dimethyl-

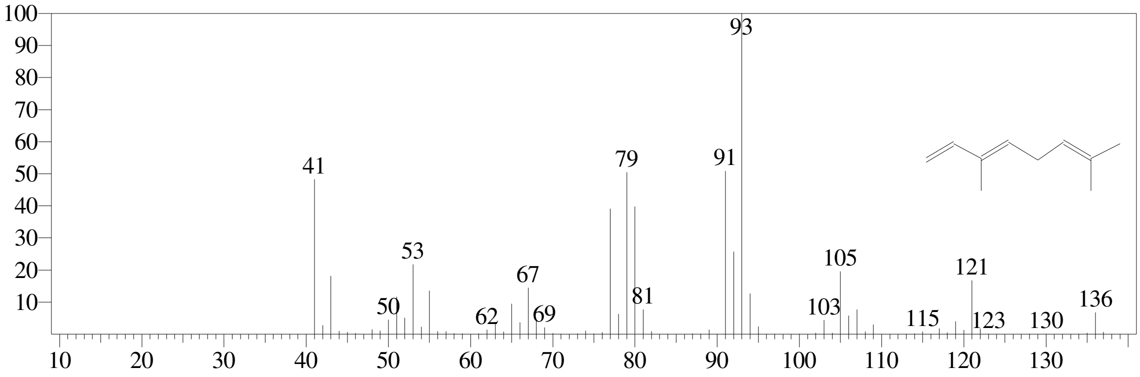

Hit#:4 Entry:11381 Library:NIST23-1.lib
SI:86 Formula:C10H16 CAS:502-99-8 MolWeight:136 RetIndex:1029
CompName:1,3,7-Octatriene, 3,7-dimethyl- $$ 2,6-Dimethyl-1,5,7-octatriene $$ 3,7-Dimethyl-1,3,7-octatriene # $$ .alph

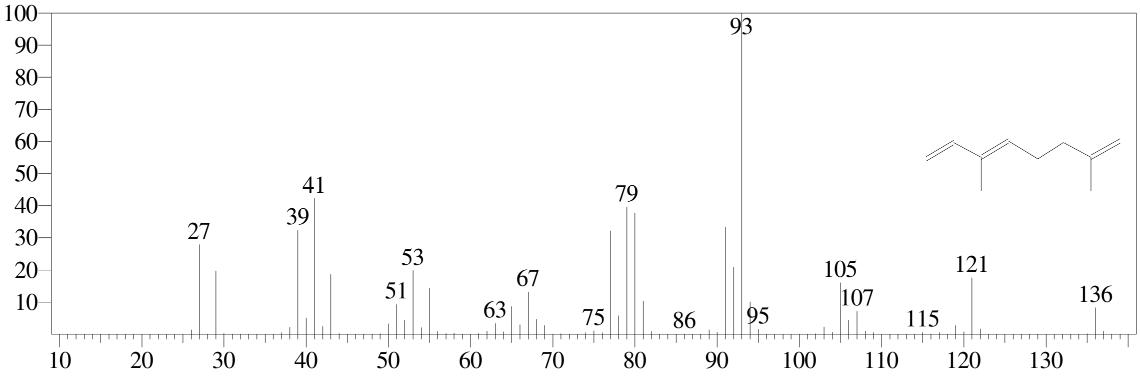

<< Target >>
Line#:16 R.Time:10.292(Scan#:936) MassPeaks:14
RawMode:Averaged 10.283-10.300(935-937) BasePeak:93.10(8676)
BG Mode:None Group 1 - Event 1 Scan

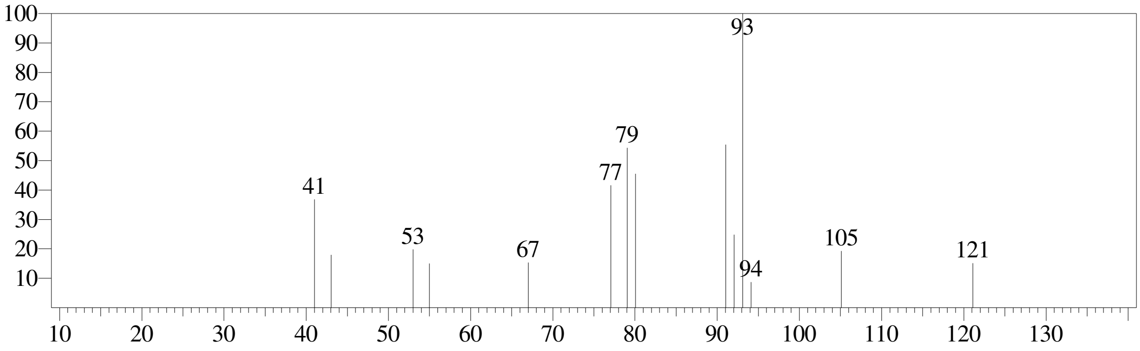

Hit#:5 Entry:8399 Library:NIST23s.lib
SI:86 Formula:C10H16 CAS:3338-55-4 MolWeight:136 RetIndex:1047
CompName:1,3,6-Octatriene, 3,7-dimethyl-, (Z)- $$ .beta.-cis-Ocimene $$ cis-.beta.-Ocimene $$ cis-3,7-Dimethyl-1,3,6-o

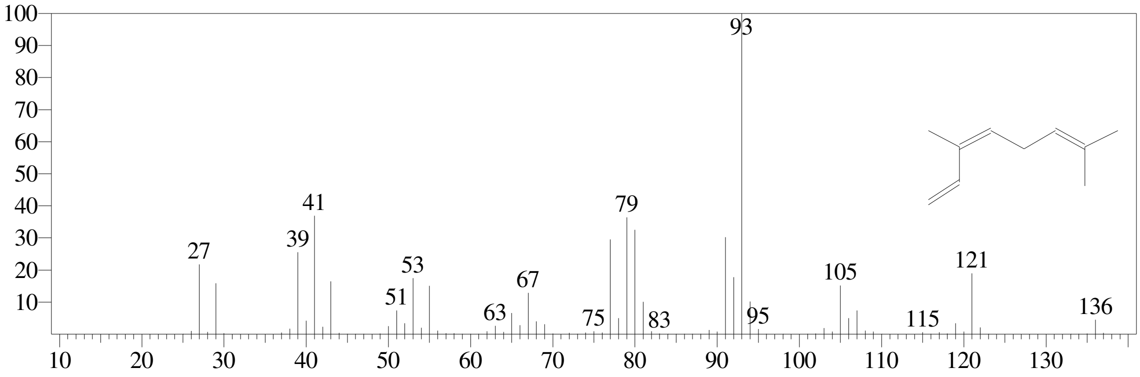

<< Target >>
Line#:17   R.Time:10.725(Scan#:988) MassPeaks:2
RawMode:Averaged 10.717-10.733(987-989) BasePeak:93.05(1702)
BG Mode:None Group 1 - Event 1 Scan

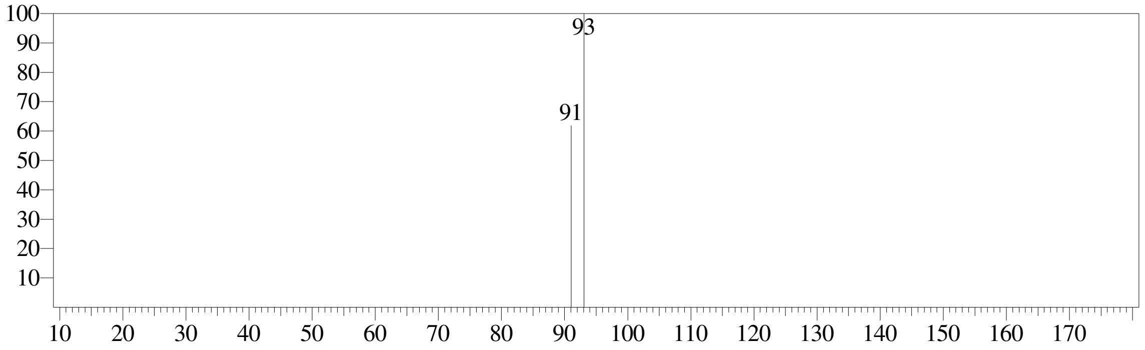

Hit#:1 Entry:33334 Library:NIST23-1.lib
SI:95 Formula:C7H9Br CAS:0-00-0 MolWeight:172 RetIndex:1083
CompName:Nortricyclyl bromide

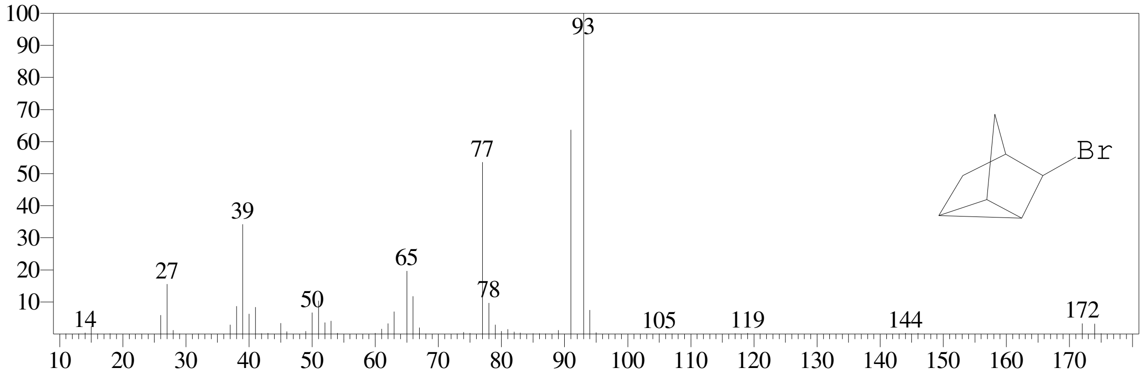

Hit#:2 Entry:3165 Library:NIST23-1.lib
SI:92 Formula:C8H12 CAS:0-00-0 MolWeight:108 RetIndex:870
CompName:Spiro[bicyclo[3.1.0]hexane-2,1'-cyclopropane]

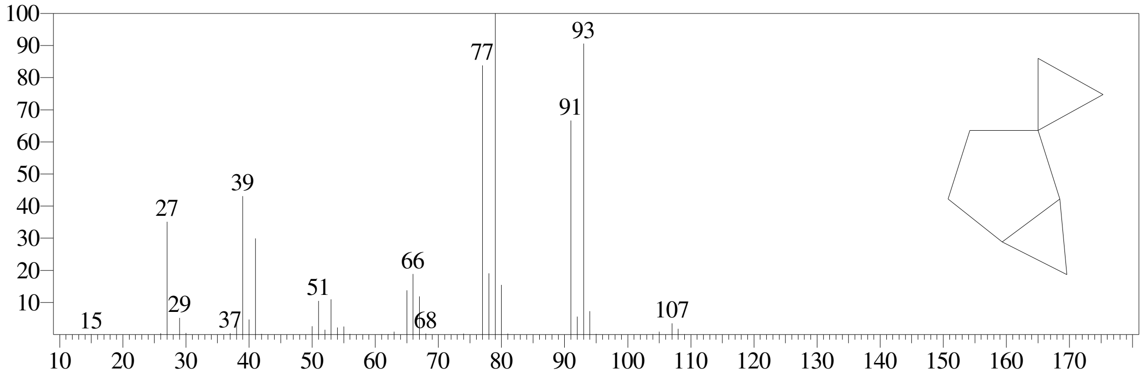

<< Target >>
Line#:17   R.Time:10.725(Scan#:988) MassPeaks:2
RawMode:Averaged 10.717-10.733(987-989) BasePeak:93.05(1702)
BG Mode:None Group 1 - Event 1 Scan

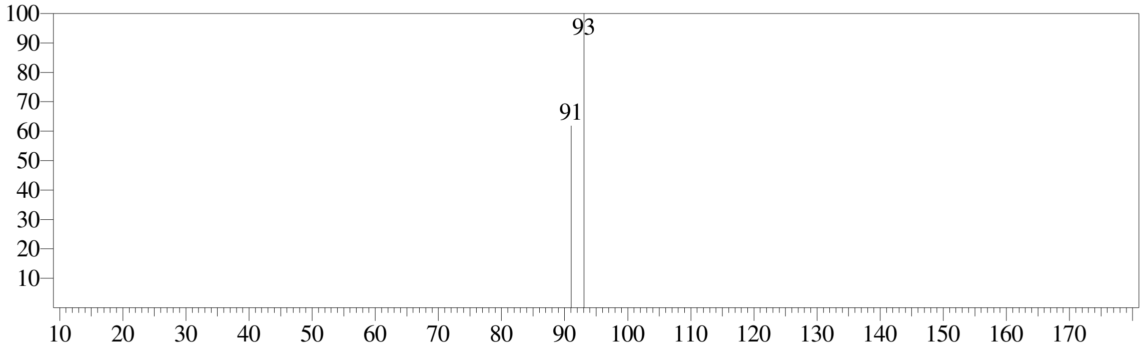

Hit#:3 Entry:3140 Library:NIST23-1.lib
SI:92 Formula:C8H12 CAS:70970-60-4 MolWeight:108 RetIndex:890
CompName:Tricyclo[4.1.1.0(2,5)]octane

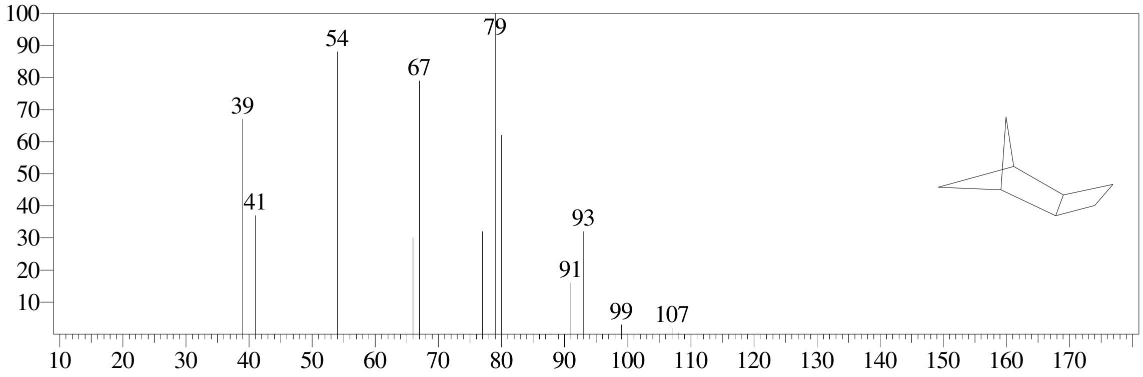

Hit#:4 Entry:11134 Library:NIST23-1.lib
SI:91 Formula:C8H12N2 CAS:109746-10-3 MolWeight:136 RetIndex:1191
CompName:1,4-Methano-1H-Cyclopropa[d]pyridazine, 4,4a,5,5a-tetrahydro-6,6-dimethyl-, (1.alpha.,4.alpha.,4a.alpha.,5a

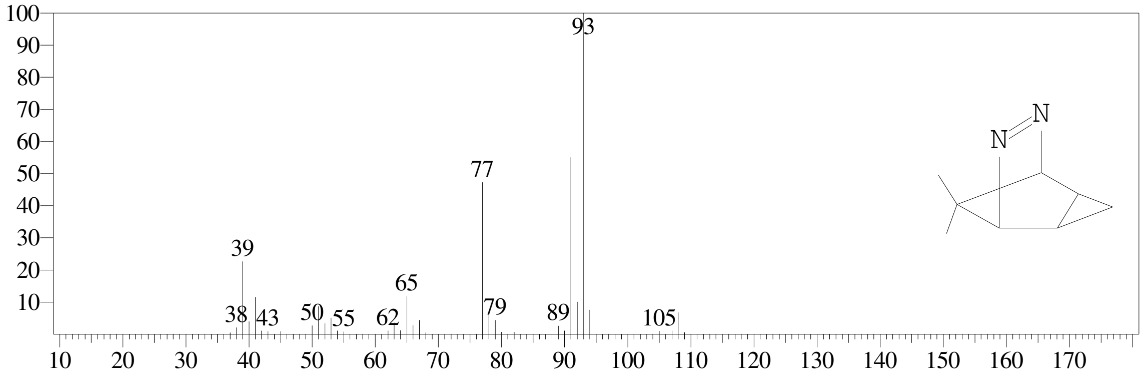

<< Target >>
Line#:17   R.Time:10.725(Scan#:988) MassPeaks:2
RawMode:Averaged 10.717-10.733(987-989) BasePeak:93.05(1702)
BG Mode:None Group 1 - Event 1 Scan

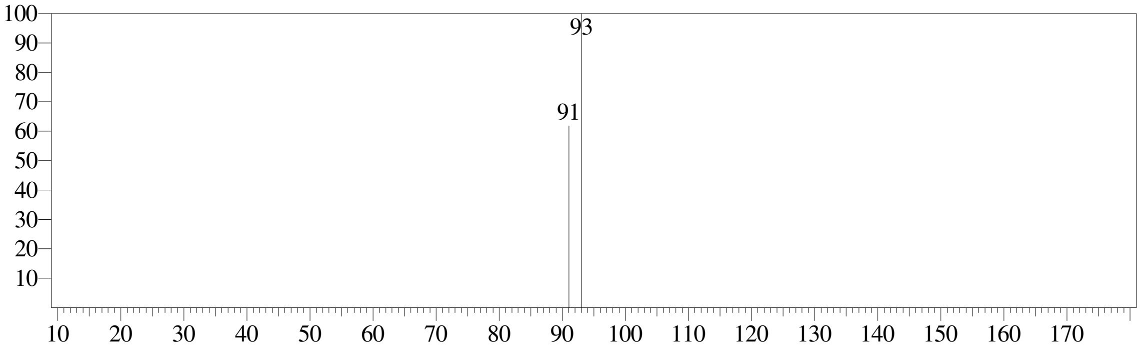

Hit#:5 Entry:3188 Library:NIST23-1.lib
SI:91 Formula:C8H12 CAS:61142-26-5 MolWeight:108 RetIndex:813
CompName:Cyclopropene, 1-methyl-3-(2-methylcyclopropyl)- $$ 1-Methyl-3-(2-methylcyclopropyl)-1-cyclopropene # $

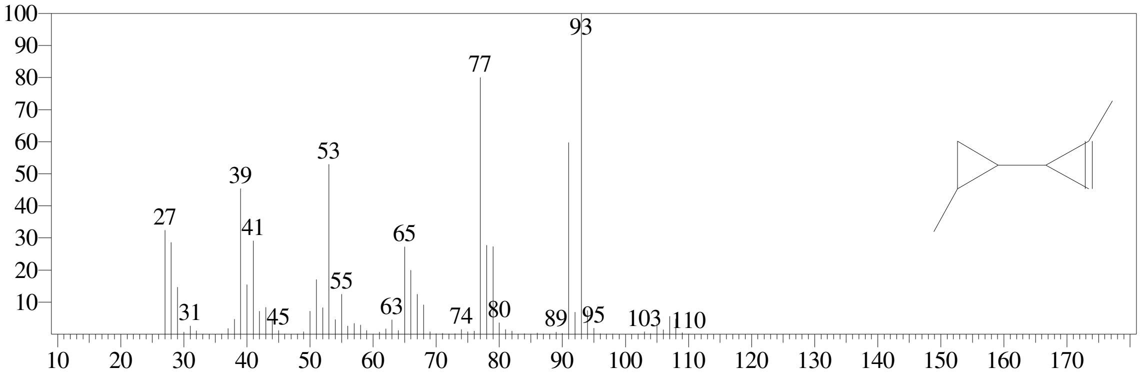

<< Target >>
Line#:18 R.Time:13.408(Scan#:1310) MassPeaks:1
RawMode:Averaged 13.400-13.417(1309-1311) BasePeak:81.05(1999)
BG Mode:None Group 1 - Event 1 Scan

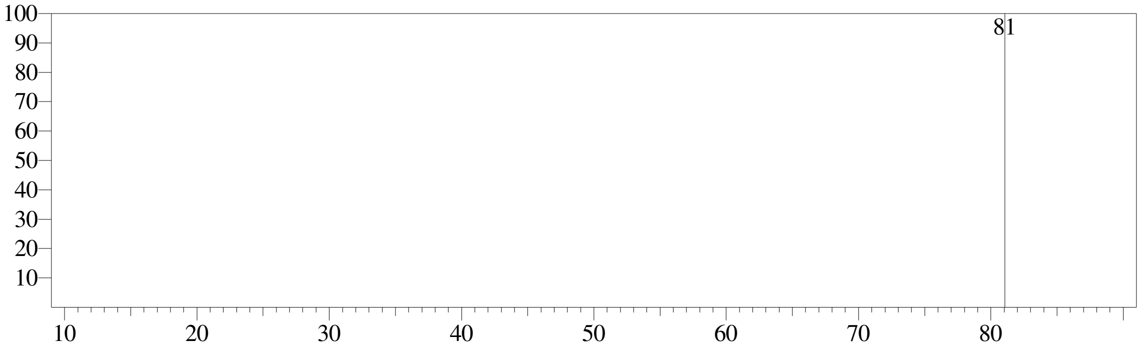

Hit#:1 Entry:722 Library:NIST23s.lib
SI:100 Formula:B3H6N3 CAS:6569-51-3 MolWeight:81 RetIndex:1132
CompName:s-Triazaborane $$ Borazine $$ s-Triazatriborine, hexahydro- $$ Borazole $$ Borazyne, cyclic trimer $$ Hexa

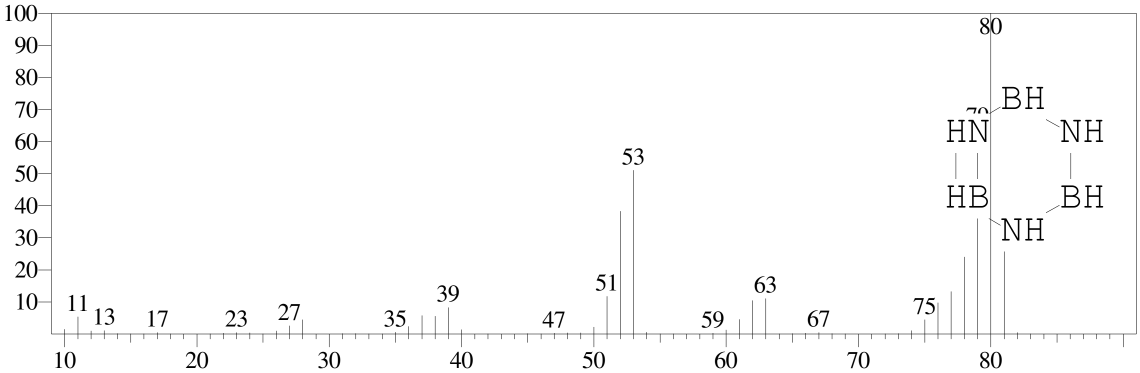

Hit#:2 Entry:544 Library:NIST23-1.lib
SI:100 Formula:B3H6N3 CAS:6569-51-3 MolWeight:81 RetIndex:1132
CompName:s-Triazaborane $$ Borazine $$ s-Triazatriborine, hexahydro- $$ Borazole $$ Borazyne, cyclic trimer $$ Hexa

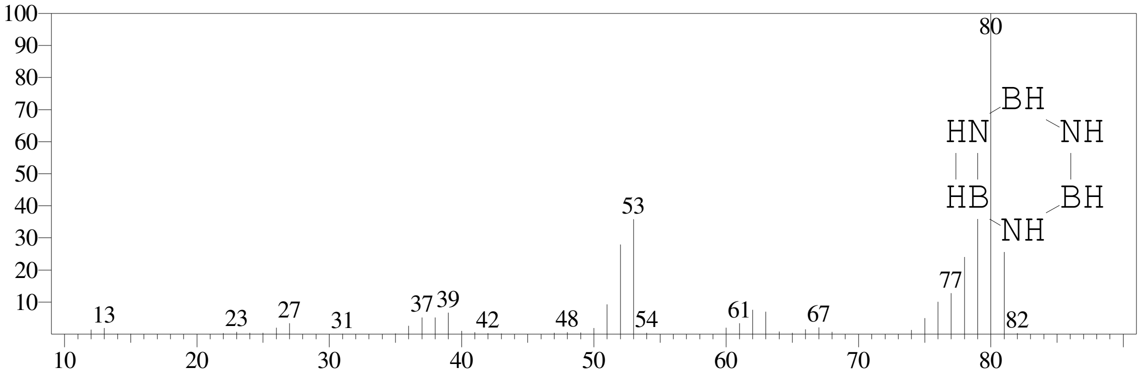

<< Target >>
Line#:18 R.Time:13.408(Scan#:1310) MassPeaks:1
RawMode:Averaged 13.400-13.417(1309-1311) BasePeak:81.05(1999)
BG Mode:None Group 1 - Event 1 Scan

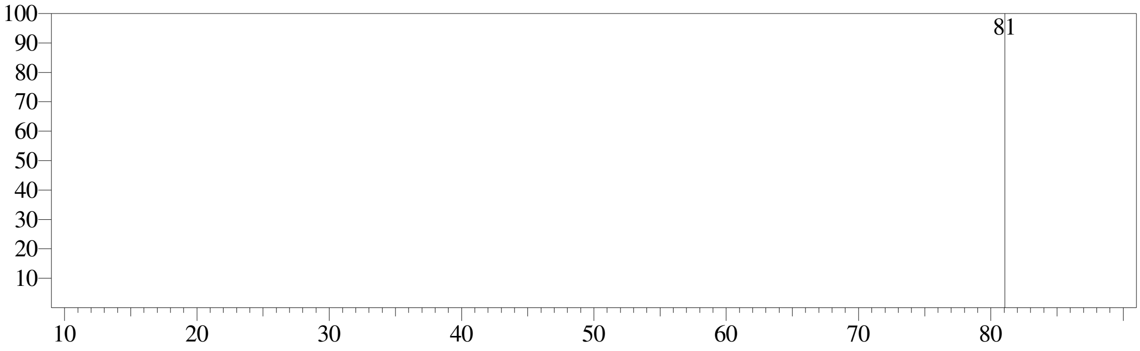

Hit#:3 Entry:561 Library:NIST23-1.lib
SI:98 Formula:C5H7N CAS:636-41-9 MolWeight:81 RetIndex:822
CompName:1H-Pyrrole, 2-methyl- $$ Pyrrole, 2-methyl- $$ .alpha.-Methylpyrrole $$ 2-Methylpyrrole $$ 2-Methyl-1H-p

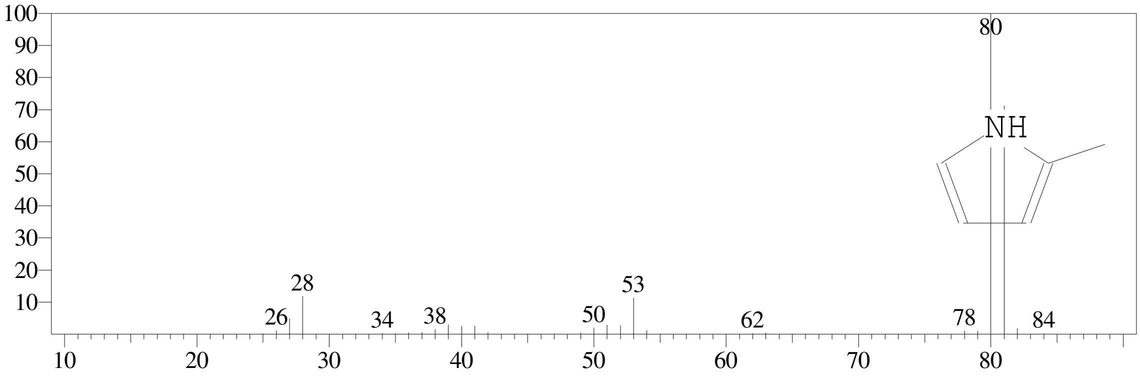

Hit#:4 Entry:547 Library:NIST23-1.lib
SI:98 Formula:C3H3N3 CAS:290-87-9 MolWeight:81 RetIndex:770
CompName:1,3,5-Triazine $$ s-Triazine $$ Cyanidine $$ Vedita 250 $$ s-Triazine-(1,3,5) $$ sym-Triazine $$

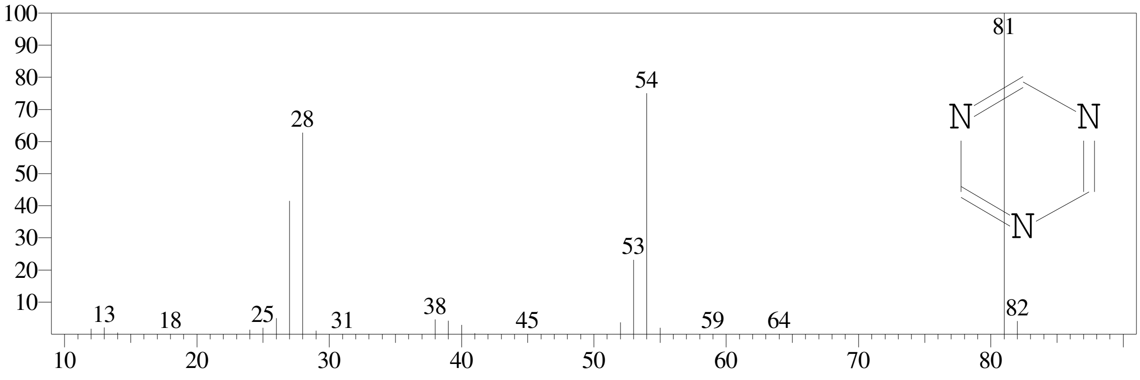

<< Target >>
Line#:18   R.Time:13.408(Scan#:1310) MassPeaks:1
RawMode:Averaged 13.400-13.417(1309-1311) BasePeak:81.05(1999)
BG Mode:None Group 1 - Event 1 Scan

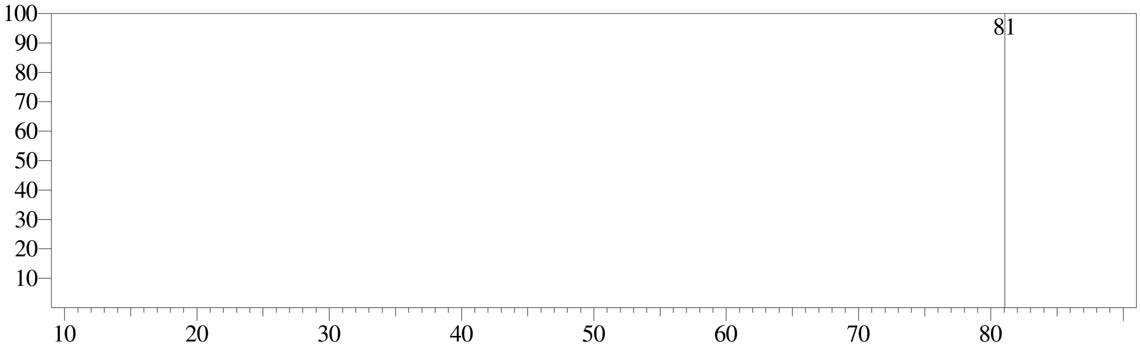

Hit#:5 Entry:732 Library:NIST23s.lib
SI:98 Formula:C5H7N CAS:13284-42-9 MolWeight:81 RetIndex:771
CompName:2-Pentenenitrile $$ 1-Cyano-1-butene $$ 2-Pentenonitrile $$ Pent-2-enenitrile $$

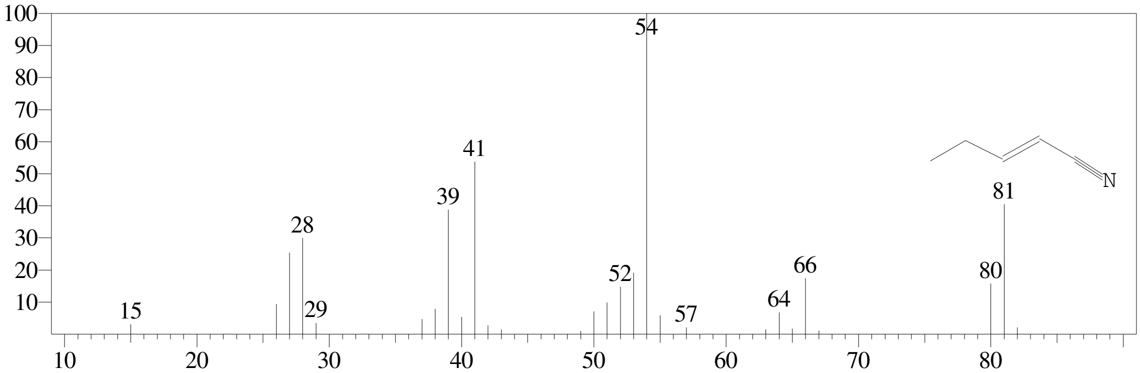

<< Target >>
Line#:19 R.Time:15.100(Scan#:1513) MassPeaks:10
RawMode:Averaged 15.092-15.108(1512-1514) BasePeak:95.10(6207)
BG Mode:None Group 1 - Event 1 Scan

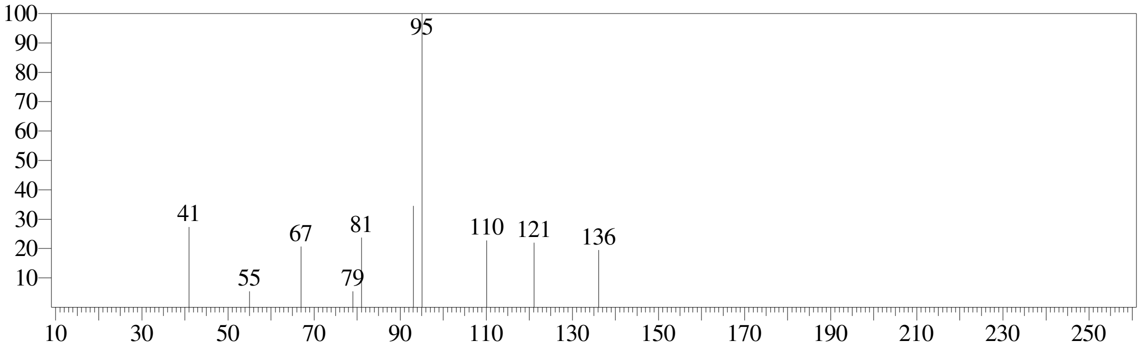

Hit#:1 Entry:11436 Library:NIST23-1.lib
SI:74 Formula:C10H16 CAS:2633-80-9 MolWeight:136 RetIndex:1023
CompName:Bicyclo[2.2.1]heptane, 2-(2-propenyl)- $$ 2-Allylbicyclo[2.2.1]heptane # $$

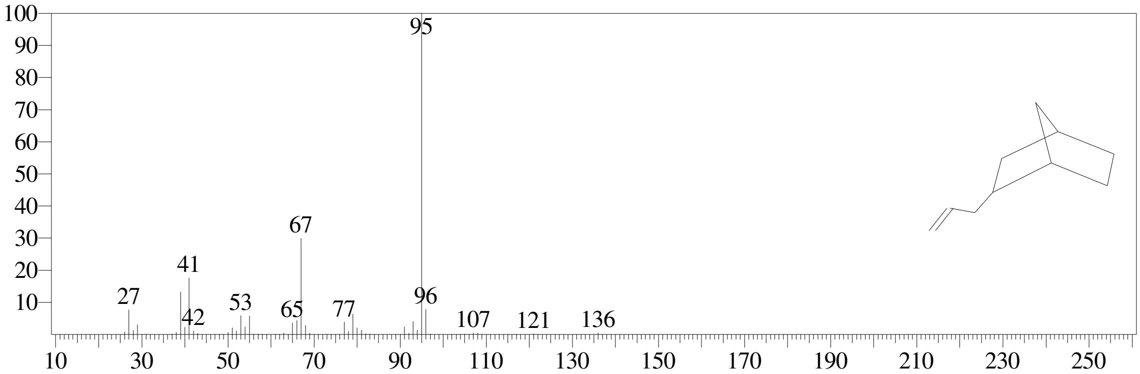

Hit#:2 Entry:116904 Library:NIST23-1.lib
SI:74 Formula:C12H17F3O2 CAS:28587-55-5 MolWeight:250 RetIndex:1141
CompName:Borneol, trifluoroacetate (ester) $$ 1,7,7-Trimethylbicyclo[2.2.1]hept-2-yl trifluoroacetate # $$

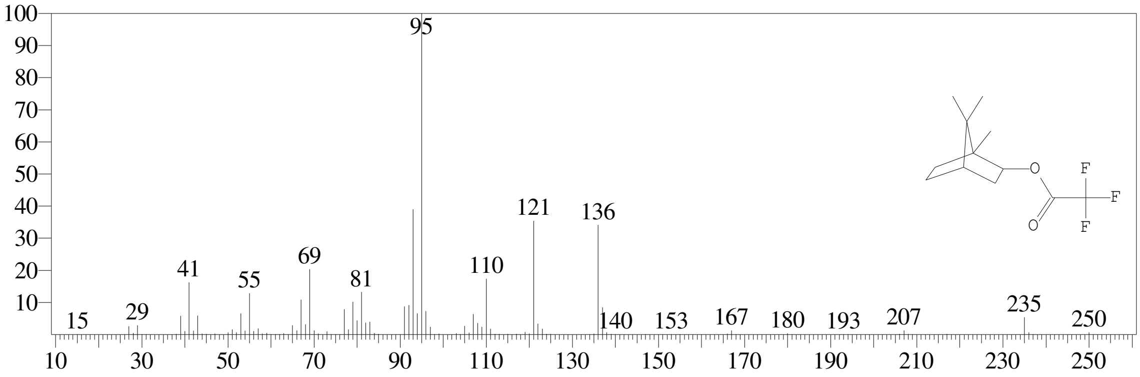

<< Target >>
Line#:19   R.Time:15.100(Scan#:1513) MassPeaks:10
RawMode:Averaged 15.092-15.108(1512-1514) BasePeak:95.10(6207)
BG Mode:None Group 1 - Event 1 Scan

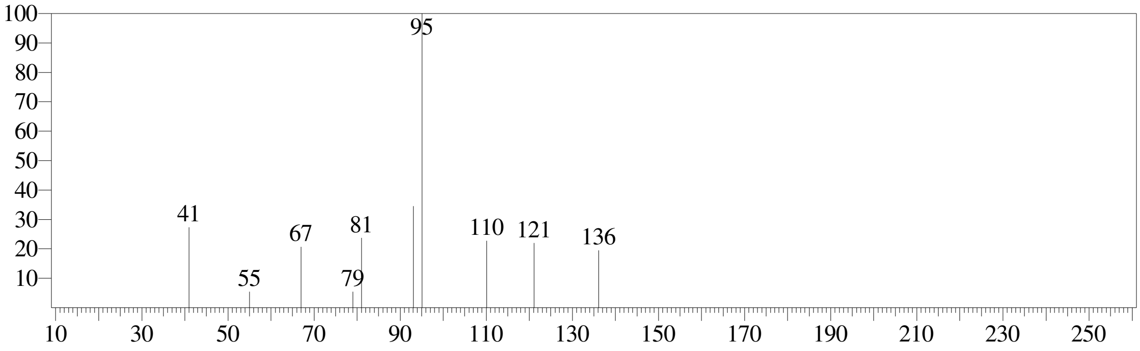

Hit#:3 Entry:18500 Library:NIST23-1.lib
SI:73 Formula:C11H18 CAS:55170-90-6 MolWeight:150 RetIndex:1098
CompName:Bicyclo[2.2.1]heptane, 2-(1-buten-3-yl)- $$ 2-(1-Methyl-2-propenyl)bicyclo[2.2.1]heptane # $$

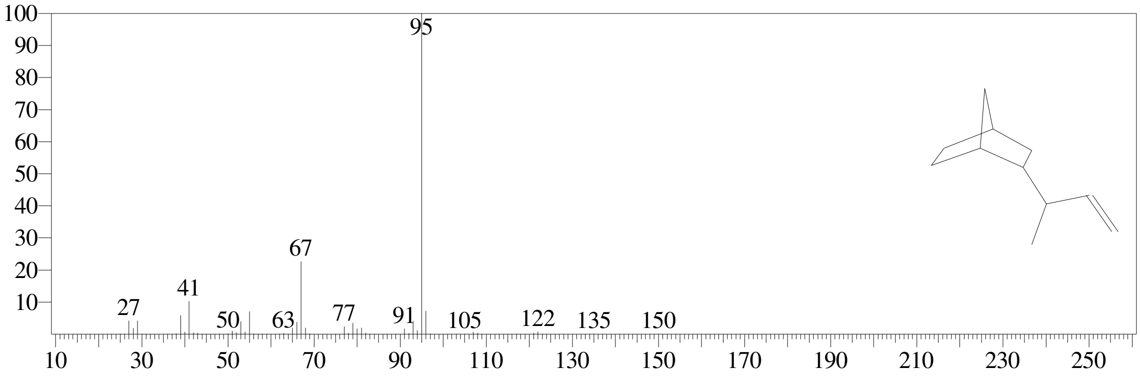

Hit#:4 Entry:13018 Library:NIST23s.lib
SI:73 Formula:C10H18O CAS:464-45-9 MolWeight:154 RetIndex:1159
CompName:Bicyclo[2.2.1]heptan-2-ol, 1,7,7-trimethyl-, (1S-endo)- $$ Borneol, (1S,2R,4S)-(-)- $$ (-)-Borneol $$ L-Born

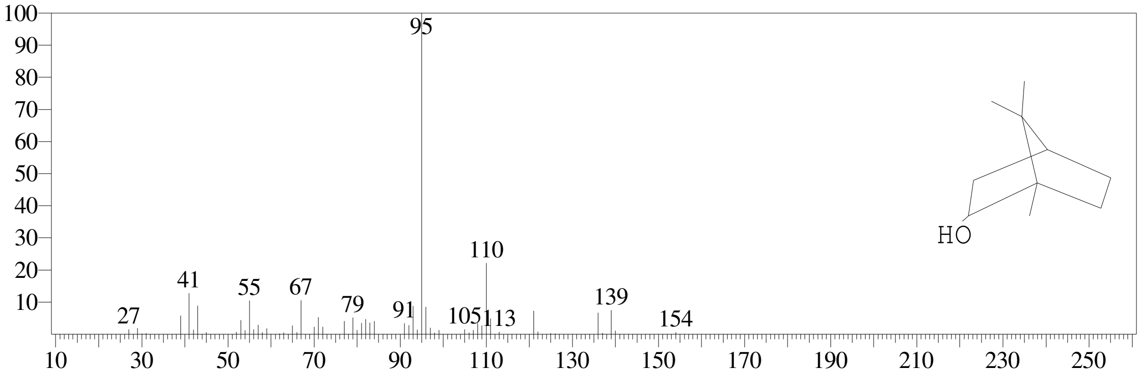

<< Target >>
Line#:19   R.Time:15.100(Scan#:1513) MassPeaks:10
RawMode:Averaged 15.092-15.108(1512-1514) BasePeak:95.10(6207)
BG Mode:None Group 1 - Event 1 Scan

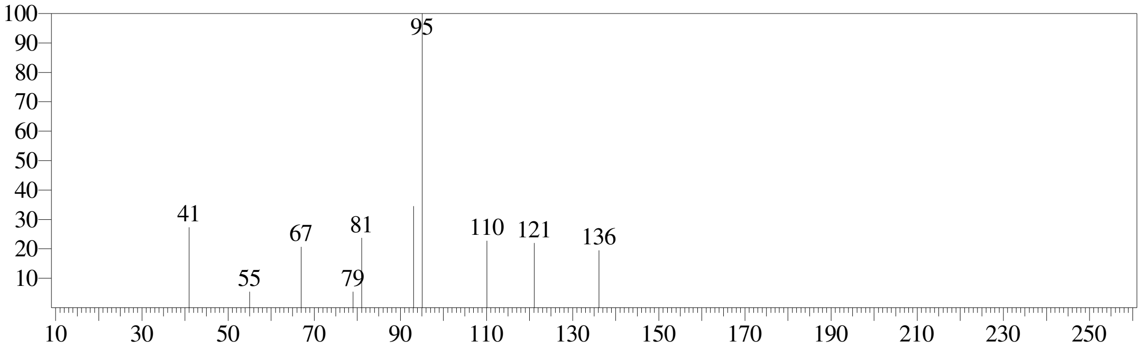

Hit#:5 Entry:32803 Library:NIST23s.lib
SI:73 Formula:C12H17F3O2 CAS:28587-55-5 MolWeight:250 RetIndex:1141
CompName:Borneol, trifluoroacetate (ester) $$ 1,7,7-Trimethylbicyclo[2.2.1]hept-2-yl trifluoroacetate # $$

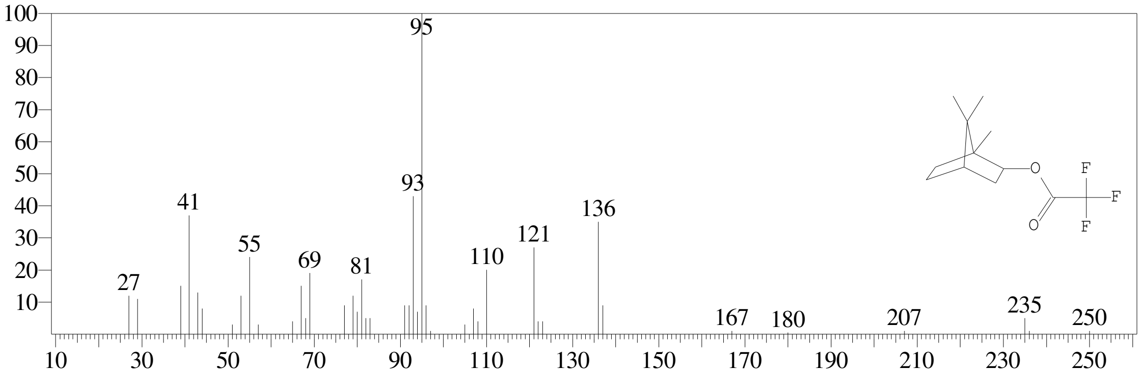

<< Target >>
Line#:20 R.Time:24.317(Scan#:2619) MassPeaks:5
RawMode:Averaged 24.308-24.325(2618-2620) BasePeak:105.10(2305)
BG Mode:None Group 1 - Event 1 Scan

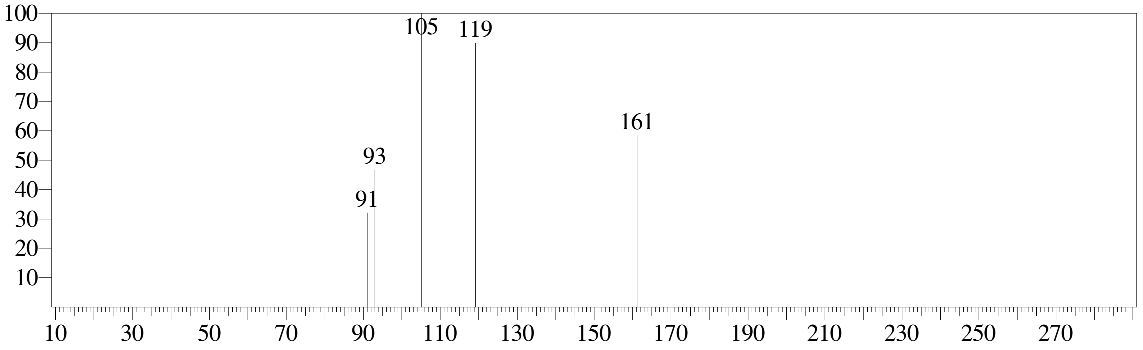

Hit#:1 Entry:5871 Library:NIST23-1.lib
SI:80 Formula:C4H12O2Si CAS:0-00-0 MolWeight:120 RetIndex:635
CompName:Ethoxy(methoxy)methylsilane

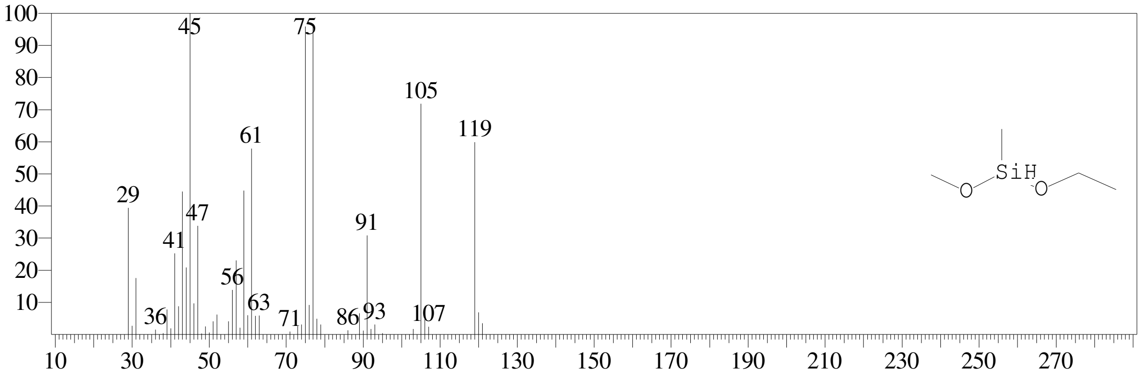

Hit#:2 Entry:24948 Library:NIST23s.lib
SI:76 Formula:C15H24 CAS:17699-14-8 MolWeight:204 RetIndex:1381
CompName:.alpha.-Cubebene $$ 1H-Cyclopenta[1,3]cyclopropa[1,2]benzene, 3a,3b,4,5,6,7-hexahydro-3,7-dimethyl-4-(1-

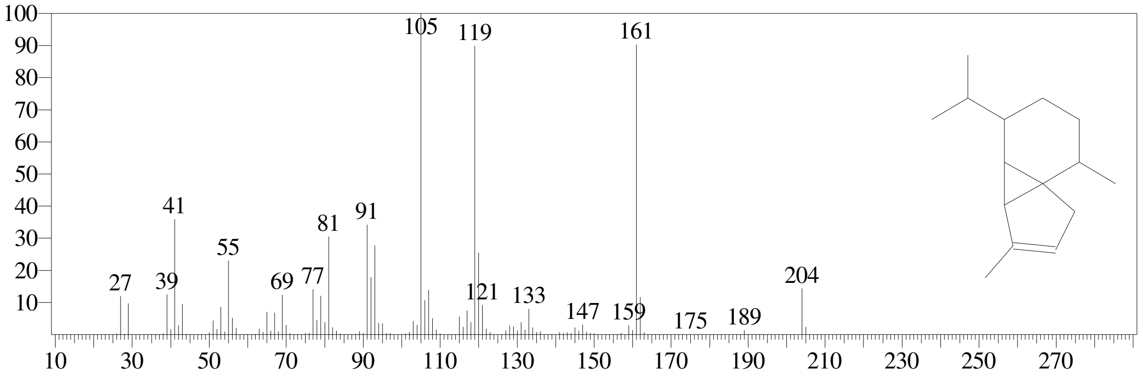

<< Target >>
Line#:20   R.Time:24.317(Scan#:2619) MassPeaks:5
RawMode:Averaged 24.308-24.325(2618-2620) BasePeak:105.10(2305)
BG Mode:None Group 1 - Event 1 Scan

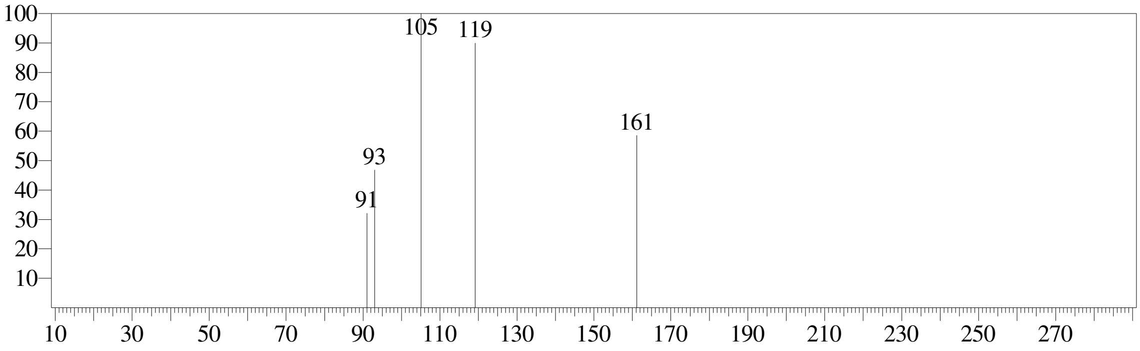

Hit#:3 Entry:14475 Library:NIST23s.lib
SI:76 Formula:C9H7NO2 CAS:5715-02-6 MolWeight:161 RetIndex:1312
CompName:2-Acetoxybenzonitrile $$ Benzonitrile, 2-(acetyloxy)- $$ 2-Cyanophenyl acetate # $$

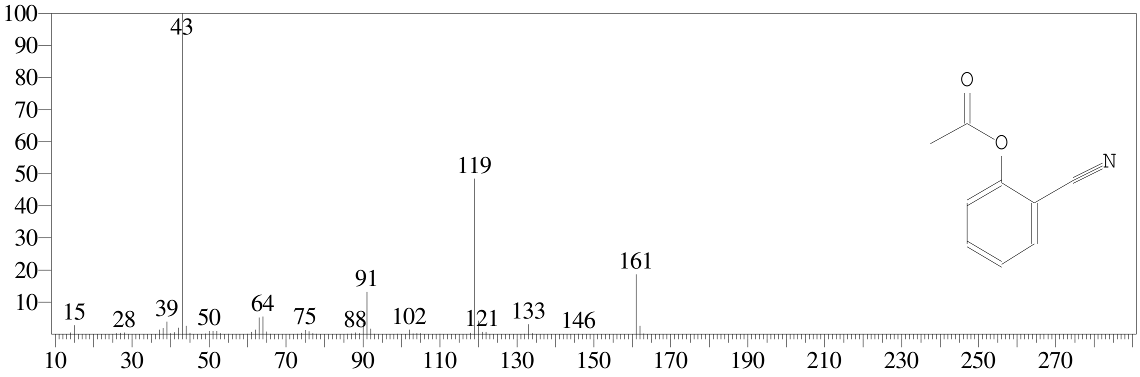

Hit#:4 Entry:25750 Library:NIST23-1.lib
SI:76 Formula:C7H18O2Si CAS:0-00-0 MolWeight:162 RetIndex:927
CompName:Methoxy(n-pentyloxy)methylsilane $$ Methoxy(methyl)(pentyloxy)silane # $$

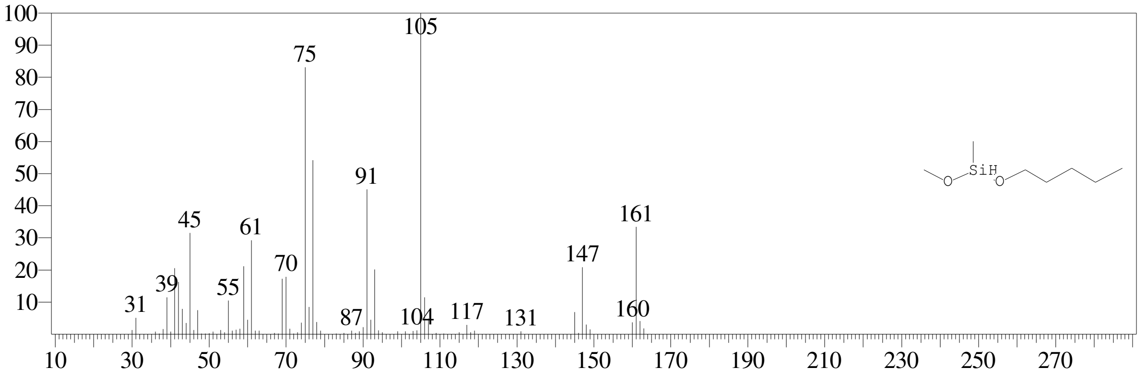

<< Target >>
Line#:20   R.Time:24.317(Scan#:2619) MassPeaks:5
RawMode:Averaged 24.308-24.325(2618-2620) BasePeak:105.10(2305)
BG Mode:None Group 1 - Event 1 Scan

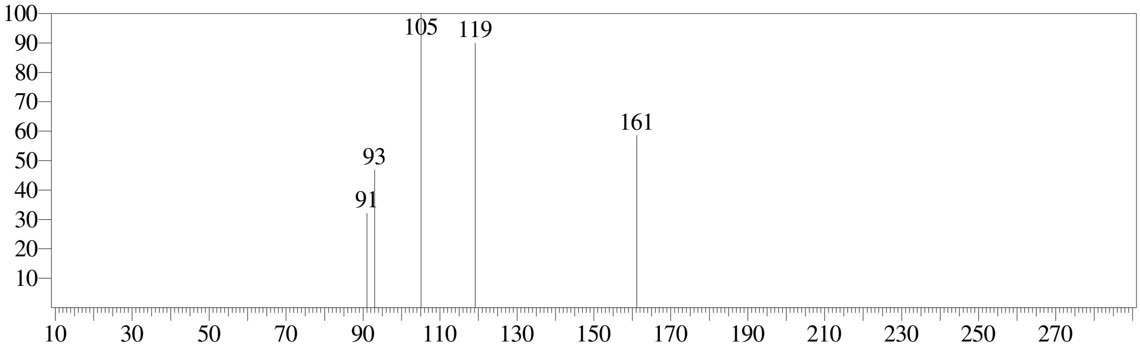

Hit#:5 Entry:160510 Library:NIST23-1.lib
SI:75 Formula:C16H13NO4 CAS:0-00-0 MolWeight:283 RetIndex:2883
CompName:2-Methyl-2-phenyl-5-(1,4-dihydropyridin-4-ylidene)-1,3-dioxan-4,6-dione $$ 2-Methyl-2-phenyl-5-(4(1H)-py

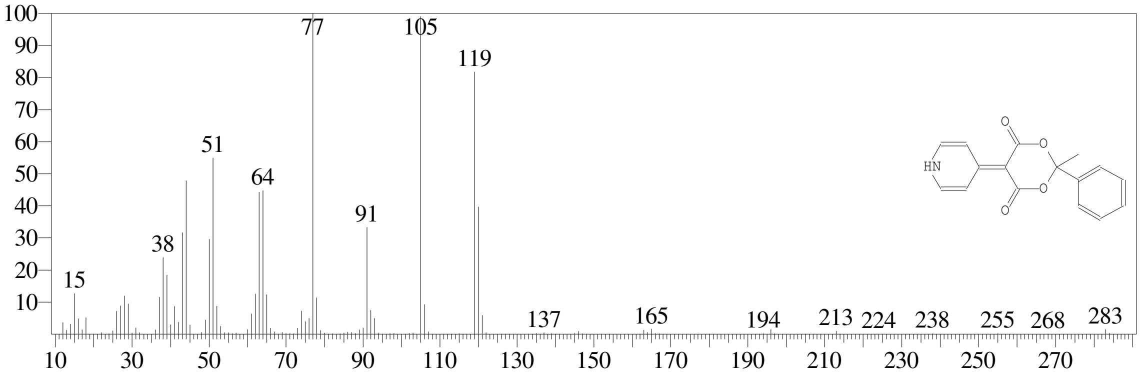

<< Target >>
Line#:21 R.Time:26.142(Scan#:2838) MassPeaks:28
RawMode:Averaged 26.133-26.150(2837-2839) BasePeak:41.00(7392)
BG Mode:None Group 1 - Event 1 Scan

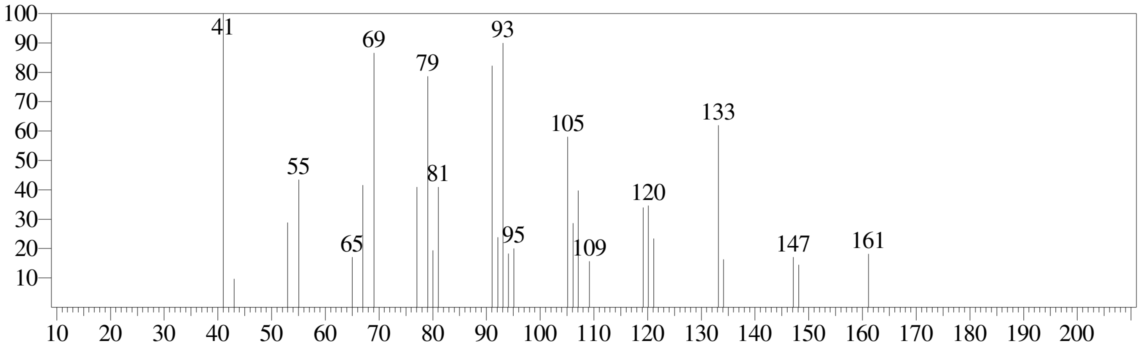

Hit#:1 Entry:24803 Library:NIST23s.lib
SI:91 Formula:C15H24 CAS:118-65-0 MolWeight:204 RetIndex:1448
CompName:Bicyclo[7.2.0]undec-4-ene, 4,11,11-trimethyl-8-methylene-,[1R-(1R*,4Z,9S*)]- $$ Isocaryophyllene $$ 4,11,

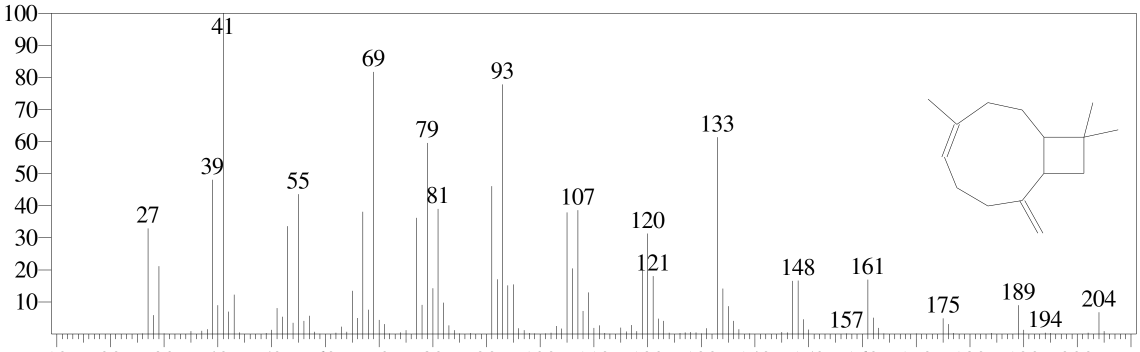

Hit#:2 Entry:24827 Library:NIST23s.lib
SI:91 Formula:C15H24 CAS:87-44-5 MolWeight:204 RetIndex:1448
CompName:Caryophyllene $$ Bicyclo[7.2.0]undec-4-ene, 4,11,11-trimethyl-8-methylene-, [1R-(1R*,4E,9S*)]- $$ Bicycl

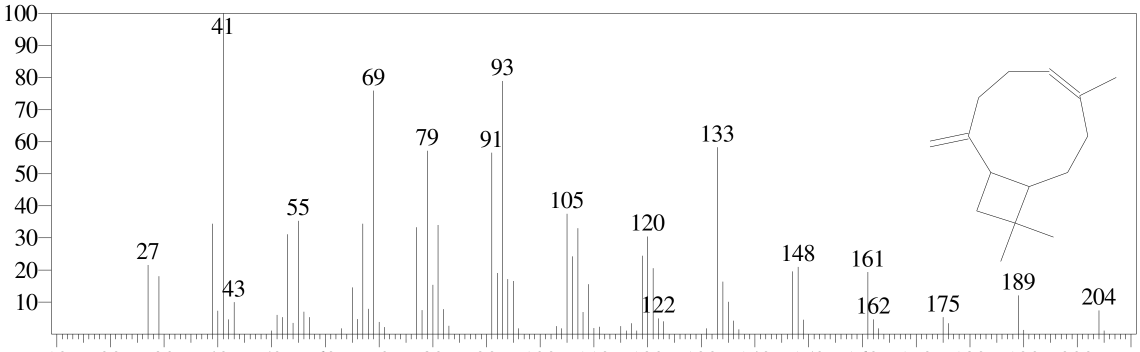

<< Target >>
Line#:21   R.Time:26.142(Scan#:2838) MassPeaks:28
RawMode:Averaged 26.133-26.150(2837-2839) BasePeak:41.00(7392)
BG Mode:None Group 1 - Event 1 Scan

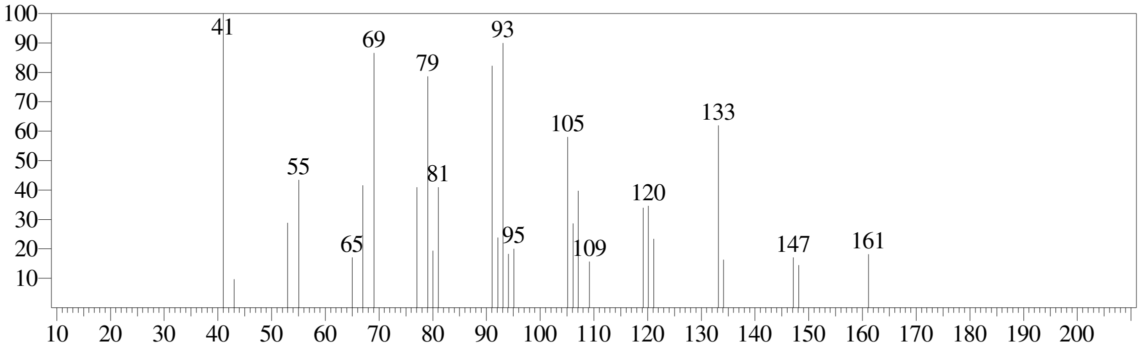

Hit#:3 Entry:24804 Library:NIST23s.lib
SI:90 Formula:C15H24 CAS:87-44-5 MolWeight:204 RetIndex:1448
CompName:Caryophyllene $$ Bicyclo[7.2.0]undec-4-ene, 4,11,11-trimethyl-8-methylene-, [1R-(1R*,4E,9S*)]- $$ Bicycl

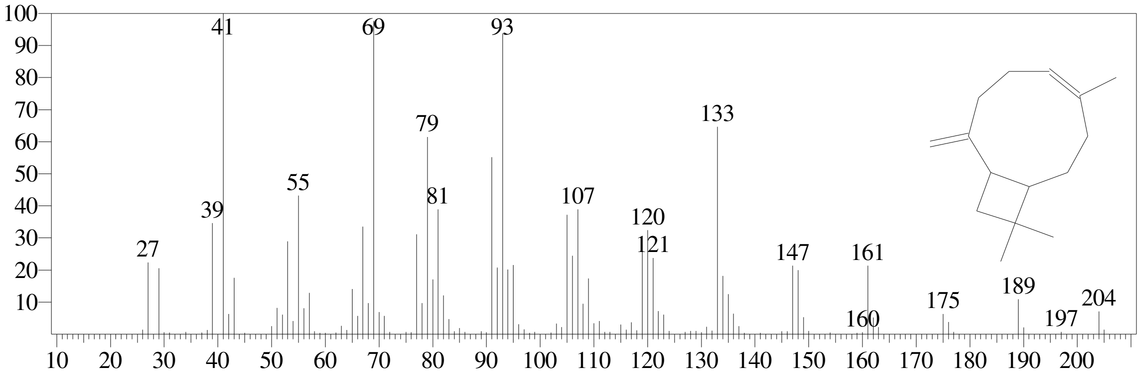

Hit#:4 Entry:62827 Library:NIST23-1.lib
SI:89 Formula:C15H24 CAS:87-44-5 MolWeight:204 RetIndex:1448
CompName:Caryophyllene $$ Bicyclo[7.2.0]undec-4-ene, 4,11,11-trimethyl-8-methylene-, [1R-(1R*,4E,9S*)]- $$ Bicycl

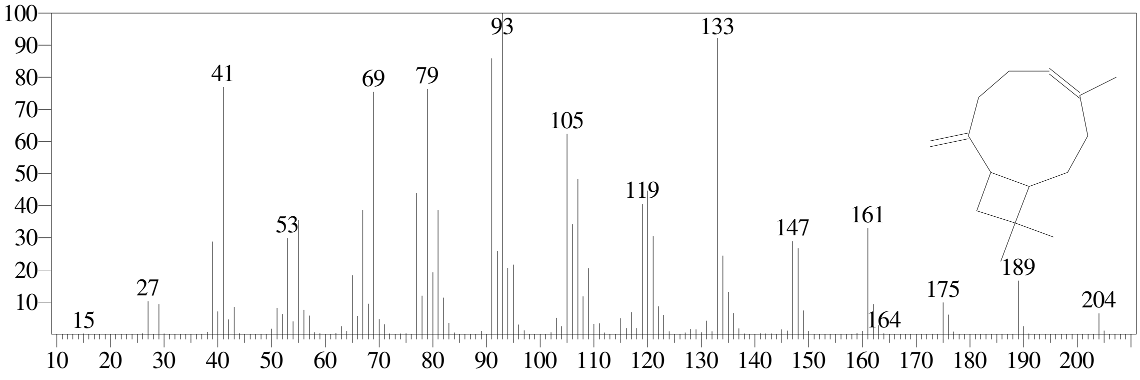

<< Target >>
Line#:21   R.Time:26.142(Scan#:2838) MassPeaks:28
RawMode:Averaged 26.133-26.150(2837-2839) BasePeak:41.00(7392)
BG Mode:None Group 1 - Event 1 Scan

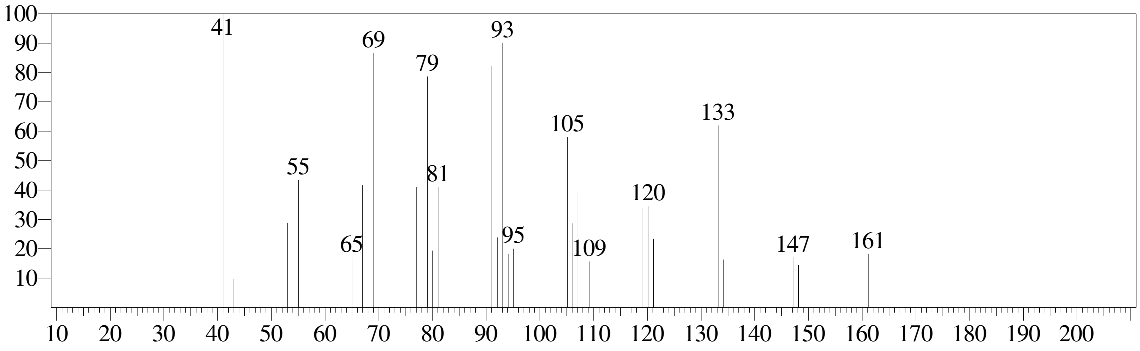

Hit#:5 Entry:24884 Library:NIST23s.lib
SI:89 Formula:C15H24 CAS:13877-93-5 MolWeight:204 RetIndex:1448
CompName:Bicyclo[7.2.0]undec-4-ene, 4,11,11-trimethyl-8-methylene- $$ Bicyclo[7.2.0]undec-4-ene, 4,11,11-trimethyl-

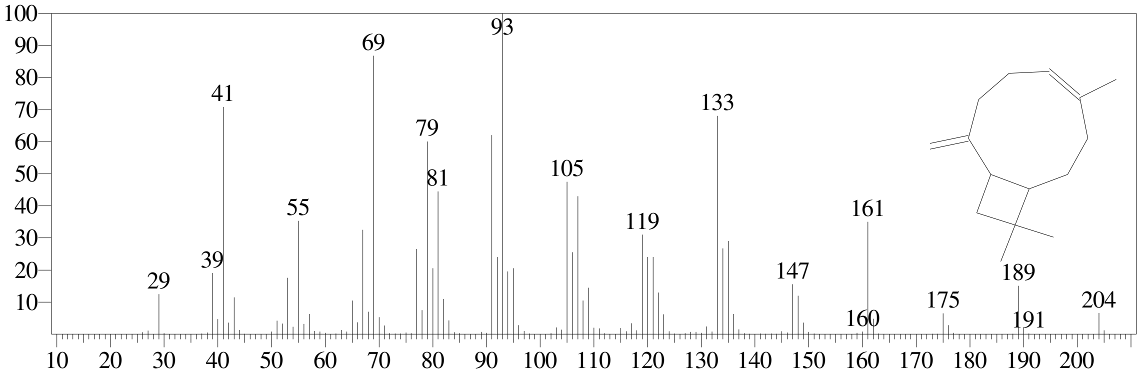

<< Target >>
Line#:22   R.Time:26.942(Scan#:2934) MassPeaks:15
RawMode:Averaged 26.933-26.950(2933-2935) BasePeak:41.00(3063)
BG Mode:None Group 1 - Event 1 Scan

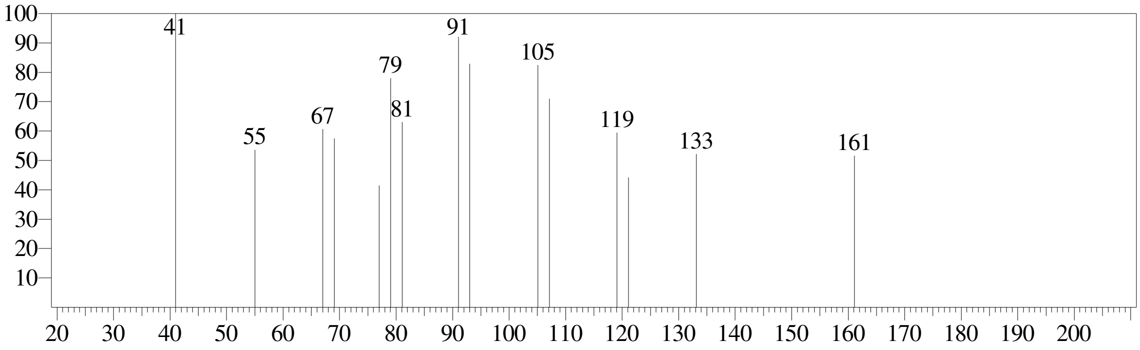

Hit#:1 Entry:24814 Library:NIST23s.lib
SI:77 Formula:C15H24 CAS:25246-27-9 MolWeight:204 RetIndex:1424
CompName:Alloaromadendrene $$ 1H-Cycloprop[e]azulene, decahydro-1,1,7-trimethyl-4-methylene-, [1aR-(1a.alpha.,4a

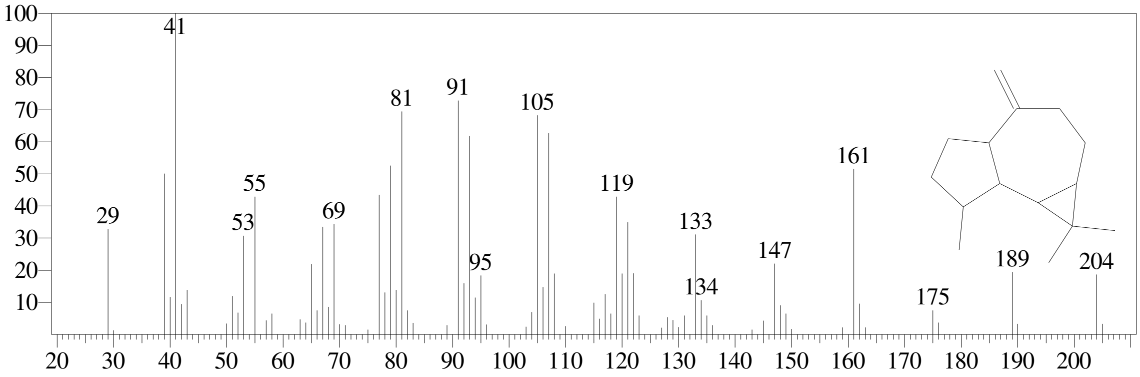

Hit#:2 Entry:62757 Library:NIST23-1.lib
SI:76 Formula:C15H24 CAS:28973-99-1 MolWeight:204 RetIndex:1509
CompName:(Z,Z)-.alpha.-Farnesene $$ (3Z,6Z)-3,7,11-Trimethyl-1,3,6,10-dodecatetraene # $$

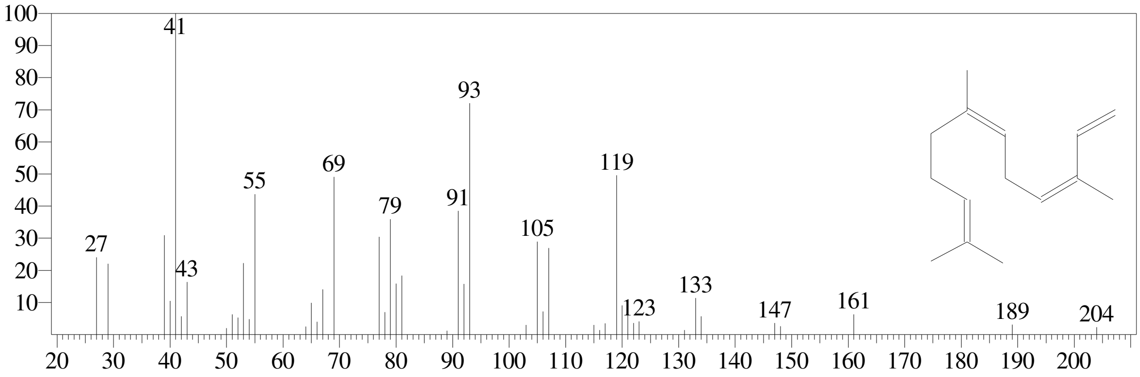

<< Target >>
Line#:22   R.Time:26.942(Scan#:2934) MassPeaks:15
RawMode:Averaged 26.933-26.950(2933-2935) BasePeak:41.00(3063)
BG Mode:None Group 1 - Event 1 Scan

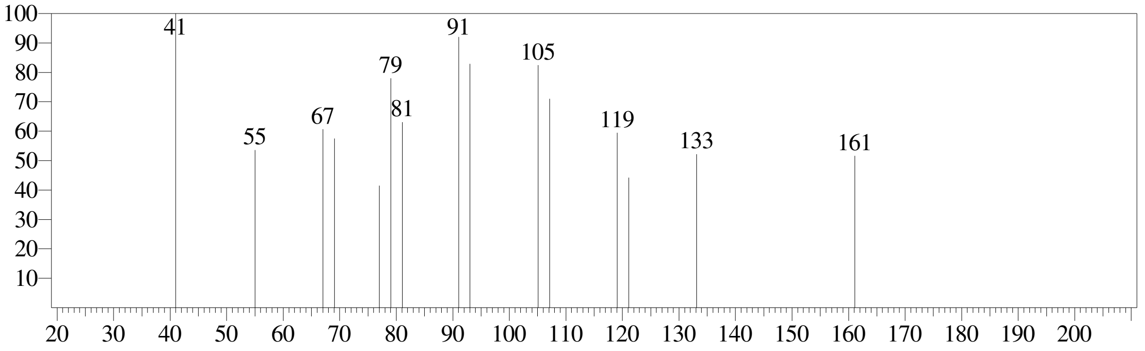

Hit#:3 Entry:62671 Library:NIST23-1.lib
SI:76 Formula:C13H20N2 CAS:0-00-0 MolWeight:204 RetIndex:1704
CompName:1,4-Methanocycloocta[d]pyridazine, 1,4,4a,5,6,9,10,10a-octahydro-11,11-dimethyl-, (1.alpha.,4.alpha.,4a.alpl

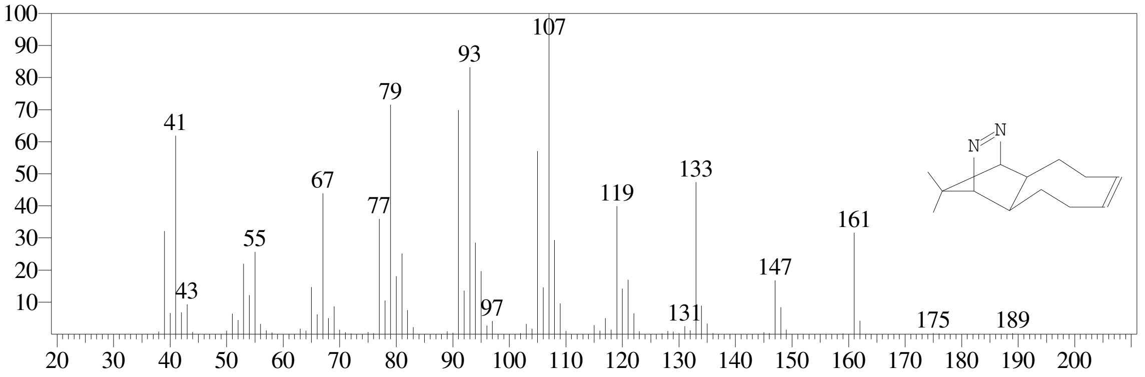

Hit#:4 Entry:24831 Library:NIST23s.lib
SI:76 Formula:C15H24 CAS:26560-14-5 MolWeight:204 RetIndex:1509
CompName:1,3,6,10-Dodecatetraene, 3,7,11-trimethyl-, (Z,E)- $$ (Z,E)-.alpha.-Farnesene $$ (3Z,6E)-3,7,11-Trimethyl-1.

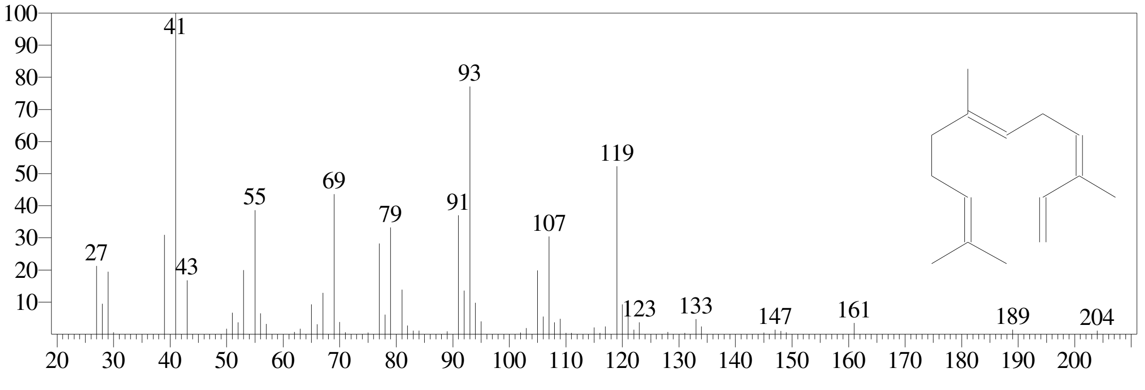

<< Target >>
Line#:22   R.Time:26.942(Scan#:2934) MassPeaks:15
RawMode:Averaged 26.933-26.950(2933-2935) BasePeak:41.00(3063)
BG Mode:None Group 1 - Event 1 Scan

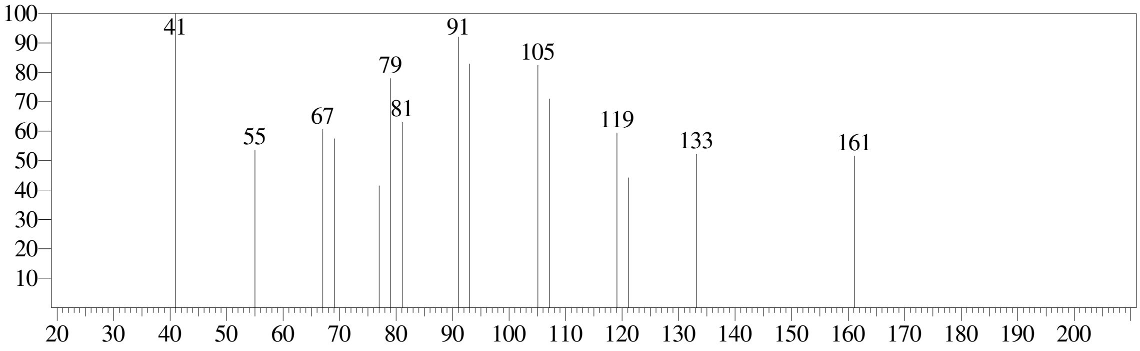

Hit#:5 Entry:62756 Library:NIST23-1.lib
SI:76 Formula:C15H24 CAS:26560-14-5 MolWeight:204 RetIndex:1509
CompName:1,3,6,10-Dodecatetraene, 3,7,11-trimethyl-, (Z,E)- $$ (Z,E)-.alpha.-Farnesene $$ (3Z,6E)-3,7,11-Trimethyl-1

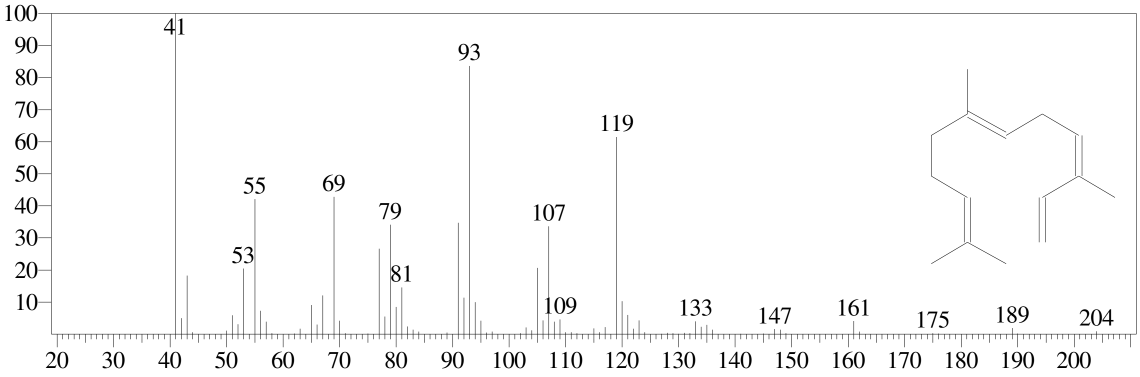

<< Target >>
Line#:23 R.Time:27.542(Scan#:3006) MassPeaks:2
RawMode:Averaged 27.533-27.550(3005-3007) BasePeak:93.05(3310)
BG Mode:None Group 1 - Event 1 Scan

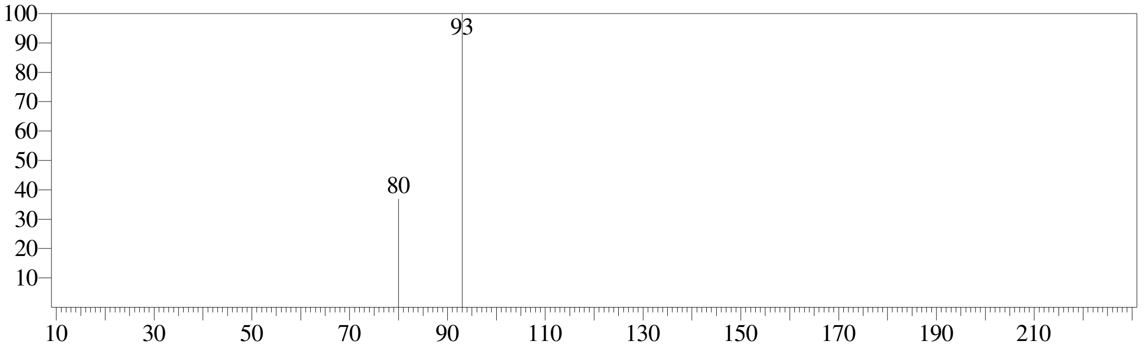

Hit#:1 Entry:1325 Library:NIST23-1.lib
SI:90 Formula:C5H6N2 CAS:7321-55-3 MolWeight:94 RetIndex:835
CompName:Propanedinitrile, dimethyl- $$ Malononitrile, dimethyl- $$ Dimethylmalononitrile $$ 2,2-Dicyanopropane $$

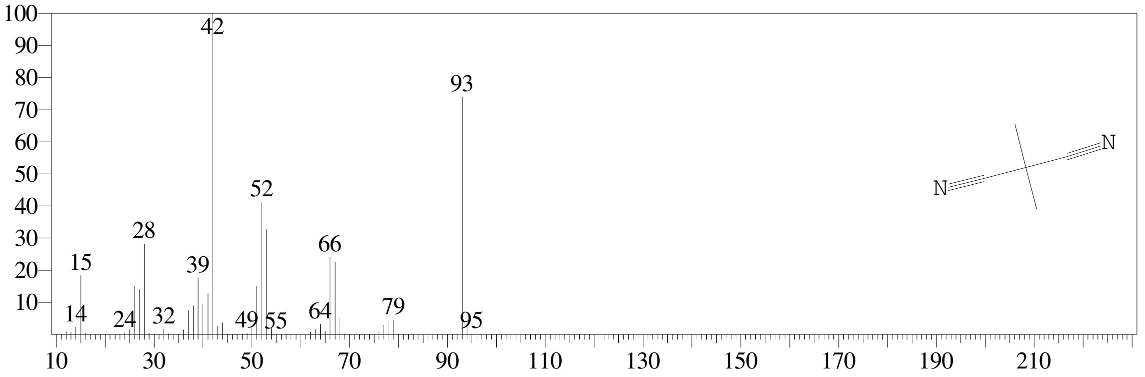

Hit#:2 Entry:3193 Library:NIST23s.lib
SI:90 Formula:C8H12 CAS:54211-14-2 MolWeight:108 RetIndex:846
CompName:Bicyclo[4.1.0]heptane, 7-methylene- $$ 7-Methylenenorcarane $$ 7-Methylenebicyclo[4.1.0]heptane $$

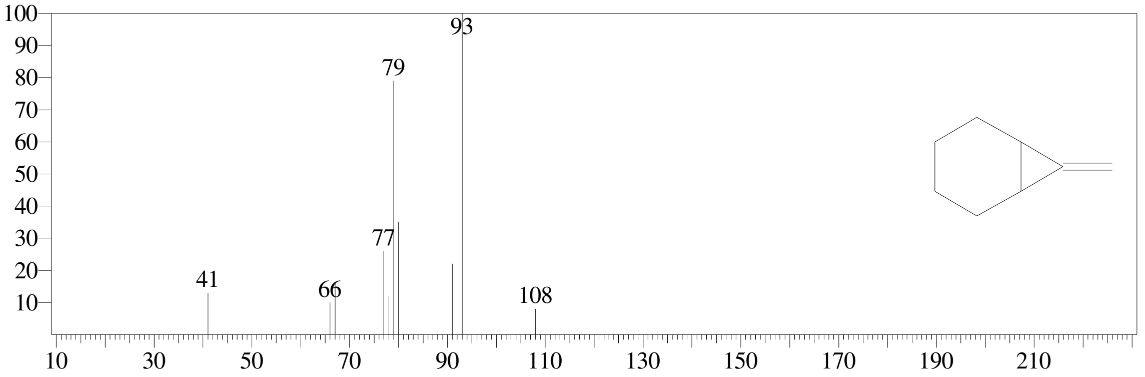

<< Target >>
Line#:23   R.Time:27.542(Scan#:3006) MassPeaks:2
RawMode:Averaged 27.533-27.550(3005-3007) BasePeak:93.05(3310)
BG Mode:None Group 1 - Event 1 Scan

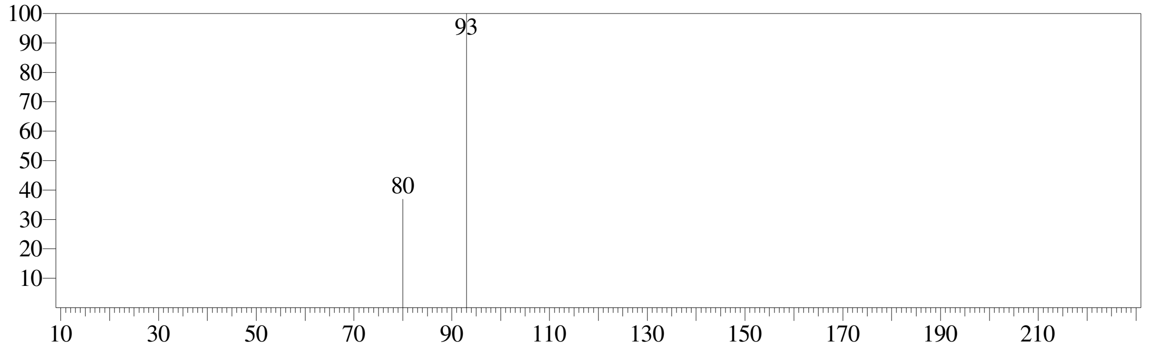

Hit#:3 Entry:3129 Library:NIST23-1.lib
SI:88 Formula:C8H12 CAS:116279-08-4 MolWeight:108 RetIndex:838
CompName:3-Cyclopentyl-1-propyne $$ Cyclopentylpropyne $$ 2-Propynylcyclopentane # $$

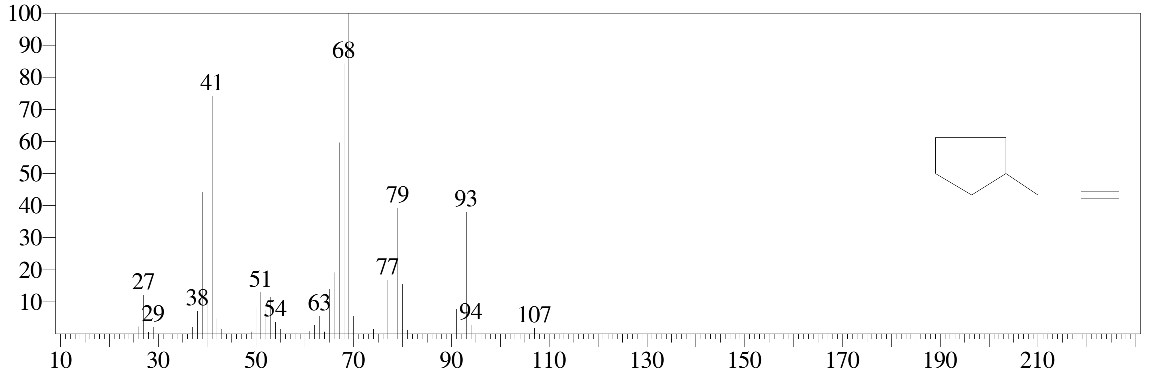

Hit#:4 Entry:80058 Library:NIST23-1.lib
SI:87 Formula:C11H12N2O3 CAS:0-00-0 MolWeight:220 RetIndex:2120
CompName:3-Pyrrolidinecarboxylic acid, 5-oxo-1-(2-pyridinylmethyl)-

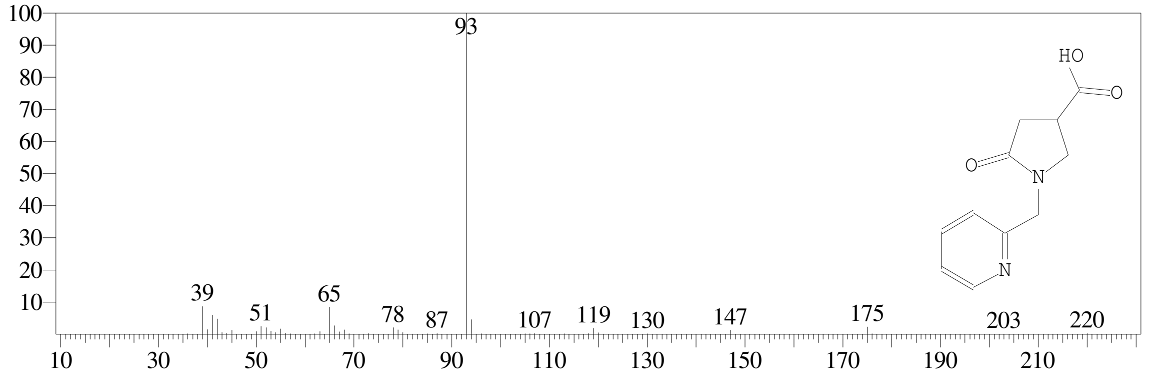

<< Target >>
Line#:23   R.Time:27.542(Scan#:3006) MassPeaks:2
RawMode:Averaged 27.533-27.550(3005-3007) BasePeak:93.05(3310)
BG Mode:None Group 1 - Event 1 Scan

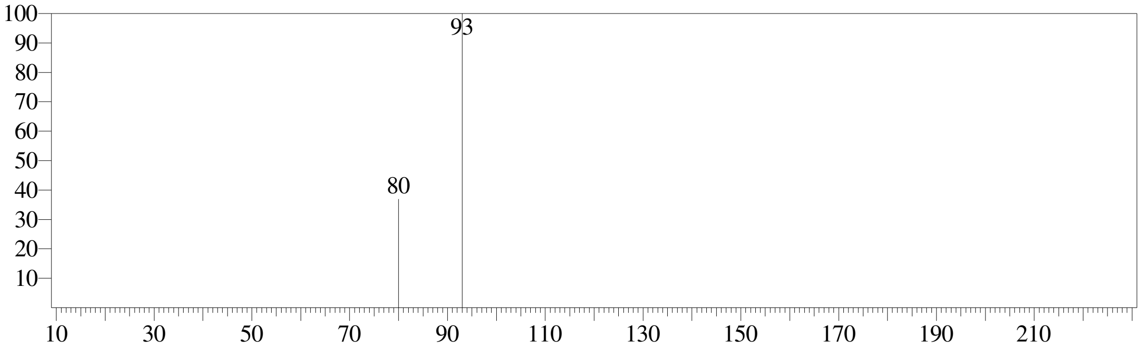

Hit#:5 Entry:11126 Library:NIST23-1.lib
SI:86 Formula:C8H12N2 CAS:55496-55-4 MolWeight:136 RetIndex:1284
CompName:4-(2-Methylamino)ethyl)pyridine $$ Pyridine, 4-(2-methylaminoethyl)- $$ N-Methyl-2-(4-pyridinyl)ethanam

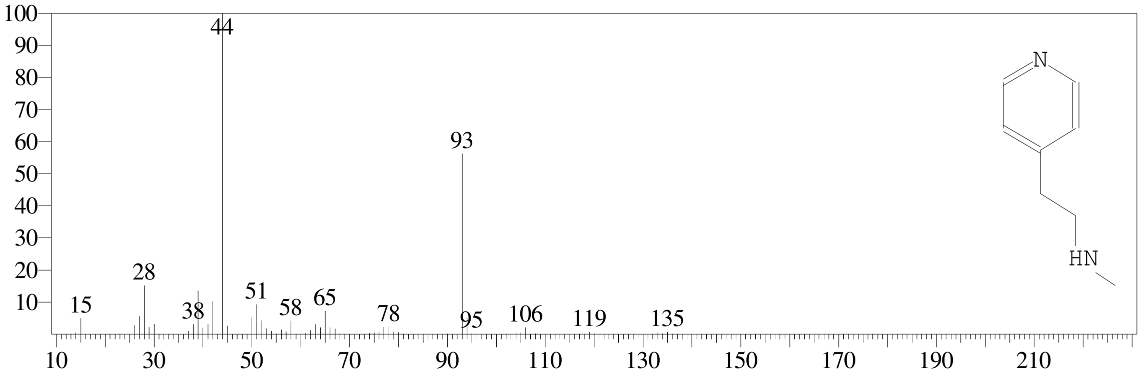

<< Target >>
Line#:24 R.Time:29.250(Scan#:3211) MassPeaks:18
RawMode:Averaged 29.242-29.258(3210-3212) BasePeak:107.10(4839)
BG Mode:None Group 1 - Event 1 Scan

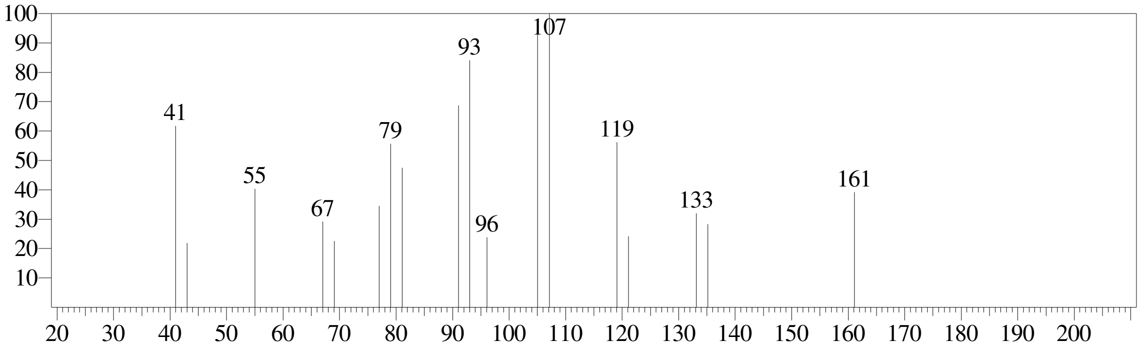

Hit#:1 Entry:37014 Library:NIST23-1.lib
SI:79 Formula:C13H20 CAS:0-00-0 MolWeight:176 RetIndex:1193
CompName:(+)-3-Carene, 2-.alpha.-isopropenyl- $$ 2-Isopropenyl-3,7,7-trimethylbicyclo[4.1.0]hept-3-ene # $$

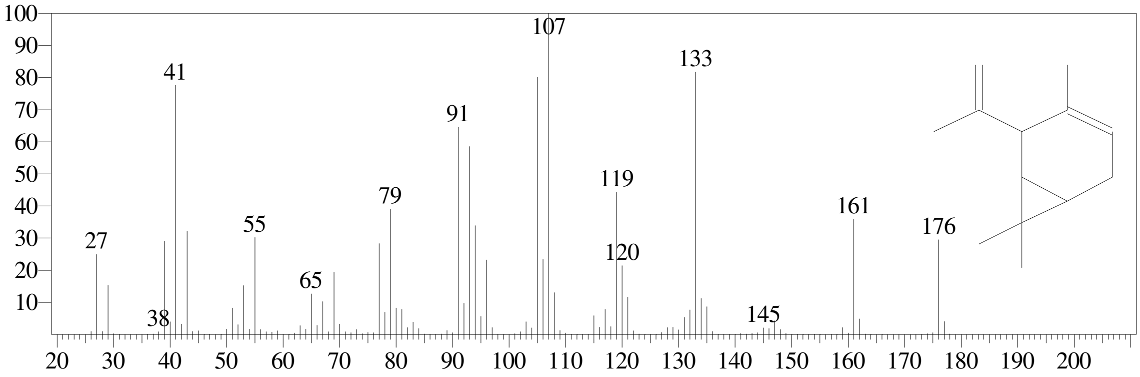

Hit#:2 Entry:24960 Library:NIST23s.lib
SI:78 Formula:C15H24 CAS:13062-00-5 MolWeight:204 RetIndex:1538
CompName:(Z)-1-Methyl-4-(6-methylhept-5-en-2-ylidene)cyclohex-1-ene $$ Cyclohexene, 4-(1,5-dimethyl-4-hexen-1-yl

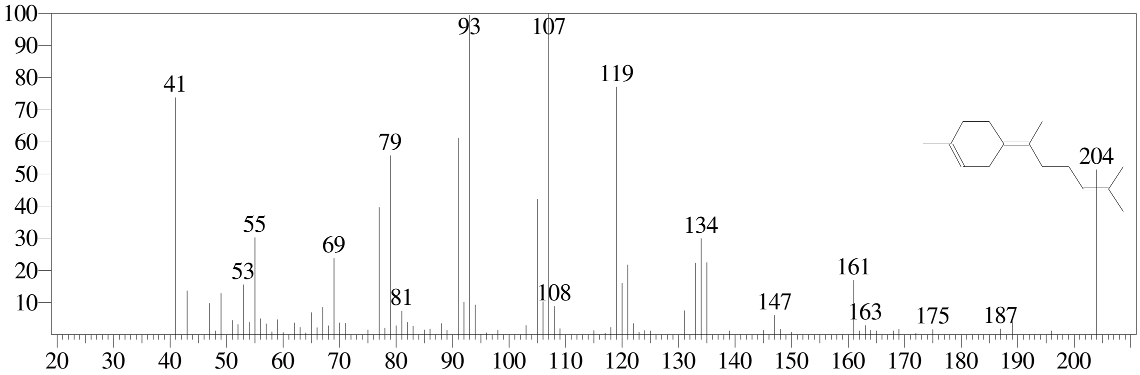

<< Target >>
Line#:24   R.Time:29.250(Scan#:3211) MassPeaks:18
RawMode:Averaged 29.242-29.258(3210-3212) BasePeak:107.10(4839)
BG Mode:None Group 1 - Event 1 Scan

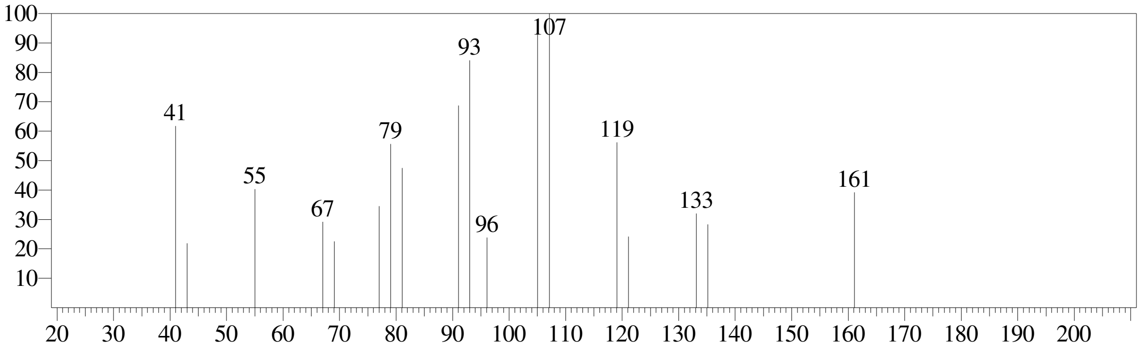

Hit#:3 Entry:62671 Library:NIST23-1.lib
SI:78 Formula:C13H20N2 CAS:0-00-0 MolWeight:204 RetIndex:1704
CompName:1,4-Methanocycloocta[d]pyridazine, 1,4,4a,5,6,9,10,10a-octahydro-11,11-dimethyl-, (1.alpha.,4.alpha.,4a.alpl

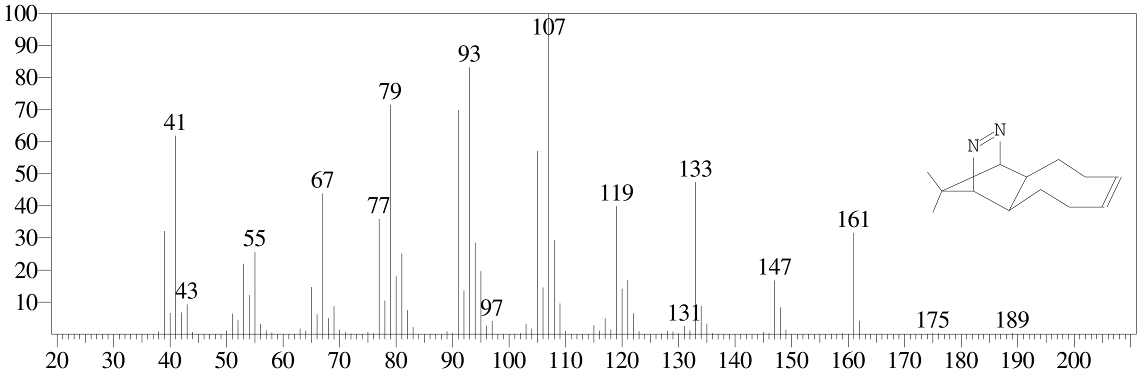

Hit#:4 Entry:24893 Library:NIST23s.lib
SI:77 Formula:C15H24 CAS:53585-13-0 MolWeight:204 RetIndex:1541
CompName:(E)-1-Methyl-4-(6-methylhept-5-en-2-ylidene)cyclohex-1-ene $$ Cyclohexene, 4-(1,5-dimethyl-4-hexen-1-yl

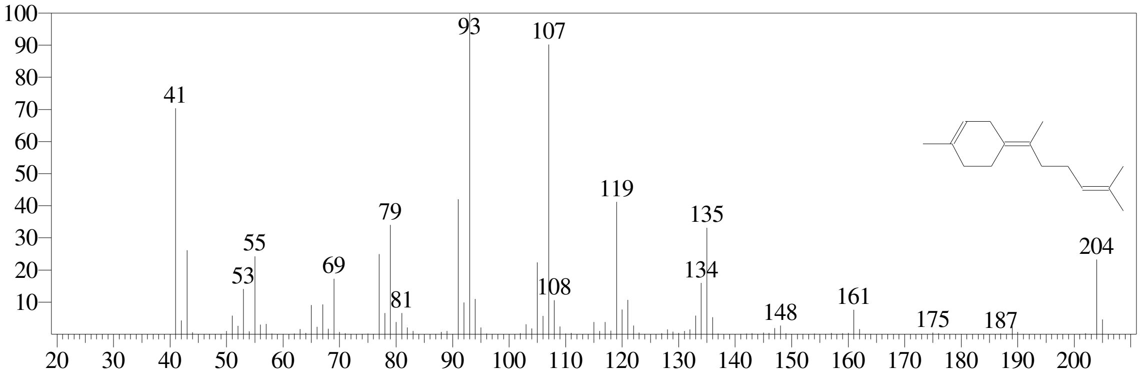

<< Target >>
Line#:24 R.Time:29.250(Scan#:3211) MassPeaks:18
RawMode:Averaged 29.242-29.258(3210-3212) BasePeak:107.10(4839)
BG Mode:None Group 1 - Event 1 Scan

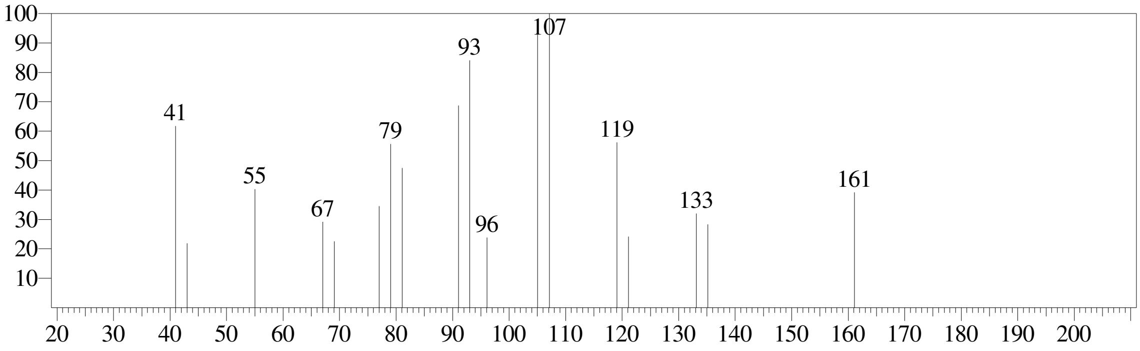

Hit#:5 Entry:24964 Library:NIST23s.lib
SI:76 Formula:C15H24 CAS:21747-46-6 MolWeight:204 RetIndex:1451
CompName:1H-Cycloprop[e]azulene, 1a,2,3,5,6,7,7a,7b-octahydro-1,1,4,7-tetramethyl-, [1aR-(1a.alpha.,7.alpha.,7a.beta.,

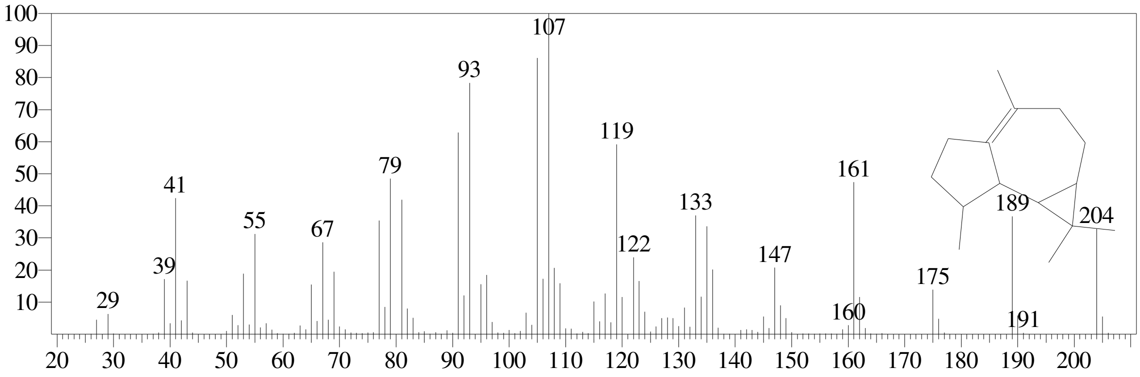

<< Target >>
Line#:25 R.Time:30.375(Scan#:3346) MassPeaks:8
RawMode:Averaged 30.367-30.383(3345-3347) BasePeak:119.10(2766)
BG Mode:None Group 1 - Event 1 Scan

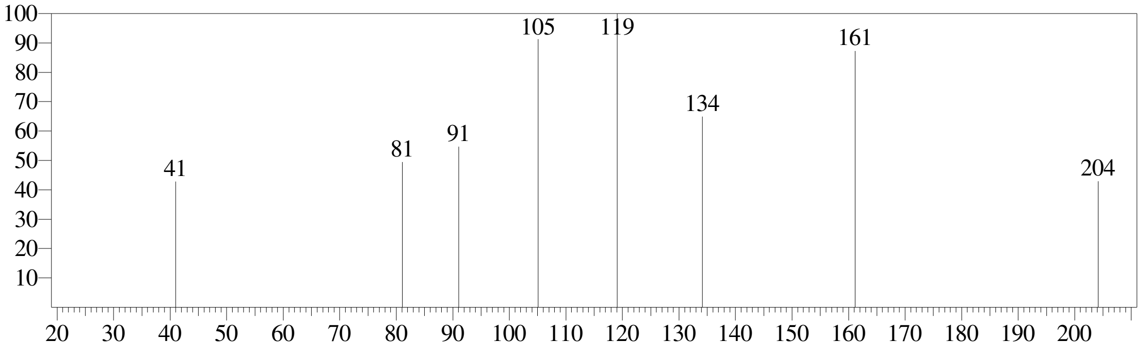

Hit#:1 Entry:25084 Library:NIST23s.lib
SI:71 Formula:C15H24 CAS:157374-44-2 MolWeight:204 RetIndex:1449
CompName:cis-muurola-3,5-diene

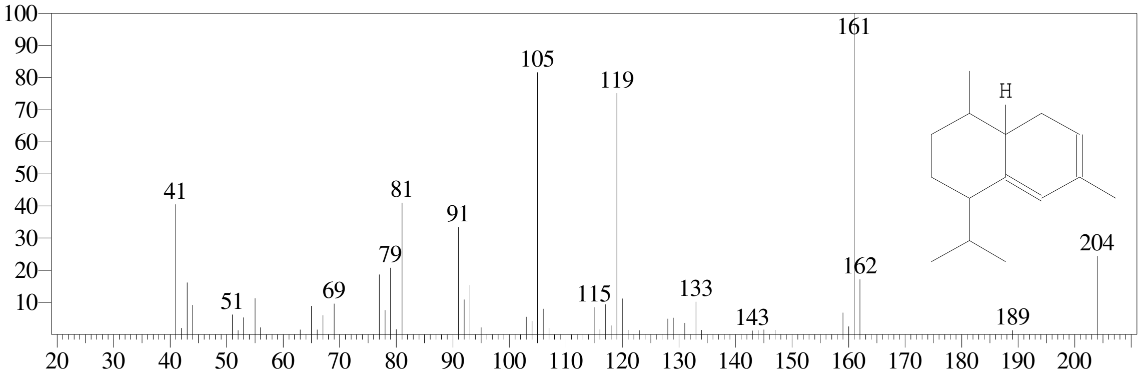

Hit#:2 Entry:63011 Library:NIST23-1.lib
SI:70 Formula:C15H24 CAS:483-76-1 MolWeight:204 RetIndex:1526
CompName:Naphthalene, 1,2,3,5,6,8a-hexahydro-4,7-dimethyl-1-(1-methylethyl)-, (1S-cis)- $$ Cadina-1(10),4-diene $$.

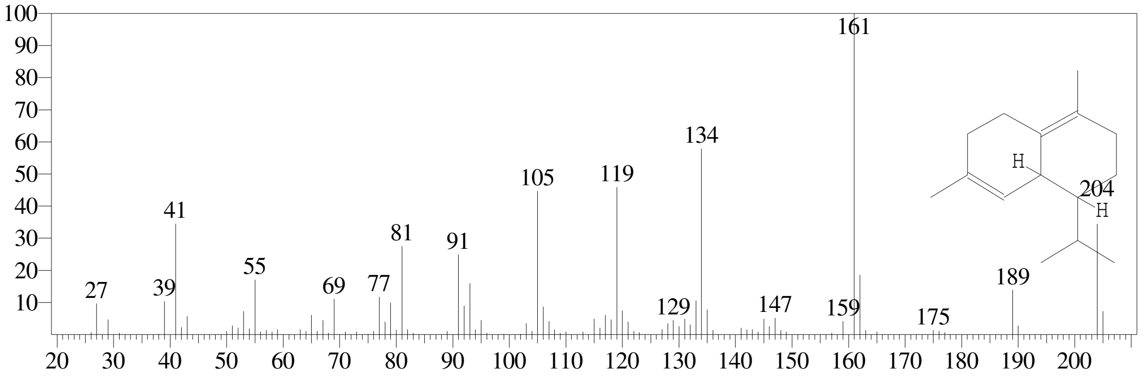

<< Target >>
Line#:25   R.Time:30.375(Scan#:3346) MassPeaks:8
RawMode:Averaged 30.367-30.383(3345-3347) BasePeak:119.10(2766)
BG Mode:None Group 1 - Event 1 Scan

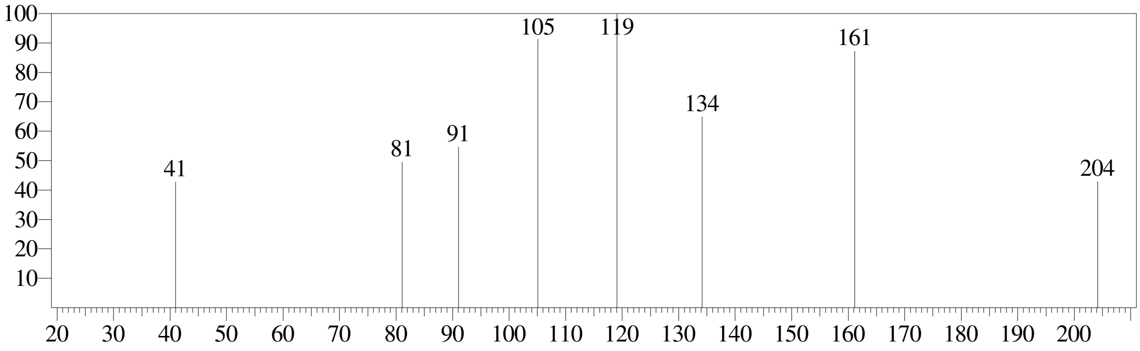

Hit#:3 Entry:62987 Library:NIST23-1.lib
SI:70 Formula:C15H24 CAS:267665-20-3 MolWeight:204 RetIndex:1465
CompName:(1S,4S,4aS)-1-Isopropyl-4,7-dimethyl-1,2,3,4,4a,5-hexahydronaphthalene $$ Naphthalene, 1,2,3,4,4a,5-hexal

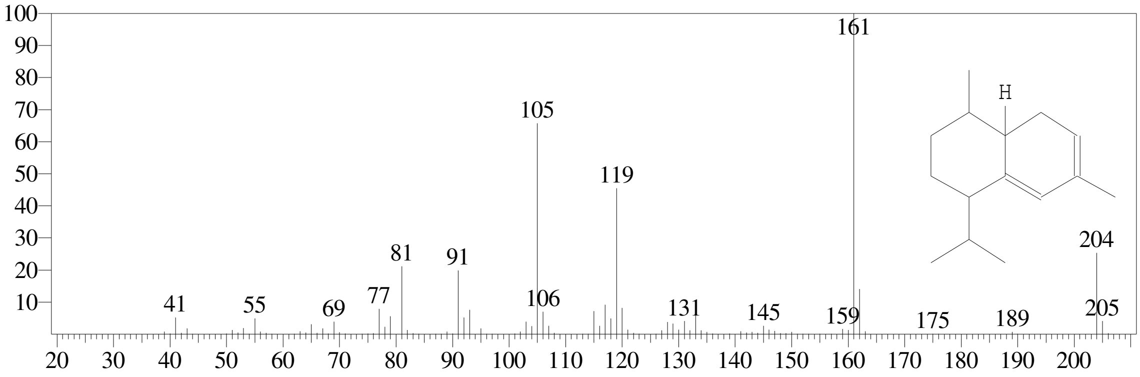

Hit#:4 Entry:62988 Library:NIST23-1.lib
SI:69 Formula:C15H24 CAS:17699-14-8 MolWeight:204 RetIndex:1381
CompName:.alpha.-Cubebene $$ 1H-Cyclopenta[1,3]cyclopropa[1,2]benzene, 3a,3b,4,5,6,7-hexahydro-3,7-dimethyl-4-(1-

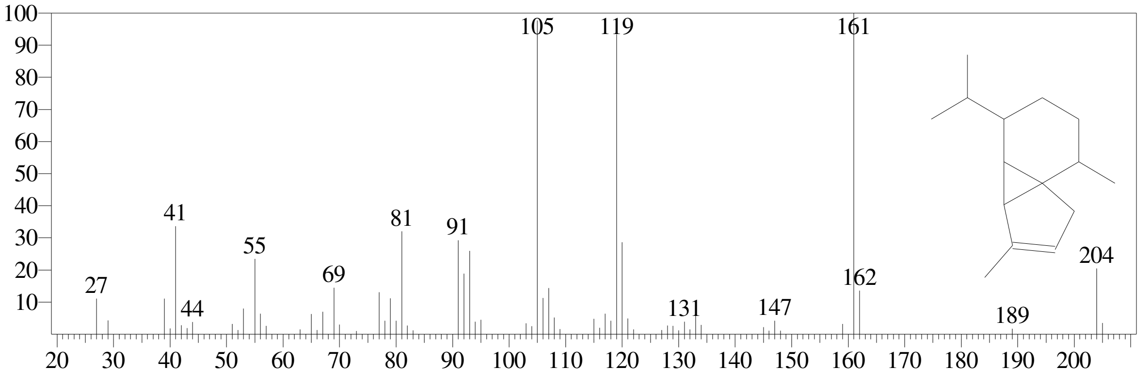

<< Target >>
Line#:25   R.Time:30.375(Scan#:3346) MassPeaks:8
RawMode:Averaged 30.367-30.383(3345-3347) BasePeak:119.10(2766)
BG Mode:None Group 1 - Event 1 Scan

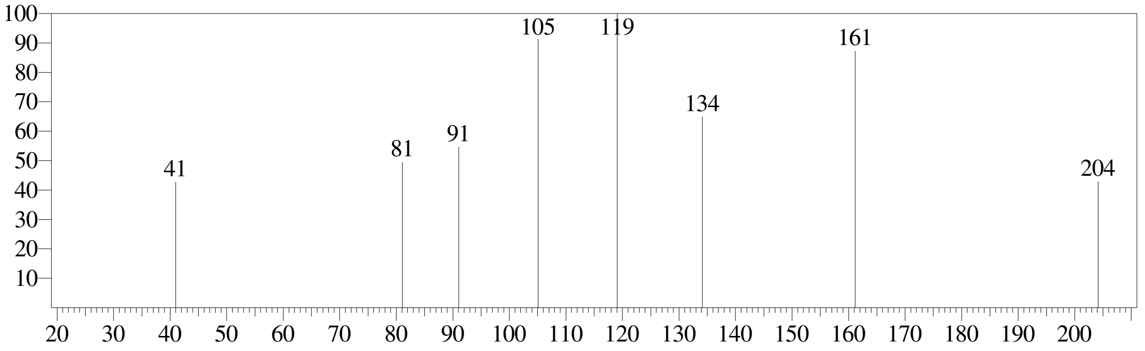

Hit#:5 Entry:24949 Library:NIST23s.lib
SI:69 Formula:C15H24 CAS:17699-14-8 MolWeight:204 RetIndex:1381
CompName:.alpha.-Cubebene $$ 1H-Cyclopenta[1,3]cyclopropa[1,2]benzene, 3a,3b,4,5,6,7-hexahydro-3,7-dimethyl-4-(1-

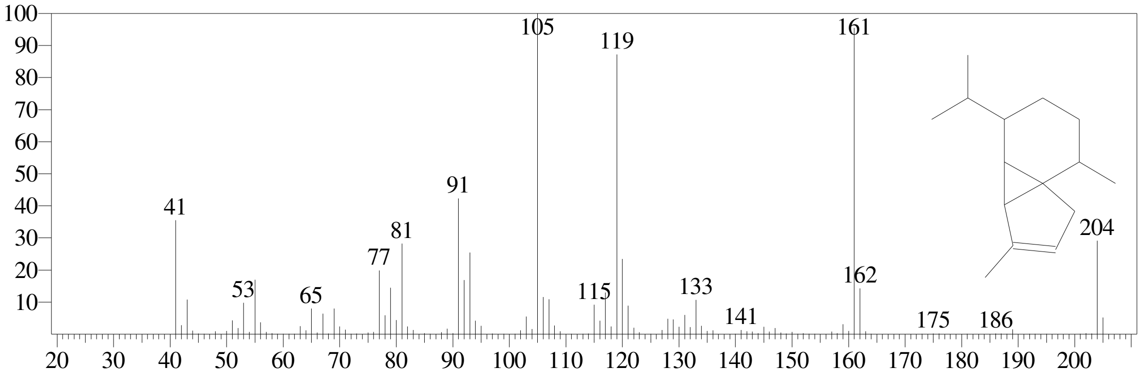

Supplement: Supplementary file 1 [file plants-15-01406-s001.zip › EP bb.pdf]
