# Supplementary material for: Unveiling Chemical Profile and Insecticidal Potential of Essential Oils from Leaves of Seven Eugenia L. Species (Myrtaceae)
Source: Plants (Basel). 2026 May 5;15(9):1406. doi: 10.3390/plants15091406 (PMC13165059; doi:10.3390/plants15091406)

CGMS

Analyzed by: Cristiane Cardoso
Analyzed: 17/6/2025
Solicitante:Douglas
Sample Name: EU
Injection Volume: 1,0 uL   Solvente: Diclorometano
Data File: C:\GCMSsolution\Data\Project1\Douglas\2025\MLLENA\170625\EU.qgd
Method File: C:\GCMSsolution\Data\Project1\Douglas\Essencial Adams-Inj.qgm
EQUIPAMENTO: Modelo:GCMS-QP2010 Plus (Shimadzu)
Coluna: VF-5m (30X0.25X0.25)

Chromatogram EU C:\GCMSsolution\Data\Project1\Douglas\2025\MLLENA\170625\EU.qgd

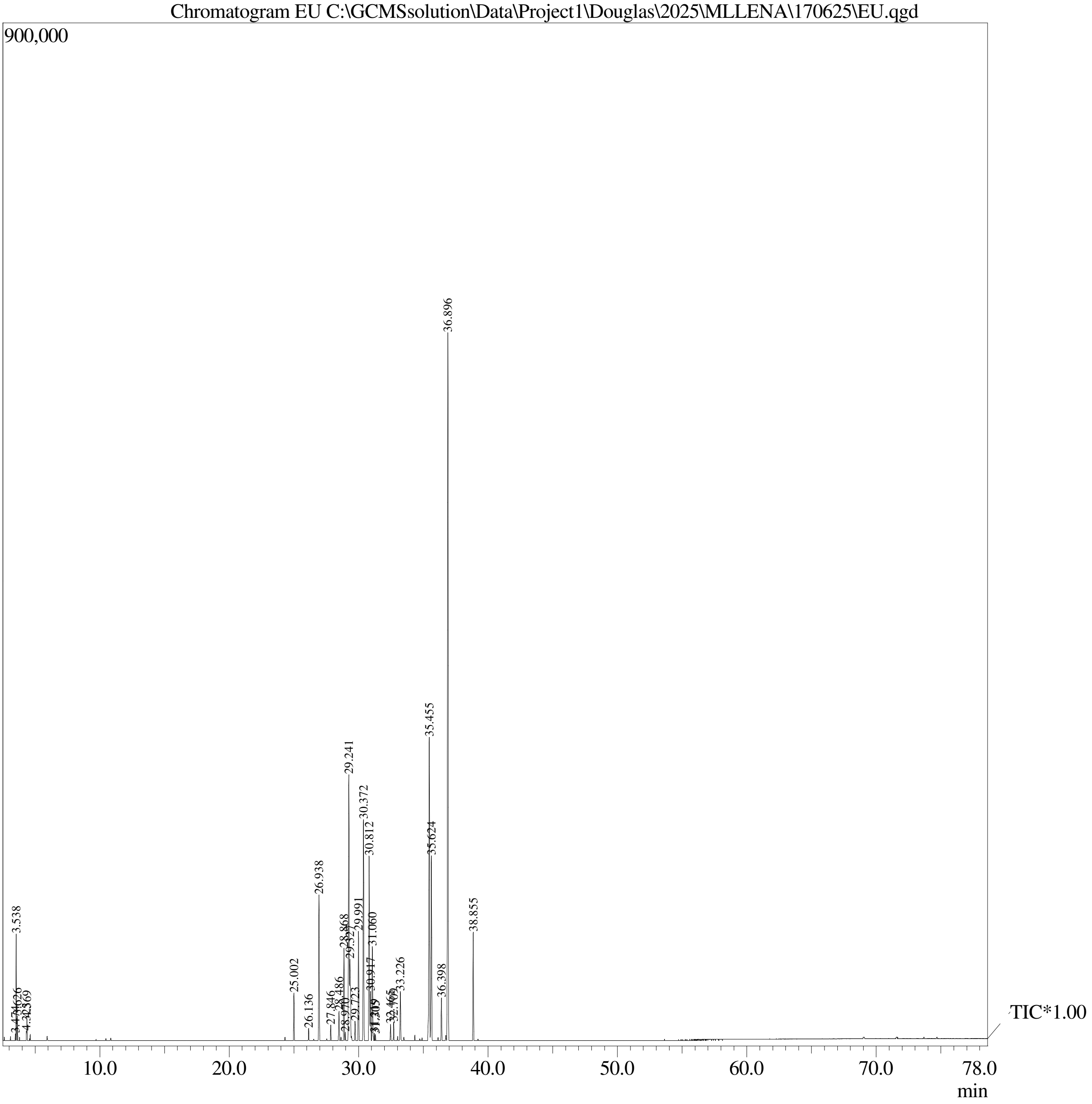

Library

<< Target >>
Line#:1   R.Time:3.475(Scan#:118) MassPeaks:2
RawMode:Averaged 3.467-3.483(117-119) BasePeak:59.00(3372)
BG Mode:None Group 1 - Event 1 Scan

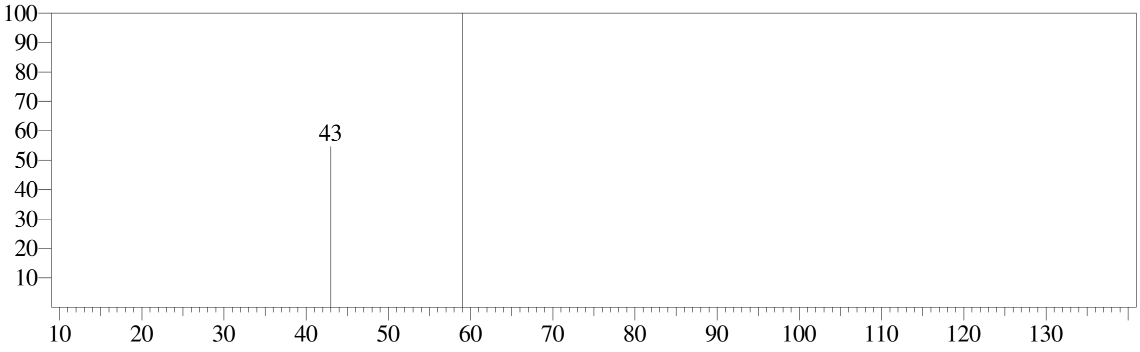

Hit#:1 Entry:2768 Library:NIST23s.lib
SI:93 Formula:C4H8O3 CAS:594-61-6 MolWeight:104 RetIndex:932
CompName:Propanoic acid, 2-hydroxy-2-methyl- $$ 2-Hydroxyisobutyric acid $$ Lactic acid, 2-methyl- $$ .alpha.-Hydrc

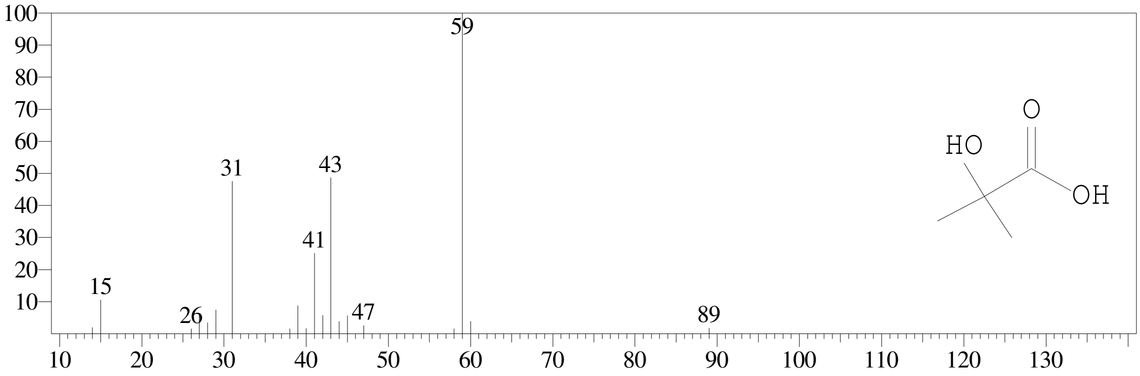

Hit#:2 Entry:152 Library:NIST23s.lib
SI:93 Formula:CH5N3 CAS:113-00-8 MolWeight:59 RetIndex:1188
CompName:Guanidine $$ Aminoformamidine $$ Aminomethanamidine $$ Carbamamidine $$ Carbamidine $$ Guanidin

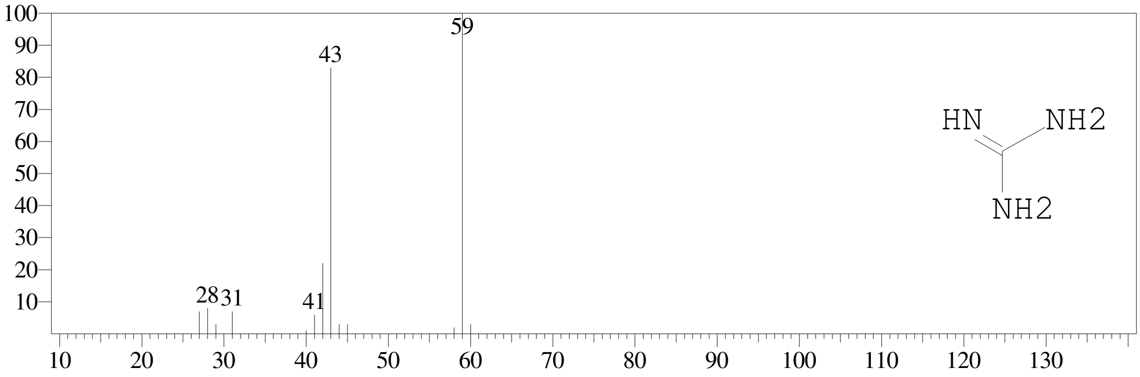

<< Target >>
Line#:1 R.Time:3.475(Scan#:118) MassPeaks:2
RawMode:Averaged 3.467-3.483(117-119) BasePeak:59.00(3372)
BG Mode:None Group 1 - Event 1 Scan

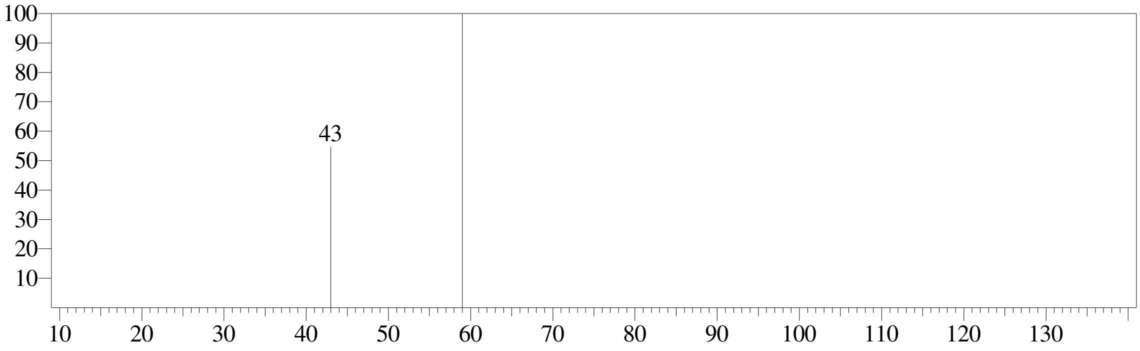

Hit#:3 Entry:9784 Library:NIST23-1.lib
SI:92 Formula:C6H12O3 CAS:80-55-7 MolWeight:132 RetIndex:843
CompName:Propanoic acid, 2-hydroxy-2-methyl-, ethyl ester $$ Lactic acid, 2-methyl-, ethyl ester $$ Ethyl .alpha.-hydro:

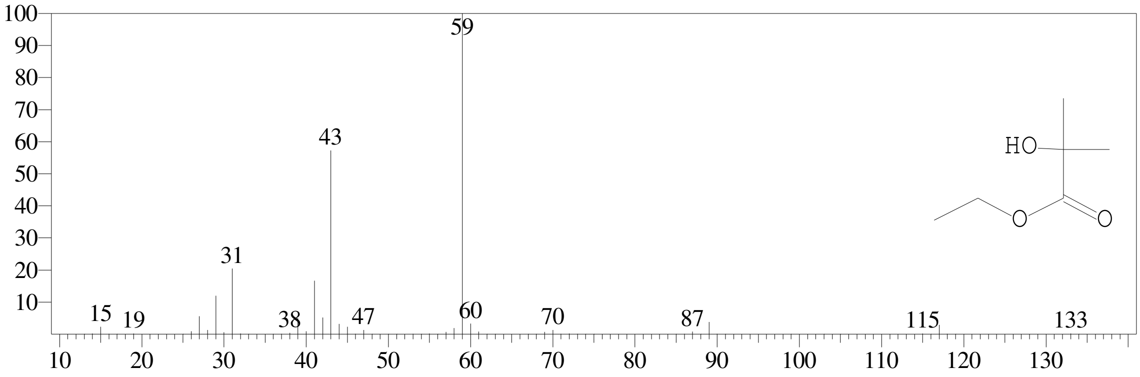

Hit#:4 Entry:121 Library:NIST23-1.lib
SI:92 Formula:CH5N3 CAS:113-00-8 MolWeight:59 RetIndex:1188
CompName:Guanidine $$ Aminoformamidine $$ Aminomethanamidine $$ Carbamamidine $$ Carbamidine $$ Guanidin

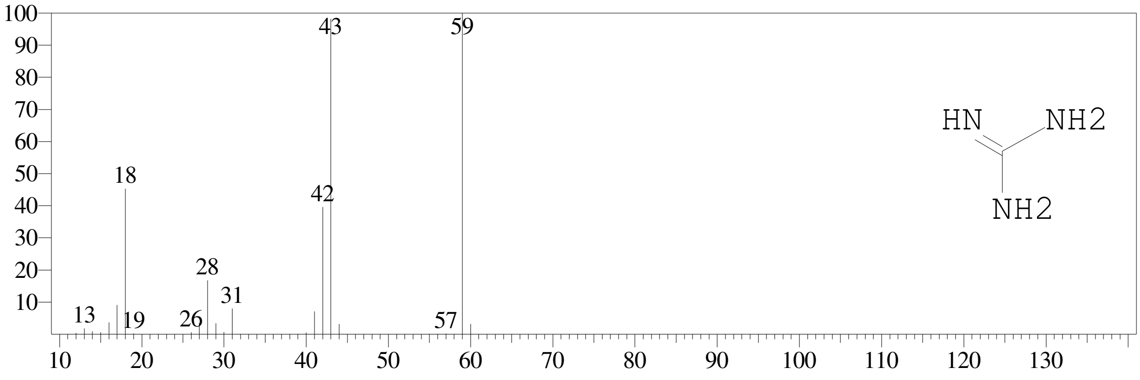

<< Target >>
Line#:1   R.Time:3.475(Scan#:118) MassPeaks:2
RawMode:Averaged 3.467-3.483(117-119) BasePeak:59.00(3372)
BG Mode:None Group 1 - Event 1 Scan

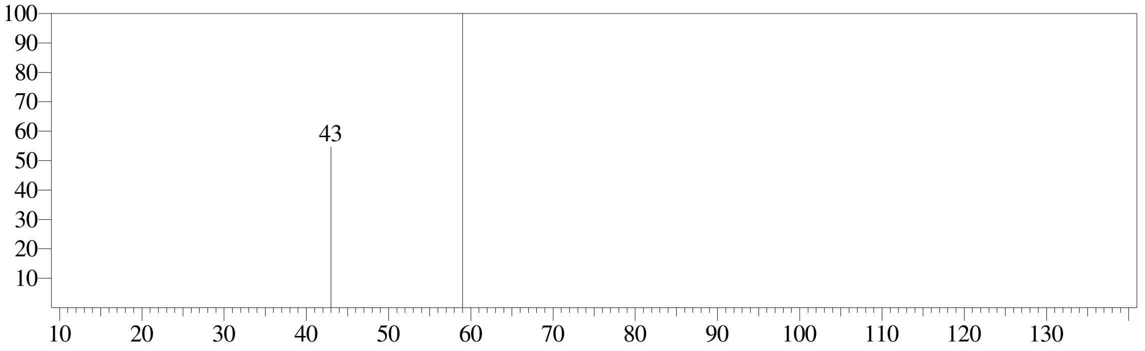

Hit#:5 Entry:2213 Library:NIST23-1.lib
SI:92 Formula:C6H12O CAS:624-97-5 MolWeight:100 RetIndex:702
CompName:4-Pentene-2-ol, 2-methyl $$ CH2=CHCH2C(CH3)2OH $$ 1-Pentene-4-ol, 4-methyl $$ 4-Penten-2-ol, 2-met

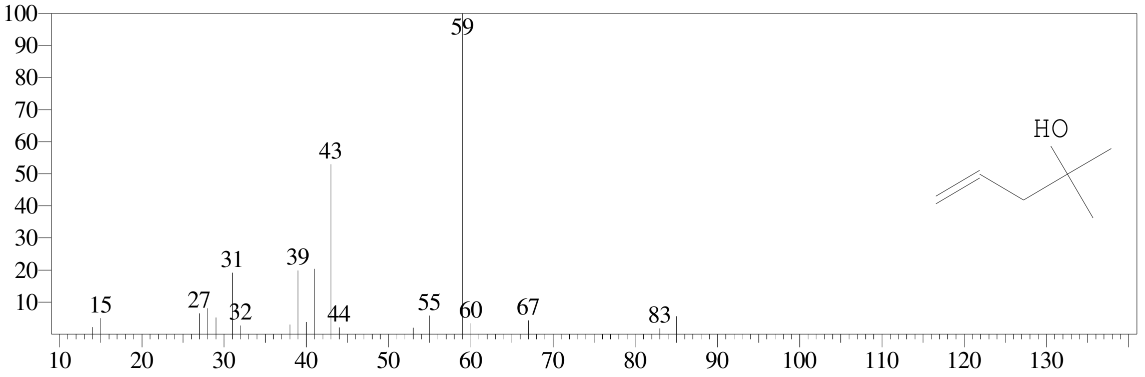

<< Target >>
Line#:2   R.Time:3.542(Scan#:126) MassPeaks:11
RawMode:Averaged 3.533-3.550(125-127) BasePeak:45.00(50491)
BG Mode:None Group 1 - Event 1 Scan

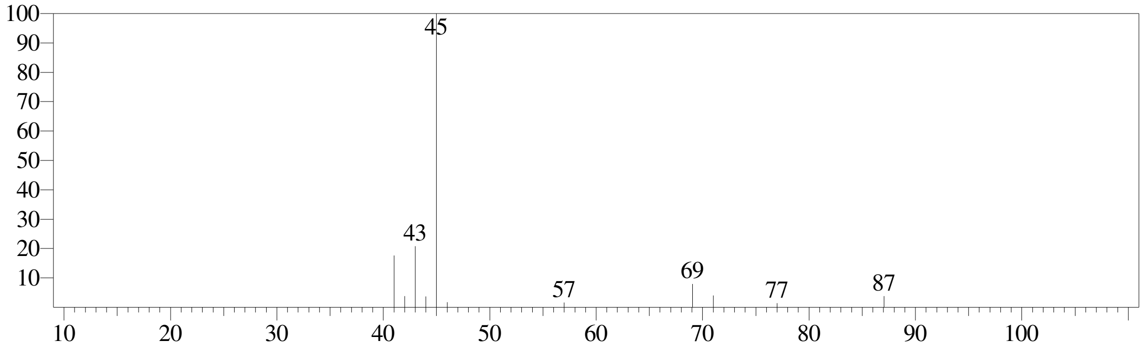

Hit#:1 Entry:2534 Library:NIST23-1.lib
SI:93 Formula:C6H14O CAS:26549-24-6 MolWeight:102 RetIndex:791
CompName:2-Hexanol, (R)- $$ (R)-(-)-2-Hexanol $$ 2-Hexanol # $$

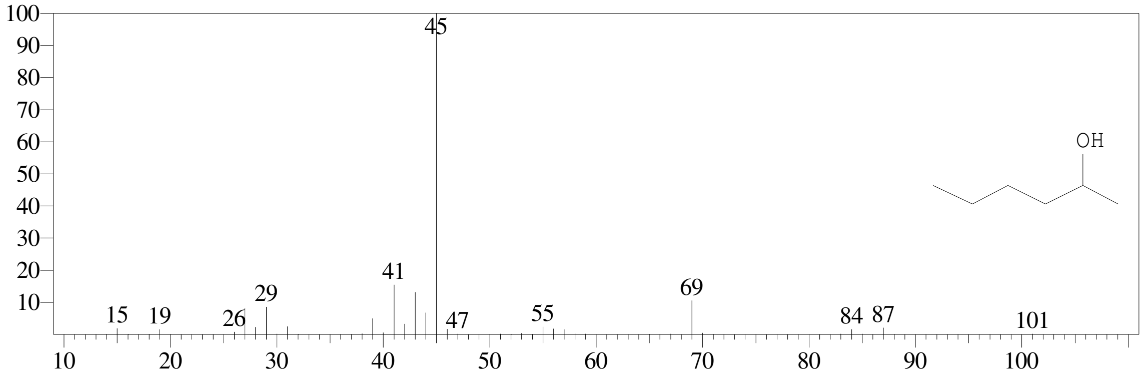

Hit#:2 Entry:2533 Library:NIST23-1.lib
SI:93 Formula:C6H14O CAS:52019-78-0 MolWeight:102 RetIndex:791
CompName:2-Hexanol, (S)- $$ (S)-(+)-2-Hexanol $$ 2-Hexanol # $$

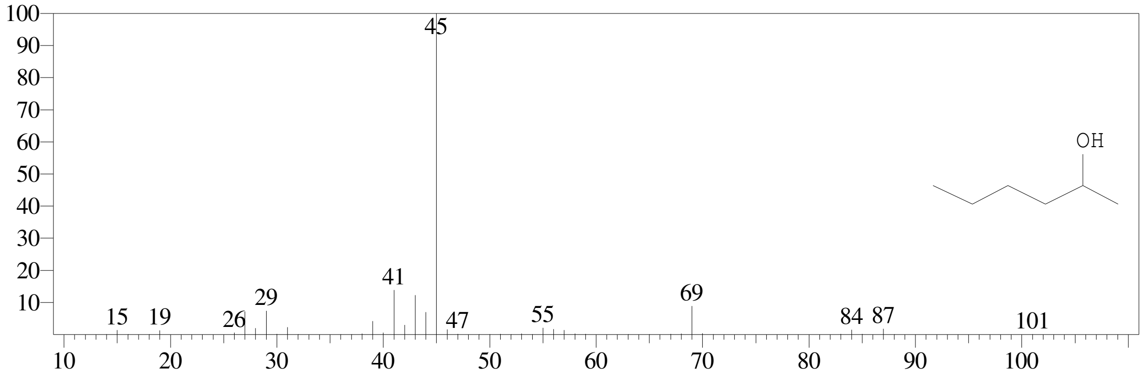

<< Target >>
Line#:2   R.Time:3.542(Scan#:126) MassPeaks:11
RawMode:Averaged 3.533-3.550(125-127) BasePeak:45.00(50491)
BG Mode:None Group 1 - Event 1 Scan

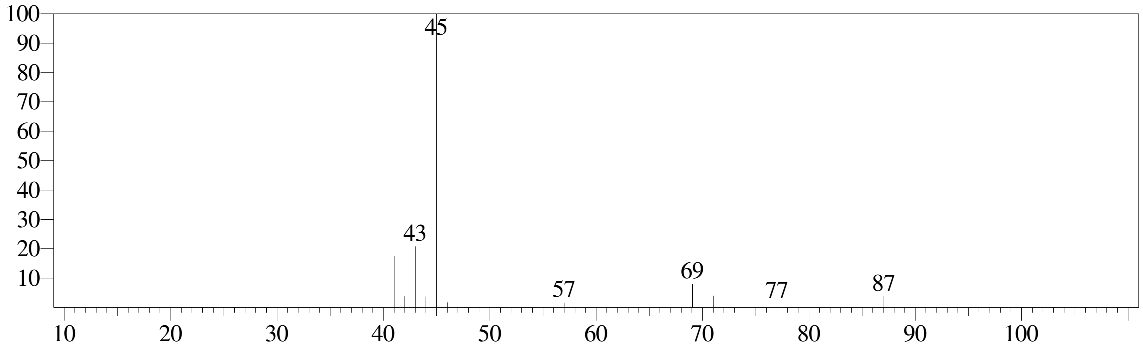

Hit#:3 Entry:2597 Library:NIST23s.lib
SI:93 Formula:C6H14O CAS:108-11-2 MolWeight:102 RetIndex:752
CompName:2-Pentanol, 4-methyl- $$ Isobutylmethylcarbinol $$ Isobutylmethylmethanol $$ Methylisobutylcarbinol $$ M

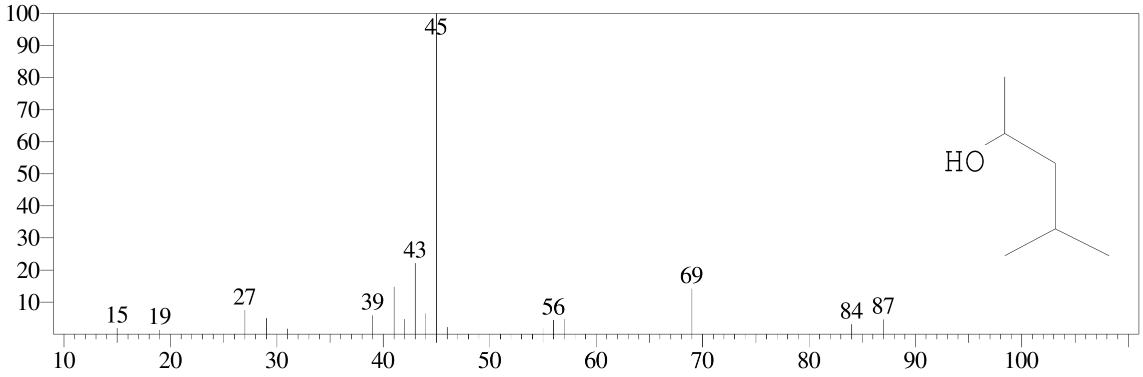

Hit#:4 Entry:1082 Library:NIST23s.lib
SI:92 Formula:C5H10O CAS:625-31-0 MolWeight:86 RetIndex:658
CompName:4-Penten-2-ol $$ 1-Penten-4-ol $$ 4-Hydroxypent-1-ene $$ CH2=CHCH2CH(OH)CH3 $$

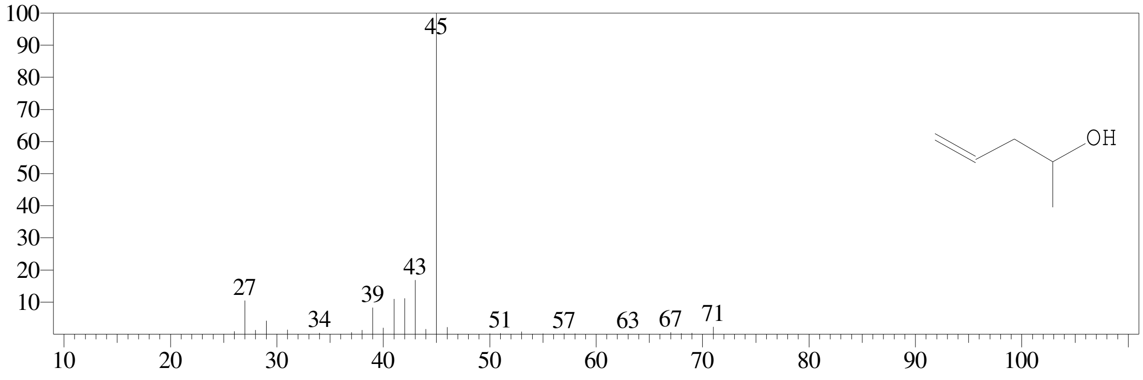

<< Target >>
Line#:2   R.Time:3.542(Scan#:126) MassPeaks:11
RawMode:Averaged 3.533-3.550(125-127) BasePeak:45.00(50491)
BG Mode:None Group 1 - Event 1 Scan

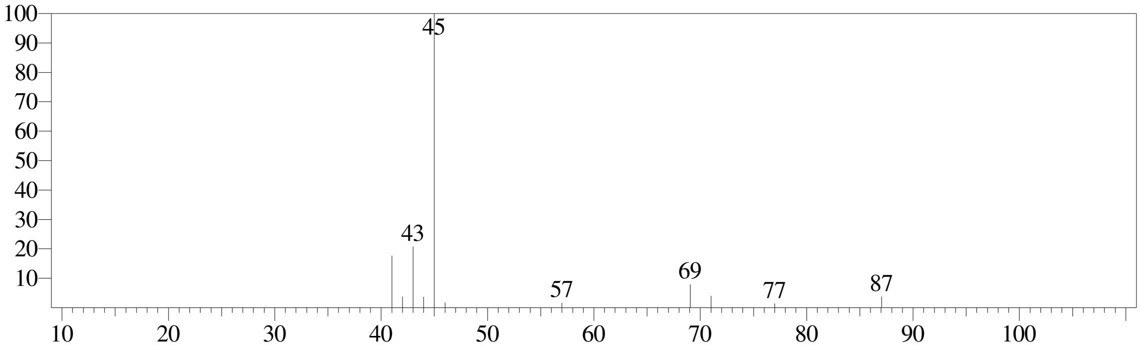

Hit#:5 Entry:897 Library:NIST23-1.lib
SI:92 Formula:C5H10O CAS:625-31-0 MolWeight:86 RetIndex:658
CompName:4-Penten-2-ol $$ 1-Penten-4-ol $$ 4-Hydroxypent-1-ene $$ CH2=CHCH2CH(OH)CH3 $$

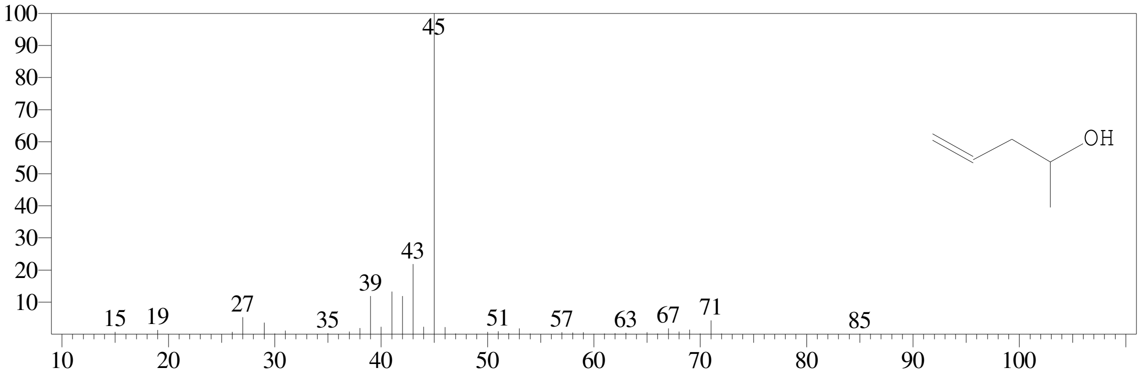

<< Target >>
Line#:3   R.Time:3.625(Scan#:136) MassPeaks:5
RawMode:Averaged 3.617-3.633(135-137) BasePeak:59.00(10989)
BG Mode:None Group 1 - Event 1 Scan

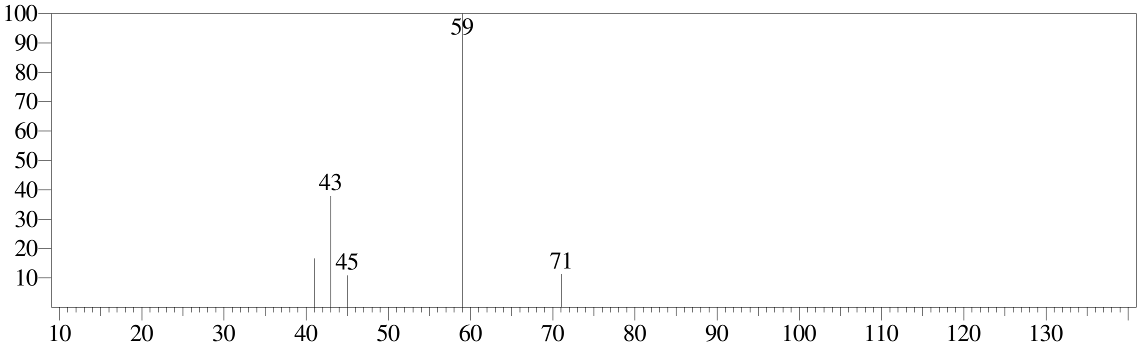

Hit#:1 Entry:2726 Library:NIST23-1.lib
SI:90 Formula:C4H8O3 CAS:594-61-6 MolWeight:104 RetIndex:932
CompName:Propanoic acid, 2-hydroxy-2-methyl- $$ 2-Hydroxyisobutyric acid $$ Lactic acid, 2-methyl- $$ .alpha.-Hydrc

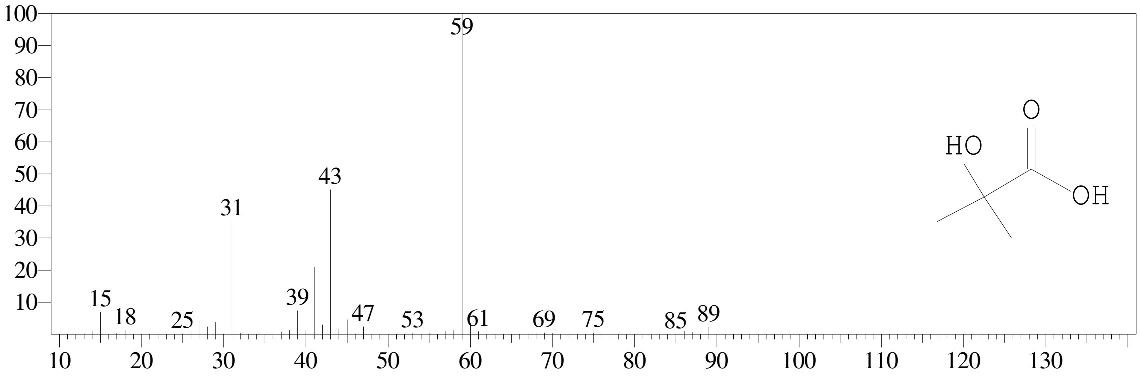

Hit#:2 Entry:2466 Library:NIST23-1.lib
SI:90 Formula:C5H10O2 CAS:115-22-0 MolWeight:102 RetIndex:737
CompName:3-Hydroxy-3-methyl-2-butanone $$ 2-Butanone, 3-hydroxy-3-methyl- $$ Dimethylacetylcarbinol $$ 3-Hydrc

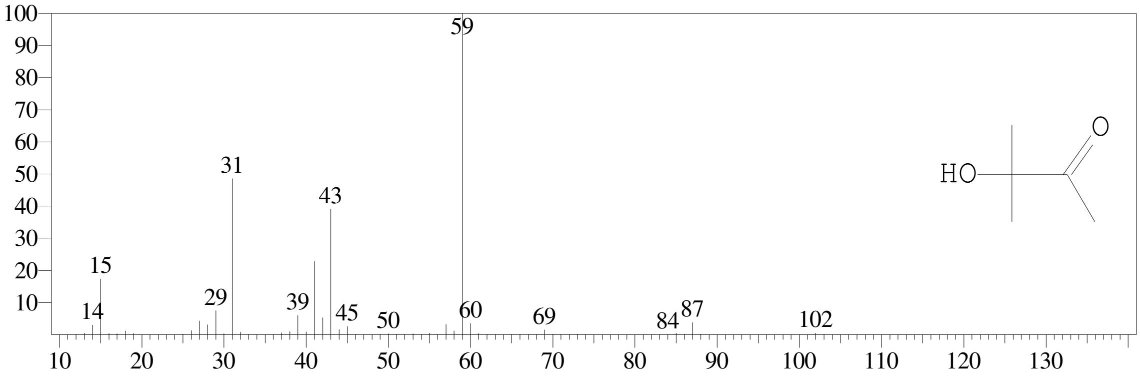

<< Target >>
Line#:3   R.Time:3.625(Scan#:136) MassPeaks:5
RawMode:Averaged 3.617-3.633(135-137) BasePeak:59.00(10989)
BG Mode:None Group 1 - Event 1 Scan

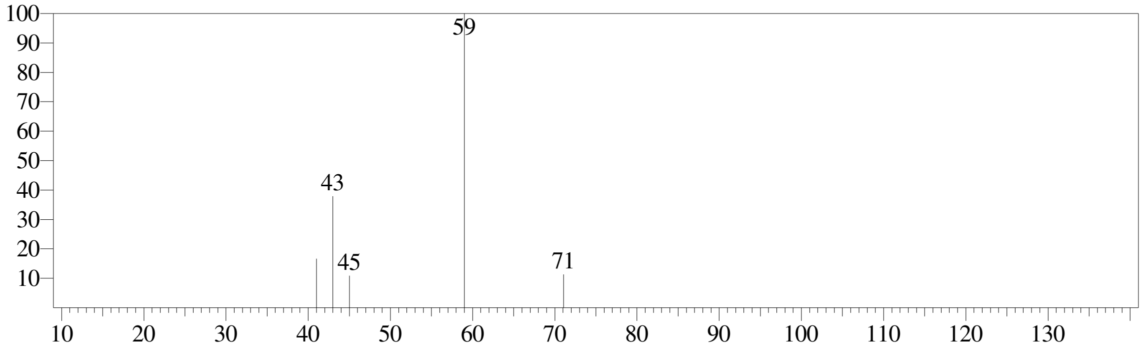

Hit#:3 Entry:2775 Library:NIST23-1.lib
SI:90 Formula:C5H12O2 CAS:5396-58-7 MolWeight:104 RetIndex:812
CompName:2-Methyl-2,3-butanediol $$ 2-Methylbutane-2,3-diol $$ 2,3-Dihydroxy-2-methylbutane $$

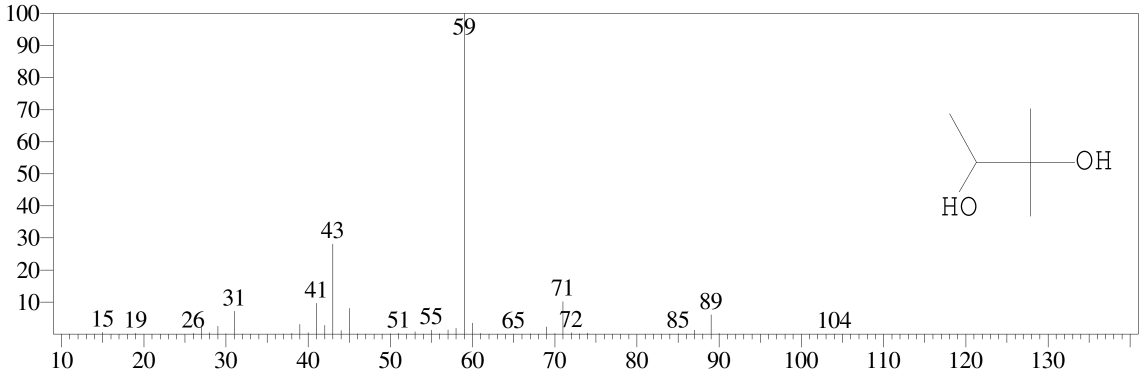

Hit#:4 Entry:9783 Library:NIST23-1.lib
SI:89 Formula:C6H12O3 CAS:70657-70-4 MolWeight:132 RetIndex:880
CompName:2-Methoxypropyl acetate $$ 1-Propanol, 2-methoxy-, 1-acetate $$ 1-Propanol, 2-methoxy-, acetate $$ 2-Meth

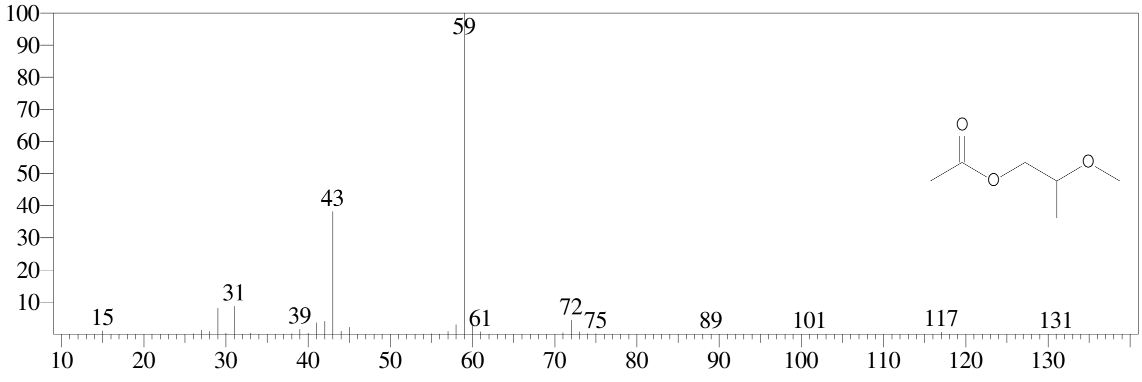

<< Target >>
Line#:3   R.Time:3.625(Scan#:136) MassPeaks:5
RawMode:Averaged 3.617-3.633(135-137) BasePeak:59.00(10989)
BG Mode:None Group 1 - Event 1 Scan

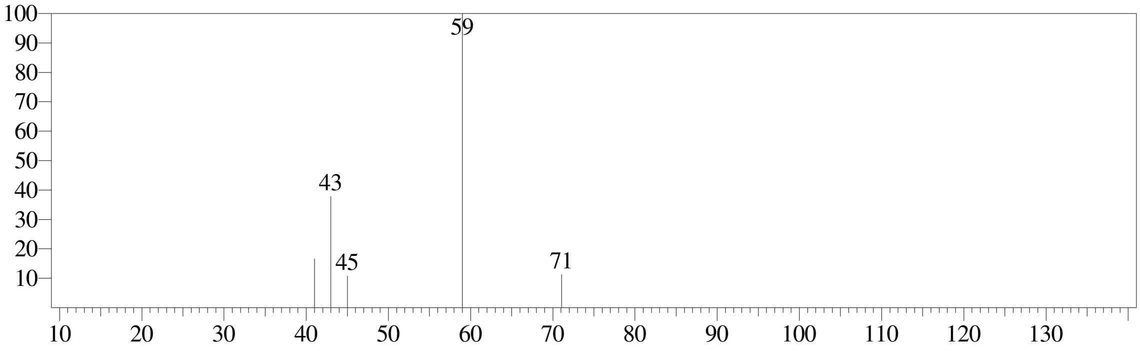

Hit#:5 Entry:583 Library:NIST23s.lib
SI:89 Formula:C4H10O CAS:598-53-8 MolWeight:74 RetIndex:471
CompName:Propane, 2-methoxy- $$ Ether, isopropyl methyl $$ Isopropyl methyl ether $$ Isopryl $$ Methyl isopropyl et

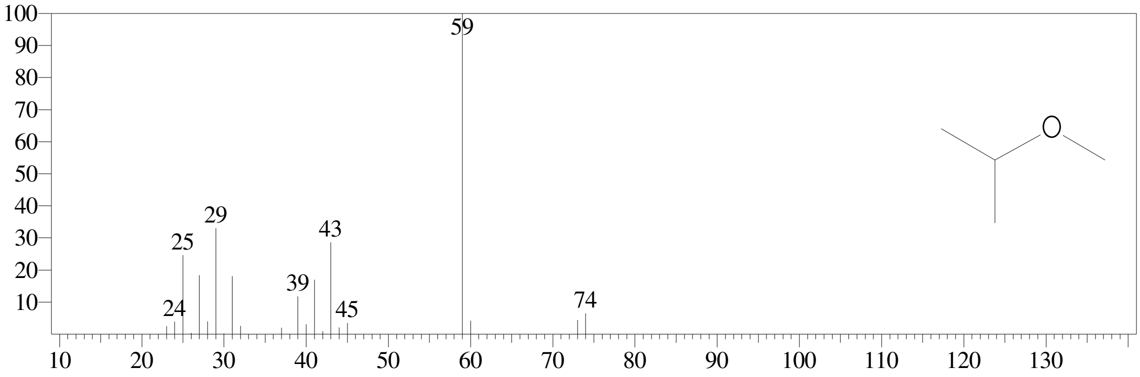

<< Target >>
Line#:4 R.Time:4.325(Scan#:220) MassPeaks:5
RawMode:Averaged 4.317-4.333(219-221) BasePeak:55.00(938)
BG Mode:Calc. from Peak Group 1 - Event 1 Scan

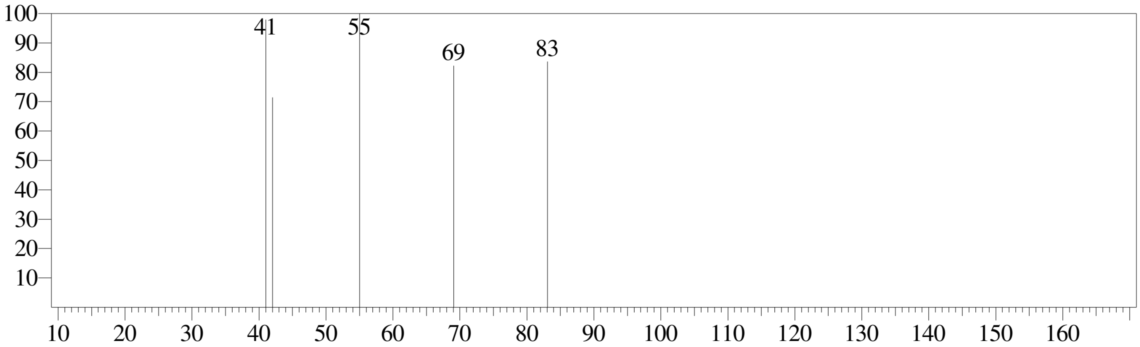

Hit#:1 Entry:654 Library:NIST23-1.lib
SI:80 Formula:C5H9N CAS:505-18-0 MolWeight:83 RetIndex:787
CompName:Pyridine, 2,3,4,5-tetrahydro- $$ .delta.1-Piperideine $$ 1-Piperideine $$ 2,3,4,5-Tetrahydropyridine $$ Tetrah

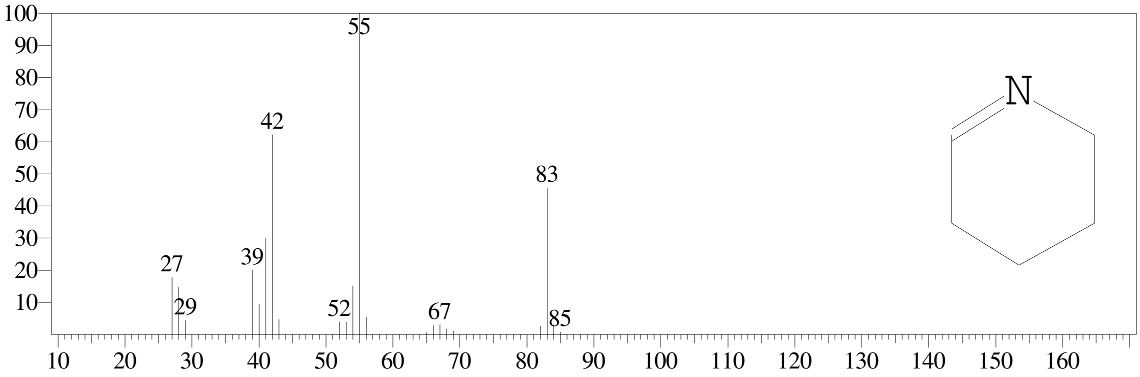

Hit#:2 Entry:25622 Library:NIST23-1.lib
SI:79 Formula:C6H11Br CAS:2270-59-9 MolWeight:162 RetIndex:944
CompName:5-Bromo-2-methyl-2-pentene

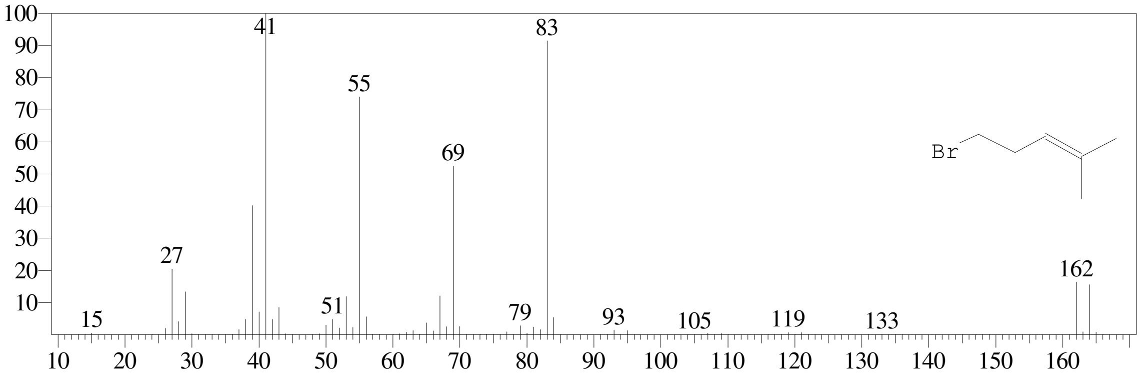

<< Target >>
Line#:4 R.Time:4.325(Scan#:220) MassPeaks:5
RawMode:Averaged 4.317-4.333(219-221) BasePeak:55.00(938)
BG Mode:Calc. from Peak Group 1 - Event 1 Scan

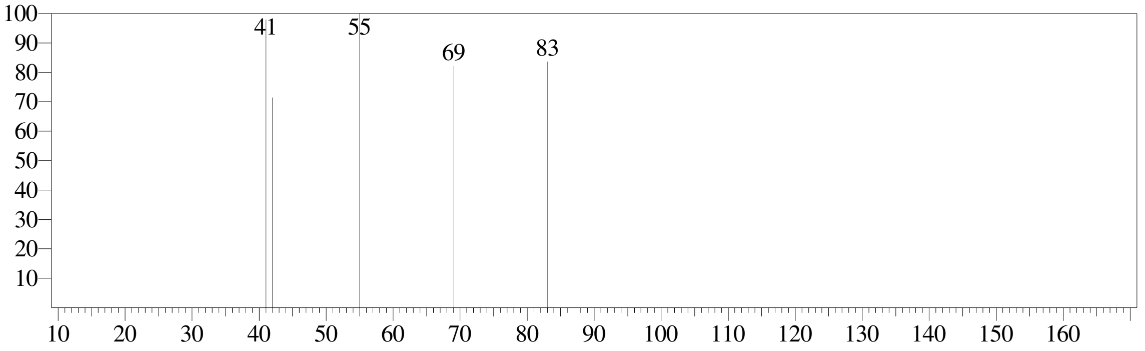

Hit#:3 Entry:649 Library:NIST23-1.lib
SI:79 Formula:C5H9N CAS:80839-91-4 MolWeight:83 RetIndex:793
CompName:Aziridine, 1-(1-propenyl)-, (E)- $$ (E)-1-Propenylaziridine $$ (E)-1-Aziridino-1-propene $$

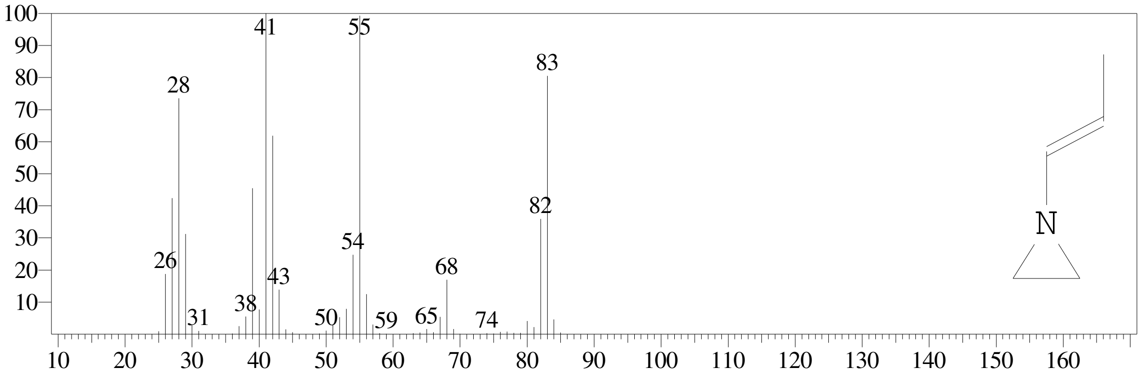

Hit#:4 Entry:14597 Library:NIST23s.lib
SI:78 Formula:C6H11Br CAS:2270-59-9 MolWeight:162 RetIndex:944
CompName:5-Bromo-2-methyl-2-pentene

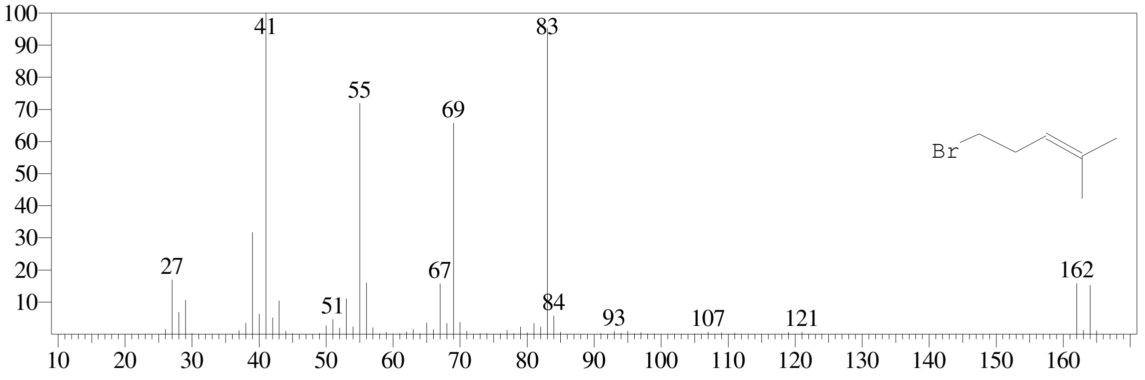

<< Target >>
Line#:4   R.Time:4.325(Scan#:220) MassPeaks:5
RawMode:Averaged 4.317-4.333(219-221) BasePeak:55.00(938)
BG Mode:Calc. from Peak Group 1 - Event 1 Scan

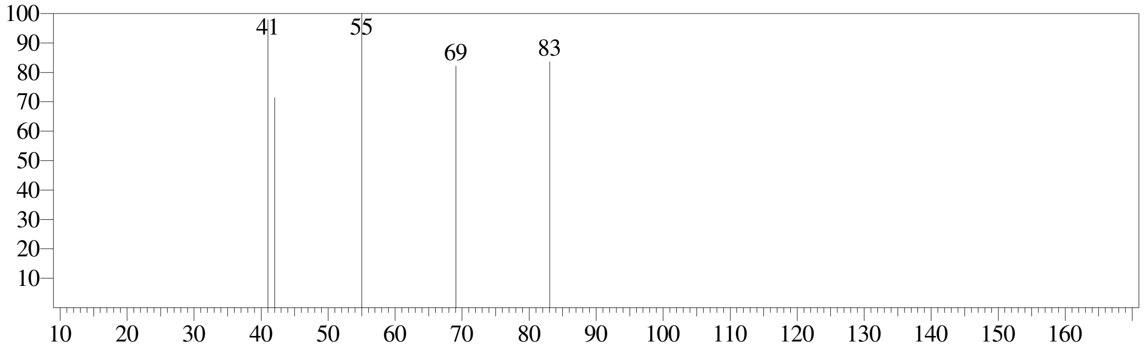

Hit#:5 Entry:1983 Library:NIST23s.lib
SI:77 Formula:C7H14 CAS:4127-47-3 MolWeight:98 RetIndex:626
CompName:Cyclopropane, 1,1,2,2-tetramethyl- $$ 1,1,2,2-Tetramethylcyclopropane $$

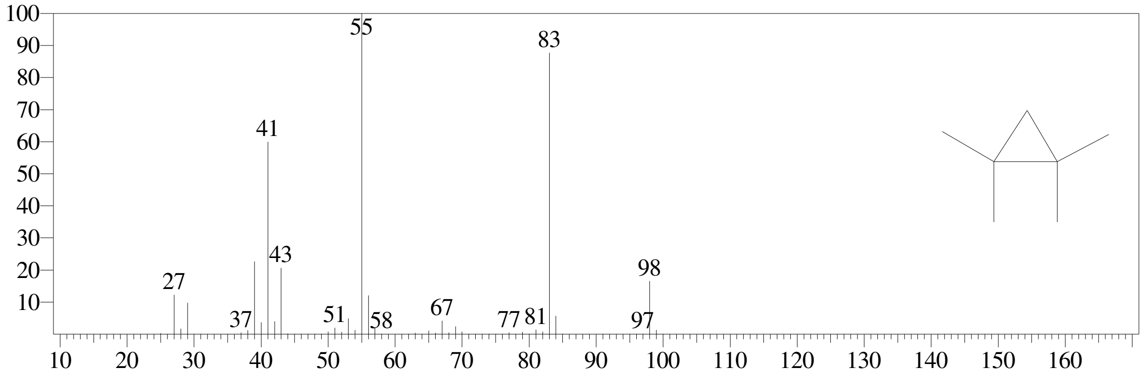

<< Target >>
Line#:5 R.Time:4.367(Scan#:225) MassPeaks:6
RawMode:Averaged 4.358-4.375(224-226) BasePeak:67.00(4649)
BG Mode:Calc. from Peak Group 1 - Event 1 Scan

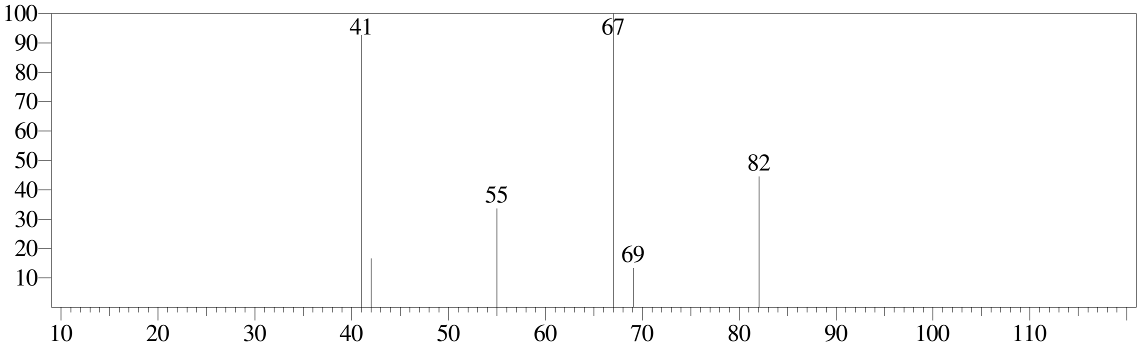

Hit#:1 Entry:6482 Library:NIST23s.lib
SI:86 Formula:C7H12O2 CAS:33467-73-1 MolWeight:128 RetIndex:931
CompName:3-Hexen-1-ol, formate, (Z)- $$ cis-.beta.-Hexenyl formate $$ cis-3-Hexenyl formate $$ cis-3-Hexen-1-ol forr

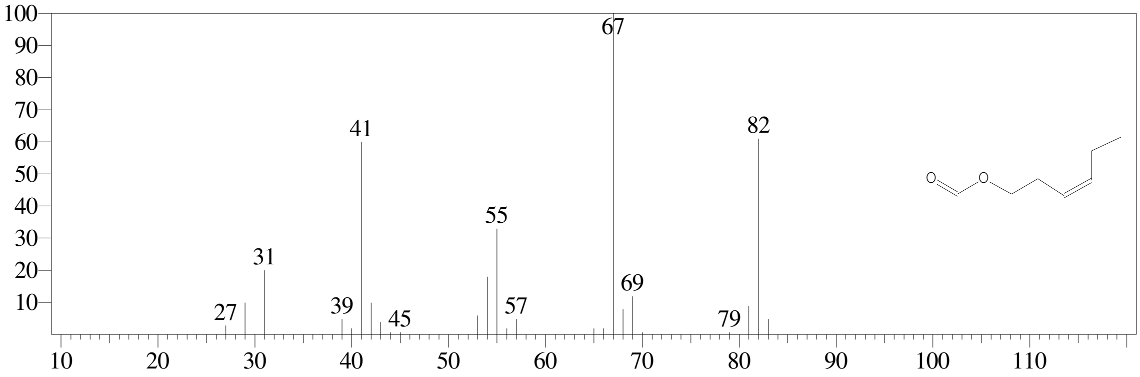

Hit#:2 Entry:6479 Library:NIST23s.lib
SI:83 Formula:C7H12O2 CAS:33467-73-1 MolWeight:128 RetIndex:931
CompName:3-Hexen-1-ol, formate, (Z)- $$ cis-.beta.-Hexenyl formate $$ cis-3-Hexenyl formate $$ cis-3-Hexen-1-ol forr

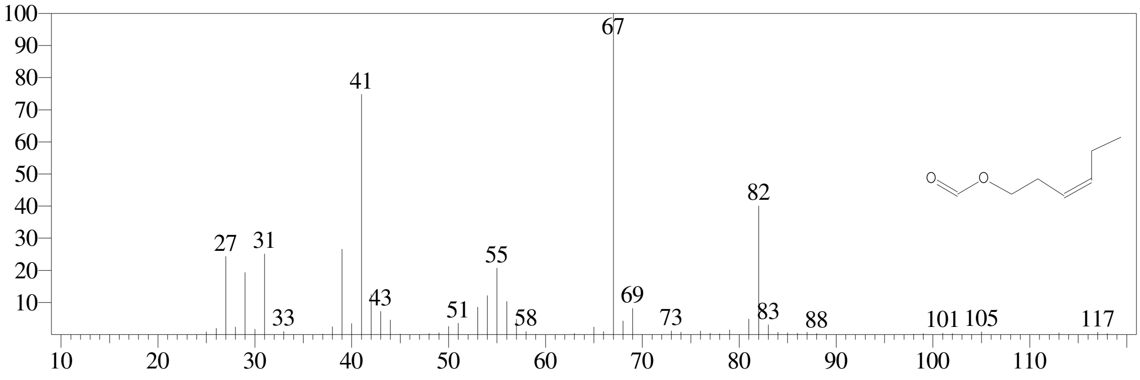

<< Target >>
Line#:5   R.Time:4.367(Scan#:225) MassPeaks:6
RawMode:Averaged 4.358-4.375(224-226) BasePeak:67.00(4649)
BG Mode:Calc. from Peak Group 1 - Event 1 Scan

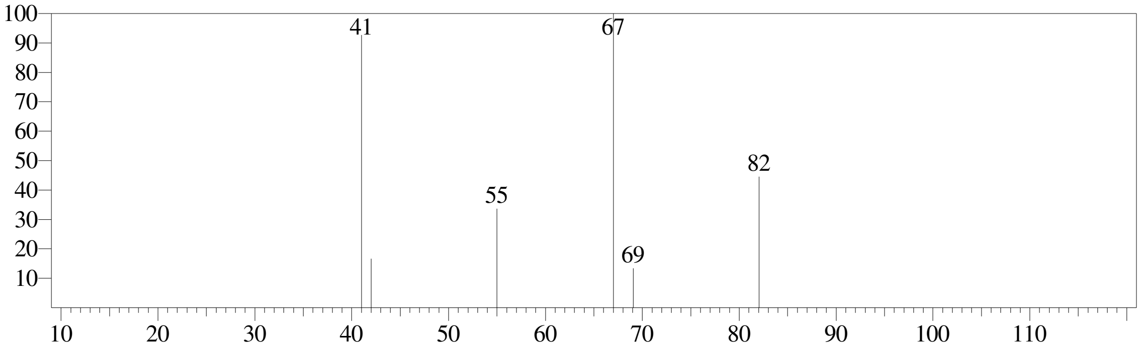

Hit#:3 Entry:8273 Library:NIST23-1.lib
SI:83 Formula:C7H12O2 CAS:33467-73-1 MolWeight:128 RetIndex:931
CompName:3-Hexen-1-ol, formate, (Z)- $$ cis-.beta.-Hexenyl formate $$ cis-3-Hexenyl formate $$ cis-3-Hexen-1-ol forr

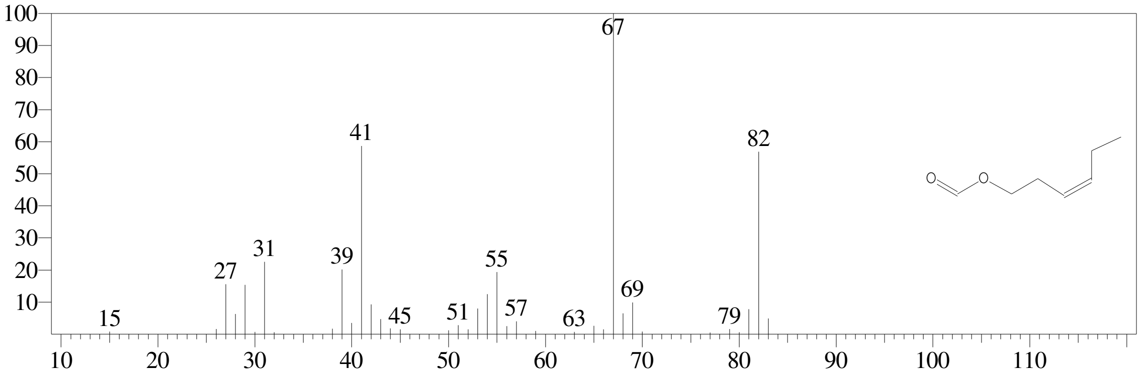

Hit#:4 Entry:6483 Library:NIST23s.lib
SI:83 Formula:C7H12O2 CAS:33467-73-1 MolWeight:128 RetIndex:931
CompName:3-Hexen-1-ol, formate, (Z)- $$ cis-.beta.-Hexenyl formate $$ cis-3-Hexenyl formate $$ cis-3-Hexen-1-ol forr

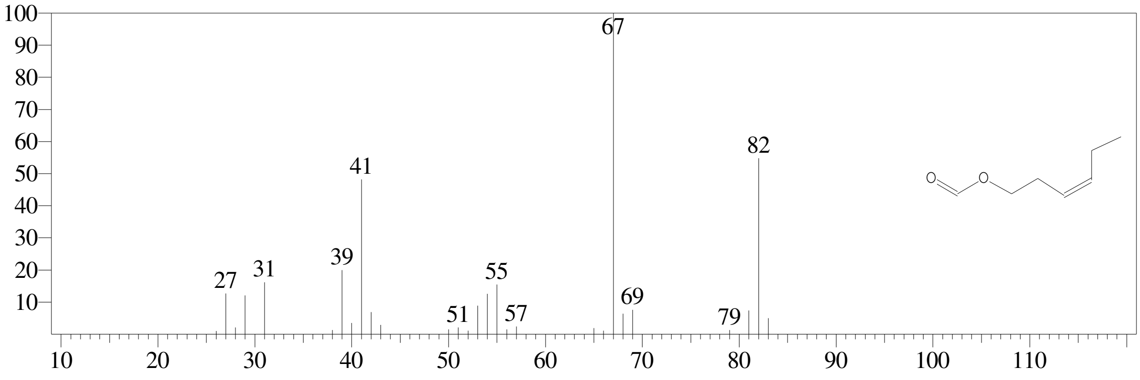

<< Target >>
Line#:5 R.Time:4.367(Scan#:225) MassPeaks:6
RawMode:Averaged 4.358-4.375(224-226) BasePeak:67.00(4649)
BG Mode:Calc. from Peak Group 1 - Event 1 Scan

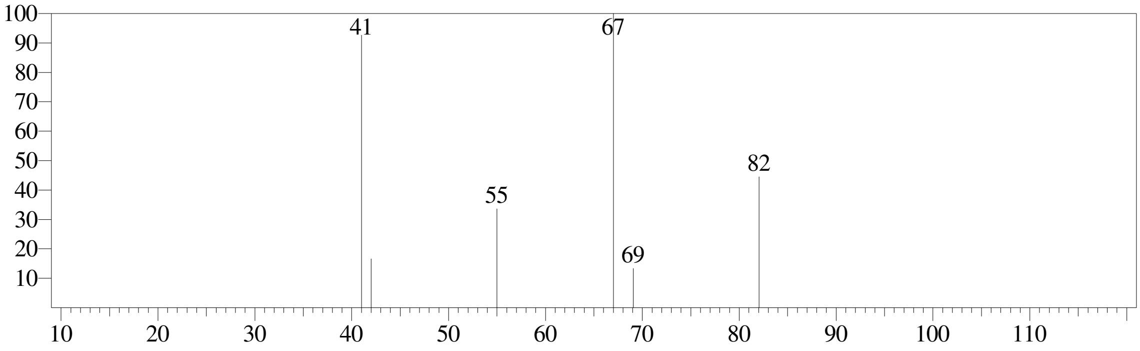

Hit#:5 Entry:2307 Library:NIST23s.lib
SI:82 Formula:C6H12O CAS:928-97-2 MolWeight:100 RetIndex:857
CompName:3-Hexen-1-ol, (E)- $$ trans-3-Hexen-1-ol $$ trans-3-Hexenol $$ E-3-Hexenol $$ (E)-Hex-3-en-1-ol $$ (3E)-

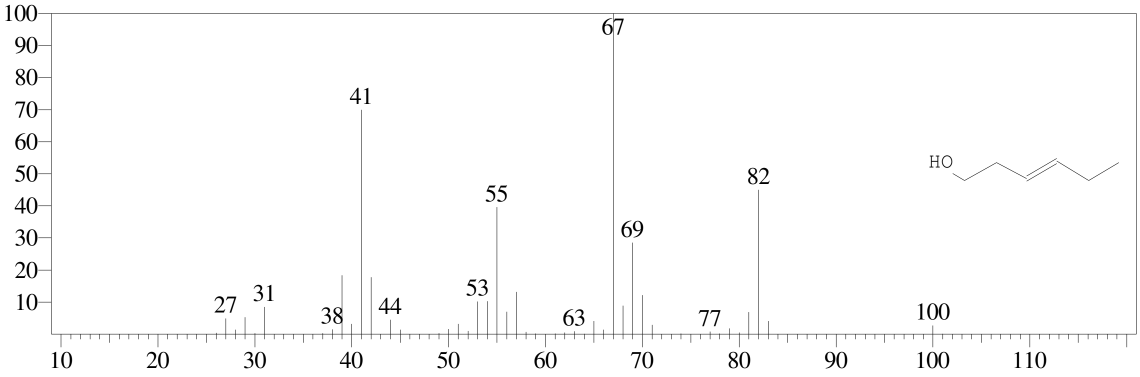

<< Target >>
Line#:6 R.Time:25.000(Scan#:2701) MassPeaks:19
RawMode:Averaged 24.992-25.008(2700-2702) BasePeak:93.05(4266)
BG Mode:None Group 1 - Event 1 Scan

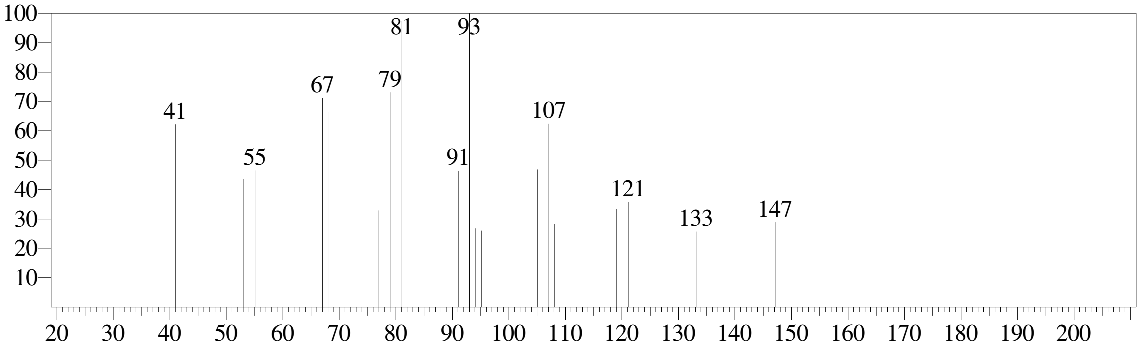

Hit#:1 Entry:24863 Library:NIST23s.lib
SI:84 Formula:C15H24 CAS:515-13-9 MolWeight:204 RetIndex:1398
CompName:Cyclohexane, 1-ethenyl-1-methyl-2,4-bis(1-methylethenyl)-, [1S-(1.alpha.,2.beta.,4.beta.)]- $$ Cyclohexane, 1

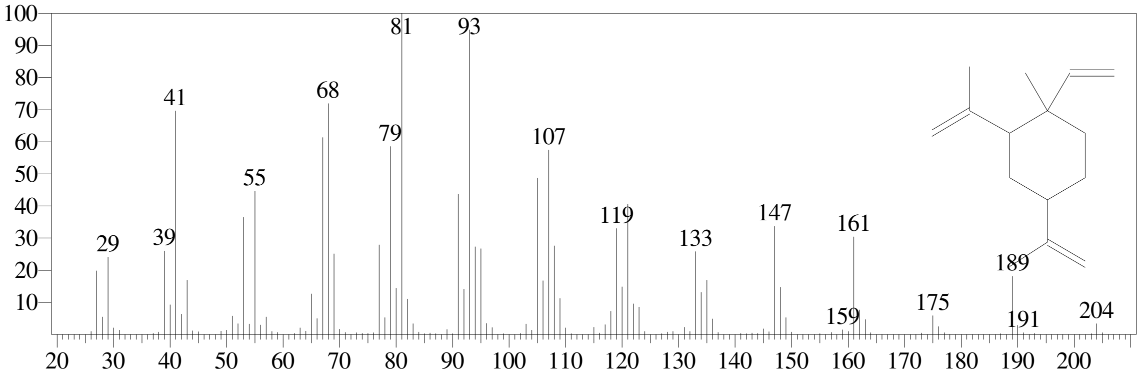

Hit#:2 Entry:24889 Library:NIST23s.lib
SI:83 Formula:C15H24 CAS:515-13-9 MolWeight:204 RetIndex:1398
CompName:Cyclohexane, 1-ethenyl-1-methyl-2,4-bis(1-methylethenyl)-, [1S-(1.alpha.,2.beta.,4.beta.)]- $$ Cyclohexane, 1

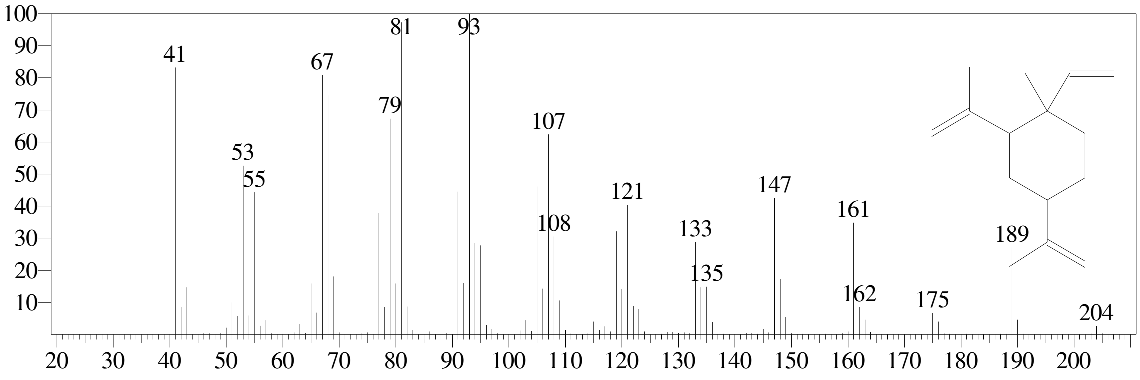

<< Target >>
Line#:6 R.Time:25.000(Scan#:2701) MassPeaks:19
RawMode:Averaged 24.992-25.008(2700-2702) BasePeak:93.05(4266)
BG Mode:None Group 1 - Event 1 Scan

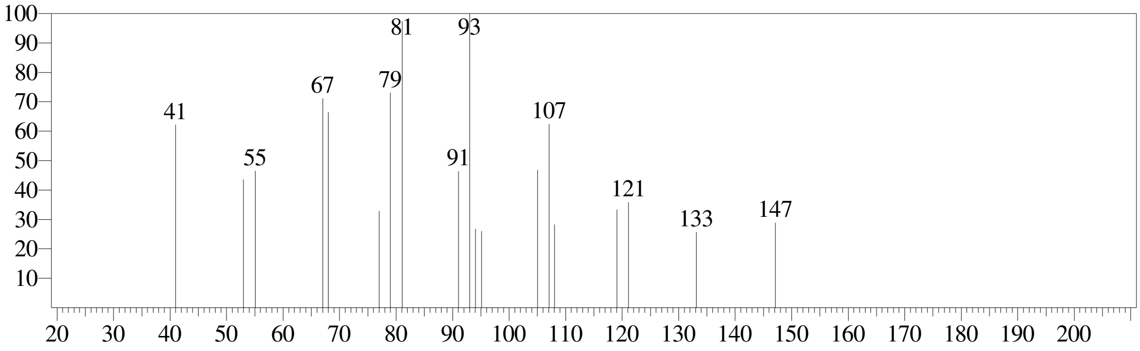

Hit#:3 Entry:62785 Library:NIST23-1.lib
SI:82 Formula:C15H24 CAS:110823-68-2 MolWeight:204 RetIndex:1398
CompName:Cyclohexane, 1-ethenyl-1-methyl-2,4-bis(1-methylethenyl)- $$ 2,4-Diisopropenyl-1-methyl-1-vinylcyclohexa

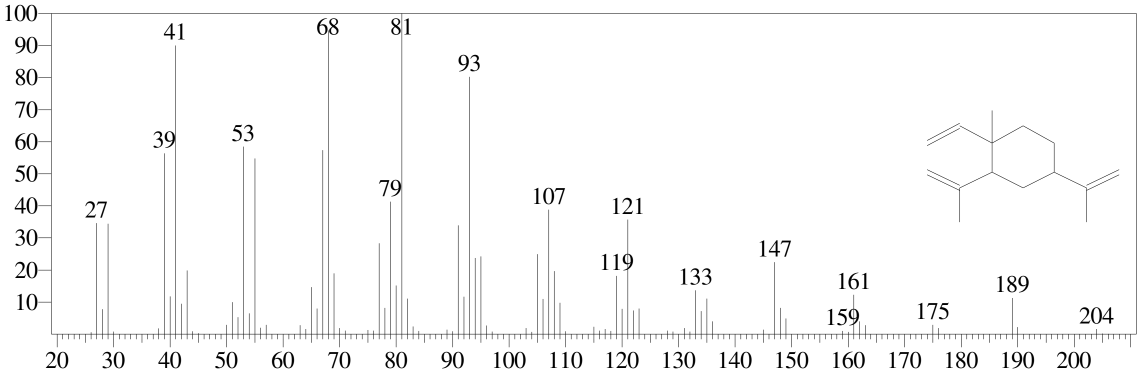

Hit#:4 Entry:62789 Library:NIST23-1.lib
SI:82 Formula:C15H24 CAS:515-13-9 MolWeight:204 RetIndex:1398
CompName:Cyclohexane, 1-ethenyl-1-methyl-2,4-bis(1-methylethenyl)-, [1S-(1.alpha.,2.beta.,4.beta.)]- $$ Cyclohexane, 1

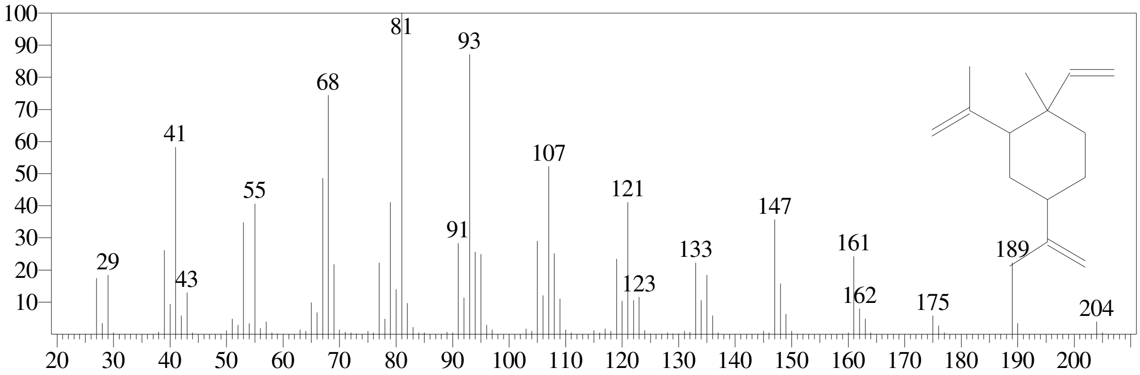

<< Target >>
Line#:6 R.Time:25.000(Scan#:2701) MassPeaks:19
RawMode:Averaged 24.992-25.008(2700-2702) BasePeak:93.05(4266)
BG Mode:None Group 1 - Event 1 Scan

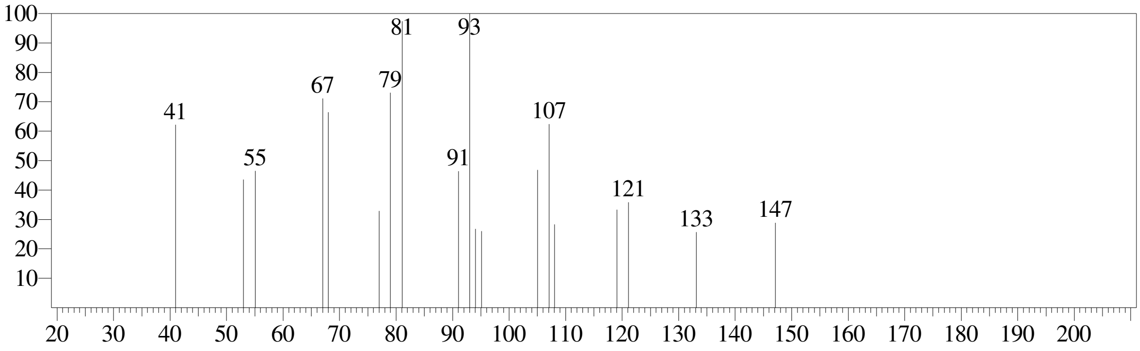

Hit#:5 Entry:62786 Library:NIST23-1.lib
SI:81 Formula:C15H24 CAS:33880-83-0 MolWeight:204 RetIndex:1398
CompName:Cyclohexane, 1-ethenyl-1-methyl-2,4-bis(1-methylethenyl)-, (1.alpha.,2.beta.,4.beta.)- $$ 2,4-Diisopropenyl-1

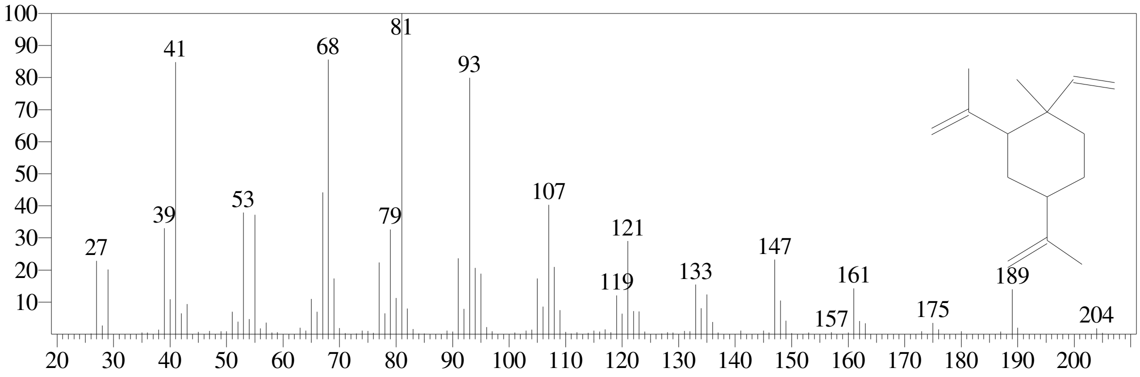

<< Target >>
Line#:7 R.Time:26.133(Scan#:2837) MassPeaks:7
RawMode:Averaged 26.125-26.142(2836-2838) BasePeak:41.00(1868)
BG Mode:None Group 1 - Event 1 Scan

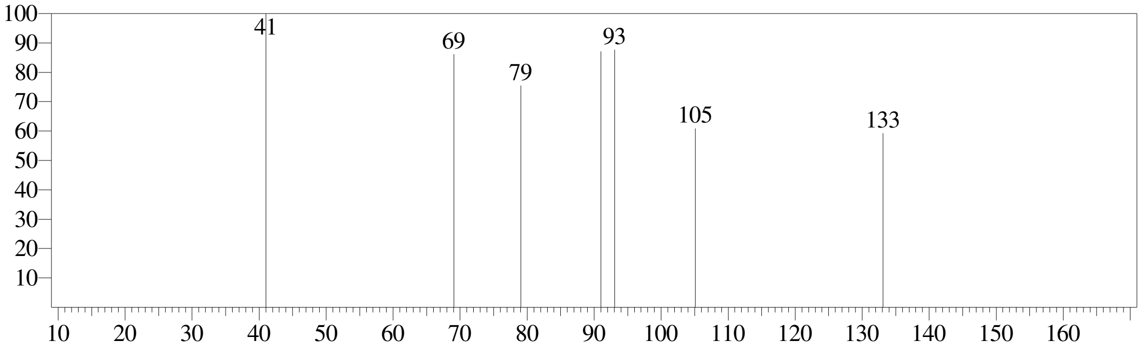

Hit#:1 Entry:25390 Library:NIST23-1.lib
SI:71 Formula:C10H11NO CAS:57276-33-2 MolWeight:161 RetIndex:1472
CompName:Ethanone, 1-cyclopropyl-2-(3-pyridinyl)- $$ Cyclopropyl 3-picolyl ketone $$ 1-Cyclopropyl-2-(3-pyridinyl)e

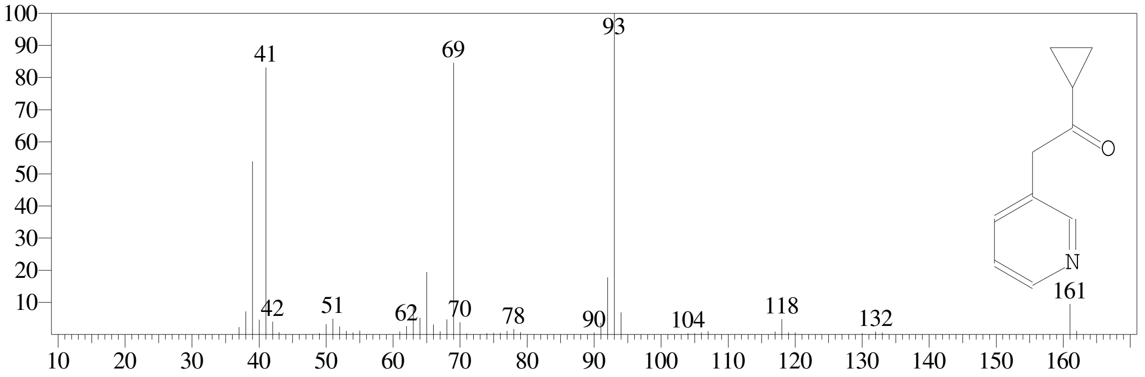

Hit#:2 Entry:8395 Library:NIST23s.lib
SI:71 Formula:C10H16 CAS:123-35-3 MolWeight:136 RetIndex:993
CompName:.beta.-Myrcene $$ 1,6-Octadiene, 7-methyl-3-methylene- $$ Myrcene $$ 7-Methyl-3-methylene-1,6-octadien

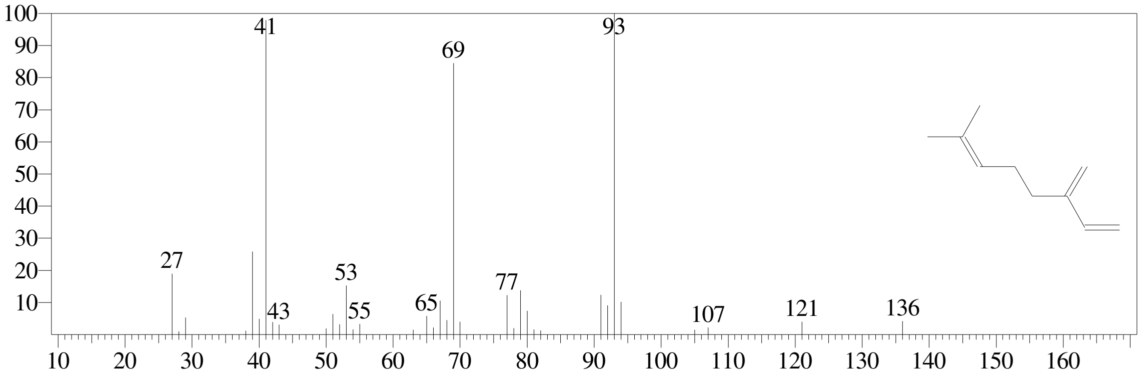

<< Target >>
Line#:7 R.Time:26.133(Scan#:2837) MassPeaks:7
RawMode:Averaged 26.125-26.142(2836-2838) BasePeak:41.00(1868)
BG Mode:None Group 1 - Event 1 Scan

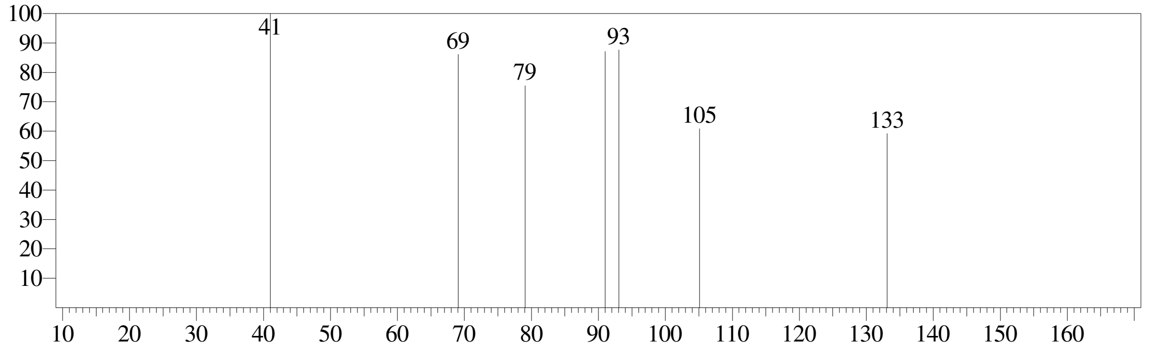

Hit#:3 Entry:11317 Library:NIST23-1.lib
SI:70 Formula:C10H16 CAS:123-35-3 MolWeight:136 RetIndex:993
CompName:.beta.-Myrcene $$ 1,6-Octadiene, 7-methyl-3-methylene- $$ Myrcene $$ 7-Methyl-3-methylene-1,6-octadien

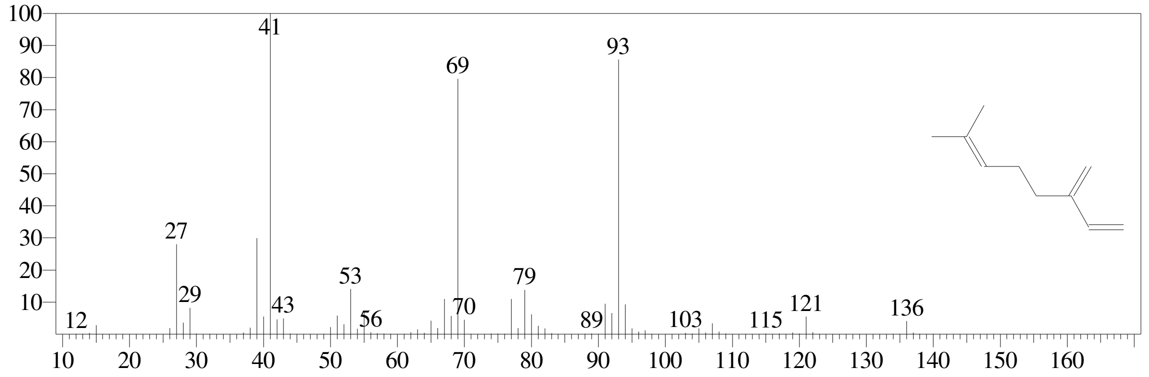

Hit#:4 Entry:8406 Library:NIST23s.lib
SI:69 Formula:C10H16 CAS:123-35-3 MolWeight:136 RetIndex:993
CompName:.beta.-Myrcene $$ 1,6-Octadiene, 7-methyl-3-methylene- $$ Myrcene $$ 7-Methyl-3-methylene-1,6-octadien

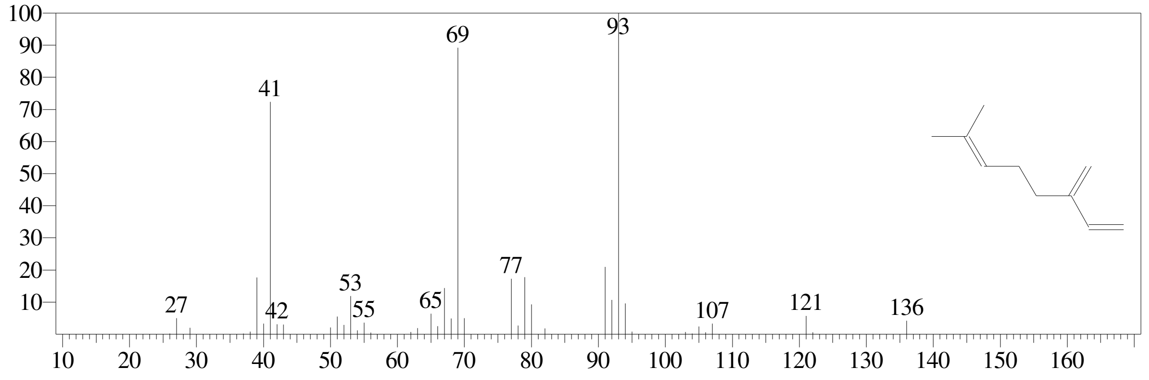

<< Target >>
Line#:7   R.Time:26.133(Scan#:2837) MassPeaks:7
RawMode:Averaged 26.125-26.142(2836-2838) BasePeak:41.00(1868)
BG Mode:None Group 1 - Event 1 Scan

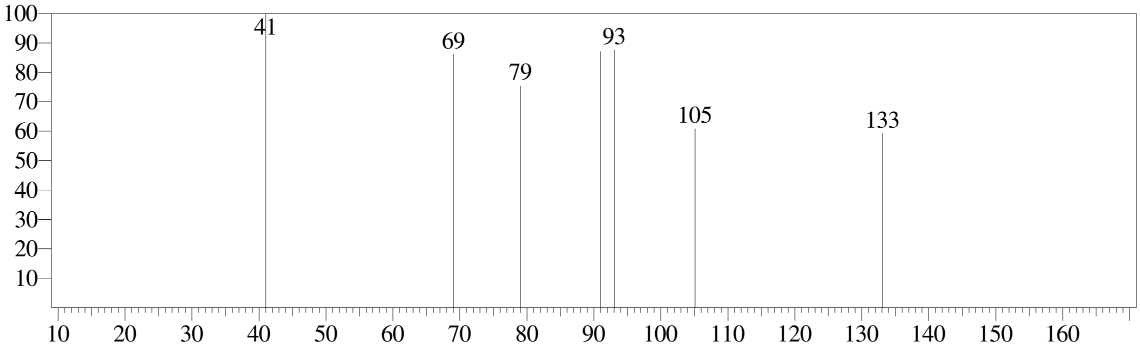

Hit#:5 Entry:25391 Library:NIST23-1.lib
SI:69 Formula:C10H11NO CAS:6580-95-6 MolWeight:161 RetIndex:1471
CompName:Ethanone, 1-cyclopropyl-2-(4-pyridinyl)- $$ Ketone, cyclopropyl 4-pyridylmethyl $$ Cyclopropyl 4-picolyl k

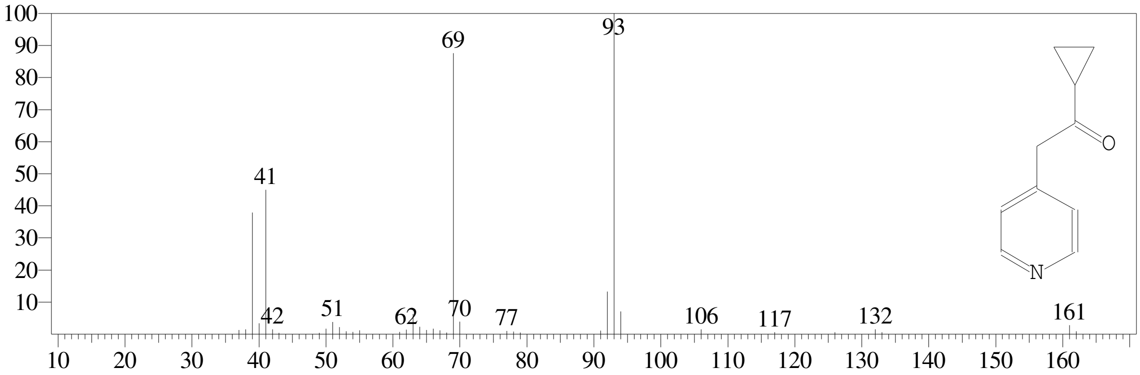

<< Target >>
Line#:8 R.Time:26.942(Scan#:2934) MassPeaks:36
RawMode:Averaged 26.933-26.950(2933-2935) BasePeak:41.00(8611)
BG Mode:None Group 1 - Event 1 Scan

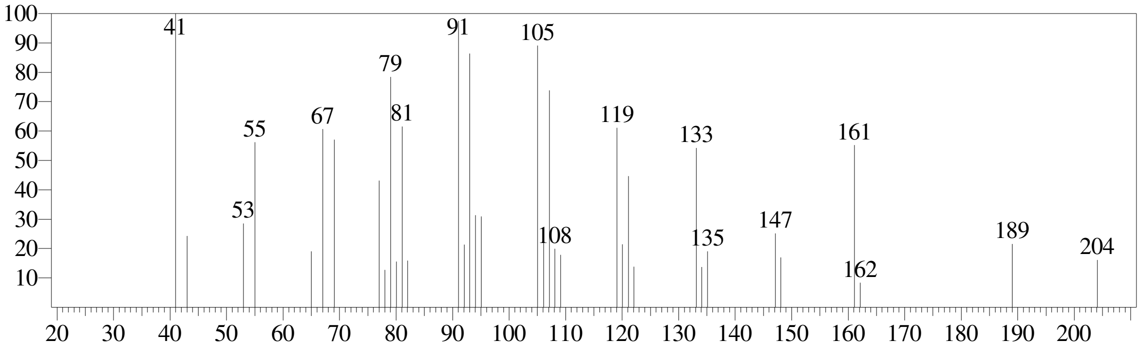

Hit#:1 Entry:62764 Library:NIST23-1.lib
SI:93 Formula:C15H24 CAS:489-39-4 MolWeight:204 RetIndex:1424
CompName:Aromandendrene $$ 1H-Cycloprop[e]azulene, decahydro-1,1,7-trimethyl-4-methylene-, [1aR-(1a.alpha.,4a.al

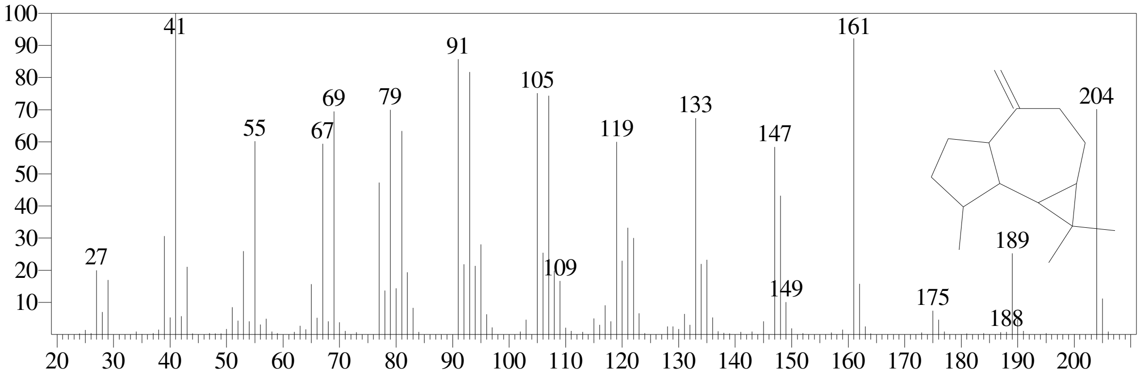

Hit#:2 Entry:62794 Library:NIST23-1.lib
SI:92 Formula:C15H24 CAS:68832-35-9 MolWeight:204 RetIndex:1450
CompName:(1R,9R,E)-4,11,11-Trimethyl-8-methylenebicyclo[7.2.0]undec-4-ene $$ Bicyclo[7.2.0]undec-4-ene, 4,11,11-t

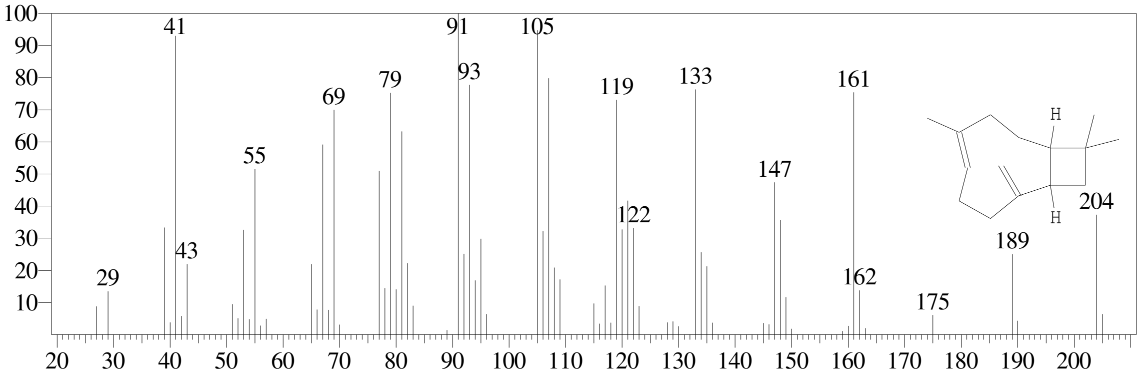

<< Target >>
Line#:8 R.Time:26.942(Scan#:2934) MassPeaks:36
RawMode:Averaged 26.933-26.950(2933-2935) BasePeak:41.00(8611)
BG Mode:None Group 1 - Event 1 Scan

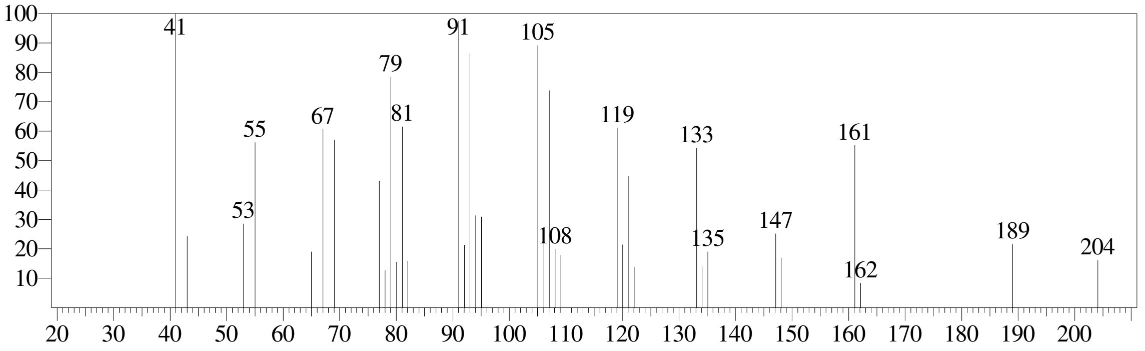

Hit#:3 Entry:24868 Library:NIST23s.lib
SI:92 Formula:C15H24 CAS:25246-27-9 MolWeight:204 RetIndex:1424
CompName:Alloaromadendrene $$ 1H-Cycloprop[e]azulene, decahydro-1,1,7-trimethyl-4-methylene-, [1aR-(1a.alpha.,4a

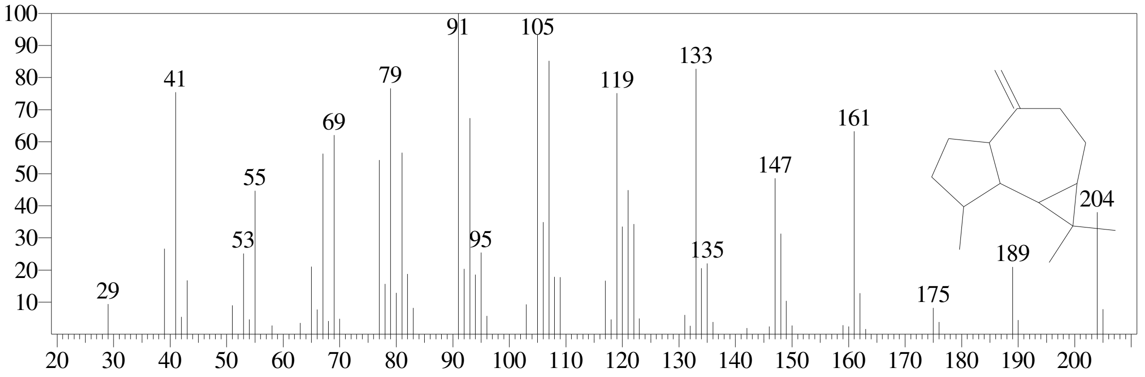

Hit#:4 Entry:24930 Library:NIST23s.lib
SI:92 Formula:C15H24 CAS:25246-27-9 MolWeight:204 RetIndex:1424
CompName:Alloaromadendrene $$ 1H-Cycloprop[e]azulene, decahydro-1,1,7-trimethyl-4-methylene-, [1aR-(1a.alpha.,4a

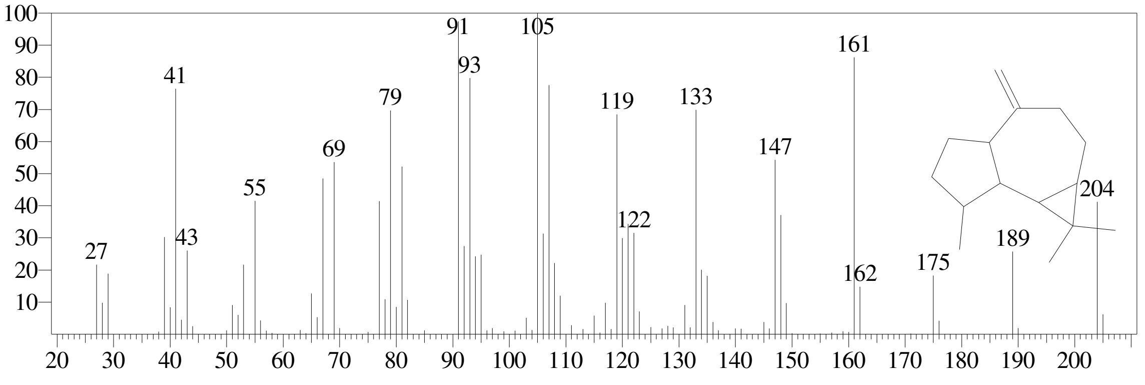

<< Target >>
Line#:8 R.Time:26.942(Scan#:2934) MassPeaks:36
RawMode:Averaged 26.933-26.950(2933-2935) BasePeak:41.00(8611)
BG Mode:None Group 1 - Event 1 Scan

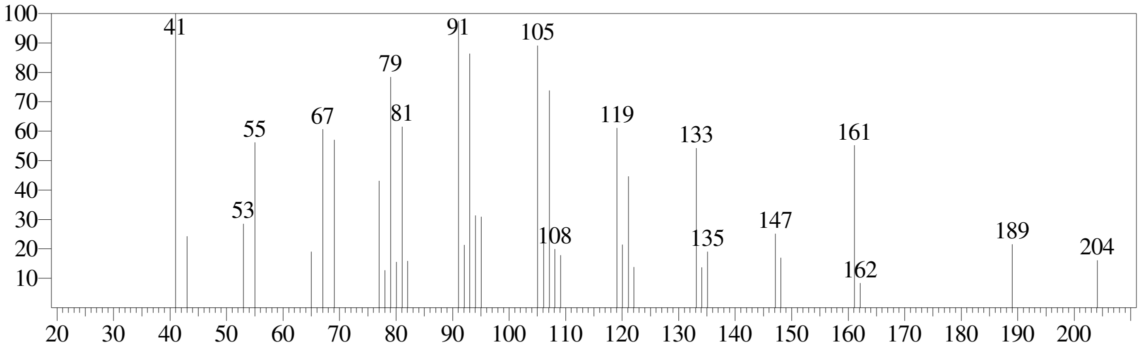

Hit#:5 Entry:24815 Library:NIST23s.lib
SI:92 Formula:C15H24 CAS:0-00-0 MolWeight:204 RetIndex:1459
CompName:1R,3Z,9S-4,11,11-Trimethyl-8-methylenebicyclo[7.2.0]undec-3-ene $$ 4,11,11-Trimethyl-8-methylenebicycl

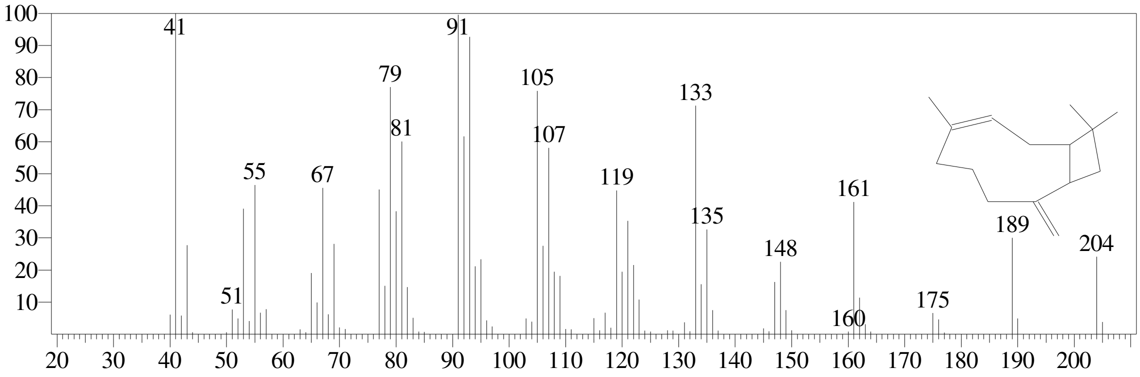

<< Target >>
Line#:9 R.Time:27.842(Scan#:3042) MassPeaks:10
RawMode:Averaged 27.833-27.850(3041-3043) BasePeak:93.05(1986)
BG Mode:None Group 1 - Event 1 Scan

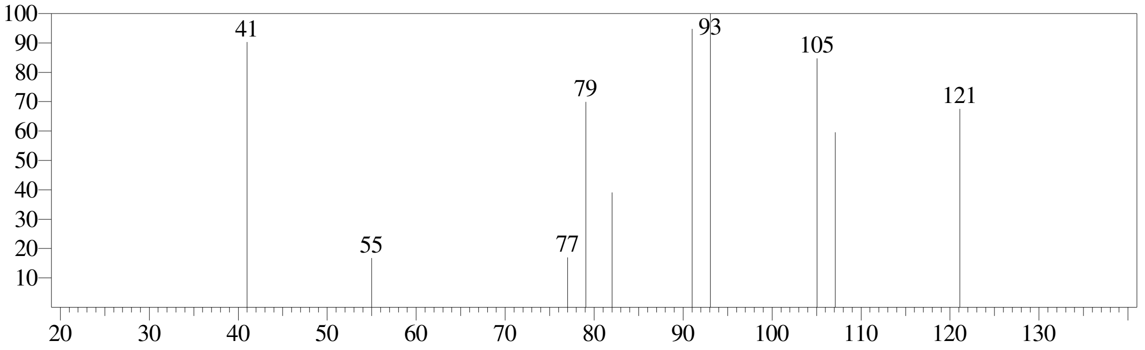

Hit#:1 Entry:11318 Library:NIST23-1.lib
SI:75 Formula:C10H16 CAS:74663-83-5 MolWeight:136 RetIndex:980
CompName:1,5-Heptadiene, 2,5-dimethyl-3-methylene- $$ (5E)-2,5-Dimethyl-3-methylene-1,5-heptadiene # $$

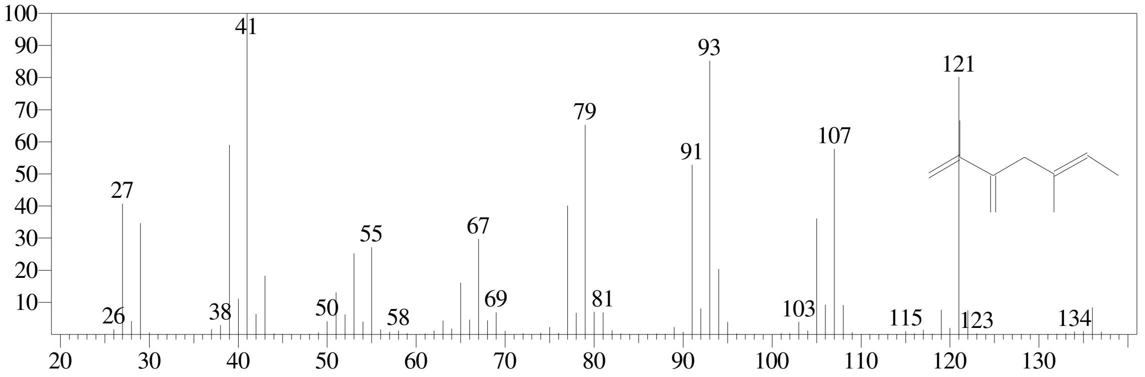

Hit#:2 Entry:8462 Library:NIST23s.lib
SI:74 Formula:C10H16 CAS:29548-02-5 MolWeight:136 RetIndex:923
CompName:1,3,6-Heptatriene, 2,5,5-trimethyl- $$ 2,5,5-Trimethyl-1,3,6-heptatriene $$ 3,3,6-Trimethyl-1,4,6-heptatriene

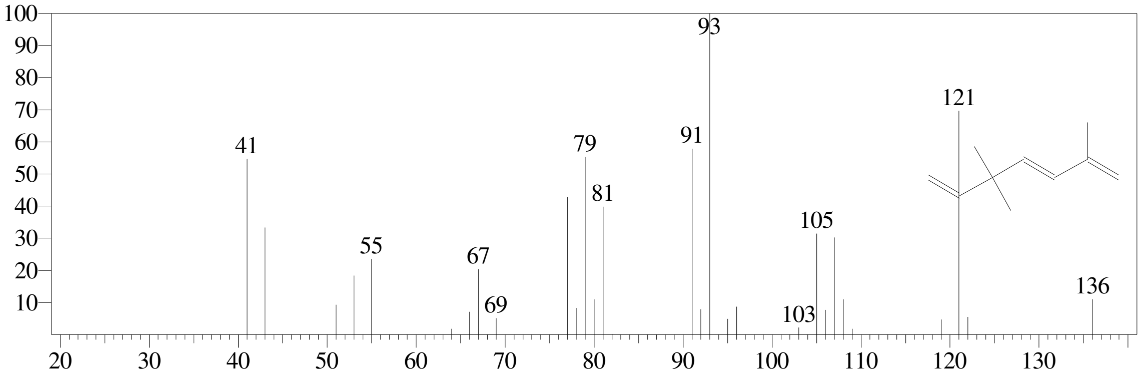

<< Target >>
Line#:9   R.Time:27.842(Scan#:3042) MassPeaks:10
RawMode:Averaged 27.833-27.850(3041-3043) BasePeak:93.05(1986)
BG Mode:None Group 1 - Event 1 Scan

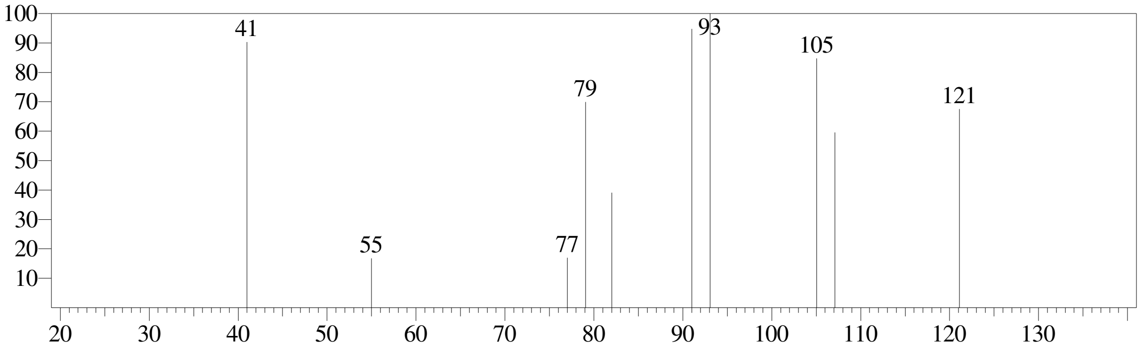

Hit#:3 Entry:8457 Library:NIST23s.lib
SI:72 Formula:C10H16 CAS:586-63-0 MolWeight:136 RetIndex:1083
CompName:Cyclohexene, 3-methyl-6-(1-methylethylidene)- $$ p-Mentha-2,4(8)-diene $$ Isoterpinolene $$ 3-Methyl-6-(

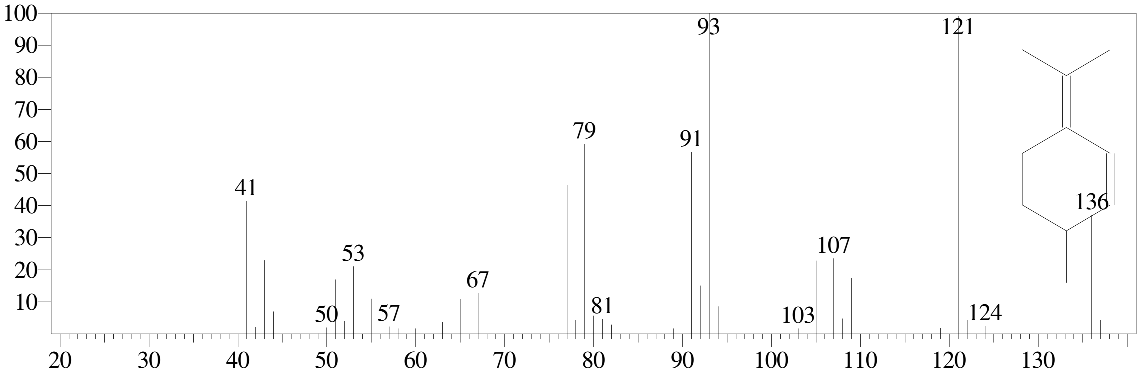

Hit#:4 Entry:11420 Library:NIST23-1.lib
SI:72 Formula:C10H16 CAS:42123-66-0 MolWeight:136 RetIndex:963
CompName:1,3,6-Heptatriene, 2,5,6-trimethyl- $$ 2,5,6-Trimethyl-1,3,6-heptatriene $$ 2,5,6-Trimethyl-hepta-1,3,6-triene

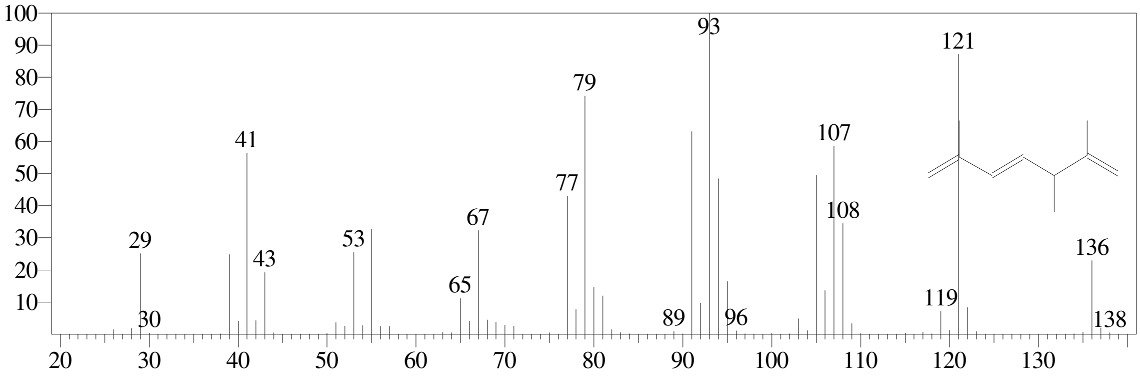

<< Target >>
Line#:9 R.Time:27.842(Scan#:3042) MassPeaks:10
RawMode:Averaged 27.833-27.850(3041-3043) BasePeak:93.05(1986)
BG Mode:None Group 1 - Event 1 Scan

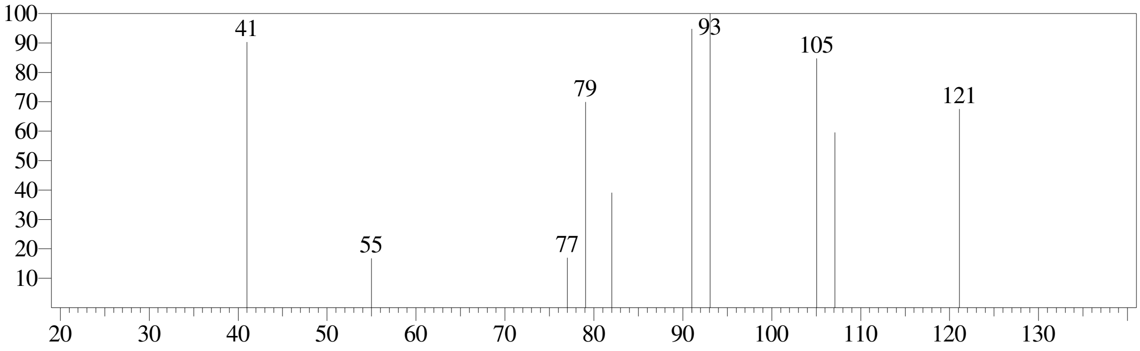

Hit#:5 Entry:6359 Library:NIST23-1.lib
SI:71 Formula:C9H14 CAS:59131-13-4 MolWeight:122 RetIndex:865
CompName:1,5-Hexadiene, 2,5-dimethyl-3-methylene- $$ 2,5-Dimethyl-3-methylene-1,5-hexadiene # $$

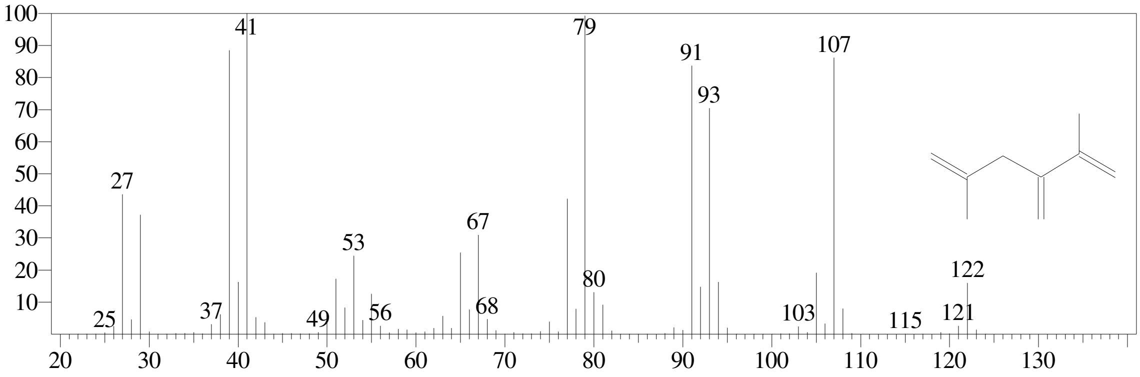

<< Target >>
Line#:10 R.Time:28.483(Scan#:3119) MassPeaks:11
RawMode:Averaged 28.475-28.492(3118-3120) BasePeak:105.05(3633)
BG Mode:None Group 1 - Event 1 Scan

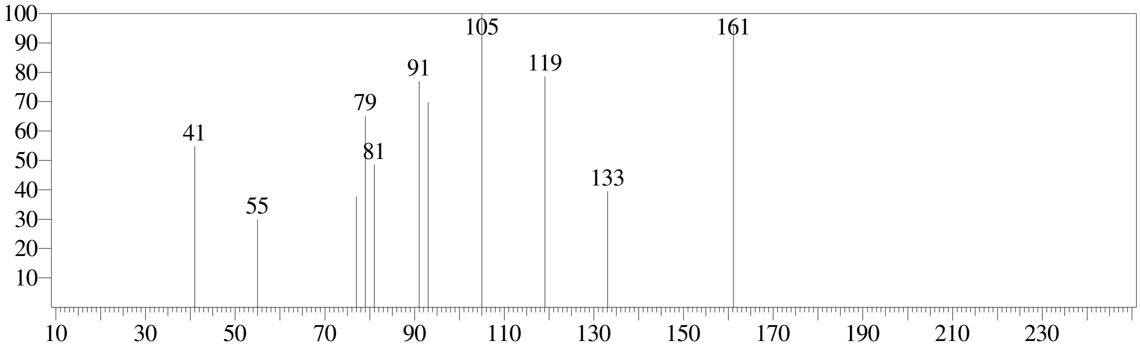

Hit#:1 Entry:25067 Library:NIST23s.lib
SI:75 Formula:C15H24 CAS:23986-74-5 MolWeight:204 RetIndex:1478
CompName:Germacrene D $$ (S,1Z,6Z)-8-Isopropyl-1-methyl-5-methylenecyclodeca-1,6-diene $$ D-Germacrene $$ 1(1

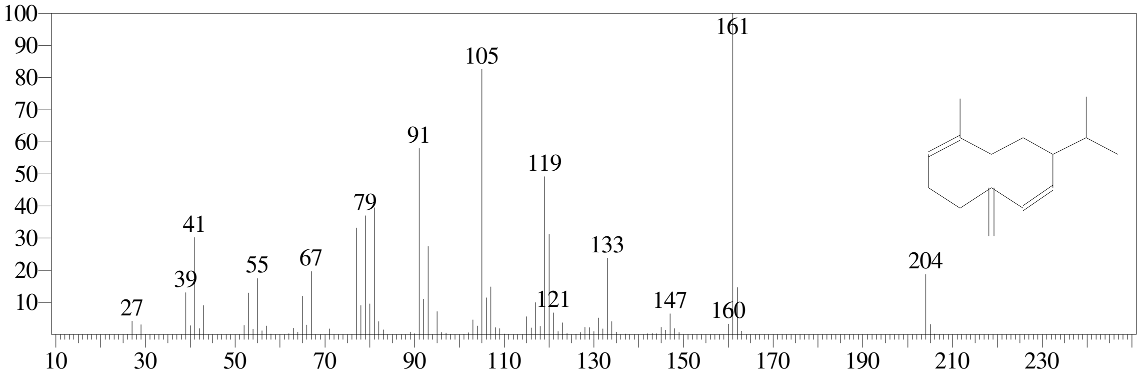

Hit#:2 Entry:62910 Library:NIST23-1.lib
SI:75 Formula:C15H24 CAS:3856-25-5 MolWeight:204 RetIndex:1407
CompName:Copaene $$ Tricyclo[4.4.0.02,7]dec-3-ene, 1,3-dimethyl-8-(1-methylethyl)-, stereoisomer $$ Tricyclo[4.4.0.0

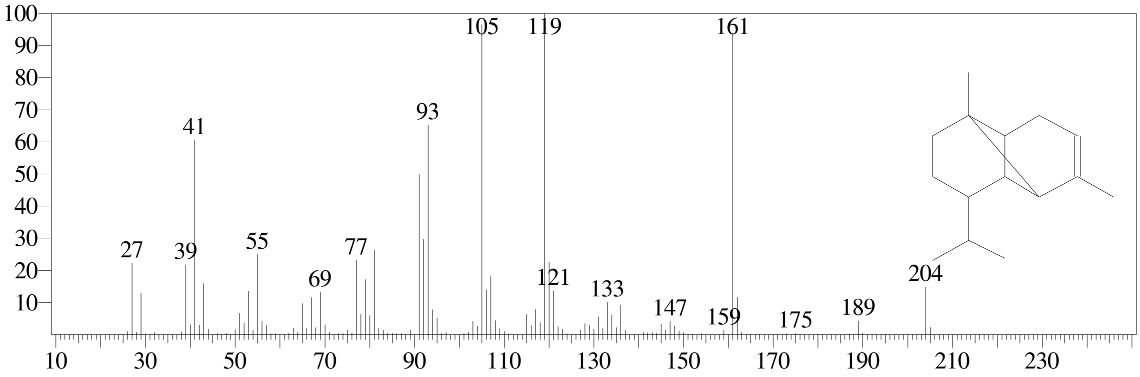

<< Target >>
Line#:10 R.Time:28.483(Scan#:3119) MassPeaks:11
RawMode:Averaged 28.475-28.492(3118-3120) BasePeak:105.05(3633)
BG Mode:None Group 1 - Event 1 Scan

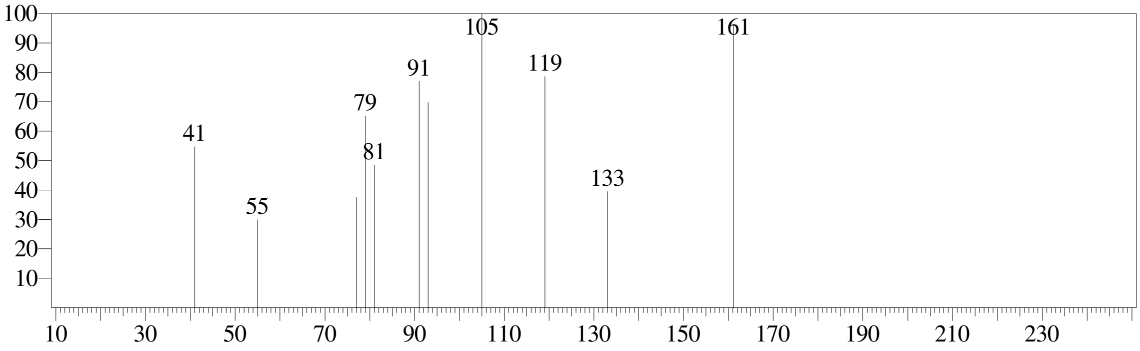

Hit#:3 Entry:31431 Library:NIST23s.lib
SI:75 Formula:C12H17Br CAS:57040-44-5 MolWeight:240 RetIndex:1581
CompName:Adamantane, 1-(2-bromoethenyl)- $$ 1-(2-Bromovinyl)-adamantane $$ 1-[(E)-2-Bromoethenyl]adamantane

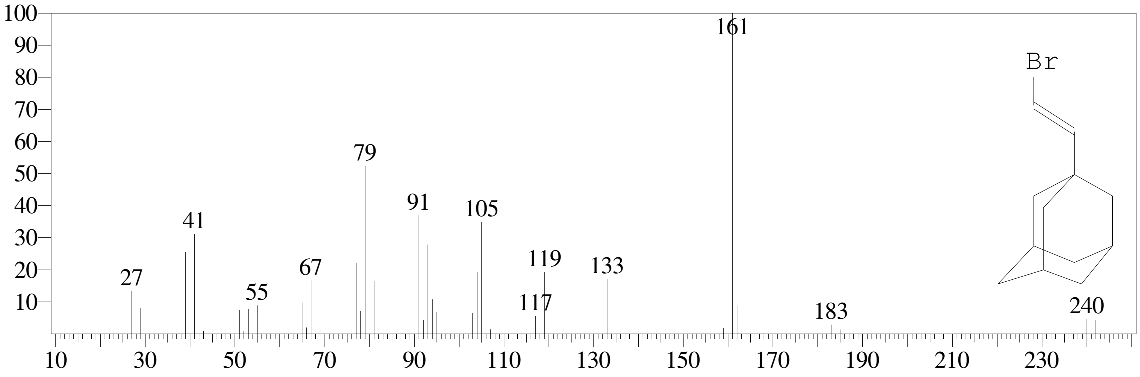

Hit#:4 Entry:62981 Library:NIST23-1.lib
SI:75 Formula:C15H24 CAS:23986-74-5 MolWeight:204 RetIndex:1478
CompName:Germacrene D $$ (S,1Z,6Z)-8-Isopropyl-1-methyl-5-methylenecyclodeca-1,6-diene $$ D-Germacrene $$ 1(1

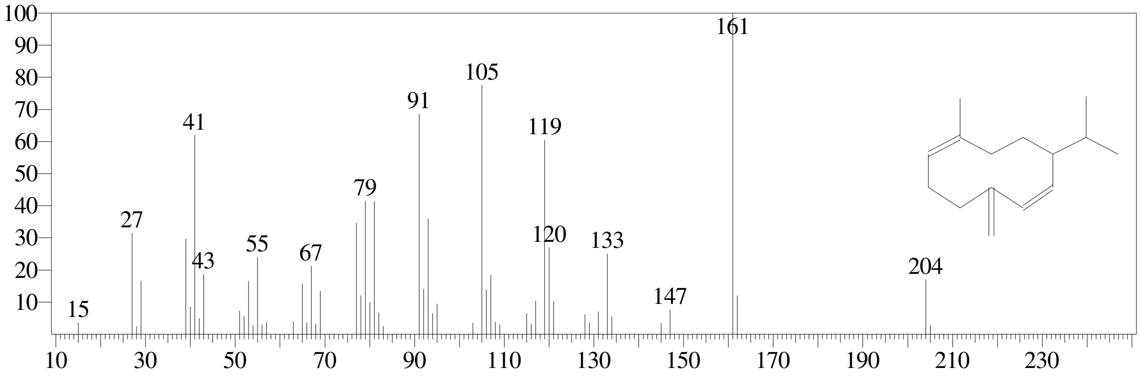

<< Target >>
Line#:10   R.Time:28.483(Scan#:3119) MassPeaks:11
RawMode:Averaged 28.475-28.492(3118-3120) BasePeak:105.05(3633)
BG Mode:None Group 1 - Event 1 Scan

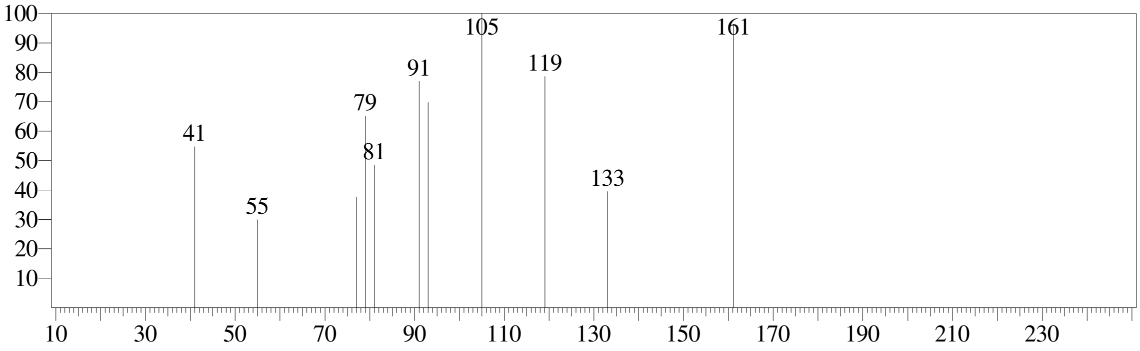

Hit#:5 Entry:25084 Library:NIST23s.lib
SI:75 Formula:C15H24 CAS:157374-44-2 MolWeight:204 RetIndex:1449
CompName:cis-muurola-3,5-diene

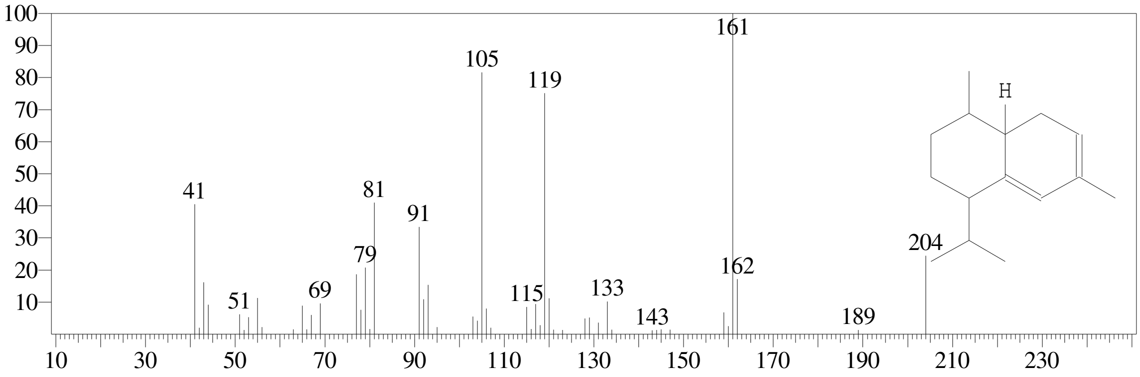

<< Target >>
Line#:11 R.Time:28.867(Scan#:3165) MassPeaks:33
RawMode:Averaged 28.858-28.875(3164-3166) BasePeak:93.05(5536)
BG Mode:None Group 1 - Event 1 Scan

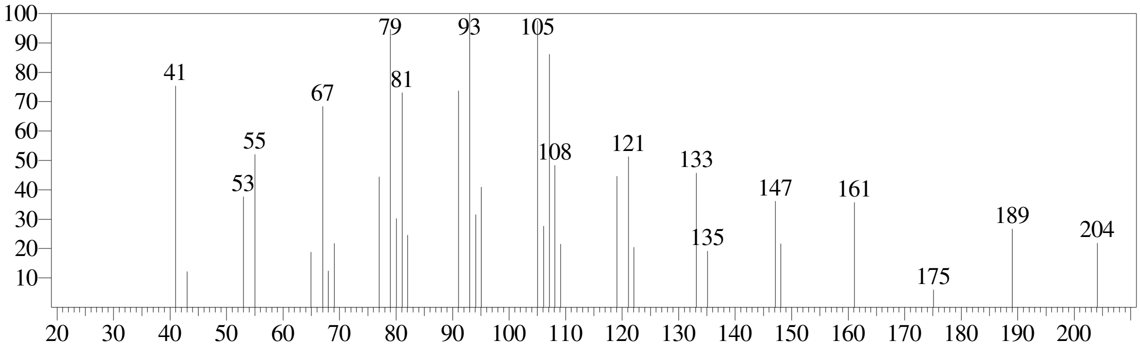

Hit#:1 Entry:24936 Library:NIST23s.lib
SI:91 Formula:C15H24 CAS:17066-67-0 MolWeight:204 RetIndex:1489
CompName:Naphthalene, decahydro-4a-methyl-1-methylene-7-(1-methylethenyl)-, [4aR-(4a.alpha.,7.alpha.,8a.beta.)]- $$

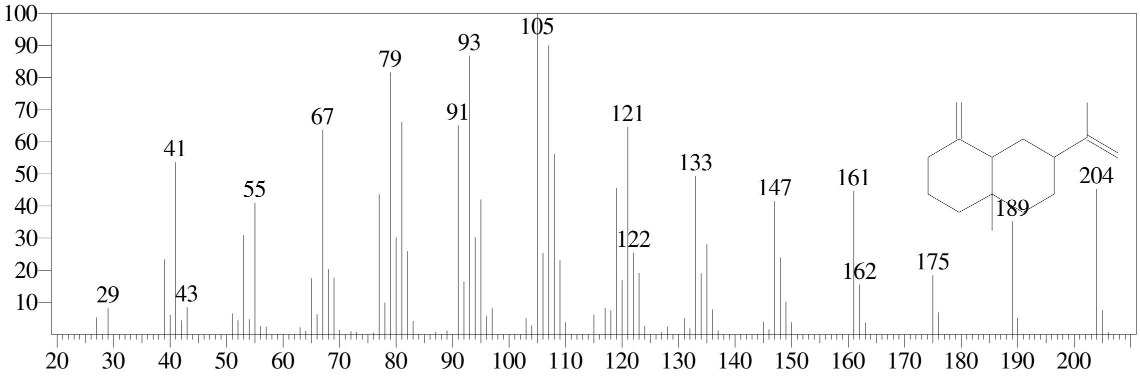

Hit#:2 Entry:24892 Library:NIST23s.lib
SI:91 Formula:C15H24 CAS:17066-67-0 MolWeight:204 RetIndex:1489
CompName:Naphthalene, decahydro-4a-methyl-1-methylene-7-(1-methylethenyl)-, [4aR-(4a.alpha.,7.alpha.,8a.beta.)]- $$

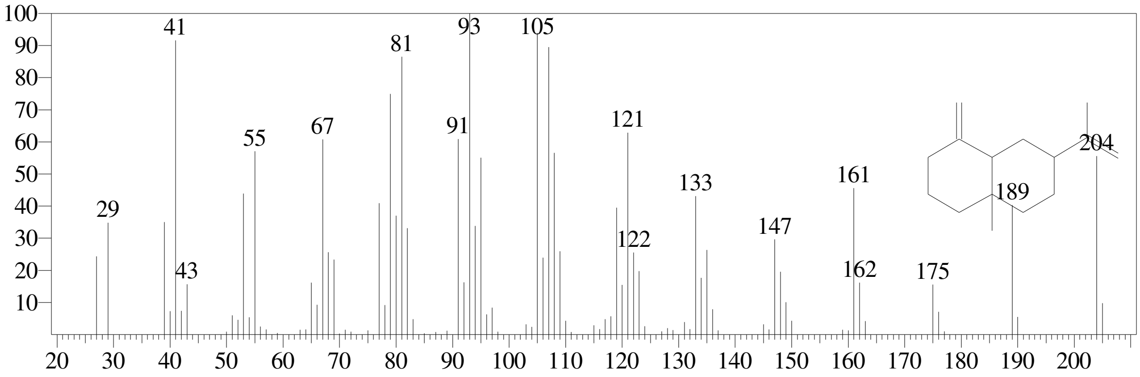

<< Target >>
Line#:11 R.Time:28.867(Scan#:3165) MassPeaks:33
RawMode:Averaged 28.858-28.875(3164-3166) BasePeak:93.05(5536)
BG Mode:None Group 1 - Event 1 Scan

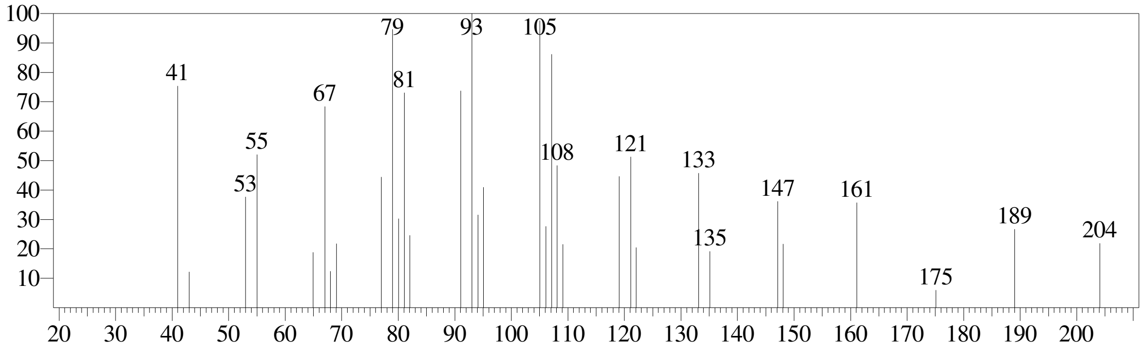

Hit#:3 Entry:62812 Library:NIST23-1.lib
SI:90 Formula:C15H24 CAS:0-00-0 MolWeight:204 RetIndex:1475
CompName:Bicyclo[5.3.0]decane, 2-methylene-5-(1-methylvinyl)-8-methyl- $$ 7-Isopropenyl-1-methyl-4-methylenedeca

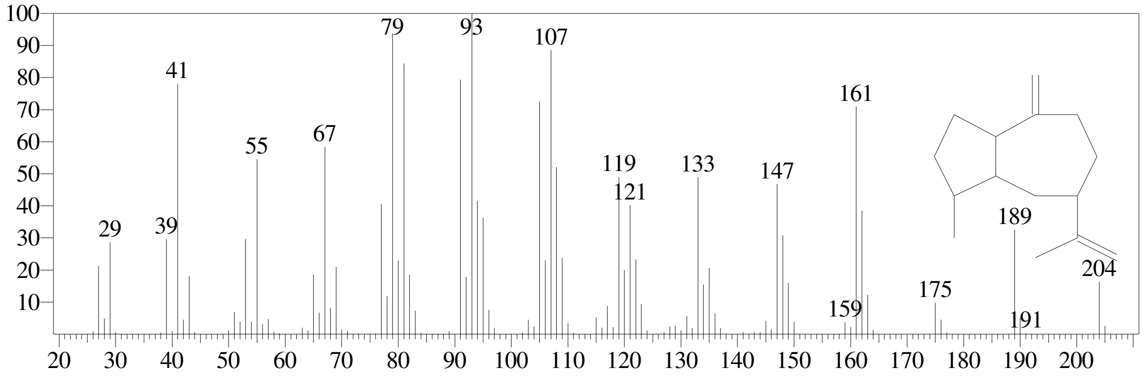

Hit#:4 Entry:24958 Library:NIST23s.lib
SI:89 Formula:C15H24 CAS:3691-11-0 MolWeight:204 RetIndex:1498
CompName:Azulene, 1,2,3,5,6,7,8,8a-octahydro-1,4-dimethyl-7-(1-methylethenyl)-, [1S-(1.alpha.,7.alpha.,8a.beta.)]- $$ (

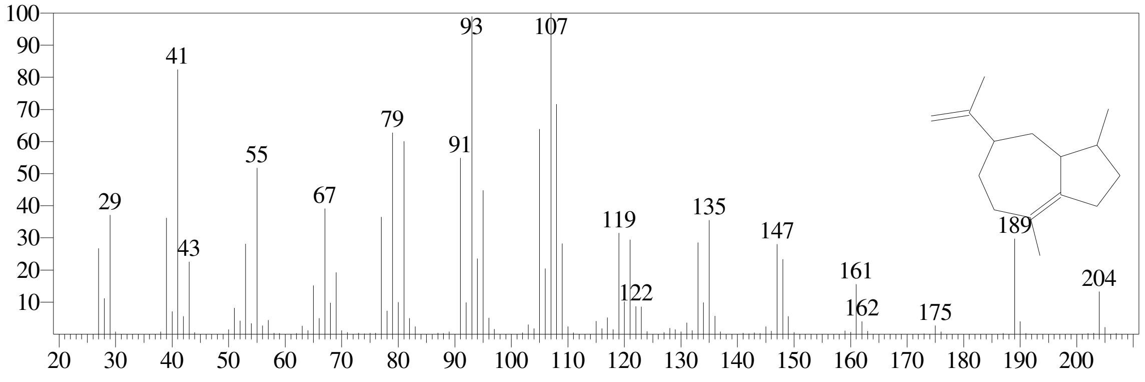

<< Target >>
Line#:11 R.Time:28.867(Scan#:3165) MassPeaks:33
RawMode:Averaged 28.858-28.875(3164-3166) BasePeak:93.05(5536)
BG Mode:None Group 1 - Event 1 Scan

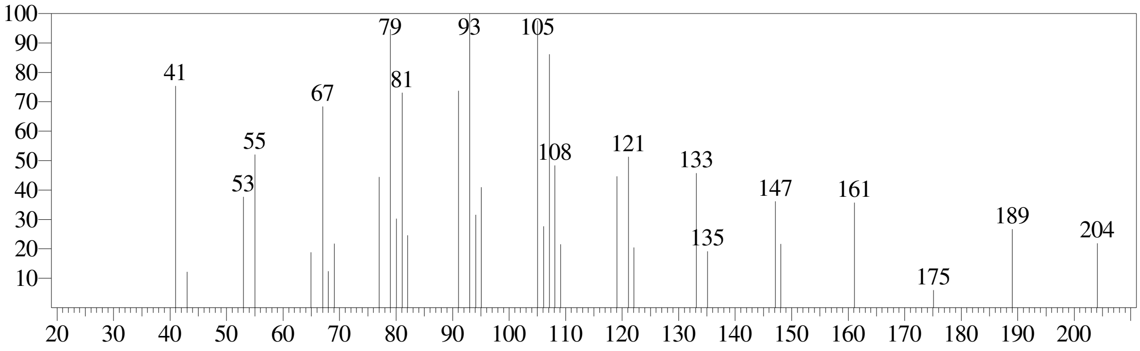

Hit#:5 Entry:62867 Library:NIST23-1.lib
SI:89 Formula:C15H24 CAS:0-00-0 MolWeight:204 RetIndex:1394
CompName:Cycloheptane, 4-methylene-1-methyl-2-(2-methyl-1-propen-1-yl)-1-vinyl- $$ 1-Methyl-4-methylene-2-(2-me

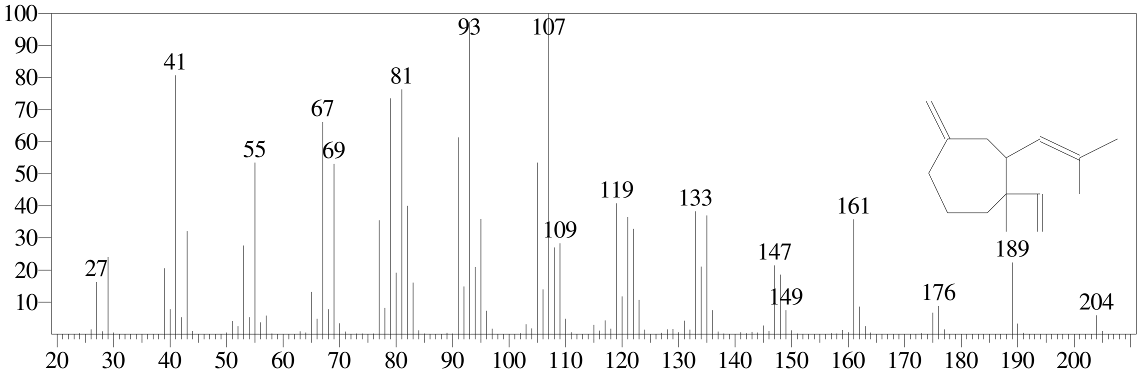

<< Target >>
Line#:12 R.Time:28.967(Scan#:3177) MassPeaks:6
RawMode:Averaged 28.958-28.975(3176-3178) BasePeak:105.05(1661)
BG Mode:None Group 1 - Event 1 Scan

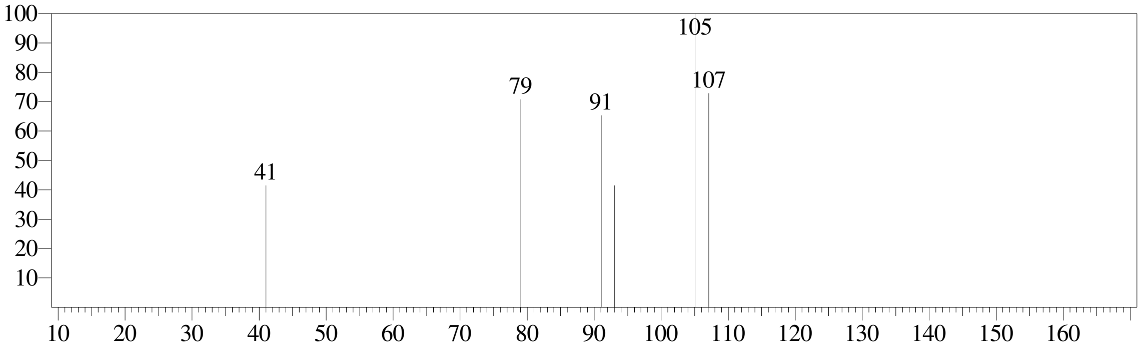

Hit#:1 Entry:2868 Library:NIST23s.lib
SI:76 Formula:CBrN CAS:506-68-3 MolWeight:105 RetIndex:585
CompName:Cyanogen bromide $$ Bromine cyanide $$ Bromocyan $$ Bromocyanide $$ Bromocyanogen $$ Campilit $$

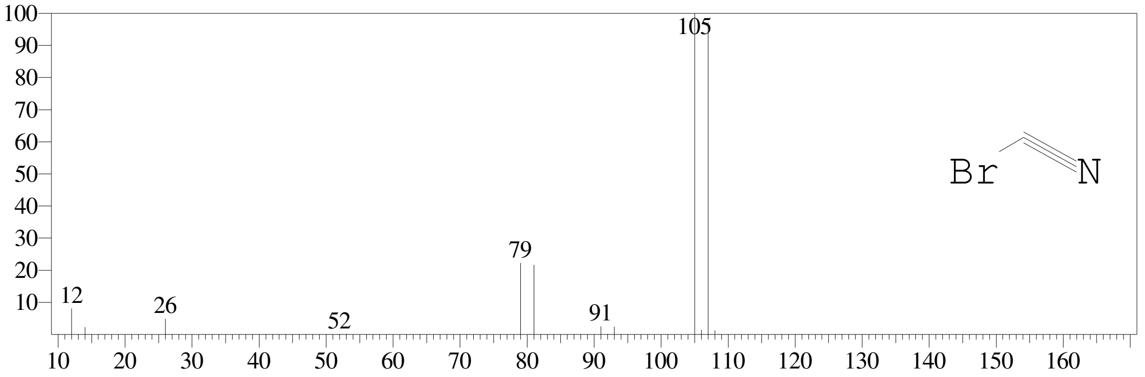

Hit#:2 Entry:18484 Library:NIST23-1.lib
SI:73 Formula:C11H18 CAS:85615-64-1 MolWeight:150 RetIndex:1239
CompName:(2Z,4Z,6E)-2,4,6-Undecatriene $$ (2E,4Z,6E)-2,4,6-Undecatriene # $$

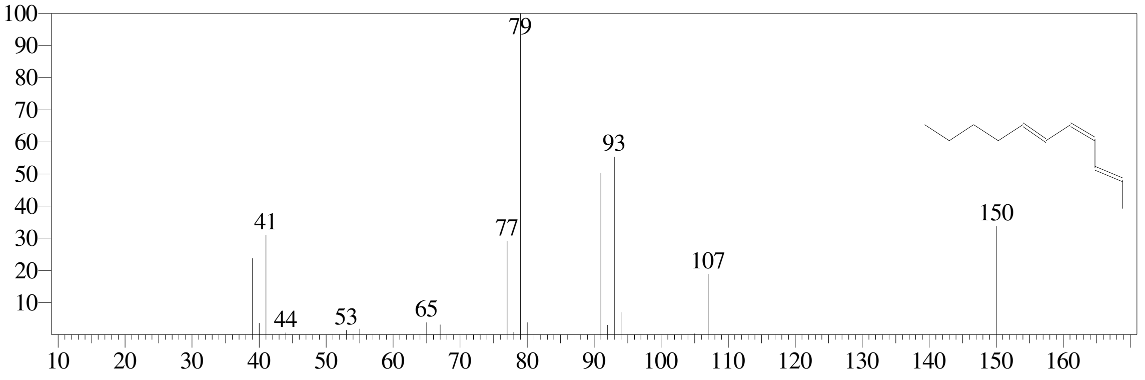

<< Target >>
Line#:12   R.Time:28.967(Scan#:3177) MassPeaks:6
RawMode:Averaged 28.958-28.975(3176-3178) BasePeak:105.05(1661)
BG Mode:None Group 1 - Event 1 Scan

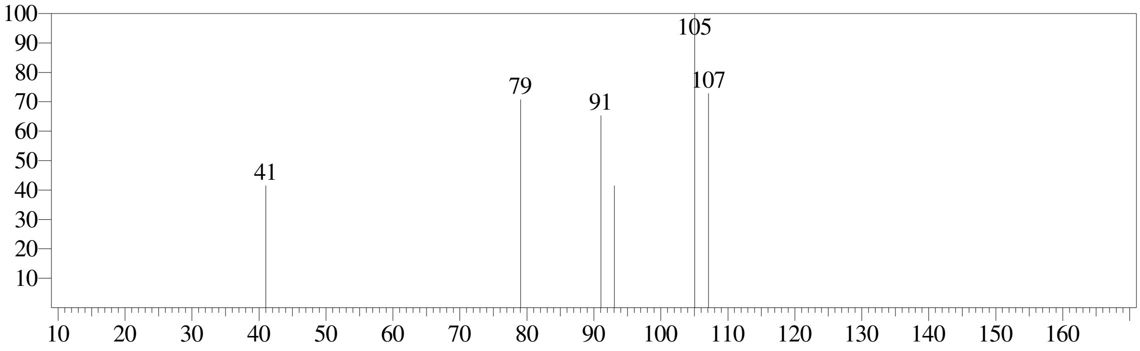

Hit#:3 Entry:18293 Library:NIST23-1.lib
SI:71 Formula:C10H14O CAS:66930-01-6 MolWeight:150 RetIndex:1207
CompName:Tricyclo[3.2.1.0(2,4)]octan-8-one, 3,3-dimethyl-, (1.alpha.,2.alpha.,4.alpha.,5.alpha.)-

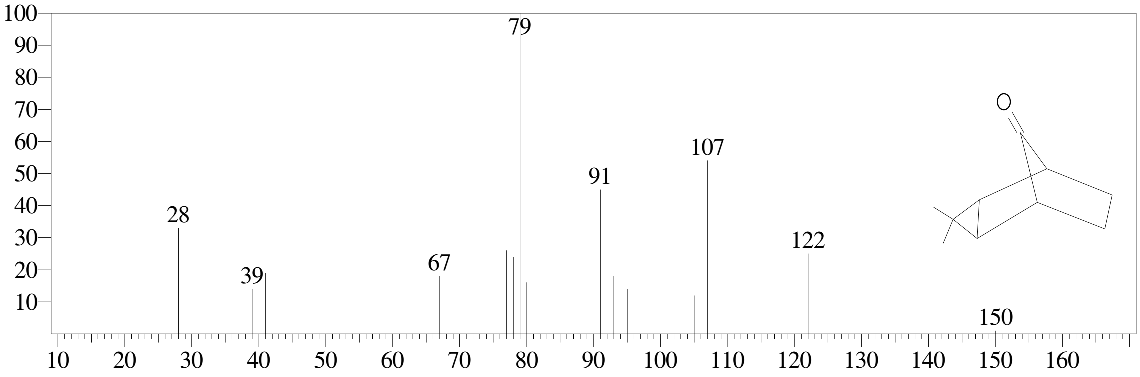

Hit#:4 Entry:27256 Library:NIST23-1.lib
SI:70 Formula:C10H12O2 CAS:2930-05-4 MolWeight:164 RetIndex:1375
CompName:Oxirane, [(phenylmethoxy)methyl]- $$ Propane, 1-(benzyloxy)-2,3-epoxy- $$ Benzyl glycidyl ether $$ 1-(Be

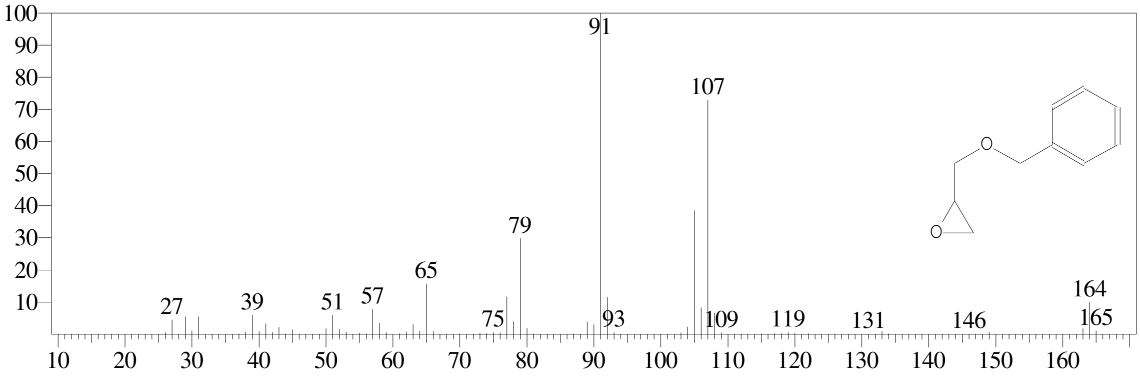

<< Target >>
Line#:12   R.Time:28.967(Scan#:3177) MassPeaks:6
RawMode:Averaged 28.958-28.975(3176-3178) BasePeak:105.05(1661)
BG Mode:None Group 1 - Event 1 Scan

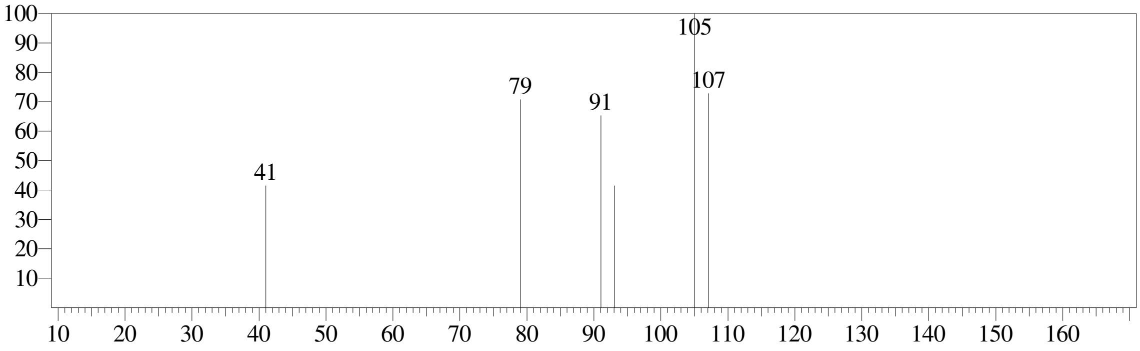

Hit#:5 Entry:5869 Library:NIST23-1.lib
SI:69 Formula:C4H9ClSi CAS:1719-58-0 MolWeight:120 RetIndex:652
CompName:Silane, chloroethenyldimethyl- $$ Dimethylvinylchlorosilane $$ Vinyldimethylchlorosilane $$ CV-4720 $$ (

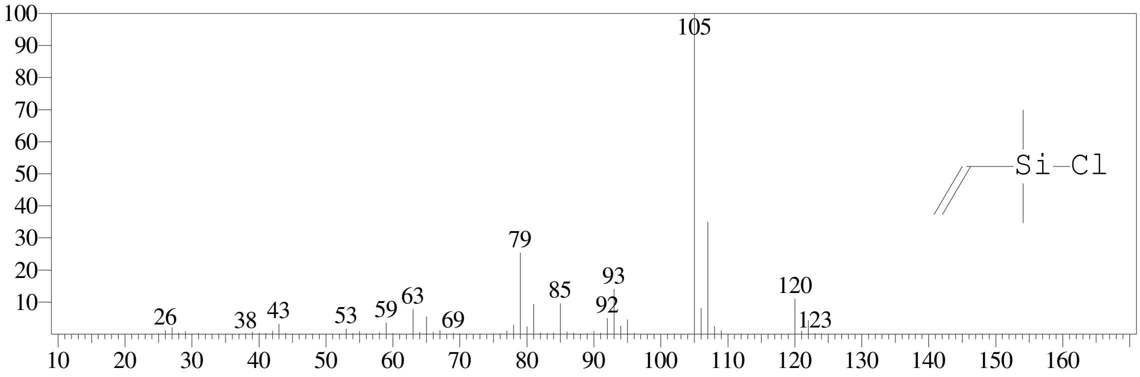

<< Target >>
Line#:13 R.Time:29.242(Scan#:3210) MassPeaks:46
RawMode:Averaged 29.233-29.250(3209-3211) BasePeak:105.05(15722)
BG Mode:Calc. from Peak Group 1 - Event 1 Scan

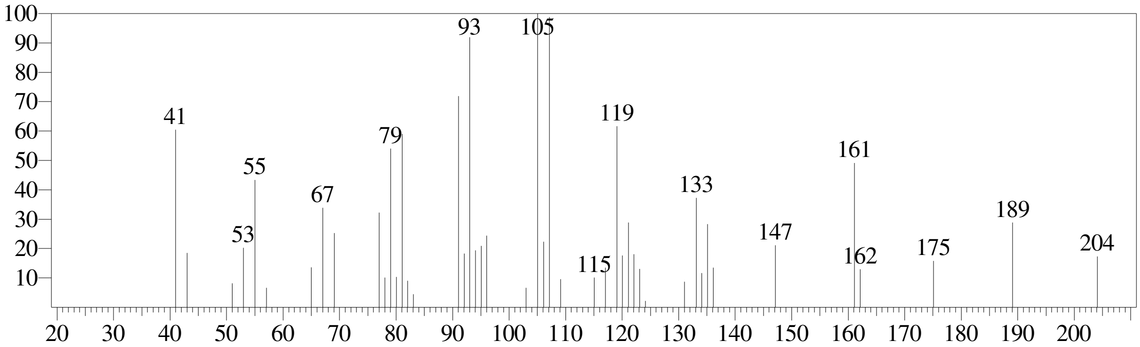

Hit#:1 Entry:24962 Library:NIST23s.lib
SI:93 Formula:C15H24 CAS:21747-46-6 MolWeight:204 RetIndex:1451
CompName:1H-Cycloprop[e]azulene, 1a,2,3,5,6,7,7a,7b-octahydro-1,1,4,7-tetramethyl-, [1aR-(1a.alpha.,7.alpha.,7a.beta.,

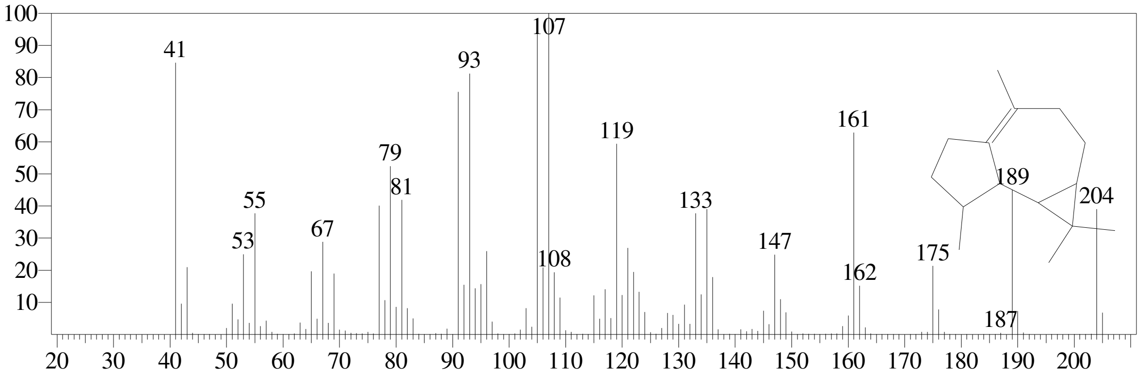

Hit#:2 Entry:24957 Library:NIST23s.lib
SI:91 Formula:C15H24 CAS:21747-46-6 MolWeight:204 RetIndex:1451
CompName:1H-Cycloprop[e]azulene, 1a,2,3,5,6,7,7a,7b-octahydro-1,1,4,7-tetramethyl-, [1aR-(1a.alpha.,7.alpha.,7a.beta.,

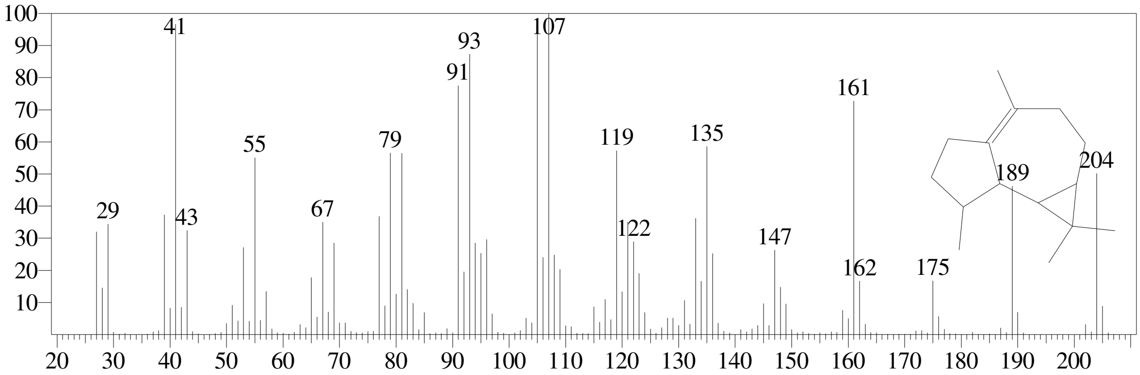

<< Target >>
Line#:13 R.Time:29.242(Scan#:3210) MassPeaks:46
RawMode:Averaged 29.233-29.250(3209-3211) BasePeak:105.05(15722)
BG Mode:Calc. from Peak Group 1 - Event 1 Scan

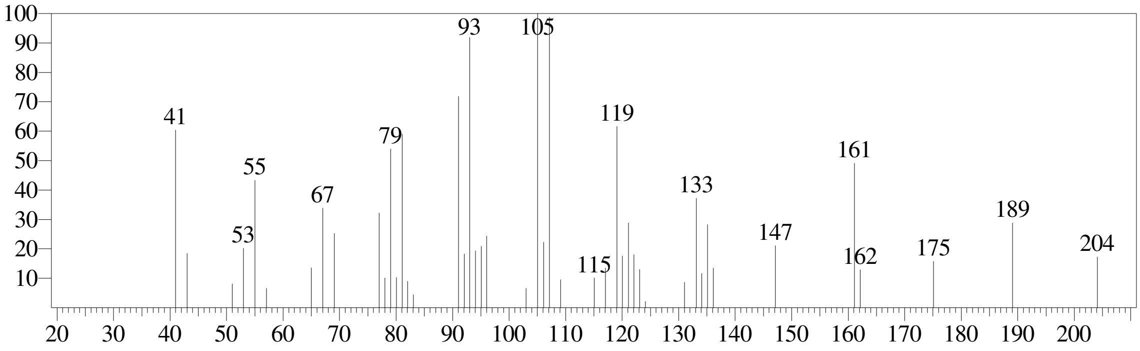

Hit#:3 Entry:24964 Library:NIST23s.lib
SI:91 Formula:C15H24 CAS:21747-46-6 MolWeight:204 RetIndex:1451
CompName:1H-Cycloprop[e]azulene, 1a,2,3,5,6,7,7a,7b-octahydro-1,1,4,7-tetramethyl-, [1aR-(1a.alpha.,7.alpha.,7a.beta.,

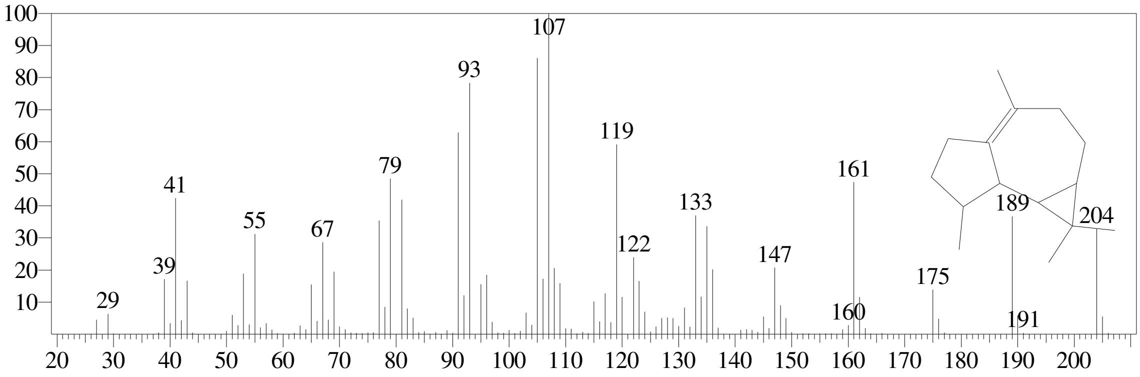

Hit#:4 Entry:24894 Library:NIST23s.lib
SI:91 Formula:C15H24 CAS:3691-11-0 MolWeight:204 RetIndex:1498
CompName:Azulene, 1,2,3,5,6,7,8,8a-octahydro-1,4-dimethyl-7-(1-methylethenyl)-, [1S-(1.alpha.,7.alpha.,8a.beta.)]- $$ (

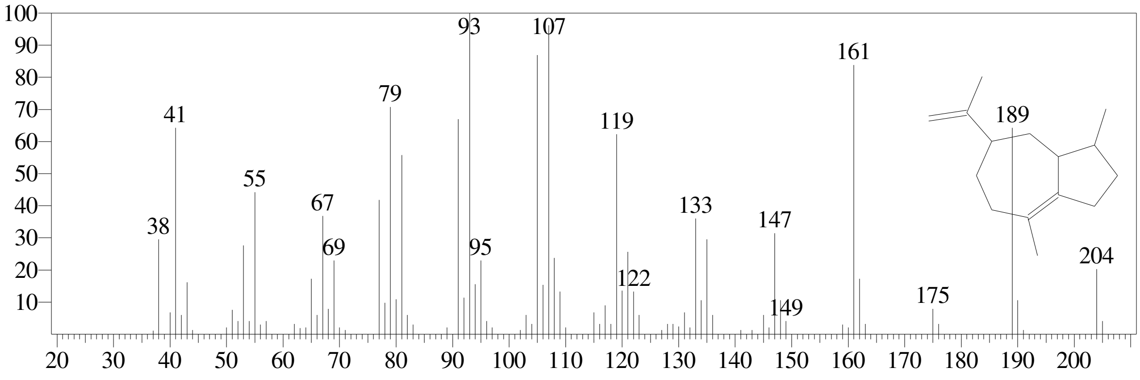

<< Target >>
Line#:13 R.Time:29.242(Scan#:3210) MassPeaks:46
RawMode:Averaged 29.233-29.250(3209-3211) BasePeak:105.05(15722)
BG Mode:Calc. from Peak Group 1 - Event 1 Scan

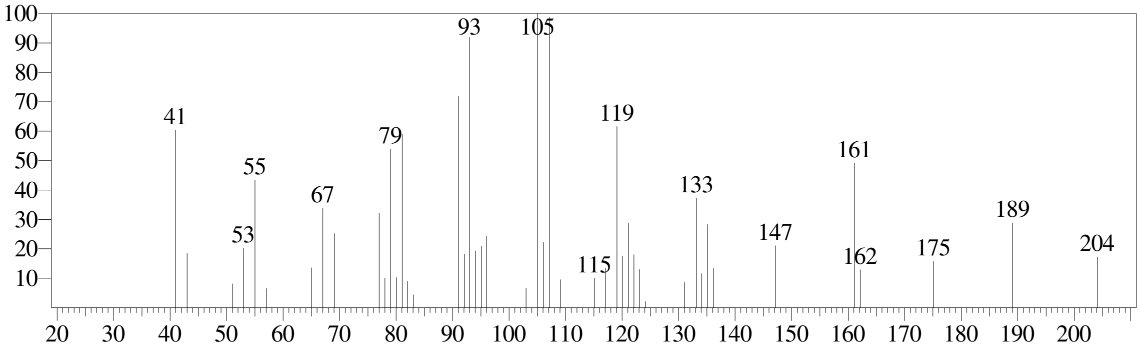

Hit#:5 Entry:62871 Library:NIST23-1.lib
SI:91 Formula:C15H24 CAS:21747-46-6 MolWeight:204 RetIndex:1451
CompName:1H-Cycloprop[e]azulene, 1a,2,3,5,6,7,7a,7b-octahydro-1,1,4,7-tetramethyl-, [1aR-(1a.alpha.,7.alpha.,7a.beta.,

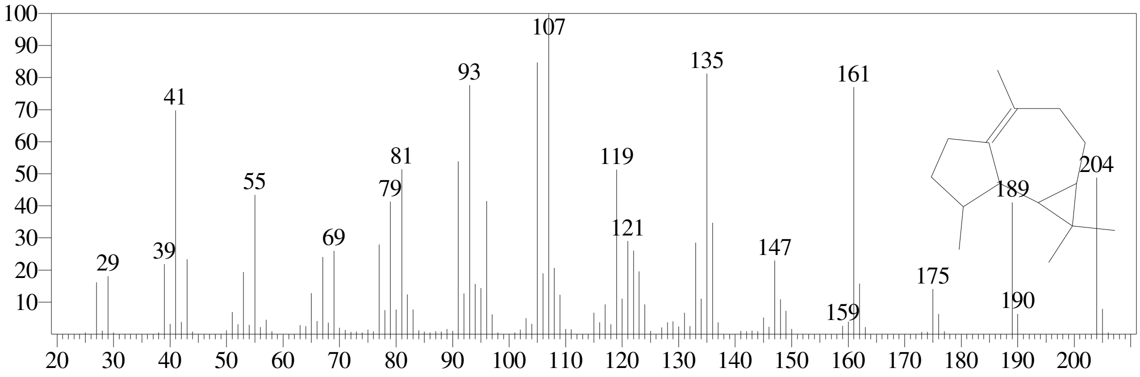

<< Target >>
Line#:14 R.Time:29.325(Scan#:3220) MassPeaks:26
RawMode:Averaged 29.317-29.333(3219-3221) BasePeak:108.05(8235)
BG Mode:Calc. from Peak Group 1 - Event 1 Scan

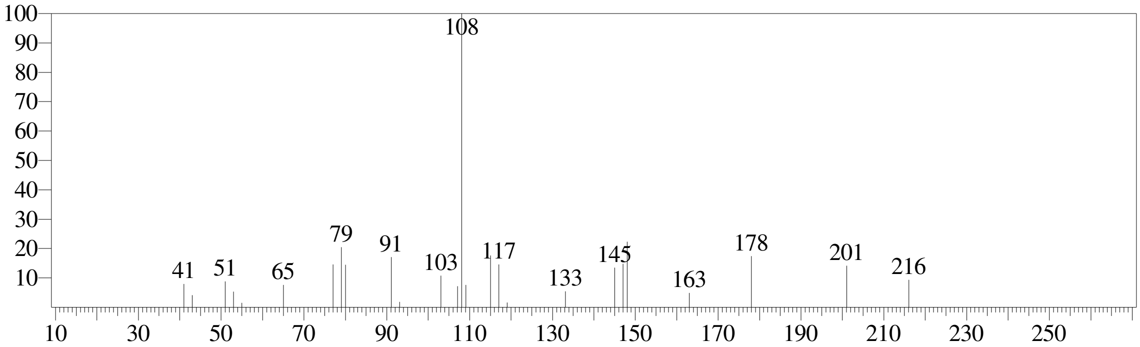

Hit#:1 Entry:27471 Library:NIST23s.lib
SI:78 Formula:C15H20O CAS:17910-09-7 MolWeight:216 RetIndex:1524
CompName:Benzofuran, 6-ethenyl-4,5,6,7-tetrahydro-3,6-dimethyl-5-isopropenyl-, trans- $$ 5-Isopropenyl-3,6-dimethyl-

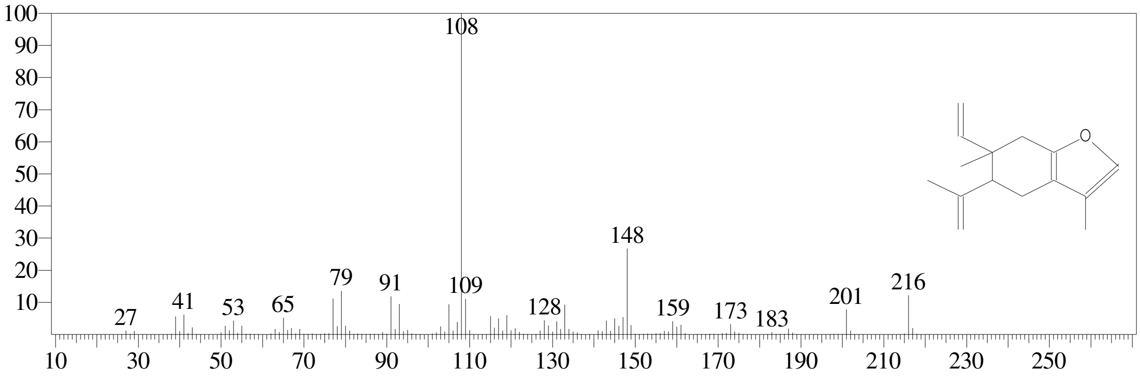

Hit#:2 Entry:76133 Library:NIST23-1.lib
SI:77 Formula:C15H20O CAS:17910-09-7 MolWeight:216 RetIndex:1524
CompName:Benzofuran, 6-ethenyl-4,5,6,7-tetrahydro-3,6-dimethyl-5-isopropenyl-, trans- $$ 5-Isopropenyl-3,6-dimethyl-

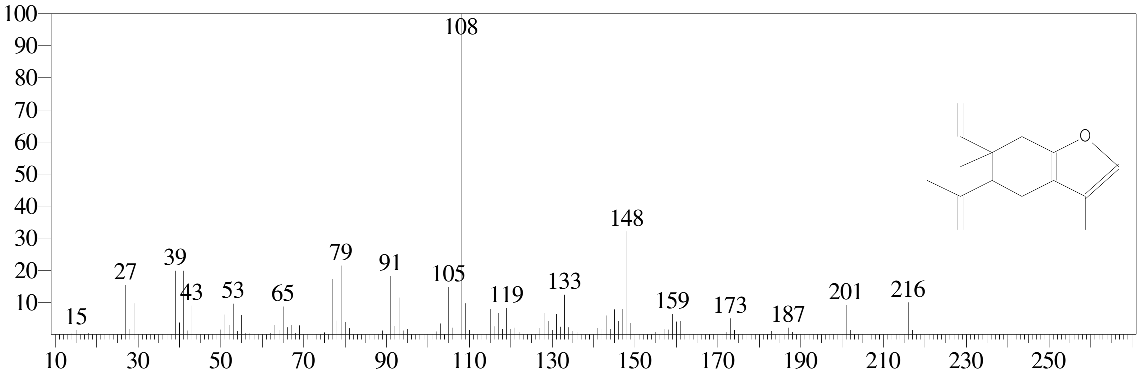

<< Target >>
Line#:14 R.Time:29.325(Scan#:3220) MassPeaks:26
RawMode:Averaged 29.317-29.333(3219-3221) BasePeak:108.05(8235)
BG Mode:Calc. from Peak Group 1 - Event 1 Scan

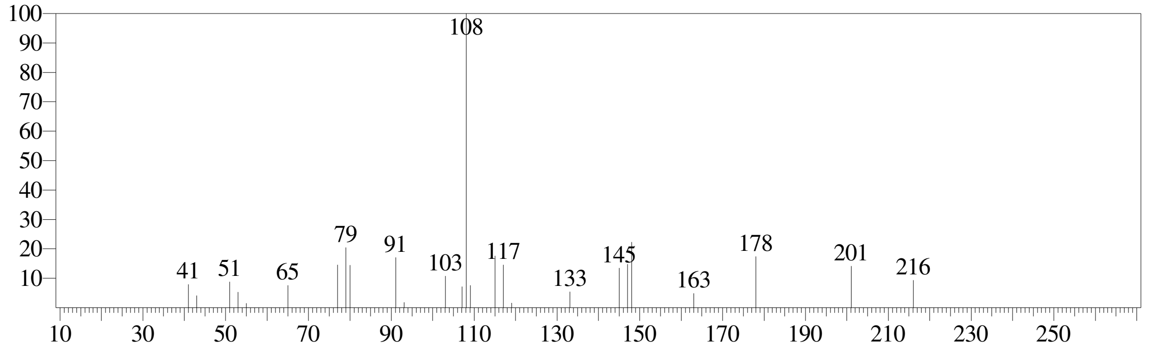

Hit#:3 Entry:27472 Library:NIST23s.lib
SI:76 Formula:C15H20O CAS:17910-09-7 MolWeight:216 RetIndex:1524
CompName:Benzofuran, 6-ethenyl-4,5,6,7-tetrahydro-3,6-dimethyl-5-isopropenyl-, trans- $$ 5-Isopropenyl-3,6-dimethyl-

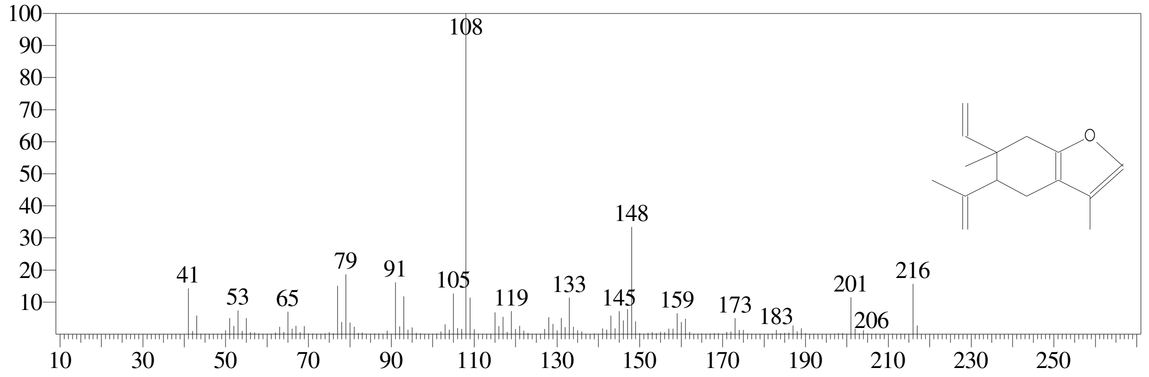

Hit#:4 Entry:76134 Library:NIST23-1.lib
SI:70 Formula:C15H20O CAS:6989-21-5 MolWeight:216 RetIndex:1653
CompName:(4aS,8aR)-3,8a-Dimethyl-5-methylene-4,4a,5,6,7,8,8a,9-octahydronaphtho[2,3-b]furan $$ Naphtho[2,3-b]fur

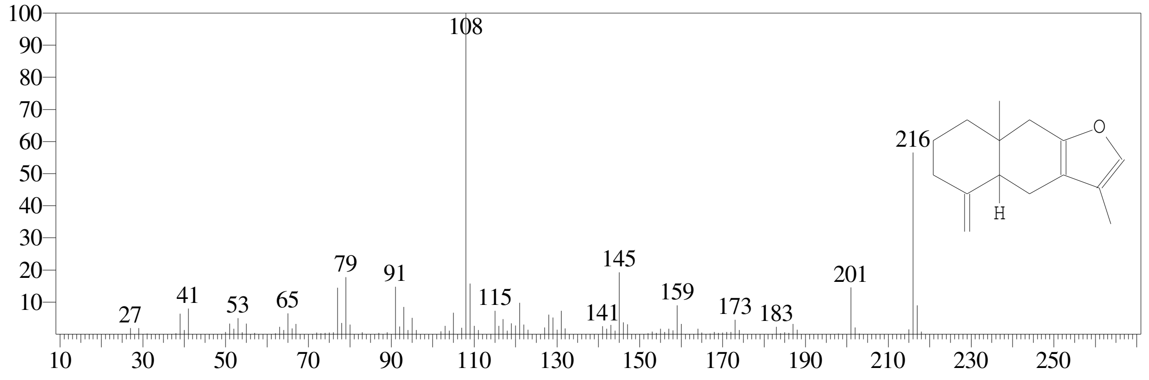

<< Target >>
Line#:14   R.Time:29.325(Scan#:3220) MassPeaks:26
RawMode:Averaged 29.317-29.333(3219-3221) BasePeak:108.05(8235)
BG Mode:Calc. from Peak Group 1 - Event 1 Scan

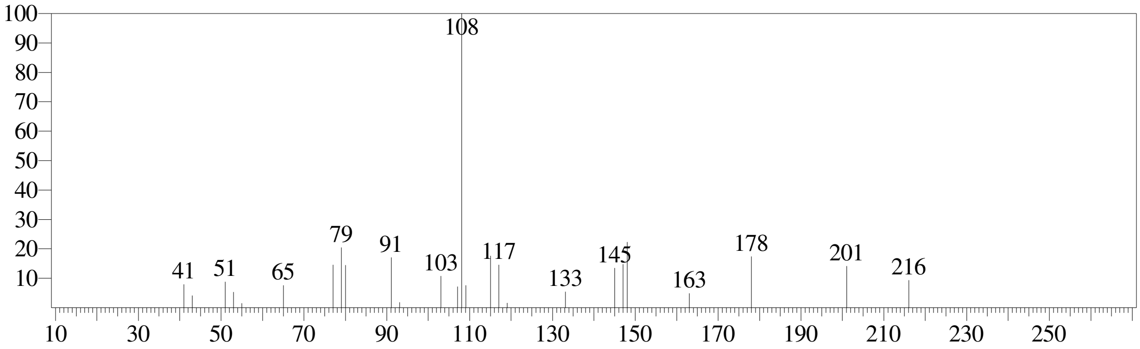

Hit#:5 Entry:130363 Library:NIST23-1.lib
SI:70 Formula:C16H20O3 CAS:19912-86-8 MolWeight:260 RetIndex:1794
CompName:5-Benzofuranacetic acid, 6-ethenyl-4,5,6,7-tetrahydro-3,6-dimethyl-.alpha.-methylene-, methyl ester $$ p-Me

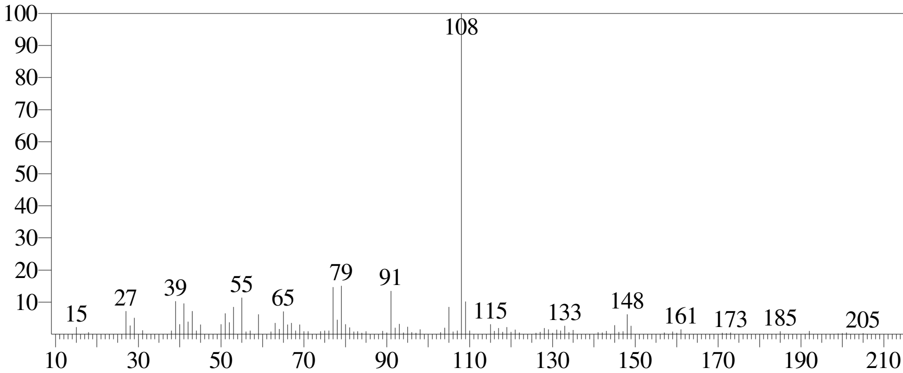

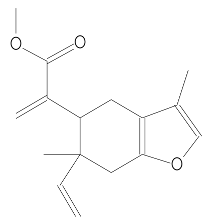

<< Target >>
Line#:15 R.Time:29.725(Scan#:3268) MassPeaks:10
RawMode:Averaged 29.717-29.733(3267-3269) BasePeak:105.05(3058)
BG Mode:None Group 1 - Event 1 Scan

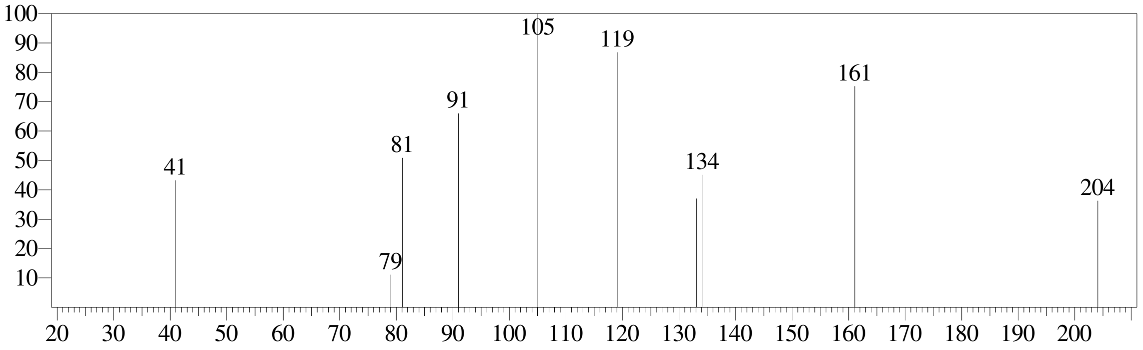

Hit#:1 Entry:25084 Library:NIST23s.lib
SI:73 Formula:C15H24 CAS:157374-44-2 MolWeight:204 RetIndex:1449
CompName:cis-muurola-3,5-diene

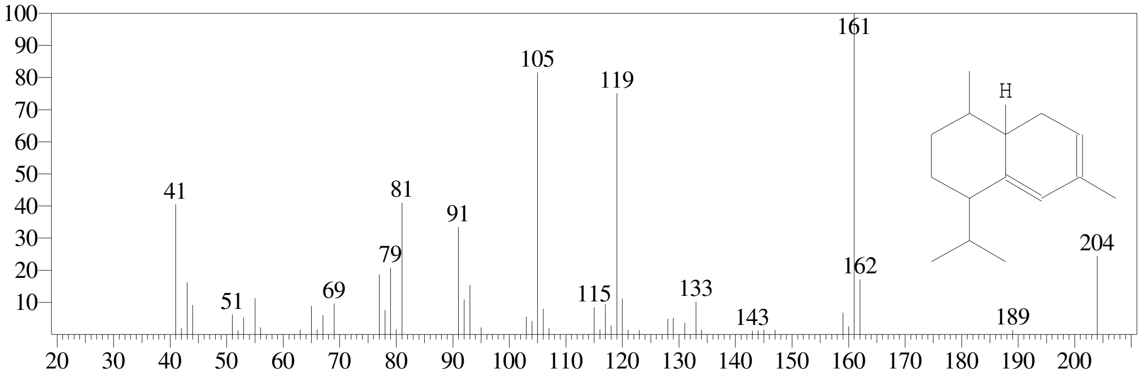

Hit#:2 Entry:62859 Library:NIST23-1.lib
SI:72 Formula:C15H24 CAS:95910-36-4 MolWeight:204 RetIndex:1396
CompName:isoledene $$ 1,1,4,7-Tetramethyl-1a,2,3,4,5,6,7,7b-octahydro-1H-cyclopropa[e]azulene # $$ (1aR,4R,7R,7b

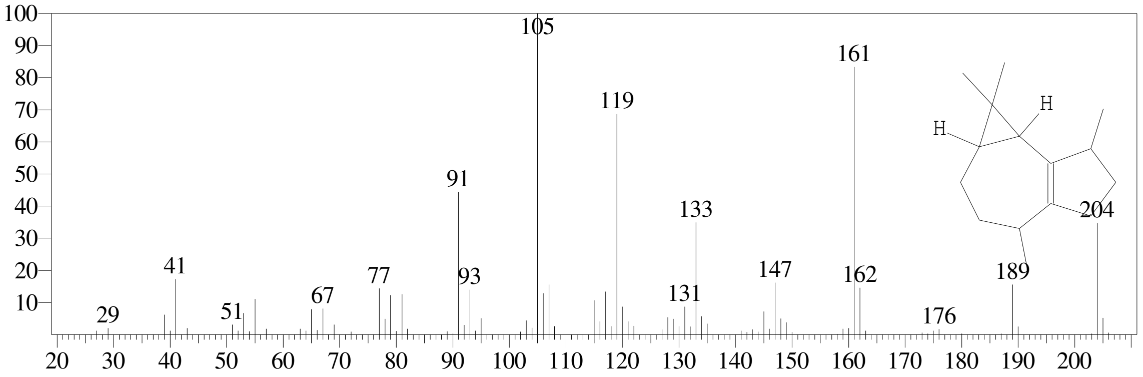

<< Target >>
Line#:15   R.Time:29.725(Scan#:3268) MassPeaks:10
RawMode:Averaged 29.717-29.733(3267-3269) BasePeak:105.05(3058)
BG Mode:None Group 1 - Event 1 Scan

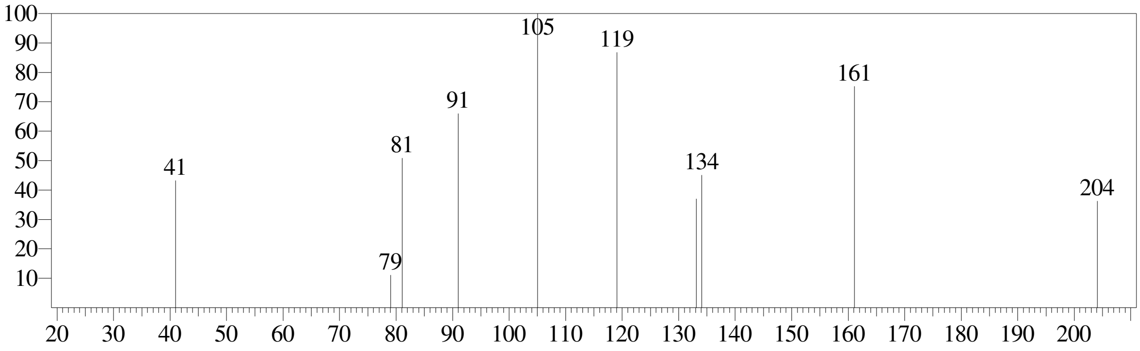

Hit#:3 Entry:24949 Library:NIST23s.lib
SI:71 Formula:C15H24 CAS:17699-14-8 MolWeight:204 RetIndex:1381
CompName:.alpha.-Cubebene $$ 1H-Cyclopenta[1,3]cyclopropa[1,2]benzene, 3a,3b,4,5,6,7-hexahydro-3,7-dimethyl-4-(1

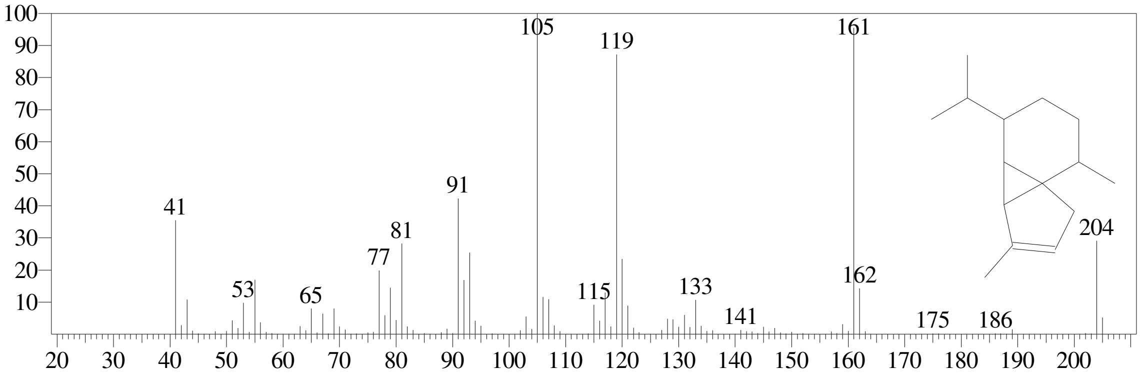

Hit#:4 Entry:24948 Library:NIST23s.lib
SI:71 Formula:C15H24 CAS:17699-14-8 MolWeight:204 RetIndex:1381
CompName:.alpha.-Cubebene $$ 1H-Cyclopenta[1,3]cyclopropa[1,2]benzene, 3a,3b,4,5,6,7-hexahydro-3,7-dimethyl-4-(1

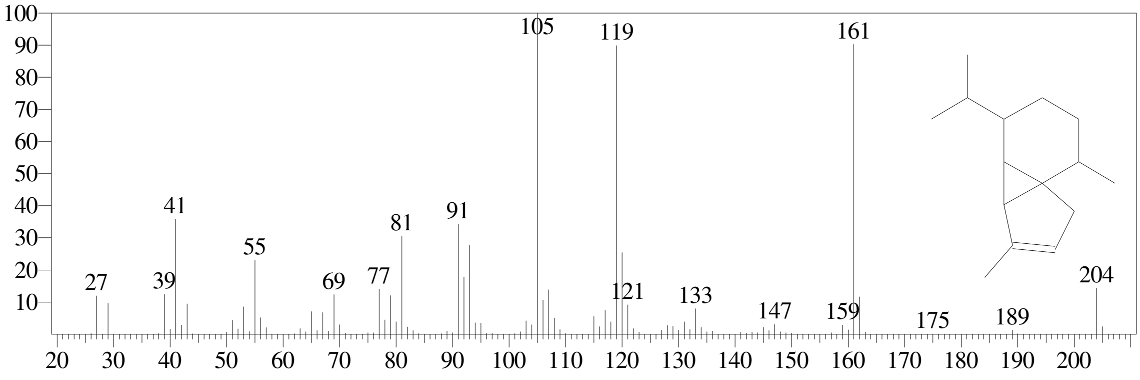

<< Target >>
Line#:15 R.Time:29.725(Scan#:3268) MassPeaks:10
RawMode:Averaged 29.717-29.733(3267-3269) BasePeak:105.05(3058)
BG Mode:None Group 1 - Event 1 Scan

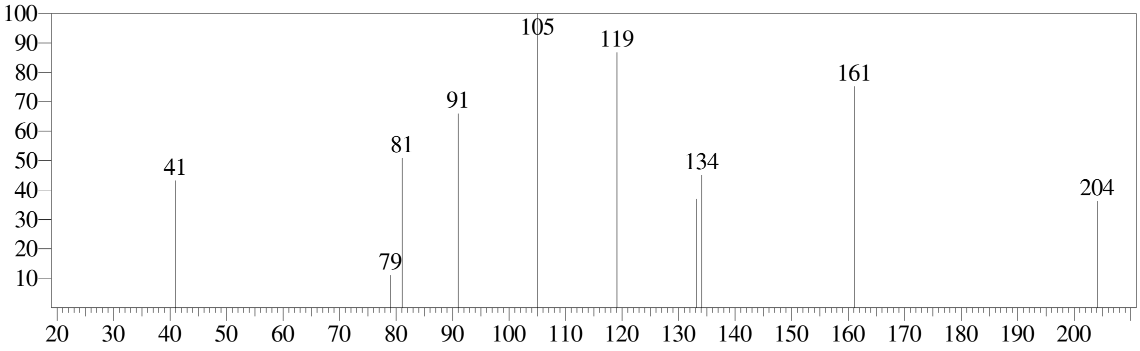

Hit#:5 Entry:62987 Library:NIST23-1.lib
SI:70 Formula:C15H24 CAS:267665-20-3 MolWeight:204 RetIndex:1465
CompName:(1S,4S,4aS)-1-Isopropyl-4,7-dimethyl-1,2,3,4,4a,5-hexahydronaphthalene $$ Naphthalene, 1,2,3,4,4a,5-hexah

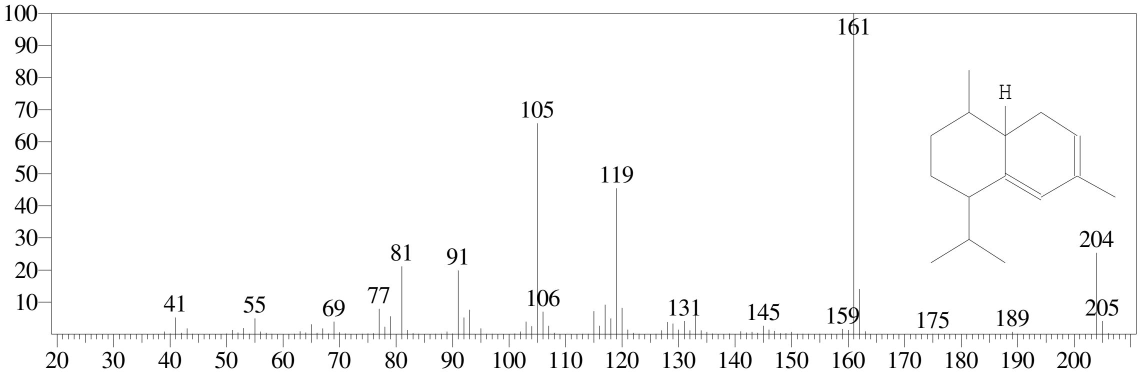

<< Target >>
Line#:16   R.Time:29.992(Scan#:3300) MassPeaks:28
RawMode:Averaged 29.983-30.000(3299-3301) BasePeak:161.10(11356)
BG Mode:None Group 1 - Event 1 Scan

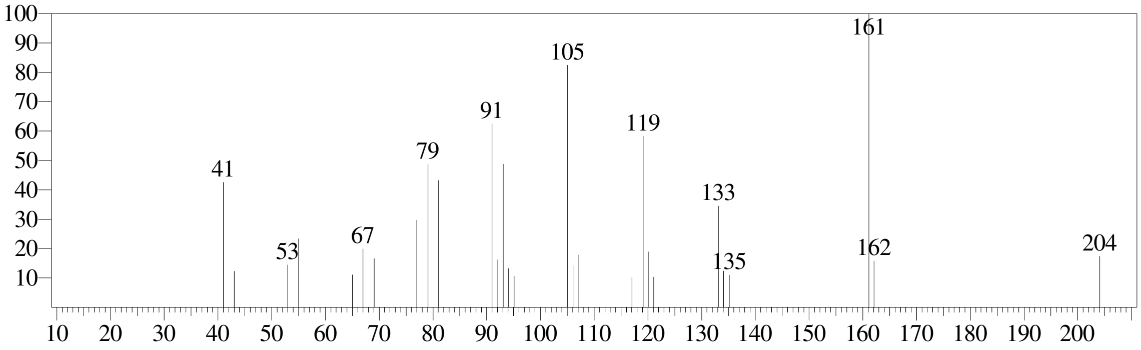

Hit#:1 Entry:25086 Library:NIST23s.lib
SI:90 Formula:C15H24 CAS:30021-74-0 MolWeight:204 RetIndex:1483
CompName:.gamma.-Muurolene $$ Naphthalene, 1,2,3,4,4a,5,6,8a-octahydro-7-methyl-4-methylene-1-(1-methylethyl)-, (

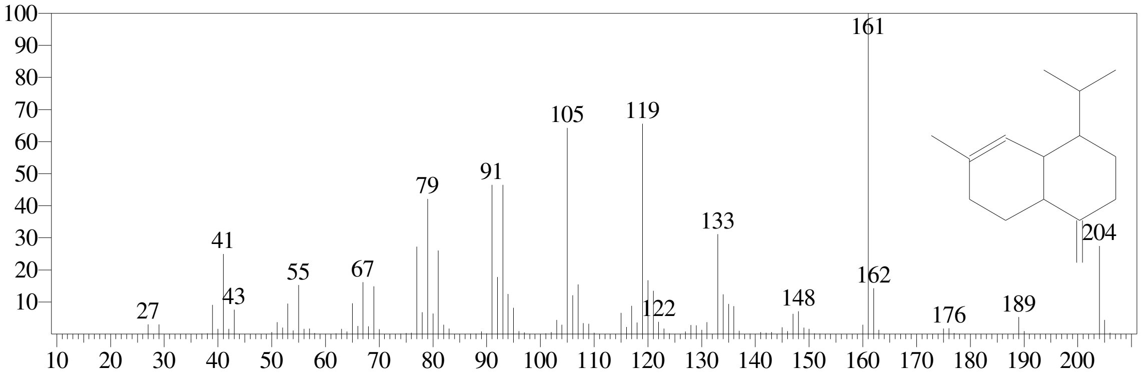

Hit#:2 Entry:62981 Library:NIST23-1.lib
SI:90 Formula:C15H24 CAS:23986-74-5 MolWeight:204 RetIndex:1478
CompName:Germacrene D $$ (S,1Z,6Z)-8-Isopropyl-1-methyl-5-methylenecyclodeca-1,6-diene $$ D-Germacrene $$ 1(1

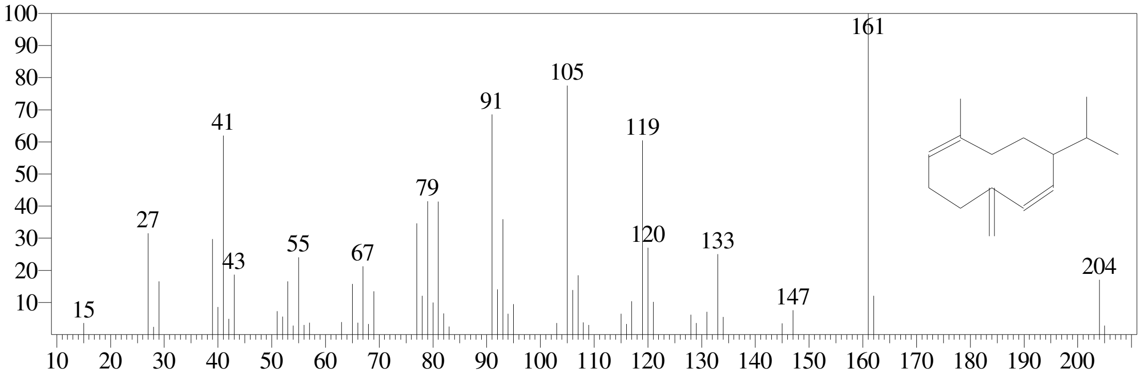

<< Target >>
Line#:16   R.Time:29.992(Scan#:3300) MassPeaks:28
RawMode:Averaged 29.983-30.000(3299-3301) BasePeak:161.10(11356)
BG Mode:None Group 1 - Event 1 Scan

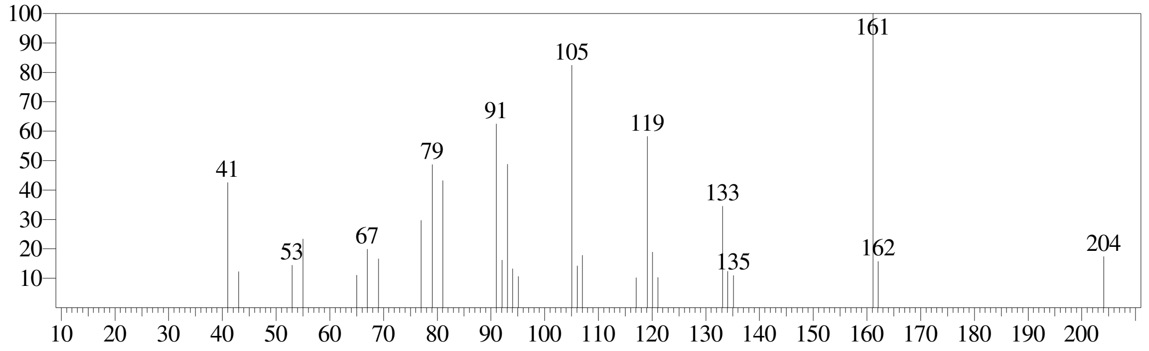

Hit#:3 Entry:25047 Library:NIST23s.lib
SI:90 Formula:C15H24 CAS:30021-74-0 MolWeight:204 RetIndex:1483
CompName:.gamma.-Muurolene $$ Naphthalene, 1,2,3,4,4a,5,6,8a-octahydro-7-methyl-4-methylene-1-(1-methylethyl)-, (

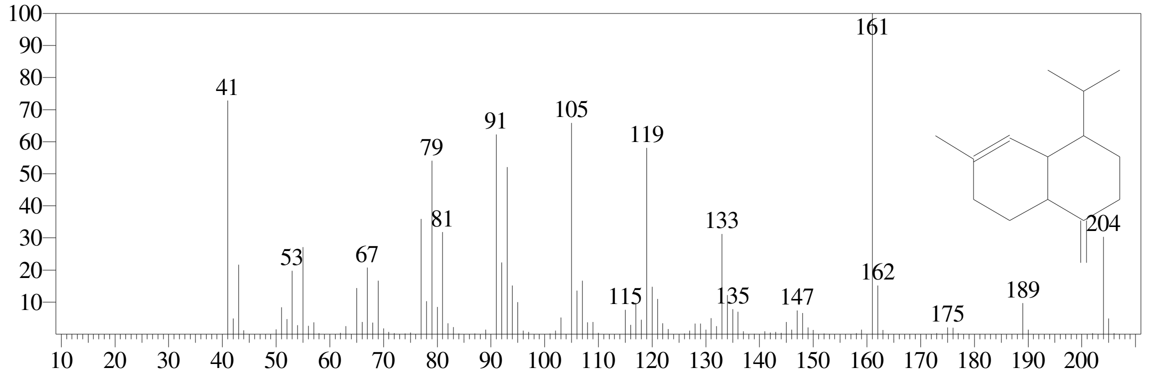

Hit#:4 Entry:25071 Library:NIST23s.lib
SI:89 Formula:C15H24 CAS:483-75-0 MolWeight:204 RetIndex:1500
CompName:Naphthalene, 1,2,4a,5,6,8a-hexahydro-4,7-dimethyl-1-(1-methylethyl)- $$ 1-Isopropyl-4,7-dimethyl-1,2,4a,5,

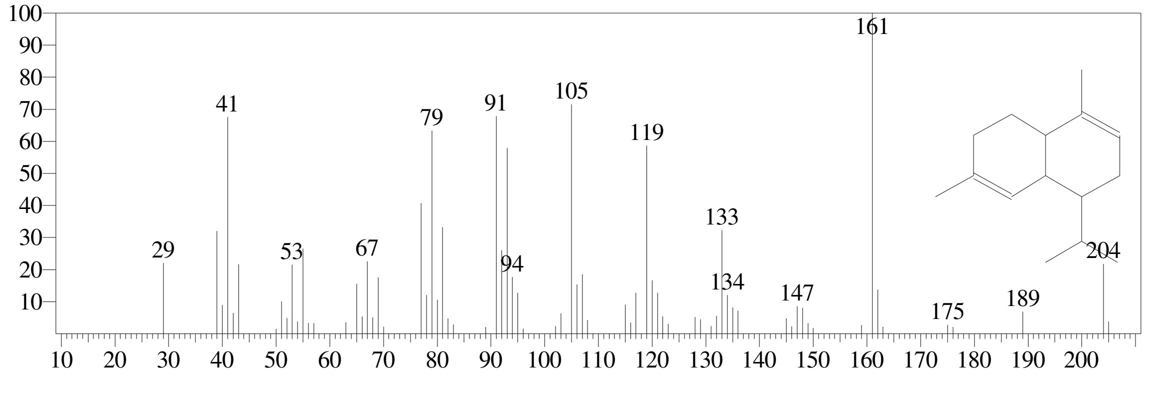

<< Target >>
Line#:16 R.Time:29.992(Scan#:3300) MassPeaks:28
RawMode:Averaged 29.983-30.000(3299-3301) BasePeak:161.10(11356)
BG Mode:None Group 1 - Event 1 Scan

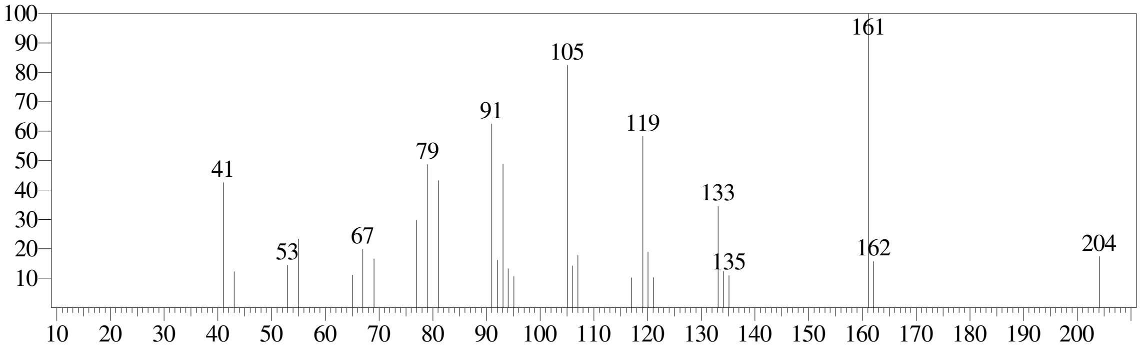

Hit#:5 Entry:62990 Library:NIST23-1.lib
SI:89 Formula:C15H24 CAS:6980-46-7 MolWeight:204 RetIndex:1483
CompName:(1S,4aR,8aS)-1-Isopropyl-7-methyl-4-methylene-1,2,3,4,4a,5,6,8a-octahydronaphthalene $$ .gamma.-Amorp

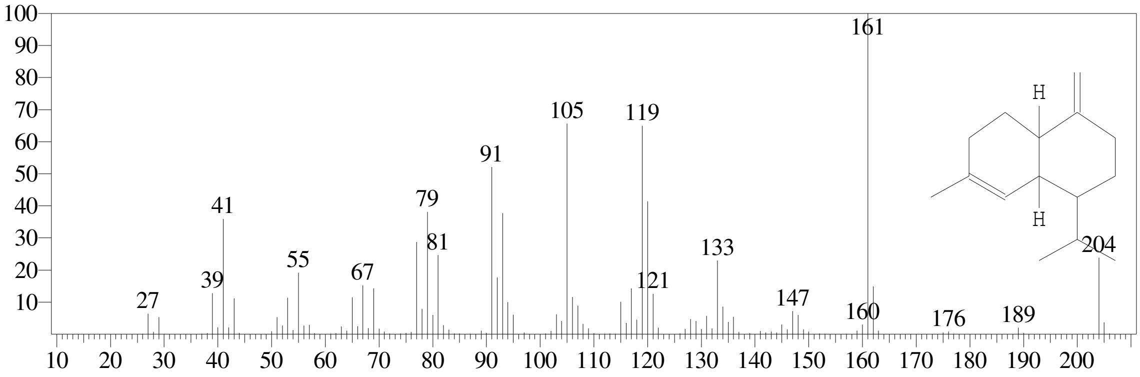

<< Target >>
Line#:17 R.Time:30.375(Scan#:3346) MassPeaks:40
RawMode:Averaged 30.367-30.383(3345-3347) BasePeak:119.10(20970)
BG Mode:None Group 1 - Event 1 Scan

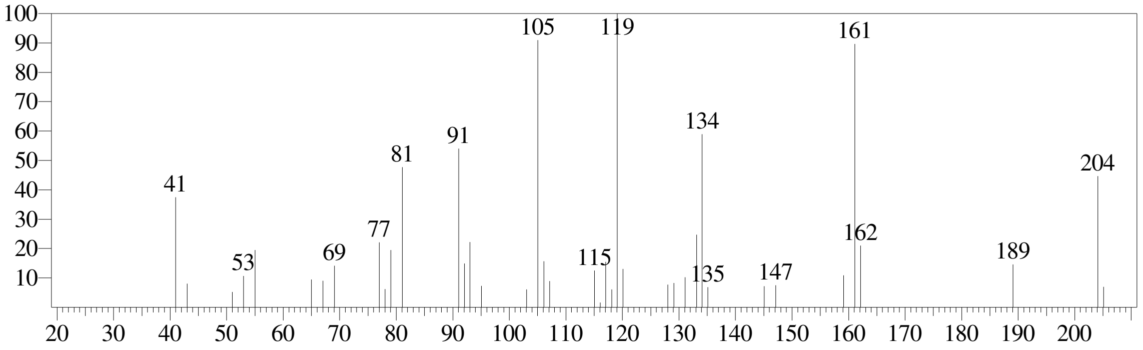

Hit#:1 Entry:63006 Library:NIST23-1.lib
SI:92 Formula:C15H24 CAS:16729-01-4 MolWeight:204 RetIndex:1526
CompName:1-Isopropyl-4,7-dimethyl-1,2,3,5,6,8a-hexahydronaphthalene $$ Cadina-1(10),4-diene $$

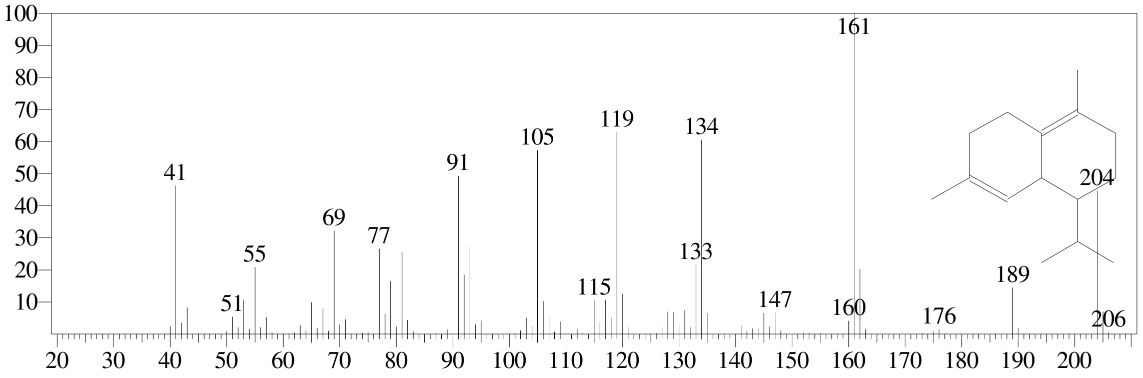

Hit#:2 Entry:63005 Library:NIST23-1.lib
SI:92 Formula:C15H24 CAS:189165-79-5 MolWeight:204 RetIndex:1526
CompName:Amorphene,delta-

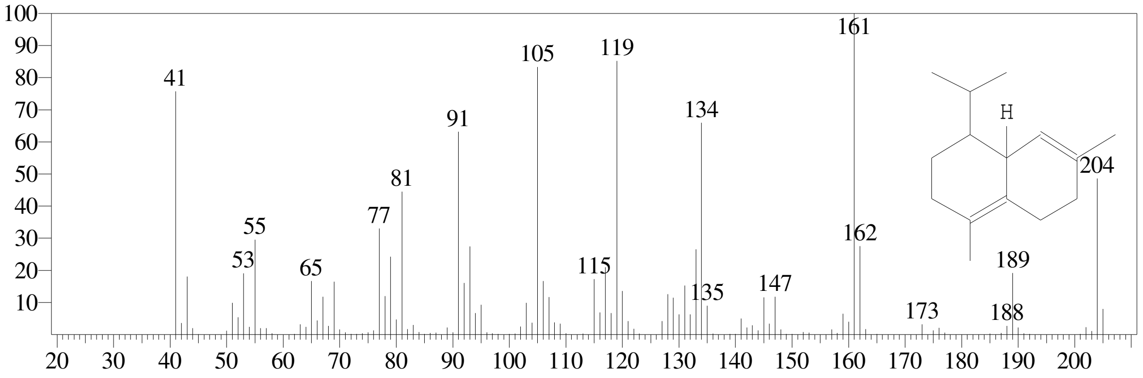

<< Target >>
Line#:17   R.Time:30.375(Scan#:3346) MassPeaks:40
RawMode:Averaged 30.367-30.383(3345-3347) BasePeak:119.10(20970)
BG Mode:None Group 1 - Event 1 Scan

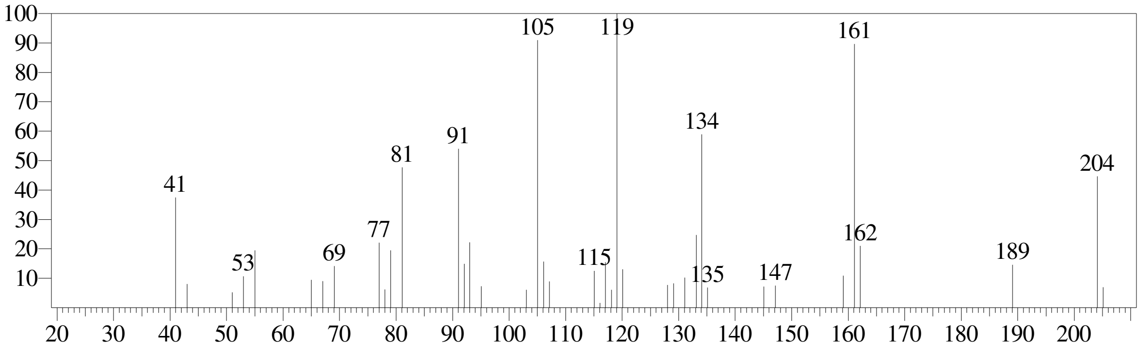

Hit#:3 Entry:25090 Library:NIST23s.lib
SI:91 Formula:C15H24 CAS:483-76-1 MolWeight:204 RetIndex:1526
CompName:Naphthalene, 1,2,3,5,6,8a-hexahydro-4,7-dimethyl-1-(1-methylethyl)-, (1S-cis)- $$ Cadina-1(10),4-diene $$.

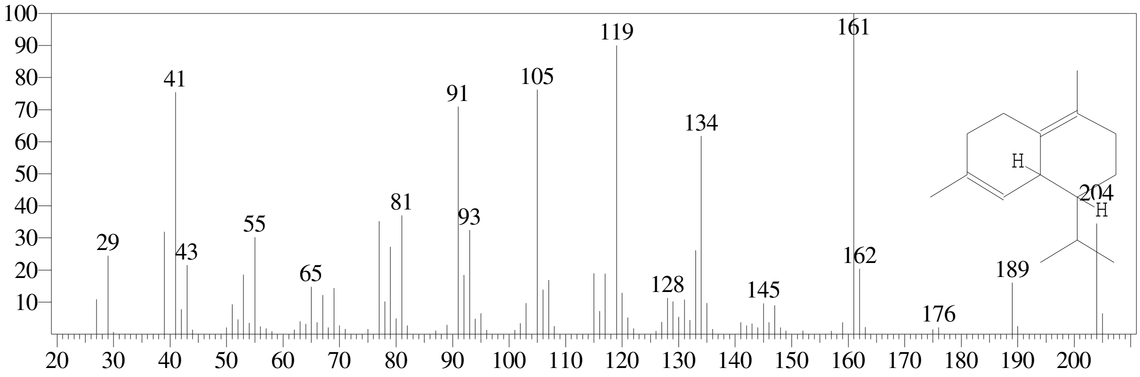

Hit#:4 Entry:25112 Library:NIST23s.lib
SI:90 Formula:C15H24 CAS:483-76-1 MolWeight:204 RetIndex:1526
CompName:Naphthalene, 1,2,3,5,6,8a-hexahydro-4,7-dimethyl-1-(1-methylethyl)-, (1S-cis)- $$ Cadina-1(10),4-diene $$.

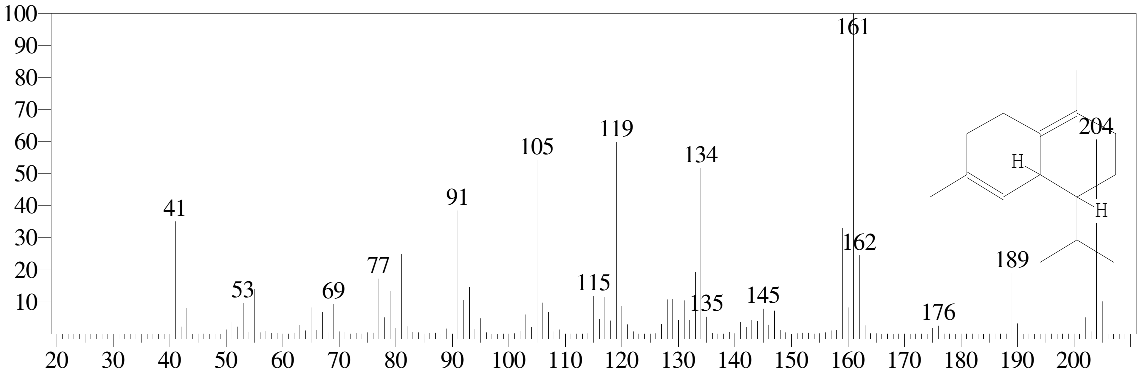

<< Target >>
Line#:17   R.Time:30.375(Scan#:3346) MassPeaks:40
RawMode:Averaged 30.367-30.383(3345-3347) BasePeak:119.10(20970)
BG Mode:None Group 1 - Event 1 Scan

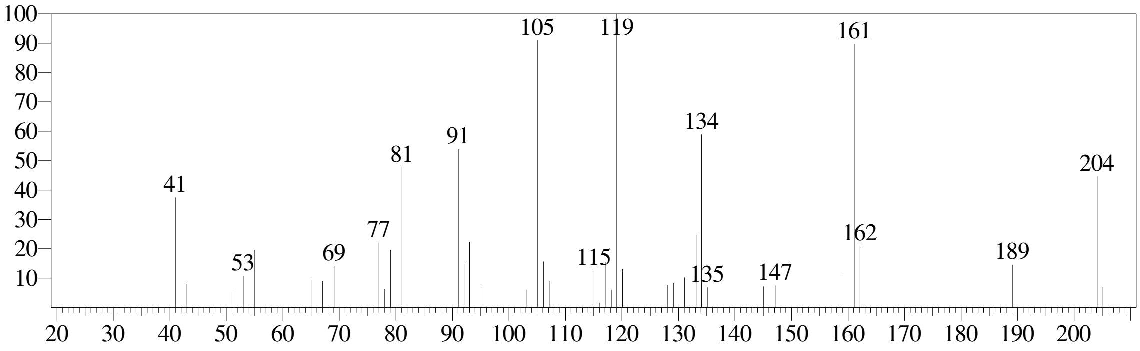

Hit#:5 Entry:24949 Library:NIST23s.lib
SI:89 Formula:C15H24 CAS:17699-14-8 MolWeight:204 RetIndex:1381
CompName:.alpha.-Cubebene $$ 1H-Cyclopenta[1,3]cyclopropa[1,2]benzene, 3a,3b,4,5,6,7-hexahydro-3,7-dimethyl-4-(1

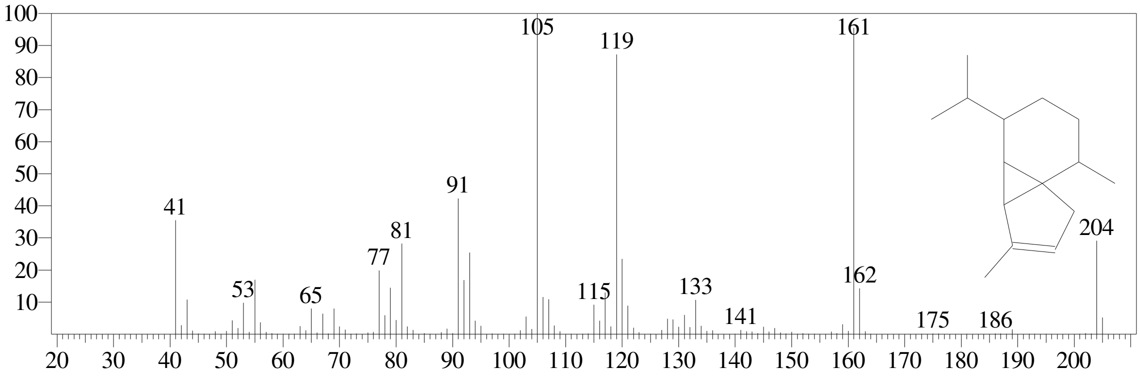

<< Target >>
Line#:18 R.Time:30.808(Scan#:3398) MassPeaks:38
RawMode:Averaged 30.800-30.817(3397-3399) BasePeak:105.05(10215)
BG Mode:Calc. from Peak Group 1 - Event 1 Scan

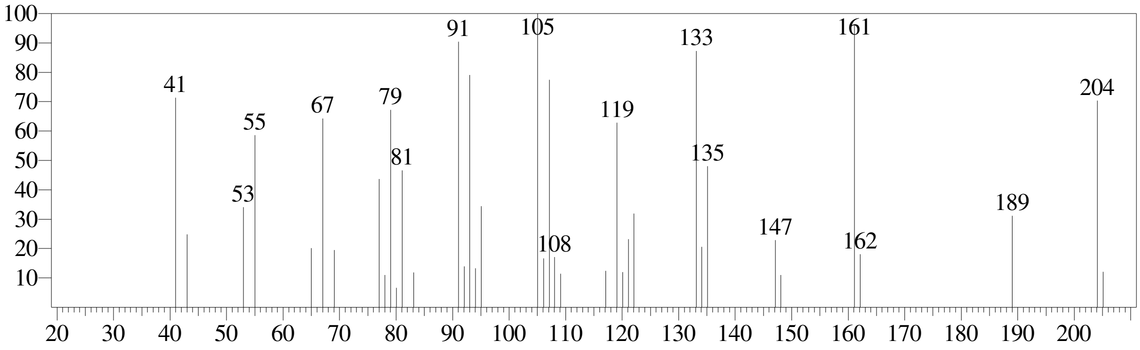

Hit#:1 Entry:62764 Library:NIST23-1.lib
SI:92 Formula:C15H24 CAS:489-39-4 MolWeight:204 RetIndex:1424
CompName:Aromandendrene $$ 1H-Cycloprop[e]azulene, decahydro-1,1,7-trimethyl-4-methylene-, [1aR-(1a.alpha.,4a.al

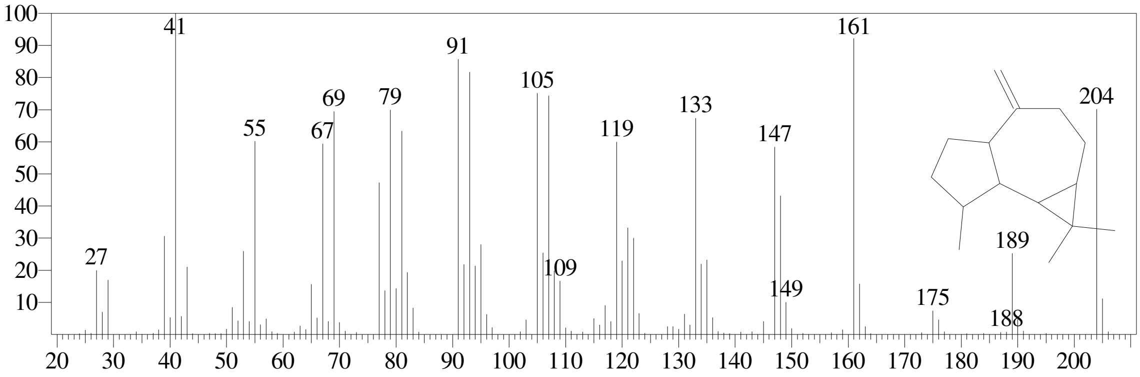

Hit#:2 Entry:24930 Library:NIST23s.lib
SI:91 Formula:C15H24 CAS:25246-27-9 MolWeight:204 RetIndex:1424
CompName:Alloaromadendrene $$ 1H-Cycloprop[e]azulene, decahydro-1,1,7-trimethyl-4-methylene-, [1aR-(1a.alpha.,4a

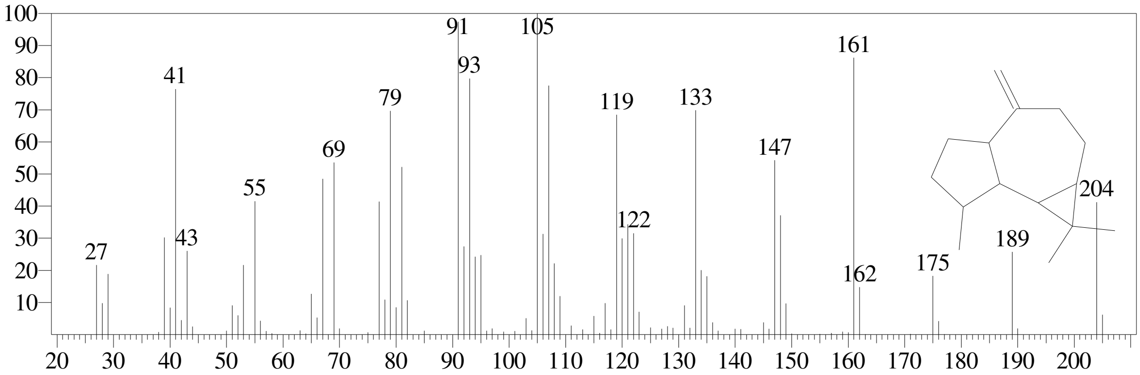

<< Target >>
Line#:18   R.Time:30.808(Scan#:3398) MassPeaks:38
RawMode:Averaged 30.800-30.817(3397-3399) BasePeak:105.05(10215)
BG Mode:Calc. from Peak Group 1 - Event 1 Scan

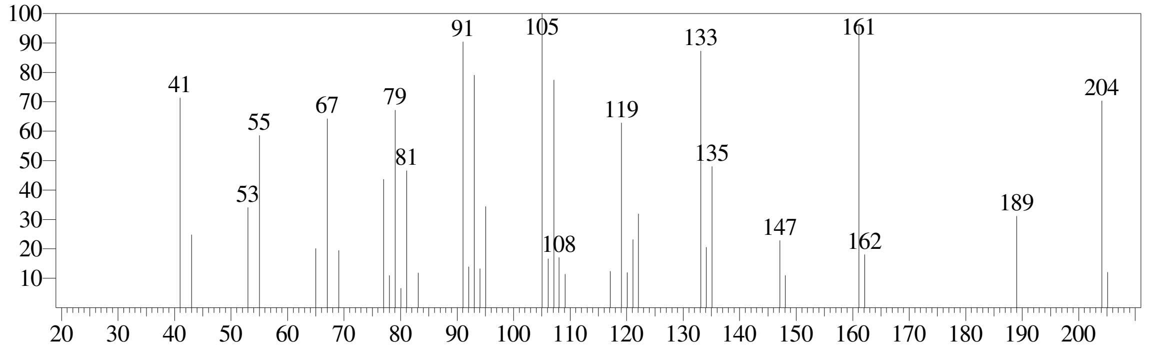

Hit#:3 Entry:62966 Library:NIST23-1.lib
SI:91 Formula:C15H24 CAS:25246-27-9 MolWeight:204 RetIndex:1424
CompName:Alloaromadendrene $$ 1H-Cycloprop[e]azulene, decahydro-1,1,7-trimethyl-4-methylene-, [1aR-(1a.alpha.,4a

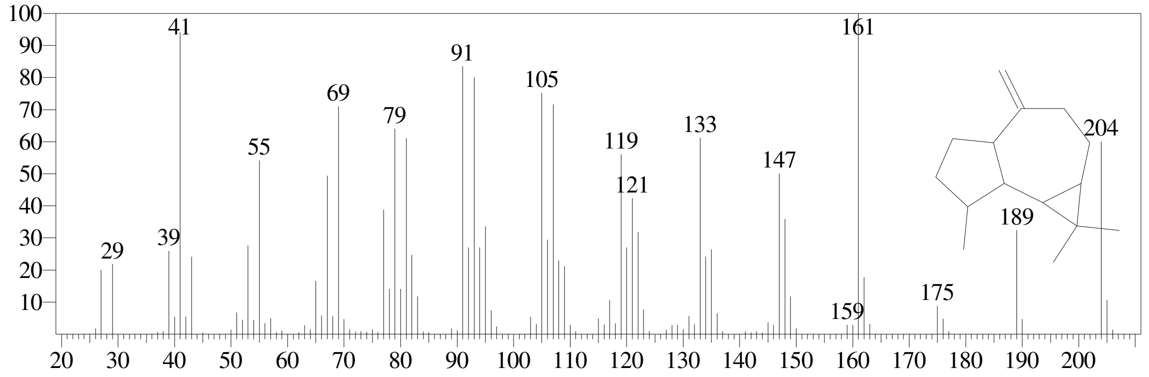

Hit#:4 Entry:62794 Library:NIST23-1.lib
SI:91 Formula:C15H24 CAS:68832-35-9 MolWeight:204 RetIndex:1450
CompName:(1R,9R,E)-4,11,11-Trimethyl-8-methylenebicyclo[7.2.0]undec-4-ene $$ Bicyclo[7.2.0]undec-4-ene, 4,11,11-t

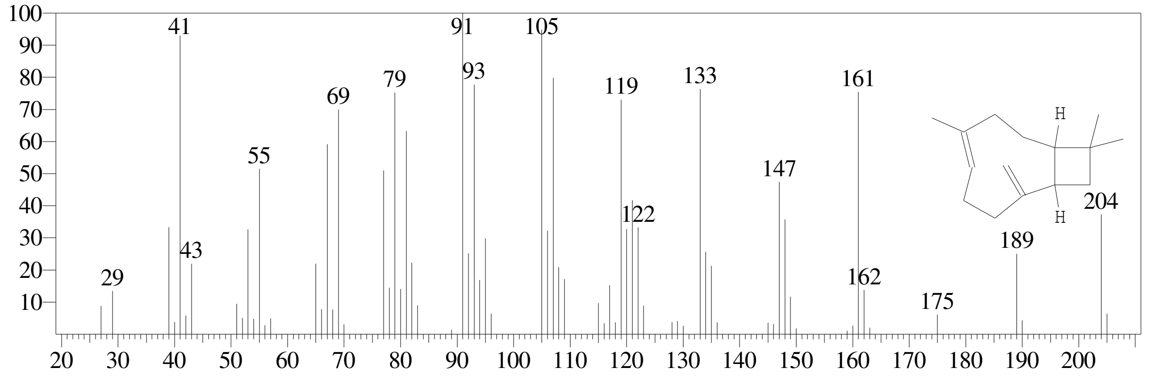

<< Target >>
Line#:18   R.Time:30.808(Scan#:3398) MassPeaks:38
RawMode:Averaged 30.800-30.817(3397-3399) BasePeak:105.05(10215)
BG Mode:Calc. from Peak Group 1 - Event 1 Scan

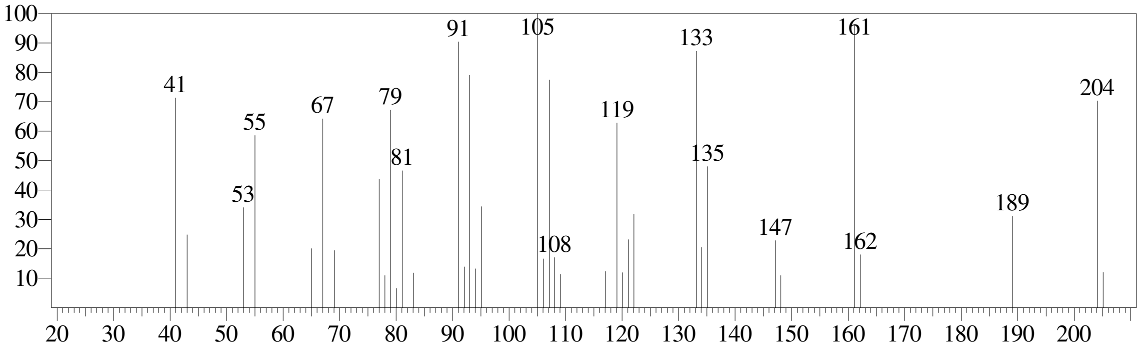

Hit#:5 Entry:25106 Library:NIST23s.lib
SI:91 Formula:C15H24 CAS:20071-49-2 MolWeight:204 RetIndex:1421
CompName:1H-Cyclopropa[a]naphthalene, decahydro-1,1,3a-trimethyl-7-methylene-, [1aS-(1a.alpha.,3a.alpha.,7a.beta.,7l

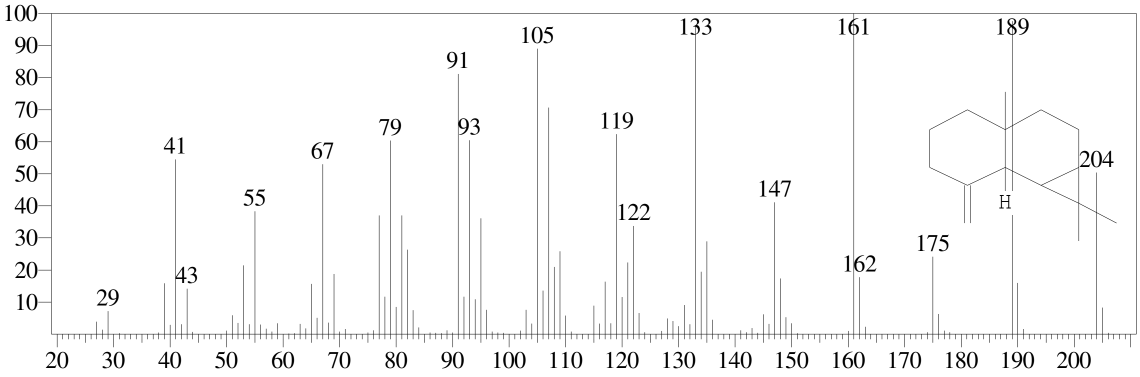

<< Target >>
Line#:19 R.Time:30.917(Scan#:3411) MassPeaks:19
RawMode:Averaged 30.908-30.925(3410-3412) BasePeak:105.05(7132)
BG Mode:Calc. from Peak Group 1 - Event 1 Scan

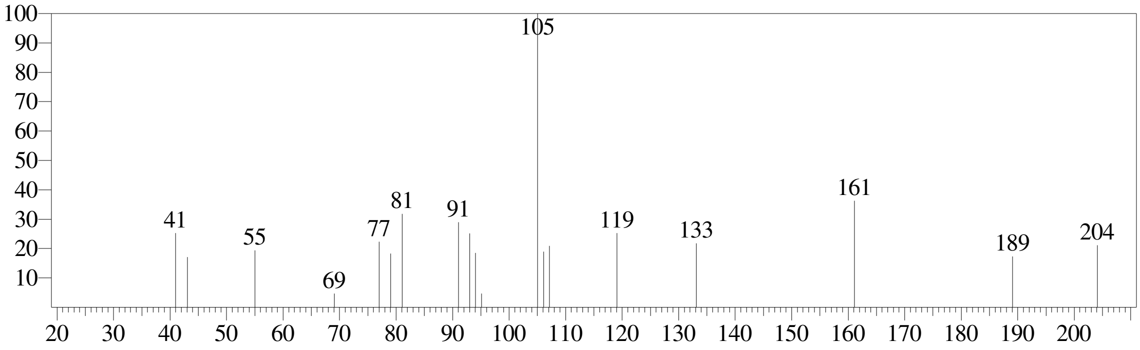

Hit#:1 Entry:62856 Library:NIST23-1.lib
SI:82 Formula:C15H24 CAS:10208-80-7 MolWeight:204 RetIndex:1500
CompName:.alpha.-Muurolene $$ Naphthalene, 1,2,4a,5,6,8a-hexahydro-4,7-dimethyl-1-(1-methylethyl)-, (1S,4aS,8aR)- $

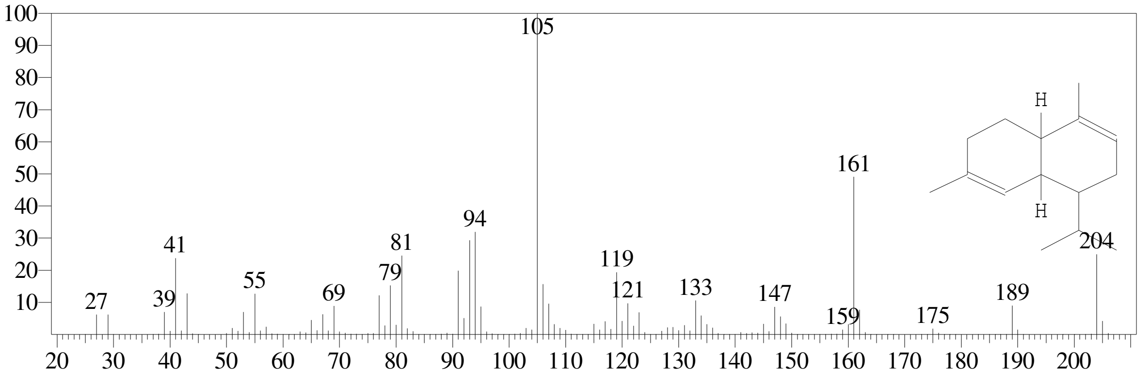

Hit#:2 Entry:62857 Library:NIST23-1.lib
SI:82 Formula:C15H24 CAS:483-75-0 MolWeight:204 RetIndex:1500
CompName:Naphthalene, 1,2,4a,5,6,8a-hexahydro-4,7-dimethyl-1-(1-methylethyl)- $$ 1-Isopropyl-4,7-dimethyl-1,2,4a,5,

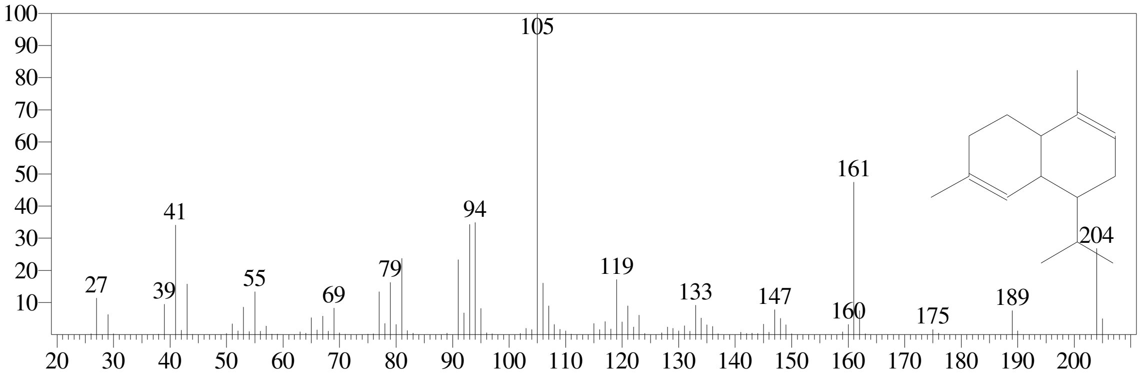

<< Target >>
Line#:19   R.Time:30.917(Scan#:3411) MassPeaks:19
RawMode:Averaged 30.908-30.925(3410-3412) BasePeak:105.05(7132)
BG Mode:Calc. from Peak Group 1 - Event 1 Scan

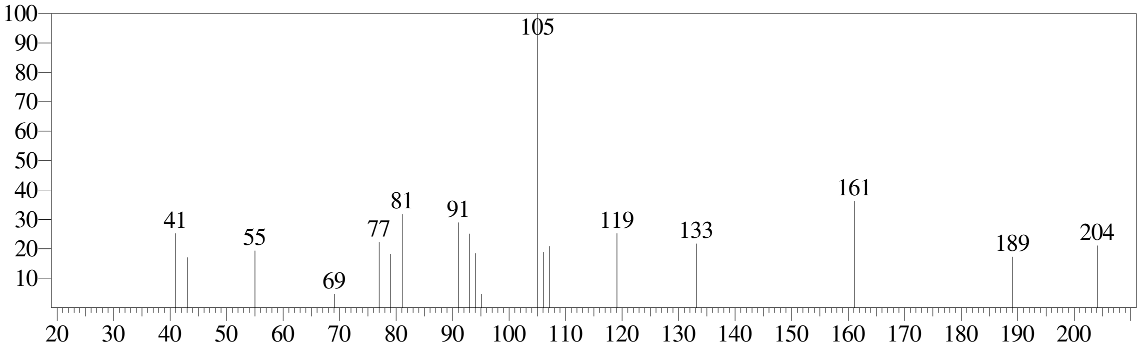

Hit#:3 Entry:24947 Library:NIST23s.lib
SI:82 Formula:C15H24 CAS:31983-22-9 MolWeight:204 RetIndex:1498
CompName:Naphthalene, 1,2,4a,5,6,8a-hexahydro-4,7-dimethyl-1-(1-methylethyl)-, (1.alpha.,4a.alpha.,8a.alpha.)- $$ [1.a

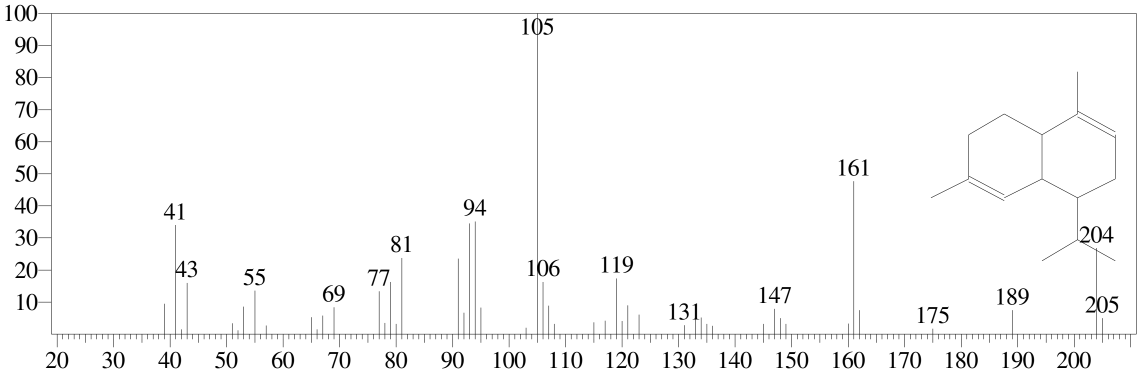

Hit#:4 Entry:62854 Library:NIST23-1.lib
SI:81 Formula:C15H24 CAS:17627-24-6 MolWeight:204 RetIndex:1500
CompName:Naphthalene, 1,2,4a,5,6,8a-hexahydro-4,7-dimethyl-1-(1-methylethyl)-,[1R-(1.alpha.,4a.alpha.,8a.alpha.)]- $$

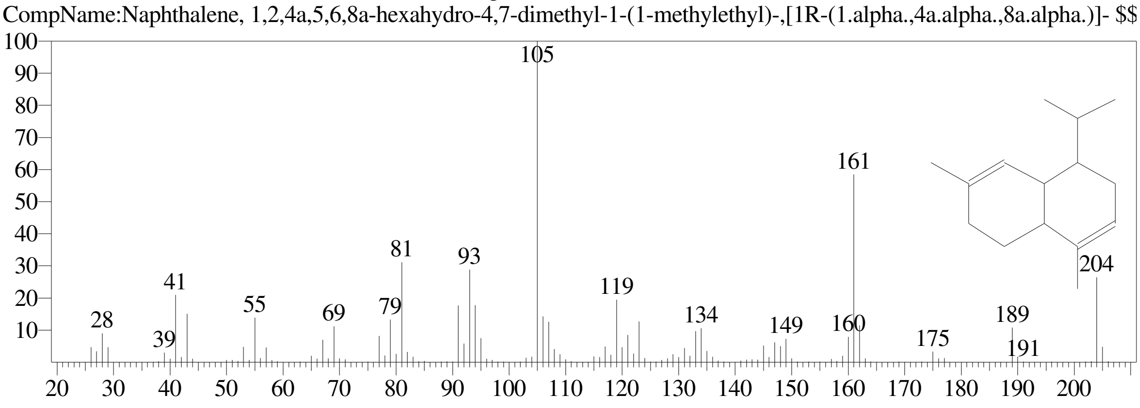

<< Target >>
Line#:19   R.Time:30.917(Scan#:3411) MassPeaks:19
RawMode:Averaged 30.908-30.925(3410-3412) BasePeak:105.05(7132)
BG Mode:Calc. from Peak Group 1 - Event 1 Scan

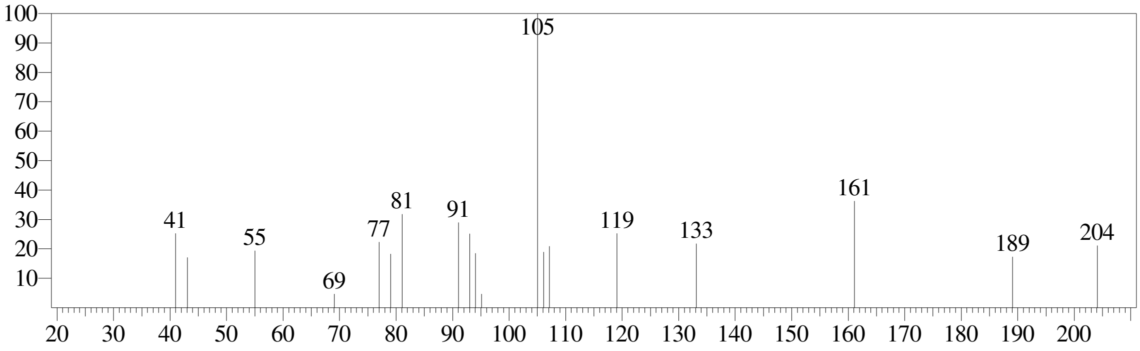

Hit#:5 Entry:24946 Library:NIST23s.lib
SI:80 Formula:C15H24 CAS:10208-80-7 MolWeight:204 RetIndex:1500
CompName:.alpha.-Muurolene $$ Naphthalene, 1,2,4a,5,6,8a-hexahydro-4,7-dimethyl-1-(1-methylethyl)-, (1S,4aS,8aR)- $

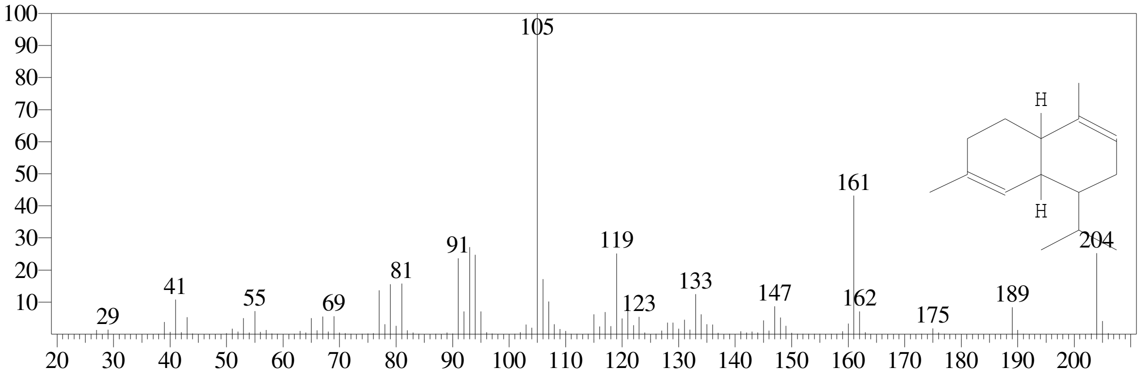

<< Target >>
Line#:20 R.Time:31.058(Scan#:3428) MassPeaks:25
RawMode:Averaged 31.050-31.067(3427-3429) BasePeak:107.10(7757)
BG Mode:Calc. from Peak Group 1 - Event 1 Scan

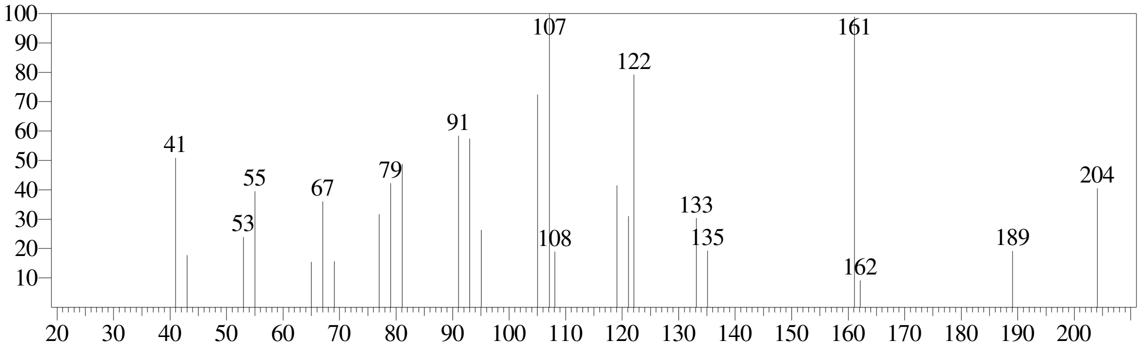

Hit#:1 Entry:25028 Library:NIST23s.lib
SI:85 Formula:C15H24 CAS:56633-28-4 MolWeight:204 RetIndex:1410
CompName:(-)-.alpha.-Panasinsen $$ (2aR,4aS,8aR)-2,2,4a,8-Tetramethyl-1,2,2a,3,4,4a,5,6-octahydrocyclobuta[c]indene

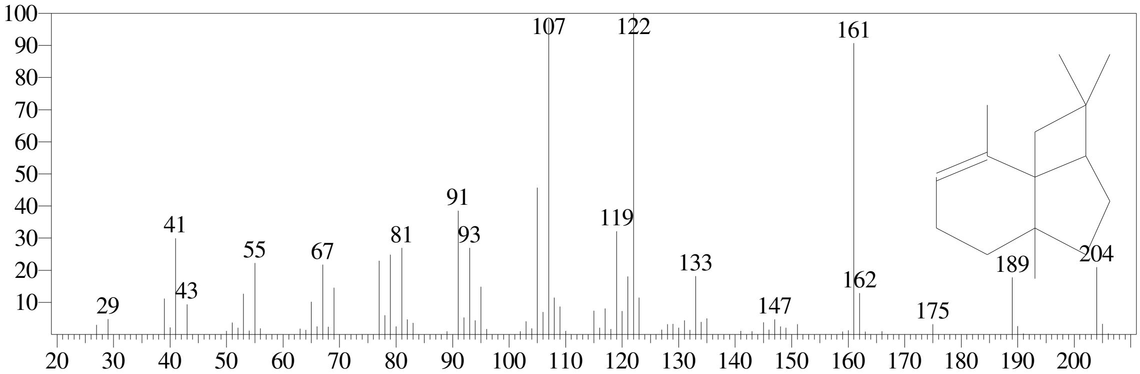

Hit#:2 Entry:63002 Library:NIST23-1.lib
SI:84 Formula:C15H24 CAS:6813-21-4 MolWeight:204 RetIndex:1522
CompName:Selina-3,7(11)-diene $$ Naphthalene, 1,2,3,4,4a,5,6,8a-octahydro-4a,8-dimethyl-2-(1-methylethylidene)-, (4a

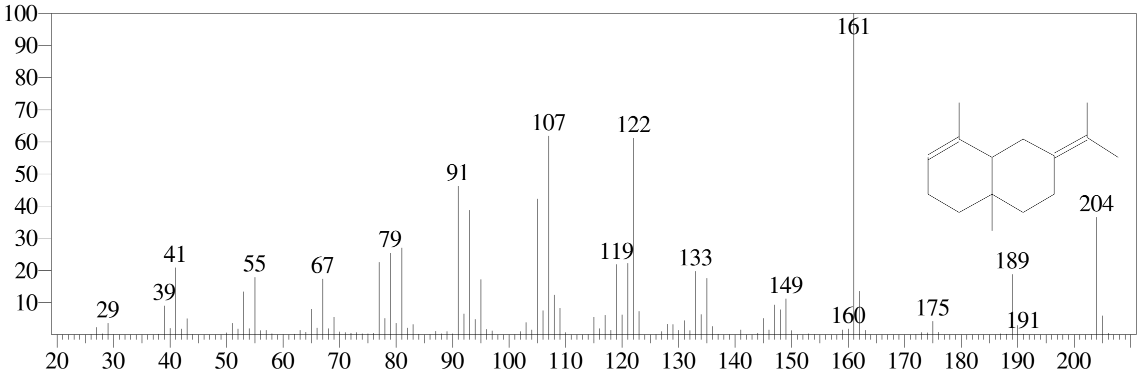

<< Target >>
Line#:20 R.Time:31.058(Scan#:3428) MassPeaks:25
RawMode:Averaged 31.050-31.067(3427-3429) BasePeak:107.10(7757)
BG Mode:Calc. from Peak Group 1 - Event 1 Scan

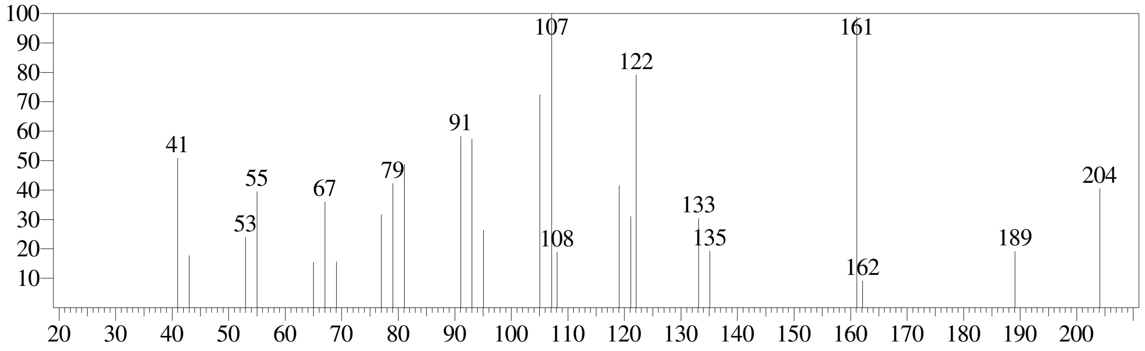

Hit#:3 Entry:25092 Library:NIST23s.lib
SI:83 Formula:C15H24 CAS:6813-21-4 MolWeight:204 RetIndex:1522
CompName:Selina-3,7(11)-diene $$ Naphthalene, 1,2,3,4,4a,5,6,8a-octahydro-4a,8-dimethyl-2-(1-methylethylidene)-, (4a

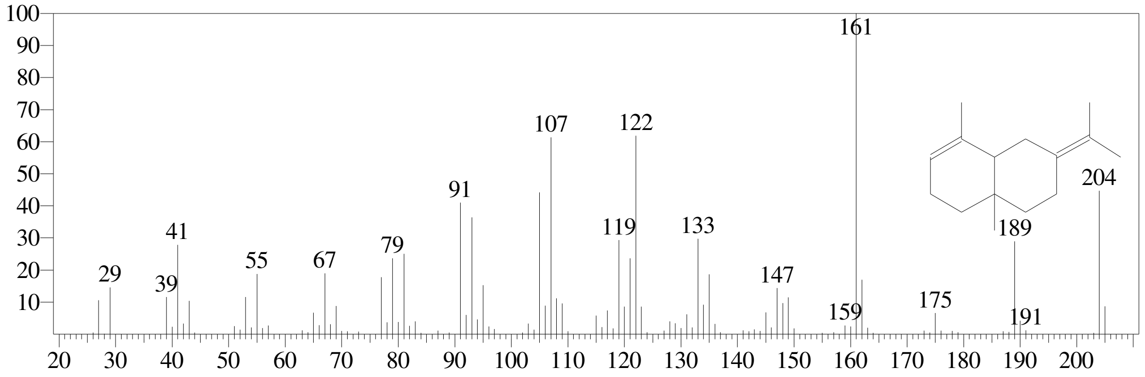

Hit#:4 Entry:24894 Library:NIST23s.lib
SI:83 Formula:C15H24 CAS:3691-11-0 MolWeight:204 RetIndex:1498
CompName:Azulene, 1,2,3,5,6,7,8,8a-octahydro-1,4-dimethyl-7-(1-methylethenyl)-, [1S-(1.alpha.,7.alpha.,8a.beta.)]- $$ (

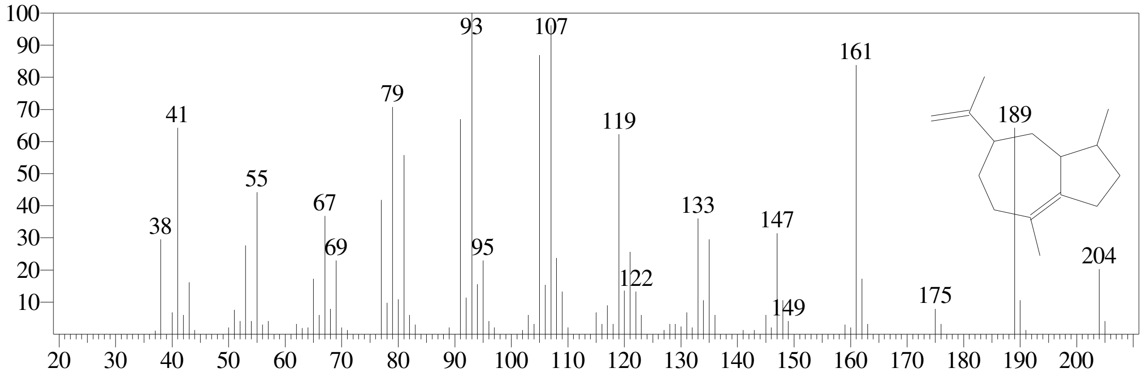

<< Target >>
Line#:20 R.Time:31.058(Scan#:3428) MassPeaks:25
RawMode:Averaged 31.050-31.067(3427-3429) BasePeak:107.10(7757)
BG Mode:Calc. from Peak Group 1 - Event 1 Scan

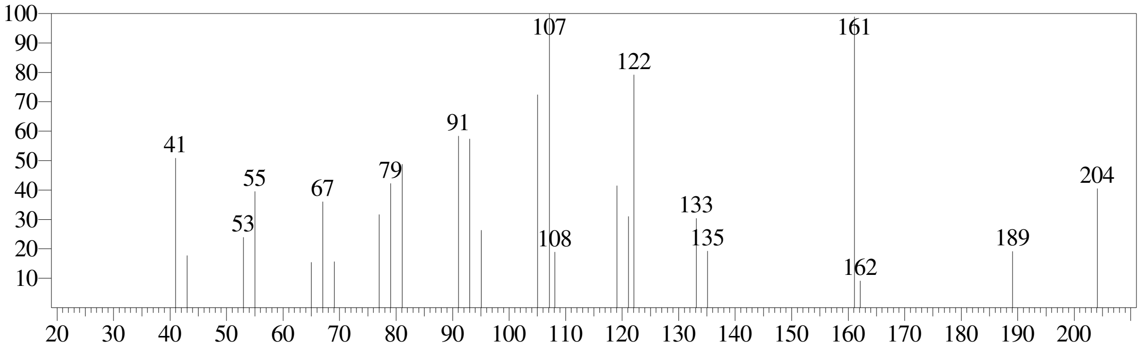

Hit#:5 Entry:25041 Library:NIST23s.lib
SI:83 Formula:C15H24 CAS:4630-07-3 MolWeight:204 RetIndex:1496
CompName:Naphthalene, 1,2,3,5,6,7,8,8a-octahydro-1,8a-dimethyl-7-(1-methylethenyl)-, [1R-(1.alpha.,7.beta.,8a.alpha.)]

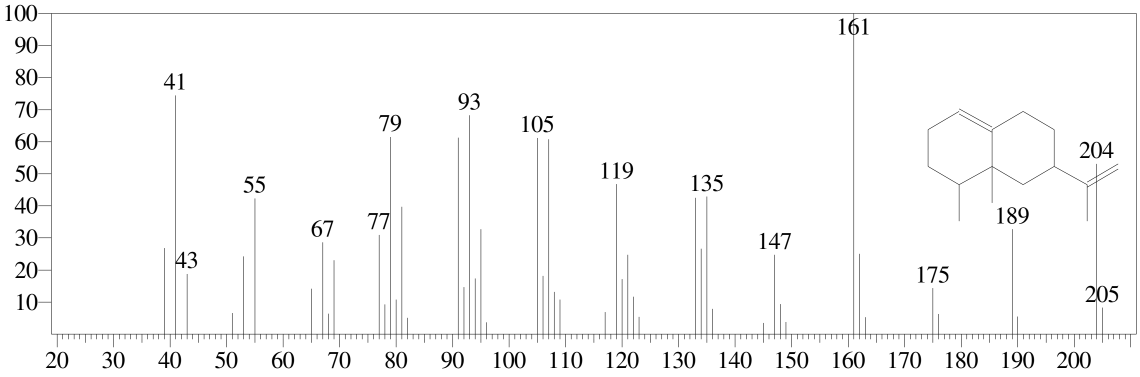

<< Target >>
Line#:21   R.Time:31.217(Scan#:3447) MassPeaks:5
RawMode:Averaged 31.208-31.225(3446-3448) BasePeak:91.00(1507)
BG Mode:None Group 1 - Event 1 Scan

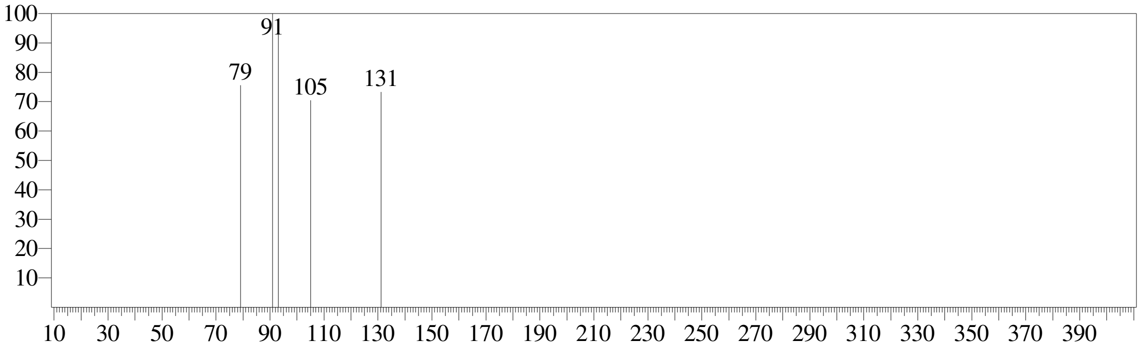

Hit#:1 Entry:3165 Library:NIST23-1.lib
SI:75 Formula:C8H12 CAS:0-00-0 MolWeight:108 RetIndex:870
CompName:Spiro[bicyclo[3.1.0]hexane-2,1'-cyclopropane]

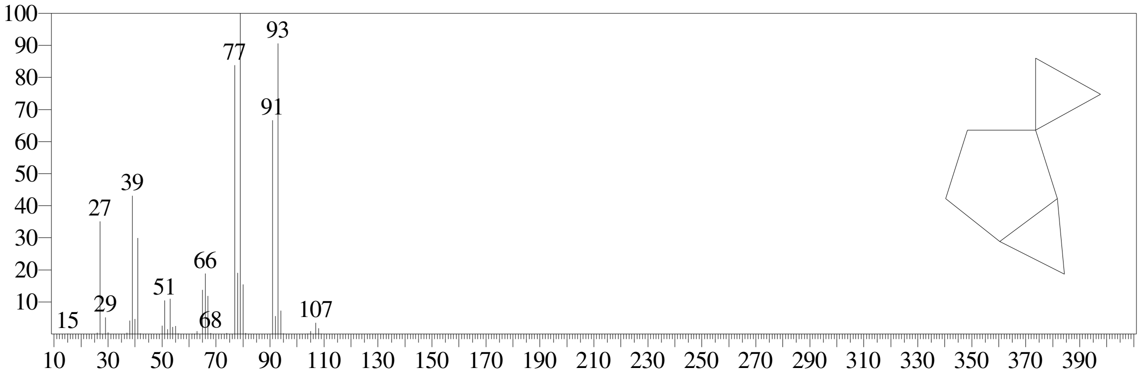

Hit#:2 Entry:3192 Library:NIST23-1.lib
SI:72 Formula:C8H12 CAS:6196-78-7 MolWeight:108 RetIndex:788
CompName:1-Methylene-2-vinylcyclopentane $$ Cyclopentane, 1-ethenyl-2-methylene- $$ 2-Methylenevinylcyclopentan

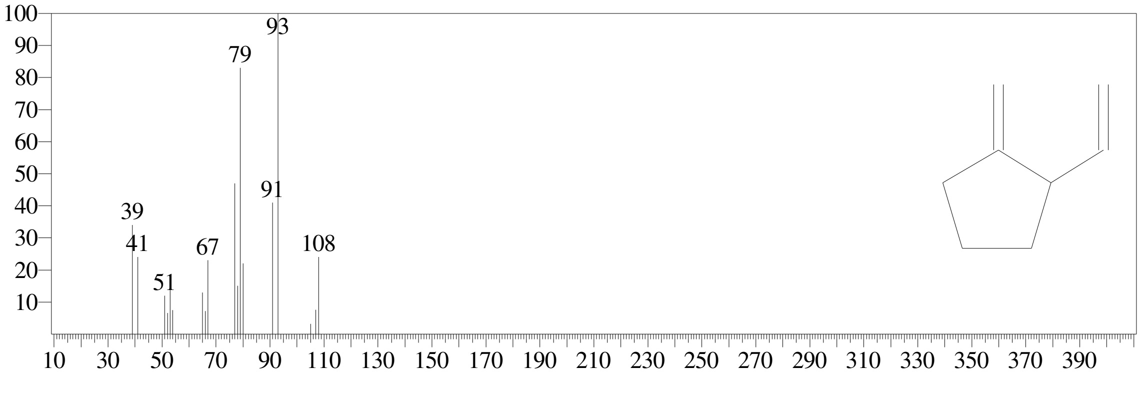

<< Target >>
Line#:21   R.Time:31.217(Scan#:3447) MassPeaks:5
RawMode:Averaged 31.208-31.225(3446-3448) BasePeak:91.00(1507)
BG Mode:None Group 1 - Event 1 Scan

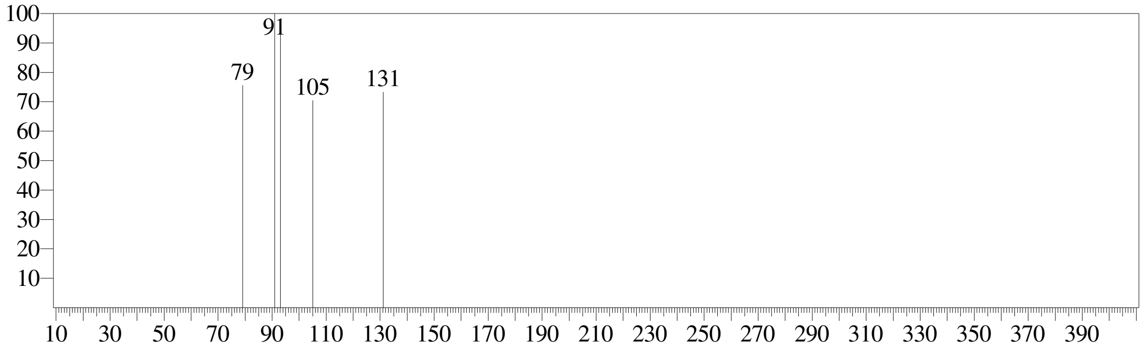

Hit#:3 Entry:51019 Library:NIST23-2.lib
SI:72 Formula:C23H23NO6 CAS:17037-55-7 MolWeight:409 RetIndex:3061
CompName:7-Azabicyclo[2.2.1]heptane-2,7-dicarboxylic acid, 3-hydroxy-, 7-benzyl methyl ester, benzoate (ester), endo-2

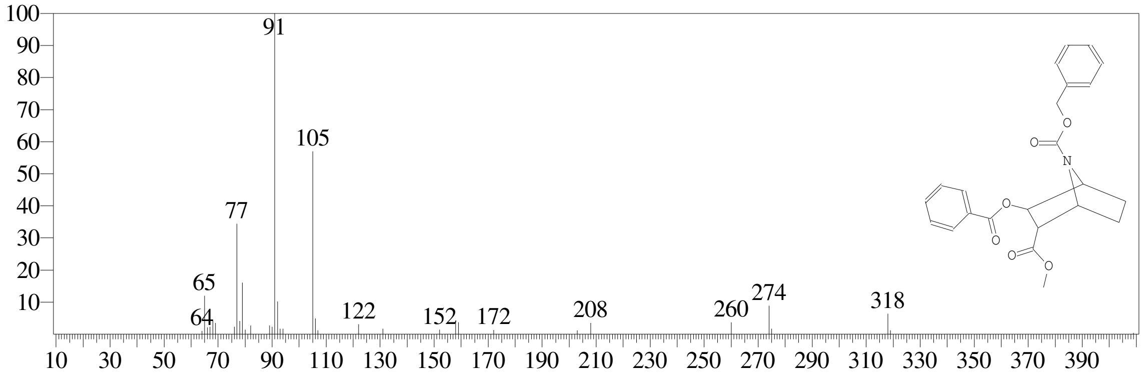

Hit#:4 Entry:36844 Library:NIST23-1.lib
SI:71 Formula:C11H16N2 CAS:0-00-0 MolWeight:176 RetIndex:1645
CompName:Spiro[3,4-diazatricyclo[5.2.2.0(2,6)]undec-3-ene-5,1'-cyclopropane]

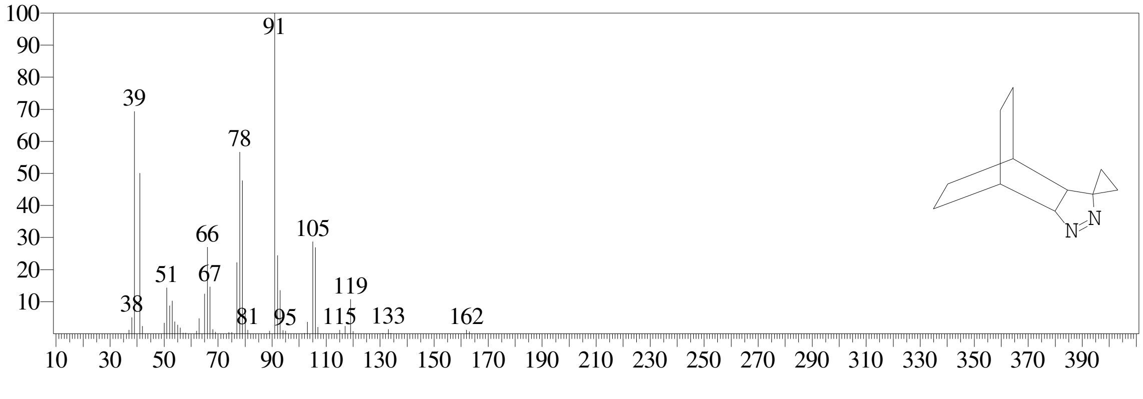

<< Target >>
Line#:21   R.Time:31.217(Scan#:3447) MassPeaks:5
RawMode:Averaged 31.208-31.225(3446-3448) BasePeak:91.00(1507)
BG Mode:None Group 1 - Event 1 Scan

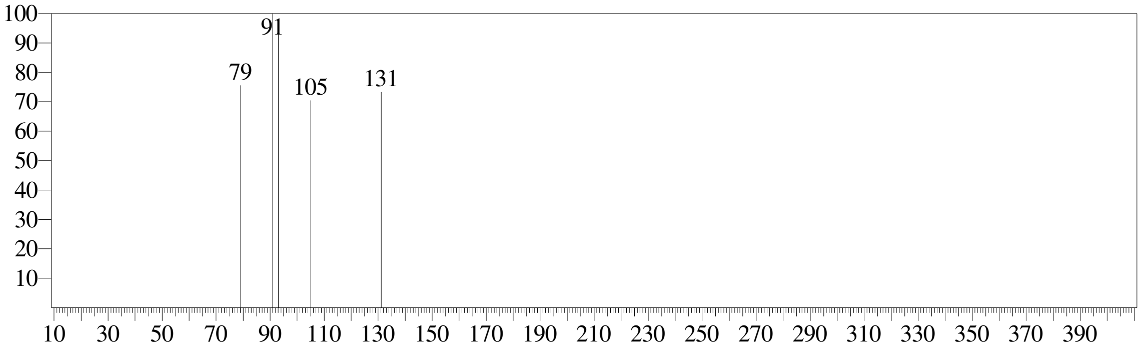

Hit#:5 Entry:3188 Library:NIST23-1.lib
SI:71 Formula:C8H12 CAS:61142-26-5 MolWeight:108 RetIndex:813
CompName:Cyclopropene, 1-methyl-3-(2-methylcyclopropyl)- $$ 1-Methyl-3-(2-methylcyclopropyl)-1-cyclopropene # $

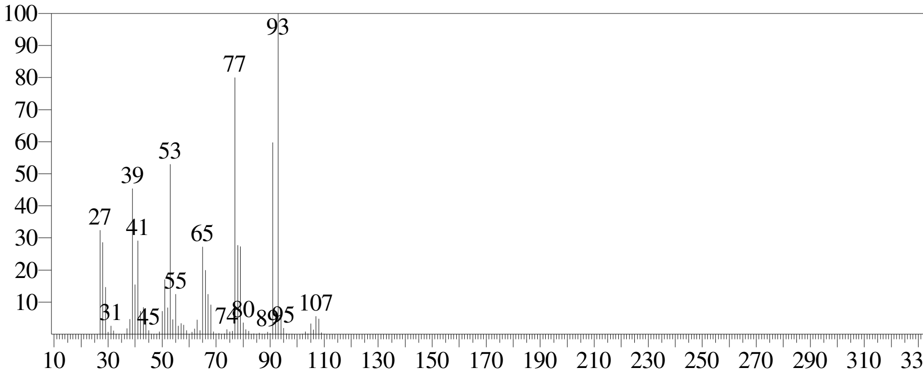

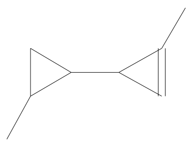

<< Target >>
Line#:22 R.Time:31.308(Scan#:3458) MassPeaks:5
RawMode:Averaged 31.300-31.317(3457-3459) BasePeak:93.05(1214)
BG Mode:None Group 1 - Event 1 Scan

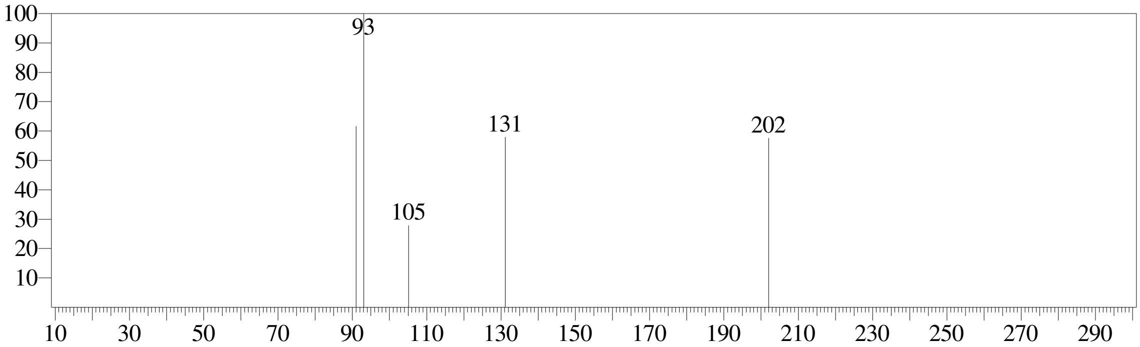

Hit#:1 Entry:3165 Library:NIST23-1.lib
SI:69 Formula:C8H12 CAS:0-00-0 MolWeight:108 RetIndex:870
CompName:Spiro[bicyclo[3.1.0]hexane-2,1'-cyclopropane]

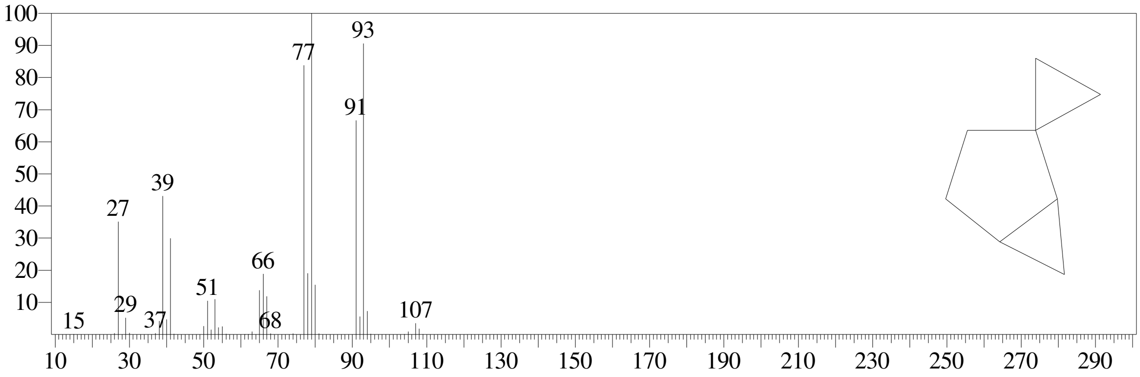

Hit#:2 Entry:174557 Library:NIST23-1.lib
SI:69 Formula:C19H19NO2 CAS:0-00-0 MolWeight:293 RetIndex:2270
CompName:Carbonic acid, monoamide, N-benzyl-N-phenethyl-, propargyl ester

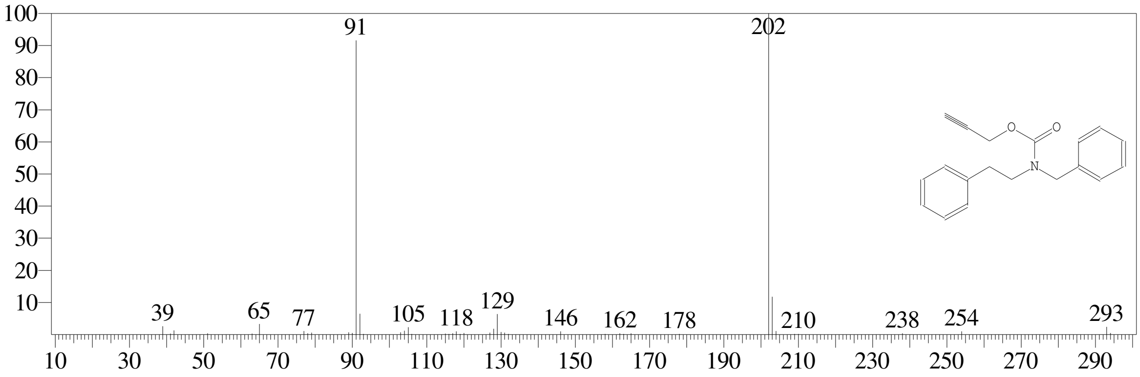

<< Target >>
Line#:22 R.Time:31.308(Scan#:3458) MassPeaks:5
RawMode:Averaged 31.300-31.317(3457-3459) BasePeak:93.05(1214)
BG Mode:None Group 1 - Event 1 Scan

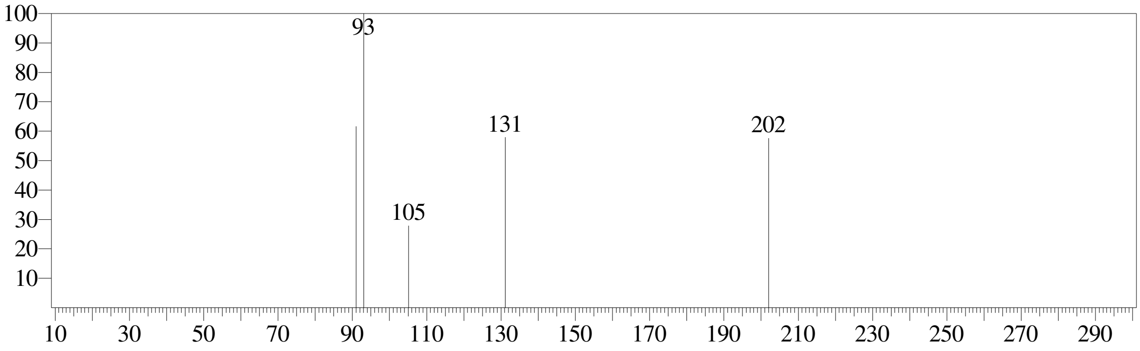

Hit#:3 Entry:3188 Library:NIST23-1.lib
SI:68 Formula:C8H12 CAS:61142-26-5 MolWeight:108 RetIndex:813
CompName:Cyclopropene, 1-methyl-3-(2-methylcyclopropyl)- $$ 1-Methyl-3-(2-methylcyclopropyl)-1-cyclopropene # $

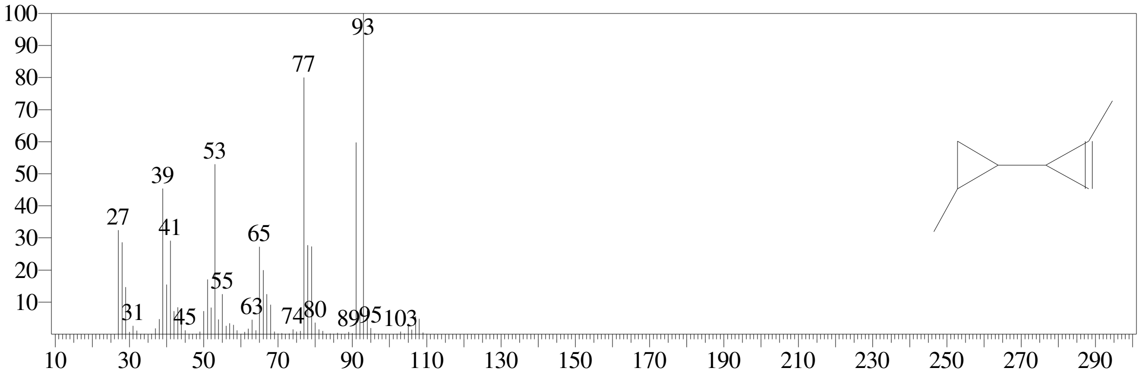

Hit#:4 Entry:3156 Library:NIST23-1.lib
SI:68 Formula:C8H12 CAS:25399-32-0 MolWeight:108 RetIndex:859
CompName:Dispiro[2.1.2.1]octane

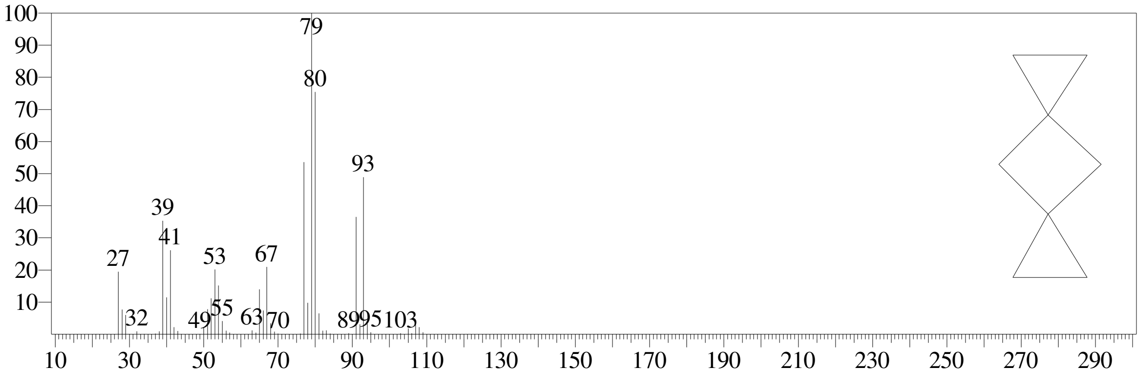

<< Target >>
Line#:22   R.Time:31.308(Scan#:3458) MassPeaks:5
RawMode:Averaged 31.300-31.317(3457-3459) BasePeak:93.05(1214)
BG Mode:None Group 1 - Event 1 Scan

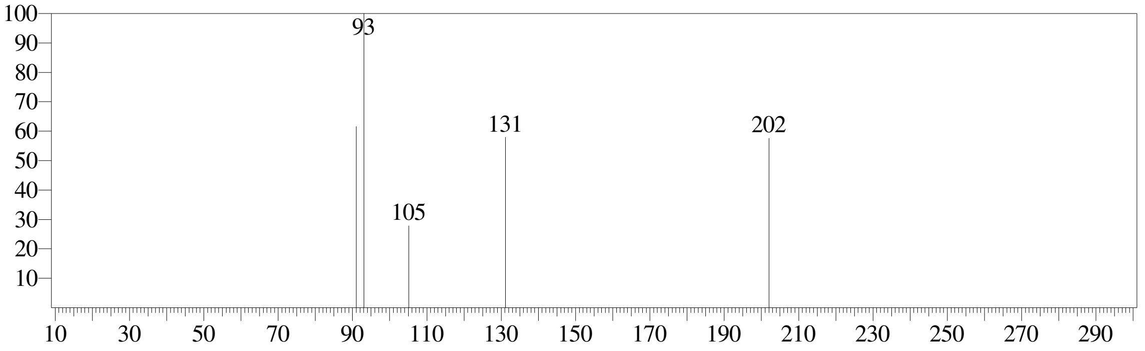

Hit#:5 Entry:86979 Library:NIST23-1.lib
SI:68 Formula:C15H15NO CAS:3271-81-6 MolWeight:225 RetIndex:2098
CompName:Hydrocinnamanilide $$ N,3-Diphenylpropanamide # $$

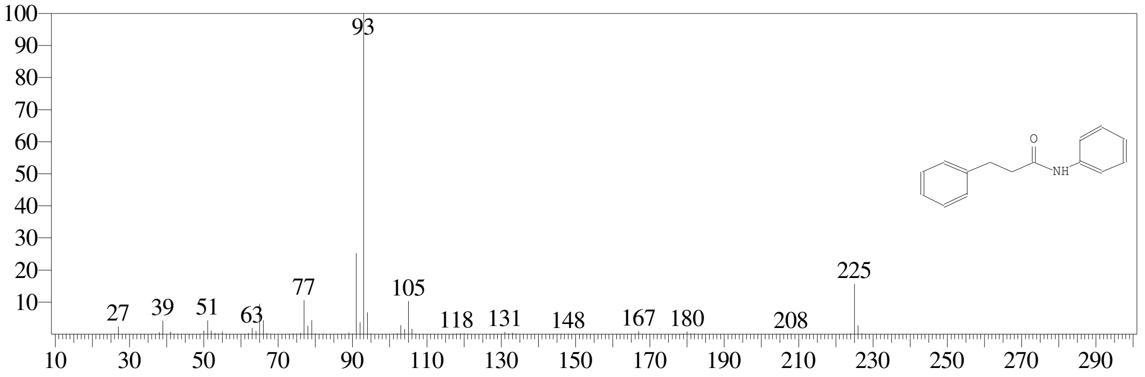

<< Target >>
Line#:23 R.Time:32.467(Scan#:3597) MassPeaks:9
RawMode:Averaged 32.458-32.475(3596-3598) BasePeak:43.00(2469)
BG Mode:None Group 1 - Event 1 Scan

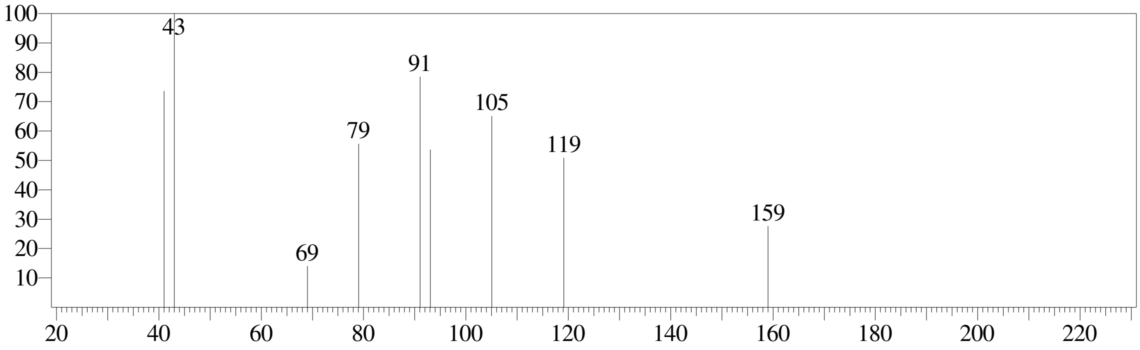

Hit#:1 Entry:50762 Library:NIST23-1.lib
SI:67 Formula:C13H20O CAS:163886-28-0 MolWeight:192 RetIndex:1472
CompName:(+)-3-Carene, 10-(acetylmethyl)- $$ 4-(7,7-Dimethylbicyclo[4.1.0]hept-3-en-3-yl)-2-butanone # $$

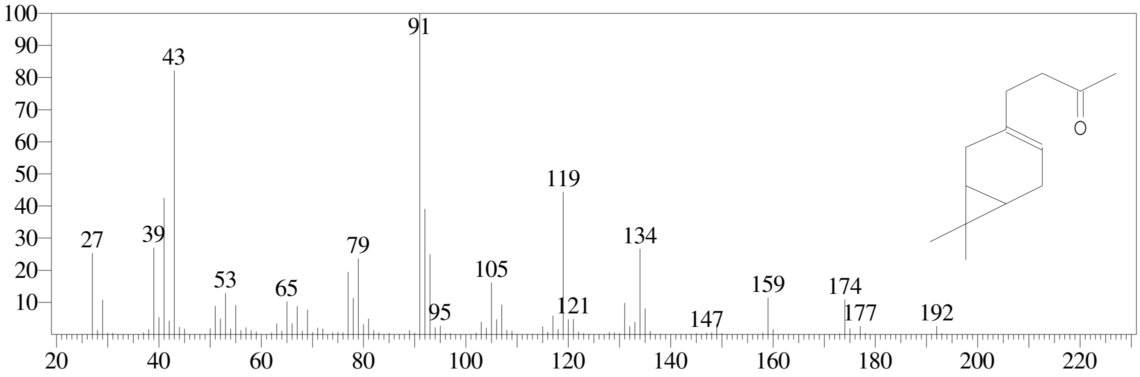

Hit#:2 Entry:50761 Library:NIST23-1.lib
SI:67 Formula:C13H20O CAS:0-00-0 MolWeight:192 RetIndex:1437
CompName:.alpha.-Pinene, 10-(2-oxopropyl)- $$ 4-(6,6-Dimethylbicyclo[3.1.1]hept-2-en-2-yl)-2-butanone # $$

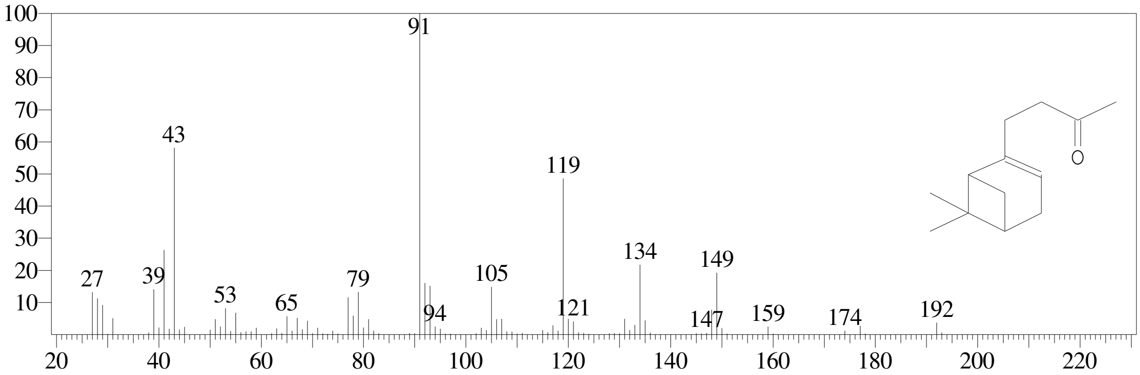

<< Target >>
Line#:23   R.Time:32.467(Scan#:3597) MassPeaks:9
RawMode:Averaged 32.458-32.475(3596-3598) BasePeak:43.00(2469)
BG Mode:None Group 1 - Event 1 Scan

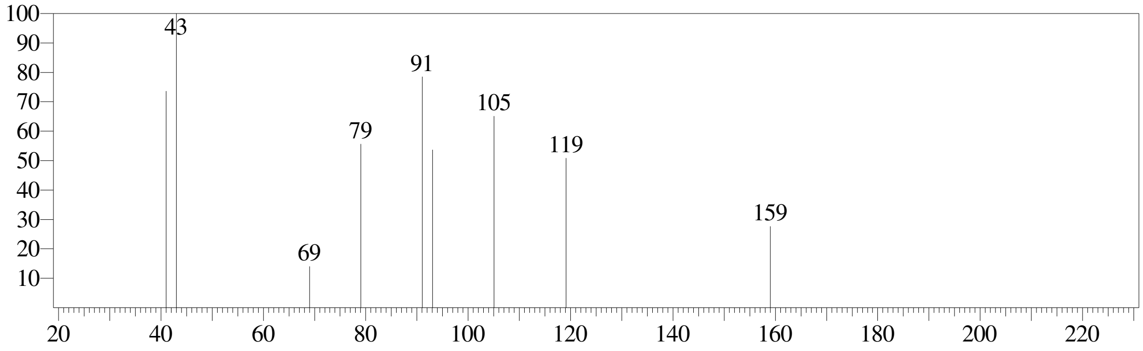

Hit#:3 Entry:38526 Library:NIST23-1.lib
SI:67 Formula:C12H18O CAS:0-00-0 MolWeight:178 RetIndex:1282
CompName:3-Carene, 2-acetyl- $$ 1-(3,7,7-Trimethylbicyclo[4.1.0]hept-3-en-2-yl)ethanone # $$

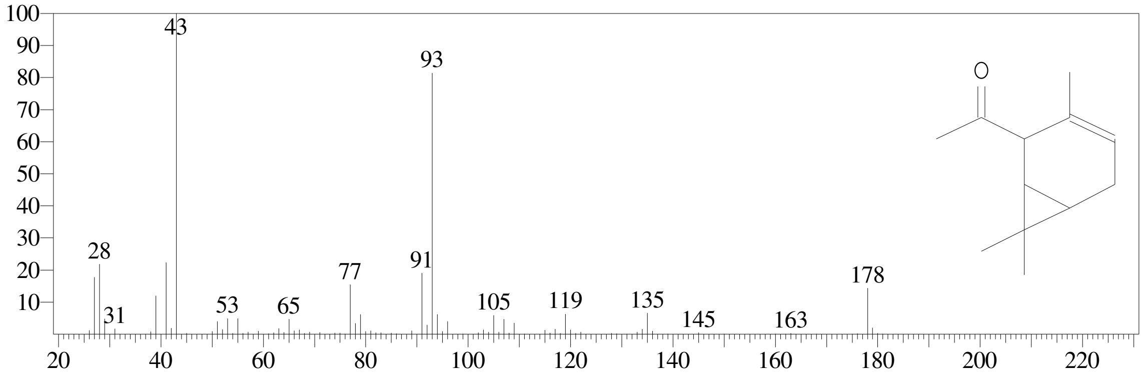

Hit#:4 Entry:52709 Library:NIST23-1.lib
SI:66 Formula:C12H18O2 CAS:73366-18-4 MolWeight:194 RetIndex:1292
CompName:Pinocarvyl acetate, cis-

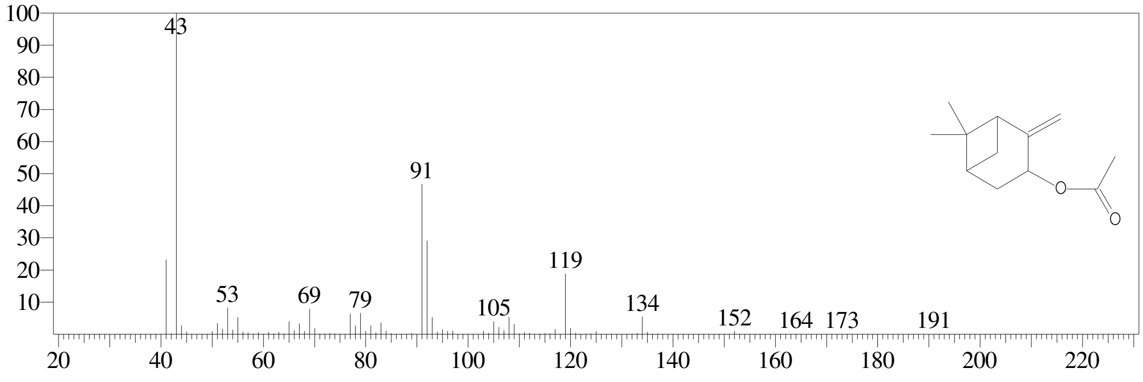

<< Target >>
Line#:23   R.Time:32.467(Scan#:3597) MassPeaks:9
RawMode:Averaged 32.458-32.475(3596-3598) BasePeak:43.00(2469)
BG Mode:None Group 1 - Event 1 Scan

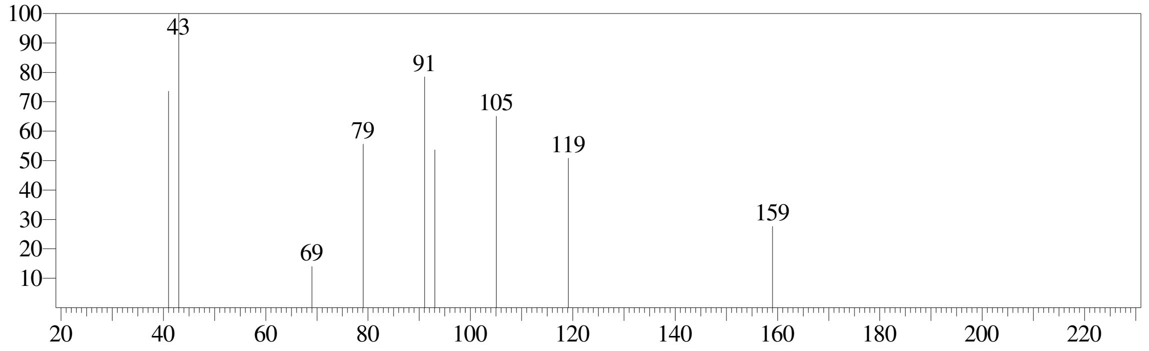

Hit#:5 Entry:83254 Library:NIST23-1.lib
SI:66 Formula:C14H22O2 CAS:74810-39-2 MolWeight:222 RetIndex:1427
CompName:Tricyclo[5.1.0.0(2,4)]octane-5-carboxylic acid, 3,3,8,8-tetramethyl-, methyl ester

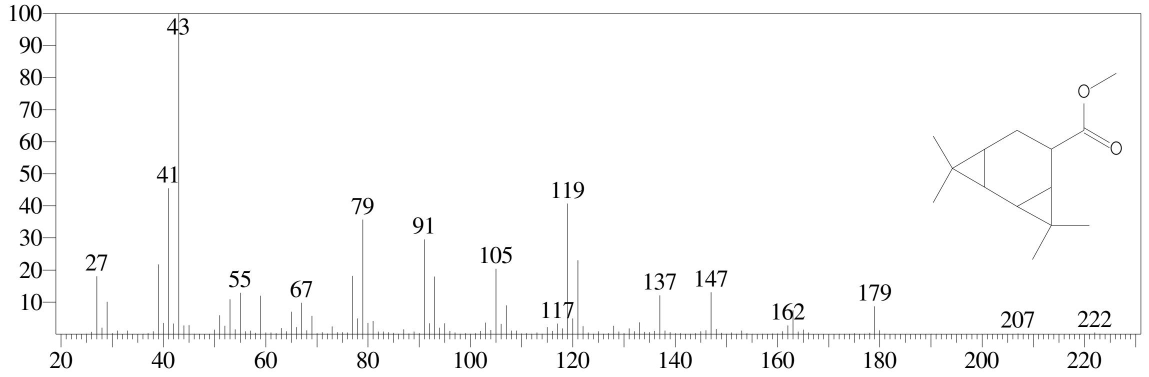

<< Target >>
Line#:24 R.Time:32.708(Scan#:3626) MassPeaks:12
RawMode:Averaged 32.700-32.717(3625-3627) BasePeak:43.00(2248)
BG Mode:None Group 1 - Event 1 Scan

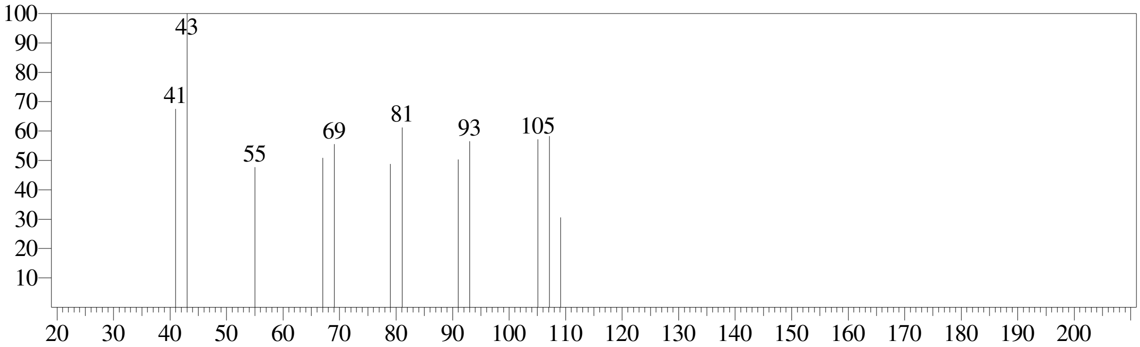

Hit#:1 Entry:30459 Library:NIST23-1.lib
SI:76 Formula:C10H16O2 CAS:0-00-0 MolWeight:168 RetIndex:1351
CompName:(2S,4R)-p-Mentha-[1(7),8]-diene 2-hydroperoxide $$ 5-Isopropenyl-2-methylenecyclohexyl hydroperoxide #

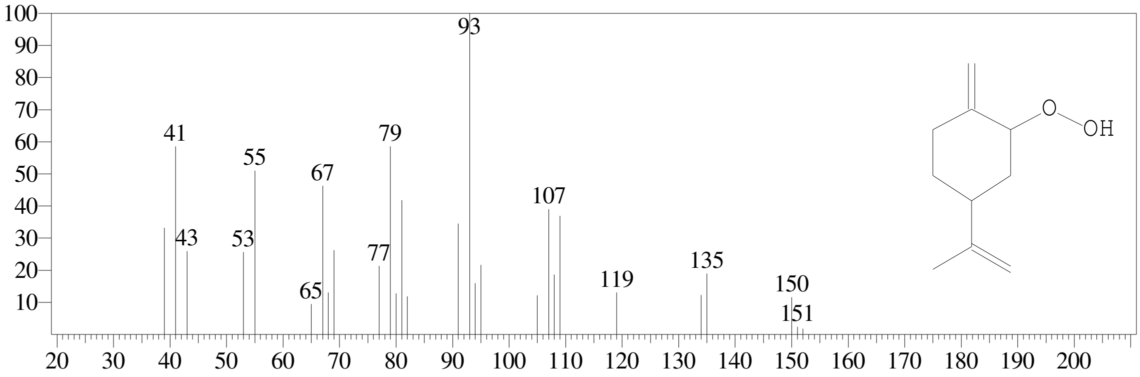

Hit#:2 Entry:27627 Library:NIST23-1.lib
SI:74 Formula:C12H20 CAS:0-00-0 MolWeight:164 RetIndex:1080
CompName:1-Pentene, 5-(2,2-dimethylcyclopropyl)-2-methyl-4-methylene- $$ 1,1-Dimethyl-2-(4-methyl-2-methylene-4-

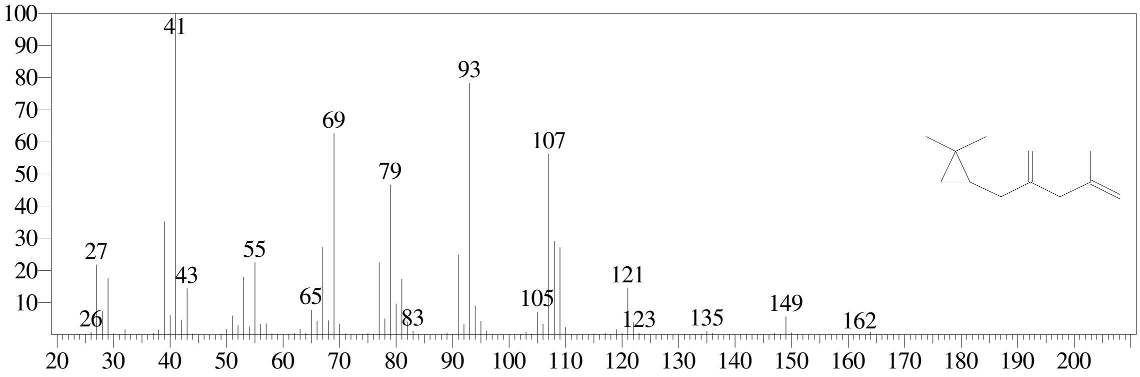

<< Target >>
Line#:24 R.Time:32.708(Scan#:3626) MassPeaks:12
RawMode:Averaged 32.700-32.717(3625-3627) BasePeak:43.00(2248)
BG Mode:None Group 1 - Event 1 Scan

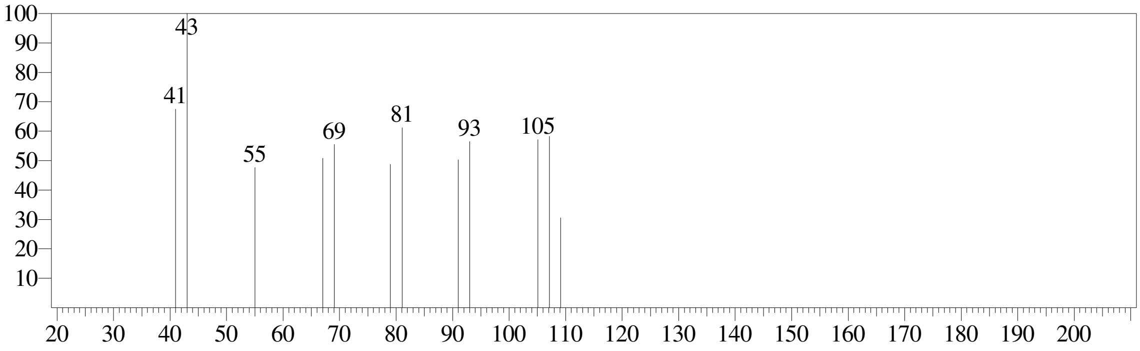

Hit#:3 Entry:50740 Library:NIST23-1.lib
SI:73 Formula:C13H20O CAS:63922-50-9 MolWeight:192 RetIndex:1472
CompName:3,4-Heptadien-2-one, 3-cyclopentyl-6-methyl- $$ 3-Cyclopentyl-6-methyl-3,4-heptadien-2-one # $$

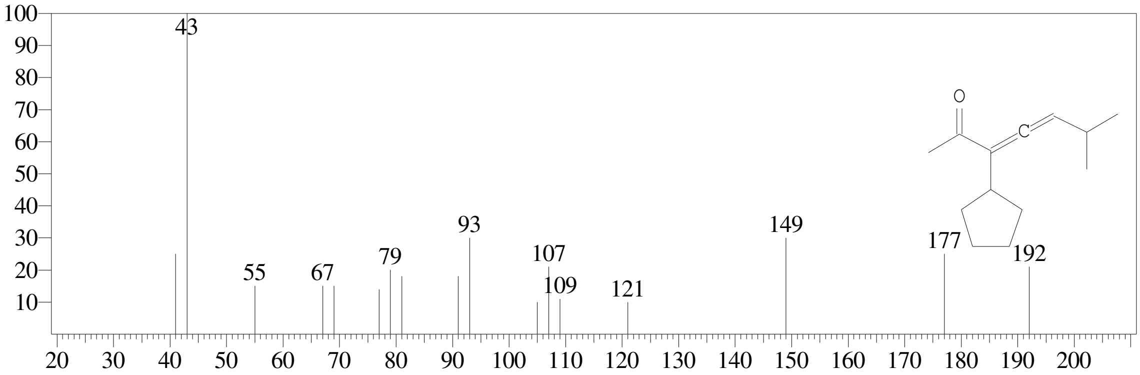

Hit#:4 Entry:24823 Library:NIST23s.lib
SI:73 Formula:C15H24 CAS:26560-14-5 MolWeight:204 RetIndex:1509
CompName:1,3,6,10-Dodecatetraene, 3,7,11-trimethyl-, (Z,E)- $$ (Z,E)-.alpha.-Farnesene $$ (3Z,6E)-3,7,11-Trimethyl-1.

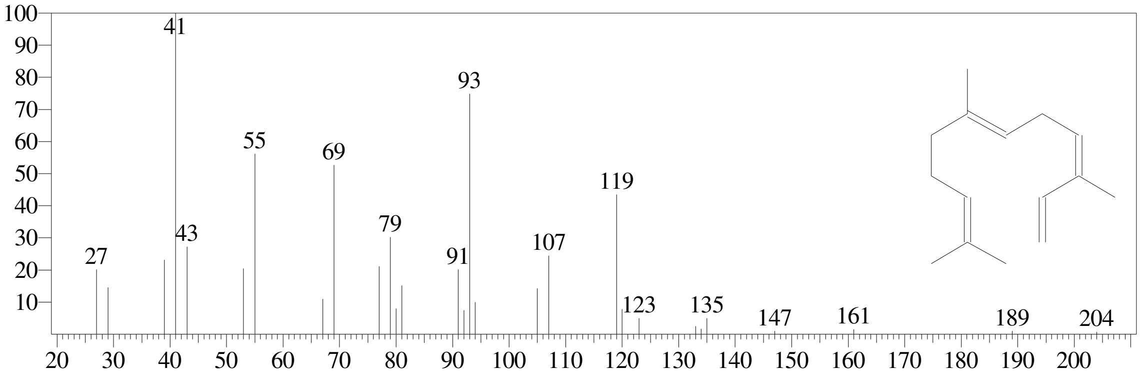

<< Target >>
Line#:24   R.Time:32.708(Scan#:3626) MassPeaks:12
RawMode:Averaged 32.700-32.717(3625-3627) BasePeak:43.00(2248)
BG Mode:None Group 1 - Event 1 Scan

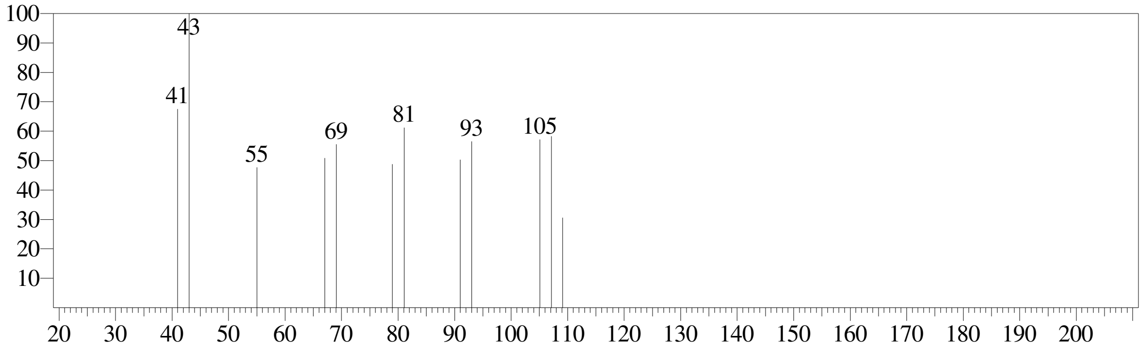

Hit#:5 Entry:28692 Library:NIST23s.lib
SI:72 Formula:C15H26O CAS:142-50-7 MolWeight:222 RetIndex:1574
CompName:Nerolidol, cis- $$ 1,6,10-Dodecatrien-3-ol, 3,7,11-trimethyl-, [S-(Z)]- $$ 1,6,10-Dodecatrien-3-ol, 3,7,11-trim

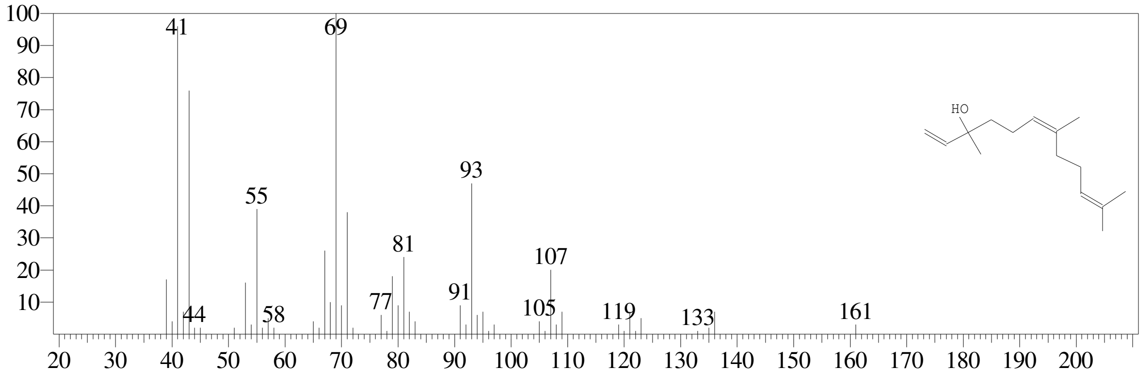

<< Target >>
Line#:25 R.Time:33.225(Scan#:3688) MassPeaks:12
RawMode:Averaged 33.217-33.233(3687-3689) BasePeak:93.05(15667)
BG Mode:None Group 1 - Event 1 Scan

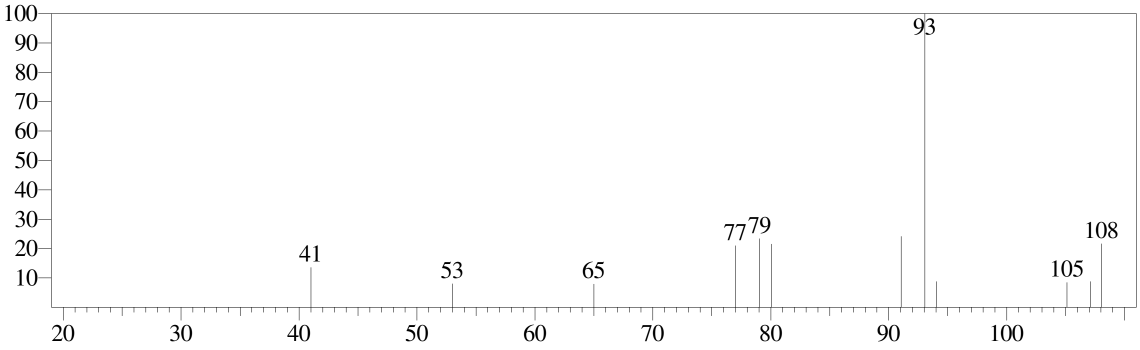

Hit#:1 Entry:3206 Library:NIST23-1.lib
SI:84 Formula:C8H12 CAS:62338-00-5 MolWeight:108 RetIndex:892
CompName:Cyclopentene, 3-ethylidene-1-methyl- $$ (3E)-3-Ethylidene-1-methyl-1-cyclopentene # $$

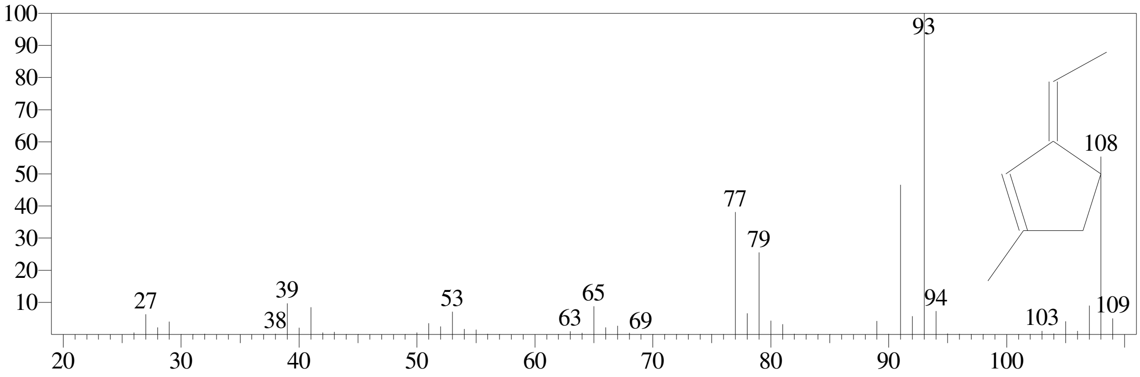

Hit#:2 Entry:3198 Library:NIST23-1.lib
SI:83 Formula:C8H12 CAS:34564-56-2 MolWeight:108 RetIndex:889
CompName:3-Methylenecycloheptene $$ 3-Methylene-1-cycloheptene # $$

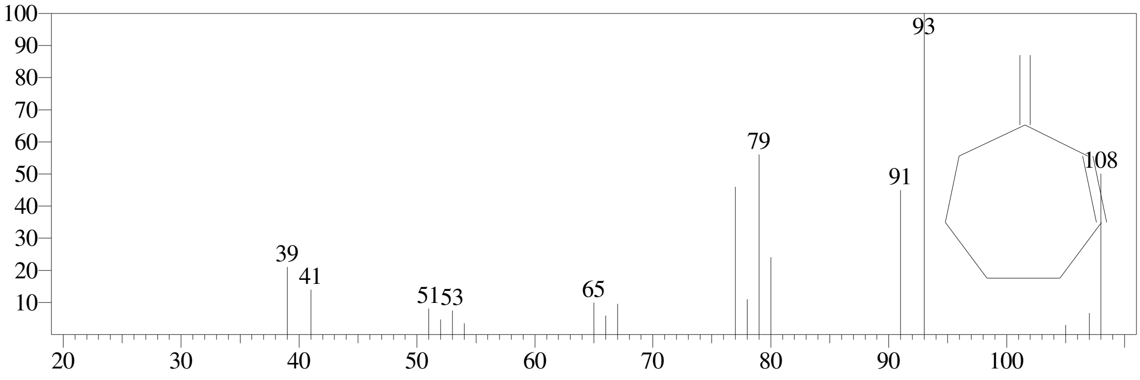

<< Target >>
Line#:25   R.Time:33.225(Scan#:3688) MassPeaks:12
RawMode:Averaged 33.217-33.233(3687-3689) BasePeak:93.05(15667)
BG Mode:None Group 1 - Event 1 Scan

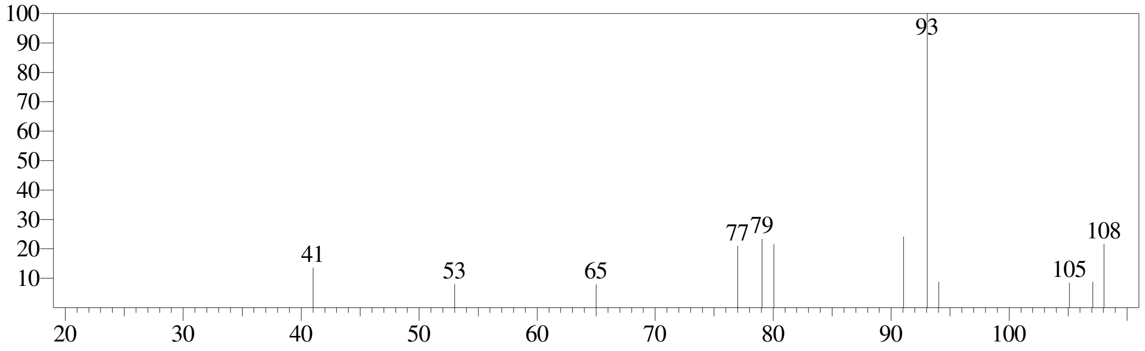

Hit#:3 Entry:3200 Library:NIST23-1.lib
SI:83 Formula:C8H12 CAS:5715-27-5 MolWeight:108 RetIndex:816
CompName:1,3-Cyclohexadiene, 5,6-dimethyl- $$ 5,6-Dimethyl-1,3-cyclohexadiene # $$

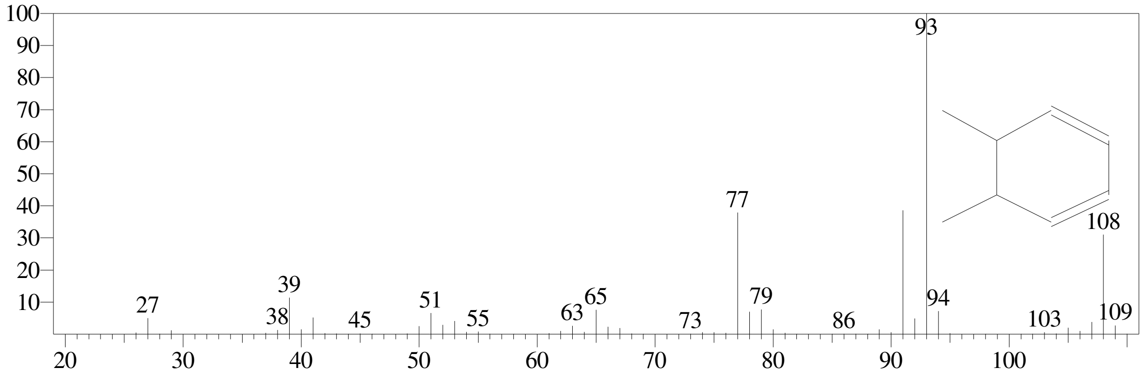

Hit#:4 Entry:3199 Library:NIST23-1.lib
SI:82 Formula:C8H12 CAS:4249-09-6 MolWeight:108 RetIndex:766
CompName:Cyclopentadiene, 1,5,5-trimethyl- $$ 1,5,5-Trimethylcyclopentadiene $$

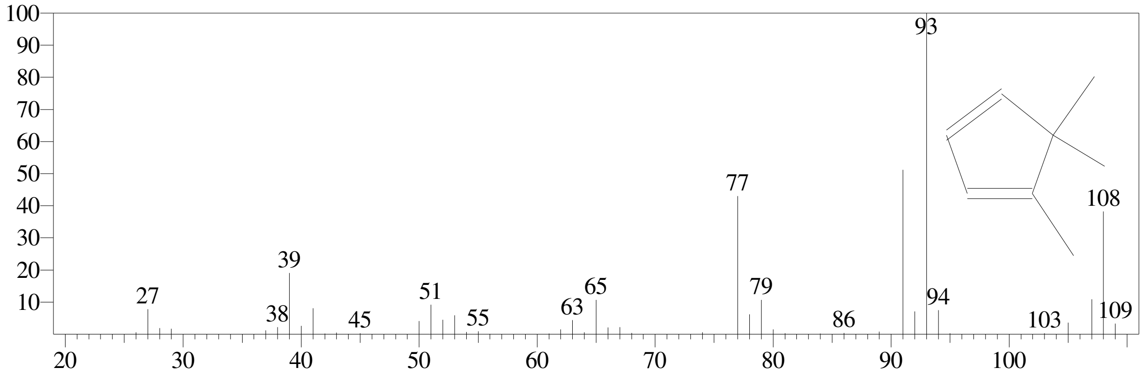

<< Target >>
Line#:25 R.Time:33.225(Scan#:3688) MassPeaks:12
RawMode:Averaged 33.217-33.233(3687-3689) BasePeak:93.05(15667)
BG Mode:None Group 1 - Event 1 Scan

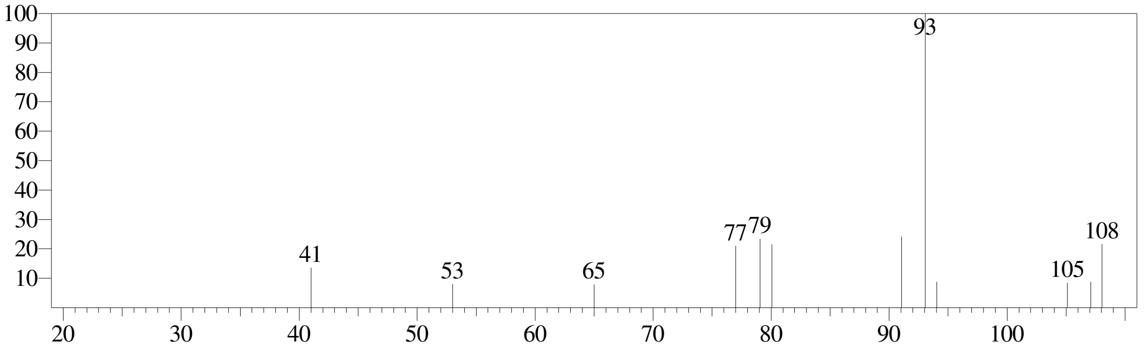

Hit#:5 Entry:3201 Library:NIST23-1.lib
SI:82 Formula:C8H12 CAS:7086-15-9 MolWeight:108 RetIndex:750
CompName:Cyclopentadiene, 2,5,5-trimethyl- $$ 2,5,5-Trimethyl-1,3-cyclopentadiene # $$

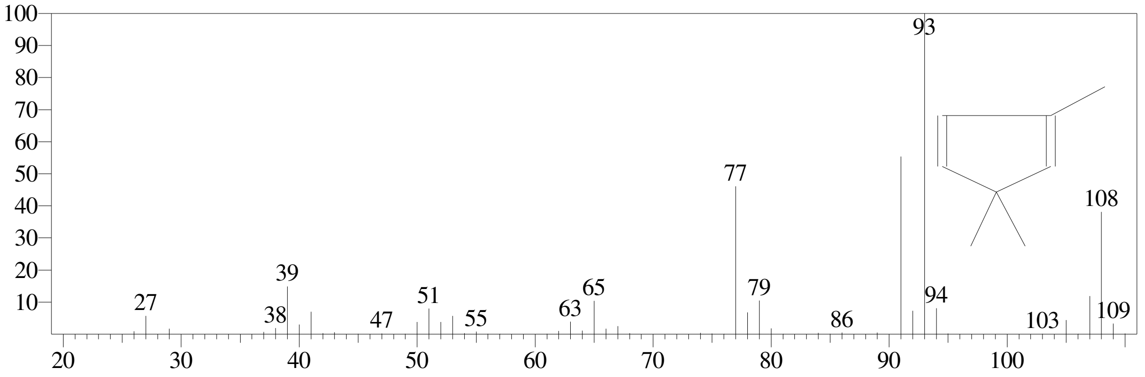

<< Target >>
Line#:26   R.Time:35.458(Scan#:3956) MassPeaks:57
RawMode:Averaged 35.450-35.467(3955-3957) BasePeak:108.05(60701)
BG Mode:Calc. from Peak Group 1 - Event 1 Scan

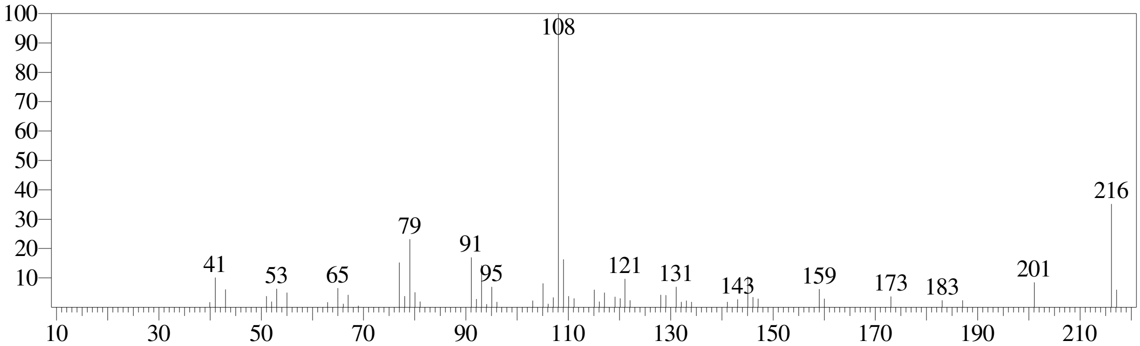

Hit#:1 Entry:76134 Library:NIST23-1.lib
SI:92 Formula:C15H20O CAS:6989-21-5 MolWeight:216 RetIndex:1653
CompName:(4aS,8aR)-3,8a-Dimethyl-5-methylene-4,4a,5,6,7,8,8a,9-octahydronaphtho[2,3-b]furan $$ Naphtho[2,3-b]fura

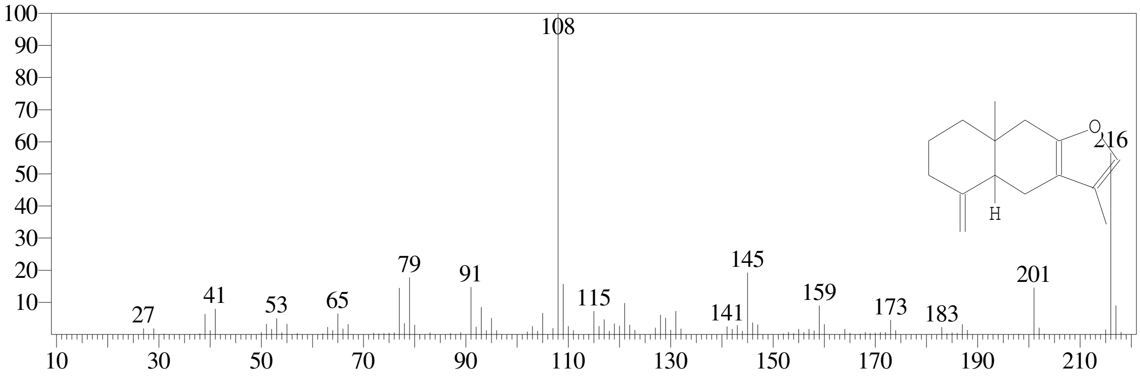

Hit#:2 Entry:27473 Library:NIST23s.lib
SI:90 Formula:C15H20O CAS:6989-21-5 MolWeight:216 RetIndex:1653
CompName:(4aS,8aR)-3,8a-Dimethyl-5-methylene-4,4a,5,6,7,8,8a,9-octahydronaphtho[2,3-b]furan $$ Naphtho[2,3-b]fura

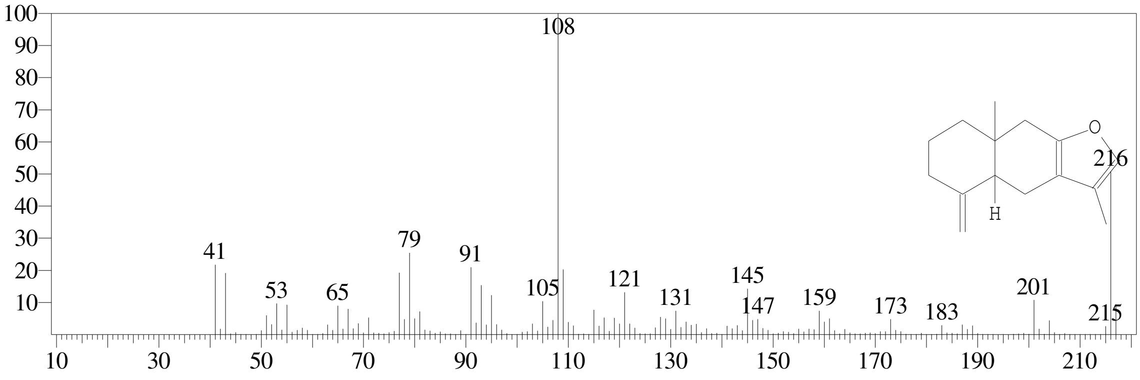

<< Target >>
Line#:26 R.Time:35.458(Scan#:3956) MassPeaks:57
RawMode:Averaged 35.450-35.467(3955-3957) BasePeak:108.05(60701)
BG Mode:Calc. from Peak Group 1 - Event 1 Scan

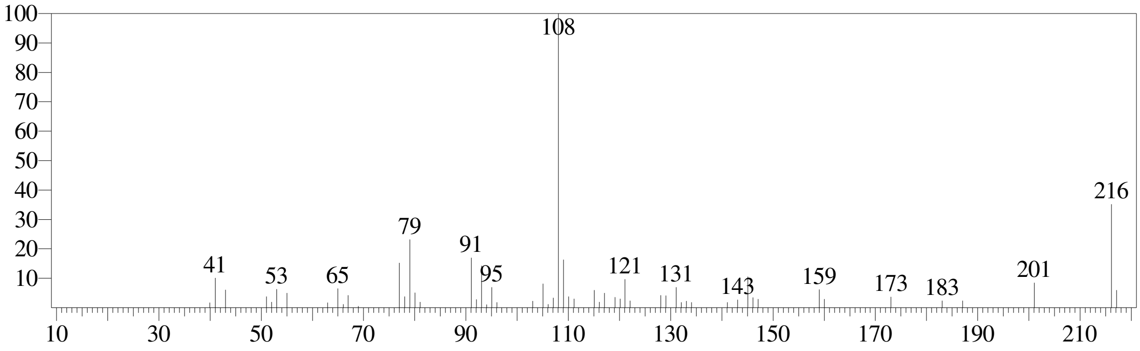

Hit#:3 Entry:27472 Library:NIST23s.lib
SI:86 Formula:C15H20O CAS:17910-09-7 MolWeight:216 RetIndex:1524
CompName:Benzofuran, 6-ethenyl-4,5,6,7-tetrahydro-3,6-dimethyl-5-isopropenyl-, trans- $$ 5-Isopropenyl-3,6-dimethyl-

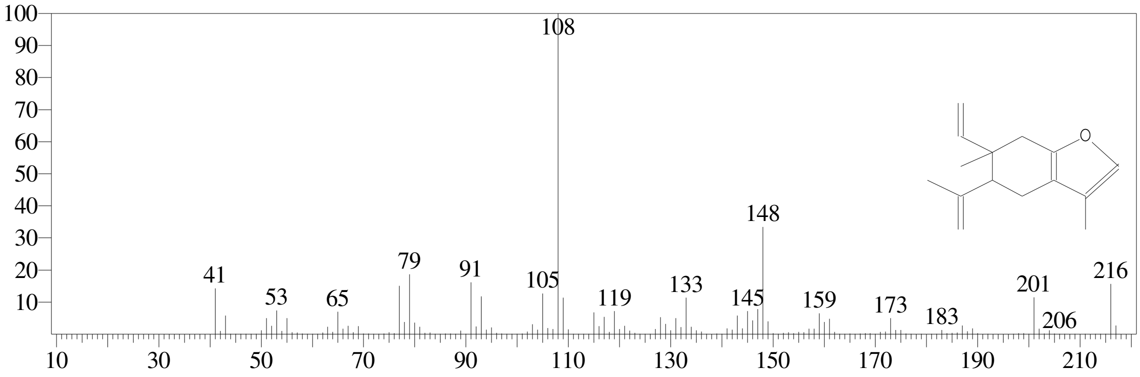

Hit#:4 Entry:27471 Library:NIST23s.lib
SI:84 Formula:C15H20O CAS:17910-09-7 MolWeight:216 RetIndex:1524
CompName:Benzofuran, 6-ethenyl-4,5,6,7-tetrahydro-3,6-dimethyl-5-isopropenyl-, trans- $$ 5-Isopropenyl-3,6-dimethyl-

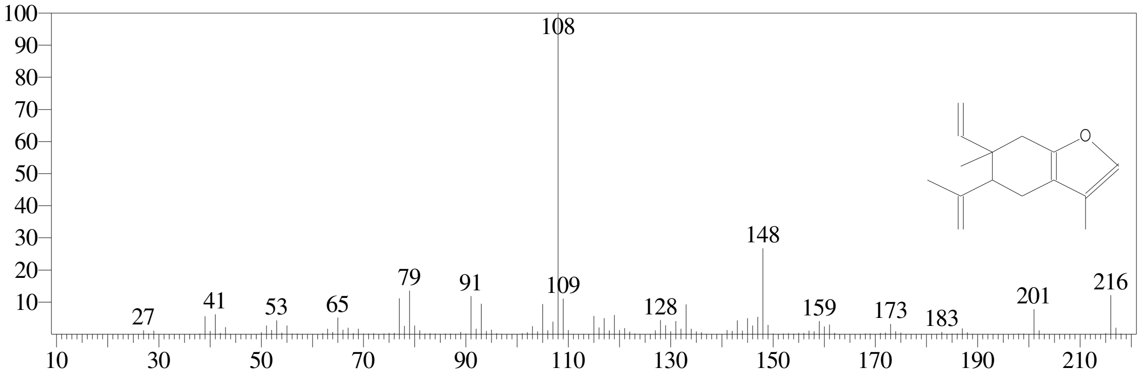

<< Target >>
Line#:26   R.Time:35.458(Scan#:3956) MassPeaks:57
RawMode:Averaged 35.450-35.467(3955-3957) BasePeak:108.05(60701)
BG Mode:Calc. from Peak Group 1 - Event 1 Scan

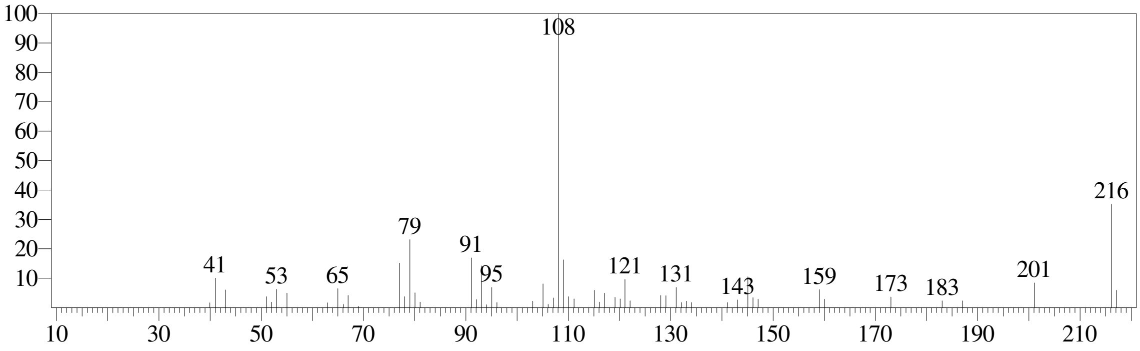

Hit#:5 Entry:76133 Library:NIST23-1.lib
SI:82 Formula:C15H20O CAS:17910-09-7 MolWeight:216 RetIndex:1524
CompName:Benzofuran, 6-ethenyl-4,5,6,7-tetrahydro-3,6-dimethyl-5-isopropenyl-, trans- $$ 5-Isopropenyl-3,6-dimethyl-6

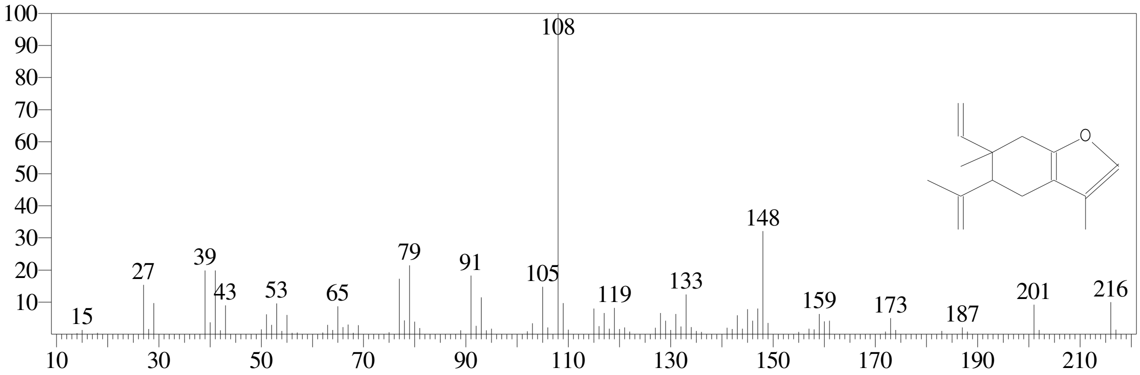

<< Target >>
Line#:27 R.Time:35.625(Scan#:3976) MassPeaks:38
RawMode:Averaged 35.617-35.633(3975-3977) BasePeak:108.05(26326)
BG Mode:Calc. from Peak Group 1 - Event 1 Scan

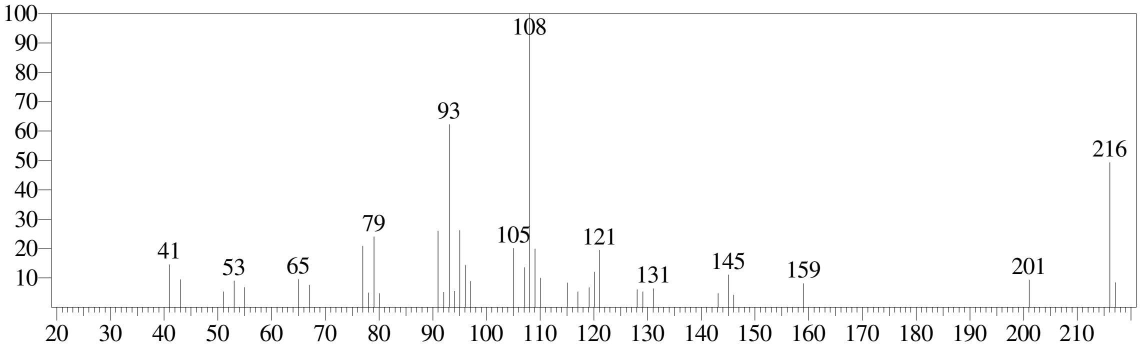

Hit#:1 Entry:27473 Library:NIST23s.lib
SI:87 Formula:C15H20O CAS:6989-21-5 MolWeight:216 RetIndex:1653
CompName:(4aS,8aR)-3,8a-Dimethyl-5-methylene-4,4a,5,6,7,8,8a,9-octahydronaphtho[2,3-b]furan $$ Naphtho[2,3-b]fur

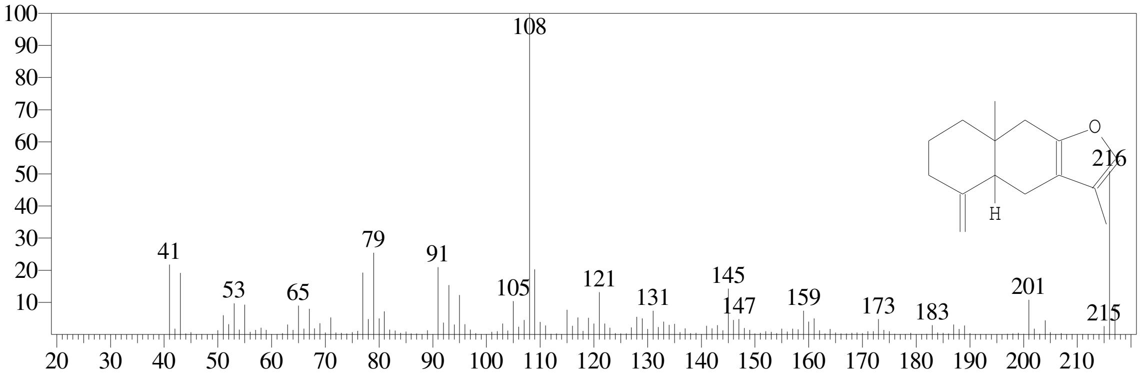

Hit#:2 Entry:76134 Library:NIST23-1.lib
SI:82 Formula:C15H20O CAS:6989-21-5 MolWeight:216 RetIndex:1653
CompName:(4aS,8aR)-3,8a-Dimethyl-5-methylene-4,4a,5,6,7,8,8a,9-octahydronaphtho[2,3-b]furan $$ Naphtho[2,3-b]fur

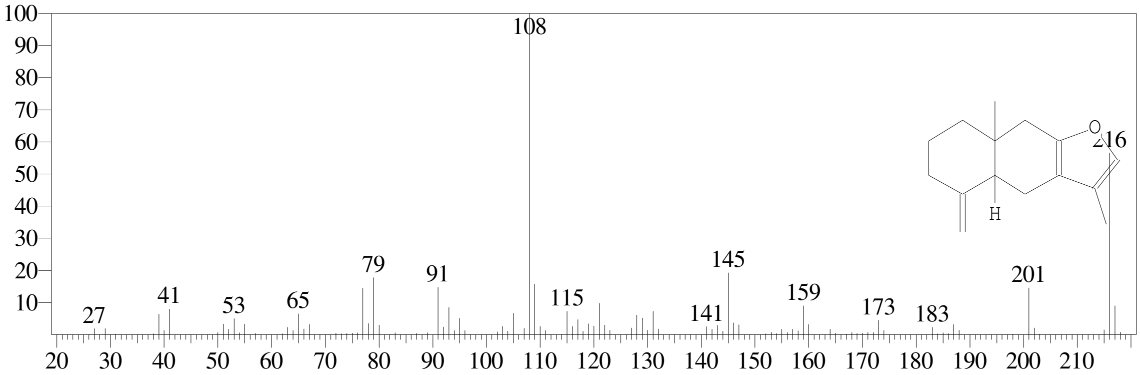

<< Target >>
Line#:27 R.Time:35.625(Scan#:3976) MassPeaks:38
RawMode:Averaged 35.617-35.633(3975-3977) BasePeak:108.05(26326)
BG Mode:Calc. from Peak Group 1 - Event 1 Scan

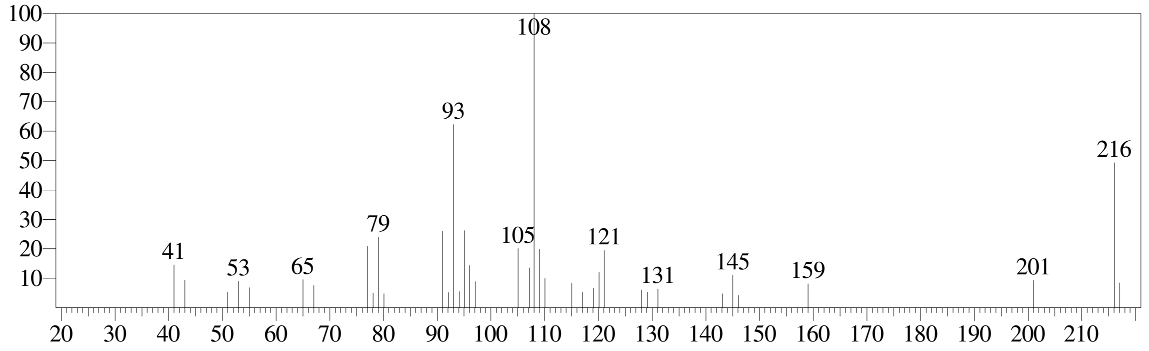

Hit#:3 Entry:12368 Library:NIST23s.lib
SI:75 Formula:C10H16O CAS:4501-58-0 MolWeight:152 RetIndex:1131
CompName:.alpha.-Campholenal $$ (R)-.alpha.-Campholene aldehyde $$ (R)-2-(2,2,3-Trimethylcyclopent-3-en-1-yl)acet

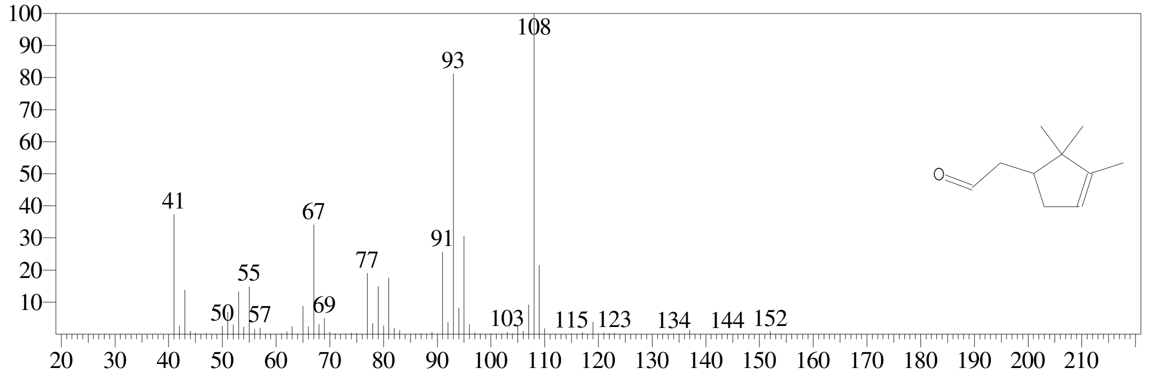

Hit#:4 Entry:27472 Library:NIST23s.lib
SI:75 Formula:C15H20O CAS:17910-09-7 MolWeight:216 RetIndex:1524
CompName:Benzofuran, 6-ethenyl-4,5,6,7-tetrahydro-3,6-dimethyl-5-isopropenyl-, trans- $$ 5-Isopropenyl-3,6-dimethyl-

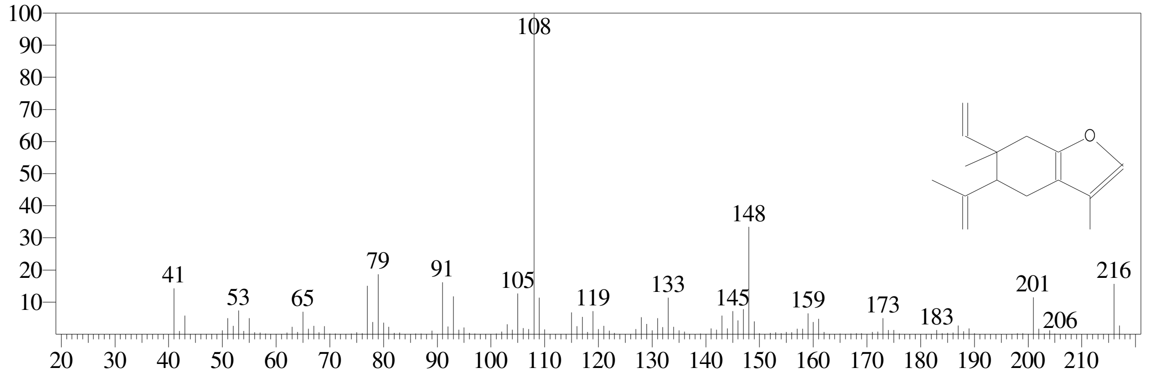

<< Target >>
Line#:27   R.Time:35.625(Scan#:3976) MassPeaks:38
RawMode:Averaged 35.617-35.633(3975-3977) BasePeak:108.05(26326)
BG Mode:Calc. from Peak Group 1 - Event 1 Scan

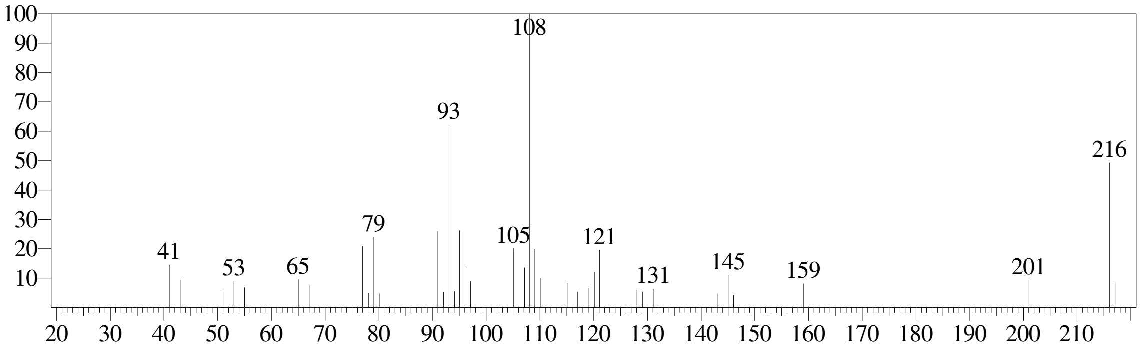

Hit#:5 Entry:12338 Library:NIST23s.lib
SI:74 Formula:C10H16O CAS:3570-04-5 MolWeight:152 RetIndex:1168
CompName:Camphenol, 6- $$ 5,5-Dimethyl-6-methylenebicyclo[2.2.1]heptan-2-ol # $$ Camphen-6-ol $$ Camphenol $$

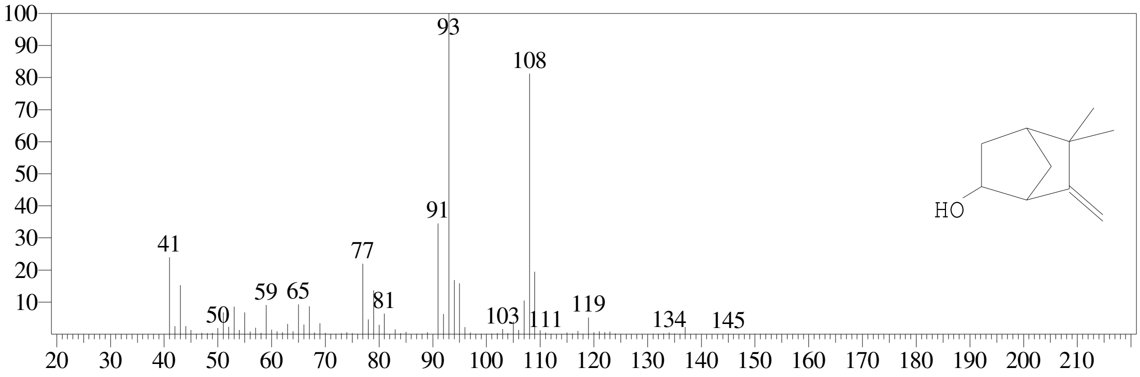

<< Target >>
Line#:28   R.Time:36.400(Scan#:4069) MassPeaks:18
RawMode:Averaged 36.392-36.408(4068-4070) BasePeak:105.05(4933)
BG Mode:None Group 1 - Event 1 Scan

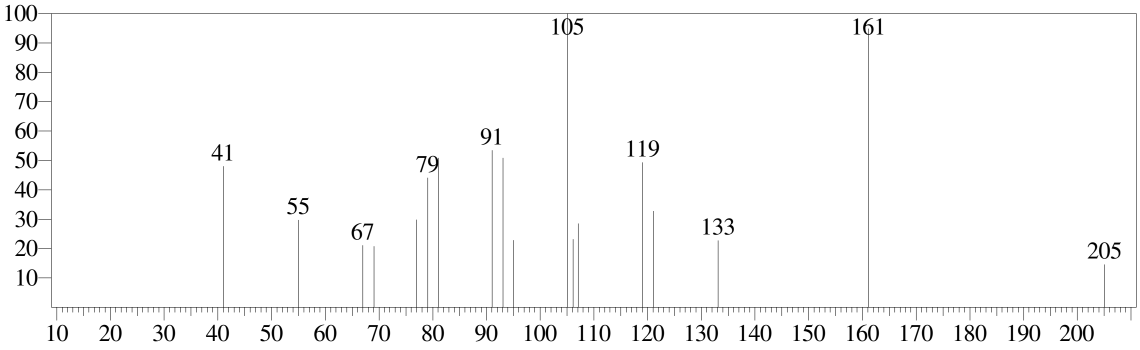

Hit#:1 Entry:62981 Library:NIST23-1.lib
SI:79 Formula:C15H24 CAS:23986-74-5 MolWeight:204 RetIndex:1478
CompName:Germacrene D $$ (S,1Z,6Z)-8-Isopropyl-1-methyl-5-methylenecyclodeca-1,6-diene $$ D-Germacrene $$ 1(1

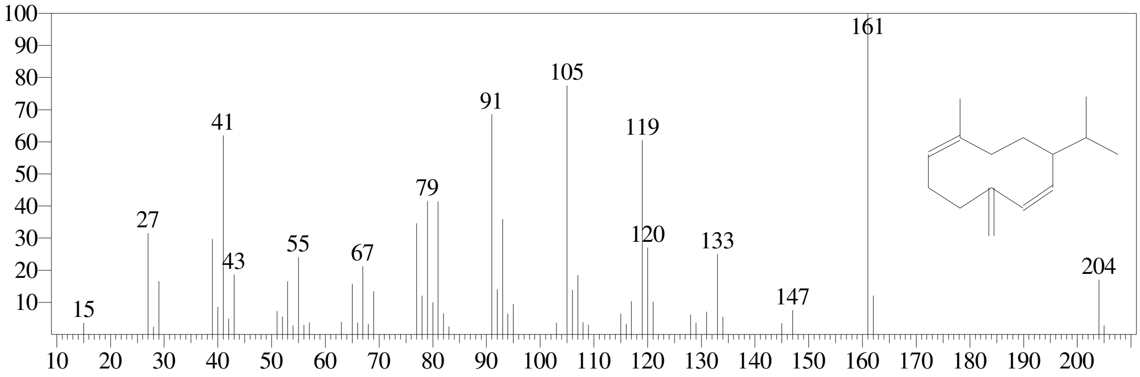

Hit#:2 Entry:25067 Library:NIST23s.lib
SI:79 Formula:C15H24 CAS:23986-74-5 MolWeight:204 RetIndex:1478
CompName:Germacrene D $$ (S,1Z,6Z)-8-Isopropyl-1-methyl-5-methylenecyclodeca-1,6-diene $$ D-Germacrene $$ 1(1

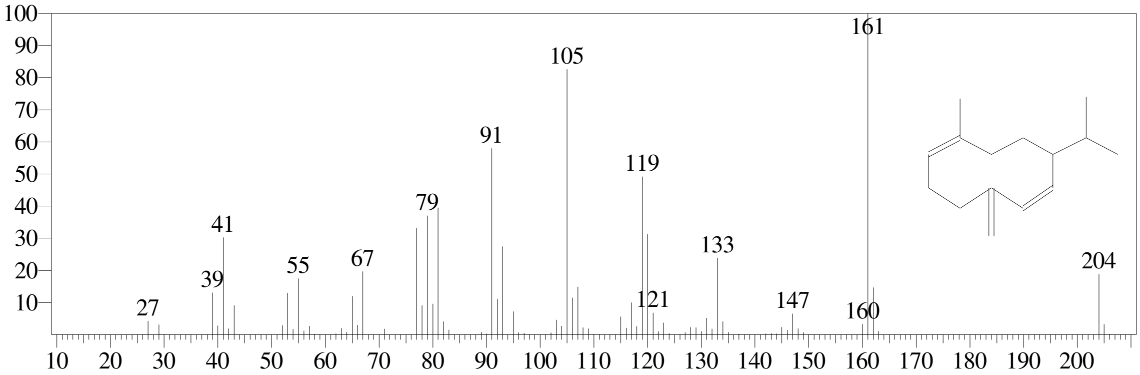

<< Target >>
Line#:28 R.Time:36.400(Scan#:4069) MassPeaks:18
RawMode:Averaged 36.392-36.408(4068-4070) BasePeak:105.05(4933)
BG Mode:None Group 1 - Event 1 Scan

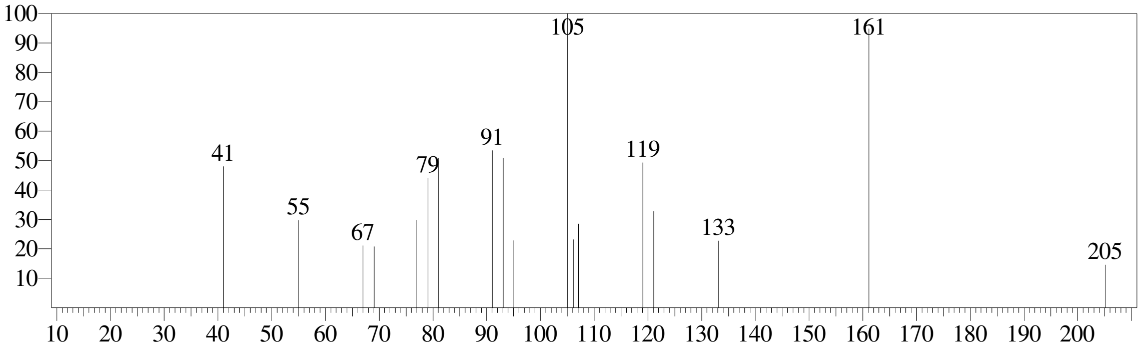

Hit#:3 Entry:25083 Library:NIST23s.lib
SI:78 Formula:C15H24 CAS:95910-36-4 MolWeight:204 RetIndex:1396
CompName:isoledene $$ 1,1,4,7-Tetramethyl-1a,2,3,4,5,6,7,7b-octahydro-1H-cyclopropa[e]azulene # $$ (1aR,4R,7R,7bS

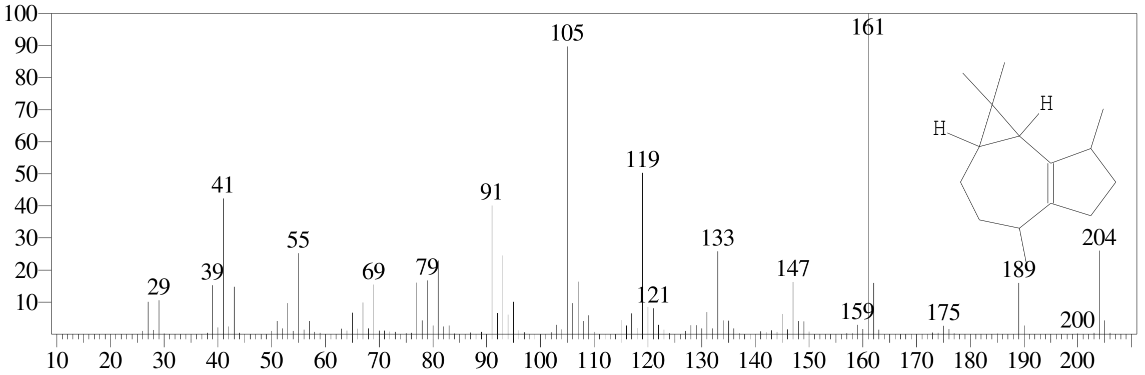

Hit#:4 Entry:25086 Library:NIST23s.lib
SI:78 Formula:C15H24 CAS:30021-74-0 MolWeight:204 RetIndex:1483
CompName:.gamma.-Muurolene $$ Naphthalene, 1,2,3,4,4a,5,6,8a-octahydro-7-methyl-4-methylene-1-(1-methylethyl)-, (

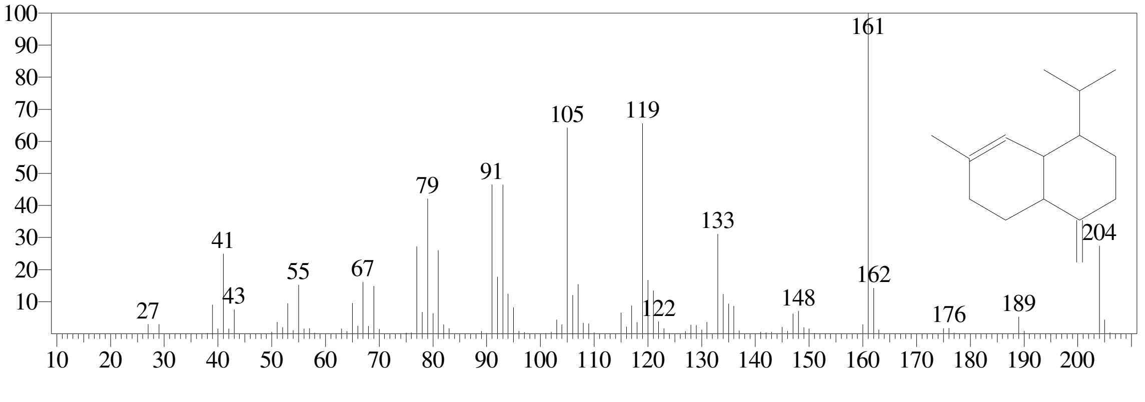

<< Target >>
Line#:28   R.Time:36.400(Scan#:4069) MassPeaks:18
RawMode:Averaged 36.392-36.408(4068-4070) BasePeak:105.05(4933)
BG Mode:None Group 1 - Event 1 Scan

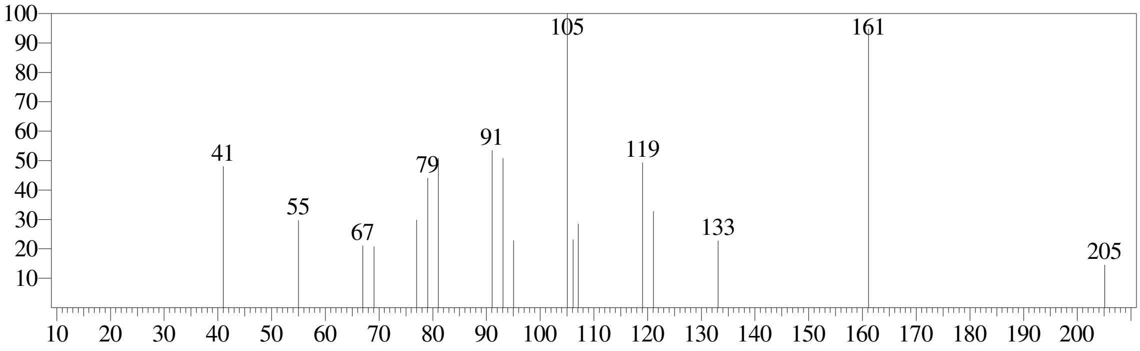

Hit#:5 Entry:62910 Library:NIST23-1.lib
SI:78 Formula:C15H24 CAS:3856-25-5 MolWeight:204 RetIndex:1407
CompName:Copaene $$ Tricyclo[4.4.0.02,7]dec-3-ene, 1,3-dimethyl-8-(1-methylethyl)-, stereoisomer $$ Tricyclo[4.4.0.0

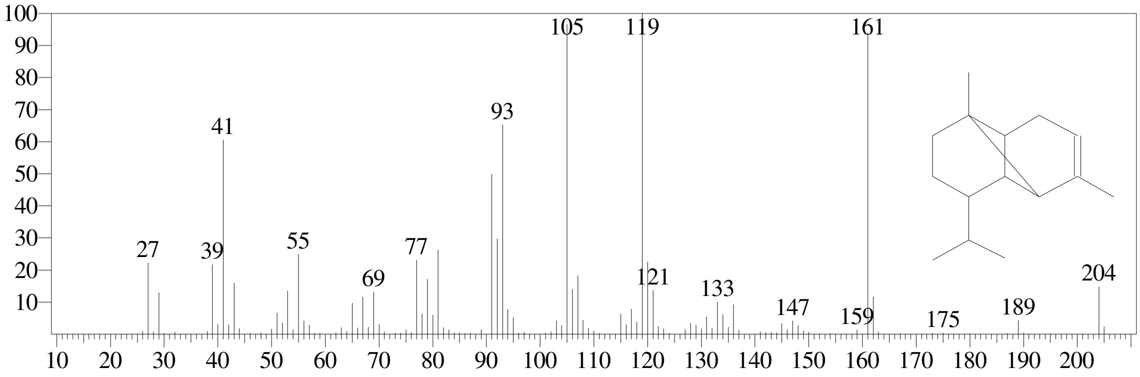

<< Target >>
Line#:29   R.Time:36.900(Scan#:4129) MassPeaks:68
RawMode:Averaged 36.892-36.908(4128-4130) BasePeak:107.10(75856)
BG Mode:None Group 1 - Event 1 Scan

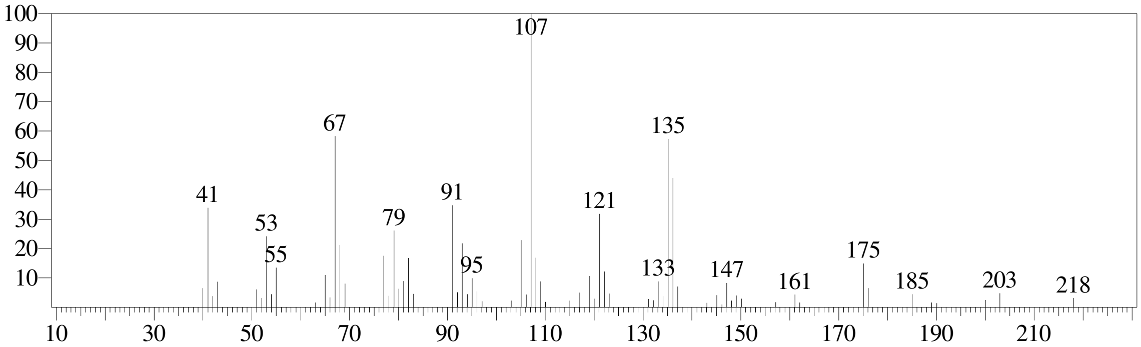

Hit#:1 Entry:27830 Library:NIST23s.lib
SI:95 Formula:C15H22O CAS:6902-91-6 MolWeight:218 RetIndex:1699
CompName:3,7-Cyclodecadien-1-one, 3,7-dimethyl-10-(1-methylethylidene)-, (E,E)- $$ Germacra-3,7(11),9-trien-6-one,

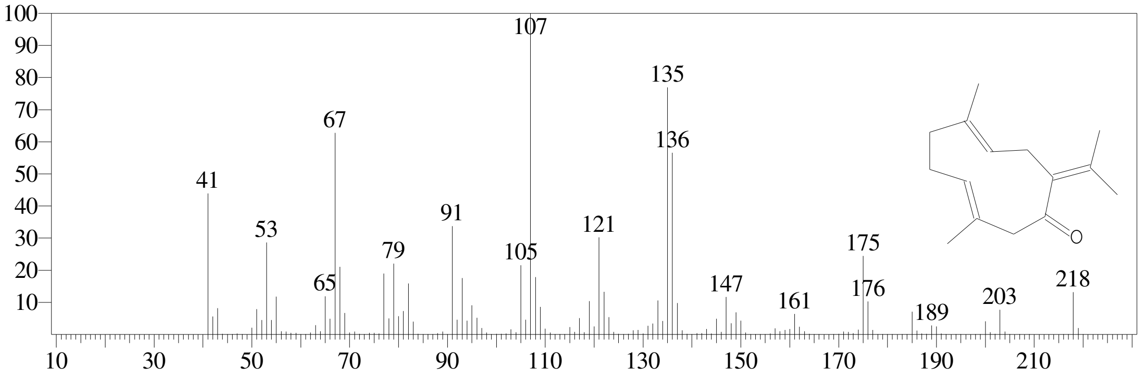

Hit#:2 Entry:27840 Library:NIST23s.lib
SI:93 Formula:C15H22O CAS:6902-91-6 MolWeight:218 RetIndex:1699
CompName:3,7-Cyclodecadien-1-one, 3,7-dimethyl-10-(1-methylethylidene)-, (E,E)- $$ Germacra-3,7(11),9-trien-6-one,

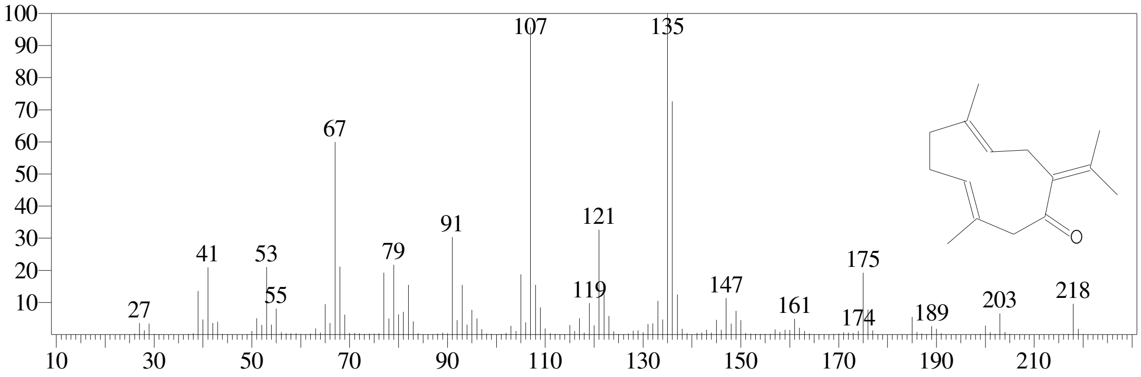

<< Target >>
Line#:29   R.Time:36.900(Scan#:4129) MassPeaks:68
RawMode:Averaged 36.892-36.908(4128-4130) BasePeak:107.10(75856)
BG Mode:None Group 1 - Event 1 Scan

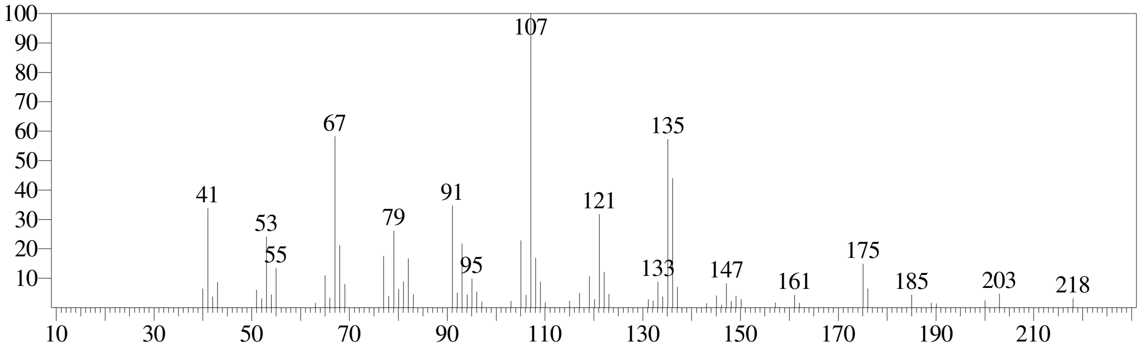

Hit#:3 Entry:27829 Library:NIST23s.lib
SI:93 Formula:C15H22O CAS:6902-91-6 MolWeight:218 RetIndex:1699
CompName:3,7-Cyclodecadien-1-one, 3,7-dimethyl-10-(1-methylethylidene)-, (E,E)- $$ Germacra-3,7(11),9-trien-6-one,

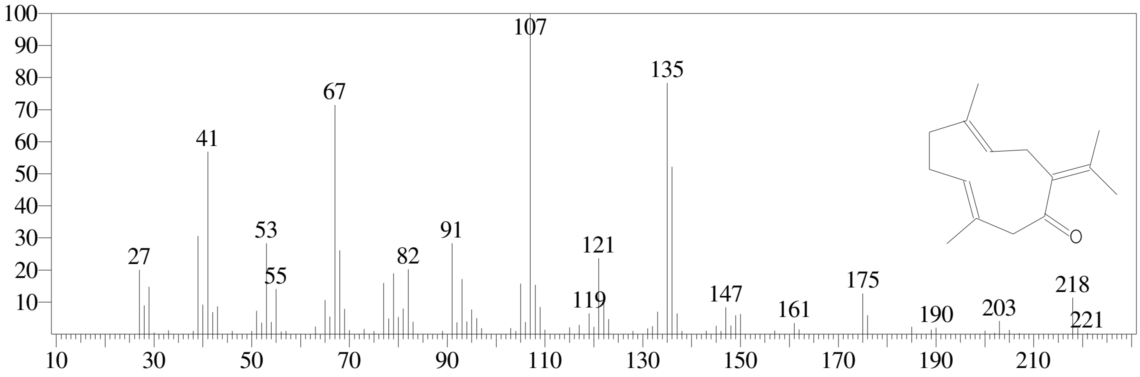

Hit#:4 Entry:78394 Library:NIST23-1.lib
SI:93 Formula:C15H22O CAS:6902-91-6 MolWeight:218 RetIndex:1699
CompName:3,7-Cyclodecadien-1-one, 3,7-dimethyl-10-(1-methylethylidene)-, (E,E)- $$ Germacra-3,7(11),9-trien-6-one,

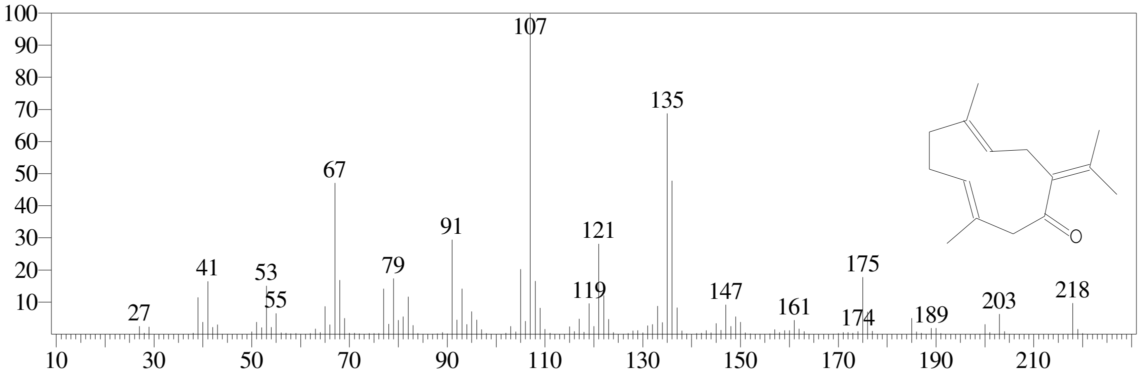

<< Target >>
Line#:29 R.Time:36.900(Scan#:4129) MassPeaks:68
RawMode:Averaged 36.892-36.908(4128-4130) BasePeak:107.10(75856)
BG Mode:None Group 1 - Event 1 Scan

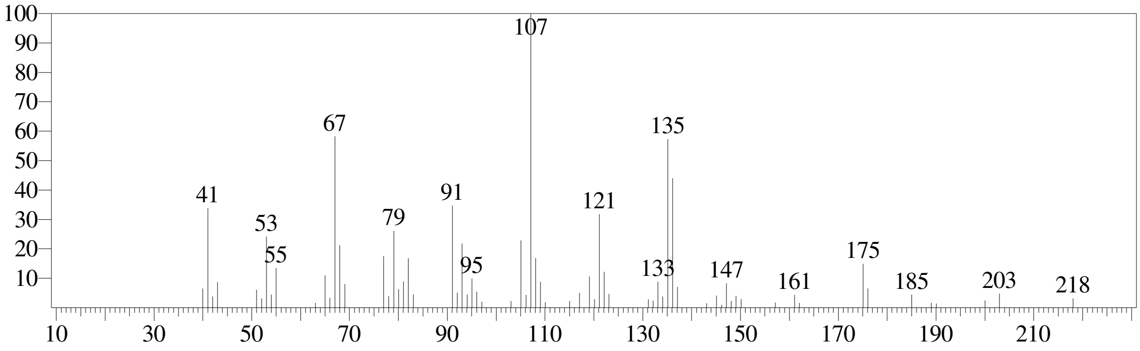

Hit#:5 Entry:48901 Library:NIST23-1.lib
SI:93 Formula:C13H18O CAS:55521-11-4 MolWeight:190 RetIndex:1480
CompName:3,7-Cyclodecadien-1-one, 10-(1-methylethenyl)-, (E,E)- $$ 10-Isopropenyl-3,7-cyclodecadien-1-one # $$

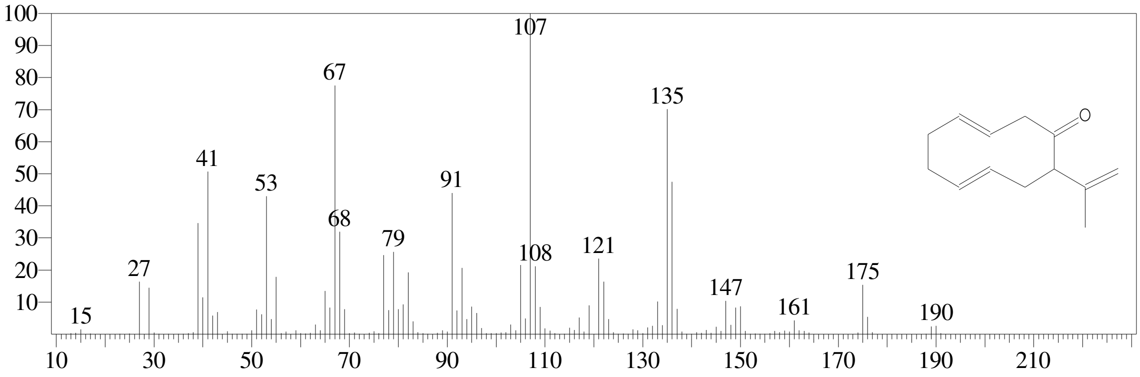

<< Target >>
Line#:30 R.Time:38.858(Scan#:4364) MassPeaks:39
RawMode:Averaged 38.850-38.867(4363-4365) BasePeak:66.00(8814)
BG Mode:None Group 1 - Event 1 Scan

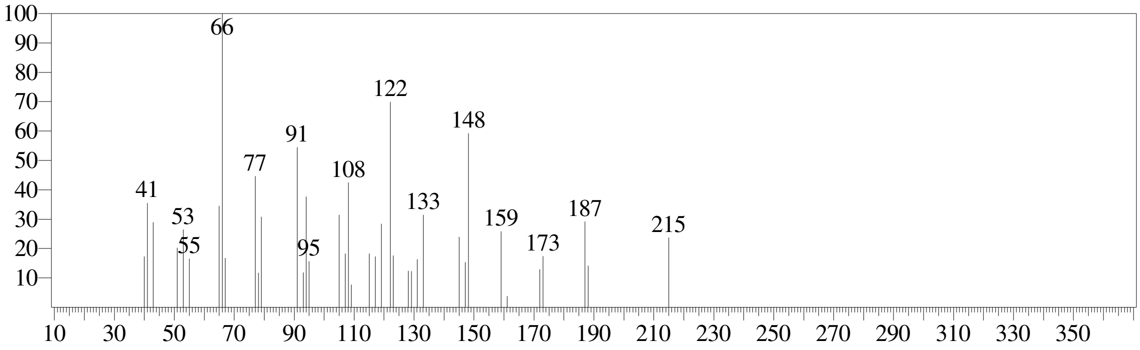

Hit#:1 Entry:92983 Library:NIST23-1.lib
SI:69 Formula:C15H18O2 CAS:151590-41-9 MolWeight:230 RetIndex:1823
CompName:(R)-3,5,8a-Trimethyl-7,8,8a,9-tetrahydronaphtho[2,3-b]furan-4(6H)-one $$ Naphtho[2,3-b]furan-4(6H)-one,

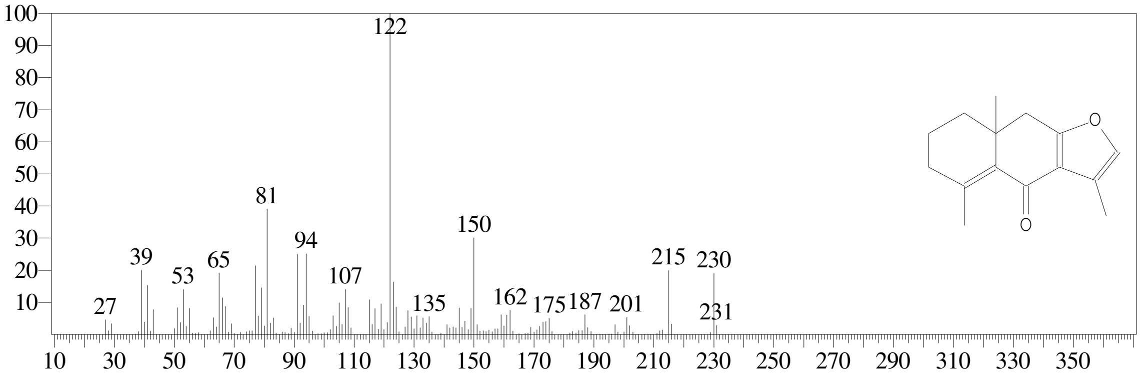

Hit#:2 Entry:24789 Library:NIST23s.lib
SI:69 Formula:C14H20O CAS:30557-76-7 MolWeight:204 RetIndex:1614
CompName:(3aR,6R,8aR)-7,7-Dimethyl-8-methylenehexahydro-1H-3a,6-methanoazulen-3(2H)-one $$ Khusimone $$ 12

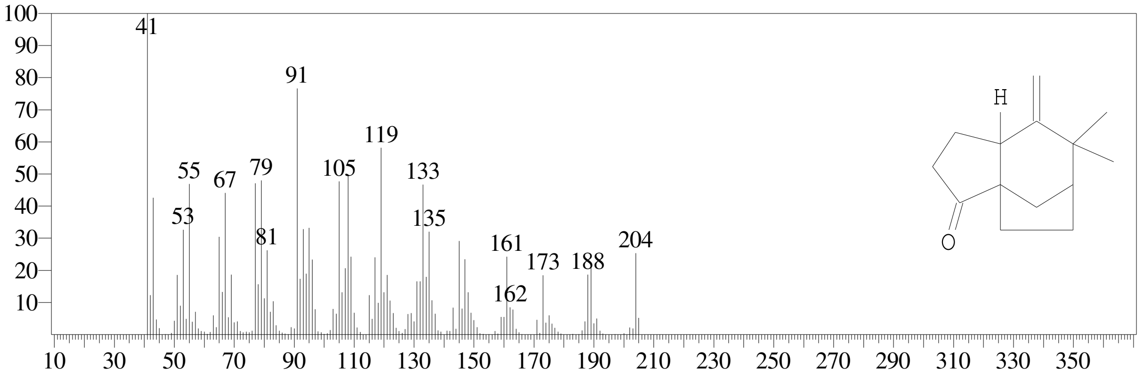

<< Target >>
Line#:30 R.Time:38.858(Scan#:4364) MassPeaks:39
RawMode:Averaged 38.850-38.867(4363-4365) BasePeak:66.00(8814)
BG Mode:None Group 1 - Event 1 Scan

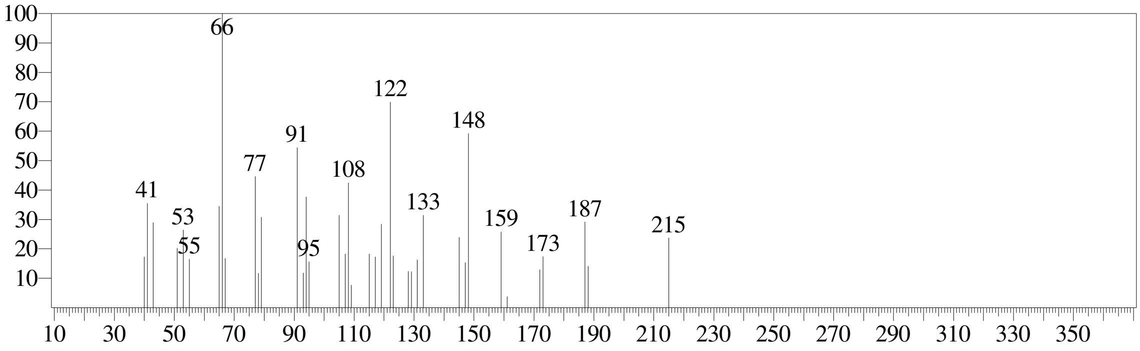

Hit#:3 Entry:115031 Library:NIST23-1.lib
SI:68 Formula:C16H24O2 CAS:1345675-64-0 MolWeight:248 RetIndex:1754
CompName:.alpha.-Santalyl formate $$ (Z)-5-((1R,3R,6S)-2,3-Dimethyltricyclo[2.2.1.02,6]heptan-3-yl)-2-methylpent-2-e

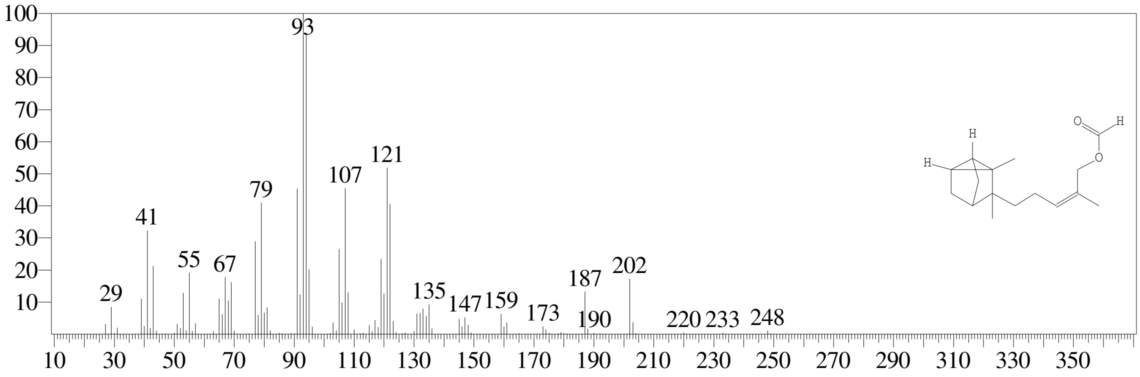

Hit#:4 Entry:14387 Library:NIST23-2.lib
SI:67 Formula:C20H28O6 CAS:52557-29-6 MolWeight:364 RetIndex:3134
CompName:1H-2,8a-Methanocyclopenta[a]cyclopropa[e]cyclodecen-11-one, 1a,2,5,5a,6,9,10,10a-octahydro-5,5a,6-trihyc

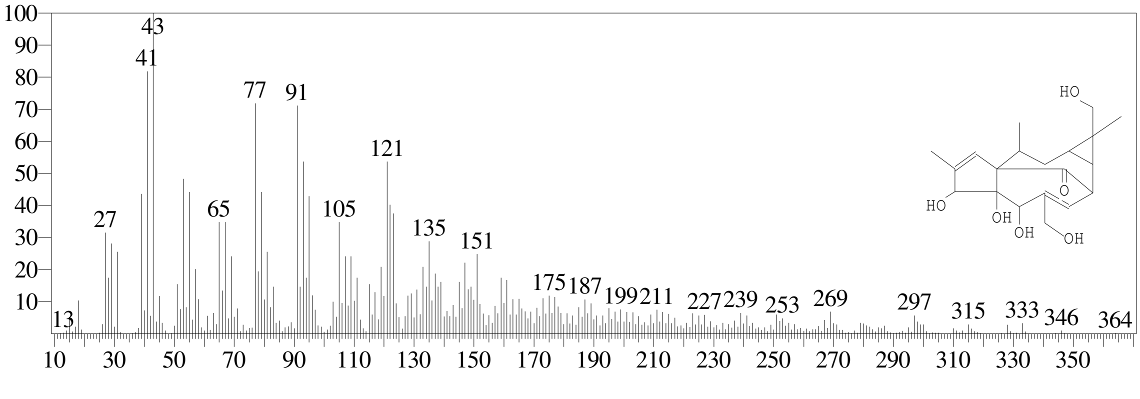

<< Target >>
Line#:30   R.Time:38.858(Scan#:4364) MassPeaks:39
RawMode:Averaged 38.850-38.867(4363-4365) BasePeak:66.00(8814)
BG Mode:None Group 1 - Event 1 Scan

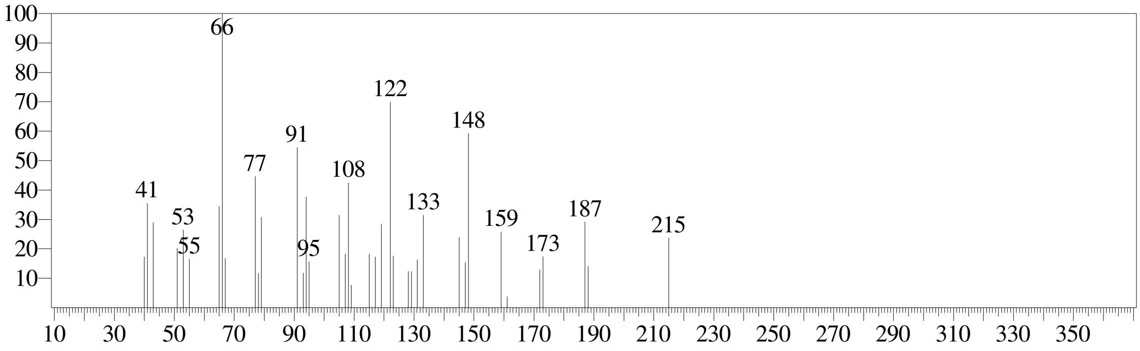

Hit#:5 Entry:24970 Library:NIST23s.lib
SI:67 Formula:C15H24 CAS:3650-28-0 MolWeight:204 RetIndex:1411
CompName:1,4-Methano-1H-indene, octahydro-4-methyl-8-methylene-7-(1-methylethyl)-, [1S-(1.alpha.,3a.beta.,4.alpha.,

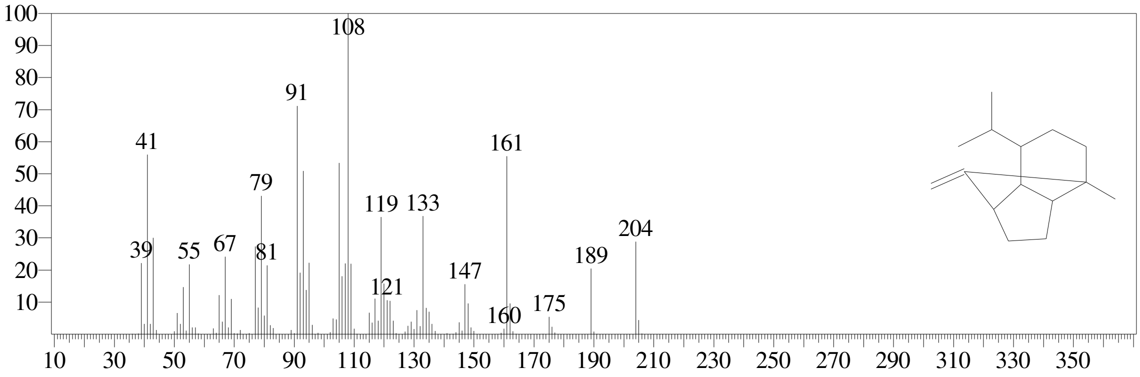

Supplement: Supplementary file 1 [file plants-15-01406-s001.zip › EU bb.pdf]
