# Supplementary material for: Unveiling Chemical Profile and Insecticidal Potential of Essential Oils from Leaves of Seven Eugenia L. Species (Myrtaceae)
Source: Plants (Basel). 2026 May 5;15(9):1406. doi: 10.3390/plants15091406 (PMC13165059; doi:10.3390/plants15091406)
Supplement: Supplementary file 1 [file plants-15-01406-s001.zip › Suplementar materials Figure S1.pdf]

Unveiling Chemical Profile and Insecticidal Potential of Essential Oils from Leaves of Seven *Eugenia* L. Species (Myrtaceae)

Lorene Armstrong [1,2,*], Nayana Figueiredo Pereira [3], Diefrey Ribeiro Campos [4], Yara Peluso Cid [4], Irailson Thierry Monchak [1], Neide Mara Menezes Epifânio [5], Douglas Siqueira Almeida Chaves [5] and Jane Manfron [1,2]

[1] Postgraduate Program in Pharmaceutical Sciences, State University of Ponta Grossa, Ponta Grossa 84030900, Paraná, Brazil
[2] Postgraduate Program in Health Sciences, State University of Ponta Grossa, Ponta Grossa 84030900, Paraná, Brazil
[3] Programa de Pós-graduação em Química, Universidade Federal Rural do Rio de Janeiro, Seropédica, Rio de Janeiro23897000, Brazil
[4] Clinical Research and Technological Innovation Center—Laerte Grisi, Federal Rural University of Rio de Janeiro, Seropédica, Rio de Janeiro23897000, Brazil
[5] Laboratório de Farmacognosia, Universidade Federal Rural do Rio de Janeiro, Seropédica, Rio de Janeiro23897000, Brazil
* Correspondence: larmstrong@uepg.br

Figure S1. Loadings calculated for the multivariate analysis of essential oils from *Eugenia* species.

	PC 1	PC 2	PC 3	PC 4	PC 5	PC 6
Hexanol <2->	-0.0037116	0.029065	-0.022969	0.07705	0.053641	-0.1619
Hexenal <(2E)->	-0.00077103	0.0040707	0.00060675	0.0087649	-0.019556	-0.037541
Hexenol <(3Z)->	-0.0014136	-0.0053972	0.009743	0.0072666	-0.017871	-0.033274
Santene	0.0061319	-0.0032283	-0.0065663	0.054579	0.057236	-0.0026367
Pinene <α->	0.9488	0.054144	0.011048	-0.21374	-0.014726	-0.038429
Camphene	0.0033774	-8,33E-01	-1,56E-02	-0.0055996	-0.00089333	0.001421
Pinene <β->	0.047275	-0.0052394	-0.0087106	0.0073312	0.065412	0.012984
Myrcene	0.0059282	-0.0090772	-0.033368	0.037781	0.049595	-0.0057092
Carene <δ-2->	0.0019341	0.0028563	0.0022153	0.03011	-0.011931	-0.045543
Phellandrene <α->	0.0081758	-0.0043044	-0.008755	0.072772	0.076314	-0.0035157
Cymene <ρ->	0.0014781	0.023034	0.024761	0.09241	-0.17048	-0.27542
Limonene	0.097583	-0.017769	-0.058438	0.64847	0.55451	-0.056373
Cineole <1.8->	-0.0076092	0.10131	0.092002	0.30955	-0.55075	-0.33398
Ocimene <(Z)-β->	0.009811	-0.0051652	-0.010506	0.087326	0.091577	-0.0042188
Campholenal <α->	0.018804	-0.0099001	-0.020137	0.16738	0.17552	-0.008086
Terpinene <γ->	0.0011674	0.014041	0.01242	0.033329	-0.07273	-0.018237
Isoborneol	0.0054603	-0.0028747	-0.0058471	0.048601	0.050967	-0.002348
Terpineol <γ->	0.041163	-0.0010153	-1,90E-01	-0.068246	-0.010887	0.017318
Borneol	0.00018038	0.0028109	0.0030216	0.011277	-0.020804	-0.033611
Terpineol <α->	0.0031629	0.0048098	0.0052446	0.014854	-0.036872	-0.057153
Cubebene <α->	-0.015226	-0.055612	0.0048713	-0.0074326	-0.040514	-0.15084
Isoledene	-0.0057168	-0.018492	0.021486	-0.013369	0.0089024	-0.012484
Copaene <α->	-0.0072429	0.019693	0.012137	0.017999	-0.039038	0.25529
Panasinsene <β->	-0.0040738	0.015271	0.0056157	-0.014151	0.015914	-0.02319
Elemene <β->	-0.05846	-0.016168	-0.21031	-0.124	0.0056593	-0.014294
Bornyl acetate	0.0026386	-6,51E-01	-1,22E-02	-0.0043747	-0.00069791	0.0011101
Ylangene <α->	0.016043	-0.0003957	-7,41E-02	-0.026598	-0.0042433	0.0067497
Caryophyllene <(E)-	-0.12121	0.43922	0.16695	0.052172	-0.10048	-0.0086143
Guaiene <α->	-0.048405	-0.099248	-0.4398	-0.017232	0.048393	-0.081329
Aromadendrene	-0.089032	0.32491	0.12528	-0.25818	0.26809	-0.15582
Farnesene-(Z)	0.00016534	0.0025767	0.0027698	0.010337	-0.01907	-0.03081
Muurola-3.5-diene <	-0.0023022	0.018268	0.012212	0.02144	-0.046559	-0.10884
Himachalene	-0.00032347	-0.001639	0.0014178	-0.00066014	0.00030434	-0.00040859
Muurola-3.5-diene <	0.0029199	-0.0015373	-0.0031268	0.02599	0.027255	-0.0012556
Humulene <α->	-0.030056	0.054355	-0.046843	-0.021502	-0.052963	0.028179
Farnesene<β->	0.00029199	-0.00015373	-0.00031268	0.002599	0.0027255	-0.00012556
Aromadendrene <all	-0.010068	-0.091076	0.048339	0.037578	0.092417	-0.027066
Aromadendrane <del	-0.0095336	0.025922	0.015976	0.023691	-0.051385	0.33602
Caryophyllene <9-ep	-0.0039269	0.010677	0.0065805	0.0097583	-0.021165	0.13841
Gurjunene<γ->	-0.011488	-0.017776	-0.081602	-0.023247	-0.011684	-0.01457
Muurolene <γ->	-0.022524	-0.039788	-0.1439	-0.045606	-0.020165	-0.028561
Germacrene D	-0.014647	-0.028765	0.053537	-0.025127	0.0060249	-0.076964
Amorphene <α->	-0.0022529	-0.008546	0.00051	-0.0045838	0.00052343	-0.0028499
Selinene <β->	-0.12126	-0.028128	-0.54061	-0.28347	0.030484	-0.23301
Selinene<d->	-0.001418	0.0038556	0.0023763	0.0035238	-0.007643	0.049981
Amorphene <γ->	-0.012014	0.0021147	-0.0098639	0.021663	-0.073214	0.11639
Viridiflorene	-0.1315	0.48175	0.19643	-0.30603	0.26876	-0.013756

Curzerene	-0.040254	-0.20396	0.17643	-0.082151	0.037874	-0.050846
Muurolene <α->	-0.0097841	-0.025198	0.0074057	-0.011135	-0.0082378	0.058515
Guaiene <trans-β->	-0.0037523	0.010203	0.006288	0.0093246	-0.020225	0.13226
Bisabolene <β->	-0.015864	0.072264	0.052686	0.11963	-0.23874	0.24019
Germacrene A	-0.0031498	-0.0048742	-0.022375	-0.0063743	-0.0032038	-0.003995
Amorphene <δ->	-0.081837	-0.18068	-0.18702	-0.1228	-0.072363	0.24685
Selinene <7-epi-α->	-0.00062997	-0.00097484	-0.004475	-0.0012749	-0.00064075	-0.000799
Cadinene <δ->	-0.016138	0.073377	0.034328	-0.016711	-0.011214	-0.21983
Zonarene	-0.0016144	0.0043895	0.0027053	0.0040118	-0.0087013	0.056901
Nerolidol <(Z)->	-0.0056427	-0.028591	0.024732	-0.011516	0.0053091	-0.0071276
Cadinene <α->	-0.022858	-0.11582	0.10019	-0.04665	0.021507	-0.028874
Selina-3.7(11)-diene	-0.01071	-0.054269	0.046943	-0.021858	0.010077	-0.013529
Spathulenol	-0.0022705	0.028504	0.026287	0.084951	-0.15953	-0.10431
Globulol	-0.0077883	0.021176	0.013051	0.019354	-0.041978	0.27451
Viridiflorol	-0.0044505	0.012101	0.0074579	0.011059	-0.023987	0.15686
Cubeban-11-ol	-0.0019198	0.0052199	0.0032171	0.0047707	-0.010347	0.067666
Rosifoliol	-0.0039705	0.010796	0.0066536	0.0098668	-0.0214	0.13995
Cubenol <1-epi->	-0.0039487	0.010736	0.0066171	0.0098126	-0.021283	0.13918
Cedranone	-0.0028856	0.010817	0.0039778	-0.010024	0.011272	-0.016427
Muurolol <epi-α->	-0.0037742	0.010262	0.0063246	0.0093789	-0.020342	0.13303
Cadinol <α->	-0.0050768	-0.007856	-0.036063	-0.010274	-0.0051637	-0.006439
Intermedol <neo->	-0.0073006	0.013368	-0.0011046	0.011292	-0.032705	0.20184
Atractylone	-0.057793	-0.29283	0.25331	-0.11795	0.054376	-0.073001
Selin-11-en-4-α-ol	-0.015786	-0.024428	-0.11214	-0.031947	-0.016056	-0.020022
Germacrone	-0.095172	-0.48223	0.41714	-0.19423	0.089545	-0.12022
